# Chiral Supramolecular Hydrogel Enhanced Transdermal Delivery of Sodium Aescinate to Modulate M1 Macrophage Polarization Against Lymphedema

**DOI:** 10.1002/advs.202303495

**Published:** 2023-12-01

**Authors:** Xueqian Wang, Chunxiao Cui, Xinxian Meng, Chengyao Han, Beibei Wu, Xiaoqiu Dou, Changli Zhao, Yixin Zhang, Ke Li, Chuanliang Feng

**Affiliations:** ^1^ State Key Lab of Metal Matrix Composites Shanghai Key Laboratory for Molecular Engineering of Chiral Drugs School of Materials Science and Engineering Shanghai Jiao Tong University Shanghai 200240 China; ^2^ Department of Burns and Plastic Surgery Shanghai Children's Medical Center Shanghai Jiao Tong University Shanghai 200127 China; ^3^ Department of Plastic and Reconstructive Surgery Shanghai Ninth People's Hospital Shanghai Jiao Tong University School of Medicine Shanghai 200011 China

**Keywords:** chiral hydrogels, lymphedema, sodium aescinate, transdermal drug delivery

## Abstract

Sodium aescinate (SA) shows great potential for treating lymphedema since it can regulate the expression of cytokines in M1 macrophages, however, it is commonly administered intravenously in clinical practice and often accompanied by severe toxic side effects and short metabolic cycles. Herein, SA‐loaded chiral supramolecular hydrogels are prepared to prove the curative effects of SA on lymphedema and enhance its safety and transdermal transmission efficiency. In vitro studies demonstrate that SA‐ loaded chiral supramolecular hydrogels can modulate local immune responses by inhibiting M1 macrophage polarization. Typically, these chiral hydrogels can significantly increase the permeability of SA with good biocompatibility due to the high enantioselectivity between chiral gelators and stratum corneum and L‐type hydrogels are found to have preferable drug penetration over D‐type hydrogels. In vivo studies show that topical delivery of SA via chiral hydrogels results in dramatic therapeutic effects on lymphedema. Specifically, it can downregulate the level of inflammatory cytokines, reduce the development of fibrosis, and promote the regeneration of lymphatic vessels. This study initiates the use of SA for lymphedema treatment and for the creation of an effective chiral biological platform for improved topical administration.

## Graphical abstract

Illustration of the major skin structure (epidermis, dermis, and subcutaneous tissue) and the penetration mechanism of drug molecules. These chiral hydrogels can significantly increase the permeability of SA (sodium aescinate) with good biocompatibility due to the high enantioselectivity between chiral gelators and stratum corneum (the main barrier of skin). And these SA‐loaded chiral supramolecular hydrogels can modulate local immune responses by inhibiting M1 macrophage polarization to relieve lymphedema.

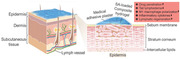

## 1 Introduction

Lymphedema is a debilitating and complex disease caused by lymphatic insufficiency.^[^ ^1^, ^2^ ^]^ Characterized by the accumulation of extracellular fluid in affected tissues, lymphedema can lead to progressive skin and subcutaneous tissue swelling, intradermal fibrosis, and chronic tissue inflammation, causing functional and psychological burdens for patients.^[^ ^3^ ^]^ Currently, a conservative palliative approach remains the mainstay of treatment for this disease but is rarely curative; thus, better therapy approaches are urgently required. In the development of lymphedema, inflammatory cell infiltration has been strongly associated to the onset of that.^[^ ^4^, ^5^, ^6^ ^]^ Studies have revealed that lymphedema has a large accumulation of macrophages, which can be a key regulator of the regeneration of collateral lymphatics following lymphatic injury.^[^ ^7^, ^8^ ^]^ The polarization and accumulation of M1 macrophages further contribute to local inflammation,^[^ ^9^ ^]^ which often results in abnormal tissue lipid deposition and high fibrotic manifestation.^[^ ^10^ ^]^ As a result, proper regulation of macrophage M1 polarization and deposition can be a curative method for lymphedema. When compared to the medications frequently used to treat edema induced by trauma or surgery, sodium aescinate (SA), which has anti‐inflammatory and anti‐swelling properties, may be a potential lymphedema treatment.^[^ ^11^, ^12^, ^13^, ^14^ ^]^ Previous research has found that SA can help decrease the levels of inflammatory cytokines such as tumor necrosis factor‐alpha (TNF‐α),^[^ ^15^ ^]^ which are effector cytokines of M1 macrophages. Nevertheless, conventional administration routes of SA are associated with numerous drawbacks,^[^ ^16^, ^17^ ^]^ including severe toxic side effects and short metabolic cycles.^[^ ^18^, ^19^, ^20^ ^]^ Thus, to prove and achieve better therapeutic effects, an alternative route for delivering SA to specific sites is needed.

Recently, the rapid development of transdermal drug delivery systems has attracted considerable attention.^[^ ^21^, ^22^, ^23^, ^24^, ^25^ ^]^ Such transdermal drug delivery methods make it possible to provide drugs locally at the site of the injury, reducing systemic side effects and enhancing the effectiveness of therapy. However, the skin, being the largest organ in the human body, is a primary barrier for foreign substances to penetrate, which may tremendously hinder drug delivery and limit its therapeutic effect.^[^ ^26^, ^27^, ^28^ ^]^ To overcome this barrier and enhance drug transdermal delivery, both chemical and physical approaches are being intensively researched. Physical techniques such as electroporation, lasers, and magneto/sonophoresis have been utilized to improve transdermal efficiency. Yet, these techniques have limitations on the types of drugs, and the additional external equipment is not convenient for practical use.^[^ ^29^, ^30^, ^31^, ^32^, ^33^ ^]^ Chemical methods usually use some penetration enhancers, such as lipids, alcohols, organic salts, etc., but they have irritating effects on the skin, and their use alone is not ideal.^[^ ^34^ ^]^ Among the various strategies to enhance the transdermal transmission of drugs, hydrogels have been described as a desirable drug delivery system when compared to other topically applied forms.^[^ ^35^, ^36^, ^37^, ^38^ ^]^ In particular, some chemically cross‐linked polymer hydrogels, such as hyaluronic acid, cellulose, chitosan, and so on, have been widely used as transdermal delivery systems. But for supramolecular hydrogels, which are formed by physical cross‐linking are particularly preferred in clinical practice because of the fact that these kinds of hydrogels have high skin biocompatibility without using chemical cross‐linking agents.^[^ ^39^, ^40^ ^]^ Various drugs can be efficiently stored in the hydrogel network and slowly released at specific sites; nevertheless, relatively little study on these hydrogels as transdermal delivery systems has been explored.^[^ ^41^, ^42^, ^43^, ^44^, ^45^, ^46^, ^47^ ^]^ Among the well‐known skin layers, the stratum corneum (SC) represents challenging horizontally packed layers in the outermost layer of the skin, mainly composed of chiral molecules (e.g., proteins and ceramides), and it is a responsive substance that can be altered by changes in the skin environment.^[^ ^48^, ^49^, ^50^ ^]^ In addition to being a typical characteristic of SC, the chiral structure is a beneficial structural component of hydrogels. Chiral compounds are very important in the development of transdermal delivery, and good correlativity of in vivo and in vitro has become an ideal requirement in the development of drug penetrants. This is particularly important for regulating drug penetration by interacting with and modifying the conformation of intercellular proteins.^[^ ^51^, ^52^ ^]^ So far, many studies have explored the differences in the transdermal penetration of drug enantiomers; however, to date, little attention has been paid to the stereoselectivity effects of chiral supramolecular hydrogels in transdermal delivery and their mechanism of drug penetration. Based on this, it is necessary to study the differences and mechanisms of chiral supramolecular hydrogels in promoting drug penetration in order to better enhance drug transdermal delivery.

**Scheme 1:**
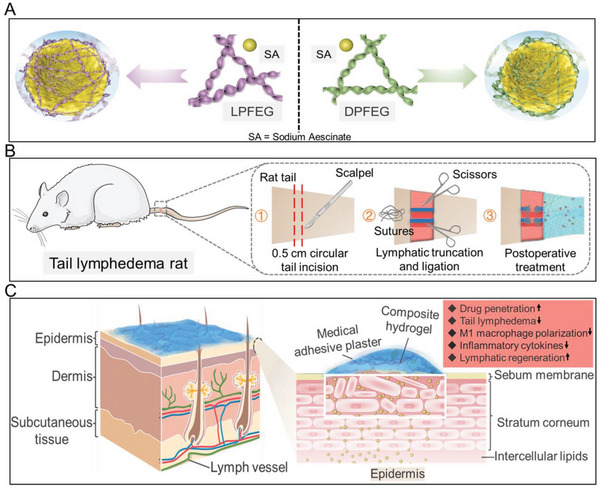
A) Schematic illustration of co‐assemblies of supramolecular gelators (L/DPFEG) and drugs (SA). B) The establishment of a rat model with tail lymphedema and its postoperative treatment. C) Illustration of the major skin structure (epidermis, dermis, and subcutaneous tissue) and the penetration mechanism of drug molecules. The composite hydrogel promotes drug penetration by disturbing the alignment of stratum corneum (the main barrier of skin).

Previous research by our group demonstrated that chiral supramolecular hydrogels formed by L‐phenylalanine gelators (LPFEG) and D‐phenylalanine gelators (DPFEG) could carry drug molecules and achieve gradual release with high biosafety.^[^ ^53^ ^]^ In this study, chiral supramolecular hydrogels are prepared using a cooperativity approach based on co‐assemblies of gelators and drug molecules (SA) for the efficient delivery of SA against lymphedema. SA‐loaded chiral composite hydrogels can regulate inflammatory reactions by reducing the M1 polarization status of macrophages in vitro and may play an anti‐inflammatory role in the progression of lymphedema. In addition, the chirality property of L/DPFEG is beneficial for SA transdermal delivery due to the enantioselective interaction with the stratum corneum. LPFEG‐based hydrogels exhibit preferable drug penetration than DPFEG‐based hydrogels with the maximum value of Q_12h_ (12 h cumulative transmission) being 205.45 ± 5.65 µg cm^−2^, which is 1.34 times higher than that of the SA solution. In vivo experiments further demonstrate that these composite hydrogels can significantly relieve the tail lymphedema of rats by promoting better SA delivery due to reduced levels of inflammatory factors **Scheme** **1**.

## 2 Results and Discussion

### 2.1 Preparation and Characterization of SA, L‐SA, and D‐SA

**Figure 1:**
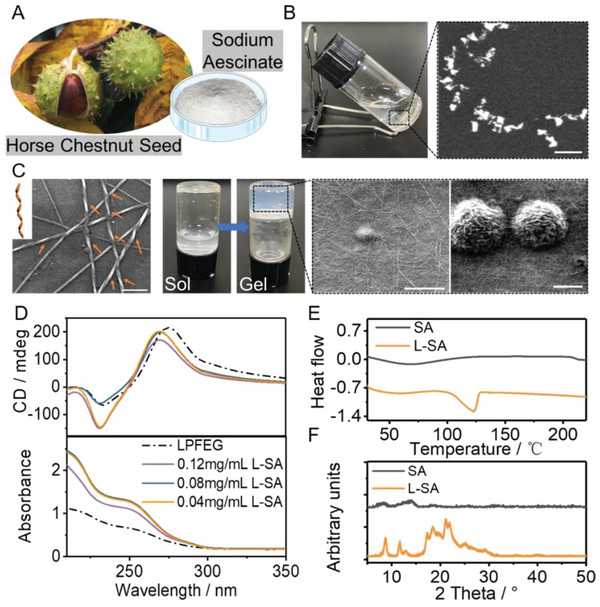
Characterization of SA and L‐SA. A) Illustration of SA. B) Photograph and SEM image of the SA solution. Scale bar: 10 µm. C) Photograph and SEM of L‐SA. Scale bar = 5 µm. The SEM enlargement of L‐SA is in the lower right corner (Scale bar = 10 µm). D) CD and corresponding UV‐vis spectra of pure LPFEG and L‐SA with different SA concentration. E) DSC thermograms and F) XRD patterns of SA and L‐SA.

SA is a kind of sodium salt saponin that is isolated from horse chestnut seeds and appears as a white powder (**Figure** **1**A). As a saponin, the free SA in solution existed in an amorphous state (Figure 1B). It was found that other saponins also existed in a similar amorphous form.^[^ ^54^, ^55^, ^56^ ^]^ However, this amorphous state may raise concerns about the stability of the preparation process and long‐term storage. SA is available in a variety of therapeutic forms (e.g., oral tablets, injections), and hydrogels have been prepared in this study by a facile heating‐cooling method.

Based on the reported literature (Scheme S1, Figure S1‐S4, Supporting Information), L/DPFEG was synthesized by a conventional liquid phase reaction. 1, 4‐ diamide benzene was chosen as a gel scaffold because its symmetrical structure could provide strong parallel interactions between hydrogen‐bonded amide parts and hydrophobic interactions between hydrophobic nuclei. This was necessary for self‐assembly and therefore allowed gelation to occur. The connection of phenylalanine side chains further introduced the hydrophobic interaction force of gelation. Hydrophobic benzene ring and phenylalanine could shield the contact between amide and water, thus promoting the intermolecular hydrogen bond stack, which stabilized hydrogel structures.^[^ ^57^ ^]^ SEM (scanning electron microscopy) images revealed that the pure LPFEG hydrogel showed a left‐handed helical fiber microstructure (Figure 1C). By contrast, its enantiomer DPFEG showed opposite homogeneous right‐handed helical nanofibers (Figure S5, Supporting Information). When the ultrasonic dispersion of L/DPFEG was mixed with SA (abbreviated as L/D‐SA), the turbidly flowing liquid was transformed into a semi‐transparent homogeneous composite hydrogel after heating and cooling, which could stably exist at room temperature for at least 2 weeks (Figure S6‐S7, Supporting Information). To further observe the morphology of composite hydrogels, SEM was performed. As shown in Figure 1C, the L‐SA composite hydrogel exhibited flower‐like morphologies accompanied by chiral fibers under SEM. The same morphologies occurred in the D‐SA composite hydrogel (Figure S8, Supporting Information). The circular dichroism (CD) spectrum (Figure 1D) showed that the LPFEG hydrogel had one positive CD signal at around 272 nm and a negative one at 228 nm. For the DPFEG hydrogel, the positive CD signal was observed at about 231 nm, while 264 nm corresponded to the negative CD signal (Figure S9, Supporting Information). The different concentrations of L/D‐SA hydrogels showed obvious characteristic peaks similar to those of L/DPFEG, indicating that chiral properties were introduced into the composite system. Additionally, the diffraction peaks in the X‐ray diffraction (XRD) pattern of the amorphous SA were not obvious. Compared with SA, the L‐SA hydrogel displayed more diffraction peaks in the XRD pattern and an extra melting endotherm of about 123°C (Figure 1E‐F), which were the characteristic features of crystalline SA.^[^ ^58^ ^]^ Similarly, the D‐SA hydrogel displayed an additional melting endotherm around 132°C as well as sharp diffraction peaks (Figure S10‐S11, Supplemental Information). The crystallization of SA in hydrogels contributed to the attractive appearance and potentially increased physical stability of products.

### 2.2 In vitro Biocompatibility of L/D‐SA Composite Hydrogels

**Figure 2:**
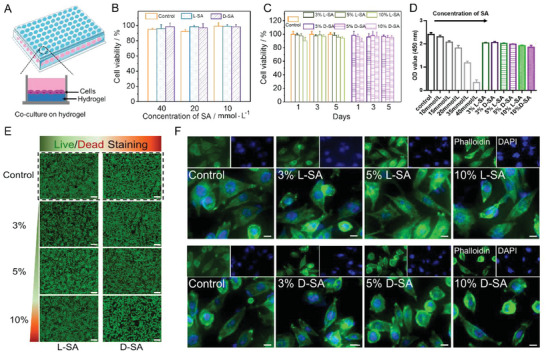
Biocompatibility of composite hydrogels in vitro. A) Illustration of cells co‐cultured with hydrogel. B) CCK‐8 assay of L929 cells co‐cultured with various concentrations of SA composite hydrogels for three days (*n* = 3). C) CCK‐8 test of L929 cells co‐cultured with equal volumes of PBS and composite hydrogels after one, three, and five days (SA = 20 mmol L^−1^, *n* = 3). D) CCK‐8 test of macrophages co‐cultured with various concentrations of SA and composite hydrogels (3% L/D was chosen, *n* = 3). E) Fluorescence images of a live/dead assay of L929 cells cultured for 3 days with different concentrations of L‐SA and D‐SA. Scale bar: 50 µm. F) Fluorescence microscopy images of L929 cells at days 3 with different concentrations of L/D‐SA, blue (DAPI) for nuclei, and green (Fluoresceine Isothiocyanate (FITC) labeled phalloidin) for F‐actin. Scale bar: 10 µm.

Good biocompatibility is an important feature of biomaterials for long‐term use. To assess the cytotoxicity of L‐SA and D‐SA composite hydrogels in vitro, the cell counting kit‐8 (CCK‐8) assay and live/dead staining were used. First, the effect of different concentrations of SA on cells was carried out. As shown in the illustration, L929 cells (mouse fibroblast cells) were co‐cultured with hydrogel (**Figure** **2**A). After a 3‐day incubation, the cell viability percentage of L929 did not significantly decline even at a high SA concentration, indicating that the concentration of SA had no effect on L929 cell viability (Figure 2B). Following incubation with various concentrations of SA hydrogels, the morphologies of L929 cells were also evaluated using fluorescence microscopy. The images found that the majority of cells grew well and exhibited a fiber‐like morphology (Figure S12, Supplemental Information). However, there were differences in the sensitivity of macrophages to SA. The cell viability was observed to decrease in an obvious manner with increased concentrations of SA (Figure 2D; Figure S13, Supplemental Information). Thus, SA at a concentration of 20 mmol mL^−1^ was selected for further study under the premise of ensuring safety. Then, the concentration of L/DPFEG was varied to investigate its effect on L929 cells. The results of the CCK‐8 assay revealed that L929 cells had high cell viability in relatively low concentrations of L/DPFEG (3% and 5%) and maintained more than 90% cell viability over the course of the 5‐day cell‐hydrogel co‐culture. At a higher L/DPFEG concentration (10%), cell viability dropped but remained above 80% (Figure 2C). In the representative images of live/dead staining (Figure 2E) and actin cytoskeleton staining (Figure 2F; Figure S15, Supplemental Information), a high intensity of green fluorescence was observed, and the cellular morphologies were well maintained in all groups when the cells were cultured on plates in the presence of hydrogels. This observation was in good agreement with the CCK‐8 result, suggesting that all of the composite hydrogels had good biocompatibility to normal cells and could be a promising candidate in the biomedical areas. At the same time, with the treatment of L‐SA and D‐SA composite hydrogels (SA = 20 mmol L^−1^, 3% L/D was chosen) on RAW264.7, no significant difference was found in the cell vitality compared with L929 cells (Figure 2D; Figure S14, Supplemental Information).

### 2.3 In vitro Impact of L/D‐SA Composite Hydrogels on Macrophage M1 Polarization

**Figure 3:**
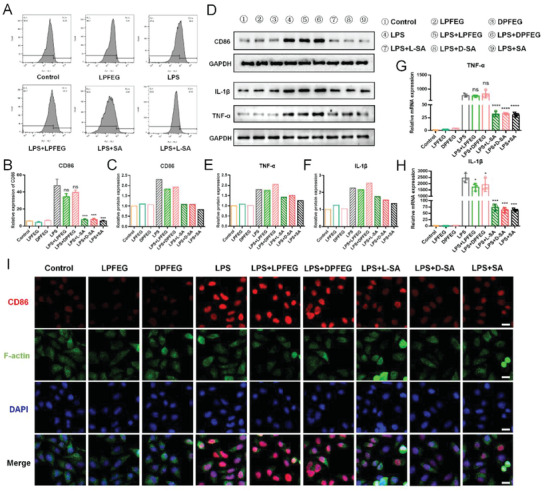
Influence of L/D‐SA composite hydrogels on macrophage M1 polarization. A) Effect of L/D‐SA composite hydrogels on M1 polarization on LPS‐induced M1 polarization in RAW264.7 cells (SA = 20 mmol/L, 3% L/D was chosen). B) The CD86 expression was detected by flow cytometry. C) The corresponding signal intensity quantification of CD86 in D). D) Western blot analysis results of CD86 (M1 marker) and IL‐1β and TNF‐α (the special functional markers for M1) by macrophages after various treatments. GAPDH (glyceraldehyde‐3‐phosphate dehydrogenase) was used as the protein loading control. The corresponding signal intensity quantification of E) TNF‐α and F) IL‐1β in D). The gene level of G) TNF‐α and H) IL‐1β (n = 3). I) Immunofluorescent staining images of CD86 expressed by macrophages after different treatments. Red (CD86), green (F‐actin), and blue (DAPI). Scale bar: 50 µm. The results are expressed as the mean ± SD, n = 3 per group. The P value is calculated by t‐test. *p < 0.05, **p < 0.01, ***p < 0.001, and ****p < 0.0001, respectively.

Regulating the excitation and accumulation of macrophages, especially by inhibiting the M1 polarization, is crucial in the treatment of lymphedema. The impact of pure LPFEG/DPFEG hydrogels on RAW264.7 (the mouse mononuclear macrophage leukemia cell line) under M0 status and LPS‐induced M1 phenotype was firstly detected in vitro. It was discovered that the mere LPFEG/DPFEG hydrogel had almost no effect on macrophage M1 polarization compared to the control group. At the same time, the relative expression of CD86 (leukocyte differentiation antigen 86), the special surface phenotype marker of M1, and IL‐1β (interleukin 1 beta) and TNF‐α (tumor necrosis factor alpha), the special functional markers for M1, was not obviously decreased with pure LPFEG/DPFEG hydrogels (**Figures** **3**A‐B,G‐H; Figure S16, Supplemental Information). No proinflammatory or inflammatory regulating effects were noticed with the treatment of LPFEG/DPFEG composite hydrogels. Therefore, interference caused by hydrogels with the influence of macrophage M1 polarization had been excluded. Then, the efficacy of L/D‐SA composite hydrogels in inhibiting LPS‐induced M1 polarization in RAW264.7 was tested. LPS (lipopolysaccharide)‐induced M1 polarization was applied as a positive control to detect whether L/D‐SA composite hydrogels could reverse the M1 polarization of macrophages. After being stimulated with LPS (100 ng mL^−1^) after 24 hours, flow cytometry showed that LPS‐stimulated RAW264.7 cells expressed considerably more CD86 than the negative control (Figure 3A‐B). Immunofluorescent staining against CD86 was positive in LPS‐activated macrophages (M1) (Figure 3I; Figure S17, Supplemental Information). Furthermore, mRNA (messenger RNA) expression and secretion of IL‐1β and TNF‐α were significantly increased. By contrast, LPS‐promoted CD86 expression was significantly reversed with the treatment of SA‐loaded composite hydrogels (Figure 3A‐D; Figure S18, Supplemental Information). In contrast to the positive control, macrophages in all SA‐treated groups displayed decreased fluorescence expression of CD86 (Figure 3I). Additionally, the mRNA and protein expression of IL‐1β and TNF‐α were also declined, indicating the possible suppressive effects of SA on macrophage M1 polarization (Figure 3D‐H; Figure S19, Supplemental Information). Besides, a comparison of SA and L/D‐SA composite hydrogels was made. The results indicated that the modification conducted on SA would not impact the efficacity of SA on macrophage polarization. To conclude, the treatment with L/D‐SA composite hydrogels could inhibit the M1 polarization of macrophages and may play an anti‐inflammatory role in the progression of lymphedema.

### 2.4 In vitro Transdermal Drug Release Test

**Figure 4:**
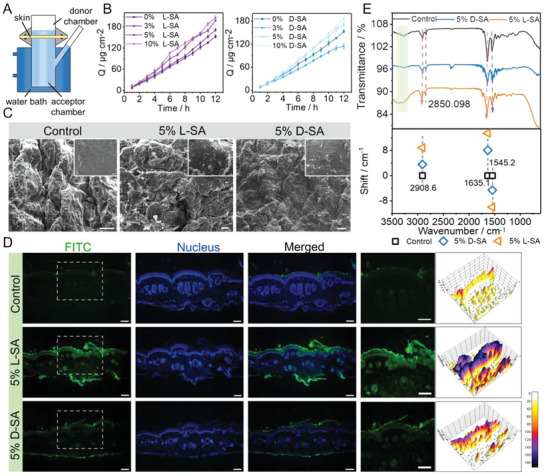
Effects of composite hydrogels on drug penetration. A) Schematic illustration of the Franz diffusion cell system for the skin permeation study in vitro. B) Cumulative amount of SA permeated from different composite hydrogels across excised rat tail skin over time (n = 3). C) SEM of skin treated with different composite hydrogels. Scale bar = 100 µm. Enlarged images is shown in the upper right corner. Scale bar: 10 µm. D) Fluorescent images of the skin after incubation with different composite hydrogels for 4 h. The fluorescent intensity in the gray box is converted to a 3D stack using surface plotting from ImageJ software. Scale bar = 100 µm. E) ATR‐FTIR of skin after various composite hydrogel treatments (the upper image) and the corresponding shift in wavenumber (the following image).

The vertical Franz diffusion cell system was employed to evaluate the permeation capability of composite hydrogels through the tail skin of the rat in vitro (**Figure** **4**A). Fluorescein isothiocyanate isomer (FITC) was grafted onto SA to prepare composite hydrogels and its potential ability to promote transdermal drug delivery was investigated by UV‐vis spectrophotometry based on the standard curves (Figure S20, Supplemental Information). FITC could be used to visualize the transdermal penetration process of drugs. The L/D‐SA hydrogels were first added into the donor chamber, and the amounts of SA molecules permeating across the skin in the medium of the acceptor chamber was collected every hour. During the experiment, the systems were placed in a water bath at 37°C and gently stirred. By comparing the composite hydrogels with various L/DPFEG concentrations, it was discovered that LPFEG‐based hydrogels have better drug penetration ability than DPFEG‐based hydrogels. The 3% D‐SA hydrogel exhibited less permeation, that was, 114.67 µg cm^−2^ after 12 h as shown in Figure 4B, while the 3% L‐SA hydrogel showed slightly higher SA permeation through the skin, that was, 171.72 µg cm^−2^ compared with the equal concentration of SA in solution (153.37 µg cm^−2^). The 5% L‐SA hydrogel exhibited significantly higher permeation of SA across the skin, that was, 198.37 µg cm^−2^ as compared with the 5% D‐SA hydrogel, and this value was further increased to 205.45 µg cm^−2^ when 10% LPFEG was added as a penetration enhancer in the hydrogel. Additionally, the permeability parameters (steady infiltration rate J_S_ and infiltration ratio ER) of samples containing LPFEG enhancer were obviously increased, and the amount of drug penetration displayed concentration‐dependent (**Table** **1**). The experiment had shown that the effect of penetration enhancers was directly proportional to the concentration, except for 3% DPFEG. However, some studies indicated that when the penetration enhancer effect became stable, the higher the concentration, the less active the penetration enhancer was, and it could also cause severe irritation.^[^ ^59^ ^]^ Thus, 5% L/D‐SA hydrogel was chosen for the subsequent study based on its higher permeation enhancement activity and cell viability results. For the purpose of visualization and qualitative analysis, skin tissues with different treatments were collected after 4 hours, cryosectioned, and detected by a fluorescence microscope. The fluorescent images demonstrated that the SA solution showed a negligible fluorescence signal, while 5% D‐SA hydrogel showed slightly higher fluorescence after 4 hours (Figure 4D; Figure S21, Supplemental Information). In contrast to those incubated with either the 5% D‐SA hydrogel or the SA solution, incubations with the 5% L‐SA hydrogel uniformly dispersed a stronger fluorescent signal of SA throughout the skin. Furthermore, the SA signal of the 5% L‐SA hydrogel was visible in both the epidermis and dermis of note, indicating that the LPFEG could effectively penetrate drugs into the deep layers of the skin, particularly through the stratum corneum (the major barrier for transdermal drug delivery). The qualitative results of fluorescence microscopy were consistent with the quantitative statistics, demonstrating that LPFEG‐based chiral supramolecular hydrogels had a superior ability to promote drug penetration.

**Table 1:** Permeation parameters of different concentrations of L/D‐type gelators through rat tail skin to sodium aescinate (n = 3).

Penetration enhancer	*Q*_12h_ [µg cm^−2^]	*J_S_* [µg cm^−2^ h^−1^]	*T*_lag_ [h]	ER
Control	153.37±3.91	13.06±0.29	0.74	1.00
3% L‐SA	171.72±3.48	14.58±0.43	0.82	1.12
3% D‐SA	114.67±7.20	10.17±0.15	0.46	0.75
5% L‐SA	198.37±2.32	17.02±0.35	0.72	1.29
5% D‐SA	171.63±4.61	15.53±0.26	0.86	1.12
10% L‐SA	205.45±5.65	18.10±0.42	0.68	1.34
10% D‐SA	185.36±3.14	18.48±0.48	1.08	1.21

To explore the potential mechanism of composite hydrogels in promoting drug penetration, the tail skin of rats was monitored under a SEM following various treatments. According to the SEM images, the skin surface of the SA group was smooth and free of any obvious abnormalities. The stratum corneum was arranged regularly and tightly with narrow cracks, and some secretions were adhered to the epidermis (Figure 4C). In the 5% D‐SA group, the cracks between the epidermis began to widen, and the outermost stratum corneum fell off compared with the SA group. From the longitudinal section of fluorescence images, it could be found that the stratum corneum structure was loose and appeared to have cavitation structures (Figure S21, Supplemental Information). The most significant changes could be found in the 5% L‐SA group, which had increased inter‐epidermal fissures and obvious stratum corneum shedding. The longitudinal section illustrated that the stratum corneum structure was obviously loosened and abscissed, and the stratum corneum was separated from the epidermis. From the H&E (hematoxylin and eosin) staining, it could also be observed that the cuticle architecture was loose and presented a free state in the 5% L‐SA group, while the 5% D‐SA group demonstrated a slight change in the cuticle (Figure S22, Supplemental Information). Then, ATR‐FTIR (attenuated total reflection infrared) spectroscopy was applied on different treated skins. In the SA group, the ‐OH stretching vibration of stratum corneum lipids appeared at around 3273 cm^−1^, and the initial peak positions of V*_s_* CH2 and V*_as_* CH2 were 2850 cm^−1^ and 2908 cm^−1^, respectively (Figure 4E). The ATR‐FTIR spectrum gave clear proof that both the 5% L‐SA group and the 5% D‐SA group presented a slight shift at V*_s_* CH2, while the peak positions of V*_as_* CH2 shifted to high wavenumbers with an increase of 8.9 units and 3.6 units, respectively, compared with the SA group. At the same time, the ‐OH stretching vibration moved to higher wavenumbers coupled with the peak broadened. It had been reported that the frequency of the ‐OH stretching vibration was about 3200–3300 cm^−1^. When the peak value of the ‐OH vibration shifted to a higher wavenumber, the skin lipids transitioned into a comparatively loose state, and the hydration of the stratum corneum increased, which was beneficial for drug molecules to penetrate. The characteristic peak of the amide bands was also discovered to be varied. A distinct shift from 1635 cm^−1^ in the original SA group to 1643 cm^−1^ in the D‐SA group and 1650 cm^−1^ in the L‐SA group occurred for the amide I band. Similarly, the amide II band displayed a clear decrease from 1545 to 1540 cm^−1^ in the D‐SA group while a significant shift occurred from 1545 to 1535 cm^−1^ in the L‐SA group. The transformation from a α‐helical to a β‐folded conformation of proteins in the skin is reflected by the change of peak location in amide bands, which increased the permeability of the keratinocytes to better drug molecules penetration.^[^ ^60^, ^61^, ^62^, ^63^ ^]^ As a result, the fact that L/DPFEG promoted drug penetration could be attributed to enhancing stratum corneum hydration and opening the keratin dense structure to reduce the barrier resistance. In addition to shielding the skin from external damage, the stratum corneum also maintains the skin with a constant resistance value.^[^ ^64^ ^]^ However, the resistance value of skin will change in response to outside factors. After different treatments were applied to the isolated stratum corneum, it was found that the resistance value of the LPFEG group significantly decreased after 6 hours, and the change in the LPFEG group was more noticeable than that in the DPFEG group (Figure S23, Supplemental Information). This indicated that LPFEG could disturb the barrier effect of the stratum corneum and enhance the permeability of the skin by reducing the resistance value, making it easier for drug molecules to penetrate the skin. Moreover, the stratum corneum of the skin contains a variety of chiral compounds (e.g., ceramides, keratin), which can interact with chiral molecules to develop a range of stereoselective effects.^[^ ^65^ ^]^ Ceramides are the predominant substances in the stratum corneum and are essential for skin permeability barrier function. Based on this, the interaction between L/DPFEG and ceramides was assessed at the absorbance of 450 nm by the Elisa kit (Figure S24, Supplemental Information). The results showed that ceramide content was enormously decreased in the LPFEG group after 6 hours, indicating a stronger interaction between LPFEG and ceramide. DSC (differential scanning calorimetry) was also carried out to investigate the interaction mentioned above. The DSC curves of each component and mixture sample of ceramide and L/DPFEG were shown in Figure S25. It was unmistakably indicated that there were three endothermic peaks at 82.7, 128.5, and 126.3°C; each peak was associated with the melting points of ceramide, LPFEG, and DPFEG, respectively. DSC scans of mixtures revealed broadening of the ceramide melting peak with a lowered onset and decreased melting enthalpy, which were positive indicators of interaction between ceramide and L/DPFEG. The melting enthalpy and onset temperature of the LPFEG and ceramide mixture (70.13°C and 16.44 J g^−1^) were lower than those of the DPFEG and ceramide mixture (70.53°C and 21.89 J g^−1^). It might be related to the stronger interaction between LPFEG and ceramide. This was also the reason for the differentiation between LPFEG‐based hydrogels and DPFEG‐based hydrogels in terms of facilitating penetration.

### 2.5 Evaluation of Rat Tail Lymphedema in Vivo

Although SA can reduce edema, its therapeutic impact on lymphedema has not been reported. Currently, there is no commercially available SA gel that has been shown to treat lymphedema. Additionally, no research to far have mentioned the use of chiral supramolecular hydrogels as penetrants to enhance medicinal molecules. Due to its distinct skin safety and dynamic responsiveness, supramolecular hydrogels differ from polymer hydrogels and are an effective transdermal drugs carrier. Furthermore, the unique chiral structure of the skin and its distinctive stereoselectivity between chiral compounds are crucial, which is also the reason why there are variations in skin penetration following the combination of the same medicinal molecule with various chiral supramolecular hydrogels. This will enable us to develop a new SA gel for lymphedema treatment. It had been demonstrated in the aforementioned experiments that L/D‐SA composite hydrogels could prevent macrophage M1 polarization. Therefore, in this section, we show whether SA and SA‐loaded composite hydrogels effect lymphedema via in vivo experiments. In the experiment, the untreated tail lymphedema model served as the negative control group, while the normal tail without any treatment was established as the blank control group. The pure drug (SA) solution without hydrogel, the L‐SA, and the D‐SA groups were also set. The primary objectives of the study are to ascertain if SA proves effective in treating lymphedema and whether there are any distinctions between the L‐SA and D‐SA groups.

**Figure 5:**
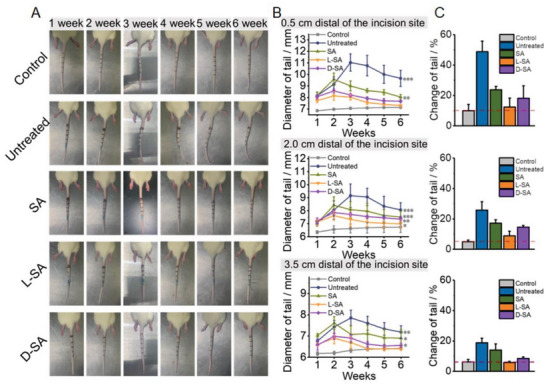
Effects of composite hydrogels on a rat tail lymphedema model. A) Representative photographs of the rat tail at 1, 2, 3, 4, 5, and 6 weeks after surgery with different treatments. B) Quantification of tail diameter change (n = 5). C) Changes in tail diameter after six weeks in different groups. The data are expressed as the mean ± SD, n = 5 animals per group. The P value is calculated by t‐test. *p < 0.05, **p < 0.01, and ***p < 0.001.

A rat‐tail lymphedema model was successfully established by ring‐resection of a 0.5‐mm‐wide skin layer and truncation of lymphatic vessels (Figure S26, Supplemental Information). Methylene blue was subcutaneously injected to locate lymphatic vessels and separate them from the blood vessels. The rats were subjected to different treatments the following day after surgery, and we measured the tail diameters at the +0.5 cm, +2 cm, and +3.5 cm sites every week. In the untreated group, the diameter of tails gradually increased over time and peaked in the third week, followed by slowly resolved. In addition to lymphedema, some stiff and curled tail shapes were also observed. On the contrary, in the SA, D‐SA, and L‐SA groups, the tail diameter increased at the beginning, but obviously decreased after week 2 as the repair effect became more and more apparent (**Figure** **5**A). Meanwhile, the wound at the surgical site gradually healed as the lymphedema subsided. Notably, although the tail diameter grew and then reduced after surgery due to the successful establishment of lymphedema, the degree of tail swelling was lower in the L‐SA group than in the other groups owing to enhanced SA penetration. And in the L‐SA group, the rat tail diameters from 0.5 cm to 3.5 cm distal of the incision site were basically restored to the level of the control group after 6 weeks, in agreement with the experiment in vitro (Figure 5B‐C). Of note, the farther away from the surgical site, the smaller the difference between each group.

### 2.6 Histological Analysis of Rat Tail Lymphedema

**Figure 6:**
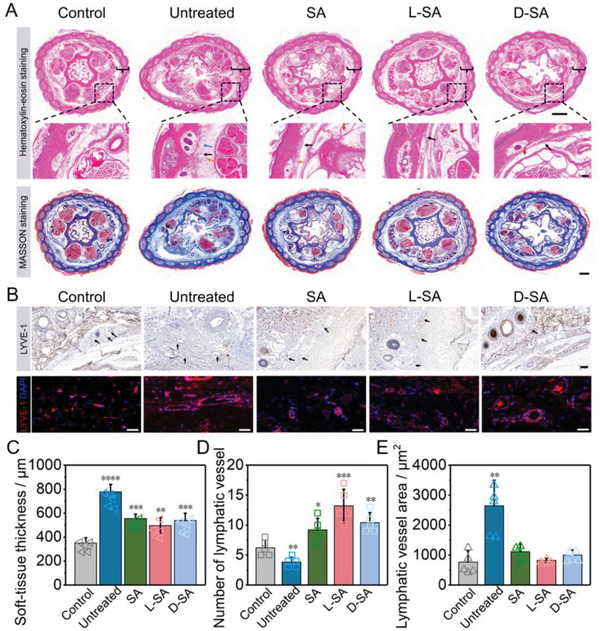
Pathological changes of tail. A) Representative H&E staining images of tail cross‐sections (scale bar = 500 µm) with brackets indicating fibroadipose tissue and with dotted boxes indicating local magnification (black arrow: inflammatory cell, blue arrow: edematous tissue, red arrow: regeneration vessel, orange arrow: granulation tissue, scale bar = 200 µm) and Masson staining of tail cross‐sections. Scale bar = 500 µm. B) Representative immunohistochemical and immunofluorescence images of tissue sections stained with LYVE‐1 in various groups. Scale bar = 100 µm. Statistical results of C) soft‐tissue thickness, D) lymphatic vessel count, and E) lymphatic vessel area (n = 5). The results are expressed as the mean ± SD, n = 5 per group. The P value is calculated by t‐test. *p < 0.05, **p < 0.01, ***p < 0.001, and ****p < 0.0001.

The rat tail tissue was collected at 6 weeks, and the proximal whole‐tail cross sections were taken for H&E and Masson staining analyses. As shown in **Figure** **6**A and Figure S27 (Supplemental Information), the dermal layer structure in the untreated group was disordered, and the thickness of the soft tissues was significantly increased, which was approximately 2‐fold that of that in the control group (Figure 6C). Tissue edema, hyperkeratosis, and hyperplasia were visible in the epidermis. Furthermore, massive cell infiltration and granulation tissue were observed in most regions, representing the inflammatory changes in these tissues. Whereas histopathological examination from the SA, L‐SA, and D‐SA groups indicated that the overall structure of the skin was nearly normal, and the organization of the dermis was complete and orderly along with neovascularization form. Following therapy, the lymphedema subsided, the skin thickness was markedly reduced, and no prominent infiltration of inflammatory cells was observed in the tissue. Among them, skin tissue from the L‐SA group was nearly identical to that of the control group. A statistical comparison showed that the thickness of soft tissue after treatment was smaller in the L‐SA group than in the D‐SA group and in the SA group, and the difference between the L‐SA group and the control group was minimal (Figure 6C). Then, the collagen deposition in subcutaneous tissue was evaluated by Masson staining. These findings revealed that the distribution of collagen fibers in the dermis was arranged in a dense and orderly manner, and the subcutaneous tissue was dispersed across the blue‐stained fiber region in the SA, D‐SA, and L‐SA groups (Figure 6A; Figure S27, Supplemental Information). However, in the untreated group, collagen fibers were deposited, and the percentage of fibrotic areas increased in comparison to the control group, indicating the development of subcutaneous fibrosis in the tails. Fibrosis is a histological hallmark of lymphedema, which may be caused by local inflammatory activation and plays an inhibiting role in relieving lymphedema.^[^ ^66^, ^67^ ^]^ Thus, the relative severity of fibrosis in the untreated group was also one of the important factors hindering the alleviation of lymphedema.

Next, lymphatic remodeling was qualitatively and quantitatively assessed using immunofluorescence and immunohistochemical staining for LYVE‐1 (lymphatic vessel endothelial hyaluronan receptor 1), which was a lymphangiogenesis marker.^[^ ^68^ ^]^ Formation of LYVE‐1‐positive lymphatic structures (red) was evident in all groups at 6 weeks after surgery (Figure S28, Supplemental Information). The results demonstrated a significant enlargement of lymphatic vessels in the dermis and subdermis in the untreated group as compared to the control group (Figure 6B; Figure S29, Supplemental Information). On the contrary, lymphatics returned substantially to normal with small and collapsed structures following SA, L‐SA, and D‐SA treatments. A quantitative examination of mean lymphatic vessel number and area revealed that the lymphatic vessel marker in the untreated group was lower than that of the other groups (Figure 6D‐E). However, the LYVE‐1‐positive lymphatic area in the untreated group was larger than those in other groups. After L‐SA therapy, the number of lymphatic vessels grew the highest; and the area of lymphatic vessels reduced in all treatment groups due to the remission effect, but the L‐SA group had the greatest decline. As a result, it was possible to hypothesize that the therapeutic effects of SA, L‐SA, and D‐SA groups on lymphedema were attained by reducing inflammatory responses, diminishing tissue fibrosis, and promoting lymphatic vessel regeneration. Moreover, a prominent therapeutic effect could be observed in the L‐SA group.

### 2.7 Mechanism of drug‐loaded Hydrogel Beneficial Effects Against Lymphedema

**Figure 7:**
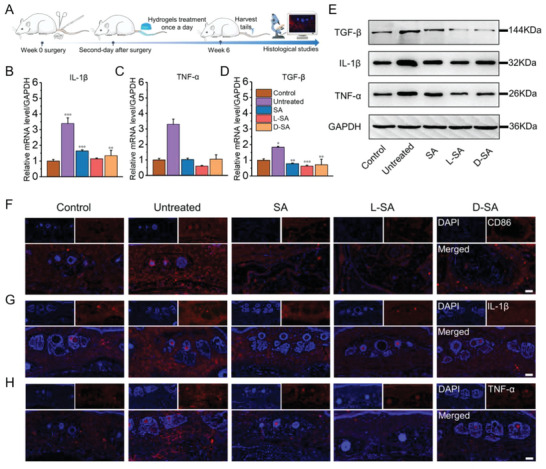
L/D‐SA hydrogels can relieve tail lymphedema. A) Schematic illustration of establishment of the rat tail lymphedema model and postoperative therapy. Real‐time PCR results of B) IL‐1β, C) TNF‐α, and D) TGF‐β (n = 3). E) The expression levels of inflammation‐related proteins (IL‐1β, TNF‐α, and TGF‐β) in the rat tail lymphedema analyzed by Western blotting (n = 3). The representative images in the tail lymphedema site after immunofluorescence labeling with F) CD86, G) IL‐1β, and H) TNF‐α. Scale bar = 100 µm. The results are expressed as the mean ± SD, n = 3 per group. The P value is calculated by t‐test. *p < 0.05, **p < 0.01, and ***p < 0.001.

Histologically, lymphedema is characterized by an altered abundance of immune cells and inflammatory responses, which are associated with the polarization of macrophages and a series of local cytokines released by macrophages. Among them, the inflammatory cytokines can be divided into pro‐inflammatory factors (e.g., TNF‐α, IL‐1β, IL‐6 (interleukin 6)) and anti‐inflammatory factors (e.g., IL‐10 (interleukin 10)), which regulate the inflammatory response in opposite manners.^[^ ^69^ ^]^ In order to explore the molecular mechanisms of drug‐loaded hydrogel's beneficial effects against lymphedema, the expression of those inflammatory cytokines was evaluated in week 6 (**Figure** **7**A). The expression of proinflammatory cytokines, as depicted in Figure 7B‐C, was higher in the untreated group than in the control group at week 6. While in the SA, L‐SA, and D‐SA groups, the expression of inflammatory factors showed a downward trend, indicating less severe inflammatory responses in these three groups. Notably, the relative mRNA expression of TNF‐α and IL‐6 in the L‐SA group was significantly lower compared with the control group. Furthermore, treatments with SA, L‐SA, and D‐SA also led to a marked reduction in the red fluorescence intensity compared with the untreated group (Figure 7F‐H; Figure S30, Supplemental Information). As an anti‐inflammatory factor, IL‐10 can antagonize the impacts of proinflammatory cytokines and inhibit inflammatory cells. As shown in Figure S31, the expression level of IL‐10 in the SA, L‐SA, and D‐SA groups was higher than that of the control group and the untreated group. The immunofluorescence staining further confirmed that IL‐10 showed enhanced red fluorescence in the SA, L‐SA, and D‐SA groups (Figure S32, Supplemental Information). These findings suggested that the SA, L‐SA, and D‐SA groups could be capable of downregulating the expression of pro‐inflammatory factors and upregulating the expression of anti‐inflammatory factors after the induction of lymphedema. TGF‐β (transforming growth factor‐beta) stimulates mesenchymal cells to differentiate into myofibroblasts, which aids in the secretion of collagen and the synthesis of extracellular matrix (ECM). However, an excessive release of TGF‐β may also result in a significant buildup of ECM and fibrosis.^[^ ^70^ ^]^ In this study, TGF‐β expression was found to be significantly elevated in the untreated group on the sixth week following a lymphatic operation (Figure 7D‐E). This was also consistent with the results of excessive collagen precipitation in Masson staining. By contrast, the secretion of TGF‐β in the SA, L‐SA, and D‐SA groups showed a decreasing trend, with the L‐SA group experiencing the greatest reduction, even lower than that in the control group, which could be attributed to the antifibrotic effect of IL‐10. Such a difference in the TGF‐β levels may contribute to reducing the development of fibrosis.

Lymphedema induces inflammation by causing pro‐inflammatory cells to become activated. Increased pro‐inflammatory cell infiltration surrounding the surgical site prolongs inflammation, inhibits lymphangiogenesis, and perpetuates lymphedema.^[^ ^71^ ^]^ The study demonstrated for the first time that SA and SA‐loaded hydrogels could improve lymphedema, which was achieved by dynamically modulating the level of inflammatory cytokines, thus help alleviating the fibrosis, oedema or abnormal fat deposition caused by inflammation. Within the treatment groups, outstanding results were observed in the L‐SA group. The most noticeable effects of L‐SA hydrogel administration were the near‐normalization of tissue structure and resolution of inflammatory responses, which were associated with the better drug penetration promoted by the LPFEG‐based hydrogel at the same duration.

## 3 Conclusion

In conclusion, the co‐assemblies of SA and chiral supramolecular gelators offer a promising approach to improve the stability and therapeutic efficacy of SA for the treatment of lymphedema. In vitro studies reveal that SA and SA‐loaded composite hydrogels can regulate local immunological status by reducing the M1 polarization of macrophages, which has beneficial effects for lymphedema progression. Notably, these chiral supramolecular hydrogels achieve superior transdermal delivery of SA compared to the SA solution, with the L‐SA treatment showing outstanding results due to the high enantioselectivity between L‐type chiral gelators and stratum corneum. In vivo studies further demonstrate the efficacy of these hydrogels in reducing fibrosis and edema caused by inflammation and promoting lymphatic vessel regeneration. Collectively, these findings demonstrate for the first time that SA can be considered as a potential candidate for lymphedema therapy. In addition, an effective chiral biomaterial platform is being developed to enhance percutaneous delivery and enable SA to play a more active role in the treatment of lymphedema. Furthermore, this study emphasizes the importance of stereoselective effects between chiral hydrogels and stratum corneum for improved medication penetration. Therefore, these kinds of drug‐loaded chiral supramolecular hydrogels provide significant insights for the development of new formulations to improve lymphedema treatment, which has great practical significance and can be explored for the non‐invasive treatment of other diseases.

## 4 Experimental Section

### Materials

All material can be found in supplementary information.

### Synthesis of L/DPFEG Gelator

The detailed procedures for the synthesis of L/DPFEG gelators are available in supplementary information.

### Preparation of L/D‐SA Hydrogels

SA was uniformly dispersed in deionized (DI) water and diluted from 40 mmol mL^−1^ to 10 mmol mL^−1^ for further use. Then, L/DPFEG gelators (3 mg mL^−1^, abbreviated as 3% L/D) were mixed with different concentrations of SA solutions to form hydrogels by heating‐cooling method. Similarly, the concentration of SA aqueous solution was fixed and 3% L/D‐SA, 5% L/D‐SA and 10% L/D‐SA hydrogel was prepared by changing the concentration of L/DPFEG gelators. The pure L/DPFEG hydrogels were constructed by the same approach.

### Characterization of L/D‐SA Hydrogels

The surface morphology of L/DPFEG hydrogels and L/D‐SA composite hydrogels were investigated by field emission scanning electron microscope (FM‐SEM, FEI Quanta 250). Before the measurement, the specimens were diluted and dripped onto the silicon wafer. After drying, Au plating was sputter coated on the surface of samples.

Circular dichroism (CD) spectroscopy was utilized to determine the optical activity and secondary features of substances. The experiment was performed on a CD spectrometer (JASCO J‐1500) in the ultraviolet range of 190–500 nm using a quartz colorimetric dish (optical diameter: 0.2 mm) at N_2_ atmosphere.

The crystal structure of the SA and composite hydrogel was investigated by X‐ray diffraction (XRD, Mini Flex 600, Japan). SA, L‐SA, and D‐SA freeze‐dried powders were respectively filled into the dishes of the X‐ray diffractometer, using CuKα radiation source, 40 kV voltage and 15 mA current, scanning at a rate of 10° min^−1^ within the 2θ range of 5°−60°.

SA, L‐SA, and D‐SA freeze‐dried powders were placed in aluminum crucibles of differential scanning calorimeter (DSC) (DSC 2500, America) in a temperature range of 30–230 °C at a 10 °C min^−1^ heating rate.

### Cell Cultures

Mouse L929 fibroblasts were purchased from Procell (Procell Life Science & Technology Co., Ltd., Wuhan). Mouse Raw 264.7 macrophages were purchased from Procell. L929 cells were cultured in MEM (Gibco, 10% FBS, 1% NEAA, and 1% penicillin‐streptomycin) and Raw 264.7 cells were cultured in α‐MEM (Gibco, 5% FBS, 1% penicillin‐streptomycin) and placed in an incubator (37°C, 5% CO_2_).

### In vitro Cytotoxicity Study

The L929 fibroblasts were seeded into 96‐well plates (2×10^4^ cells per well) and incubated for 24 h. Various concentrations of L/D‐SA hydrogels and equal concentrations of SA solutions were co‐cultured with cells for 1, 3 and 5 day, respectively. Subsequently, CCK‐8 assay was performed on cells and the absorbance at 450 nm was examined using a microplate reader (TECAN Infinite 200 PRO). The relative cell viability (%) was calculated by Equation (1) in supplementary information.

The live/dead staining tests were carried out in 24‐well plates with 1×10^5^ cells per well. After being cultured for 3 days, the cells were stained with a solution of AM/PI (2 µM/4 µM) for 40 min. For analysis of cellular morphology, cells were first seeded onto 12‐well plates at a density of 1×10^5^ cells per well and co‐cultured with various groups of hydrogels. Following that, the cells were fixed with 4% paraformaldehyde (PFA) for 30 min and stained with Phalloidin‐iFluor 488 conjugate according to the instructions. The results of live/dead staining and cell morphology were able to be visualized by a fluorescence microscope (Olympus IX73).

### Effect of the SA and the L/D‐SA Hydrogels on Macrophages

RAW264.7 cells were treated with SA, L‐SA, and D‐SA to study the in vitro impact of the drug on macrophages. When they reached 80% confluence, the cells were scraped, dissociated, and counted, and then plated in 6‐well plates at a concentration of 2×10^5^ mL^−1^ (in RAW264.7 cells). When reaching 60% confluence, cells were stimulated by 100 ng mL^−1^ P.g.‐LPS (InvivoGen, San Diego, CA, USA) with or without different forms of SA.

### Flow Cytometry

The surface markers of stimulated cells were detected by flow cytometry. Briefly, RAW264.7 cells were collected from 6‐well plates after stimulation for 24 h and washed three times with PBS. The cell suspension respectively containing 1×10^6^ M0‐unpolarized or M1‐polarized cells was then divided into 1.5 ml EP tubes and incubated with blocking antibody CD16/32 (Biolegend, San Diego, CA, USA) on ice for 10 min. After being washed twice, cells were incubated in PBS plus Intrapore Permeabilization Reagent with the following antibodies (FITC anti‐mouse CD86) (from Biolegend) on ice for 30 min in the dark. After being washed twice, cells were suspended in 500 µl PBS with 3% FBS and then detected by flow cytometry (BD Biosciences, San Diego, CA, USA).

### Enzyme‐linked Immunosorbent Assay (ELISA)

Cell supernatant was collected from RAW264.7 cells, centrifuged at 12,000 rpm at 4°C for 10 min and the concentrations of TNF‐α, IL‐1β were measured with ELISA kits (Anogen, Canada). All samples were assayed in triplicate and measured at a 450 nm wavelength.

### Isolation of Rat Tail Skins

Rats weighing between 150 and 250 g were chosen, sacrificed, and their tail skin was promptly separated. Spread the skin on a clean glass plate, let the stratum corneum downward, with a knife or other blunt instrument dipping physiological saline to remove subcutaneous fat, tissue and capillaries. Then rinsed with saline repeatedly, wiped dry, wrapped in aluminum foil and stored in the fridge for later use.

### Skin Penetration Test

The transdermal permeation study was carried out by the vertical Franz diffusion cell system (Shanghai Kaikai Technology Trading Co., Ltd., China) with a diffusional area of 1.766 cm^2^ and a receptor chamber volume of 7 mL. The excised tail skin of rats was placed between donor and acceptor chambers of the system with the stratum corneum facing the donor compartment. 2 mL portion of different hydrogels and equal concentration SA solutions were deposited onto the surface of skin in the donor chamber. The acceptor chamber was filled with phosphate‐buffered saline (PBS) and constantly stirred in a water bath thermostatically controlled at 37°C. At specified time intervals, the acceptor solution (1 mL) was collected and an equal volume of PBS was replenished, which was then measured with an ultraviolet‐visible spectrophotometer (Evolution 201 Thermo Fisher Scientific, USA) at 485 nm. The cumulative percentage of the SA permeated across the skin (Q_n_) was calculated according to the Equation (2) in supplementary information.

### In vitro Assays on Isolated Rat Tail Skins

The change morphology of rat tail skin treated with different hydrogels was characterized by FM‐SEM. The skin was collected after 4 h of treatment, rinsed with normal saline repeatedly, fixed with 2.5% glutaraldehyde, dehydrated with a series of ethanol gradients, and dried at the critical point for CO_2_. Then, Au plating was sputtered onto the surface of samples and photographed.

The infrared spectrum of the rat tail skin before and after treatment was obtained by using Attenuated Total Reflection Fourier transform spectrometer (ATR‐FTIR, Bruck EQUINOX55). Excised skin was incubated in 0.25% trypsin solution for 24 hours at 37°C. The stratum corneum was separated with cotton swabs, washed with DI water, and dried with filter paper. Then, the stratum corneum was immersed in different hydrogels or equal concentration of SA aqueous solution and was stirred at a constant speed of 400 r min^−1^ for 6 h. After that, the sample was washed with DI water, sucked with filter paper, and placed in a vacuum dryer for detection.

The resistance value was performed on the isolated stratum corneum, which was immersed in various hydrogels after 1 and 6 hours. Then, the sample was washed with DI water, dried with filter paper, and detected by the multimeter.

The hydrogel‐treated or SA‐treated rat tail skin was fixed in 4% PFA, stained with DAPI and cryo‐sectioned at 10‐µm thickness. The fluorescence images of samples were obtained using a fluorescence microscope. The two‐dimensional (2D) fluorescence images were converted into three‐dimensional (3D) reconstructed images by the fluorescent intensity‐based surface plotting using Image J Software.

### Animals

SD rats (male, 151–200 g) were purchased from SLAC Laboratory Animal Co.,Ltd (Shanghai, China). All animal trials were performed following protocols approved by the Ethical Committee for Animal Experiments of Shanghai Jiao Tong University (China, A2021146).

### Establishment of Rat Tail Lymphedema Model

Post‐surgical lymphedema was experimentally created in the tails of rats (SD, male, 151–200 g). Prior and during surgeries, rats were anesthetized with isofluorane inhalation anesthetic and their vital signs were closely monitored. For each intervention, a 0.5 cm circumferential incision was created at the proximal end after measuring the tail from tip to body base at 13 cm. The main lymphatic trunks were identified via subcutaneous injection of methylene blue distal to the incision site, followed by truncation and ligation. At the end of the procedure, sterile gauze was covered around the incision to keep it clean, and gauze was taken off 24 h later. The rats were randomly divided into five groups (n = 5) and subjected to different treatments the following day after surgery.

### Lymphedema Rat Tail Evaluation

The diameter of the tail in the lymphedema rat tail model was measured with a Vernier caliper (n = 5). Measurements were taken weekly following surgery for 6 weeks.

### Quantitative real‐time PCR Measurement

Rat tails were collected from the surgical site on week 6 and then homogenized to extract the total RNA of tissue with Trizol reagent (Solarbio, China) according to the manufacturer's instructions. The total extracted RNA was transformed into cDNA using TransScript cDNA synthesis kit (TransGen Biotech, China). Finally, the real‐time PCR was performed on ABI QuantStudio 1 Real‐Time RCR system (Thermofisher, USA) for gene expression analysis. Primer sequence of glyceraldehyde 3‐phosphate dehydrogenase (GAPDH, housekeeping gene), Tumor necrosis factor‐alpha (TNF‐α), interleukin‐6 (IL‐6), interleukin 1 beta (IL‐1β), interleukin‐10 (IL‐10), and transforming growth factor‐β (TGF‐β) are listed in Table S1 (Supplementary Information). The relative gene expression was calculated by −2^ΔΔCt^ method.

### Western Blotting Assay

According to the manufacturer's instructions, total proteins were extracted from Raw 264.7 macrophages using RIPA lysis buffer (Bryotime, China) and quantified using the BCA kit (Bryotime, China). Protein samples were then separated by SDS‐PAGE (sodium dodecyl sulfate‐polyacrylamide gel electrophoresis) and transferred to polyvinylidene fluoride (PVDF) membranes (Millipore, USA). After the membranes were blocked with 5% non‐fat dried milk for 2 h at RT (room temperature), they were incubated with particular primary antibodies overnight at 4°C. Subsequently, the PVDF membranes were treated with corresponding secondary antibodies for 1 h at RT. The membrane was washed again with TBST and was imaged by a gel imaging system (Fusion FX7, France).

The rat tails on the surgical site on week 6 after surgery were collected and were shipped in dry ice. Tissues were washed 2–3 times with cold TBS to remove blood stains, then cut into small pieces and placed in a homogenizer. RIPA lysate was added and the mixture is thoroughly homogenized on ice. After centrifugation at 12 000 g for 5 min, the supernatant was collected and the total protein content was quantified using a BCA assay kit. Equal amount of extracted total proteins were separated using gel electrophoresis and transferred to a polyvinylidene difluoride (PVDF) membrane. The membrane containing proteins was incubated with primary antibodies at 4°C overnight and a secondary antibody at room temperature for 30 min. The membrane was washed again with TBST and was imaged by a gel imaging system. GAPDH was used as control. The categories of all reagent used in this study are shown in Table S2 (Supplementary Information).

### Histological Analysis

The rat tails were harvested 1.5 cm distal to the surgical site on week 6. The samples were fixed with 4% PFA and were decalcified with 10% ethylenediaminetetraacetic acid (EDTA) to prepare histological sections. The sections were further stained with hematoxylin and eosin (H&E) for pathological analysis and Masson staining for collagen deposition. Based on H&E images, the soft‐tissue thickness was quantified by Image J.

For immunofluorescence and immumohistochemical staining, the sections were respectively incubated with antibodies to IL‐6, TNF‐α, IL‐1β, IL‐10 and LYVE‐1 (Lymphatic Vessel Endothelial Hyaluronic Acid Receptor 1) according to a standard procedure (Thermo, USA). The stained slides were photographed using a fluorescence microscope (80i, Nikon).

### Statistical Analysis

All data values were presented as the mean ± standard deviation (SD). The between‐group differences in test were analyzed by t‐test (2‐tailed); and comparisons between more than two groups were performed by one‐way analysis of variance (ANOVA) using Prism (Version 6, GraphPad Software), where the statistical significance was assigned as *p < 0.05, **p < 0.01, ***p < 0.001, and ****p < 0.0001, respectively. The figure legends included a complete list of the experiments' statistical information.

## Conflict of Interest

The authors declare no conflict of interest.

## Supporting information

Supporting Information

Supporting Information

## Data Availability

The data that support the findings of this study are available from the corresponding author upon reasonable request.
