# Supplementary material for: Chiral Supramolecular Hydrogel Enhanced Transdermal Delivery of Sodium Aescinate to Modulate M1 Macrophage Polarization Against Lymphedema
Source: Adv Sci (Weinh). 2023 Dec 1;11(5):2303495. doi: 10.1002/advs.202303495 (PMC10837362; doi:10.1002/advs.202303495)
Supplement: Supplementary file 1 — Supporting Information [file ADVS-11-2303495-s001.pdf]

ADVANCED SCIENCE
Open Access

Supporting Information

for *Adv. Sci.*, DOI 10.1002/advs.202303495

Chiral Supramolecular Hydrogel Enhanced Transdermal Delivery of Sodium Aescinate to Modulate M1 Macrophage Polarization Against Lymphedema

Xueqian Wang, Chunxiao Cui, Xinxian Meng, Chengyao Han, Beibei Wu, Xiaoqiu Dou, Changli Zhao, Yixin Zhang, Ke Li* and Chuanliang Feng**

Supporting information

Chiral supramolecular hydrogel enhanced transdermal delivery of sodium aescinate to modulate M1 macrophage polarization against lymphedema

Xueqian Wang[1#], Chunxiao Cui[2#], Xinxian Meng[3#], Chengyao Han[3], Beibei Wu[1], Xiaoqiu Dou[1], Changli Zhao[1], Yixin Zhang[3], Ke Li[3]* and Chuanliang Feng[1]**

#The first three authors equally contributed to this work.

[1]State Key Lab of Metal Matrix Composites, Shanghai Key Laboratory for Molecular Engineering of Chiral Drugs, School of Materials Science and Engineering, Shanghai Jiao Tong University, Shanghai 200240, China

[2] Department of Facial Plastic and Reconstructive Surgery, ENT Institute, Eye and ENT Hospital,Fudan University, Shanghai, China

[3] Department of Plastic and Reconstructive Surgery, Shanghai Ninth People's Hospital, Shanghai JiaoTong University School of Medicine, Shanghai, China

*Corresponding author

CHUANLIANG FENG

E-mail: clfeng@sjtu.edu.cn

YIXIN ZHANG, M.D.

Professor, Chief，Division of Reconstructive Microsurgery, Vice Chief Department of Plastic and Reconstructive Surgery, Shanghai Ninth People's Hospital Shanghai Jiao Tong University School of Medicine (China)

Phone number: +86-21-23271699 Mobile phone number: +86 130-6177-5858

Fax number: +86-21-63051858 E-mail address: zhangyixin6688@hotmail.com

Ke Li, M.D.

Department of Plastic and Reconstructive Surgery, Shanghai Ninth People's Hospital

Shanghai Jiao Tong University School of Medicine (China).

E-mail address: 18817821624@163.com

1. Experimental section

1.1. Materials

Sodium aescinate (SA, Escin Sodium Salt) were purchased from Aladdin Chemistry (Shanghai) Co., Ltd.; Terephthaloyl Chloride, D/L-Phenylalanine Methyl Ester Hydrochloride, were purchased from Sigma-Aldrich Company; Ethanol (C_2H_5OH), Methanol (CH_3 OH), Diethylene Glycol ($C_4H_{10}O_3$), Dichloromethane (CH_2Cl_2), Triethylamine ($C_6H_{15}N$) and Ammonia were bought from Macklin Biochemical (Shanghai) Co., Ltd. All aqueous solutions were prepared using ultrapure water (18 MU) from a Milli-Q system (Millipore). Calcein-AM/propidium iodide (PI), Hoechst 33258, Dojindo's cell counting kit-8 (CCK-8), and Trypsin (0.25%) were bought from Shanghai Shaoxin Biotechnology Co., Ltd.; Fetal bovine serum (FBS), MEM (Minimum Essential Medium), NEAA (non-essential amino acids), phosphate-buffered saline (PBS), normal saline, 4% paraformaldehyde, and glutaraldehyde (2.5%) were purchased from Thermo Fisher Scientific (China) Co., Ltd.; Phalloidin-iFluor 488 conjugate was bought from ATT Bioquest; Ceramides mixture was obtained from Target Molecule Corp.; Human Ceramide ELISA Kit was

bought from Shanghai TITAN Technology Co., Ltd.; L929 fibroblasts were obtained from ATCC. All of the chemicals were used as received without further purification.

1.2. Synthesis of D/LPFEG gelator

L/D-phenylalanine chiral gelator (L/DPFEG) was synthesized based on a previous synthetic method [1]. Under magnetic stirring, terephthaloyl chloride (2.6 g, 13.0 mmol) was dissolved in DCM (20 mL) and slowly added to a combination containing L-phenylalanine methyl ester hydrochloride (100 mL, 6.0 g, 26.2 mmol) and triethylamine (8.0 mL, 58.4 mmol). Rotavapor was used to concentrate the mixture after it had been stirred overnight to remove the surplus solvent. The insoluble materials p-Ph(D-Phe-OMe)$_2$ was obtained after the residual substance in the flask was dissolved in ethanol and filtered and dried. To obtain a clear solution, the intermediate product (5.1 g, 10.5 mmol, 85%) was dissolved in methanol and NaOH aqueous solution (15 mL) was added under agitation for 24 hours. The pH of the solution was then adjusted with HCl (3.0 M) to achieve gel precipitation. Then, the gel was filtered and washed multiple times with DI water, and the p-Ph(D-Phe-OH)$_2$ (4.6 g, 9.9 mmol, 90%) was obtained after vacuum drying. Overall yield of DPF: 76.5%. Similarly, LPF was obtained as a white solid after freeze-drying (4.2 g, 82%).

The cleared solution was put into the frozen water after reacting at 130 °C for 3.5 hours, resulting in gel precipitation. Finally, the sediment was filtered and rinsed with DI water multiple times before being baked to get DPFEG gelator (4.2 g, 6.6 mmol, 91%). LPFEG gelator (3.6 g, 5.6 mmol, 85%) was synthesized in the same way.

1.3. In vitro cytotoxicity study.

Mouse fibroblasts (L929) are used to evaluate the biotoxicity of the materials. The cytotoxicity of the materials to L929 cells was detected by CCK-8 method and Live/Dead staining after culture within the specified time. The relative cell viability was calculated as following Equation (1):

Cell viability (%) = [(As-Ab) / (Ac-Ab)] × 100%   Equation (1)

Where As is the absorbance of experimental well, containing cell culture medium, CCK-8 and the substance to be tested. Ab is a blank well, without cell culture medium, CCK-8 and substance. Ac is the control group, containing cell culture medium and CCK-8, without substance to be tested.

1.4. Skin penetration test

The cumulative percentage of the SA permeated across the skin (Qn) was calculated according to the Equation (2):

$$Q_n\ (\%) = \frac{VC_n + \sum_{i=1}^{n-1} C_i V_i}{A} \times 100 \qquad \text{Equation (2)}$$

where Q_n is the cumulative percentage of SA permeated across the skin, V is the volume of the medium in the acceptor chamber (7 mL), V_i is the volume of the medium sampled at various time points (1 mL), C_n is the concentration of SA in the medium in the acceptor chamber at various time points, C_i is the concentration of SA in the medium in the acceptor chamber at the *i*th (n − 1) time point, and A is the feeding quantity of the SA.

1.5. Quantitative real-time PCR measurement

Primer sequence of glyceraldehyde 3-phosphate dehydrogenase (GAPDH, housekeeping gene), Tumor necrosis factor-alpha (TNF-α), interleukin-6 (IL-6),

interleukin 1 beta (IL-1β), interleukin-10 (IL-10), and transforming growth factor-β (TGF-β) are listed below.

Table S1 Primers used for real-time PCR

Target gene	Primer sequences (5′-3′)	
GAPDH	Forward	ACTCTACCCACGGCAAGTTC
	Reverse	TGGGTTTCCCGTTGATGA CC
TNF-α	Forward	AAATGGGCTCCCTCTATCAGTTC
	Reverse	TCTGCTTGGTGGTTTGCTACGAC
IL-6	Forward	ACCCCAACTTCCAATGCTCT
	Reverse	GGTTTGCCGAGTAGACCTCA
IL-1β	Forward	ACTATGGCAACTGTCCCTGAAC
	Reverse	GTGCTTGGGTCCTCATCCTG
IL-10	Forward	AATTGAACCACCCGGCATCT
	Reverse	TTTCCAAGGAGTTGCTCCCG
TGF-β	Forward	AGTGCTGAGGAGAAACCGTG
	Reverse	TTTGTGCATCGGCTGAAAGC

1.6. Western blotting assay

The categories of all reagent used in western blotting assay are shown in Table S2.

Table S2 Reagent used in western blotting

Reagent	Company	Cat.ID
RIPA Lysis Buffer	Bryotime	P0013B
PMSF（100mM）	Biosharp	BL507A

Phosphorylated Protease Inhibitor	Bryotime	P1081
BCA protein assay kit	Bryotime	P0012
SDS-PAGE loading buffer (5×)	Bryotime	P0015
SDS-PAGE Gel Kit	Biosharp	BL508A
Protein Marker	Therm（Fermentas）	26616
TRIS	BIOFROXX	1115GR500
Glycine	BIOFROXX	1275KG2P5
SDS	BIOFROXX	3250GR500
BSA	Roche	G5001
TWEEN 20	Solarbio	T8220
ECL	Biosharp	BL520A
CD86	Proteintech	13395-1-AP
TGF-β	Bioss	BSM-33287
IL-1β	Bioss	BS-0812R
IL-6	Boster	BA4339
TNF-α	Proteintech	60291-Ig
IL-10	Proteintech	60269-Ig
GAPDH	HUABIO	ET1601-4
HRP, Goat Anti-Rabbit IgG	Jackson	111-035-003
HRP, Rabbit Anti-Goat IgG	Jackson	305-035-003
HRP, Goat Anti-Mouse IgG	Jackson	115-035-003
HRP, Goat Anti-Rat IgG	Jackson	112-035-003

2. Additional Experimental Data and Figures

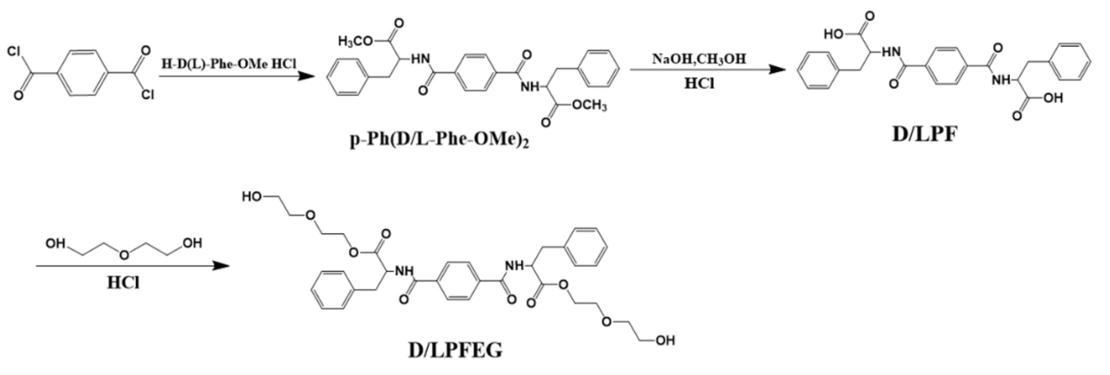

Scheme S1. Synthesis procedures of D/LPFEG. D/LPFEG was synthesized by H-D-Phe-OMe and H-L-Phe-OMe, respectively [1].

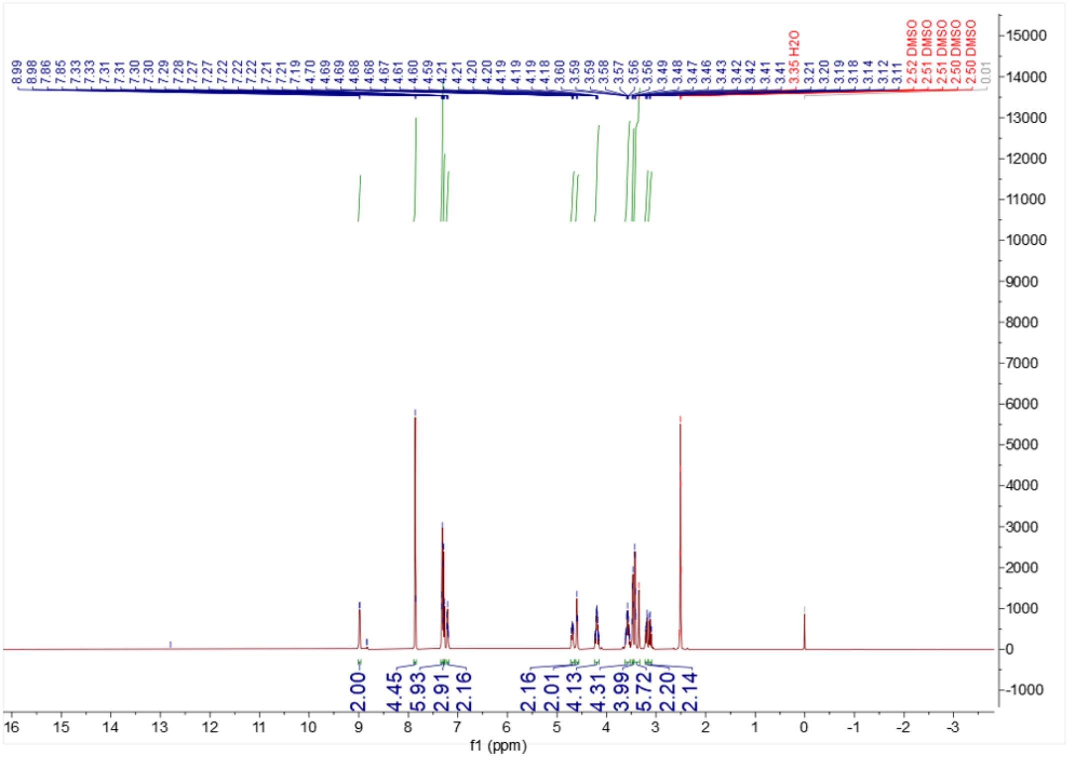

Figure S1. ^{1}H-NMR spectrum of LPFEG in DMSO-*d*6.

^{1}H NMR (500 MHz, DMSO-*d6*) δ 8.98 (d, J = 7.8 Hz, 2H), 7.86 (s, 4H), 7.34 – 7.29 (m, 6H), 7.28 (d, J = 7.9 Hz, 3H), 7.23 – 7.18 (m, 2H), 4.69 (ddd, J = 10.1, 7.8, 5.3 Hz, 2H), 4.60 (t, J = 5.4 Hz, 2H), 4.20 (qdd, J = 11.9, 5.9, 3.7 Hz, 4H), 3.63 – 3.53 (m, 4H), 3.47 (q, J = 5.3 Hz, 4H), 3.42 (dd, J = 5.5, 3.9 Hz, 6H), 3.19 (dd, J = 13.8, 5.3 Hz, 2H), 3.12 (dd, J = 13.8, 10.0 Hz, 2H).

Acquisition Parameter

Source Type	ESI	Ion Polarity	Positive	Set Nebulizer	2.0 Bar
Focus	Active	Set Capillary	4500 V	Set Dry Heater	220 °C
Scan Begin	50 m/z	Set End Plate Offset	-500 V	Set Dry Gas	8.0 l/min
Scan End	1300 m/z	Set Charging Voltage	2000 V	Set Divert Valve	Waste
		Set Corona	0 nA	Set APCI Heater	0 °C

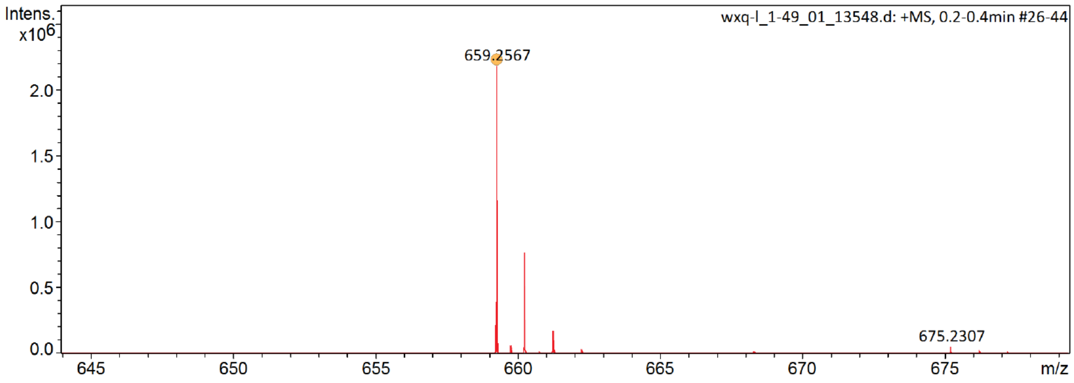

Figure S2. EI-HRMS spectrum of LPFEG [2].

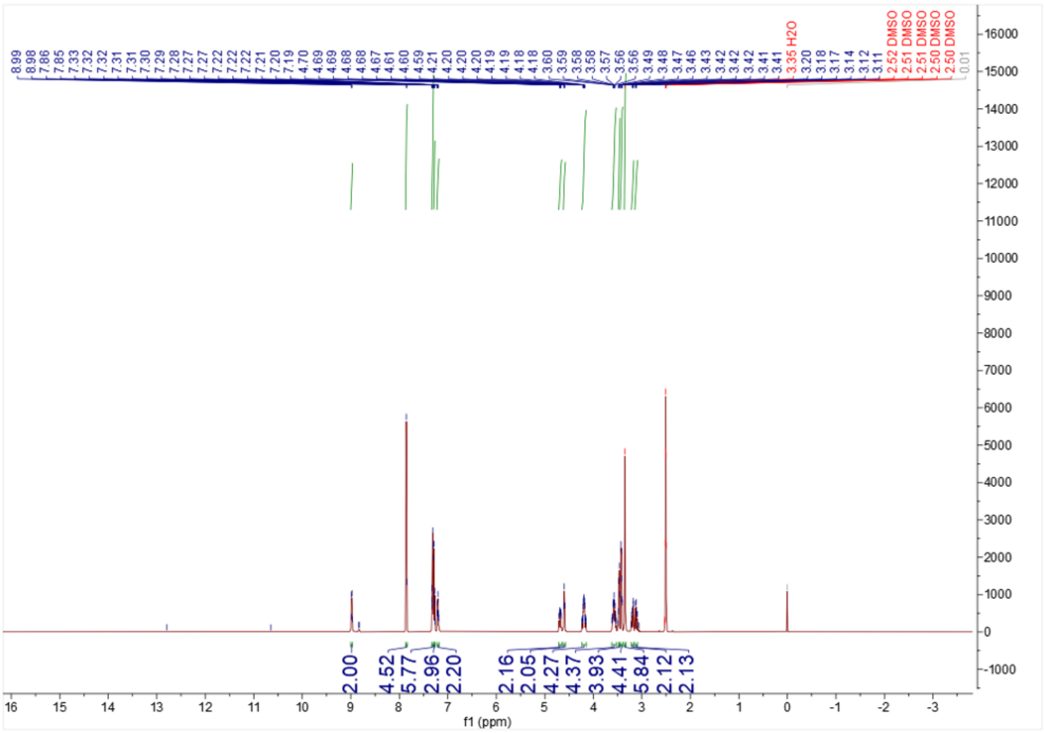

Figure S3. ^{1}H-NMR spectrum of DPFEG in DMSO-*d*6.

^{1}H NMR (500 MHz, DMSO-*d6*) δ 8.98 (d, J = 7.8 Hz, 2H), 7.85 (d, J = 3.4 Hz, 5H), 7.33 – 7.29 (m, 6H), 7.28 (d, J = 7.8 Hz, 3H), 7.23 – 7.18 (m, 2H), 4.68 (ddd, J = 10.0, 7.8, 5.4 Hz, 2H), 4.60 (t, J = 5.4 Hz, 2H), 4.19 (qdd, J = 12.0, 5.9, 3.7 Hz, 4H), 3.58 (tdd, J = 11.5, 8.7, 4.8 Hz, 4H), 3.47 (t, J = 5.3 Hz, 4H), 3.42 (dd, J = 5.5, 4.0 Hz, 4H), 3.19 (dd, J = 13.9, 5.3 Hz, 2H), 3.12 (dd, J = 13.8, 10.0 Hz, 2H).

Acquisition Parameter

Source Type	ESI	Ion Polarity	Positive	Set Nebulizer	2.0 Bar
Focus	Active	Set Capillary	4500 V	Set Dry Heater	220 °C
Scan Begin	50 m/z	Set End Plate Offset	-500 V	Set Dry Gas	8.0 l/min
Scan End	1300 m/z	Set Charging Voltage	2000 V	Set Divert Valve	Waste
		Set Corona	0 nA	Set APCI Heater	0 °C

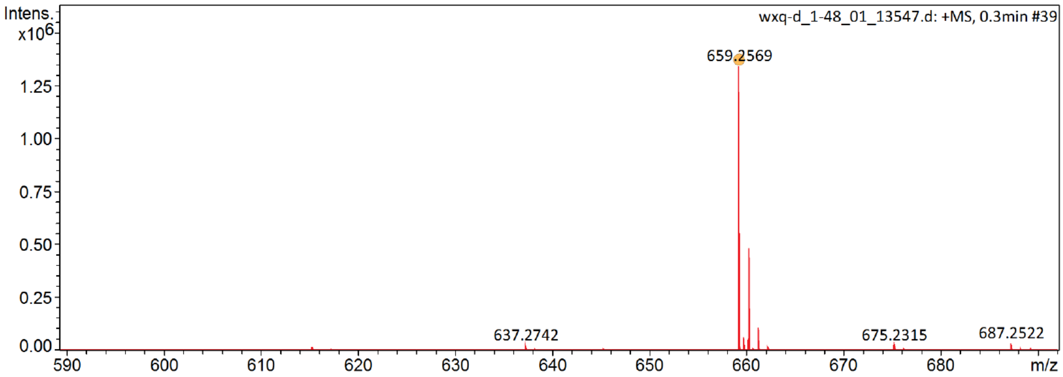

Figure S4. EI-HRMS spectrum of DPFEG.

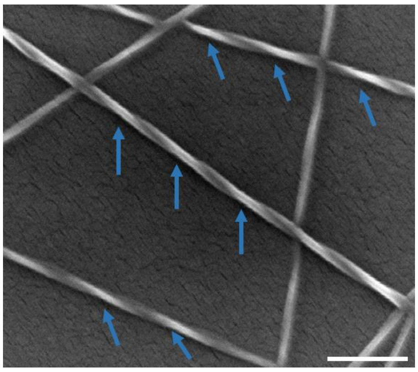

Figure S5. SEM image of DPFEG. Scale bar: 500 nm.

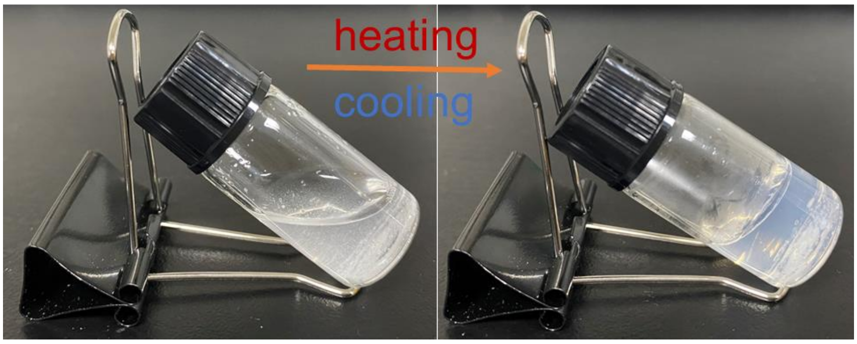

Figure S6. Photographs of D-SA before and after the heating/cooling method.

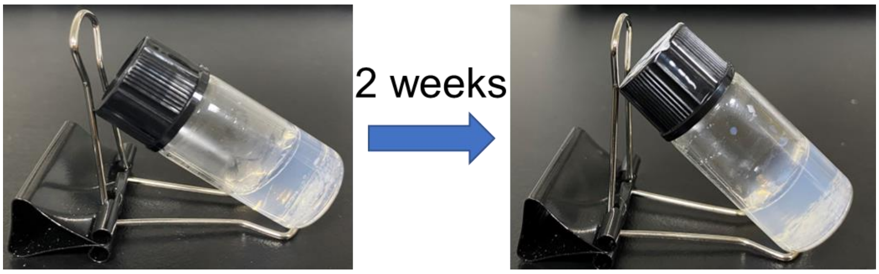

Figure S7. The stability of L-SA hydrogel after 2 weeks.

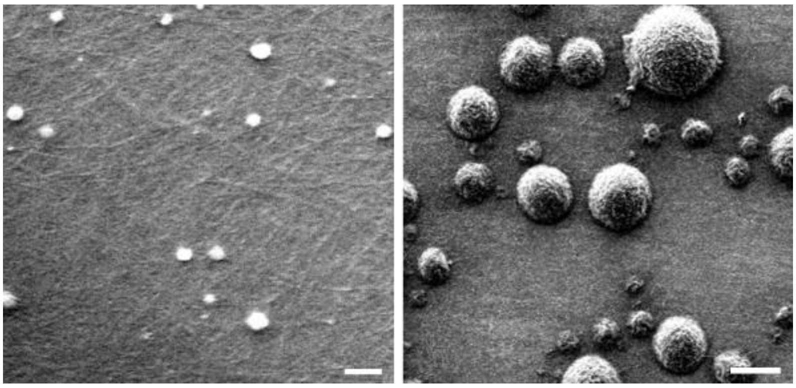

Figure S8. SEM of D-SA hydrogel. Scale bar 20 μm.

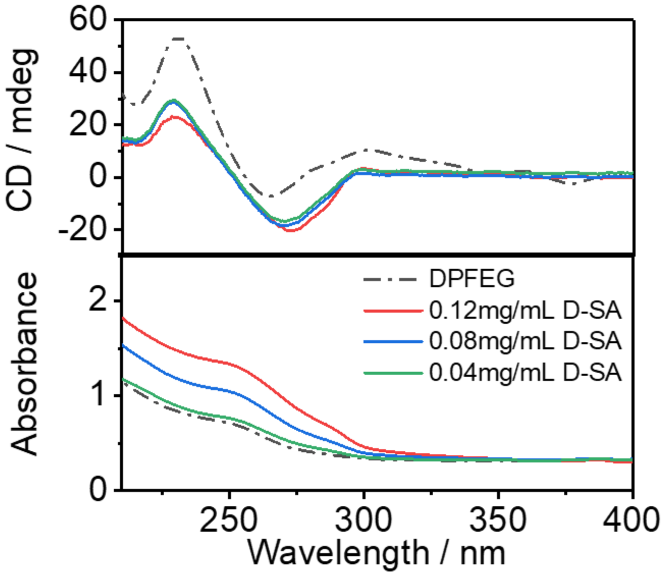

Figure S9. CD and corresponding UV-vis spectra of pure DPFEG and D-SA with different SA concentration.

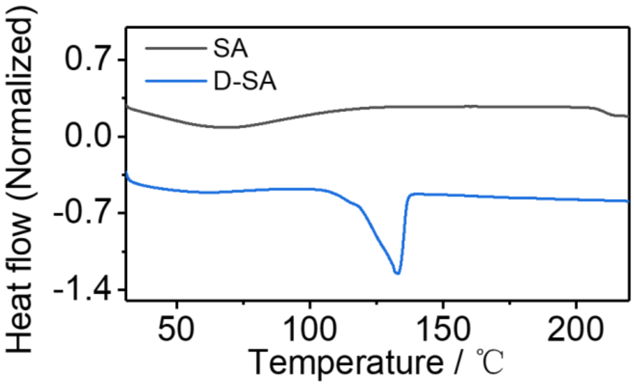

Figure S10. DSC thermograms of SA and D-SA.

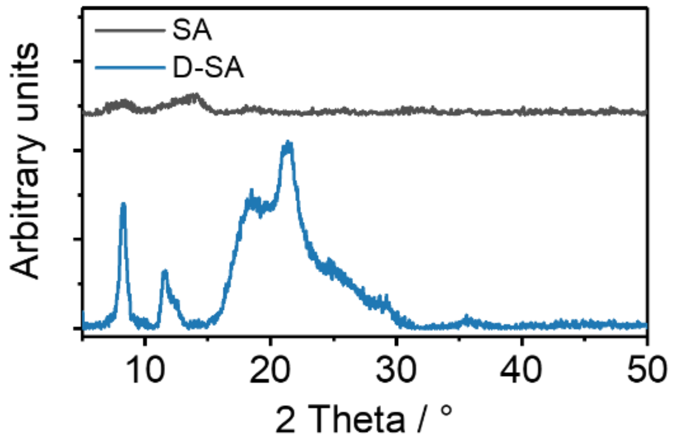

Figure S11. XRD patterns of SA and D-SA.

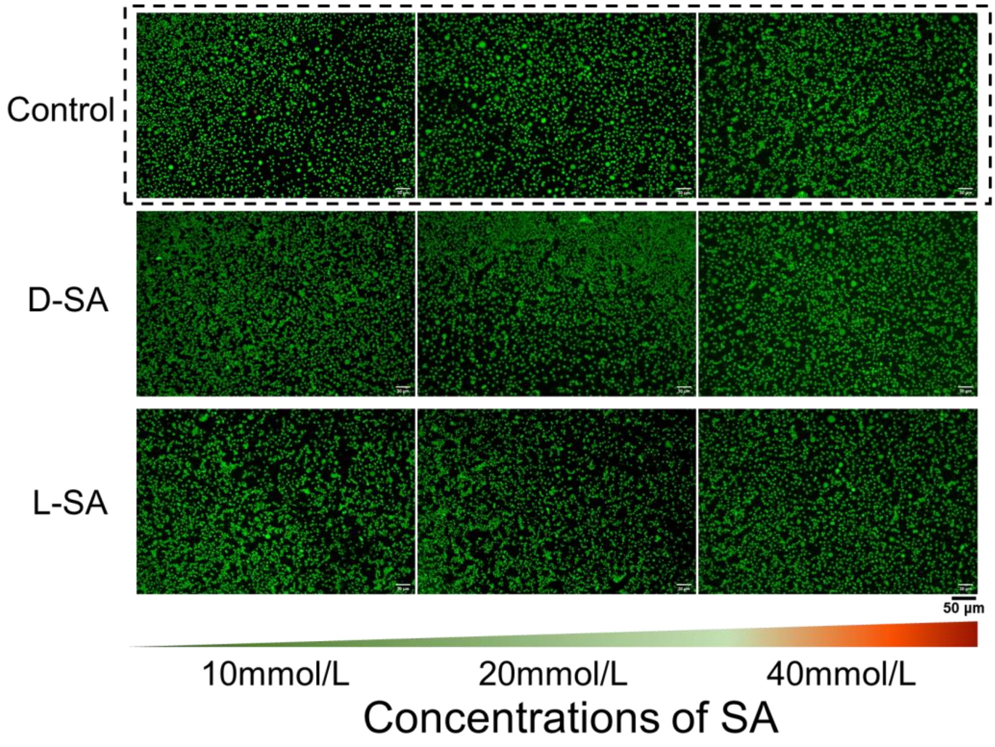

Figure S12. Fluorescence images of a live/dead assay of L929 cells cultured for 3 days with different SA composition hydrogels. Scale bar: 50 μm.

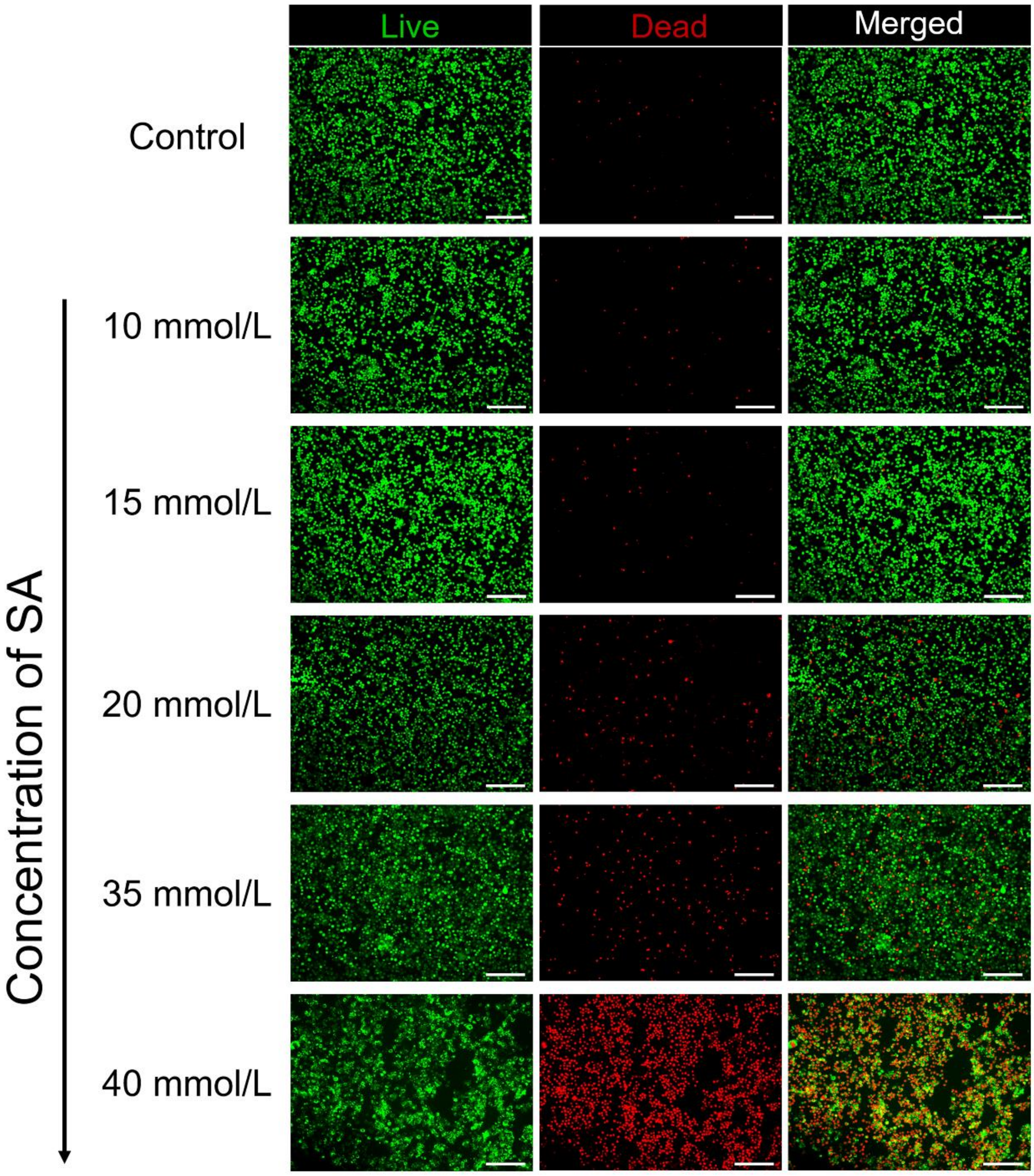

Figure S13. Fluorescence microscopy images of RAW264.7 with different concentrations of SA, green (Calcein-AM) for living cells and red (PI) for dead cells. Scale bar: 200 μm.

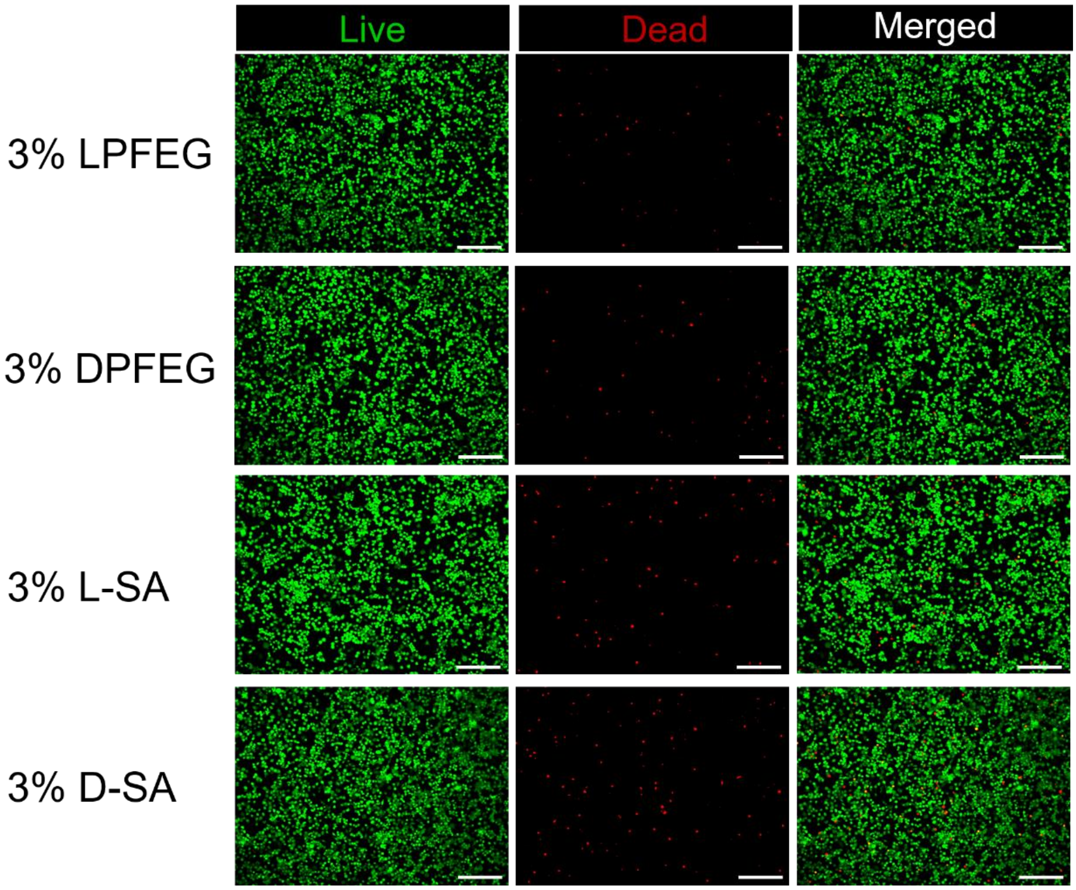

Figure S14. Fluorescence microscopy images of RAW264.7 treated with 3% L, 3% D, 3% L-SA, and 3% D-SA, green (Calcein-AM) for living cells and red (PI) for dead cells. Scale bar: 200 μm.

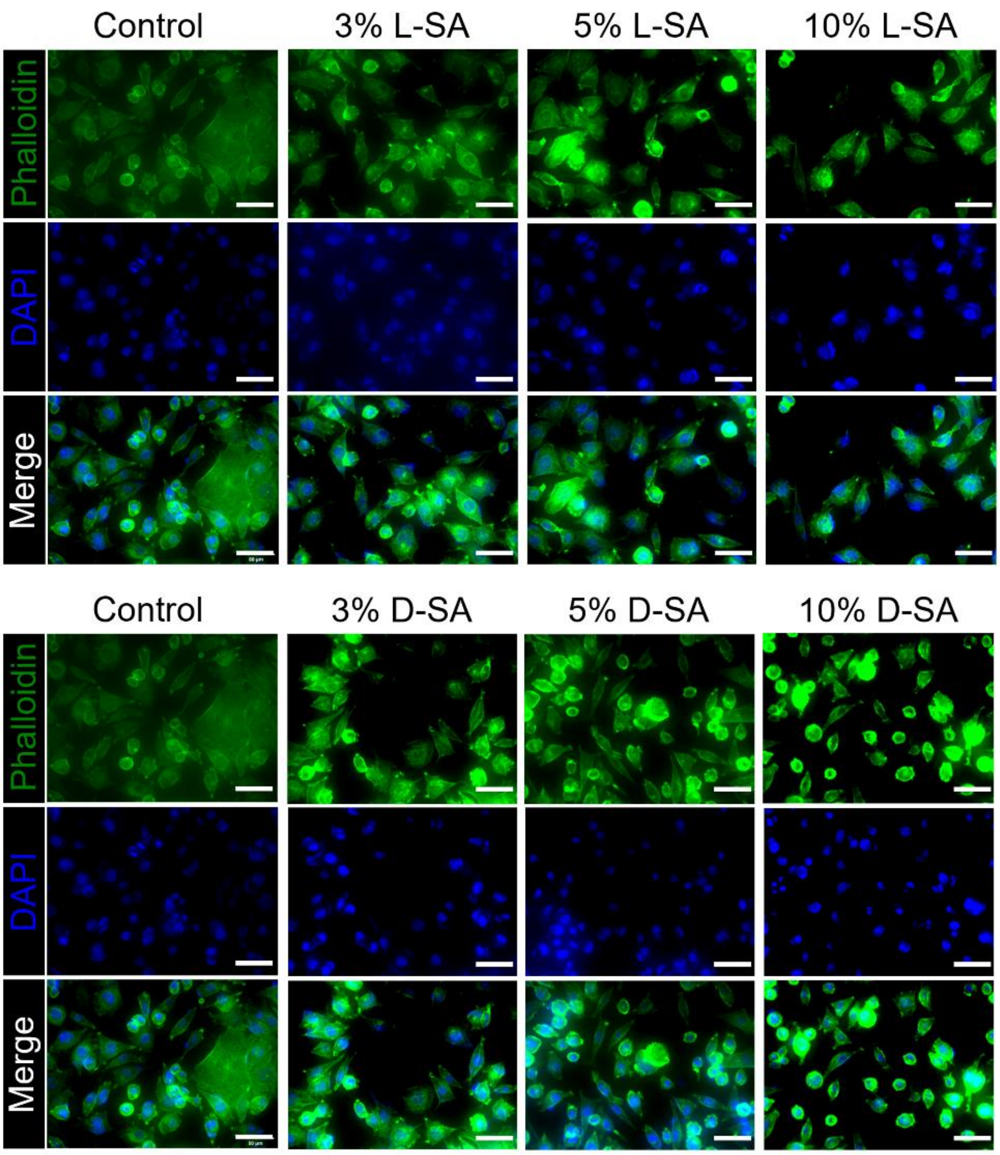

Figure S15. Fluorescence microscopy images of L929 cells at days 3 with different concentrations of L/D-SA, blue (DAPI) for nuclei and green (Fluoresceine Isothiocyanate (FITC) labeled phalloidin) for F-actin. Scale bar: 50 μm.

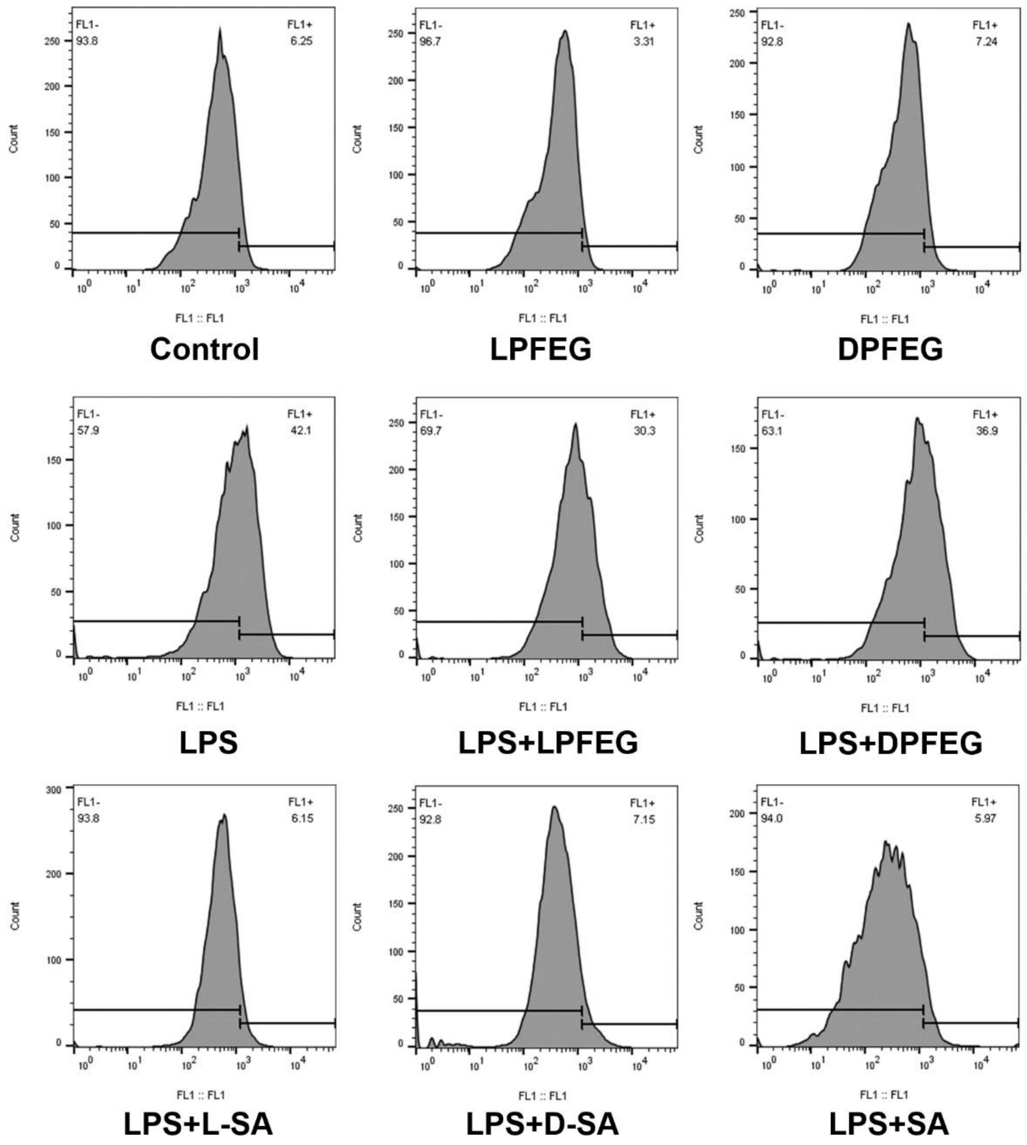

Figure S16. Effect of L/D-SA composite hydrogels on M1 polarization on LPS-induced M1 polarization in RAW264.7 cells (SA = 20 mmol/L, 3% L/D was chosen).

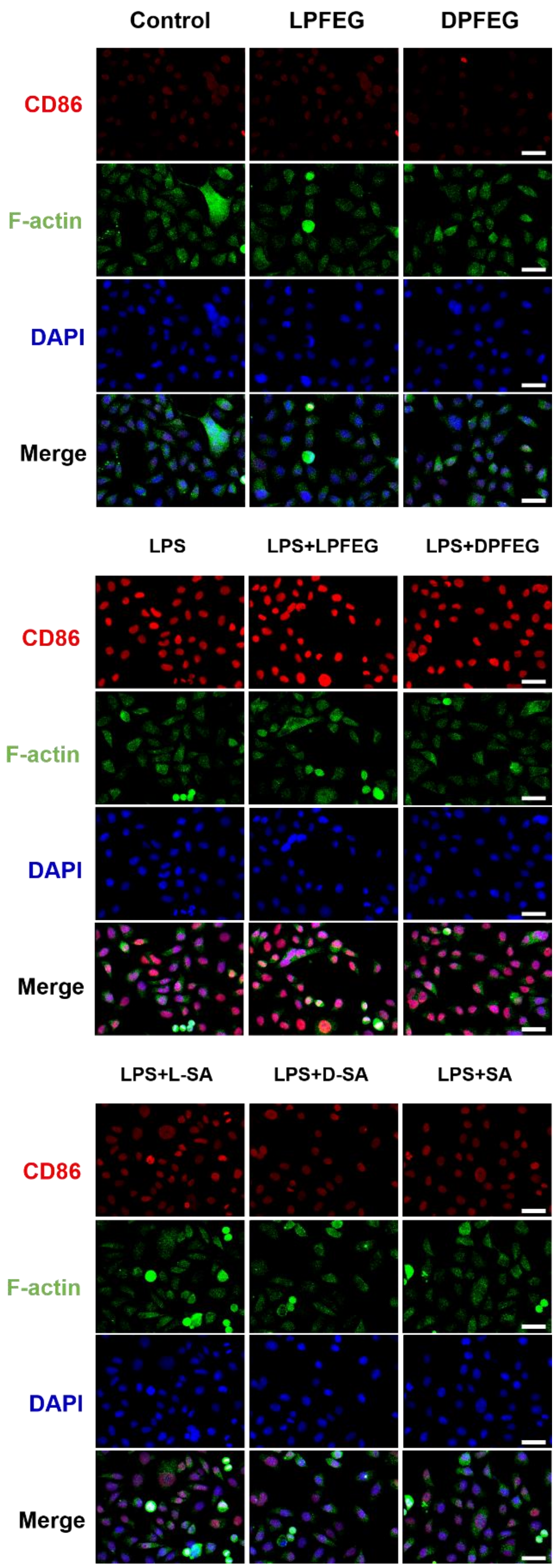

Figure S17. Immunofluorescent staining images of CD86 expressed by macrophages after different treatments. Red (CD86), green (F-actin), and blue (DAPI). Scale bar: 100 μm.

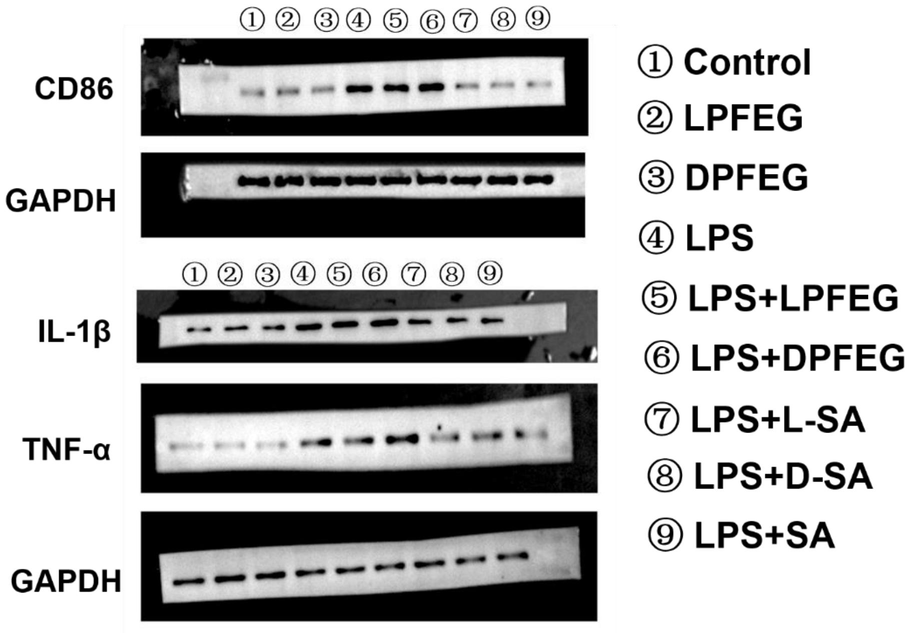

Figure S18. Western blot analysis results of CD86 (M1 marker) and IL-1β and TNF-α (the special functional markers for M1) by macrophages with various treatment. GAPDH was used as the protein loading control.

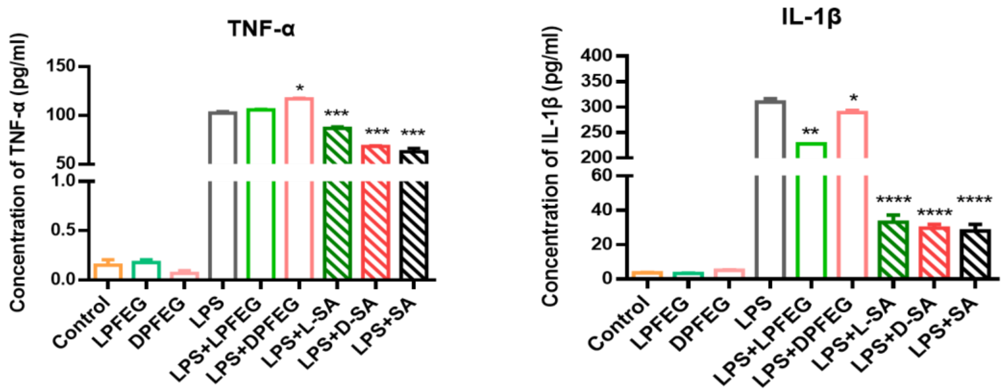

Figure S19. TNF-α and IL-1β secretion expression were evaluated by ELISA (n = 3). The results are expressed as the mean ± SD, n = 3 per group. The P value is calculated by t-test. *p < 0.05, **p < 0.01, ***p < 0.001, and ****p < 0.0001, respectively.

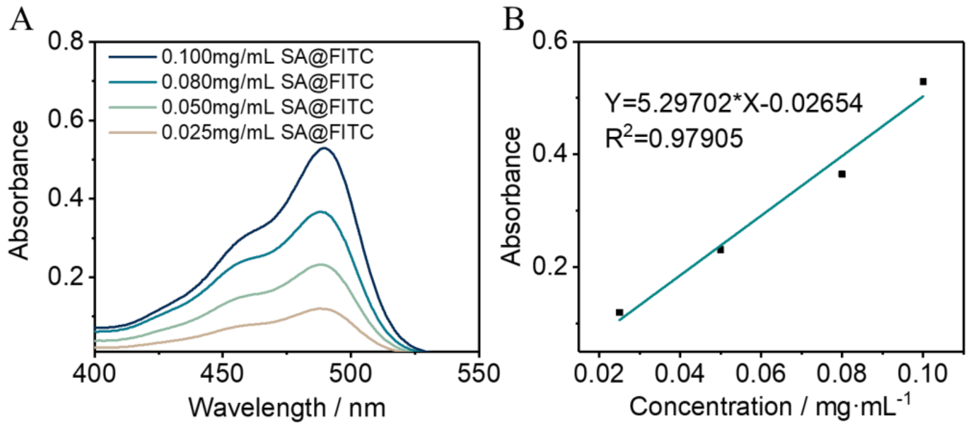

Figure S20. (A) UV-vis absorption of SA labelled with FITC (SA@FITC) at different concentrations. (B) Standard calibration curve of SA@FITC.

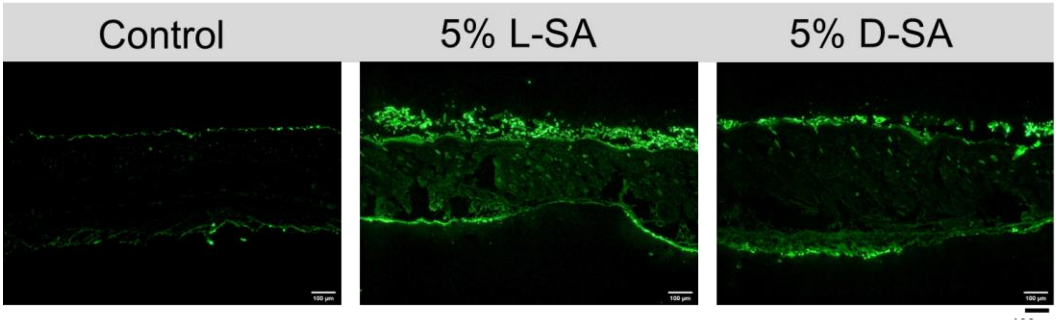

Figure S21. Fluorescent images of the skin after incubation with different materials after 4h. Scale bar: 100 μm.

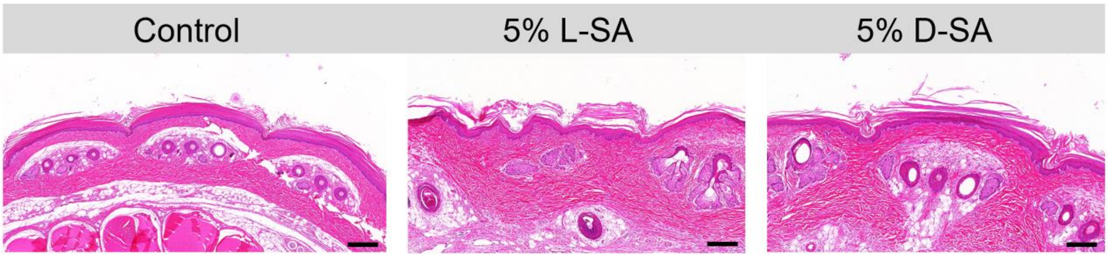

Figure S22. Hematoxylin-eosin staining of the skin treated with different materials after 4h. Scale bar: 200 μm.

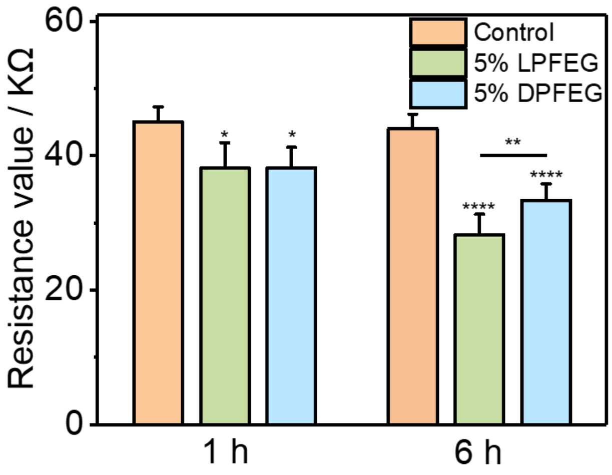

Figure S23. The resistance value of the stratum corneum of the skin after treatment with various penetration enhancers at 1 and 6 hours. The results are expressed as the mean ± SD, n = 3 per group. The P value is calculated by t-test. *p < 0.05, **p < 0.01, ***p < 0.001, and ****p < 0.0001, respectively.

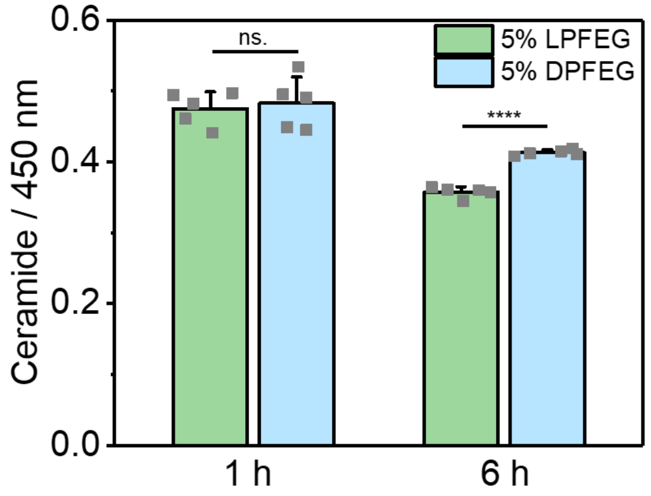

Figure S24. An Elisa test for the detection of ceramide interacted with various penetration enhancers at the absorption of 450 nm (n = 5). The results are expressed as the mean ± SD, n = 3 per group. The P value is calculated by t-test. *p < 0.05, **p

< 0.01, ***p < 0.001, and ****p < 0.0001, respectively.

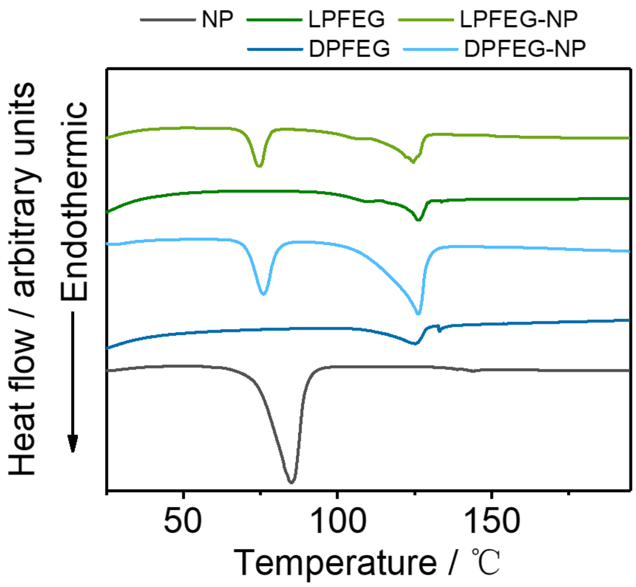

Figure S25. DSC curves of ceramide, LPFEG, DPFEG and the mixture of ceramide and L/DPFEG.

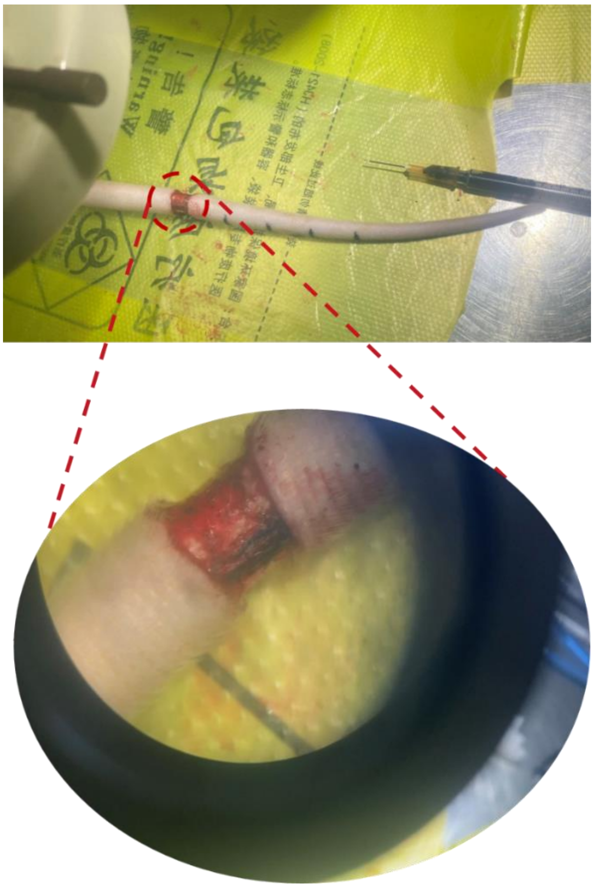

Figure S26. A surgical photograph of rat tail lymphedema model.

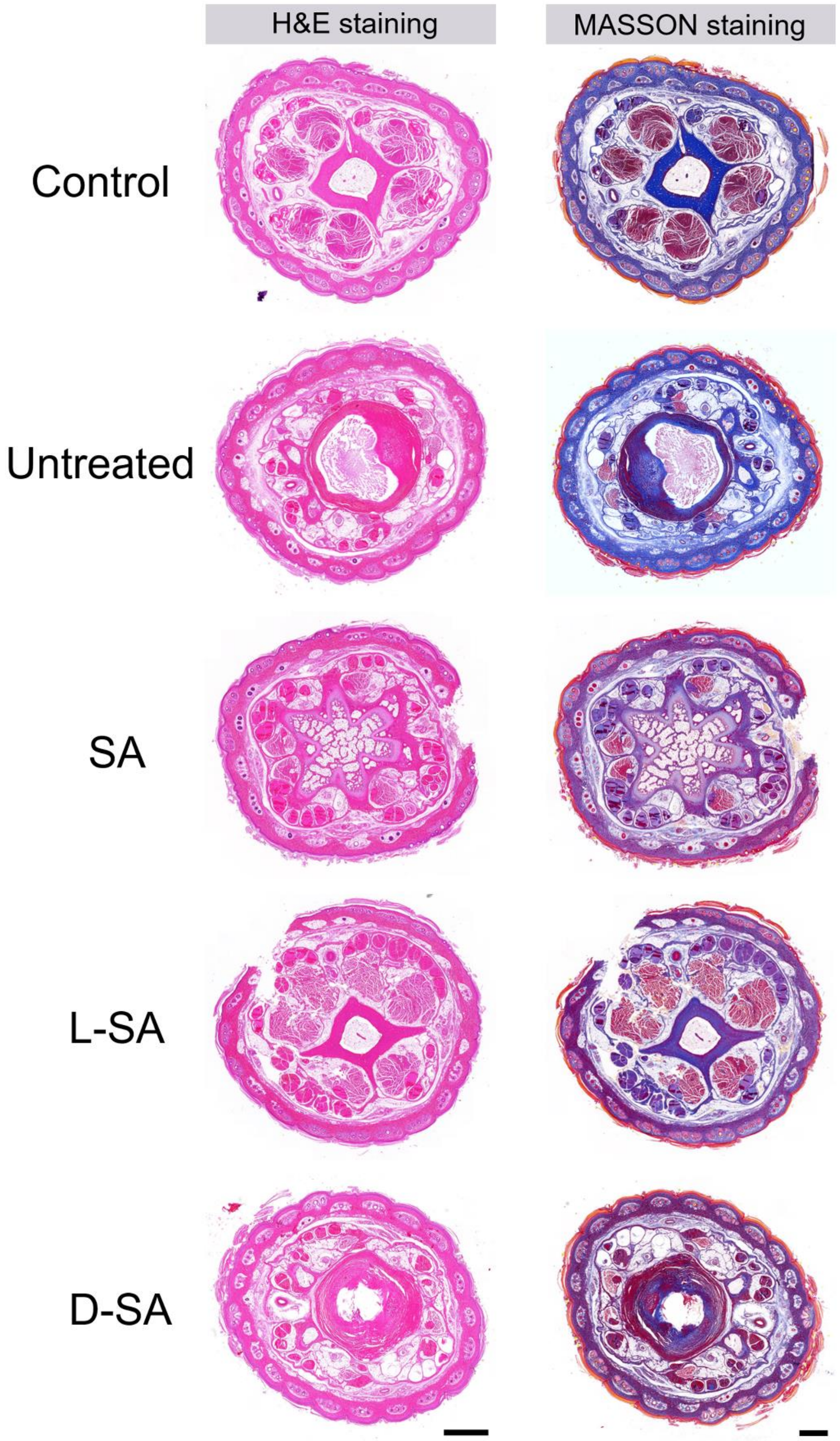

Figure S27. H&E staining and Masson staining images of tail cross-sections with various treatments after 6 weeks. Scale bar = 500 μm.

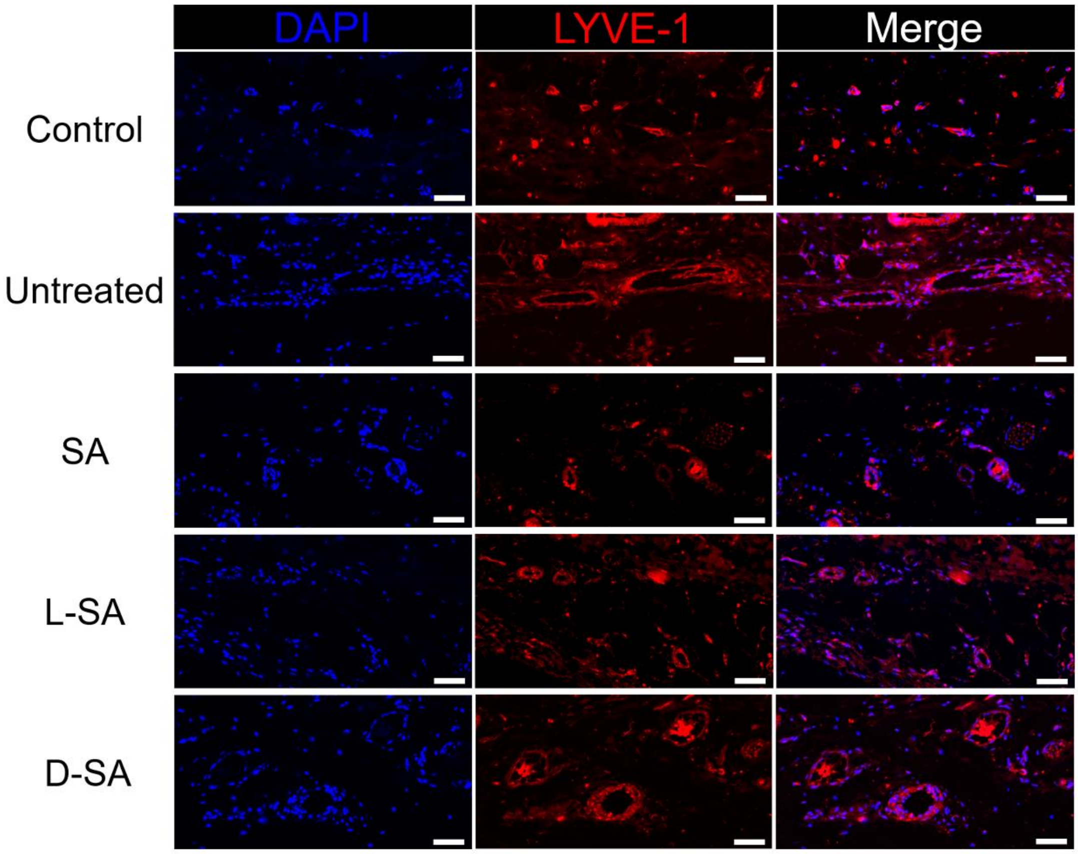

Figure S28. Immunofluorescence staining for LYVE-1 after different treatments. Scale bar = 50 μm.

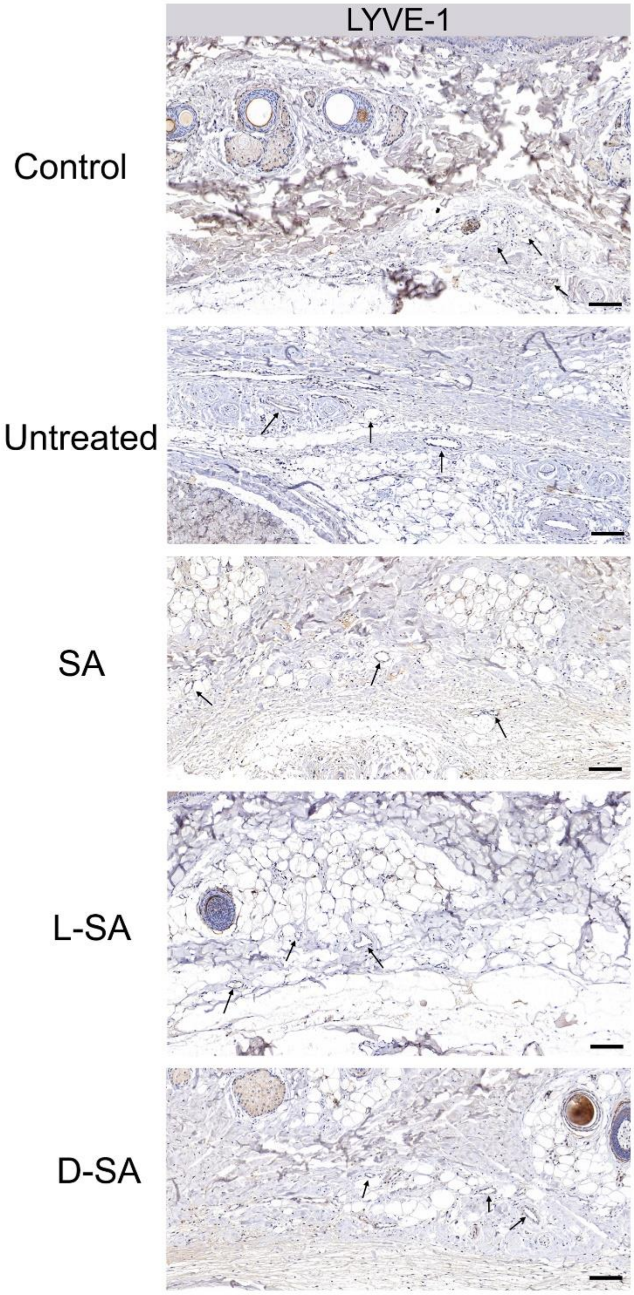

Figure S29. Immunohistochemical images of tissue sections stained with LYVE-1 in various groups. Scale bar = 100 μm.

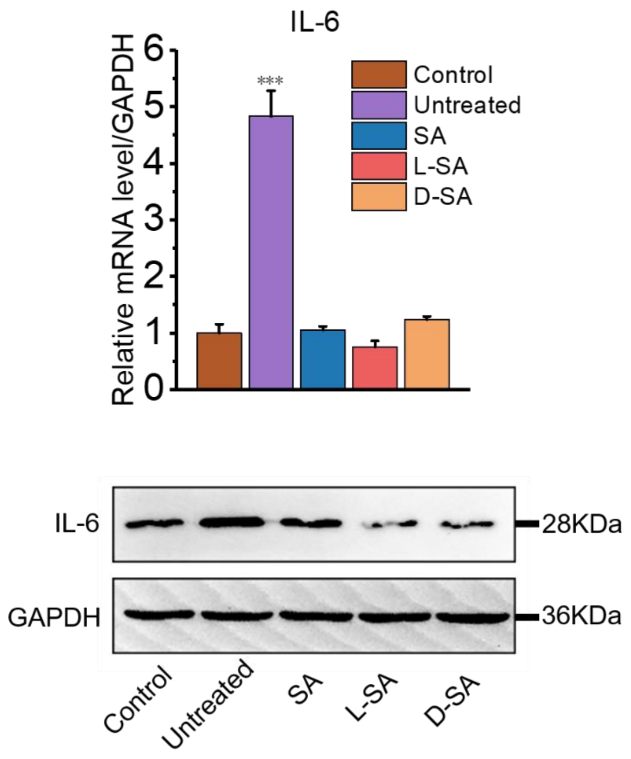

Figure S30. Real-time PCR results of IL-6 and the expression levels of inflammation-related proteins (IL-6) in the rat tail lymphedema analyzed by Western blotting (n = 3). The results are expressed as the mean ± SD, n = 3 per group. The P value is calculated by t-test. *$p < 0.05$, **$p < 0.01$, and ***$p < 0.001$.

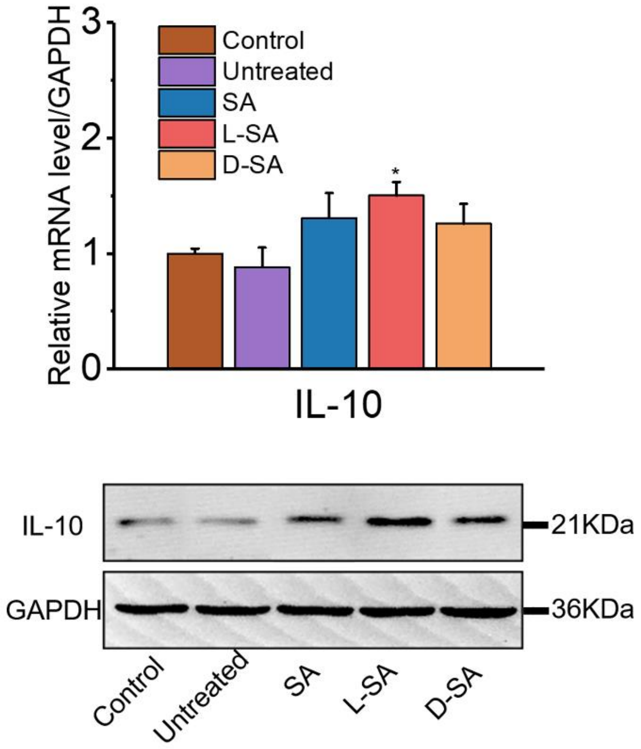

Figure S31. Real-time PCR results of IL-10 and the expression levels of inflammation-related proteins (IL-10) in the rat tail lymphedema analyzed by Western blotting (n = 3). The results are expressed as the mean ± SD, n = 3 per group. The P value is calculated by t-test. *p < 0.05, **p < 0.01, ***p < 0.001, and ****p < 0.0001.

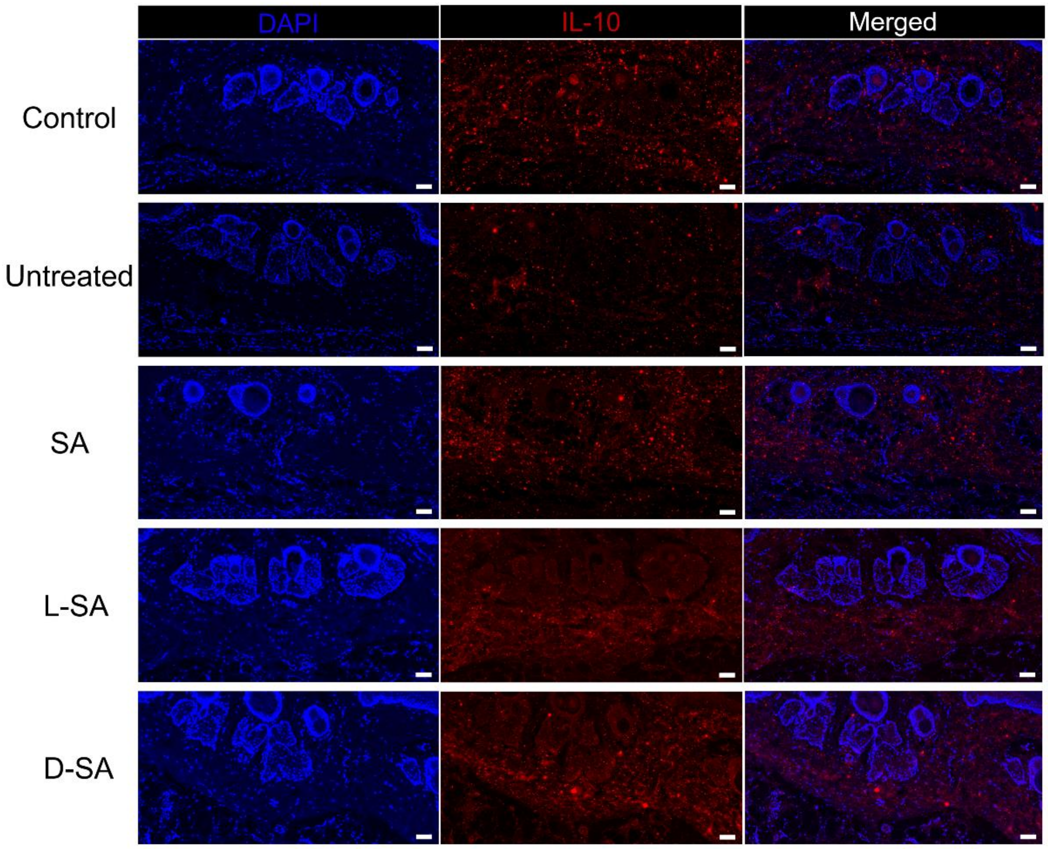

Figure S32. The representative images in the tail lymphedema site after immunofluorescence labeling with IL-10. Scale bar = 100 μm.

References

[1] G. F. Li, X. Li, J. H. Sheng, P. Z. Li, W. K. Ong, S. Z. F. Phua, H. Ågren, L. L. Zhu, Y. L. Zhao, *ACS Nano* **2017,** *11*, 11880.

[2] X. Q. Wang, B. B. Wu, Y. Q. Zhang, *Polym. Chem.* **2022,** *13*, 1685.
