# Supplementary material for: Chiral Supramolecular Hydrogel Enhanced Transdermal Delivery of Sodium Aescinate to Modulate M1 Macrophage Polarization Against Lymphedema
Source: Adv Sci (Weinh). 2023 Dec 1;11(5):2303495. doi: 10.1002/advs.202303495 (PMC10837362; doi:10.1002/advs.202303495)
Supplement: Supplementary file 2 — Supporting Information [file ADVS-11-2303495-s002.pdf]

ADVANCED SCIENCE
Open Access

Supporting Information

for *Adv. Sci.*, DOI 10.1002/advs.202303495

Chiral Supramolecular Hydrogel Enhanced Transdermal Delivery of Sodium Aescinate to Modulate M1 Macrophage Polarization Against Lymphedema

Xueqian Wang, Chunxiao Cui, Xinxian Meng, Chengyao Han, Beibei Wu, Xiaoqiu Dou, Changli Zhao, Yixin Zhang, Ke Li* and Chuanliang Feng**

Figure 1

For CD and corresponding UV-vis spectra:

Wavelength LPFEG 0.12 L-SA 0.08 L-SA 0.04 L-SA (CD data) and the corresponding UV-vis data

400.0	16.7788	7.30525	9.76091	8.00362	9.9	9.49256	9.40724	9.3141
399.5	16.776	7.3788	9.80421	8.03528	9.89641	9.49016	9.40517	9.31205
399.0	16.9134	7.42389	9.86871	8.08717	9.89192	9.48837	9.40343	9.31033
398.5	17.1208	7.27502	9.93988	8.12738	9.89078	9.48691	9.40203	9.30894
398.0	17.2953	7.2659.98141	8.1729	9.88899	9.48569	9.40074	9.30766	
397.5	17.2961	7.29076	10.04479	8.22752	9.88761	9.48474	9.39995	9.30688
397.0	17.4625	7.29924	10.10538	8.29566	9.88598	9.48407	9.39931	9.30625
396.5	17.6096	7.35626	10.1925	8.35931	9.88236	9.48357	9.39904	9.30598
396.0	17.6505	7.3616	10.24838	8.38517	9.8798	9.48357	9.39896	9.3059
395.5	17.7255	7.37849	10.31065	8.44769	9.88042	9.48372	9.39894	9.30588
395.0	17.9958	7.49103	10.38368	8.50048	9.88312	9.48415	9.39942	9.30636
394.5	18.1623	7.47587	10.45371	8.53848	9.8822	9.48475	9.4002	9.30713
394.0	18.1819	7.47867	10.52929	8.61648	9.88061	9.48595	9.40139	9.30831
393.5	18.0914	7.57563	10.58453	8.66538	9.88132	9.48758	9.40289	9.30979
393.0	18.197.55198	10.63891	8.67838	9.88254	9.48923	9.40417	9.31106	
392.5	18.1487	7.64118	10.731	8.75169	9.88558	9.4909	9.40598	9.31285
392.0	18.2661	7.70696	10.77524	8.79041	9.88684	9.49285	9.40751	9.31437
391.5	18.4547	7.79824	10.82788	8.83803	9.88629	9.49463	9.40919	9.31603
391.0	18.6128	7.90091	10.87653	8.9149	9.88898	9.49623	9.41055	9.31738
390.5	18.5875	7.91881	10.95597	8.92548	9.89194	9.4972	9.4116	9.31842
390.0	18.6742	8.01183	10.99835	8.99786	9.89289	9.49804	9.41226	9.31907
389.5	18.7335	8.03553	11.07835	9.05893	9.89382	9.49851	9.41299	9.31979
389.0	18.9007	8.14749	11.14676	9.10524	9.89501	9.49882	9.41338	9.32018
388.5	18.9599	8.20074	11.2255	9.14059	9.89672	9.49905	9.41356	9.32036
388.0	18.9938	8.22015	11.30665	9.20614	9.89884	9.49924	9.41369	9.32049
387.5	19.0264	8.20239	11.36388	9.30079	9.89957	9.49899	9.41373	9.32052
387.0	19.0537	8.32627	11.44032	9.36334	9.89871	9.49875	9.41363	9.32043

386.5	19.0735	8.28511	11.46879	9.41276		9.89807	9.49862	9.41334	9.32014
386.0	19.2045	8.39441	11.53535	9.48169		9.89838	9.49812	9.41299	9.31979
385.5	19.365	8.42962	11.61556	9.52869		9.8999	9.49747	9.41243	9.31924
385.0	19.6824	8.49818	11.7205	9.57648		9.89955	9.49672	9.4115	9.31832
384.5	19.8509	8.63134	11.77876	9.67607		9.9003	9.49585	9.41068	9.3175
384.0	20.1095	8.63375	11.84071	9.72659		9.90079	9.49477	9.40976	9.31659
383.5	20.2502	8.66926	11.91941	9.8229		9.90094	9.49368	9.40878	9.31562
383.0	20.3035	8.69512	12.01909	9.87483		9.8998	9.49252	9.40785	9.3147
382.5	20.4942	8.73943	12.1225	9.90959		9.89918	9.49138	9.40683	9.31369
382.0	20.635	8.82606	12.18606	9.97531		9.89755	9.4903	9.4057	9.31257
381.5	20.7319	8.91584	12.2735	10.03455		9.89535	9.48923	9.40476	9.31164
381.0	20.8941	8.99692	12.34997	10.1039		9.8948	9.48839	9.40364	9.31053
380.5	21.0008	8.93595	12.41344	10.19893		9.8947	9.48746	9.40308	9.30998
380.0	21.1454	8.92315	12.49115	10.23169		9.89387	9.48671	9.40236	9.30927
379.5	21.3268	9.00675	12.54812	10.24969		9.89324	9.48585	9.4015	9.30842
379.0	21.5685	8.99976	12.62762	10.32117		9.89443	9.48518	9.40077	9.30769
378.5	21.8028	9.04347	12.73706	10.37038		9.89433	9.48468	9.40022	9.30715
378.0	21.9861	9.10643	12.82409	10.42959		9.89423	9.48424	9.39971	9.30664
377.5	22.0363	9.21996	12.90588	10.48614		9.89114	9.48362	9.39932	9.30626
377.0	22.2997	9.1277	13.01203	10.58597		9.89155	9.48314	9.39917	9.30611
376.5	22.345	9.22781	13.07076	10.67348		9.89191	9.48277	9.39903	9.30597
376.0	22.3106	9.20146	13.179	10.748		9.89025	9.48253	9.39865	9.30559
375.5	22.3177	9.27647	13.30753	10.80634		9.88912	9.48191	9.39842	9.30537
375.0	22.4007	9.26982	13.41953	10.87341		9.88919	9.48139	9.39803	9.30498
374.5	22.599.34094	13.49582	10.99086		9.88826	9.48097	9.39745	9.30441	
374.0	22.7249	9.47284	13.60565	11.06107		9.88685	9.48058	9.3972	9.30416
373.5	22.7923	9.55986	13.70815	11.16952		9.88578	9.48006	9.39683	9.30379
373.0	22.9254	9.65418	13.77918	11.2631		9.88395	9.47967	9.39654	9.3035
372.5	23.1787	9.61206	13.90724	11.34476		9.88315	9.47906	9.39629	9.30326
372.0	23.4163	9.83441	14.00597	11.41676		9.88136	9.47854	9.39563	9.3026

371.5	23.5445	9.79767	14.11644	11.50645	9.8808	9.47808	9.39527	9.30225
371.0	23.6234	9.86896	14.23947	11.58662	9.87893	9.47771	9.39473	9.30171
370.5	23.6527	9.99012	14.34524	11.65469	9.877	9.4774	9.39442	9.30141
370.0	23.6851	10.0242	14.41744	11.73107	9.87756	9.47701	9.39432	9.30131
369.5	23.8114	10.1009	14.53956	11.81407	9.87833	9.47658	9.39419	9.30118
369.0	24.1267	10.2905	14.69862	11.96455	9.87924	9.4764	9.39372	9.30071
368.5	24.2786	10.3079	14.78541	12.02376	9.87778	9.47627	9.39365	9.30064
368.0	24.443	10.3153	14.88079	12.06334	9.87715	9.47626	9.39389	9.30088
367.5	24.817	10.4405	14.96721	12.16138	9.876	9.47616	9.39387	9.30086
367.0	24.9636	10.55	15.05247	12.26731	9.8771	9.47617	9.39395	9.30094
366.5	25.1302	10.7235	15.12762	12.352	9.87705	9.47624	9.39408	9.30107
366.0	25.149	10.6298	15.22788	12.48034	9.87942	9.47621	9.39414	9.30113
365.5	25.421	10.6659	15.32435	12.57269	9.88149	9.47618	9.39423	9.30122
365.0	25.5432	10.7224	15.41635	12.67176	9.88139	9.47617	9.39424	9.30123
364.5	25.6862	10.9113	15.51374	12.75824	9.88015	9.47621	9.39416	9.30115
364.0	25.8388	11.0501	15.64724	12.83566	9.87909	9.47644	9.39427	9.30126
363.5	25.9377	11.1156	15.74579	12.9201	9.87932	9.47655	9.39446	9.30145
363.0	26.1091	11.2761	15.86706	13.02007	9.88043	9.47672	9.39454	9.30152
362.5	26.4603	11.3552	15.96959	13.112	9.88181	9.47675	9.3949	9.30188
362.0	26.6764	11.4338	16.13347	13.22583	9.87979	9.47676	9.39503	9.30201
361.5	26.9221	11.5344	16.23262	13.35328	9.87689	9.47675	9.39506	9.30204
361.0	27.2381	11.6115	16.35853	13.45172	9.87616	9.47688	9.39518	9.30216
360.5	27.3447	11.6593	16.49026	13.54948	9.87516	9.47683	9.39513	9.30211
360.0	27.6285	11.8111	16.62776	13.69828	9.87429	9.47669	9.39485	9.30183
359.5	27.7385	11.8938	16.79335	13.81128	9.87382	9.47669	9.39473	9.30171
359.0	27.8751	12.0428	16.94294	13.91459	9.87376	9.47674	9.39479	9.30177
358.5	28.2602	12.1054	17.07815	14.02631	9.87335	9.47668	9.39482	9.3018
358.0	28.6569	12.29	17.21644	14.14093	9.87187	9.47672	9.39486	9.30184
357.5	28.8904	12.499	17.38297	14.22003	9.86877	9.47692	9.39496	9.30194
357.0	28.9626	12.6426	17.53232	14.3271	9.86899	9.47709	9.39513	9.30211

356.5	29.2419	12.6935	17.68765	14.4109	9.87031	9.47753	9.39518	9.30216
356.0	29.4357	12.675	17.84179	14.53062	9.87096	9.4776	9.39518	9.30216
355.5	29.707	12.7505	18.00932	14.65921	9.87267	9.47761	9.39536	9.30234
355.0	30.0075	12.758	18.13256	14.7871	9.87236	9.47777	9.39571	9.30268
354.5	30.2757	12.8714	18.25938	14.90607	9.87215	9.47799	9.39565	9.30262
354.0	30.3807	12.9199	18.36318	15.01097	9.87366	9.47827	9.39607	9.30304
353.5	30.6076	13.0568	18.47847	15.13097	9.8747	9.47832	9.39616	9.30313
353.0	30.7165	13.181	18.65926	15.26224	9.87652	9.47867	9.39648	9.30345
352.5	30.9594	13.2893	18.78126	15.39652	9.88074	9.47888	9.39682	9.30378
352.0	31.2085	13.3606	18.92297	15.5061	9.88134	9.47906	9.39728	9.30424
351.5	31.5904	13.5434	19.06147	15.63228	9.88311	9.4793	9.39759	9.30454
351.0	31.8481	13.5349	19.20968	15.74783	9.88366	9.47927	9.39777	9.30472
350.5	32.1032	13.7774	19.36285	15.87969	9.8844	9.47925	9.39783	9.30478
350.0	32.3785	13.8839	19.54032	16.02441	9.88576	9.4796	9.39802	9.30497
349.5	32.5769	13.9791	19.66729	16.18859	9.88516	9.47951	9.39818	9.30513
349.0	32.9488	14.0157	19.82876	16.31734	9.88292	9.47952	9.39844	9.30539
348.5	33.2865	14.1064	20.03124	16.4839	9.88318	9.47948	9.39857	9.30551
348.0	33.6277	14.1925	20.18656	16.63238	9.88328	9.47933	9.39882	9.30576
347.5	33.8556	14.3442	20.34279	16.78834	9.88401	9.47926	9.39908	9.30602
347.0	34.1297	14.6197	20.52203	16.93097	9.88287	9.47932	9.39905	9.30599
346.5	34.3809	14.8657	20.70044	17.07114	9.88178	9.47916	9.39906	9.306
346.0	34.6217	15.07	20.87247	17.25621	9.88114	9.47891	9.39922	9.30616
345.5	35.0419	15.2027	21.06453	17.41703	9.87915	9.47886	9.39908	9.30602
345.0	35.3621	15.3493	21.26118	17.58683	9.8791	9.47895	9.39911	9.30605
344.5	35.6879	15.4653	21.42612	17.74231	9.87731	9.47903	9.399	9.30594
344.0	36.0141	15.6286	21.64309	17.91303	9.8752	9.47904	9.39896	9.3059
343.5	36.2252	15.719	21.845	18.07207	9.8748	9.47885	9.39878	9.30572
343.0	36.4969	15.8489	22.06485	18.27714	9.87328	9.47852	9.39886	9.3058
342.5	36.8842	16.0445	22.23612	18.46097	9.87248	9.47847	9.39883	9.30577
342.0	37.1201	16.1582	22.42632	18.68172	9.87273	9.4785	9.39894	9.30588

341.5	37.499	16.2324	22.61515	18.78028	9.8726	9.47831	9.39877	9.30571
341.0	37.7402	16.3593	22.81503	18.93969	9.87315	9.47832	9.39854	9.30549
340.5	38.1435	16.5183	22.99444	19.09834	9.87579	9.47824	9.39848	9.30543
340.0	38.51	16.6415	23.17447	19.25331	9.87531	9.47809	9.39831	9.30526
339.5	38.712	16.7664	23.36982	19.40279	9.87509	9.47825	9.39822	9.30517
339.0	39.0491	16.8893	23.57138	19.61424	9.8749	9.47824	9.3983	9.30525
338.5	39.2697	16.9774	23.78359	19.78752	9.87457	9.47815	9.39838	9.30533
338.0	39.5405	17.0432	24.00324	19.99024	9.87476	9.47815	9.39834	9.30529
337.5	39.9374	17.1393	24.25624	20.14969	9.87583	9.4782	9.39844	9.30539
337.0	40.3248	17.3148	24.44632	20.31986	9.87745	9.47828	9.39839	9.30534
336.5	40.6676	17.3372	24.67624	20.51617	9.87857	9.47802	9.39848	9.30543
336.0	40.9765	17.543	24.88853	20.72831	9.87802	9.47813	9.39858	9.30552
335.5	41.3486	17.6345	25.11921	20.91734	9.87812	9.47807	9.39867	9.30561
335.0	41.6863	17.8774	25.33576	21.07159	9.87896	9.47803	9.39885	9.30579
334.5	41.9374	18.05	25.56859	21.22941	9.88002	9.47819	9.39885	9.30579
334.0	42.2861	18.1305	25.81997	21.41855	9.88029	9.47818	9.39894	9.30588
333.5	42.6385	18.3393	26.10256	21.59293	9.87845	9.47835	9.39919	9.30613
333.0	42.9693	18.5892	26.35665	21.82731	9.87819	9.47834	9.39936	9.3063
332.5	43.4117	18.8049	26.63182	22.05593	9.87966	9.47854	9.39984	9.30677
332.0	43.6835	18.9954	26.90521	22.31493	9.87896	9.47875	9.40001	9.30694
331.5	44.1044	19.2845	27.17697	22.50893	9.87927	9.47872	9.40043	9.30736
331.0	44.5839	19.4553	27.42879	22.72583	9.87924	9.47871	9.40066	9.30758
330.5	45.0148	19.5834	27.72238	22.9591	9.87965	9.47882	9.40103	9.30795
330.0	45.5052	19.7321	27.99129	23.21979	9.88146	9.47881	9.40123	9.30815
329.5	45.9362	19.9549	28.25115	23.43621	9.88286	9.47887	9.4018	9.30871
329.0	46.3469	20.1157	28.52344	23.68093	9.88094	9.47903	9.40223	9.30914
328.5	46.6505	20.3528	28.77224	23.89897	9.88071	9.47886	9.4025	9.30941
328.0	47.1229	20.6397	29.03626	24.15431	9.88079	9.47897	9.40257	9.30948
327.5	47.5982	20.9055	29.35759	24.36817	9.88124	9.47878	9.40265	9.30955
327.0	48.0976	21.1519	29.64735	24.63452	9.88105	9.47849	9.40263	9.30953

326.5	48.425	21.3544	29.92294	24.90124	9.87903	9.47832	9.4025	9.30941
326.0	48.8215	21.6153	30.19235	25.17721	9.87902	9.47826	9.40242	9.30933
325.5	49.4467	21.9181	30.46118	25.45638	9.88031	9.47826	9.40248	9.30939
325.0	49.993	22.02	30.78588	25.67707	9.88086	9.47828	9.40274	9.30964
324.5	50.4017	22.1787	31.09794	25.944	9.88142	9.47833	9.40298	9.30988
324.0	50.854	22.4016	31.39206	26.20286	9.88032	9.4782	9.40301	9.30991
323.5	51.2655	22.579	31.67059	26.45655	9.8805	9.47837	9.40328	9.31018
323.0	51.6321	22.7032	31.99559	26.75245	9.87959	9.47852	9.40326	9.31016
322.5	52.1746	22.8164	32.34235	27.06214	9.87981	9.47858	9.40344	9.31034
322.0	52.755	23.1443	32.66353	27.3561	9.87969	9.47868	9.40348	9.31038
321.5	53.2333	23.5051	33.03059	27.66928	9.87764	9.479	9.40365	9.31054
321.0	53.8091	23.7534	33.36324	27.96038	9.87641	9.47918	9.40366	9.31055
320.5	54.4534	23.9553	33.74382	28.29221	9.87775	9.47948	9.40392	9.31081
320.0	54.919	24.3742	34.16265	28.56262	9.87722	9.47959	9.40421	9.3111
319.5	55.3891	24.6214	34.525	28.89276	9.87759	9.4798	9.40436	9.31125
319.0	56.0704	24.8894	34.895	29.25652	9.87675	9.48005	9.40463	9.31151
318.5	56.5949	25.1072	35.23971	29.5441	9.87623	9.48055	9.40511	9.31199
318.0	57.1368	25.3196	35.53706	29.82166	9.87714	9.48098	9.40536	9.31224
317.5	57.6575	25.5751	35.91353	30.10262	9.87639	9.48119	9.40574	9.31261
317.0	58.3338	25.8951	36.53559	30.62214	9.87373	9.48102	9.40579	9.31266
316.5	58.9674	26.2412	36.94147	30.97283	9.87399	9.48086	9.40585	9.31272
316.0	59.6358	26.5554	37.34706	31.3099	9.87331	9.48091	9.40602	9.31289
315.5	60.2035	26.8683	37.76971	31.70934	9.87284	9.48111	9.4063	9.31317
315.0	61.0317	27.2833	38.21529	32.08279	9.87434	9.48134	9.40677	9.31363
314.5	61.6971	27.677	38.64706	32.43041	9.87503	9.48165	9.40713	9.31399
314.0	62.4709	28.0618	39.10529	32.81566	9.87696	9.48226	9.40725	9.31411
313.5	63.1224	28.3603	39.53294	33.16993	9.87738	9.48242	9.40768	9.31453
313.0	63.6969	28.5893	40.01059	33.51321	9.87761	9.4826	9.40806	9.31491
312.5	64.4163	28.8471	40.47971	33.91414	9.8786	9.48278	9.40825	9.3151
312.0	65.0015	29.2459	40.93206	34.31641	9.87811	9.48311	9.40861	9.31546

311.5	65.8957	29.4673	41.43882	34.79552	9.87844	9.48349	9.40907	9.31591
311.0	66.5503	29.8358	41.92882	35.21483	9.87723	9.48369	9.40943	9.31627
310.5	67.2379	30.1227	42.45412	35.62	9.87825	9.48393	9.41001	9.31684
310.0	67.9779	30.5441	43.03088	36.05759	9.87842	9.4842	9.41066	9.31749
309.5	68.7079	30.9362	43.54941	36.51897	9.87925	9.48457	9.4115	9.31832
309.0	69.5456	31.2486	44.07706	36.98241	9.88192	9.48522	9.41216	9.31897
308.5	70.4908	31.6612	44.61353	37.45138	9.88506	9.48601	9.41285	9.31965
308.0	71.353	32.0476	45.21029	37.96759	9.88655	9.48668	9.41353	9.32033
307.5	72.0801	32.4435	45.77324	38.44759	9.88719	9.48739	9.41428	9.32107
307.0	72.898	32.821	46.30059	38.87483	9.88874	9.48766	9.41472	9.3215
306.5	73.667	33.3501	46.96824	39.47897	9.88871	9.48808	9.41523	9.32201
306.0	74.8198	33.877	47.74882	40.18	9.88897	9.48861	9.41617	9.32294
305.5	75.4729	34.3696	48.37088	40.6831	9.88771	9.48911	9.41704	9.3238
305.0	76.3667	34.7388	48.89176	41.16759	9.88855	9.48984	9.41762	9.32438
304.5	77.2665	35.2999	49.55412	41.71483	9.88948	9.49042	9.41827	9.32502
304.0	78.224	35.7893	50.20794	42.29621	9.89192	9.49109	9.4191	9.32584
303.5	79.2497	36.1735	50.8742.82759	9.89366	9.49175	9.42017	9.3269	
303.0	80.047	36.6583	51.50794	43.3631	9.89485	9.49268	9.42132	9.32804
302.5	80.8772	37.2114	52.12147	43.91	9.89573	9.49373	9.42246	9.32917
302.0	81.7393	37.6653	52.73647	44.48069	9.89763	9.49483	9.42356	9.33026
301.5	82.6463	38.1256	53.42324	45.05793	9.89953	9.49623	9.42483	9.33151
301.0	83.4858	38.584	54.09059	45.60724	9.90146	9.49765	9.4263	9.33297
300.5	84.3197	39.2668	54.76941	46.17931	9.90107	9.49918	9.42799	9.33464
300.0	85.0276	39.7866	55.45324	46.77517	9.90148	9.50104	9.43001	9.33664
299.5	85.6156	40.3475	56.135	47.38414	9.90264	9.50292	9.4323	9.33891
299.0	86.3215	40.7956.86706	47.99069	9.90451	9.5059.43476	9.34135		
298.5	87.1416	41.347	57.53882	48.56448	9.90654	9.5075	9.43746	9.34402
298.0	87.8317	41.9084	58.32824	49.19103	9.90771	9.51008	9.44074	9.34727
297.5	88.5781	42.5057	59.06118	49.83138	9.90973	9.51288	9.44407	9.35056
297.0	89.4036	43.0581	59.82618	50.55241	9.91386	9.51575	9.44762	9.35408

296.5	90.4072	43.7388	60.73529	51.33759	9.91672	9.51898	9.45183	9.35825
296.0	91.4639	44.5973	61.76882	52.25828	9.91678	9.52271	9.45655	9.36292
295.5	92.199	45.5315	62.89353	53.20517	9.91805	9.52624	9.46078	9.36711
295.0	93.4725	46.4183	64.00059	54.16586	9.92145	9.52944	9.46454	9.37083
294.5	94.7022	47.4988	65.1955.21621		9.92331	9.53313	9.46898	9.37523
294.0	96.0772	48.5456	66.51588	56.45034	9.92543	9.53732	9.47409	9.38029
293.5	97.4641	49.7367	67.99147	57.79207	9.92691	9.54195	9.4798	9.38594
293.0	99.1586	51.0912	69.58441	59.25586	9.92896	9.54683	9.48554	9.39162
292.5	100.889	52.5504	71.27882	60.80345	9.92925	9.55189	9.49123	9.39726
292.0	102.722	53.8267	72.99088	62.39276	9.93131	9.5564	9.49677	9.40274
291.5	104.79	55.4232	74.9264.08655		9.93352	9.56101	9.50272	9.40863
291.0	106.988	56.9967	76.85618	65.82241	9.93685	9.56577	9.50792	9.41378
290.5	109.605	58.8166	79.13147	67.8731	9.94026	9.57087	9.51423	9.42003
290.0	112.348	60.5808	81.45206	69.96414	9.94321	9.57571	9.52012	9.42586
289.5	115.117	62.4696	83.84382	72.14931	9.94689	9.58058	9.52583	9.43151
289.0	117.911	64.4732	86.34735	74.40276	9.94931	9.58509	9.53135	9.43698
288.5	120.719	66.5428	88.81441	76.59517	9.95091	9.58936	9.53656	9.44214
288.0	123.872	68.6879	91.50971	79.02345	9.95393	9.59414	9.54209	9.44761
287.5	127.108	70.8394	94.21176	81.45517	9.95784	9.59891	9.54768	9.45315
287.0	130.394	72.9717	96.95735	83.98138	9.9594	9.60401	9.55367	9.45908
286.5	133.677	75.4505	99.93735	86.74034	9.96223	9.60943	9.56008	9.46543
286.0	136.881	77.6621	102.89912	89.44862	9.96617	9.61435	9.56577	9.47106
285.5	140.34	79.8329	105.75059	92.0269	9.97011	9.61867	9.57079	9.47603
285.0	143.94	82.0786	108.66088	94.6469	9.97424	9.62307	9.57596	9.48115
284.5	147.389	84.646	111.76941	97.52379	9.97738	9.6273	9.58158	9.48671
284.0	150.964	87.1316	115.10765	100.57034	9.98273	9.63217	9.58767	9.49274
283.5	155.067	89.8034	118.45853	103.69897	9.98783	9.63711	9.5938	9.49881
283.0	158.812	92.2976	121.75265	106.79207	9.99158	9.64195	9.59982	9.50477
282.5	162.338	94.876	124.92912	109.80448	9.99435	9.64664	9.60579	9.51068
282.0	165.938	97.6248	128.28382	113.00828	9.99538	9.65186	9.61226	9.51709

281.5	169.891	100.346	131.70206	116.25138	10.00004	9.65707	9.61872	9.52349
281.0	173.605	103.149	135.18941	119.56862	10.00351	9.66237	9.6254	9.5301
280.5	177.377	106.062	138.87824	123.09586	10.0083	9.66853	9.63278	9.53741
280.0	181.097	109.085	142.43912	126.5669	10.01155	9.67441	9.6399	9.54446
279.5	184.612	112.007	146.02559	130.04724	10.01623	9.68048	9.64744	9.55192
279.0	188.289	114.932	149.62441	133.66103	10.02183	9.68681	9.6554	9.5598
278.5	191.952	118.055	153.39529	137.46655	10.02709	9.69364	9.66403	9.56835
278.0	195.473	121.242	157.18765	141.33414	10.03181	9.70088	9.67321	9.57744
277.5	198.504	124.309	160.73441	144.98828	10.03615	9.70796	9.68219	9.58633
277.0	201.734	127.497	164.39735	148.80828	10.04024	9.71578	9.69219	9.59623
276.5	204.052	130.438	167.85529	152.46655	10.04398	9.72359	9.702	9.60594
276.0	206.581	133.296	171.11118	155.95862	10.04901	9.7313	9.71179	9.61563
275.5	208.796	136.257	174.21735	159.43586	10.05214	9.73947	9.722	9.62574
275.0	210.774	139.125	177.33706	162.99621	10.05546	9.74833	9.73343	9.63706
274.5	212.278	142.038	180.30824	166.54586	10.06153	9.75773	9.74543	9.64894
274.0	213.555	144.683	183.105	169.95207	10.06805	9.76756	9.75762	9.66101
273.5	214.572	147.344	185.75176	173.32724	10.07314	9.77787	9.77068	9.67394
273.0	215.023	150.165	188.18824	176.65034	10.07805	9.78881	9.78434	9.68747
272.5	215.611	152.656	190.41765	179.73828	10.08413	9.79955	9.79777	9.70076
272.0	215.803	155.118	192.52353	182.93966	10.09082	9.81108	9.81244	9.71529
271.5	215.761	157.711	194.41706	185.92483	10.0979	9.82317	9.8276	9.7303
271.0	215.311	160.044	195.99324	188.74793	10.10484	9.83579	9.84325	9.74579
270.5	214.006	162.22	197.29765	191.35103	10.11091	9.84866	9.85902	9.76141
270.0	212.485	163.955	198.14118	193.71621	10.11756	9.862	9.8756	9.77782
269.5	210.696	165.64	198.66265	195.76621	10.12505	9.87588	9.89264	9.79469
269.0	208.602	167.143	198.85765	197.5369	10.1332	9.88999	9.91027	9.81215
268.5	206.104	168.384	198.68706	199.15483	10.14156	9.90458	9.92828	9.82998
268.0	203.646	169.418	198.12147	200.32862	10.1502	9.91891	9.94565	9.84718
267.5	200.28	170.142	197.17265	201.24724	10.15731	9.93311	9.96334	9.86469
267.0	196.608	170.464	195.86824	202.00241	10.16625	9.94845	9.98228	9.88345

266.5	193.033	170.67	194.08941	202.2931	10.17571	9.96414	10.00161	9.90258
266.0	188.863	170.68	191.92676	202.25931	10.18282	9.98011	10.02094	9.92172
265.5	184.602	170.492	189.58647	201.85138	10.18976	9.99509	10.03959	9.94019
265.0	179.787	169.597	186.69412	201.11172	10.19802	10.01115	10.05908	9.95949
264.5	174.781	168.402	183.41853	199.96207	10.2063	10.02722	10.07874	9.97895
264.0	169.307	166.682	179.69941	198.33241	10.21313	10.04339	10.09844	9.99846
263.5	163.225	164.752	175.53529	196.29862	10.21853	10.05971	10.11848	10.0183
263.0	157.911	162.422	171.27794	194.06586	10.22561	10.07518	10.13727	10.0369
262.5	151.709	160.263	166.59412	191.20138	10.23222	10.09139	10.15717	10.0566
262.0	145.888	157.439	161.76294	188.07931	10.24091	10.10746	10.17666	10.0759
261.5	140.507	154.614	156.88441	184.79172	10.24951	10.12249	10.19474	10.0938
261.0	134.685	151.333	151.64059	180.97034	10.25625	10.13813	10.21342	10.1123
260.5	128.151	147.561	145.94618	176.64414	10.26174	10.15462	10.23271	10.1314
260.0	122.096	143.755	140.29824	172.28897	10.26832	10.16992	10.25079	10.1493
259.5	115.934	139.587	134.58441	167.66552	10.27322	10.18496	10.26887	10.1672
259.0	110.068	135.561	128.85088	162.71207	10.27958	10.19994	10.28645	10.1846
258.5	104.145	130.975	123.01882	157.41931	10.28551	10.21459	10.30362	10.2016
258.0	98.0271	126.189	116.91294	151.74621	10.29242	10.22948	10.32079	10.2186
257.5	91.4311	120.576	110.29647	145.34759	10.29915	10.24529	10.33917	10.2368
257.0	85.2135	115.049	103.65265	138.58586	10.30821	10.26049	10.35694	10.2544
256.5	79.7825	109.988	97.97941	132.51138	10.31522	10.27344	10.37189	10.2692
256.0	74.7891	105.292.61206	126.73586		10.32099	10.28517	10.38543	10.2826
255.5	69.8676	99.8002	86.86088	120.18586	10.32599	10.29772	10.39967	10.2967
255.0	64.8733	94.433	81.45588	113.83172	10.3309	10.30914	10.4126	10.3095
254.5	60.0848	88.737	76.09529	107.46069	10.33531	10.31995	10.42492	10.3217
254.0	55.7352	83.6227	71.06235	101.38207	10.33817	10.32985	10.43603	10.3327
253.5	51.3039	78.351	66.27059	95.29862	10.34116	10.33913	10.44683	10.3434
253.0	46.7267	73.062	61.39559	88.93069	10.34507	10.34872	10.45784	10.3543
252.5	42.3525	67.2088	56.37471	82.36586	10.35072	10.3579	10.46875	10.3651
252.0	37.825	61.1387	51.22235	75.38759	10.35733	10.36708	10.47915	10.3754

251.5	33.581	55.2028	46.25529	68.29759	10.36186	10.37564	10.48885	10.385
251.0	29.9358	50.0852	42.15735	62.44103	10.36483	10.38258	10.49673	10.3928
250.5	26.5498	45.2321	38.16265	56.76759	10.36804	10.38901	10.5038	10.3998
250.0	23.2444	40.2487	34.41235	51.09828	10.37089	10.39462	10.51057	10.4065
249.5	19.6076	34.6215	30.45676	44.89	10.37371	10.40063	10.51753	10.4134
249.0	16.5183	29.4768	26.72474	39.0669	10.37582	10.40604	10.52329	10.4191
248.5	13.3465	24.6216	23.19059	33.48817	10.37825	10.41104	10.52875	10.4245
248.0	10.4072	20.0906	19.86738	28.37852	10.38167	10.41522	10.53309	10.4288
247.5	7.35543	14.8761	16.61832	22.88983	10.3844	10.4192	10.53753	10.4332
247.0	4.56762	10.3536	13.55821	17.89948	10.3875	10.42277	10.54157	10.4372
246.5	1.86418	5.86755	10.51506	12.88566	10.38992	10.42613	10.54551	10.4411
246.0	-0.84439	2.13116	7.81106	8.227	10.39347	10.42909	10.54884	10.4444
245.5	-3.32781	-2.40678	5.20256	3.69555	10.39622	10.43185	10.55187	10.4474
245.0	-5.9104	-6.79858	2.28193	-1.10658	10.39916	10.43491	10.55501	10.4505
244.5	-8.21169	-10.9534	-0.32741	-5.69703	10.40262	10.43756	10.55773	10.4532
244.0	-10.5917	-15.5721	-3.01294	-10.35352	10.40344	10.44021	10.56056	10.456
243.5	-12.7735	-19.5524	-5.33771	-14.621	10.40548	10.44266	10.56319	10.4586
243.0	-15.0051	-23.899	-7.8635	-19.24783	10.40931	10.44541	10.56601	10.4614
242.5	-17.255	-28.1951	-10.26462	-23.59679	10.4128	10.44796	10.56874	10.4641
242.0	-19.5598	-32.1875	-12.53835	-27.96007	10.41544	10.45082	10.57167	10.467
241.5	-21.4003	-35.7934	-14.67691	-31.81817	10.41858	10.45357	10.5745	10.4698
241.0	-23.436	-39.5586	-16.84432	-35.72828	10.42346	10.45633	10.57773	10.473
240.5	-25.6105	-43.6586	-18.91062	-39.59793	10.42866	10.45928	10.58116	10.4764
240.0	-27.4959	-47.5724	-20.92303	-43.44655	10.43215	10.46214	10.5845	10.4797
239.5	-29.5071	-51.5959	-22.97847	-47.36931	10.43491	10.4654	10.58854	10.4837
239.0	-31.4069	-55.0612	-25.06512	-51.06552	10.43872	10.46887	10.59268	10.4878
238.5	-33.4257	-58.7265	-27.00503	-54.89207	10.44299	10.47244	10.59692	10.492
238.0	-35.3242	-62.4959	-28.93326	-58.63448	10.44912	10.47632	10.60116	10.4962
237.5	-37.3241	-66.7545	-30.96441	-62.60414	10.45493	10.4803	10.60601	10.501
237.0	-39.2161	-70.9239	-33.12471	-66.87448	10.46074	10.48489	10.61157	10.5065

236.5	-41.3557	-75.7607	-35.40441	-71.5	10.46519	10.48999	10.61783	10.5127
236.0	-43.8286	-80.1109	-37.76559	-76.17138	10.47069	10.4958	10.6249	10.5197
235.5	-45.7882	-83.8633	-39.66618	-80.33931	10.47854	10.5011	10.63156	10.5263
235.0	-47.6112	-87.7553	-41.41324	-84.08483	10.48373	10.506	10.63762	10.5323
234.5	-49.6154	-91.7764	-43.32059	-88.24138	10.49036	10.5113	10.64439	10.539
234.0	-51.765	-96.4437	-45.31353	-92.88069	10.49712	10.51753	10.65257	10.5471
233.5	-53.8955	-101.291	-47.34912	-97.68	10.50441	10.52405	10.66095	10.5554
233.0	-55.9491	-105.923	-49.33676	-102.48	10.51197	10.53099	10.67004	10.5644
232.5	-57.9911	-111.013	-51.23882	-107.58069	10.51649	10.53823	10.67954	10.5738
232.0	-59.7097	-115.9	-53.05265	-112.57448	10.52363	10.54609	10.68974	10.5839
231.5	-61.9007	-120.985	-54.83735	-117.7769	10.53066	10.55557	10.70226	10.5963
231.0	-63.2933	-125.712	-56.32265	-122.77966	10.53927	10.56506	10.71509	10.609
230.5	-64.7018	-129.942	-57.39059	-127.16207	10.54935	10.57424	10.72741	10.6212
230.0	-65.7631	-134.491	-58.38206	-131.53483	10.55849	10.58434	10.74155	10.6352
229.5	-66.4576	-138.451	-58.96853	-135.31586	10.56594	10.59515	10.7569	10.6504
229.0	-66.9108	-141.553	-59.34059	-138.61828	10.57361	10.60708	10.77347	10.6668
228.5	-66.9403	-144.425	-59.23265	-141.59862	10.58222	10.61983	10.79145	10.6846
228.0	-66.8146	-146.959	-58.67412	-143.92345	10.59481	10.63411	10.81205	10.705
227.5	-66.0857	-149.044	-57.64559	-145.25345	10.60427	10.6487	10.83336	10.7261
227.0	-64.6694	-149.897	-56.21618	-145.66138	10.61361	10.6642	10.85599	10.7485
226.5	-63.2794	-149.973	-54.49176	-144.84586	10.62674	10.68103	10.88063	10.7729
226.0	-60.7976	-148.975	-52.31735	-142.94552	10.6364	10.69909	10.90669	10.7987
225.5	-58.0623	-147.261	-49.80294	-139.95655	10.64438	10.71806	10.93436	10.8261
225.0	-55.3557	-144.449	-46.93441	-136.22897	10.65435	10.73907	10.96527	10.8567
224.5	-52.0945	-140.861	-43.70559	-131.48483	10.66454	10.76131	10.99789	10.889
224.0	-48.8824	-136.571	-40.51294	-126.46655	10.6721	10.78262	11.02849	10.9193
223.5	-45.1882	-131.41	-37.27029	-120.5069	10.68197	10.80568	11.06182	10.9523
223.0	-41.61	-125.643	-33.56618	-113.6831	10.69173	10.83087	11.09768	10.9878
222.5	-38.2444	-119.768	-30.49824	-106.70966	10.69814	10.85433	11.13071	11.0205
222.0	-34.8868	-113.873	-27.35265	-99.49897	10.70706	10.8784	11.16383	11.0533

221.5	-30.6633	-106.556	-23.87435	-91.34034	10.71515	10.90564	11.2007	11.0898
221.0	-26.9976	-98.8189	-20.45579	-82.57448	10.7247	10.9345	11.23908	11.1278
220.5	-23.7901	-91.3118	-17.36606	-74.12345	10.73447	10.96214	11.27503	11.1634
220.0	-21.1408	-84.7001	-14.94541	-66.67241	10.74077	10.9855	11.30503	11.1931
219.5	-18.3771	-78.0049	-12.67241	-59.25103	10.74455	11.01008	11.33553	11.2233
219.0	-16.3232	-72.26	-10.92294	-53.04793	10.75095	11.03171	11.36189	11.2494
218.5	-13.9958	-65.8941	-9.13426	-46.53241	10.75547	11.05558	11.39028	11.2775
218.0	-11.5485	-59.5784	-7.43194	-40.11862	10.75893	11.08118	11.42007	11.307
217.5	-10.3536	-54.2758	-6.1715	-34.27321	10.7645	11.10515	11.44694	11.3336
217.0	-8.93954	-49.5122	-5.10359	-29.89748	10.77048	11.12616	11.46966	11.3561
216.5	-7.3268	-44.5673	-4.26321	-25.56117	10.77668	11.14789	11.49198	11.3782
216.0	-6.08459	-39.9055	-3.64903	-21.41414	10.78235	11.17012	11.5138	11.3998
215.5	-5.09258	-35.8322	-3.28276	-17.77748	10.79054	11.19277	11.53471	11.4205
215.0	-4.21232	-31.9431	-2.83082	-14.51903	10.7961	11.2148	11.554	11.4396
214.5	-3.22452	-29.3085	-2.48952	-12.06438	10.80051	11.23438	11.56995	11.4554
214.0	-2.83188	-27.6156	-1.92086	-9.97717	10.8044	11.25254	11.58399	11.4693
213.5	-1.98424	-24.7344	-1.52136	-7.39231	10.80797	11.27528	11.60036	11.4855
213.0	-1.61771	-22.1063	-1.60616	-5.52683	10.81185	11.29487	11.61349	11.4985
212.5	-1.12965	-21.522	-1.4758	-4.3011	10.81458	11.31221	11.62419	11.5091
212.0	-0.67363	-20.6059	-1.35384	-3.4601	10.81595	11.33169	11.6354	11.5202
211.5	-0.26639	-19.8043	-1.54553	-2.93961	10.82078	11.3526	11.64651	11.5312
211.0	-0.13889	-19.5	-1.48109	-2.61213	10.82518	11.37504	11.65762	11.5422
210.5	0.27167	-19.2403	-1.59484	-2.69204	10.82729	11.39717	11.66792	11.5524
210.0	0.31567	-18.6805	-1.74001	-3.03714	10.82981	11.41696	11.67621	11.5606
209.5	0.22879	-18.4512	-1.80204	-3.43524	10.83285	11.44297	11.68651	11.5708
209.0	0.71823	-18.7827	-1.99	-4.51666	10.83569	11.46919	11.6961	11.5803
208.5	0.67046	-20.5727	-1.90684	-5.05717	10.83842	11.4952	11.70529	11.5894
208.0	0.40756	-22.0006	-1.92037	-5.53969	10.84094	11.52223	11.71428	11.5983
207.5	0.28155	-22.5051	-1.91658	-6.55131	10.8422	11.54864	11.72267	11.6066
207.0	-0.00791	-24.1432	-1.71305	-6.89486	10.84367	11.57578	11.73075	11.6146

206.5	-7.20E-04	-25.3948	-2.01306	-7.55514	10.84514	11.60362	11.73862	11.6224
206.0	0.19037	-26.7645	-2.11893	-8.611	10.84629	11.63198	11.7462	11.6299
205.5	0.11169	-27.9821	-2.08097	-9.985	10.84745	11.6636	11.75418	11.6378
205.0	-0.01737	-29.7841	-2.1322	-10.72572	10.8486	11.69389	11.76125	11.6448
204.5	-0.17027	-30.8808	-2.13164	-11.41714	10.84976	11.72449	11.76781	11.6513
204.0	-0.30575	-33.0265	-2.05992	-11.87955	10.85028	11.75601	11.77418	11.6576
203.5	-0.51896	-33.3112	-2.01366	-12.48045	10.84839	11.78834	11.78024	11.6636
203.0	-0.13522	-33.3535	-1.90982	-12.67748	10.84692	11.81425	11.78448	11.6678
202.5	-0.34585	-33.6325	-1.73671	-12.79907	10.84629	11.84189	11.78791	11.6712
202.0	-0.06766	-33.8152	-1.48774	-12.50238	10.84587	11.87188	11.79064	11.6739
201.5	1.98E-04	-33.8294	-1.21841	-11.73152	10.84503	11.90044	11.79215	11.6754
201.0	-0.07333	-32.21	-1.00607	-10.63893	10.84188	11.92737	11.79215	11.6754
200.5	0.12739	-30.4792	-0.81418	-9.89128	10.83978	11.95154	11.79084	11.6741
200.0	0.52902	-28.9407	-0.74017	-8.37545	10.83548	11.97245	11.78832	11.6716
199.5	0.84219	-25.3854	-0.55193	-7.46679	10.83086	11.99296	11.78448	11.6678
199.0	0.81068	-20.4154	-0.06509	-6.14976	10.8254	12.01458	11.77862	11.662
198.5	0.90485	-14.3877	0.18601	-4.25683	10.81973	12.03243	11.77216	11.6556
198.0	1.02617	-6.43841	0.69117	-2.11757	10.8128	12.05079	11.76347	11.647
197.5	1.21486	-0.25467	0.96338	-0.48005	10.8065	12.06527	11.75438	11.638
197.0	1.20231	8.73197	1.46076	1.26365	10.79936	12.07813	11.74448	11.6282
196.5	1.5953	18.8792	2.03356	3.73269	10.79211	12.09067	11.73216	11.616
196.0	1.72789	28.2586	2.52347	6.32848	10.78434	12.10036	11.71883	11.6028
195.5	1.51443	37.8707	2.56383	9.07059	10.77563	12.10699	11.70519	11.5893
195.0	1.21038	47.684	2.98279	10.92679	10.76618	12.11128	11.69004	11.5743
194.5	1.15519	58.2257	3.27485	12.53403	10.75715	12.1125	11.6751	11.5595
194.0	0.64943	67.3117	3.69726	14.27686	10.74969	12.11087	11.66075	11.5453
193.5	0.05905	75.8	4.10756	15.95797	10.73866	12.10546	11.645	11.5297
193.0	-0.30181	83.0183	4.65391	16.78934	10.72911	12.09669	11.62874	11.5136
192.5	-1.09964	90.4786	4.91341	17.9229	10.72029	12.08476	11.61237	11.4974
192.0	-1.41997	94.7385	4.97247	18.9531	10.71147	12.06844	11.59601	11.4812

191.5	-2.13451	98.3553	4.77815	19.43803	10.70244	12.04763	11.57884	11.4642
191.0	-2.42337	99.3717	4.98941	20.16648	10.6952	12.02682	11.5644	11.4499
190.5	-2.59195	99.9294	4.9705	20.195	10.69121	12.00938	11.5544	11.44
190.0	-3.33147	99.0067	5.17947	20.07014	10.68774	11.99061	11.54571	11.4314

For DSC thermograms:

Temperature	SA	L-SA
30.002	-0.03293	-0.04059
30.002	-0.03303	-0.04065
30.002	-0.03311	-0.04071
30.002	-0.0332	-0.04077
30.001	-0.03328	-0.04082
30.002	-0.03336	-0.04087
30.002	-0.03345	-0.04093
30.003	-0.03356	-0.04099
30.003	-0.03369	-0.04102
30.002	-0.03381	-0.04106
30.002	-0.03388	-0.04111
30.002	-0.03396	-0.04116
30.003	-0.03403	-0.0412
30.002	-0.03409	-0.04124
30.002	-0.0342	-0.04128
30.004	-0.03433	-0.04134
30.003	-0.03446	-0.04143
30.004	-0.03462	-0.04155
30.004	-0.03478	-0.04164
30.004	-0.03496	-0.04172
30.006	-0.03516	-0.04179
30.006	-0.03541	-0.04188
30.006	-0.03564	-0.04197

30.006	-0.03589	-0.04207
30.007	-0.03617	-0.04217
30.008	-0.03651	-0.04227
30.009	-0.03691	-0.04243
30.009	-0.03741	-0.04263
30.01	-0.03796	-0.04285
30.011	-0.03868	-0.04306
30.013	-0.03963	-0.04332
30.014	-0.0408	-0.04366
30.016	-0.04223	-0.04412
30.018	-0.04418	-0.04475
30.019	-0.04626	-0.04547
30.021	-0.04869	-0.04632
30.023	-0.05167	-0.04739
30.026	-0.05504	-0.04877
30.028	-0.05858	-0.05036
30.03	-0.06219	-0.05209
30.034	-0.06568	-0.05367
30.036	-0.06962	-0.0554
30.04	-0.07287	-0.05725
30.043	-0.07691	-0.05867
30.046	-0.08013	-0.06033
30.05	-0.0846	-0.0621
30.054	-0.08905	-0.06389
30.058	-0.094	-0.06578
30.062	-0.09886	-0.06746
30.068	-0.10397	-0.0697
30.072	-0.10942	-0.0718
30.078	-0.11477	-0.07383
30.083	-0.12045	-0.07605

30.088	-0.12637	-0.07859
30.094	-0.13214	-0.08081
30.101	-0.13814	-0.0833
30.107	-0.14433	-0.08567
30.113	-0.15002	-0.08806
30.121	-0.15644	-0.09077
30.127	-0.16309	-0.09358
30.135	-0.16953	-0.09638
30.142	-0.17589	-0.09907
30.15	-0.18238	-0.10201
30.158	-0.1889	-0.10458
30.166	-0.1954	-0.10758
30.175	-0.20196	-0.11047
30.184	-0.20851	-0.11345
30.193	-0.2154	-0.11635
30.202	-0.2218	-0.11897
30.212	-0.22808	-0.12191
30.221	-0.23519	-0.12457
30.233	-0.24216	-0.12736
30.242	-0.24876	-0.1299
30.252	-0.2554	-0.13258
30.263	-0.26175	-0.13535
30.273	-0.26844	-0.13788
30.283	-0.27484	-0.14062
30.295	-0.28071	-0.1433
30.307	-0.28698	-0.14591
30.318	-0.29277	-0.1484
30.329	-0.29805	-0.15108
30.34	-0.30321	-0.15363
30.352	-0.30816	-0.15571

30.365	-0.31349	-0.15801
30.377	-0.3188	-0.16034
30.389	-0.32361	-0.1625
30.402	-0.32885	-0.16451
30.415	-0.33405	-0.16657
30.428	-0.33851	-0.16841
30.44	-0.34299	-0.17026
30.453	-0.3475	-0.17202
30.466	-0.35157	-0.17381
30.478	-0.35551	-0.17567
30.492	-0.35876	-0.17724
30.505	-0.36193	-0.17897
30.518	-0.36607	-0.18082
30.532	-0.36957	-0.18213
30.545	-0.37274	-0.18371
30.558	-0.37628	-0.18506
30.573	-0.37958	-0.18648
30.587	-0.38278	-0.18779
30.601	-0.38538	-0.18904
30.614	-0.38849	-0.19029
30.628	-0.39062	-0.1913
30.642	-0.3933	-0.19235
30.656	-0.39559	-0.19322
30.671	-0.39842	-0.19418
30.685	-0.40028	-0.19504
30.699	-0.402	-0.19617
30.713	-0.4034	-0.19705
30.728	-0.40523	-0.19802
30.742	-0.40764	-0.19883
30.756	-0.40918	-0.19956

30.771	-0.41058	-0.20037
30.785	-0.41228	-0.20123
30.8	-0.41404	-0.202
30.814	-0.4158	-0.20248
30.829	-0.41744	-0.20303
30.842	-0.41908	-0.20373
30.858	-0.42022	-0.2046
30.872	-0.42113	-0.20536
30.887	-0.42209	-0.20612
30.902	-0.42329	-0.2065
30.916	-0.42429	-0.20711
30.931	-0.42567	-0.20771
30.947	-0.42742	-0.20828
30.961	-0.42931	-0.20901
30.976	-0.43017	-0.20942
30.992	-0.43194	-0.20994
31.006	-0.43323	-0.2104
31.021	-0.43415	-0.21091
31.036	-0.43538	-0.21141
31.051	-0.43652	-0.21197
31.066	-0.43795	-0.21234
31.08	-0.43929	-0.2127
31.096	-0.44022	-0.21345
31.111	-0.44122	-0.2138
31.126	-0.44183	-0.2143
31.142	-0.4422	-0.21461
31.156	-0.44309	-0.21512
31.172	-0.44401	-0.21573
31.186	-0.44433	-0.21611
31.202	-0.44525	-0.21669

31.217	-0.44649	-0.21712
31.233	-0.44811	-0.21769
31.249	-0.4494	-0.21829
31.263	-0.45066	-0.21898
31.28	-0.45143	-0.21933
31.294	-0.45292	-0.2197
31.309	-0.45388	-0.22019
31.325	-0.45526	-0.22062
31.341	-0.45637	-0.22114
31.356	-0.45741	-0.22165
31.371	-0.45817	-0.22205
31.388	-0.45941	-0.22234
31.403	-0.46041	-0.22261
31.418	-0.46115	-0.22295
31.434	-0.4614	-0.22377
31.45	-0.46304	-0.22425
31.465	-0.46346	-0.22454
31.481	-0.46451	-0.22481
31.496	-0.46525	-0.22546
31.513	-0.46651	-0.22552
31.527	-0.46727	-0.22612
31.543	-0.46854	-0.22663
31.558	-0.46937	-0.22706
31.576	-0.47036	-0.22732
31.59	-0.47088	-0.22752
31.606	-0.4713	-0.22799
31.622	-0.47216	-0.22835
31.638	-0.47279	-0.22858
31.654	-0.47349	-0.22892
31.67	-0.47408	-0.22941

31.686	-0.47455	-0.2297
31.701	-0.47508	-0.22996
31.717	-0.47573	-0.2302
31.733	-0.47667	-0.23057
31.749	-0.47735	-0.23097
31.765	-0.47795	-0.23137
31.78	-0.47831	-0.23177
31.796	-0.47862	-0.23221
31.812	-0.47926	-0.23242
31.828	-0.47971	-0.2327
31.844	-0.48047	-0.23298
31.86	-0.48127	-0.23325
31.876	-0.48187	-0.2335
31.892	-0.48247	-0.23371
31.908	-0.48303	-0.23388
31.924	-0.48386	-0.23409
31.939	-0.48448	-0.23459
31.957	-0.48506	-0.23514
31.972	-0.48586	-0.23558
31.988	-0.48656	-0.23586
32.005	-0.48702	-0.236
32.021	-0.48746	-0.2362
32.037	-0.48797	-0.23633
32.053	-0.48835	-0.23636
32.069	-0.48896	-0.23641
32.084	-0.48941	-0.23647
32.101	-0.48978	-0.23661
32.117	-0.49004	-0.23689
32.132	-0.4903	-0.23722
32.149	-0.49075	-0.23758

32.165	-0.49128	-0.23795
32.182	-0.49177	-0.23831
32.198	-0.49209	-0.23857
32.213	-0.49252	-0.23879
32.23	-0.49287	-0.2389
32.246	-0.49325	-0.23896
32.261	-0.49361	-0.23896
32.277	-0.49397	-0.23897
32.294	-0.49412	-0.23907
32.31	-0.49425	-0.2392
32.327	-0.4945	-0.23935
32.342	-0.4949	-0.23954
32.359	-0.49536	-0.23976
32.374	-0.49593	-0.23999
32.391	-0.49641	-0.24017
32.407	-0.49688	-0.24041
32.423	-0.49732	-0.24069
32.44	-0.49764	-0.24094
32.455	-0.49797	-0.24118
32.472	-0.49822	-0.24144
32.488	-0.4984	-0.24169
32.504	-0.49856	-0.24195
32.521	-0.49882	-0.24217
32.537	-0.49918	-0.24237
32.553	-0.49955	-0.24251
32.568	-0.49987	-0.24254
32.586	-0.50016	-0.24263
32.602	-0.50046	-0.24272
32.618	-0.50074	-0.24284
32.634	-0.50102	-0.24296

32.65	-0.50132	-0.24308
32.666	-0.5016	-0.24319
32.683	-0.50179	-0.24333
32.699	-0.50203	-0.24353
32.715	-0.50228	-0.24373
32.731	-0.5025	-0.24388
32.747	-0.50269	-0.244
32.764	-0.50288	-0.24414
32.78	-0.50316	-0.24427
32.797	-0.5034	-0.24446
32.813	-0.50362	-0.24465
32.828	-0.50389	-0.24482
32.845	-0.5041	-0.24496
32.861	-0.50435	-0.24507
32.877	-0.50457	-0.24522
32.893	-0.50479	-0.24537
32.91	-0.50504	-0.24553
32.926	-0.50527	-0.24564
32.943	-0.50546	-0.24576
32.959	-0.50572	-0.24589
32.975	-0.50602	-0.246
32.992	-0.50637	-0.24611
33.007	-0.50667	-0.24622
33.024	-0.50704	-0.24631
33.04	-0.50742	-0.24641
33.055	-0.50775	-0.24655
33.072	-0.50814	-0.24674
33.088	-0.50844	-0.24694
33.105	-0.50875	-0.24712
33.121	-0.50895	-0.24732

33.138	-0.50913	-0.24745
33.155	-0.50935	-0.2476
33.17	-0.50956	-0.24779
33.186	-0.50979	-0.24799
33.203	-0.51004	-0.24817
33.218	-0.51036	-0.24835
33.236	-0.51068	-0.24854
33.252	-0.51095	-0.24869
33.268	-0.51121	-0.2488
33.284	-0.5114	-0.24891
33.301	-0.51152	-0.249
33.316	-0.51168	-0.24906
33.333	-0.51184	-0.24913
33.349	-0.51201	-0.24926
33.366	-0.51218	-0.24943
33.383	-0.51243	-0.24964
33.398	-0.51263	-0.24987
33.415	-0.51281	-0.25004
33.431	-0.51299	-0.25017
33.447	-0.51324	-0.25026
33.463	-0.51353	-0.25036
33.48	-0.51377	-0.25044
33.496	-0.51399	-0.25051
33.512	-0.51425	-0.25056
33.528	-0.51451	-0.25065
33.545	-0.51477	-0.25078
33.561	-0.51506	-0.25092
33.577	-0.51533	-0.25106
33.593	-0.51554	-0.25116
33.611	-0.5157	-0.25131

33.627	-0.51581	-0.25143
33.643	-0.51594	-0.25154
33.658	-0.51608	-0.25165
33.675	-0.51624	-0.25178
33.692	-0.51641	-0.25192
33.708	-0.51662	-0.25205
33.724	-0.51685	-0.25217
33.741	-0.51711	-0.25229
33.758	-0.51741	-0.25241
33.774	-0.51769	-0.25252
33.79	-0.51794	-0.25262
33.806	-0.51814	-0.25277
33.823	-0.51829	-0.25292
33.84	-0.51844	-0.25308
33.856	-0.51858	-0.25328
33.872	-0.51869	-0.25344
33.889	-0.5188	-0.25358
33.904	-0.51896	-0.25371
33.921	-0.51916	-0.25383
33.937	-0.51943	-0.25395
33.952	-0.51964	-0.25405
33.969	-0.51985	-0.25414
33.986	-0.52003	-0.25425
34.002	-0.52022	-0.25435
34.019	-0.52035	-0.25445
34.036	-0.52047	-0.25455
34.052	-0.52058	-0.25465
34.068	-0.52068	-0.25473
34.084	-0.52078	-0.25483
34.101	-0.52092	-0.2549

34.116	-0.52104	-0.25499
34.134	-0.52117	-0.25509
34.149	-0.52128	-0.25521
34.167	-0.52139	-0.25535
34.183	-0.52155	-0.25547
34.199	-0.5217	-0.25558
34.215	-0.5219	-0.25567
34.231	-0.52213	-0.25576
34.247	-0.52232	-0.25583
34.265	-0.52251	-0.25592
34.281	-0.52274	-0.25603
34.297	-0.52297	-0.25614
34.313	-0.52315	-0.25625
34.329	-0.52329	-0.25641
34.346	-0.52347	-0.25657
34.363	-0.52366	-0.25672
34.379	-0.5239	-0.25683
34.395	-0.52417	-0.25693
34.412	-0.5245	-0.25703
34.427	-0.52476	-0.25713
34.444	-0.52497	-0.25725
34.459	-0.52516	-0.25742
34.476	-0.52533	-0.25767
34.493	-0.52547	-0.25787
34.509	-0.52559	-0.25809
34.526	-0.52568	-0.25823
34.543	-0.52576	-0.25834
34.56	-0.52584	-0.25841
34.575	-0.52594	-0.25848
34.592	-0.52608	-0.25853

34.607	-0.52621	-0.25858
34.623	-0.52632	-0.25864
34.641	-0.52646	-0.25873
34.657	-0.52662	-0.25884
34.673	-0.52682	-0.25897
34.69	-0.52707	-0.25914
34.706	-0.52729	-0.25925
34.723	-0.52752	-0.25932
34.739	-0.52776	-0.25937
34.755	-0.52793	-0.25945
34.771	-0.52807	-0.25952
34.788	-0.52819	-0.25958
34.804	-0.52826	-0.25967
34.82	-0.52834	-0.25981
34.836	-0.52844	-0.25995
34.853	-0.52854	-0.2601
34.869	-0.52871	-0.26026
34.886	-0.52895	-0.26039
34.902	-0.52924	-0.26048
34.918	-0.5296	-0.26056
34.935	-0.52993	-0.26067
34.951	-0.53026	-0.26079
34.968	-0.53047	-0.26089
34.985	-0.53065	-0.26098
35.001	-0.53082	-0.26106
35.017	-0.53098	-0.26115
35.033	-0.53107	-0.26126
35.05	-0.53112	-0.26137
35.066	-0.53117	-0.26146
35.082	-0.53126	-0.26156

35.099	-0.53137	-0.26166
35.116	-0.5315	-0.26179
35.131	-0.53159	-0.26193
35.148	-0.53168	-0.26205
35.165	-0.53181	-0.26217
35.181	-0.53195	-0.26228
35.197	-0.53214	-0.26241
35.214	-0.53243	-0.26256
35.23	-0.53277	-0.26271
35.246	-0.533	-0.26284
35.263	-0.53328	-0.26297
35.28	-0.53346	-0.26309
35.295	-0.53372	-0.26323
35.312	-0.53389	-0.26335
35.328	-0.53397	-0.26345
35.346	-0.534	-0.26355
35.361	-0.53405	-0.26365
35.378	-0.53413	-0.2638
35.394	-0.53423	-0.26394
35.411	-0.53435	-0.26405
35.428	-0.53448	-0.26415
35.443	-0.53461	-0.26425
35.46	-0.53475	-0.26437
35.477	-0.53491	-0.26448
35.492	-0.53509	-0.26458
35.509	-0.53532	-0.26466
35.525	-0.53547	-0.26475
35.542	-0.53561	-0.26484
35.558	-0.53577	-0.2649
35.573	-0.5359	-0.26498

35.591	-0.53601	-0.26508
35.607	-0.53614	-0.26521
35.624	-0.53624	-0.26533
35.64	-0.53637	-0.26548
35.657	-0.53651	-0.26565
35.673	-0.5367	-0.26577
35.689	-0.53686	-0.26591
35.705	-0.53699	-0.26603
35.722	-0.53716	-0.26613
35.738	-0.5373	-0.2662
35.755	-0.53746	-0.26626
35.771	-0.53759	-0.26633
35.788	-0.53777	-0.26642
35.804	-0.5379	-0.26649
35.82	-0.53802	-0.26655
35.836	-0.53821	-0.26664
35.854	-0.53847	-0.26673
35.87	-0.53866	-0.26682
35.886	-0.53887	-0.26693
35.902	-0.53913	-0.26706
35.918	-0.53935	-0.2672
35.935	-0.5395	-0.26731
35.952	-0.53968	-0.26746
35.968	-0.53983	-0.26759
35.984	-0.53994	-0.26773
36.001	-0.54004	-0.2679
36.018	-0.54017	-0.26802
36.034	-0.54024	-0.26811
36.05	-0.5403	-0.26817
36.067	-0.54038	-0.26825

36.083	-0.54046	-0.26832
36.1	-0.54059	-0.26838
36.116	-0.54071	-0.26846
36.132	-0.54088	-0.26858
36.149	-0.54104	-0.26873
36.165	-0.5412	-0.26888
36.182	-0.54144	-0.26912
36.198	-0.54171	-0.2693
36.215	-0.54193	-0.26942
36.232	-0.54217	-0.26949
36.247	-0.54241	-0.26955
36.264	-0.54263	-0.26963
36.281	-0.54288	-0.2697
36.297	-0.54308	-0.26978
36.313	-0.54326	-0.26987
36.329	-0.54339	-0.26998
36.346	-0.54353	-0.27006
36.363	-0.5437	-0.27019
36.379	-0.54387	-0.27031
36.395	-0.54402	-0.27041
36.412	-0.54421	-0.27051
36.428	-0.5444	-0.27059
36.444	-0.54466	-0.2707
36.461	-0.54485	-0.27081
36.478	-0.54503	-0.27097
36.494	-0.54515	-0.27113
36.51	-0.54521	-0.27134
36.527	-0.54526	-0.27147
36.544	-0.54534	-0.27164
36.56	-0.54543	-0.27182

36.577	-0.54556	-0.27196
36.593	-0.54575	-0.2721
36.609	-0.54592	-0.27223
36.626	-0.54607	-0.27235
36.643	-0.54618	-0.27246
36.659	-0.54634	-0.27256
36.675	-0.54641	-0.27263
36.692	-0.54647	-0.27271
36.708	-0.54654	-0.27277
36.724	-0.54662	-0.27284
36.741	-0.54672	-0.27292
36.756	-0.54687	-0.27301
36.774	-0.54713	-0.27309
36.79	-0.5474	-0.2732
36.807	-0.54765	-0.27332
36.823	-0.54787	-0.27344
36.839	-0.54807	-0.27352
36.856	-0.54818	-0.27361
36.873	-0.54828	-0.27371
36.889	-0.54843	-0.27385
36.906	-0.54857	-0.27399
36.922	-0.54865	-0.2741
36.939	-0.54875	-0.27421
36.955	-0.54886	-0.27431
36.972	-0.54898	-0.27439
36.988	-0.5491	-0.27447
37.005	-0.54915	-0.27453
37.02	-0.54921	-0.27459
37.037	-0.54928	-0.27465
37.053	-0.54938	-0.27472

37.07	-0.54961	-0.27483
37.087	-0.54987	-0.27494
37.103	-0.55005	-0.27505
37.118	-0.55022	-0.27515
37.136	-0.55039	-0.27528
37.152	-0.55061	-0.27542
37.17	-0.55078	-0.2756
37.185	-0.55093	-0.27577
37.202	-0.5511	-0.27592
37.219	-0.55125	-0.27607
37.236	-0.55137	-0.27621
37.251	-0.55146	-0.27633
37.268	-0.55152	-0.27641
37.285	-0.55157	-0.27647
37.301	-0.5517	-0.27652
37.318	-0.55183	-0.27657
37.334	-0.55189	-0.27664
37.349	-0.55194	-0.27672
37.367	-0.55202	-0.27686
37.383	-0.55214	-0.27697
37.398	-0.55225	-0.27707
37.415	-0.55239	-0.27717
37.433	-0.5525	-0.27724
37.448	-0.55261	-0.2773
37.465	-0.55269	-0.27737
37.482	-0.55278	-0.27747
37.499	-0.55288	-0.27755
37.515	-0.55303	-0.27761
37.532	-0.55312	-0.27771
37.548	-0.55323	-0.27783

37.564	-0.55335	-0.278
37.58	-0.55345	-0.27815
37.597	-0.55354	-0.27826
37.613	-0.55364	-0.27838
37.63	-0.55378	-0.27846
37.647	-0.55393	-0.27854
37.663	-0.5541	-0.27864
37.68	-0.55426	-0.27874
37.697	-0.55445	-0.27887
37.713	-0.55462	-0.279
37.729	-0.55474	-0.27909
37.745	-0.55484	-0.27919
37.762	-0.55492	-0.2793
37.778	-0.55498	-0.27939
37.794	-0.55505	-0.27948
37.812	-0.55512	-0.27957
37.828	-0.55519	-0.27965
37.845	-0.55527	-0.27972
37.861	-0.55536	-0.27984
37.877	-0.55546	-0.28
37.893	-0.55567	-0.28011
37.91	-0.55581	-0.28021
37.926	-0.55596	-0.28031
37.943	-0.55607	-0.28043
37.96	-0.55621	-0.28053
37.977	-0.55635	-0.28061
37.993	-0.55644	-0.28069
38.009	-0.55653	-0.28076
38.026	-0.5566	-0.28083
38.043	-0.55668	-0.28093

38.059	-0.55679	-0.28106
38.075	-0.55694	-0.28121
38.092	-0.55705	-0.28136
38.109	-0.55718	-0.28151
38.125	-0.55731	-0.2816
38.141	-0.55752	-0.28171
38.158	-0.55769	-0.28182
38.175	-0.55786	-0.28195
38.191	-0.55798	-0.28207
38.207	-0.55806	-0.28218
38.224	-0.55814	-0.28228
38.241	-0.55825	-0.28238
38.256	-0.55837	-0.28249
38.273	-0.55844	-0.28262
38.289	-0.5585	-0.28278
38.307	-0.55857	-0.2829
38.322	-0.55865	-0.28301
38.34	-0.55876	-0.28311
38.356	-0.55896	-0.28319
38.372	-0.55914	-0.28329
38.389	-0.55934	-0.28339
38.404	-0.55952	-0.2835
38.422	-0.55967	-0.2836
38.438	-0.55982	-0.2837
38.455	-0.55994	-0.2838
38.471	-0.56011	-0.28393
38.488	-0.5602	-0.28409
38.505	-0.56026	-0.28423
38.521	-0.56033	-0.28436
38.538	-0.5604	-0.28445

38.555	-0.56047	-0.28454
38.571	-0.56055	-0.2846
38.587	-0.56065	-0.28467
38.603	-0.56079	-0.28475
38.62	-0.56093	-0.28482
38.636	-0.56113	-0.28487
38.653	-0.56131	-0.28496
38.669	-0.56147	-0.28505
38.686	-0.56158	-0.28515
38.703	-0.56172	-0.28527
38.719	-0.56186	-0.2854
38.735	-0.56202	-0.28556
38.752	-0.56226	-0.2857
38.769	-0.56245	-0.28586
38.786	-0.5626	-0.28597
38.802	-0.56276	-0.28606
38.818	-0.56289	-0.28612
38.834	-0.56299	-0.28618
38.851	-0.56306	-0.28624
38.867	-0.56312	-0.28631
38.883	-0.56319	-0.28639
38.9	-0.56326	-0.28645
38.917	-0.56338	-0.28652
38.934	-0.56356	-0.28659
38.95	-0.56374	-0.2867
38.967	-0.56387	-0.2868
38.983	-0.56403	-0.28688
39.001	-0.56412	-0.28697
39.017	-0.56422	-0.28706
39.032	-0.5643	-0.28715

39.05	-0.56437	-0.28723
39.066	-0.56447	-0.28732
39.082	-0.56459	-0.28739
39.099	-0.56475	-0.28747
39.116	-0.56495	-0.28756
39.131	-0.56511	-0.2877
39.149	-0.56523	-0.28787
39.165	-0.56532	-0.288
39.181	-0.56539	-0.28814
39.198	-0.56546	-0.28827
39.215	-0.56554	-0.28841
39.231	-0.5656	-0.2885
39.248	-0.56568	-0.28858
39.263	-0.56574	-0.28865
39.281	-0.56581	-0.28872
39.298	-0.56588	-0.28877
39.313	-0.566	-0.28885
39.33	-0.56607	-0.28897
39.346	-0.56614	-0.28912
39.363	-0.56634	-0.28931
39.38	-0.56657	-0.28954
39.396	-0.56684	-0.28972
39.413	-0.56713	-0.2899
39.43	-0.56739	-0.29003
39.446	-0.56757	-0.29014
39.462	-0.56771	-0.29024
39.479	-0.56779	-0.29033
39.496	-0.56786	-0.29042
39.512	-0.56793	-0.29052
39.529	-0.56806	-0.29063

39.546	-0.56812	-0.29078
39.562	-0.56819	-0.2909
39.578	-0.56825	-0.291
39.595	-0.56832	-0.29109
39.612	-0.56839	-0.29117
39.629	-0.56846	-0.29127
39.645	-0.56858	-0.29135
39.661	-0.56878	-0.29144
39.677	-0.56913	-0.29154
39.695	-0.56947	-0.29161
39.711	-0.56974	-0.29169
39.728	-0.56992	-0.29176
39.744	-0.57004	-0.29184
39.761	-0.57012	-0.29191
39.777	-0.57019	-0.292
39.793	-0.57025	-0.2921
39.81	-0.57032	-0.29221
39.826	-0.57038	-0.29232
39.843	-0.57044	-0.29247
39.86	-0.57051	-0.2926
39.876	-0.57058	-0.29274
39.892	-0.57065	-0.29286
39.908	-0.57071	-0.293
39.926	-0.57078	-0.29314
39.943	-0.57085	-0.29327
39.959	-0.57095	-0.29337
39.975	-0.57109	-0.29349
39.992	-0.57121	-0.29358
40.008	-0.57132	-0.29365
40.024	-0.5714	-0.29372

40.04	-0.57148	-0.2938
40.058	-0.57157	-0.29388
40.075	-0.57166	-0.29399
40.091	-0.57173	-0.29412
40.107	-0.57179	-0.29427
40.124	-0.57187	-0.2944
40.14	-0.57196	-0.29452
40.158	-0.57203	-0.29462
40.175	-0.5721	-0.29474
40.19	-0.57221	-0.29482
40.207	-0.57233	-0.29487
40.223	-0.57241	-0.29493
40.24	-0.57248	-0.29498
40.256	-0.57255	-0.29506
40.272	-0.57263	-0.29512
40.29	-0.5727	-0.29521
40.306	-0.57277	-0.2953
40.322	-0.57289	-0.29539
40.338	-0.573	-0.29547
40.356	-0.57308	-0.29557
40.372	-0.57317	-0.29569
40.39	-0.57327	-0.2958
40.406	-0.57335	-0.29595
40.422	-0.57343	-0.29604
40.439	-0.5735	-0.29613
40.454	-0.57359	-0.29621
40.472	-0.57366	-0.2963
40.488	-0.57376	-0.29639
40.505	-0.57387	-0.29647
40.521	-0.57397	-0.29653

40.537	-0.57405	-0.2966
40.554	-0.57412	-0.2967
40.571	-0.57419	-0.2968
40.587	-0.57426	-0.29693
40.604	-0.57436	-0.29702
40.62	-0.57444	-0.2971
40.637	-0.57451	-0.29715
40.654	-0.57458	-0.29722
40.67	-0.57465	-0.29729
40.686	-0.57472	-0.29739
40.704	-0.57479	-0.29749
40.719	-0.57486	-0.29759
40.736	-0.57493	-0.29771
40.753	-0.57501	-0.29788
40.769	-0.57508	-0.29805
40.786	-0.57519	-0.29816
40.802	-0.57532	-0.29827
40.818	-0.57544	-0.29838
40.836	-0.5756	-0.29856
40.852	-0.57572	-0.29873
40.869	-0.5758	-0.29887
40.885	-0.57587	-0.29899
40.901	-0.57595	-0.29912
40.918	-0.57602	-0.29919
40.936	-0.57609	-0.29927
40.951	-0.57616	-0.29935
40.969	-0.57624	-0.2994
40.985	-0.57631	-0.2995
41	-0.5764	-0.29958
41.018	-0.5765	-0.29969

41.034	-0.57665	-0.29982
41.051	-0.57674	-0.29992
41.068	-0.57685	-0.30002
41.083	-0.57695	-0.3001
41.1	-0.5771	-0.30018
41.117	-0.57721	-0.30028
41.134	-0.57731	-0.30039
41.15	-0.57739	-0.30048
41.166	-0.57748	-0.30054
41.184	-0.57755	-0.30062
41.2	-0.57762	-0.30069
41.217	-0.5777	-0.30077
41.233	-0.57778	-0.30084
41.25	-0.57785	-0.30093
41.266	-0.57792	-0.301
41.282	-0.578	-0.30108
41.299	-0.57808	-0.30117
41.316	-0.57816	-0.30127
41.333	-0.57824	-0.30141
41.349	-0.57832	-0.30151
41.365	-0.57843	-0.30162
41.382	-0.57855	-0.30172
41.398	-0.57863	-0.30184
41.415	-0.57871	-0.30194
41.432	-0.5788	-0.30205
41.448	-0.57891	-0.30214
41.464	-0.57901	-0.30224
41.481	-0.57918	-0.30236
41.498	-0.5793	-0.30246
41.514	-0.57938	-0.30257

41.531	-0.57946	-0.30271
41.547	-0.57956	-0.30284
41.564	-0.57964	-0.30295
41.58	-0.57974	-0.30307
41.597	-0.57982	-0.30319
41.613	-0.57992	-0.30331
41.63	-0.58	-0.30342
41.647	-0.58011	-0.30353
41.663	-0.58027	-0.30364
41.68	-0.58047	-0.30371
41.696	-0.58065	-0.3038
41.712	-0.58076	-0.3039
41.729	-0.58086	-0.30399
41.745	-0.58095	-0.30408
41.762	-0.58105	-0.30416
41.779	-0.58114	-0.30423
41.796	-0.58122	-0.30431
41.813	-0.5813	-0.3044
41.829	-0.58138	-0.30453
41.845	-0.58146	-0.30466
41.862	-0.58154	-0.30479
41.879	-0.58162	-0.30496
41.895	-0.58169	-0.30515
41.912	-0.58177	-0.30537
41.929	-0.58184	-0.30556
41.945	-0.58193	-0.30574
41.962	-0.58201	-0.30586
41.978	-0.58209	-0.30596
41.995	-0.58222	-0.30607
42.011	-0.58241	-0.30621

42.027	-0.58258	-0.30633
42.045	-0.58271	-0.3064
42.061	-0.58282	-0.30647
42.077	-0.5829	-0.30654
42.094	-0.58298	-0.30663
42.11	-0.58306	-0.30668
42.127	-0.58315	-0.30672
42.144	-0.58323	-0.30676
42.16	-0.58331	-0.3068
42.177	-0.58339	-0.30687
42.194	-0.58347	-0.30698
42.21	-0.58355	-0.3071
42.227	-0.58363	-0.30724
42.243	-0.58371	-0.30738
42.26	-0.58378	-0.30755
42.276	-0.58386	-0.30774
42.293	-0.58394	-0.30791
42.309	-0.58402	-0.30805
42.326	-0.58413	-0.30813
42.343	-0.58426	-0.3082
42.361	-0.58436	-0.30826
42.377	-0.58444	-0.30833
42.393	-0.58452	-0.30843
42.409	-0.58461	-0.30854
42.426	-0.58468	-0.30864
42.443	-0.58476	-0.30875
42.459	-0.58485	-0.30884
42.476	-0.58492	-0.30892
42.492	-0.585	-0.30899
42.509	-0.58507	-0.30905

42.525	-0.58515	-0.3091
42.542	-0.58525	-0.30914
42.558	-0.58533	-0.30918
42.574	-0.58545	-0.30923
42.591	-0.58557	-0.30931
42.608	-0.58568	-0.30941
42.625	-0.58576	-0.30953
42.641	-0.58584	-0.30961
42.657	-0.58592	-0.30972
42.673	-0.586	-0.30985
42.691	-0.58608	-0.30996
42.708	-0.58616	-0.31011
42.724	-0.58624	-0.31026
42.74	-0.58634	-0.31039
42.757	-0.58644	-0.31047
42.773	-0.58653	-0.31055
42.79	-0.58662	-0.31061
42.806	-0.5867	-0.31066
42.823	-0.58678	-0.31071
42.84	-0.58686	-0.31076
42.856	-0.58694	-0.31083
42.872	-0.58702	-0.31094
42.89	-0.5871	-0.31107
42.906	-0.58717	-0.31123
42.922	-0.58725	-0.31137
42.939	-0.58735	-0.31151
42.955	-0.58745	-0.31164
42.972	-0.58753	-0.31178
42.989	-0.58762	-0.31188
43.005	-0.5877	-0.31197

43.023	-0.58778	-0.31206
43.038	-0.58786	-0.31214
43.055	-0.58794	-0.31222
43.072	-0.58802	-0.31231
43.088	-0.5881	-0.31242
43.105	-0.58818	-0.31255
43.121	-0.58826	-0.31267
43.139	-0.58834	-0.31276
43.156	-0.58842	-0.31285
43.172	-0.5885	-0.31296
43.189	-0.58858	-0.31308
43.205	-0.58866	-0.31323
43.221	-0.58874	-0.31338
43.237	-0.58883	-0.31349
43.254	-0.58891	-0.31358
43.27	-0.58899	-0.31369
43.288	-0.58906	-0.31381
43.303	-0.58914	-0.31395
43.32	-0.58922	-0.31407
43.337	-0.58931	-0.31418
43.354	-0.58939	-0.31431
43.371	-0.58947	-0.31442
43.387	-0.58955	-0.31457
43.405	-0.58963	-0.31469
43.42	-0.58971	-0.31479
43.437	-0.58979	-0.31488
43.453	-0.58988	-0.31497
43.47	-0.58998	-0.31505
43.487	-0.59006	-0.31515
43.503	-0.59014	-0.31523

43.519	-0.59022	-0.31531
43.536	-0.5903	-0.31543
43.553	-0.59037	-0.31554
43.569	-0.59045	-0.31567
43.586	-0.59052	-0.3158
43.603	-0.59059	-0.31592
43.619	-0.59066	-0.31601
43.636	-0.59074	-0.31609
43.652	-0.59082	-0.31616
43.67	-0.5909	-0.31628
43.686	-0.59098	-0.31637
43.703	-0.59106	-0.31646
43.72	-0.59115	-0.31656
43.736	-0.59123	-0.31665
43.753	-0.59131	-0.31675
43.768	-0.59139	-0.31683
43.785	-0.59147	-0.31691
43.802	-0.59155	-0.31698
43.819	-0.59163	-0.31704
43.836	-0.59172	-0.31713
43.852	-0.5918	-0.31725
43.869	-0.59188	-0.31737
43.884	-0.592	-0.31749
43.901	-0.59209	-0.31761
43.917	-0.59218	-0.31772
43.934	-0.59226	-0.31784
43.951	-0.59234	-0.31792
43.968	-0.59242	-0.318
43.984	-0.5925	-0.31807
44.001	-0.59257	-0.31812

44.017	-0.59265	-0.31818
44.034	-0.59273	-0.31826
44.05	-0.59281	-0.31836
44.067	-0.59289	-0.31851
44.084	-0.59297	-0.31871
44.101	-0.59305	-0.31889
44.117	-0.59313	-0.31901
44.133	-0.59322	-0.31911
44.151	-0.5933	-0.31925
44.167	-0.59338	-0.31936
44.183	-0.59346	-0.31945
44.2	-0.59354	-0.31953
44.216	-0.59363	-0.31959
44.233	-0.59371	-0.31964
44.25	-0.59379	-0.31973
44.265	-0.59389	-0.3198
44.282	-0.594	-0.31988
44.299	-0.59409	-0.31997
44.315	-0.59418	-0.32005
44.332	-0.59427	-0.32012
44.349	-0.59435	-0.32021
44.366	-0.59444	-0.3203
44.382	-0.59452	-0.32041
44.4	-0.59459	-0.3205
44.416	-0.59467	-0.32059
44.432	-0.59474	-0.32071
44.448	-0.59481	-0.32082
44.466	-0.59488	-0.32097
44.481	-0.59496	-0.32111
44.498	-0.59503	-0.32128

44.515	-0.59512	-0.32144
44.531	-0.59521	-0.32157
44.548	-0.59532	-0.32169
44.565	-0.59542	-0.32179
44.581	-0.59553	-0.32187
44.598	-0.59565	-0.32192
44.614	-0.59573	-0.32197
44.631	-0.59582	-0.32204
44.648	-0.5959	-0.3221
44.664	-0.59598	-0.32219
44.681	-0.59609	-0.32229
44.697	-0.59623	-0.32244
44.714	-0.59635	-0.32259
44.731	-0.59648	-0.32271
44.747	-0.59659	-0.32286
44.765	-0.59667	-0.32298
44.78	-0.59676	-0.32308
44.798	-0.59684	-0.32318
44.814	-0.59692	-0.3233
44.831	-0.59699	-0.32341
44.847	-0.59708	-0.32353
44.864	-0.59716	-0.32364
44.88	-0.59724	-0.32374
44.897	-0.59732	-0.32384
44.913	-0.5974	-0.32392
44.93	-0.59748	-0.32401
44.947	-0.59756	-0.32408
44.963	-0.59763	-0.32417
44.979	-0.59771	-0.32424
44.996	-0.59778	-0.32433

45.013	-0.59785	-0.32442
45.03	-0.59793	-0.32453
45.046	-0.598	-0.32462
45.062	-0.59808	-0.32472
45.08	-0.59816	-0.32481
45.095	-0.59825	-0.32489
45.112	-0.59833	-0.32496
45.128	-0.5984	-0.32502
45.146	-0.59848	-0.32508
45.162	-0.59856	-0.32515
45.18	-0.59864	-0.32526
45.196	-0.59872	-0.32538
45.212	-0.5988	-0.32551
45.228	-0.59888	-0.32562
45.245	-0.59895	-0.32576
45.262	-0.59903	-0.32591
45.278	-0.59911	-0.32608
45.295	-0.59919	-0.32625
45.311	-0.59927	-0.32639
45.328	-0.59935	-0.32651
45.345	-0.59942	-0.32661
45.361	-0.59951	-0.32673
45.377	-0.59961	-0.32686
45.393	-0.59969	-0.32699
45.411	-0.59978	-0.3271
45.427	-0.59986	-0.32722
45.443	-0.59993	-0.32732
45.46	-0.60001	-0.3274
45.477	-0.60008	-0.32747
45.493	-0.60015	-0.32753

45.511	-0.60023	-0.32761
45.527	-0.6003	-0.32769
45.543	-0.60037	-0.32777
45.56	-0.60045	-0.32787
45.578	-0.60052	-0.32795
45.593	-0.6006	-0.32802
45.609	-0.60067	-0.32808
45.628	-0.60074	-0.32814
45.644	-0.60081	-0.3282
45.66	-0.60089	-0.32826
45.677	-0.60097	-0.32832
45.693	-0.60104	-0.32841
45.71	-0.60112	-0.32852
45.727	-0.60119	-0.32867
45.743	-0.60127	-0.32882
45.759	-0.60134	-0.32898
45.776	-0.60141	-0.32913
45.792	-0.60149	-0.32928
45.809	-0.60156	-0.32942
45.826	-0.60163	-0.32958
45.843	-0.6017	-0.32969
45.86	-0.60178	-0.32977
45.876	-0.60186	-0.32983
45.891	-0.60193	-0.32988
45.909	-0.60201	-0.32996
45.925	-0.60208	-0.33004
45.942	-0.60216	-0.33011
45.959	-0.60223	-0.33021
45.975	-0.60232	-0.33033
45.991	-0.60243	-0.33045

46.008	-0.60251	-0.33059
46.024	-0.60259	-0.33071
46.041	-0.60267	-0.33081
46.058	-0.60274	-0.33089
46.075	-0.60282	-0.33096
46.092	-0.60289	-0.33109
46.108	-0.60296	-0.33124
46.125	-0.60303	-0.33142
46.142	-0.6031	-0.33157
46.158	-0.60317	-0.33175
46.175	-0.60324	-0.33192
46.191	-0.60331	-0.33208
46.208	-0.60339	-0.33225
46.224	-0.60347	-0.33234
46.241	-0.60355	-0.33244
46.257	-0.60362	-0.33251
46.274	-0.60369	-0.33259
46.291	-0.60377	-0.33268
46.307	-0.60384	-0.33278
46.325	-0.60391	-0.33288
46.341	-0.60399	-0.33298
46.357	-0.60406	-0.33308
46.374	-0.60414	-0.33318
46.391	-0.60421	-0.33329
46.407	-0.60428	-0.33339
46.424	-0.60435	-0.33346
46.44	-0.60442	-0.33353
46.457	-0.60449	-0.3336
46.474	-0.60456	-0.33368
46.49	-0.60463	-0.33376

46.507	-0.6047	-0.33385
46.524	-0.60478	-0.33396
46.54	-0.60485	-0.33404
46.556	-0.60493	-0.33412
46.573	-0.60501	-0.33422
46.59	-0.60508	-0.33432
46.606	-0.60514	-0.33439
46.622	-0.60521	-0.33445
46.639	-0.60527	-0.33453
46.655	-0.60533	-0.33459
46.672	-0.6054	-0.33466
46.688	-0.60546	-0.33474
46.705	-0.60552	-0.33483
46.722	-0.60559	-0.33491
46.738	-0.60567	-0.33499
46.755	-0.60576	-0.3351
46.772	-0.60583	-0.3352
46.789	-0.60591	-0.33529
46.805	-0.60599	-0.3354
46.822	-0.60606	-0.33551
46.839	-0.60617	-0.33565
46.855	-0.60626	-0.33579
46.871	-0.60634	-0.33596
46.889	-0.60641	-0.33612
46.904	-0.60649	-0.33627
46.921	-0.60655	-0.3364
46.938	-0.60663	-0.33652
46.954	-0.60669	-0.3366
46.97	-0.60676	-0.33668
46.988	-0.60682	-0.33677

47.004	-0.60688	-0.33686
47.021	-0.60694	-0.33693
47.038	-0.607	-0.337
47.054	-0.60707	-0.33708
47.071	-0.60713	-0.33716
47.089	-0.6072	-0.33729
47.105	-0.60727	-0.33738
47.122	-0.60734	-0.33748
47.138	-0.6074	-0.33756
47.154	-0.60747	-0.33764
47.171	-0.60754	-0.33771
47.187	-0.60761	-0.33781
47.204	-0.60768	-0.33791
47.221	-0.60775	-0.33799
47.237	-0.60782	-0.33808
47.254	-0.60789	-0.33818
47.271	-0.60797	-0.3383
47.287	-0.60807	-0.33843
47.304	-0.60815	-0.33859
47.321	-0.60823	-0.33875
47.337	-0.6083	-0.33898
47.354	-0.60838	-0.33917
47.37	-0.60845	-0.33936
47.387	-0.60852	-0.33951
47.404	-0.60859	-0.33962
47.42	-0.60865	-0.33969
47.437	-0.60873	-0.33975
47.452	-0.60879	-0.33981
47.469	-0.60886	-0.33985
47.486	-0.60893	-0.33991

47.502	-0.60903	-0.33998
47.519	-0.60912	-0.34005
47.536	-0.6092	-0.34012
47.552	-0.60928	-0.34018
47.57	-0.60935	-0.34025
47.586	-0.60941	-0.3403
47.602	-0.60948	-0.34037
47.62	-0.60955	-0.34045
47.636	-0.60961	-0.34059
47.653	-0.60967	-0.3407
47.669	-0.60974	-0.34087
47.686	-0.60981	-0.34103
47.702	-0.60987	-0.34117
47.719	-0.60994	-0.34131
47.735	-0.61001	-0.34148
47.753	-0.61007	-0.34164
47.769	-0.61013	-0.3418
47.785	-0.6102	-0.34191
47.802	-0.61026	-0.34202
47.819	-0.61032	-0.34213
47.836	-0.61038	-0.3422
47.852	-0.61044	-0.34227
47.869	-0.6105	-0.34234
47.885	-0.61057	-0.3424
47.902	-0.61064	-0.34246
47.918	-0.61071	-0.34253
47.935	-0.61077	-0.3426
47.951	-0.61084	-0.34266
47.968	-0.61092	-0.34272
47.984	-0.61099	-0.3428

48.001	-0.61106	-0.34287
48.017	-0.61113	-0.34294
48.034	-0.61119	-0.34299
48.05	-0.61125	-0.34307
48.067	-0.6113	-0.34317
48.083	-0.61136	-0.34326
48.1	-0.61142	-0.34337
48.116	-0.61147	-0.34346
48.133	-0.61153	-0.34357
48.15	-0.61159	-0.3437
48.166	-0.61165	-0.34385
48.182	-0.61171	-0.34398
48.199	-0.61178	-0.34413
48.216	-0.61184	-0.34425
48.233	-0.61191	-0.34437
48.25	-0.61197	-0.34452
48.266	-0.61203	-0.34462
48.283	-0.6121	-0.34469
48.299	-0.61216	-0.34477
48.315	-0.61223	-0.34485
48.333	-0.61228	-0.34492
48.349	-0.61234	-0.34501
48.365	-0.6124	-0.34509
48.383	-0.61246	-0.34517
48.399	-0.61253	-0.34522
48.415	-0.61259	-0.34529
48.433	-0.61266	-0.34538
48.449	-0.61273	-0.34549
48.466	-0.61279	-0.34562
48.482	-0.61286	-0.34573

48.5	-0.61294	-0.34585
48.515	-0.61301	-0.34602
48.533	-0.61308	-0.34622
48.548	-0.61314	-0.34636
48.565	-0.6132	-0.34649
48.582	-0.61325	-0.34658
48.599	-0.61331	-0.34668
48.615	-0.61337	-0.34682
48.631	-0.61343	-0.34694
48.649	-0.61349	-0.34704
48.665	-0.61355	-0.34713
48.682	-0.61362	-0.34721
48.699	-0.61369	-0.34729
48.714	-0.61375	-0.34739
48.731	-0.61382	-0.34749
48.748	-0.61389	-0.34758
48.764	-0.61396	-0.34767
48.782	-0.61403	-0.34778
48.798	-0.6141	-0.34794
48.815	-0.61417	-0.34807
48.832	-0.61424	-0.34821
48.848	-0.6143	-0.34836
48.865	-0.61437	-0.34852
48.882	-0.61444	-0.34869
48.898	-0.61451	-0.34882
48.915	-0.61458	-0.34894
48.931	-0.61464	-0.34904
48.948	-0.6147	-0.3491
48.964	-0.61476	-0.34917
48.981	-0.61481	-0.34924

48.998	-0.61487	-0.34932
49.014	-0.61493	-0.34941
49.031	-0.61499	-0.34949
49.047	-0.61506	-0.3496
49.063	-0.61512	-0.34973
49.081	-0.61519	-0.34985
49.097	-0.61526	-0.35001
49.115	-0.61532	-0.35017
49.131	-0.61539	-0.35034
49.148	-0.61545	-0.3505
49.164	-0.61552	-0.35062
49.18	-0.61559	-0.35073
49.197	-0.61565	-0.35081
49.213	-0.61572	-0.35088
49.231	-0.61578	-0.35093
49.247	-0.61584	-0.35096
49.263	-0.6159	-0.351
49.28	-0.61595	-0.35104
49.297	-0.616	-0.35107
49.313	-0.61605	-0.3511
49.33	-0.6161	-0.35116
49.346	-0.61615	-0.35122
49.363	-0.61621	-0.3513
49.379	-0.61626	-0.35138
49.396	-0.61632	-0.35146
49.413	-0.61637	-0.35152
49.429	-0.61643	-0.35159
49.446	-0.61648	-0.35167
49.464	-0.61654	-0.35175
49.48	-0.6166	-0.35181

49.496	-0.61665	-0.35187
49.513	-0.61671	-0.35194
49.53	-0.61677	-0.35203
49.546	-0.61684	-0.35213
49.562	-0.6169	-0.35221
49.579	-0.61696	-0.35229
49.595	-0.61702	-0.35237
49.612	-0.61707	-0.35246
49.628	-0.61713	-0.35255
49.646	-0.61718	-0.35265
49.662	-0.61724	-0.35274
49.679	-0.61729	-0.35283
49.695	-0.61734	-0.35292
49.711	-0.6174	-0.35302
49.728	-0.61745	-0.35313
49.745	-0.6175	-0.35323
49.761	-0.61756	-0.35332
49.778	-0.61761	-0.35342
49.795	-0.61766	-0.3535
49.811	-0.61772	-0.35358
49.827	-0.61777	-0.35365
49.845	-0.61783	-0.35374
49.862	-0.61788	-0.35385
49.878	-0.61794	-0.35401
49.894	-0.618	-0.35413
49.911	-0.61807	-0.35425
49.928	-0.61813	-0.35438
49.944	-0.61819	-0.35449
49.962	-0.61825	-0.35456
49.978	-0.6183	-0.3546

49.994	-0.61836	-0.35467
50.011	-0.61841	-0.35475
50.026	-0.61846	-0.35481
50.043	-0.61851	-0.35487
50.061	-0.61857	-0.35493
50.077	-0.61862	-0.35503
50.094	-0.61868	-0.35513
50.111	-0.61874	-0.35524
50.127	-0.6188	-0.35531
50.143	-0.61886	-0.35539
50.16	-0.61891	-0.3555
50.176	-0.61896	-0.35565
50.194	-0.61901	-0.35577
50.21	-0.61907	-0.35588
50.226	-0.61912	-0.35598
50.244	-0.61917	-0.35606
50.261	-0.61923	-0.35614
50.278	-0.61928	-0.35621
50.294	-0.61932	-0.35628
50.31	-0.61937	-0.35637
50.327	-0.61942	-0.35646
50.344	-0.61947	-0.35661
50.359	-0.61952	-0.35677
50.376	-0.61957	-0.35692
50.393	-0.61962	-0.35707
50.41	-0.61968	-0.35718
50.427	-0.61973	-0.3573
50.443	-0.61979	-0.3574
50.46	-0.61984	-0.3575
50.476	-0.6199	-0.35757

50.493	-0.61995	-0.35766
50.509	-0.62001	-0.35776
50.526	-0.62006	-0.35789
50.542	-0.62011	-0.35802
50.559	-0.62016	-0.35816
50.576	-0.62022	-0.35828
50.592	-0.62027	-0.35841
50.61	-0.62032	-0.35853
50.626	-0.62037	-0.35862
50.642	-0.62042	-0.35868
50.659	-0.62046	-0.35873
50.676	-0.62051	-0.35877
50.693	-0.62055	-0.35882
50.71	-0.62059	-0.35886
50.725	-0.62063	-0.35893
50.743	-0.62067	-0.35897
50.758	-0.62072	-0.35901
50.775	-0.62076	-0.35906
50.792	-0.62081	-0.35913
50.808	-0.62086	-0.35921
50.824	-0.62092	-0.35932
50.842	-0.62098	-0.3594
50.858	-0.62103	-0.35951
50.875	-0.62108	-0.35963
50.891	-0.62113	-0.35977
50.907	-0.62119	-0.35991
50.925	-0.62123	-0.36003
50.942	-0.62128	-0.36012
50.958	-0.62132	-0.36023
50.975	-0.62136	-0.36033

50.991	-0.6214	-0.36044
51.008	-0.62145	-0.36058
51.024	-0.62149	-0.36068
51.041	-0.62153	-0.3608
51.058	-0.62157	-0.36089
51.075	-0.62161	-0.36098
51.091	-0.62166	-0.36107
51.108	-0.6217	-0.36112
51.124	-0.62175	-0.36115
51.14	-0.6218	-0.36117
51.157	-0.62185	-0.36119
51.174	-0.6219	-0.36121
51.19	-0.62195	-0.36123
51.207	-0.622	-0.36128
51.223	-0.62205	-0.36137
51.24	-0.6221	-0.3615
51.257	-0.62215	-0.36166
51.273	-0.6222	-0.36187
51.29	-0.62225	-0.36204
51.307	-0.6223	-0.36218
51.323	-0.62234	-0.36234
51.34	-0.62239	-0.36247
51.356	-0.62243	-0.36259
51.373	-0.62248	-0.36267
51.389	-0.62253	-0.36275
51.406	-0.62258	-0.36285
51.423	-0.62262	-0.36296
51.44	-0.62267	-0.36307
51.456	-0.62272	-0.36319
51.472	-0.62277	-0.36334

51.489	-0.62281	-0.36346
51.506	-0.62285	-0.36354
51.522	-0.62289	-0.36362
51.54	-0.62293	-0.36371
51.555	-0.62297	-0.36378
51.572	-0.62302	-0.36384
51.589	-0.62307	-0.36391
51.607	-0.62312	-0.36398
51.623	-0.62317	-0.36405
51.64	-0.62322	-0.36412
51.656	-0.62327	-0.36417
51.672	-0.62332	-0.36422
51.688	-0.62337	-0.36429
51.705	-0.62341	-0.36437
51.721	-0.62346	-0.36447
51.738	-0.6235	-0.36454
51.755	-0.62354	-0.36468
51.772	-0.62359	-0.36487
51.788	-0.62363	-0.36509
51.805	-0.62368	-0.36529
51.822	-0.62372	-0.36542
51.839	-0.62376	-0.3655
51.854	-0.6238	-0.36557
51.871	-0.62385	-0.36563
51.889	-0.62389	-0.36569
51.905	-0.62393	-0.36578
51.921	-0.62397	-0.36587
51.937	-0.62401	-0.36597
51.955	-0.62405	-0.36607
51.971	-0.62408	-0.36617

51.988	-0.62412	-0.36627
52.004	-0.62416	-0.3664
52.021	-0.62419	-0.36647
52.037	-0.62423	-0.36655
52.054	-0.62427	-0.36661
52.071	-0.62431	-0.36667
52.087	-0.62435	-0.36673
52.104	-0.62439	-0.36682
52.121	-0.62443	-0.3669
52.137	-0.62448	-0.36704
52.155	-0.62453	-0.36715
52.17	-0.62457	-0.36726
52.187	-0.62462	-0.36737
52.204	-0.62466	-0.36749
52.221	-0.6247	-0.3676
52.237	-0.62474	-0.36771
52.254	-0.62478	-0.36779
52.271	-0.62481	-0.36784
52.287	-0.62485	-0.3679
52.303	-0.62488	-0.36795
52.321	-0.62491	-0.36804
52.337	-0.62495	-0.36814
52.353	-0.62499	-0.36828
52.37	-0.62502	-0.36846
52.386	-0.62506	-0.36865
52.404	-0.6251	-0.36885
52.421	-0.62513	-0.36899
52.437	-0.62517	-0.36911
52.453	-0.62521	-0.36921
52.47	-0.62526	-0.36927

52.487	-0.6253	-0.36932
52.503	-0.62533	-0.36937
52.52	-0.62537	-0.36941
52.537	-0.6254	-0.36947
52.554	-0.62544	-0.36952
52.57	-0.62547	-0.36958
52.586	-0.62551	-0.36964
52.603	-0.62554	-0.36969
52.62	-0.62558	-0.36976
52.637	-0.62562	-0.36982
52.653	-0.62566	-0.36989
52.67	-0.62569	-0.36998
52.686	-0.62573	-0.37007
52.703	-0.62577	-0.37016
52.719	-0.6258	-0.37028
52.736	-0.62584	-0.37037
52.753	-0.62588	-0.37049
52.769	-0.62593	-0.37056
52.786	-0.62597	-0.37063
52.803	-0.62601	-0.37071
52.819	-0.62606	-0.37078
52.836	-0.6261	-0.37086
52.852	-0.62614	-0.37096
52.87	-0.62618	-0.37105
52.886	-0.62621	-0.37112
52.902	-0.62625	-0.3712
52.92	-0.62628	-0.37126
52.936	-0.62632	-0.37134
52.951	-0.62636	-0.3714
52.969	-0.62641	-0.37144

52.984	-0.62645	-0.37147
53.002	-0.62649	-0.37152
53.018	-0.62653	-0.37159
53.034	-0.62657	-0.37166
53.052	-0.62661	-0.37176
53.068	-0.62665	-0.37189
53.085	-0.62667	-0.37206
53.101	-0.6267	-0.37223
53.117	-0.62673	-0.37238
53.134	-0.62676	-0.37251
53.152	-0.62679	-0.37265
53.168	-0.62683	-0.37272
53.185	-0.62687	-0.3728
53.201	-0.62691	-0.37287
53.218	-0.62695	-0.37293
53.234	-0.62699	-0.37299
53.252	-0.62704	-0.37306
53.268	-0.62708	-0.37314
53.284	-0.62711	-0.37325
53.3	-0.62715	-0.37336
53.317	-0.62719	-0.37348
53.335	-0.62723	-0.37358
53.351	-0.62727	-0.37367
53.368	-0.62731	-0.37376
53.384	-0.62734	-0.37384
53.4	-0.62737	-0.3739
53.418	-0.6274	-0.37395
53.434	-0.62744	-0.37399
53.45	-0.62747	-0.37403
53.467	-0.6275	-0.37408

53.483	-0.62753	-0.37413
53.501	-0.62756	-0.37419
53.517	-0.6276	-0.37423
53.533	-0.62764	-0.37429
53.55	-0.62768	-0.37436
53.567	-0.62772	-0.37444
53.583	-0.62776	-0.37453
53.6	-0.62781	-0.37463
53.617	-0.62784	-0.37475
53.633	-0.62788	-0.37487
53.65	-0.62791	-0.37503
53.667	-0.62794	-0.3752
53.682	-0.62797	-0.37538
53.7	-0.628	-0.37549
53.715	-0.62803	-0.3756
53.732	-0.62807	-0.3757
53.749	-0.62811	-0.37579
53.766	-0.62815	-0.3759
53.783	-0.62819	-0.376
53.799	-0.62823	-0.37607
53.816	-0.62826	-0.37613
53.832	-0.6283	-0.37619
53.85	-0.62833	-0.37627
53.866	-0.62836	-0.37636
53.882	-0.62839	-0.37644
53.899	-0.62841	-0.37652
53.916	-0.62844	-0.37665
53.932	-0.62847	-0.37679
53.949	-0.6285	-0.37694
53.965	-0.62852	-0.37706

53.982	-0.62855	-0.37717
53.999	-0.62858	-0.37726
54.015	-0.62861	-0.37735
54.032	-0.62864	-0.37743
54.049	-0.62866	-0.37752
54.065	-0.62869	-0.37757
54.081	-0.62872	-0.37761
54.098	-0.62875	-0.37767
54.115	-0.62877	-0.37774
54.132	-0.6288	-0.37783
54.148	-0.62882	-0.37794
54.165	-0.62885	-0.37806
54.181	-0.62888	-0.37818
54.198	-0.62892	-0.3783
54.214	-0.62896	-0.3784
54.231	-0.62899	-0.37848
54.248	-0.62903	-0.37853
54.265	-0.62906	-0.37857
54.282	-0.6291	-0.37861
54.298	-0.62913	-0.37864
54.313	-0.62916	-0.37867
54.331	-0.62919	-0.37874
54.348	-0.62921	-0.37881
54.364	-0.62924	-0.37892
54.38	-0.62926	-0.37906
54.396	-0.62929	-0.37923
54.413	-0.62931	-0.37934
54.431	-0.62934	-0.37947
54.448	-0.62936	-0.3796
54.464	-0.62939	-0.37971

54.48	-0.62941	-0.37979
54.497	-0.62944	-0.37986
54.514	-0.62947	-0.37992
54.53	-0.6295	-0.37997
54.547	-0.62953	-0.38001
54.563	-0.62955	-0.38007
54.58	-0.62958	-0.3801
54.596	-0.62961	-0.38015
54.613	-0.62963	-0.38022
54.629	-0.62966	-0.3803
54.646	-0.62968	-0.38036
54.662	-0.62971	-0.38044
54.68	-0.62973	-0.38054
54.697	-0.62976	-0.38066
54.712	-0.62978	-0.38077
54.729	-0.62981	-0.38089
54.747	-0.62983	-0.38101
54.763	-0.62986	-0.3811
54.779	-0.62988	-0.3812
54.796	-0.6299	-0.38131
54.814	-0.62992	-0.3814
54.83	-0.62995	-0.38146
54.846	-0.62997	-0.3815
54.863	-0.62999	-0.38154
54.879	-0.63002	-0.38156
54.895	-0.63004	-0.38159
54.914	-0.63007	-0.38165
54.929	-0.6301	-0.38173
54.945	-0.63012	-0.38181
54.962	-0.63015	-0.38189

54.979	-0.63017	-0.38195
54.996	-0.6302	-0.38201
55.013	-0.63024	-0.38211
55.029	-0.63027	-0.3822
55.045	-0.63031	-0.38228
55.062	-0.63034	-0.38236
55.078	-0.63036	-0.38246
55.095	-0.63039	-0.38257
55.113	-0.63041	-0.3827
55.128	-0.63043	-0.38285
55.146	-0.63044	-0.38301
55.162	-0.63046	-0.3831
55.178	-0.63047	-0.38317
55.196	-0.63049	-0.38326
55.212	-0.63051	-0.38334
55.229	-0.63053	-0.38341
55.245	-0.63055	-0.38347
55.262	-0.63058	-0.38353
55.279	-0.63061	-0.38359
55.295	-0.63063	-0.38363
55.312	-0.63066	-0.38368
55.328	-0.63068	-0.38373
55.345	-0.6307	-0.38377
55.362	-0.63072	-0.38382
55.377	-0.63074	-0.38388
55.394	-0.63076	-0.38397
55.41	-0.63078	-0.38407
55.428	-0.6308	-0.38417
55.444	-0.63083	-0.3843
55.462	-0.63086	-0.38445

55.479	-0.63089	-0.38459
55.495	-0.63092	-0.38475
55.512	-0.63094	-0.38486
55.527	-0.63096	-0.38496
55.545	-0.63098	-0.38505
55.562	-0.631	-0.38514
55.578	-0.63102	-0.38521
55.595	-0.63104	-0.38529
55.611	-0.63106	-0.38536
55.628	-0.63109	-0.38541
55.645	-0.63111	-0.38546
55.661	-0.63113	-0.38552
55.678	-0.63115	-0.38559
55.694	-0.63117	-0.38565
55.711	-0.63119	-0.3857
55.727	-0.63121	-0.38577
55.744	-0.63123	-0.38581
55.761	-0.63125	-0.38585
55.777	-0.63126	-0.38591
55.793	-0.63128	-0.38597
55.81	-0.6313	-0.38605
55.827	-0.63132	-0.38617
55.843	-0.63134	-0.38628
55.86	-0.63136	-0.3864
55.877	-0.63137	-0.38649
55.894	-0.63139	-0.38656
55.91	-0.63141	-0.38661
55.927	-0.63144	-0.38666
55.944	-0.63146	-0.38671
55.96	-0.63149	-0.38674

55.976	-0.63151	-0.38679
55.993	-0.63154	-0.38685
56.01	-0.63156	-0.38693
56.028	-0.63158	-0.38703
56.044	-0.6316	-0.38717
56.06	-0.63161	-0.38729
56.077	-0.63162	-0.3874
56.094	-0.63163	-0.3875
56.111	-0.63165	-0.38759
56.127	-0.63167	-0.38765
56.144	-0.63169	-0.38773
56.16	-0.63171	-0.38782
56.177	-0.63173	-0.38789
56.193	-0.63175	-0.38796
56.21	-0.63177	-0.38802
56.227	-0.63179	-0.38809
56.244	-0.63181	-0.38817
56.26	-0.63183	-0.38829
56.276	-0.63185	-0.38835
56.293	-0.63187	-0.3884
56.309	-0.63189	-0.38845
56.326	-0.63191	-0.3885
56.343	-0.63192	-0.38856
56.36	-0.63193	-0.38859
56.375	-0.63195	-0.38863
56.392	-0.63196	-0.38867
56.409	-0.63197	-0.38873
56.426	-0.63198	-0.38881
56.442	-0.632	-0.3889
56.459	-0.63201	-0.38897

56.475	-0.63202	-0.38903
56.492	-0.63204	-0.38912
56.509	-0.63205	-0.38923
56.526	-0.63207	-0.38933
56.542	-0.63208	-0.3894
56.559	-0.63209	-0.38947
56.576	-0.63211	-0.38957
56.592	-0.63212	-0.38966
56.608	-0.63213	-0.38976
56.625	-0.63214	-0.38984
56.643	-0.63215	-0.38991
56.659	-0.63216	-0.38994
56.675	-0.63218	-0.38997
56.692	-0.63219	-0.38999
56.708	-0.63221	-0.39002
56.725	-0.63223	-0.39005
56.742	-0.63225	-0.39008
56.758	-0.63228	-0.39011
56.775	-0.63231	-0.39016
56.791	-0.63233	-0.39022
56.808	-0.63235	-0.3903
56.825	-0.63238	-0.39039
56.841	-0.6324	-0.3905
56.858	-0.63242	-0.39061
56.874	-0.63244	-0.3907
56.891	-0.63245	-0.39079
56.909	-0.63247	-0.39086
56.924	-0.63248	-0.39095
56.942	-0.6325	-0.39103
56.958	-0.63251	-0.39114

56.975	-0.63253	-0.39121
56.991	-0.63254	-0.39127
57.009	-0.63255	-0.39132
57.024	-0.63257	-0.39135
57.041	-0.63258	-0.39138
57.057	-0.63259	-0.39143
57.074	-0.6326	-0.39148
57.091	-0.63261	-0.39153
57.108	-0.63262	-0.39163
57.124	-0.63263	-0.39176
57.141	-0.63264	-0.39189
57.158	-0.63265	-0.39203
57.174	-0.63267	-0.39214
57.191	-0.63268	-0.39225
57.207	-0.63269	-0.39232
57.224	-0.63271	-0.39237
57.241	-0.63272	-0.39242
57.258	-0.63273	-0.39246
57.274	-0.63274	-0.39249
57.291	-0.63275	-0.39254
57.307	-0.63276	-0.39259
57.323	-0.63277	-0.39264
57.34	-0.6328	-0.3927
57.358	-0.63282	-0.39277
57.374	-0.63283	-0.39286
57.391	-0.63285	-0.39295
57.407	-0.63287	-0.39301
57.424	-0.63288	-0.39308
57.441	-0.63289	-0.39315
57.458	-0.6329	-0.39324

57.474	-0.6329	-0.39333
57.49	-0.63291	-0.39339
57.507	-0.63293	-0.39346
57.523	-0.63294	-0.39354
57.54	-0.63295	-0.39362
57.557	-0.63296	-0.39369
57.574	-0.63298	-0.39373
57.591	-0.63299	-0.39376
57.607	-0.633	-0.39383
57.624	-0.63301	-0.39386
57.64	-0.63302	-0.39389
57.657	-0.63303	-0.39392
57.674	-0.63303	-0.39395
57.69	-0.63304	-0.39399
57.706	-0.63305	-0.39407
57.723	-0.63306	-0.39419
57.74	-0.63307	-0.39435
57.757	-0.63308	-0.3945
57.774	-0.63308	-0.3946
57.79	-0.63309	-0.39466
57.807	-0.6331	-0.39471
57.824	-0.6331	-0.39477
57.84	-0.63311	-0.39484
57.856	-0.63312	-0.39488
57.873	-0.63313	-0.39496
57.89	-0.63314	-0.39504
57.907	-0.63314	-0.3951
57.923	-0.63315	-0.39517
57.94	-0.63315	-0.39524
57.957	-0.63316	-0.39529

57.974	-0.63316	-0.39532
57.989	-0.63316	-0.39541
58.006	-0.63317	-0.39543
58.023	-0.63318	-0.39546
58.039	-0.63319	-0.39549
58.056	-0.63321	-0.39553
58.072	-0.63323	-0.39559
58.089	-0.63324	-0.39564
58.106	-0.63325	-0.39571
58.122	-0.63326	-0.3958
58.139	-0.63326	-0.39586
58.156	-0.63327	-0.39592
58.172	-0.63328	-0.39597
58.189	-0.63328	-0.39603
58.205	-0.63328	-0.39606
58.222	-0.63329	-0.39613
58.239	-0.6333	-0.39617
58.256	-0.63331	-0.39624
58.272	-0.63332	-0.3963
58.288	-0.63333	-0.39636
58.305	-0.63334	-0.39642
58.322	-0.63335	-0.39649
58.339	-0.63336	-0.39655
58.355	-0.63337	-0.39661
58.371	-0.63338	-0.39668
58.389	-0.63339	-0.39675
58.406	-0.63339	-0.3968
58.422	-0.6334	-0.39683
58.438	-0.63341	-0.39686
58.456	-0.63341	-0.3969

58.472	-0.63342	-0.39693
58.488	-0.63343	-0.39697
58.505	-0.63343	-0.39703
58.522	-0.63343	-0.39706
58.537	-0.63344	-0.39709
58.555	-0.63344	-0.39712
58.571	-0.63344	-0.39717
58.587	-0.63344	-0.39721
58.604	-0.63343	-0.39727
58.62	-0.63344	-0.39732
58.637	-0.63344	-0.39738
58.654	-0.63345	-0.39746
58.671	-0.63345	-0.39756
58.688	-0.63345	-0.39767
58.705	-0.63345	-0.39777
58.722	-0.63345	-0.39783
58.738	-0.63345	-0.39788
58.754	-0.63346	-0.39791
58.771	-0.63346	-0.39795
58.788	-0.63346	-0.39799
58.803	-0.63347	-0.39803
58.821	-0.63347	-0.39809
58.837	-0.63348	-0.39816
58.855	-0.63349	-0.39822
58.871	-0.6335	-0.39831
58.887	-0.6335	-0.39843
58.904	-0.63351	-0.39855
58.921	-0.63352	-0.39871
58.937	-0.63352	-0.39879
58.954	-0.63352	-0.39889

58.972	-0.63353	-0.39896
58.988	-0.63353	-0.39899
59.005	-0.63353	-0.39902
59.02	-0.63353	-0.39906
59.038	-0.63352	-0.39909
59.055	-0.63352	-0.39912
59.07	-0.63351	-0.39915
59.087	-0.6335	-0.39919
59.105	-0.6335	-0.39926
59.121	-0.63349	-0.39933
59.138	-0.63349	-0.39943
59.153	-0.6335	-0.39953
59.169	-0.6335	-0.39962
59.188	-0.63351	-0.3997
59.203	-0.63352	-0.39979
59.221	-0.63352	-0.39986
59.237	-0.63352	-0.39994
59.254	-0.63352	-0.40001
59.27	-0.63353	-0.40007
59.288	-0.63352	-0.40012
59.304	-0.63352	-0.40016
59.321	-0.63352	-0.4002
59.337	-0.63352	-0.40023
59.353	-0.63352	-0.40028
59.37	-0.63352	-0.40031
59.387	-0.63352	-0.40035
59.403	-0.63352	-0.40039
59.42	-0.63351	-0.40045
59.436	-0.6335	-0.40051
59.453	-0.6335	-0.40059

59.47	-0.63349	-0.40069
59.487	-0.63348	-0.40079
59.503	-0.63348	-0.40087
59.52	-0.63347	-0.40092
59.538	-0.63347	-0.40096
59.554	-0.63347	-0.40101
59.57	-0.63347	-0.40104
59.587	-0.63348	-0.40107
59.604	-0.63348	-0.40112
59.62	-0.63348	-0.40115
59.637	-0.63348	-0.4012
59.654	-0.63348	-0.40126
59.67	-0.63348	-0.40134
59.687	-0.63347	-0.40143
59.703	-0.63347	-0.4015
59.72	-0.63347	-0.40156
59.738	-0.63347	-0.40163
59.754	-0.63347	-0.4017
59.77	-0.63346	-0.40173
59.787	-0.63345	-0.40177
59.803	-0.63345	-0.4018
59.82	-0.63345	-0.40184
59.836	-0.63346	-0.40187
59.853	-0.63346	-0.40192
59.871	-0.63347	-0.40197
59.887	-0.63347	-0.40202
59.904	-0.63348	-0.40208
59.92	-0.63349	-0.40216
59.937	-0.6335	-0.40223
59.953	-0.6335	-0.40228

59.97	-0.63349	-0.40234
59.987	-0.63348	-0.40238
60.004	-0.63348	-0.40241
60.02	-0.63347	-0.40246
60.036	-0.63347	-0.4025
60.054	-0.63346	-0.40253
60.071	-0.63346	-0.40259
60.086	-0.63345	-0.40262
60.103	-0.63345	-0.40265
60.12	-0.63345	-0.40269
60.136	-0.63344	-0.40272
60.153	-0.63344	-0.40276
60.169	-0.63343	-0.40279
60.186	-0.63343	-0.40283
60.203	-0.63343	-0.40286
60.219	-0.63343	-0.40291
60.236	-0.63342	-0.40294
60.253	-0.63341	-0.403
60.269	-0.63341	-0.40305
60.287	-0.6334	-0.40309
60.303	-0.63338	-0.40313
60.319	-0.63337	-0.40316
60.337	-0.63336	-0.4032
60.353	-0.63336	-0.40324
60.369	-0.63335	-0.40327
60.386	-0.63335	-0.40332
60.402	-0.63335	-0.40337
60.42	-0.63335	-0.40341
60.436	-0.63335	-0.40346
60.453	-0.63335	-0.40352

60.469	-0.63335	-0.40357
60.486	-0.63335	-0.40361
60.503	-0.63334	-0.40365
60.52	-0.63333	-0.40369
60.536	-0.63331	-0.40372
60.553	-0.6333	-0.40376
60.569	-0.63329	-0.4038
60.586	-0.63327	-0.40383
60.603	-0.63326	-0.40387
60.619	-0.63325	-0.40391
60.637	-0.63324	-0.40396
60.653	-0.63324	-0.404
60.669	-0.63324	-0.40404
60.685	-0.63323	-0.40407
60.702	-0.63322	-0.40411
60.719	-0.63321	-0.40415
60.734	-0.6332	-0.40419
60.752	-0.6332	-0.40423
60.769	-0.6332	-0.40428
60.785	-0.63319	-0.40433
60.802	-0.63319	-0.40438
60.819	-0.6332	-0.40444
60.836	-0.6332	-0.40449
60.853	-0.63319	-0.40453
60.869	-0.63318	-0.40457
60.885	-0.63316	-0.40462
60.902	-0.63314	-0.40466
60.918	-0.63312	-0.40469
60.935	-0.63309	-0.40473
60.952	-0.63307	-0.40477

60.968	-0.63306	-0.40481
60.985	-0.63304	-0.40486
61.002	-0.63304	-0.40494
61.019	-0.63304	-0.40504
61.035	-0.63303	-0.40512
61.052	-0.63302	-0.40516
61.068	-0.63302	-0.4052
61.085	-0.63301	-0.40524
61.102	-0.63301	-0.40529
61.118	-0.633	-0.40532
61.135	-0.633	-0.40536
61.152	-0.633	-0.40539
61.169	-0.63299	-0.40543
61.185	-0.63299	-0.40546
61.202	-0.63298	-0.4055
61.218	-0.63298	-0.40554
61.234	-0.63297	-0.40557
61.251	-0.63296	-0.40561
61.268	-0.63294	-0.40565
61.284	-0.63293	-0.40569
61.301	-0.63292	-0.40572
61.318	-0.63292	-0.40576
61.335	-0.63291	-0.4058
61.351	-0.63291	-0.40583
61.368	-0.63291	-0.40587
61.385	-0.63291	-0.40592
61.401	-0.6329	-0.40597
61.418	-0.63289	-0.40603
61.434	-0.63288	-0.40611
61.451	-0.63287	-0.40619

61.468	-0.63286	-0.40626
61.485	-0.63284	-0.4063
61.501	-0.63283	-0.40634
61.518	-0.63282	-0.40638
61.535	-0.63281	-0.40641
61.551	-0.63281	-0.40645
61.568	-0.6328	-0.40649
61.585	-0.6328	-0.40653
61.602	-0.63279	-0.40656
61.619	-0.63278	-0.4066
61.635	-0.63277	-0.40664
61.651	-0.63276	-0.40668
61.668	-0.63275	-0.40672
61.684	-0.63273	-0.40675
61.701	-0.63272	-0.40679
61.717	-0.63271	-0.40683
61.734	-0.63269	-0.40687
61.751	-0.63269	-0.40691
61.768	-0.63268	-0.40694
61.784	-0.63267	-0.40699
61.802	-0.63266	-0.40705
61.818	-0.63265	-0.40712
61.834	-0.63265	-0.40719
61.851	-0.63264	-0.40725
61.867	-0.63263	-0.40729
61.884	-0.63263	-0.40733
61.901	-0.63262	-0.40736
61.918	-0.63262	-0.4074
61.934	-0.63261	-0.40743
61.951	-0.6326	-0.40747

61.968	-0.63259	-0.4075
61.984	-0.63257	-0.40754
62.001	-0.63256	-0.40758
62.017	-0.63254	-0.40761
62.035	-0.63252	-0.40766
62.051	-0.6325	-0.4077
62.069	-0.63249	-0.40774
62.085	-0.63248	-0.40777
62.1	-0.63247	-0.40781
62.118	-0.63246	-0.40786
62.134	-0.63245	-0.40791
62.151	-0.63244	-0.40796
62.167	-0.63243	-0.40801
62.185	-0.63243	-0.40805
62.201	-0.63241	-0.40809
62.217	-0.6324	-0.40813
62.233	-0.63238	-0.40816
62.251	-0.63236	-0.4082
62.268	-0.63234	-0.40823
62.284	-0.63231	-0.40827
62.301	-0.6323	-0.4083
62.317	-0.63229	-0.40834
62.333	-0.63228	-0.40838
62.35	-0.63227	-0.40841
62.367	-0.63226	-0.40845
62.385	-0.63225	-0.40849
62.402	-0.63224	-0.40853
62.417	-0.63223	-0.40857
62.434	-0.63222	-0.4086
62.45	-0.6322	-0.40864

62.467	-0.63219	-0.40868
62.484	-0.63217	-0.40871
62.502	-0.63216	-0.40875
62.517	-0.63214	-0.40879
62.534	-0.63213	-0.40882
62.55	-0.63211	-0.40886
62.567	-0.63209	-0.40889
62.584	-0.63208	-0.40893
62.6	-0.63206	-0.40896
62.617	-0.63204	-0.409
62.634	-0.63202	-0.40903
62.65	-0.63201	-0.40907
62.667	-0.63199	-0.40911
62.684	-0.63197	-0.40914
62.7	-0.63195	-0.40917
62.716	-0.63193	-0.40921
62.733	-0.63191	-0.40924
62.749	-0.63189	-0.40928
62.766	-0.63187	-0.40931
62.782	-0.63186	-0.40935
62.8	-0.63184	-0.40939
62.816	-0.63183	-0.40942
62.833	-0.63182	-0.40946
62.849	-0.63181	-0.4095
62.866	-0.63179	-0.40954
62.883	-0.63178	-0.40957
62.9	-0.63176	-0.40961
62.916	-0.63175	-0.40965
62.933	-0.63173	-0.40969
62.95	-0.63171	-0.40972

62.966	-0.63169	-0.40976
62.983	-0.63166	-0.40979
62.999	-0.63164	-0.40983
63.016	-0.63162	-0.40987
63.032	-0.63159	-0.40991
63.049	-0.63157	-0.40994
63.066	-0.63154	-0.40998
63.083	-0.63152	-0.41002
63.099	-0.6315	-0.41005
63.116	-0.63148	-0.41009
63.133	-0.63147	-0.41012
63.149	-0.63146	-0.41016
63.166	-0.63145	-0.41019
63.183	-0.63144	-0.41023
63.2	-0.63143	-0.41026
63.216	-0.63143	-0.4103
63.232	-0.63141	-0.41033
63.249	-0.6314	-0.4104
63.266	-0.63138	-0.41047
63.283	-0.63136	-0.41053
63.299	-0.63134	-0.41057
63.316	-0.63132	-0.41061
63.333	-0.6313	-0.41064
63.35	-0.63128	-0.41068
63.366	-0.63126	-0.41071
63.383	-0.63124	-0.41074
63.399	-0.63123	-0.41078
63.417	-0.63121	-0.41081
63.432	-0.63119	-0.41085
63.45	-0.63118	-0.4109

63.466	-0.63116	-0.41094
63.483	-0.63114	-0.41098
63.5	-0.63112	-0.41104
63.518	-0.63109	-0.41108
63.534	-0.63108	-0.41111
63.549	-0.63106	-0.41115
63.567	-0.63103	-0.41119
63.583	-0.63101	-0.41123
63.599	-0.63099	-0.41126
63.616	-0.63097	-0.4113
63.632	-0.63095	-0.41133
63.65	-0.63094	-0.41136
63.667	-0.63092	-0.41139
63.684	-0.63091	-0.41142
63.7	-0.63089	-0.41145
63.716	-0.63087	-0.41148
63.733	-0.63085	-0.41151
63.75	-0.63083	-0.41154
63.767	-0.63081	-0.41157
63.783	-0.63079	-0.41161
63.8	-0.63076	-0.41164
63.817	-0.63074	-0.41168
63.833	-0.63071	-0.41171
63.85	-0.63069	-0.41175
63.866	-0.63067	-0.41179
63.883	-0.63065	-0.41182
63.9	-0.63063	-0.41185
63.916	-0.63061	-0.41188
63.933	-0.63059	-0.41191
63.95	-0.63057	-0.41194

63.967	-0.63055	-0.41197
63.984	-0.63053	-0.412
64	-0.6305	-0.41204
64.017	-0.63048	-0.41207
64.033	-0.63045	-0.4121
64.05	-0.63043	-0.41213
64.066	-0.63041	-0.41216
64.084	-0.63038	-0.41219
64.1	-0.63036	-0.41223
64.117	-0.63034	-0.41226
64.133	-0.63032	-0.41229
64.15	-0.6303	-0.41232
64.166	-0.63027	-0.41235
64.183	-0.63025	-0.41238
64.2	-0.63022	-0.41241
64.216	-0.63019	-0.41244
64.233	-0.63015	-0.41247
64.25	-0.63012	-0.4125
64.267	-0.63009	-0.41253
64.283	-0.63007	-0.41256
64.3	-0.63005	-0.41259
64.317	-0.63002	-0.41261
64.333	-0.63	-0.41264
64.349	-0.62997	-0.41267
64.366	-0.62995	-0.41269
64.383	-0.62992	-0.41272
64.399	-0.6299	-0.41275
64.416	-0.62987	-0.41277
64.434	-0.62985	-0.4128
64.45	-0.62982	-0.41283

64.466	-0.6298	-0.41287
64.483	-0.62977	-0.4129
64.499	-0.62974	-0.41293
64.516	-0.6297	-0.41296
64.532	-0.62968	-0.41299
64.549	-0.62965	-0.41302
64.566	-0.62962	-0.41305
64.584	-0.62959	-0.41308
64.6	-0.62957	-0.4131
64.616	-0.62954	-0.41313
64.633	-0.62952	-0.41316
64.649	-0.6295	-0.41318
64.666	-0.62948	-0.41321
64.683	-0.62945	-0.41323
64.699	-0.62943	-0.41326
64.716	-0.6294	-0.41329
64.733	-0.62938	-0.41332
64.75	-0.62935	-0.41335
64.767	-0.62933	-0.41337
64.784	-0.62931	-0.4134
64.8	-0.62929	-0.41343
64.817	-0.62926	-0.41345
64.834	-0.62924	-0.41347
64.85	-0.62921	-0.4135
64.866	-0.62918	-0.41353
64.883	-0.62916	-0.41355
64.9	-0.62913	-0.41358
64.917	-0.6291	-0.41361
64.933	-0.62907	-0.41365
64.95	-0.62904	-0.41369

64.967	-0.62901	-0.41371
64.984	-0.62898	-0.41374
65	-0.62896	-0.41377
65.016	-0.62893	-0.4138
65.033	-0.6289	-0.41382
65.05	-0.62888	-0.41385
65.066	-0.62885	-0.41387
65.083	-0.62882	-0.4139
65.1	-0.6288	-0.41393
65.117	-0.62877	-0.41395
65.133	-0.62874	-0.41398
65.15	-0.6287	-0.41401
65.166	-0.62868	-0.41403
65.182	-0.62865	-0.41406
65.2	-0.62862	-0.41409
65.217	-0.6286	-0.41412
65.233	-0.62857	-0.41415
65.25	-0.62855	-0.41418
65.266	-0.62852	-0.4142
65.282	-0.6285	-0.41423
65.301	-0.62847	-0.41424
65.317	-0.62844	-0.41427
65.334	-0.62841	-0.41429
65.35	-0.62838	-0.41431
65.366	-0.62835	-0.41433
65.384	-0.62832	-0.41436
65.4	-0.62829	-0.41438
65.417	-0.62827	-0.41441
65.433	-0.62824	-0.41443
65.449	-0.62821	-0.41446

65.466	-0.62818	-0.41448
65.483	-0.62815	-0.4145
65.5	-0.62812	-0.41452
65.517	-0.62809	-0.41454
65.532	-0.62806	-0.41457
65.55	-0.62803	-0.41459
65.567	-0.628	-0.41461
65.583	-0.62797	-0.41464
65.6	-0.62794	-0.41466
65.616	-0.62791	-0.41469
65.634	-0.62788	-0.41471
65.65	-0.62785	-0.41473
65.666	-0.62782	-0.41475
65.682	-0.62779	-0.41477
65.7	-0.62776	-0.41479
65.716	-0.62773	-0.41481
65.733	-0.6277	-0.41483
65.75	-0.62767	-0.41485
65.766	-0.62764	-0.41487
65.784	-0.62762	-0.41489
65.8	-0.62759	-0.41491
65.816	-0.62756	-0.41493
65.833	-0.62754	-0.41496
65.85	-0.62751	-0.41498
65.866	-0.62748	-0.415
65.883	-0.62745	-0.41502
65.9	-0.62742	-0.41504
65.916	-0.62739	-0.41506
65.933	-0.62736	-0.41508
65.949	-0.62732	-0.4151

65.966	-0.6273	-0.41511
65.983	-0.62726	-0.41513
65.999	-0.62723	-0.41515
66.016	-0.6272	-0.41517
66.033	-0.62716	-0.41519
66.05	-0.62713	-0.41521
66.067	-0.62709	-0.41523
66.083	-0.62706	-0.41526
66.1	-0.62703	-0.41528
66.117	-0.627	-0.4153
66.133	-0.62696	-0.41531
66.15	-0.62693	-0.41533
66.166	-0.6269	-0.41534
66.183	-0.62687	-0.41536
66.201	-0.62683	-0.41537
66.217	-0.6268	-0.41539
66.232	-0.62677	-0.41541
66.25	-0.62673	-0.41543
66.266	-0.6267	-0.41544
66.283	-0.62667	-0.41546
66.3	-0.62663	-0.41548
66.317	-0.6266	-0.4155
66.334	-0.62657	-0.41552
66.351	-0.62653	-0.41554
66.367	-0.6265	-0.41556
66.384	-0.62648	-0.41557
66.4	-0.62645	-0.41559
66.417	-0.62642	-0.41561
66.432	-0.6264	-0.41563
66.449	-0.62638	-0.41564

66.466	-0.62636	-0.41566
66.483	-0.62633	-0.41568
66.499	-0.6263	-0.41569
66.516	-0.62627	-0.41571
66.534	-0.62623	-0.41572
66.55	-0.6262	-0.41574
66.566	-0.62617	-0.41575
66.583	-0.62614	-0.41577
66.6	-0.62611	-0.41578
66.617	-0.62607	-0.41579
66.634	-0.62603	-0.41581
66.65	-0.62598	-0.41583
66.667	-0.62594	-0.41585
66.684	-0.62589	-0.41587
66.701	-0.62582	-0.41589
66.717	-0.62577	-0.4159
66.733	-0.62574	-0.41592
66.75	-0.6257	-0.41593
66.767	-0.62567	-0.41595
66.785	-0.62565	-0.41596
66.801	-0.62562	-0.41597
66.817	-0.62559	-0.41598
66.833	-0.62556	-0.41599
66.851	-0.62553	-0.416
66.866	-0.6255	-0.41601
66.884	-0.62547	-0.41603
66.9	-0.62544	-0.41605
66.916	-0.62541	-0.41606
66.934	-0.62538	-0.41608
66.95	-0.62536	-0.41609

66.966	-0.62533	-0.4161
66.984	-0.6253	-0.41611
67	-0.62527	-0.41613
67.016	-0.62524	-0.41614
67.033	-0.62521	-0.41615
67.05	-0.62517	-0.41616
67.067	-0.62514	-0.41617
67.083	-0.6251	-0.41619
67.101	-0.62507	-0.4162
67.117	-0.62503	-0.41621
67.134	-0.62499	-0.41623
67.151	-0.62495	-0.41624
67.167	-0.62491	-0.41625
67.184	-0.62487	-0.41627
67.2	-0.62484	-0.41628
67.218	-0.62481	-0.41629
67.233	-0.62478	-0.41631
67.25	-0.62475	-0.41632
67.267	-0.62472	-0.41633
67.284	-0.6247	-0.41634
67.301	-0.62467	-0.41636
67.317	-0.62464	-0.41637
67.334	-0.62461	-0.41638
67.351	-0.62458	-0.41639
67.368	-0.62455	-0.4164
67.384	-0.62451	-0.41641
67.4	-0.62447	-0.41641
67.417	-0.62443	-0.41642
67.433	-0.6244	-0.41643
67.451	-0.62436	-0.41645

67.468	-0.62433	-0.41646
67.484	-0.6243	-0.41647
67.501	-0.62426	-0.41648
67.518	-0.62422	-0.41649
67.534	-0.62418	-0.4165
67.551	-0.62414	-0.41651
67.568	-0.6241	-0.41653
67.584	-0.62406	-0.41653
67.601	-0.62402	-0.41654
67.618	-0.62399	-0.41654
67.634	-0.62396	-0.41655
67.651	-0.62393	-0.41656
67.667	-0.6239	-0.41657
67.684	-0.62387	-0.41658
67.701	-0.62385	-0.41659
67.718	-0.62382	-0.4166
67.734	-0.62378	-0.41661
67.751	-0.62375	-0.41661
67.767	-0.62371	-0.41662
67.785	-0.62367	-0.41663
67.801	-0.62363	-0.41663
67.817	-0.6236	-0.41664
67.834	-0.62356	-0.41664
67.851	-0.62352	-0.41665
67.868	-0.62349	-0.41666
67.885	-0.62346	-0.41666
67.901	-0.62343	-0.41666
67.918	-0.6234	-0.41667
67.934	-0.62336	-0.41667
67.951	-0.62333	-0.41668

67.967	-0.6233	-0.41669
67.985	-0.62326	-0.41669
68.001	-0.62322	-0.4167
68.018	-0.62319	-0.4167
68.035	-0.62315	-0.41671
68.051	-0.62311	-0.41671
68.067	-0.62307	-0.41671
68.085	-0.62303	-0.41672
68.102	-0.62299	-0.41672
68.118	-0.62296	-0.41672
68.135	-0.62292	-0.41673
68.152	-0.62289	-0.41673
68.168	-0.62285	-0.41674
68.185	-0.62282	-0.41674
68.201	-0.62278	-0.41674
68.217	-0.62274	-0.41674
68.234	-0.6227	-0.41675
68.251	-0.62265	-0.41675
68.268	-0.62261	-0.41676
68.284	-0.62257	-0.41676
68.302	-0.62253	-0.41676
68.318	-0.62248	-0.41676
68.334	-0.62245	-0.41677
68.351	-0.62241	-0.41677
68.368	-0.62237	-0.41677
68.385	-0.62234	-0.41678
68.402	-0.6223	-0.41678
68.418	-0.62225	-0.41679
68.435	-0.62221	-0.41679
68.452	-0.62217	-0.41679

68.468	-0.62213	-0.41679
68.485	-0.62209	-0.41678
68.501	-0.62205	-0.41678
68.518	-0.62202	-0.41678
68.535	-0.62198	-0.41678
68.551	-0.62194	-0.41678
68.568	-0.62191	-0.41677
68.585	-0.62187	-0.41677
68.601	-0.62182	-0.41678
68.618	-0.62177	-0.41678
68.634	-0.62173	-0.41678
68.651	-0.62168	-0.41678
68.667	-0.62164	-0.41679
68.684	-0.6216	-0.41679
68.701	-0.62157	-0.41679
68.718	-0.62154	-0.41679
68.734	-0.62151	-0.41679
68.751	-0.62148	-0.41678
68.767	-0.62145	-0.41678
68.785	-0.62142	-0.41678
68.801	-0.62138	-0.41678
68.818	-0.62134	-0.41678
68.834	-0.6213	-0.41678
68.851	-0.62125	-0.41679
68.867	-0.62121	-0.41679
68.884	-0.62116	-0.41679
68.902	-0.62111	-0.41679
68.917	-0.62106	-0.41678
68.935	-0.62101	-0.41678
68.951	-0.62096	-0.41678

68.968	-0.62091	-0.41677
68.984	-0.62086	-0.41677
69.001	-0.62081	-0.41677
69.017	-0.62076	-0.41676
69.035	-0.62071	-0.41676
69.051	-0.62067	-0.41676
69.068	-0.62061	-0.41676
69.084	-0.62055	-0.41676
69.101	-0.6205	-0.41676
69.117	-0.62045	-0.41676
69.135	-0.6204	-0.41676
69.152	-0.62035	-0.41676
69.168	-0.6203	-0.41676
69.184	-0.62024	-0.41676
69.202	-0.62019	-0.41676
69.218	-0.62013	-0.41676
69.235	-0.62007	-0.41675
69.251	-0.62001	-0.41674
69.269	-0.61996	-0.41674
69.285	-0.61991	-0.41673
69.302	-0.61986	-0.41673
69.318	-0.61982	-0.41672
69.335	-0.61977	-0.41672
69.352	-0.61973	-0.41672
69.368	-0.61968	-0.41671
69.384	-0.61964	-0.41671
69.401	-0.61958	-0.41671
69.419	-0.61953	-0.4167
69.435	-0.61948	-0.4167
69.452	-0.61943	-0.4167

69.468	-0.61938	-0.41669
69.485	-0.61933	-0.41669
69.502	-0.61928	-0.41668
69.518	-0.61922	-0.41668
69.535	-0.61917	-0.41667
69.551	-0.61911	-0.41667
69.568	-0.61906	-0.41667
69.585	-0.61901	-0.41666
69.602	-0.61896	-0.41666
69.618	-0.61892	-0.41666
69.635	-0.61887	-0.41666
69.653	-0.61882	-0.41666
69.669	-0.61878	-0.41665
69.686	-0.61873	-0.41665
69.702	-0.61868	-0.41664
69.718	-0.61863	-0.41663
69.735	-0.61858	-0.41661
69.751	-0.61853	-0.4166
69.769	-0.61848	-0.41659
69.785	-0.61842	-0.41659
69.801	-0.61837	-0.41658
69.818	-0.61831	-0.41657
69.835	-0.61826	-0.41657
69.852	-0.61821	-0.41656
69.869	-0.61816	-0.41656
69.886	-0.61812	-0.41656
69.902	-0.61808	-0.41656
69.918	-0.61803	-0.41655
69.936	-0.61798	-0.41654
69.952	-0.61793	-0.41653

69.969	-0.61788	-0.41653
69.986	-0.61783	-0.41652
70.002	-0.61778	-0.41651
70.019	-0.61772	-0.4165
70.035	-0.61767	-0.4165
70.053	-0.61762	-0.41649
70.069	-0.61757	-0.41648
70.086	-0.61751	-0.41647
70.103	-0.61746	-0.41646
70.12	-0.61741	-0.41644
70.136	-0.61736	-0.41644
70.153	-0.61731	-0.41643
70.169	-0.61726	-0.41642
70.185	-0.61721	-0.41641
70.203	-0.61715	-0.4164
70.22	-0.6171	-0.4164
70.236	-0.61705	-0.41639
70.253	-0.61699	-0.41639
70.269	-0.61694	-0.41637
70.286	-0.61689	-0.41636
70.303	-0.61685	-0.41634
70.319	-0.6168	-0.41633
70.336	-0.61675	-0.41631
70.352	-0.61669	-0.4163
70.369	-0.61663	-0.41629
70.386	-0.61657	-0.41628
70.403	-0.61651	-0.41627
70.42	-0.61646	-0.41626
70.436	-0.6164	-0.41625
70.453	-0.61635	-0.41624

70.469	-0.61629	-0.41623
70.487	-0.61624	-0.41621
70.503	-0.61619	-0.41619
70.52	-0.61614	-0.41618
70.536	-0.61609	-0.41617
70.553	-0.61604	-0.41616
70.569	-0.61598	-0.41614
70.586	-0.61593	-0.41614
70.602	-0.61588	-0.41613
70.619	-0.61583	-0.41612
70.637	-0.61578	-0.41611
70.653	-0.61574	-0.4161
70.671	-0.61569	-0.41609
70.687	-0.61565	-0.41608
70.703	-0.61559	-0.41606
70.72	-0.61554	-0.41605
70.736	-0.61549	-0.41603
70.754	-0.61543	-0.41602
70.77	-0.61538	-0.416
70.788	-0.61532	-0.41599
70.802	-0.61527	-0.41597
70.82	-0.61521	-0.41596
70.836	-0.61516	-0.41594
70.853	-0.61511	-0.41593
70.87	-0.61505	-0.41592
70.887	-0.61501	-0.41591
70.904	-0.61496	-0.4159
70.92	-0.61491	-0.41589
70.937	-0.61486	-0.41587
70.954	-0.61481	-0.41586

70.971	-0.61476	-0.41585
70.987	-0.61471	-0.41584
71.003	-0.61466	-0.41582
71.021	-0.61461	-0.4158
71.037	-0.61456	-0.41578
71.054	-0.61451	-0.41576
71.071	-0.61447	-0.41574
71.087	-0.61443	-0.41573
71.104	-0.61438	-0.41571
71.12	-0.61434	-0.4157
71.137	-0.61429	-0.41569
71.154	-0.61423	-0.41567
71.172	-0.61417	-0.41566
71.188	-0.61412	-0.41565
71.204	-0.61406	-0.41563
71.221	-0.61401	-0.41562
71.238	-0.61395	-0.4156
71.255	-0.61389	-0.41558
71.27	-0.61384	-0.41556
71.287	-0.61379	-0.41554
71.305	-0.61374	-0.41553
71.322	-0.61369	-0.41551
71.338	-0.61364	-0.41549
71.355	-0.61359	-0.41547
71.371	-0.61353	-0.41544
71.388	-0.61348	-0.41541
71.406	-0.61343	-0.4154
71.422	-0.61338	-0.41538
71.439	-0.61332	-0.41536
71.456	-0.61326	-0.41534

71.472	-0.6132	-0.41533
71.488	-0.61315	-0.41531
71.505	-0.61309	-0.41529
71.522	-0.61304	-0.41528
71.538	-0.61299	-0.41526
71.555	-0.61294	-0.41524
71.571	-0.61289	-0.41522
71.587	-0.61284	-0.41519
71.604	-0.61279	-0.41516
71.621	-0.61274	-0.41514
71.638	-0.61268	-0.41511
71.655	-0.61262	-0.4151
71.671	-0.61256	-0.41507
71.688	-0.61251	-0.41505
71.704	-0.61245	-0.41503
71.721	-0.6124	-0.41501
71.738	-0.61235	-0.41499
71.754	-0.6123	-0.41497
71.771	-0.61225	-0.41495
71.788	-0.61219	-0.41493
71.806	-0.61213	-0.41491
71.821	-0.61207	-0.41489
71.838	-0.61202	-0.41487
71.855	-0.61196	-0.41485
71.872	-0.6119	-0.41483
71.888	-0.61185	-0.41481
71.905	-0.6118	-0.41479
71.922	-0.61174	-0.41477
71.939	-0.61169	-0.41475
71.955	-0.61164	-0.41473

71.973	-0.61159	-0.4147
71.989	-0.61154	-0.41468
72.006	-0.61149	-0.41465
72.023	-0.61144	-0.41461
72.038	-0.61138	-0.41457
72.055	-0.61133	-0.41455
72.072	-0.61128	-0.41453
72.088	-0.61123	-0.4145
72.105	-0.61118	-0.41447
72.122	-0.61114	-0.41444
72.138	-0.61109	-0.41441
72.155	-0.61105	-0.41439
72.172	-0.61101	-0.41437
72.189	-0.61096	-0.41435
72.206	-0.61092	-0.41433
72.222	-0.61087	-0.4143
72.239	-0.61082	-0.41429
72.255	-0.61076	-0.41427
72.273	-0.61071	-0.41424
72.289	-0.61065	-0.41422
72.306	-0.6106	-0.4142
72.322	-0.61054	-0.41418
72.339	-0.61049	-0.41415
72.355	-0.61044	-0.41413
72.372	-0.61039	-0.4141
72.39	-0.61034	-0.41408
72.406	-0.61029	-0.41405
72.422	-0.61024	-0.41403
72.439	-0.61018	-0.414
72.456	-0.61013	-0.41397

72.473	-0.61008	-0.41394
72.489	-0.61003	-0.41391
72.506	-0.60998	-0.41389
72.523	-0.60993	-0.41386
72.539	-0.60989	-0.41383
72.556	-0.60984	-0.41377
72.573	-0.60979	-0.41373
72.589	-0.60974	-0.41369
72.605	-0.60969	-0.41366
72.623	-0.60965	-0.41363
72.64	-0.6096	-0.4136
72.656	-0.60955	-0.41357
72.672	-0.60949	-0.41355
72.69	-0.60945	-0.41353
72.706	-0.6094	-0.4135
72.723	-0.60935	-0.41348
72.739	-0.6093	-0.41345
72.756	-0.60925	-0.41343
72.773	-0.60919	-0.4134
72.789	-0.60915	-0.41338
72.806	-0.6091	-0.41335
72.824	-0.60906	-0.41332
72.84	-0.60901	-0.41329
72.856	-0.60897	-0.41326
72.874	-0.60892	-0.41323
72.889	-0.60888	-0.4132
72.906	-0.60882	-0.41317
72.923	-0.60877	-0.41314
72.94	-0.60869	-0.41311
72.956	-0.60863	-0.41309

72.973	-0.60858	-0.41306
72.99	-0.60852	-0.41303
73.006	-0.60847	-0.413
73.023	-0.60842	-0.41297
73.04	-0.60837	-0.41295
73.056	-0.60833	-0.41292
73.073	-0.60829	-0.41289
73.09	-0.60825	-0.41286
73.107	-0.6082	-0.41283
73.124	-0.60815	-0.41281
73.14	-0.6081	-0.41278
73.157	-0.60805	-0.41275
73.174	-0.608	-0.41271
73.191	-0.60795	-0.41268
73.207	-0.6079	-0.41265
73.224	-0.60786	-0.41262
73.24	-0.60781	-0.41259
73.257	-0.60777	-0.41256
73.274	-0.60773	-0.41253
73.291	-0.60769	-0.4125
73.308	-0.60765	-0.41247
73.324	-0.6076	-0.41244
73.341	-0.60756	-0.41239
73.358	-0.60751	-0.41232
73.376	-0.60746	-0.41227
73.391	-0.60741	-0.41223
73.408	-0.60736	-0.4122
73.424	-0.60731	-0.41217
73.44	-0.60726	-0.41214
73.457	-0.60722	-0.41211

73.474	-0.60716	-0.41208
73.491	-0.60711	-0.41205
73.508	-0.60706	-0.41202
73.524	-0.60701	-0.41199
73.54	-0.60696	-0.41196
73.558	-0.60691	-0.41193
73.574	-0.60686	-0.4119
73.591	-0.60681	-0.41187
73.607	-0.60677	-0.41184
73.625	-0.60672	-0.41181
73.642	-0.60668	-0.41178
73.658	-0.60663	-0.41175
73.675	-0.60658	-0.41172
73.691	-0.60653	-0.41169
73.707	-0.60648	-0.41166
73.725	-0.60643	-0.41162
73.742	-0.60639	-0.41158
73.759	-0.60634	-0.41154
73.775	-0.60629	-0.41147
73.791	-0.60624	-0.41142
73.808	-0.60619	-0.41135
73.824	-0.60615	-0.41125
73.842	-0.6061	-0.41117
73.859	-0.60606	-0.41114
73.875	-0.60601	-0.4111
73.892	-0.60596	-0.41107
73.908	-0.60591	-0.41104
73.925	-0.60585	-0.41101
73.942	-0.6058	-0.41098
73.959	-0.60574	-0.41095

73.975	-0.60569	-0.41092
73.991	-0.60564	-0.41089
74.008	-0.60559	-0.41086
74.025	-0.60554	-0.41083
74.042	-0.6055	-0.41079
74.059	-0.60545	-0.41076
74.075	-0.6054	-0.41071
74.092	-0.60535	-0.41066
74.109	-0.60531	-0.41061
74.125	-0.60526	-0.41057
74.143	-0.60521	-0.41054
74.158	-0.60516	-0.4105
74.176	-0.60511	-0.41047
74.193	-0.60507	-0.41044
74.21	-0.60502	-0.4104
74.226	-0.60497	-0.41037
74.242	-0.60492	-0.41034
74.257	-0.60486	-0.41031
74.274	-0.60481	-0.41025
74.292	-0.60475	-0.4102
74.309	-0.60469	-0.41014
74.326	-0.60464	-0.4101
74.342	-0.60458	-0.41007
74.36	-0.60453	-0.41003
74.376	-0.60448	-0.40999
74.393	-0.60443	-0.40993
74.408	-0.60439	-0.40989
74.426	-0.60434	-0.40986
74.443	-0.6043	-0.40982
74.459	-0.60425	-0.40978

74.475	-0.60421	-0.40975
74.493	-0.60417	-0.40971
74.51	-0.60412	-0.40968
74.526	-0.60407	-0.40964
74.543	-0.60402	-0.40961
74.56	-0.60397	-0.40956
74.576	-0.60391	-0.4095
74.593	-0.60386	-0.40944
74.61	-0.60381	-0.4094
74.626	-0.60376	-0.40936
74.643	-0.60371	-0.40932
74.66	-0.60365	-0.40928
74.676	-0.6036	-0.40924
74.693	-0.60355	-0.40921
74.709	-0.6035	-0.40917
74.727	-0.60345	-0.40914
74.744	-0.60341	-0.4091
74.76	-0.60336	-0.40905
74.776	-0.60331	-0.40901
74.794	-0.60327	-0.40898
74.81	-0.60322	-0.40894
74.826	-0.60317	-0.40891
74.844	-0.60313	-0.40887
74.86	-0.60308	-0.40884
74.876	-0.60303	-0.4088
74.893	-0.60298	-0.40876
74.911	-0.60294	-0.40873
74.927	-0.6029	-0.40868
74.944	-0.60285	-0.40861
74.96	-0.6028	-0.40854

74.976	-0.60276	-0.40846
74.994	-0.60271	-0.40837
75.01	-0.60267	-0.4083
75.026	-0.60262	-0.40823
75.044	-0.60257	-0.40818
75.061	-0.60252	-0.40814
75.077	-0.60248	-0.4081
75.093	-0.60243	-0.40806
75.11	-0.60238	-0.40802
75.127	-0.60233	-0.40799
75.144	-0.60228	-0.40795
75.16	-0.60222	-0.40791
75.178	-0.60217	-0.40787
75.194	-0.60212	-0.40783
75.211	-0.60207	-0.40778
75.228	-0.60202	-0.40773
75.244	-0.60197	-0.40768
75.261	-0.60193	-0.40762
75.277	-0.60188	-0.40755
75.294	-0.60183	-0.40751
75.311	-0.60178	-0.40747
75.328	-0.60173	-0.40744
75.345	-0.60169	-0.40741
75.361	-0.60164	-0.40736
75.379	-0.6016	-0.40733
75.395	-0.60155	-0.40726
75.411	-0.60151	-0.40717
75.429	-0.60147	-0.40709
75.445	-0.60142	-0.40701
75.462	-0.60137	-0.40693

75.478	-0.60133	-0.40687
75.496	-0.60128	-0.40683
75.511	-0.60123	-0.40679
75.528	-0.60117	-0.40676
75.545	-0.60112	-0.40671
75.561	-0.60108	-0.40666
75.578	-0.60103	-0.40663
75.595	-0.60098	-0.40659
75.612	-0.60092	-0.40653
75.628	-0.60087	-0.40647
75.645	-0.60082	-0.40642
75.661	-0.60077	-0.40638
75.678	-0.60072	-0.40634
75.697	-0.60068	-0.40631
75.713	-0.60063	-0.40627
75.729	-0.60059	-0.40624
75.746	-0.60055	-0.4062
75.762	-0.60051	-0.40617
75.779	-0.60048	-0.40611
75.796	-0.60044	-0.40604
75.812	-0.60039	-0.40595
75.828	-0.60035	-0.40584
75.846	-0.6003	-0.40575
75.863	-0.60025	-0.40567
75.878	-0.6002	-0.40557
75.895	-0.60015	-0.40553
75.913	-0.6001	-0.40548
75.929	-0.60006	-0.40541
75.946	-0.60001	-0.40536
75.963	-0.59996	-0.40531

75.98	-0.59991	-0.40525
75.996	-0.59986	-0.40521
76.014	-0.59981	-0.40517
76.029	-0.59976	-0.40511
76.046	-0.59971	-0.40506
76.063	-0.59966	-0.40501
76.08	-0.59962	-0.40496
76.097	-0.59957	-0.40487
76.113	-0.59953	-0.4048
76.13	-0.5995	-0.40469
76.147	-0.59946	-0.40463
76.163	-0.59942	-0.40458
76.179	-0.59938	-0.40454
76.197	-0.59933	-0.4045
76.213	-0.59928	-0.40447
76.23	-0.59924	-0.40443
76.247	-0.59919	-0.40439
76.263	-0.59913	-0.40435
76.281	-0.59909	-0.4043
76.297	-0.59904	-0.40423
76.313	-0.59898	-0.40412
76.33	-0.59893	-0.40404
76.346	-0.59887	-0.40396
76.363	-0.59882	-0.4039
76.38	-0.59877	-0.40384
76.395	-0.59871	-0.40379
76.413	-0.59866	-0.40376
76.43	-0.59861	-0.4037
76.446	-0.59857	-0.40364
76.463	-0.59852	-0.40361

76.48	-0.59848	-0.40357
76.496	-0.59844	-0.40353
76.513	-0.5984	-0.40349
76.53	-0.59835	-0.40346
76.547	-0.59831	-0.4034
76.563	-0.59826	-0.40333
76.58	-0.59818	-0.40326
76.597	-0.5981	-0.40321
76.614	-0.59804	-0.4031
76.63	-0.59798	-0.40301
76.647	-0.59789	-0.40293
76.664	-0.59783	-0.40284
76.681	-0.59777	-0.40281
76.697	-0.59772	-0.40277
76.714	-0.59767	-0.40273
76.73	-0.59763	-0.40269
76.747	-0.59758	-0.40264
76.764	-0.59754	-0.4026
76.781	-0.59749	-0.40255
76.797	-0.59745	-0.40249
76.813	-0.59741	-0.40243
76.831	-0.59736	-0.40235
76.847	-0.59731	-0.40226
76.864	-0.59727	-0.4022
76.881	-0.59723	-0.40214
76.897	-0.59718	-0.4021
76.914	-0.59713	-0.40206
76.93	-0.59709	-0.40201
76.947	-0.59705	-0.40198
76.964	-0.597	-0.40194

76.98	-0.59695	-0.4019
76.998	-0.5969	-0.40186
77.014	-0.59685	-0.40183
77.03	-0.5968	-0.40179
77.047	-0.59675	-0.40175
77.064	-0.59671	-0.40169
77.082	-0.59666	-0.4016
77.098	-0.59662	-0.40152
77.114	-0.59659	-0.40146
77.132	-0.59655	-0.40138
77.147	-0.59651	-0.4013
77.164	-0.59647	-0.40121
77.181	-0.59644	-0.40113
77.198	-0.59639	-0.40109
77.215	-0.59635	-0.40101
77.231	-0.5963	-0.40095
77.248	-0.59626	-0.40088
77.264	-0.59621	-0.40082
77.282	-0.59615	-0.40078
77.298	-0.59611	-0.40074
77.315	-0.59606	-0.4007
77.331	-0.59601	-0.40066
77.348	-0.59597	-0.40061
77.365	-0.59593	-0.40057
77.383	-0.59589	-0.40053
77.397	-0.59585	-0.40049
77.415	-0.59581	-0.40045
77.432	-0.59577	-0.40041
77.45	-0.59573	-0.40037
77.465	-0.59569	-0.40033

77.482	-0.59565	-0.40029
77.499	-0.59561	-0.40024
77.516	-0.59557	-0.4002
77.532	-0.59553	-0.40015
77.549	-0.59549	-0.40011
77.565	-0.59545	-0.40006
77.583	-0.59541	-0.40002
77.6	-0.59537	-0.39998
77.616	-0.59533	-0.39994
77.633	-0.59529	-0.3999
77.65	-0.59525	-0.39985
77.666	-0.59521	-0.39979
77.683	-0.59518	-0.39974
77.699	-0.59514	-0.39967
77.717	-0.5951	-0.39957
77.732	-0.59506	-0.39946
77.749	-0.59503	-0.39939
77.766	-0.59499	-0.39931
77.784	-0.59496	-0.39926
77.8	-0.59492	-0.39921
77.817	-0.59489	-0.39917
77.834	-0.59486	-0.39913
77.85	-0.59482	-0.39909
77.866	-0.59479	-0.39903
77.883	-0.59475	-0.39897
77.9	-0.59471	-0.39891
77.917	-0.59467	-0.39884
77.933	-0.59462	-0.39877
77.95	-0.59458	-0.39869
77.967	-0.59454	-0.39862

77.983	-0.59449	-0.39857
78	-0.59445	-0.39851
78.017	-0.59441	-0.39847
78.034	-0.59437	-0.39841
78.051	-0.59432	-0.39836
78.067	-0.59428	-0.39831
78.084	-0.59424	-0.39827
78.1	-0.5942	-0.39823
78.117	-0.59415	-0.39816
78.134	-0.59411	-0.39807
78.151	-0.59407	-0.39796
78.167	-0.59403	-0.39788
78.184	-0.59399	-0.39779
78.2	-0.59395	-0.39773
78.217	-0.59391	-0.39762
78.233	-0.59388	-0.39751
78.25	-0.59384	-0.39744
78.267	-0.5938	-0.39735
78.284	-0.59376	-0.39729
78.301	-0.59372	-0.39721
78.317	-0.59368	-0.39713
78.334	-0.59364	-0.39704
78.351	-0.5936	-0.39696
78.368	-0.59356	-0.39692
78.385	-0.59352	-0.39688
78.401	-0.59347	-0.39683
78.418	-0.59343	-0.39675
78.435	-0.59339	-0.39669
78.452	-0.59334	-0.39663
78.468	-0.59329	-0.39656

78.485	-0.59324	-0.39646
78.502	-0.59319	-0.39637
78.518	-0.59315	-0.39629
78.533	-0.5931	-0.39616
78.551	-0.59306	-0.39607
78.567	-0.59302	-0.396
78.585	-0.59298	-0.39593
78.601	-0.59295	-0.39586
78.618	-0.59291	-0.39581
78.635	-0.59288	-0.39576
78.652	-0.59284	-0.39571
78.668	-0.59281	-0.39564
78.685	-0.59277	-0.3956
78.702	-0.59273	-0.39556
78.718	-0.59269	-0.39552
78.735	-0.59265	-0.39548
78.751	-0.59261	-0.39544
78.769	-0.59257	-0.3954
78.785	-0.59253	-0.39535
78.803	-0.59248	-0.39529
78.819	-0.59244	-0.39521
78.836	-0.5924	-0.39513
78.852	-0.59236	-0.39503
78.869	-0.59232	-0.39493
78.887	-0.59228	-0.39487
78.903	-0.59223	-0.39482
78.919	-0.59219	-0.39478
78.936	-0.59215	-0.39474
78.953	-0.59211	-0.3947
78.969	-0.59206	-0.39466

78.985	-0.59202	-0.39458
79.002	-0.59198	-0.39449
79.019	-0.59194	-0.39436
79.036	-0.59189	-0.39422
79.053	-0.59185	-0.39404
79.069	-0.59181	-0.39388
79.087	-0.59176	-0.39376
79.102	-0.59172	-0.39367
79.119	-0.59167	-0.3936
79.136	-0.59163	-0.39354
79.153	-0.59159	-0.39349
79.169	-0.59155	-0.39343
79.186	-0.59151	-0.39337
79.203	-0.59147	-0.39331
79.219	-0.59143	-0.39326
79.236	-0.5914	-0.39323
79.252	-0.59135	-0.39319
79.27	-0.59132	-0.39313
79.287	-0.59127	-0.39306
79.303	-0.59123	-0.39298
79.32	-0.59119	-0.3929
79.337	-0.59115	-0.3928
79.353	-0.5911	-0.39271
79.37	-0.59106	-0.39265
79.387	-0.59102	-0.39258
79.404	-0.59097	-0.39249
79.42	-0.59093	-0.39238
79.437	-0.5909	-0.39229
79.453	-0.59087	-0.3922
79.47	-0.59084	-0.39209

79.487	-0.5908	-0.39199
79.504	-0.59077	-0.39193
79.521	-0.59073	-0.39187
79.537	-0.5907	-0.39184
79.554	-0.59066	-0.3918
79.57	-0.59062	-0.39177
79.587	-0.59058	-0.39171
79.604	-0.59053	-0.39162
79.621	-0.59049	-0.3915
79.637	-0.59045	-0.39137
79.654	-0.59041	-0.39123
79.671	-0.59038	-0.39111
79.687	-0.59034	-0.39103
79.705	-0.5903	-0.39098
79.721	-0.59027	-0.39094
79.737	-0.59024	-0.39091
79.754	-0.5902	-0.39087
79.77	-0.59017	-0.39084
79.787	-0.59013	-0.3908
79.805	-0.59009	-0.39077
79.821	-0.59006	-0.39072
79.838	-0.59002	-0.39064
79.854	-0.58998	-0.39054
79.872	-0.58994	-0.39041
79.888	-0.5899	-0.39027
79.905	-0.58986	-0.39016
79.921	-0.58981	-0.39009
79.938	-0.58978	-0.39005
79.956	-0.58974	-0.38999
79.971	-0.5897	-0.38993

79.989	-0.58967	-0.38989
80.004	-0.58963	-0.38984
80.022	-0.58959	-0.38977
80.038	-0.58955	-0.38969
80.055	-0.58951	-0.38964
80.072	-0.58947	-0.3896
80.089	-0.58943	-0.38954
80.106	-0.5894	-0.38949
80.122	-0.58936	-0.38944
80.139	-0.58933	-0.38938
80.156	-0.5893	-0.38932
80.172	-0.58926	-0.38926
80.189	-0.58923	-0.38918
80.206	-0.58919	-0.38908
80.223	-0.58914	-0.38898
80.239	-0.5891	-0.38889
80.255	-0.58905	-0.3888
80.273	-0.58901	-0.38874
80.289	-0.58898	-0.38869
80.306	-0.58894	-0.38865
80.323	-0.5889	-0.38861
80.339	-0.58886	-0.38856
80.356	-0.58882	-0.38849
80.372	-0.58878	-0.38841
80.39	-0.58874	-0.3883
80.407	-0.5887	-0.3882
80.423	-0.58866	-0.38809
80.439	-0.58862	-0.38796
80.456	-0.58859	-0.38785
80.474	-0.58855	-0.38773

80.49	-0.58851	-0.38767
80.507	-0.58848	-0.38762
80.523	-0.58844	-0.38757
80.54	-0.5884	-0.38749
80.557	-0.58837	-0.38741
80.573	-0.58833	-0.3873
80.59	-0.58829	-0.38723
80.606	-0.58825	-0.38718
80.623	-0.58822	-0.38712
80.64	-0.58818	-0.38708
80.657	-0.58814	-0.38704
80.675	-0.5881	-0.387
80.69	-0.58807	-0.38697
80.707	-0.58803	-0.38693
80.724	-0.588	-0.38687
80.741	-0.58796	-0.3868
80.758	-0.58792	-0.38669
80.773	-0.58788	-0.38659
80.791	-0.58784	-0.38645
80.807	-0.5878	-0.3863
80.824	-0.58776	-0.38619
80.841	-0.58772	-0.38609
80.858	-0.58768	-0.38601
80.875	-0.58764	-0.38594
80.891	-0.58761	-0.38589
80.908	-0.58757	-0.38584
80.924	-0.58754	-0.3858
80.941	-0.58751	-0.38575
80.957	-0.58747	-0.3857
80.973	-0.58744	-0.38563

80.99	-0.58741	-0.38558
81.007	-0.58738	-0.38554
81.025	-0.58734	-0.3855
81.042	-0.58731	-0.38546
81.057	-0.58728	-0.3854
81.074	-0.58725	-0.38534
81.091	-0.58721	-0.38528
81.108	-0.58717	-0.3852
81.125	-0.58713	-0.3851
81.141	-0.58709	-0.38498
81.158	-0.58706	-0.38484
81.175	-0.58702	-0.3847
81.191	-0.58698	-0.3846
81.208	-0.58695	-0.38452
81.224	-0.58692	-0.38445
81.24	-0.58689	-0.38437
81.258	-0.58686	-0.38429
81.275	-0.58682	-0.38423
81.292	-0.58679	-0.38417
81.308	-0.58676	-0.38412
81.324	-0.58673	-0.38406
81.341	-0.58669	-0.38401
81.358	-0.58665	-0.38396
81.375	-0.58662	-0.38391
81.392	-0.58659	-0.38386
81.408	-0.58656	-0.38381
81.425	-0.58653	-0.38375
81.441	-0.58651	-0.3837
81.458	-0.58648	-0.38364
81.475	-0.58645	-0.38358

81.491	-0.58642	-0.38352
81.508	-0.58639	-0.38347
81.525	-0.58635	-0.38341
81.542	-0.58631	-0.38334
81.559	-0.58627	-0.38326
81.575	-0.58623	-0.38319
81.592	-0.5862	-0.3831
81.609	-0.58618	-0.38304
81.626	-0.58615	-0.383
81.642	-0.58612	-0.38296
81.659	-0.58609	-0.38293
81.675	-0.58605	-0.3829
81.692	-0.58603	-0.38286
81.71	-0.586	-0.38281
81.726	-0.58597	-0.38272
81.743	-0.58593	-0.38267
81.759	-0.5859	-0.38261
81.776	-0.58587	-0.38249
81.793	-0.58584	-0.38238
81.809	-0.58581	-0.38227
81.826	-0.58578	-0.3822
81.843	-0.58575	-0.38213
81.86	-0.58571	-0.38208
81.876	-0.58568	-0.38201
81.892	-0.58565	-0.38194
81.91	-0.58562	-0.38189
81.926	-0.58559	-0.38184
81.943	-0.58555	-0.3818
81.959	-0.58551	-0.38176
81.977	-0.58547	-0.3817

81.993	-0.58543	-0.38162
82.01	-0.5854	-0.38155
82.027	-0.58536	-0.38147
82.043	-0.58533	-0.38141
82.059	-0.5853	-0.38134
82.078	-0.58527	-0.38122
82.094	-0.58523	-0.38109
82.11	-0.5852	-0.38095
82.127	-0.58517	-0.38081
82.144	-0.58514	-0.38071
82.16	-0.5851	-0.38063
82.178	-0.58507	-0.38057
82.195	-0.58505	-0.3805
82.211	-0.58502	-0.38047
82.228	-0.585	-0.38043
82.245	-0.58497	-0.38035
82.261	-0.58495	-0.38031
82.278	-0.58492	-0.38027
82.295	-0.58489	-0.38021
82.311	-0.58486	-0.38013
82.328	-0.58483	-0.38006
82.345	-0.58479	-0.37997
82.361	-0.58476	-0.37987
82.378	-0.58472	-0.37977
82.395	-0.58469	-0.37968
82.412	-0.58465	-0.3796
82.429	-0.58462	-0.37953
82.445	-0.58459	-0.37944
82.462	-0.58456	-0.37937
82.479	-0.58452	-0.37929

82.496	-0.58449	-0.3792
82.512	-0.58445	-0.37912
82.529	-0.58441	-0.37902
82.545	-0.58437	-0.37892
82.562	-0.58434	-0.37882
82.579	-0.5843	-0.37871
82.595	-0.58426	-0.37861
82.612	-0.58423	-0.37853
82.629	-0.5842	-0.37847
82.645	-0.58417	-0.37839
82.662	-0.58415	-0.37831
82.679	-0.58412	-0.37826
82.695	-0.58409	-0.37821
82.713	-0.58406	-0.37816
82.729	-0.58403	-0.3781
82.746	-0.584	-0.37804
82.763	-0.58397	-0.37796
82.779	-0.58393	-0.37788
82.796	-0.58391	-0.37777
82.812	-0.58388	-0.37769
82.829	-0.58385	-0.37761
82.846	-0.58382	-0.37754
82.862	-0.58379	-0.37747
82.879	-0.58376	-0.37738
82.895	-0.58373	-0.37727
82.912	-0.5837	-0.37719
82.928	-0.58367	-0.3771
82.946	-0.58364	-0.37705
82.962	-0.58362	-0.377
82.98	-0.58359	-0.37694

82.996	-0.58357	-0.3769
83.013	-0.58353	-0.37686
83.029	-0.5835	-0.3768
83.046	-0.58347	-0.37672
83.063	-0.58345	-0.37663
83.08	-0.58342	-0.3765
83.097	-0.5834	-0.37639
83.112	-0.58337	-0.37629
83.13	-0.58334	-0.37616
83.147	-0.58332	-0.37604
83.162	-0.58329	-0.37596
83.179	-0.58327	-0.37591
83.196	-0.58325	-0.37587
83.213	-0.58322	-0.37584
83.229	-0.58319	-0.3758
83.246	-0.58317	-0.37577
83.263	-0.58314	-0.37574
83.28	-0.58311	-0.3757
83.297	-0.58308	-0.37565
83.314	-0.58304	-0.37557
83.33	-0.58301	-0.37545
83.347	-0.58297	-0.3753
83.364	-0.58294	-0.37516
83.38	-0.58291	-0.37501
83.397	-0.58289	-0.37487
83.414	-0.58286	-0.37478
83.43	-0.58284	-0.3747
83.448	-0.58283	-0.37466
83.463	-0.58281	-0.37459
83.479	-0.58278	-0.37453

83.498	-0.58276	-0.37448
83.515	-0.58272	-0.37443
83.53	-0.58269	-0.37435
83.547	-0.58266	-0.37429
83.564	-0.58263	-0.37423
83.581	-0.58261	-0.37413
83.597	-0.58258	-0.37406
83.614	-0.58255	-0.37398
83.63	-0.58252	-0.3739
83.647	-0.58249	-0.37381
83.663	-0.58246	-0.37371
83.681	-0.58243	-0.3736
83.698	-0.5824	-0.3735
83.715	-0.58237	-0.37341
83.731	-0.58235	-0.37332
83.748	-0.58233	-0.37326
83.765	-0.58231	-0.37319
83.782	-0.58229	-0.37312
83.798	-0.58227	-0.37306
83.814	-0.58225	-0.37299
83.832	-0.58222	-0.37291
83.849	-0.58219	-0.37283
83.865	-0.58216	-0.37275
83.882	-0.58213	-0.3727
83.899	-0.58211	-0.37264
83.915	-0.58208	-0.37258
83.931	-0.58206	-0.37251
83.948	-0.58203	-0.37243
83.965	-0.58201	-0.37232
83.981	-0.58198	-0.37221

83.999	-0.58195	-0.37214
84.015	-0.58193	-0.37201
84.032	-0.58191	-0.37186
84.048	-0.58189	-0.37171
84.066	-0.58186	-0.37157
84.082	-0.58184	-0.37145
84.099	-0.58183	-0.37136
84.116	-0.5818	-0.37127
84.132	-0.58178	-0.3712
84.148	-0.58175	-0.37113
84.165	-0.58172	-0.37107
84.182	-0.58169	-0.37104
84.199	-0.58167	-0.37101
84.216	-0.58164	-0.37098
84.231	-0.58162	-0.37095
84.249	-0.58159	-0.37092
84.265	-0.58158	-0.37089
84.282	-0.58156	-0.37086
84.299	-0.58155	-0.37083
84.316	-0.58153	-0.37079
84.333	-0.58151	-0.37072
84.349	-0.58149	-0.3706
84.366	-0.58146	-0.37039
84.383	-0.58144	-0.37016
84.4	-0.58141	-0.36999
84.416	-0.58137	-0.36986
84.433	-0.58135	-0.36976
84.449	-0.58132	-0.36968
84.465	-0.58129	-0.3696
84.483	-0.58127	-0.36955

84.499	-0.58124	-0.36951
84.516	-0.58122	-0.36947
84.533	-0.5812	-0.36942
84.549	-0.58118	-0.36938
84.566	-0.58116	-0.36932
84.583	-0.58113	-0.36928
84.6	-0.58111	-0.36923
84.617	-0.58109	-0.36919
84.633	-0.58107	-0.36915
84.65	-0.58105	-0.3691
84.666	-0.58103	-0.36903
84.683	-0.581	-0.36895
84.699	-0.58098	-0.36886
84.716	-0.58095	-0.36878
84.734	-0.58093	-0.36873
84.75	-0.58091	-0.3687
84.767	-0.58088	-0.36865
84.783	-0.58086	-0.3686
84.801	-0.58084	-0.36856
84.817	-0.58082	-0.36851
84.834	-0.58081	-0.36846
84.85	-0.58079	-0.36841
84.867	-0.58077	-0.36836
84.884	-0.58075	-0.36829
84.902	-0.58072	-0.36822
84.917	-0.58069	-0.36812
84.935	-0.58067	-0.36804
84.951	-0.58065	-0.36798
84.968	-0.58062	-0.3679
84.983	-0.5806	-0.36785

85.002	-0.58059	-0.3678
85.018	-0.58057	-0.36775
85.036	-0.58055	-0.3677
85.051	-0.58054	-0.36767
85.068	-0.58052	-0.36764
85.085	-0.5805	-0.36761
85.101	-0.58049	-0.36756
85.118	-0.58047	-0.36747
85.135	-0.58045	-0.36738
85.152	-0.58044	-0.36727
85.168	-0.58042	-0.36718
85.185	-0.5804	-0.36704
85.202	-0.58038	-0.3669
85.218	-0.58036	-0.36674
85.235	-0.58034	-0.3666
85.252	-0.58032	-0.36647
85.269	-0.5803	-0.36636
85.286	-0.58028	-0.36627
85.302	-0.58026	-0.3661
85.318	-0.58025	-0.36593
85.336	-0.58023	-0.36584
85.352	-0.58022	-0.36578
85.37	-0.5802	-0.36573
85.386	-0.58018	-0.36569
85.403	-0.58017	-0.36566
85.42	-0.58015	-0.36562
85.436	-0.58013	-0.36557
85.453	-0.58011	-0.36551
85.47	-0.5801	-0.36545
85.487	-0.58008	-0.36536

85.503	-0.58007	-0.36526
85.519	-0.58005	-0.36517
85.537	-0.58003	-0.36509
85.554	-0.58001	-0.36501
85.57	-0.57999	-0.36492
85.586	-0.57998	-0.36484
85.604	-0.57996	-0.36476
85.619	-0.57995	-0.36468
85.636	-0.57993	-0.36457
85.653	-0.57991	-0.36445
85.67	-0.57989	-0.36437
85.687	-0.57986	-0.36428
85.703	-0.57985	-0.36423
85.72	-0.57983	-0.36418
85.737	-0.57982	-0.36415
85.753	-0.5798	-0.36412
85.771	-0.57979	-0.36404
85.787	-0.57978	-0.36396
85.803	-0.57976	-0.36389
85.82	-0.57975	-0.36379
85.837	-0.57974	-0.36363
85.853	-0.57973	-0.36348
85.87	-0.57971	-0.36333
85.887	-0.5797	-0.3632
85.903	-0.57969	-0.36312
85.92	-0.57967	-0.36308
85.937	-0.57966	-0.36302
85.954	-0.57964	-0.36296
85.97	-0.57962	-0.3629
85.987	-0.5796	-0.36285

86.005	-0.57958	-0.36281
86.021	-0.57957	-0.36277
86.038	-0.57955	-0.36273
86.054	-0.57953	-0.36267
86.07	-0.57951	-0.36258
86.087	-0.57949	-0.36249
86.104	-0.57947	-0.36241
86.121	-0.57946	-0.36234
86.138	-0.57945	-0.36224
86.154	-0.57944	-0.36215
86.171	-0.57943	-0.36209
86.188	-0.57942	-0.36202
86.204	-0.57942	-0.36194
86.222	-0.57941	-0.36189
86.239	-0.5794	-0.36181
86.255	-0.57939	-0.36169
86.272	-0.57938	-0.36159
86.289	-0.57937	-0.36149
86.305	-0.57936	-0.3614
86.322	-0.57935	-0.36133
86.338	-0.57932	-0.36124
86.356	-0.57931	-0.36116
86.373	-0.57929	-0.3611
86.389	-0.57928	-0.36104
86.405	-0.57926	-0.36099
86.422	-0.57924	-0.36091
86.439	-0.57923	-0.3608
86.455	-0.57922	-0.36072
86.472	-0.57921	-0.36066
86.488	-0.5792	-0.36058

86.505	-0.57918	-0.36053
86.523	-0.57917	-0.36049
86.54	-0.57916	-0.36045
86.556	-0.57915	-0.3604
86.572	-0.57914	-0.36036
86.589	-0.57914	-0.36032
86.606	-0.57913	-0.36027
86.623	-0.57912	-0.36015
86.639	-0.57912	-0.35999
86.656	-0.57911	-0.35987
86.672	-0.5791	-0.35972
86.689	-0.57909	-0.35961
86.705	-0.57908	-0.35958
86.723	-0.57907	-0.35953
86.74	-0.57906	-0.35949
86.756	-0.57905	-0.35944
86.772	-0.57904	-0.35939
86.789	-0.57903	-0.35935
86.806	-0.57902	-0.3593
86.823	-0.579	-0.35925
86.84	-0.57899	-0.35918
86.857	-0.57898	-0.35908
86.874	-0.57898	-0.35897
86.891	-0.57897	-0.3589
86.907	-0.57896	-0.35883
86.923	-0.57896	-0.35876
86.94	-0.57896	-0.35867
86.957	-0.57895	-0.35856
86.974	-0.57894	-0.35848
86.991	-0.57893	-0.35839

87.007	-0.57893	-0.35833
87.023	-0.57892	-0.35829
87.041	-0.57891	-0.35823
87.058	-0.57891	-0.35815
87.075	-0.5789	-0.35805
87.091	-0.5789	-0.35794
87.108	-0.57889	-0.35786
87.124	-0.57888	-0.35777
87.14	-0.57888	-0.35771
87.157	-0.57888	-0.35766
87.174	-0.57887	-0.35761
87.192	-0.57886	-0.35756
87.208	-0.57886	-0.35753
87.225	-0.57886	-0.3575
87.242	-0.57886	-0.35747
87.259	-0.57886	-0.35741
87.276	-0.57886	-0.35732
87.292	-0.57885	-0.35724
87.309	-0.57884	-0.35717
87.326	-0.57883	-0.35709
87.342	-0.57881	-0.35705
87.359	-0.5788	-0.35699
87.375	-0.57879	-0.35691
87.392	-0.57878	-0.35683
87.408	-0.57877	-0.35675
87.426	-0.57876	-0.35666
87.442	-0.57875	-0.35653
87.458	-0.57874	-0.35638
87.476	-0.57873	-0.35623
87.492	-0.57872	-0.35609

87.509	-0.57872	-0.35594
87.526	-0.57871	-0.35582
87.543	-0.5787	-0.35573
87.559	-0.57869	-0.35561
87.576	-0.57868	-0.35554
87.593	-0.57867	-0.35547
87.61	-0.57866	-0.3554
87.626	-0.57865	-0.35534
87.643	-0.57863	-0.35526
87.659	-0.57862	-0.35518
87.676	-0.57861	-0.35511
87.692	-0.57861	-0.35503
87.71	-0.5786	-0.35495
87.726	-0.57859	-0.35489
87.743	-0.57859	-0.35483
87.76	-0.57858	-0.35478
87.777	-0.57858	-0.35472
87.794	-0.57857	-0.35466
87.81	-0.57857	-0.35459
87.826	-0.57856	-0.35448
87.843	-0.57855	-0.35437
87.86	-0.57855	-0.35426
87.877	-0.57855	-0.35415
87.893	-0.57855	-0.35405
87.91	-0.57854	-0.35396
87.926	-0.57854	-0.35388
87.942	-0.57852	-0.35381
87.96	-0.57852	-0.35373
87.977	-0.57851	-0.35366
87.994	-0.5785	-0.35357

88.011	-0.57849	-0.35346
88.027	-0.57847	-0.35337
88.044	-0.57847	-0.35327
88.06	-0.57846	-0.35314
88.078	-0.57846	-0.35298
88.094	-0.57845	-0.35284
88.11	-0.57845	-0.35275
88.128	-0.57844	-0.35268
88.143	-0.57844	-0.35261
88.161	-0.57843	-0.35253
88.177	-0.57843	-0.35246
88.194	-0.57843	-0.35239
88.211	-0.57843	-0.35236
88.227	-0.57842	-0.35232
88.244	-0.57842	-0.35226
88.262	-0.57841	-0.35219
88.277	-0.5784	-0.35212
88.295	-0.57839	-0.35205
88.311	-0.57838	-0.35199
88.328	-0.57837	-0.35193
88.345	-0.57835	-0.35184
88.361	-0.57834	-0.35175
88.378	-0.57833	-0.35169
88.395	-0.57832	-0.35163
88.412	-0.57831	-0.35158
88.427	-0.5783	-0.35152
88.444	-0.5783	-0.35147
88.462	-0.5783	-0.35138
88.479	-0.57829	-0.35127
88.495	-0.57829	-0.35115

88.512	-0.57829	-0.351
88.529	-0.57829	-0.35085
88.545	-0.57829	-0.35076
88.562	-0.57828	-0.35067
88.578	-0.57828	-0.35059
88.595	-0.57827	-0.35052
88.612	-0.57827	-0.35045
88.629	-0.57827	-0.35041
88.645	-0.57826	-0.35036
88.662	-0.57826	-0.35032
88.679	-0.57825	-0.35028
88.695	-0.57824	-0.35024
88.712	-0.57823	-0.35017
88.728	-0.57822	-0.35012
88.746	-0.57822	-0.35005
88.763	-0.57821	-0.34997
88.779	-0.5782	-0.3499
88.796	-0.57819	-0.34983
88.813	-0.57819	-0.34975
88.829	-0.57819	-0.34968
88.846	-0.57818	-0.34962
88.863	-0.57818	-0.34957
88.879	-0.57817	-0.3495
88.895	-0.57817	-0.34942
88.913	-0.57817	-0.34933
88.93	-0.57817	-0.34921
88.946	-0.57817	-0.34908
88.963	-0.57818	-0.34897
88.98	-0.57818	-0.34885
88.997	-0.57819	-0.34869

89.013	-0.57819	-0.34856
89.03	-0.5782	-0.34848
89.046	-0.5782	-0.34844
89.063	-0.57821	-0.3484
89.079	-0.57821	-0.34837
89.096	-0.57821	-0.34829
89.113	-0.5782	-0.34826
89.13	-0.5782	-0.34823
89.146	-0.5782	-0.3482
89.163	-0.57819	-0.34815
89.181	-0.57819	-0.34805
89.196	-0.57818	-0.34793
89.214	-0.57818	-0.34783
89.23	-0.57818	-0.34772
89.246	-0.57819	-0.34759
89.264	-0.57819	-0.34751
89.28	-0.5782	-0.34743
89.298	-0.5782	-0.34736
89.313	-0.5782	-0.34729
89.331	-0.57821	-0.34724
89.348	-0.57821	-0.34716
89.364	-0.57822	-0.34709
89.38	-0.57822	-0.34702
89.396	-0.57822	-0.34698
89.414	-0.57822	-0.34694
89.43	-0.57822	-0.34692
89.448	-0.57822	-0.34689
89.464	-0.57823	-0.34684
89.481	-0.57824	-0.34679
89.498	-0.57824	-0.3467

89.515	-0.57825	-0.3466
89.531	-0.57826	-0.3464
89.548	-0.57827	-0.34625
89.564	-0.57828	-0.34615
89.581	-0.57829	-0.34606
89.598	-0.5783	-0.34597
89.615	-0.5783	-0.34591
89.631	-0.57831	-0.34585
89.648	-0.57832	-0.34582
89.665	-0.57832	-0.34578
89.682	-0.57833	-0.34573
89.699	-0.57833	-0.34565
89.715	-0.57834	-0.34555
89.732	-0.57835	-0.34547
89.749	-0.57835	-0.3454
89.765	-0.57836	-0.34535
89.781	-0.57838	-0.34527
89.799	-0.57839	-0.34517
89.816	-0.5784	-0.34507
89.832	-0.57842	-0.34497
89.849	-0.57843	-0.34485
89.865	-0.57844	-0.34477
89.882	-0.57844	-0.3447
89.898	-0.57845	-0.3446
89.915	-0.57845	-0.34454
89.932	-0.57845	-0.3445
89.949	-0.57846	-0.34447
89.966	-0.57846	-0.34442
89.982	-0.57847	-0.34438
90	-0.57847	-0.34433

90.016	-0.57848	-0.34425
90.033	-0.57849	-0.34414
90.05	-0.57849	-0.34403
90.066	-0.5785	-0.34395
90.083	-0.57851	-0.34383
90.1	-0.57853	-0.34368
90.116	-0.57854	-0.34358
90.134	-0.57855	-0.34349
90.15	-0.57856	-0.34341
90.166	-0.57857	-0.34332
90.183	-0.57858	-0.34326
90.199	-0.57859	-0.34317
90.217	-0.5786	-0.34308
90.233	-0.57861	-0.34301
90.25	-0.57861	-0.34297
90.266	-0.57861	-0.3429
90.283	-0.57861	-0.34282
90.3	-0.5786	-0.34274
90.317	-0.5786	-0.34269
90.333	-0.57859	-0.34262
90.35	-0.57859	-0.34256
90.368	-0.57859	-0.34251
90.383	-0.57859	-0.34242
90.4	-0.57859	-0.34233
90.417	-0.5786	-0.34227
90.434	-0.57861	-0.3422
90.451	-0.57861	-0.34213
90.468	-0.57861	-0.34205
90.484	-0.57861	-0.34195
90.501	-0.5786	-0.34186

90.517	-0.5786	-0.34177
90.534	-0.5786	-0.34168
90.55	-0.57859	-0.3416
90.567	-0.57859	-0.34153
90.586	-0.57859	-0.34144
90.601	-0.57859	-0.34135
90.617	-0.57859	-0.34125
90.634	-0.57859	-0.34117
90.651	-0.5786	-0.3411
90.667	-0.5786	-0.34102
90.685	-0.5786	-0.34094
90.701	-0.57861	-0.34088
90.718	-0.57862	-0.34081
90.734	-0.57862	-0.34073
90.751	-0.57863	-0.34064
90.768	-0.57863	-0.34057
90.784	-0.57863	-0.34049
90.801	-0.57864	-0.34042
90.817	-0.57864	-0.34035
90.834	-0.57864	-0.3403
90.851	-0.57865	-0.34025
90.868	-0.57866	-0.3402
90.885	-0.57867	-0.34014
90.902	-0.57868	-0.3401
90.918	-0.57869	-0.34005
90.935	-0.57871	-0.33999
90.952	-0.57872	-0.33993
90.969	-0.57873	-0.33985
90.985	-0.57873	-0.33976
91.002	-0.57874	-0.33967

91.019	-0.57874	-0.33957
91.037	-0.57874	-0.33949
91.053	-0.57875	-0.33942
91.069	-0.57876	-0.33932
91.086	-0.57877	-0.33922
91.103	-0.57878	-0.33909
91.119	-0.57879	-0.33893
91.135	-0.57881	-0.33878
91.152	-0.57882	-0.33865
91.169	-0.57883	-0.33853
91.185	-0.57884	-0.33847
91.203	-0.57884	-0.33842
91.219	-0.57885	-0.33838
91.236	-0.57886	-0.33835
91.253	-0.57887	-0.33833
91.269	-0.57887	-0.3383
91.286	-0.57888	-0.33827
91.302	-0.57889	-0.33822
91.32	-0.5789	-0.33815
91.337	-0.57891	-0.33806
91.352	-0.57893	-0.33797
91.369	-0.57894	-0.33789
91.386	-0.57895	-0.33782
91.402	-0.57896	-0.33774
91.419	-0.57898	-0.33764
91.436	-0.57899	-0.33755
91.454	-0.57901	-0.33746
91.47	-0.57902	-0.33737
91.487	-0.57903	-0.33729
91.504	-0.57904	-0.33722

91.52	-0.57905	-0.33715
91.537	-0.57906	-0.33709
91.554	-0.57907	-0.33704
91.57	-0.57908	-0.33701
91.588	-0.5791	-0.33696
91.603	-0.57911	-0.33692
91.62	-0.57912	-0.33687
91.637	-0.57913	-0.33684
91.654	-0.57915	-0.33678
91.67	-0.57916	-0.3367
91.687	-0.57918	-0.33665
91.704	-0.5792	-0.33659
91.721	-0.57922	-0.3365
91.737	-0.57924	-0.33641
91.754	-0.57926	-0.33632
91.771	-0.57927	-0.33621
91.787	-0.57929	-0.33609
91.805	-0.57931	-0.33601
91.822	-0.57932	-0.33594
91.838	-0.57934	-0.33587
91.855	-0.57936	-0.33583
91.871	-0.57938	-0.33578
91.888	-0.5794	-0.33573
91.906	-0.57942	-0.33567
91.922	-0.57944	-0.33563
91.938	-0.57946	-0.33556
91.955	-0.57947	-0.33548
91.971	-0.57949	-0.33538
91.987	-0.57951	-0.33528
92.005	-0.57954	-0.33517

92.021	-0.57956	-0.33502
92.038	-0.57958	-0.33491
92.054	-0.5796	-0.3348
92.072	-0.57963	-0.33471
92.089	-0.57965	-0.3346
92.105	-0.57968	-0.33453
92.122	-0.5797	-0.33448
92.139	-0.57973	-0.33445
92.155	-0.57976	-0.33441
92.172	-0.57978	-0.33437
92.188	-0.57981	-0.33432
92.205	-0.57983	-0.33425
92.221	-0.57985	-0.33417
92.238	-0.57986	-0.33406
92.255	-0.57988	-0.33397
92.273	-0.57989	-0.33389
92.289	-0.57991	-0.3338
92.305	-0.57993	-0.33373
92.323	-0.57996	-0.33368
92.339	-0.57998	-0.33363
92.355	-0.58	-0.33356
92.373	-0.58003	-0.33351
92.389	-0.58006	-0.33342
92.406	-0.58009	-0.33329
92.423	-0.58012	-0.33317
92.438	-0.58015	-0.33308
92.455	-0.58017	-0.33297
92.473	-0.58021	-0.33289
92.489	-0.58024	-0.33282
92.506	-0.58026	-0.33276

92.523	-0.58029	-0.33269
92.539	-0.58031	-0.33264
92.556	-0.58033	-0.33258
92.572	-0.58035	-0.33252
92.59	-0.58037	-0.33246
92.607	-0.58039	-0.33239
92.623	-0.58042	-0.33233
92.64	-0.58045	-0.33229
92.657	-0.58049	-0.33224
92.673	-0.58052	-0.33221
92.69	-0.58056	-0.33218
92.707	-0.58059	-0.33212
92.723	-0.58062	-0.33205
92.739	-0.58065	-0.33196
92.757	-0.58069	-0.33187
92.773	-0.58072	-0.33173
92.79	-0.58075	-0.3316
92.807	-0.58079	-0.33147
92.824	-0.58082	-0.33137
92.84	-0.58085	-0.33129
92.857	-0.58087	-0.33125
92.873	-0.58089	-0.33122
92.89	-0.58092	-0.33113
92.907	-0.58094	-0.33107
92.924	-0.58097	-0.33099
92.94	-0.581	-0.33097
92.957	-0.58102	-0.33093
92.975	-0.58105	-0.33088
92.992	-0.58107	-0.33083
93.008	-0.5811	-0.33079

93.025	-0.58112	-0.3307
93.041	-0.58115	-0.33061
93.058	-0.58117	-0.33048
93.074	-0.5812	-0.33033
93.091	-0.58124	-0.33016
93.107	-0.5813	-0.32994
93.124	-0.58134	-0.32978
93.142	-0.58138	-0.32969
93.158	-0.58142	-0.32962
93.176	-0.58146	-0.32957
93.191	-0.58149	-0.32954
93.208	-0.58152	-0.32949
93.225	-0.58155	-0.32947
93.242	-0.58158	-0.32941
93.258	-0.58161	-0.32936
93.276	-0.58163	-0.3293
93.292	-0.58166	-0.32924
93.309	-0.58167	-0.32918
93.324	-0.58169	-0.32913
93.342	-0.58172	-0.32909
93.359	-0.58174	-0.32903
93.375	-0.58177	-0.32898
93.392	-0.58181	-0.32892
93.409	-0.58184	-0.32887
93.426	-0.58188	-0.32874
93.443	-0.58192	-0.32861
93.459	-0.58196	-0.32848
93.476	-0.582	-0.32838
93.492	-0.58203	-0.32825
93.509	-0.58206	-0.32815

93.526	-0.58209	-0.32802
93.543	-0.58212	-0.32791
93.56	-0.58214	-0.32786
93.576	-0.58217	-0.32782
93.592	-0.5822	-0.32778
93.609	-0.58223	-0.32773
93.626	-0.58227	-0.32768
93.643	-0.5823	-0.32761
93.66	-0.58233	-0.32756
93.676	-0.58236	-0.32748
93.693	-0.5824	-0.32738
93.709	-0.58244	-0.32727
93.726	-0.58247	-0.32714
93.742	-0.58251	-0.327
93.759	-0.58254	-0.32687
93.776	-0.58257	-0.32674
93.792	-0.58261	-0.32666
93.81	-0.58264	-0.3266
93.827	-0.58268	-0.32654
93.842	-0.58272	-0.32648
93.86	-0.58276	-0.32644
93.876	-0.58281	-0.32641
93.893	-0.58285	-0.32637
93.91	-0.58289	-0.32633
93.926	-0.58293	-0.32628
93.944	-0.58297	-0.32621
93.961	-0.58301	-0.32615
93.977	-0.58305	-0.32608
93.994	-0.58308	-0.32603
94.011	-0.58312	-0.32598

94.027	-0.58315	-0.32591
94.043	-0.58319	-0.32585
94.06	-0.58322	-0.3258
94.077	-0.58326	-0.32575
94.093	-0.5833	-0.32566
94.11	-0.58333	-0.32556
94.127	-0.58337	-0.32542
94.143	-0.58341	-0.3253
94.16	-0.58345	-0.32521
94.178	-0.58349	-0.32515
94.194	-0.58353	-0.32511
94.21	-0.58357	-0.32507
94.227	-0.58361	-0.32503
94.245	-0.58365	-0.32499
94.261	-0.58368	-0.32495
94.278	-0.58372	-0.32488
94.293	-0.58375	-0.3248
94.311	-0.58379	-0.32469
94.327	-0.58382	-0.32458
94.344	-0.58386	-0.32448
94.36	-0.58389	-0.32441
94.378	-0.58393	-0.32435
94.394	-0.58397	-0.32432
94.411	-0.58401	-0.32429
94.428	-0.58405	-0.32424
94.444	-0.58408	-0.32418
94.461	-0.58412	-0.32412
94.477	-0.58415	-0.32407
94.494	-0.58419	-0.324
94.511	-0.58422	-0.32394

94.528	-0.58425	-0.32386
94.546	-0.58429	-0.3238
94.562	-0.58433	-0.32374
94.579	-0.58438	-0.32369
94.595	-0.58442	-0.32359
94.612	-0.58449	-0.32347
94.629	-0.58456	-0.32337
94.645	-0.58461	-0.32329
94.662	-0.58465	-0.32323
94.678	-0.58469	-0.32315
94.695	-0.58473	-0.32308
94.711	-0.58477	-0.32303
94.728	-0.58481	-0.323
94.745	-0.58484	-0.32297
94.762	-0.58488	-0.32292
94.778	-0.58493	-0.32289
94.795	-0.58497	-0.32284
94.812	-0.58501	-0.32276
94.829	-0.58505	-0.32269
94.845	-0.58509	-0.3226
94.861	-0.58513	-0.32252
94.878	-0.58518	-0.32246
94.895	-0.58522	-0.32239
94.912	-0.58527	-0.3223
94.929	-0.58531	-0.32222
94.945	-0.58535	-0.3221
94.962	-0.5854	-0.32201
94.979	-0.58544	-0.32192
94.996	-0.58548	-0.32184
95.012	-0.58553	-0.32177

95.03	-0.58557	-0.32171
95.046	-0.58562	-0.32164
95.062	-0.58567	-0.32159
95.079	-0.58571	-0.32153
95.096	-0.58576	-0.32145
95.113	-0.5858	-0.32136
95.13	-0.58584	-0.32128
95.146	-0.58588	-0.32121
95.163	-0.58592	-0.32113
95.18	-0.58596	-0.32106
95.196	-0.586	-0.32096
95.213	-0.58604	-0.32087
95.229	-0.58609	-0.3208
95.247	-0.58613	-0.32073
95.263	-0.58618	-0.32068
95.281	-0.58623	-0.32064
95.297	-0.58627	-0.32059
95.313	-0.58632	-0.32055
95.33	-0.58636	-0.32049
95.348	-0.5864	-0.3204
95.364	-0.58644	-0.32028
95.38	-0.58647	-0.32014
95.396	-0.58652	-0.31997
95.413	-0.58657	-0.31981
95.43	-0.58662	-0.31968
95.446	-0.58672	-0.31954
95.462	-0.58678	-0.31947
95.48	-0.58683	-0.31942
95.497	-0.58688	-0.31936
95.514	-0.58693	-0.31928

95.53	-0.58697	-0.31921
95.547	-0.58702	-0.31916
95.564	-0.58707	-0.31911
95.58	-0.58711	-0.31904
95.597	-0.58716	-0.31897
95.614	-0.58721	-0.3189
95.631	-0.58726	-0.31879
95.647	-0.58731	-0.31871
95.664	-0.58735	-0.31863
95.68	-0.5874	-0.31855
95.697	-0.58745	-0.31847
95.714	-0.5875	-0.31842
95.731	-0.58756	-0.31838
95.747	-0.58761	-0.31835
95.763	-0.58766	-0.31832
95.781	-0.58771	-0.31829
95.799	-0.58776	-0.31824
95.815	-0.5878	-0.31818
95.831	-0.58784	-0.31813
95.848	-0.58788	-0.31804
95.865	-0.58793	-0.31791
95.881	-0.58797	-0.31778
95.898	-0.58801	-0.31767
95.915	-0.58805	-0.31758
95.931	-0.5881	-0.31747
95.948	-0.58814	-0.31741
95.965	-0.58818	-0.31736
95.981	-0.58823	-0.31729
95.997	-0.58827	-0.31718
96.015	-0.58831	-0.31711

96.032	-0.58835	-0.31705
96.048	-0.5884	-0.31699
96.065	-0.58844	-0.31694
96.08	-0.58849	-0.31687
96.098	-0.58853	-0.31683
96.114	-0.58858	-0.31676
96.131	-0.58863	-0.31668
96.148	-0.58868	-0.31664
96.165	-0.58873	-0.31661
96.181	-0.58878	-0.31658
96.198	-0.58884	-0.31652
96.216	-0.58889	-0.31646
96.232	-0.58895	-0.31639
96.249	-0.58901	-0.31634
96.266	-0.58906	-0.31629
96.281	-0.5891	-0.31624
96.298	-0.58916	-0.31616
96.315	-0.58921	-0.31608
96.331	-0.58927	-0.31604
96.349	-0.58933	-0.31599
96.366	-0.5894	-0.31596
96.382	-0.58946	-0.31592
96.399	-0.58954	-0.31589
96.416	-0.58963	-0.31584
96.433	-0.58971	-0.31578
96.45	-0.58978	-0.3157
96.466	-0.58984	-0.31561
96.483	-0.58989	-0.31552
96.499	-0.58994	-0.31544
96.515	-0.59	-0.31536

96.532	-0.59004	-0.31525
96.549	-0.59009	-0.31515
96.566	-0.59013	-0.31505
96.583	-0.59018	-0.31498
96.599	-0.59023	-0.31487
96.616	-0.59028	-0.31482
96.632	-0.59033	-0.31476
96.649	-0.59039	-0.31468
96.666	-0.59044	-0.3146
96.683	-0.5905	-0.31454
96.701	-0.59055	-0.31449
96.716	-0.59061	-0.31443
96.733	-0.59067	-0.31438
96.75	-0.59077	-0.31435
96.766	-0.59085	-0.31431
96.784	-0.59094	-0.31427
96.801	-0.59101	-0.31423
96.816	-0.59108	-0.31418
96.833	-0.59118	-0.31415
96.85	-0.59128	-0.31412
96.867	-0.59134	-0.31406
96.884	-0.5914	-0.31399
96.9	-0.59145	-0.31393
96.918	-0.59151	-0.31387
96.934	-0.59157	-0.31382
96.95	-0.59164	-0.31377
96.967	-0.59169	-0.31372
96.984	-0.59175	-0.31365
97	-0.59181	-0.31358
97.017	-0.59188	-0.31348

97.033	-0.59195	-0.31341
97.05	-0.59202	-0.31334
97.068	-0.5921	-0.31327
97.085	-0.59216	-0.31317
97.101	-0.59222	-0.3131
97.117	-0.59228	-0.31302
97.133	-0.59233	-0.31292
97.151	-0.59238	-0.31283
97.167	-0.59243	-0.31275
97.184	-0.59249	-0.31268
97.202	-0.59255	-0.3126
97.217	-0.59261	-0.3125
97.235	-0.59267	-0.31241
97.251	-0.59276	-0.31232
97.268	-0.59284	-0.31223
97.285	-0.59292	-0.31218
97.3	-0.593	-0.31212
97.318	-0.59306	-0.31205
97.334	-0.59313	-0.31199
97.352	-0.59319	-0.31194
97.368	-0.59325	-0.31188
97.385	-0.59335	-0.31179
97.402	-0.59341	-0.31167
97.419	-0.59349	-0.31151
97.435	-0.59356	-0.31134
97.451	-0.59363	-0.31121
97.468	-0.59369	-0.31108
97.484	-0.59376	-0.31101
97.502	-0.59383	-0.31091
97.519	-0.59391	-0.31087

97.535	-0.59397	-0.31084
97.552	-0.59404	-0.31081
97.569	-0.5941	-0.31078
97.585	-0.59417	-0.31073
97.603	-0.59424	-0.31069
97.619	-0.5943	-0.31061
97.636	-0.59438	-0.31056
97.652	-0.59446	-0.3105
97.668	-0.59456	-0.31043
97.685	-0.59467	-0.31033
97.702	-0.59477	-0.31024
97.719	-0.59486	-0.31012
97.736	-0.59496	-0.31002
97.752	-0.59505	-0.30996
97.769	-0.59517	-0.30989
97.786	-0.59524	-0.30985
97.802	-0.5953	-0.30981
97.818	-0.59537	-0.30976
97.836	-0.59544	-0.3097
97.852	-0.5955	-0.30964
97.869	-0.59556	-0.30957
97.886	-0.59562	-0.3095
97.902	-0.59568	-0.30943
97.919	-0.59574	-0.30937
97.936	-0.59581	-0.30932
97.954	-0.59591	-0.30925
97.97	-0.59601	-0.30919
97.987	-0.59617	-0.30912
98.003	-0.59632	-0.30903
98.021	-0.59649	-0.30894

98.037	-0.59669	-0.30882
98.053	-0.59683	-0.30873
98.07	-0.59692	-0.30866
98.086	-0.59699	-0.30859
98.103	-0.59705	-0.3085
98.119	-0.59712	-0.30847
98.136	-0.5972	-0.30841
98.153	-0.59728	-0.30836
98.17	-0.59736	-0.30832
98.186	-0.59743	-0.30828
98.204	-0.5975	-0.30825
98.22	-0.59757	-0.30821
98.237	-0.59763	-0.30817
98.254	-0.59771	-0.30813
98.27	-0.59777	-0.30809
98.288	-0.59784	-0.30801
98.303	-0.59791	-0.30796
98.32	-0.59798	-0.30787
98.337	-0.59805	-0.30776
98.354	-0.59815	-0.30767
98.37	-0.59828	-0.30761
98.388	-0.59847	-0.30754
98.404	-0.59867	-0.30746
98.421	-0.59883	-0.3074
98.437	-0.59892	-0.30734
98.454	-0.59899	-0.30727
98.47	-0.59907	-0.30719
98.487	-0.59914	-0.3071
98.504	-0.59921	-0.30703
98.522	-0.59928	-0.30696

98.538	-0.59935	-0.30689
98.554	-0.59942	-0.30682
98.571	-0.59949	-0.30675
98.588	-0.59955	-0.30668
98.604	-0.59962	-0.30664
98.621	-0.59969	-0.3066
98.638	-0.59976	-0.30652
98.655	-0.59983	-0.30643
98.671	-0.59992	-0.30637
98.688	-0.60008	-0.30631
98.705	-0.60021	-0.30625
98.722	-0.60034	-0.30618
98.738	-0.60047	-0.30609
98.756	-0.60054	-0.30604
98.771	-0.60062	-0.30601
98.789	-0.60069	-0.30597
98.804	-0.60076	-0.30593
98.822	-0.60086	-0.30587
98.839	-0.60094	-0.30578
98.855	-0.60101	-0.30571
98.871	-0.60108	-0.30561
98.889	-0.60115	-0.30551
98.905	-0.60122	-0.30541
98.922	-0.6013	-0.30533
98.938	-0.60138	-0.30527
98.955	-0.60145	-0.30522
98.972	-0.60155	-0.30518
98.989	-0.60166	-0.30515
99.006	-0.6018	-0.30511
99.022	-0.60189	-0.30507

99.039	-0.60196	-0.30498
99.056	-0.60206	-0.30489
99.073	-0.60213	-0.3048
99.089	-0.6022	-0.3047
99.106	-0.60228	-0.3046
99.122	-0.60238	-0.30452
99.139	-0.60255	-0.30443
99.156	-0.60269	-0.30435
99.172	-0.60286	-0.30428
99.189	-0.60304	-0.30422
99.206	-0.60314	-0.30417
99.222	-0.60326	-0.30412
99.239	-0.60334	-0.30408
99.256	-0.60342	-0.30402
99.273	-0.60349	-0.30397
99.289	-0.60357	-0.30392
99.307	-0.60371	-0.30388
99.323	-0.60384	-0.30384
99.339	-0.60398	-0.30377
99.357	-0.60417	-0.30369
99.373	-0.60432	-0.3036
99.389	-0.6045	-0.30352
99.406	-0.60465	-0.30344
99.423	-0.60474	-0.30336
99.44	-0.60485	-0.3033
99.456	-0.60493	-0.30324
99.474	-0.60504	-0.30318
99.491	-0.60516	-0.30314
99.506	-0.60525	-0.30311
99.525	-0.60532	-0.30307

99.54	-0.6054	-0.30304
99.557	-0.60557	-0.30296
99.574	-0.60574	-0.30287
99.59	-0.60585	-0.30275
99.607	-0.60593	-0.3026
99.623	-0.60601	-0.30249
99.64	-0.60608	-0.3024
99.657	-0.60616	-0.30228
99.673	-0.60625	-0.30221
99.691	-0.60633	-0.30217
99.707	-0.6064	-0.30213
99.723	-0.60648	-0.3021
99.741	-0.60656	-0.30207
99.757	-0.60674	-0.30203
99.774	-0.60697	-0.30199
99.791	-0.60714	-0.30195
99.808	-0.60731	-0.3019
99.825	-0.6075	-0.30185
99.842	-0.60776	-0.3018
99.858	-0.60794	-0.30176
99.875	-0.60806	-0.30171
99.89	-0.60819	-0.30164
99.908	-0.6083	-0.30156
99.925	-0.60842	-0.30147
99.94	-0.60855	-0.30138
99.957	-0.60862	-0.30129
99.974	-0.60873	-0.30118
99.991	-0.60881	-0.30108
100.007	-0.60889	-0.30099
100.025	-0.60898	-0.30091

100.042	-0.6091	-0.30083
100.058	-0.6092	-0.30077
100.075	-0.60933	-0.30069
100.091	-0.60951	-0.3006
100.109	-0.60969	-0.30049
100.124	-0.60988	-0.30041
100.142	-0.61006	-0.30032
100.159	-0.61023	-0.30022
100.175	-0.61035	-0.30016
100.192	-0.61043	-0.30008
100.209	-0.61051	-0.30001
100.226	-0.61059	-0.29994
100.242	-0.61067	-0.29988
100.259	-0.61074	-0.29983
100.275	-0.61083	-0.29977
100.293	-0.6109	-0.29972
100.308	-0.61099	-0.29968
100.326	-0.61107	-0.29962
100.343	-0.61115	-0.29956
100.359	-0.61126	-0.29951
100.377	-0.61137	-0.29943
100.393	-0.61151	-0.29936
100.41	-0.61163	-0.2993
100.426	-0.61174	-0.29924
100.443	-0.61186	-0.2992
100.46	-0.61198	-0.29916
100.476	-0.61206	-0.29913
100.493	-0.61214	-0.2991
100.51	-0.61222	-0.29905
100.527	-0.6123	-0.29898

100.543	-0.61238	-0.29888
100.56	-0.61248	-0.29876
100.576	-0.61257	-0.29858
100.592	-0.61267	-0.29843
100.609	-0.61276	-0.2983
100.626	-0.61293	-0.29823
100.643	-0.61313	-0.29818
100.66	-0.61328	-0.29813
100.677	-0.61344	-0.2981
100.692	-0.6136	-0.29807
100.71	-0.61371	-0.29803
100.726	-0.61381	-0.298
100.743	-0.61391	-0.29796
100.76	-0.61399	-0.2979
100.777	-0.61413	-0.29782
100.793	-0.6142	-0.29769
100.81	-0.61428	-0.29754
100.827	-0.6144	-0.29739
100.844	-0.61453	-0.29724
100.86	-0.61472	-0.29707
100.877	-0.61489	-0.29695
100.893	-0.61502	-0.29687
100.91	-0.61518	-0.29681
100.927	-0.61537	-0.29675
100.943	-0.61555	-0.29669
100.96	-0.61574	-0.29664
100.977	-0.61603	-0.29658
100.994	-0.61629	-0.29646
101.01	-0.6166	-0.29638
101.027	-0.61679	-0.2963

101.043	-0.61694	-0.29621
101.061	-0.61705	-0.29613
101.078	-0.61712	-0.29606
101.094	-0.6172	-0.29602
101.11	-0.61734	-0.29599
101.128	-0.6175	-0.29596
101.143	-0.61757	-0.29593
101.161	-0.61765	-0.29586
101.177	-0.61775	-0.29579
101.194	-0.61795	-0.29576
101.211	-0.61813	-0.29573
101.228	-0.61829	-0.29568
101.244	-0.61855	-0.2956
101.262	-0.61892	-0.2955
101.277	-0.61925	-0.29539
101.294	-0.61953	-0.29527
101.311	-0.61974	-0.29518
101.328	-0.61987	-0.29509
101.345	-0.62001	-0.295
101.36	-0.62017	-0.29496
101.377	-0.62026	-0.29493
101.394	-0.6204	-0.2949
101.412	-0.62049	-0.29486
101.429	-0.62059	-0.29483
101.446	-0.62077	-0.29478
101.462	-0.62096	-0.29473
101.478	-0.6211	-0.29468
101.496	-0.62124	-0.29461
101.512	-0.62142	-0.29453
101.528	-0.62156	-0.29444

101.544	-0.62174	-0.29436
101.562	-0.6219	-0.29429
101.578	-0.62209	-0.29423
101.595	-0.6223	-0.2942
101.612	-0.62248	-0.29416
101.628	-0.62267	-0.29412
101.645	-0.62291	-0.29408
101.662	-0.62309	-0.29403
101.678	-0.62323	-0.29399
101.695	-0.62343	-0.29396
101.712	-0.62362	-0.29391
101.728	-0.62379	-0.29384
101.746	-0.62394	-0.29374
101.762	-0.6241	-0.29359
101.779	-0.62435	-0.29342
101.795	-0.62457	-0.29332
101.812	-0.62476	-0.29318
101.829	-0.62503	-0.29308
101.845	-0.6253	-0.29297
101.862	-0.62556	-0.29285
101.879	-0.62585	-0.29279
101.895	-0.62606	-0.29275
101.913	-0.62628	-0.29272
101.929	-0.62651	-0.29269
101.946	-0.62679	-0.29266
101.963	-0.62717	-0.29262
101.979	-0.6275	-0.29259
101.997	-0.62781	-0.29256
102.013	-0.62807	-0.29253
102.03	-0.62825	-0.2925

102.046	-0.62841	-0.29246
102.063	-0.62855	-0.29242
102.08	-0.62861	-0.29235
102.096	-0.62867	-0.2923
102.114	-0.62874	-0.29221
102.13	-0.6288	-0.29214
102.146	-0.62887	-0.29208
102.163	-0.62901	-0.29204
102.179	-0.62911	-0.29198
102.197	-0.62923	-0.29192
102.213	-0.62935	-0.29189
102.229	-0.62957	-0.29184
102.246	-0.6298	-0.2918
102.264	-0.63003	-0.29174
102.28	-0.63027	-0.2917
102.297	-0.63059	-0.29165
102.313	-0.63094	-0.2916
102.33	-0.63129	-0.29155
102.347	-0.63173	-0.2915
102.364	-0.63211	-0.29142
102.38	-0.63242	-0.29133
102.398	-0.63277	-0.29124
102.414	-0.63299	-0.29112
102.43	-0.63318	-0.29101
102.448	-0.63326	-0.29091
102.464	-0.63334	-0.29083
102.481	-0.63343	-0.29079
102.498	-0.63352	-0.29076
102.514	-0.63363	-0.29072
102.531	-0.6338	-0.29065

102.548	-0.63405	-0.29062
102.564	-0.63425	-0.29059
102.582	-0.63443	-0.29055
102.598	-0.6346	-0.29051
102.615	-0.6347	-0.29047
102.631	-0.63478	-0.29041
102.648	-0.63485	-0.29034
102.665	-0.63491	-0.29027
102.682	-0.63496	-0.29023
102.699	-0.63502	-0.29019
102.715	-0.63511	-0.29013
102.732	-0.63524	-0.29007
102.749	-0.63537	-0.29
102.765	-0.63551	-0.28989
102.782	-0.63565	-0.28977
102.798	-0.6359	-0.28966
102.815	-0.63625	-0.28957
102.831	-0.63655	-0.28949
102.848	-0.63681	-0.28939
102.865	-0.63709	-0.28933
102.881	-0.63741	-0.2893
102.898	-0.63765	-0.28926
102.916	-0.6378	-0.28921
102.932	-0.63794	-0.28918
102.949	-0.63806	-0.28913
102.965	-0.63815	-0.28908
102.982	-0.6383	-0.28902
103	-0.63848	-0.28895
103.016	-0.6387	-0.28888
103.032	-0.63895	-0.28882

103.05	-0.63916	-0.28876
103.066	-0.6394	-0.2887
103.083	-0.6396	-0.28862
103.1	-0.63974	-0.28855
103.116	-0.63982	-0.28848
103.133	-0.63989	-0.2884
103.148	-0.64	-0.28831
103.166	-0.64017	-0.28823
103.183	-0.6404	-0.28814
103.199	-0.64073	-0.28806
103.215	-0.64105	-0.28799
103.232	-0.64128	-0.28794
103.25	-0.64152	-0.28789
103.266	-0.6417	-0.28785
103.282	-0.64188	-0.28781
103.3	-0.64209	-0.28778
103.315	-0.64226	-0.28775
103.333	-0.64243	-0.28771
103.349	-0.64264	-0.28768
103.367	-0.64289	-0.28762
103.382	-0.64318	-0.28756
103.4	-0.6435	-0.28748
103.416	-0.6438	-0.28739
103.433	-0.64408	-0.28728
103.449	-0.64435	-0.28717
103.465	-0.64461	-0.28704
103.483	-0.64478	-0.28694
103.5	-0.64489	-0.28688
103.516	-0.64501	-0.28685
103.534	-0.64514	-0.28681

103.549	-0.6453	-0.28677
103.566	-0.64547	-0.28673
103.583	-0.64565	-0.28668
103.599	-0.64581	-0.28659
103.615	-0.64599	-0.28651
103.634	-0.64624	-0.28645
103.651	-0.6465	-0.28636
103.667	-0.64677	-0.28628
103.683	-0.64699	-0.28619
103.702	-0.64728	-0.28615
103.717	-0.64756	-0.28612
103.734	-0.64787	-0.28607
103.751	-0.64815	-0.28602
103.767	-0.64847	-0.28596
103.785	-0.64875	-0.2859
103.801	-0.649	-0.28585
103.816	-0.64926	-0.28577
103.834	-0.64954	-0.28569
103.85	-0.64985	-0.28562
103.867	-0.65017	-0.28557
103.883	-0.65055	-0.28554
103.9	-0.65086	-0.28551
103.917	-0.6511	-0.28547
103.933	-0.65129	-0.28544
103.952	-0.65152	-0.2854
103.968	-0.65171	-0.28537
103.985	-0.65189	-0.28533
104.001	-0.6521	-0.28529
104.017	-0.65228	-0.28523
104.036	-0.65247	-0.28514

104.051	-0.65269	-0.28507
104.069	-0.65295	-0.28499
104.085	-0.6532	-0.28491
104.102	-0.65343	-0.28485
104.119	-0.65373	-0.2848
104.135	-0.654	-0.28476
104.151	-0.65435	-0.28469
104.169	-0.65468	-0.28465
104.185	-0.65493	-0.28462
104.202	-0.65517	-0.28457
104.219	-0.65536	-0.28454
104.235	-0.65556	-0.2845
104.252	-0.65579	-0.28443
104.269	-0.65603	-0.28436
104.285	-0.65636	-0.2843
104.302	-0.65671	-0.28427
104.317	-0.65705	-0.28423
104.335	-0.65738	-0.2842
104.352	-0.65776	-0.28416
104.368	-0.65803	-0.28412
104.386	-0.65834	-0.28407
104.402	-0.65859	-0.284
104.418	-0.65884	-0.28392
104.436	-0.65906	-0.28383
104.452	-0.65941	-0.28374
104.47	-0.65976	-0.28361
104.486	-0.66014	-0.28348
104.502	-0.66056	-0.2834
104.519	-0.66089	-0.28335
104.535	-0.66112	-0.28329

104.552	-0.6613	-0.28324
104.569	-0.66147	-0.2832
104.586	-0.66163	-0.28316
104.603	-0.6618	-0.28312
104.62	-0.66201	-0.28308
104.637	-0.66232	-0.28304
104.653	-0.66258	-0.28299
104.67	-0.66279	-0.28293
104.686	-0.66307	-0.28286
104.703	-0.66338	-0.28281
104.72	-0.66365	-0.28276
104.737	-0.66396	-0.28272
104.753	-0.66428	-0.28267
104.77	-0.66453	-0.28262
104.786	-0.66473	-0.28258
104.804	-0.66497	-0.28255
104.82	-0.66533	-0.28251
104.837	-0.66572	-0.28247
104.854	-0.66607	-0.28241
104.871	-0.66641	-0.28232
104.887	-0.66664	-0.28223
104.904	-0.66695	-0.28215
104.921	-0.66718	-0.28209
104.937	-0.66738	-0.28203
104.953	-0.66752	-0.28196
104.97	-0.66762	-0.28191
104.987	-0.66771	-0.28187
105.004	-0.66788	-0.28182
105.02	-0.66807	-0.28179
105.038	-0.6683	-0.28175

105.055	-0.66856	-0.2817
105.071	-0.66881	-0.28165
105.087	-0.66913	-0.2816
105.105	-0.66949	-0.28157
105.121	-0.66986	-0.28153
105.138	-0.67018	-0.28149
105.155	-0.67044	-0.28145
105.172	-0.6707	-0.28142
105.189	-0.67109	-0.28137
105.205	-0.67146	-0.2813
105.221	-0.67174	-0.28121
105.238	-0.67202	-0.28113
105.254	-0.6724	-0.28103
105.271	-0.67274	-0.28091
105.288	-0.67314	-0.28079
105.305	-0.67352	-0.28065
105.321	-0.6738	-0.28051
105.338	-0.67401	-0.28042
105.355	-0.67424	-0.28032
105.372	-0.67444	-0.28027
105.388	-0.67465	-0.28021
105.405	-0.67476	-0.28017
105.422	-0.67489	-0.28013
105.438	-0.67504	-0.2801
105.455	-0.67519	-0.28006
105.472	-0.67539	-0.28002
105.49	-0.6756	-0.27997
105.506	-0.67578	-0.27989
105.522	-0.67596	-0.2798
105.539	-0.67613	-0.27972

105.555	-0.67636	-0.27963
105.571	-0.67663	-0.27954
105.589	-0.67683	-0.27947
105.606	-0.67709	-0.27942
105.622	-0.67742	-0.27937
105.639	-0.67773	-0.27934
105.656	-0.67816	-0.2793
105.672	-0.67857	-0.27927
105.689	-0.67902	-0.27923
105.707	-0.67942	-0.27919
105.723	-0.67968	-0.27915
105.74	-0.67994	-0.2791
105.755	-0.68026	-0.27902
105.773	-0.68054	-0.27895
105.789	-0.68087	-0.27888
105.806	-0.68116	-0.27881
105.822	-0.68143	-0.27875
105.839	-0.68176	-0.27866
105.856	-0.68204	-0.27855
105.872	-0.68228	-0.27843
105.889	-0.68257	-0.27833
105.906	-0.68282	-0.27826
105.923	-0.6831	-0.27819
105.939	-0.68357	-0.27812
105.956	-0.68401	-0.27806
105.973	-0.68446	-0.27801
105.989	-0.68487	-0.27797
106.006	-0.6852	-0.27793
106.022	-0.68547	-0.27788
106.04	-0.68571	-0.27782

106.056	-0.68592	-0.27776
106.073	-0.6861	-0.27773
106.09	-0.68625	-0.2777
106.107	-0.6864	-0.27766
106.124	-0.6866	-0.27761
106.14	-0.68687	-0.27756
106.156	-0.68711	-0.27747
106.173	-0.68743	-0.27738
106.191	-0.68776	-0.27731
106.207	-0.68821	-0.27722
106.223	-0.68855	-0.27712
106.24	-0.68891	-0.27703
106.257	-0.68922	-0.27693
106.273	-0.68953	-0.27685
106.29	-0.6898	-0.2768
106.307	-0.69003	-0.27676
106.323	-0.69028	-0.27671
106.339	-0.69056	-0.27663
106.357	-0.69096	-0.27658
106.373	-0.69132	-0.27654
106.391	-0.69184	-0.2765
106.407	-0.69233	-0.27644
106.424	-0.6928	-0.2764
106.441	-0.69322	-0.27635
106.457	-0.69358	-0.27631
106.474	-0.69391	-0.27627
106.49	-0.69419	-0.27624
106.506	-0.69441	-0.2762
106.525	-0.69465	-0.27617
106.541	-0.69496	-0.27613

106.556	-0.69522	-0.2761
106.574	-0.6955	-0.27603
106.591	-0.69582	-0.27595
106.607	-0.69612	-0.27587
106.624	-0.69639	-0.27579
106.64	-0.69672	-0.2757
106.657	-0.69708	-0.27561
106.674	-0.69749	-0.27554
106.691	-0.69788	-0.27544
106.707	-0.69838	-0.27536
106.724	-0.699	-0.27528
106.741	-0.69954	-0.27524
106.757	-0.70004	-0.27521
106.774	-0.7004	-0.27517
106.791	-0.70067	-0.27514
106.807	-0.70088	-0.2751
106.825	-0.7011	-0.27507
106.841	-0.70138	-0.27503
106.858	-0.70173	-0.27499
106.874	-0.7021	-0.27496
106.891	-0.70249	-0.27492
106.908	-0.70291	-0.27489
106.924	-0.7033	-0.27485
106.941	-0.70377	-0.27481
106.957	-0.70423	-0.27478
106.974	-0.70458	-0.27474
106.992	-0.70488	-0.27468
107.008	-0.7052	-0.27463
107.024	-0.70552	-0.27455
107.041	-0.70583	-0.27448

107.058	-0.70618	-0.2744
107.074	-0.70656	-0.27431
107.092	-0.70691	-0.27423
107.108	-0.70736	-0.27416
107.124	-0.70781	-0.27411
107.141	-0.70821	-0.27407
107.158	-0.70859	-0.27403
107.176	-0.70889	-0.27399
107.192	-0.70912	-0.27395
107.209	-0.70948	-0.27391
107.225	-0.7099	-0.27388
107.242	-0.7103	-0.27384
107.26	-0.7108	-0.27381
107.275	-0.71134	-0.27377
107.292	-0.71185	-0.27372
107.309	-0.7124	-0.27365
107.326	-0.71281	-0.27359
107.343	-0.71322	-0.27353
107.359	-0.71358	-0.27346
107.376	-0.71385	-0.27339
107.392	-0.71411	-0.2733
107.408	-0.71435	-0.27324
107.426	-0.71461	-0.2732
107.441	-0.71481	-0.27316
107.459	-0.71498	-0.27311
107.475	-0.71516	-0.27308
107.492	-0.71538	-0.27303
107.509	-0.71561	-0.27298
107.526	-0.71591	-0.27293
107.543	-0.71629	-0.27286

107.559	-0.7167	-0.27282
107.575	-0.71718	-0.27278
107.591	-0.71759	-0.27274
107.609	-0.71801	-0.27271
107.625	-0.71842	-0.27267
107.641	-0.71884	-0.27264
107.66	-0.71927	-0.2726
107.676	-0.71973	-0.27256
107.692	-0.7202	-0.27253
107.709	-0.7207	-0.27246
107.726	-0.72128	-0.27235
107.744	-0.72178	-0.27224
107.76	-0.72233	-0.27213
107.776	-0.72272	-0.27202
107.793	-0.723	-0.27195
107.81	-0.72319	-0.27186
107.826	-0.72345	-0.27179
107.843	-0.72375	-0.27172
107.859	-0.72405	-0.27166
107.876	-0.72442	-0.2716
107.892	-0.72482	-0.27155
107.91	-0.72521	-0.27146
107.926	-0.72552	-0.27139
107.944	-0.72587	-0.27133
107.959	-0.72631	-0.27129
107.977	-0.72676	-0.27126
107.994	-0.72716	-0.27122
108.01	-0.72752	-0.27119
108.027	-0.72788	-0.27115
108.043	-0.72821	-0.27112

108.06	-0.72855	-0.27107
108.077	-0.72887	-0.27104
108.092	-0.72921	-0.271
108.111	-0.7296	-0.27096
108.127	-0.73005	-0.27091
108.144	-0.73071	-0.27087
108.161	-0.73124	-0.27084
108.177	-0.73178	-0.2708
108.194	-0.73231	-0.27076
108.212	-0.73279	-0.27071
108.228	-0.73319	-0.27068
108.245	-0.73364	-0.27064
108.261	-0.73406	-0.27057
108.278	-0.73441	-0.2705
108.294	-0.73487	-0.27043
108.311	-0.73532	-0.27038
108.327	-0.7358	-0.27034
108.343	-0.73624	-0.27027
108.361	-0.73673	-0.2702
108.378	-0.73722	-0.27016
108.395	-0.73771	-0.27012
108.411	-0.73824	-0.27006
108.428	-0.73874	-0.27
108.444	-0.73918	-0.26995
108.461	-0.73967	-0.26989
108.478	-0.7402	-0.26983
108.494	-0.74067	-0.26978
108.512	-0.74121	-0.26973
108.527	-0.74177	-0.26969
108.544	-0.74226	-0.26964

108.562	-0.7427	-0.26958
108.578	-0.74316	-0.26953
108.594	-0.74366	-0.26947
108.612	-0.74413	-0.26942
108.629	-0.7445	-0.26938
108.645	-0.74481	-0.26934
108.662	-0.74504	-0.2693
108.678	-0.74525	-0.26925
108.695	-0.74549	-0.2692
108.712	-0.74582	-0.26917
108.728	-0.7462	-0.26913
108.745	-0.74678	-0.26909
108.762	-0.74739	-0.26905
108.778	-0.74802	-0.26901
108.795	-0.74873	-0.26897
108.812	-0.74949	-0.26893
108.827	-0.75022	-0.2689
108.845	-0.75085	-0.26886
108.861	-0.75128	-0.26881
108.878	-0.75168	-0.26876
108.895	-0.75202	-0.26872
108.911	-0.75232	-0.26868
108.928	-0.75264	-0.26863
108.946	-0.7531	-0.26858
108.962	-0.75351	-0.2685
108.979	-0.75394	-0.26843
108.995	-0.75438	-0.26835
109.012	-0.75474	-0.26829
109.029	-0.75505	-0.26822
109.045	-0.75547	-0.26815

109.062	-0.75598	-0.26806
109.078	-0.75646	-0.26801
109.096	-0.75701	-0.26797
109.112	-0.75756	-0.26792
109.128	-0.75831	-0.26786
109.145	-0.7591	-0.26781
109.162	-0.75976	-0.26775
109.179	-0.7604	-0.2677
109.196	-0.7611	-0.26766
109.213	-0.7615	-0.26762
109.229	-0.76183	-0.26758
109.246	-0.76207	-0.2675
109.262	-0.76223	-0.26744
109.279	-0.76234	-0.2674
109.296	-0.76248	-0.26736
109.311	-0.76267	-0.26733
109.329	-0.76287	-0.26729
109.346	-0.7631	-0.26725
109.362	-0.76336	-0.26721
109.38	-0.76369	-0.26717
109.396	-0.76411	-0.26713
109.412	-0.76463	-0.2671
109.429	-0.76514	-0.26706
109.445	-0.76568	-0.26702
109.462	-0.76623	-0.26698
109.48	-0.76684	-0.26694
109.496	-0.76747	-0.2669
109.513	-0.76807	-0.26686
109.529	-0.76871	-0.26682
109.547	-0.76927	-0.26678

109.563	-0.76979	-0.26674
109.58	-0.77031	-0.2667
109.597	-0.77085	-0.26666
109.614	-0.77118	-0.26662
109.63	-0.77137	-0.26658
109.647	-0.77154	-0.26653
109.663	-0.77172	-0.26648
109.68	-0.77189	-0.26644
109.697	-0.77224	-0.2664
109.712	-0.7726	-0.26636
109.73	-0.77301	-0.26631
109.746	-0.77355	-0.26627
109.764	-0.77409	-0.26623
109.78	-0.77468	-0.26619
109.797	-0.77526	-0.26615
109.813	-0.77582	-0.26609
109.83	-0.77639	-0.26603
109.847	-0.77706	-0.26597
109.864	-0.77756	-0.26591
109.88	-0.77813	-0.26583
109.898	-0.7786	-0.26574
109.914	-0.77915	-0.26564
109.931	-0.77973	-0.26557
109.946	-0.78022	-0.2655
109.963	-0.78063	-0.26545
109.98	-0.78116	-0.26541
109.996	-0.78176	-0.26537
110.014	-0.78236	-0.26533
110.031	-0.78316	-0.26529
110.047	-0.78384	-0.26525

110.064	-0.78434	-0.26521
110.08	-0.78481	-0.26515
110.097	-0.78519	-0.2651
110.114	-0.78551	-0.26506
110.131	-0.78576	-0.26499
110.146	-0.786	-0.26494
110.164	-0.78626	-0.26489
110.181	-0.78655	-0.26485
110.198	-0.78693	-0.26481
110.214	-0.78751	-0.26477
110.231	-0.78815	-0.26473
110.247	-0.7888	-0.26469
110.264	-0.78955	-0.26465
110.281	-0.79004	-0.26461
110.297	-0.79053	-0.26457
110.313	-0.79104	-0.26453
110.331	-0.79147	-0.26447
110.347	-0.79183	-0.26441
110.365	-0.79218	-0.26432
110.381	-0.79265	-0.26428
110.397	-0.79307	-0.26423
110.414	-0.79353	-0.26418
110.431	-0.79396	-0.26414
110.447	-0.79461	-0.26409
110.465	-0.79533	-0.26405
110.481	-0.79603	-0.26401
110.498	-0.79673	-0.26397
110.515	-0.79745	-0.26393
110.531	-0.79797	-0.26389
110.548	-0.79843	-0.26381

110.565	-0.79888	-0.26373
110.581	-0.79934	-0.26368
110.597	-0.79971	-0.2636
110.615	-0.80013	-0.26354
110.631	-0.80064	-0.26346
110.647	-0.80105	-0.26341
110.665	-0.80148	-0.26335
110.681	-0.80193	-0.26331
110.698	-0.80241	-0.26327
110.714	-0.8029	-0.26323
110.731	-0.80338	-0.26319
110.749	-0.80382	-0.26315
110.766	-0.80428	-0.2631
110.781	-0.80475	-0.26306
110.798	-0.8053	-0.26302
110.814	-0.8058	-0.26298
110.831	-0.80625	-0.26294
110.848	-0.80666	-0.2629
110.864	-0.80712	-0.26286
110.882	-0.80753	-0.26281
110.898	-0.8079	-0.26278
110.915	-0.80823	-0.26274
110.931	-0.80851	-0.2627
110.948	-0.8089	-0.26266
110.966	-0.80929	-0.26262
110.982	-0.80984	-0.26258
110.998	-0.8106	-0.26254
111.015	-0.81137	-0.2625
111.032	-0.81231	-0.26242
111.049	-0.81331	-0.26237

111.065	-0.81428	-0.26233
111.082	-0.815	-0.26229
111.098	-0.81542	-0.26225
111.114	-0.81574	-0.26221
111.133	-0.81604	-0.26217
111.149	-0.81621	-0.26213
111.165	-0.81641	-0.26209
111.182	-0.81673	-0.26205
111.199	-0.81705	-0.26201
111.215	-0.81755	-0.26197
111.233	-0.81823	-0.26193
111.25	-0.81891	-0.26188
111.265	-0.81948	-0.26182
111.283	-0.81993	-0.26177
111.298	-0.82034	-0.26173
111.315	-0.82078	-0.26169
111.332	-0.82123	-0.26164
111.349	-0.82158	-0.26158
111.366	-0.82209	-0.26152
111.383	-0.82262	-0.26148
111.4	-0.8231	-0.26144
111.417	-0.82364	-0.2614
111.433	-0.82411	-0.26136
111.45	-0.82461	-0.26132
111.465	-0.82512	-0.26127
111.482	-0.82567	-0.26123
111.499	-0.82634	-0.26119
111.515	-0.82704	-0.26115
111.533	-0.82761	-0.26111
111.549	-0.82834	-0.26107

111.566	-0.82906	-0.26103
111.584	-0.82938	-0.26099
111.6	-0.82963	-0.26095
111.616	-0.82983	-0.26091
111.634	-0.82998	-0.26088
111.65	-0.83024	-0.26084
111.667	-0.83051	-0.2608
111.684	-0.83094	-0.26075
111.7	-0.83147	-0.2607
111.717	-0.83214	-0.26065
111.733	-0.83281	-0.26059
111.75	-0.83349	-0.26051
111.767	-0.83413	-0.26044
111.783	-0.83471	-0.2604
111.8	-0.83533	-0.26035
111.817	-0.83591	-0.26031
111.833	-0.8364	-0.26027
111.85	-0.83693	-0.26022
111.868	-0.83752	-0.26018
111.883	-0.83798	-0.26014
111.901	-0.83851	-0.2601
111.917	-0.83888	-0.26006
111.933	-0.8392	-0.26001
111.95	-0.83955	-0.25996
111.967	-0.83996	-0.25992
111.983	-0.84034	-0.25988
112.001	-0.84079	-0.25984
112.017	-0.84119	-0.2598
112.034	-0.84168	-0.25976
112.05	-0.8423	-0.25971

112.067	-0.84303	-0.25967
112.084	-0.84377	-0.25963
112.1	-0.84435	-0.25957
112.116	-0.8449	-0.25953
112.133	-0.84543	-0.25948
112.15	-0.84597	-0.25944
112.168	-0.84636	-0.2594
112.184	-0.84671	-0.25936
112.2	-0.84711	-0.25932
112.218	-0.84741	-0.25928
112.233	-0.84779	-0.25924
112.25	-0.84828	-0.2592
112.268	-0.84873	-0.25916
112.284	-0.84917	-0.25912
112.301	-0.84978	-0.25908
112.317	-0.85029	-0.25903
112.334	-0.85099	-0.25899
112.35	-0.8515	-0.25894
112.367	-0.85189	-0.2589
112.385	-0.85237	-0.25885
112.401	-0.85279	-0.2588
112.419	-0.85334	-0.25875
112.434	-0.8539	-0.25871
112.45	-0.8545	-0.25867
112.468	-0.85507	-0.25863
112.484	-0.85562	-0.25859
112.501	-0.85615	-0.25854
112.518	-0.85667	-0.25851
112.534	-0.85708	-0.25847
112.551	-0.85745	-0.25843

112.569	-0.85778	-0.25839
112.586	-0.85809	-0.25835
112.602	-0.85847	-0.25831
112.618	-0.85882	-0.25827
112.635	-0.85912	-0.25823
112.652	-0.85936	-0.25819
112.668	-0.85966	-0.25815
112.685	-0.86007	-0.2581
112.701	-0.86059	-0.25806
112.718	-0.86114	-0.25803
112.734	-0.86176	-0.25798
112.752	-0.8624	-0.25794
112.769	-0.86316	-0.2579
112.785	-0.86387	-0.25786
112.802	-0.86452	-0.25782
112.819	-0.86513	-0.25778
112.836	-0.86562	-0.25774
112.852	-0.86605	-0.2577
112.869	-0.86657	-0.25766
112.885	-0.86717	-0.2576
112.901	-0.86765	-0.25754
112.918	-0.86809	-0.25748
112.935	-0.86855	-0.25743
112.952	-0.86904	-0.25738
112.968	-0.86954	-0.25734
112.985	-0.87003	-0.25731
113.002	-0.87043	-0.25727
113.019	-0.87075	-0.25723
113.036	-0.87098	-0.25719
113.053	-0.87128	-0.25715

113.069	-0.87169	-0.25711
113.086	-0.87204	-0.25707
113.103	-0.87235	-0.25702
113.119	-0.87275	-0.25696
113.137	-0.8731	-0.2569
113.152	-0.87345	-0.25684
113.169	-0.87377	-0.25679
113.187	-0.87407	-0.25676
113.203	-0.87449	-0.25672
113.219	-0.87495	-0.25668
113.235	-0.87538	-0.25664
113.253	-0.87584	-0.2566
113.27	-0.87635	-0.25656
113.286	-0.87675	-0.25652
113.303	-0.87713	-0.25647
113.319	-0.87748	-0.25641
113.336	-0.87779	-0.25633
113.354	-0.87811	-0.25628
113.369	-0.87846	-0.25624
113.386	-0.87898	-0.2562
113.402	-0.8796	-0.25616
113.419	-0.88028	-0.25612
113.436	-0.88106	-0.25608
113.452	-0.88176	-0.25604
113.469	-0.88228	-0.256
113.487	-0.88285	-0.25596
113.503	-0.88346	-0.25591
113.52	-0.88406	-0.25587
113.537	-0.88452	-0.25583
113.553	-0.88501	-0.25579

113.569	-0.88546	-0.25574
113.585	-0.88585	-0.25568
113.603	-0.88618	-0.2556
113.619	-0.88651	-0.25556
113.635	-0.88676	-0.25552
113.652	-0.88704	-0.25548
113.67	-0.88741	-0.25544
113.686	-0.88779	-0.2554
113.702	-0.88822	-0.25536
113.72	-0.88868	-0.25532
113.737	-0.88917	-0.25528
113.753	-0.88967	-0.25525
113.769	-0.89018	-0.25521
113.787	-0.89069	-0.25517
113.803	-0.89123	-0.25513
113.82	-0.8917	-0.2551
113.837	-0.89215	-0.25506
113.853	-0.89253	-0.25501
113.87	-0.89289	-0.25497
113.886	-0.89332	-0.25492
113.903	-0.89364	-0.25484
113.919	-0.894	-0.25478
113.935	-0.89445	-0.25473
113.953	-0.89489	-0.25469
113.969	-0.89532	-0.25465
113.987	-0.89577	-0.2546
114.002	-0.89615	-0.25456
114.02	-0.89657	-0.25452
114.036	-0.89689	-0.25448
114.053	-0.89723	-0.25444

114.069	-0.89766	-0.25441
114.086	-0.89798	-0.25437
114.103	-0.89834	-0.25433
114.12	-0.89866	-0.25429
114.137	-0.89904	-0.25426
114.153	-0.89945	-0.25422
114.17	-0.89988	-0.25417
114.186	-0.90029	-0.25413
114.203	-0.90065	-0.25409
114.22	-0.90106	-0.25405
114.237	-0.90136	-0.25401
114.253	-0.90165	-0.25397
114.271	-0.90202	-0.25393
114.286	-0.90242	-0.2539
114.304	-0.90277	-0.25386
114.32	-0.90322	-0.25383
114.337	-0.90373	-0.25379
114.353	-0.90421	-0.25375
114.369	-0.90483	-0.25371
114.386	-0.90534	-0.25367
114.402	-0.90586	-0.25363
114.42	-0.90631	-0.25359
114.437	-0.9067	-0.25355
114.454	-0.90703	-0.25351
114.47	-0.90739	-0.25348
114.487	-0.90766	-0.25344
114.503	-0.90795	-0.25341
114.52	-0.9083	-0.25337
114.537	-0.90867	-0.25333
114.553	-0.9091	-0.25329

114.57	-0.90966	-0.25326
114.588	-0.91011	-0.25322
114.603	-0.91065	-0.25318
114.62	-0.91109	-0.25314
114.637	-0.91142	-0.2531
114.653	-0.91173	-0.25307
114.67	-0.91195	-0.25303
114.687	-0.91212	-0.253
114.703	-0.91234	-0.25296
114.72	-0.91269	-0.25292
114.737	-0.91297	-0.25289
114.754	-0.91335	-0.25285
114.77	-0.91385	-0.25282
114.787	-0.91453	-0.25278
114.804	-0.91528	-0.25274
114.821	-0.91595	-0.2527
114.837	-0.91665	-0.25266
114.854	-0.91713	-0.25263
114.871	-0.91748	-0.25259
114.888	-0.91776	-0.25255
114.904	-0.91797	-0.25251
114.92	-0.91811	-0.25246
114.937	-0.91829	-0.25242
114.954	-0.91859	-0.25238
114.969	-0.91895	-0.25234
114.987	-0.91942	-0.2523
115.004	-0.91997	-0.25227
115.021	-0.9206	-0.25223
115.038	-0.92134	-0.25219
115.054	-0.92206	-0.25215

115.071	-0.92263	-0.25212
115.088	-0.92305	-0.25208
115.104	-0.92342	-0.25205
115.121	-0.9238	-0.25201
115.139	-0.92404	-0.25198
115.154	-0.92425	-0.25194
115.172	-0.92445	-0.25191
115.188	-0.92464	-0.25187
115.205	-0.9249	-0.25184
115.221	-0.92523	-0.2518
115.238	-0.92559	-0.25177
115.253	-0.92588	-0.25173
115.271	-0.92626	-0.25169
115.288	-0.92664	-0.25166
115.305	-0.92704	-0.25162
115.32	-0.92752	-0.25159
115.338	-0.928	-0.25155
115.355	-0.92846	-0.25152
115.371	-0.92912	-0.25148
115.387	-0.9298	-0.25145
115.405	-0.93042	-0.25142
115.421	-0.93103	-0.25138
115.438	-0.93157	-0.25135
115.455	-0.93208	-0.25132
115.472	-0.93246	-0.25128
115.488	-0.93281	-0.25125
115.504	-0.93316	-0.25122
115.522	-0.93354	-0.25118
115.539	-0.934	-0.25115
115.555	-0.93457	-0.25112

115.572	-0.93526	-0.25108
115.589	-0.93569	-0.25105
115.605	-0.93616	-0.25101
115.622	-0.93659	-0.25098
115.638	-0.93707	-0.25094
115.655	-0.9375	-0.25091
115.672	-0.9378	-0.25087
115.688	-0.93816	-0.25083
115.705	-0.93857	-0.2508
115.722	-0.93906	-0.25076
115.739	-0.9395	-0.25073
115.755	-0.93989	-0.25069
115.773	-0.94023	-0.25066
115.788	-0.94063	-0.25062
115.805	-0.94096	-0.25059
115.822	-0.94141	-0.25056
115.839	-0.94184	-0.25052
115.856	-0.94233	-0.25049
115.872	-0.94282	-0.25045
115.889	-0.94325	-0.2504
115.906	-0.94356	-0.2503
115.923	-0.94381	-0.25022
115.939	-0.9441	-0.25015
115.956	-0.94442	-0.25009
115.972	-0.9448	-0.25
115.989	-0.94519	-0.24993
116.006	-0.94564	-0.24986
116.022	-0.94602	-0.24981
116.039	-0.94657	-0.24978
116.056	-0.94715	-0.24974

116.073	-0.94769	-0.2497
116.089	-0.94811	-0.24967
116.106	-0.9486	-0.24963
116.123	-0.94905	-0.2496
116.139	-0.9495	-0.24956
116.156	-0.94995	-0.24953
116.173	-0.95033	-0.24949
116.189	-0.95074	-0.24945
116.207	-0.95111	-0.24941
116.223	-0.95139	-0.24937
116.24	-0.95165	-0.24934
116.256	-0.95194	-0.2493
116.273	-0.95218	-0.24926
116.289	-0.9526	-0.24921
116.306	-0.95312	-0.24917
116.323	-0.95369	-0.24911
116.338	-0.95436	-0.24906
116.356	-0.95489	-0.24896
116.372	-0.95546	-0.24889
116.388	-0.95588	-0.24884
116.405	-0.95621	-0.2488
116.422	-0.95655	-0.24876
116.439	-0.95687	-0.24873
116.456	-0.95722	-0.24869
116.472	-0.95776	-0.24866
116.49	-0.95826	-0.24862
116.506	-0.95864	-0.24859
116.523	-0.95901	-0.24855
116.54	-0.95952	-0.24851
116.557	-0.96009	-0.24848

116.573	-0.96056	-0.24844
116.59	-0.96096	-0.2484
116.606	-0.96144	-0.24836
116.623	-0.96194	-0.24832
116.639	-0.9624	-0.24829
116.655	-0.96286	-0.24825
116.673	-0.96332	-0.24821
116.688	-0.96373	-0.24818
116.706	-0.96399	-0.24814
116.722	-0.96422	-0.2481
116.739	-0.96451	-0.24807
116.756	-0.96474	-0.24803
116.773	-0.96501	-0.24799
116.789	-0.96532	-0.24796
116.807	-0.96566	-0.24793
116.823	-0.96596	-0.24789
116.84	-0.96635	-0.24786
116.856	-0.96675	-0.24783
116.872	-0.96724	-0.24779
116.89	-0.96785	-0.24776
116.907	-0.96848	-0.24772
116.923	-0.96914	-0.24769
116.94	-0.96976	-0.24765
116.957	-0.97026	-0.24761
116.974	-0.9707	-0.24758
116.991	-0.97122	-0.24755
117.007	-0.97156	-0.24752
117.024	-0.97186	-0.24749
117.04	-0.97212	-0.24745
117.057	-0.97239	-0.24743

117.073	-0.97277	-0.24739
117.09	-0.97322	-0.24736
117.107	-0.97369	-0.24733
117.124	-0.97431	-0.24729
117.141	-0.97485	-0.24726
117.157	-0.97539	-0.24722
117.174	-0.97581	-0.24719
117.192	-0.97615	-0.24716
117.207	-0.97639	-0.24713
117.225	-0.97662	-0.24709
117.241	-0.97682	-0.24706
117.258	-0.97709	-0.24703
117.275	-0.97738	-0.247
117.291	-0.9777	-0.24697
117.308	-0.97811	-0.24694
117.324	-0.97855	-0.24691
117.341	-0.9791	-0.24688
117.357	-0.97988	-0.24684
117.374	-0.98067	-0.24681
117.39	-0.98139	-0.24678
117.408	-0.9821	-0.24675
117.424	-0.98257	-0.24671
117.441	-0.98295	-0.24667
117.457	-0.98331	-0.24664
117.475	-0.98358	-0.2466
117.491	-0.98377	-0.24657
117.508	-0.984	-0.24653
117.524	-0.98426	-0.2465
117.541	-0.98456	-0.24646
117.558	-0.98489	-0.24643

117.575	-0.98515	-0.2464
117.592	-0.98548	-0.24636
117.609	-0.98583	-0.24633
117.625	-0.98626	-0.2463
117.642	-0.98665	-0.24626
117.659	-0.98692	-0.24623
117.676	-0.98719	-0.2462
117.692	-0.98746	-0.24616
117.709	-0.98778	-0.24613
117.725	-0.98819	-0.24609
117.742	-0.98864	-0.24606
117.759	-0.98897	-0.24602
117.776	-0.98936	-0.24599
117.793	-0.98974	-0.24595
117.81	-0.99022	-0.24592
117.825	-0.99065	-0.24588
117.842	-0.99101	-0.24585
117.859	-0.99125	-0.2458
117.876	-0.99143	-0.24575
117.892	-0.99162	-0.24568
117.909	-0.99192	-0.24562
117.926	-0.99219	-0.24558
117.941	-0.99239	-0.24554
117.959	-0.99259	-0.24551
117.976	-0.99281	-0.24547
117.992	-0.99308	-0.24544
118.009	-0.99341	-0.24541
118.025	-0.99384	-0.24538
118.042	-0.99417	-0.24535
118.06	-0.99449	-0.24532

118.076	-0.99479	-0.24528
118.093	-0.99515	-0.24525
118.109	-0.99552	-0.24521
118.126	-0.99591	-0.24518
118.144	-0.99625	-0.24515
118.16	-0.99661	-0.24511
118.176	-0.99695	-0.24507
118.193	-0.99719	-0.24504
118.21	-0.99747	-0.245
118.226	-0.99772	-0.24497
118.243	-0.99798	-0.24493
118.26	-0.9982	-0.24489
118.276	-0.99852	-0.24486
118.293	-0.99882	-0.24482
118.309	-0.99919	-0.24479
118.327	-0.99953	-0.24475
118.343	-0.99983	-0.24472
118.359	-1.00018	-0.24469
118.376	-1.00059	-0.24465
118.393	-1.00102	-0.24462
118.41	-1.00148	-0.24459
118.426	-1.00204	-0.24455
118.443	-1.0025	-0.24451
118.46	-1.00289	-0.24447
118.476	-1.00321	-0.24442
118.492	-1.00356	-0.24436
118.51	-1.00391	-0.24432
118.526	-1.00422	-0.24426
118.543	-1.00448	-0.24422
118.559	-1.00478	-0.24419

118.576	-1.00508	-0.24415
118.592	-1.00544	-0.24412
118.609	-1.0058	-0.24409
118.626	-1.00625	-0.24406
118.643	-1.00657	-0.24403
118.66	-1.00686	-0.244
118.677	-1.0072	-0.24396
118.693	-1.00759	-0.24393
118.711	-1.00796	-0.24389
118.726	-1.00843	-0.24385
118.743	-1.0089	-0.2438
118.759	-1.00922	-0.24377
118.776	-1.00952	-0.24373
118.792	-1.00975	-0.2437
118.81	-1.01011	-0.24367
118.826	-1.01046	-0.24364
118.842	-1.01079	-0.2436
118.86	-1.01108	-0.24357
118.877	-1.01134	-0.24353
118.893	-1.01162	-0.2435
118.91	-1.01197	-0.24344
118.926	-1.01247	-0.24338
118.943	-1.01302	-0.24334
118.959	-1.01362	-0.2433
118.976	-1.01417	-0.24327
118.992	-1.0147	-0.24324
119.009	-1.01511	-0.24321
119.026	-1.01546	-0.24318
119.042	-1.01575	-0.24315
119.059	-1.01602	-0.24312

119.076	-1.01639	-0.24309
119.092	-1.01662	-0.24306
119.109	-1.01681	-0.24303
119.126	-1.01716	-0.24299
119.142	-1.0176	-0.24296
119.16	-1.01804	-0.24292
119.175	-1.01843	-0.24288
119.194	-1.01882	-0.24285
119.209	-1.01922	-0.24281
119.225	-1.01958	-0.24278
119.243	-1.01995	-0.24274
119.26	-1.02032	-0.24271
119.276	-1.0206	-0.24268
119.293	-1.0208	-0.24264
119.31	-1.02099	-0.24261
119.327	-1.02118	-0.24258
119.344	-1.0214	-0.24255
119.36	-1.02168	-0.24252
119.377	-1.02198	-0.24249
119.393	-1.02235	-0.24246
119.41	-1.02281	-0.24243
119.426	-1.02337	-0.2424
119.444	-1.02399	-0.24237
119.46	-1.02446	-0.24234
119.477	-1.02488	-0.24231
119.494	-1.02529	-0.24228
119.511	-1.02568	-0.24225
119.527	-1.02603	-0.24222
119.543	-1.02634	-0.24219
119.56	-1.0266	-0.24216

119.577	-1.02687	-0.24213
119.593	-1.02709	-0.2421
119.611	-1.0274	-0.24206
119.626	-1.02782	-0.24203
119.644	-1.0282	-0.242
119.66	-1.02858	-0.24197
119.676	-1.02896	-0.24193
119.693	-1.02934	-0.2419
119.71	-1.02966	-0.24187
119.727	-1.03 -0.24184	
119.744	-1.0303	-0.24181
119.76	-1.03055	-0.24178
119.777	-1.03076	-0.24174
119.794	-1.03093	-0.24171
119.811	-1.0311	-0.24168
119.827	-1.03132	-0.24165
119.845	-1.03165	-0.24162
119.861	-1.03201	-0.24159
119.878	-1.03237	-0.24156
119.894	-1.03263	-0.24153
119.912	-1.03287	-0.2415
119.928	-1.03304	-0.24147
119.944	-1.03328	-0.24144
119.961	-1.03356	-0.24141
119.977	-1.0338	-0.24138
119.994	-1.03402	-0.24135
120.01	-1.03423	-0.24131
120.027	-1.03446	-0.24128
120.043	-1.03473	-0.24125
120.061	-1.03503	-0.24122

120.077	-1.03537	-0.24119
120.095	-1.03569	-0.24116
120.111	-1.03593	-0.24113
120.127	-1.03616	-0.2411
120.145	-1.03638	-0.24107
120.161	-1.03665	-0.24104
120.178	-1.03691	-0.24101
120.194	-1.03708	-0.24098
120.211	-1.03728	-0.24095
120.227	-1.03746	-0.24091
120.244	-1.03767	-0.24088
120.26	-1.0379	-0.24085
120.277	-1.03819	-0.24082
120.295	-1.03851	-0.24079
120.311	-1.03891	-0.24076
120.327	-1.03924	-0.24073
120.344	-1.0396	-0.2407
120.361	-1.03995	-0.24067
120.379	-1.04026	-0.24064
120.395	-1.04052	-0.24061
120.412	-1.0407	-0.24058
120.429	-1.04088	-0.24055
120.445	-1.0411	-0.24052
120.461	-1.04126	-0.24049
120.478	-1.0414	-0.24046
120.495	-1.04152	-0.24043
120.511	-1.04166	-0.2404
120.529	-1.04183	-0.24037
120.545	-1.04199	-0.24035
120.561	-1.04214	-0.24031

120.578	-1.04225	-0.24028
120.595	-1.04239	-0.24025
120.611	-1.04257	-0.24022
120.628	-1.04273	-0.24019
120.645	-1.04295	-0.24016
120.661	-1.04315	-0.24013
120.678	-1.04331	-0.24009
120.696	-1.04344	-0.24006
120.712	-1.04363	-0.24003
120.728	-1.04381	-0.24
120.745	-1.04396	-0.23996
120.762	-1.04413	-0.23993
120.78	-1.04433	-0.2399
120.795	-1.04456	-0.23988
120.811	-1.04479	-0.23985
120.828	-1.04499	-0.23982
120.845	-1.04517	-0.23979
120.862	-1.04533	-0.23976
120.878	-1.04547	-0.23973
120.895	-1.04569	-0.2397
120.911	-1.04591	-0.23967
120.928	-1.04614	-0.23964
120.946	-1.04637	-0.23961
120.962	-1.04661	-0.23958
120.978	-1.0468	-0.23956
120.995	-1.04697	-0.23953
121.013	-1.04707	-0.23951
121.029	-1.04716	-0.23948
121.046	-1.04727	-0.23945
121.062	-1.04738	-0.23942

121.078	-1.04756	-0.23939
121.095	-1.04777	-0.23936
121.113	-1.04798	-0.23933
121.129	-1.04819	-0.2393
121.146	-1.04834	-0.23927
121.162	-1.04845	-0.23924
121.179	-1.04857	-0.23922
121.195	-1.04867	-0.23919
121.212	-1.04875	-0.23916
121.229	-1.04893	-0.23913
121.246	-1.04918	-0.2391
121.263	-1.04949	-0.23908
121.279	-1.04985	-0.23905
121.296	-1.05026	-0.23902
121.313	-1.05071	-0.23899
121.329	-1.05108	-0.23896
121.346	-1.05146	-0.23893
121.363	-1.05182	-0.2389
121.38	-1.05223	-0.23887
121.396	-1.05263	-0.23884
121.413	-1.05301	-0.23881
121.429	-1.05342	-0.23878
121.446	-1.05382	-0.23876
121.463	-1.05423	-0.23873
121.48	-1.05453	-0.2387
121.495	-1.05481	-0.23868
121.513	-1.05503	-0.23865
121.529	-1.05523	-0.23863
121.546	-1.0554	-0.2386
121.563	-1.05559	-0.23857

121.58	-1.05582	-0.23854
121.597	-1.05606	-0.23852
121.614	-1.0564	-0.23849
121.63	-1.05686	-0.23846
121.646	-1.05741	-0.23843
121.663	-1.05799	-0.2384
121.68	-1.05854	-0.23837
121.696	-1.059	-0.23835
121.713	-1.05944	-0.23832
121.73	-1.05973	-0.23829
121.746	-1.05999	-0.23826
121.763	-1.06021	-0.23823
121.779	-1.06044	-0.2382
121.797	-1.06075	-0.23818
121.813	-1.06107	-0.23815
121.83	-1.0614	-0.23812
121.847	-1.06185	-0.2381
121.863	-1.06232	-0.23807
121.88	-1.06273	-0.23805
121.897	-1.06313	-0.23802
121.913	-1.0635	-0.238
121.93	-1.06389	-0.23797
121.947	-1.06426	-0.23794
121.964	-1.06467	-0.23791
121.981	-1.06513	-0.23788
121.998	-1.06554	-0.23785
122.013	-1.06618	-0.23782
122.03	-1.06706	-0.23779
122.047	-1.06789	-0.23776
122.064	-1.06865	-0.23773

122.08	-1.06926	-0.23771
122.097	-1.0698	-0.23768
122.114	-1.07034	-0.23766
122.131	-1.07084	-0.23763
122.148	-1.0714	-0.23761
122.164	-1.07203	-0.23759
122.181	-1.07271	-0.23756
122.198	-1.07337	-0.23754
122.214	-1.07396	-0.23752
122.231	-1.07451	-0.23749
122.248	-1.0751	-0.23746
122.265	-1.07567	-0.23743
122.28	-1.07631	-0.2374
122.297	-1.07688	-0.23737
122.314	-1.07736	-0.23734
122.329	-1.07788	-0.23731
122.347	-1.07831	-0.23728
122.364	-1.07869	-0.23725
122.381	-1.07896	-0.23722
122.398	-1.07908	-0.2372
122.415	-1.07916	-0.23717
122.43	-1.07918	-0.23715
122.447	-1.0792	-0.23712
122.463	-1.07921	-0.2371
122.481	-1.0792	-0.23707
122.498	-1.07913	-0.23705
122.515	-1.07904	-0.23702
122.532	-1.07896	-0.237
122.548	-1.07891	-0.23697
122.565	-1.07886	-0.23694

122.581	-1.07879	-0.23691
122.597	-1.07872	-0.23688
122.614	-1.07865	-0.23685
122.631	-1.07856	-0.23683
122.648	-1.07845	-0.2368
122.664	-1.07833	-0.23677
122.681	-1.07822	-0.23675
122.697	-1.07813	-0.23672
122.714	-1.07804	-0.2367
122.731	-1.07795	-0.23668
122.748	-1.07786	-0.23665
122.765	-1.07776	-0.23663
122.781	-1.07765	-0.23661
122.798	-1.07757	-0.23658
122.814	-1.07747	-0.23655
122.83	-1.07734	-0.23651
122.848	-1.07719	-0.23648
122.863	-1.07705	-0.23645
122.881	-1.07697	-0.23642
122.897	-1.07684	-0.2364
122.914	-1.0767	-0.23638
122.93	-1.07647	-0.23635
122.947	-1.07621	-0.23633
122.963	-1.07577	-0.23631
122.981	-1.0753	-0.23629
122.997	-1.07483	-0.23626
123.014	-1.07432	-0.23623
123.03	-1.07381	-0.23621
123.047	-1.07338	-0.23618
123.063	-1.07312	-0.23615

123.081	-1.07285	-0.23612
123.097	-1.07256	-0.23609
123.114	-1.07228	-0.23607
123.131	-1.072	-0.23604
123.148	-1.07161	-0.23602
123.164	-1.07108	-0.23599
123.181	-1.07058	-0.23597
123.198	-1.07013	-0.23594
123.214	-1.06977	-0.23592
123.232	-1.0694	-0.23589
123.248	-1.06907	-0.23587
123.265	-1.0688	-0.23584
123.281	-1.06849	-0.23582
123.299	-1.06806	-0.23579
123.314	-1.0677	-0.23577
123.332	-1.06715	-0.23575
123.348	-1.06652	-0.23573
123.364	-1.06598	-0.2357
123.381	-1.06533	-0.23568
123.397	-1.06462	-0.23565
123.414	-1.06384	-0.23563
123.431	-1.06301	-0.2356
123.448	-1.06219	-0.23557
123.465	-1.06146	-0.23554
123.481	-1.06083	-0.23552
123.497	-1.06032	-0.23549
123.513	-1.05972	-0.23547
123.531	-1.05913	-0.23545
123.548	-1.05872	-0.23543
123.565	-1.05836	-0.2354

123.581	-1.058	-0.23538
123.598	-1.05752	-0.23535
123.615	-1.05675	-0.23533
123.631	-1.05593	-0.2353
123.648	-1.0551	-0.23528
123.665	-1.05439	-0.23526
123.681	-1.0536	-0.23524
123.698	-1.05278	-0.23522
123.715	-1.05204	-0.2352
123.732	-1.0512	-0.23518
123.747	-1.05021	-0.23516
123.765	-1.04929	-0.23514
123.782	-1.04837	-0.23512
123.798	-1.04754	-0.23509
123.815	-1.04655	-0.23507
123.832	-1.0456	-0.23504
123.847	-1.04474	-0.23501
123.865	-1.04414	-0.23499
123.882	-1.04359	-0.23496
123.898	-1.04304	-0.23494
123.915	-1.04238	-0.23492
123.931	-1.04169	-0.2349
123.948	-1.04108	-0.23488
123.965	-1.04049	-0.23486
123.982	-1.03998	-0.23484
123.999	-1.03944	-0.23482
124.015	-1.03885	-0.2348
124.032	-1.0381	-0.23478
124.048	-1.03737	-0.23476
124.065	-1.03675	-0.23474

124.081	-1.03608	-0.23471
124.099	-1.03547	-0.23469
124.115	-1.03469	-0.23467
124.131	-1.03408	-0.23465
124.148	-1.03333	-0.23463
124.165	-1.03239	-0.23461
124.182	-1.03143	-0.23459
124.199	-1.03059	-0.23456
124.214	-1.02968	-0.23454
124.231	-1.02892	-0.23452
124.248	-1.02808	-0.23449
124.265	-1.02729	-0.23447
124.283	-1.02641	-0.23445
124.298	-1.02529	-0.23442
124.315	-1.02409	-0.2344
124.331	-1.02279	-0.23438
124.349	-1.02121	-0.23436
124.366	-1.01975	-0.23434
124.382	-1.01819	-0.23433
124.399	-1.01675	-0.23431
124.415	-1.01582	-0.23429
124.431	-1.01474	-0.23427
124.449	-1.01393	-0.23425
124.465	-1.0128	-0.23423
124.482	-1.0111	-0.23421
124.499	-1.00987	-0.23418
124.515	-1.00897	-0.23417
124.532	-1.00787	-0.23414
124.548	-1.00635	-0.23413
124.566	-1.00515	-0.23411

124.582	-1.00364	-0.23409
124.599	-1.00219	-0.23407
124.616	-1.00081	-0.23405
124.632	-0.99994	-0.23403
124.649	-0.99848	-0.23401
124.666	-0.99667	-0.23399
124.683	-0.99474	-0.23396
124.7	-0.99279	-0.23394
124.716	-0.99167	-0.23392
124.732	-0.99002	-0.2339
124.749	-0.9887	-0.23388
124.765	-0.9868	-0.23386
124.782	-0.98472	-0.23385
124.799	-0.98384	-0.23383
124.815	-0.98275	-0.23381
124.833	-0.98133	-0.23379
124.849	-0.97959	-0.23377
124.866	-0.97787	-0.23376
124.883	-0.97667	-0.23374
124.9	-0.97524	-0.23372
124.916	-0.97303	-0.2337
124.933	-0.97125	-0.23368
124.95	-0.96954	-0.23367
124.966	-0.96732	-0.23364
124.983	-0.9656	-0.23362
124.998	-0.96405	-0.2336
125.017	-0.96223	-0.23358
125.032	-0.9604	-0.23356
125.05	-0.95845	-0.23354
125.066	-0.95654	-0.23352

125.082	-0.95494	-0.2335
125.099	-0.95286	-0.23348
125.115	-0.95109	-0.23346
125.133	-0.94942	-0.23345
125.15	-0.94743	-0.23343
125.167	-0.94567	-0.23342
125.183	-0.9439	-0.2334
125.201	-0.94165	-0.23338
125.216	-0.93924	-0.23336
125.234	-0.93707	-0.23334
125.25	-0.9349	-0.23332
125.267	-0.93294	-0.2333
125.283	-0.93089	-0.23328
125.299	-0.92905	-0.23327
125.317	-0.927	-0.23325
125.333	-0.92492	-0.23323
125.349	-0.92323	-0.23321
125.366	-0.9214	-0.2332
125.383	-0.91934	-0.23318
125.4	-0.91699	-0.23316
125.416	-0.91499	-0.23314
125.434	-0.91278	-0.23313
125.449	-0.91061	-0.23311
125.466	-0.90819	-0.23309
125.482	-0.90599	-0.23308
125.5	-0.90378	-0.23306
125.516	-0.90152	-0.23305
125.533	-0.89973	-0.23303
125.55	-0.897	-0.23301
125.568	-0.89436	-0.233

125.583	-0.89197	-0.23299
125.6	-0.88987	-0.23297
125.616	-0.88749	-0.23296
125.634	-0.8858	-0.23294
125.65	-0.88321	-0.23292
125.667	-0.88039	-0.2329
125.683	-0.87775	-0.23289
125.7	-0.87525	-0.23287
125.717	-0.87305	-0.23285
125.732	-0.87068	-0.23283
125.751	-0.86736	-0.23282
125.768	-0.86474	-0.2328
125.784	-0.86221	-0.23279
125.801	-0.85907	-0.23277
125.817	-0.85705	-0.23276
125.834	-0.85451	-0.23274
125.851	-0.85176	-0.23272
125.867	-0.84881	-0.2327
125.884	-0.84661	-0.23268
125.9	-0.84397	-0.23266
125.917	-0.84205	-0.23264
125.934	-0.83964	-0.23262
125.95	-0.83714	-0.23261
125.966	-0.83492	-0.23259
125.983	-0.83245	-0.23258
126	-0.83018	-0.23256
126.017	-0.82809	-0.23255
126.034	-0.82593	-0.23254
126.051	-0.82318	-0.23253
126.066	-0.82064	-0.23252

126.083	-0.81779	-0.2325
126.101	-0.81556	-0.23248
126.117	-0.8131	-0.23246
126.134	-0.80962	-0.23245
126.15	-0.80689	-0.23243
126.167	-0.80357	-0.23241
126.183	-0.80083	-0.23238
126.201	-0.79853	-0.23236
126.217	-0.79576	-0.23235
126.234	-0.79347	-0.23233
126.25	-0.79066	-0.23232
126.267	-0.78774	-0.2323
126.284	-0.78499	-0.23229
126.301	-0.78246	-0.23228
126.318	-0.77953	-0.23227
126.334	-0.77653	-0.23225
126.35	-0.77399	-0.23223
126.368	-0.77074	-0.23222
126.384	-0.76814	-0.2322
126.4	-0.76544	-0.23218
126.418	-0.7632	-0.23217
126.434	-0.76119	-0.23215
126.45	-0.75853	-0.23214
126.467	-0.75651	-0.23213
126.485	-0.7544	-0.23212
126.5	-0.75171	-0.2321
126.518	-0.74949	-0.23209
126.535	-0.74741	-0.23207
126.55	-0.74518	-0.23205
126.567	-0.74316	-0.23204

126.585	-0.7402	-0.23202
126.6	-0.73808	-0.232
126.619	-0.73611	-0.23199
126.635	-0.734	-0.23198
126.652	-0.73163	-0.23197
126.667	-0.72941	-0.23196
126.684	-0.727	-0.23195
126.701	-0.72394	-0.23194
126.718	-0.72242	-0.23192
126.734	-0.72023	-0.23191
126.751	-0.71797	-0.23189
126.768	-0.71571	-0.23188
126.785	-0.71381	-0.23186
126.801	-0.71166	-0.23184
126.818	-0.71034	-0.23183
126.834	-0.70809	-0.23181
126.85	-0.70651	-0.2318
126.868	-0.70475	-0.23179
126.884	-0.70283	-0.23178
126.901	-0.70099	-0.23177
126.918	-0.69888	-0.23176
126.934	-0.69636	-0.23174
126.951	-0.69453	-0.23173
126.967	-0.69278	-0.23171
126.984	-0.69083	-0.2317
127.001	-0.68926	-0.23169
127.018	-0.68783	-0.23168
127.034	-0.68607	-0.23167
127.05	-0.68461	-0.23166
127.068	-0.68301	-0.23165

127.085	-0.6815	-0.23164
127.1	-0.67995	-0.23162
127.117	-0.67893	-0.23161
127.134	-0.67735	-0.2316
127.151	-0.67596	-0.23159
127.167	-0.67446	-0.23158
127.184	-0.67264	-0.23156
127.201	-0.67115	-0.23155
127.218	-0.66939	-0.23154
127.234	-0.6675	-0.23152
127.251	-0.66545	-0.23151
127.267	-0.66406	-0.2315
127.285	-0.66298	-0.23148
127.301	-0.66155	-0.23147
127.317	-0.66106	-0.23146
127.334	-0.66044	-0.23145
127.351	-0.65953	-0.23144
127.368	-0.65862	-0.23143
127.384	-0.65677	-0.23142
127.401	-0.65565	-0.23141
127.418	-0.65452	-0.23139
127.434	-0.65304	-0.23138
127.451	-0.65186	-0.23137
127.468	-0.65079	-0.23136
127.484	-0.64934	-0.23135
127.501	-0.64849	-0.23134
127.518	-0.6477	-0.23133
127.534	-0.64671	-0.23132
127.551	-0.64623	-0.23131
127.567	-0.64521	-0.2313

127.585	-0.64434	-0.23129
127.602	-0.64334	-0.23128
127.618	-0.64209	-0.23127
127.635	-0.64116	-0.23126
127.651	-0.63962	-0.23126
127.669	-0.63871	-0.23125
127.684	-0.63779	-0.23124
127.702	-0.63718	-0.23123
127.718	-0.63577	-0.23122
127.735	-0.63502	-0.23121
127.751	-0.63497	-0.23121
127.768	-0.63451	-0.2312
127.784	-0.6338	-0.23119
127.801	-0.63314	-0.23117
127.818	-0.63201	-0.23116
127.834	-0.63168	-0.23115
127.851	-0.63094	-0.23113
127.868	-0.63055	-0.23112
127.884	-0.62973	-0.23111
127.9	-0.6288	-0.2311
127.917	-0.62799	-0.23109
127.934	-0.62769	-0.23108
127.951	-0.62744	-0.23107
127.967	-0.62701	-0.23106
127.984	-0.62644	-0.23105
128.002	-0.62577	-0.23104
128.018	-0.62502	-0.23104
128.034	-0.62418	-0.23103
128.051	-0.62341	-0.23102
128.068	-0.62279	-0.23101

128.085	-0.62209	-0.231
128.101	-0.62139	-0.231
128.119	-0.62073	-0.23099
128.134	-0.62032	-0.23098
128.152	-0.62007	-0.23097
128.168	-0.61985	-0.23096
128.185	-0.6197	-0.23095
128.201	-0.61947	-0.23094
128.217	-0.61909	-0.23093
128.234	-0.61858	-0.23093
128.252	-0.61825	-0.23092
128.267	-0.61795	-0.23091
128.285	-0.61742	-0.23091
128.302	-0.61697	-0.2309
128.317	-0.6167	-0.2309
128.334	-0.61637	-0.23089
128.351	-0.61598	-0.23088
128.367	-0.61569	-0.23087
128.385	-0.61536	-0.23086
128.401	-0.61503	-0.23085
128.418	-0.61473	-0.23084
128.434	-0.61438	-0.23083
128.452	-0.61409	-0.23082
128.468	-0.61375	-0.23081
128.484	-0.61352	-0.23081
128.501	-0.61321	-0.2308
128.519	-0.61297	-0.2308
128.535	-0.61281	-0.23079
128.552	-0.61264	-0.23078
128.569	-0.6125	-0.23077

128.584	-0.61243	-0.23077
128.601	-0.61233	-0.23076
128.617	-0.61223	-0.23075
128.634	-0.6121	-0.23074
128.651	-0.61199	-0.23074
128.668	-0.6119	-0.23073
128.685	-0.61179	-0.23073
128.701	-0.61161	-0.23072
128.718	-0.6114	-0.23071
128.734	-0.6112	-0.2307
128.751	-0.61101	-0.2307
128.768	-0.61084	-0.23069
128.784	-0.61066	-0.23069
128.801	-0.61048	-0.23068
128.818	-0.61036	-0.23068
128.836	-0.61026	-0.23067
128.852	-0.6102	-0.23067
128.869	-0.61012	-0.23066
128.885	-0.61004	-0.23066
128.902	-0.60997	-0.23065
128.918	-0.6099	-0.23065
128.934	-0.60981	-0.23064
128.952	-0.60969	-0.23064
128.968	-0.60953	-0.23063
128.985	-0.60937	-0.23063
129.002	-0.60916	-0.23063
129.018	-0.60897	-0.23062
129.035	-0.60884	-0.23062
129.051	-0.60871	-0.23061
129.069	-0.60865	-0.23061

129.085	-0.60859	-0.23061
129.101	-0.60853	-0.2306
129.118	-0.60849	-0.2306
129.135	-0.60845	-0.23059
129.151	-0.6084	-0.23059
129.168	-0.60834	-0.23058
129.185	-0.60827	-0.23057
129.202	-0.60823	-0.23057
129.219	-0.60818	-0.23057
129.236	-0.60812	-0.23056
129.251	-0.60808	-0.23056
129.268	-0.60805	-0.23055
129.285	-0.60802	-0.23055
129.302	-0.60784	-0.23055
129.318	-0.60782	-0.23054
129.335	-0.60779	-0.23054
129.352	-0.6077	-0.23053
129.368	-0.60762	-0.23053
129.385	-0.60751	-0.23052
129.401	-0.60744	-0.23052
129.418	-0.60737	-0.23051
129.435	-0.60733	-0.23051
129.452	-0.6073	-0.2305
129.468	-0.60728	-0.2305
129.485	-0.60713	-0.2305
129.502	-0.60701	-0.23049
129.518	-0.60692	-0.23049
129.535	-0.60683	-0.23049
129.553	-0.60675	-0.23048
129.569	-0.60673	-0.23048

129.585	-0.60671	-0.23048
129.601	-0.60668	-0.23047
129.618	-0.60664	-0.23047
129.635	-0.60662	-0.23047
129.651	-0.6066	-0.23046
129.669	-0.60657	-0.23046
129.685	-0.60655	-0.23046
129.701	-0.60653	-0.23046
129.719	-0.60647	-0.23046
129.735	-0.60644	-0.23046
129.752	-0.6064	-0.23046
129.768	-0.60635	-0.23046
129.786	-0.6063	-0.23045
129.802	-0.60628	-0.23045
129.819	-0.60627	-0.23044
129.835	-0.60625	-0.23044
129.852	-0.60623	-0.23043
129.869	-0.6062	-0.23043
129.886	-0.60618	-0.23042
129.902	-0.60616	-0.23042
129.919	-0.60614	-0.23042
129.935	-0.60611	-0.23042
129.952	-0.60607	-0.23042
129.969	-0.60606	-0.23042
129.986	-0.60605	-0.23042
130.002	-0.60604	-0.23041
130.019	-0.60603	-0.23041
130.035	-0.60601	-0.23041
130.051	-0.606	-0.23041
130.07	-0.60598	-0.2304

130.087	-0.60596	-0.2304
130.103	-0.60593	-0.2304
130.119	-0.6059	-0.2304
130.135	-0.60588	-0.2304
130.152	-0.60585	-0.23039
130.169	-0.60583	-0.23039
130.185	-0.60581	-0.23039
130.202	-0.60579	-0.23039
130.219	-0.60577	-0.23038
130.235	-0.60576	-0.23038
130.252	-0.60575	-0.23038
130.269	-0.60574	-0.23038
130.284	-0.60572	-0.23038
130.301	-0.60571	-0.23038
130.319	-0.60568	-0.23037
130.335	-0.60566	-0.23037
130.351	-0.60564	-0.23037
130.369	-0.60562	-0.23037
130.385	-0.6056	-0.23036
130.402	-0.60558	-0.23036
130.417	-0.60556	-0.23036
130.435	-0.60555	-0.23035
130.452	-0.60554	-0.23034
130.468	-0.60553	-0.23034
130.485	-0.60552	-0.23034
130.502	-0.60551	-0.23034
130.518	-0.6055	-0.23033
130.534	-0.60548	-0.23033
130.552	-0.60546	-0.23033
130.568	-0.60544	-0.23033

130.585	-0.60542	-0.23033
130.601	-0.60541	-0.23033
130.618	-0.60539	-0.23033
130.634	-0.60538	-0.23033
130.651	-0.60536	-0.23033
130.668	-0.60534	-0.23033
130.685	-0.60533	-0.23033
130.702	-0.60531	-0.23033
130.718	-0.60529	-0.23033
130.735	-0.60528	-0.23034
130.751	-0.60526	-0.23033
130.768	-0.60524	-0.23034
130.785	-0.60523	-0.23034
130.802	-0.60521	-0.23034
130.818	-0.60519	-0.23034
130.836	-0.60517	-0.23034
130.852	-0.60515	-0.23034
130.869	-0.60514	-0.23034
130.885	-0.60512	-0.23033
130.902	-0.6051	-0.23034
130.918	-0.60509	-0.23034
130.935	-0.60508	-0.23034
130.952	-0.60508	-0.23034
130.968	-0.60507	-0.23034
130.986	-0.60506	-0.23035
131.002	-0.60505	-0.23035
131.018	-0.60504	-0.23035
131.035	-0.60503	-0.23034
131.051	-0.60502	-0.23034
131.068	-0.60501	-0.23034

131.085	-0.605	-0.23034
131.102	-0.60499	-0.23034
131.118	-0.60498	-0.23034
131.134	-0.60498	-0.23035
131.152	-0.60498	-0.23035
131.17	-0.60497	-0.23035
131.185	-0.60497	-0.23035
131.201	-0.60496	-0.23035
131.219	-0.60495	-0.23035
131.235	-0.60494	-0.23036
131.252	-0.60492	-0.23036
131.268	-0.6049	-0.23036
131.285	-0.60489	-0.23036
131.302	-0.60487	-0.23036
131.318	-0.60487	-0.23037
131.336	-0.60486	-0.23036
131.352	-0.60485	-0.23036
131.369	-0.60485	-0.23036
131.385	-0.60484	-0.23036
131.401	-0.60484	-0.23037
131.418	-0.60484	-0.23037
131.435	-0.60483	-0.23037
131.452	-0.60483	-0.23037
131.468	-0.60482	-0.23038
131.485	-0.60482	-0.23038
131.502	-0.60481	-0.23038
131.518	-0.60481	-0.23038
131.535	-0.60481	-0.23039
131.552	-0.60481	-0.23039
131.568	-0.6048	-0.23039

131.585	-0.60479	-0.2304
131.602	-0.60478	-0.2304
131.618	-0.60477	-0.23041
131.635	-0.60477	-0.23042
131.653	-0.60476	-0.23042
131.669	-0.60476	-0.23042
131.686	-0.60475	-0.23042
131.702	-0.60474	-0.23043
131.718	-0.60473	-0.23043
131.736	-0.60472	-0.23043
131.752	-0.60472	-0.23043
131.768	-0.60471	-0.23043
131.785	-0.60469	-0.23044
131.803	-0.60468	-0.23044
131.818	-0.60467	-0.23045
131.836	-0.60466	-0.23046
131.851	-0.60465	-0.23046
131.869	-0.60464	-0.23046
131.885	-0.60464	-0.23046
131.902	-0.60464	-0.23046
131.918	-0.60464	-0.23046
131.936	-0.60464	-0.23046
131.951	-0.60464	-0.23046
131.968	-0.60463	-0.23046
131.985	-0.60462	-0.23046
132.001	-0.60461	-0.23046
132.018	-0.60461	-0.23046
132.034	-0.6046	-0.23046
132.051	-0.6046	-0.23046
132.068	-0.6046	-0.23046

132.084	-0.6046	-0.23046
132.101	-0.6046	-0.23046
132.118	-0.60461	-0.23046
132.134	-0.60461	-0.23046
132.151	-0.60461	-0.23046
132.168	-0.6046	-0.23047
132.184	-0.6046	-0.23047
132.2	-0.60459	-0.23047
132.217	-0.60459	-0.23047
132.234	-0.60458	-0.23047
132.25	-0.60458	-0.23047
132.268	-0.60457	-0.23047
132.285	-0.60457	-0.23048
132.302	-0.60457	-0.23048
132.318	-0.60457	-0.23048
132.334	-0.60458	-0.23048
132.351	-0.60457	-0.23049
132.368	-0.60458	-0.23049
132.385	-0.60458	-0.2305
132.401	-0.60459	-0.2305
132.417	-0.60459	-0.23051
132.435	-0.6046	-0.23051
132.451	-0.60461	-0.23052
132.467	-0.60462	-0.23052
132.485	-0.60462	-0.23052
132.5	-0.60463	-0.23052
132.518	-0.60464	-0.23052
132.534	-0.60465	-0.23052
132.551	-0.60466	-0.23052
132.567	-0.60467	-0.23052

132.585	-0.60468	-0.23053
132.602	-0.60471	-0.23053
132.618	-0.60473	-0.23054
132.634	-0.60475	-0.23054
132.651	-0.60476	-0.23055
132.669	-0.60478	-0.23055
132.684	-0.60479	-0.23055
132.701	-0.6048	-0.23055
132.718	-0.60482	-0.23056
132.733	-0.60483	-0.23056
132.751	-0.60484	-0.23056
132.768	-0.60486	-0.23057
132.785	-0.60488	-0.23057
132.801	-0.6049	-0.23057
132.818	-0.60491	-0.23058
132.835	-0.60493	-0.23058
132.852	-0.60495	-0.23058
132.868	-0.60497	-0.23058
132.885	-0.60498	-0.23058
132.901	-0.60499	-0.23058
132.917	-0.60501	-0.23059
132.934	-0.60502	-0.23059
132.951	-0.60503	-0.2306
132.968	-0.60505	-0.2306
132.985	-0.60506	-0.23061
133.001	-0.60507	-0.23061
133.018	-0.60508	-0.23061
133.035	-0.60509	-0.23061
133.051	-0.6051	-0.23062
133.069	-0.6051	-0.23062

133.084	-0.60511	-0.23062
133.101	-0.60512	-0.23062
133.118	-0.60513	-0.23062
133.135	-0.60515	-0.23063
133.152	-0.60517	-0.23063
133.168	-0.60518	-0.23063
133.185	-0.6052	-0.23064
133.202	-0.60522	-0.23064
133.218	-0.60523	-0.23065
133.234	-0.60524	-0.23065
133.252	-0.60525	-0.23066
133.268	-0.60525	-0.23066
133.285	-0.60526	-0.23067
133.302	-0.60526	-0.23067
133.318	-0.60527	-0.23067
133.335	-0.60528	-0.23067
133.351	-0.60528	-0.23068
133.368	-0.60529	-0.23068
133.385	-0.6053	-0.23068
133.402	-0.60532	-0.23068
133.418	-0.60533	-0.23068
133.435	-0.60535	-0.23068
133.451	-0.60536	-0.23069
133.468	-0.60538	-0.23069
133.485	-0.60539	-0.2307
133.501	-0.6054	-0.2307
133.518	-0.60542	-0.23071
133.535	-0.60542	-0.23072
133.551	-0.60543	-0.23072
133.569	-0.60543	-0.23073

133.586	-0.60544	-0.23073
133.602	-0.60545	-0.23073
133.619	-0.60545	-0.23074
133.635	-0.60547	-0.23074
133.651	-0.60548	-0.23074
133.668	-0.6055	-0.23074
133.685	-0.60552	-0.23075
133.702	-0.60554	-0.23075
133.719	-0.60556	-0.23076
133.735	-0.60557	-0.23077
133.752	-0.60558	-0.23077
133.768	-0.60559	-0.23077
133.785	-0.6056	-0.23077
133.801	-0.60561	-0.23077
133.817	-0.60562	-0.23077
133.835	-0.60563	-0.23077
133.852	-0.60564	-0.23077
133.868	-0.60565	-0.23077
133.885	-0.60567	-0.23078
133.902	-0.60569	-0.23078
133.919	-0.6057	-0.23079
133.935	-0.60572	-0.23079
133.952	-0.60572	-0.2308
133.969	-0.60572	-0.2308
133.986	-0.60572	-0.23081
134.003	-0.60573	-0.23081
134.019	-0.60573	-0.23081
134.036	-0.60574	-0.23082
134.052	-0.60574	-0.23082
134.068	-0.60575	-0.23082

134.086	-0.60576	-0.23082
134.102	-0.60577	-0.23082
134.119	-0.60579	-0.23083
134.136	-0.6058	-0.23083
134.152	-0.60582	-0.23083
134.168	-0.60583	-0.23083
134.186	-0.60584	-0.23083
134.202	-0.60586	-0.23083
134.219	-0.60586	-0.23083
134.235	-0.60588	-0.23083
134.252	-0.60589	-0.23083
134.268	-0.60591	-0.23083
134.285	-0.60592	-0.23084
134.302	-0.60593	-0.23084
134.318	-0.60593	-0.23084
134.336	-0.60594	-0.23084
134.352	-0.60594	-0.23085
134.369	-0.60594	-0.23085
134.386	-0.60595	-0.23085
134.402	-0.60596	-0.23085
134.419	-0.60597	-0.23085
134.435	-0.60599	-0.23085
134.452	-0.60601	-0.23085
134.468	-0.60603	-0.23085
134.485	-0.60605	-0.23085
134.502	-0.60607	-0.23085
134.519	-0.60611	-0.23084
134.535	-0.60617	-0.23084
134.551	-0.60622	-0.23084
134.569	-0.60626	-0.23084

134.584	-0.60632	-0.23084
134.602	-0.60636	-0.23084
134.618	-0.60638	-0.23084
134.635	-0.60641	-0.23084
134.652	-0.60643	-0.23084
134.669	-0.60646	-0.23084
134.685	-0.60648	-0.23084
134.702	-0.60651	-0.23084
134.719	-0.60653	-0.23084
134.735	-0.60656	-0.23084
134.751	-0.60658	-0.23084
134.769	-0.60661	-0.23084
134.786	-0.60664	-0.23085
134.802	-0.60667	-0.23085
134.819	-0.60669	-0.23086
134.836	-0.60671	-0.23086
134.852	-0.60674	-0.23086
134.869	-0.60676	-0.23086
134.885	-0.60678	-0.23085
134.903	-0.6068	-0.23085
134.919	-0.60682	-0.23085
134.935	-0.60684	-0.23085
134.952	-0.60686	-0.23085
134.969	-0.60689	-0.23085
134.986	-0.60691	-0.23085
135.002	-0.60694	-0.23085
135.019	-0.60696	-0.23085
135.035	-0.60698	-0.23085
135.052	-0.607	-0.23085
135.069	-0.60702	-0.23085

135.086	-0.60703	-0.23085
135.103	-0.60705	-0.23085
135.119	-0.60706	-0.23085
135.136	-0.60708	-0.23086
135.153	-0.6071	-0.23086
135.169	-0.60711	-0.23086
135.185	-0.60712	-0.23086
135.201	-0.60713	-0.23086
135.218	-0.60714	-0.23085
135.235	-0.60715	-0.23085
135.251	-0.60716	-0.23085
135.268	-0.60717	-0.23084
135.285	-0.60717	-0.23084
135.303	-0.60718	-0.23084
135.318	-0.60718	-0.23084
135.336	-0.60719	-0.23084
135.352	-0.6072	-0.23084
135.368	-0.60721	-0.23084
135.385	-0.60722	-0.23084
135.402	-0.60722	-0.23084
135.418	-0.60723	-0.23084
135.436	-0.60724	-0.23084
135.451	-0.60725	-0.23084
135.469	-0.60727	-0.23084
135.485	-0.60728	-0.23084
135.502	-0.6073	-0.23085
135.519	-0.60732	-0.23085
135.536	-0.60733	-0.23085
135.553	-0.60735	-0.23085
135.569	-0.60736	-0.23084

135.585	-0.60737	-0.23084
135.602	-0.60738	-0.23084
135.619	-0.60739	-0.23083
135.636	-0.6074	-0.23083
135.652	-0.60741	-0.23083
135.669	-0.60742	-0.23083
135.686	-0.60743	-0.23083
135.702	-0.60744	-0.23083
135.719	-0.60745	-0.23083
135.736	-0.60746	-0.23083
135.752	-0.60746	-0.23083
135.769	-0.60747	-0.23083
135.785	-0.60748	-0.23083
135.802	-0.60749	-0.23083
135.818	-0.60751	-0.23083
135.835	-0.60752	-0.23082
135.853	-0.60754	-0.23082
135.87	-0.60755	-0.23082
135.885	-0.60756	-0.23082
135.902	-0.60757	-0.23082
135.918	-0.60758	-0.23082
135.935	-0.60759	-0.23082
135.952	-0.6076	-0.23081
135.968	-0.60761	-0.23081
135.986	-0.60763	-0.23081
136.001	-0.60764	-0.2308
136.018	-0.60766	-0.2308
136.035	-0.60767	-0.2308
136.052	-0.60769	-0.2308
136.069	-0.6077	-0.2308

136.085	-0.60771	-0.2308
136.102	-0.60771	-0.2308
136.12	-0.60772	-0.2308
136.136	-0.60772	-0.2308
136.153	-0.60773	-0.2308
136.169	-0.60774	-0.2308
136.186	-0.60776	-0.2308
136.203	-0.60777	-0.2308
136.219	-0.60778	-0.2308
136.236	-0.6078	-0.23079
136.252	-0.60782	-0.23079
136.268	-0.60784	-0.23078
136.285	-0.6079	-0.23078
136.302	-0.60805	-0.23077
136.32	-0.60845	-0.23077
136.336	-0.60921	-0.23076
136.351	-0.61066	-0.23076
136.368	-0.61322	-0.23076
136.386	-0.61705	-0.23076
136.403	-0.62086	-0.23076
136.419	-0.6245	-0.23076
136.435	-0.62725	-0.23076
136.453	-0.62886	-0.23076
136.469	-0.62957	-0.23076
136.485	-0.62982	-0.23075
136.503	-0.62971	-0.23074
136.519	-0.62943	-0.23073
136.535	-0.6291	-0.23073
136.552	-0.62881	-0.23072
136.569	-0.62857	-0.23072

136.585	-0.62837	-0.23072
136.602	-0.62822	-0.23072
136.619	-0.62779	-0.23071
136.636	-0.62749	-0.23071
136.652	-0.62728	-0.23071
136.669	-0.62716	-0.2307
136.686	-0.62709	-0.2307
136.703	-0.62699	-0.23069
136.719	-0.62686	-0.23069
136.736	-0.62669	-0.23069
136.752	-0.6265	-0.23068
136.769	-0.62634	-0.23068
136.785	-0.6262	-0.23067
136.802	-0.626	-0.23067
136.82	-0.62578	-0.23067
136.836	-0.62558	-0.23067
136.852	-0.62536	-0.23067
136.869	-0.62508	-0.23067
136.885	-0.62486	-0.23066
136.903	-0.62458	-0.23066
136.919	-0.62435	-0.23066
136.935	-0.62396	-0.23065
136.951	-0.62361	-0.23065
136.969	-0.62342	-0.23064
136.985	-0.62324	-0.23063
137.002	-0.62309	-0.23063
137.019	-0.62295	-0.23062
137.037	-0.62279	-0.23061
137.052	-0.62261	-0.23061
137.069	-0.62241	-0.2306

137.086	-0.62218	-0.23059
137.103	-0.62199	-0.23059
137.119	-0.62181	-0.23059
137.136	-0.62166	-0.23058
137.152	-0.62153	-0.23058
137.169	-0.62143	-0.23058
137.185	-0.62134	-0.23057
137.202	-0.62127	-0.23057
137.218	-0.62121	-0.23057
137.235	-0.62115	-0.23057
137.252	-0.62109	-0.23057
137.268	-0.62098	-0.23057
137.286	-0.62086	-0.23057
137.302	-0.62076	-0.23057
137.319	-0.62068	-0.23056
137.335	-0.62062	-0.23056
137.352	-0.62055	-0.23055
137.369	-0.62048	-0.23054
137.386	-0.62042	-0.23054
137.402	-0.62036	-0.23053
137.419	-0.6203	-0.23053
137.435	-0.62024	-0.23052
137.452	-0.62018	-0.23052
137.469	-0.62012	-0.23051
137.486	-0.62005	-0.23051
137.502	-0.61999	-0.23051
137.518	-0.61992	-0.23051
137.536	-0.61986	-0.23051
137.552	-0.6198	-0.23051
137.569	-0.61974	-0.23051

137.586	-0.61969	-0.2305
137.602	-0.61964	-0.2305
137.62	-0.61959	-0.2305
137.636	-0.61954	-0.23049
137.653	-0.61949	-0.23049
137.669	-0.61943	-0.23048
137.686	-0.61938	-0.23047
137.703	-0.61933	-0.23046
137.719	-0.61927	-0.23046
137.737	-0.61922	-0.23045
137.752	-0.61917	-0.23045
137.77	-0.61912	-0.23045
137.786	-0.61908	-0.23045
137.804	-0.61905	-0.23045
137.82	-0.61901	-0.23045
137.837	-0.61897	-0.23045
137.853	-0.61892	-0.23045
137.87	-0.61888	-0.23045
137.887	-0.61884	-0.23045
137.903	-0.61879	-0.23045
137.92	-0.61874	-0.23044
137.937	-0.61869	-0.23044
137.954	-0.61864	-0.23044
137.97	-0.61859	-0.23044
137.987	-0.61854	-0.23043
138.003	-0.61849	-0.23043
138.021	-0.61844	-0.23042
138.037	-0.61839	-0.23042
138.054	-0.61834	-0.23042
138.071	-0.6183	-0.23042

138.087	-0.61826	-0.23041
138.104	-0.61822	-0.23041
138.121	-0.61818	-0.23041
138.137	-0.61814	-0.23041
138.154	-0.6181	-0.23041
138.17	-0.61806	-0.23041
138.187	-0.61803	-0.2304
138.204	-0.61799	-0.2304
138.221	-0.61795	-0.23039
138.237	-0.61792	-0.23038
138.253	-0.61788	-0.23037
138.27	-0.61784	-0.23037
138.286	-0.61781	-0.23036
138.303	-0.61777	-0.23035
138.321	-0.61774	-0.23034
138.337	-0.6177	-0.23034
138.354	-0.61766	-0.23034
138.371	-0.61761	-0.23034
138.388	-0.61757	-0.23034
138.405	-0.61752	-0.23034
138.421	-0.61748	-0.23034
138.438	-0.61743	-0.23033
138.454	-0.61739	-0.23032
138.472	-0.61735	-0.23032
138.488	-0.61731	-0.23031
138.504	-0.61727	-0.2303
138.521	-0.61724	-0.2303
138.537	-0.61721	-0.23029
138.554	-0.61717	-0.23028
138.571	-0.61714	-0.23027

138.588	-0.61711	-0.23026
138.604	-0.61708	-0.23026
138.621	-0.61705	-0.23025
138.638	-0.61701	-0.23025
138.655	-0.61698	-0.23024
138.671	-0.61694	-0.23023
138.688	-0.6169	-0.23023
138.705	-0.61685	-0.23022
138.721	-0.6168	-0.23022
138.737	-0.61676	-0.23021
138.754	-0.61671	-0.23021
138.771	-0.61667	-0.2302
138.787	-0.61662	-0.23019
138.804	-0.61658	-0.23018
138.821	-0.61653	-0.23018
138.837	-0.61649	-0.23017
138.854	-0.61645	-0.23016
138.871	-0.61641	-0.23015
138.887	-0.61637	-0.23014
138.904	-0.61633	-0.23013
138.921	-0.61629	-0.23013
138.938	-0.61626	-0.23012
138.954	-0.61622	-0.23011
138.97	-0.61619	-0.23011
138.987	-0.61615	-0.2301
139.005	-0.61611	-0.23009
139.021	-0.61608	-0.23008
139.038	-0.61605	-0.23007
139.054	-0.61601	-0.23006
139.071	-0.61598	-0.23005

139.088	-0.61595	-0.23004
139.104	-0.61591	-0.23004
139.121	-0.61589	-0.23003
139.137	-0.61586	-0.23002
139.154	-0.61583	-0.23002
139.171	-0.6158	-0.23001
139.187	-0.61577	-0.23001
139.204	-0.61575	-0.23
139.221	-0.61573	-0.22999
139.237	-0.6157	-0.22998
139.253	-0.61568	-0.22998
139.269	-0.61565	-0.22997
139.287	-0.61563	-0.22996
139.305	-0.6156	-0.22995
139.321	-0.61557	-0.22994
139.338	-0.61555	-0.22993
139.355	-0.61552	-0.22992
139.371	-0.6155	-0.22991
139.387	-0.61548	-0.2299
139.404	-0.61546	-0.22989
139.42	-0.61544	-0.22988
139.437	-0.61542	-0.22987
139.454	-0.6154	-0.22986
139.47	-0.61538	-0.22986
139.487	-0.61536	-0.22985
139.503	-0.61534	-0.22984
139.52	-0.61531	-0.22984
139.537	-0.61529	-0.22983
139.553	-0.61528	-0.22983
139.57	-0.61526	-0.22982

139.587	-0.61525	-0.22982
139.604	-0.61525	-0.22981
139.62	-0.61524	-0.2298
139.637	-0.61523	-0.22979
139.653	-0.61523	-0.22979
139.669	-0.61521	-0.22978
139.686	-0.61519	-0.22977
139.703	-0.61518	-0.22977
139.719	-0.61516	-0.22976
139.737	-0.61515	-0.22976
139.753	-0.61514	-0.22976
139.77	-0.61513	-0.22975
139.787	-0.61511	-0.22975
139.804	-0.61509	-0.22974
139.82	-0.61508	-0.22973
139.837	-0.61507	-0.22972
139.853	-0.61506	-0.22971
139.87	-0.61506	-0.2297
139.886	-0.61506	-0.2297
139.904	-0.61505	-0.22969
139.92	-0.61505	-0.22968
139.936	-0.61504	-0.22967
139.953	-0.61504	-0.22967
139.971	-0.61504	-0.22966
139.987	-0.61502	-0.22965
140.004	-0.61501	-0.22964
140.021	-0.61499	-0.22963
140.036	-0.61499	-0.22963
140.054	-0.61498	-0.22962
140.069	-0.61498	-0.22961

140.086	-0.61497	-0.22961
140.104	-0.61496	-0.22961
140.121	-0.61496	-0.22961
140.137	-0.61495	-0.22961
140.154	-0.61495	-0.22961
140.171	-0.61494	-0.22961
140.188	-0.61493	-0.2296
140.204	-0.61492	-0.2296
140.221	-0.61491	-0.2296
140.238	-0.6149	-0.22959
140.253	-0.61489	-0.22958
140.27	-0.61488	-0.22957
140.288	-0.61487	-0.22957
140.304	-0.61487	-0.22956
140.32	-0.61487	-0.22955
140.337	-0.61487	-0.22954
140.354	-0.61487	-0.22953
140.371	-0.61487	-0.22952
140.388	-0.61487	-0.22952
140.405	-0.61487	-0.22951
140.42	-0.61487	-0.22951
140.438	-0.61486	-0.2295
140.455	-0.61484	-0.2295
140.471	-0.61483	-0.2295
140.488	-0.61483	-0.2295
140.504	-0.61482	-0.2295
140.521	-0.61482	-0.2295
140.537	-0.61481	-0.22949
140.554	-0.61481	-0.22949
140.572	-0.61481	-0.22949

140.588	-0.61481	-0.22948
140.604	-0.61481	-0.22947
140.621	-0.61481	-0.22947
140.638	-0.6148	-0.22946
140.655	-0.61478	-0.22945
140.67	-0.61477	-0.22944
140.688	-0.61476	-0.22943
140.703	-0.61474	-0.22942
140.721	-0.61474	-0.22941
140.737	-0.61473	-0.2294
140.754	-0.61473	-0.2294
140.771	-0.61473	-0.22939
140.788	-0.61473	-0.22938
140.805	-0.61473	-0.22938
140.821	-0.61473	-0.22938
140.838	-0.61473	-0.22937
140.855	-0.61472	-0.22936
140.871	-0.61472	-0.22936
140.887	-0.61472	-0.22935
140.904	-0.61472	-0.22935
140.921	-0.61472	-0.22934
140.938	-0.61471	-0.22933
140.955	-0.61471	-0.22933
140.971	-0.6147	-0.22932
140.988	-0.61469	-0.22931
141.005	-0.61469	-0.2293
141.021	-0.61468	-0.22929
141.038	-0.61467	-0.22928
141.055	-0.61467	-0.22927
141.071	-0.61467	-0.22926

141.088	-0.61468	-0.22925
141.104	-0.61468	-0.22924
141.122	-0.61469	-0.22923
141.138	-0.61468	-0.22923
141.154	-0.61469	-0.22922
141.171	-0.61469	-0.22922
141.188	-0.61469	-0.22921
141.204	-0.61468	-0.22921
141.222	-0.61468	-0.2292
141.238	-0.61467	-0.22919
141.254	-0.61466	-0.22918
141.271	-0.61465	-0.22918
141.288	-0.61464	-0.22917
141.304	-0.61463	-0.22916
141.322	-0.61463	-0.22915
141.338	-0.61463	-0.22914
141.354	-0.61463	-0.22913
141.371	-0.61464	-0.22912
141.388	-0.61465	-0.22912
141.404	-0.61466	-0.22911
141.421	-0.61467	-0.2291
141.438	-0.61467	-0.2291
141.455	-0.61467	-0.22909
141.471	-0.61467	-0.22909
141.488	-0.61468	-0.22909
141.504	-0.61468	-0.22908
141.522	-0.61469	-0.22908
141.538	-0.6147	-0.22907
141.555	-0.61472	-0.22906
141.572	-0.61473	-0.22906

141.588	-0.61475	-0.22905
141.605	-0.61478	-0.22904
141.622	-0.61479	-0.22904
141.638	-0.61481	-0.22903
141.654	-0.61482	-0.22902
141.671	-0.61483	-0.22902
141.688	-0.61484	-0.22901
141.705	-0.61485	-0.229
141.721	-0.61486	-0.22899
141.737	-0.61487	-0.22899
141.755	-0.61489	-0.22898
141.771	-0.61489	-0.22897
141.788	-0.61491	-0.22897
141.804	-0.61492	-0.22897
141.822	-0.61493	-0.22896
141.839	-0.61494	-0.22896
141.855	-0.61496	-0.22895
141.872	-0.61497	-0.22894
141.889	-0.61498	-0.22893
141.905	-0.615	-0.22892
141.921	-0.61501	-0.22891
141.938	-0.61502	-0.2289
141.955	-0.61503	-0.22889
141.971	-0.61504	-0.22888
141.988	-0.61505	-0.22887
142.004	-0.61506	-0.22887
142.022	-0.61506	-0.22886
142.038	-0.61507	-0.22886
142.055	-0.61508	-0.22886
142.072	-0.61508	-0.22885

142.089	-0.61508	-0.22885
142.105	-0.61509	-0.22884
142.122	-0.61509	-0.22884
142.139	-0.61509	-0.22883
142.155	-0.61509	-0.22882
142.171	-0.61509	-0.22881
142.189	-0.6151	-0.22881
142.206	-0.6151	-0.2288
142.222	-0.6151	-0.2288
142.239	-0.61511	-0.22879
142.255	-0.61511	-0.22879
142.272	-0.61511	-0.22879
142.289	-0.61511	-0.22878
142.306	-0.61511	-0.22877
142.323	-0.61511	-0.22876
142.339	-0.61511	-0.22875
142.355	-0.61512	-0.22874
142.371	-0.61513	-0.22873
142.389	-0.61514	-0.22872
142.405	-0.61515	-0.22871
142.423	-0.61516	-0.2287
142.438	-0.61518	-0.22869
142.455	-0.61518	-0.22868
142.471	-0.61519	-0.22868
142.489	-0.6152	-0.22867
142.506	-0.6152	-0.22867
142.522	-0.61521	-0.22866
142.538	-0.61521	-0.22866
142.556	-0.61522	-0.22865
142.572	-0.61522	-0.22864

142.589	-0.61523	-0.22863
142.605	-0.61523	-0.22863
142.622	-0.61523	-0.22862
142.64	-0.61523	-0.22861
142.656	-0.61523	-0.22861
142.672	-0.61523	-0.2286
142.689	-0.61523	-0.2286
142.705	-0.61524	-0.22859
142.722	-0.61524	-0.22858
142.74	-0.61525	-0.22858
142.756	-0.61526	-0.22857
142.773	-0.61526	-0.22856
142.789	-0.61526	-0.22855
142.806	-0.61527	-0.22854
142.822	-0.61527	-0.22853
142.839	-0.61528	-0.22852
142.855	-0.61529	-0.22852
142.872	-0.6153	-0.22851
142.889	-0.61531	-0.2285
142.904	-0.61532	-0.22849
142.922	-0.61533	-0.22849
142.939	-0.61535	-0.22848
142.956	-0.61537	-0.22847
142.972	-0.61538	-0.22846
142.989	-0.61539	-0.22844
143.006	-0.6154	-0.22843
143.022	-0.6154	-0.22842
143.039	-0.61541	-0.22842
143.055	-0.61542	-0.22841
143.072	-0.61543	-0.2284

143.09	-0.61544	-0.2284
143.106	-0.61545	-0.22839
143.122	-0.61545	-0.22839
143.139	-0.61546	-0.22839
143.155	-0.61547	-0.22838
143.173	-0.61547	-0.22837
143.189	-0.61548	-0.22837
143.205	-0.61549	-0.22836
143.222	-0.61549	-0.22835
143.239	-0.6155	-0.22834
143.256	-0.6155	-0.22833
143.271	-0.6155	-0.22832
143.289	-0.61551	-0.22831
143.305	-0.61552	-0.2283
143.323	-0.61553	-0.2283
143.338	-0.61554	-0.22829
143.356	-0.61554	-0.22828
143.372	-0.61555	-0.22828
143.389	-0.61556	-0.22828
143.406	-0.61558	-0.22827
143.422	-0.61559	-0.22827
143.438	-0.6156	-0.22826
143.456	-0.61561	-0.22825
143.473	-0.61563	-0.22824
143.49	-0.61564	-0.22823
143.506	-0.61566	-0.22823
143.522	-0.61568	-0.22822
143.539	-0.61569	-0.22822
143.556	-0.61571	-0.22821
143.573	-0.61572	-0.22821

143.589	-0.61573	-0.2282
143.606	-0.61575	-0.2282
143.623	-0.61576	-0.22819
143.639	-0.61577	-0.22818
143.656	-0.61578	-0.22817
143.674	-0.61578	-0.22816
143.69	-0.61579	-0.22816
143.707	-0.61579	-0.22815
143.723	-0.6158	-0.22814
143.739	-0.6158	-0.22813
143.756	-0.61581	-0.22813
143.773	-0.61582	-0.22812
143.79	-0.61582	-0.22811
143.806	-0.61583	-0.22811
143.823	-0.61583	-0.22811
143.84	-0.61584	-0.2281
143.857	-0.61584	-0.2281
143.873	-0.61585	-0.2281
143.891	-0.61586	-0.2281
143.906	-0.61587	-0.22809
143.923	-0.61588	-0.22809
143.94	-0.61589	-0.22808
143.956	-0.61589	-0.22807
143.973	-0.6159	-0.22806
143.989	-0.61592	-0.22806
144.006	-0.61592	-0.22805
144.023	-0.61593	-0.22804
144.039	-0.61594	-0.22804
144.057	-0.61595	-0.22803
144.073	-0.61596	-0.22803

144.089	-0.61596	-0.22802
144.106	-0.61597	-0.22802
144.123	-0.61597	-0.22801
144.14	-0.61598	-0.228
144.156	-0.61598	-0.22799
144.172	-0.61599	-0.22798
144.189	-0.61599	-0.22797
144.207	-0.61599	-0.22796
144.223	-0.61599	-0.22795
144.24	-0.616	-0.22795
144.257	-0.616	-0.22794
144.274	-0.61601	-0.22794
144.29	-0.61601	-0.22793
144.306	-0.61601	-0.22793
144.324	-0.61602	-0.22792
144.34	-0.61602	-0.22792
144.356	-0.61603	-0.22791
144.373	-0.61604	-0.2279
144.39	-0.61604	-0.2279
144.406	-0.61605	-0.22789
144.423	-0.61606	-0.22788
144.44	-0.61606	-0.22787
144.456	-0.61606	-0.22786
144.473	-0.61607	-0.22785
144.49	-0.61607	-0.22784
144.506	-0.61608	-0.22783
144.523	-0.61608	-0.22782
144.54	-0.61609	-0.22781
144.555	-0.6161	-0.2278
144.572	-0.61611	-0.2278

144.589	-0.61611	-0.22779
144.607	-0.61611	-0.22778
144.622	-0.61612	-0.22777
144.639	-0.61612	-0.22776
144.656	-0.61613	-0.22776
144.673	-0.61613	-0.22775
144.69	-0.61613	-0.22775
144.706	-0.61614	-0.22774
144.723	-0.61615	-0.22773
144.741	-0.61616	-0.22772
144.757	-0.61617	-0.22772
144.773	-0.61618	-0.22771
144.79	-0.6162	-0.22769
144.806	-0.61621	-0.22768
144.823	-0.61623	-0.22767
144.84	-0.61624	-0.22765
144.856	-0.61625	-0.22764
144.873	-0.61626	-0.22763
144.889	-0.61626	-0.22762
144.907	-0.61626	-0.22761
144.923	-0.61626	-0.2276
144.939	-0.61627	-0.22759
144.956	-0.61627	-0.22758
144.973	-0.61628	-0.22757
144.989	-0.61629	-0.22756
145.006	-0.6163	-0.22755
145.023	-0.61631	-0.22755
145.04	-0.61632	-0.22754
145.057	-0.61633	-0.22753
145.074	-0.61634	-0.22752

145.088	-0.61635	-0.22751
145.106	-0.61637	-0.2275
145.123	-0.61638	-0.2275
145.14	-0.6164	-0.22749
145.156	-0.61641	-0.22748
145.173	-0.61642	-0.22748
145.189	-0.61643	-0.22748
145.207	-0.61644	-0.22747
145.223	-0.61645	-0.22747
145.24	-0.61646	-0.22746
145.257	-0.61647	-0.22746
145.273	-0.61648	-0.22745
145.29	-0.6165	-0.22744
145.306	-0.61652	-0.22743
145.323	-0.61654	-0.22742
145.341	-0.61655	-0.22741
145.357	-0.61657	-0.2274
145.373	-0.61658	-0.22739
145.39	-0.61659	-0.22738
145.406	-0.6166	-0.22738
145.423	-0.61661	-0.22737
145.44	-0.61662	-0.22737
145.456	-0.61663	-0.22736
145.473	-0.61666	-0.22736
145.49	-0.61667	-0.22736
145.506	-0.61669	-0.22735
145.524	-0.6167	-0.22734
145.541	-0.61672	-0.22734
145.557	-0.61673	-0.22733
145.574	-0.61674	-0.22732

145.591	-0.61676	-0.22731
145.607	-0.61677	-0.2273
145.623	-0.61677	-0.2273
145.639	-0.61678	-0.22729
145.656	-0.61679	-0.22729
145.674	-0.61679	-0.22728
145.689	-0.61681	-0.22728
145.707	-0.61681	-0.22727
145.722	-0.61682	-0.22727
145.74	-0.61683	-0.22726
145.757	-0.61684	-0.22726
145.774	-0.61685	-0.22725
145.79	-0.61687	-0.22724
145.807	-0.61688	-0.22724
145.824	-0.61689	-0.22723
145.84	-0.61691	-0.22722
145.858	-0.61692	-0.22721
145.874	-0.61693	-0.22721
145.89	-0.61694	-0.2272
145.907	-0.61695	-0.22719
145.923	-0.61696	-0.22719
145.94	-0.61698	-0.22718
145.957	-0.61699	-0.22717
145.973	-0.617	-0.22716
145.99	-0.61702	-0.22716
146.007	-0.61704	-0.22715
146.023	-0.61705	-0.22714
146.04	-0.61707	-0.22714
146.057	-0.61708	-0.22713
146.074	-0.61709	-0.22713

146.091	-0.61711	-0.22712
146.108	-0.61712	-0.22711
146.123	-0.61713	-0.22711
146.14	-0.61714	-0.2271
146.157	-0.61715	-0.22709
146.174	-0.61717	-0.22709
146.19	-0.61718	-0.22708
146.207	-0.6172	-0.22708
146.224	-0.61722	-0.22707
146.241	-0.61724	-0.22706
146.257	-0.61726	-0.22705
146.274	-0.61728	-0.22704
146.29	-0.61729	-0.22704
146.307	-0.6173	-0.22703
146.324	-0.6173	-0.22702
146.341	-0.61731	-0.22701
146.356	-0.61732	-0.22701
146.374	-0.61733	-0.227
146.391	-0.61735	-0.22699
146.407	-0.61736	-0.22699
146.423	-0.61738	-0.22698
146.44	-0.61739	-0.22697
146.457	-0.6174	-0.22697
146.474	-0.61742	-0.22696
146.49	-0.61743	-0.22696
146.507	-0.61745	-0.22696
146.525	-0.61746	-0.22695
146.54	-0.61748	-0.22695
146.558	-0.6175	-0.22694
146.574	-0.61751	-0.22694

146.59	-0.61753	-0.22693
146.608	-0.61755	-0.22692
146.624	-0.61757	-0.22692
146.64	-0.61758	-0.22691
146.657	-0.6176	-0.2269
146.674	-0.61761	-0.2269
146.69	-0.61763	-0.22689
146.708	-0.61765	-0.22688
146.724	-0.61767	-0.22687
146.741	-0.6177	-0.22686
146.758	-0.61772	-0.22685
146.774	-0.61774	-0.22685
146.791	-0.61776	-0.22684
146.808	-0.61777	-0.22683
146.824	-0.61778	-0.22683
146.841	-0.61779	-0.22682
146.858	-0.6178	-0.22682
146.875	-0.61781	-0.22682
146.891	-0.61782	-0.22682
146.909	-0.61784	-0.22682
146.924	-0.61785	-0.22682
146.941	-0.61787	-0.22681
146.958	-0.6179	-0.2268
146.974	-0.61792	-0.2268
146.992	-0.61794	-0.22679
147.007	-0.61796	-0.22678
147.024	-0.61799	-0.22677
147.041	-0.61801	-0.22677
147.058	-0.61802	-0.22677
147.074	-0.61803	-0.22677

147.091	-0.61804	-0.22676
147.108	-0.61806	-0.22676
147.124	-0.61807	-0.22675
147.142	-0.61809	-0.22675
147.158	-0.6181	-0.22674
147.176	-0.61812	-0.22674
147.192	-0.61814	-0.22673
147.209	-0.61816	-0.22672
147.225	-0.61817	-0.22671
147.242	-0.61819	-0.2267
147.258	-0.6182	-0.22669
147.275	-0.61822	-0.22669
147.292	-0.61823	-0.22668
147.308	-0.61825	-0.22668
147.325	-0.61826	-0.22667
147.342	-0.61828	-0.22667
147.359	-0.61829	-0.22666
147.374	-0.61831	-0.22666
147.392	-0.61832	-0.22665
147.408	-0.61834	-0.22664
147.425	-0.61836	-0.22664
147.443	-0.61838	-0.22663
147.459	-0.61839	-0.22663
147.476	-0.6184	-0.22662
147.492	-0.61842	-0.22662
147.509	-0.61844	-0.22661
147.526	-0.61845	-0.2266
147.542	-0.61847	-0.2266
147.559	-0.61849	-0.22659
147.575	-0.6185	-0.22658

147.592	-0.61852	-0.22656
147.608	-0.61853	-0.22656
147.625	-0.61853	-0.22655
147.642	-0.61855	-0.22654
147.658	-0.61856	-0.22654
147.675	-0.61857	-0.22654
147.691	-0.61858	-0.22653
147.708	-0.61859	-0.22652
147.725	-0.6186	-0.22652
147.742	-0.61862	-0.22651
147.758	-0.61863	-0.2265
147.775	-0.61864	-0.22649
147.792	-0.61865	-0.22649
147.809	-0.61867	-0.22648
147.825	-0.61867	-0.22647
147.842	-0.61869	-0.22647
147.859	-0.6187	-0.22646
147.875	-0.61871	-0.22646
147.893	-0.61872	-0.22645
147.909	-0.61873	-0.22645
147.925	-0.61875	-0.22644
147.941	-0.61876	-0.22643
147.959	-0.61877	-0.22643
147.975	-0.61879	-0.22642
147.992	-0.6188	-0.22641
148.008	-0.61882	-0.2264
148.025	-0.61883	-0.22639
148.042	-0.61885	-0.22638
148.058	-0.61887	-0.22637
148.075	-0.61888	-0.22636

148.09	-0.6189	-0.22635
148.108	-0.61891	-0.22634
148.126	-0.61892	-0.22634
148.141	-0.61893	-0.22633
148.158	-0.61894	-0.22632
148.174	-0.61895	-0.22631
148.192	-0.61895	-0.2263
148.208	-0.61896	-0.22629
148.225	-0.61897	-0.22629
148.241	-0.61898	-0.22628
148.259	-0.61899	-0.22627
148.275	-0.619	-0.22627
148.292	-0.61901	-0.22626
148.308	-0.61903	-0.22625
148.325	-0.61903	-0.22625
148.341	-0.61904	-0.22624
148.358	-0.61905	-0.22623
148.375	-0.61906	-0.22622
148.391	-0.61907	-0.22622
148.408	-0.61908	-0.22621
148.424	-0.61909	-0.2262
148.441	-0.6191	-0.22619
148.458	-0.61911	-0.22618
148.474	-0.61913	-0.22618
148.49	-0.61914	-0.22617
148.507	-0.61916	-0.22617
148.524	-0.61917	-0.22617
148.541	-0.61918	-0.22617
148.558	-0.61919	-0.22616
148.574	-0.6192	-0.22616

148.591	-0.61921	-0.22616
148.608	-0.61922	-0.22615
148.625	-0.61923	-0.22614
148.641	-0.61924	-0.22614
148.657	-0.61925	-0.22613
148.674	-0.61926	-0.22612
148.691	-0.61928	-0.22612
148.707	-0.61929	-0.22611
148.725	-0.6193	-0.2261
148.741	-0.61931	-0.22609
148.758	-0.61932	-0.22608
148.774	-0.61933	-0.22607
148.79	-0.61934	-0.22607
148.808	-0.61935	-0.22606
148.825	-0.61936	-0.22605
148.841	-0.61937	-0.22605
148.858	-0.61938	-0.22605
148.874	-0.6194	-0.22604
148.892	-0.61941	-0.22604
148.908	-0.61943	-0.22604
148.925	-0.61945	-0.22603
148.941	-0.61946	-0.22603
148.958	-0.61948	-0.22602
148.974	-0.61949	-0.22601
148.991	-0.61951	-0.226
149.008	-0.61952	-0.226
149.025	-0.61954	-0.22599
149.042	-0.61955	-0.22599
149.058	-0.61957	-0.22599
149.074	-0.61959	-0.22598

149.091	-0.6196	-0.22598
149.108	-0.61962	-0.22598
149.125	-0.61964	-0.22598
149.142	-0.61967	-0.22598
149.158	-0.61969	-0.22598
149.175	-0.61971	-0.22597
149.191	-0.61973	-0.22597
149.209	-0.61975	-0.22597
149.225	-0.61976	-0.22596
149.242	-0.61978	-0.22596
149.258	-0.61979	-0.22595
149.274	-0.61981	-0.22595
149.291	-0.61983	-0.22594
149.308	-0.61985	-0.22594
149.324	-0.61986	-0.22593
149.341	-0.61988	-0.22592
149.358	-0.61991	-0.22592
149.374	-0.61994	-0.22591
149.391	-0.61996	-0.22591
149.408	-0.61998	-0.2259
149.425	-0.62001	-0.22589
149.441	-0.62003	-0.22589
149.458	-0.62005	-0.22589
149.475	-0.62007	-0.22589
149.491	-0.62009	-0.22589
149.509	-0.6201	-0.22589
149.525	-0.62011	-0.22589
149.541	-0.62012	-0.22589
149.558	-0.62014	-0.22589
149.575	-0.62016	-0.22588

149.592	-0.62018	-0.22588
149.609	-0.6202	-0.22587
149.626	-0.62022	-0.22587
149.642	-0.62025	-0.22586
149.658	-0.62027	-0.22586
149.676	-0.6203	-0.22585
149.693	-0.62033	-0.22584
149.709	-0.62035	-0.22584
149.726	-0.62037	-0.22584
149.742	-0.6204	-0.22583
149.759	-0.62042	-0.22583
149.775	-0.62044	-0.22582
149.792	-0.62046	-0.22581
149.809	-0.62048	-0.22581
149.826	-0.6205	-0.2258
149.842	-0.62052	-0.2258
149.858	-0.62055	-0.22579
149.875	-0.62058	-0.22579
149.891	-0.6206	-0.22578
149.908	-0.62063	-0.22578
149.925	-0.62066	-0.22577
149.941	-0.62068	-0.22577
149.958	-0.6207	-0.22577
149.976	-0.62072	-0.22577
149.992	-0.62073	-0.22577
150.009	-0.62074	-0.22577
150.026	-0.62076	-0.22576
150.042	-0.62077	-0.22576
150.058	-0.62079	-0.22575
150.075	-0.62081	-0.22574

150.092	-0.62082	-0.22574
150.109	-0.62084	-0.22573
150.124	-0.62086	-0.22572
150.142	-0.62088	-0.22571
150.159	-0.62089	-0.22571
150.175	-0.62091	-0.2257
150.193	-0.62093	-0.2257
150.209	-0.62095	-0.22569
150.225	-0.62096	-0.22569
150.242	-0.62098	-0.22569
150.259	-0.62099	-0.22569
150.275	-0.62101	-0.22569
150.293	-0.62102	-0.22568
150.309	-0.62104	-0.22568
150.325	-0.62105	-0.22568
150.342	-0.62106	-0.22567
150.359	-0.62108	-0.22567
150.375	-0.6211	-0.22566
150.392	-0.62111	-0.22566
150.408	-0.62113	-0.22565
150.425	-0.62114	-0.22565
150.442	-0.62115	-0.22564
150.458	-0.62117	-0.22564
150.476	-0.62118	-0.22563
150.492	-0.6212	-0.22563
150.508	-0.62121	-0.22563
150.525	-0.62123	-0.22562
150.541	-0.62125	-0.22562
150.559	-0.62127	-0.22561
150.576	-0.62129	-0.22561

150.593	-0.62131	-0.22561
150.609	-0.62133	-0.2256
150.625	-0.62135	-0.2256
150.642	-0.62136	-0.22559
150.659	-0.62137	-0.22559
150.675	-0.62138	-0.22558
150.691	-0.6214	-0.22558
150.709	-0.62142	-0.22557
150.724	-0.62143	-0.22557
150.741	-0.62144	-0.22556
150.759	-0.62145	-0.22556
150.776	-0.62147	-0.22555
150.792	-0.62148	-0.22555
150.809	-0.6215	-0.22554
150.825	-0.62152	-0.22554
150.842	-0.62153	-0.22553
150.857	-0.62154	-0.22553
150.875	-0.62155	-0.22553
150.892	-0.62157	-0.22552
150.908	-0.62159	-0.22552
150.924	-0.6216	-0.22551
150.941	-0.62162	-0.22551
150.958	-0.62164	-0.2255
150.975	-0.62167	-0.2255
150.991	-0.62168	-0.22549
151.008	-0.6217	-0.22549
151.024	-0.62171	-0.22548
151.042	-0.62172	-0.22548
151.058	-0.62173	-0.22548
151.074	-0.62174	-0.22547

151.092	-0.62175	-0.22547
151.108	-0.62176	-0.22546
151.125	-0.62177	-0.22545
151.141	-0.62178	-0.22545
151.158	-0.6218	-0.22544
151.175	-0.62181	-0.22543
151.191	-0.62183	-0.22543
151.208	-0.62184	-0.22543
151.225	-0.62186	-0.22543
151.241	-0.62188	-0.22543
151.259	-0.6219	-0.22543
151.275	-0.62192	-0.22542
151.291	-0.62194	-0.22542
151.308	-0.62197	-0.22542
151.325	-0.62199	-0.22541
151.342	-0.62201	-0.22541
151.359	-0.62203	-0.2254
151.375	-0.62205	-0.2254
151.391	-0.62207	-0.22539
151.408	-0.62208	-0.22539
151.425	-0.6221	-0.22539
151.442	-0.62211	-0.22539
151.458	-0.62213	-0.22539
151.475	-0.62215	-0.22539
151.492	-0.62217	-0.22539
151.508	-0.62219	-0.22538
151.525	-0.62221	-0.22537
151.542	-0.62223	-0.22537
151.558	-0.62224	-0.22536
151.575	-0.62226	-0.22536

151.591	-0.62229	-0.22535
151.608	-0.6223	-0.22535
151.625	-0.62232	-0.22534
151.642	-0.62233	-0.22534
151.658	-0.62234	-0.22534
151.675	-0.62235	-0.22534
151.691	-0.62237	-0.22534
151.708	-0.62239	-0.22533
151.725	-0.6224	-0.22533
151.742	-0.62242	-0.22533
151.758	-0.62245	-0.22533
151.775	-0.62248	-0.22533
151.792	-0.6225	-0.22533
151.809	-0.62253	-0.22533
151.825	-0.62255	-0.22533
151.842	-0.62256	-0.22533
151.859	-0.62258	-0.22533
151.876	-0.62259	-0.22532
151.892	-0.62261	-0.22531
151.909	-0.62262	-0.22531
151.925	-0.62264	-0.2253
151.942	-0.62265	-0.2253
151.958	-0.62268	-0.2253
151.974	-0.62271	-0.22529
151.992	-0.62274	-0.22529
152.008	-0.62276	-0.22529
152.024	-0.62278	-0.22529
152.041	-0.62279	-0.22528
152.058	-0.62281	-0.22528
152.075	-0.62282	-0.22528

152.092	-0.62284	-0.22527
152.109	-0.62286	-0.22527
152.125	-0.62287	-0.22527
152.14	-0.62288	-0.22527
152.158	-0.6229	-0.22527
152.175	-0.62292	-0.22527
152.192	-0.62294	-0.22526
152.208	-0.62296	-0.22526
152.225	-0.62298	-0.22525
152.242	-0.62301	-0.22524
152.259	-0.62303	-0.22524
152.275	-0.62306	-0.22523
152.293	-0.62308	-0.22523
152.309	-0.62309	-0.22523
152.325	-0.62311	-0.22523
152.342	-0.62313	-0.22523
152.359	-0.62315	-0.22523
152.375	-0.62317	-0.22523
152.391	-0.62318	-0.22523
152.409	-0.62319	-0.22523
152.425	-0.62321	-0.22523
152.443	-0.62323	-0.22522
152.458	-0.62324	-0.22522
152.475	-0.62326	-0.22521
152.491	-0.62327	-0.22521
152.509	-0.62328	-0.22521
152.525	-0.62329	-0.22521
152.542	-0.62331	-0.22521
152.558	-0.62332	-0.2252
152.575	-0.62333	-0.2252

152.592	-0.62335	-0.2252
152.608	-0.62336	-0.2252
152.624	-0.62337	-0.22519
152.641	-0.62338	-0.22519
152.658	-0.6234	-0.22518
152.675	-0.62343	-0.22518
152.692	-0.62345	-0.22517
152.708	-0.62347	-0.22517
152.725	-0.62349	-0.22517
152.742	-0.62351	-0.22517
152.758	-0.62353	-0.22517
152.775	-0.62355	-0.22517
152.792	-0.62357	-0.22517
152.809	-0.62358	-0.22517
152.825	-0.62359	-0.22516
152.841	-0.6236	-0.22516
152.858	-0.62362	-0.22516
152.875	-0.62364	-0.22515
152.891	-0.62365	-0.22515
152.908	-0.62367	-0.22515
152.925	-0.62369	-0.22514
152.942	-0.62371	-0.22514
152.959	-0.62373	-0.22514
152.974	-0.62375	-0.22514
152.992	-0.62376	-0.22514
153.008	-0.62377	-0.22514
153.025	-0.62379	-0.22514
153.042	-0.6238	-0.22514
153.059	-0.62381	-0.22515
153.076	-0.62381	-0.22514

153.093	-0.62382	-0.22514
153.109	-0.62383	-0.22514
153.125	-0.62384	-0.22513
153.143	-0.62386	-0.22513
153.159	-0.62387	-0.22512
153.176	-0.62388	-0.22512
153.192	-0.6239	-0.22511
153.209	-0.62392	-0.22511
153.225	-0.62393	-0.22511
153.241	-0.62395	-0.22511
153.258	-0.62396	-0.22511
153.276	-0.62398	-0.2251
153.292	-0.624	-0.2251
153.309	-0.62402	-0.2251
153.326	-0.62404	-0.2251
153.341	-0.62406	-0.2251
153.359	-0.62409	-0.22509
153.375	-0.62411	-0.22509
153.391	-0.62414	-0.22508
153.408	-0.62415	-0.22508
153.425	-0.62416	-0.22508
153.442	-0.62417	-0.22508
153.458	-0.62418	-0.22508
153.475	-0.62419	-0.22508
153.492	-0.6242	-0.22509
153.509	-0.6242	-0.22509
153.526	-0.62422	-0.22509
153.543	-0.62423	-0.22509
153.559	-0.62425	-0.22509
153.576	-0.62428	-0.22508

153.592	-0.6243	-0.22508
153.609	-0.62431	-0.22508
153.626	-0.62432	-0.22508
153.642	-0.62434	-0.22508
153.658	-0.62435	-0.22508
153.675	-0.62437	-0.22508
153.692	-0.62438	-0.22508
153.709	-0.62439	-0.22508
153.726	-0.62441	-0.22509
153.742	-0.62442	-0.22509
153.759	-0.62444	-0.22509
153.776	-0.62446	-0.22509
153.793	-0.62447	-0.22509
153.809	-0.62448	-0.22509
153.826	-0.6245	-0.22509
153.842	-0.62451	-0.22509
153.859	-0.62452	-0.22508
153.876	-0.62453	-0.22508
153.893	-0.62454	-0.22508
153.91	-0.62456	-0.22508
153.926	-0.62458	-0.22508
153.943	-0.6246	-0.22508
153.96	-0.62462	-0.22508
153.976	-0.62464	-0.22508
153.992	-0.62465	-0.22508
154.009	-0.62467	-0.22508
154.026	-0.62469	-0.22507
154.042	-0.62471	-0.22507
154.06	-0.62473	-0.22507
154.076	-0.62475	-0.22507

154.093	-0.62477	-0.22507
154.109	-0.62479	-0.22507
154.127	-0.62481	-0.22508
154.142	-0.62482	-0.22508
154.16	-0.62484	-0.22508
154.177	-0.62485	-0.22508
154.193	-0.62486	-0.22508
154.211	-0.62487	-0.22508
154.227	-0.62489	-0.22507
154.243	-0.6249	-0.22507
154.26	-0.62492	-0.22506
154.277	-0.62493	-0.22506
154.294	-0.62495	-0.22506
154.31	-0.62496	-0.22506
154.326	-0.62498	-0.22506
154.343	-0.62499	-0.22506
154.361	-0.62501	-0.22507
154.377	-0.62503	-0.22507
154.394	-0.62505	-0.22507
154.411	-0.62507	-0.22507
154.427	-0.62509	-0.22507
154.444	-0.62511	-0.22507
154.46	-0.62514	-0.22507
154.478	-0.62516	-0.22507
154.493	-0.62519	-0.22507
154.51	-0.62521	-0.22507
154.527	-0.62523	-0.22506
154.544	-0.62524	-0.22506
154.561	-0.62526	-0.22506
154.577	-0.62527	-0.22506

154.593	-0.62529	-0.22507
154.61	-0.6253	-0.22507
154.627	-0.62532	-0.22507
154.644	-0.62533	-0.22507
154.66	-0.62534	-0.22508
154.677	-0.62536	-0.22508
154.694	-0.62538	-0.22508
154.71	-0.6254	-0.22508
154.728	-0.62542	-0.22507
154.744	-0.62544	-0.22507
154.76	-0.62546	-0.22507
154.777	-0.62548	-0.22507
154.793	-0.62551	-0.22506
154.811	-0.62553	-0.22506
154.827	-0.62555	-0.22505
154.844	-0.62557	-0.22505
154.86	-0.62559	-0.22504
154.876	-0.62561	-0.22504
154.894	-0.62562	-0.22504
154.911	-0.62563	-0.22504
154.927	-0.62565	-0.22504
154.944	-0.62566	-0.22504
154.96	-0.62567	-0.22504
154.977	-0.62569	-0.22504
154.993	-0.6257	-0.22504
155.01	-0.62572	-0.22503
155.027	-0.62574	-0.22503
155.045	-0.62575	-0.22503
155.06	-0.62577	-0.22502
155.078	-0.62579	-0.22502

155.094	-0.62581	-0.22501
155.11	-0.62583	-0.22501
155.127	-0.62585	-0.22501
155.144	-0.62587	-0.225
155.16	-0.62589	-0.225
155.177	-0.62591	-0.225
155.194	-0.62593	-0.225
155.211	-0.62594	-0.225
155.228	-0.62596	-0.225
155.244	-0.62598	-0.225
155.26	-0.62599	-0.225
155.277	-0.62601	-0.22499
155.294	-0.62602	-0.22499
155.311	-0.62603	-0.22498
155.328	-0.62604	-0.22498
155.344	-0.62605	-0.22497
155.36	-0.62606	-0.22497
155.376	-0.62608	-0.22496
155.393	-0.62609	-0.22496
155.41	-0.6261	-0.22495
155.427	-0.62611	-0.22495
155.444	-0.62613	-0.22494
155.46	-0.62614	-0.22494
155.476	-0.62615	-0.22494
155.493	-0.62616	-0.22494
155.51	-0.62617	-0.22494
155.527	-0.62619	-0.22494
155.543	-0.62621	-0.22494
155.56	-0.62622	-0.22494
155.576	-0.62624	-0.22494

155.594	-0.62626	-0.22494
155.61	-0.62628	-0.22494
155.627	-0.62629	-0.22494
155.643	-0.62631	-0.22493
155.66	-0.62632	-0.22493
155.676	-0.62633	-0.22492
155.694	-0.62634	-0.22492
155.71	-0.62635	-0.22491
155.727	-0.62636	-0.22491
155.744	-0.62638	-0.2249
155.761	-0.62639	-0.2249
155.777	-0.6264	-0.2249
155.794	-0.62641	-0.2249
155.81	-0.62642	-0.2249
155.827	-0.62644	-0.2249
155.843	-0.62645	-0.22489
155.861	-0.62646	-0.22489
155.878	-0.62648	-0.22489
155.894	-0.62649	-0.22489
155.911	-0.62651	-0.22489
155.928	-0.62653	-0.22488
155.944	-0.62654	-0.22488
155.96	-0.62656	-0.22488
155.976	-0.62657	-0.22488
155.994	-0.62658	-0.22488
156.01	-0.6266	-0.22488
156.028	-0.62662	-0.22488
156.044	-0.62664	-0.22488
156.06	-0.62666	-0.22488
156.076	-0.62668	-0.22488

156.093	-0.6267	-0.22488
156.11	-0.62672	-0.22488
156.127	-0.62674	-0.22488
156.144	-0.62676	-0.22488
156.161	-0.62677	-0.22489
156.177	-0.62678	-0.22489
156.194	-0.6268	-0.22489
156.21	-0.62682	-0.22489
156.228	-0.62684	-0.2249
156.244	-0.62687	-0.2249
156.261	-0.62689	-0.22489
156.277	-0.62691	-0.22489
156.295	-0.62694	-0.22488
156.31	-0.62697	-0.22488
156.327	-0.62699	-0.22487
156.344	-0.62701	-0.22487
156.36	-0.62703	-0.22487
156.378	-0.62704	-0.22487
156.393	-0.62706	-0.22487
156.411	-0.62708	-0.22487
156.428	-0.62709	-0.22487
156.444	-0.62711	-0.22488
156.46	-0.62713	-0.22488
156.478	-0.62715	-0.22488
156.495	-0.62717	-0.22487
156.511	-0.62719	-0.22487
156.527	-0.62721	-0.22487
156.544	-0.62723	-0.22486
156.561	-0.62725	-0.22486
156.578	-0.62727	-0.22486

156.595	-0.6273	-0.22485
156.611	-0.62733	-0.22485
156.629	-0.62736	-0.22485
156.644	-0.62738	-0.22485
156.661	-0.6274	-0.22485
156.678	-0.62742	-0.22485
156.694	-0.62744	-0.22485
156.711	-0.62745	-0.22485
156.729	-0.62747	-0.22486
156.744	-0.62749	-0.22486
156.761	-0.62751	-0.22486
156.777	-0.62752	-0.22486
156.795	-0.62754	-0.22486
156.811	-0.62756	-0.22485
156.828	-0.62757	-0.22485
156.844	-0.62758	-0.22485
156.862	-0.6276	-0.22485
156.878	-0.62762	-0.22485
156.894	-0.62763	-0.22485
156.911	-0.62765	-0.22485
156.928	-0.62767	-0.22485
156.945	-0.62768	-0.22484
156.961	-0.6277	-0.22484
156.977	-0.62772	-0.22483
156.995	-0.62774	-0.22483
157.011	-0.62776	-0.22482
157.028	-0.62778	-0.22482
157.045	-0.6278	-0.22482
157.061	-0.62782	-0.22482
157.078	-0.62784	-0.22482

157.095	-0.62786	-0.22482
157.111	-0.62788	-0.22482
157.128	-0.62789	-0.22482
157.146	-0.6279	-0.22482
157.162	-0.62791	-0.22482
157.178	-0.62793	-0.22482
157.195	-0.62794	-0.22482
157.212	-0.62795	-0.22481
157.228	-0.62797	-0.22481
157.245	-0.62798	-0.22481
157.262	-0.628	-0.22481
157.278	-0.62803	-0.2248
157.294	-0.62805	-0.2248
157.312	-0.62806	-0.2248
157.328	-0.62808	-0.2248
157.346	-0.6281	-0.2248
157.361	-0.62812	-0.2248
157.378	-0.62814	-0.2248
157.395	-0.62816	-0.22479
157.411	-0.62818	-0.22479
157.428	-0.62819	-0.22479
157.444	-0.62821	-0.22478
157.461	-0.62823	-0.22478
157.478	-0.62825	-0.22478
157.495	-0.62826	-0.22478
157.511	-0.62827	-0.22478
157.527	-0.62828	-0.22478
157.545	-0.62828	-0.22478
157.561	-0.62829	-0.22479
157.577	-0.62831	-0.22479

157.594	-0.62832	-0.22479
157.611	-0.62834	-0.22479
157.627	-0.62836	-0.22479
157.644	-0.62838	-0.22479
157.662	-0.6284	-0.22479
157.678	-0.62841	-0.22479
157.695	-0.62843	-0.22478
157.712	-0.62845	-0.22478
157.728	-0.62846	-0.22478
157.745	-0.62847	-0.22477
157.762	-0.62849	-0.22476
157.778	-0.6285	-0.22476
157.795	-0.62852	-0.22475
157.812	-0.62853	-0.22475
157.827	-0.62855	-0.22475
157.844	-0.62857	-0.22474
157.861	-0.62859	-0.22474
157.878	-0.62861	-0.22474
157.895	-0.62863	-0.22474
157.911	-0.62865	-0.22474
157.928	-0.62866	-0.22473
157.944	-0.62868	-0.22473
157.961	-0.6287	-0.22473
157.978	-0.62871	-0.22473
157.994	-0.62873	-0.22473
158.01	-0.62874	-0.22473
158.028	-0.62875	-0.22473
158.045	-0.62877	-0.22473
158.061	-0.62879	-0.22473
158.078	-0.62881	-0.22473

158.094	-0.62883	-0.22473
158.112	-0.62885	-0.22472
158.127	-0.62887	-0.22472
158.145	-0.62889	-0.22472
158.161	-0.62891	-0.22471
158.179	-0.62893	-0.22471
158.194	-0.62894	-0.22471
158.212	-0.62894	-0.2247
158.228	-0.62896	-0.2247
158.245	-0.62896	-0.2247
158.262	-0.62897	-0.2247
158.278	-0.62898	-0.2247
158.294	-0.629	-0.2247
158.311	-0.62902	-0.2247
158.328	-0.62904	-0.2247
158.344	-0.62906	-0.2247
158.361	-0.62908	-0.2247
158.377	-0.6291	-0.2247
158.395	-0.62911	-0.2247
158.412	-0.62913	-0.22469
158.428	-0.62914	-0.22469
158.444	-0.62916	-0.22469
158.461	-0.62917	-0.22468
158.478	-0.62918	-0.22468
158.494	-0.6292	-0.22468
158.511	-0.62921	-0.22468
158.528	-0.62923	-0.22468
158.544	-0.62924	-0.22468
158.561	-0.62925	-0.22469
158.578	-0.62926	-0.22469

158.595	-0.62927	-0.22469
158.611	-0.62928	-0.22469
158.628	-0.6293	-0.22469
158.645	-0.62932	-0.22469
158.662	-0.62933	-0.22469
158.678	-0.62936	-0.22468
158.694	-0.62937	-0.22468
158.712	-0.62939	-0.22467
158.727	-0.62941	-0.22467
158.744	-0.62943	-0.22467
158.762	-0.62945	-0.22467
158.778	-0.62946	-0.22467
158.795	-0.62947	-0.22467
158.811	-0.62948	-0.22466
158.827	-0.6295	-0.22466
158.843	-0.62952	-0.22466
158.861	-0.62953	-0.22466
158.877	-0.62955	-0.22466
158.893	-0.62958	-0.22466
158.91	-0.62959	-0.22466
158.928	-0.6296	-0.22466
158.943	-0.62962	-0.22465
158.961	-0.62964	-0.22466
158.978	-0.62965	-0.22465
158.995	-0.62966	-0.22465
159.011	-0.62968	-0.22465
159.028	-0.6297	-0.22465
159.044	-0.62973	-0.22465
159.061	-0.62975	-0.22465
159.077	-0.62978	-0.22465

159.094	-0.62981	-0.22465
159.111	-0.62984	-0.22465
159.127	-0.62986	-0.22465
159.145	-0.62988	-0.22465
159.161	-0.6299	-0.22465
159.177	-0.62992	-0.22466
159.194	-0.62993	-0.22466
159.212	-0.62995	-0.22466
159.228	-0.62997	-0.22466
159.245	-0.62999	-0.22466
159.262	-0.63002	-0.22466
159.278	-0.63004	-0.22466
159.295	-0.63006	-0.22466
159.311	-0.63008	-0.22466
159.329	-0.6301	-0.22466
159.345	-0.63012	-0.22467
159.362	-0.63014	-0.22467
159.378	-0.63016	-0.22468
159.395	-0.63019	-0.22468
159.412	-0.63021	-0.22469
159.428	-0.63025	-0.22469
159.445	-0.63027	-0.22469
159.461	-0.63029	-0.22469
159.478	-0.63031	-0.22469
159.496	-0.63034	-0.22469
159.512	-0.63036	-0.22469
159.529	-0.63038	-0.22469
159.544	-0.63041	-0.22469
159.562	-0.63043	-0.22469
159.578	-0.63046	-0.22469

159.595	-0.63049	-0.22469
159.612	-0.63052	-0.22469
159.628	-0.63054	-0.22469
159.645	-0.63057	-0.22469
159.662	-0.63059	-0.22469
159.678	-0.63061	-0.2247
159.695	-0.63063	-0.2247
159.713	-0.63065	-0.2247
159.73	-0.63067	-0.2247
159.746	-0.6307	-0.2247
159.763	-0.63073	-0.2247
159.779	-0.63075	-0.2247
159.795	-0.63077	-0.22469
159.812	-0.63079	-0.22469
159.829	-0.63081	-0.22469
159.846	-0.63084	-0.22469
159.862	-0.63087	-0.22469
159.879	-0.63089	-0.2247
159.896	-0.63092	-0.2247
159.913	-0.63096	-0.2247
159.929	-0.63099	-0.2247
159.945	-0.63103	-0.22471
159.962	-0.63105	-0.22471
159.979	-0.63108	-0.22471
159.996	-0.6311	-0.22471
160.013	-0.63112	-0.22471
160.029	-0.63114	-0.22471
160.045	-0.63115	-0.22471
160.062	-0.63117	-0.22471
160.079	-0.63119	-0.2247

160.096	-0.63122	-0.2247
160.113	-0.63125	-0.2247
160.128	-0.63128	-0.22471
160.147	-0.6313	-0.22471
160.163	-0.63133	-0.22472
160.179	-0.63135	-0.22472
160.197	-0.63136	-0.22473
160.213	-0.63138	-0.22473
160.229	-0.6314	-0.22473
160.246	-0.63142	-0.22473
160.263	-0.63144	-0.22473
160.279	-0.63146	-0.22473
160.296	-0.63148	-0.22473
160.313	-0.6315	-0.22473
160.329	-0.63153	-0.22472
160.346	-0.63155	-0.22472
160.363	-0.63157	-0.22472
160.38	-0.63158	-0.22472
160.396	-0.6316	-0.22472
160.413	-0.63163	-0.22472
160.429	-0.63165	-0.22472
160.446	-0.63168	-0.22472
160.463	-0.63171	-0.22472
160.48	-0.63174	-0.22472
160.496	-0.63176	-0.22472
160.513	-0.63178	-0.22472
160.53	-0.6318	-0.22472
160.546	-0.63181	-0.22472
160.563	-0.63183	-0.22472
160.58	-0.63185	-0.22471

160.595	-0.63187	-0.22471
160.613	-0.63189	-0.22471
160.63	-0.6319	-0.2247
160.646	-0.63192	-0.2247
160.662	-0.63193	-0.2247
160.68	-0.63195	-0.2247
160.697	-0.63196	-0.2247
160.712	-0.63198	-0.2247
160.729	-0.63199	-0.2247
160.746	-0.63202	-0.2247
160.763	-0.63204	-0.2247
160.78	-0.63206	-0.2247
160.796	-0.63209	-0.2247
160.813	-0.63211	-0.2247
160.83	-0.63212	-0.2247
160.846	-0.63214	-0.2247
160.863	-0.63215	-0.22469
160.879	-0.63217	-0.22469
160.896	-0.63219	-0.22469
160.913	-0.63221	-0.22469
160.93	-0.63223	-0.22469
160.946	-0.63224	-0.2247
160.963	-0.63226	-0.2247
160.979	-0.63228	-0.2247
160.996	-0.6323	-0.2247
161.013	-0.63232	-0.2247
161.029	-0.63234	-0.2247
161.046	-0.63236	-0.2247
161.063	-0.63238	-0.22469
161.079	-0.63241	-0.22469

161.096	-0.63243	-0.22469
161.113	-0.63246	-0.22469
161.129	-0.63248	-0.22469
161.146	-0.6325	-0.22469
161.162	-0.63252	-0.2247
161.18	-0.63253	-0.2247
161.196	-0.63255	-0.2247
161.213	-0.63256	-0.22471
161.23	-0.63257	-0.22471
161.246	-0.63258	-0.22471
161.263	-0.63259	-0.22471
161.28	-0.6326	-0.22471
161.297	-0.63261	-0.2247
161.312	-0.63263	-0.2247
161.33	-0.63264	-0.22469
161.346	-0.63266	-0.22469
161.362	-0.63269	-0.22469
161.379	-0.63271	-0.22469
161.396	-0.63274	-0.22469
161.413	-0.63276	-0.22469
161.429	-0.63278	-0.2247
161.446	-0.6328	-0.2247
161.463	-0.63282	-0.2247
161.48	-0.63283	-0.2247
161.497	-0.63285	-0.22471
161.512	-0.63286	-0.22471
161.529	-0.63287	-0.2247
161.546	-0.63288	-0.2247
161.563	-0.6329	-0.2247
161.58	-0.63291	-0.2247

161.596	-0.63293	-0.2247
161.613	-0.63294	-0.2247
161.63	-0.63296	-0.2247
161.646	-0.63297	-0.2247
161.663	-0.63299	-0.2247
161.679	-0.63301	-0.2247
161.696	-0.63303	-0.2247
161.713	-0.63305	-0.2247
161.73	-0.63306	-0.2247
161.746	-0.63308	-0.2247
161.763	-0.6331	-0.2247
161.78	-0.63312	-0.2247
161.796	-0.63314	-0.22469
161.813	-0.63315	-0.22469
161.83	-0.63315	-0.22469
161.846	-0.63317	-0.22469
161.863	-0.63318	-0.22469
161.88	-0.63319	-0.22468
161.896	-0.63321	-0.22468
161.912	-0.63322	-0.22469
161.929	-0.63324	-0.22469
161.946	-0.63326	-0.22469
161.962	-0.63328	-0.22469
161.979	-0.63329	-0.2247
161.996	-0.63331	-0.2247
162.013	-0.63332	-0.2247
162.029	-0.63333	-0.2247
162.046	-0.63333	-0.22469
162.062	-0.63333	-0.22469
162.079	-0.63334	-0.22469

162.096	-0.63334	-0.22469
162.112	-0.63335	-0.22468
162.129	-0.63336	-0.22468
162.146	-0.63336	-0.22468
162.161	-0.63338	-0.22468
162.178	-0.63339	-0.22468
162.195	-0.63341	-0.22468
162.212	-0.63342	-0.22468
162.228	-0.63344	-0.22468
162.245	-0.63346	-0.22468
162.261	-0.63348	-0.22468
162.279	-0.63349	-0.22468
162.294	-0.6335	-0.22468
162.311	-0.63352	-0.22467
162.329	-0.63353	-0.22467
162.345	-0.63353	-0.22466
162.361	-0.63355	-0.22466
162.379	-0.63356	-0.22466
162.395	-0.63357	-0.22465
162.412	-0.63359	-0.22465
162.428	-0.63361	-0.22465
162.445	-0.63363	-0.22465
162.462	-0.63365	-0.22465
162.478	-0.63367	-0.22465
162.495	-0.63369	-0.22465
162.512	-0.63371	-0.22466
162.528	-0.63373	-0.22466
162.545	-0.63375	-0.22466
162.561	-0.63377	-0.22466
162.578	-0.6338	-0.22466

162.595	-0.63382	-0.22466
162.612	-0.63384	-0.22466
162.628	-0.63385	-0.22466
162.645	-0.63387	-0.22467
162.662	-0.63389	-0.22467
162.678	-0.63391	-0.22467
162.696	-0.63393	-0.22467
162.712	-0.63395	-0.22468
162.729	-0.63397	-0.22468
162.745	-0.63399	-0.22468
162.762	-0.63401	-0.22468
162.778	-0.63402	-0.22469
162.795	-0.63404	-0.22469
162.811	-0.63407	-0.22469
162.828	-0.63409	-0.22469
162.845	-0.63412	-0.2247
162.862	-0.63415	-0.2247
162.879	-0.63417	-0.22471
162.895	-0.6342	-0.22471
162.912	-0.63422	-0.22472
162.929	-0.63425	-0.22472
162.946	-0.63427	-0.22472
162.962	-0.6343	-0.22473
162.979	-0.63432	-0.22474
162.996	-0.63435	-0.22474
163.012	-0.63437	-0.22474
163.029	-0.6344	-0.22475
163.046	-0.63443	-0.22475
163.063	-0.63445	-0.22476
163.079	-0.63448	-0.22476

163.096	-0.63451	-0.22476
163.112	-0.63454	-0.22477
163.129	-0.63456	-0.22477
163.145	-0.63459	-0.22478
163.162	-0.63461	-0.22478
163.179	-0.63463	-0.22478
163.196	-0.63465	-0.22478
163.212	-0.63467	-0.22478
163.228	-0.63469	-0.22478
163.246	-0.63472	-0.22478
163.263	-0.63475	-0.22479
163.279	-0.63478	-0.22479
163.295	-0.63481	-0.2248
163.313	-0.63484	-0.2248
163.33	-0.63487	-0.22481
163.347	-0.6349	-0.22482
163.362	-0.63492	-0.22483
163.38	-0.63494	-0.22483
163.396	-0.63496	-0.22484
163.413	-0.63498	-0.22484
163.429	-0.635	-0.22484
163.446	-0.63502	-0.22484
163.462	-0.63503	-0.22484
163.479	-0.63505	-0.22484
163.496	-0.63508	-0.22484
163.513	-0.6351	-0.22484
163.53	-0.63513	-0.22485
163.546	-0.63516	-0.22485
163.564	-0.63519	-0.22486
163.58	-0.63522	-0.22486

163.597	-0.63525	-0.22487
163.613	-0.63527	-0.22487
163.63	-0.6353	-0.22487
163.647	-0.63533	-0.22487
163.663	-0.63535	-0.22487
163.68	-0.63537	-0.22487
163.697	-0.63539	-0.22488
163.713	-0.63541	-0.22488
163.729	-0.63543	-0.22488
163.746	-0.63545	-0.22489
163.763	-0.63547	-0.22489
163.779	-0.63549	-0.22489
163.797	-0.63551	-0.22488
163.812	-0.63552	-0.22488
163.829	-0.63554	-0.22488
163.847	-0.63555	-0.22487
163.863	-0.63557	-0.22487
163.879	-0.63558	-0.22486
163.896	-0.6356	-0.22486
163.912	-0.63561	-0.22486
163.929	-0.63563	-0.22486
163.945	-0.63564	-0.22485
163.962	-0.63565	-0.22485
163.978	-0.63566	-0.22486
163.995	-0.63568	-0.22486
164.012	-0.63569	-0.22486
164.03	-0.63571	-0.22486
164.046	-0.63572	-0.22486
164.062	-0.63573	-0.22486
164.079	-0.63575	-0.22486

164.095	-0.63576	-0.22486
164.112	-0.63579	-0.22486
164.13	-0.63581	-0.22486
164.146	-0.63582	-0.22486
164.162	-0.63584	-0.22486
164.179	-0.63586	-0.22486
164.196	-0.63588	-0.22486
164.212	-0.63589	-0.22486
164.228	-0.63591	-0.22486
164.245	-0.63592	-0.22485
164.262	-0.63594	-0.22485
164.279	-0.63595	-0.22485
164.295	-0.63597	-0.22485
164.312	-0.63599	-0.22486
164.329	-0.63602	-0.22486
164.346	-0.63604	-0.22486
164.363	-0.63606	-0.22486
164.379	-0.63609	-0.22487
164.395	-0.63611	-0.22487
164.412	-0.63613	-0.22487
164.429	-0.63614	-0.22488
164.445	-0.63615	-0.22487
164.462	-0.63616	-0.22488
164.479	-0.63617	-0.22488
164.496	-0.63618	-0.22488
164.513	-0.6362	-0.22488
164.528	-0.63621	-0.22488
164.546	-0.63623	-0.22488
164.563	-0.63626	-0.22489
164.579	-0.63628	-0.22489

164.595	-0.63631	-0.22489
164.612	-0.63634	-0.22488
164.629	-0.63636	-0.22488
164.646	-0.63637	-0.22488
164.663	-0.63639	-0.22488
164.679	-0.63641	-0.22488
164.696	-0.63643	-0.22487
164.713	-0.63644	-0.22488
164.729	-0.63646	-0.22488
164.746	-0.63648	-0.22488
164.762	-0.6365	-0.22489
164.779	-0.63652	-0.22489
164.796	-0.63654	-0.2249
164.812	-0.63656	-0.2249
164.829	-0.63658	-0.22491
164.845	-0.63661	-0.22491
164.862	-0.63663	-0.22492
164.879	-0.63666	-0.22492
164.896	-0.63668	-0.22492
164.913	-0.6367	-0.22493
164.929	-0.63672	-0.22493
164.946	-0.63674	-0.22493
164.962	-0.63676	-0.22493
164.98	-0.63678	-0.22493
164.996	-0.6368	-0.22493
165.012	-0.63681	-0.22493
165.028	-0.63683	-0.22494
165.046	-0.63685	-0.22494
165.062	-0.63688	-0.22495
165.08	-0.6369	-0.22495

165.096	-0.63692	-0.22495
165.113	-0.63695	-0.22496
165.129	-0.63697	-0.22496
165.146	-0.63699	-0.22496
165.163	-0.63701	-0.22496
165.179	-0.63702	-0.22496
165.197	-0.63704	-0.22497
165.212	-0.63706	-0.22497
165.229	-0.63707	-0.22497
165.246	-0.63709	-0.22497
165.263	-0.63711	-0.22497
165.279	-0.63713	-0.22498
165.296	-0.63715	-0.22498
165.312	-0.63717	-0.22498
165.328	-0.6372	-0.22498
165.346	-0.63722	-0.22498
165.362	-0.63724	-0.22498
165.38	-0.63725	-0.22498
165.396	-0.63727	-0.22498
165.411	-0.63729	-0.22498
165.429	-0.63731	-0.22498
165.445	-0.63733	-0.22498
165.461	-0.63736	-0.22497
165.478	-0.63738	-0.22497
165.496	-0.6374	-0.22498
165.512	-0.63742	-0.22498
165.529	-0.63744	-0.22498
165.545	-0.63746	-0.22499
165.562	-0.63748	-0.22499
165.579	-0.6375	-0.225

165.596	-0.63752	-0.225
165.612	-0.63755	-0.22501
165.629	-0.63757	-0.22501
165.646	-0.63759	-0.22501
165.662	-0.63761	-0.225
165.679	-0.63763	-0.225
165.695	-0.63764	-0.225
165.711	-0.63765	-0.22499
165.729	-0.63767	-0.22499
165.745	-0.63769	-0.22499
165.763	-0.63771	-0.22499
165.778	-0.63773	-0.225
165.795	-0.63775	-0.225
165.811	-0.63777	-0.22501
165.827	-0.6378	-0.22501
165.845	-0.63782	-0.22501
165.861	-0.63783	-0.22501
165.879	-0.63785	-0.22501
165.896	-0.63787	-0.22501
165.912	-0.63788	-0.225
165.928	-0.6379	-0.225
165.944	-0.63791	-0.22499
165.961	-0.63793	-0.22499
165.978	-0.63794	-0.225
165.994	-0.63796	-0.225
166.011	-0.63797	-0.22501
166.028	-0.63799	-0.22501
166.045	-0.63801	-0.22502
166.061	-0.63803	-0.22503
166.079	-0.63805	-0.22503

166.095	-0.63808	-0.22504
166.112	-0.63811	-0.22504
166.129	-0.63814	-0.22504
166.145	-0.63817	-0.22504
166.162	-0.63819	-0.22504
166.178	-0.63821	-0.22505
166.196	-0.63824	-0.22505
166.212	-0.63826	-0.22505
166.229	-0.63828	-0.22505
166.245	-0.6383	-0.22505
166.262	-0.63831	-0.22506
166.278	-0.63832	-0.22506
166.295	-0.63834	-0.22507
166.312	-0.63836	-0.22507
166.328	-0.63838	-0.22508
166.345	-0.6384	-0.22508
166.363	-0.63842	-0.22509
166.379	-0.63845	-0.22509
166.395	-0.63847	-0.22509
166.411	-0.6385	-0.2251
166.429	-0.63853	-0.2251
166.445	-0.63856	-0.2251
166.462	-0.63858	-0.2251
166.478	-0.6386	-0.22511
166.496	-0.63862	-0.22511
166.512	-0.63863	-0.22512
166.529	-0.63865	-0.22512
166.546	-0.63866	-0.22512
166.562	-0.63867	-0.22512
166.579	-0.63869	-0.22512

166.595	-0.63872	-0.22512
166.612	-0.63875	-0.22513
166.629	-0.63878	-0.22513
166.645	-0.63881	-0.22513
166.662	-0.63884	-0.22514
166.679	-0.63887	-0.22514
166.696	-0.63889	-0.22515
166.712	-0.63892	-0.22516
166.729	-0.63894	-0.22516
166.746	-0.63897	-0.22517
166.763	-0.639	-0.22517
166.779	-0.63902	-0.22517
166.795	-0.63905	-0.22518
166.812	-0.63907	-0.22518
166.83	-0.63909	-0.22518
166.846	-0.63912	-0.22518
166.862	-0.63914	-0.22519
166.879	-0.63917	-0.22519
166.897	-0.63919	-0.2252
166.912	-0.63922	-0.2252
166.929	-0.63924	-0.2252
166.946	-0.63926	-0.22521
166.962	-0.63929	-0.22521
166.98	-0.63931	-0.22521
166.997	-0.63933	-0.22521
167.013	-0.63935	-0.22521
167.03	-0.63937	-0.22522
167.046	-0.63939	-0.22522
167.063	-0.63941	-0.22522
167.08	-0.63943	-0.22522

167.096	-0.63946	-0.22523
167.113	-0.63948	-0.22524
167.129	-0.63951	-0.22524
167.146	-0.63954	-0.22525
167.163	-0.63956	-0.22525
167.18	-0.63959	-0.22525
167.197	-0.63961	-0.22526
167.212	-0.63963	-0.22526
167.23	-0.63965	-0.22527
167.246	-0.63968	-0.22527
167.263	-0.6397	-0.22527
167.28	-0.63972	-0.22527
167.296	-0.63974	-0.22527
167.312	-0.63977	-0.22527
167.33	-0.63979	-0.22526
167.345	-0.63981	-0.22526
167.363	-0.63983	-0.22526
167.38	-0.63985	-0.22526
167.396	-0.63987	-0.22526
167.413	-0.6399	-0.22527
167.43	-0.63993	-0.22527
167.447	-0.63995	-0.22527
167.462	-0.63997	-0.22527
167.48	-0.63999	-0.22527
167.497	-0.64001	-0.22527
167.514	-0.64003	-0.22527
167.53	-0.64004	-0.22527
167.546	-0.64005	-0.22528
167.562	-0.64006	-0.22528
167.58	-0.64008	-0.22527

167.596	-0.6401	-0.22527
167.613	-0.64012	-0.22528
167.629	-0.64014	-0.22528
167.646	-0.64016	-0.22529
167.662	-0.64018	-0.22529
167.68	-0.6402	-0.2253
167.697	-0.64021	-0.2253
167.713	-0.64023	-0.22531
167.73	-0.64024	-0.22531
167.747	-0.64026	-0.22531
167.763	-0.64027	-0.22531
167.779	-0.64029	-0.22531
167.797	-0.6403	-0.2253
167.814	-0.64032	-0.2253
167.83	-0.64034	-0.2253
167.847	-0.64036	-0.2253
167.863	-0.64038	-0.2253
167.88	-0.64039	-0.22531
167.896	-0.6404	-0.22531
167.913	-0.64041	-0.22531
167.93	-0.64042	-0.22531
167.945	-0.64043	-0.22531
167.963	-0.64045	-0.22532
167.98	-0.64046	-0.22532
167.996	-0.64048	-0.22532
168.013	-0.6405	-0.22532
168.029	-0.64052	-0.22532
168.046	-0.64054	-0.22532
168.063	-0.64056	-0.22532
168.079	-0.64058	-0.22532

168.096	-0.64059	-0.22532
168.113	-0.6406	-0.22532
168.129	-0.64062	-0.22532
168.147	-0.64064	-0.22532
168.163	-0.64065	-0.22532
168.179	-0.64067	-0.22533
168.196	-0.64069	-0.22533
168.213	-0.6407	-0.22533
168.23	-0.64071	-0.22534
168.247	-0.64072	-0.22534
168.263	-0.64073	-0.22534
168.279	-0.64074	-0.22534
168.296	-0.64074	-0.22535
168.313	-0.64075	-0.22535
168.329	-0.64076	-0.22535
168.346	-0.64077	-0.22535
168.363	-0.64079	-0.22535
168.38	-0.6408	-0.22535
168.396	-0.64082	-0.22535
168.413	-0.64084	-0.22535
168.43	-0.64086	-0.22535
168.446	-0.64089	-0.22535
168.463	-0.64092	-0.22535
168.479	-0.64095	-0.22535
168.497	-0.64099	-0.22536
168.513	-0.64102	-0.22536
168.53	-0.64104	-0.22536
168.546	-0.64107	-0.22536
168.563	-0.64108	-0.22536
168.58	-0.64109	-0.22536

168.596	-0.64109	-0.22536
168.613	-0.6411	-0.22536
168.63	-0.64112	-0.22536
168.647	-0.64113	-0.22537
168.664	-0.64114	-0.22538
168.679	-0.64116	-0.22538
168.696	-0.64118	-0.22539
168.713	-0.6412	-0.22539
168.73	-0.64122	-0.2254
168.746	-0.64124	-0.2254
168.763	-0.64126	-0.2254
168.779	-0.64129	-0.2254
168.795	-0.64131	-0.2254
168.813	-0.64134	-0.2254
168.83	-0.64137	-0.2254
168.847	-0.6414	-0.22541
168.863	-0.64142	-0.22541
168.88	-0.64144	-0.22541
168.897	-0.64145	-0.22541
168.913	-0.64147	-0.22541
168.93	-0.64149	-0.22541
168.947	-0.6415	-0.22542
168.963	-0.64153	-0.22542
168.98	-0.64155	-0.22543
168.997	-0.64158	-0.22544
169.014	-0.6416	-0.22544
169.031	-0.64163	-0.22545
169.046	-0.64166	-0.22545
169.064	-0.64168	-0.22545
169.08	-0.64171	-0.22545

169.098	-0.64174	-0.22545
169.113	-0.64176	-0.22545
169.13	-0.64179	-0.22545
169.147	-0.64182	-0.22545
169.163	-0.64184	-0.22545
169.179	-0.64187	-0.22545
169.197	-0.64189	-0.22546
169.214	-0.64192	-0.22546
169.23	-0.64193	-0.22547
169.246	-0.64195	-0.22547
169.263	-0.64197	-0.22547
169.28	-0.64198	-0.22547
169.297	-0.64199	-0.22547
169.313	-0.64201	-0.22547
169.33	-0.64202	-0.22548
169.346	-0.64204	-0.22548
169.364	-0.64206	-0.22548
169.381	-0.64208	-0.22548
169.397	-0.64211	-0.22548
169.413	-0.64213	-0.22548
169.43	-0.64216	-0.22548
169.446	-0.64219	-0.22548
169.463	-0.64222	-0.22548
169.48	-0.64225	-0.22548
169.496	-0.64227	-0.22548
169.512	-0.64229	-0.22549
169.53	-0.64231	-0.22549
169.547	-0.64233	-0.22549
169.564	-0.64234	-0.22549
169.58	-0.64236	-0.2255

169.597	-0.64238	-0.2255
169.613	-0.6424	-0.2255
169.63	-0.64242	-0.2255
169.646	-0.64244	-0.2255
169.663	-0.64246	-0.2255
169.679	-0.64248	-0.22549
169.696	-0.6425	-0.22549
169.713	-0.64251	-0.22549
169.73	-0.64252	-0.22549
169.747	-0.64254	-0.22549
169.763	-0.64255	-0.22549
169.781	-0.64257	-0.2255
169.797	-0.64259	-0.2255
169.813	-0.64261	-0.2255
169.83	-0.64263	-0.22551
169.847	-0.64266	-0.22551
169.864	-0.64268	-0.22551
169.88	-0.6427	-0.22551
169.896	-0.64272	-0.22551
169.913	-0.64274	-0.22551
169.93	-0.64277	-0.22551
169.947	-0.6428	-0.22551
169.963	-0.64283	-0.22551
169.98	-0.64285	-0.22552
169.997	-0.64288	-0.22552
170.013	-0.64289	-0.22551
170.03	-0.6429	-0.22551
170.046	-0.64292	-0.22551
170.064	-0.64293	-0.22551
170.08	-0.64294	-0.22551

170.096	-0.64296	-0.22551
170.111	-0.64298	-0.22551
170.129	-0.643	-0.22551
170.146	-0.64303	-0.22551
170.163	-0.64305	-0.22552
170.18	-0.64308	-0.22552
170.197	-0.6431	-0.22553
170.213	-0.64312	-0.22553
170.23	-0.64314	-0.22554
170.246	-0.64316	-0.22554
170.263	-0.64318	-0.22554
170.28	-0.6432	-0.22554
170.296	-0.64321	-0.22554
170.314	-0.64322	-0.22554
170.33	-0.64323	-0.22554
170.346	-0.64324	-0.22554
170.363	-0.64326	-0.22555
170.379	-0.64327	-0.22555
170.396	-0.64329	-0.22555
170.412	-0.64331	-0.22555
170.43	-0.64334	-0.22556
170.446	-0.64336	-0.22556
170.463	-0.64339	-0.22556
170.48	-0.64342	-0.22556
170.497	-0.64344	-0.22556
170.513	-0.64347	-0.22556
170.529	-0.64349	-0.22557
170.546	-0.64351	-0.22557
170.563	-0.64353	-0.22557
170.58	-0.64355	-0.22557

170.597	-0.64356	-0.22557
170.613	-0.64358	-0.22558
170.63	-0.64359	-0.22558
170.647	-0.6436	-0.22558
170.663	-0.64362	-0.22559
170.68	-0.64363	-0.22559
170.697	-0.64364	-0.22559
170.713	-0.64366	-0.22559
170.73	-0.64368	-0.2256
170.746	-0.64369	-0.2256
170.763	-0.64371	-0.2256
170.779	-0.64372	-0.2256
170.797	-0.64374	-0.22561
170.814	-0.64376	-0.22561
170.83	-0.64379	-0.22561
170.847	-0.64381	-0.22561
170.863	-0.64384	-0.22561
170.879	-0.64387	-0.22562
170.896	-0.64389	-0.22562
170.914	-0.64392	-0.22562
170.93	-0.64394	-0.22562
170.947	-0.64396	-0.22563
170.963	-0.64397	-0.22563
170.981	-0.64399	-0.22563
170.997	-0.64401	-0.22563
171.013	-0.64403	-0.22563
171.03	-0.64406	-0.22563
171.047	-0.64408	-0.22563
171.064	-0.6441	-0.22563
171.08	-0.64412	-0.22563

171.097	-0.64414	-0.22563
171.113	-0.64415	-0.22563
171.13	-0.64417	-0.22564
171.147	-0.64419	-0.22564
171.163	-0.6442	-0.22564
171.18	-0.64423	-0.22564
171.196	-0.64426	-0.22564
171.213	-0.64429	-0.22565
171.23	-0.64432	-0.22565
171.246	-0.64435	-0.22565
171.264	-0.64437	-0.22566
171.28	-0.6444	-0.22566
171.297	-0.64442	-0.22566
171.314	-0.64444	-0.22566
171.329	-0.64446	-0.22567
171.347	-0.64448	-0.22567
171.364	-0.6445	-0.22567
171.38	-0.64452	-0.22567
171.397	-0.64455	-0.22567
171.413	-0.64457	-0.22567
171.43	-0.6446	-0.22568
171.447	-0.64462	-0.22568
171.463	-0.64464	-0.22568
171.48	-0.64466	-0.22568
171.497	-0.64468	-0.22568
171.513	-0.6447	-0.22568
171.531	-0.64471	-0.22568
171.546	-0.64473	-0.22568
171.563	-0.64475	-0.22568
171.58	-0.64477	-0.22568

171.597	-0.6448	-0.22568
171.614	-0.64482	-0.22568
171.63	-0.64485	-0.22568
171.647	-0.64487	-0.22568
171.663	-0.64489	-0.22568
171.68	-0.64492	-0.22569
171.697	-0.64494	-0.22569
171.713	-0.64496	-0.2257
171.73	-0.64498	-0.2257
171.747	-0.64499	-0.2257
171.763	-0.64501	-0.22571
171.781	-0.64503	-0.22571
171.798	-0.64505	-0.22572
171.813	-0.64507	-0.22572
171.831	-0.6451	-0.22572
171.847	-0.64512	-0.22572
171.864	-0.64514	-0.22572
171.88	-0.64517	-0.22572
171.897	-0.64519	-0.22572
171.913	-0.64521	-0.22573
171.931	-0.64524	-0.22573
171.946	-0.64525	-0.22572
171.964	-0.64526	-0.22572
171.981	-0.64527	-0.22573
171.997	-0.64528	-0.22573
172.014	-0.6453	-0.22573
172.031	-0.64532	-0.22573
172.047	-0.64535	-0.22574
172.064	-0.64538	-0.22574
172.081	-0.6454	-0.22574

172.097	-0.64543	-0.22574
172.114	-0.64545	-0.22574
172.13	-0.64547	-0.22574
172.147	-0.64548	-0.22574
172.164	-0.64549	-0.22574
172.18	-0.6455	-0.22574
172.197	-0.64551	-0.22574
172.213	-0.64552	-0.22574
172.23	-0.64554	-0.22574
172.247	-0.64555	-0.22574
172.264	-0.64557	-0.22574
172.28	-0.6456	-0.22574
172.296	-0.64562	-0.22574
172.313	-0.64565	-0.22574
172.33	-0.64568	-0.22574
172.347	-0.6457	-0.22574
172.363	-0.64572	-0.22574
172.38	-0.64574	-0.22574
172.397	-0.64576	-0.22575
172.413	-0.64577	-0.22575
172.43	-0.64578	-0.22575
172.447	-0.6458	-0.22575
172.463	-0.64581	-0.22575
172.48	-0.64582	-0.22575
172.496	-0.64583	-0.22575
172.513	-0.64584	-0.22575
172.53	-0.64585	-0.22574
172.546	-0.64587	-0.22574
172.564	-0.64588	-0.22574
172.58	-0.64589	-0.22574

172.597	-0.6459	-0.22575
172.613	-0.64591	-0.22575
172.63	-0.64592	-0.22575
172.646	-0.64593	-0.22575
172.663	-0.64595	-0.22576
172.68	-0.64596	-0.22576
172.697	-0.64598	-0.22576
172.714	-0.64599	-0.22576
172.731	-0.646	-0.22576
172.747	-0.64602	-0.22577
172.764	-0.64603	-0.22577
172.78	-0.64605	-0.22577
172.797	-0.64607	-0.22577
172.814	-0.64609	-0.22577
172.831	-0.64611	-0.22578
172.848	-0.64613	-0.22578
172.864	-0.64616	-0.22579
172.881	-0.64618	-0.22579
172.898	-0.6462	-0.2258
172.914	-0.64622	-0.2258
172.931	-0.64624	-0.22581
172.947	-0.64626	-0.22581
172.964	-0.64627	-0.22582
172.98	-0.64629	-0.22582
172.997	-0.64631	-0.22583
173.014	-0.64633	-0.22583
173.031	-0.64635	-0.22583
173.047	-0.64637	-0.22584
173.064	-0.6464	-0.22584
173.081	-0.64642	-0.22585

173.097	-0.64645	-0.22585
173.115	-0.64647	-0.22586
173.132	-0.64649	-0.22586
173.148	-0.64652	-0.22586
173.165	-0.64655	-0.22587
173.181	-0.64657	-0.22587
173.198	-0.6466	-0.22587
173.214	-0.64663	-0.22587
173.231	-0.64666	-0.22587
173.248	-0.6467	-0.22588
173.265	-0.64672	-0.22588
173.282	-0.64675	-0.22588
173.298	-0.64678	-0.22589
173.315	-0.64681	-0.2259
173.331	-0.64684	-0.22591
173.348	-0.64687	-0.22592
173.364	-0.64689	-0.22592
173.38	-0.64691	-0.22593
173.398	-0.64693	-0.22594
173.415	-0.64695	-0.22595
173.431	-0.64698	-0.22595
173.448	-0.647	-0.22596
173.464	-0.64703	-0.22597
173.482	-0.64706	-0.22597
173.498	-0.64709	-0.22598
173.515	-0.64712	-0.22599
173.531	-0.64714	-0.22599
173.548	-0.64717	-0.226
173.565	-0.64719	-0.226
173.582	-0.64722	-0.22601

173.598	-0.64724	-0.22601
173.615	-0.64726	-0.22601
173.631	-0.64729	-0.22601
173.649	-0.64732	-0.22601
173.665	-0.64735	-0.22602
173.682	-0.64737	-0.22602
173.699	-0.6474	-0.22602
173.715	-0.64743	-0.22603
173.731	-0.64746	-0.22603
173.749	-0.64749	-0.22604
173.765	-0.64753	-0.22604
173.781	-0.64756	-0.22604
173.799	-0.64759	-0.22605
173.815	-0.64762	-0.22605
173.832	-0.64765	-0.22606
173.849	-0.64767	-0.22606
173.865	-0.6477	-0.22606
173.882	-0.64772	-0.22606
173.898	-0.64775	-0.22606
173.915	-0.64777	-0.22606
173.932	-0.6478	-0.22606
173.949	-0.64783	-0.22606
173.965	-0.64786	-0.22606
173.981	-0.64788	-0.22606
173.998	-0.64791	-0.22606
174.014	-0.64793	-0.22606
174.032	-0.64795	-0.22606
174.048	-0.64797	-0.22607
174.065	-0.648	-0.22607
174.082	-0.64802	-0.22607

174.098	-0.64805	-0.22607
174.115	-0.64807	-0.22607
174.131	-0.6481	-0.22607
174.15	-0.64812	-0.22607
174.166	-0.64815	-0.22608
174.182	-0.64817	-0.22608
174.199	-0.64819	-0.22608
174.215	-0.64821	-0.22608
174.232	-0.64822	-0.22609
174.249	-0.64824	-0.22609
174.266	-0.64826	-0.22609
174.283	-0.64829	-0.22609
174.299	-0.64832	-0.22609
174.315	-0.64834	-0.22608
174.332	-0.64837	-0.22608
174.349	-0.6484	-0.22608
174.366	-0.64842	-0.22607
174.382	-0.64844	-0.22607
174.398	-0.64846	-0.22607
174.415	-0.64848	-0.22606
174.432	-0.64849	-0.22606
174.449	-0.64851	-0.22607
174.465	-0.64852	-0.22607
174.482	-0.64854	-0.22607
174.499	-0.64856	-0.22607
174.514	-0.64858	-0.22607
174.531	-0.64859	-0.22607
174.549	-0.64861	-0.22608
174.565	-0.64863	-0.22608
174.582	-0.64865	-0.22608

174.598	-0.64866	-0.22608
174.616	-0.64868	-0.22608
174.632	-0.64869	-0.22608
174.649	-0.64871	-0.22608
174.665	-0.64872	-0.22608
174.682	-0.64874	-0.22608
174.698	-0.64875	-0.22607
174.715	-0.64877	-0.22607
174.732	-0.64879	-0.22607
174.748	-0.6488	-0.22607
174.764	-0.64882	-0.22607
174.781	-0.64884	-0.22608
174.798	-0.64886	-0.22608
174.815	-0.64888	-0.22608
174.833	-0.6489	-0.22608
174.849	-0.64891	-0.22608
174.866	-0.64893	-0.22608
174.882	-0.64895	-0.22608
174.898	-0.64897	-0.22608
174.916	-0.64899	-0.22608
174.931	-0.649	-0.22608
174.948	-0.64902	-0.22608
174.964	-0.64903	-0.22608
174.982	-0.64904	-0.22608
174.998	-0.64906	-0.22609
175.015	-0.64908	-0.22609
175.031	-0.6491	-0.22609
175.048	-0.64911	-0.22609
175.065	-0.64913	-0.22609
175.081	-0.64915	-0.22609

175.099	-0.64916	-0.22609
175.115	-0.64918	-0.22608
175.131	-0.64919	-0.22608
175.148	-0.64921	-0.22608
175.165	-0.64922	-0.22609
175.182	-0.64923	-0.22609
175.198	-0.64924	-0.22609
175.215	-0.64925	-0.2261
175.231	-0.64926	-0.2261
175.248	-0.64927	-0.22611
175.265	-0.64928	-0.22611
175.282	-0.6493	-0.22611
175.298	-0.64931	-0.2261
175.315	-0.64933	-0.2261
175.332	-0.64934	-0.2261
175.348	-0.64936	-0.2261
175.364	-0.64938	-0.2261
175.381	-0.6494	-0.2261
175.398	-0.64942	-0.22611
175.414	-0.64944	-0.22611
175.431	-0.64945	-0.22611
175.447	-0.64947	-0.22611
175.465	-0.64949	-0.22611
175.482	-0.64951	-0.22611
175.498	-0.64954	-0.22612
175.515	-0.64956	-0.22612
175.532	-0.64957	-0.22612
175.549	-0.64958	-0.22612
175.566	-0.64959	-0.22612
175.581	-0.6496	-0.22612

175.599	-0.6496	-0.22612
175.615	-0.64961	-0.22613
175.632	-0.64962	-0.22613
175.649	-0.64963	-0.22613
175.665	-0.64964	-0.22613
175.681	-0.64966	-0.22613
175.698	-0.64968	-0.22614
175.714	-0.6497	-0.22614
175.731	-0.64971	-0.22614
175.748	-0.64972	-0.22614
175.764	-0.64974	-0.22614
175.781	-0.64975	-0.22615
175.798	-0.64976	-0.22615
175.814	-0.64978	-0.22614
175.831	-0.64979	-0.22614
175.847	-0.6498	-0.22614
175.864	-0.64982	-0.22614
175.881	-0.64983	-0.22614
175.897	-0.64985	-0.22615
175.914	-0.64986	-0.22615
175.931	-0.64987	-0.22616
175.948	-0.64988	-0.22616
175.965	-0.64989	-0.22616
175.981	-0.6499	-0.22616
175.998	-0.64992	-0.22617
176.014	-0.64993	-0.22617
176.032	-0.64995	-0.22617
176.048	-0.64997	-0.22617
176.065	-0.64999	-0.22618
176.081	-0.65 -0.22618	

176.098	-0.65001	-0.22618
176.115	-0.65003	-0.22618
176.131	-0.65004	-0.22619
176.147	-0.65005	-0.22619
176.164	-0.65007	-0.22619
176.181	-0.65008	-0.22619
176.198	-0.6501	-0.22619
176.214	-0.65011	-0.22619
176.231	-0.65013	-0.22619
176.248	-0.65015	-0.2262
176.265	-0.65017	-0.2262
176.281	-0.65019	-0.2262
176.298	-0.65021	-0.22621
176.315	-0.65023	-0.22621
176.332	-0.65025	-0.22621
176.348	-0.65027	-0.22621
176.365	-0.65029	-0.22622
176.381	-0.65031	-0.22622
176.399	-0.65032	-0.22622
176.415	-0.65033	-0.22623
176.432	-0.65034	-0.22623
176.447	-0.65036	-0.22624
176.465	-0.65037	-0.22624
176.481	-0.65039	-0.22625
176.498	-0.65041	-0.22626
176.514	-0.65043	-0.22627
176.531	-0.65045	-0.22627
176.548	-0.65046	-0.22627
176.566	-0.65048	-0.22628
176.582	-0.6505	-0.22629

176.599	-0.65052	-0.22629
176.615	-0.65055	-0.2263
176.632	-0.65057	-0.2263
176.648	-0.65059	-0.2263
176.665	-0.65062	-0.22631
176.681	-0.65064	-0.22631
176.699	-0.65067	-0.22631
176.715	-0.6507	-0.22631
176.732	-0.65073	-0.22631
176.748	-0.65075	-0.22632
176.766	-0.65078	-0.22632
176.781	-0.6508	-0.22632
176.799	-0.65082	-0.22632
176.815	-0.65085	-0.22633
176.832	-0.65087	-0.22633
176.849	-0.65089	-0.22634
176.866	-0.65092	-0.22634
176.881	-0.65095	-0.22634
176.898	-0.65097	-0.22635
176.915	-0.651	-0.22635
176.932	-0.65102	-0.22635
176.949	-0.65105	-0.22635
176.965	-0.65107	-0.22635
176.984	-0.65109	-0.22635
177	-0.65112	-0.22636
177.016	-0.65114	-0.22636
177.033	-0.65116	-0.22637
177.049	-0.65118	-0.22637
177.066	-0.65121	-0.22637
177.083	-0.65123	-0.22638

177.099	-0.65126	-0.22638
177.116	-0.65129	-0.22639
177.132	-0.65132	-0.22639
177.15	-0.65135	-0.22639
177.166	-0.65137	-0.2264
177.184	-0.6514	-0.2264
177.199	-0.65142	-0.2264
177.216	-0.65143	-0.2264
177.233	-0.65145	-0.2264
177.249	-0.65147	-0.2264
177.266	-0.6515	-0.2264
177.282	-0.65152	-0.2264
177.299	-0.65155	-0.2264
177.316	-0.65157	-0.2264
177.333	-0.6516	-0.2264
177.35	-0.65162	-0.22641
177.366	-0.65165	-0.22641
177.382	-0.65167	-0.22641
177.399	-0.6517	-0.22641
177.416	-0.65172	-0.22642
177.432	-0.65174	-0.22642
177.45	-0.65177	-0.22643
177.467	-0.6518	-0.22643
177.482	-0.65182	-0.22643
177.5	-0.65185	-0.22643
177.517	-0.65188	-0.22643
177.533	-0.6519	-0.22643
177.549	-0.65193	-0.22643
177.566	-0.65195	-0.22643
177.583	-0.65197	-0.22643

177.599	-0.65199	-0.22643
177.616	-0.65201	-0.22643
177.632	-0.65203	-0.22644
177.649	-0.65205	-0.22644
177.666	-0.65206	-0.22644
177.683	-0.65208	-0.22644
177.699	-0.6521	-0.22644
177.715	-0.65212	-0.22644
177.733	-0.65214	-0.22644
177.749	-0.65217	-0.22645
177.766	-0.65219	-0.22644
177.783	-0.65221	-0.22644
177.799	-0.65223	-0.22644
177.816	-0.65225	-0.22644
177.832	-0.65228	-0.22644
177.85	-0.6523	-0.22644
177.866	-0.65231	-0.22644
177.882	-0.65233	-0.22645
177.899	-0.65235	-0.22645
177.916	-0.65236	-0.22645
177.933	-0.65239	-0.22646
177.949	-0.65241	-0.22647
177.966	-0.65244	-0.22647
177.983	-0.65246	-0.22647
177.999	-0.65249	-0.22647
178.016	-0.65251	-0.22647
178.032	-0.65253	-0.22647
178.049	-0.65255	-0.22647
178.067	-0.65257	-0.22647
178.082	-0.65258	-0.22647

178.1	-0.6526	-0.22648
178.116	-0.65263	-0.22648
178.133	-0.65265	-0.22648
178.149	-0.65268	-0.22648
178.166	-0.6527	-0.22648
178.182	-0.65273	-0.22648
178.199	-0.65275	-0.22649
178.216	-0.65278	-0.22649
178.232	-0.6528	-0.22649
178.249	-0.65283	-0.22649
178.266	-0.65285	-0.22649
178.283	-0.65287	-0.2265
178.299	-0.6529	-0.2265
178.316	-0.65293	-0.2265
178.332	-0.65295	-0.2265
178.349	-0.65297	-0.2265
178.366	-0.653	-0.22651
178.383	-0.65302	-0.22651
178.399	-0.65304	-0.22651
178.416	-0.65306	-0.22652
178.432	-0.65309	-0.22652
178.448	-0.6531	-0.22652
178.465	-0.65312	-0.22652
178.482	-0.65314	-0.22652
178.5	-0.65316	-0.22653
178.516	-0.65318	-0.22653
178.532	-0.6532	-0.22653
178.549	-0.65323	-0.22654
178.566	-0.65326	-0.22654
178.582	-0.65329	-0.22655

178.6	-0.65332	-0.22655
178.615	-0.65335	-0.22655
178.633	-0.65338	-0.22656
178.649	-0.65341	-0.22655
178.666	-0.65344	-0.22655
178.682	-0.65346	-0.22655
178.7	-0.65348	-0.22655
178.716	-0.65349	-0.22655
178.733	-0.65351	-0.22655
178.75	-0.65353	-0.22656
178.766	-0.65355	-0.22656
178.783	-0.65358	-0.22656
178.8	-0.6536	-0.22657
178.816	-0.65363	-0.22657
178.833	-0.65366	-0.22658
178.849	-0.65368	-0.22658
178.866	-0.65371	-0.22658
178.883	-0.65374	-0.22658
178.9	-0.65376	-0.22658
178.916	-0.65379	-0.22658
178.933	-0.65381	-0.22658
178.949	-0.65383	-0.22658
178.967	-0.65385	-0.22659
178.983	-0.65386	-0.22659
178.999	-0.65387	-0.22659
179.015	-0.65389	-0.2266
179.032	-0.6539	-0.2266
179.049	-0.65391	-0.2266
179.066	-0.65393	-0.2266
179.083	-0.65395	-0.2266

179.1	-0.65397	-0.2266
179.117	-0.654	-0.2266
179.133	-0.65402	-0.2266
179.149	-0.65404	-0.2266
179.166	-0.65406	-0.2266
179.182	-0.65407	-0.2266
179.199	-0.65409	-0.2266
179.216	-0.6541	-0.2266
179.232	-0.65411	-0.2266
179.249	-0.65412	-0.22661
179.265	-0.65413	-0.22661
179.282	-0.65414	-0.22661
179.298	-0.65416	-0.22661
179.315	-0.65417	-0.22661
179.332	-0.65418	-0.22661
179.349	-0.6542	-0.22661
179.365	-0.65422	-0.22661
179.382	-0.65424	-0.22661
179.399	-0.65427	-0.22662
179.416	-0.65429	-0.22662
179.432	-0.65432	-0.22662
179.448	-0.65435	-0.22662
179.466	-0.65436	-0.22662
179.482	-0.65438	-0.22662
179.499	-0.65439	-0.22662
179.516	-0.65441	-0.22662
179.532	-0.65442	-0.22662
179.549	-0.65444	-0.22662
179.565	-0.65446	-0.22662
179.582	-0.65448	-0.22662

179.598	-0.6545	-0.22662
179.616	-0.65452	-0.22662
179.632	-0.65455	-0.22663
179.649	-0.65457	-0.22663
179.666	-0.65459	-0.22663
179.682	-0.65461	-0.22664
179.698	-0.65463	-0.22664
179.715	-0.65466	-0.22665
179.731	-0.65468	-0.22665
179.749	-0.65471	-0.22665
179.765	-0.65474	-0.22665
179.783	-0.65476	-0.22665
179.798	-0.65478	-0.22666
179.815	-0.65481	-0.22666
179.832	-0.65484	-0.22666
179.848	-0.65487	-0.22666
179.865	-0.65489	-0.22667
179.881	-0.65492	-0.22667
179.899	-0.65494	-0.22668
179.916	-0.65497	-0.22668
179.932	-0.655	-0.22668
179.949	-0.65502	-0.22668
179.965	-0.65505	-0.22669
179.982	-0.65507	-0.22669
180	-0.6551	-0.22669
180.016	-0.65513	-0.2267
180.033	-0.65516	-0.2267
180.05	-0.65519	-0.2267
180.065	-0.65522	-0.22671
180.082	-0.65526	-0.22671

180.099	-0.6553	-0.22672
180.116	-0.65535	-0.22672
180.132	-0.65539	-0.22673
180.149	-0.65543	-0.22674
180.166	-0.65546	-0.22674
180.183	-0.6555	-0.22675
180.199	-0.65553	-0.22676
180.216	-0.65556	-0.22676
180.232	-0.65558	-0.22677
180.248	-0.65561	-0.22678
180.266	-0.65563	-0.22678
180.282	-0.65566	-0.22679
180.299	-0.65569	-0.2268
180.315	-0.65573	-0.2268
180.333	-0.65575	-0.2268
180.349	-0.65578	-0.2268
180.366	-0.65581	-0.2268
180.383	-0.65584	-0.2268
180.399	-0.65587	-0.22681
180.416	-0.6559	-0.22681
180.433	-0.65594	-0.22681
180.449	-0.65597	-0.22682
180.466	-0.65599	-0.22683
180.483	-0.65602	-0.22683
180.5	-0.65605	-0.22684
180.517	-0.65608	-0.22685
180.532	-0.65611	-0.22685
180.55	-0.65614	-0.22686
180.566	-0.65617	-0.22686
180.583	-0.6562	-0.22686

180.6	-0.65623	-0.22687
180.616	-0.65627	-0.22687
180.633	-0.6563	-0.22688
180.65	-0.65633	-0.22688
180.667	-0.65636	-0.22688
180.684	-0.65638	-0.22689
180.7	-0.65641	-0.22689
180.716	-0.65644	-0.22689
180.733	-0.65646	-0.22689
180.75	-0.65649	-0.2269
180.766	-0.65652	-0.2269
180.783	-0.65655	-0.2269
180.8	-0.65658	-0.22691
180.816	-0.65661	-0.22691
180.833	-0.65663	-0.22692
180.849	-0.65666	-0.22692
180.867	-0.65668	-0.22693
180.882	-0.6567	-0.22694
180.9	-0.65673	-0.22694
180.916	-0.65675	-0.22695
180.933	-0.65677	-0.22695
180.949	-0.65678	-0.22696
180.967	-0.6568	-0.22696
180.983	-0.65682	-0.22697
180.999	-0.65684	-0.22697
181.017	-0.65687	-0.22697
181.034	-0.65689	-0.22698
181.051	-0.65691	-0.22698
181.068	-0.65693	-0.22698
181.084	-0.65695	-0.22699

181.1	-0.65697	-0.22699
181.117	-0.657	-0.22699
181.133	-0.65702	-0.22699
181.149	-0.65704	-0.22699
181.166	-0.65707	-0.22699
181.183	-0.65709	-0.22699
181.2	-0.65711	-0.22699
181.216	-0.65713	-0.227
181.233	-0.65715	-0.227
181.25	-0.65717	-0.227
181.266	-0.65719	-0.22701
181.283	-0.65721	-0.22701
181.299	-0.65722	-0.22701
181.316	-0.65724	-0.22701
181.333	-0.65726	-0.22701
181.349	-0.65728	-0.227
181.366	-0.65729	-0.227
181.382	-0.65731	-0.227
181.398	-0.65733	-0.227
181.415	-0.65734	-0.227
181.432	-0.65736	-0.22701
181.45	-0.65738	-0.22701
181.466	-0.65739	-0.22701
181.482	-0.6574	-0.22702
181.499	-0.65741	-0.22702
181.516	-0.65741	-0.22702
181.533	-0.65743	-0.22703
181.55	-0.65744	-0.22703
181.566	-0.65745	-0.22703
181.582	-0.65747	-0.22703

181.599	-0.65749	-0.22703
181.617	-0.65751	-0.22703
181.633	-0.65753	-0.22703
181.65	-0.65756	-0.22703
181.664	-0.65758	-0.22703
181.683	-0.65761	-0.22704
181.698	-0.65763	-0.22704
181.715	-0.65765	-0.22704
181.732	-0.65767	-0.22704
181.748	-0.65769	-0.22705
181.765	-0.65771	-0.22705
181.782	-0.65773	-0.22705
181.799	-0.65774	-0.22705
181.815	-0.65776	-0.22706
181.831	-0.65778	-0.22706
181.848	-0.6578	-0.22706
181.865	-0.65782	-0.22706
181.882	-0.65783	-0.22707
181.9	-0.65785	-0.22707
181.916	-0.65786	-0.22708
181.933	-0.65789	-0.22708
181.949	-0.65792	-0.22709
181.966	-0.65795	-0.22709
181.982	-0.65798	-0.2271
182	-0.65801	-0.2271
182.015	-0.65804	-0.2271
182.032	-0.65807	-0.2271
182.048	-0.6581	-0.22711
182.065	-0.65813	-0.22711
182.082	-0.65815	-0.22712

182.099	-0.65817	-0.22712
182.116	-0.65818	-0.22713
182.132	-0.65819	-0.22713
182.15	-0.65821	-0.22714
182.166	-0.65824	-0.22715
182.182	-0.65826	-0.22715
182.199	-0.65829	-0.22715
182.215	-0.65831	-0.22716
182.233	-0.65834	-0.22716
182.249	-0.65837	-0.22716
182.266	-0.65839	-0.22717
182.283	-0.65842	-0.22717
182.3	-0.65844	-0.22717
182.316	-0.65846	-0.22717
182.333	-0.65848	-0.22718
182.349	-0.6585	-0.22718
182.366	-0.65852	-0.22719
182.383	-0.65855	-0.22719
182.399	-0.65857	-0.2272
182.417	-0.6586	-0.22721
182.433	-0.65862	-0.22721
182.45	-0.65865	-0.22721
182.466	-0.65868	-0.22722
182.484	-0.6587	-0.22723
182.5	-0.65873	-0.22723
182.517	-0.65876	-0.22724
182.534	-0.65878	-0.22724
182.55	-0.65881	-0.22725
182.566	-0.65883	-0.22725
182.584	-0.65886	-0.22726

182.599	-0.65889	-0.22726
182.616	-0.65891	-0.22727
182.632	-0.65893	-0.22727
182.649	-0.65895	-0.22727
182.667	-0.65897	-0.22728
182.683	-0.65899	-0.22728
182.7	-0.65901	-0.22729
182.716	-0.65903	-0.22729
182.732	-0.65904	-0.2273
182.75	-0.65906	-0.2273
182.767	-0.65909	-0.22731
182.783	-0.65911	-0.22731
182.8	-0.65913	-0.22731
182.816	-0.65914	-0.22732
182.832	-0.65916	-0.22732
182.85	-0.65918	-0.22732
182.866	-0.6592	-0.22733
182.883	-0.65922	-0.22733
182.9	-0.65925	-0.22733
182.916	-0.65926	-0.22733
182.933	-0.65929	-0.22734
182.949	-0.65931	-0.22734
182.967	-0.65933	-0.22734
182.982	-0.65935	-0.22734
183	-0.65937	-0.22735
183.016	-0.65939	-0.22735
183.033	-0.65941	-0.22735
183.049	-0.65943	-0.22736
183.067	-0.65946	-0.22736
183.083	-0.65948	-0.22737

183.099	-0.6595	-0.22737
183.117	-0.65952	-0.22737
183.133	-0.65955	-0.22737
183.149	-0.65957	-0.22737
183.166	-0.65959	-0.22738
183.184	-0.65961	-0.22738
183.2	-0.65962	-0.22739
183.216	-0.65964	-0.22739
183.234	-0.65966	-0.2274
183.25	-0.65968	-0.2274
183.267	-0.6597	-0.2274
183.284	-0.65972	-0.2274
183.301	-0.65974	-0.2274
183.317	-0.65977	-0.2274
183.333	-0.65979	-0.2274
183.35	-0.65981	-0.2274
183.366	-0.65983	-0.22741
183.384	-0.65984	-0.22741
183.399	-0.65986	-0.22742
183.416	-0.65987	-0.22743
183.433	-0.65989	-0.22743
183.449	-0.65991	-0.22744
183.466	-0.65993	-0.22745
183.483	-0.65995	-0.22745
183.5	-0.65997	-0.22745
183.517	-0.66	-0.22745
183.533	-0.66002	-0.22745
183.55	-0.66005	-0.22746
183.567	-0.66008	-0.22746
183.583	-0.66011	-0.22746

183.6	-0.66014	-0.22746
183.617	-0.66016	-0.22746
183.632	-0.66019	-0.22746
183.65	-0.66022	-0.22746
183.667	-0.66024	-0.22747
183.683	-0.66026	-0.22747
183.7	-0.66029	-0.22747
183.717	-0.66031	-0.22747
183.733	-0.66032	-0.22748
183.75	-0.66034	-0.22748
183.766	-0.66036	-0.22749
183.783	-0.66038	-0.22749
183.8	-0.66041	-0.22749
183.816	-0.66043	-0.2275
183.833	-0.66046	-0.2275
183.85	-0.66049	-0.22751
183.867	-0.66053	-0.22751
183.883	-0.66057	-0.22752
183.9	-0.6606	-0.22752
183.917	-0.66064	-0.22753
183.933	-0.66067	-0.22753
183.951	-0.6607	-0.22754
183.967	-0.66073	-0.22754
183.983	-0.66075	-0.22755
184	-0.66078	-0.22755
184.016	-0.6608	-0.22755
184.033	-0.66082	-0.22756
184.049	-0.66083	-0.22756
184.066	-0.66086	-0.22756
184.083	-0.66089	-0.22756

184.1	-0.66092	-0.22757
184.117	-0.66094	-0.22757
184.133	-0.66097	-0.22758
184.151	-0.661	-0.22758
184.166	-0.66103	-0.22758
184.183	-0.66106	-0.22758
184.199	-0.66109	-0.22758
184.217	-0.66112	-0.22758
184.233	-0.66114	-0.22759
184.249	-0.66116	-0.22759
184.266	-0.66118	-0.2276
184.282	-0.6612	-0.2276
184.299	-0.66122	-0.22761
184.316	-0.66124	-0.22762
184.332	-0.66127	-0.22762
184.35	-0.6613	-0.22762
184.366	-0.66133	-0.22762
184.383	-0.66136	-0.22762
184.399	-0.6614	-0.22762
184.416	-0.66143	-0.22762
184.433	-0.66146	-0.22763
184.449	-0.66149	-0.22763
184.465	-0.66151	-0.22764
184.483	-0.66153	-0.22765
184.499	-0.66155	-0.22765
184.515	-0.66157	-0.22766
184.532	-0.66159	-0.22766
184.549	-0.6616	-0.22767
184.566	-0.66162	-0.22767
184.583	-0.66165	-0.22768

184.6	-0.66167	-0.22768
184.616	-0.6617	-0.22768
184.632	-0.66173	-0.22768
184.649	-0.66175	-0.22769
184.666	-0.66178	-0.22769
184.683	-0.66181	-0.22769
184.7	-0.66183	-0.22769
184.716	-0.66186	-0.22769
184.733	-0.66187	-0.22769
184.749	-0.66189	-0.2277
184.766	-0.66191	-0.2277
184.782	-0.66193	-0.22769
184.8	-0.66195	-0.22769
184.816	-0.66197	-0.2277
184.833	-0.66199	-0.2277
184.85	-0.66202	-0.22771
184.866	-0.66204	-0.22771
184.883	-0.66206	-0.22771
184.9	-0.66208	-0.22771
184.915	-0.6621	-0.22771
184.933	-0.66211	-0.22772
184.949	-0.66213	-0.22772
184.966	-0.66215	-0.22772
184.983	-0.66217	-0.22772
184.999	-0.66219	-0.22772
185.015	-0.66221	-0.22773
185.032	-0.66223	-0.22773
185.049	-0.66225	-0.22773
185.066	-0.66227	-0.22774
185.083	-0.66229	-0.22774

185.098	-0.66231	-0.22774
185.116	-0.66233	-0.22775
185.133	-0.66234	-0.22775
185.149	-0.66236	-0.22775
185.166	-0.66238	-0.22776
185.182	-0.6624	-0.22776
185.2	-0.66242	-0.22777
185.216	-0.66244	-0.22777
185.233	-0.66245	-0.22778
185.248	-0.66247	-0.22778
185.266	-0.66249	-0.22778
185.282	-0.66251	-0.22779
185.299	-0.66253	-0.22779
185.316	-0.66255	-0.22779
185.332	-0.66258	-0.2278
185.349	-0.6626	-0.22781
185.365	-0.66263	-0.22781
185.383	-0.66265	-0.22782
185.399	-0.66268	-0.22783
185.415	-0.66271	-0.22784
185.432	-0.66273	-0.22785
185.45	-0.66276	-0.22785
185.465	-0.66278	-0.22786
185.481	-0.6628	-0.22786
185.499	-0.66282	-0.22786
185.516	-0.66284	-0.22786
185.533	-0.66286	-0.22787
185.55	-0.66288	-0.22787
185.566	-0.66291	-0.22788
185.583	-0.66294	-0.22788

185.6	-0.66296	-0.22789
185.616	-0.663	-0.2279
185.633	-0.66303	-0.2279
185.649	-0.66307	-0.22791
185.666	-0.66309	-0.22792
185.682	-0.66312	-0.22792
185.699	-0.66314	-0.22793
185.716	-0.66317	-0.22793
185.732	-0.66319	-0.22794
185.75	-0.66321	-0.22794
185.766	-0.66324	-0.22795
185.783	-0.66326	-0.22795
185.799	-0.66328	-0.22796
185.816	-0.66331	-0.22796
185.833	-0.66334	-0.22796
185.85	-0.66337	-0.22797
185.866	-0.6634	-0.22797
185.883	-0.66343	-0.22798
185.9	-0.66346	-0.22799
185.916	-0.66349	-0.228
185.933	-0.66352	-0.22801
185.95	-0.66355	-0.22802
185.967	-0.66357	-0.22802
185.983	-0.66359	-0.22803
186	-0.66362	-0.22804
186.017	-0.66364	-0.22804
186.034	-0.66366	-0.22805
186.05	-0.66368	-0.22805
186.067	-0.66371	-0.22806
186.084	-0.66374	-0.22807

186.1	-0.66377	-0.22808
186.117	-0.6638	-0.22808
186.133	-0.66383	-0.22809
186.15	-0.66385	-0.2281
186.166	-0.66388	-0.2281
186.184	-0.66391	-0.22811
186.2	-0.66394	-0.22811
186.217	-0.66396	-0.22812
186.234	-0.66398	-0.22812
186.251	-0.664	-0.22812
186.268	-0.66402	-0.22813
186.285	-0.66404	-0.22813
186.3	-0.66406	-0.22813
186.317	-0.66409	-0.22814
186.334	-0.66411	-0.22814
186.351	-0.66414	-0.22815
186.367	-0.66417	-0.22816
186.384	-0.6642	-0.22817
186.4	-0.66423	-0.22817
186.418	-0.66424	-0.22817
186.434	-0.66427	-0.22818
186.451	-0.66429	-0.22818
186.467	-0.66431	-0.22818
186.484	-0.66433	-0.22818
186.5	-0.66436	-0.22818
186.517	-0.66438	-0.22818
186.534	-0.66441	-0.22819
186.551	-0.66443	-0.22819
186.568	-0.66446	-0.2282
186.584	-0.66448	-0.2282

186.601	-0.6645	-0.22821
186.618	-0.66453	-0.22821
186.634	-0.66455	-0.22821
186.651	-0.66457	-0.22821
186.666	-0.66458	-0.22821
186.684	-0.6646	-0.22821
186.699	-0.66462	-0.22821
186.717	-0.66464	-0.22821
186.734	-0.66466	-0.22821
186.75	-0.66468	-0.22821
186.767	-0.6647	-0.22822
186.784	-0.66472	-0.22822
186.8	-0.66474	-0.22822
186.817	-0.66476	-0.22822
186.833	-0.66478	-0.22823
186.85	-0.6648	-0.22823
186.867	-0.66482	-0.22823
186.884	-0.66485	-0.22823
186.901	-0.66487	-0.22824
186.917	-0.66489	-0.22824
186.934	-0.66491	-0.22825
186.951	-0.66493	-0.22825
186.967	-0.66494	-0.22826
186.983	-0.66496	-0.22826
187	-0.66497	-0.22826
187.016	-0.66499	-0.22826
187.033	-0.665	-0.22826
187.05	-0.66501	-0.22827
187.066	-0.66502	-0.22827
187.084	-0.66504	-0.22828

187.1	-0.66506	-0.22828
187.117	-0.66507	-0.22829
187.133	-0.66509	-0.22829
187.15	-0.66511	-0.2283
187.167	-0.66513	-0.2283
187.183	-0.66514	-0.22831
187.2	-0.66516	-0.22831
187.216	-0.66518	-0.22832
187.233	-0.6652	-0.22832
187.25	-0.66522	-0.22832
187.266	-0.66524	-0.22832
187.283	-0.66526	-0.22833
187.3	-0.66528	-0.22833
187.316	-0.6653	-0.22833
187.334	-0.66532	-0.22834
187.349	-0.66534	-0.22834
187.366	-0.66535	-0.22835
187.384	-0.66537	-0.22835
187.4	-0.66539	-0.22835
187.417	-0.66541	-0.22835
187.433	-0.66542	-0.22835
187.45	-0.66544	-0.22835
187.466	-0.66545	-0.22835
187.483	-0.66547	-0.22835
187.5	-0.6655	-0.22835
187.516	-0.66553	-0.22835
187.532	-0.66555	-0.22836
187.55	-0.66558	-0.22836
187.566	-0.66561	-0.22836
187.583	-0.66564	-0.22837

187.599	-0.66566	-0.22837
187.617	-0.66569	-0.22837
187.633	-0.66571	-0.22838
187.65	-0.66573	-0.22838
187.667	-0.66575	-0.22839
187.683	-0.66577	-0.2284
187.699	-0.6658	-0.22841
187.716	-0.66582	-0.22842
187.733	-0.66584	-0.22843
187.749	-0.66586	-0.22844
187.767	-0.66588	-0.22844
187.784	-0.6659	-0.22845
187.8	-0.66592	-0.22845
187.817	-0.66594	-0.22845
187.833	-0.66596	-0.22846
187.851	-0.66599	-0.22846
187.867	-0.66601	-0.22847
187.884	-0.66604	-0.22847
187.901	-0.66606	-0.22848
187.917	-0.66609	-0.22848
187.934	-0.66612	-0.22849
187.95	-0.66615	-0.22849
187.967	-0.66617	-0.2285
187.984	-0.6662	-0.22851
188.001	-0.66622	-0.22851
188.018	-0.66625	-0.22852
188.034	-0.66627	-0.22853
188.051	-0.66629	-0.22854
188.068	-0.66631	-0.22854
188.085	-0.66633	-0.22855

188.101	-0.66635	-0.22856
188.118	-0.66638	-0.22856
188.134	-0.6664	-0.22857
188.151	-0.66643	-0.22857
188.168	-0.66647	-0.22857
188.185	-0.6665	-0.22857
188.201	-0.66653	-0.22857
188.217	-0.66656	-0.22858
188.234	-0.6666	-0.22858
188.252	-0.66662	-0.22858
188.268	-0.66665	-0.22859
188.284	-0.66667	-0.22859
188.301	-0.66669	-0.22859
188.317	-0.66671	-0.2286
188.334	-0.66673	-0.2286
188.351	-0.66675	-0.2286
188.368	-0.66677	-0.2286
188.384	-0.66678	-0.2286
188.4	-0.6668	-0.22861
188.417	-0.66682	-0.22861
188.435	-0.66683	-0.22862
188.451	-0.66685	-0.22862
188.468	-0.66687	-0.22862
188.483	-0.66689	-0.22863
188.5	-0.6669	-0.22863
188.517	-0.66692	-0.22863
188.534	-0.66694	-0.22863
188.55	-0.66696	-0.22863
188.567	-0.66699	-0.22864
188.584	-0.66701	-0.22864

188.6	-0.66703	-0.22864
188.617	-0.66705	-0.22864
188.633	-0.66707	-0.22865
188.651	-0.66709	-0.22865
188.667	-0.66712	-0.22865
188.684	-0.66715	-0.22865
188.7	-0.66717	-0.22866
188.717	-0.66719	-0.22866
188.733	-0.66722	-0.22866
188.751	-0.66724	-0.22866
188.767	-0.66726	-0.22867
188.784	-0.66728	-0.22867
188.801	-0.6673	-0.22867
188.818	-0.66732	-0.22868
188.834	-0.66734	-0.22867
188.85	-0.66736	-0.22867
188.868	-0.66738	-0.22867
188.883	-0.6674	-0.22867
188.9	-0.66741	-0.22867
188.916	-0.66743	-0.22867
188.934	-0.66745	-0.22867
188.951	-0.66747	-0.22867
188.967	-0.66748	-0.22868
188.984	-0.6675	-0.22869
189.001	-0.66753	-0.22869
189.017	-0.66755	-0.2287
189.034	-0.66758	-0.2287
189.051	-0.66761	-0.2287
189.067	-0.66764	-0.2287
189.084	-0.66766	-0.2287

189.099	-0.66769	-0.2287
189.117	-0.6677	-0.2287
189.133	-0.66772	-0.2287
189.15	-0.66774	-0.22871
189.166	-0.66776	-0.22871
189.183	-0.66778	-0.22871
189.201	-0.66779	-0.22872
189.217	-0.66781	-0.22872
189.234	-0.66783	-0.22873
189.25	-0.66786	-0.22873
189.267	-0.66789	-0.22874
189.284	-0.66791	-0.22874
189.301	-0.66793	-0.22874
189.318	-0.66796	-0.22874
189.334	-0.66798	-0.22874
189.352	-0.668	-0.22874
189.368	-0.66802	-0.22874
189.384	-0.66804	-0.22875
189.4	-0.66805	-0.22875
189.417	-0.66807	-0.22876
189.434	-0.66808	-0.22877
189.451	-0.6681	-0.22877
189.467	-0.66812	-0.22877
189.482	-0.66814	-0.22878
189.501	-0.66816	-0.22878
189.518	-0.66818	-0.22878
189.534	-0.66821	-0.22878
189.55	-0.66823	-0.22878
189.567	-0.66825	-0.22878
189.583	-0.66828	-0.22879

189.599	-0.66831	-0.22879
189.616	-0.66833	-0.22879
189.633	-0.66836	-0.22879
189.649	-0.66838	-0.22879
189.666	-0.6684	-0.22879
189.682	-0.66842	-0.22879
189.699	-0.66844	-0.22879
189.716	-0.66846	-0.2288
189.733	-0.66848	-0.2288
189.749	-0.6685	-0.22881
189.766	-0.66852	-0.22881
189.783	-0.66854	-0.22882
189.8	-0.66856	-0.22882
189.816	-0.66858	-0.22883
189.833	-0.6686	-0.22883
189.85	-0.66862	-0.22883
189.866	-0.66864	-0.22883
189.883	-0.66865	-0.22884
189.899	-0.66867	-0.22884
189.916	-0.66869	-0.22884
189.933	-0.6687	-0.22884
189.95	-0.66872	-0.22884
189.966	-0.66874	-0.22884
189.981	-0.66875	-0.22884
189.999	-0.66877	-0.22884
190.016	-0.66879	-0.22885
190.032	-0.6688	-0.22885
190.05	-0.66883	-0.22885
190.066	-0.66885	-0.22886
190.083	-0.66887	-0.22886

190.099	-0.66889	-0.22887
190.116	-0.66891	-0.22887
190.133	-0.66893	-0.22888
190.149	-0.66895	-0.22888
190.166	-0.66896	-0.22889
190.183	-0.66897	-0.22889
190.199	-0.66898	-0.2289
190.216	-0.669	-0.2289
190.233	-0.66902	-0.22891
190.249	-0.66904	-0.22892
190.266	-0.66905	-0.22892
190.283	-0.66907	-0.22892
190.299	-0.66909	-0.22892
190.316	-0.66911	-0.22893
190.332	-0.66913	-0.22893
190.35	-0.66915	-0.22893
190.366	-0.66917	-0.22894
190.382	-0.66919	-0.22894
190.399	-0.6692	-0.22895
190.416	-0.66922	-0.22895
190.433	-0.66923	-0.22896
190.449	-0.66925	-0.22896
190.466	-0.66927	-0.22897
190.483	-0.66929	-0.22898
190.499	-0.66931	-0.22899
190.517	-0.66933	-0.229
190.533	-0.66935	-0.22901
190.55	-0.66938	-0.22901
190.566	-0.66941	-0.22902
190.583	-0.66943	-0.22902

190.6	-0.66945	-0.22903
190.617	-0.66947	-0.22903
190.634	-0.66949	-0.22903
190.65	-0.6695	-0.22904
190.667	-0.66951	-0.22905
190.683	-0.66953	-0.22906
190.7	-0.66954	-0.22907
190.717	-0.66956	-0.22908
190.734	-0.66958	-0.22908
190.751	-0.66961	-0.22909
190.766	-0.66964	-0.2291
190.784	-0.66967	-0.2291
190.8	-0.66971	-0.22911
190.817	-0.66974	-0.22912
190.834	-0.66977	-0.22912
190.85	-0.66979	-0.22913
190.867	-0.66982	-0.22914
190.884	-0.66984	-0.22915
190.901	-0.66987	-0.22915
190.918	-0.66989	-0.22916
190.934	-0.66992	-0.22916
190.951	-0.66995	-0.22917
190.968	-0.66998	-0.22918
190.984	-0.67001	-0.22919
191.001	-0.67004	-0.2292
191.018	-0.67007	-0.22921
191.035	-0.6701	-0.22922
191.052	-0.67014	-0.22923
191.068	-0.67017	-0.22924
191.084	-0.67021	-0.22924

191.102	-0.67024	-0.22925
191.118	-0.67027	-0.22926
191.135	-0.6703	-0.22926
191.152	-0.67033	-0.22927
191.168	-0.67037	-0.22927
191.184	-0.67041	-0.22927
191.201	-0.67044	-0.22927
191.217	-0.67048	-0.22928
191.235	-0.67052	-0.22928
191.252	-0.67056	-0.22929
191.268	-0.6706	-0.22929
191.285	-0.67063	-0.2293
191.302	-0.67067	-0.22931
191.318	-0.6707	-0.22932
191.336	-0.67074	-0.22933
191.352	-0.67078	-0.22933
191.369	-0.67082	-0.22934
191.385	-0.67085	-0.22934
191.401	-0.67088	-0.22935
191.418	-0.67092	-0.22936
191.435	-0.67095	-0.22936
191.452	-0.67098	-0.22936
191.468	-0.67102	-0.22937
191.485	-0.67106	-0.22937
191.502	-0.6711	-0.22938
191.519	-0.67114	-0.22939
191.535	-0.67118	-0.22939
191.552	-0.67123	-0.2294
191.569	-0.67127	-0.2294
191.585	-0.67131	-0.2294

191.603	-0.67134	-0.22941
191.618	-0.67137	-0.22941
191.636	-0.67141	-0.22941
191.653	-0.67144	-0.22942
191.668	-0.67146	-0.22942
191.685	-0.6715	-0.22942
191.702	-0.67153	-0.22943
191.719	-0.67157	-0.22943
191.735	-0.67161	-0.22943
191.752	-0.67165	-0.22944
191.768	-0.67168	-0.22944
191.785	-0.67172	-0.22944
191.802	-0.67175	-0.22944
191.819	-0.67177	-0.22944
191.835	-0.6718	-0.22944
191.852	-0.67183	-0.22945
191.869	-0.67186	-0.22945
191.885	-0.67189	-0.22945
191.902	-0.67192	-0.22946
191.919	-0.67195	-0.22946
191.935	-0.67199	-0.22947
191.952	-0.67201	-0.22947
191.968	-0.67204	-0.22947
191.986	-0.67206	-0.22948
192.002	-0.67208	-0.22948
192.018	-0.6721	-0.22948
192.036	-0.67213	-0.22949
192.052	-0.67215	-0.22949
192.069	-0.67217	-0.22949
192.084	-0.6722	-0.22949

192.102	-0.67223	-0.22949
192.118	-0.67227	-0.22948
192.135	-0.6723	-0.22948
192.152	-0.67233	-0.22948
192.168	-0.67236	-0.22948
192.185	-0.67238	-0.22948
192.202	-0.67241	-0.22948
192.219	-0.67244	-0.22949
192.235	-0.67246	-0.22949
192.252	-0.67248	-0.22949
192.268	-0.67249	-0.2295
192.285	-0.67251	-0.2295
192.302	-0.67253	-0.2295
192.319	-0.67255	-0.2295
192.335	-0.67257	-0.2295
192.351	-0.67259	-0.2295
192.368	-0.67261	-0.2295
192.384	-0.67263	-0.2295
192.402	-0.67264	-0.22951
192.419	-0.67266	-0.22951
192.435	-0.67267	-0.22951
192.452	-0.67269	-0.22951
192.468	-0.6727	-0.22952
192.484	-0.67271	-0.22952
192.502	-0.67272	-0.22952
192.519	-0.67274	-0.22952
192.535	-0.67276	-0.22952
192.552	-0.67278	-0.22952
192.569	-0.6728	-0.22952
192.585	-0.67281	-0.22952

192.602	-0.67283	-0.22952
192.618	-0.67285	-0.22952
192.635	-0.67286	-0.22952
192.651	-0.67288	-0.22952
192.667	-0.67289	-0.22952
192.684	-0.67291	-0.22952
192.701	-0.67292	-0.22952
192.719	-0.67293	-0.22952
192.735	-0.67295	-0.22953
192.75	-0.67296	-0.22953
192.768	-0.67296	-0.22953
192.784	-0.67297	-0.22953
192.801	-0.67298	-0.22954
192.818	-0.67299	-0.22954
192.835	-0.673	-0.22954
192.852	-0.673	-0.22954
192.868	-0.67301	-0.22954
192.885	-0.67302	-0.22954
192.902	-0.67303	-0.22954
192.918	-0.67304	-0.22955
192.935	-0.67306	-0.22955
192.951	-0.67307	-0.22956
192.968	-0.67309	-0.22956
192.985	-0.6731	-0.22957
193.001	-0.67312	-0.22957
193.018	-0.67314	-0.22958
193.035	-0.67316	-0.22958
193.051	-0.67317	-0.22958
193.068	-0.67319	-0.22958
193.085	-0.67319	-0.22959

193.102	-0.6732	-0.22959
193.118	-0.67321	-0.22959
193.135	-0.67321	-0.22959
193.151	-0.67322	-0.2296
193.168	-0.67324	-0.2296
193.185	-0.67326	-0.2296
193.201	-0.67328	-0.2296
193.219	-0.6733	-0.2296
193.234	-0.67333	-0.2296
193.252	-0.67337	-0.22961
193.268	-0.6734	-0.22961
193.285	-0.67342	-0.22962
193.303	-0.67345	-0.22963
193.319	-0.67347	-0.22963
193.335	-0.6735	-0.22964
193.352	-0.67352	-0.22964
193.368	-0.67353	-0.22964
193.384	-0.67355	-0.22964
193.402	-0.67357	-0.22964
193.417	-0.6736	-0.22965
193.434	-0.67362	-0.22965
193.451	-0.67364	-0.22965
193.468	-0.67367	-0.22966
193.485	-0.6737	-0.22967
193.502	-0.67373	-0.22968
193.519	-0.67375	-0.22969
193.535	-0.67377	-0.22969
193.551	-0.6738	-0.2297
193.568	-0.67382	-0.2297
193.584	-0.67384	-0.22971

193.602	-0.67387	-0.22971
193.618	-0.6739	-0.22971
193.635	-0.67392	-0.22972
193.652	-0.67395	-0.22972
193.669	-0.67398	-0.22972
193.685	-0.67402	-0.22973
193.702	-0.67405	-0.22973
193.719	-0.67407	-0.22974
193.735	-0.6741	-0.22974
193.751	-0.67413	-0.22974
193.768	-0.67415	-0.22974
193.785	-0.67418	-0.22974
193.801	-0.67421	-0.22975
193.818	-0.67424	-0.22975
193.834	-0.67427	-0.22976
193.851	-0.67429	-0.22977
193.868	-0.67432	-0.22978
193.885	-0.67434	-0.22978
193.902	-0.67436	-0.22979
193.918	-0.67439	-0.22979
193.935	-0.67442	-0.22979
193.951	-0.67445	-0.22979
193.969	-0.67448	-0.22979
193.984	-0.67451	-0.22979
194.001	-0.67454	-0.22979
194.018	-0.67456	-0.22979
194.035	-0.67458	-0.2298
194.052	-0.67461	-0.2298
194.068	-0.67462	-0.22981
194.085	-0.67464	-0.22981

194.101	-0.67466	-0.22982
194.118	-0.67469	-0.22982
194.134	-0.67471	-0.22983
194.151	-0.67475	-0.22983
194.168	-0.67478	-0.22984
194.184	-0.67482	-0.22984
194.201	-0.67485	-0.22984
194.218	-0.67488	-0.22985
194.234	-0.67492	-0.22985
194.251	-0.67494	-0.22985
194.268	-0.67497	-0.22985
194.285	-0.675	-0.22985
194.301	-0.67503	-0.22985
194.317	-0.67505	-0.22986
194.335	-0.67508	-0.22986
194.35	-0.6751	-0.22986
194.367	-0.67512	-0.22986
194.384	-0.67514	-0.22986
194.4	-0.67516	-0.22987
194.417	-0.67518	-0.22987
194.434	-0.67521	-0.22988
194.45	-0.67523	-0.22989
194.467	-0.67527	-0.2299
194.483	-0.67529	-0.2299
194.501	-0.67532	-0.22991
194.518	-0.67535	-0.22992
194.535	-0.67537	-0.22993
194.551	-0.6754	-0.22993
194.568	-0.67542	-0.22993
194.585	-0.67544	-0.22993

194.601	-0.67547	-0.22993
194.617	-0.67549	-0.22993
194.635	-0.67551	-0.22994
194.651	-0.67554	-0.22994
194.668	-0.67556	-0.22994
194.684	-0.67558	-0.22995
194.701	-0.6756	-0.22996
194.718	-0.67563	-0.22997
194.735	-0.67566	-0.22997
194.751	-0.67568	-0.22998
194.768	-0.67571	-0.22998
194.784	-0.67574	-0.22999
194.802	-0.67577	-0.22999
194.818	-0.6758	-0.22999
194.835	-0.67583	-0.23
194.852	-0.67587	-0.23
194.869	-0.6759	-0.23001
194.885	-0.67593	-0.23002
194.901	-0.67596	-0.23002
194.918	-0.67599	-0.23003
194.935	-0.67602	-0.23004
194.952	-0.67605	-0.23005
194.968	-0.67609	-0.23006
194.985	-0.67612	-0.23007
195.002	-0.67616	-0.23008
195.019	-0.67619	-0.23009
195.036	-0.67623	-0.2301
195.052	-0.67627	-0.2301
195.068	-0.67631	-0.23011
195.085	-0.67634	-0.23011

195.102	-0.67637	-0.23012
195.119	-0.6764	-0.23012
195.135	-0.67643	-0.23013
195.153	-0.67646	-0.23013
195.167	-0.67649	-0.23014
195.185	-0.67652	-0.23015
195.202	-0.67655	-0.23016
195.22	-0.67658	-0.23017
195.235	-0.67661	-0.23018
195.253	-0.67665	-0.23019
195.269	-0.67668	-0.2302
195.286	-0.67671	-0.2302
195.302	-0.67675	-0.23021
195.319	-0.67678	-0.23021
195.335	-0.67681	-0.23022
195.352	-0.67685	-0.23022
195.369	-0.67688	-0.23023
195.386	-0.67691	-0.23023
195.403	-0.67695	-0.23024
195.419	-0.67698	-0.23025
195.436	-0.67701	-0.23026
195.453	-0.67705	-0.23027
195.469	-0.67708	-0.23027
195.486	-0.67712	-0.23028
195.503	-0.67715	-0.23029
195.519	-0.67719	-0.2303
195.535	-0.67723	-0.2303
195.553	-0.67727	-0.23031
195.569	-0.6773	-0.23032
195.586	-0.67733	-0.23032

195.602	-0.67737	-0.23033
195.619	-0.6774	-0.23033
195.636	-0.67743	-0.23034
195.653	-0.67746	-0.23034
195.67	-0.67749	-0.23035
195.686	-0.67751	-0.23035
195.703	-0.67754	-0.23036
195.72	-0.67757	-0.23037
195.736	-0.6776	-0.23037
195.754	-0.67763	-0.23037
195.77	-0.67766	-0.23038
195.786	-0.67769	-0.23038
195.802	-0.67773	-0.23039
195.819	-0.67776	-0.23039
195.836	-0.6778	-0.23039
195.852	-0.67783	-0.23039
195.869	-0.67786	-0.2304
195.886	-0.6779	-0.2304
195.903	-0.67793	-0.23041
195.92	-0.67796	-0.23041
195.937	-0.67799	-0.23042
195.952	-0.67802	-0.23043
195.97	-0.67805	-0.23043
195.986	-0.67807	-0.23044
196.003	-0.6781	-0.23044
196.019	-0.67813	-0.23045
196.036	-0.67816	-0.23046
196.052	-0.67819	-0.23046
196.07	-0.67822	-0.23047
196.086	-0.67824	-0.23047

196.102	-0.67827	-0.23047
196.12	-0.6783	-0.23048
196.137	-0.67832	-0.23048
196.153	-0.67835	-0.23048
196.169	-0.67838	-0.23048
196.186	-0.67841	-0.23049
196.202	-0.67843	-0.23049
196.219	-0.67846	-0.2305
196.235	-0.67849	-0.23051
196.252	-0.67852	-0.23051
196.268	-0.67854	-0.23052
196.286	-0.67857	-0.23053
196.303	-0.6786	-0.23053
196.319	-0.67863	-0.23054
196.336	-0.67866	-0.23054
196.352	-0.6787	-0.23054
196.368	-0.67873	-0.23055
196.387	-0.67877	-0.23055
196.402	-0.6788	-0.23055
196.419	-0.67883	-0.23055
196.435	-0.67885	-0.23055
196.453	-0.67887	-0.23056
196.47	-0.67889	-0.23056
196.486	-0.67891	-0.23056
196.503	-0.67893	-0.23057
196.519	-0.67896	-0.23057
196.536	-0.67899	-0.23058
196.552	-0.67902	-0.23058
196.568	-0.67905	-0.23058
196.586	-0.67908	-0.23059

196.603	-0.6791	-0.23059
196.619	-0.67913	-0.23059
196.636	-0.67915	-0.2306
196.652	-0.67917	-0.2306
196.668	-0.67919	-0.23061
196.685	-0.67921	-0.23061
196.703	-0.67923	-0.23062
196.719	-0.67925	-0.23062
196.736	-0.67926	-0.23063
196.752	-0.67928	-0.23063
196.769	-0.67929	-0.23063
196.785	-0.67931	-0.23063
196.802	-0.67933	-0.23063
196.818	-0.67936	-0.23063
196.835	-0.67939	-0.23064
196.851	-0.67942	-0.23064
196.868	-0.67946	-0.23064
196.885	-0.67949	-0.23065
196.901	-0.67952	-0.23065
196.919	-0.67956	-0.23066
196.935	-0.67958	-0.23066
196.952	-0.67961	-0.23067
196.969	-0.67964	-0.23068
196.986	-0.67967	-0.23069
197.002	-0.67969	-0.2307
197.018	-0.67971	-0.23071
197.035	-0.67974	-0.23072
197.052	-0.67975	-0.23072
197.069	-0.67977	-0.23073
197.084	-0.6798	-0.23074

197.102	-0.67982	-0.23075
197.118	-0.67984	-0.23075
197.135	-0.67986	-0.23076
197.151	-0.67989	-0.23076
197.169	-0.67992	-0.23076
197.184	-0.67995	-0.23077
197.201	-0.67998	-0.23077
197.218	-0.68001	-0.23078
197.235	-0.68003	-0.23078
197.25	-0.68006	-0.23078
197.268	-0.68008	-0.23079
197.284	-0.6801	-0.23079
197.301	-0.68012	-0.23079
197.318	-0.68015	-0.2308
197.335	-0.68017	-0.23081
197.352	-0.68019	-0.23081
197.368	-0.68022	-0.23082
197.386	-0.68024	-0.23083
197.402	-0.68026	-0.23084
197.418	-0.68029	-0.23084
197.434	-0.68031	-0.23085
197.451	-0.68034	-0.23085
197.468	-0.68037	-0.23086
197.485	-0.6804	-0.23087
197.501	-0.68043	-0.23087
197.518	-0.68047	-0.23088
197.534	-0.68051	-0.23089
197.551	-0.68054	-0.2309
197.568	-0.68058	-0.23091
197.585	-0.68061	-0.23091

197.602	-0.68064	-0.23092
197.618	-0.68066	-0.23093
197.634	-0.68068	-0.23094
197.651	-0.6807	-0.23095
197.668	-0.68072	-0.23095
197.685	-0.68073	-0.23096
197.701	-0.68076	-0.23097
197.718	-0.68078	-0.23097
197.735	-0.68081	-0.23097
197.751	-0.68084	-0.23098
197.768	-0.68087	-0.23098
197.785	-0.6809	-0.23099
197.801	-0.68093	-0.23099
197.817	-0.68096	-0.231
197.835	-0.681	-0.23101
197.851	-0.68103	-0.23102
197.869	-0.68106	-0.23104
197.885	-0.68108	-0.23105
197.903	-0.68111	-0.23106
197.918	-0.68113	-0.23107
197.935	-0.68115	-0.23108
197.952	-0.68118	-0.23109
197.969	-0.68121	-0.2311
197.985	-0.68124	-0.2311
198.002	-0.68127	-0.23111
198.018	-0.6813	-0.23111
198.036	-0.68134	-0.23112
198.051	-0.68138	-0.23112
198.068	-0.68141	-0.23113
198.085	-0.68144	-0.23113

198.102	-0.68147	-0.23114
198.118	-0.6815	-0.23114
198.136	-0.68153	-0.23115
198.152	-0.68156	-0.23116
198.168	-0.68159	-0.23117
198.185	-0.68162	-0.23118
198.202	-0.68165	-0.23119
198.219	-0.68167	-0.2312
198.236	-0.6817	-0.23121
198.253	-0.68173	-0.23122
198.268	-0.68176	-0.23123
198.286	-0.68178	-0.23123
198.301	-0.68181	-0.23124
198.319	-0.68183	-0.23124
198.335	-0.68185	-0.23124
198.351	-0.68188	-0.23125
198.369	-0.6819	-0.23126
198.385	-0.68193	-0.23126
198.402	-0.68195	-0.23127
198.419	-0.68198	-0.23127
198.435	-0.682	-0.23128
198.452	-0.68203	-0.23129
198.468	-0.68206	-0.23129
198.485	-0.68209	-0.2313
198.502	-0.68212	-0.23131
198.519	-0.68215	-0.23132
198.535	-0.68218	-0.23132
198.552	-0.68222	-0.23133
198.569	-0.68225	-0.23133
198.585	-0.68228	-0.23134

198.601	-0.68231	-0.23135
198.617	-0.68234	-0.23135
198.636	-0.68237	-0.23136
198.651	-0.68241	-0.23137
198.667	-0.68244	-0.23137
198.684	-0.68247	-0.23138
198.701	-0.68251	-0.23139
198.718	-0.68254	-0.2314
198.734	-0.68257	-0.23141
198.752	-0.68261	-0.23142
198.768	-0.68264	-0.23142
198.785	-0.68267	-0.23143
198.801	-0.68271	-0.23144
198.817	-0.68274	-0.23145
198.834	-0.68276	-0.23146
198.852	-0.68279	-0.23147
198.868	-0.68281	-0.23147
198.885	-0.68284	-0.23148
198.901	-0.68287	-0.23149
198.918	-0.68289	-0.23149
198.934	-0.68292	-0.2315
198.952	-0.68295	-0.23151
198.967	-0.68298	-0.23151
198.985	-0.68302	-0.23152
199.001	-0.68304	-0.23153
199.018	-0.68307	-0.23154
199.035	-0.68309	-0.23154
199.051	-0.68312	-0.23155
199.068	-0.68315	-0.23156
199.084	-0.68318	-0.23156

199.102	-0.6832	-0.23157
199.118	-0.68323	-0.23158
199.134	-0.68326	-0.23158
199.151	-0.68329	-0.23159
199.168	-0.68332	-0.2316
199.184	-0.68335	-0.2316
199.201	-0.68338	-0.23161
199.218	-0.68342	-0.23162
199.236	-0.68346	-0.23163
199.251	-0.68349	-0.23163
199.269	-0.68353	-0.23164
199.285	-0.68356	-0.23165
199.302	-0.68359	-0.23166
199.318	-0.68361	-0.23167
199.335	-0.68364	-0.23168
199.351	-0.68366	-0.23169
199.367	-0.68368	-0.2317
199.384	-0.6837	-0.23171
199.401	-0.68372	-0.23172
199.418	-0.68375	-0.23173
199.434	-0.68378	-0.23174
199.451	-0.68381	-0.23174
199.468	-0.68383	-0.23175
199.484	-0.68386	-0.23176
199.501	-0.68389	-0.23177
199.518	-0.68392	-0.23178
199.534	-0.68394	-0.2318
199.552	-0.68397	-0.2318
199.568	-0.68399	-0.23181
199.585	-0.68402	-0.23182

199.601	-0.68405	-0.23183
199.618	-0.68408	-0.23183
199.634	-0.68411	-0.23184
199.651	-0.68415	-0.23185
199.668	-0.68418	-0.23185
199.685	-0.68421	-0.23186
199.701	-0.68424	-0.23187
199.718	-0.68427	-0.23188
199.734	-0.6843	-0.23189
199.751	-0.68434	-0.23191
199.768	-0.68437	-0.23192
199.785	-0.68439	-0.23193
199.801	-0.68441	-0.23193
199.818	-0.68443	-0.23194
199.835	-0.68445	-0.23195
199.85	-0.68446	-0.23196
199.868	-0.68448	-0.23197
199.884	-0.6845	-0.23198
199.901	-0.68452	-0.23199
199.918	-0.68455	-0.232
199.935	-0.68458	-0.23201
199.952	-0.68461	-0.23203
199.968	-0.68465	-0.23204
199.985	-0.68468	-0.23205
200.001	-0.68472	-0.23206
200.018	-0.68476	-0.23206
200.035	-0.68479	-0.23207
200.052	-0.68482	-0.23208
200.068	-0.68485	-0.23209
200.084	-0.68489	-0.2321

200.102	-0.68492	-0.23211
200.118	-0.68495	-0.23212
200.134	-0.68498	-0.23214
200.151	-0.68501	-0.23215
200.168	-0.68504	-0.23216
200.185	-0.68507	-0.23218
200.202	-0.68509	-0.23219
200.218	-0.68512	-0.2322
200.234	-0.68515	-0.23221
200.251	-0.68517	-0.23222
200.267	-0.6852	-0.23223
200.284	-0.68522	-0.23224
200.301	-0.68525	-0.23225
200.318	-0.68529	-0.23226
200.335	-0.68532	-0.23227
200.352	-0.68535	-0.23229
200.368	-0.68539	-0.2323
200.384	-0.68542	-0.23232
200.401	-0.68546	-0.23233
200.417	-0.6855	-0.23235
200.435	-0.68553	-0.23236
200.452	-0.68557	-0.23237
200.468	-0.6856	-0.23238
200.485	-0.68563	-0.23239
200.502	-0.68566	-0.2324
200.518	-0.68569	-0.23241
200.535	-0.68572	-0.23242
200.552	-0.68575	-0.23243
200.569	-0.68578	-0.23245
200.585	-0.68581	-0.23246

200.601	-0.68584	-0.23247
200.618	-0.68587	-0.23248
200.635	-0.6859	-0.23249
200.651	-0.68594	-0.2325
200.669	-0.68597	-0.23252
200.685	-0.686	-0.23253
200.702	-0.68603	-0.23255
200.718	-0.68605	-0.23256
200.735	-0.68608	-0.23258
200.752	-0.68611	-0.23259
200.768	-0.68614	-0.23261
200.786	-0.68617	-0.23262
200.801	-0.68621	-0.23263
200.818	-0.68624	-0.23264
200.835	-0.68627	-0.23265
200.852	-0.68631	-0.23266
200.868	-0.68634	-0.23267
200.885	-0.68637	-0.23269
200.902	-0.6864	-0.2327
200.919	-0.68643	-0.23271
200.935	-0.68646	-0.23272
200.952	-0.68649	-0.23273
200.968	-0.68652	-0.23274
200.985	-0.68656	-0.23276
201.001	-0.68659	-0.23277
201.018	-0.68662	-0.23278
201.035	-0.68666	-0.2328
201.052	-0.68669	-0.23281
201.068	-0.68672	-0.23283
201.085	-0.68675	-0.23284

201.103	-0.68678	-0.23286
201.119	-0.6868	-0.23287
201.136	-0.68683	-0.23288
201.152	-0.68686	-0.23289
201.168	-0.68689	-0.23289
201.185	-0.68692	-0.2329
201.201	-0.68695	-0.23291
201.218	-0.68697	-0.23292
201.235	-0.687	-0.23293
201.252	-0.68702	-0.23294
201.269	-0.68705	-0.23296
201.285	-0.68708	-0.23297
201.301	-0.68712	-0.23299
201.319	-0.68715	-0.23301
201.335	-0.68718	-0.23303
201.352	-0.68721	-0.23304
201.368	-0.68724	-0.23305
201.385	-0.68727	-0.23307
201.402	-0.68729	-0.23308
201.418	-0.68731	-0.23309
201.435	-0.68733	-0.2331
201.451	-0.68735	-0.23311
201.468	-0.68737	-0.23312
201.486	-0.6874	-0.23312
201.501	-0.68742	-0.23313
201.518	-0.68745	-0.23314
201.535	-0.68748	-0.23315
201.551	-0.68751	-0.23316
201.568	-0.68754	-0.23317
201.585	-0.68757	-0.23319

201.602	-0.6876	-0.2332
201.618	-0.68762	-0.23322
201.635	-0.68765	-0.23323
201.652	-0.68768	-0.23325
201.668	-0.6877	-0.23326
201.685	-0.68773	-0.23327
201.701	-0.68776	-0.23329
201.718	-0.68778	-0.2333
201.734	-0.68781	-0.23331
201.751	-0.68784	-0.23333
201.768	-0.68787	-0.23334
201.785	-0.6879	-0.23335
201.802	-0.68793	-0.23336
201.817	-0.68795	-0.23337
201.835	-0.68798	-0.23338
201.851	-0.68801	-0.23339
201.868	-0.68805	-0.2334
201.884	-0.68808	-0.23342
201.901	-0.68811	-0.23343
201.918	-0.68814	-0.23345
201.935	-0.68816	-0.23347
201.952	-0.68819	-0.23349
201.969	-0.68821	-0.23351
201.985	-0.68823	-0.23352
202.001	-0.68825	-0.23354
202.017	-0.68828	-0.23355
202.034	-0.68831	-0.23357
202.05	-0.68833	-0.23358
202.068	-0.68835	-0.23359
202.084	-0.68838	-0.2336

202.101	-0.68841	-0.23362
202.118	-0.68843	-0.23363
202.134	-0.68846	-0.23365
202.152	-0.68849	-0.23366
202.168	-0.68851	-0.23367
202.185	-0.68854	-0.23369
202.201	-0.68856	-0.2337
202.218	-0.68859	-0.23371
202.234	-0.68862	-0.23372
202.251	-0.68864	-0.23374
202.268	-0.68867	-0.23375
202.284	-0.68869	-0.23377
202.301	-0.68871	-0.23379
202.318	-0.68874	-0.2338
202.334	-0.68877	-0.23382
202.351	-0.6888	-0.23384
202.368	-0.68883	-0.23385
202.384	-0.68885	-0.23387
202.401	-0.68888	-0.23388
202.417	-0.6889	-0.2339
202.434	-0.68893	-0.23391
202.45	-0.68895	-0.23392
202.467	-0.68897	-0.23394
202.484	-0.68899	-0.23396
202.501	-0.68901	-0.23397
202.517	-0.68904	-0.23399
202.534	-0.68907	-0.23401
202.551	-0.6891	-0.23403
202.567	-0.68913	-0.23406
202.584	-0.68918	-0.23408

202.6	-0.68922	-0.2341
202.618	-0.68926	-0.23412
202.634	-0.68929	-0.23413
202.651	-0.68932	-0.23415
202.667	-0.68935	-0.23416
202.684	-0.68938	-0.23418
202.701	-0.68941	-0.2342
202.718	-0.68944	-0.23421
202.734	-0.68947	-0.23423
202.751	-0.68949	-0.23424
202.767	-0.68952	-0.23426
202.784	-0.68955	-0.23428
202.8	-0.68957	-0.2343
202.817	-0.68959	-0.23431
202.834	-0.68961	-0.23433
202.851	-0.68963	-0.23435
202.868	-0.68965	-0.23437
202.884	-0.68967	-0.23439
202.901	-0.68969	-0.2344
202.917	-0.68971	-0.23442
202.934	-0.68974	-0.23444
202.95	-0.68977	-0.23446
202.967	-0.68981	-0.23448
202.983	-0.68983	-0.2345
203	-0.68986	-0.23452
203.017	-0.68989	-0.23454
203.034	-0.68991	-0.23455
203.051	-0.68994	-0.23457
203.067	-0.68997	-0.23459
203.084	-0.68999	-0.23461

203.101	-0.69002	-0.23463
203.118	-0.69005	-0.23465
203.135	-0.69007	-0.23467
203.151	-0.69009	-0.23469
203.168	-0.69011	-0.23471
203.184	-0.69013	-0.23473
203.201	-0.69014	-0.23474
203.218	-0.69016	-0.23476
203.234	-0.69019	-0.23478
203.252	-0.69022	-0.2348
203.267	-0.69025	-0.23482
203.284	-0.69029	-0.23484
203.301	-0.69032	-0.23486
203.318	-0.69036	-0.23488
203.334	-0.69039	-0.2349
203.35	-0.69042	-0.23492
203.368	-0.69044	-0.23494
203.384	-0.69047	-0.23496
203.402	-0.6905	-0.23498
203.418	-0.69052	-0.235
203.434	-0.69055	-0.23502
203.45	-0.69058	-0.23504
203.467	-0.69061	-0.23507
203.483	-0.69064	-0.2351
203.5	-0.69068	-0.23513
203.518	-0.69071	-0.23515
203.534	-0.69074	-0.23517
203.551	-0.69077	-0.23519
203.566	-0.6908	-0.23521
203.584	-0.69082	-0.23523

203.601	-0.69085	-0.23525
203.617	-0.69088	-0.23526
203.634	-0.69091	-0.23528
203.651	-0.69094	-0.2353
203.667	-0.69097	-0.23532
203.684	-0.691	-0.23534
203.701	-0.69103	-0.23536
203.717	-0.69107	-0.23538
203.734	-0.6911	-0.2354
203.751	-0.69113	-0.23542
203.767	-0.69116	-0.23544
203.784	-0.69119	-0.23548
203.801	-0.69121	-0.2355
203.817	-0.69124	-0.23552
203.834	-0.69126	-0.23554
203.85	-0.69129	-0.23556
203.868	-0.69132	-0.23558
203.885	-0.69136	-0.23561
203.901	-0.69139	-0.23563
203.918	-0.69142	-0.23565
203.934	-0.69145	-0.23567
203.951	-0.69148	-0.23569
203.967	-0.69151	-0.23571
203.984	-0.69153	-0.23573
204	-0.69156	-0.23575
204.017	-0.69159	-0.23576
204.034	-0.69163	-0.23578
204.05	-0.69166	-0.23581
204.068	-0.69171	-0.23583
204.084	-0.69174	-0.23586

204.1	-0.69178	-0.2359
204.117	-0.69181	-0.23592
204.134	-0.69184	-0.23595
204.151	-0.69186	-0.23598
204.168	-0.69188	-0.236
204.184	-0.69191	-0.23602
204.201	-0.69193	-0.23604
204.217	-0.69195	-0.23606
204.234	-0.69197	-0.23608
204.25	-0.692	-0.2361
204.267	-0.69203	-0.23612
204.284	-0.69207	-0.23614
204.301	-0.6921	-0.23616
204.318	-0.69213	-0.23618
204.334	-0.69216	-0.2362
204.349	-0.69219	-0.23622
204.367	-0.69222	-0.23625
204.383	-0.69225	-0.23627
204.4	-0.69228	-0.23629
204.417	-0.6923	-0.23631
204.433	-0.69233	-0.23633
204.449	-0.69236	-0.23636
204.466	-0.69239	-0.23638
204.483	-0.69242	-0.2364
204.499	-0.69244	-0.23643
204.516	-0.69247	-0.23645
204.533	-0.69251	-0.23648
204.55	-0.69253	-0.23653
204.567	-0.69255	-0.23656
204.583	-0.69258	-0.23658

204.6	-0.6926	-0.23661
204.616	-0.69262	-0.23663
204.633	-0.69265	-0.23666
204.649	-0.69267	-0.23668
204.666	-0.69269	-0.2367
204.683	-0.69271	-0.23672
204.699	-0.69273	-0.23674
204.716	-0.69276	-0.23676
204.733	-0.69278	-0.23679
204.749	-0.69281	-0.23681
204.766	-0.69283	-0.23683
204.783	-0.69285	-0.23686
204.8	-0.69288	-0.2369
204.817	-0.69291	-0.23693
204.833	-0.69293	-0.23696
204.85	-0.69296	-0.23699
204.867	-0.69298	-0.23702
204.883	-0.693	-0.23705
204.899	-0.69302	-0.23707
204.916	-0.69304	-0.23711
204.933	-0.69307	-0.23715
204.949	-0.69309	-0.23718
204.966	-0.69312	-0.23721
204.983	-0.69315	-0.23723
205	-0.69317	-0.23726
205.016	-0.6932	-0.23729
205.032	-0.69322	-0.23732
205.049	-0.69324	-0.23735
205.066	-0.69327	-0.23738
205.083	-0.69329	-0.23742

205.1	-0.69331	-0.23748
205.116	-0.69333	-0.23757
205.133	-0.69335	-0.23766
205.15	-0.69337	-0.23775
205.166	-0.69339	-0.23784
205.183	-0.69342	-0.2379
205.2	-0.69346	-0.23794
205.216	-0.69349	-0.23797
205.233	-0.69352	-0.238
205.25	-0.69356	-0.23803
205.266	-0.6936	-0.23806
205.283	-0.69363	-0.23808
205.3	-0.69366	-0.23811
205.317	-0.6937	-0.23814
205.333	-0.69373	-0.23817
205.349	-0.69376	-0.2382
205.366	-0.69379	-0.23823
205.383	-0.69382	-0.23826
205.399	-0.69386	-0.23829
205.417	-0.69388	-0.23832
205.433	-0.69392	-0.23835
205.45	-0.69395	-0.23839
205.467	-0.69398	-0.23842
205.483	-0.69402	-0.23845
205.5	-0.69405	-0.23848
205.517	-0.69408	-0.23851
205.533	-0.69411	-0.23854
205.551	-0.69414	-0.23857
205.567	-0.69418	-0.23862
205.584	-0.69422	-0.23869

205.601	-0.69426	-0.23875
205.618	-0.6943	-0.23879
205.633	-0.69434	-0.23883
205.65	-0.69438	-0.23886
205.666	-0.69441	-0.23889
205.684	-0.69444	-0.23893
205.7	-0.69448	-0.23896
205.717	-0.69451	-0.23899
205.733	-0.69454	-0.23902
205.75	-0.69458	-0.23905
205.767	-0.69462	-0.23909
205.783	-0.69466	-0.23912
205.8	-0.6947	-0.23915
205.817	-0.69475	-0.23919
205.833	-0.69479	-0.23923
205.85	-0.69482	-0.23927
205.867	-0.69485	-0.23931
205.883	-0.69488	-0.23936
205.9	-0.69491	-0.2394
205.917	-0.69494	-0.23943
205.933	-0.69497	-0.23947
205.95	-0.695	-0.23951
205.966	-0.69503	-0.23955
205.982	-0.69506	-0.23958
206	-0.6951	-0.23962
206.016	-0.69514	-0.23965
206.033	-0.69517	-0.23969
206.05	-0.69521	-0.23972
206.066	-0.69525	-0.23975
206.083	-0.69528	-0.23978

206.1	-0.69531	-0.23982
206.116	-0.69534	-0.23985
206.132	-0.69537	-0.23988
206.149	-0.6954	-0.23993
206.166	-0.69544	-0.23998
206.183	-0.69547	-0.24004
206.199	-0.69551	-0.24013
206.217	-0.69554	-0.24021
206.234	-0.69558	-0.24028
206.25	-0.69562	-0.24032
206.267	-0.69565	-0.24036
206.282	-0.69569	-0.2404
206.299	-0.69571	-0.24044
206.316	-0.69575	-0.24048
206.332	-0.69578	-0.24053
206.349	-0.69581	-0.24058
206.366	-0.69584	-0.24061
206.383	-0.69587	-0.24065
206.399	-0.6959	-0.24068
206.416	-0.69593	-0.24072
206.433	-0.69596	-0.24077
206.449	-0.69599	-0.24081
206.466	-0.69601	-0.24085
206.482	-0.69604	-0.24088
206.5	-0.69606	-0.24093
206.516	-0.69608	-0.24099
206.533	-0.69611	-0.24104
206.548	-0.69613	-0.2411
206.566	-0.69616	-0.24113
206.583	-0.69619	-0.24117

206.598	-0.69622	-0.2412
206.615	-0.69626	-0.24124
206.632	-0.69629	-0.24128
206.648	-0.69633	-0.24134
206.666	-0.69636	-0.24141
206.682	-0.6964	-0.24151
206.699	-0.69643	-0.24162
206.715	-0.69647	-0.24171
206.731	-0.6965	-0.24185
206.749	-0.69653	-0.24192
206.766	-0.69655	-0.24201
206.782	-0.69658	-0.24209
206.798	-0.6966	-0.24215
206.815	-0.69662	-0.24218
206.831	-0.69664	-0.24222
206.848	-0.69666	-0.24225
206.864	-0.69669	-0.24229
206.882	-0.69672	-0.24235
206.898	-0.69674	-0.24241
206.915	-0.69676	-0.24249
206.932	-0.69679	-0.24255
206.948	-0.69682	-0.24264
206.966	-0.69685	-0.24273
206.981	-0.69688	-0.24284
206.998	-0.6969	-0.24293
207.015	-0.69693	-0.24301
207.032	-0.69696	-0.24309
207.048	-0.69699	-0.24315
207.065	-0.69701	-0.24324
207.082	-0.69704	-0.24333

207.097	-0.69706	-0.24346
207.115	-0.69709	-0.24354
207.131	-0.69712	-0.24363
207.148	-0.69714	-0.24371
207.165	-0.69717	-0.24376
207.181	-0.69719	-0.24384
207.198	-0.69722	-0.24392
207.215	-0.69725	-0.24397
207.23	-0.69727	-0.24401
207.247	-0.6973	-0.24407
207.264	-0.69732	-0.24416
207.281	-0.69735	-0.24425
207.298	-0.69737	-0.24433
207.314	-0.6974	-0.2444
207.331	-0.69744	-0.24448
207.347	-0.69747	-0.24453
207.365	-0.69749	-0.24458
207.381	-0.69752	-0.24463
207.398	-0.69754	-0.24469
207.414	-0.69756	-0.24475
207.43	-0.69759	-0.24483
207.447	-0.69761	-0.24494
207.463	-0.69764	-0.24516
207.48	-0.69767	-0.24533
207.497	-0.6977	-0.2455
207.514	-0.69773	-0.24565
207.53	-0.69776	-0.24582
207.547	-0.69779	-0.24592
207.564	-0.69782	-0.24602
207.58	-0.69785	-0.24608

207.597	-0.69787	-0.24614
207.614	-0.6979	-0.24624
207.63	-0.69793	-0.24633
207.647	-0.69795	-0.24644
207.663	-0.69798	-0.24655
207.68	-0.69801	-0.24669
207.696	-0.69803	-0.24683
207.713	-0.69807	-0.24699
207.73	-0.6981	-0.24719
207.747	-0.69813	-0.24736
207.763	-0.69816	-0.24747
207.781	-0.69819	-0.24753
207.797	-0.69822	-0.24758
207.813	-0.69824	-0.24762
207.83	-0.69827	-0.24765
207.846	-0.69829	-0.24771
207.863	-0.69831	-0.2478
207.879	-0.69833	-0.24789
207.896	-0.69834	-0.24801
207.912	-0.69837	-0.24823
207.93	-0.69839	-0.24849
207.946	-0.69841	-0.24874
207.963	-0.69844	-0.24893
207.979	-0.69846	-0.24909
207.997	-0.69848	-0.24927
208.013	-0.69851	-0.24939
208.03	-0.69854	-0.24953
208.046	-0.69857	-0.24964
208.063	-0.6986	-0.24973
208.08	-0.69862	-0.2498

208.096	-0.69865	-0.24987
208.113	-0.69867	-0.24994
208.129	-0.69869	-0.25
208.145	-0.69872	-0.25004
208.163	-0.69874	-0.25012
208.179	-0.69877	-0.25024
208.196	-0.69879	-0.25039
208.212	-0.69882	-0.25055
208.229	-0.69884	-0.2507
208.246	-0.69886	-0.25087
208.262	-0.69888	-0.25108
208.279	-0.69891	-0.25127
208.295	-0.69893	-0.25144
208.312	-0.69895	-0.25162
208.328	-0.69896	-0.25181
208.345	-0.69898	-0.25197
208.361	-0.699	-0.25212
208.378	-0.69903	-0.25223
208.395	-0.69906	-0.25236
208.412	-0.69909	-0.25246
208.428	-0.69912	-0.25255
208.444	-0.69915	-0.25265
208.461	-0.69919	-0.25278
208.478	-0.69922	-0.25286
208.494	-0.69925	-0.253
208.512	-0.69927	-0.25318
208.529	-0.6993	-0.25335
208.545	-0.69932	-0.25349
208.561	-0.69934	-0.2536
208.578	-0.69936	-0.25372

208.595	-0.69937	-0.25383
208.611	-0.6994	-0.25393
208.627	-0.69942	-0.25404
208.644	-0.69945	-0.25418
208.66	-0.69948	-0.25436
208.678	-0.69951	-0.2546
208.694	-0.69954	-0.2548
208.711	-0.69956	-0.25498
208.727	-0.6996	-0.25516
208.745	-0.69963	-0.25537
208.761	-0.69966	-0.25557
208.777	-0.69968	-0.25582
208.794	-0.69971	-0.25602
208.811	-0.69974	-0.2562
208.827	-0.69977	-0.25643
208.845	-0.6998	-0.25665
208.861	-0.69983	-0.25691
208.878	-0.69987	-0.25715
208.894	-0.6999	-0.25734
208.911	-0.69995	-0.25746
208.928	-0.69999	-0.25761
208.944	-0.70003	-0.25776
208.961	-0.70007	-0.25789
208.978	-0.70012	-0.25801
208.993	-0.70016	-0.25813
209.011	-0.7002	-0.25826
209.027	-0.70023	-0.25838
209.045	-0.70027	-0.25855
209.061	-0.70031	-0.25871
209.077	-0.70034	-0.25889

209.094	-0.70038	-0.25908
209.11	-0.70042	-0.2593
209.128	-0.70045	-0.25947
209.144	-0.70048	-0.25962
209.16	-0.70051	-0.25974
209.177	-0.70054	-0.25986
209.194	-0.70057	-0.26
209.21	-0.70061	-0.26015
209.227	-0.70065	-0.26031
209.244	-0.70069	-0.2605
209.26	-0.70072	-0.26068
209.277	-0.70076	-0.26083
209.294	-0.7008	-0.26095
209.31	-0.70083	-0.26112
209.326	-0.70087	-0.26127
209.343	-0.7009	-0.26142
209.361	-0.70093	-0.26155
209.377	-0.70097	-0.26167
209.394	-0.701	-0.26184
209.41	-0.70103	-0.26202
209.427	-0.70107	-0.2622
209.443	-0.7011	-0.26235
209.46	-0.70113	-0.26251
209.476	-0.70117	-0.26265
209.493	-0.70121	-0.26282
209.51	-0.70124	-0.26301
209.526	-0.70127	-0.26318
209.543	-0.7013	-0.26336
209.559	-0.70134	-0.26353
209.577	-0.70137	-0.26368

209.593	-0.7014	-0.26382
209.609	-0.70143	-0.26394
209.626	-0.70146	-0.26405
209.644	-0.70149	-0.2642
209.66	-0.70152	-0.26434
209.676	-0.70154	-0.26453
209.693	-0.70157	-0.26475
209.71	-0.70161	-0.26498
209.726	-0.70164	-0.26519
209.743	-0.70168	-0.26538
209.759	-0.70171	-0.26552
209.776	-0.70175	-0.26563
209.793	-0.70178	-0.26572
209.809	-0.70181	-0.26581
209.826	-0.70184	-0.26592
209.843	-0.70186	-0.26605
209.858	-0.70188	-0.26624
209.875	-0.7019	-0.26645
209.891	-0.70193	-0.26669
209.908	-0.70196	-0.26694
209.925	-0.70199	-0.26716
209.942	-0.70202	-0.26735
209.959	-0.70206	-0.26752
209.976	-0.7021	-0.26767
209.992	-0.70214	-0.26783
210.008	-0.70218	-0.26802
210.025	-0.70222	-0.26821
210.042	-0.70225	-0.26837
210.058	-0.70228	-0.26851
210.075	-0.70231	-0.26864

210.091	-0.70233	-0.26876
210.109	-0.70236	-0.26886
210.125	-0.7024	-0.26894
210.142	-0.70243	-0.26904
210.158	-0.70246	-0.26914
210.175	-0.70249	-0.26927
210.191	-0.70252	-0.26943
210.208	-0.70255	-0.26961
210.224	-0.70257	-0.26976
210.24	-0.70259	-0.26995
210.257	-0.70261	-0.27014
210.273	-0.70263	-0.27031
210.29	-0.70265	-0.27049
210.308	-0.70268	-0.27065
210.324	-0.70271	-0.27078
210.341	-0.70274	-0.2709
210.357	-0.70277	-0.27103
210.373	-0.7028	-0.27118
210.39	-0.70283	-0.27135
210.407	-0.70286	-0.27151
210.423	-0.70288	-0.27164
210.44	-0.7029	-0.27181
210.457	-0.70292	-0.27199
210.473	-0.70295	-0.27219
210.489	-0.70298	-0.27239
210.505	-0.70301	-0.27261
210.523	-0.70303	-0.27277
210.538	-0.70305	-0.27294
210.556	-0.70307	-0.27315
210.572	-0.7031	-0.27338

210.589	-0.70312	-0.27361
210.606	-0.70313	-0.27381
210.622	-0.70315	-0.27402
210.638	-0.70316	-0.27423
210.655	-0.70317	-0.27438
210.672	-0.70318	-0.27452
210.689	-0.7032	-0.27469
210.705	-0.70321	-0.27482
210.722	-0.70323	-0.275
210.739	-0.70325	-0.27516
210.755	-0.70328	-0.27537
210.772	-0.7033	-0.27556
210.787	-0.70333	-0.27573
210.804	-0.70336	-0.27589
210.821	-0.70339	-0.27608
210.837	-0.70342	-0.27627
210.853	-0.70344	-0.27646
210.87	-0.70346	-0.27666
210.887	-0.70348	-0.27685
210.904	-0.7035	-0.27707
210.921	-0.70352	-0.27735
210.936	-0.70354	-0.27768
210.954	-0.70356	-0.27797
210.97	-0.70358	-0.27815
210.986	-0.7036	-0.27831
211.004	-0.70362	-0.27847
211.02	-0.70364	-0.27867
211.036	-0.70367	-0.27888
211.052	-0.70369	-0.27906
211.07	-0.70371	-0.27924

211.086	-0.70374	-0.27941
211.103	-0.70376	-0.27961
211.12	-0.70379	-0.27983
211.136	-0.70382	-0.28002
211.152	-0.70385	-0.28017
211.169	-0.70388	-0.28035
211.186	-0.7039	-0.28054
211.202	-0.70393	-0.28075
211.219	-0.70396	-0.28098
211.235	-0.70398	-0.28123
211.252	-0.704	-0.28148
211.268	-0.70402	-0.28169
211.285	-0.70404	-0.28189
211.302	-0.70406	-0.28208
211.318	-0.70409	-0.2822
211.335	-0.70413	-0.28232
211.351	-0.70416	-0.28247
211.368	-0.70419	-0.28262
211.385	-0.70422	-0.28282
211.401	-0.70425	-0.28309
211.418	-0.70428	-0.28337
211.434	-0.7043	-0.28363
211.451	-0.70433	-0.28392
211.467	-0.70435	-0.28418
211.484	-0.70438	-0.28447
211.5	-0.70441	-0.28472
211.517	-0.70444	-0.28492
211.534	-0.70447	-0.28506
211.55	-0.7045	-0.2852
211.567	-0.70452	-0.28533

211.583	-0.70455	-0.28546
211.6	-0.70458	-0.28557
211.617	-0.7046	-0.28568
211.634	-0.70462	-0.28578
211.65	-0.70465	-0.28588
211.666	-0.70467	-0.28606
211.683	-0.7047	-0.28625
211.7	-0.70472	-0.28649
211.716	-0.70475	-0.2868
211.733	-0.70479	-0.2871
211.75	-0.70482	-0.28734
211.766	-0.70486	-0.28753
211.783	-0.7049	-0.28766
211.799	-0.70493	-0.2878
211.816	-0.70496	-0.2879
211.833	-0.70499	-0.28798
211.85	-0.70502	-0.28808
211.865	-0.70505	-0.28816
211.883	-0.70508	-0.28829
211.899	-0.7051	-0.28848
211.915	-0.70513	-0.28868
211.932	-0.70516	-0.28889
211.949	-0.70519	-0.28916
211.966	-0.70523	-0.28939
211.983	-0.70526	-0.28965
211.998	-0.70529	-0.28989
212.015	-0.70532	-0.29013
212.032	-0.70535	-0.29036
212.048	-0.70538	-0.29051
212.064	-0.7054	-0.2907

212.081	-0.70543	-0.29087
212.098	-0.70545	-0.29103
212.115	-0.70548	-0.29116
212.131	-0.70551	-0.29131
212.148	-0.70554	-0.29143
212.164	-0.70557	-0.29157
212.181	-0.70561	-0.29169
212.198	-0.70564	-0.29183
212.214	-0.70567	-0.29199
212.231	-0.7057	-0.29218
212.248	-0.70573	-0.29239
212.264	-0.70575	-0.29255
212.281	-0.70577	-0.2927
212.297	-0.7058	-0.29283
212.314	-0.70582	-0.29299
212.331	-0.70584	-0.29314
212.347	-0.70586	-0.29329
212.364	-0.70589	-0.29345
212.381	-0.70592	-0.29357
212.397	-0.70595	-0.29366
212.413	-0.70598	-0.29375
212.43	-0.70601	-0.29386
212.446	-0.70604	-0.29394
212.463	-0.70607	-0.29404
212.48	-0.7061	-0.29415
212.496	-0.70613	-0.29433
212.513	-0.70615	-0.29452
212.53	-0.70617	-0.29477
212.545	-0.70619	-0.295
212.562	-0.70622	-0.29525

212.579	-0.70624	-0.29547
212.595	-0.70627	-0.29567
212.613	-0.7063	-0.29583
212.628	-0.70634	-0.29598
212.645	-0.70636	-0.29611
212.662	-0.70639	-0.29623
212.678	-0.70642	-0.29633
212.695	-0.70645	-0.29643
212.712	-0.70648	-0.29662
212.728	-0.70651	-0.29682
212.745	-0.70654	-0.29702
212.762	-0.70658	-0.29717
212.778	-0.70661	-0.2973
212.795	-0.70665	-0.29742
212.811	-0.70668	-0.29753
212.828	-0.70671	-0.29759
212.844	-0.70673	-0.29764
212.861	-0.70676	-0.29769
212.877	-0.70678	-0.29777
212.895	-0.7068	-0.29788
212.91	-0.70683	-0.29803
212.928	-0.70685	-0.2982
212.944	-0.70688	-0.29837
212.961	-0.7069	-0.29855
212.978	-0.70693	-0.29873
212.994	-0.70695	-0.29887
213.01	-0.70698	-0.29897
213.027	-0.70701	-0.29907
213.043	-0.70704	-0.29916
213.059	-0.70707	-0.29926

213.077	-0.7071	-0.29937
213.094	-0.70713	-0.29949
213.11	-0.70715	-0.2996
213.126	-0.70718	-0.29971
213.144	-0.7072	-0.29989
213.159	-0.70723	-0.30005
213.177	-0.70725	-0.30019
213.193	-0.70728	-0.30032
213.21	-0.70731	-0.30045
213.227	-0.70734	-0.30056
213.243	-0.70737	-0.30068
213.259	-0.7074	-0.30081
213.276	-0.70744	-0.30093
213.292	-0.70747	-0.30102
213.309	-0.70751	-0.30111
213.326	-0.70754	-0.30122
213.342	-0.70757	-0.30133
213.359	-0.7076	-0.30148
213.375	-0.70763	-0.30163
213.392	-0.70766	-0.30176
213.409	-0.70769	-0.3019
213.426	-0.70771	-0.30202
213.441	-0.70773	-0.30211
213.459	-0.70776	-0.3022
213.476	-0.70779	-0.3023
213.492	-0.70782	-0.3024
213.508	-0.70785	-0.30251
213.525	-0.70789	-0.30262
213.542	-0.70792	-0.30272
213.558	-0.70795	-0.30281

213.575	-0.70798	-0.30288
213.592	-0.70801	-0.30295
213.609	-0.70804	-0.30303
213.625	-0.70806	-0.30314
213.642	-0.70808	-0.30323
213.659	-0.70811	-0.30331
213.676	-0.70814	-0.30337
213.692	-0.70817	-0.30343
213.708	-0.7082	-0.30351
213.725	-0.70823	-0.3036
213.742	-0.70826	-0.30368
213.758	-0.7083	-0.30377
213.775	-0.70834	-0.30386
213.792	-0.70838	-0.30394
213.808	-0.70842	-0.30403
213.826	-0.70846	-0.30411
213.842	-0.70849	-0.30419
213.858	-0.70852	-0.30425
213.875	-0.70856	-0.3043
213.892	-0.7086	-0.30435
213.908	-0.70863	-0.30441
213.925	-0.70867	-0.30446
213.941	-0.7087	-0.30452
213.959	-0.70874	-0.30461
213.975	-0.70877	-0.30467
213.992	-0.7088	-0.30472
214.008	-0.70883	-0.30476
214.025	-0.70887	-0.30479
214.041	-0.7089	-0.30485
214.058	-0.70892	-0.30492

214.075	-0.70895	-0.305
214.091	-0.70898	-0.30509
214.108	-0.70901	-0.30517
214.126	-0.70905	-0.30522
214.141	-0.70909	-0.30528
214.158	-0.70913	-0.30533
214.174	-0.70916	-0.30537
214.191	-0.70919	-0.30541
214.208	-0.70923	-0.30544
214.224	-0.70927	-0.30547
214.241	-0.7093	-0.3055
214.257	-0.70934	-0.30553
214.274	-0.70937	-0.30557
214.291	-0.70941	-0.30561
214.307	-0.70944	-0.30565
214.324	-0.70947	-0.30569
214.341	-0.7095	-0.30573
214.358	-0.70952	-0.30578
214.374	-0.70954	-0.3058
214.39	-0.70957	-0.30584
214.407	-0.70959	-0.30586
214.423	-0.70963	-0.30589
214.441	-0.70966	-0.30592
214.458	-0.70969	-0.30595
214.474	-0.70973	-0.30598
214.491	-0.70977	-0.30602
214.507	-0.7098	-0.30605
214.524	-0.70984	-0.30609
214.54	-0.70987	-0.30612
214.556	-0.7099	-0.30615

214.573	-0.70993	-0.30617
214.59	-0.70996	-0.30619
214.607	-0.70999	-0.30621
214.624	-0.71003	-0.30623
214.64	-0.71006	-0.30625
214.657	-0.71009	-0.30627
214.674	-0.71011	-0.30629
214.69	-0.71014	-0.30631
214.707	-0.71016	-0.30633
214.724	-0.71018	-0.30635
214.74	-0.71021	-0.30637
214.756	-0.71023	-0.3064
214.773	-0.71025	-0.30643
214.791	-0.71028	-0.30645
214.806	-0.71032	-0.30649
214.824	-0.71035	-0.30652
214.84	-0.71039	-0.30655
214.857	-0.71042	-0.30657
214.874	-0.71045	-0.3066
214.89	-0.71047	-0.30662
214.906	-0.7105	-0.30666
214.923	-0.71052	-0.30669
214.94	-0.71055	-0.30671
214.957	-0.71058	-0.30673
214.973	-0.71061	-0.30675
214.99	-0.71064	-0.30678
215.007	-0.71067	-0.3068
215.022	-0.7107	-0.30682
215.039	-0.71072	-0.30685
215.056	-0.71074	-0.30687

215.074	-0.71076	-0.30689
215.09	-0.71078	-0.30692
215.106	-0.7108	-0.30694
215.124	-0.71082	-0.30697
215.14	-0.71084	-0.30699
215.157	-0.71086	-0.30701
215.173	-0.71089	-0.30704
215.19	-0.71092	-0.30707
215.206	-0.71096	-0.30709
215.223	-0.71099	-0.30711
215.24	-0.71102	-0.30713
215.255	-0.71106	-0.30715
215.273	-0.71109	-0.30717
215.29	-0.71112	-0.30719
215.306	-0.71114	-0.30721
215.323	-0.71117	-0.30724
215.339	-0.71119	-0.30726
215.356	-0.71121	-0.30729
215.373	-0.71123	-0.30731
215.39	-0.71125	-0.30734
215.406	-0.71127	-0.30736
215.423	-0.7113	-0.30738
215.439	-0.71133	-0.3074
215.456	-0.71136	-0.30742
215.473	-0.71139	-0.30745
215.489	-0.71143	-0.30747
215.506	-0.71146	-0.30749
215.522	-0.71149	-0.30751
215.54	-0.71153	-0.30753
215.556	-0.71156	-0.30755

215.573	-0.71159	-0.30757
215.59	-0.71161	-0.30759
215.606	-0.71163	-0.30762
215.622	-0.71165	-0.30764
215.639	-0.71167	-0.30766
215.656	-0.71169	-0.30768
215.672	-0.71172	-0.3077
215.689	-0.71175	-0.30772
215.706	-0.71177	-0.30775
215.722	-0.7118	-0.30777
215.739	-0.71183	-0.30779
215.755	-0.71186	-0.30781
215.773	-0.71189	-0.30783
215.79	-0.71192	-0.30786
215.806	-0.71195	-0.30788
215.823	-0.71198	-0.30791
215.839	-0.712	-0.30793
215.856	-0.71203	-0.30795
215.872	-0.71206	-0.30797
215.889	-0.7121	-0.308
215.907	-0.71213	-0.30802
215.923	-0.71217	-0.30805
215.939	-0.7122	-0.30807
215.956	-0.71223	-0.30809
215.973	-0.71227	-0.30812
215.989	-0.7123	-0.30814
216.006	-0.71232	-0.30816
216.022	-0.71235	-0.30818
216.039	-0.71237	-0.3082
216.056	-0.7124	-0.30822

216.072	-0.71244	-0.30825
216.089	-0.71247	-0.30827
216.106	-0.7125	-0.30829
216.123	-0.71253	-0.30831
216.14	-0.71257	-0.30834
216.156	-0.7126	-0.30836
216.173	-0.71263	-0.30839
216.191	-0.71266	-0.30841
216.206	-0.71268	-0.30843
216.223	-0.71271	-0.30846
216.24	-0.71275	-0.30848
216.256	-0.71278	-0.30851
216.273	-0.71282	-0.30854
216.29	-0.71285	-0.30856
216.306	-0.71289	-0.30859
216.322	-0.71292	-0.30861
216.339	-0.71296	-0.30864
216.356	-0.71299	-0.30866
216.372	-0.71303	-0.30868
216.39	-0.71306	-0.3087
216.406	-0.7131	-0.30872
216.422	-0.71313	-0.30875
216.439	-0.71316	-0.30877
216.456	-0.71319	-0.3088
216.474	-0.71323	-0.30882
216.49	-0.71326	-0.30885
216.507	-0.7133	-0.30887
216.524	-0.71334	-0.3089
216.54	-0.71338	-0.30892
216.558	-0.71342	-0.30894

216.573	-0.71346	-0.30897
216.589	-0.7135	-0.30899
216.607	-0.71355	-0.30901
216.623	-0.71359	-0.30903
216.64	-0.71363	-0.30905
216.656	-0.71366	-0.30907
216.673	-0.71369	-0.30909
216.689	-0.71373	-0.30911
216.707	-0.71376	-0.30913
216.723	-0.71379	-0.30915
216.74	-0.71384	-0.30917
216.756	-0.71388	-0.3092
216.774	-0.71392	-0.30922
216.79	-0.71396	-0.30924
216.807	-0.714	-0.30927
216.823	-0.71404	-0.30929
216.84	-0.71408	-0.30931
216.856	-0.71411	-0.30933
216.874	-0.71414	-0.30935
216.89	-0.71417	-0.30937
216.906	-0.7142	-0.30939
216.923	-0.71423	-0.30941
216.94	-0.71427	-0.30943
216.956	-0.7143	-0.30945
216.972	-0.71434	-0.30946
216.989	-0.71438	-0.30948
217.006	-0.71442	-0.3095
217.022	-0.71445	-0.30952
217.038	-0.71449	-0.30954
217.057	-0.71452	-0.30956

217.073	-0.71455	-0.30959
217.089	-0.71458	-0.30961
217.106	-0.71461	-0.30963
217.123	-0.71463	-0.30965
217.138	-0.71466	-0.30966
217.156	-0.71468	-0.30968
217.172	-0.71471	-0.30971
217.189	-0.71475	-0.30973
217.206	-0.71478	-0.30975
217.223	-0.71481	-0.30977
217.24	-0.71483	-0.30979
217.257	-0.71486	-0.30981
217.274	-0.71489	-0.30983
217.29	-0.71492	-0.30984
217.307	-0.71495	-0.30986
217.322	-0.71498	-0.30987
217.339	-0.715	-0.30989
217.355	-0.71502	-0.30991
217.373	-0.71505	-0.30993
217.389	-0.71507	-0.30995
217.407	-0.71509	-0.30997
217.422	-0.71511	-0.30999
217.44	-0.71513	-0.31002
217.455	-0.71516	-0.31004
217.473	-0.71518	-0.31006
217.489	-0.71521	-0.31008
217.506	-0.71523	-0.3101
217.522	-0.71527	-0.31013
217.539	-0.7153	-0.31015
217.556	-0.71533	-0.31017

217.572	-0.71536	-0.31019
217.589	-0.71538	-0.31021
217.605	-0.71541	-0.31023
217.622	-0.71543	-0.31025
217.639	-0.71546	-0.31028
217.656	-0.71548	-0.3103
217.673	-0.71551	-0.31033
217.689	-0.71553	-0.31036
217.706	-0.71555	-0.31038
217.722	-0.71557	-0.3104
217.738	-0.7156	-0.31043
217.755	-0.71562	-0.31045
217.772	-0.71564	-0.31048
217.789	-0.71566	-0.3105
217.806	-0.71568	-0.31053
217.822	-0.7157	-0.31055
217.839	-0.71573	-0.31058
217.855	-0.71576	-0.3106
217.871	-0.71579	-0.31063
217.888	-0.71582	-0.31065
217.905	-0.71585	-0.31068
217.922	-0.71588	-0.3107
217.938	-0.71591	-0.31073
217.955	-0.71594	-0.31076
217.971	-0.71596	-0.31078
217.988	-0.71599	-0.31082
218.005	-0.71601	-0.31086
218.022	-0.71604	-0.31089
218.039	-0.71608	-0.31092
218.055	-0.71611	-0.31094

218.071	-0.71615	-0.31097
218.088	-0.71619	-0.311
218.104	-0.71623	-0.31102
218.121	-0.71627	-0.31105
218.137	-0.71631	-0.31108
218.154	-0.71634	-0.31111
218.171	-0.71636	-0.31114
218.187	-0.71639	-0.31119
218.204	-0.71641	-0.31123
218.221	-0.71644	-0.31128
218.238	-0.71646	-0.31134
218.254	-0.71648	-0.3114
218.271	-0.71651	-0.31148
218.289	-0.71654	-0.31155
218.305	-0.71658	-0.31161
218.321	-0.71662	-0.3117
218.338	-0.71666	-0.31178
218.355	-0.71669	-0.31183
218.371	-0.71673	-0.31186
218.387	-0.71677	-0.31189
218.404	-0.71681	-0.31192
218.421	-0.71684	-0.31195
218.438	-0.71687	-0.31198
218.454	-0.71691	-0.31202
218.471	-0.71695	-0.31205
218.487	-0.71699	-0.31208
218.504	-0.71703	-0.31213
218.521	-0.71707	-0.31217
218.537	-0.71711	-0.31221
218.554	-0.71714	-0.31225

218.571	-0.71718	-0.31231
218.587	-0.71722	-0.31235
218.604	-0.71726	-0.31238
218.621	-0.7173	-0.31241
218.637	-0.71733	-0.31244
218.654	-0.71737	-0.31247
218.67	-0.71742	-0.31251
218.687	-0.71746	-0.31256
218.704	-0.7175	-0.31261
218.72	-0.71755	-0.31267
218.737	-0.71758	-0.31272
218.754	-0.71761	-0.31282
218.771	-0.71764	-0.3129
218.788	-0.71767	-0.31294
218.804	-0.71771	-0.31298
218.821	-0.71773	-0.31301
218.838	-0.71777	-0.31305
218.854	-0.71781	-0.31308
218.871	-0.71784	-0.31311
218.887	-0.71789	-0.31315
218.903	-0.71794	-0.31318
218.921	-0.71801	-0.31321
218.938	-0.71805	-0.31324
218.954	-0.71811	-0.31327
218.971	-0.71815	-0.31331
218.987	-0.7182	-0.31334
219.004	-0.71823	-0.31337
219.02	-0.71827	-0.31344
219.037	-0.71831	-0.31352
219.053	-0.71834	-0.31361

219.07	-0.71837	-0.31367
219.088	-0.71841	-0.31372
219.104	-0.71844	-0.31376
219.121	-0.71847	-0.31379
219.137	-0.7185	-0.31383
219.153	-0.71852	-0.31386
219.17	-0.71856	-0.31389
219.187	-0.71859	-0.31393
219.203	-0.71862	-0.31399
219.22	-0.71865	-0.31406
219.237	-0.71868	-0.31413
219.254	-0.71872	-0.31417
219.27	-0.71875	-0.3142
219.288	-0.71879	-0.31424
219.304	-0.71883	-0.31427
219.321	-0.71887	-0.3143
219.337	-0.71891	-0.31433
219.354	-0.71895	-0.31437
219.37	-0.71899	-0.3144
219.386	-0.71903	-0.31445
219.403	-0.71907	-0.31451
219.42	-0.71911	-0.31458
219.436	-0.71914	-0.31461
219.453	-0.71917	-0.31465
219.47	-0.71919	-0.31468
219.487	-0.71922	-0.31472
219.504	-0.71925	-0.31475
219.52	-0.71928	-0.3148
219.536	-0.71931	-0.31484
219.554	-0.71935	-0.31488

219.57	-0.71938	-0.31492
219.586	-0.71941	-0.31499
219.603	-0.71945	-0.31508
219.62	-0.71947	-0.31515
219.636	-0.7195	-0.3152
219.653	-0.71954	-0.31527
219.669	-0.71957	-0.31533
219.687	-0.71961	-0.31538
219.703	-0.71965	-0.31542
219.72	-0.71969	-0.31545
219.737	-0.71973	-0.31549
219.753	-0.71977	-0.31552
219.769	-0.71981	-0.31557
219.786	-0.71984	-0.31561
219.803	-0.71987	-0.31566
219.82	-0.71989	-0.31571
219.836	-0.71992	-0.31575
219.853	-0.71995	-0.31578
219.87	-0.71997	-0.31583
219.886	-0.72	-0.31587
219.904	-0.72003	-0.3159
219.92	-0.72007	-0.31594
219.936	-0.7201	-0.31599
219.953	-0.72014	-0.31604
219.97	-0.72018	-0.31608
219.987	-0.72021	-0.31612
220.003	-0.72024	-0.31615
220.02	-0.72027	-0.31619
220.036	-0.72031	-0.31622
220.053	-0.72034	-0.31626

220.069	-0.72038	-0.31629
220.086	-0.72041	-0.31633
220.102	-0.72045	-0.31637
220.119	-0.72048	-0.31646
220.136	-0.72052	-0.31661
220.153	-0.72056	-0.31673
220.17	-0.72059	-0.31684
220.186	-0.72062	-0.31693
220.203	-0.72065	-0.317
220.22	-0.72069	-0.31708
220.237	-0.72072	-0.31713
220.253	-0.72075	-0.31716
220.27	-0.72078	-0.3172
220.287	-0.72081	-0.31723
220.303	-0.72084	-0.31726
220.32	-0.72087	-0.3173
220.336	-0.7209	-0.31733
220.353	-0.72093	-0.31737
220.37	-0.72096	-0.3174
220.387	-0.72099	-0.31744
220.403	-0.72102	-0.31748
220.42	-0.72106	-0.31752
220.437	-0.72109	-0.31756
220.453	-0.72113	-0.3176
220.47	-0.72117	-0.31763
220.487	-0.7212	-0.31767
220.503	-0.72124	-0.31773
220.52	-0.72127	-0.31781
220.536	-0.72131	-0.31791
220.552	-0.72134	-0.31797

220.57	-0.72137	-0.31802
220.586	-0.72141	-0.31806
220.603	-0.72144	-0.3181
220.62	-0.72147	-0.31814
220.636	-0.72151	-0.31817
220.652	-0.72154	-0.31823
220.67	-0.72157	-0.31826
220.686	-0.72159	-0.3183
220.703	-0.72162	-0.31833
220.718	-0.72165	-0.31837
220.736	-0.72168	-0.31842
220.752	-0.72171	-0.31847
220.769	-0.72174	-0.31852
220.786	-0.72177	-0.31856
220.803	-0.72181	-0.3186
220.82	-0.72185	-0.31865
220.836	-0.72189	-0.31869
220.853	-0.72193	-0.31874
220.87	-0.72197	-0.31877
220.886	-0.72201	-0.31882
220.903	-0.72206	-0.31887
220.92	-0.7221	-0.3189
220.936	-0.72213	-0.31894
220.953	-0.72216	-0.31897
220.969	-0.72219	-0.31901
220.986	-0.72223	-0.31905
221.003	-0.72227	-0.31909
221.019	-0.72231	-0.31913
221.036	-0.72235	-0.31917
221.053	-0.72239	-0.31921

221.069	-0.72243	-0.31924
221.086	-0.72248	-0.31928
221.102	-0.72252	-0.31931
221.119	-0.72255	-0.31935
221.136	-0.72258	-0.31939
221.153	-0.72261	-0.31944
221.169	-0.72264	-0.31951
221.186	-0.72267	-0.31959
221.202	-0.72271	-0.31966
221.219	-0.72275	-0.3197
221.236	-0.72278	-0.31974
221.252	-0.72282	-0.31978
221.269	-0.72285	-0.31982
221.286	-0.72289	-0.31986
221.302	-0.72293	-0.31989
221.319	-0.72297	-0.31993
221.336	-0.72301	-0.31997
221.353	-0.72305	-0.32001
221.37	-0.72308	-0.32005
221.385	-0.72311	-0.32012
221.403	-0.72315	-0.32017
221.419	-0.72319	-0.32022
221.436	-0.72323	-0.32027
221.453	-0.72327	-0.32032
221.469	-0.72331	-0.32038
221.486	-0.72335	-0.32042
221.504	-0.7234	-0.32046
221.52	-0.72344	-0.32052
221.537	-0.72348	-0.32056
221.553	-0.72351	-0.3206

221.569	-0.72354	-0.32064
221.587	-0.72356	-0.32067
221.603	-0.72359	-0.32071
221.62	-0.72362	-0.32075
221.637	-0.72365	-0.32079
221.653	-0.72369	-0.32083
221.67	-0.72372	-0.32087
221.686	-0.72376	-0.32092
221.704	-0.72379	-0.32097
221.72	-0.72383	-0.32102
221.737	-0.72387	-0.32108
221.754	-0.7239	-0.32114
221.769	-0.72394	-0.32117
221.786	-0.72397	-0.32121
221.802	-0.724	-0.32126
221.82	-0.72403	-0.3213
221.836	-0.72407	-0.32134
221.852	-0.7241	-0.32137
221.869	-0.72414	-0.3214
221.885	-0.72418	-0.32144
221.902	-0.72423	-0.32147
221.919	-0.72427	-0.32151
221.936	-0.7243	-0.32155
221.952	-0.72434	-0.32159
221.968	-0.72437	-0.32163
221.985	-0.7244	-0.32168
222.002	-0.72443	-0.32173
222.019	-0.72446	-0.32178
222.035	-0.7245	-0.32183
222.053	-0.72454	-0.32187

222.069	-0.72458	-0.32191
222.085	-0.72462	-0.32194
222.102	-0.72466	-0.32198
222.118	-0.7247	-0.32201
222.135	-0.72472	-0.32205
222.151	-0.72476	-0.32208
222.168	-0.72479	-0.32212
222.185	-0.72481	-0.32215
222.202	-0.72484	-0.32219
222.218	-0.72487	-0.32223
222.235	-0.72491	-0.32227
222.251	-0.72495	-0.32231
222.268	-0.72498	-0.32235
222.285	-0.72502	-0.32239
222.302	-0.72506	-0.32243
222.319	-0.7251	-0.32247
222.335	-0.72513	-0.3225
222.352	-0.72517	-0.32254
222.368	-0.7252	-0.32258
222.384	-0.72524	-0.32262
222.401	-0.72527	-0.32267
222.418	-0.7253	-0.32271
222.435	-0.72535	-0.32275
222.452	-0.72539	-0.32279
222.467	-0.72543	-0.32283
222.485	-0.72547	-0.32288
222.501	-0.72551	-0.32292
222.517	-0.72555	-0.32296
222.535	-0.7256	-0.323
222.551	-0.72564	-0.32304

222.568	-0.72568	-0.32307
222.584	-0.72572	-0.32311
222.601	-0.72576	-0.32315
222.618	-0.7258	-0.32319
222.634	-0.72584	-0.32323
222.651	-0.72588	-0.32327
222.668	-0.72592	-0.32331
222.685	-0.72596	-0.32335
222.701	-0.726	-0.32338
222.718	-0.72606	-0.32342
222.734	-0.72614	-0.32345
222.751	-0.72629	-0.32349
222.767	-0.72644	-0.32352
222.784	-0.72658	-0.32356
222.801	-0.72673	-0.3236
222.817	-0.7268	-0.32364
222.835	-0.72686	-0.32367
222.851	-0.7269	-0.32371
222.867	-0.72694	-0.32375
222.885	-0.72697	-0.32379
222.901	-0.72701	-0.32382
222.917	-0.72704	-0.32386
222.934	-0.72708	-0.3239
222.95	-0.72711	-0.32394
222.967	-0.72714	-0.32397
222.984	-0.72717	-0.32401
223.001	-0.7272	-0.32405
223.017	-0.72724	-0.32409
223.034	-0.72728	-0.32413
223.05	-0.72731	-0.32418

223.067	-0.72735	-0.32422
223.084	-0.72739	-0.32426
223.101	-0.72742	-0.3243
223.117	-0.72746	-0.32434
223.135	-0.7275	-0.32437
223.149	-0.72753	-0.32441
223.166	-0.72757	-0.32445
223.184	-0.72761	-0.32449
223.2	-0.72764	-0.32452
223.217	-0.72767	-0.32456
223.234	-0.72771	-0.3246
223.251	-0.72775	-0.32464
223.267	-0.72779	-0.32468
223.284	-0.72782	-0.32472
223.3	-0.72785	-0.32475
223.317	-0.72789	-0.32479
223.334	-0.72792	-0.32483
223.35	-0.72796	-0.32487
223.368	-0.72801	-0.3249
223.384	-0.72805	-0.32494
223.4	-0.72809	-0.32498
223.417	-0.72814	-0.32502
223.434	-0.72818	-0.32506
223.45	-0.72821	-0.32509
223.468	-0.72825	-0.32513
223.483	-0.72828	-0.32517
223.5	-0.72831	-0.32521
223.518	-0.72833	-0.32525
223.534	-0.72836	-0.32529
223.551	-0.72839	-0.32533

223.567	-0.72842	-0.32537
223.584	-0.72845	-0.32541
223.601	-0.72849	-0.32546
223.617	-0.72852	-0.32549
223.634	-0.72856	-0.32553
223.65	-0.72859	-0.32557
223.667	-0.72864	-0.32561
223.684	-0.72868	-0.32564
223.701	-0.72873	-0.32568
223.718	-0.72877	-0.32571
223.734	-0.7288	-0.32574
223.751	-0.72884	-0.32578
223.768	-0.72888	-0.32582
223.785	-0.72891	-0.32585
223.8	-0.72895	-0.32589
223.817	-0.72898	-0.32597
223.834	-0.72902	-0.32605
223.85	-0.72906	-0.32613
223.867	-0.7291	-0.32617
223.884	-0.72913	-0.32621
223.9	-0.72917	-0.32625
223.917	-0.72921	-0.32629
223.934	-0.72925	-0.32633
223.951	-0.72929	-0.32636
223.968	-0.72933	-0.3264
223.984	-0.72938	-0.32644
224.001	-0.72942	-0.32647
224.017	-0.72946	-0.32651
224.035	-0.72951	-0.32655
224.051	-0.72956	-0.32659

224.068	-0.7296	-0.32662
224.084	-0.72965	-0.32666
224.101	-0.7297	-0.3267
224.117	-0.72975	-0.32673
224.134	-0.72979	-0.32677
224.151	-0.72984	-0.3268
224.168	-0.72989	-0.32684
224.184	-0.72994	-0.32687
224.201	-0.72998	-0.32691
224.217	-0.73002	-0.32694
224.234	-0.73006	-0.32698
224.251	-0.7301	-0.32701
224.268	-0.73014	-0.32705
224.284	-0.73018	-0.32708
224.301	-0.73023	-0.32711
224.318	-0.73027	-0.32715
224.334	-0.73031	-0.32718
224.351	-0.73035	-0.32722
224.368	-0.73039	-0.32725
224.384	-0.73043	-0.32728
224.4	-0.73047	-0.32732
224.418	-0.73051	-0.32735
224.434	-0.73055	-0.32738
224.451	-0.73059	-0.32742
224.468	-0.73063	-0.32745
224.484	-0.73067	-0.32749
224.501	-0.73071	-0.32752
224.517	-0.73075	-0.32755
224.535	-0.73079	-0.32758
224.551	-0.73082	-0.32761

224.568	-0.73085	-0.32765
224.584	-0.73088	-0.32768
224.601	-0.73091	-0.32771
224.618	-0.73094	-0.32774
224.634	-0.73098	-0.32777
224.651	-0.73101	-0.32781
224.667	-0.73104	-0.32784
224.685	-0.73106	-0.32787
224.701	-0.73109	-0.3279
224.718	-0.73112	-0.32793
224.735	-0.73116	-0.32797
224.751	-0.73119	-0.32799
224.767	-0.73121	-0.32802
224.784	-0.73123	-0.32805
224.802	-0.73125	-0.32808
224.818	-0.73127	-0.32811
224.835	-0.73129	-0.32814
224.852	-0.73131	-0.32817
224.868	-0.73133	-0.3282
224.884	-0.73135	-0.32823
224.901	-0.73137	-0.32826
224.918	-0.73139	-0.32829
224.934	-0.73141	-0.32832
224.951	-0.73143	-0.32835
224.967	-0.73145	-0.32838
224.984	-0.73146	-0.32841
225	-0.73148	-0.32843
225.017	-0.7315	-0.32846
225.033	-0.73151	-0.32849
225.051	-0.73153	-0.32852

225.068	-0.73156	-0.32855
225.084	-0.73158	-0.32858
225.101	-0.7316	-0.32861
225.118	-0.73162	-0.32864
225.133	-0.73163	-0.32867
225.151	-0.73164	-0.3287
225.167	-0.73166	-0.32872
225.184	-0.73167	-0.32875
225.201	-0.73169	-0.32877
225.217	-0.7317	-0.32879
225.234	-0.73172	-0.32881
225.251	-0.73174	-0.32884
225.267	-0.73176	-0.32886
225.285	-0.73177	-0.32889
225.301	-0.73179	-0.32891
225.318	-0.73181	-0.32894
225.334	-0.73183	-0.32897
225.351	-0.73185	-0.32899
225.368	-0.73187	-0.32902
225.384	-0.73189	-0.32905
225.4	-0.73191	-0.32908
225.418	-0.73192	-0.32911
225.433	-0.73193	-0.32913
225.451	-0.73194	-0.32916
225.468	-0.73196	-0.32918
225.485	-0.73197	-0.32921
225.501	-0.73198	-0.32923
225.517	-0.73199	-0.32926
225.535	-0.73201	-0.32929
225.551	-0.73202	-0.32932

225.568	-0.73204	-0.32934
225.585	-0.73205	-0.32937
225.6	-0.73206	-0.32939
225.617	-0.73208	-0.32942
225.634	-0.73209	-0.32944
225.651	-0.7321	-0.32946
225.667	-0.73211	-0.32948
225.684	-0.73213	-0.32951
225.701	-0.73215	-0.32953
225.717	-0.73217	-0.32955
225.734	-0.73219	-0.32957
225.751	-0.73221	-0.32959
225.768	-0.73223	-0.32962
225.783	-0.73225	-0.32964
225.8	-0.73227	-0.32967
225.818	-0.73228	-0.32969
225.834	-0.73229	-0.32971
225.852	-0.7323	-0.32973
225.867	-0.73231	-0.32975
225.884	-0.73232	-0.32977
225.901	-0.73233	-0.32979
225.917	-0.73234	-0.32981
225.934	-0.73235	-0.32983
225.95	-0.73237	-0.32985
225.967	-0.73239	-0.32988
225.984	-0.7324	-0.3299
226	-0.73242	-0.32992
226.018	-0.73244	-0.32994
226.034	-0.73245	-0.32996
226.051	-0.73247	-0.32998

226.067	-0.73248	-0.33
226.084	-0.73249	-0.33002
226.101	-0.7325	-0.33004
226.117	-0.7325	-0.33006
226.135	-0.73252	-0.33007
226.151	-0.73253	-0.33009
226.167	-0.73254	-0.3301
226.184	-0.73255	-0.33012
226.2	-0.73256	-0.33014
226.217	-0.73257	-0.33015
226.234	-0.73258	-0.33017
226.25	-0.73259	-0.33018
226.266	-0.73261	-0.3302
226.284	-0.73262	-0.33021
226.3	-0.73263	-0.33022
226.317	-0.73264	-0.33023
226.334	-0.73265	-0.33025
226.35	-0.73267	-0.33026
226.367	-0.73268	-0.33027
226.383	-0.7327	-0.33028
226.4	-0.73272	-0.3303
226.417	-0.73273	-0.33031
226.433	-0.73275	-0.33032
226.449	-0.73277	-0.33033
226.466	-0.73278	-0.33034
226.484	-0.73279	-0.33035
226.5	-0.73281	-0.33036
226.517	-0.73282	-0.33037
226.534	-0.73284	-0.33039
226.551	-0.73285	-0.3304

226.567	-0.73287	-0.3304
226.583	-0.7329	-0.33041
226.6	-0.73292	-0.33042
226.617	-0.73294	-0.33043
226.633	-0.73296	-0.33044
226.65	-0.73298	-0.33045
226.667	-0.733	-0.33046
226.684	-0.73301	-0.33047
226.701	-0.73303	-0.33048
226.717	-0.73305	-0.33049
226.734	-0.73308	-0.3305
226.75	-0.73311	-0.33051
226.767	-0.73314	-0.33052
226.783	-0.73317	-0.33052
226.8	-0.73319	-0.33053
226.818	-0.73322	-0.33054
226.833	-0.73324	-0.33054
226.85	-0.73325	-0.33054
226.867	-0.73327	-0.33055
226.884	-0.73329	-0.33054
226.9	-0.73331	-0.33054
226.917	-0.73334	-0.33055
226.934	-0.73337	-0.33055
226.95	-0.7334	-0.33055
226.967	-0.73343	-0.33056
226.984	-0.73346	-0.33056
227	-0.73349	-0.33057
227.017	-0.73352	-0.33057
227.034	-0.73355	-0.33058
227.05	-0.73358	-0.33059

227.067	-0.7336	-0.3306
227.084	-0.73363	-0.3306
227.101	-0.73366	-0.33061
227.118	-0.73369	-0.33062
227.135	-0.73371	-0.33062
227.152	-0.73373	-0.33062
227.168	-0.73375	-0.33062
227.185	-0.73378	-0.33062
227.201	-0.73381	-0.33062
227.218	-0.73384	-0.33061
227.235	-0.73387	-0.33061
227.251	-0.73391	-0.33059
227.268	-0.73396	-0.33058
227.285	-0.73401	-0.33057
227.301	-0.73404	-0.33057
227.318	-0.73408	-0.33057
227.335	-0.73412	-0.33057
227.352	-0.73416	-0.33057
227.368	-0.73419	-0.33057
227.385	-0.73423	-0.33057
227.402	-0.73426	-0.33057
227.419	-0.73429	-0.33056
227.435	-0.73433	-0.33055
227.451	-0.73437	-0.33052
227.469	-0.73441	-0.33049
227.486	-0.73445	-0.33047
227.503	-0.73449	-0.33044
227.519	-0.73452	-0.33043
227.536	-0.73455	-0.33041
227.553	-0.73458	-0.3304

227.57	-0.73462	-0.33038
227.587	-0.73465	-0.33037
227.603	-0.73468	-0.33035
227.619	-0.73471	-0.33032
227.636	-0.73475	-0.33028
227.653	-0.73479	-0.33025
227.67	-0.73483	-0.33022
227.687	-0.73487	-0.33017
227.703	-0.73491	-0.33013
227.719	-0.73495	-0.33008
227.737	-0.73499	-0.33005
227.754	-0.73503	-0.33
227.77	-0.73506	-0.32994
227.786	-0.73509	-0.32985
227.804	-0.73512	-0.32975
227.82	-0.73515	-0.32964
227.836	-0.73519	-0.32951
227.853	-0.73522	-0.3294
227.87	-0.73525	-0.32928
227.887	-0.73529	-0.32919
227.904	-0.73533	-0.32913
227.92	-0.73537	-0.32909
227.936	-0.73541	-0.32906
227.953	-0.73545	-0.32899
227.971	-0.73548	-0.32889
227.987	-0.73552	-0.32879
228.003	-0.73555	-0.32867
228.02	-0.73559	-0.3285
228.037	-0.73562	-0.32831
228.053	-0.73566	-0.32807

228.071	-0.7357	-0.32784
228.087	-0.73574	-0.32765
228.104	-0.73578	-0.32748
228.121	-0.73581	-0.32732
228.138	-0.73584	-0.32713
228.155	-0.73587	-0.32684

For XRD patterns:

2 Theta	SA	L-SA
5	0	0.10667
5.02	0	0.10667
5.04	0.122	0.10667
5.06	0.08	0.10667
5.08	0	0.10667
5.1	0	0.12667
5.12	0	0.15333
5.14	0.09	0.17333
5.16	0.078	0.16
5.18	0.028	0.13333
5.2	0.11	0.1
5.22	0	0.06
5.24	0	0.03333
5.26	0	0.02
5.28	0	0.00667
5.3	0	0.00667
5.32	0	0
5.34	0	0.01333
5.36	0	0.04
5.38	0	0.06
5.4	0	0.08

5.42	0	0.08667
5.44	0	0.07333
5.46	0	0.05333
5.48	0	0.02667
5.5	0	0.00667
5.52	0	0
5.54	0	0.01333
5.56	0	0.04667
5.58	0	0.08
5.6	0	0.11333
5.62	0	0.12667
5.64	0	0.11333
5.66	0	0.08
5.68	0	0.04667
5.7	0	0.02
5.72	0	0
5.74	0	0
5.76	0.004	0
5.78	0	0
5.8	0	0
5.82	0	0
5.84	0.002	0
5.86	0.082	0
5.88	0	0.00667
5.9	0.006	0.02
5.92	0.032	0.04
5.94	0	0.05333
5.96	0	0.06
5.98	0	0.06667
6	0.14	0.05333

6.02	0	0.02667
6.04	0	0.00667
6.06	0.042	0.00667
6.08	0.042	0.04667
6.1	0	0.12
6.12	0	0.20667
6.14	0.014	0.28667
6.16	0	0.30667
6.18	0	0.26667
6.2	0	0.18
6.22	0	0.09333
6.24	0	0.03333
6.26	0.02	0.01333
6.28	0	0.02667
6.3	0	0.06667
6.32	0	0.09333
6.34	0.062	0.12667
6.36	0	0.15333
6.38	0	0.17333
6.4	0.044	0.18667
6.42	0.032	0.17333
6.44	0	0.12667
6.46	0.042	0.08667
6.48	0.072	0.04
6.5	0	0.02
6.52	0.134	0.02667
6.54	0.122	0.05333
6.56	0.018	0.08
6.58	0	0.09333
6.6	0	0.10667

6.62	0	0.12
6.64	0	0.12667
6.66	0	0.16
6.68	0.008	0.18667
6.7	0.052	0.24
6.72	0.04	0.27333
6.74	0.27	0.3
6.76	0.184	0.36
6.78	0.21	0.35333
6.8	0.048	0.34
6.82	0	0.33333
6.84	0	0.3
6.86	0.196	0.29333
6.88	0.13	0.30667
6.9	0	0.36667
6.92	0	0.48667
6.94	0.098	0.55333
6.96	0.034	0.62
6.98	0.084	0.62
7	0	0.56
7.02	0.056	0.45333
7.04	0	0.32667
7.06	0.012	0.22
7.08	0.17	0.16
7.1	0	0.09333
7.12	0	0.09333
7.14	0.01	0.12667
7.16	0.006	0.19333
7.18	0.134	0.29333
7.2	0.15	0.40667

7.22	0.096	0.49333
7.24	0	0.55333
7.26	0.024	0.58667
7.28	0	0.62667
7.3	0.028	0.66
7.32	0	0.67333
7.34	0.074	0.66667
7.36	0	0.62667
7.38	0.176	0.55333
7.4	0.128	0.46667
7.42	0.208	0.38
7.44	0	0.33333
7.46	0.108	0.32
7.48	0.014	0.36667
7.5	0.068	0.46
7.52	0.176	0.54
7.54	0.136	0.62
7.56	0.216	0.64
7.58	0.098	0.59333
7.6	0.22	0.54667
7.62	0.126	0.54
7.64	0.27	0.59333
7.66	0.268	0.69333
7.68	0.436	0.73333
7.7	0.312	0.72
7.72	0.064	0.59333
7.74	0.254	0.45333
7.76	0.58	0.35333
7.78	0.386	0.32667
7.8	0.418	0.38667

7.82	0.15	0.50667
7.84	0.18	0.6
7.86	0.34	0.61333
7.88	0.208	0.56667
7.9	0.614	0.54667
7.92	0.31	0.46667
7.94	0.534	0.42667
7.96	0.224	0.42
7.98	0.536	0.47333
8	0.35	0.54
8.02	0.406	0.58
8.04	0.564	0.60667
8.06	0.526	0.66
8.08	0.486	0.64667
8.1	0.388	0.68
8.12	0.574	0.72
8.14	0.37	0.76
8.16	0.776	0.79333
8.18	0.502	0.77333
8.2	0.92	0.7
8.22	0.342	0.62667
8.24	0.836	0.53333
8.26	0.732	0.47333
8.28	0.864	0.47333
8.3	0.888	0.47333
8.32	0.946	0.52
8.34	1.016	0.61333
8.36	1.254	0.7
8.38	1.18	0.78667
8.4	1.072	0.81333

8.42	1.426	0.82
8.44	1.596	0.81333
8.46	1.592	0.72667
8.48	1.71	0.61333
8.5	1.746	0.54667
8.52	1.82	0.47333
8.54	2.096	0.44
8.56	1.942	0.43333
8.58	2.384	0.51333
8.6	2.036	0.62667
8.62	2.266	0.7
8.64	2.488	0.71333
8.66	2.424	0.7
8.68	2.834	0.61333
8.7	2.58	0.52
8.72	2.87	0.42667
8.74	2.622	0.37333
8.76	2.516	0.35333
8.78	2.724	0.35333
8.8	2.344	0.37333
8.82	2.742	0.41333
8.84	2.122	0.47333
8.86	2.212	0.57333
8.88	1.964	0.66
8.9	2.016	0.7
8.92	1.89	0.67333
8.94	1.616	0.60667
8.96	1.532	0.48
8.98	1.672	0.34667
9	0.944	0.23333

9.02	1.234	0.17333
9.04	0.8	0.18
9.06	1.076	0.23333
9.08	0.886	0.30667
9.1	0.672	0.40667
9.12	0.626	0.48667
9.14	0.412	0.54
9.16	0.39	0.51333
9.18	0.508	0.45333
9.2	0.592	0.38
9.22	0.392	0.32
9.24	0.448	0.26
9.26	0.528	0.24
9.28	0.344	0.22667
9.3	0.314	0.20667
9.32	0.212	0.17333
9.34	0.318	0.14667
9.36	0.152	0.12
9.38	0.218	0.14
9.4	0.026	0.16
9.42	0.106	0.22
9.44	0.2	0.29333
9.46	0.086	0.34
9.48	0.204	0.38
9.5	0.204	0.38667
9.52	0.11	0.36
9.54	0.164	0.34
9.56	0.224	0.28
9.58	0.068	0.22667
9.6	0.11	0.18667

9.62	0.216	0.14667
9.64	0.062	0.10667
9.66	0.08	0.1
9.68	0.18	0.11333
9.7	0.046	0.16
9.72	0.344	0.19333
9.74	0.228	0.24667
9.76	0.11	0.26667
9.78	0.118	0.27333
9.8	0.128	0.26
9.82	0.014	0.24667
9.84	0.084	0.23333
9.86	0.006	0.23333
9.88	0.002	0.26
9.9	0.188	0.30667
9.92	0.056	0.31333
9.94	0.188	0.32667
9.96	0.014	0.32667
9.98	0.13	0.26667
10	0.164	0.21333
10.02	0.07	0.17333
10.04	0.104	0.14
10.06	0.166	0.11333
10.08	0.02	0.10667
10.1	0.05	0.12
10.12	0.01	0.14667
10.14	0.024	0.18
10.16	0.112	0.25333
10.18	0	0.31333
10.2	0	0.36667

10.22	0	0.34667
10.24	0.166	0.28667
10.26	0	0.22667
10.28	0	0.21333
10.3	0.17	0.22667
10.32	0.028	0.28667
10.34	0.026	0.35333
10.36	0.046	0.39333
10.38	0	0.35333
10.4	0	0.30667
10.42	0	0.27333
10.44	0.072	0.27333
10.46	0	0.24667
10.48	0.1	0.23333
10.5	0.05	0.20667
10.52	0.08	0.16
10.54	0	0.12
10.56	0	0.10667
10.58	0	0.10667
10.6	0	0.11333
10.62	0	0.15333
10.64	0	0.2
10.66	0.178	0.24667
10.68	0.08	0.3
10.7	0	0.35333
10.72	0.072	0.34667
10.74	0.108	0.26667
10.76	0.04	0.19333
10.78	0	0.16667
10.8	0.05	0.15333

10.82	0.082	0.18
10.84	0.024	0.25333
10.86	0.142	0.31333
10.88	0.05	0.36667
10.9	0	0.36
10.92	0.1	0.36667
10.94	0.068	0.41333
10.96	0.062	0.42
10.98	0.006	0.44667
11	0.086	0.48
11.02	0.084	0.5
11.04	0	0.51333
11.06	0.18	0.48
11.08	0.16	0.46
11.1	0.114	0.45333
11.12	0.2	0.43333
11.14	0.232	0.44
11.16	0.108	0.44667
11.18	0.252	0.46667
11.2	0.418	0.44
11.22	0.26	0.36667
11.24	0.422	0.32
11.26	0.45	0.31333
11.28	0.536	0.35333
11.3	0.602	0.44667
11.32	0.808	0.54
11.34	0.65	0.62667
11.36	0.972	0.64
11.38	1.114	0.57333
11.4	1.05	0.50667

11.42	1.182	0.44
11.44	1.322	0.44667
11.46	1.63	0.46667
11.48	1.48	0.5
11.5	1.704	0.52667
11.52	1.68	0.55333
11.54	1.844	0.54
11.56	1.838	0.50667
11.58	1.808	0.46667
11.6	1.954	0.47333
11.62	2.15	0.44667
11.64	1.892	0.44
11.66	1.994	0.45333
11.68	2.354	0.49333
11.7	1.91	0.54667
11.72	1.946	0.56667
11.74	1.832	0.56667
11.76	1.782	0.56667
11.78	1.786	0.50667
11.8	1.396	0.45333
11.82	1.642	0.40667
11.84	1.442	0.4
11.86	1.304	0.45333
11.88	1.032	0.48
11.9	0.994	0.54
11.92	0.944	0.6
11.94	1.06	0.60667
11.96	0.548	0.58
11.98	0.888	0.52667
12	0.946	0.49333

12.02	0.594	0.48667
12.04	0.69	0.46
12.06	0.686	0.48
12.08	0.624	0.49333
12.1	0.452	0.54
12.12	0.624	0.6
12.14	0.544	0.67333
12.16	0.444	0.77333
12.18	0.588	0.86
12.2	0.496	0.87333
12.22	0.57	0.81333
12.24	0.532	0.72667
12.26	0.448	0.64667
12.28	0.376	0.56667
12.3	0.504	0.52667
12.32	0.596	0.57333
12.34	0.532	0.63333
12.36	0.498	0.69333
12.38	0.564	0.74
12.4	0.692	0.82667
12.42	0.564	0.88667
12.44	0.286	0.88667
12.46	0.436	0.86
12.48	0.696	0.86
12.5	0.39	0.80667
12.52	0.512	0.72667
12.54	0.606	0.65333
12.56	0.532	0.66667
12.58	0.642	0.67333
12.6	0.58	0.65333

12.62	0.514	0.66667
12.64	0.646	0.72667
12.66	0.676	0.76667
12.68	0.498	0.78667
12.7	0.75	0.78
12.72	0.83	0.82
12.74	0.83	0.83333
12.76	0.628	0.82
12.78	0.68	0.80667
12.8	0.784	0.81333
12.82	0.89	0.84667
12.84	0.694	0.85333
12.86	0.786	0.81333
12.88	0.84	0.78
12.9	0.676	0.75333
12.92	0.654	0.7
12.94	0.892	0.64
12.96	0.698	0.63333
12.98	0.712	0.69333
13	0.816	0.77333
13.02	0.75	0.84667
13.04	0.84	0.90667
13.06	0.732	0.95333
13.08	0.706	0.94
13.1	0.672	0.88
13.12	0.672	0.83333
13.14	0.59	0.78
13.16	0.58	0.75333
13.18	0.662	0.71333
13.2	0.648	0.68

13.22	0.578	0.68
13.24	0.324	0.68
13.26	0.444	0.73333
13.28	0.522	0.80667
13.3	0.284	0.88667
13.32	0.35	0.98667
13.34	0.274	1.04
13.36	0.432	1.07333
13.38	0.448	1.07333
13.4	0.376	1.00667
13.42	0.322	0.93333
13.44	0.288	0.82667
13.46	0.14	0.69333
13.48	0.334	0.63333
13.5	0.244	0.62
13.52	0.122	0.68667
13.54	0.35	0.8
13.56	0.228	0.90667
13.58	0.344	1.04
13.6	0.194	1.11333
13.62	0.184	1.10667
13.64	0.342	1.09333
13.66	0.148	1.07333
13.68	0.046	1.06
13.7	0.118	1.02667
13.72	0.406	1.00667
13.74	0.154	1.04667
13.76	0.138	1.09333
13.78	0.154	1.12
13.8	0.2	1.12667

13.82	0.17	1.12
13.84	0.12	1.06667
13.86	0.124	0.92
13.88	0.208	0.78667
13.9	0.036	0.71333
13.92	0.1	0.73333
13.94	0.082	0.80667
13.96	0.026	0.91333
13.98	0.07	1.00667
14	0.046	1.05333
14.02	0.202	1.04667
14.04	0.238	1.04667
14.06	0.086	1.03333
14.08	0.106	1.07333
14.1	0.158	1.13333
14.12	0	1.15333
14.14	0.028	1.12
14.16	0.096	1.02667
14.18	0.082	0.96667
14.2	0.096	0.98
14.22	0	0.97333
14.24	0	0.98
14.26	0.082	1.01333
14.28	0.134	0.99333
14.3	0.034	0.93333
14.32	0.054	0.84
14.34	0	0.79333
14.36	0.01	0.80667
14.38	0.066	0.78667
14.4	0.014	0.74667

14.42	0	0.72667
14.44	0.096	0.66667
14.46	0.122	0.60667
14.48	0.054	0.58
14.5	0.16	0.6
14.52	0.182	0.68667
14.54	0.06	0.76667
14.56	0.056	0.83333
14.58	0.1	0.84667
14.6	0.012	0.76667
14.62	0.108	0.62667
14.64	0.052	0.50667
14.66	0.036	0.44
14.68	0.006	0.47333
14.7	0	0.53333
14.72	0.21	0.6
14.74	0.12	0.64667
14.76	0.082	0.66
14.78	0.036	0.62667
14.8	0.032	0.58
14.82	0.122	0.54
14.84	0.224	0.51333
14.86	0.136	0.44667
14.88	0.064	0.36667
14.9	0	0.33333
14.92	0.116	0.30667
14.94	0.158	0.30667
14.96	0.216	0.34667
14.98	0.05	0.38667
15	0.048	0.40667

15.02	0.096	0.38
15.04	0.134	0.36667
15.06	0.06	0.38667
15.08	0.076	0.38667
15.1	0.076	0.41333
15.12	0.062	0.45333
15.14	0.06	0.43333
15.16	0.112	0.35333
15.18	0.116	0.24667
15.2	0.118	0.18667
15.22	0.142	0.16667
15.24	0.046	0.18667
15.26	0.09	0.26
15.28	0.006	0.31333
15.3	0	0.30667
15.32	0.092	0.26
15.34	0.422	0.22
15.36	0.294	0.21333
15.38	0.008	0.20667
15.4	0	0.22
15.42	0.242	0.24
15.44	0.198	0.20667
15.46	0.186	0.16667
15.48	0.044	0.14
15.5	0.254	0.13333
15.52	0.244	0.14
15.54	0.202	0.14
15.56	0.088	0.14
15.58	0.066	0.12
15.6	0.176	0.07333

15.62	0.176	0.04667
15.64	0.17	0.04
15.66	0.278	0.06
15.68	0.28	0.09333
15.7	0.128	0.14667
15.72	0.076	0.18
15.74	0.368	0.18
15.76	0.272	0.14
15.78	0.122	0.11333
15.8	0.354	0.11333
15.82	0.466	0.12
15.84	0.192	0.12
15.86	0.21	0.13333
15.88	0.384	0.13333
15.9	0.32	0.11333
15.92	0.254	0.10667
15.94	0.39	0.14
15.96	0.414	0.19333
15.98	0.24	0.22667
16	0.22	0.22667
16.02	0.294	0.22667
16.04	0.412	0.17333
16.06	0.386	0.12667
16.08	0.296	0.11333
16.1	0.328	0.12
16.12	0.382	0.12667
16.14	0.362	0.13333
16.16	0.428	0.14
16.18	0.396	0.14
16.2	0.424	0.11333

16.22	0.438	0.09333
16.24	0.422	0.08667
16.26	0.474	0.08
16.28	0.49	0.09333
16.3	0.454	0.11333
16.32	0.586	0.13333
16.34	0.35	0.12667
16.36	0.258	0.09333
16.38	0.592	0.06667
16.4	0.814	0.06
16.42	0.462	0.07333
16.44	0.288	0.12
16.46	0.678	0.18667
16.48	0.726	0.24667
16.5	0.504	0.25333
16.52	0.586	0.20667
16.54	0.594	0.14
16.56	0.526	0.08667
16.58	0.59	0.04667
16.6	0.768	0.04667
16.62	0.782	0.07333
16.64	0.63	0.11333
16.66	0.538	0.14
16.68	0.824	0.14
16.7	0.974	0.12
16.72	0.762	0.10667
16.74	0.746	0.1
16.76	0.974	0.10667
16.78	1.016	0.10667
16.8	1.094	0.10667

16.82	0.992	0.13333
16.84	1.136	0.16667
16.86	1.348	0.16667
16.88	1.356	0.18
16.9	1.548	0.2
16.92	1.58	0.17333
16.94	1.58	0.14
16.96	1.694	0.12667
16.98	1.924	0.14
17	2.158	0.18667
17.02	2.18	0.19333
17.04	2.294	0.2
17.06	2.364	0.20667
17.08	2.41	0.15333
17.1	2.614	0.11333
17.12	2.96	0.09333
17.14	2.786	0.12
17.16	2.98	0.16
17.18	3.066	0.16
17.2	3.268	0.17333
17.22	3.292	0.18667
17.24	3.224	0.16667
17.26	3.402	0.16667
17.28	3.648	0.18667
17.3	3.326	0.22667
17.32	3.234	0.24667
17.34	3.468	0.24
17.36	3.552	0.24667
17.38	3.336	0.24667
17.4	3.286	0.22667

17.42	3.002	0.22
17.44	3.17	0.23333
17.46	3.186	0.21333
17.48	3.09	0.16
17.5	2.896	0.10667
17.52	2.756	0.08667
17.54	2.722	0.09333
17.56	2.848	0.12
17.58	2.946	0.17333
17.6	2.806	0.21333
17.62	2.816	0.21333
17.64	2.862	0.16
17.66	2.978	0.10667
17.68	2.912	0.08
17.7	2.672	0.09333
17.72	2.702	0.14
17.74	2.81	0.21333
17.76	2.678	0.28667
17.78	2.858	0.34667
17.8	2.982	0.34
17.82	2.854	0.3
17.84	2.816	0.25333
17.86	3.134	0.22
17.88	3.06	0.18667
17.9	2.944	0.15333
17.92	2.96	0.15333
17.94	3.068	0.20667
17.96	3.258	0.26667
17.98	3.086	0.33333
18	3.242	0.39333

18.02	3.342	0.46
18.04	3.39	0.5
18.06	3.488	0.48667
18.08	3.572	0.46
18.1	3.516	0.43333
18.12	3.484	0.38
18.14	3.614	0.31333
18.16	3.718	0.25333
18.18	3.816	0.22667
18.2	4.12	0.22667
18.22	3.898	0.24667
18.24	3.95	0.26667
18.26	4.234	0.26667
18.28	3.96	0.24667
18.3	4.206	0.24667
18.32	4.056	0.26
18.34	4.304	0.27333
18.36	4.254	0.31333
18.38	4.216	0.35333
18.4	4.088	0.36
18.42	4.09	0.33333
18.44	4.266	0.30667
18.46	4.592	0.29333
18.48	4.294	0.26
18.5	4.17	0.22667
18.52	4.07	0.22667
18.54	4.35	0.24
18.56	4.498	0.24
18.58	4.436	0.24
18.6	4.254	0.26667

18.62	4.092	0.29333
18.64	4.218	0.31333
18.66	4.232	0.35333
18.68	4.098	0.40667
18.7	4.12	0.45333
18.72	4.186	0.44667
18.74	4.148	0.42667
18.76	4.074	0.42
18.78	3.834	0.38
18.8	3.816	0.34667
18.82	3.748	0.32667
18.84	3.762	0.30667
18.86	3.706	0.27333
18.88	3.518	0.23333
18.9	3.642	0.21333
18.92	3.758	0.21333
18.94	3.638	0.22
18.96	3.616	0.25333
18.98	3.474	0.29333
19	3.482	0.31333
19.02	3.388	0.32
19.04	3.29	0.32
19.06	3.412	0.32667
19.08	3.318	0.34667
19.1	3.47	0.38
19.12	3.268	0.41333
19.14	3.354	0.38667
19.16	3.324	0.32
19.18	3.406	0.21333
19.2	3.174	0.12667

19.22	3.2	0.07333
19.24	3.384	0.09333
19.26	3.298	0.14667
19.28	3.452	0.22
19.3	3.698	0.26667
19.32	3.48	0.28667
19.34	3.124	0.26667
19.36	3.486	0.25333
19.38	3.63	0.26
19.4	3.752	0.28667
19.42	3.504	0.29333
19.44	3.528	0.28667
19.46	3.322	0.24667
19.48	3.222	0.19333
19.5	3.722	0.13333
19.52	3.736	0.10667
19.54	3.644	0.11333
19.56	3.504	0.14
19.58	3.416	0.16667
19.6	3.562	0.19333
19.62	3.532	0.19333
19.64	3.666	0.18
19.66	3.566	0.16667
19.68	3.406	0.15333
19.7	3.512	0.14667
19.72	3.44	0.15333
19.74	3.614	0.18667
19.76	3.476	0.22
19.78	3.548	0.24
19.8	3.446	0.25333

19.82	3.214	0.26
19.84	3.182	0.25333
19.86	3.46	0.24667
19.88	3.39	0.24
19.9	3.414	0.24
19.92	3.448	0.22
19.94	3.314	0.20667
19.96	3.216	0.22667
19.98	3.488	0.25333
20	3.428	0.28667
20.02	3.412	0.29333
20.04	3.134	0.27333
20.06	3.054	0.22
20.08	3.16	0.14667
20.1	3.45	0.09333
20.12	3.222	0.08
20.14	3.07	0.07333
20.16	3.206	0.06667
20.18	3.164	0.06
20.2	3.25	0.06667
20.22	2.936	0.09333
20.24	3.02	0.11333
20.26	3.114	0.13333
20.28	3.044	0.16667
20.3	3.046	0.18
20.32	2.976	0.17333
20.34	2.872	0.17333
20.36	2.88	0.2
20.38	3.018	0.22667
20.4	2.896	0.22667

20.42	2.994	0.20667
20.44	2.994	0.19333
20.46	2.762	0.17333
20.48	2.696	0.15333
20.5	2.926	0.12667
20.52	2.98	0.13333
20.54	2.956	0.16
20.56	2.952	0.16
20.58	2.952	0.16667
20.6	2.746	0.15333
20.62	2.962	0.13333
20.64	3.182	0.09333
20.66	3.278	0.05333
20.68	3.008	0.02667
20.7	3.14	0.03333
20.72	3.458	0.03333
20.74	3.712	0.06
20.76	3.67	0.08667
20.78	3.42	0.12
20.8	3.868	0.13333
20.82	3.938	0.14667
20.84	4.112	0.14
20.86	4.116	0.14
20.88	4.254	0.12
20.9	4.558	0.12667
20.92	4.618	0.13333
20.94	4.784	0.15333
20.96	5.054	0.16667
20.98	5.152	0.18
21	5.074	0.16

21.02	5.066	0.14
21.04	5.34	0.1
21.06	5.406	0.06
21.08	5.678	0.03333
21.1	5.734	0.03333
21.12	5.33	0.06
21.14	5.712	0.10667
21.16	5.738	0.15333
21.18	5.718	0.19333
21.2	5.502	0.18667
21.22	5.404	0.14
21.24	5.3	0.08667
21.26	5.474	0.06
21.28	5.508	0.04667
21.3	5.35	0.06667
21.32	5.218	0.11333
21.34	4.976	0.16
21.36	4.952	0.16
21.38	4.932	0.12667
21.4	4.692	0.08667
21.42	4.828	0.05333
21.44	4.668	0.01333
21.46	4.574	0
21.48	4.43	0.00667
21.5	4.688	0.04
21.52	4.424	0.06667
21.54	4.688	0.08667
21.56	4.34	0.11333
21.58	4.472	0.14
21.6	4.506	0.13333

21.62	4.654	0.12
21.64	4.714	0.12
21.66	4.72	0.13333
21.68	4.704	0.14667
21.7	4.572	0.15333
21.72	4.682	0.16667
21.74	4.7	0.16
21.76	5.008	0.12667
21.78	5.024	0.08667
21.8	4.776	0.06667
21.82	4.732	0.07333
21.84	4.618	0.08667
21.86	4.988	0.10667
21.88	5.234	0.11333
21.9	5.056	0.1
21.92	4.872	0.08
21.94	4.728	0.06667
21.96	4.764	0.08
21.98	4.704	0.1
22	4.766	0.14
22.02	4.758	0.16667
22.04	4.866	0.16
22.06	4.746	0.13333
22.08	4.58	0.10667
22.1	4.428	0.08667
22.12	4.34	0.06667
22.14	4.24	0.05333
22.16	4.308	0.06667
22.18	4.066	0.06667
22.2	3.996	0.08

22.22	4.094	0.1
22.24	4.116	0.13333
22.26	3.65	0.16667
22.28	3.816	0.16
22.3	3.694	0.13333
22.32	3.72	0.1
22.34	3.71	0.06667
22.36	3.53	0.03333
22.38	3.372	0.01333
22.4	3.222	0.00667
22.42	3.362	0.01333
22.44	3.386	0.00667
22.46	3.172	0
22.48	3.114	0
22.5	3.066	0
22.52	3.068	0.00667
22.54	2.95	0.02
22.56	2.894	0.04
22.58	3.038	0.06
22.6	2.896	0.08667
22.62	2.762	0.09333
22.64	2.71	0.07333
22.66	2.694	0.06
22.68	2.676	0.05333
22.7	2.588	0.06
22.72	2.484	0.07333
22.74	2.74	0.09333
22.76	2.684	0.10667
22.78	2.518	0.08667
22.8	2.728	0.06

22.82	2.444	0.05333
22.84	2.48	0.06667
22.86	2.49	0.08
22.88	2.498	0.10667
22.9	2.52	0.12667
22.92	2.536	0.13333
22.94	2.52	0.11333
22.96	2.272	0.08667
22.98	2.372	0.07333
23	2.416	0.08
23.02	2.454	0.06
23.04	2.364	0.05333
23.06	2.29	0.07333
23.08	2.408	0.10667
23.1	2.25	0.14
23.12	2.014	0.16667
23.14	2.14	0.18
23.16	2.27	0.18667
23.18	2.228	0.14
23.2	2.208	0.08667
23.22	2.144	0.03333
23.24	2.152	0.02667
23.26	2.01	0.07333
23.28	2.278	0.16
23.3	2.092	0.26
23.32	2.144	0.34667
23.34	1.928	0.37333
23.36	1.984	0.33333
23.38	1.884	0.24667
23.4	2.034	0.16

23.42	2.166	0.10667
23.44	1.922	0.08667
23.46	1.876	0.08667
23.48	1.746	0.12
23.5	1.75	0.14
23.52	1.862	0.15333
23.54	1.87	0.13333
23.56	1.754	0.11333
23.58	1.82	0.1
23.6	1.854	0.1
23.62	1.74	0.10667
23.64	1.776	0.14
23.66	1.732	0.14667
23.68	1.868	0.14667
23.7	1.756	0.11333
23.72	1.712	0.06667
23.74	1.538	0.02667
23.76	1.572	0.00667
23.78	1.75	0.01333
23.8	1.688	0.03333
23.82	1.592	0.05333
23.84	1.616	0.08667
23.86	1.6	0.11333
23.88	1.476	0.12667
23.9	1.466	0.13333
23.92	1.674	0.12667
23.94	1.436	0.1
23.96	1.528	0.06667
23.98	1.456	0.06667
24	1.534	0.09333

24.02	1.486	0.14
24.04	1.634	0.19333
24.06	1.478	0.21333
24.08	1.57	0.17333
24.1	1.584	0.12
24.12	1.514	0.08
24.14	1.382	0.08667
24.16	1.462	0.11333
24.18	1.584	0.16667
24.2	1.65	0.22
24.22	1.446	0.24
24.24	1.37	0.22
24.26	1.56	0.21333
24.28	1.696	0.22
24.3	1.556	0.26
24.32	1.546	0.26667
24.34	1.522	0.28667
24.36	1.47	0.28667
24.38	1.584	0.26667
24.4	1.572	0.22667
24.42	1.714	0.18
24.44	1.666	0.12667
24.46	1.716	0.1
24.48	1.652	0.07333
24.5	1.574	0.08667
24.52	1.65	0.10667
24.54	1.668	0.11333
24.56	1.724	0.11333
24.58	1.89	0.12667
24.6	1.594	0.14667

24.62	1.66	0.18
24.64	1.678	0.21333
24.66	1.76	0.24667
24.68	1.924	0.24667
24.7	1.89	0.2
24.72	1.764	0.14667
24.74	1.828	0.11333
24.76	1.702	0.11333
24.78	1.83	0.10667
24.8	1.912	0.10667
24.82	1.948	0.11333
24.84	1.706	0.09333
24.86	1.676	0.07333
24.88	1.776	0.08
24.9	1.752	0.12
24.92	1.918	0.17333
24.94	1.742	0.2
24.96	1.898	0.21333
24.98	1.896	0.20667
25	1.832	0.15333
25.02	1.792	0.10667
25.04	1.88	0.06667
25.06	1.8	0.06667
25.08	1.874	0.08667
25.1	1.728	0.12667
25.12	1.734	0.17333
25.14	1.854	0.22
25.16	1.824	0.24667
25.18	2.12	0.24
25.2	1.924	0.20667

25.22	1.872	0.18
25.24	1.72	0.17333
25.26	1.798	0.19333
25.28	1.886	0.2
25.3	1.932	0.23333
25.32	1.734	0.26667
25.34	1.68	0.25333
25.36	1.836	0.20667
25.38	1.72	0.17333
25.4	1.784	0.16667
25.42	1.848	0.18
25.44	1.76	0.19333
25.46	1.596	0.21333
25.48	1.496	0.22667
25.5	1.552	0.19333
25.52	1.596	0.14
25.54	1.772	0.12
25.56	1.7	0.12
25.58	1.402	0.15333
25.6	1.55	0.19333
25.62	1.524	0.22667
25.64	1.692	0.26667
25.66	1.562	0.3
25.68	1.448	0.31333
25.7	1.496	0.31333
25.72	1.24	0.28
25.74	1.4	0.23333
25.76	1.542	0.19333
25.78	1.54	0.18667
25.8	1.56	0.23333

25.82	1.404	0.32
25.84	1.448	0.37333
25.86	1.206	0.38
25.88	1.576	0.32667
25.9	1.348	0.24
25.92	1.346	0.15333
25.94	1.25	0.1
25.96	1.38	0.08667
25.98	1.34	0.12
26	1.416	0.14667
26.02	1.17	0.2
26.04	1.406	0.25333
26.06	1.44	0.3
26.08	1.508	0.32
26.1	1.196	0.3
26.12	1.278	0.24667
26.14	1.132	0.2
26.16	1.4	0.16
26.18	1.32	0.14
26.2	1.442	0.11333
26.22	1.334	0.1
26.24	1.44	0.07333
26.26	1.428	0.04
26.28	1.544	0.03333
26.3	1.316	0.05333
26.32	1.218	0.09333
26.34	1.258	0.13333
26.36	1.312	0.14667
26.38	1.52	0.14667
26.4	1.324	0.12667

26.42	1.368	0.12
26.44	1.402	0.13333
26.46	1.448	0.16
26.48	1.222	0.19333
26.5	1.228	0.21333
26.52	1.182	0.21333
26.54	1.18	0.2
26.56	1.258	0.18
26.58	1.24	0.18
26.6	1.222	0.19333
26.62	1.256	0.21333
26.64	1.166	0.25333
26.66	1.386	0.26
26.68	1.26	0.25333
26.7	1.21	0.22
26.72	1.142	0.16667
26.74	1.196	0.12
26.76	1.088	0.08667
26.78	1.162	0.06
26.8	1.26	0.06
26.82	1.3	0.05333
26.84	1.164	0.05333
26.86	1.154	0.05333
26.88	1.218	0.06
26.9	1.272	0.06
26.92	1.082	0.05333
26.94	1.148	0.04
26.96	1.264	0.02667
26.98	1.2	0.01333
27	1.2	0

27.02	1.074	0.00667
27.04	1.158	0.02667
27.06	1.182	0.06
27.08	1.206	0.09333
27.1	1.212	0.12667
27.12	1.242	0.14667
27.14	1.242	0.14
27.16	1.052	0.10667
27.18	1.088	0.06
27.2	1.224	0.02667
27.22	1.212	0.00667
27.24	1.176	0.01333
27.26	1.06	0.04667
27.28	0.98	0.1
27.3	1.126	0.15333
27.32	1.114	0.19333
27.34	1.136	0.20667
27.36	1.102	0.19333
27.38	1.092	0.16
27.4	1.11	0.12
27.42	0.956	0.10667
27.44	1.006	0.11333
27.46	0.996	0.13333
27.48	1.036	0.16
27.5	0.99	0.15333
27.52	1.012	0.12667
27.54	0.826	0.08
27.56	0.932	0.04
27.58	0.98	0.02
27.6	1.042	0.00667

27.62	0.94	0.02
27.64	0.816	0.04
27.66	0.848	0.06
27.68	0.732	0.07333
27.7	0.944	0.07333
27.72	1.03	0.06667
27.74	0.978	0.06
27.76	0.986	0.05333
27.78	0.822	0.05333
27.8	0.898	0.07333
27.82	0.884	0.11333
27.84	0.816	0.14
27.86	0.886	0.14667
27.88	0.82	0.14
27.9	0.944	0.14
27.92	0.848	0.12667
27.94	0.854	0.13333
27.96	1	0.16667
27.98	0.778	0.19333
28	0.946	0.2
28.02	0.808	0.16667
28.04	0.898	0.12
28.06	0.932	0.09333
28.08	0.87	0.08
28.1	0.82	0.08667
28.12	0.804	0.1
28.14	0.682	0.11333
28.16	0.786	0.13333
28.18	0.77	0.13333
28.2	0.786	0.13333

28.22	0.908	0.14667
28.24	1.004	0.16667
28.26	1.042	0.16
28.28	0.972	0.14
28.3	0.96	0.12
28.32	0.722	0.12
28.34	0.708	0.12667
28.36	0.948	0.14667
28.38	1.018	0.15333
28.4	0.726	0.15333
28.42	0.966	0.13333
28.44	0.836	0.09333
28.46	0.86	0.06
28.48	0.82	0.04
28.5	0.758	0.03333
28.52	0.864	0.03333
28.54	0.884	0.04
28.56	0.926	0.05333
28.58	0.92	0.06
28.6	0.94	0.07333
28.62	0.752	0.06
28.64	0.902	0.05333
28.66	0.918	0.04667
28.68	0.8	0.04667
28.7	0.738	0.05333
28.72	0.674	0.06
28.74	0.8	0.08
28.76	0.968	0.11333
28.78	0.914	0.11333
28.8	0.914	0.12

28.82	0.85	0.1
28.84	0.89	0.08667
28.86	0.946	0.06
28.88	0.68	0.04667
28.9	0.93	0.06
28.92	0.948	0.08
28.94	0.874	0.1
28.96	0.996	0.10667
28.98	0.784	0.08667
29	0.968	0.06
29.02	1.008	0.04
29.04	1.008	0.02667
29.06	1.126	0.03333
29.08	0.924	0.06
29.1	1.038	0.10667
29.12	1.06	0.14667
29.14	0.878	0.17333
29.16	0.96	0.19333
29.18	0.904	0.18
29.2	0.888	0.14667
29.22	1.02	0.10667
29.24	0.924	0.08667
29.26	0.958	0.09333
29.28	0.894	0.12
29.3	0.844	0.15333
29.32	0.918	0.17333
29.34	0.896	0.15333
29.36	1.066	0.11333
29.38	0.802	0.07333
29.4	0.896	0.04

29.42	0.82	0.02
29.44	0.87	0.02
29.46	0.774	0.02667
29.48	0.92	0.05333
29.5	0.706	0.09333
29.52	0.754	0.14
29.54	0.686	0.18667
29.56	0.774	0.20667
29.58	0.72	0.2
29.6	0.766	0.16
29.62	0.632	0.1
29.64	0.692	0.04667
29.66	0.684	0.01333
29.68	0.586	0.02
29.7	0.738	0.06
29.72	0.634	0.10667
29.74	0.624	0.16
29.76	0.51	0.18667
29.78	0.466	0.19333
29.8	0.536	0.18
29.82	0.504	0.18
29.84	0.572	0.18667
29.86	0.636	0.18
29.88	0.594	0.18667
29.9	0.664	0.19333
29.92	0.562	0.17333
29.94	0.496	0.16
29.96	0.508	0.15333
29.98	0.592	0.16
30	0.5	0.18

30.02	0.578	0.21333
30.04	0.53	0.26
30.06	0.556	0.29333
30.08	0.64	0.28
30.1	0.6	0.24
30.12	0.56	0.16667
30.14	0.446	0.09333
30.16	0.42	0.04
30.18	0.444	0.03333
30.2	0.398	0.05333
30.22	0.306	0.08667
30.24	0.614	0.11333
30.26	0.436	0.11333
30.28	0.444	0.10667
30.3	0.464	0.12
30.32	0.338	0.14
30.34	0.36	0.18
30.36	0.328	0.19333
30.38	0.45	0.19333
30.4	0.428	0.18667
30.42	0.426	0.16
30.44	0.442	0.17333
30.46	0.474	0.24
30.48	0.502	0.32
30.5	0.37	0.4
30.52	0.262	0.42
30.54	0.328	0.37333
30.56	0.118	0.28
30.58	0.192	0.14667
30.6	0.346	0.06667

30.62	0.296	0.05333
30.64	0.518	0.1
30.66	0.268	0.20667
30.68	0.302	0.3
30.7	0.258	0.34667
30.72	0.152	0.32
30.74	0.282	0.24
30.76	0.158	0.18667
30.78	0.266	0.14667
30.8	0.268	0.16667
30.82	0.186	0.23333
30.84	0.264	0.30667
30.86	0.248	0.36
30.88	0.148	0.37333
30.9	0.172	0.35333
30.92	0.226	0.32667
30.94	0.126	0.24667
30.96	0.198	0.18
30.98	0.194	0.10667
31	0.056	0.04667
31.02	0.15	0.03333
31.04	0.138	0.06667
31.06	0.256	0.15333
31.08	0.156	0.25333
31.1	0.05	0.34
31.12	0.104	0.42
31.14	0.01	0.42667
31.16	0.006	0.41333
31.18	0.19	0.40667
31.2	0.216	0.38

31.22	0.314	0.38
31.24	0.26	0.36
31.26	0.042	0.34
31.28	0.148	0.33333
31.3	0.106	0.28
31.32	0.04	0.24
31.34	0.27	0.21333
31.36	0.296	0.16
31.38	0.166	0.12667
31.4	0.314	0.1
31.42	0.038	0.09333
31.44	0.04	0.10667
31.46	0.222	0.11333
31.48	0.016	0.15333
31.5	0.164	0.20667
31.52	0.144	0.22667
31.54	0.058	0.26
31.56	0.262	0.27333
31.58	0.018	0.27333
31.6	0.002	0.26667
31.62	0.028	0.27333
31.64	0.038	0.31333
31.66	0.26	0.35333
31.68	0.098	0.36667
31.7	0.32	0.35333
31.72	0.156	0.29333
31.74	0.036	0.23333
31.76	0.148	0.17333
31.78	0.066	0.15333
31.8	0.082	0.17333

31.82	0.074	0.2
31.84	0.098	0.20667
31.86	0.184	0.20667
31.88	0.114	0.18667
31.9	0.07	0.19333
31.92	0.08	0.21333
31.94	0.092	0.25333
31.96	0.094	0.31333
31.98	0.212	0.35333
32	0.07	0.36
32.02	0.068	0.36667
32.04	0.056	0.35333
32.06	0.126	0.36
32.08	0.108	0.36
32.1	0.138	0.37333
32.12	0.004	0.37333
32.14	0	0.32667
32.16	0.036	0.28
32.18	0.014	0.21333
32.2	0.062	0.16667
32.22	0.13	0.15333
32.24	0.022	0.17333
32.26	0.132	0.22
32.28	0.146	0.24667
32.3	0	0.23333
32.32	0.134	0.22
32.34	0.078	0.17333
32.36	0.01	0.14667
32.38	0.116	0.16
32.4	0	0.18

32.42	0.052	0.22667
32.44	0.038	0.24667
32.46	0.018	0.24
32.48	0.078	0.22667
32.5	0.02	0.20667
32.52	0.048	0.19333
32.54	0.064	0.18
32.56	0.096	0.17333
32.58	0.024	0.17333
32.6	0	0.15333
32.62	0.016	0.13333
32.64	0.052	0.12
32.66	0	0.13333
32.68	0.038	0.15333
32.7	0.004	0.18667
32.72	0.002	0.22
32.74	0.106	0.26
32.76	0.008	0.26
32.78	0	0.26
32.8	0	0.26
32.82	0	0.28
32.84	0	0.32667
32.86	0	0.36667
32.88	0	0.39333
32.9	0	0.41333
32.92	0	0.36
32.94	0.042	0.26667
32.96	0	0.17333
32.98	0	0.11333
33	0.042	0.09333

33.02	0.028	0.12
33.04	0	0.18
33.06	0.08	0.26667
33.08	0.078	0.29333
33.1	0.04	0.28
33.12	0.13	0.24
33.14	0	0.2
33.16	0	0.16667
33.18	0	0.15333
33.2	0	0.14667
33.22	0	0.14667
33.24	0	0.10667
33.26	0.128	0.06667
33.28	0.1	0.04667
33.3	0.132	0.04
33.32	0	0.05333
33.34	0	0.08667
33.36	0	0.14
33.38	0	0.16667
33.4	0	0.16
33.42	0	0.15333
33.44	0.072	0.14
33.46	0	0.1
33.48	0.076	0.06667
33.5	0	0.04667
33.52	0	0.03333
33.54	0	0.02667
33.56	0	0.03333
33.58	0	0.06
33.6	0	0.10667

33.62	0.054	0.15333
33.64	0.012	0.19333
33.66	0	0.2
33.68	0.09	0.18
33.7	0.028	0.14
33.72	0.01	0.11333
33.74	0.058	0.10667
33.76	0.024	0.13333
33.78	0.048	0.16667
33.8	0	0.18
33.82	0	0.15333
33.84	0	0.11333
33.86	0	0.09333
33.88	0.072	0.09333
33.9	0.064	0.11333
33.92	0.106	0.14
33.94	0.034	0.16
33.96	0.018	0.14667
33.98	0.022	0.11333
34	0.094	0.08667
34.02	0.038	0.08
34.04	0.14	0.08
34.06	0	0.08
34.08	0	0.08
34.1	0	0.07333
34.12	0	0.06
34.14	0.03	0.06
34.16	0.09	0.06667
34.18	0	0.08
34.2	0.05	0.07333

34.22	0.006	0.05333
34.24	0.018	0.04
34.26	0.008	0.04667
34.28	0	0.08
34.3	0.068	0.15333
34.32	0.022	0.22667
34.34	0.008	0.28
34.36	0.036	0.29333
34.38	0.12	0.25333
34.4	0.054	0.18667
34.42	0	0.12667
34.44	0.074	0.09333
34.46	0	0.07333
34.48	0	0.06
34.5	0.036	0.05333
34.52	0.016	0.05333
34.54	0.03	0.05333
34.56	0.104	0.06
34.58	0	0.07333
34.6	0	0.07333
34.62	0.034	0.05333
34.64	0	0.03333
34.66	0	0.03333
34.68	0	0.06667
34.7	0.052	0.12
34.72	0.08	0.18
34.74	0.152	0.21333
34.76	0.05	0.2
34.78	0.04	0.13333
34.8	0.078	0.07333

34.82	0.036	0.04667
34.84	0.034	0.06
34.86	0.022	0.11333
34.88	0	0.18667
34.9	0.106	0.24667
34.92	0.086	0.25333
34.94	0.02	0.21333
34.96	0	0.14667
34.98	0.082	0.09333
35	0.012	0.06667
35.02	0.058	0.06667
35.04	0.13	0.10667
35.06	0.062	0.15333
35.08	0.002	0.17333
35.1	0.03	0.19333
35.12	0.068	0.2
35.14	0.12	0.21333
35.16	0.052	0.21333
35.18	0	0.2
35.2	0.012	0.19333
35.22	0.054	0.17333
35.24	0.03	0.14
35.26	0.072	0.12
35.28	0.122	0.09333
35.3	0.146	0.10667
35.32	0.232	0.12
35.34	0.102	0.13333
35.36	0.06	0.15333
35.38	0.018	0.16667
35.4	0.004	0.17333

35.42	0.078	0.18
35.44	0.158	0.18
35.46	0.094	0.2
35.48	0.146	0.2
35.5	0.26	0.19333
35.52	0.068	0.18667
35.54	0.098	0.18
35.56	0.066	0.17333
35.58	0.094	0.17333
35.6	0.128	0.18667
35.62	0.164	0.20667
35.64	0.162	0.21333
35.66	0.242	0.21333
35.68	0.194	0.20667
35.7	0.222	0.20667
35.72	0.2	0.20667
35.74	0.16	0.22667
35.76	0.188	0.26
35.78	0.232	0.29333
35.8	0.082	0.29333
35.82	0.042	0.28667
35.84	0.184	0.25333
35.86	0.254	0.22667
35.88	0.034	0.20667
35.9	0.244	0.22
35.92	0.14	0.25333
35.94	0.074	0.28
35.96	0.144	0.26667
35.98	0.066	0.23333
36	0.03	0.18

36.02	0.094	0.12667
36.04	0.138	0.1
36.06	0.086	0.1
36.08	0.126	0.12
36.1	0.184	0.13333
36.12	0.186	0.14
36.14	0.122	0.12667
36.16	0.102	0.11333
36.18	0.116	0.11333
36.2	0.05	0.12667
36.22	0.104	0.14667
36.24	0.144	0.16
36.26	0.064	0.16
36.28	0.13	0.13333
36.3	0.056	0.08667
36.32	0.002	0.04667
36.34	0.154	0.01333
36.36	0.146	0.00667
36.38	0.096	0.01333
36.4	0.166	0.03333
36.42	0.19	0.06667
36.44	0.166	0.1
36.46	0.016	0.12667
36.48	0.032	0.13333
36.5	0.14	0.13333
36.52	0.012	0.12667
36.54	0.04	0.11333
36.56	0.08	0.12
36.58	0.04	0.14667
36.6	0	0.18

36.62	0.11	0.2
36.64	0.068	0.19333
36.66	0.09	0.19333
36.68	0	0.17333
36.7	0.026	0.16667
36.72	0.07	0.18667
36.74	0.008	0.2
36.76	0.046	0.18667
36.78	0.08	0.15333
36.8	0.032	0.1
36.82	0.162	0.05333
36.84	0	0.02
36.86	0.078	0.02
36.88	0.07	0.06
36.9	0	0.1
36.92	0	0.13333
36.94	0	0.16
36.96	0.014	0.14667
36.98	0.084	0.11333
37	0.078	0.06667
37.02	0.086	0.03333
37.04	0.026	0.02667
37.06	0	0.06
37.08	0	0.11333
37.1	0	0.18
37.12	0.052	0.21333
37.14	0	0.21333
37.16	0	0.16667
37.18	0.062	0.10667
37.2	0.064	0.04667

37.22	0	0.02
37.24	0.022	0.02667
37.26	0.06	0.06
37.28	0	0.11333
37.3	0.012	0.17333
37.32	0	0.20667
37.34	0.022	0.21333
37.36	0.102	0.18667
37.38	0.078	0.16
37.4	0.042	0.11333
37.42	0	0.08
37.44	0.06	0.07333
37.46	0	0.08
37.48	0	0.08
37.5	0.006	0.08667
37.52	0.008	0.08
37.54	0	0.06667
37.56	0	0.05333
37.58	0.036	0.06667
37.6	0.036	0.08
37.62	0	0.10667
37.64	0.096	0.12
37.66	0.074	0.13333
37.68	0.084	0.12
37.7	0.054	0.11333
37.72	0.03	0.12
37.74	0.026	0.13333
37.76	0.02	0.14
37.78	0	0.14
37.8	0.092	0.14

37.82	0.056	0.12
37.84	0.07	0.1
37.86	0	0.08667
37.88	0.192	0.09333
37.9	0.032	0.10667
37.92	0.052	0.11333
37.94	0.024	0.12667
37.96	0.084	0.14
37.98	0	0.14667
38	0	0.15333
38.02	0	0.18
38.04	0.058	0.21333
38.06	0.046	0.24
38.08	0	0.24
38.1	0.06	0.23333
38.12	0	0.21333
38.14	0.072	0.16667
38.16	0.058	0.13333
38.18	0.03	0.13333
38.2	0	0.16
38.22	0	0.19333
38.24	0	0.22
38.26	0	0.22667
38.28	0	0.22
38.3	0	0.19333
38.32	0.096	0.18667
38.34	0	0.19333
38.36	0	0.21333
38.38	0.094	0.20667
38.4	0.002	0.17333

38.42	0	0.11333
38.44	0.026	0.06
38.46	0.038	0.02667
38.48	0.01	0.02667
38.5	0.028	0.06
38.52	0.038	0.10667
38.54	0	0.14667
38.56	0.044	0.16
38.58	0.012	0.15333
38.6	0.086	0.14
38.62	0.036	0.12667
38.64	0	0.1
38.66	0.004	0.08
38.68	0.066	0.06667
38.7	0.006	0.06
38.72	0.014	0.06
38.74	0	0.05333
38.76	0	0.06667
38.78	0	0.06667
38.8	0.088	0.06
38.82	0.02	0.07333
38.84	0.046	0.1
38.86	0.028	0.13333
38.88	0.094	0.17333
38.9	0	0.20667
38.92	0.054	0.24
38.94	0.074	0.23333
38.96	0.018	0.2
38.98	0.134	0.15333
39	0.116	0.11333

39.02	0.148	0.06667
39.04	0.116	0.04667
39.06	0.048	0.04667
39.08	0.04	0.07333
39.1	0.02	0.1
39.12	0.042	0.12667
39.14	0.2	0.13333
39.16	0.096	0.13333
39.18	0.044	0.10667
39.2	0.09	0.08
39.22	0.152	0.06667
39.24	0.036	0.07333
39.26	0.016	0.09333
39.28	0.18	0.11333
39.3	0.158	0.13333
39.32	0.132	0.15333
39.34	0.134	0.15333
39.36	0.152	0.15333
39.38	0.088	0.16667
39.4	0.032	0.2
39.42	0.116	0.22
39.44	0.266	0.22667
39.46	0.196	0.24
39.48	0.102	0.22
39.5	0.15	0.20667
39.52	0.232	0.2
39.54	0.128	0.21333
39.56	0.076	0.22667
39.58	0.218	0.22
39.6	0.188	0.21333

39.62	0.158	0.19333
39.64	0.152	0.15333
39.66	0.15	0.14
39.68	0.192	0.14667
39.7	0.078	0.16
39.72	0.068	0.18
39.74	0.128	0.2
39.76	0.194	0.20667
39.78	0.178	0.18
39.8	0.17	0.12667
39.82	0.272	0.07333
39.84	0.248	0.03333
39.86	0.15	0.01333
39.88	0.208	0.03333
39.9	0.118	0.1
39.92	0.08	0.17333
39.94	0.094	0.23333
39.96	0.158	0.26667
39.98	0.092	0.24667
40	0.262	0.22667
40.02	0.114	0.21333
40.04	0.288	0.24667
40.06	0.228	0.30667
40.08	0.168	0.37333
40.1	0.176	0.41333
40.12	0.072	0.40667
40.14	0.134	0.34
40.16	0.052	0.24667
40.18	0.11	0.14
40.2	0.086	0.06667

40.22	0.136	0.02667
40.24	0.148	0.01333
40.26	0.106	0.03333
40.28	0.17	0.06
40.3	0.204	0.07333
40.32	0.216	0.08667
40.34	0.174	0.1
40.36	0.072	0.1
40.38	0.054	0.09333
40.4	0.018	0.06667
40.42	0.086	0.04667
40.44	0.044	0.02667
40.46	0.062	0.02667
40.48	0.238	0.06667
40.5	0.186	0.13333
40.52	0.144	0.2
40.54	0.116	0.24
40.56	0.16	0.24667
40.58	0.088	0.23333
40.6	0.102	0.20667
40.62	0.126	0.2
40.64	0.146	0.23333
40.66	0.122	0.26
40.68	0.164	0.28
40.7	0.062	0.27333
40.72	0.278	0.22667
40.74	0.086	0.16
40.76	0.152	0.09333
40.78	0.082	0.05333
40.8	0.016	0.05333

40.82	0.046	0.08667
40.84	0.116	0.14
40.86	0.09	0.18667
40.88	0.054	0.22
40.9	0.022	0.20667
40.92	0.088	0.16
40.94	0.198	0.09333
40.96	0.196	0.04
40.98	0.112	0.02
41	0.136	0.02667
41.02	0.112	0.07333
41.04	0.05	0.14667
41.06	0	0.21333
41.08	0.022	0.26
41.1	0.056	0.29333
41.12	0.064	0.30667
41.14	0.174	0.28667
41.16	0.114	0.24667
41.18	0.156	0.2
41.2	0.006	0.16667
41.22	0	0.12667
41.24	0.014	0.09333
41.26	0.032	0.1
41.28	0	0.11333
41.3	0.06	0.12
41.32	0.09	0.12
41.34	0.132	0.13333
41.36	0.022	0.15333
41.38	0	0.16667
41.4	0.056	0.19333

41.42	0.068	0.24
41.44	0.018	0.25333
41.46	0.078	0.24
41.48	0.012	0.18667
41.5	0.09	0.13333
41.52	0	0.08
41.54	0	0.04667
41.56	0	0.04667
41.58	0.044	0.06
41.6	0	0.07333
41.62	0.06	0.08667
41.64	0	0.08667
41.66	0.044	0.09333
41.68	0	0.1
41.7	0	0.11333
41.72	0.01	0.12667
41.74	0.11	0.13333
41.76	0.046	0.12
41.78	0	0.11333
41.8	0	0.11333
41.82	0.002	0.14
41.84	0.028	0.18
41.86	0.018	0.21333
41.88	0.054	0.25333
41.9	0.062	0.26667
41.92	0.062	0.24
41.94	0.006	0.2
41.96	0	0.17333
41.98	0	0.16
42	0	0.17333

42.02	0	0.19333
42.04	0	0.23333
42.06	0	0.24
42.08	0	0.19333
42.1	0.034	0.14667
42.12	0.002	0.10667
42.14	0	0.07333
42.16	0	0.08
42.18	0	0.13333
42.2	0	0.21333
42.22	0	0.28
42.24	0	0.32667
42.26	0.05	0.32
42.28	0	0.27333
42.3	0.05	0.18
42.32	0.04	0.1
42.34	0.058	0.07333
42.36	0.052	0.08
42.38	0	0.13333
42.4	0	0.19333
42.42	0.068	0.23333
42.44	0.022	0.25333
42.46	0.006	0.23333
42.48	0	0.22
42.5	0.006	0.22
42.52	0	0.22667
42.54	0	0.23333
42.56	0	0.22
42.58	0.018	0.19333
42.6	0.052	0.15333

42.62	0.076	0.10667
42.64	0.066	0.08667
42.66	0.074	0.08667
42.68	0	0.12
42.7	0.042	0.16667
42.72	0.03	0.2
42.74	0.01	0.22667
42.76	0.074	0.23333
42.78	0.008	0.22667
42.8	0.008	0.2
42.82	0	0.16667
42.84	0.066	0.13333
42.86	0.082	0.12
42.88	0	0.1
42.9	0	0.10667
42.92	0.01	0.12
42.94	0.056	0.14667
42.96	0	0.15333
42.98	0	0.15333
43	0.066	0.15333
43.02	0.07	0.14667
43.04	0.13	0.11333
43.06	0.104	0.09333
43.08	0.124	0.09333
43.1	0.078	0.08
43.12	0.064	0.06
43.14	0.074	0.05333
43.16	0.052	0.04667
43.18	0.02	0.03333
43.2	0.006	0.02667

43.22	0.014	0.05333
43.24	0.07	0.11333
43.26	0.04	0.17333
43.28	0.134	0.24
43.3	0.038	0.29333
43.32	0.068	0.30667
43.34	0.06	0.28667
43.36	0.08	0.23333
43.38	0.098	0.18667
43.4	0.024	0.18
43.42	0.12	0.18667
43.44	0.074	0.24
43.46	0.112	0.29333
43.48	0.076	0.33333
43.5	0.03	0.33333
43.52	0.022	0.28667
43.54	0.012	0.22667
43.56	0.018	0.18
43.58	0.012	0.15333
43.6	0.002	0.17333
43.62	0.03	0.20667
43.64	0.004	0.22667
43.66	0	0.23333
43.68	0.052	0.21333
43.7	0.072	0.17333
43.72	0.1	0.11333
43.74	0.062	0.06667
43.76	0.096	0.04667
43.78	0.03	0.03333
43.8	0.092	0.04

43.82	0.028	0.07333
43.84	0.132	0.12667
43.86	0.158	0.17333
43.88	0.058	0.2
43.9	0	0.21333
43.92	0	0.19333
43.94	0	0.14
43.96	0	0.07333
43.98	0.022	0.04
44	0.028	0.03333
44.02	0.036	0.06
44.04	0	0.10667
44.06	0	0.17333
44.08	0.048	0.22
44.1	0	0.22667
44.12	0	0.20667
44.14	0.052	0.16667
44.16	0.048	0.12
44.18	0.01	0.08
44.2	0.028	0.05333
44.22	0.058	0.06667
44.24	0.026	0.1
44.26	0.016	0.15333
44.28	0.036	0.2
44.3	0.046	0.22
44.32	0.076	0.21333
44.34	0.036	0.2
44.36	0.008	0.16667
44.38	0.046	0.16
44.4	0.162	0.18

44.42	0.062	0.2
44.44	0.012	0.22667
44.46	0.036	0.23333
44.48	0.066	0.20667
44.5	0.004	0.17333
44.52	0.05	0.13333
44.54	0.026	0.10667
44.56	0.082	0.06667
44.58	0.066	0.04
44.6	0.068	0.04
44.62	0.06	0.06667
44.64	0.048	0.10667
44.66	0	0.17333
44.68	0.044	0.25333
44.7	0	0.31333
44.72	0.05	0.30667
44.74	0	0.26667
44.76	0.044	0.20667
44.78	0.012	0.14
44.8	0.13	0.07333
44.82	0.116	0.04
44.84	0.03	0.03333
44.86	0.052	0.04667
44.88	0	0.07333
44.9	0.028	0.1
44.92	0.002	0.12
44.94	0	0.13333
44.96	0.026	0.12667
44.98	0.01	0.12
45	0.008	0.10667

45.02	0	0.08
45.04	0	0.08
45.06	0	0.08
45.08	0.12	0.09333
45.1	0.01	0.12
45.12	0	0.14
45.14	0	0.16
45.16	0	0.18
45.18	0	0.18
45.2	0.034	0.18667
45.22	0	0.18
45.24	0	0.16
45.26	0	0.14
45.28	0.02	0.11333
45.3	0.024	0.1
45.32	0	0.10667
45.34	0	0.13333
45.36	0	0.17333
45.38	0.022	0.21333
45.4	0	0.24
45.42	0	0.23333
45.44	0	0.21333
45.46	0	0.16667
45.48	0	0.11333
45.5	0	0.08667
45.52	0	0.08
45.54	0.024	0.08667
45.56	0	0.11333
45.58	0.016	0.14
45.6	0.016	0.15333

45.62	0.142	0.13333
45.64	0.068	0.09333
45.66	0	0.06
45.68	0	0.04
45.7	0	0.04
45.72	0	0.06
45.74	0.002	0.08667
45.76	0	0.11333
45.78	0.04	0.10667
45.8	0	0.08
45.82	0	0.04667
45.84	0	0.02
45.86	0.09	0.00667
45.88	0.09	0.01333
45.9	0.044	0.03333
45.92	0.046	0.06667
45.94	0.108	0.1
45.96	0.022	0.13333
45.98	0.004	0.14
46	0	0.14
46.02	0	0.13333
46.04	0	0.12
46.06	0.024	0.10667
46.08	0.01	0.1
46.1	0.072	0.1
46.12	0.038	0.10667
46.14	0.058	0.12
46.16	0.038	0.13333
46.18	0.01	0.17333
46.2	0.07	0.21333

46.22	0.024	0.25333
46.24	0	0.28667
46.26	0	0.28
46.28	0.078	0.25333
46.3	0	0.21333
46.32	0	0.17333
46.34	0	0.16
46.36	0.014	0.16
46.38	0.038	0.19333
46.4	0	0.23333
46.42	0	0.26
46.44	0.028	0.26667
46.46	0.02	0.24667
46.48	0.07	0.2
46.5	0.002	0.14
46.52	0.068	0.08
46.54	0.084	0.03333
46.56	0.166	0.02
46.58	0.092	0.02667
46.6	0.054	0.08
46.62	0	0.13333
46.64	0.046	0.19333
46.66	0	0.24
46.68	0	0.24667
46.7	0	0.22
46.72	0.07	0.16667
46.74	0.042	0.12
46.76	0.02	0.1
46.78	0.018	0.10667
46.8	0.05	0.14667

46.82	0.202	0.2
46.84	0.048	0.22667
46.86	0.104	0.22
46.88	0.046	0.17333
46.9	0.024	0.12
46.92	0	0.05333
46.94	0.012	0.02
46.96	0.044	0.02667
46.98	0.028	0.08667
47	0.042	0.18
47.02	0	0.26667
47.04	0.102	0.35333
47.06	0.048	0.40667
47.08	0.028	0.40667
47.1	0.044	0.38
47.12	0	0.30667
47.14	0.082	0.24
47.16	0.074	0.17333
47.18	0.086	0.11333
47.2	0.004	0.09333
47.22	0.012	0.1
47.24	0.034	0.14667
47.26	0.046	0.21333
47.28	0.054	0.27333
47.3	0.084	0.32667
47.32	0.05	0.35333
47.34	0.086	0.34667
47.36	0.088	0.3
47.38	0	0.22667
47.4	0	0.16

47.42	0.04	0.11333
47.44	0.082	0.1
47.46	0.078	0.12667
47.48	0.076	0.18
47.5	0.018	0.24
47.52	0.04	0.28667
47.54	0.024	0.28
47.56	0.018	0.26
47.58	0.014	0.24667
47.6	0.002	0.24
47.62	0.016	0.22667
47.64	0.046	0.21333
47.66	0.084	0.2
47.68	0.128	0.19333
47.7	0	0.16
47.72	0.072	0.14667
47.74	0	0.16
47.76	0.006	0.16
47.78	0.004	0.16667
47.8	0	0.14667
47.82	0	0.12667
47.84	0.066	0.11333
47.86	0.01	0.09333
47.88	0.06	0.08
47.9	0.016	0.08
47.92	0	0.08667
47.94	0.012	0.10667
47.96	0.04	0.12667
47.98	0.012	0.16
48	0	0.19333

48.02	0.004	0.22
48.04	0	0.22
48.06	0.058	0.18667
48.08	0.044	0.14
48.1	0.018	0.09333
48.12	0	0.07333
48.14	0.106	0.06667
48.16	0	0.08667
48.18	0	0.12667
48.2	0	0.16667
48.22	0.03	0.18667
48.24	0.04	0.2
48.26	0.014	0.2
48.28	0.044	0.2
48.3	0.1	0.17333
48.32	0.034	0.14
48.34	0.02	0.12667
48.36	0.008	0.12
48.38	0	0.12
48.4	0.064	0.12667
48.42	0	0.15333
48.44	0	0.16667
48.46	0	0.17333
48.48	0.03	0.18
48.5	0.04	0.19333
48.52	0.012	0.19333
48.54	0.016	0.16
48.56	0	0.11333
48.58	0	0.07333
48.6	0	0.04667

48.62	0.07	0.04
48.64	0.058	0.07333
48.66	0.006	0.14
48.68	0.094	0.22
48.7	0.002	0.28
48.72	0.022	0.29333
48.74	0.008	0.28667
48.76	0.004	0.24
48.78	0.086	0.18
48.8	0.09	0.13333
48.82	0.04	0.12
48.84	0	0.12
48.86	0.02	0.14667
48.88	0.052	0.15333
48.9	0.082	0.16667
48.92	0	0.15333
48.94	0	0.14667
48.96	0.046	0.14
48.98	0.024	0.14
49	0	0.14
49.02	0	0.14
49.04	0.032	0.14667
49.06	0.05	0.14667
49.08	0.074	0.14
49.1	0.036	0.14
49.12	0.08	0.15333
49.14	0.044	0.16667
49.16	0.06	0.18
49.18	0.016	0.18
49.2	0.004	0.17333

49.22	0.032	0.14
49.24	0	0.1
49.26	0.002	0.05333
49.28	0.03	0.02667
49.3	0	0.02
49.32	0	0.02
49.34	0	0.02667
49.36	0.048	0.04667
49.38	0.02	0.06
49.4	0.008	0.08
49.42	0	0.09333
49.44	0.056	0.10667
49.46	0.038	0.10667
49.48	0.076	0.08667
49.5	0	0.06
49.52	0.106	0.03333
49.54	0	0.00667
49.56	0.006	0
49.58	0.05	0.02
49.6	0.022	0.05333
49.62	0.014	0.1
49.64	0.012	0.13333
49.66	0.046	0.15333
49.68	0	0.13333
49.7	0	0.1
49.72	0	0.05333
49.74	0	0.02667
49.76	0.022	0.01333
49.78	0.072	0.02667
49.8	0	0.06667

49.82	0.03	0.12
49.84	0	0.16
49.86	0	0.19333
49.88	0.064	0.19333
49.9	0.072	0.18667
49.92	0.002	0.16
49.94	0.096	0.13333
49.96	0.09	0.11333
49.98	0.174	0.09333
50	0.05	0.08667
50.02	0.02	0.07333
50.04	0	0.04667
50.06	0	0.04
50.08	0	0.04667
50.1	0.006	0.08
50.12	0.004	0.11333
50.14	0.036	0.14
50.16	0	0.16
50.18	0	0.15333
50.2	0.006	0.13333
50.22	0.06	0.13333
50.24	0.03	0.14667
50.26	0	0.18
50.28	0.066	0.2
50.3	0.056	0.21333
50.32	0	0.19333
50.34	0.072	0.15333
50.36	0	0.11333
50.38	0	0.10667
50.4	0.028	0.11333

50.42	0	0.13333
50.44	0	0.14667
50.46	0.098	0.16667
50.48	0.082	0.15333
50.5	0.094	0.11333
50.52	0.084	0.07333
50.54	0.102	0.04
50.56	0	0.02
50.58	0.03	0.01333
50.6	0.01	0.02667
50.62	0.002	0.04667
50.64	0	0.06667
50.66	0.056	0.08
50.68	0.012	0.08667
50.7	0.062	0.1
50.72	0.014	0.11333
50.74	0	0.11333
50.76	0.108	0.12667
50.78	0.03	0.12667
50.8	0	0.13333
50.82	0	0.13333
50.84	0	0.12
50.86	0	0.11333
50.88	0	0.09333
50.9	0.01	0.06667
50.92	0.018	0.04667
50.94	0.02	0.02
50.96	0	0.00667
50.98	0.08	0.00667
51	0.014	0.01333

51.02	0.054	0.02667
51.04	0	0.04
51.06	0.02	0.06
51.08	0.02	0.08
51.1	0.038	0.08667
51.12	0	0.09333
51.14	0	0.09333
51.16	0	0.07333
51.18	0	0.04667
51.2	0.012	0.02667
51.22	0.002	0.01333
51.24	0.016	0.02
51.26	0.07	0.04
51.28	0.056	0.08
51.3	0.026	0.12667
51.32	0.114	0.16
51.34	0.096	0.16
51.36	0.032	0.15333
51.38	0	0.13333
51.4	0	0.10667
51.42	0	0.08667
51.44	0	0.06
51.46	0	0.04667
51.48	0	0.04667
51.5	0.074	0.03333
51.52	0.066	0.03333
51.54	0	0.02667
51.56	0.024	0.03333
51.58	0	0.03333
51.6	0	0.02667

51.62	0.028	0.02667
51.64	0.042	0.02667
51.66	0	0.02
51.68	0.018	0.03333
51.7	0	0.05333
51.72	0.032	0.06667
51.74	0	0.08667
51.76	0.03	0.10667
51.78	0.036	0.14
51.8	0	0.14667
51.82	0	0.14667
51.84	0	0.14667
51.86	0.024	0.12
51.88	0.05	0.08667
51.9	0.088	0.06
51.92	0.016	0.04667
51.94	0	0.04
51.96	0.106	0.04
51.98	0.01	0.03333
52	0	0.02667
52.02	0	0.02
52.04	0.032	0.01333
52.06	0.018	0.01333
52.08	0	0.02
52.1	0.048	0.02667
52.12	0.072	0.02667
52.14	0	0.02667
52.16	0	0.03333
52.18	0.046	0.04
52.2	0.068	0.04

52.22	0.034	0.04
52.24	0	0.04
52.26	0.052	0.04
52.28	0.046	0.04
52.3	0.078	0.04667
52.32	0.002	0.06
52.34	0	0.05333
52.36	0	0.04667
52.38	0.086	0.03333
52.4	0	0.01333
52.42	0	0.00667
52.44	0	0.02
52.46	0	0.04667
52.48	0.012	0.07333
52.5	0	0.1
52.52	0	0.11333
52.54	0.096	0.12667
52.56	0.11	0.12
52.58	0.024	0.10667
52.6	0.044	0.09333
52.62	0.038	0.08667
52.64	0.038	0.06667
52.66	0.07	0.04667
52.68	0.016	0.02667
52.7	0.008	0.01333
52.72	0.004	0.00667
52.74	0	0.01333
52.76	0.002	0.03333
52.78	0.014	0.06
52.8	0.058	0.08667

52.82	0.068	0.11333
52.84	0.074	0.13333
52.86	0.092	0.14
52.88	0.072	0.13333
52.9	0.136	0.11333
52.92	0.026	0.09333
52.94	0	0.06667
52.96	0	0.04
52.98	0	0.02667
53	0.06	0.02
53.02	0.044	0.02
53.04	0	0.01333
53.06	0.052	0.02
53.08	0.126	0.04
53.1	0.062	0.06667
53.12	0.116	0.08667
53.14	0.046	0.12
53.16	0.02	0.15333
53.18	0.036	0.17333
53.2	0.118	0.15333
53.22	0.062	0.14
53.24	0.058	0.12667
53.26	0.014	0.11333
53.28	0.04	0.1
53.3	0.044	0.10667
53.32	0	0.12
53.34	0.012	0.14
53.36	0.008	0.14
53.38	0.016	0.13333
53.4	0.02	0.13333

53.42	0.014	0.14667
53.44	0	0.15333
53.46	0.018	0.14667
53.48	0.074	0.13333
53.5	0.01	0.12667
53.52	0.09	0.10667
53.54	0.086	0.1
53.56	0.086	0.11333
53.58	0	0.13333
53.6	0.05	0.14
53.62	0.022	0.12667
53.64	0.062	0.1
53.66	0.012	0.08
53.68	0	0.06
53.7	0	0.06667
53.72	0.026	0.09333
53.74	0.036	0.12667
53.76	0.01	0.14667
53.78	0.056	0.16
53.8	0.074	0.15333
53.82	0	0.12667
53.84	0.156	0.08667
53.86	0.134	0.04667
53.88	0.062	0.02
53.9	0.116	0
53.92	0.022	0
53.94	0.024	0.00667
53.96	0.092	0.02667
53.98	0.052	0.04
54	0.024	0.05333

54.02	0.064	0.06
54.04	0.022	0.06
54.06	0.08	0.06667
54.08	0.07	0.08
54.1	0.018	0.09333
54.12	0.066	0.12
54.14	0.074	0.12
54.16	0.026	0.11333
54.18	0.108	0.1
54.2	0.026	0.07333
54.22	0.056	0.04667
54.24	0.058	0.03333
54.26	0.036	0.02
54.28	0.032	0.02
54.3	0.024	0.04
54.32	0.032	0.07333
54.34	0.082	0.12
54.36	0.034	0.14667
54.38	0.118	0.16667
54.4	0.04	0.18
54.42	0.018	0.16667
54.44	0.048	0.15333
54.46	0	0.14667
54.48	0	0.12667
54.5	0.016	0.10667
54.52	0.034	0.08
54.54	0.038	0.05333
54.56	0.024	0.04667
54.58	0	0.04667
54.6	0.102	0.04

54.62	0.054	0.04
54.64	0.02	0.02667
54.66	0.004	0.02
54.68	0.06	0.01333
54.7	0.03	0.00667
54.72	0.044	0.01333
54.74	0.062	0.04667
54.76	0.07	0.09333
54.78	0.07	0.14667
54.8	0	0.19333
54.82	0.066	0.22667
54.84	0.078	0.22
54.86	0.094	0.18
54.88	0.084	0.13333
54.9	0.004	0.08667
54.92	0.018	0.06
54.94	0.022	0.04667
54.96	0.088	0.04
54.98	0.056	0.04
55	0.04	0.04
55.02	0.07	0.04
55.04	0.048	0.05333
55.06	0.084	0.06
55.08	0	0.07333
55.1	0.024	0.1
55.12	0.08	0.13333
55.14	0.08	0.17333
55.16	0.022	0.20667
55.18	0.052	0.22667
55.2	0.046	0.21333

55.22	0.056	0.16667
55.24	0.018	0.11333
55.26	0.116	0.08
55.28	0.094	0.06667
55.3	0	0.08
55.32	0	0.1
55.34	0.02	0.1
55.36	0.008	0.08
55.38	0.05	0.05333
55.4	0.07	0.02667
55.42	0.05	0
55.44	0.09	0
55.46	0.102	0
55.48	0.04	0.00667
55.5	0.064	0.00667
55.52	0	0.01333
55.54	0	0.03333
55.56	0.062	0.05333
55.58	0.12	0.07333
55.6	0.016	0.1
55.62	0.046	0.12667
55.64	0.012	0.14667
55.66	0.108	0.14
55.68	0.084	0.12667
55.7	0.07	0.10667
55.72	0.064	0.08667
55.74	0.042	0.06667
55.76	0.088	0.06
55.78	0.004	0.06
55.8	0	0.06667

55.82	0	0.08
55.84	0.074	0.10667
55.86	0.014	0.12667
55.88	0.002	0.15333
55.9	0.068	0.18667
55.92	0.07	0.22667
55.94	0.03	0.26667
55.96	0.1	0.30667
55.98	0.06	0.35333
56	0.068	0.38
56.02	0.078	0.34667
56.04	0.048	0.30667
56.06	0.076	0.24
56.08	0.062	0.17333
56.1	0.066	0.12
56.12	0	0.08667
56.14	0.036	0.08
56.16	0.03	0.09333
56.18	0.01	0.10667
56.2	0.118	0.14
56.22	0.124	0.18667
56.24	0.002	0.22
56.26	0.106	0.24667
56.28	0.128	0.25333
56.3	0.064	0.23333
56.32	0.132	0.2
56.34	0.046	0.17333
56.36	0.002	0.16
56.38	0.038	0.16
56.4	0.086	0.16667

56.42	0.08	0.16
56.44	0	0.14
56.46	0.098	0.10667
56.48	0.014	0.07333
56.5	0.018	0.05333
56.52	0.026	0.04667
56.54	0.062	0.04
56.56	0	0.03333
56.58	0.03	0.02
56.6	0	0.00667
56.62	0.112	0
56.64	0.064	0.00667
56.66	0.024	0.04
56.68	0.016	0.08667
56.7	0.036	0.14
56.72	0.16	0.19333
56.74	0.124	0.22
56.76	0.062	0.21333
56.78	0.042	0.17333
56.8	0.026	0.13333
56.82	0.026	0.08667
56.84	0	0.04667
56.86	0.024	0.02
56.88	0.02	0.01333
56.9	0.038	0.00667
56.92	0.036	0.02
56.94	0.024	0.04667
56.96	0.016	0.08667
56.98	0.064	0.13333
57	0.04	0.17333

57.02	0.05	0.2
57.04	0.056	0.20667
57.06	0.03	0.18667
57.08	0.04	0.16
57.1	0.034	0.11333
57.12	0.03	0.06667
57.14	0.054	0.03333
57.16	0.02	0.02667
57.18	0.022	0.03333
57.2	0.02	0.05333
57.22	0.002	0.08
57.24	0.036	0.1
57.26	0.07	0.1
57.28	0	0.1
57.3	0.078	0.1
57.32	0.09	0.10667
57.34	0.018	0.10667
57.36	0.058	0.11333
57.38	0.054	0.11333
57.4	0.066	0.1
57.42	0.052	0.07333
57.44	0.006	0.06667
57.46	0.014	0.08
57.48	0.096	0.09333
57.5	0.052	0.10667
57.52	0.086	0.10667
57.54	0.044	0.1
57.56	0	0.08
57.58	0.11	0.06
57.6	0.032	0.05333

57.62	0.046	0.06667
57.64	0.024	0.08667
57.66	0.068	0.12
57.68	0.02	0.14667
57.7	0.046	0.16
57.72	0.08	0.15333
57.74	0.072	0.14
57.76	0.028	0.11333
57.78	0.056	0.1
57.8	0.102	0.08667
57.82	0.108	0.1
57.84	0.06	0.10667
57.86	0.074	0.11333
57.88	0.086	0.10667
57.9	0.104	0.09333
57.92	0.084	0.08
57.94	0.038	0.06667
57.96	0.04	0.06
57.98	0.138	0.06
58	0.062	0.07333
58.02	0.002	0.08
58.04	0	0.08
58.06	0	0.06667
58.08	0.016	0.06667
58.1	0.062	0.07333
58.12	0.038	0.07333
58.14	0	0.08667
58.16	0.018	0.1
58.18	0.078	0.10667
58.2	0	0.09333

58.22	0.012	0.08
58.24	0.014	0.08667
58.26	0.01	0.09333
58.28	0	0.1
58.3	0	0.10667
58.32	0.022	0.12
58.34	0.002	0.13333
58.36	0	0.12667
58.38	0.004	0.10667
58.4	0	0.1
58.42	0	0.08667
58.44	0	0.08667
58.46	0.048	0.09333
58.48	0.058	0.12
58.5	0.072	0.15333
58.52	0.052	0.17333
58.54	0	0.16667
58.56	0.018	0.16
58.58	0	0.14
58.6	0	0.12
58.62	0.004	0.1
58.64	0.034	0.09333
58.66	0.024	0.1
58.68	0.002	0.10667
58.7	0.008	0.11333
58.72	0.056	0.10667
58.74	0.04	0.1
58.76	0.094	0.09333
58.78	0	0.08667
58.8	0	0.08667

58.82	0.024	0.08667
58.84	0.014	0.08
58.86	0	0.05333
58.88	0	0.03333
58.9	0	0.02
58.92	0	0.02
58.94	0.044	0.04
58.96	0.012	0.06667
58.98	0	0.10667
59	0	0.13333
59.02	0	0.14
59.04	0.002	0.14
59.06	0.074	0.14667
59.08	0.048	0.15333
59.1	0.01	0.15333
59.12	0	0.15333
59.14	0.006	0.16
59.16	0.044	0.14667
59.18	0.002	0.12667
59.2	0.034	0.1
59.22	0	0.07333
59.24	0	0.05333
59.26	0.012	0.03333
59.28	0	0.02667
59.3	0	0.02
59.32	0	0.02
59.34	0	0.02667
59.36	0	0.04
59.38	0.054	0.06
59.4	0	0.08

59.42	0	0.1
59.44	0	0.12
59.46	0.014	0.12
59.48	0	0.11333
59.5	0	0.10667
59.52	0.016	0.10667
59.54	0.01	0.10667
59.56	0.002	0.12667
59.58	0.056	0.15333
59.6	0	0.17333
59.62	0	0.19333
59.64	0.05	0.21333
59.66	0.024	0.18667
59.68	0.006	0.15333
59.7	0	0.10667
59.72	0	0.06667
59.74	0	0.04
59.76	0	0.02
59.78	0.082	0.03333
59.8	0.02	0.05333
59.82	0	0.07333
59.84	0	0.09333
59.86	0.032	0.1
59.88	0	0.09333
59.9	0.024	0.08
59.92	0.088	0.05333
59.94	0.006	0.04
59.96	0	0.03333
59.98	0.05	0.04667
60	0.028	0.06

Figure 2

For CCK-8 assay of L929 cells co-cultured with various concentrations of SA composite hydrogels:

Control	SD	0.012 L-SA	SD	0.012 D-SA	SD
94.9	1.6	96.1	5.1	98.6	4.8

Control	SD	0.012 L-SA	SD	0.012 D-SA	SD
92.3	2.7	98.6	1.352	97.1	5.4

Control	SD	0.012 L-SA	SD	0.012 D-SA	SD
99.3	4.4	99.1	2.3	98.5	2.7

For CCK-8 test of L929 cells co-cultured with equal volumes of PBS and composite hydrogels after one, three, and five days:

One day

Control	SD	3% L-SA	SD	5% L-SA	SD	10% L-SA	SD	3% D-SA	SD	5% D-SA	SD	10% D-SA	SD
100	4.1	99.074	2.2	97.37	3.3	90.126	5	98.355	5.7	96.312	3.9	88.908	5.1

Three day

Control	SD	3% L-SA	SD	5% L-SA	SD	10% L-SA	SD	3% D-SA	SD	5% D-SA	SD	10% D-SA	SD
100	4.1	97.441	2	99.136	5	97.087	2.4	95.787	5.2	97.894	6.4	96.783	6.3

Five day

Control	SD	3% L-SA	SD	5% L-SA	SD	10% L-SA	SD	3% D-SA	SD	5% D-SA	SD	10% D-SA	SD
100	3	98.428	3.7	95.681	3.1	93.982	2.4	96.991	2	94.82	3.3	95.453	2.5

For CCK-8 test of macrophages co-cultured with various concentrations of SA and composite hydrogels:

control	10mmol/L	15mmol/L	20mmol/L	35mmol/L	40mmol/L	LPFEG	DPFEG	L-SA	D-SA
2.32518	2.36641	2.02739	1.83497	1.10610	0.208293	2.02190	2.03161	1.91991	1.79645
2.46941	2.31947	2.13993	1.70059	1.19241	0.405673	2.07125	2.04799	1.93427	1.80494

2.43493	2.23487	2.05462	1.92266	1.24541	0.396510	2.02899	2.10943	1.90298	1.83293

Figure 3

For the CD86 expression was detected by flow cytometry:

Control	LPFEG	DPFEG	LPS	LPS+LPFEG	LPS+DPFEG	LPS+L-SA	LPS+D-SA	LPS+SA
6.25	3.31	7.24	42.1	30.3	36.90	6.15	7.15	5.97
5.35	5.27	5.68	44.5	35.2	42.60	7.68	6.75	5.21
6.01	4.36	6.59	56.2	37.6	38.92	8.35	9.35	6.87

For the corresponding signal intensity quantification of CD86:

Control	LPFEG	DPFEG	LPS	LPS+LPFEG	LPS+DPFEG	LPS+L-SA	LPS+D-SA	LPS+SA
1.0	1.10	1.067	2.3	1.83	1.93	1.08	1.077	0.83

For the corresponding signal intensity quantification of TNF-α:

Control	LPFEG	DPFEG	LPS	LPS+LPFEG	LPS+DPFEG	LPS+L-SA	LPS+D-SA	LPS+SA
1.0	1.075	1.025	1.8	1.75	2.05	1.425	1.500	1.25

For the corresponding signal intensity quantification of IL-1β:

Control	LPFEG	DPFEG	LPS	LPS+LPFEG	LPS+DPFEG	LPS+L-SA	LPS+D-SA	LPS+SA
1.0	1.250	1.050	2.25	2.15	2.55	1.750	1.550	1.35

For the gene level of TNF-α:

Control	LPFEG	DPFEG	LPS	LPS+LPFEG	LPS+DPFEG	LPS+L-SA	LPS+D-SA	LPS+SA
1.111265000	1.737729000	4.388932000	753.3641000	763.8806000	732.7633000	29.59340000	34.82867000	30.832110
0.960730300	1.928131000	4.123502000	835.9098000	712.7258000	994.0698000	28.98437000	32.94993000	27.643100
0.928004300	1.968646000	5.041560000	732.7633000	779.9314000	769.1938000	37.98093000	31.17253000	33.563910

For the gene level of IL-1β:

Control	LPFEG	DPFEG	LPS	LPS+LPFEG	LPS+DPFEG	LPS+L-SA	LPS+D-SA	LPS+SA
1.181456000	0.446139400	2.892332000	2313.027000	1852.896000	1568.931000	59.53089000	53.46662000	66.356100
0.858902600	0.512479600	2.892332000	2867.478000	1931.581000	1635.557000	76.93491000	72.53304000	58.631200
0.959640900	0.372565600	2.994330000	2158.131000	1375.334000	2531.131000	83.60785000	64.91889000	61.456300

Figure 4

For the Cumulative amount of SA:

Time	0% L-SA	SD	3% L-SA	SD	5% L-SA	SD	10% L-SA	SD
1	9.903	3.3	8.563	2.6	9.896	5.6	10.231	7.4
2	20.726	4.1	18.632	3.1	23.633	3.3	21.556	5.5
3	32.475	5.6	29.331	5.8	38.639	2.6	38.996	5.6
4	44.794	6.9	40.686	6.9	52.26	4.8	54.365	5.8
5	57.602	5.8	52.319	1.7	69.663	4.5	74.489	6.2
6	71.193	5.9	68.262	5.4	90.226	7.2	105.637	3.5
7	85.532	3.4	79.339	3.9	101.354	3.8	120.258	3.1
8	97.105	5.72	92.832	4.7	123.023	5.7	134.697	3.7
9	116.659	0.7	105.301	5.6	146.223	5.5	148.253	4.4
10	134.058	3.8	122.286	5.5	158.397	3.2	162.031	3.8
11	152.448	3.6	135.552	2.3	169.652	4.3	188.619	4.6
12	171.719	3.4	153.369	3.9	198.367	2.3	205.445	5.6

Time	0% D-SA	SD	3% D-SA	SD	5% D-SA	SD	10% D-SA	SD
1	7.847	0.6	8.896	4.2	9.016	7.2	8.563	2.6
2	16.078	2.3	17.665	6.8	15.344	2.8	18.632	3.1
3	25.059	2.1	31.603	5.5	27.629	6.3	29.331	5.8
4	34.652	1.6	45.221	5.6	44.338	5.8	40.686	6.9

5	44.487	2.1	58.365	5.5	63.626	5.7	52.319	1.7
6	54.829	2.9	79.201	2.3	79.639	5.5	68.262	5.4
7	65.591	2.2	90.736	4.7	93.552	4.9	79.339	3.9
8	76.85	2.89	110.341	3.6	105.589	5.6	92.832	4.7
9	88.364	3.6	125.776	2.2	130.221	3.6	105.301	5.6
10	99.192	4	140.065	7.6	148.354	4.4	122.286	5.5
11	109.424	6.5	158.316	4.8	164.489	6.8	135.552	2.3
12	114.67	7.2	171.621	4.6	185.361	3.1	153.369	3.9

For the ATR-FTIR of skin:

Wavenumber	Control	5% D-SA	5% L-SA
599.7532	95.34308	98.54034	89.71731
601.6816	95.47642	98.61529	89.67815
603.6101	95.53568	98.64847	89.76616
605.5386	95.40501	98.68076	89.87518
607.467	95.34811	98.74042	89.90058
609.3955	95.43034	98.74515	89.92559
611.324	95.56259	98.74014	89.92279
613.2524	95.7	98.69336	89.95678
615.1809	95.70531	98.56444	89.93939
617.1094	95.60858	98.63364	90.03861
619.0378	95.49502	98.70528	90.15672
620.9663	95.49243	98.74752	90.23209
622.8948	95.69622	98.76217	90.16689
624.8232	95.78516	98.81844	90.20665
626.7517	95.798	98.92694	90.23001
628.6802	95.80521	98.97798	90.31313
630.6086	95.84868	98.90622	90.32799
632.5371	95.8596	98.93774	90.32052
634.4656	95.87243	99.05968	90.42191

636.394	95.95332	99.01443	90.56701
638.3225	96.04126	98.8603	90.60921
640.251	95.99437	98.86176	90.62687
642.1794	95.87952	98.91111	90.59393
644.1079	95.92653	98.87844	90.67067
646.0364	95.98973	98.91032	90.74551
647.9648	96.10609	98.84261	90.71048
649.8933	96.08631	98.78758	90.71904
651.8218	96.02918	98.82909	90.71692
653.7502	96.02467	98.93603	90.75858
655.6787	96.06044	99.02055	90.84187
657.6072	96.10039	98.88737	90.81389
659.5356	96.1296	98.81248	90.82784
661.4641	96.10428	98.8521	90.84328
663.3926	96.09913	98.80834	90.83986
665.321	96.09624	98.80255	90.82939
667.2495	96.07081	98.5528	90.77843
669.178	96.20177	98.66026	90.8839
671.1064	96.33469	98.94614	91.05139
673.0349	96.36559	98.99612	91.14956
674.9634	96.40552	98.95737	91.14059
676.8918	96.38036	98.96938	91.18114
678.8203	96.37582	99.00292	91.24157
680.7488	96.50243	99.07721	91.2737
682.6772	96.62466	99.10851	91.31168
684.6057	96.66813	99.07686	91.3382
686.5342	96.71568	99.06532	91.41937
688.4626	96.75179	99.09618	91.45988
690.3911	96.70798	99.15002	91.47106
692.3196	96.73195	99.20232	91.54408

694.248	96.75769	99.21214	91.60056
696.1765	96.78821	99.15168	91.63138
698.105	96.7911	99.1834	91.65668
700.0334	96.76259	99.22829	91.68491
701.9619	96.79895	99.25728	91.76509
703.8904	96.89886	99.22924	91.81874
705.8188	96.96915	99.24482	91.86353
707.7473	97.02232	99.30729	91.92724
709.6758	97.09229	99.33128	92.00965
711.6042	97.13918	99.32804	92.0622
713.5327	97.20305	99.32677	92.09179
715.4612	97.23537	99.37212	92.1021
717.3896	97.21583	99.39858	92.08948
719.3181	97.16777	99.35565	92.09655
721.2466	97.16408	99.32566	92.15816
723.175	97.29098	99.4284	92.2804
725.1035	97.40771	99.55928	92.45969
727.032	97.55419	99.61043	92.61163
728.9604	97.66562	99.60403	92.72176
730.8889	97.68881	99.67194	92.87284
732.8174	97.73516	99.683	93.01415
734.7458	97.77112	99.64367	93.08562
736.6743	97.79329	99.68803	93.14484
738.6028	97.79311	99.77219	93.259
740.5313	97.85479	99.86012	93.40276
742.4597	97.93837	99.87268	93.4733
744.3882	97.92612	99.86003	93.53919
746.3167	97.9278	99.93741	93.6825
748.2451	97.99612	99.97442	93.77827
750.1736	98.08247	99.99896	93.87057

752.1021	98.17654	100.0098	93.94159
754.0305	98.21099	99.97918	93.99241
755.959	98.22249	99.99197	94.0307
757.8875	98.24725	100.0352	94.11723
759.8159	98.27911	100.031	94.19253
761.7444	98.33225	100.0617	94.24142
763.6729	98.39887	100.0403	94.30157
765.6013	98.40678	100.0143	94.41695
767.5298	98.41211	100.0805	94.48698
769.4583	98.42207	100.1888	94.58334
771.3867	98.44457	100.2313	94.66936
773.3152	98.48801	100.2067	94.73635
775.2437	98.53495	100.23	94.82545
777.1721	98.60314	100.2524	94.88667
779.1006	98.64941	100.265	94.9483
781.0291	98.71408	100.2739	95.04816
782.9575	98.76476	100.2813	95.13322
784.886	98.76847	100.3139	95.21084
786.8145	98.76453	100.3539	95.2648
788.7429	98.83105	100.3761	95.39445
790.6714	98.93686	100.3835	95.4876
792.5999	98.95148	100.4092	95.50027
794.5283	98.92391	100.4205	95.53062
796.4568	98.93653	100.4489	95.6025
798.3853	98.97133	100.4526	95.63995
800.3137	99.00053	100.447	95.68846
802.2422	98.9929	100.4562	95.78786
804.1707	98.97603	100.4594	95.90935
806.0991	99.0243	100.4498	95.96372
808.0276	99.08411	100.4644	96.03414

809.9561	99.12129	100.528	96.15664
811.8845	99.14513	100.5586	96.23165
813.813	99.14482	100.5529	96.27811
815.7415	99.13656	100.531	96.31197
817.6699	99.17179	100.5437	96.39771
819.5984	99.23765	100.5914	96.48962
821.5269	99.29935	100.5876	96.52097
823.4553	99.3252	100.5849	96.58221
825.3838	99.28547	100.5864	96.62659
827.3123	99.26692	100.6102	96.70625
829.2407	99.30582	100.6503	96.79147
831.1692	99.36211	100.6656	96.87201
833.0977	99.38926	100.6866	96.95818
835.0261	99.42184	100.6769	96.99754
836.9546	99.42555	100.6591	97.009
838.8831	99.41982	100.7088	97.10961
840.8115	99.48235	100.7346	97.20098
842.74	99.56331	100.731	97.28501
844.6685	99.5918	100.7602	97.356
846.5969	99.57535	100.7886	97.3993
848.5254	99.57092	100.7822	97.4446
850.4539	99.59963	100.7831	97.51709
852.3823	99.67281	100.8232	97.62227
854.3108	99.75063	100.8668	97.71999
856.2393	99.77811	100.8638	97.74702
858.1677	99.75072	100.8294	97.77972
860.0962	99.77007	100.8379	97.82366
862.0247	99.79422	100.8984	97.90122
863.9531	99.80717	100.9126	97.96224
865.8816	99.83154	100.8882	97.96291

867.8101	99.85459	100.892	97.98089
869.7385	99.85325	100.931	98.05227
871.667	99.85275	100.9166	98.11861
873.5955	99.84595	100.8827	98.15851
875.5239	99.83906	100.8682	98.20341
877.4524	99.86258	100.8585	98.25152
879.3809	99.91312	100.8908	98.31445
881.3093	99.92593	100.9185	98.37505
883.2378	99.91197	100.9137	98.42165
885.1663	99.93617	100.8923	98.44991
887.0947	99.97926	100.9168	98.50394
889.0232	100.0094	100.9481	98.56705
890.9517	99.98947	100.9346	98.60361
892.8801	99.98807	100.9454	98.63012
894.8086	99.9971	100.9574	98.66571
896.7371	100.0082	100.9459	98.69672
898.6655	100.0268	100.9196	98.71626
900.594	100.0098	100.929	98.75915
902.5225	100.0077	100.9729	98.82084
904.4509	100.029	100.9759	98.85063
906.3794	100.0425	100.9742	98.89608
908.3079	100.0519	100.9705	98.91934
910.2363	100.0825	100.9484	98.9107
912.1648	100.0995	100.9274	98.92472
914.0933	100.0874	100.9227	98.95052
916.0217	100.0877	100.9418	98.97177
917.9502	100.0682	100.9842	99.03059
919.8787	100.0389	100.982	99.04253
921.8071	100.0673	100.9555	99.05807
923.7356	100.089	100.9691	99.13034

925.6641	100.0972	100.9985	99.18616
927.5925	100.0903	100.9999	99.19468
929.521	100.0497	100.9872	99.2036
931.4495	100.0333	100.9654	99.22456
933.3779	100.0599	100.9386	99.20535
935.3064	100.0972	100.9421	99.20737
937.2349	100.134	100.967	99.26157
939.1633	100.1506	100.9597	99.27808
941.0918	100.146	100.9775	99.3086
943.0203	100.1593	101.0114	99.36512
944.9487	100.1713	101.0336	99.40565
946.8772	100.1797	101.0413	99.41337
948.8057	100.1629	101.0219	99.39991
950.7341	100.1325	100.9973	99.39095
952.6626	100.1207	101.003	99.39297
954.5911	100.1276	101.0108	99.40845
956.5195	100.1093	100.9996	99.4183
958.448	100.1024	100.9819	99.40288
960.3765	100.0897	100.9613	99.38385
962.3049	100.0495	100.9268	99.36903
964.2334	100.0037	100.8949	99.3317
966.1619	99.97346	100.8445	99.27269
968.0903	99.94495	100.8234	99.24058
970.0188	99.9189	100.8251	99.25624
971.9473	99.91566	100.8328	99.2844
973.8757	99.95413	100.8487	99.31582
975.8042	99.99478	100.8484	99.33456
977.7327	99.99693	100.8469	99.33578
979.6611	99.97623	100.8599	99.35159
981.5896	99.97552	100.8784	99.37546

983.5181	100.0217	100.8709	99.38129
985.4465	100.0394	100.8674	99.40364
987.375	100.0161	100.8695	99.42386
989.3035	100.0137	100.8626	99.41875
991.2319	100.0184	100.8585	99.42398
993.1604	100.0271	100.8701	99.43601
995.0889	100.017	100.8782	99.42734
997.0173	100.0048	100.8776	99.43546
998.9458	100.0101	100.8629	99.46822
1000.874	100.0081	100.8419	99.46288
1002.803	100.0257	100.8384	99.45383
1004.731	100.0436	100.8369	99.43768
1006.66	100.0027	100.8405	99.42648
1008.588	99.9539	100.849	99.42402
1010.517	99.92013	100.8426	99.41283
1012.445	99.90129	100.8347	99.43594
1014.374	99.86749	100.834	99.44187
1016.302	99.83116	100.838	99.42374
1018.23	99.79018	100.8136	99.41219
1020.159	99.74402	100.7793	99.38824
1022.087	99.69391	100.7758	99.37962
1024.016	99.65946	100.7764	99.38514
1025.944	99.63805	100.7544	99.35445
1027.873	99.5847	100.7262	99.32186
1029.801	99.55013	100.7449	99.35429
1031.73	99.53998	100.7585	99.3818
1033.658	99.50469	100.7333	99.37924
1035.587	99.47857	100.7229	99.3868
1037.515	99.45764	100.7318	99.39665
1039.444	99.42979	100.7012	99.38122

1041.372	99.41225	100.6804	99.36113
1043.301	99.39617	100.6791	99.37081
1045.229	99.32594	100.6752	99.37595
1047.157	99.2401	100.6647	99.36166
1049.086	99.21688	100.6625	99.36868
1051.014	99.19437	100.671	99.37688
1052.943	99.15813	100.6596	99.35677
1054.871	99.12984	100.6582	99.35519
1056.8	99.12914	100.667	99.37601
1058.728	99.11021	100.6798	99.40487
1060.657	99.0977	100.6706	99.40338
1062.585	99.0849	100.6588	99.3865
1064.514	99.06365	100.66	99.39042
1066.442	99.06055	100.6521	99.40228
1068.371	99.06127	100.6359	99.39865
1070.299	99.05225	100.6399	99.38402
1072.228	99.03416	100.6332	99.37602
1074.156	99.00801	100.6369	99.37988
1076.084	98.99784	100.6279	99.36932
1078.013	98.99656	100.6066	99.36337
1079.941	99.0078	100.6133	99.35763
1081.87	99.03348	100.6242	99.34845
1083.798	99.06224	100.6394	99.36906
1085.727	99.06356	100.6473	99.37366
1087.655	99.07464	100.6452	99.37662
1089.584	99.12184	100.661	99.39857
1091.512	99.14339	100.6585	99.39794
1093.441	99.18623	100.6535	99.41322
1095.369	99.22732	100.672	99.43827
1097.298	99.24509	100.7136	99.45631

1099.226	99.29004	100.7374	99.47691
1101.155	99.30978	100.7346	99.49081
1103.083	99.32267	100.7459	99.50878
1105.011	99.34364	100.7477	99.52442
1106.94	99.35049	100.7385	99.5322
1108.868	99.36183	100.7626	99.5399
1110.797	99.39919	100.7664	99.54185
1112.725	99.43362	100.7398	99.53337
1114.654	99.43002	100.7446	99.53447
1116.582	99.43951	100.7536	99.54979
1118.511	99.46677	100.7602	99.57228
1120.439	99.4846	100.7848	99.60165
1122.368	99.50071	100.7889	99.61908
1124.296	99.50584	100.8027	99.63141
1126.225	99.51339	100.8338	99.64381
1128.153	99.53207	100.8292	99.64484
1130.082	99.55721	100.806	99.63612
1132.01	99.58588	100.7986	99.65231
1133.938	99.61066	100.7865	99.66553
1135.867	99.62054	100.8023	99.67751
1137.795	99.64108	100.8384	99.69283
1139.724	99.65021	100.8596	99.67806
1141.652	99.64721	100.8466	99.65021
1143.581	99.64803	100.8319	99.63335
1145.509	99.64063	100.8177	99.63219
1147.438	99.58112	100.7811	99.59659
1149.366	99.53982	100.7431	99.5215
1151.295	99.52061	100.7156	99.45601
1153.223	99.49745	100.6804	99.41056
1155.151	99.46533	100.6411	99.34303

1157.08	99.43029	100.6013	99.27135
1159.008	99.39434	100.5722	99.21917
1160.937	99.3616	100.5533	99.18259
1162.865	99.33323	100.5236	99.15405
1164.794	99.29187	100.4906	99.11414
1166.722	99.24915	100.4589	99.05819
1168.651	99.22501	100.4208	98.99756
1170.579	99.21051	100.3925	98.95702
1172.508	99.19476	100.3801	98.94654
1174.436	99.21157	100.3959	98.97783
1176.365	99.26009	100.4369	99.02606
1178.293	99.3052	100.4655	99.06062
1180.222	99.34408	100.4846	99.09637
1182.15	99.36742	100.516	99.14849
1184.078	99.38186	100.5297	99.18182
1186.007	99.40296	100.5421	99.20306
1187.935	99.41136	100.5554	99.2316
1189.864	99.42195	100.571	99.25704
1191.792	99.42503	100.5857	99.28395
1193.721	99.40337	100.5851	99.30106
1195.649	99.39059	100.5836	99.3046
1197.578	99.37988	100.5936	99.31619
1199.506	99.35973	100.5843	99.32655
1201.435	99.35647	100.5655	99.33614
1203.363	99.33945	100.5682	99.33849
1205.292	99.31363	100.5807	99.34852
1207.22	99.2916	100.5741	99.36074
1209.149	99.26356	100.5574	99.36199
1211.077	99.2337	100.5399	99.34647
1213.005	99.19806	100.5353	99.34745

1214.934	99.15966	100.5346	99.35344
1216.862	99.11693	100.519	99.3415
1218.791	99.07469	100.5016	99.32468
1220.719	99.02777	100.4931	99.30914
1222.648	98.965	100.465	99.2787
1224.576	98.91776	100.4359	99.24467
1226.505	98.88101	100.4212	99.22228
1228.433	98.83312	100.3917	99.18644
1230.362	98.77955	100.3742	99.15383
1232.29	98.73106	100.3593	99.11543
1234.219	98.69629	100.3407	99.07108
1236.147	98.67187	100.3113	99.0358
1238.076	98.64355	100.2927	99.00381
1240.004	98.62827	100.2873	98.97856
1241.932	98.61648	100.2778	98.96664
1243.861	98.61905	100.2808	98.95784
1245.789	98.6245	100.2937	98.95324
1247.718	98.63672	100.2786	98.93724
1249.646	98.65713	100.2666	98.93604
1251.575	98.67818	100.2714	98.95679
1253.503	98.69619	100.2868	98.97049
1255.432	98.71346	100.299	98.98969
1257.36	98.72914	100.3069	99.00688
1259.289	98.7502	100.3157	99.03767
1261.217	98.77735	100.3397	99.08495
1263.146	98.80506	100.3635	99.12453
1265.074	98.84043	100.3712	99.14258
1267.003	98.87012	100.3745	99.16483
1268.931	98.89529	100.384	99.18639
1270.859	98.90281	100.3891	99.19849

1272.788	98.91724	100.4023	99.21502
1274.716	98.93532	100.4177	99.23593
1276.645	98.94551	100.4339	99.26654
1278.573	98.94859	100.4423	99.2917
1280.502	98.94751	100.4525	99.30273
1282.43	98.94926	100.4561	99.30294
1284.359	98.93829	100.4507	99.30238
1286.287	98.92375	100.4395	99.309
1288.216	98.92957	100.4358	99.32173
1290.144	98.93561	100.4351	99.33324
1292.073	98.92194	100.4373	99.3403
1294.001	98.90923	100.4393	99.34609
1295.93	98.90602	100.4361	99.34552
1297.858	98.91772	100.4372	99.34462
1299.786	98.92602	100.4433	99.35154
1301.715	98.92318	100.4421	99.36182
1303.643	98.91566	100.4493	99.37086
1305.572	98.92839	100.4601	99.38081
1307.5	98.94956	100.4567	99.38683
1309.429	98.97324	100.4554	99.40402
1311.357	98.98125	100.4402	99.40263
1313.286	98.98814	100.4481	99.4072
1315.214	99.01047	100.4744	99.43143
1317.143	99.02582	100.4528	99.42505
1319.071	99.04742	100.4646	99.42437
1321	99.08351	100.5335	99.46912
1322.928	99.11334	100.5591	99.48868
1324.857	99.14948	100.5574	99.50195
1326.785	99.16306	100.5385	99.50004
1328.714	99.16374	100.5219	99.47964

1330.642	99.17746	100.5402	99.47607
1332.57	99.18736	100.5581	99.49157
1334.499	99.16776	100.5076	99.48769
1336.427	99.13549	100.3803	99.42873
1338.356	99.11299	100.2655	99.37538
1340.284	99.1696	100.3884	99.43678
1342.213	99.23895	100.5547	99.51881
1344.141	99.25955	100.5908	99.54784
1346.07	99.26333	100.5973	99.56518
1347.998	99.26268	100.5789	99.5578
1349.927	99.26902	100.5898	99.55507
1351.855	99.27365	100.5952	99.53882
1353.784	99.26685	100.5734	99.5236
1355.712	99.26888	100.5726	99.53249
1357.641	99.25841	100.5311	99.50323
1359.569	99.18398	100.3637	99.38841
1361.497	99.07644	100.1455	99.22407
1363.426	99.0748	100.2162	99.20145
1365.354	99.09933	100.3453	99.24715
1367.283	99.06892	100.3112	99.24529
1369.211	99.03996	100.3257	99.27402
1371.14	98.96672	100.2303	99.22444
1373.068	98.85424	99.99863	99.06463
1374.997	98.80795	100.0387	99.02116
1376.925	98.78539	100.1365	99.04849
1378.854	98.74866	100.1551	99.05955
1380.782	98.71304	100.1982	99.10022
1382.711	98.661	100.2129	99.13807
1384.639	98.60055	100.1051	99.14433
1386.568	98.55338	99.98812	99.13899

1388.496	98.58631	100.1468	99.24364
1390.424	98.58	100.223	99.28781
1392.353	98.47063	100.0262	99.19804
1394.281	98.35889	99.82794	99.10588
1396.21	98.35645	99.88011	99.14478
1398.138	98.36484	99.92661	99.15944
1400.067	98.45132	100.1472	99.27219
1401.995	98.492	100.212	99.33245
1403.924	98.4743	100.1196	99.28649
1405.852	98.5416	100.236	99.34045
1407.781	98.61887	100.33	99.38686
1409.709	98.66762	100.2997	99.35982
1411.638	98.71885	100.3093	99.36394
1413.566	98.73908	100.307	99.3608
1415.495	98.6856	100.1194	99.24939
1417.423	98.6047	99.78237	99.07098
1419.351	98.71843	99.96249	99.18406
1421.28	98.86473	100.2575	99.37032
1423.208	98.9119	100.277	99.3952
1425.137	98.9896	100.4255	99.47729
1427.065	99.00873	100.4037	99.46127
1428.994	98.96292	100.2555	99.37276
1430.922	98.95495	100.2917	99.37875
1432.851	98.896	100.2578	99.33903
1434.779	98.72807	99.92952	99.11121
1436.708	98.67829	99.90854	99.03542
1438.636	98.7513	100.234	99.21172
1440.565	98.75077	100.3052	99.23492
1442.493	98.71133	100.2718	99.18105
1444.422	98.64702	100.1798	99.11452

1446.35	98.55565	99.99152	98.99649
1448.278	98.54041	100.0005	98.96601
1450.207	98.53896	100.0592	98.95065
1452.135	98.44714	99.90931	98.81313
1454.064	98.27196	99.49124	98.58112
1455.992	98.05231	98.84531	98.20223
1457.921	98.21181	99.19479	98.3159
1459.849	98.44822	99.78159	98.61362
1461.778	98.47132	99.85249	98.6316
1463.706	98.34594	99.58726	98.42287
1465.635	98.36769	99.68652	98.44064
1467.563	98.4772	99.87943	98.62286
1469.492	98.51423	99.72933	98.69265
1471.42	98.55158	99.39738	98.66808
1473.349	98.80312	99.66643	98.94695
1475.277	99.03637	100.0318	99.25667
1477.205	99.24319	100.3948	99.5404
1479.134	99.33381	100.5414	99.68205
1481.062	99.36164	100.5635	99.74077
1482.991	99.38966	100.5883	99.79084
1484.919	99.29358	100.3649	99.68562
1486.848	99.09541	99.96598	99.46516
1488.776	98.93638	99.69518	99.30357
1490.705	98.97087	100.0044	99.461
1492.633	98.98218	100.2497	99.60114
1494.562	98.78083	99.94569	99.42416
1496.49	98.55543	99.65839	99.21059
1498.419	98.47454	99.76263	99.24823
1500.347	98.4304	99.92484	99.32381
1502.276	98.26615	99.80238	99.24394

1504.204	97.87701	99.04337	98.83354
1506.132	97.35151	98.04331	98.14538
1508.061	97.33305	98.58205	98.30663
1509.989	97.33133	99.06637	98.52659
1511.918	97.2018	99.15201	98.51369
1513.846	96.9643	98.91478	98.33489
1515.775	96.66222	98.50496	98.01414
1517.703	96.5125	98.45702	97.92365
1519.632	96.35471	98.19086	97.75541
1521.56	96.25251	98.15015	97.64581
1523.489	96.24567	98.44109	97.77247
1525.417	96.13689	98.46143	97.73396
1527.346	96.05499	98.62943	97.75443
1529.274	95.95574	98.75532	97.77243
1531.203	95.72755	98.45782	97.59415
1533.131	95.50948	98.23903	97.38531
1535.059	95.45404	98.47794	97.46101
1536.988	95.19385	98.05217	97.222
1538.916	94.76898	97.12027	96.57743
1540.845	94.69653	97.28742	96.5497
1542.773	94.79202	97.75861	96.8159
1544.702	94.89221	98.19497	97.00603
1546.63	95.02579	98.52166	97.16577
1548.559	95.0808	98.44125	97.11382
1550.487	95.24619	98.54723	97.17468
1552.416	96.54378	98.55357	97.22156
1554.344	96.90892	98.41405	97.19009
1556.273	97.71605	97.94869	97.01845
1558.201	97.71621	97.40335	96.73425
1560.13	97.74003	98.69442	97.46152

1562.058	97.97269	99.30775	97.88967
1563.986	98.12292	99.43518	98.03622
1565.915	98.19582	99.5305	98.16334
1567.843	98.26957	99.3429	98.12294
1569.772	98.32558	99.5004	98.23056
1571.7	98.34865	99.81802	98.45763
1573.629	98.36034	99.62459	98.4128
1575.557	98.35788	99.40273	98.29147
1577.486	98.33473	99.82403	98.55016
1579.414	98.32408	100.2222	98.76424
1581.343	98.31631	100.2141	98.83772
1583.271	98.29031	100.1984	98.86709
1585.2	98.20387	100.1817	98.86942
1587.128	98.09598	100.1305	98.85397
1589.057	98.0031	100.1145	98.825
1590.985	97.88689	100.1659	98.81354
1592.913	97.7427	100.1476	98.75031
1594.842	97.59152	100.0494	98.69549
1596.77	97.3879	99.98624	98.67683
1598.699	97.07701	99.94699	98.61765
1600.627	96.75019	99.82531	98.50121
1602.556	96.63208	99.7333	98.38467
1604.484	96.4239	99.7147	98.26906
1606.413	96.02703	99.62566	98.09723
1608.341	95.71347	99.23861	97.91309
1610.27	95.4361	98.87541	97.78204
1612.198	95.10896	99.07475	97.61464
1614.127	94.84025	99.04568	97.29784
1616.055	94.53778	98.6598	96.88471
1617.984	94.10224	98.56908	96.82046

1619.912	93.63467	98.5343	96.72943
1621.84	93.53611	98.41113	96.36942
1623.769	93.4212	98.41882	96.13042
1625.697	93.16331	98.22163	95.9856
1627.626	92.85604	97.59695	95.76359
1629.554	92.44585	97.11776	95.66325
1631.483	92.20296	97.48286	95.53259
1633.411	92.18909	97.67887	95.14568
1635.34	92.06488	97.60465	94.70497
1637.268	92.24848	97.29331	94.82801
1639.197	92.84557	96.58412	94.93549
1641.125	93.27733	96.37262	94.89611
1643.054	93.67986	96.69913	94.7486
1644.982	94.02533	96.3239	94.32594
1646.911	94.45905	95.94534	94.15975
1648.839	94.8902	96.94624	94.46466
1650.767	95.13443	97.54575	94.41328
1652.696	95.35617	97.78942	94.17572
1654.624	95.71622	97.77277	94.84882
1656.553	96.01199	97.87907	95.37099
1658.481	96.2498	98.25171	95.67036
1660.41	96.6065	98.23363	95.78645
1662.338	96.84782	97.98997	95.94565
1664.267	97.00842	98.21098	96.28398
1666.195	97.04349	98.5128	96.41695
1668.124	97.23495	98.53142	96.38842
1670.052	97.75364	98.861	96.61564
1671.981	98.05849	99.0836	96.93172
1673.909	98.35585	99.01196	97.06631
1675.838	98.62939	98.61166	97.34221

1677.766	98.70933	98.37008	97.58939
1679.694	98.80461	99.19379	97.66142
1681.623	98.96851	99.53873	97.56239
1683.551	99.06578	99.77573	97.5026
1685.48	99.44158	100.0003	98.06252
1687.408	99.64198	99.80788	98.36583
1689.337	99.74761	99.45835	98.59492
1691.265	99.95005	99.5209	98.82716
1693.194	100.0658	99.41369	98.80888
1695.122	100.1263	99.91328	98.66209
1697.051	100.0882	100.1651	98.77667
1698.979	99.97722	100.154	98.77321
1700.908	99.90552	100.4715	99.06667
1702.836	100.0824	100.6875	99.27148
1704.765	100.233	100.7109	99.30394
1706.693	100.2415	100.4753	99.50862
1708.621	100.2213	100.0652	99.65585
1710.55	100.1753	99.82379	99.70737
1712.478	100.0394	100.2718	99.59554
1714.407	99.88067	100.7279	99.38403
1716.335	99.69708	100.7642	99.26195
1718.264	99.63644	100.7544	99.4799
1720.192	99.82008	100.655	99.73668
1722.121	99.88101	100.3528	99.7559
1724.049	99.94683	100.0202	99.70091
1725.978	100.056	99.60691	99.58459
1727.906	100.1731	99.51887	99.35308
1729.835	100.2469	100.0698	99.10672
1731.763	100.2417	100.151	98.84539
1733.692	100.2862	100.2865	98.7255

1735.62	100.4315	100.5427	99.07127
1737.548	100.5906	100.7089	99.23938
1739.477	100.603	100.7665	99.43691
1741.405	100.6335	100.6414	99.69922
1743.334	100.6636	100.6487	99.91345
1745.262	100.6418	100.9683	100.0631
1747.191	100.7164	101.2844	100.0691
1749.119	100.7676	101.2013	100.1204
1751.048	100.7044	101.1747	100.3358
1752.976	100.662	101.211	100.5631
1754.905	100.5802	101.1024	100.5473
1756.833	100.6085	101.2671	100.543
1758.762	100.7367	101.3629	100.5934
1760.69	100.7979	101.1841	100.5578
1762.619	100.7898	101.0264	100.6557
1764.547	100.7663	100.7627	100.7207
1766.475	100.7881	100.8293	100.6228
1768.404	100.7909	101.1776	100.5506
1770.332	100.8156	101.3441	100.4165
1772.261	100.8143	101.3128	100.4624
1774.189	100.7073	101.2647	100.6633
1776.118	100.6662	101.3516	100.7552
1778.046	100.7665	101.3616	100.7554
1779.975	100.8215	101.4306	100.7424
1781.903	100.8039	101.3858	100.7871
1783.832	100.7779	101.076	100.7875
1785.76	100.7959	100.9197	100.8398
1787.689	100.8406	101.1615	100.8273
1789.617	100.8657	101.2978	100.6648
1791.546	100.8613	101.253	100.5717

1793.474	100.8286	101.1548	100.7034
1795.402	100.8404	101.2511	100.7884
1797.331	100.8903	101.4285	100.7612
1799.259	100.9021	101.4748	100.7118
1801.188	100.9027	101.4117	100.7619
1803.116	100.9086	101.3176	100.8628
1805.045	100.8927	101.3326	100.8927
1806.973	100.8422	101.4669	100.8601
1808.902	100.8126	101.5059	100.8238
1810.83	100.8299	101.4896	100.8415
1812.759	100.7708	101.5126	100.8998
1814.687	100.8059	101.508	100.9207
1816.616	100.8902	101.3644	100.9201
1818.544	100.9004	101.2363	100.9499
1820.473	100.8938	101.2488	100.9557
1822.401	100.9066	101.0795	100.8709
1824.329	100.8966	101.2091	100.788
1826.258	100.8137	101.4765	100.8067
1828.186	100.7651	101.516	100.729
1830.115	100.8302	101.4829	100.7842
1832.043	100.8782	101.4958	100.9304
1833.972	100.9135	101.4729	100.9503
1835.9	100.9311	101.2354	100.9272
1837.829	100.9325	101.0927	100.9524
1839.757	100.9255	101.2777	100.9587
1841.686	100.9379	101.4072	100.8227
1843.614	100.9519	101.5079	100.7179
1845.543	100.943	101.5449	100.822
1847.471	100.923	101.5575	100.9028
1849.4	100.8566	101.5571	100.9616

1851.328	100.7569	101.5357	100.9826
1853.256	100.7803	101.5283	100.9886
1855.185	100.8764	101.5386	100.9916
1857.113	100.9123	101.5147	100.9825
1859.042	100.9278	101.372	100.9679
1860.97	100.929	101.1321	100.9715
1862.899	100.9177	101.1753	100.9771
1864.827	100.9137	101.4454	100.9001
1866.756	100.9151	101.5528	100.7688
1868.684	100.915	101.5591	100.7887
1870.613	100.9057	101.5496	100.9389
1872.541	100.8692	101.5532	100.9969
1874.47	100.8866	101.5523	100.9977
1876.398	100.9177	101.5341	100.9958
1878.327	100.9119	101.5323	100.9985
1880.255	100.9159	101.4992	100.9925
1882.183	100.936	101.3839	100.9935
1884.112	100.9351	101.43	100.9972
1886.04	100.9212	101.5129	100.9803
1887.969	100.9166	101.4651	100.9101
1889.897	100.9151	101.5059	100.9272
1891.826	100.9041	101.5762	100.983
1893.754	100.9032	101.5824	100.96
1895.683	100.9228	101.5548	100.9741
1897.611	100.9219	101.551	101.0076
1899.54	100.8893	101.5329	101.0051
1901.468	100.8549	101.4833	100.9993
1903.396	100.8786	101.4604	100.9969
1905.325	100.8888	101.5198	100.9821
1907.253	100.893	101.5517	100.9583

1909.182	100.9212	101.4717	100.9604
1911.11	100.9184	101.3709	100.9961
1913.039	100.918	101.4403	101.0122
1914.967	100.9319	101.4129	100.9675
1916.896	100.9415	101.4102	100.9053
1918.824	100.9302	101.5152	100.9519
1920.753	100.9146	101.563	100.9435
1922.681	100.9066	101.5805	100.9362
1924.61	100.8833	101.5868	100.9936
1926.538	100.858	101.5866	101.0174
1928.467	100.8817	101.5785	101.0152
1930.395	100.8963	101.5596	101.009
1932.323	100.9178	101.5391	101.0101
1934.252	100.9247	101.4333	101.0173
1936.18	100.9142	101.3687	101.0135
1938.109	100.9128	101.4596	100.996
1940.037	100.9246	101.526	100.9316
1941.966	100.9257	101.5548	100.8818
1943.894	100.9223	101.5708	100.927
1945.823	100.9351	101.5757	100.9592
1947.751	100.9238	101.5614	100.9796
1949.68	100.8942	101.5643	101.0043
1951.608	100.8949	101.5723	101.0114
1953.537	100.9136	101.5716	101.0068
1955.465	100.9221	101.5748	101.0129
1957.394	100.9215	101.5491	101.0012
1959.322	100.9245	101.4802	100.9977
1961.25	100.9221	101.4916	101.0108
1963.179	100.9151	101.5637	100.9991
1965.107	100.9213	101.567	100.9555

1967.036	100.9146	101.5629	100.9609
1968.964	100.8992	101.5729	101.0027
1970.893	100.8862	101.5679	101.0037
1972.821	100.8801	101.5683	100.991
1974.75	100.8597	101.5775	100.9879
1976.678	100.861	101.5785	100.9843
1978.607	100.8974	101.5624	100.9833
1980.535	100.9197	101.5312	100.9811
1982.464	100.9127	101.5165	100.9806
1984.392	100.9024	101.4674	100.9799
1986.321	100.9135	101.4923	100.9629
1988.249	100.9234	101.5761	100.9458
1990.177	100.9088	101.5911	100.9156
1992.106	100.9149	101.5754	100.931
1994.034	100.9316	101.5771	100.9827
1995.963	100.9412	101.5848	100.9809
1997.891	100.9192	101.5812	100.9642
1999.82	100.8951	101.5615	100.9734
2001.748	100.8827	101.5619	100.9884
2003.677	100.8886	101.575	100.9895
2005.605	100.8993	101.5813	100.9781
2007.534	100.9015	101.5671	100.9825
2009.462	100.9045	101.5234	100.9899
2011.391	100.9015	101.5167	100.9881
2013.319	100.8944	101.5401	100.98
2015.248	100.8919	101.5674	100.9525
2017.176	100.8934	101.5838	100.9411
2019.104	100.8993	101.5742	100.9561
2021.033	100.9102	101.5691	100.9648
2022.961	100.9018	101.5692	100.9716

2024.89	100.878	101.5583	100.9773
2026.818	100.884	101.5563	100.9786
2028.747	100.8968	101.5638	100.9793
2030.675	100.9014	101.5585	100.9683
2032.604	100.897	101.5295	100.9482
2034.532	100.9101.5183	100.9379	
2036.461	100.9056	101.5491	100.9471
2038.389	100.8812	101.5747	100.9438
2040.318	100.8546	101.5786	100.9339
2042.246	100.8711	101.578	100.9423
2044.175	100.8891	101.5772	100.9541
2046.103	100.8951	101.5763	100.9588
2048.031	100.8848	101.5706	100.9543
2049.96	100.8755	101.5616	100.9348
2051.888	100.8786	101.5629	100.9426
2053.817	100.8798	101.5652	100.9659
2055.745	100.8865	101.5532	100.9674
2057.674	100.8966	101.5373	100.9494
2059.602	100.8867	101.5552	100.9223
2061.531	100.8765	101.5723	100.9092
2063.459	100.8736	101.5685	100.9186
2065.388	100.8831	101.5776	100.927
2067.316	100.8868	101.5713	100.9178
2069.245	100.8835	101.5518	100.9189
2071.173	100.8831	101.5405	100.9342
2073.102	100.8901	101.5596	100.9327
2075.03	100.8911	101.5744	100.9168
2076.958	100.8828	101.5669	100.9162
2078.887	100.8761	101.5561	100.9303
2080.815	100.8796	101.5439	100.9381

2082.744	100.8599	101.5379	100.9376
2084.672	100.8395	101.5586	100.9267
2086.601	100.8497	101.5694	100.9127
2088.529	100.8642	101.5707	100.9071
2090.458	100.8671	101.5686	100.9087
2092.386	100.8699	101.5708	100.9116
2094.315	100.8704	101.5772	100.9179
2096.243	100.8692	101.575	100.9255
2098.172	100.8749	101.5734	100.9272
2100.1	100.8856	101.5658	100.9234
2102.029	100.8911	101.5585	100.9199
2103.957	100.8873	101.565	100.9225
2105.885	100.8768	101.5724	100.9129
2107.814	100.8619	101.5716	100.9073
2109.742	100.8496	101.5789	100.9138
2111.671	100.8575	101.5925	100.9225
2113.599	100.8687	101.5877	100.9283
2115.528	100.8613	101.5786	100.9278
2117.456	100.8664	101.5775	100.9244
2119.385	100.8778	101.5947	100.9271
2121.313	100.8781	101.5897	100.9262
2123.242	100.8748	101.5747	100.9153
2125.17	100.8686	101.5714	100.9219
2127.099	100.865	101.5742	100.9232
2129.027	100.8566	101.5815	100.9215
2130.956	100.8547	101.5949	100.9229
2132.884	100.8614	101.6016	100.9179
2134.813	100.8753	101.5817	100.9321
2136.741	100.8734	101.568	100.9439
2138.669	100.8651	101.5799	100.9423

2140.598	100.8669	101.5883	100.9324
2142.526	100.8693	101.5903	100.9296
2144.455	100.8689	101.5959	100.928
2146.383	100.8718	101.5972	100.9243
2148.312	100.8749	101.5905	100.9228
2150.24	100.8775	101.5835	100.9328
2152.169	100.8844	101.5793	100.9425
2154.097	100.8839	101.57	100.9332
2156.026	100.8741	101.5747	100.9245
2157.954	100.8905	101.5785	100.9233
2159.883	100.8886	101.5687	100.9247
2161.811	100.8825	101.5765	100.9416
2163.74	100.8732	101.5908	100.9454
2165.668	100.8715	101.5792	100.9247
2167.596	100.8811	101.5772	100.933
2169.525	100.8819	101.5789	100.9419
2171.453	100.8806	101.5663	100.9268
2173.382	100.8854	101.5642	100.9193
2175.31	100.8777	101.5758	100.9254
2177.239	100.8671	101.59	100.9243
2179.167	100.8649	101.6022	100.9221
2181.096	100.8699	101.6006	100.9247
2183.024	100.8794	101.5836	100.9413
2184.953	100.8903	101.5728	100.9565
2186.881	100.898	101.5685	100.9624
2188.81	100.9057	101.5756	100.9512
2190.738	100.9003	101.5867	100.9444
2192.667	100.8897	101.5892	100.94
2194.595	100.8698	101.588	100.9437
2196.523	100.8688	101.5977	100.9583

2198.452	100.8755	101.5909	100.9688
2200.38	100.8749	101.5814	100.9613
2202.309	100.8765	101.5798	100.9667
2204.237	100.8749	101.5908	100.9694
2206.166	100.8822	101.5937	100.9691
2208.094	100.889	101.5864	100.9599
2210.023	100.8811	101.5881	100.9765
2211.951	100.8772	101.584	100.9957
2213.88	100.8893	101.5854	100.9821
2215.808	100.8951	101.5943	100.9828
2217.737	100.8953	101.5837	100.9903
2219.665	100.8929	101.5781	100.9928
2221.594	100.8812	101.588	100.9969
2223.522	100.885	101.5978	101.0046
2225.45	100.8938	101.5923	101.009
2227.379	100.8828	101.5905	101.0005
2229.307	100.8782	101.5822	100.9956
2231.236	100.893	101.5846	100.9971
2233.164	100.901	101.5785	101.0031
2235.093	100.8893	101.5673	101.0053
2237.021	100.8715	101.5646	100.9988
2238.95	100.8778	101.5678	100.9911
2240.878	100.8898	101.5669	100.9885
2242.807	100.8814	101.575	100.9879
2244.735	100.876	101.5858	100.994
2246.664	100.8857	101.5792	100.9993
2248.592	100.8858	101.5765	101.0051
2250.521	100.8781	101.5581	101.0129
2252.449	100.8799	101.5413	101.0079
2254.377	100.8788	101.5442	101.0134

2256.306	100.8782	101.5514	101.0227
2258.234	100.8749	101.5601	101.0168
2260.163	100.8724	101.5661	101.0076
2262.091	100.8763	101.5613	101.0169
2264.02	100.8761	101.5656	101.0261
2265.948	100.8784	101.5738	101.0286
2267.877	100.8934	101.5795	101.0233
2269.805	100.8805	101.584	101.0223
2271.734	100.8749	101.5844	101.0161
2273.662	100.8803	101.582	101.0194
2275.591	100.8684	101.5725	101.0353
2277.519	100.8647	101.5735	101.0362
2279.448	100.8654	101.5756	101.0286
2281.376	100.8567	101.5607	101.0188
2283.304	100.8575	101.5438	101.0218
2285.233	100.8631	101.5279	101.0371
2287.161	100.8634	101.5035	101.0367
2289.09	100.839	101.4914	101.0186
2291.018	100.8134	101.494	101.0121
2292.947	100.8185	101.4698	101.0018
2294.875	100.8178	101.4467	100.997
2296.804	100.8097	101.4266	100.9969
2298.732	100.7998	101.3914	100.9933
2300.661	100.7582	101.3443	100.9845
2302.589	100.7261	101.2793	100.9646
2304.518	100.7362	101.2236	100.9549
2306.446	100.7563	101.1902	100.9594
2308.375	100.7605	101.1684	100.9467
2310.303	100.734	101.1261	100.9369
2312.231	100.7049	101.0765	100.9305

2314.16	100.7107	101.0137	100.9292
2316.088	100.7106	100.9442	100.9046
2318.017	100.7049	100.9242	100.8868
2319.945	100.6936	100.9028	100.8787
2321.874	100.6884	100.8506	100.86
2323.802	100.6844	100.8319	100.8496
2325.731	100.7054	100.8069	100.843
2327.659	100.7393	100.7928	100.8346
2329.588	100.7521	100.7815	100.8304
2331.516	100.7468	100.7705	100.8238
2333.445	100.7221	100.7636	100.8131
2335.373	100.6972	100.7533	100.8167
2337.302	100.6577	100.7791	100.8405
2339.23	100.5964	100.8854	100.8491
2341.158	100.576	101.0112	100.8219
2343.087	100.5819	100.9884	100.791
2345.015	100.5924	100.8358	100.8052
2346.944	100.6278	100.6738	100.864
2348.872	100.6135	100.5537	100.9177
2350.801	100.6093	100.478	100.8929
2352.729	100.6414	100.4325	100.8284
2354.658	100.6677	100.4389	100.7875
2356.586	100.6808	100.4489	100.7481
2358.515	100.7257	100.4948	100.7248
2360.443	100.7675	100.6083	100.7203
2362.372	100.7906	100.7202	100.6997
2364.3	100.8226	100.8131	100.6856
2366.229	100.8507	100.9112	100.7448
2368.157	100.8654	101.0185	100.7978
2370.085	100.8558	101.1622	100.8323

2372.014	100.8578	101.3074	100.8694
2373.942	100.8694	101.414	100.8804
2375.871	100.866	101.4951	100.907
2377.799	100.8761	101.5402	100.9499
2379.728	100.88	101.5649	100.9963
2381.656	100.8768	101.5771	101.0336
2383.585	100.8832	101.5732	101.0509
2385.513	100.8831	101.5813	101.0677
2387.442	100.8719	101.6014	101.0859
2389.37	100.8745	101.6173	101.0848
2391.299	100.8764	101.6042	101.0792
2393.227	100.8825	101.5776	101.087
2395.156	100.884	101.574	101.1044
2397.084	100.8755	101.5732	101.1071
2399.012	100.8691	101.563	101.0936
2400.941	100.8691	101.5776	101.0874
2402.869	100.8729	101.5862	101.0838
2404.798	100.8599	101.5657	101.0742
2406.726	100.851	101.5541	101.0722
2408.655	100.86	101.5751	101.0728
2410.583	100.8624	101.6089	101.0692
2412.512	100.8588	101.621	101.0703
2414.44	100.8603	101.6096	101.0752
2416.369	100.8803	101.596	101.0806
2418.297	100.8716	101.5907	101.0806
2420.226	100.8745	101.5888	101.0847
2422.154	100.8767	101.5819	101.0827
2424.083	100.861	101.5808	101.0838
2426.011	100.8659	101.597	101.0876
2427.939	100.8644	101.6089	101.0738

2429.868	100.8689	101.5958	101.0722
2431.796	100.8697	101.5846	101.0835
2433.725	100.8635	101.5948	101.091
2435.653	100.859	101.6053	101.0918
2437.582	100.858	101.6126	101.088
2439.51	100.8613	101.5985	101.0838
2441.439	100.8559	101.5893	101.0879
2443.367	100.8583	101.591	101.1044
2445.296	100.858	101.5838	101.1112
2447.224	100.8684	101.5896	101.1011
2449.153	100.8894	101.6097	101.1056
2451.081	100.872	101.617	101.1219
2453.01	100.842	101.6157	101.1362
2454.938	100.8363	101.6126	101.1444
2456.866	100.8419	101.6096	101.1309
2458.795	100.8503	101.6182	101.1104
2460.723	100.8566	101.605	101.0962
2462.652	100.8337	101.5915	101.0852
2464.58	100.8328	101.598	101.1002
2466.509	100.8553	101.614	101.1161
2468.437	100.8527	101.6201	101.1214
2470.366	100.8508	101.6101	101.1156
2472.294	100.849	101.5957	101.1093
2474.223	100.8347	101.5939	101.1272
2476.151	100.8393	101.6178	101.1205
2478.08	100.859	101.6199	101.0965
2480.008	100.8672	101.6098	101.1042
2481.937	100.8682	101.6052	101.1262
2483.865	100.8569	101.6124	101.1266
2485.793	100.85	101.6094	101.1203

2487.722	100.8483	101.6055	101.1189
2489.65	100.8449	101.6118	101.1345
2491.579	100.8405	101.6239	101.1339
2493.507	100.848	101.6221	101.1239
2495.436	100.8517	101.6186	101.1267
2497.364	100.8403	101.6196	101.1317
2499.293	100.8279	101.6123	101.1362
2501.221	100.8275	101.5982	101.1376
2503.15	100.827	101.5968	101.1444
2505.078	100.8287	101.606	101.1406
2507.007	100.8406	101.5946	101.1325
2508.935	100.841	101.5836	101.1277
2510.864	100.8344	101.5866	101.1334
2512.792	100.8355	101.5836	101.1262
2514.72	100.8216	101.5636	101.1094
2516.649	100.8167	101.5712	101.1124
2518.577	100.8307	101.5915	101.1179
2520.506	100.8416	101.591	101.1154
2522.434	100.832	101.586	101.1204
2524.363	100.8155	101.587	101.1198
2526.291	100.8131	101.6017	101.1239
2528.22	100.8358	101.6152	101.1371
2530.148	100.8391	101.5946	101.1315
2532.077	100.8086	101.5715	101.1221
2534.005	100.818	101.582	101.1373
2535.934	100.828	101.5946	101.1267
2537.862	100.8124	101.584	101.1232
2539.791	100.8061	101.5852	101.1373
2541.719	100.8	101.592	101.1415
2543.647	100.8055	101.587	101.1351

2545.576	100.8084	101.5896	101.13
2547.504	100.8028	101.5943	101.1472
2549.433	100.7935	101.5916	101.1482
2551.361	100.8047	101.5935	101.1428
2553.29	100.8034	101.6056	101.1444
2555.218	100.8045	101.6123	101.15
2557.147	100.8091	101.6042	101.1622
2559.075	100.8123	101.6005	101.1729
2561.004	100.8043	101.6077	101.1699
2562.932	100.7871	101.6033	101.1462
2564.861	100.7852	101.5833	101.137
2566.789	100.7941	101.5833	101.1513
2568.718	100.7878	101.5891	101.1605
2570.646	100.7622	101.6062	101.1548
2572.574	100.7786	101.6182	101.1428
2574.503	100.8063	101.6008	101.1351
2576.431	100.7967	101.5877	101.1434
2578.36	100.7807	101.5931	101.1547
2580.288	100.7918	101.5904	101.1439
2582.217	100.7849	101.5849	101.1289
2584.145	100.7663	101.5764	101.129
2586.074	100.7757	101.5726	101.1262
2588.002	100.7941	101.5837	101.1311
2589.931	100.7804	101.5922	101.1365
2591.859	100.7703	101.5895	101.1477
2593.788	100.7681	101.579	101.1629
2595.716	100.767	101.5675	101.1659
2597.645	100.7871	101.5605	101.1377
2599.573	100.8011	101.5667	101.1004
2601.501	100.7947	101.5722	101.1007

2603.43	100.7795	101.5564	101.1095
2605.358	100.7485	101.5612	101.1098
2607.287	100.7402	101.5799	101.1183
2609.215	100.7567	101.5767	101.113
2611.144	100.7666	101.5699	101.1001
2613.072	100.7724	101.576	101.1006
2615.001	100.7613	101.5581	101.0994
2616.929	100.7647	101.5458	101.1094
2618.858	100.7729	101.5565	101.1279
2620.786	100.7646	101.5607	101.1251
2622.715	100.7594	101.5579	101.1103
2624.643	100.7754	101.5691	101.0958
2626.572	100.7756	101.5715	101.0972
2628.5	100.7649	101.5683	101.1169
2630.428	100.7727	101.5542	101.1201
2632.357	100.7734	101.5592	101.1174
2634.285	100.7743	101.5767	101.1199
2636.214	100.7713	101.579	101.1146
2638.142	100.7497	101.5838	101.1224
2640.071	100.7278	101.5862	101.1454
2641.999	100.7324	101.5755	101.1372
2643.928	100.7284	101.5736	101.1315
2645.856	100.7169	101.5658	101.1398
2647.785	100.7238	101.5634	101.1259
2649.713	100.7281	101.5823	101.1176
2651.642	100.7171	101.5823	101.1227
2653.57	100.7153	101.5649	101.1309
2655.499	100.7263	101.5742	101.127
2657.427	100.7283	101.5676	101.1098
2659.355	100.7271	101.5582	101.1067

2661.284	100.7395	101.5631	101.1119
2663.212	100.7397	101.565	101.1116
2665.141	100.738	101.5763	101.1152
2667.069	100.7343	101.5873	101.1138
2668.998	100.7279	101.5762	101.1172
2670.926	100.7304	101.5672	101.1216
2672.855	100.7414	101.5597	101.1314
2674.783	100.722	101.5729	101.1353
2676.712	100.6942	101.5761	101.1263
2678.64	100.7016	101.5728	101.1122
2680.569	100.7272	101.5808	101.1194
2682.497	100.7374	101.5885	101.1338
2684.426	100.7354	101.5758	101.1312
2686.354	100.7247	101.5673	101.1398
2688.282	100.7345	101.5759	101.1517
2690.211	100.7346	101.5909	101.1383
2692.139	100.7231	101.5824	101.1198
2694.068	100.7162	101.5656	101.1093
2695.996	100.7081	101.5633	101.1134
2697.925	100.705	101.5559	101.1096
2699.853	100.7024	101.5616	101.1057
2701.782	100.6847	101.5789	101.1199
2703.71	100.7031	101.579	101.1029
2705.639	100.7076	101.571	101.0906
2707.567	100.6894	101.5565	101.1065
2709.496	100.6871	101.5586	101.1188
2711.424	100.6875	101.5704	101.1201
2713.353	100.6818	101.5662	101.1105
2715.281	100.6741	101.5605	101.1009
2717.209	100.6612	101.5668	101.1087

2719.138	100.6653	101.5468	101.1233
2721.066	100.6798	101.5447	101.1119
2722.995	100.6986	101.5702	101.1063
2724.923	100.6782	101.5829	101.1113
2726.852	100.66	101.5755	101.1072
2728.78	100.66	101.5812	101.1179
2730.709	100.6514	101.5721	101.1278
2732.637	100.6473	101.5553	101.1292
2734.566	100.6552	101.5657	101.1257
2736.494	100.6595	101.5659	101.1002
2738.423	100.6753	101.5478	101.0835
2740.351	100.677	101.5439	101.0971
2742.28	100.6594	101.5519	101.1122
2744.208	100.6594	101.5551	101.1081
2746.136	100.6576	101.5477	101.0764
2748.065	100.6489	101.5387	101.0507
2749.993	100.6634	101.5467	101.0659
2751.922	100.6568	101.5631	101.0971
2753.85	100.6482	101.5703	101.0989
2755.779	100.6484	101.5575	101.1028
2757.707	100.6301	101.5388	101.1163
2759.636	100.6236	101.5246	101.1054
2761.564	100.608	101.5158	101.086
2763.493	100.5972	101.5018	101.063
2765.421	100.6136	101.5086	101.0439
2767.35	100.622	101.5279	101.0512
2769.278	100.6301	101.5298	101.0516
2771.207	100.6315	101.5295	101.0554
2773.135	100.6289	101.5206	101.078
2775.063	100.6045	101.5156	101.0705

2776.992	100.5962	101.5168	101.052
2778.92	100.6104	101.506	101.0397
2780.849	100.6057	101.4927	101.0429
2782.777	100.568	101.507	101.0506
2784.706	100.5382	101.5161	101.0401
2786.634	100.5335	101.4948	101.0319
2788.563	100.5478	101.4846	101.0406
2790.491	100.544	101.494	101.0315
2792.42	100.5232	101.4894	101.0164
2794.348	100.5011	101.4692	101.0068
2796.277	100.4722	101.4612	101.012
2798.205	100.4598	101.4484	101.0019
2800.134	100.4687	101.4401	100.9868
2802.062	100.4639	101.4361	100.9851
2803.99	100.447	101.4079	100.9781
2805.919	100.4236	101.3872	100.9513
2807.847	100.4007	101.3961	100.9315
2809.776	100.3799	101.3978	100.9253
2811.704	100.3376	101.3883	100.9172
2813.633	100.3139	101.3611	100.9041
2815.561	100.3042	101.3477	100.8925
2817.49	100.2831	101.3198	100.8724
2819.418	100.2456	101.2957	100.8565
2821.347	100.1779	101.2891	100.8323
2823.275	100.099	101.2626	100.7974
2825.204	99.98862	101.2271	100.7713
2827.132	99.84364	101.2022	100.7464
2829.061	99.65464	101.1428	100.7085
2830.989	99.39747	101.0841	100.6669
2832.917	99.18673	101.0071	100.6145

2834.846	99.132	100.9204	100.5373
2836.774	99.20211	100.7979	100.4593
2838.703	99.34438	100.6132	100.3592
2840.631	99.49522	100.3932	100.2303
2842.56	99.60892	100.1988	100.0391
2844.488	99.67914	100.1471	99.78012
2846.417	99.71017	100.2036	99.49045
2848.345	99.73933	100.3103	99.25294
2850.274	99.75304	100.4399	99.19417
2852.202	99.73887	100.5735	99.28462
2854.131	99.75168	100.654	99.44562
2856.059	99.76771	100.6906	99.62589
2857.988	99.80596	100.707	99.79713
2859.916	99.84256	100.7045	99.92284
2861.844	99.86678	100.6999	100.0036
2863.773	99.86954	100.7107	100.0277
2865.701	99.87787	100.752	100.0173
2867.63	99.87043	100.8196	100.009
2869.558	99.84016	100.8446	100.0316
2871.487	99.78355	100.8393	100.0804
2873.415	99.75001	100.8355	100.1242
2875.344	99.74023	100.8398	100.1526
2877.272	99.68892	100.8581	100.1642
2879.201	99.61841	100.8144	100.1567
2881.129	99.56782	100.7783	100.1303
2883.058	99.53329	100.7609	100.1086
2884.986	99.47553	100.7195	100.0822
2886.915	99.40887	100.6828	100.0384
2888.843	99.33527	100.6578	100.005
2890.771	99.23032	100.6102	99.95979

2892.7	99.09427	100.5373	99.91362
2894.628	98.93079	100.4722	99.8803
2896.557	98.69913	100.426	99.80922
2898.485	98.45348	100.3766	99.71782
2900.414	98.2588	100.2891	99.66323
2902.342	98.1282	100.1631	99.6004
2904.271	98.08663	99.30836	99.50242
2906.199	98.1158	99.24734	99.37619
2908.128	98.16743	99.23888	99.18608
2910.056	98.24621	99.21545	98.95361
2911.985	98.35018	99.20387	98.68868
2913.913	98.48495	99.20903	98.42273
2915.842	98.6144	99.19942	98.20901
2917.77	98.7246	99.6814	98.06903
2919.698	98.85334	99.78835	97.9928
2921.627	99.00362	99.90036	97.98463
2923.555	99.09272	100.0294	98.03603
2925.484	99.13694	100.1311	98.16808
2927.412	99.20277	100.2041	98.34572
2929.341	99.23682	100.2537	98.50255
2931.269	99.25144	100.2654	98.63528
2933.198	99.24738	100.2771	98.75605
2935.126	99.23604	100.2969	98.90012
2937.055	99.24196	100.3253	99.07913
2938.983	99.23651	100.372	99.20264
2940.912	99.23968	100.4176	99.26685
2942.84	99.28622	100.4162	99.31228
2944.769	99.35996	100.4091	99.34827
2946.697	99.41148	100.4505	99.36237
2948.625	99.45	100.5275	99.37347

2950.554	99.53405	100.6024	99.4217
2952.482	99.62479	100.6665	99.50116
2954.411	99.69917	100.7416	99.54482
2956.339	99.78407	100.8156	99.52751
2958.268	99.84595	100.8857	99.54723
2960.196	99.87143	100.9383	99.6038
2962.125	99.91295	100.979	99.67415
2964.053	99.94395	101.0155	99.74734
2965.982	99.98649	101.0454	99.83437
2967.91	100.0424	101.0908	99.92576
2969.839	100.0849	101.1335	100.0182
2971.767	100.1057	101.1535	100.0993
2973.696	100.1202	101.1791	100.1551
2975.624	100.1489	101.2332	100.1873
2977.552	100.178	101.2534	100.2145
2979.481	100.1626	101.2434	100.2373
2981.409	100.1624	101.261	100.2788
2983.338	100.1727	101.2561	100.3464
2985.266	100.1568	101.218	100.3785
2987.195	100.1291	101.2223	100.3727
2989.123	100.1433	101.2544	100.4118
2991.052	100.16	101.2933	100.4493
2992.98	100.1411	101.3033	100.4425
2994.909	100.132	101.297	100.4207
2996.837	100.1287	101.2798	100.399
2998.766	100.1391	101.2769	100.3977
3000.694	100.1663	101.3123	100.4068
3002.623	100.2049	101.3043	100.4232
3004.551	100.2036	101.303	100.4504
3006.479	100.1418	101.3409	100.4357

3008.408	100.1214	101.3495	100.3918
3010.336	100.1372	101.3306	100.3732
3012.265	100.1301	101.3122	100.3835
3014.193	100.1184	101.3209	100.4046
3016.122	100.1095	101.3401	100.4027
3018.05	100.1021	101.3318	100.4081
3019.979	100.101	101.315	100.4441
3021.907	100.1142	101.307	100.4368
3023.836	100.1237	101.3181	100.3824
3025.764	100.1096	101.309	100.3649
3027.693	100.0802	101.3062	100.3939
3029.621	100.0825	101.3245	100.3717
3031.55	100.0679	101.3235	100.325
3033.478	100.0413	101.2988	100.3226
3035.406	100.0396	101.2945	100.3027
3037.335	100.0248	101.316	100.2831
3039.263	100.0021	101.3178	100.2792
3041.192	100.0074	101.2942	100.2656
3043.12	100.0137	101.2871	100.2457
3045.049	99.98138	101.3162	100.2523
3046.977	99.99993	101.323	100.2493
3048.906	100.0353	101.3003	100.227
3050.834	100.0083	101.2734	100.2075
3052.763	99.98085	101.2569	100.1916
3054.691	100.0061	101.258	100.1771
3056.62	100.0174	101.2738	100.1398
3058.548	100.0054	101.273	100.1288
3060.477	100.0023	101.2583	100.1308
3062.405	99.99901	101.2668	100.1161
3064.333	100.0036	101.2779	100.1051

3066.262	100.0096	101.2788	100.0687
3068.19	100.0096	101.3035	100.038
3070.119	100.0089	101.2961	100.0326
3072.047	99.99587	101.2894	100.0216
3073.976	100.0219	101.3161	99.98609
3075.904	100.0329	101.3202	99.95934
3077.833	99.99538	101.302	99.94223
3079.761	100.0152	101.2792	99.93787
3081.69	100.0542	101.2832	99.94228
3083.618	100.0475	101.2926	99.93279
3085.547	100.0325	101.2771	99.92004
3087.475	100.0202	101.2888	99.90553
3089.404	100.0152	101.3073	99.88737
3091.332	100.007	101.2725	99.87497
3093.26	100.0037	101.2643	99.85271
3095.189	100.0033	101.2967	99.82909
3097.117	99.98658	101.2882	99.82203
3099.046	99.97162	101.2809	99.80404
3100.974	99.976	101.2774	99.8075
3102.903	99.97362	101.2862	99.79184
3104.831	99.97909	101.2854	99.74709
3106.76	99.98858	101.2957	99.74048
3108.688	99.98723	101.3095	99.7588
3110.617	99.99296	101.2947	99.7378
3112.545	99.99075	101.2868	99.69482
3114.474	99.97146	101.3074	99.65976
3116.402	99.95904	101.3358	99.64595
3118.331	99.95138	101.3351	99.64173
3120.259	99.91451	101.3069	99.62611
3122.188	99.92547	101.2653	99.57996

3124.116	99.93861	101.2813	99.54558
3126.044	99.93346	101.3138	99.5572
3127.973	99.91837	101.3121	99.53521
3129.901	99.89647	101.2895	99.52339
3131.83	99.89274	101.2621	99.50344
3133.758	99.87623	101.2501	99.44359
3135.687	99.87789	101.2639	99.39741
3137.615	99.84974	101.2614	99.38512
3139.544	99.79115	101.25	99.35568
3141.472	99.79939	101.2423	99.3218
3143.401	99.8246	101.2504	99.28022
3145.329	99.80901	101.2434	99.24454
3147.258	99.79438	101.2437	99.21682
3149.186	99.77918	101.2297	99.21127
3151.115	99.75327	101.203	99.18553
3153.043	99.72047	101.1896	99.15514
3154.971	99.70589	101.1864	99.14737
3156.9	99.67413	101.1825	99.12782
3158.828	99.64492	101.1784	99.08522
3160.757	99.61552	101.1651	99.06136
3162.685	99.59557	101.1481	99.02784
3164.614	99.60702	101.1394	98.97418
3166.542	99.59809	101.1184	98.92118
3168.471	99.58552	101.108	98.8701
3170.399	99.59666	101.105	98.81821
3172.328	99.6207	101.1099	98.78857
3174.256	99.59815	101.1286	98.77601
3176.185	99.53884	101.1335	98.73255
3178.113	99.51532	101.1019	98.684
3180.042	99.5205	101.0771	98.62494

3181.97	99.53478	101.066	98.61601
3183.898	99.50686	101.0534	98.62186
3185.827	99.4614	101.0547	98.57262
3187.755	99.45609	101.067	98.53967
3189.684	99.44701	101.047	98.54656
3191.612	99.44794	101.0339	98.48593
3193.541	99.45448	101.0064	98.40724
3195.469	99.44982	100.9864	98.37482
3197.398	99.43015	100.9903	98.36201
3199.326	99.4153	101.0148	98.35036
3201.255	99.40685	101.0228	98.32452
3203.183	99.4104	101.014	98.27595
3205.112	99.42781	101.0042	98.23002
3207.04	99.40971	101.0193	98.20663
3208.969	99.3837	101.0176	98.16166
3210.897	99.37851	100.9864	98.08498
3212.825	99.38309	100.9756	98.04212
3214.754	99.38388	100.9682	98.04302
3216.682	99.36068	100.965	98.07991
3218.611	99.30843	100.983	98.0715
3220.539	99.29976	100.9725	98.03952
3222.468	99.30447	100.9637	97.97451
3224.396	99.26976	100.9657	97.92258
3226.325	99.23408	100.9641	97.9152
3228.253	99.22897	100.9878	97.88743
3230.182	99.22115	101.002	97.85582
3232.11	99.19283	100.9632	97.84499
3234.039	99.17514	100.9571	97.82616
3235.967	99.15552	100.966	97.8364
3237.896	99.10936	100.9047	97.8307

3239.824	99.05367	100.8752	97.79231
3241.752	99.04576	100.8934	97.7569
3243.681	99.06141	100.9097	97.72356
3245.609	99.0112	100.8682	97.68304
3247.538	98.94524	100.8206	97.68089
3249.466	98.91709	100.7944	97.68484
3251.395	98.90672	100.8067	97.61349
3253.323	98.91438	100.8251	97.57888
3255.252	98.90754	100.8175	97.57238
3257.18	98.88902	100.8013	97.51763
3259.109	98.84383	100.7988	97.45651
3261.037	98.78644	100.7766	97.42634
3262.966	98.79389	100.7733	97.41357
3264.894	98.82285	100.7956	97.45149
3266.823	98.8072	100.7873	97.45707
3268.751	98.79245	100.7736	97.40636
3270.679	98.80874	100.761	97.36611
3272.608	98.8383	100.7681	97.33044
3274.536	98.83966	100.7715	97.27044
3276.465	98.83103	100.7739	97.24038
3278.393	98.84061	100.7914	97.25336
3280.322	98.85605	100.7598	97.25368
3282.25	98.85722	100.7553	97.23434
3284.179	98.84795	100.8007	97.21117
3286.107	98.85237	100.8222	97.18494
3288.036	98.86727	100.8082	97.19285
3289.964	98.86326	100.8013	97.20609
3291.893	98.87788	100.7918	97.21445
3293.821	98.93438	100.7927	97.20002
3295.75	98.96217	100.8189	97.20753

3297.678	98.93829	100.8142	97.2266
3299.606	98.93301	100.8315	97.20659
3301.535	98.96626	100.8575	97.18469
3303.463	99.02012	100.8712	97.17358
3305.392	99.05563	100.852	97.17422
3307.32	99.04267	100.8324	97.18152
3309.249	99.04459	100.8253	97.17265
3311.177	99.0802	100.8996	97.17627
3313.106	99.10468	100.9287	97.18693
3315.034	99.13177	100.9215	97.19477
3316.963	99.1545	100.928	97.18992
3318.891	99.14766	100.9082	97.15187
3320.82	99.12852	100.9128	97.12456
3322.748	99.10443	100.9318	97.16629
3324.677	99.11497	100.953	97.2326
3326.605	99.13866	100.9831	97.19302
3328.533	99.18549	100.9887	97.16565
3330.462	99.24451	100.9679	97.17426
3332.39	99.2343	100.9265	97.14214
3334.319	99.19328	100.9058	97.15929
3336.247	99.21037	100.9573	97.17942
3338.176	99.28916	101.0009	97.1649
3340.104	99.31712	100.9628	97.15161
3342.033	99.30903	100.9592	97.16738
3343.961	99.30378	100.9812	97.15843
3345.89	99.30458	101.0094	97.15192
3347.818	99.32666	100.9951	97.12778
3349.747	99.35287	100.9836	97.12774
3351.675	99.36485	101.0138	97.12632
3353.604	99.35593	101.0528	97.07443

3355.532	99.35666	101.0614	97.08346
3357.46	99.3541	101.0216	97.10818
3359.389	99.37186	101.0145	97.13771
3361.317	99.4116	101.0629	97.11674
3363.246	99.43793	101.114	97.06917
3365.174	99.41184	101.1083	97.08945
3367.103	99.3943	101.0409	97.08401
3369.031	99.41334	101.0324	97.03758
3370.959	99.45034	101.0864	97.07742
3372.888	99.46556	101.1005	97.09522
3374.816	99.4506	101.0614	97.07935
3376.745	99.44721	101.0341	97.10204
3378.673	99.44843	101.0655	97.12833
3380.602	99.49052	101.0675	97.10746
3382.53	99.52271	101.0664	97.10019
3384.459	99.52453	101.0628	97.10204
3386.387	99.47336	101.0644	97.08311
3388.316	99.4762	101.0716	97.08751
3390.244	99.51917	101.0571	97.11517
3392.173	99.5361	101.0889	97.13388
3394.101	99.53834	101.1503	97.10825
3396.03	99.56037	101.1855	97.09749
3397.958	99.54239	101.1814	97.11834
3399.886	99.52547	101.1756	97.12983
3401.815	99.55695	101.1356	97.13448
3403.743	99.57598	101.0881	97.13758
3405.672	99.60123	101.0696	97.15984
3407.6	99.60524	101.0876	97.16341
3409.529	99.595	101.1568	97.17365
3411.457	99.6174	101.1894	97.18689

3413.386	99.61577	101.1901	97.19022
3415.314	99.6039	101.198	97.20553
3417.243	99.63594	101.1982	97.20613
3419.171	99.66801	101.1575	97.22436
3421.1	99.71535	101.1378	97.24986
3423.028	99.72659	101.1902	97.29576
3424.957	99.7065	101.1869	97.34814
3426.885	99.73454	101.1724	97.37689
3428.813	99.70585	101.1797	97.37583
3430.742	99.6658	101.1396	97.40647
3432.67	99.71162	101.1392	97.38396
3434.599	99.73578	101.2161	97.39874
3436.527	99.74086	101.2154	97.44875
3438.456	99.77318	101.2247	97.45927
3440.384	99.79745	101.2492	97.47315
3442.313	99.7695	101.2506	97.50942
3444.241	99.76789	101.2243	97.55361
3446.17	99.8498	101.2065	97.54336
3448.098	99.86575	101.2226	97.53699
3450.027	99.79321	101.2427	97.54902
3451.955	99.78334	101.2649	97.5816
3453.884	99.83816	101.2632	97.63995
3455.812	99.87454	101.2771	97.67039
3457.74	99.89466	101.295	97.65627
3459.669	99.85928	101.2942	97.69888
3461.597	99.84819	101.2987	97.73012
3463.526	99.89377	101.3067	97.74831
3465.454	99.90374	101.2794	97.79301
3467.383	99.88653	101.2347	97.80019
3469.311	99.91909	101.2835	97.85074

3471.24	99.9981	101.3331	97.89405
3473.168	100.0047	101.3279	97.90615
3475.097	99.95412	101.3484	97.95309
3477.025	99.95151	101.3465	98.01554
3478.954	99.96031	101.2971	98.02388
3480.882	99.9355	101.2732	97.99522
3482.811	99.93993	101.2892	98.01651
3484.739	99.94955	101.2825	98.07357
3486.667	99.94678	101.2614	98.10456
3488.596	99.97032	101.2876	98.14503
3490.524	99.99808	101.3542	98.19285
3492.453	100.0214	101.3547	98.20383
3494.381	100.0201	101.3058	98.21025
3496.31	100.0602	101.3368	98.24676
3498.238	100.1072	101.4074	98.2648
3500.167	100.0901	101.4361	98.25577
3502.095	100.0307	101.4051	98.30598
3504.024	100.0333	101.4033	98.39088
3505.952	100.0837	101.3935	98.40909
3507.881	100.0838	101.3207	98.40688
3509.809	100.0847	101.2908	98.4526
3511.738	100.1067	101.2827	98.50455
3513.666	100.1343	101.2949	98.54601
3515.594	100.1996	101.3678	98.53899
3517.523	100.2154	101.4563	98.54324
3519.451	100.2196	101.4902	98.61261
3521.38	100.2799	101.4845	98.61847
3523.308	100.2912	101.5073	98.65105
3525.237	100.248	101.4723	98.71178
3527.165	100.1898	101.3726	98.72395

3529.094	100.172	101.2252	98.71845
3531.022	100.1924	101.1842	98.7954
3532.951	100.2462	101.3229	98.88103
3534.879	100.277	101.3847	98.89493
3536.808	100.3113	101.3811	98.88904
3538.736	100.3502	101.472	98.8968
3540.665	100.3459	101.5529	98.93291
3542.593	100.3333	101.5012	98.89073
3544.521	100.3371	101.426	98.85561
3546.45	100.3098	101.3837	98.94019
3548.378	100.2459	101.2428	99.02212
3550.307	100.2178	100.9658	99.04703
3552.235	100.2946	100.8867	99.15139
3554.164	100.3712	101.2158	99.27094
3556.092	100.4156	101.4526	99.26074
3558.021	100.4241	101.5068	99.22089
3559.949	100.4429	101.5236	99.22895
3561.878	100.4505	101.5071	99.16686
3563.806	100.4269	101.4742	99.05831
3565.735	100.4213	101.471	99.05094
3567.663	100.3827	101.4301	99.23833
3569.592	100.3159	101.2264	99.40363
3571.52	100.3597	101.004	99.48933
3573.448	100.4544	101.1442	99.52544
3575.377	100.4721	101.3819	99.55718
3577.305	100.4589	101.3723	99.58704
3579.234	100.4846	101.2777	99.58522
3581.162	100.5129	101.3096	99.5686
3583.091	100.5189	101.3572	99.51044
3585.019	100.5569	101.401	99.40751

3586.948	100.5466	101.4739	99.49397
3588.876	100.4924	101.4888	99.67061
3590.805	100.4613	101.3078	99.69188
3592.733	100.4706	101.147	99.64622
3594.662	100.4917	101.1793	99.65685
3596.59	100.5058	101.1618	99.71774
3598.519	100.5167	101.1546	99.80313
3600.447	100.4802	101.1653	99.87767
3602.375	100.5119	101.0343	99.91291
3604.304	100.6405	101.0137	99.85544
3606.232	100.6971	101.2966	99.76576
3608.161	100.6005	101.4793	99.75478
3610.089	100.4125	101.3424	99.77287
3612.018	100.4008	100.9414	99.78129
3613.946	100.6118	100.8297	99.83686
3615.875	100.6824	101.2507	99.79324
3617.803	100.7321	101.4248	99.74722
3619.732	100.7802	101.4939	99.95764
3621.66	100.7516	101.5993	100.1234
3623.589	100.7284	101.6086	100.1006
3625.517	100.7112	101.609	99.91637
3627.446	100.6184	101.5871	99.86372
3629.374	100.4477	101.4241	100.1084
3631.302	100.3138	100.9465	100.2406
3633.231	100.3881	100.498	100.3244
3635.159	100.6073	100.697	100.416
3637.088	100.6937	101.2215	100.44
3639.016	100.663	101.4208	100.4448
3640.945	100.7251	101.3991	100.4448
3642.873	100.7985	101.5617	100.3843

3644.802	100.8538	101.7276	100.1527
3646.73	100.8675	101.7847	99.93843
3648.659	100.8167	101.7534	100.0716
3650.587	100.7092	101.6099	100.3619
3652.516	100.6255	101.2719	100.4827
3654.444	100.6385	101.0068	100.4592
3656.373	100.5851	101.0841	100.5477
3658.301	100.4702	100.9304	100.6724
3660.229	100.5983	100.6635	100.7191
3662.158	100.7333	101.0642	100.7222
3664.086	100.7999	101.4119	100.6853
3666.015	100.8627	101.6023	100.556
3667.943	100.8561	101.6866	100.4301
3669.872	100.7182	101.5855	100.4781
3671.8	100.5935	101.2526	100.4233
3673.729	100.6292	100.9496	100.2841
3675.657	100.7327	101.0816	100.5151
3677.586	100.7977	101.4045	100.7259
3679.514	100.8061	101.6126	100.8337
3681.443	100.8215	101.6555	100.9179
3683.371	100.8302	101.6484	100.8969
3685.3	100.8612	101.5036	100.7403
3687.228	100.9189	101.4355	100.5777
3689.156	100.8517	101.5752	100.6307
3691.085	100.7228	101.5426	100.7989
3693.013	100.6645	101.2732	100.9408
3694.942	100.6566	101.017	101.0175
3696.87	100.7665	101.077	101.0194
3698.799	100.8775	101.3822	100.9412
3700.727	100.854	101.5786	100.9071

3702.656	100.8346	101.5693	100.9852
3704.584	100.7715	101.4723	100.9902
3706.513	100.7201	101.3373	100.8616
3708.441	100.7724	101.281	100.6965
3710.37	100.8024	101.3024	100.7023
3712.298	100.7831	101.4122	100.8938
3714.227	100.7	101.4332	101.0312
3716.155	100.5735	101.1267	101.0393
3718.083	100.5196	100.7086	100.9678
3720.012	100.5908	100.6277	100.8785
3721.94	100.6957	100.9488	100.822
3723.869	100.7267	101.2691	100.8235
3725.797	100.6409	101.2701	100.9771
3727.726	100.5946	101.0125	101.0357
3729.654	100.6347	100.7729	100.8626
3731.583	100.4985	100.8396	100.5902
3733.511	100.4115	100.7311	100.5435
3735.44	100.6129	100.6622	100.7543
3737.368	100.7802	100.9686	100.9205
3739.297	100.7913	101.2862	100.9184
3741.225	100.7526	101.3162	100.8173
3743.154	100.7904	101.287	100.6648
3745.082	100.8676	101.4502	100.7001
3747.01	100.8468	101.5727	100.6882
3748.939	100.763	101.5083	100.6012
3750.867	100.7383	101.4581	100.7091
3752.796	100.7668	101.4631	100.8997
3754.724	100.8607	101.4429	100.9531
3756.653	100.8882	101.6284	100.9241
3758.581	100.8764	101.7934	100.9877

3760.51	100.8069	101.7841	101.0566
3762.438	100.7626	101.6135	101.0408
3764.367	100.8435	101.5199	101.0215
3766.295	100.8983	101.5981	101.0393
3768.224	100.8797	101.5803	101.0271
3770.152	100.8614	101.5833	101.0966
3772.081	100.8466	101.6843	101.1738
3774.009	100.863	101.7346	101.2019
3775.938	100.898	101.7586	101.1423
3777.866	100.8182	101.7253	101.0652
3779.794	100.714	101.531	101.0704
3781.723	100.7123	101.335	101.0753
3783.651	100.7172	101.2264	101.1098
3785.58	100.7192	101.0426	101.1597
3787.508	100.7642	101.1495	101.1655
3789.437	100.7399	101.3496	101.1699
3791.365	100.818	101.3517	101.1354
3793.294	100.8833	101.5255	101.0397
3795.222	100.8576	101.7078	100.9569
3797.151	100.7626	101.6067	100.9073
3799.079	100.6981	101.3401	100.7977
3801.008	100.7295	101.1842	100.8514
3802.936	100.6301	101.1217	100.9837
3804.865	100.6534	100.9223	100.9776
3806.793	100.8179	101.1427	101.0466
3808.721	100.8198	101.4765	101.156
3810.65	100.7992	101.4908	101.0999
3812.578	100.8255	101.5446	100.934
3814.507	100.7875	101.5173	100.8428
3816.435	100.6706	101.3372	100.8421

3818.364	100.613	101.2779	100.7633
3820.292	100.6405	101.1874	100.8191
3822.221	100.6685	100.987	101.0163
3824.149	100.6998	100.9249	101.0247
3826.078	100.75	101.0895	101.0186
3828.006	100.8268	101.3354	101.0198
3829.935	100.8725	101.5543	100.961
3831.863	100.7654	101.6114	100.9544
3833.792	100.5475	101.4436	100.9026
3835.72	100.4471	101.0655	100.7828
3837.648	100.6183	100.6728	100.7654
3839.577	100.7773	100.9486	100.8557
3841.505	100.8102	101.4026	100.9599
3843.434	100.8296	101.6027	101.1085
3845.362	100.808	101.5109	101.1395
3847.291	100.7624	101.3042	101.0238
3849.219	100.7464	101.2409	100.8474
3851.148	100.6656	101.2989	100.657
3853.076	100.6023	101.165	100.7352
3855.005	100.6739	101.0506	100.9381
3856.933	100.7433	101.236	101.0838
3858.862	100.8036	101.3342	101.0741
3860.79	100.8162	101.527	100.9812
3862.719	100.7574	101.5315	100.9457
3864.647	100.7247	101.3187	100.9813
3866.575	100.7331	101.3398	100.9345
3868.504	100.7354	101.3507	100.8439
3870.432	100.749	101.3412	100.9261
3872.361	100.7504	101.5255	100.9776
3874.289	100.7606	101.5116	101.08

3876.218	100.8339	101.4057	101.084
3878.146	100.8381	101.5349	100.9565
3880.075	100.7914	101.6523	100.942
3882.003	100.6945	101.5168	100.9612
3883.932	100.6602	101.2665	100.9632
3885.86	100.6457	101.1403	101.0484
3887.789	100.6862	101.1302	101.0551
3889.717	100.8697	101.3336	101.0052
3891.646	100.9537	101.6314	101.0777
3893.574	100.9286	101.7807	101.111
3895.502	100.8889	101.8198	101.0148
3897.431	100.8559	101.8102	100.8624
3899.359	100.8232	101.7025	100.8052
3901.288	100.854	101.5285	100.7675
3903.216	100.8794	101.504	100.8851
3905.145	100.869	101.5777	101.0934
3907.073	100.8754	101.6083	101.2081
3909.002	100.8611	101.5689	101.2253
3910.93	100.8359	101.638	101.2493
3912.859	100.833	101.6797	101.2372
3914.787	100.8116	101.6035	101.1346
3916.716	100.8277	101.5844	101.0894
3918.644	100.8687	101.6892	101.1155
3920.573	100.8512	101.8169	101.0978
3922.501	100.808	101.8584	101.0789
3924.429	100.828	101.7611	101.129
3926.358	100.8732	101.6266	101.1322
3928.286	100.8733	101.6494	101.0694
3930.215	100.8619	101.6761	101.0898
3932.143	100.8832	101.6107	101.1765

3934.072	100.8981	101.5943	101.2654
3936	100.8808	101.6952	101.28
3937.929	100.9209	101.7537	101.2107
3939.857	100.9874	101.757	101.1297
3941.786	100.9765	101.7494	101.1825
3943.714	100.8977	101.7502	101.218
3945.643	100.8753	101.7508	101.1223
3947.571	100.8879	101.7579	101.1032
3949.5	100.8981	101.7938	101.1794
3951.428	100.9318	101.827	101.1858
3953.356	100.9127	101.8634	101.1721
3955.285	100.8616	101.8603	101.196
3957.213	100.8467	101.8592	101.1931
3959.142	100.862	101.8634	101.1664
3961.07	100.8636	101.8531	101.1939
3962.999	100.9116	101.8048	101.2457
3964.927	100.9106	101.7883	101.2975
3966.856	100.9056	101.8143	101.3164
3968.784	100.8859	101.8631	101.2844
3970.713	100.8771	101.8924	101.2792
3972.641	100.8886	101.8945	101.2973
3974.57	100.9229	101.8101	101.2803
3976.498	100.9219	101.7719	101.2393
3978.427	100.8771	101.7865	101.2119
3980.355	100.8725	101.8083	101.205
3982.283	100.881	101.8287	101.2177
3984.212	100.9004	101.8538	101.2623

Figure 5

For the quantification of tail diameter change:

0.5 cm distal of the incision site

Week	Control	SD	Untreated	SD	SA	SD	L-SA	SD	D-SA	SD
1	6.856	0.06877	8.156	0.31575	8.175	0.2655	7.728	0.15205	8.068	0.13755
2	6.978	0.07918	9.146	0.63955	9.575	0.54274	8.14	0.3698	8.57	0.17393
3	7.056	0.06804	11.03	0.7505	9.005	0.42711	8.026	0.24674	8.216	0.13202
4	7.096	0.05459	10.756	0.8861	8.596	0.17397	7.558	0.15418	7.926	0.09607
5	7.128	0.04764	10.008	0.78509	8.455	0.22664	7.41	0.10149	7.714	0.19769
6	7.138	0.04207	9.66	0.69663	8.035	0.22591	7.298	0.05891	7.668	0.08167

And the corresponding changes in tail diameter after six weeks

Control	SD	Untreated	SD	SA	SD	L-SA	SD	D-SA	SD
9.98	4.207	48.8	6.9336	23.8	2.2591	12.4	5.891	18.2	8.167

2.0 cm distal of the incision site

Week	Control	SD	Untreated	SD	SA	SD	L-SA	SD	D-SA	SD
1	6.35	0.11195	7.076	0.12542	7.0275	0.22066	7.132	0.26621	7.222	0.22797
2	6.565	0.28172	8.056	0.70599	8.425	0.64469	7.618	0.36128	7.856	0.17757
3	6.635	0.34064	9.156	0.90611	8.08	0.62466	7.336	0.535	7.714	0.13353
4	6.6875	0.39803	9.058	0.67136	7.965	0.58341	7.104	0.32516	7.546	0.10968
5	6.7175	0.38973	8.346	0.76179	7.6325	0.45006	7.034	0.323	7.456	0.05595
6	6.7325	0.40227	8.064	0.55343	7.515	0.43852	6.982	0.30261	7.352	0.10569

And the corresponding changes in tail diameter after six weeks

Control	SD	Untreated	SD	SA	SD	L-SA	SD	D-SA	SD
5.03	1.0227	25.8	5.5343	17.2	2.2591	8.9	3.0261	14.6	1.0569

3.5 cm distal of the incision site

Week	Control	SD	Untreated	SD	SA	SD	L-SA	SD	D-SA	SD
1	6.175	0.09147	6.768	0.09311	7.025	0.09708	6.606	0.07956	6.576	0.24234

2	6.2	0.07789	7.43	0.30936	7.61	0.30735	6.898	0.26574	6.99	0.15281
3	6.3275	0.09743	7.854	0.37004	7.08	0.41142	6.69	0.29656	6.902	0.17079
4	6.38	0.08124	7.604	0.3286	7.11	0.39933	6.41	0.09834	6.622	0.12458
5	6.4	0.065	7.338	0.29525	6.92	0.41231	6.392	0.08319	6.53	0.07314
6	6.42	0.06608	7.186	0.30146	6.9025	0.4036	6.401	0.06723	6.56	0.10075

And the corresponding changes in tail diameter after six weeks

Control	SD	Untreated	SD	SA	SD	L-SA	SD	D-SA	SD
6.2	1.6608	18.8	3.0146	14.1	4.0362	5.9	0.6723	8.5	1.0075

Figure 6

For the statistical results of soft-tissue thickness:

Thickness	Control	Untreated	SA	L-SA	D-SA
	361.593	829.627	575.766	446.457	553.333
	390.152	841.817	524.295	505.326	604.346
	302.155	788.847	523.975	491.601	482.421
	393.065	701.121	539.901	429.608	582.365
	304.484	723.583	607.557	603.381	465.788

Thickness	Control SD	Untreated	SD	SA	SD	L-SA	SD	D-SA	SD
350.2898	44.61391	776.999	62.6941	554.2988	36.48403	495.2746	68.00143	537.6506	61.04944

For the statistical results of lymphatic vessel count:

Number	Control	Untreated	SA	L-SA	D-SA
	8	3	9	15	9
	5	4	12	12	11
	7	4	7	17	13
	6	5	10	12	9
	5	3	8	10	10

Number	Control	SD	Untreated	SD	SA	SD	L-SA	SD	D-SA	SD
	6.2	1.30384	3.8	0.83666	9.2	1.92354	13.2	2.77489	10.4	1.67332

For the statistical results of lymphatic vessel area:

area	Control	Untreated	SA	L-SA	D-SA
	501.73	3585.64	1390.03	749.351	845.639
	448.205	3160.683	922.902	826.369	1163.946
	1339.749	1755.515	1136.462	823.421	873.464
	553.633	1717.669	777.465	933.175	1205.657
	955.342	2997.405	1333.261	772.016	915.861

area	Control	SD	Untreated	SD	SA	SD	L-SA	SD	D-SA	SD
	759.7318	381.0426	2643.382	855.2828	1112.024	261.9767	820.8664	70.97682	1000.913	170.3577

Figure 7

For the Real-time PCR results of IL-1β:

Control	SD	Untreated	SD	SA	SD	L-SA	SD	D-SA	SD
1	0.09602	3.3899	0.35992	1.63452	0.08062	1.1402	0.05089	1.3436	0.34204

For the Real-time PCR results of TNF-α:

Control	SD	Untreated	SD	SA	SD	L-SA	SD	D-SA	SD
1	0.10468	3.29784	0.33481	1.02504	0.09488	0.59879	0.04268	1.0535	0.27596

For the Real-time PCR results of TGF-β:

Control	SD	Untreated	SD	SA	SD	L-SA	SD	D-SA	SD
1	0.08531	1.82465	0.06426	0.78279	0.03768	0.62527	0.0558	0.71502	0.34268

Supporting information

Figure S9

Wavelength LPFEG 0.12 D-SA 0.08 D-SA 0.04 D-SA (CD data) and the corresponding UV-vis data

400	2.10103	-0.14159	0.47199	0.46255	9.97993	9.40482	9.62079	9.33826
399.5	2.0586	-0.15624	0.32769	0.32114	9.97768	9.40326	9.61879	9.33653
399	2.02516	-0.15398	0.24902	0.24404	9.97594	9.40202	9.61707	9.33459
398.5	1.99951	-0.03149	0.30389	0.29782	9.97426	9.40086	9.61539	9.33348
398	2.00646	0.03097	0.37062	0.36321	9.97286	9.3999	9.61428	9.33228
397.5	2.00881	0.00777	0.3745	0.36701	9.97185	9.39937	9.61323	9.33159
397	2.05933	0.07898	0.28485	0.27915	9.97115	9.39876	9.6126	9.33087
396.5	2.10698	0.1049	0.44154	0.43271	9.97062	9.39896	9.61257	9.33068
396	2.17278	-0.05364	0.45716	0.44802	9.97047	9.39903	9.61232	9.33086
395.5	2.23638	-0.00458	0.45689	0.44775	9.97054	9.39914	9.61245	9.3309
395	2.28185	0.01083	0.51744	0.50709	9.97098	9.39998	9.61299	9.33138
394.5	2.28303	-0.02537	0.46171	0.45247	9.97172	9.40127	9.61367	9.33226
394	2.28146	0.07727	0.36784	0.36048	9.97298	9.4027	9.61474	9.3337
393.5	2.25636	0.14529	0.29957	0.29358	9.97457	9.40464	9.61626	9.3354
393	2.23836	0.11817	0.32641	0.31988	9.97678	9.40669	9.6181	9.3371
392.5	2.2346	0.07615	0.34598	0.33906	9.97877	9.40878	9.62022	9.33887
392	2.25476	0.05016	0.42737	0.41882	9.98057	9.4107	9.62196	9.34035
391.5	2.27475	0.02875	0.52582	0.51531	9.98239	9.41254	9.62359	9.34188
391	2.28364	-0.05926	0.49837	0.4884	9.98411	9.41413	9.62519	9.34321
390.5	2.28938	0.00408	0.42785	0.41929	9.98529	9.41537	9.62625	9.34422
390	2.27243	-0.07122	0.40091	0.39289	9.98615	9.41636	9.62723	9.34496
389.5	2.25591	-0.12488	0.30437	0.29828	9.98672	9.41739	9.62804	9.34559
389	2.23754	-0.12809	0.21407	0.20979	9.9871	9.41805	9.6286	9.34592
388.5	2.16867	-0.19884	0.23582	0.23111	9.98736	9.41872	9.6287	9.34618
388	2.07912	-0.25847	0.03961	0.03882	9.98734	9.41883	9.62875	9.34611
387.5	1.98491	-0.18782	-0.01015	-0.00994	9.98736	9.41929	9.62884	9.34593

387	1.91801	-0.14557	-0.01887	-0.01849	9.98721	9.41966	9.62847	9.3457
386.5	1.83284	-0.03115	-0.0321	-0.03145	9.98682	9.4197	9.62844	9.34562
386	1.73491	0.07801	0.01669	0.01636	9.98649	9.41953	9.62843	9.34518
385.5	1.63173	0.1274	0.05959	0.0584	9.98572	9.41919	9.62819	9.34466
385	1.48168	0.07414	0.14533	0.14242	9.98472	9.41873	9.62747	9.34377
384.5	1.33638	0.10121	0.11446	0.11217	9.98362	9.41808	9.62653	9.34315
384	1.18123	0.09397	0.20138	0.19735	9.98239	9.41761	9.62559	9.34207
383.5	1.01572	0.20657	0.13525	0.13254	9.9814	9.41691	9.62508	9.3413
383	0.83167	0.28475	0.07896	0.07738	9.98014	9.41619	9.62427	9.34035
382.5	0.66731	0.38964	0.2928	0.28694	9.97897	9.41536	9.62304	9.33924
382	0.50261	0.38567	0.23555	0.23084	9.97764	9.41492	9.62202	9.33827
381.5	0.31972	0.45665	0.22291	0.21845	9.97673	9.41437	9.62119	9.33721
381	0.1386	0.56835	0.33207	0.32543	9.9757	9.41376	9.62042	9.33653
380.5	-0.00879	0.56769	0.31234	0.30609	9.97506	9.41343	9.61978	9.33575
380	-0.15036	0.61179	0.37172	0.36429	9.97435	9.41331	9.61914	9.33523
379.5	-0.29825	0.64099	0.44212	0.43328	9.97364	9.41304	9.61862	9.33479
379	-0.39787	0.53072	0.41146	0.40323	9.97287	9.41271	9.61817	9.33436
378.5	-0.48088	0.51206	0.36239	0.35514	9.97228	9.41237	9.61755	9.33396
378	-0.55803	0.50181	0.45041	0.4414	9.9717	9.41217	9.61733	9.33334
377.5	-0.59788	0.44497	0.47325	0.46379	9.97148	9.41243	9.61705	9.33262
377	-0.62504	0.35529	0.49539	0.48548	9.97097	9.41248	9.61689	9.33237
376.5	-0.61119	0.24321	0.52222	0.51177	9.97071	9.41238	9.61649	9.33199
376	-0.56677	0.29303	0.40099	0.39297	9.97019	9.41253	9.6162	9.33153
375.5	-0.4838	0.19616	0.42414	0.41565	9.96961	9.41232	9.61581	9.33134
375	-0.42067	0.10462	0.40052	0.39251	9.96916	9.41234	9.6154	9.33081
374.5	-0.31214	0.14515	0.30019	0.29419	9.96875	9.41232	9.61512	9.33029
374	-0.20915	0.17129	0.21028	0.20607	9.96826	9.41238	9.61496	9.33033
373.5	-0.08814	0.15942	0.141	0.13818	9.96759	9.41212	9.61465	9.3302
373	0.04547	0.15706	0.2762	0.27068	9.96716	9.41203	9.61437	9.32971
372.5	0.18756	0.10306	0.25004	0.24504	9.96687	9.41196	9.61416	9.32905

372	0.33743	0.02231	0.23139	0.22677	9.96588	9.41178	9.61392	9.32866
371.5	0.49979	0.1225	0.21235	0.2081	9.96534	9.41172	9.61342	9.32819
371	0.69047	0.166	0.25965	0.25446	9.96487	9.41164	9.61322	9.32734
370.5	0.84986	0.07724	0.32458	0.31809	9.96455	9.41163	9.6129	9.32694
370	1.01791	0.11032	0.3313	0.32468	9.96425	9.41171	9.61275	9.3266
369.5	1.20846	-0.02268	0.23432	0.22963	9.96385	9.41192	9.61239	9.32646
369	1.40371	-0.06208	0.11547	0.11316	9.96321	9.41194	9.61181	9.32631
368.5	1.60597	0.08545	0.04964	0.04865	9.9631	9.41203	9.61154	9.32613
368	1.80793	0.18053	0.07431	0.07282	9.96305	9.41226	9.61142	9.32623
367.5	2.00524	0.2061	0.05747	0.05632	9.96307	9.41225	9.6113	9.32642
367	2.20116	0.13848	0.14441	0.14152	9.96307	9.4126	9.61142	9.32642
366.5	2.39633	0.06115	0.17392	0.17045	9.96328	9.41299	9.61126	9.32647
366	2.59941	-0.02541	0.25311	0.24804	9.96292	9.41344	9.61149	9.32665
365.5	2.80326	-0.03305	0.25423	0.24915	9.96286	9.4137	9.61162	9.32664
365	2.97564	-0.05752	0.29265	0.28679	9.96306	9.41416	9.61184	9.32644
364.5	3.13566	-0.074	0.26221	0.25697	9.96301	9.41444	9.61195	9.32653
364	3.29407	0.03839	0.2586	0.25343	9.96308	9.41515	9.61204	9.32697
363.5	3.44982	0.02074	0.26953	0.26414	9.96338	9.41579	9.61243	9.32721
363	3.60927	-0.09664	0.20858	0.20441	9.96371	9.41627	9.61279	9.32742
362.5	3.7507	-0.07034	0.17127	0.16784	9.964	9.41676	9.6132	9.32788
362	3.86746	0.11218	0.20488	0.20078	9.96404	9.41736	9.61354	9.32813
361.5	3.94384	0.18665	0.24656	0.24162	9.96413	9.41795	9.61404	9.32833
361	4.00444	0.34735	0.40012	0.39212	9.96421	9.4185	9.61462	9.32868
360.5	4.05331	0.27223	0.38503	0.37733	9.96429	9.41909	9.61498	9.32911
360	4.08956	0.25432	0.35128	0.34425	9.96436	9.41964	9.61517	9.32934
359.5	4.13036	0.23238	0.33037	0.32376	9.96428	9.4204	9.6155	9.32913
359	4.16194	0.28505	0.32945	0.32286	9.96431	9.42111	9.61577	9.3293
358.5	4.18438	0.3113	0.20416	0.20008	9.96451	9.42185	9.61596	9.3294
358	4.17775	0.34233	0.15248	0.14943	9.96452	9.42257	9.61622	9.32977
357.5	4.15502	0.55119	0.20954	0.20535	9.96464	9.42328	9.61658	9.32997

357	4.10953	0.59917	0.26168	0.25645	9.96502	9.42403	9.61668	9.33014
356.5	4.06195	0.61917	0.30436	0.29827	9.965	9.42496	9.61746	9.33062
356	4.04368	0.62448	0.38978	0.38199	9.96515	9.42571	9.61772	9.33084
355.5	3.99812	0.62596	0.47162	0.46219	9.96535	9.42614	9.61791	9.33095
355	3.92881	0.79044	0.39683	0.3889	9.96551	9.42715	9.61826	9.33105
354.5	3.8481	0.89913	0.55031	0.5393	9.96589	9.42792	9.61863	9.33134
354	3.80799	0.86806	0.46653	0.4572	9.96617	9.42865	9.61889	9.33179
353.5	3.77889	0.82003	0.54515	0.53425	9.96645	9.42935	9.61933	9.33194
353	3.7693	0.75324	0.5415	0.53067	9.96695	9.43021	9.62	9.33213
352.5	3.79522	0.84171	0.53481	0.52411	9.96753	9.431	9.62026	9.33238
352	3.8071	1.02753	0.60704	0.5949	9.96779	9.43179	9.62074	9.33261
351.5	3.79817	1.09247	0.73315	0.71849	9.96818	9.43277	9.62141	9.333
351	3.79053	0.97042	0.67271	0.65925	9.96813	9.43352	9.62192	9.33319
350.5	3.76918	1.05152	0.81251	0.79625	9.96856	9.43423	9.62227	9.33367
350	3.72541	1.09174	0.81458	0.79829	9.96868	9.4348	9.62261	9.33357
349.5	3.70538	1.12924	0.94284	0.92398	9.96883	9.43535	9.62304	9.33361
349	3.67542	1.00616	0.96625	0.94693	9.96913	9.43596	9.62359	9.33387
348.5	3.64141	1.00159	0.93798	0.91922	9.96897	9.4367	9.62387	9.33413
348	3.6101	1.10632	0.91233	0.89408	9.96921	9.43693	9.62376	9.33387
347.5	3.58579	1.0387	0.82089	0.80447	9.9692	9.43763	9.62419	9.33403
347	3.55847	1.07833	0.8664	0.84908	9.96915	9.43801	9.62444	9.33407
346.5	3.55411	1.08789	0.89816	0.8802	9.96919	9.43855	9.62463	9.33403
346	3.53655	1.18761	0.83977	0.82297	9.96899	9.43919	9.62491	9.33397
345.5	3.5167	1.18582	0.92564	0.90713	9.96864	9.43967	9.62521	9.33392
345	3.51749	1.29347	0.80838	0.79221	9.96857	9.44022	9.62531	9.33397
344.5	3.53664	1.3308	0.77001	0.75461	9.96829	9.44063	9.62539	9.33399
344	3.54139	1.26248	0.70334	0.68927	9.96825	9.44102	9.62538	9.33375
343.5	3.58801	1.18724	0.67842	0.66485	9.96827	9.44148	9.62542	9.33358
343	3.66142	1.27851	0.67602	0.6625	9.96811	9.44168	9.62544	9.33358
342.5	3.73602	1.37261	0.69124	0.67741	9.96806	9.44229	9.62563	9.33343

342	3.82992	1.27418	0.71595	0.70163	9.96786	9.44285	9.62602	9.33344
341.5	3.95297	1.25496	0.67189	0.65845	9.96783	9.44355	9.62631	9.3337
341	4.0835	1.31165	0.61184	0.5996	9.96783	9.44419	9.62631	9.33387
340.5	4.22338	1.33304	0.67455	0.66106	9.96746	9.44483	9.62639	9.33391
340	4.36063	1.3021	0.63152	0.61889	9.96748	9.44532	9.62651	9.33377
339.5	4.52163	1.1974	0.59023	0.57842	9.96755	9.44638	9.62693	9.33386
339	4.67953	1.19548	0.64117	0.62834	9.96768	9.44684	9.62708	9.33397
338.5	4.84604	1.20731	0.55982	0.54862	9.96784	9.44774	9.62751	9.33397
338	4.99441	1.15626	0.56032	0.54911	9.96756	9.44822	9.62755	9.33424
337.5	5.1392	1.08778	0.55696	0.54582	9.9675	9.44894	9.62782	9.33401
337	5.32023	1.16734	0.46814	0.45877	9.96784	9.44964	9.62801	9.33412
336.5	5.49617	0.98755	0.53025	0.51965	9.96785	9.45028	9.62832	9.3342
336	5.66693	0.98795	0.64184	0.62901	9.968	9.45089	9.62878	9.3344
335.5	5.84311	1.06198	0.67142	0.65799	9.96787	9.45186	9.62925	9.33476
335	6.01904	1.10986	0.71732	0.70297	9.96793	9.45268	9.62953	9.33509
334.5	6.16526	1.0227	0.79995	0.78395	9.96821	9.45375	9.6298	9.33515
334	6.29849	1.03165	0.75764	0.74249	9.96841	9.45449	9.63011	9.33532
333.5	6.42183	1.12823	0.69628	0.68235	9.96841	9.45528	9.63038	9.33555
333	6.52619	1.144	0.70017	0.68617	9.96864	9.45614	9.63093	9.33557
332.5	6.60936	1.14923	0.78448	0.76879	9.96894	9.45697	9.6314	9.33565
332	6.70077	1.14294	0.81007	0.79387	9.96919	9.45797	9.63172	9.33581
331.5	6.8029	1.078	0.7742	0.75872	9.96947	9.45884	9.63211	9.33619
331	6.88445	1.21956	0.78501	0.76931	9.96971	9.45991	9.63258	9.33632
330.5	6.94084	1.2323	0.80455	0.78846	9.96996	9.46101	9.63303	9.33662
330	7.03902	1.34432	0.79087	0.77506	9.97008	9.46186	9.63355	9.33672
329.5	7.15736	1.27093	0.86193	0.8447	9.97072	9.46292	9.63398	9.3371
329	7.29371	1.36292	1.01031	0.9901	9.97096	9.46373	9.63435	9.33749
328.5	7.40997	1.35864	0.98614	0.96641	9.97117	9.46491	9.63475	9.33765
328	7.50304	1.54657	0.89369	0.87581	9.97117	9.46573	9.63501	9.3377
327.5	7.58441	1.50484	0.92131	0.90289	9.97111	9.46662	9.63515	9.33775

327	7.6329	1.63332	0.97521	0.9557	9.97108	9.46736	9.63557	9.33776
326.5	7.67331	1.6062	0.90994	0.89174	9.97083	9.46795	9.6357	9.3372
326	7.71962	1.7282	0.82926	0.81267	9.9706	9.46856	9.63602	9.33718
325.5	7.77021	1.854	0.84174	0.82491	9.97087	9.46954	9.63634	9.33753
325	7.81626	2.04441	0.93265	0.914	9.97106	9.47041	9.63675	9.33762
324.5	7.86979	2.12947	0.77294	0.75748	9.97113	9.47146	9.63722	9.3379
324	7.934	2.04327	0.76303	0.74777	9.97165	9.47242	9.63758	9.33813
323.5	8.02098	2.10889	0.74148	0.72665	9.9717	9.47355	9.63818	9.33836
323	8.12309	2.18144	0.78019	0.76459	9.97193	9.47442	9.63863	9.33853
322.5	8.22978	2.16429	0.89484	0.87694	9.97182	9.47531	9.63884	9.33873
322	8.33677	2.20959	0.96266	0.9434	9.97197	9.47626	9.63939	9.33881
321.5	8.44004	2.27222	1.02071	1.0003	9.97224	9.47737	9.64005	9.33907
321	8.52669	2.24315	0.87094	0.85352	9.97226	9.47849	9.64062	9.3394
320.5	8.5746	2.2339	0.78253	0.76688	9.97261	9.47988	9.64108	9.33965
320	8.61113	2.3452	1.02779	1.00723	9.97292	9.48091	9.64174	9.33998
319.5	8.67505	2.27406	1.09844	1.07647	9.97327	9.48211	9.64243	9.34034
319	8.73995	2.16175	1.01306	0.9928	9.9738	9.48348	9.64297	9.34074
318.5	8.8218	2.23974	0.99651	0.97658	9.97407	9.48457	9.64359	9.34126
318	8.90937	2.25225	1.03876	1.01798	9.97437	9.4857	9.64429	9.34169
317.5	9.0019	2.17385	1.15296	1.1299	9.97456	9.48694	9.6449	9.342
317	9.10103	2.2085	1.1228	1.10034	9.97463	9.48861	9.6457	9.34192
316.5	9.21484	2.04945	1.19474	1.17085	9.97476	9.48971	9.64594	9.34208
316	9.30158	2.10136	1.26773	1.24238	9.97461	9.49082	9.64638	9.34209
315.5	9.37342	2.09925	1.22473	1.20024	9.97449	9.49215	9.64695	9.34226
315	9.45788	2.03146	1.24167	1.21684	9.97479	9.49335	9.64791	9.34263
314.5	9.53762	2.0516	1.32132	1.29489	9.97513	9.4947	9.64864	9.34276
314	9.60601	2.05339	1.22369	1.19922	9.9756	9.49603	9.64942	9.34337
313.5	9.72002	2.07091	1.16939	1.146	9.9759	9.49737	9.65003	9.34391
313	9.81599	2.05342	1.10036	1.07835	9.97612	9.49892	9.65085	9.3443
312.5	9.94733	1.9636	1.14086	1.11804	9.97655	9.50047	9.65182	9.34476

312	10.0942	2.15117	1.2026	1.17855	9.97687	9.50194	9.65268	9.34513
311.5	10.20353	2.03614	1.07095	1.04953	9.9772	9.50355	9.6534	9.34558
311	10.32477	2.04231	1.02817	1.00761	9.97758	9.50491	9.65428	9.34593
310.5	10.45603	2.1391	1.02112	1.0007	9.97784	9.5065	9.65536	9.34649
310	10.5797	2.0853	0.9824	0.96275	9.97849	9.50822	9.65653	9.34689
309.5	10.69892	2.08006	0.94659	0.92766	9.97897	9.5099	9.65755	9.34758
309	10.84293	2.04956	0.88585	0.86813	9.97969	9.51201	9.65872	9.3484
308.5	10.99601	2.01199	0.89621	0.87829	9.98045	9.51401	9.65999	9.34926
308	11.15196	2.08233	0.96574	0.94642	9.98098	9.51605	9.66136	9.35033
307.5	11.28943	2.10921	1.00866	0.98849	9.98172	9.51784	9.66248	9.35104
307	11.44112	2.23073	1.02442	1.00393	9.98206	9.51958	9.66354	9.35172
306.5	11.58114	2.31572	1.0038	0.98372	9.9827	9.52186	9.66444	9.35248
306	11.7119	2.45663	0.9994	0.97941	9.98341	9.52453	9.66594	9.3533
305.5	11.84812	2.44001	0.94099	0.92217	9.98399	9.52651	9.66707	9.35392
305	11.94108	2.60213	1.02634	1.00581	9.98437	9.52857	9.66845	9.35443
304.5	12.02592	2.54578	1.12246	1.10001	9.98493	9.53062	9.66963	9.35513
304	12.09313	2.71501	1.15037	1.12736	9.98504	9.53319	9.67097	9.35604
303.5	12.13418	2.79984	1.23032	1.20571	9.98585	9.53583	9.67262	9.35691
303	12.16425	2.98893	1.28457	1.25888	9.98637	9.53873	9.67437	9.35785
302.5	12.16975	3.08186	1.40177	1.37373	9.98702	9.54201	9.67606	9.35889
302	12.16018	3.10418	1.46526	1.43595	9.98748	9.54556	9.67776	9.3599
301.5	12.14605	3.17008	1.48912	1.45934	9.98806	9.54955	9.67987	9.36103
301	12.12921	3.19957	1.54625	1.51532	9.98927	9.55373	9.68215	9.36223
300.5	12.08731	3.43266	1.60831	1.57614	9.98995	9.55815	9.6849	9.36367
300	11.99456	3.57313	1.56008	1.52888	9.9909	9.56284	9.6875	9.36516
299.5	11.92023	3.49844	1.59273	1.56088	9.99207	9.56844	9.69051	9.36687
299	11.8599	3.37579	1.58847	1.5567	9.99335	9.57425	9.69393	9.36867
298.5	11.77089	3.33221	1.59714	1.5652	9.9946	9.58051	9.69789	9.37059
298	11.64398	3.15714	1.58984	1.55804	9.9957	9.58753	9.70194	9.37286
297.5	11.47128	3.04875	1.46921	1.43983	9.9971	9.59475	9.70595	9.37517

297	11.2729	2.86133	1.46138	1.43215	9.99835	9.60236	9.71025	9.37733
296.5	11.10452	2.66256	1.3113	1.28507	9.9997	9.61052	9.71502	9.37996
296	10.89091	2.31424	1.03872	1.01795	10.00139	9.61998	9.72032	9.38262
295.5	10.68244	1.82953	0.84646	0.82953	10.00271	9.62883	9.72512	9.38501
295	10.49782	1.38311	0.42312	0.41465	10.0036	9.63717	9.72946	9.38724
294.5	10.3258	0.89769	-0.01239	-0.01214	10.00493	9.64608	9.73439	9.38972
294	10.1215	0.26339	-0.45679	-0.44766	10.0068	9.6554	9.7399	9.39276
293.5	9.90094	-0.21323	-0.70197	-0.68793	10.00922	9.66556	9.74604	9.3961
293	9.69796	-0.75573	-1.20615	-1.18203	10.01137	9.67575	9.75206	9.39953
292.5	9.45229	-1.53579	-1.67627	-1.64274	10.01317	9.68571	9.75809	9.4028
292	9.20948	-2.34015	-2.08845	-2.04668	10.01522	9.69511	9.76333	9.40579
291.5	8.95521	-3.13442	-2.56178	-2.51054	10.017	9.70484	9.76896	9.40911
291	8.69212	-3.90958	-3.03022	-2.96962	10.01856	9.71381	9.77427	9.41194
290.5	8.42983	-4.77114	-3.62695	-3.55441	10.02057	9.72368	9.78005	9.41507
290	8.19846	-5.66125	-4.11992	-4.03752	10.02241	9.73278	9.78554	9.41809
289.5	7.96404	-6.30144	-4.76482	-4.66952	10.02412	9.74164	9.79084	9.42085
289	7.74047	-7.04601	-5.25045	-5.14544	10.02591	9.74984	9.79586	9.4235
288.5	7.55042	-7.69777	-5.73768	-5.62293	10.02785	9.75774	9.80042	9.42646
288	7.32218	-8.4691	-6.20585	-6.08173	10.02972	9.76563	9.80533	9.42938
287.5	7.11096	-9.0009	-6.72426	-6.58977	10.03161	9.7734	9.81002	9.43214
287	6.89199	-9.71995	-7.20029	-7.05628	10.03386	9.78136	9.81519	9.43535
286.5	6.69621	-10.4195	-7.61733	-7.46498	10.0365	9.78983	9.82078	9.43869
286	6.5222	-10.8938	-7.98697	-7.82723	10.03852	9.79741	9.82574	9.44164
285.5	6.38811	-11.4276	-8.39504	-8.22714	10.04008	9.80399	9.82986	9.44406
285	6.25622	-11.8063	-8.79285	-8.61699	10.04164	9.81048	9.83423	9.44637
284.5	6.12548	-12.3031	-9.19223	-9.00839	10.04333	9.81736	9.83836	9.44893
284	6.00653	-12.6785	-9.5726	-9.38115	10.0454	9.82462	9.84311	9.45156
283.5	5.839	-13.1651	-10.0696	-9.86821	10.0474	9.83212	9.84813	9.45418
283	5.66358	-13.7322	-10.4022	-10.19416	10.04942	9.83938	9.85291	9.45669
282.5	5.4704	-13.9348	-10.896	-10.67808	10.05139	9.84627	9.8576	9.45926

282	5.27453	-14.4084	-11.3157	-11.08939	10.05343	9.85375	9.86256	9.46197
281.5	5.04599	-14.9135	-11.8599	-11.6227	10.05572	9.86121	9.86733	9.46487
281	4.82228	-15.2457	-12.096	-11.85408	10.05791	9.86871	9.87247	9.46781
280.5	4.56475	-15.543	-12.6294	-12.37681	10.06015	9.87678	9.87762	9.47095
280	4.24995	-15.9834	-13.1019	-12.83986	10.06256	9.88442	9.8828	9.47363
279.5	3.93378	-16.5063	-13.3887	-13.12093	10.06478	9.89233	9.88804	9.47652
279	3.57423	-17.0349	-13.9388	-13.66002	10.06726	9.90044	9.89378	9.47979
278.5	3.1873	-17.2607	-14.5021	-14.21206	10.07004	9.90924	9.89958	9.4832
278	2.76418	-17.771	-14.9056	-14.60749	10.07292	9.91789	9.90551	9.48646
277.5	2.33805	-18.2064	-15.2363	-14.93157	10.07574	9.92651	9.91132	9.48961
277	1.91681	-18.5609	-15.7507	-15.43569	10.07849	9.93583	9.9175	9.49331
276.5	1.46456	-18.9092	-16.1206	-15.79819	10.08153	9.94503	9.92377	9.49685
276	1.00579	-19.4633	-16.526	-16.19548	10.08443	9.95397	9.92998	9.50055
275.5	0.54777	-19.6213	-16.9516	-16.61257	10.08746	9.96306	9.93612	9.50406
275	0.15024	-19.9046	-17.1292	-16.78662	10.09057	9.97256	9.9428	9.50802
274.5	-0.25424	-19.9148	-17.2319	-16.88726	10.09404	9.98241	9.94979	9.5122
274	-0.67112	-20.0474	-17.4129	-17.06464	10.09766	9.99265	9.95691	9.51647
273.5	-1.12309	-20.1966	-17.5025	-17.15245	10.1016	10.003	9.9644	9.52083
273	-1.54994	-20.1959	-17.6965	-17.34257	10.10551	10.0137	9.9722	9.52534
272.5	-1.97326	-20.2146	-17.8512	-17.49418	10.10961	10.0241	9.97979	9.5299
272	-2.33123	-20.3465	-18.0212	-17.66078	10.1138	10.035	9.98785	9.53483
271.5	-2.65363	-20.1843	-18.2482	-17.88324	10.11862	10.0461	9.99612	9.53983
271	-2.97433	-20.0218	-18.4053	-18.03719	10.12361	10.0573	10.00455	9.54508
270.5	-3.26762	-19.6783	-18.3346	-17.96791	10.12841	10.0684	10.01297	9.55014
270	-3.57199	-19.3199	-18.3698	-18.0024	10.13373	10.0799	10.02171	9.55556
269.5	-3.87644	-18.986	-18.2073	-17.84315	10.13914	10.0914	10.0308	9.56125
269	-4.17662	-18.7213	-18.4109	-18.04268	10.14438	10.1031	10.03982	9.56698
268.5	-4.42633	-18.7575	-18.07	-17.7086	10.15012	10.1147	10.04932	9.57277
268	-4.64904	-18.4197	-17.7764	-17.42087	10.15576	10.1257	10.05822	9.57829
267.5	-4.87255	-17.9231	-17.7567	-17.40157	10.16152	10.1367	10.06716	9.58381

267	-5.04849	-17.4829	-17.4089	-17.06072	10.16754	10.1482	10.07668	9.58985
266.5	-5.20639	-16.9857	-17.2625	-16.91725	10.17402	10.1597	10.08611	9.59624
266	-5.32667	-16.5233	-17.1593	-16.81611	10.18031	10.1709	10.09563	9.60246
265.5	-5.41228	-15.8652	-16.8788	-16.54122	10.18639	10.1813	10.1046	9.60837
265	-5.43748	-15.225	-16.5192	-16.18882	10.19301	10.1922	10.11398	9.61466
264.5	-5.4126	-14.7147	-16.0884	-15.76663	10.19961	10.2032	10.12326	9.62099
264	-5.37474	-14.1067	-15.6241	-15.31162	10.20652	10.2141	10.13259	9.62707
263.5	-5.29351	-13.7823	-15.1386	-14.83583	10.21329	10.2249	10.14217	9.63352
263	-5.16307	-13.6003	-14.5798	-14.2882	10.21963	10.2349	10.15123	9.63962
262.5	-4.98666	-13.0882	-13.952	-13.67296	10.22644	10.2453	10.16082	9.64611
262	-4.72962	-12.5811	-13.604	-13.33192	10.23344	10.2559	10.17011	9.65266
261.5	-4.38507	-12.147	-12.996	-12.73608	10.23969	10.2652	10.1787	9.6585
261	-3.99897	-11.6909	-12.628	-12.37544	10.24652	10.2749	10.18754	9.66488
260.5	-3.57825	-11.1805	-12.0227	-11.78225	10.25388	10.2852	10.19685	9.67153
260	-3.13425	-10.4339	-11.5266	-11.29607	10.26064	10.2948	10.20542	9.67779
259.5	-2.66206	-9.76911	-10.9959	-10.77598	10.26728	10.304	10.21403	9.68376
259	-2.12391	-9.11941	-10.2698	-10.0644	10.27397	10.3132	10.22227	9.69018
258.5	-1.58099	-8.60405	-9.65823	-9.46507	10.28078	10.3222	10.23056	9.696
258	-0.981	-8.187	-8.97117	-8.79175	10.28756	10.3311	10.23914	9.70196
257.5	-0.34353	-7.44098	-8.17552	-8.01201	10.29493	10.341	10.24823	9.70855
257	0.32609	-6.92882	-7.82251	-7.66606	10.30203	10.3504	10.25679	9.71515
256.5	1.00064	-6.5178	-7.13458	-6.99189	10.30803	10.3584	10.26417	9.72036
256	1.69608	-5.79147	-6.77208	-6.63664	10.31336	10.3656	10.27098	9.72563
255.5	2.38392	-4.94443	-5.89935	-5.78136	10.31926	10.3734	10.27812	9.73077
255	3.08981	-4.5991	-5.2746	-5.16911	10.32472	10.3804	10.28461	9.7356
254.5	3.87766	-3.59023	-4.61545	-4.52314	10.33017	10.3872	10.29073	9.74043
254	4.69587	-2.8311	-3.97998	-3.90038	10.33474	10.3935	10.29649	9.74456
253.5	5.54756	-2.18447	-3.2571	-3.19196	10.3394	10.3993	10.30196	9.74873
253	6.40623	-1.74569	-2.50734	-2.45719	10.34373	10.4053	10.30742	9.75287
252.5	7.34972	-1.11169	-1.64969	-1.6167	10.34868	10.4112	10.31318	9.75705

252	8.30559	-0.17881	-0.83922	-0.82243	10.35315	10.417	10.31854	9.76125
251.5	9.26852	0.38327	-0.0415	-0.04067	10.35755	10.4224	10.32359	9.76537
251	10.25445	0.87107	0.50116	0.49114	10.36115	10.4267	10.32781	9.76854
250.5	11.26218	1.60002	1.0622	1.04096	10.36456	10.431	10.33183	9.77173
250	12.30923	2.09132	1.47778	1.44822	10.36772	10.435	10.33543	9.7749
249.5	13.33864	3.09002	2.25575	2.21063	10.37121	10.4393	10.33914	9.7779
249	14.40789	3.34923	2.95804	2.89888	10.37416	10.4428	10.34254	9.78054
248.5	15.49394	4.15469	3.76145	3.68622	10.37715	10.4461	10.34583	9.78325
248	16.63349	4.84279	4.50578	4.41566	10.37979	10.449	10.34862	9.78559
247.5	17.80155	5.44339	5.19129	5.08746	10.38256	10.4519	10.3515	9.78815
247	19.02029	6.21747	5.89192	5.77408	10.38497	10.4548	10.35397	9.79031
246.5	20.26794	6.94796	6.56436	6.43307	10.38719	10.4574	10.35655	9.79236
246	21.55027	7.21092	7.26246	7.11721	10.38923	10.4599	10.35892	9.79455
245.5	22.80694	7.50535	7.83148	7.67485	10.3917	10.4622	10.36098	9.79646
245	24.10953	8.41421	8.4017	8.23367	10.39396	10.4648	10.36345	9.79882
244.5	25.46845	9.41912	9.31878	9.1324	10.39641	10.4673	10.36582	9.80097
244	26.79434	9.90473	9.96307	9.76381	10.39871	10.4698	10.36798	9.80321
243.5	28.11611	10.3608	10.4857	10.27599	10.40092	10.4722	10.37025	9.8054
243	29.49244	11.1848	11.2458	11.02088	10.40375	10.475	10.37292	9.80792
242.5	30.87403	11.7654	11.8367	11.59997	10.40605	10.4775	10.37509	9.81036
242	32.2696	12.3297	12.6165	12.36417	10.40866	10.4804	10.37766	9.81303
241.5	33.67935	12.2939	13.4273	13.15875	10.4113	10.483	10.38003	9.81549
241	35.09259	12.7927	13.9961	13.71618	10.41368	10.486	10.38281	9.81831
240.5	36.54423	13.7336	14.7608	14.46558	10.41662	10.4892	10.3859	9.82134
240	37.9986	14.6434	15.3903	15.08249	10.41938	10.4925	10.38889	9.82425
239.5	39.45502	15.2934	16.4297	16.10111	10.4224	10.4959	10.39208	9.82756
239	40.87534	16.0242	16.9103	16.57209	10.42544	10.4992	10.39528	9.83074
238.5	42.30839	16.2036	17.641	17.28818	10.42859	10.5027	10.39878	9.83436
238	43.66365	16.8866	18.1844	17.82071	10.43201	10.5062	10.40197	9.83772
237.5	44.95592	17.87	18.664	18.29072	10.43541	10.51	10.40557	9.84143

237	46.23903	18.1698	19.9039	19.50582	10.43909	10.5139	10.40949	9.84536
236.5	47.4697	18.6713	20.7556	20.34049	10.44313	10.5184	10.41392	9.8496
236	48.61868	19.2481	21.4181	20.98974	10.44728	10.5234	10.41897	9.85421
235.5	49.77848	20.0738	22.1159	21.67358	10.45145	10.528	10.4235	9.85856
235	50.84219	20.2547	22.7734	22.31793	10.45468	10.532	10.42731	9.86229
234.5	51.81395	20.4364	23.5035	23.03343	10.45805	10.5363	10.43153	9.86627
234	52.686	20.6552	24.528	24.03744	10.4619	10.541	10.43627	9.87074
233.5	53.45908	21.2252	24.9862	24.48648	10.46584	10.5459	10.44101	9.87527
233	54.13081	21.8044	25.6926	25.17875	10.46964	10.5508	10.44595	9.87992
232.5	54.65357	22.0589	26.1502	25.6272	10.47389	10.556	10.4512	9.8849
232	55.00834	22.3639	26.7513	26.21627	10.47815	10.5612	10.45594	9.88972
231.5	55.20465	22.5307	27.3026	26.75655	10.4829	10.5669	10.46181	9.89538
231	55.28458	22.796	27.5761	27.02458	10.48772	10.5727	10.46758	9.90081
230.5	55.25939	22.846	27.9723	27.41285	10.49201	10.578	10.47294	9.90598
230	55.09142	22.72	28.3404	27.77359	10.49664	10.5837	10.47881	9.91142
229.5	54.82149	22.8944	28.6725	28.09905	10.50115	10.5895	10.48478	9.91704
229	54.40158	23.1045	28.737	28.16226	10.50605	10.5956	10.49117	9.92268
228.5	53.85856	23.3182	28.564	27.99272	10.51085	10.6019	10.49755	9.92864
228	53.1491	22.5046	28.2958	27.72988	10.51617	10.6085	10.50415	9.93487
227.5	52.31903	22.1686	28.2557	27.69059	10.52121	10.6148	10.51084	9.94101
227	51.24785	22.0596	27.934	27.37532	10.52655	10.6211	10.51764	9.94719
226.5	50.07287	21.6193	27.544	26.99312	10.53212	10.6277	10.52464	9.95354
226	48.83364	21.4232	26.9622	26.42296	10.53824	10.6346	10.53206	9.9603
225.5	47.55331	21.362	26.1753	25.65179	10.54425	10.6416	10.53978	9.96706
225	46.26217	20.6034	25.4512	24.94218	10.55112	10.6491	10.54823	9.97449
224.5	44.93841	20.4028	24.6205	24.12809	10.55817	10.6568	10.55678	9.98182
224	43.56046	19.4737	23.9176	23.43925	10.56458	10.6641	10.56471	9.98856
223.5	42.18398	18.1535	23.3697	22.90231	10.57162	10.6717	10.57316	9.99585
223	40.81877	17.7107	22.2685	21.82313	10.57929	10.68	10.58243	10.0035
222.5	39.43681	16.8623	21.2025	20.77845	10.58637	10.6876	10.59098	10.0106

222	38.13125	16.526	20.1623	19.75905	10.59378	10.6956	10.59963	10.0178
221.5	36.93717	15.941	19.1977	18.81375	10.60208	10.7044	10.60931	10.026
221	35.74464	15.207	18.3692	18.00182	10.61116	10.7137	10.61961	10.0345
220.5	34.63002	14.2001	17.1611	16.81788	10.61974	10.723	10.6295	10.0427
220	33.6401	13.8199	16.7442	16.40932	10.62737	10.731	10.63794	10.0497
219.5	32.73382	13.3863	16.3979	16.06994	10.63541	10.7394	10.64701	10.0571
219	31.96022	13.2688	15.8897	15.57191	10.64255	10.747	10.65494	10.0637
218.5	31.32404	12.9531	15.1069	14.80476	10.65056	10.7553	10.6639	10.071
218	30.80682	12.6543	14.8325	14.53585	10.65985	10.7648	10.67399	10.0789
217.5	30.3154	12.456	14.3557	14.06859	10.66845	10.7739	10.68378	10.0864
217	29.8946	12.5135	13.9729	13.69344	10.67637	10.7821	10.69222	10.0932
216.5	29.55104	12.2481	13.8233	13.54683	10.6847	10.791	10.70119	10.1002
216	29.37767	12.4489	13.654	13.38092	10.69368	10.8005	10.71056	10.1073
215.5	29.3182	12.3114	13.3427	13.07585	10.703	10.8105	10.72045	10.115
215	29.41153	12.7573	13.017	12.75666	10.71263	10.8206	10.73044	10.1224
214.5	29.58515	13.1144	13.2042	12.94012	10.7214	10.83	10.7394	10.1292
214	29.78977	13.2141	13.5889	13.31712	10.72964	10.8388	10.74784	10.1354
213.5	30.03822	12.7265	13.6666	13.39327	10.74055	10.8503	10.75866	10.1431
213	30.36536	12.8397	13.7807	13.50509	10.75008	10.8605	10.76824	10.1501
212.5	30.74531	12.5724	13.6201	13.3477	10.75842	10.8698	10.77689	10.1561
212	31.18174	12.5278	13.9886	13.70883	10.76805	10.8802	10.78668	10.1628
211.5	31.65456	12.638	13.8022	13.52616	10.77864	10.8915	10.79728	10.1699
211	32.27782	12.4154	13.8077	13.53155	10.7902	10.9039	10.80892	10.1776
210.5	32.98883	12.7271	13.8669	13.58956	10.80186	10.9164	10.82046	10.1852
210	33.72511	12.4827	13.9248	13.6463	10.81203	10.9274	10.83086	10.1919
209.5	34.35663	11.7322	13.852	13.57496	10.82562	10.942	10.84436	10.2007
209	34.98455	11.8195	14.1972	13.91326	10.83963	10.9566	10.85785	10.2097
208.5	35.69507	11.6102	14.4146	14.12631	10.85333	10.9711	10.87165	10.2184
208	36.24988	11.594	14.9106	14.61239	10.86756	10.986	10.88586	10.2277
207.5	36.64299	12.1316	14.9301	14.6315	10.88137	11.0006	10.90008	10.2365

207	37.00789	11.5826	14.8508	14.55378	10.89592	11.0158	10.9145	10.2455
206.5	37.37525	10.4822	15.093	14.79114	10.91058	11.0312	10.92923	10.255
206	37.51615	10.7272	14.5611	14.26988	10.92598	11.047	10.94447	10.2645
205.5	37.66685	10.3804	14.1132	13.83094	10.94332	11.0648	10.96188	10.2754
205	37.709	10.2024	13.6667	13.39337	10.96055	11.0819	10.97898	10.2858
204.5	37.5507	10.9564	13.5718	13.30036	10.97799	11.0993	10.99618	10.2965
204	37.10436	11.9073	13.4834	13.21373	10.99682	11.1176	11.01472	10.3079
203.5	36.38325	11.4839	12.8744	12.61691	11.0164	11.1368	11.03418	10.3199
203	35.48167	11.6034	12.3391	12.09232	11.03256	11.1525	11.04994	10.3297
202.5	34.39687	10.733	11.8041	11.56802	11.05032	11.1694	11.06725	10.3403
202	33.33627	10.5136	10.6553	10.44219	11.07054	11.1884	11.08671	10.3524
201.5	32.16366	9.57867	10.1321	9.92946	11.09044	11.2068	11.10608	10.3643
201	30.87906	8.43884	8.75981	8.58461	11.11056	11.2246	11.12503	10.3757
200.5	29.48245	6.83875	7.34874	7.20177	11.13014	11.2415	11.14316	10.3867
200	27.97385	5.11412	5.52498	5.41448	11.14822	11.2566	11.15974	10.3965
199.5	26.35325	3.84731	4.21763	4.13328	11.16738	11.2722	11.17725	10.4069
199	24.62065	2.9965	1.80662	1.77049	11.18995	11.2899	11.19775	10.419
198.5	22.77605	0.83146	0.31711	0.31077	11.21071	11.3056	11.21608	10.4295
198	20.81946	0.59596	-1.54471	-1.51382	11.23564	11.3236	11.23771	10.4418
197.5	18.75086	-1.57779	-3.71955	-3.64516	11.25854	11.3395	11.25728	10.4529
197	16.57027	-5.29342	-6.05684	-5.9357	11.28304	11.3556	11.27788	10.4643
196.5	14.27767	-9.11674	-8.70208	-8.52804	11.31258	11.374	11.30209	10.4776
196	11.87308	-11.9249	-11.0229	-10.80244	11.34296	11.3919	11.3267	10.4914
195.5	9.35649	-13.7064	-12.9756	-12.71609	11.37282	11.4082	11.3506	10.5046
195	6.7279	-16.7172	-16.0592	-15.73802	11.40556	11.4249	11.37656	10.5191
194.5	3.98731	-19.6529	-19.6648	-19.2715	11.43702	11.4399	11.40117	10.5334
194	1.13473	-21.1356	-22.6287	-22.17613	11.46644	11.4527	11.42394	10.5467
193.5	-1.82986	-22.055	-26.1964	-25.67247	11.49533	11.4636	11.44588	10.5597
193	-4.90644	-24.3313	-27.4151	-26.8668	11.52294	11.4726	11.46658	10.573
192.5	-8.09503	-25.7276	-29.6671	-29.07376	11.55172	11.4804	11.48831	10.5879

192	-11.39561	-26.1283	-31.2222	-30.59776	11.5805	11.4862	11.50974	10.6035
191.5	-14.80819	-28.4813	-31.7726	-31.13715	11.60511	11.4877	11.52766	10.6178
191	-18.33277	-29.2981	-32.1494	-31.50641	11.62405	11.4863	11.54177	10.6313
190.5	-21.96934	-28.7155	-32.9952	-32.3353	11.63882	11.4842	11.553	10.644
190	-25.71792	-28.271	-32.911	-32.25278	11.65241	11.4803	11.56371	10.6576

Figure S10

Temperature	D-SA	SA
30.002	-0.01542	-0.04059
30.002	-0.01531	-0.04065
30.002	-0.01533	-0.04071
30.002	-0.01543	-0.04077
30.001	-0.01559	-0.04082
30.002	-0.0158	-0.04087
30.002	-0.01598	-0.04093
30.003	-0.01621	-0.04099
30.003	-0.01642	-0.04102
30.002	-0.01671	-0.04106
30.002	-0.01692	-0.04111
30.002	-0.01709	-0.04116
30.003	-0.01731	-0.0412
30.002	-0.01755	-0.04124
30.002	-0.01785	-0.04128
30.004	-0.01816	-0.04134
30.003	-0.01845	-0.04143
30.004	-0.01877	-0.04155
30.004	-0.0191	-0.04164
30.004	-0.01942	-0.04172
30.006	-0.01977	-0.04179
30.006	-0.02015	-0.04188

30.006	-0.02037	-0.04197
30.006	-0.02068	-0.04207
30.007	-0.02101	-0.04217
30.008	-0.02138	-0.04227
30.009	-0.02167	-0.04243
30.009	-0.02204	-0.04263
30.01	-0.0225	-0.04285
30.011	-0.0231	-0.04306
30.013	-0.02382	-0.04332
30.014	-0.02477	-0.04366
30.016	-0.02574	-0.04412
30.018	-0.02674	-0.04475
30.019	-0.02808	-0.04547
30.021	-0.02953	-0.04632
30.023	-0.03113	-0.04739
30.026	-0.0326	-0.04877
30.028	-0.03437	-0.05036
30.03	-0.03631	-0.05209
30.034	-0.03837	-0.05367
30.036	-0.04034	-0.0554
30.04	-0.04243	-0.05725
30.043	-0.04456	-0.05867
30.046	-0.04701	-0.06033
30.05	-0.04928	-0.0621
30.054	-0.05205	-0.06389
30.058	-0.05445	-0.06578
30.062	-0.05734	-0.06746
30.068	-0.06056	-0.0697
30.072	-0.06382	-0.0718
30.078	-0.06721	-0.07383

30.083	-0.07075	-0.07605
30.088	-0.07439	-0.07859
30.094	-0.07789	-0.08081
30.101	-0.08176	-0.0833
30.107	-0.08567	-0.08567
30.113	-0.08953	-0.08806
30.121	-0.0934	-0.09077
30.127	-0.09737	-0.09358
30.135	-0.10152	-0.09638
30.142	-0.1059	-0.09907
30.15	-0.11021	-0.10201
30.158	-0.11456	-0.10458
30.166	-0.11937	-0.10758
30.175	-0.12407	-0.11047
30.184	-0.12875	-0.11345
30.193	-0.13357	-0.11635
30.202	-0.13815	-0.11897
30.212	-0.1426	-0.12191
30.221	-0.14739	-0.12457
30.233	-0.15161	-0.12736
30.242	-0.1564	-0.1299
30.252	-0.16082	-0.13258
30.263	-0.1654	-0.13535
30.273	-0.16997	-0.13788
30.283	-0.17474	-0.14062
30.295	-0.17938	-0.1433
30.307	-0.18403	-0.14591
30.318	-0.18877	-0.1484
30.329	-0.19302	-0.15108
30.34	-0.19727	-0.15363

30.352	-0.20163	-0.15571
30.365	-0.20592	-0.15801
30.377	-0.20983	-0.16034
30.389	-0.21409	-0.1625
30.402	-0.21768	-0.16451
30.415	-0.22158	-0.16657
30.428	-0.22543	-0.16841
30.44	-0.22922	-0.17026
30.453	-0.23302	-0.17202
30.466	-0.23667	-0.17381
30.478	-0.24052	-0.17567
30.492	-0.24396	-0.17724
30.505	-0.24728	-0.17897
30.518	-0.25036	-0.18082
30.532	-0.2536	-0.18213
30.545	-0.25644	-0.18371
30.558	-0.25959	-0.18506
30.573	-0.26246	-0.18648
30.587	-0.26541	-0.18779
30.601	-0.26804	-0.18904
30.614	-0.27112	-0.19029
30.628	-0.27413	-0.1913
30.642	-0.27666	-0.19235
30.656	-0.27918	-0.19322
30.671	-0.28158	-0.19418
30.685	-0.28393	-0.19504
30.699	-0.28607	-0.19617
30.713	-0.28811	-0.19705
30.728	-0.29029	-0.19802
30.742	-0.29274	-0.19883

30.756	-0.29476	-0.19956
30.771	-0.29672	-0.20037
30.785	-0.29892	-0.20123
30.8	-0.30077	-0.202
30.814	-0.30229	-0.20248
30.829	-0.30406	-0.20303
30.842	-0.30602	-0.20373
30.858	-0.30779	-0.2046
30.872	-0.30941	-0.20536
30.887	-0.31139	-0.20612
30.902	-0.3133	-0.2065
30.916	-0.31523	-0.20711
30.931	-0.31694	-0.20771
30.947	-0.31814	-0.20828
30.961	-0.31978	-0.20901
30.976	-0.32082	-0.20942
30.992	-0.32209	-0.20994
31.006	-0.32346	-0.2104
31.021	-0.32526	-0.21091
31.036	-0.32659	-0.21141
31.051	-0.32836	-0.21197
31.066	-0.32974	-0.21234
31.08	-0.33096	-0.2127
31.096	-0.33231	-0.21345
31.111	-0.33355	-0.2138
31.126	-0.33465	-0.2143
31.142	-0.3355	-0.21461
31.156	-0.33663	-0.21512
31.172	-0.33785	-0.21573
31.186	-0.33928	-0.21611

31.202	-0.34056	-0.21669
31.217	-0.34186	-0.21712
31.233	-0.34303	-0.21769
31.249	-0.34418	-0.21829
31.263	-0.34544	-0.21898
31.28	-0.34662	-0.21933
31.294	-0.34793	-0.2197
31.309	-0.34917	-0.22019
31.325	-0.35039	-0.22062
31.341	-0.35159	-0.22114
31.356	-0.35258	-0.22165
31.371	-0.35359	-0.22205
31.388	-0.3546	-0.22234
31.403	-0.35555	-0.22261
31.418	-0.35656	-0.22295
31.434	-0.35739	-0.22377
31.45	-0.35836	-0.22425
31.465	-0.35917	-0.22454
31.481	-0.3599	-0.22481
31.496	-0.36098	-0.22546
31.513	-0.36194	-0.22552
31.527	-0.36305	-0.22612
31.543	-0.36387	-0.22663
31.558	-0.36467	-0.22706
31.576	-0.36578	-0.22732
31.59	-0.36671	-0.22752
31.606	-0.36801	-0.22799
31.622	-0.36891	-0.22835
31.638	-0.3698	-0.22858
31.654	-0.37046	-0.22892

31.67	-0.37155	-0.22941
31.686	-0.37226	-0.2297
31.701	-0.37297	-0.22996
31.717	-0.3737	-0.2302
31.733	-0.37448	-0.23057
31.749	-0.37538	-0.23097
31.765	-0.37611	-0.23137
31.78	-0.37686	-0.23177
31.796	-0.37739	-0.23221
31.812	-0.378	-0.23242
31.828	-0.37879	-0.2327
31.844	-0.37932	-0.23298
31.86	-0.37993	-0.23325
31.876	-0.3806	-0.2335
31.892	-0.38144	-0.23371
31.908	-0.38237	-0.23388
31.924	-0.38312	-0.23409
31.939	-0.38391	-0.23459
31.957	-0.38458	-0.23514
31.972	-0.38488	-0.23558
31.988	-0.38544	-0.23586
32.005	-0.38622	-0.236
32.021	-0.38626	-0.2362
32.037	-0.38701	-0.23633
32.053	-0.38715	-0.23636
32.069	-0.38783	-0.23641
32.084	-0.38855	-0.23647
32.101	-0.38909	-0.23661
32.117	-0.38951	-0.23689
32.132	-0.39005	-0.23722

32.149	-0.39063	-0.23758
32.165	-0.39131	-0.23795
32.182	-0.39173	-0.23831
32.198	-0.39218	-0.23857
32.213	-0.39265	-0.23879
32.23	-0.39308	-0.2389
32.246	-0.39346	-0.23896
32.261	-0.39398	-0.23896
32.277	-0.39444	-0.23897
32.294	-0.3949	-0.23907
32.31	-0.39539	-0.2392
32.327	-0.39575	-0.23935
32.342	-0.39619	-0.23954
32.359	-0.39663	-0.23976
32.374	-0.39743	-0.23999
32.391	-0.39804	-0.24017
32.407	-0.39848	-0.24041
32.423	-0.39883	-0.24069
32.44	-0.39914	-0.24094
32.455	-0.39945	-0.24118
32.472	-0.39975	-0.24144
32.488	-0.39996	-0.24169
32.504	-0.40012	-0.24195
32.521	-0.40023	-0.24217
32.537	-0.4004	-0.24237
32.553	-0.40066	-0.24251
32.568	-0.40093	-0.24254
32.586	-0.40122	-0.24263
32.602	-0.40154	-0.24272
32.618	-0.40191	-0.24284

32.634	-0.40223	-0.24296
32.65	-0.40273	-0.24308
32.666	-0.40335	-0.24319
32.683	-0.40393	-0.24333
32.699	-0.40443	-0.24353
32.715	-0.40469	-0.24373
32.731	-0.40499	-0.24388
32.747	-0.40539	-0.244
32.764	-0.40565	-0.24414
32.78	-0.4059	-0.24427
32.797	-0.40606	-0.24446
32.813	-0.40616	-0.24465
32.828	-0.40625	-0.24482
32.845	-0.40637	-0.24496
32.861	-0.40652	-0.24507
32.877	-0.40677	-0.24522
32.893	-0.4071	-0.24537
32.91	-0.40744	-0.24553
32.926	-0.40786	-0.24564
32.943	-0.40828	-0.24576
32.959	-0.40857	-0.24589
32.975	-0.40882	-0.246
32.992	-0.40902	-0.24611
33.007	-0.40916	-0.24622
33.024	-0.40932	-0.24631
33.04	-0.40947	-0.24641
33.055	-0.40966	-0.24655
33.072	-0.40992	-0.24674
33.088	-0.41025	-0.24694
33.105	-0.41059	-0.24712

33.121	-0.41105	-0.24732
33.138	-0.41144	-0.24745
33.155	-0.41175	-0.2476
33.17	-0.41203	-0.24779
33.186	-0.41224	-0.24799
33.203	-0.41241	-0.24817
33.218	-0.41257	-0.24835
33.236	-0.41273	-0.24854
33.252	-0.41283	-0.24869
33.268	-0.41298	-0.2488
33.284	-0.41311	-0.24891
33.301	-0.41328	-0.249
33.316	-0.41342	-0.24906
33.333	-0.41358	-0.24913
33.349	-0.41376	-0.24926
33.366	-0.41392	-0.24943
33.383	-0.4141	-0.24964
33.398	-0.41429	-0.24987
33.415	-0.41447	-0.25004
33.431	-0.41468	-0.25017
33.447	-0.41498	-0.25026
33.463	-0.4153	-0.25036
33.48	-0.41557	-0.25044
33.496	-0.41579	-0.25051
33.512	-0.41597	-0.25056
33.528	-0.41612	-0.25065
33.545	-0.41632	-0.25078
33.561	-0.41648	-0.25092
33.577	-0.41661	-0.25106
33.593	-0.41676	-0.25116

33.611	-0.41687	-0.25131
33.627	-0.41697	-0.25143
33.643	-0.41709	-0.25154
33.658	-0.41729	-0.25165
33.675	-0.4175	-0.25178
33.692	-0.41769	-0.25192
33.708	-0.41788	-0.25205
33.724	-0.41805	-0.25217
33.741	-0.41821	-0.25229
33.758	-0.41834	-0.25241
33.774	-0.41851	-0.25252
33.79	-0.41868	-0.25262
33.806	-0.41882	-0.25277
33.823	-0.41892	-0.25292
33.84	-0.41903	-0.25308
33.856	-0.41919	-0.25328
33.872	-0.41934	-0.25344
33.889	-0.41948	-0.25358
33.904	-0.41962	-0.25371
33.921	-0.41972	-0.25383
33.937	-0.4198	-0.25395
33.952	-0.41991	-0.25405
33.969	-0.42005	-0.25414
33.986	-0.42022	-0.25425
34.002	-0.42039	-0.25435
34.019	-0.42059	-0.25445
34.036	-0.42083	-0.25455
34.052	-0.42112	-0.25465
34.068	-0.42137	-0.25473
34.084	-0.42165	-0.25483

34.101	-0.42189	-0.2549
34.116	-0.42205	-0.25499
34.134	-0.42215	-0.25509
34.149	-0.42223	-0.25521
34.167	-0.42229	-0.25535
34.183	-0.42236	-0.25547
34.199	-0.42244	-0.25558
34.215	-0.42254	-0.25567
34.231	-0.42267	-0.25576
34.247	-0.42279	-0.25583
34.265	-0.42294	-0.25592
34.281	-0.4231	-0.25603
34.297	-0.42323	-0.25614
34.313	-0.42336	-0.25625
34.329	-0.4235	-0.25641
34.346	-0.42363	-0.25657
34.363	-0.42377	-0.25672
34.379	-0.42391	-0.25683
34.395	-0.42403	-0.25693
34.412	-0.42415	-0.25703
34.427	-0.42424	-0.25713
34.444	-0.42432	-0.25725
34.459	-0.42439	-0.25742
34.476	-0.4245	-0.25767
34.493	-0.42467	-0.25787
34.509	-0.42485	-0.25809
34.526	-0.42507	-0.25823
34.543	-0.42527	-0.25834
34.56	-0.4255	-0.25841
34.575	-0.42572	-0.25848

34.592	-0.42586	-0.25853
34.607	-0.42596	-0.25858
34.623	-0.42603	-0.25864
34.641	-0.4261	-0.25873
34.657	-0.4262	-0.25884
34.673	-0.42632	-0.25897
34.69	-0.42647	-0.25914
34.706	-0.42666	-0.25925
34.723	-0.42679	-0.25932
34.739	-0.42696	-0.25937
34.755	-0.4271	-0.25945
34.771	-0.42721	-0.25952
34.788	-0.42732	-0.25958
34.804	-0.4274	-0.25967
34.82	-0.42747	-0.25981
34.836	-0.42754	-0.25995
34.853	-0.42763	-0.2601
34.869	-0.42773	-0.26026
34.886	-0.42785	-0.26039
34.902	-0.42799	-0.26048
34.918	-0.42816	-0.26056
34.935	-0.42835	-0.26067
34.951	-0.42851	-0.26079
34.968	-0.42865	-0.26089
34.985	-0.42878	-0.26098
35.001	-0.42889	-0.26106
35.017	-0.429	-0.26115
35.033	-0.42912	-0.26126
35.05	-0.42922	-0.26137
35.066	-0.42931	-0.26146

35.082	-0.4294	-0.26156
35.099	-0.42951	-0.26166
35.116	-0.42964	-0.26179
35.131	-0.42977	-0.26193
35.148	-0.42992	-0.26205
35.165	-0.43009	-0.26217
35.181	-0.43021	-0.26228
35.197	-0.43036	-0.26241
35.214	-0.43051	-0.26256
35.23	-0.43065	-0.26271
35.246	-0.43078	-0.26284
35.263	-0.43091	-0.26297
35.28	-0.43107	-0.26309
35.295	-0.43124	-0.26323
35.312	-0.43142	-0.26335
35.328	-0.43161	-0.26345
35.346	-0.43184	-0.26355
35.361	-0.43201	-0.26365
35.378	-0.43213	-0.2638
35.394	-0.43223	-0.26394
35.411	-0.43235	-0.26405
35.428	-0.43244	-0.26415
35.443	-0.43251	-0.26425
35.46	-0.4326	-0.26437
35.477	-0.43267	-0.26448
35.492	-0.43273	-0.26458
35.509	-0.43278	-0.26466
35.525	-0.43284	-0.26475
35.542	-0.43291	-0.26484
35.558	-0.433	-0.2649

35.573	-0.43314	-0.26498
35.591	-0.43331	-0.26508
35.607	-0.4335	-0.26521
35.624	-0.43368	-0.26533
35.64	-0.43386	-0.26548
35.657	-0.43405	-0.26565
35.673	-0.43421	-0.26577
35.689	-0.43434	-0.26591
35.705	-0.43449	-0.26603
35.722	-0.43466	-0.26613
35.738	-0.4348	-0.2662
35.755	-0.43492	-0.26626
35.771	-0.43504	-0.26633
35.788	-0.43515	-0.26642
35.804	-0.43523	-0.26649
35.82	-0.4353	-0.26655
35.836	-0.43536	-0.26664
35.854	-0.43542	-0.26673
35.87	-0.43548	-0.26682
35.886	-0.43556	-0.26693
35.902	-0.43568	-0.26706
35.918	-0.43579	-0.2672
35.935	-0.43594	-0.26731
35.952	-0.43611	-0.26746
35.968	-0.43623	-0.26759
35.984	-0.43634	-0.26773
36.001	-0.43643	-0.2679
36.018	-0.43653	-0.26802
36.034	-0.43665	-0.26811
36.05	-0.43673	-0.26817

36.067	-0.43681	-0.26825
36.083	-0.4369	-0.26832
36.1	-0.43699	-0.26838
36.116	-0.43707	-0.26846
36.132	-0.4372	-0.26858
36.149	-0.43733	-0.26873
36.165	-0.43746	-0.26888
36.182	-0.43755	-0.26912
36.198	-0.43763	-0.2693
36.215	-0.43772	-0.26942
36.232	-0.43781	-0.26949
36.247	-0.43788	-0.26955
36.264	-0.43795	-0.26963
36.281	-0.43804	-0.2697
36.297	-0.43809	-0.26978
36.313	-0.43816	-0.26987
36.329	-0.43824	-0.26998
36.346	-0.43835	-0.27006
36.363	-0.43845	-0.27019
36.379	-0.43858	-0.27031
36.395	-0.4387	-0.27041
36.412	-0.43879	-0.27051
36.428	-0.4389	-0.27059
36.444	-0.43901	-0.2707
36.461	-0.43914	-0.27081
36.478	-0.43926	-0.27097
36.494	-0.43942	-0.27113
36.51	-0.43958	-0.27134
36.527	-0.43974	-0.27147
36.544	-0.43988	-0.27164

36.56	-0.44	-0.27182
36.577	-0.44012	-0.27196
36.593	-0.44021	-0.2721
36.609	-0.44027	-0.27223
36.626	-0.44035	-0.27235
36.643	-0.44044	-0.27246
36.659	-0.44051	-0.27256
36.675	-0.44057	-0.27263
36.692	-0.44064	-0.27271
36.708	-0.44068	-0.27277
36.724	-0.44073	-0.27284
36.741	-0.44079	-0.27292
36.756	-0.44086	-0.27301
36.774	-0.44094	-0.27309
36.79	-0.44101	-0.2732
36.807	-0.44108	-0.27332
36.823	-0.44117	-0.27344
36.839	-0.44126	-0.27352
36.856	-0.4414	-0.27361
36.873	-0.44155	-0.27371
36.889	-0.44169	-0.27385
36.906	-0.44177	-0.27399
36.922	-0.44187	-0.2741
36.939	-0.44198	-0.27421
36.955	-0.44207	-0.27431
36.972	-0.44214	-0.27439
36.988	-0.44223	-0.27447
37.005	-0.44229	-0.27453
37.02	-0.44234	-0.27459
37.037	-0.44239	-0.27465

37.053	-0.44245	-0.27472
37.07	-0.44251	-0.27483
37.087	-0.44254	-0.27494
37.103	-0.44259	-0.27505
37.118	-0.44264	-0.27515
37.136	-0.44271	-0.27528
37.152	-0.44278	-0.27542
37.17	-0.44287	-0.2756
37.185	-0.44295	-0.27577
37.202	-0.44304	-0.27592
37.219	-0.44311	-0.27607
37.236	-0.44319	-0.27621
37.251	-0.44329	-0.27633
37.268	-0.44339	-0.27641
37.285	-0.4435	-0.27647
37.301	-0.44361	-0.27652
37.318	-0.44375	-0.27657
37.334	-0.4439	-0.27664
37.349	-0.4441	-0.27672
37.367	-0.44426	-0.27686
37.383	-0.44441	-0.27697
37.398	-0.44452	-0.27707
37.415	-0.44459	-0.27717
37.433	-0.44466	-0.27724
37.448	-0.44476	-0.2773
37.465	-0.44485	-0.27737
37.482	-0.44497	-0.27747
37.499	-0.44509	-0.27755
37.515	-0.44526	-0.27761
37.532	-0.44545	-0.27771

37.548	-0.44564	-0.27783
37.564	-0.4458	-0.278
37.58	-0.44592	-0.27815
37.597	-0.44601	-0.27826
37.613	-0.44609	-0.27838
37.63	-0.44614	-0.27846
37.647	-0.44619	-0.27854
37.663	-0.44623	-0.27864
37.68	-0.44628	-0.27874
37.697	-0.44633	-0.27887
37.713	-0.44638	-0.279
37.729	-0.44643	-0.27909
37.745	-0.4465	-0.27919
37.762	-0.44665	-0.2793
37.778	-0.44679	-0.27939
37.794	-0.44695	-0.27948
37.812	-0.44709	-0.27957
37.828	-0.44719	-0.27965
37.845	-0.4473	-0.27972
37.861	-0.4474	-0.27984
37.877	-0.44751	-0.28
37.893	-0.44759	-0.28011
37.91	-0.44765	-0.28021
37.926	-0.44768	-0.28031
37.943	-0.44775	-0.28043
37.96	-0.44787	-0.28053
37.977	-0.44801	-0.28061
37.993	-0.44813	-0.28069
38.009	-0.44821	-0.28076
38.026	-0.4483	-0.28083

38.043	-0.44839	-0.28093
38.059	-0.44846	-0.28106
38.075	-0.44857	-0.28121
38.092	-0.44865	-0.28136
38.109	-0.44871	-0.28151
38.125	-0.44878	-0.2816
38.141	-0.44886	-0.28171
38.158	-0.44893	-0.28182
38.175	-0.449	-0.28195
38.191	-0.44905	-0.28207
38.207	-0.4491	-0.28218
38.224	-0.44917	-0.28228
38.241	-0.44925	-0.28238
38.256	-0.44934	-0.28249
38.273	-0.44944	-0.28262
38.289	-0.44955	-0.28278
38.307	-0.44966	-0.2829
38.322	-0.44978	-0.28301
38.34	-0.44987	-0.28311
38.356	-0.44995	-0.28319
38.372	-0.45002	-0.28329
38.389	-0.45008	-0.28339
38.404	-0.45019	-0.2835
38.422	-0.45029	-0.2836
38.438	-0.45038	-0.2837
38.455	-0.4505	-0.2838
38.471	-0.45065	-0.28393
38.488	-0.45078	-0.28409
38.505	-0.45088	-0.28423
38.521	-0.45097	-0.28436

38.538	-0.45104	-0.28445
38.555	-0.4511	-0.28454
38.571	-0.45115	-0.2846
38.587	-0.45121	-0.28467
38.603	-0.45128	-0.28475
38.62	-0.45136	-0.28482
38.636	-0.45146	-0.28487
38.653	-0.45163	-0.28496
38.669	-0.45178	-0.28505
38.686	-0.45194	-0.28515
38.703	-0.45209	-0.28527
38.719	-0.45218	-0.2854
38.735	-0.45223	-0.28556
38.752	-0.45226	-0.2857
38.769	-0.4523	-0.28586
38.786	-0.45235	-0.28597
38.802	-0.45242	-0.28606
38.818	-0.45251	-0.28612
38.834	-0.45265	-0.28618
38.851	-0.4528	-0.28624
38.867	-0.45293	-0.28631
38.883	-0.4531	-0.28639
38.9	-0.45322	-0.28645
38.917	-0.45329	-0.28652
38.934	-0.45333	-0.28659
38.95	-0.45339	-0.2867
38.967	-0.45347	-0.2868
38.983	-0.45355	-0.28688
39.001	-0.45365	-0.28697
39.017	-0.45374	-0.28706

39.032	-0.45382	-0.28715
39.05	-0.45388	-0.28723
39.066	-0.45394	-0.28732
39.082	-0.454	-0.28739
39.099	-0.45406	-0.28747
39.116	-0.45411	-0.28756
39.131	-0.45416	-0.2877
39.149	-0.45419	-0.28787
39.165	-0.45425	-0.288
39.181	-0.45435	-0.28814
39.198	-0.45445	-0.28827
39.215	-0.45453	-0.28841
39.231	-0.45461	-0.2885
39.248	-0.45468	-0.28858
39.263	-0.45475	-0.28865
39.281	-0.45482	-0.28872
39.298	-0.45491	-0.28877
39.313	-0.45502	-0.28885
39.33	-0.45511	-0.28897
39.346	-0.4552	-0.28912
39.363	-0.45533	-0.28931
39.38	-0.45546	-0.28954
39.396	-0.45557	-0.28972
39.413	-0.4557	-0.2899
39.43	-0.45578	-0.29003
39.446	-0.45583	-0.29014
39.462	-0.45587	-0.29024
39.479	-0.45591	-0.29033
39.496	-0.45595	-0.29042
39.512	-0.456	-0.29052

39.529	-0.45609	-0.29063
39.546	-0.45617	-0.29078
39.562	-0.45626	-0.2909
39.578	-0.45634	-0.291
39.595	-0.45642	-0.29109
39.612	-0.45647	-0.29117
39.629	-0.45651	-0.29127
39.645	-0.45656	-0.29135
39.661	-0.45661	-0.29144
39.677	-0.45667	-0.29154
39.695	-0.45673	-0.29161
39.711	-0.4568	-0.29169
39.728	-0.45684	-0.29176
39.744	-0.45688	-0.29184
39.761	-0.45692	-0.29191
39.777	-0.45696	-0.292
39.793	-0.45703	-0.2921
39.81	-0.45709	-0.29221
39.826	-0.45718	-0.29232
39.843	-0.45729	-0.29247
39.86	-0.45741	-0.2926
39.876	-0.45754	-0.29274
39.892	-0.45766	-0.29286
39.908	-0.45776	-0.293
39.926	-0.45786	-0.29314
39.943	-0.45793	-0.29327
39.959	-0.45801	-0.29337
39.975	-0.45808	-0.29349
39.992	-0.45814	-0.29358
40.008	-0.45821	-0.29365

40.024	-0.4583	-0.29372
40.04	-0.45836	-0.2938
40.058	-0.45843	-0.29388
40.075	-0.45852	-0.29399
40.091	-0.45862	-0.29412
40.107	-0.45874	-0.29427
40.124	-0.45881	-0.2944
40.14	-0.45887	-0.29452
40.158	-0.45896	-0.29462
40.175	-0.45905	-0.29474
40.19	-0.45915	-0.29482
40.207	-0.45922	-0.29487
40.223	-0.45928	-0.29493
40.24	-0.45932	-0.29498
40.256	-0.45937	-0.29506
40.272	-0.45941	-0.29512
40.29	-0.45947	-0.29521
40.306	-0.45951	-0.2953
40.322	-0.45958	-0.29539
40.338	-0.45965	-0.29547
40.356	-0.45979	-0.29557
40.372	-0.45992	-0.29569
40.39	-0.46003	-0.2958
40.406	-0.4601	-0.29595
40.422	-0.46017	-0.29604
40.439	-0.46027	-0.29613
40.454	-0.46036	-0.29621
40.472	-0.46044	-0.2963
40.488	-0.46049	-0.29639
40.505	-0.46054	-0.29647

40.521	-0.4606	-0.29653
40.537	-0.46068	-0.2966
40.554	-0.46072	-0.2967
40.571	-0.46076	-0.2968
40.587	-0.46081	-0.29693
40.604	-0.46085	-0.29702
40.62	-0.46094	-0.2971
40.637	-0.46109	-0.29715
40.654	-0.4612	-0.29722
40.67	-0.46131	-0.29729
40.686	-0.46138	-0.29739
40.704	-0.46147	-0.29749
40.719	-0.46156	-0.29759
40.736	-0.46164	-0.29771
40.753	-0.4617	-0.29788
40.769	-0.46174	-0.29805
40.786	-0.4618	-0.29816
40.802	-0.46187	-0.29827
40.818	-0.46199	-0.29838
40.836	-0.46208	-0.29856
40.852	-0.46214	-0.29873
40.869	-0.46219	-0.29887
40.885	-0.46226	-0.29899
40.901	-0.46234	-0.29912
40.918	-0.4624	-0.29919
40.936	-0.46246	-0.29927
40.951	-0.46252	-0.29935
40.969	-0.46256	-0.2994
40.985	-0.46261	-0.2995
41	-0.46268	-0.29958

41.018	-0.46273	-0.29969
41.034	-0.46278	-0.29982
41.051	-0.46284	-0.29992
41.068	-0.46292	-0.30002
41.083	-0.46303	-0.3001
41.1	-0.46309	-0.30018
41.117	-0.46315	-0.30028
41.134	-0.46321	-0.30039
41.15	-0.46325	-0.30048
41.166	-0.4633	-0.30054
41.184	-0.46337	-0.30062
41.2	-0.46341	-0.30069
41.217	-0.46345	-0.30077
41.233	-0.4635	-0.30084
41.25	-0.46354	-0.30093
41.266	-0.46363	-0.301
41.282	-0.46376	-0.30108
41.299	-0.46392	-0.30117
41.316	-0.46416	-0.30127
41.333	-0.46436	-0.30141
41.349	-0.46451	-0.30151
41.365	-0.46463	-0.30162
41.382	-0.46471	-0.30172
41.398	-0.46477	-0.30184
41.415	-0.46482	-0.30194
41.432	-0.46487	-0.30205
41.448	-0.46494	-0.30214
41.464	-0.46499	-0.30224
41.481	-0.46506	-0.30236
41.498	-0.46511	-0.30246

41.514	-0.46515	-0.30257
41.531	-0.4652	-0.30271
41.547	-0.46528	-0.30284
41.564	-0.46532	-0.30295
41.58	-0.46536	-0.30307
41.597	-0.46541	-0.30319
41.613	-0.46548	-0.30331
41.63	-0.46555	-0.30342
41.647	-0.46565	-0.30353
41.663	-0.46574	-0.30364
41.68	-0.46583	-0.30371
41.696	-0.4659	-0.3038
41.712	-0.46594	-0.3039
41.729	-0.46598	-0.30399
41.745	-0.46602	-0.30408
41.762	-0.46607	-0.30416
41.779	-0.46611	-0.30423
41.796	-0.46616	-0.30431
41.813	-0.46621	-0.3044
41.829	-0.46626	-0.30453
41.845	-0.46634	-0.30466
41.862	-0.46644	-0.30479
41.879	-0.46658	-0.30496
41.895	-0.46669	-0.30515
41.912	-0.4668	-0.30537
41.929	-0.4669	-0.30556
41.945	-0.46698	-0.30574
41.962	-0.46707	-0.30586
41.978	-0.46716	-0.30596
41.995	-0.46721	-0.30607

42.011	-0.46725	-0.30621
42.027	-0.4673	-0.30633
42.045	-0.46737	-0.3064
42.061	-0.46746	-0.30647
42.077	-0.46753	-0.30654
42.094	-0.46759	-0.30663
42.11	-0.46766	-0.30668
42.127	-0.46773	-0.30672
42.144	-0.46777	-0.30676
42.16	-0.46781	-0.3068
42.177	-0.46786	-0.30687
42.194	-0.4679	-0.30698
42.21	-0.46797	-0.3071
42.227	-0.46803	-0.30724
42.243	-0.46808	-0.30738
42.26	-0.46812	-0.30755
42.276	-0.46817	-0.30774
42.293	-0.46821	-0.30791
42.309	-0.46827	-0.30805
42.326	-0.46831	-0.30813
42.343	-0.46836	-0.3082
42.361	-0.4684	-0.30826
42.377	-0.46845	-0.30833
42.393	-0.46849	-0.30843
42.409	-0.46855	-0.30854
42.426	-0.46864	-0.30864
42.443	-0.46876	-0.30875
42.459	-0.46887	-0.30884
42.476	-0.46896	-0.30892
42.492	-0.46903	-0.30899

42.509	-0.46908	-0.30905
42.525	-0.46913	-0.3091
42.542	-0.46917	-0.30914
42.558	-0.46921	-0.30918
42.574	-0.46926	-0.30923
42.591	-0.46931	-0.30931
42.608	-0.46935	-0.30941
42.625	-0.46942	-0.30953
42.641	-0.46952	-0.30961
42.657	-0.46959	-0.30972
42.673	-0.46965	-0.30985
42.691	-0.4697	-0.30996
42.708	-0.46975	-0.31011
42.724	-0.46979	-0.31026
42.74	-0.46984	-0.31039
42.757	-0.46991	-0.31047
42.773	-0.46997	-0.31055
42.79	-0.47002	-0.31061
42.806	-0.47008	-0.31066
42.823	-0.47018	-0.31071
42.84	-0.47027	-0.31076
42.856	-0.47035	-0.31083
42.872	-0.47041	-0.31094
42.89	-0.47046	-0.31107
42.906	-0.4705	-0.31123
42.922	-0.47055	-0.31137
42.939	-0.47059	-0.31151
42.955	-0.47064	-0.31164
42.972	-0.47069	-0.31178
42.989	-0.47073	-0.31188

43.005	-0.47077	-0.31197
43.023	-0.47082	-0.31206
43.038	-0.47091	-0.31214
43.055	-0.47101	-0.31222
43.072	-0.47109	-0.31231
43.088	-0.47114	-0.31242
43.105	-0.4712	-0.31255
43.121	-0.47127	-0.31267
43.139	-0.47135	-0.31276
43.156	-0.4714	-0.31285
43.172	-0.47146	-0.31296
43.189	-0.47151	-0.31308
43.205	-0.47155	-0.31323
43.221	-0.4716	-0.31338
43.237	-0.47165	-0.31349
43.254	-0.4717	-0.31358
43.27	-0.47175	-0.31369
43.288	-0.4718	-0.31381
43.303	-0.47185	-0.31395
43.32	-0.4719	-0.31407
43.337	-0.47196	-0.31418
43.354	-0.47201	-0.31431
43.371	-0.47206	-0.31442
43.387	-0.47211	-0.31457
43.405	-0.47216	-0.31469
43.42	-0.47221	-0.31479
43.437	-0.47226	-0.31488
43.453	-0.4723	-0.31497
43.47	-0.47236	-0.31505
43.487	-0.47241	-0.31515

43.503	-0.47246	-0.31523
43.519	-0.47251	-0.31531
43.536	-0.47256	-0.31543
43.553	-0.47261	-0.31554
43.569	-0.47266	-0.31567
43.586	-0.47271	-0.3158
43.603	-0.47275	-0.31592
43.619	-0.4728	-0.31601
43.636	-0.47285	-0.31609
43.652	-0.4729	-0.31616
43.67	-0.47295	-0.31628
43.686	-0.473	-0.31637
43.703	-0.47304	-0.31646
43.72	-0.47309	-0.31656
43.736	-0.47314	-0.31665
43.753	-0.47319	-0.31675
43.768	-0.47325	-0.31683
43.785	-0.47333	-0.31691
43.802	-0.4734	-0.31698
43.819	-0.4735	-0.31704
43.836	-0.47356	-0.31713
43.852	-0.47361	-0.31725
43.869	-0.47365	-0.31737
43.884	-0.4737	-0.31749
43.901	-0.47375	-0.31761
43.917	-0.4738	-0.31772
43.934	-0.47385	-0.31784
43.951	-0.47392	-0.31792
43.968	-0.47399	-0.318
43.984	-0.47409	-0.31807

44.001	-0.47424	-0.31812
44.017	-0.47438	-0.31818
44.034	-0.47444	-0.31826
44.05	-0.4745	-0.31836
44.067	-0.47455	-0.31851
44.084	-0.4746	-0.31871
44.101	-0.47465	-0.31889
44.117	-0.4747	-0.31901
44.133	-0.47474	-0.31911
44.151	-0.47479	-0.31925
44.167	-0.47484	-0.31936
44.183	-0.47489	-0.31945
44.2	-0.47494	-0.31953
44.216	-0.47499	-0.31959
44.233	-0.47505	-0.31964
44.25	-0.47511	-0.31973
44.265	-0.47516	-0.3198
44.282	-0.47521	-0.31988
44.299	-0.47526	-0.31997
44.315	-0.47531	-0.32005
44.332	-0.47536	-0.32012
44.349	-0.47541	-0.32021
44.366	-0.47546	-0.3203
44.382	-0.47551	-0.32041
44.4	-0.47556	-0.3205
44.416	-0.47564	-0.32059
44.432	-0.47572	-0.32071
44.448	-0.4758	-0.32082
44.466	-0.47589	-0.32097
44.481	-0.47595	-0.32111

44.498	-0.476	-0.32128
44.515	-0.47605	-0.32144
44.531	-0.4761	-0.32157
44.548	-0.47615	-0.32169
44.565	-0.4762	-0.32179
44.581	-0.47625	-0.32187
44.598	-0.4763	-0.32192
44.614	-0.47635	-0.32197
44.631	-0.4764	-0.32204
44.648	-0.47645	-0.3221
44.664	-0.47655	-0.32219
44.681	-0.47662	-0.32229
44.697	-0.47667	-0.32244
44.714	-0.47672	-0.32259
44.731	-0.47679	-0.32271
44.747	-0.47693	-0.32286
44.765	-0.47709	-0.32298
44.78	-0.47719	-0.32308
44.798	-0.47727	-0.32318
44.814	-0.47734	-0.3233
44.831	-0.47739	-0.32341
44.847	-0.47744	-0.32353
44.864	-0.47749	-0.32364
44.88	-0.47754	-0.32374
44.897	-0.47761	-0.32384
44.913	-0.47766	-0.32392
44.93	-0.47771	-0.32401
44.947	-0.47775	-0.32408
44.963	-0.4778	-0.32417
44.979	-0.47785	-0.32424

44.996	-0.4779	-0.32433
45.013	-0.47795	-0.32442
45.03	-0.478	-0.32453
45.046	-0.47805	-0.32462
45.062	-0.4781	-0.32472
45.08	-0.47815	-0.32481
45.095	-0.4782	-0.32489
45.112	-0.47825	-0.32496
45.128	-0.4783	-0.32502
45.146	-0.47835	-0.32508
45.162	-0.47843	-0.32515
45.18	-0.47851	-0.32526
45.196	-0.47859	-0.32538
45.212	-0.47867	-0.32551
45.228	-0.47873	-0.32562
45.245	-0.47878	-0.32576
45.262	-0.47884	-0.32591
45.278	-0.47889	-0.32608
45.295	-0.47893	-0.32625
45.311	-0.47898	-0.32639
45.328	-0.47902	-0.32651
45.345	-0.47907	-0.32661
45.361	-0.47912	-0.32673
45.377	-0.47916	-0.32686
45.393	-0.47921	-0.32699
45.411	-0.47926	-0.3271
45.427	-0.47931	-0.32722
45.443	-0.47935	-0.32732
45.46	-0.4794	-0.3274
45.477	-0.47945	-0.32747

45.493	-0.4795	-0.32753
45.511	-0.47955	-0.32761
45.527	-0.4796	-0.32769
45.543	-0.47965	-0.32777
45.56	-0.4797	-0.32787
45.578	-0.47974	-0.32795
45.593	-0.47979	-0.32802
45.609	-0.47984	-0.32808
45.628	-0.4799	-0.32814
45.644	-0.47997	-0.3282
45.66	-0.48003	-0.32826
45.677	-0.48009	-0.32832
45.693	-0.48017	-0.32841
45.71	-0.48023	-0.32852
45.727	-0.4803	-0.32867
45.743	-0.48035	-0.32882
45.759	-0.48039	-0.32898
45.776	-0.48044	-0.32913
45.792	-0.48049	-0.32928
45.809	-0.48054	-0.32942
45.826	-0.48058	-0.32958
45.843	-0.48063	-0.32969
45.86	-0.48067	-0.32977
45.876	-0.48072	-0.32983
45.891	-0.48076	-0.32988
45.909	-0.48081	-0.32996
45.925	-0.48086	-0.33004
45.942	-0.48092	-0.33011
45.959	-0.48099	-0.33021
45.975	-0.48104	-0.33033

45.991	-0.48109	-0.33045
46.008	-0.48114	-0.33059
46.024	-0.48119	-0.33071
46.041	-0.48124	-0.33081
46.058	-0.48129	-0.33089
46.075	-0.48134	-0.33096
46.092	-0.48139	-0.33109
46.108	-0.48144	-0.33124
46.125	-0.4815	-0.33142
46.142	-0.48155	-0.33157
46.158	-0.4816	-0.33175
46.175	-0.48165	-0.33192
46.191	-0.4817	-0.33208
46.208	-0.48177	-0.33225
46.224	-0.48182	-0.33234
46.241	-0.48187	-0.33244
46.257	-0.48192	-0.33251
46.274	-0.48197	-0.33259
46.291	-0.48201	-0.33268
46.307	-0.48206	-0.33278
46.325	-0.48211	-0.33288
46.341	-0.48216	-0.33298
46.357	-0.48221	-0.33308
46.374	-0.48226	-0.33318
46.391	-0.48231	-0.33329
46.407	-0.48236	-0.33339
46.424	-0.48241	-0.33346
46.44	-0.48247	-0.33353
46.457	-0.48252	-0.3336
46.474	-0.48257	-0.33368

46.49	-0.48261	-0.33376
46.507	-0.48266	-0.33385
46.524	-0.4827	-0.33396
46.54	-0.48275	-0.33404
46.556	-0.4828	-0.33412
46.573	-0.48284	-0.33422
46.59	-0.48289	-0.33432
46.606	-0.48294	-0.33439
46.622	-0.48301	-0.33445
46.639	-0.48312	-0.33453
46.655	-0.4832	-0.33459
46.672	-0.48326	-0.33466
46.688	-0.48333	-0.33474
46.705	-0.48338	-0.33483
46.722	-0.48343	-0.33491
46.738	-0.48348	-0.33499
46.755	-0.48352	-0.3351
46.772	-0.48357	-0.3352
46.789	-0.48361	-0.33529
46.805	-0.48365	-0.3354
46.822	-0.48369	-0.33551
46.839	-0.48374	-0.33565
46.855	-0.48378	-0.33579
46.871	-0.48383	-0.33596
46.889	-0.48388	-0.33612
46.904	-0.48397	-0.33627
46.921	-0.48405	-0.3364
46.938	-0.4841	-0.33652
46.954	-0.48416	-0.3366
46.97	-0.48421	-0.33668

46.988	-0.48426	-0.33677
47.004	-0.48431	-0.33686
47.021	-0.48435	-0.33693
47.038	-0.4844	-0.337
47.054	-0.48445	-0.33708
47.071	-0.48449	-0.33716
47.089	-0.48454	-0.33729
47.105	-0.48459	-0.33738
47.122	-0.48465	-0.33748
47.138	-0.48472	-0.33756
47.154	-0.48483	-0.33764
47.171	-0.48495	-0.33771
47.187	-0.48501	-0.33781
47.204	-0.48506	-0.33791
47.221	-0.48511	-0.33799
47.237	-0.48516	-0.33808
47.254	-0.4852	-0.33818
47.271	-0.48525	-0.3383
47.287	-0.48529	-0.33843
47.304	-0.48534	-0.33859
47.321	-0.48538	-0.33875
47.337	-0.48543	-0.33898
47.354	-0.48547	-0.33917
47.37	-0.48552	-0.33936
47.387	-0.48557	-0.33951
47.404	-0.48562	-0.33962
47.42	-0.48567	-0.33969
47.437	-0.48572	-0.33975
47.452	-0.48577	-0.33981
47.469	-0.48582	-0.33985

47.486	-0.48586	-0.33991
47.502	-0.48591	-0.33998
47.519	-0.48596	-0.34005
47.536	-0.48601	-0.34012
47.552	-0.48607	-0.34018
47.57	-0.48613	-0.34025
47.586	-0.48618	-0.3403
47.602	-0.48623	-0.34037
47.62	-0.48627	-0.34045
47.636	-0.48632	-0.34059
47.653	-0.48636	-0.3407
47.669	-0.48641	-0.34087
47.686	-0.48646	-0.34103
47.702	-0.48651	-0.34117
47.719	-0.48655	-0.34131
47.735	-0.4866	-0.34148
47.753	-0.48665	-0.34164
47.769	-0.4867	-0.3418
47.785	-0.48674	-0.34191
47.802	-0.48679	-0.34202
47.819	-0.48683	-0.34213
47.836	-0.48688	-0.3422
47.852	-0.48693	-0.34227
47.869	-0.48697	-0.34234
47.885	-0.48702	-0.3424
47.902	-0.48706	-0.34246
47.918	-0.48711	-0.34253
47.935	-0.48715	-0.3426
47.951	-0.48719	-0.34266
47.968	-0.48723	-0.34272

47.984	-0.48727	-0.3428
48.001	-0.48731	-0.34287
48.017	-0.48736	-0.34294
48.034	-0.48741	-0.34299
48.05	-0.48746	-0.34307
48.067	-0.48751	-0.34317
48.083	-0.48755	-0.34326
48.1	-0.48762	-0.34337
48.116	-0.48771	-0.34346
48.133	-0.48782	-0.34357
48.15	-0.48792	-0.3437
48.166	-0.48799	-0.34385
48.182	-0.48804	-0.34398
48.199	-0.48809	-0.34413
48.216	-0.48814	-0.34425
48.233	-0.48818	-0.34437
48.25	-0.48823	-0.34452
48.266	-0.48827	-0.34462
48.283	-0.48831	-0.34469
48.299	-0.48835	-0.34477
48.315	-0.48839	-0.34485
48.333	-0.48843	-0.34492
48.349	-0.48848	-0.34501
48.365	-0.48853	-0.34509
48.383	-0.48859	-0.34517
48.399	-0.48867	-0.34522
48.415	-0.48873	-0.34529
48.433	-0.48881	-0.34538
48.449	-0.48887	-0.34549
48.466	-0.48893	-0.34562

48.482	-0.48898	-0.34573
48.5	-0.48902	-0.34585
48.515	-0.48906	-0.34602
48.533	-0.48911	-0.34622
48.548	-0.48915	-0.34636
48.565	-0.48919	-0.34649
48.582	-0.48924	-0.34658
48.599	-0.48928	-0.34668
48.615	-0.48932	-0.34682
48.631	-0.48937	-0.34694
48.649	-0.48941	-0.34704
48.665	-0.48946	-0.34713
48.682	-0.48951	-0.34721
48.699	-0.48955	-0.34729
48.714	-0.4896	-0.34739
48.731	-0.48965	-0.34749
48.748	-0.48969	-0.34758
48.764	-0.48975	-0.34767
48.782	-0.48979	-0.34778
48.798	-0.48984	-0.34794
48.815	-0.48989	-0.34807
48.832	-0.48994	-0.34821
48.848	-0.48998	-0.34836
48.865	-0.49003	-0.34852
48.882	-0.49007	-0.34869
48.898	-0.49012	-0.34882
48.915	-0.49016	-0.34894
48.931	-0.49021	-0.34904
48.948	-0.49026	-0.3491
48.964	-0.4903	-0.34917

48.981	-0.49035	-0.34924
48.998	-0.49041	-0.34932
49.014	-0.49045	-0.34941
49.031	-0.4905	-0.34949
49.047	-0.49055	-0.3496
49.063	-0.49059	-0.34973
49.081	-0.49063	-0.34985
49.097	-0.49068	-0.35001
49.115	-0.49072	-0.35017
49.131	-0.49076	-0.35034
49.148	-0.49081	-0.3505
49.164	-0.49086	-0.35062
49.18	-0.4909	-0.35073
49.197	-0.49095	-0.35081
49.213	-0.491	-0.35088
49.231	-0.49104	-0.35093
49.247	-0.49109	-0.35096
49.263	-0.49113	-0.351
49.28	-0.49117	-0.35104
49.297	-0.49122	-0.35107
49.313	-0.49126	-0.3511
49.33	-0.4913	-0.35116
49.346	-0.49135	-0.35122
49.363	-0.49139	-0.3513
49.379	-0.49144	-0.35138
49.396	-0.49148	-0.35146
49.413	-0.49152	-0.35152
49.429	-0.49156	-0.35159
49.446	-0.4916	-0.35167
49.464	-0.49164	-0.35175

49.48	-0.49168	-0.35181
49.496	-0.49172	-0.35187
49.513	-0.49176	-0.35194
49.53	-0.4918	-0.35203
49.546	-0.49184	-0.35213
49.562	-0.49189	-0.35221
49.579	-0.49194	-0.35229
49.595	-0.49199	-0.35237
49.612	-0.49203	-0.35246
49.628	-0.49207	-0.35255
49.646	-0.49211	-0.35265
49.662	-0.49215	-0.35274
49.679	-0.49219	-0.35283
49.695	-0.49223	-0.35292
49.711	-0.49227	-0.35302
49.728	-0.4923	-0.35313
49.745	-0.49234	-0.35323
49.761	-0.49239	-0.35332
49.778	-0.49243	-0.35342
49.795	-0.49248	-0.3535
49.811	-0.49252	-0.35358
49.827	-0.49256	-0.35365
49.845	-0.49261	-0.35374
49.862	-0.49265	-0.35385
49.878	-0.49269	-0.35401
49.894	-0.49273	-0.35413
49.911	-0.49277	-0.35425
49.928	-0.49281	-0.35438
49.944	-0.49285	-0.35449
49.962	-0.4929	-0.35456

49.978	-0.49294	-0.3546
49.994	-0.49299	-0.35467
50.011	-0.49303	-0.35475
50.026	-0.49308	-0.35481
50.043	-0.49312	-0.35487
50.061	-0.49317	-0.35493
50.077	-0.49321	-0.35503
50.094	-0.49325	-0.35513
50.111	-0.49329	-0.35524
50.127	-0.49333	-0.35531
50.143	-0.49337	-0.35539
50.16	-0.49341	-0.3555
50.176	-0.49345	-0.35565
50.194	-0.49349	-0.35577
50.21	-0.49354	-0.35588
50.226	-0.49358	-0.35598
50.244	-0.49362	-0.35606
50.261	-0.49366	-0.35614
50.278	-0.49371	-0.35621
50.294	-0.49375	-0.35628
50.31	-0.4938	-0.35637
50.327	-0.49384	-0.35646
50.344	-0.49388	-0.35661
50.359	-0.49393	-0.35677
50.376	-0.49397	-0.35692
50.393	-0.49401	-0.35707
50.41	-0.49405	-0.35718
50.427	-0.49409	-0.3573
50.443	-0.49413	-0.3574
50.46	-0.49417	-0.3575

50.476	-0.49421	-0.35757
50.493	-0.49425	-0.35766
50.509	-0.49429	-0.35776
50.526	-0.49433	-0.35789
50.542	-0.49437	-0.35802
50.559	-0.49441	-0.35816
50.576	-0.49446	-0.35828
50.592	-0.4945	-0.35841
50.61	-0.49454	-0.35853
50.626	-0.49458	-0.35862
50.642	-0.49461	-0.35868
50.659	-0.49465	-0.35873
50.676	-0.49469	-0.35877
50.693	-0.49472	-0.35882
50.71	-0.49476	-0.35886
50.725	-0.4948	-0.35893
50.743	-0.49484	-0.35897
50.758	-0.49488	-0.35901
50.775	-0.49492	-0.35906
50.792	-0.49496	-0.35913
50.808	-0.495	-0.35921
50.824	-0.49503	-0.35932
50.842	-0.49507	-0.3594
50.858	-0.4951	-0.35951
50.875	-0.49514	-0.35963
50.891	-0.49517	-0.35977
50.907	-0.49521	-0.35991
50.925	-0.49525	-0.36003
50.942	-0.49529	-0.36012
50.958	-0.49534	-0.36023

50.975	-0.49539	-0.36033
50.991	-0.49543	-0.36044
51.008	-0.49546	-0.36058
51.024	-0.4955	-0.36068
51.041	-0.49554	-0.3608
51.058	-0.49557	-0.36089
51.075	-0.49561	-0.36098
51.091	-0.49564	-0.36107
51.108	-0.49568	-0.36112
51.124	-0.49572	-0.36115
51.14	-0.49575	-0.36117
51.157	-0.49579	-0.36119
51.174	-0.49583	-0.36121
51.19	-0.49587	-0.36123
51.207	-0.4959	-0.36128
51.223	-0.49594	-0.36137
51.24	-0.49598	-0.3615
51.257	-0.49601	-0.36166
51.273	-0.49605	-0.36187
51.29	-0.49609	-0.36204
51.307	-0.49613	-0.36218
51.323	-0.49617	-0.36234
51.34	-0.49621	-0.36247
51.356	-0.49625	-0.36259
51.373	-0.49629	-0.36267
51.389	-0.49633	-0.36275
51.406	-0.49637	-0.36285
51.423	-0.4964	-0.36296
51.44	-0.49644	-0.36307
51.456	-0.49648	-0.36319

51.472	-0.49651	-0.36334
51.489	-0.49655	-0.36346
51.506	-0.49659	-0.36354
51.522	-0.49663	-0.36362
51.54	-0.49667	-0.36371
51.555	-0.49671	-0.36378
51.572	-0.49675	-0.36384
51.589	-0.49679	-0.36391
51.607	-0.49683	-0.36398
51.623	-0.49689	-0.36405
51.64	-0.49693	-0.36412
51.656	-0.49697	-0.36417
51.672	-0.497	-0.36422
51.688	-0.49704	-0.36429
51.705	-0.49707	-0.36437
51.721	-0.49711	-0.36447
51.738	-0.49715	-0.36454
51.755	-0.49718	-0.36468
51.772	-0.49722	-0.36487
51.788	-0.49727	-0.36509
51.805	-0.49733	-0.36529
51.822	-0.49737	-0.36542
51.839	-0.4974	-0.3655
51.854	-0.49745	-0.36557
51.871	-0.49749	-0.36563
51.889	-0.49752	-0.36569
51.905	-0.49756	-0.36578
51.921	-0.4976	-0.36587
51.937	-0.49763	-0.36597
51.955	-0.49767	-0.36607

51.971	-0.49771	-0.36617
51.988	-0.49775	-0.36627
52.004	-0.49779	-0.3664
52.021	-0.49783	-0.36647
52.037	-0.49788	-0.36655
52.054	-0.49793	-0.36661
52.071	-0.49797	-0.36667
52.087	-0.49801	-0.36673
52.104	-0.49804	-0.36682
52.121	-0.49808	-0.3669
52.137	-0.49812	-0.36704
52.155	-0.49815	-0.36715
52.17	-0.49818	-0.36726
52.187	-0.49822	-0.36737
52.204	-0.49826	-0.36749
52.221	-0.49829	-0.3676
52.237	-0.49833	-0.36771
52.254	-0.49837	-0.36779
52.271	-0.4984	-0.36784
52.287	-0.49844	-0.3679
52.303	-0.49848	-0.36795
52.321	-0.49852	-0.36804
52.337	-0.49856	-0.36814
52.353	-0.4986	-0.36828
52.37	-0.49864	-0.36846
52.386	-0.49868	-0.36865
52.404	-0.49872	-0.36885
52.421	-0.49875	-0.36899
52.437	-0.49878	-0.36911
52.453	-0.49882	-0.36921

52.47	-0.49885	-0.36927
52.487	-0.49888	-0.36932
52.503	-0.49892	-0.36937
52.52	-0.49896	-0.36941
52.537	-0.499	-0.36947
52.554	-0.49904	-0.36952
52.57	-0.49907	-0.36958
52.586	-0.49911	-0.36964
52.603	-0.49915	-0.36969
52.62	-0.49919	-0.36976
52.637	-0.49922	-0.36982
52.653	-0.49926	-0.36989
52.67	-0.4993	-0.36998
52.686	-0.49933	-0.37007
52.703	-0.49937	-0.37016
52.719	-0.4994	-0.37028
52.736	-0.49944	-0.37037
52.753	-0.49947	-0.37049
52.769	-0.49951	-0.37056
52.786	-0.49954	-0.37063
52.803	-0.49958	-0.37071
52.819	-0.49961	-0.37078
52.836	-0.49965	-0.37086
52.852	-0.49968	-0.37096
52.87	-0.49972	-0.37105
52.886	-0.49975	-0.37112
52.902	-0.49979	-0.3712
52.92	-0.49983	-0.37126
52.936	-0.49986	-0.37134
52.951	-0.4999	-0.3714

52.969	-0.49993	-0.37144
52.984	-0.49997	-0.37147
53.002	-0.5	-0.37152
53.018	-0.50004	-0.37159
53.034	-0.50007	-0.37166
53.052	-0.50011	-0.37176
53.068	-0.50015	-0.37189
53.085	-0.50018	-0.37206
53.101	-0.50022	-0.37223
53.117	-0.50025	-0.37238
53.134	-0.50029	-0.37251
53.152	-0.50032	-0.37265
53.168	-0.50035	-0.37272
53.185	-0.50039	-0.3728
53.201	-0.50042	-0.37287
53.218	-0.50045	-0.37293
53.234	-0.50049	-0.37299
53.252	-0.50052	-0.37306
53.268	-0.50056	-0.37314
53.284	-0.5006	-0.37325
53.3	-0.50063	-0.37336
53.317	-0.50067	-0.37348
53.335	-0.50071	-0.37358
53.351	-0.50074	-0.37367
53.368	-0.50078	-0.37376
53.384	-0.50081	-0.37384
53.4	-0.50084	-0.3739
53.418	-0.50088	-0.37395
53.434	-0.50091	-0.37399
53.45	-0.50094	-0.37403

53.467	-0.50098	-0.37408
53.483	-0.50101	-0.37413
53.501	-0.50104	-0.37419
53.517	-0.50108	-0.37423
53.533	-0.50111	-0.37429
53.55	-0.50115	-0.37436
53.567	-0.50118	-0.37444
53.583	-0.50121	-0.37453
53.6	-0.50125	-0.37463
53.617	-0.50128	-0.37475
53.633	-0.50131	-0.37487
53.65	-0.50135	-0.37503
53.667	-0.50138	-0.3752
53.682	-0.50141	-0.37538
53.7	-0.50145	-0.37549
53.715	-0.50148	-0.3756
53.732	-0.50152	-0.3757
53.749	-0.50155	-0.37579
53.766	-0.50159	-0.3759
53.783	-0.50162	-0.376
53.799	-0.50166	-0.37607
53.816	-0.50169	-0.37613
53.832	-0.50172	-0.37619
53.85	-0.50175	-0.37627
53.866	-0.50178	-0.37636
53.882	-0.50181	-0.37644
53.899	-0.50184	-0.37652
53.916	-0.50188	-0.37665
53.932	-0.50191	-0.37679
53.949	-0.50194	-0.37694

53.965	-0.50198	-0.37706
53.982	-0.50201	-0.37717
53.999	-0.50204	-0.37726
54.015	-0.50208	-0.37735
54.032	-0.50211	-0.37743
54.049	-0.50214	-0.37752
54.065	-0.50218	-0.37757
54.081	-0.50221	-0.37761
54.098	-0.50224	-0.37767
54.115	-0.50227	-0.37774
54.132	-0.50231	-0.37783
54.148	-0.50234	-0.37794
54.165	-0.50237	-0.37806
54.181	-0.5024	-0.37818
54.198	-0.50243	-0.3783
54.214	-0.50245	-0.3784
54.231	-0.50248	-0.37848
54.248	-0.50251	-0.37853
54.265	-0.50253	-0.37857
54.282	-0.50256	-0.37861
54.298	-0.50259	-0.37864
54.313	-0.50262	-0.37867
54.331	-0.50265	-0.37874
54.348	-0.50268	-0.37881
54.364	-0.50271	-0.37892
54.38	-0.50274	-0.37906
54.396	-0.50277	-0.37923
54.413	-0.5028	-0.37934
54.431	-0.50283	-0.37947
54.448	-0.50286	-0.3796

54.464	-0.50289	-0.37971
54.48	-0.50292	-0.37979
54.497	-0.50295	-0.37986
54.514	-0.50299	-0.37992
54.53	-0.50302	-0.37997
54.547	-0.50306	-0.38001
54.563	-0.50309	-0.38007
54.58	-0.50313	-0.3801
54.596	-0.50316	-0.38015
54.613	-0.50319	-0.38022
54.629	-0.50321	-0.3803
54.646	-0.50323	-0.38036
54.662	-0.50326	-0.38044
54.68	-0.50328	-0.38054
54.697	-0.50331	-0.38066
54.712	-0.50334	-0.38077
54.729	-0.50337	-0.38089
54.747	-0.5034	-0.38101
54.763	-0.50343	-0.3811
54.779	-0.50347	-0.3812
54.796	-0.5035	-0.38131
54.814	-0.50353	-0.3814
54.83	-0.50356	-0.38146
54.846	-0.50359	-0.3815
54.863	-0.50362	-0.38154
54.879	-0.50365	-0.38156
54.895	-0.50368	-0.38159
54.914	-0.50371	-0.38165
54.929	-0.50375	-0.38173
54.945	-0.50378	-0.38181

54.962	-0.50381	-0.38189
54.979	-0.50384	-0.38195
54.996	-0.50386	-0.38201
55.013	-0.50389	-0.38211
55.029	-0.50392	-0.3822
55.045	-0.50394	-0.38228
55.062	-0.50397	-0.38236
55.078	-0.504	-0.38246
55.095	-0.50403	-0.38257
55.113	-0.50406	-0.3827
55.128	-0.50409	-0.38285
55.146	-0.50412	-0.38301
55.162	-0.50414	-0.3831
55.178	-0.50417	-0.38317
55.196	-0.5042	-0.38326
55.212	-0.50422	-0.38334
55.229	-0.50425	-0.38341
55.245	-0.50428	-0.38347
55.262	-0.50431	-0.38353
55.279	-0.50433	-0.38359
55.295	-0.50436	-0.38363
55.312	-0.50438	-0.38368
55.328	-0.50441	-0.38373
55.345	-0.50443	-0.38377
55.362	-0.50446	-0.38382
55.377	-0.50448	-0.38388
55.394	-0.5045	-0.38397
55.41	-0.50453	-0.38407
55.428	-0.50455	-0.38417
55.444	-0.50458	-0.3843

55.462	-0.50461	-0.38445
55.479	-0.50464	-0.38459
55.495	-0.50467	-0.38475
55.512	-0.50472	-0.38486
55.527	-0.50476	-0.38496
55.545	-0.50481	-0.38505
55.562	-0.50485	-0.38514
55.578	-0.50488	-0.38521
55.595	-0.50491	-0.38529
55.611	-0.50494	-0.38536
55.628	-0.50496	-0.38541
55.645	-0.50499	-0.38546
55.661	-0.50501	-0.38552
55.678	-0.50504	-0.38559
55.694	-0.50507	-0.38565
55.711	-0.50509	-0.3857
55.727	-0.50512	-0.38577
55.744	-0.50515	-0.38581
55.761	-0.50517	-0.38585
55.777	-0.5052	-0.38591
55.793	-0.50522	-0.38597
55.81	-0.50524	-0.38605
55.827	-0.50526	-0.38617
55.843	-0.50528	-0.38628
55.86	-0.50531	-0.3864
55.877	-0.50533	-0.38649
55.894	-0.50536	-0.38656
55.91	-0.50539	-0.38661
55.927	-0.50542	-0.38666
55.944	-0.50545	-0.38671

55.96	-0.50548	-0.38674
55.976	-0.50552	-0.38679
55.993	-0.50555	-0.38685
56.01	-0.50558	-0.38693
56.028	-0.50561	-0.38703
56.044	-0.50563	-0.38717
56.06	-0.50566	-0.38729
56.077	-0.50568	-0.3874
56.094	-0.5057	-0.3875
56.111	-0.50572	-0.38759
56.127	-0.50574	-0.38765
56.144	-0.50576	-0.38773
56.16	-0.50579	-0.38782
56.177	-0.50581	-0.38789
56.193	-0.50584	-0.38796
56.21	-0.50586	-0.38802
56.227	-0.50589	-0.38809
56.244	-0.50591	-0.38817
56.26	-0.50594	-0.38829
56.276	-0.50597	-0.38835
56.293	-0.50599	-0.3884
56.309	-0.50602	-0.38845
56.326	-0.50605	-0.3885
56.343	-0.50608	-0.38856
56.36	-0.5061	-0.38859
56.375	-0.50613	-0.38863
56.392	-0.50615	-0.38867
56.409	-0.50617	-0.38873
56.426	-0.50619	-0.38881
56.442	-0.50621	-0.3889

56.459	-0.50623	-0.38897
56.475	-0.50625	-0.38903
56.492	-0.50628	-0.38912
56.509	-0.5063	-0.38923
56.526	-0.50633	-0.38933
56.542	-0.50635	-0.3894
56.559	-0.50638	-0.38947
56.576	-0.50641	-0.38957
56.592	-0.50644	-0.38966
56.608	-0.50646	-0.38976
56.625	-0.50649	-0.38984
56.643	-0.50651	-0.38991
56.659	-0.50653	-0.38994
56.675	-0.50655	-0.38997
56.692	-0.50658	-0.38999
56.708	-0.5066	-0.39002
56.725	-0.50662	-0.39005
56.742	-0.50664	-0.39008
56.758	-0.50666	-0.39011
56.775	-0.50668	-0.39016
56.791	-0.5067	-0.39022
56.808	-0.50673	-0.3903
56.825	-0.50675	-0.39039
56.841	-0.50677	-0.3905
56.858	-0.5068	-0.39061
56.874	-0.50682	-0.3907
56.891	-0.50684	-0.39079
56.909	-0.50687	-0.39086
56.924	-0.50689	-0.39095
56.942	-0.50691	-0.39103

56.958	-0.50694	-0.39114
56.975	-0.50696	-0.39121
56.991	-0.50698	-0.39127
57.009	-0.507	-0.39132
57.024	-0.50702	-0.39135
57.041	-0.50705	-0.39138
57.057	-0.50707	-0.39143
57.074	-0.5071	-0.39148
57.091	-0.50712	-0.39153
57.108	-0.50714	-0.39163
57.124	-0.50716	-0.39176
57.141	-0.50719	-0.39189
57.158	-0.50721	-0.39203
57.174	-0.50723	-0.39214
57.191	-0.50726	-0.39225
57.207	-0.50728	-0.39232
57.224	-0.5073	-0.39237
57.241	-0.50733	-0.39242
57.258	-0.50735	-0.39246
57.274	-0.50738	-0.39249
57.291	-0.5074	-0.39254
57.307	-0.50741	-0.39259
57.323	-0.50743	-0.39264
57.34	-0.50745	-0.3927
57.358	-0.50747	-0.39277
57.374	-0.50749	-0.39286
57.391	-0.50751	-0.39295
57.407	-0.50753	-0.39301
57.424	-0.50755	-0.39308
57.441	-0.50757	-0.39315

57.458	-0.50759	-0.39324
57.474	-0.50761	-0.39333
57.49	-0.50764	-0.39339
57.507	-0.50766	-0.39346
57.523	-0.50768	-0.39354
57.54	-0.5077	-0.39362
57.557	-0.50772	-0.39369
57.574	-0.50775	-0.39373
57.591	-0.50777	-0.39376
57.607	-0.50779	-0.39383
57.624	-0.5078	-0.39386
57.64	-0.50782	-0.39389
57.657	-0.50784	-0.39392
57.674	-0.50787	-0.39395
57.69	-0.50789	-0.39399
57.706	-0.50791	-0.39407
57.723	-0.50793	-0.39419
57.74	-0.50795	-0.39435
57.757	-0.50797	-0.3945
57.774	-0.50799	-0.3946
57.79	-0.50801	-0.39466
57.807	-0.50803	-0.39471
57.824	-0.50805	-0.39477
57.84	-0.50806	-0.39484
57.856	-0.50808	-0.39488
57.873	-0.5081	-0.39496
57.89	-0.50811	-0.39504
57.907	-0.50813	-0.3951
57.923	-0.50815	-0.39517
57.94	-0.50817	-0.39524

57.957	-0.50819	-0.39529
57.974	-0.50821	-0.39532
57.989	-0.50823	-0.39541
58.006	-0.50825	-0.39543
58.023	-0.50826	-0.39546
58.039	-0.50828	-0.39549
58.056	-0.5083	-0.39553
58.072	-0.50832	-0.39559
58.089	-0.50834	-0.39564
58.106	-0.50836	-0.39571
58.122	-0.50838	-0.3958
58.139	-0.50839	-0.39586
58.156	-0.50841	-0.39592
58.172	-0.50842	-0.39597
58.189	-0.50844	-0.39603
58.205	-0.50845	-0.39606
58.222	-0.50847	-0.39613
58.239	-0.50848	-0.39617
58.256	-0.5085	-0.39624
58.272	-0.50852	-0.3963
58.288	-0.50853	-0.39636
58.305	-0.50855	-0.39642
58.322	-0.50857	-0.39649
58.339	-0.50859	-0.39655
58.355	-0.50861	-0.39661
58.371	-0.50863	-0.39668
58.389	-0.50865	-0.39675
58.406	-0.50866	-0.3968
58.422	-0.50868	-0.39683
58.438	-0.5087	-0.39686

58.456	-0.50872	-0.3969
58.472	-0.50874	-0.39693
58.488	-0.50876	-0.39697
58.505	-0.50878	-0.39703
58.522	-0.50879	-0.39706
58.537	-0.50881	-0.39709
58.555	-0.50882	-0.39712
58.571	-0.50884	-0.39717
58.587	-0.50885	-0.39721
58.604	-0.50887	-0.39727
58.62	-0.50888	-0.39732
58.637	-0.5089	-0.39738
58.654	-0.50892	-0.39746
58.671	-0.50893	-0.39756
58.688	-0.50895	-0.39767
58.705	-0.50897	-0.39777
58.722	-0.50899	-0.39783
58.738	-0.50901	-0.39788
58.754	-0.50903	-0.39791
58.771	-0.50904	-0.39795
58.788	-0.50906	-0.39799
58.803	-0.50908	-0.39803
58.821	-0.5091	-0.39809
58.837	-0.50911	-0.39816
58.855	-0.50913	-0.39822
58.871	-0.50914	-0.39831
58.887	-0.50915	-0.39843
58.904	-0.50916	-0.39855
58.921	-0.50917	-0.39871
58.937	-0.50918	-0.39879

58.954	-0.5092	-0.39889
58.972	-0.50921	-0.39896
58.988	-0.50922	-0.39899
59.005	-0.50924	-0.39902
59.02	-0.50925	-0.39906
59.038	-0.50927	-0.39909
59.055	-0.50929	-0.39912
59.07	-0.50931	-0.39915
59.087	-0.50933	-0.39919
59.105	-0.50934	-0.39926
59.121	-0.50936	-0.39933
59.138	-0.50938	-0.39943
59.153	-0.50939	-0.39953
59.169	-0.50941	-0.39962
59.188	-0.50942	-0.3997
59.203	-0.50943	-0.39979
59.221	-0.50945	-0.39986
59.237	-0.50946	-0.39994
59.254	-0.50948	-0.40001
59.27	-0.5095	-0.40007
59.288	-0.50951	-0.40012
59.304	-0.50953	-0.40016
59.321	-0.50955	-0.4002
59.337	-0.50956	-0.40023
59.353	-0.50957	-0.40028
59.37	-0.50958	-0.40031
59.387	-0.50959	-0.40035
59.403	-0.5096	-0.40039
59.42	-0.50962	-0.40045
59.436	-0.50963	-0.40051

59.453	-0.50964	-0.40059
59.47	-0.50966	-0.40069
59.487	-0.50967	-0.40079
59.503	-0.50969	-0.40087
59.52	-0.50971	-0.40092
59.538	-0.50973	-0.40096
59.554	-0.50974	-0.40101
59.57	-0.50976	-0.40104
59.587	-0.50977	-0.40107
59.604	-0.50978	-0.40112
59.62	-0.50979	-0.40115
59.637	-0.5098	-0.4012
59.654	-0.50981	-0.40126
59.67	-0.50982	-0.40134
59.687	-0.50982	-0.40143
59.703	-0.50983	-0.4015
59.72	-0.50984	-0.40156
59.738	-0.50985	-0.40163
59.754	-0.50986	-0.4017
59.77	-0.50987	-0.40173
59.787	-0.50988	-0.40177
59.803	-0.50989	-0.4018
59.82	-0.50991	-0.40184
59.836	-0.50992	-0.40187
59.853	-0.50994	-0.40192
59.871	-0.50995	-0.40197
59.887	-0.50997	-0.40202
59.904	-0.50998	-0.40208
59.92	-0.51	-0.40216
59.937	-0.51001	-0.40223

59.953	-0.51002	-0.40228
59.97	-0.51003	-0.40234
59.987	-0.51004	-0.40238
60.004	-0.51005	-0.40241
60.02	-0.51006	-0.40246
60.036	-0.51007	-0.4025
60.054	-0.51008	-0.40253
60.071	-0.51009	-0.40259
60.086	-0.51009	-0.40262
60.103	-0.5101	-0.40265
60.12	-0.51011	-0.40269
60.136	-0.51012	-0.40272
60.153	-0.51012	-0.40276
60.169	-0.51013	-0.40279
60.186	-0.51014	-0.40283
60.203	-0.51014	-0.40286
60.219	-0.51015	-0.40291
60.236	-0.51016	-0.40294
60.253	-0.51016	-0.403
60.269	-0.51017	-0.40305
60.287	-0.51017	-0.40309
60.303	-0.51018	-0.40313
60.319	-0.51018	-0.40316
60.337	-0.51019	-0.4032
60.353	-0.5102	-0.40324
60.369	-0.51021	-0.40327
60.386	-0.51021	-0.40332
60.402	-0.51022	-0.40337
60.42	-0.51022	-0.40341
60.436	-0.51023	-0.40346

60.453	-0.51023	-0.40352
60.469	-0.51024	-0.40357
60.486	-0.51024	-0.40361
60.503	-0.51025	-0.40365
60.52	-0.51026	-0.40369
60.536	-0.51027	-0.40372
60.553	-0.51028	-0.40376
60.569	-0.51029	-0.4038
60.586	-0.5103	-0.40383
60.603	-0.5103	-0.40387
60.619	-0.51031	-0.40391
60.637	-0.51031	-0.40396
60.653	-0.51032	-0.404
60.669	-0.51032	-0.40404
60.685	-0.51032	-0.40407
60.702	-0.51033	-0.40411
60.719	-0.51033	-0.40415
60.734	-0.51033	-0.40419
60.752	-0.51034	-0.40423
60.769	-0.51034	-0.40428
60.785	-0.51035	-0.40433
60.802	-0.51035	-0.40438
60.819	-0.51035	-0.40444
60.836	-0.51036	-0.40449
60.853	-0.51036	-0.40453
60.869	-0.51037	-0.40457
60.885	-0.51037	-0.40462
60.902	-0.51038	-0.40466
60.918	-0.51038	-0.40469
60.935	-0.51039	-0.40473

60.952	-0.51039	-0.40477
60.968	-0.5104	-0.40481
60.985	-0.51041	-0.40486
61.002	-0.51041	-0.40494
61.019	-0.51042	-0.40504
61.035	-0.51043	-0.40512
61.052	-0.51043	-0.40516
61.068	-0.51043	-0.4052
61.085	-0.51044	-0.40524
61.102	-0.51044	-0.40529
61.118	-0.51043	-0.40532
61.135	-0.51043	-0.40536
61.152	-0.51044	-0.40539
61.169	-0.51044	-0.40543
61.185	-0.51045	-0.40546
61.202	-0.51045	-0.4055
61.218	-0.51045	-0.40554
61.234	-0.51045	-0.40557
61.251	-0.51046	-0.40561
61.268	-0.51046	-0.40565
61.284	-0.51046	-0.40569
61.301	-0.51046	-0.40572
61.318	-0.51046	-0.40576
61.335	-0.51046	-0.4058
61.351	-0.51047	-0.40583
61.368	-0.51047	-0.40587
61.385	-0.51048	-0.40592
61.401	-0.51048	-0.40597
61.418	-0.51049	-0.40603
61.434	-0.5105	-0.40611

61.451	-0.5105	-0.40619
61.468	-0.51051	-0.40626
61.485	-0.51051	-0.4063
61.501	-0.51052	-0.40634
61.518	-0.51053	-0.40638
61.535	-0.51053	-0.40641
61.551	-0.51054	-0.40645
61.568	-0.51055	-0.40649
61.585	-0.51055	-0.40653
61.602	-0.51055	-0.40656
61.619	-0.51056	-0.4066
61.635	-0.51056	-0.40664
61.651	-0.51056	-0.40668
61.668	-0.51056	-0.40672
61.684	-0.51057	-0.40675
61.701	-0.51057	-0.40679
61.717	-0.51057	-0.40683
61.734	-0.51058	-0.40687
61.751	-0.51058	-0.40691
61.768	-0.51059	-0.40694
61.784	-0.51059	-0.40699
61.802	-0.5106	-0.40705
61.818	-0.5106	-0.40712
61.834	-0.51061	-0.40719
61.851	-0.51062	-0.40725
61.867	-0.51063	-0.40729
61.884	-0.51063	-0.40733
61.901	-0.51063	-0.40736
61.918	-0.51063	-0.4074
61.934	-0.51063	-0.40743

61.951	-0.51063	-0.40747
61.968	-0.51063	-0.4075
61.984	-0.51063	-0.40754
62.001	-0.51063	-0.40758
62.017	-0.51063	-0.40761
62.035	-0.51064	-0.40766
62.051	-0.51064	-0.4077
62.069	-0.51065	-0.40774
62.085	-0.51065	-0.40777
62.1	-0.51065	-0.40781
62.118	-0.51065	-0.40786
62.134	-0.51065	-0.40791
62.151	-0.51065	-0.40796
62.167	-0.51065	-0.40801
62.185	-0.51065	-0.40805
62.201	-0.51065	-0.40809
62.217	-0.51066	-0.40813
62.233	-0.51066	-0.40816
62.251	-0.51066	-0.4082
62.268	-0.51066	-0.40823
62.284	-0.51066	-0.40827
62.301	-0.51065	-0.4083
62.317	-0.51065	-0.40834
62.333	-0.51065	-0.40838
62.35	-0.51064	-0.40841
62.367	-0.51064	-0.40845
62.385	-0.51064	-0.40849
62.402	-0.51064	-0.40853
62.417	-0.51063	-0.40857
62.434	-0.51063	-0.4086

62.45	-0.51063	-0.40864
62.467	-0.51062	-0.40868
62.484	-0.51062	-0.40871
62.502	-0.51062	-0.40875
62.517	-0.51062	-0.40879
62.534	-0.51062	-0.40882
62.55	-0.51062	-0.40886
62.567	-0.51062	-0.40889
62.584	-0.51061	-0.40893
62.6	-0.5106	-0.40896
62.617	-0.5106	-0.409
62.634	-0.51059	-0.40903
62.65	-0.51058	-0.40907
62.667	-0.51058	-0.40911
62.684	-0.51058	-0.40914
62.7	-0.51058	-0.40917
62.716	-0.51058	-0.40921
62.733	-0.51058	-0.40924
62.749	-0.51058	-0.40928
62.766	-0.51058	-0.40931
62.782	-0.51057	-0.40935
62.8	-0.51057	-0.40939
62.816	-0.51057	-0.40942
62.833	-0.51057	-0.40946
62.849	-0.51058	-0.4095
62.866	-0.51058	-0.40954
62.883	-0.51057	-0.40957
62.9	-0.51057	-0.40961
62.916	-0.51056	-0.40965
62.933	-0.51055	-0.40969

62.95	-0.51055	-0.40972
62.966	-0.51054	-0.40976
62.983	-0.51053	-0.40979
62.999	-0.51052	-0.40983
63.016	-0.51052	-0.40987
63.032	-0.51051	-0.40991
63.049	-0.51051	-0.40994
63.066	-0.5105	-0.40998
63.083	-0.5105	-0.41002
63.099	-0.5105	-0.41005
63.116	-0.51049	-0.41009
63.133	-0.51049	-0.41012
63.149	-0.51049	-0.41016
63.166	-0.51049	-0.41019
63.183	-0.51049	-0.41023
63.2	-0.51049	-0.41026
63.216	-0.51049	-0.4103
63.232	-0.51049	-0.41033
63.249	-0.51049	-0.4104
63.266	-0.51049	-0.41047
63.283	-0.51049	-0.41053
63.299	-0.51049	-0.41057
63.316	-0.51049	-0.41061
63.333	-0.51048	-0.41064
63.35	-0.51048	-0.41068
63.366	-0.51048	-0.41071
63.383	-0.51048	-0.41074
63.399	-0.51048	-0.41078
63.417	-0.51047	-0.41081
63.432	-0.51047	-0.41085

63.45	-0.51047	-0.4109
63.466	-0.51046	-0.41094
63.483	-0.51045	-0.41098
63.5	-0.51045	-0.41104
63.518	-0.51044	-0.41108
63.534	-0.51044	-0.41111
63.549	-0.51044	-0.41115
63.567	-0.51043	-0.41119
63.583	-0.51043	-0.41123
63.599	-0.51043	-0.41126
63.616	-0.51043	-0.4113
63.632	-0.51042	-0.41133
63.65	-0.51042	-0.41136
63.667	-0.51041	-0.41139
63.684	-0.5104	-0.41142
63.7	-0.5104	-0.41145
63.716	-0.51039	-0.41148
63.733	-0.51038	-0.41151
63.75	-0.51038	-0.41154
63.767	-0.51038	-0.41157
63.783	-0.51038	-0.41161
63.8	-0.51038	-0.41164
63.817	-0.51037	-0.41168
63.833	-0.51037	-0.41171
63.85	-0.51037	-0.41175
63.866	-0.51036	-0.41179
63.883	-0.51036	-0.41182
63.9	-0.51035	-0.41185
63.916	-0.51034	-0.41188
63.933	-0.51033	-0.41191

63.95	-0.51032	-0.41194
63.967	-0.51031	-0.41197
63.984	-0.5103	-0.412
64	-0.5103	-0.41204
64.017	-0.51029	-0.41207
64.033	-0.51029	-0.4121
64.05	-0.51028	-0.41213
64.066	-0.51027	-0.41216
64.084	-0.51026	-0.41219
64.1	-0.51025	-0.41223
64.117	-0.51024	-0.41226
64.133	-0.51023	-0.41229
64.15	-0.51022	-0.41232
64.166	-0.51022	-0.41235
64.183	-0.51021	-0.41238
64.2	-0.51021	-0.41241
64.216	-0.5102	-0.41244
64.233	-0.51019	-0.41247
64.25	-0.51018	-0.4125
64.267	-0.51017	-0.41253
64.283	-0.51016	-0.41256
64.3	-0.51015	-0.41259
64.317	-0.51014	-0.41261
64.333	-0.51013	-0.41264
64.349	-0.51011	-0.41267
64.366	-0.5101	-0.41269
64.383	-0.51009	-0.41272
64.399	-0.51007	-0.41275
64.416	-0.51006	-0.41277
64.434	-0.51005	-0.4128

64.45	-0.51004	-0.41283
64.466	-0.51003	-0.41287
64.483	-0.51003	-0.4129
64.499	-0.51002	-0.41293
64.516	-0.51001	-0.41296
64.532	-0.50999	-0.41299
64.549	-0.50998	-0.41302
64.566	-0.50996	-0.41305
64.584	-0.50994	-0.41308
64.6	-0.50993	-0.4131
64.616	-0.50991	-0.41313
64.633	-0.50989	-0.41316
64.649	-0.50988	-0.41318
64.666	-0.50987	-0.41321
64.683	-0.50986	-0.41323
64.699	-0.50985	-0.41326
64.716	-0.50985	-0.41329
64.733	-0.50984	-0.41332
64.75	-0.50983	-0.41335
64.767	-0.50982	-0.41337
64.784	-0.50982	-0.4134
64.8	-0.5098	-0.41343
64.817	-0.50979	-0.41345
64.834	-0.50978	-0.41347
64.85	-0.50976	-0.4135
64.866	-0.50975	-0.41353
64.883	-0.50973	-0.41355
64.9	-0.50972	-0.41358
64.917	-0.50971	-0.41361
64.933	-0.5097	-0.41365

64.95	-0.5097	-0.41369
64.967	-0.50968	-0.41371
64.984	-0.50967	-0.41374
65	-0.50966	-0.41377
65.016	-0.50965	-0.4138
65.033	-0.50964	-0.41382
65.05	-0.50963	-0.41385
65.066	-0.50962	-0.41387
65.083	-0.50961	-0.4139
65.1	-0.50959	-0.41393
65.117	-0.50958	-0.41395
65.133	-0.50956	-0.41398
65.15	-0.50955	-0.41401
65.166	-0.50953	-0.41403
65.182	-0.50951	-0.41406
65.2	-0.5095	-0.41409
65.217	-0.50948	-0.41412
65.233	-0.50946	-0.41415
65.25	-0.50945	-0.41418
65.266	-0.50943	-0.4142
65.282	-0.50942	-0.41423
65.301	-0.50941	-0.41424
65.317	-0.50939	-0.41427
65.334	-0.50937	-0.41429
65.35	-0.50936	-0.41431
65.366	-0.50933	-0.41433
65.384	-0.50932	-0.41436
65.4	-0.5093	-0.41438
65.417	-0.50928	-0.41441
65.433	-0.50926	-0.41443

65.449	-0.50924	-0.41446
65.466	-0.50922	-0.41448
65.483	-0.5092	-0.4145
65.5	-0.50918	-0.41452
65.517	-0.50916	-0.41454
65.532	-0.50914	-0.41457
65.55	-0.50913	-0.41459
65.567	-0.50912	-0.41461
65.583	-0.50911	-0.41464
65.6	-0.5091	-0.41466
65.616	-0.50909	-0.41469
65.634	-0.50907	-0.41471
65.65	-0.50906	-0.41473
65.666	-0.50904	-0.41475
65.682	-0.50902	-0.41477
65.7	-0.50901	-0.41479
65.716	-0.50899	-0.41481
65.733	-0.50897	-0.41483
65.75	-0.50896	-0.41485
65.766	-0.50894	-0.41487
65.784	-0.50892	-0.41489
65.8	-0.5089	-0.41491
65.816	-0.50888	-0.41493
65.833	-0.50886	-0.41496
65.85	-0.50883	-0.41498
65.866	-0.50881	-0.415
65.883	-0.50879	-0.41502
65.9	-0.50877	-0.41504
65.916	-0.50875	-0.41506
65.933	-0.50874	-0.41508

65.949	-0.50872	-0.4151
65.966	-0.5087	-0.41511
65.983	-0.50869	-0.41513
65.999	-0.50867	-0.41515
66.016	-0.50865	-0.41517
66.033	-0.50863	-0.41519
66.05	-0.50862	-0.41521
66.067	-0.5086	-0.41523
66.083	-0.50859	-0.41526
66.1	-0.50857	-0.41528
66.117	-0.50856	-0.4153
66.133	-0.50855	-0.41531
66.15	-0.50853	-0.41533
66.166	-0.50852	-0.41534
66.183	-0.5085	-0.41536
66.201	-0.50849	-0.41537
66.217	-0.50847	-0.41539
66.232	-0.50846	-0.41541
66.25	-0.50845	-0.41543
66.266	-0.50843	-0.41544
66.283	-0.50842	-0.41546
66.3	-0.5084	-0.41548
66.317	-0.50839	-0.4155
66.334	-0.50837	-0.41552
66.351	-0.50835	-0.41554
66.367	-0.50833	-0.41556
66.384	-0.50831	-0.41557
66.4	-0.50829	-0.41559
66.417	-0.50827	-0.41561
66.432	-0.50825	-0.41563

66.449	-0.50823	-0.41564
66.466	-0.50821	-0.41566
66.483	-0.50819	-0.41568
66.499	-0.50817	-0.41569
66.516	-0.50815	-0.41571
66.534	-0.50813	-0.41572
66.55	-0.50811	-0.41574
66.566	-0.50809	-0.41575
66.583	-0.50808	-0.41577
66.6	-0.50806	-0.41578
66.617	-0.50804	-0.41579
66.634	-0.50802	-0.41581
66.65	-0.508	-0.41583
66.667	-0.50798	-0.41585
66.684	-0.50797	-0.41587
66.701	-0.50795	-0.41589
66.717	-0.50793	-0.4159
66.733	-0.50792	-0.41592
66.75	-0.5079	-0.41593
66.767	-0.50788	-0.41595
66.785	-0.50786	-0.41596
66.801	-0.50784	-0.41597
66.817	-0.50782	-0.41598
66.833	-0.5078	-0.41599
66.851	-0.50778	-0.416
66.866	-0.50776	-0.41601
66.884	-0.50774	-0.41603
66.9	-0.50772	-0.41605
66.916	-0.5077	-0.41606
66.934	-0.50768	-0.41608

66.95	-0.50766	-0.41609
66.966	-0.50764	-0.4161
66.984	-0.50762	-0.41611
67	-0.5076	-0.41613
67.016	-0.50758	-0.41614
67.033	-0.50755	-0.41615
67.05	-0.50753	-0.41616
67.067	-0.50751	-0.41617
67.083	-0.50748	-0.41619
67.101	-0.50746	-0.4162
67.117	-0.50744	-0.41621
67.134	-0.50742	-0.41623
67.151	-0.50741	-0.41624
67.167	-0.5074	-0.41625
67.184	-0.50738	-0.41627
67.2	-0.50736	-0.41628
67.218	-0.50735	-0.41629
67.233	-0.50733	-0.41631
67.25	-0.50731	-0.41632
67.267	-0.50729	-0.41633
67.284	-0.50728	-0.41634
67.301	-0.50726	-0.41636
67.317	-0.50723	-0.41637
67.334	-0.50722	-0.41638
67.351	-0.5072	-0.41639
67.368	-0.50718	-0.4164
67.384	-0.50715	-0.41641
67.4	-0.50713	-0.41641
67.417	-0.5071	-0.41642
67.433	-0.50707	-0.41643

67.451	-0.50705	-0.41645
67.468	-0.50702	-0.41646
67.484	-0.507	-0.41647
67.501	-0.50697	-0.41648
67.518	-0.50695	-0.41649
67.534	-0.50693	-0.4165
67.551	-0.50691	-0.41651
67.568	-0.50689	-0.41653
67.584	-0.50687	-0.41653
67.601	-0.50685	-0.41654
67.618	-0.50683	-0.41654
67.634	-0.5068	-0.41655
67.651	-0.50678	-0.41656
67.667	-0.50676	-0.41657
67.684	-0.50673	-0.41658
67.701	-0.50671	-0.41659
67.718	-0.50669	-0.4166
67.734	-0.50666	-0.41661
67.751	-0.50664	-0.41661
67.767	-0.50661	-0.41662
67.785	-0.50659	-0.41663
67.801	-0.50656	-0.41663
67.817	-0.50654	-0.41664
67.834	-0.50651	-0.41664
67.851	-0.50649	-0.41665
67.868	-0.50647	-0.41666
67.885	-0.50645	-0.41666
67.901	-0.50643	-0.41666
67.918	-0.50642	-0.41667
67.934	-0.5064	-0.41667

67.951	-0.50638	-0.41668
67.967	-0.50635	-0.41669
67.985	-0.50633	-0.41669
68.001	-0.50631	-0.4167
68.018	-0.50629	-0.4167
68.035	-0.50626	-0.41671
68.051	-0.50624	-0.41671
68.067	-0.50621	-0.41671
68.085	-0.50619	-0.41672
68.102	-0.50616	-0.41672
68.118	-0.50614	-0.41672
68.135	-0.50611	-0.41673
68.152	-0.50609	-0.41673
68.168	-0.50606	-0.41674
68.185	-0.50604	-0.41674
68.201	-0.50602	-0.41674
68.217	-0.506	-0.41674
68.234	-0.50597	-0.41675
68.251	-0.50595	-0.41675
68.268	-0.50592	-0.41676
68.284	-0.5059	-0.41676
68.302	-0.50587	-0.41676
68.318	-0.50585	-0.41676
68.334	-0.50582	-0.41677
68.351	-0.5058	-0.41677
68.368	-0.50578	-0.41677
68.385	-0.50576	-0.41678
68.402	-0.50574	-0.41678
68.418	-0.50571	-0.41679
68.435	-0.50569	-0.41679

68.452	-0.50566	-0.41679
68.468	-0.50564	-0.41679
68.485	-0.50561	-0.41678
68.501	-0.50559	-0.41678
68.518	-0.50557	-0.41678
68.535	-0.50554	-0.41678
68.551	-0.50552	-0.41678
68.568	-0.5055	-0.41677
68.585	-0.50548	-0.41677
68.601	-0.50545	-0.41678
68.618	-0.50543	-0.41678
68.634	-0.5054	-0.41678
68.651	-0.50537	-0.41678
68.667	-0.50535	-0.41679
68.684	-0.50533	-0.41679
68.701	-0.5053	-0.41679
68.718	-0.50528	-0.41679
68.734	-0.50525	-0.41679
68.751	-0.50522	-0.41678
68.767	-0.50519	-0.41678
68.785	-0.50516	-0.41678
68.801	-0.50513	-0.41678
68.818	-0.50511	-0.41678
68.834	-0.50508	-0.41678
68.851	-0.50506	-0.41679
68.867	-0.50504	-0.41679
68.884	-0.50502	-0.41679
68.902	-0.505	-0.41679
68.917	-0.50498	-0.41678
68.935	-0.50496	-0.41678

68.951	-0.50494	-0.41678
68.968	-0.50492	-0.41677
68.984	-0.5049	-0.41677
69.001	-0.50488	-0.41677
69.017	-0.50486	-0.41676
69.035	-0.50483	-0.41676
69.051	-0.50481	-0.41676
69.068	-0.50479	-0.41676
69.084	-0.50477	-0.41676
69.101	-0.50475	-0.41676
69.117	-0.50472	-0.41676
69.135	-0.50469	-0.41676
69.152	-0.50467	-0.41676
69.168	-0.50464	-0.41676
69.184	-0.50462	-0.41676
69.202	-0.5046	-0.41676
69.218	-0.50458	-0.41676
69.235	-0.50455	-0.41675
69.251	-0.50453	-0.41674
69.269	-0.50451	-0.41674
69.285	-0.50449	-0.41673
69.302	-0.50447	-0.41673
69.318	-0.50444	-0.41672
69.335	-0.50441	-0.41672
69.352	-0.50439	-0.41672
69.368	-0.50436	-0.41671
69.384	-0.50434	-0.41671
69.401	-0.50431	-0.41671
69.419	-0.50429	-0.4167
69.435	-0.50426	-0.4167

69.452	-0.50424	-0.4167
69.468	-0.50421	-0.41669
69.485	-0.50419	-0.41669
69.502	-0.50417	-0.41668
69.518	-0.50414	-0.41668
69.535	-0.50412	-0.41667
69.551	-0.50409	-0.41667
69.568	-0.50407	-0.41667
69.585	-0.50405	-0.41666
69.602	-0.50403	-0.41666
69.618	-0.504	-0.41666
69.635	-0.50398	-0.41666
69.653	-0.50396	-0.41666
69.669	-0.50394	-0.41665
69.686	-0.50393	-0.41665
69.702	-0.50391	-0.41664
69.718	-0.50389	-0.41663
69.735	-0.50387	-0.41661
69.751	-0.50385	-0.4166
69.769	-0.50383	-0.41659
69.785	-0.5038	-0.41659
69.801	-0.50378	-0.41658
69.818	-0.50376	-0.41657
69.835	-0.50374	-0.41657
69.852	-0.50371	-0.41656
69.869	-0.50369	-0.41656
69.886	-0.50366	-0.41656
69.902	-0.50364	-0.41656
69.918	-0.50362	-0.41655
69.936	-0.50359	-0.41654

69.952	-0.50357	-0.41653
69.969	-0.50354	-0.41653
69.986	-0.50352	-0.41652
70.002	-0.5035	-0.41651
70.019	-0.50348	-0.4165
70.035	-0.50346	-0.4165
70.053	-0.50344	-0.41649
70.069	-0.50342	-0.41648
70.086	-0.5034	-0.41647
70.103	-0.50338	-0.41646
70.12	-0.50335	-0.41644
70.136	-0.50333	-0.41644
70.153	-0.5033	-0.41643
70.169	-0.50327	-0.41642
70.185	-0.50324	-0.41641
70.203	-0.50321	-0.4164
70.22	-0.50319	-0.4164
70.236	-0.50317	-0.41639
70.253	-0.50315	-0.41639
70.269	-0.50313	-0.41637
70.286	-0.50312	-0.41636
70.303	-0.5031	-0.41634
70.319	-0.50308	-0.41633
70.336	-0.50306	-0.41631
70.352	-0.50304	-0.4163
70.369	-0.50301	-0.41629
70.386	-0.50299	-0.41628
70.403	-0.50296	-0.41627
70.42	-0.50294	-0.41626
70.436	-0.50291	-0.41625

70.453	-0.50289	-0.41624
70.469	-0.50287	-0.41623
70.487	-0.50286	-0.41621
70.503	-0.50284	-0.41619
70.52	-0.50282	-0.41618
70.536	-0.5028	-0.41617
70.553	-0.50277	-0.41616
70.569	-0.50275	-0.41614
70.586	-0.50273	-0.41614
70.602	-0.50271	-0.41613
70.619	-0.50268	-0.41612
70.637	-0.50266	-0.41611
70.653	-0.50264	-0.4161
70.671	-0.50262	-0.41609
70.687	-0.50259	-0.41608
70.703	-0.50257	-0.41606
70.72	-0.50254	-0.41605
70.736	-0.50251	-0.41603
70.754	-0.50248	-0.41602
70.77	-0.50246	-0.416
70.788	-0.50243	-0.41599
70.802	-0.5024	-0.41597
70.82	-0.50238	-0.41596
70.836	-0.50235	-0.41594
70.853	-0.50233	-0.41593
70.87	-0.50231	-0.41592
70.887	-0.50229	-0.41591
70.904	-0.50227	-0.4159
70.92	-0.50226	-0.41589
70.937	-0.50223	-0.41587

70.954	-0.50221	-0.41586
70.971	-0.50219	-0.41585
70.987	-0.50216	-0.41584
71.003	-0.50214	-0.41582
71.021	-0.50211	-0.4158
71.037	-0.50208	-0.41578
71.054	-0.50206	-0.41576
71.071	-0.50203	-0.41574
71.087	-0.50201	-0.41573
71.104	-0.50198	-0.41571
71.12	-0.50196	-0.4157
71.137	-0.50194	-0.41569
71.154	-0.50192	-0.41567
71.172	-0.50189	-0.41566
71.188	-0.50187	-0.41565
71.204	-0.50185	-0.41563
71.221	-0.50183	-0.41562
71.238	-0.5018	-0.4156
71.255	-0.50178	-0.41558
71.27	-0.50175	-0.41556
71.287	-0.50173	-0.41554
71.305	-0.50171	-0.41553
71.322	-0.50168	-0.41551
71.338	-0.50166	-0.41549
71.355	-0.50164	-0.41547
71.371	-0.50162	-0.41544
71.388	-0.5016	-0.41541
71.406	-0.50157	-0.4154
71.422	-0.50155	-0.41538
71.439	-0.50152	-0.41536

71.456	-0.5015	-0.41534
71.472	-0.50147	-0.41533
71.488	-0.50144	-0.41531
71.505	-0.50141	-0.41529
71.522	-0.50139	-0.41528
71.538	-0.50137	-0.41526
71.555	-0.50134	-0.41524
71.571	-0.50132	-0.41522
71.587	-0.50129	-0.41519
71.604	-0.50127	-0.41516
71.621	-0.50124	-0.41514
71.638	-0.50122	-0.41511
71.655	-0.50119	-0.4151
71.671	-0.50116	-0.41507
71.688	-0.50114	-0.41505
71.704	-0.50111	-0.41503
71.721	-0.50109	-0.41501
71.738	-0.50106	-0.41499
71.754	-0.50103	-0.41497
71.771	-0.50101	-0.41495
71.788	-0.50098	-0.41493
71.806	-0.50095	-0.41491
71.821	-0.50092	-0.41489
71.838	-0.5009	-0.41487
71.855	-0.50087	-0.41485
71.872	-0.50085	-0.41483
71.888	-0.50082	-0.41481
71.905	-0.5008	-0.41479
71.922	-0.50078	-0.41477
71.939	-0.50076	-0.41475

71.955	-0.50074	-0.41473
71.973	-0.50071	-0.4147
71.989	-0.50068	-0.41468
72.006	-0.50065	-0.41465
72.023	-0.50063	-0.41461
72.038	-0.5006	-0.41457
72.055	-0.50058	-0.41455
72.072	-0.50056	-0.41453
72.088	-0.50054	-0.4145
72.105	-0.50052	-0.41447
72.122	-0.5005	-0.41444
72.138	-0.50048	-0.41441
72.155	-0.50046	-0.41439
72.172	-0.50043	-0.41437
72.189	-0.50041	-0.41435
72.206	-0.50039	-0.41433
72.222	-0.50036	-0.4143
72.239	-0.50034	-0.41429
72.255	-0.50032	-0.41427
72.273	-0.5003	-0.41424
72.289	-0.50027	-0.41422
72.306	-0.50025	-0.4142
72.322	-0.50023	-0.41418
72.339	-0.50021	-0.41415
72.355	-0.50018	-0.41413
72.372	-0.50016	-0.4141
72.39	-0.50013	-0.41408
72.406	-0.50011	-0.41405
72.422	-0.50008	-0.41403
72.439	-0.50006	-0.414

72.456	-0.50003	-0.41397
72.473	-0.50001	-0.41394
72.489	-0.49998	-0.41391
72.506	-0.49996	-0.41389
72.523	-0.49994	-0.41386
72.539	-0.49991	-0.41383
72.556	-0.49989	-0.41377
72.573	-0.49987	-0.41373
72.589	-0.49985	-0.41369
72.605	-0.49983	-0.41366
72.623	-0.49981	-0.41363
72.64	-0.49979	-0.4136
72.656	-0.49976	-0.41357
72.672	-0.49974	-0.41355
72.69	-0.49972	-0.41353
72.706	-0.49969	-0.4135
72.723	-0.49967	-0.41348
72.739	-0.49965	-0.41345
72.756	-0.49963	-0.41343
72.773	-0.4996	-0.4134
72.789	-0.49958	-0.41338
72.806	-0.49955	-0.41335
72.824	-0.49953	-0.41332
72.84	-0.4995	-0.41329
72.856	-0.49948	-0.41326
72.874	-0.49946	-0.41323
72.889	-0.49943	-0.4132
72.906	-0.49941	-0.41317
72.923	-0.49939	-0.41314
72.94	-0.49936	-0.41311

72.956	-0.49934	-0.41309
72.973	-0.49932	-0.41306
72.99	-0.4993	-0.41303
73.006	-0.49927	-0.413
73.023	-0.49925	-0.41297
73.04	-0.49923	-0.41295
73.056	-0.49921	-0.41292
73.073	-0.49918	-0.41289
73.09	-0.49916	-0.41286
73.107	-0.49913	-0.41283
73.124	-0.49911	-0.41281
73.14	-0.49909	-0.41278
73.157	-0.49906	-0.41275
73.174	-0.49904	-0.41271
73.191	-0.49901	-0.41268
73.207	-0.49899	-0.41265
73.224	-0.49896	-0.41262
73.24	-0.49894	-0.41259
73.257	-0.49891	-0.41256
73.274	-0.49889	-0.41253
73.291	-0.49887	-0.4125
73.308	-0.49884	-0.41247
73.324	-0.49882	-0.41244
73.341	-0.4988	-0.41239
73.358	-0.49877	-0.41232
73.376	-0.49875	-0.41227
73.391	-0.49873	-0.41223
73.408	-0.4987	-0.4122
73.424	-0.49867	-0.41217
73.44	-0.49864	-0.41214

73.457	-0.49861	-0.41211
73.474	-0.49858	-0.41208
73.491	-0.49855	-0.41205
73.508	-0.49853	-0.41202
73.524	-0.4985	-0.41199
73.54	-0.49848	-0.41196
73.558	-0.49846	-0.41193
73.574	-0.49845	-0.4119
73.591	-0.49843	-0.41187
73.607	-0.49841	-0.41184
73.625	-0.49838	-0.41181
73.642	-0.49836	-0.41178
73.658	-0.49833	-0.41175
73.675	-0.4983	-0.41172
73.691	-0.49827	-0.41169
73.707	-0.49824	-0.41166
73.725	-0.49821	-0.41162
73.742	-0.49819	-0.41158
73.759	-0.49817	-0.41154
73.775	-0.49815	-0.41147
73.791	-0.49813	-0.41142
73.808	-0.4981	-0.41135
73.824	-0.49808	-0.41125
73.842	-0.49806	-0.41117
73.859	-0.49803	-0.41114
73.875	-0.49801	-0.4111
73.892	-0.49798	-0.41107
73.908	-0.49795	-0.41104
73.925	-0.49793	-0.41101
73.942	-0.4979	-0.41098

73.959	-0.49788	-0.41095
73.975	-0.49785	-0.41092
73.991	-0.49783	-0.41089
74.008	-0.4978	-0.41086
74.025	-0.49778	-0.41083
74.042	-0.49776	-0.41079
74.059	-0.49773	-0.41076
74.075	-0.49771	-0.41071
74.092	-0.49768	-0.41066
74.109	-0.49766	-0.41061
74.125	-0.49763	-0.41057
74.143	-0.49761	-0.41054
74.158	-0.49759	-0.4105
74.176	-0.49756	-0.41047
74.193	-0.49755	-0.41044
74.21	-0.49752	-0.4104
74.226	-0.4975	-0.41037
74.242	-0.49747	-0.41034
74.257	-0.49745	-0.41031
74.274	-0.49743	-0.41025
74.292	-0.4974	-0.4102
74.309	-0.49737	-0.41014
74.326	-0.49735	-0.4101
74.342	-0.49732	-0.41007
74.36	-0.49729	-0.41003
74.376	-0.49727	-0.40999
74.393	-0.49724	-0.40993
74.408	-0.49721	-0.40989
74.426	-0.49718	-0.40986
74.443	-0.49715	-0.40982

74.459	-0.49713	-0.40978
74.475	-0.49711	-0.40975
74.493	-0.49708	-0.40971
74.51	-0.49706	-0.40968
74.526	-0.49704	-0.40964
74.543	-0.49702	-0.40961
74.56	-0.497	-0.40956
74.576	-0.49698	-0.4095
74.593	-0.49696	-0.40944
74.61	-0.49694	-0.4094
74.626	-0.49692	-0.40936
74.643	-0.4969	-0.40932
74.66	-0.49688	-0.40928
74.676	-0.49685	-0.40924
74.693	-0.49683	-0.40921
74.709	-0.4968	-0.40917
74.727	-0.49677	-0.40914
74.744	-0.49675	-0.4091
74.76	-0.49672	-0.40905
74.776	-0.4967	-0.40901
74.794	-0.49667	-0.40898
74.81	-0.49665	-0.40894
74.826	-0.49663	-0.40891
74.844	-0.49661	-0.40887
74.86	-0.49659	-0.40884
74.876	-0.49657	-0.4088
74.893	-0.49655	-0.40876
74.911	-0.49652	-0.40873
74.927	-0.4965	-0.40868
74.944	-0.49648	-0.40861

74.96	-0.49646	-0.40854
74.976	-0.49644	-0.40846
74.994	-0.49642	-0.40837
75.01	-0.4964	-0.4083
75.026	-0.49638	-0.40823
75.044	-0.49636	-0.40818
75.061	-0.49633	-0.40814
75.077	-0.49631	-0.4081
75.093	-0.49628	-0.40806
75.11	-0.49626	-0.40802
75.127	-0.49623	-0.40799
75.144	-0.49621	-0.40795
75.16	-0.49618	-0.40791
75.178	-0.49616	-0.40787
75.194	-0.49613	-0.40783
75.211	-0.49611	-0.40778
75.228	-0.49608	-0.40773
75.244	-0.49606	-0.40768
75.261	-0.49604	-0.40762
75.277	-0.49602	-0.40755
75.294	-0.496	-0.40751
75.311	-0.49598	-0.40747
75.328	-0.49596	-0.40744
75.345	-0.49594	-0.40741
75.361	-0.49592	-0.40736
75.379	-0.4959	-0.40733
75.395	-0.49588	-0.40726
75.411	-0.49586	-0.40717
75.429	-0.49583	-0.40709
75.445	-0.49581	-0.40701

75.462	-0.49578	-0.40693
75.478	-0.49576	-0.40687
75.496	-0.49573	-0.40683
75.511	-0.49571	-0.40679
75.528	-0.49568	-0.40676
75.545	-0.49565	-0.40671
75.561	-0.49563	-0.40666
75.578	-0.49561	-0.40663
75.595	-0.49558	-0.40659
75.612	-0.49556	-0.40653
75.628	-0.49554	-0.40647
75.645	-0.49552	-0.40642
75.661	-0.4955	-0.40638
75.678	-0.49548	-0.40634
75.697	-0.49546	-0.40631
75.713	-0.49545	-0.40627
75.729	-0.49542	-0.40624
75.746	-0.4954	-0.4062
75.762	-0.49538	-0.40617
75.779	-0.49535	-0.40611
75.796	-0.49533	-0.40604
75.812	-0.4953	-0.40595
75.828	-0.49528	-0.40584
75.846	-0.49525	-0.40575
75.863	-0.49523	-0.40567
75.878	-0.49521	-0.40557
75.895	-0.49518	-0.40553
75.913	-0.49516	-0.40548
75.929	-0.49513	-0.40541
75.946	-0.49511	-0.40536

75.963	-0.49509	-0.40531
75.98	-0.49507	-0.40525
75.996	-0.49505	-0.40521
76.014	-0.49502	-0.40517
76.029	-0.495	-0.40511
76.046	-0.49499	-0.40506
76.063	-0.49497	-0.40501
76.08	-0.49495	-0.40496
76.097	-0.49493	-0.40487
76.113	-0.4949	-0.4048
76.13	-0.49488	-0.40469
76.147	-0.49486	-0.40463
76.163	-0.49483	-0.40458
76.179	-0.4948	-0.40454
76.197	-0.49477	-0.4045
76.213	-0.49474	-0.40447
76.23	-0.49472	-0.40443
76.247	-0.49469	-0.40439
76.263	-0.49466	-0.40435
76.281	-0.49464	-0.4043
76.297	-0.49461	-0.40423
76.313	-0.49459	-0.40412
76.33	-0.49456	-0.40404
76.346	-0.49454	-0.40396
76.363	-0.49451	-0.4039
76.38	-0.49449	-0.40384
76.395	-0.49446	-0.40379
76.413	-0.49444	-0.40376
76.43	-0.49442	-0.4037
76.446	-0.4944	-0.40364

76.463	-0.49438	-0.40361
76.48	-0.49436	-0.40357
76.496	-0.49434	-0.40353
76.513	-0.49432	-0.40349
76.53	-0.49429	-0.40346
76.547	-0.49426	-0.4034
76.563	-0.49423	-0.40333
76.58	-0.4942	-0.40326
76.597	-0.49417	-0.40321
76.614	-0.49414	-0.4031
76.63	-0.49411	-0.40301
76.647	-0.49409	-0.40293
76.664	-0.49406	-0.40284
76.681	-0.49403	-0.40281
76.697	-0.49401	-0.40277
76.714	-0.49398	-0.40273
76.73	-0.49395	-0.40269
76.747	-0.49393	-0.40264
76.764	-0.4939	-0.4026
76.781	-0.49387	-0.40255
76.797	-0.49384	-0.40249
76.813	-0.49381	-0.40243
76.831	-0.49378	-0.40235
76.847	-0.49376	-0.40226
76.864	-0.49373	-0.4022
76.881	-0.49371	-0.40214
76.897	-0.49369	-0.4021
76.914	-0.49367	-0.40206
76.93	-0.49365	-0.40201
76.947	-0.49363	-0.40198

76.964	-0.49361	-0.40194
76.98	-0.49358	-0.4019
76.998	-0.49356	-0.40186
77.014	-0.49354	-0.40183
77.03	-0.49351	-0.40179
77.047	-0.49348	-0.40175
77.064	-0.49346	-0.40169
77.082	-0.49343	-0.4016
77.098	-0.4934	-0.40152
77.114	-0.49338	-0.40146
77.132	-0.49335	-0.40138
77.147	-0.49333	-0.4013
77.164	-0.49331	-0.40121
77.181	-0.49329	-0.40113
77.198	-0.49327	-0.40109
77.215	-0.49324	-0.40101
77.231	-0.49322	-0.40095
77.248	-0.4932	-0.40088
77.264	-0.49318	-0.40082
77.282	-0.49316	-0.40078
77.298	-0.49314	-0.40074
77.315	-0.49311	-0.4007
77.331	-0.4931	-0.40066
77.348	-0.49308	-0.40061
77.365	-0.49306	-0.40057
77.383	-0.49304	-0.40053
77.397	-0.49302	-0.40049
77.415	-0.493	-0.40045
77.432	-0.49298	-0.40041
77.45	-0.49295	-0.40037

77.465	-0.49293	-0.40033
77.482	-0.49291	-0.40029
77.499	-0.49289	-0.40024
77.516	-0.49287	-0.4002
77.532	-0.49284	-0.40015
77.549	-0.49282	-0.40011
77.565	-0.4928	-0.40006
77.583	-0.49277	-0.40002
77.6	-0.49275	-0.39998
77.616	-0.49272	-0.39994
77.633	-0.4927	-0.3999
77.65	-0.49268	-0.39985
77.666	-0.49266	-0.39979
77.683	-0.49264	-0.39974
77.699	-0.49262	-0.39967
77.717	-0.4926	-0.39957
77.732	-0.49258	-0.39946
77.749	-0.49255	-0.39939
77.766	-0.49253	-0.39931
77.784	-0.49251	-0.39926
77.8	-0.49248	-0.39921
77.817	-0.49246	-0.39917
77.834	-0.49244	-0.39913
77.85	-0.49241	-0.39909
77.866	-0.49238	-0.39903
77.883	-0.49236	-0.39897
77.9	-0.49234	-0.39891
77.917	-0.49231	-0.39884
77.933	-0.49229	-0.39877
77.95	-0.49227	-0.39869

77.967	-0.49225	-0.39862
77.983	-0.49223	-0.39857
78	-0.49221	-0.39851
78.017	-0.49219	-0.39847
78.034	-0.49216	-0.39841
78.051	-0.49214	-0.39836
78.067	-0.49212	-0.39831
78.084	-0.4921	-0.39827
78.1	-0.49208	-0.39823
78.117	-0.49206	-0.39816
78.134	-0.49204	-0.39807
78.151	-0.49202	-0.39796
78.167	-0.492	-0.39788
78.184	-0.49197	-0.39779
78.2	-0.49193	-0.39773
78.217	-0.49189	-0.39762
78.233	-0.49187	-0.39751
78.25	-0.49184	-0.39744
78.267	-0.49182	-0.39735
78.284	-0.4918	-0.39729
78.301	-0.49178	-0.39721
78.317	-0.49175	-0.39713
78.334	-0.49173	-0.39704
78.351	-0.49171	-0.39696
78.368	-0.49168	-0.39692
78.385	-0.49166	-0.39688
78.401	-0.49164	-0.39683
78.418	-0.49162	-0.39675
78.435	-0.4916	-0.39669
78.452	-0.49158	-0.39663

78.468	-0.49156	-0.39656
78.485	-0.49154	-0.39646
78.502	-0.49152	-0.39637
78.518	-0.4915	-0.39629
78.533	-0.49148	-0.39616
78.551	-0.49146	-0.39607
78.567	-0.49143	-0.396
78.585	-0.4914	-0.39593
78.601	-0.49138	-0.39586
78.618	-0.49135	-0.39581
78.635	-0.49133	-0.39576
78.652	-0.4913	-0.39571
78.668	-0.49128	-0.39564
78.685	-0.49126	-0.3956
78.702	-0.49124	-0.39556
78.718	-0.49122	-0.39552
78.735	-0.49119	-0.39548
78.751	-0.49117	-0.39544
78.769	-0.49114	-0.3954
78.785	-0.49112	-0.39535
78.803	-0.49109	-0.39529
78.819	-0.49106	-0.39521
78.836	-0.49104	-0.39513
78.852	-0.49101	-0.39503
78.869	-0.49099	-0.39493
78.887	-0.49096	-0.39487
78.903	-0.49094	-0.39482
78.919	-0.49092	-0.39478
78.936	-0.4909	-0.39474
78.953	-0.49088	-0.3947

78.969	-0.49086	-0.39466
78.985	-0.49084	-0.39458
79.002	-0.49082	-0.39449
79.019	-0.4908	-0.39436
79.036	-0.49077	-0.39422
79.053	-0.49074	-0.39404
79.069	-0.49072	-0.39388
79.087	-0.49069	-0.39376
79.102	-0.49067	-0.39367
79.119	-0.49065	-0.3936
79.136	-0.49062	-0.39354
79.153	-0.4906	-0.39349
79.169	-0.49058	-0.39343
79.186	-0.49056	-0.39337
79.203	-0.49054	-0.39331
79.219	-0.49052	-0.39326
79.236	-0.4905	-0.39323
79.252	-0.49048	-0.39319
79.27	-0.49045	-0.39313
79.287	-0.49043	-0.39306
79.303	-0.49041	-0.39298
79.32	-0.49039	-0.3929
79.337	-0.49036	-0.3928
79.353	-0.49034	-0.39271
79.37	-0.49031	-0.39265
79.387	-0.49028	-0.39258
79.404	-0.49026	-0.39249
79.42	-0.49023	-0.39238
79.437	-0.49021	-0.39229
79.453	-0.49018	-0.3922

79.47	-0.49016	-0.39209
79.487	-0.49014	-0.39199
79.504	-0.49012	-0.39193
79.521	-0.4901	-0.39187
79.537	-0.49008	-0.39184
79.554	-0.49006	-0.3918
79.57	-0.49004	-0.39177
79.587	-0.49001	-0.39171
79.604	-0.48999	-0.39162
79.621	-0.48996	-0.3915
79.637	-0.48994	-0.39137
79.654	-0.48992	-0.39123
79.671	-0.4899	-0.39111
79.687	-0.48987	-0.39103
79.705	-0.48985	-0.39098
79.721	-0.48983	-0.39094
79.737	-0.4898	-0.39091
79.754	-0.48978	-0.39087
79.77	-0.48975	-0.39084
79.787	-0.48973	-0.3908
79.805	-0.4897	-0.39077
79.821	-0.48968	-0.39072
79.838	-0.48966	-0.39064
79.854	-0.48963	-0.39054
79.872	-0.48961	-0.39041
79.888	-0.48959	-0.39027
79.905	-0.48958	-0.39016
79.921	-0.48956	-0.39009
79.938	-0.48954	-0.39005
79.956	-0.48952	-0.38999

79.971	-0.4895	-0.38993
79.989	-0.48947	-0.38989
80.004	-0.48945	-0.38984
80.022	-0.48944	-0.38977
80.038	-0.48941	-0.38969
80.055	-0.4894	-0.38964
80.072	-0.48938	-0.3896
80.089	-0.48936	-0.38954
80.106	-0.48934	-0.38949
80.122	-0.48932	-0.38944
80.139	-0.48929	-0.38938
80.156	-0.48927	-0.38932
80.172	-0.48924	-0.38926
80.189	-0.48921	-0.38918
80.206	-0.48919	-0.38908
80.223	-0.48915	-0.38898
80.239	-0.48912	-0.38889
80.255	-0.4891	-0.3888
80.273	-0.48907	-0.38874
80.289	-0.48905	-0.38869
80.306	-0.48903	-0.38865
80.323	-0.48901	-0.38861
80.339	-0.48899	-0.38856
80.356	-0.48897	-0.38849
80.372	-0.48895	-0.38841
80.39	-0.48893	-0.3883
80.407	-0.48891	-0.3882
80.423	-0.48888	-0.38809
80.439	-0.48886	-0.38796
80.456	-0.48884	-0.38785

80.474	-0.48881	-0.38773
80.49	-0.48879	-0.38767
80.507	-0.48876	-0.38762
80.523	-0.48874	-0.38757
80.54	-0.48872	-0.38749
80.557	-0.48869	-0.38741
80.573	-0.48867	-0.3873
80.59	-0.48865	-0.38723
80.606	-0.48863	-0.38718
80.623	-0.48861	-0.38712
80.64	-0.48859	-0.38708
80.657	-0.48857	-0.38704
80.675	-0.48855	-0.387
80.69	-0.48852	-0.38697
80.707	-0.48849	-0.38693
80.724	-0.48847	-0.38687
80.741	-0.48844	-0.3868
80.758	-0.48841	-0.38669
80.773	-0.48839	-0.38659
80.791	-0.48836	-0.38645
80.807	-0.48834	-0.3863
80.824	-0.48831	-0.38619
80.841	-0.48829	-0.38609
80.858	-0.48828	-0.38601
80.875	-0.48826	-0.38594
80.891	-0.48824	-0.38589
80.908	-0.48823	-0.38584
80.924	-0.48821	-0.3858
80.941	-0.4882	-0.38575
80.957	-0.48818	-0.3857

80.973	-0.48816	-0.38563
80.99	-0.48813	-0.38558
81.007	-0.48811	-0.38554
81.025	-0.48808	-0.3855
81.042	-0.48806	-0.38546
81.057	-0.48803	-0.3854
81.074	-0.48801	-0.38534
81.091	-0.48799	-0.38528
81.108	-0.48798	-0.3852
81.125	-0.48796	-0.3851
81.141	-0.48795	-0.38498
81.158	-0.48794	-0.38484
81.175	-0.48792	-0.3847
81.191	-0.4879	-0.3846
81.208	-0.48788	-0.38452
81.224	-0.48786	-0.38445
81.24	-0.48784	-0.38437
81.258	-0.48782	-0.38429
81.275	-0.4878	-0.38423
81.292	-0.48778	-0.38417
81.308	-0.48776	-0.38412
81.324	-0.48774	-0.38406
81.341	-0.48773	-0.38401
81.358	-0.48771	-0.38396
81.375	-0.48769	-0.38391
81.392	-0.48768	-0.38386
81.408	-0.48766	-0.38381
81.425	-0.48764	-0.38375
81.441	-0.48762	-0.3837
81.458	-0.48759	-0.38364

81.475	-0.48757	-0.38358
81.491	-0.48755	-0.38352
81.508	-0.48753	-0.38347
81.525	-0.48752	-0.38341
81.542	-0.4875	-0.38334
81.559	-0.48748	-0.38326
81.575	-0.48746	-0.38319
81.592	-0.48744	-0.3831
81.609	-0.48742	-0.38304
81.626	-0.4874	-0.383
81.642	-0.48738	-0.38296
81.659	-0.48736	-0.38293
81.675	-0.48733	-0.3829
81.692	-0.48731	-0.38286
81.71	-0.48729	-0.38281
81.726	-0.48726	-0.38272
81.743	-0.48724	-0.38267
81.759	-0.48722	-0.38261
81.776	-0.48721	-0.38249
81.793	-0.48719	-0.38238
81.809	-0.48717	-0.38227
81.826	-0.48715	-0.3822
81.843	-0.48713	-0.38213
81.86	-0.48711	-0.38208
81.876	-0.48709	-0.38201
81.892	-0.48707	-0.38194
81.91	-0.48704	-0.38189
81.926	-0.48702	-0.38184
81.943	-0.487	-0.3818
81.959	-0.48697	-0.38176

81.977	-0.48695	-0.3817
81.993	-0.48693	-0.38162
82.01	-0.48691	-0.38155
82.027	-0.48689	-0.38147
82.043	-0.48687	-0.38141
82.059	-0.48685	-0.38134
82.078	-0.48683	-0.38122
82.094	-0.48681	-0.38109
82.11	-0.48679	-0.38095
82.127	-0.48677	-0.38081
82.144	-0.48674	-0.38071
82.16	-0.48671	-0.38063
82.178	-0.48669	-0.38057
82.195	-0.48666	-0.3805
82.211	-0.48664	-0.38047
82.228	-0.48661	-0.38043
82.245	-0.48659	-0.38035
82.261	-0.48657	-0.38031
82.278	-0.48655	-0.38027
82.295	-0.48653	-0.38021
82.311	-0.48651	-0.38013
82.328	-0.48649	-0.38006
82.345	-0.48646	-0.37997
82.361	-0.48644	-0.37987
82.378	-0.48642	-0.37977
82.395	-0.48639	-0.37968
82.412	-0.48637	-0.3796
82.429	-0.48635	-0.37953
82.445	-0.48633	-0.37944
82.462	-0.48631	-0.37937

82.479	-0.48629	-0.37929
82.496	-0.48627	-0.3792
82.512	-0.48625	-0.37912
82.529	-0.48623	-0.37902
82.545	-0.48621	-0.37892
82.562	-0.48619	-0.37882
82.579	-0.48617	-0.37871
82.595	-0.48615	-0.37861
82.612	-0.48613	-0.37853
82.629	-0.48611	-0.37847
82.645	-0.48609	-0.37839
82.662	-0.48607	-0.37831
82.679	-0.48605	-0.37826
82.695	-0.48603	-0.37821
82.713	-0.486	-0.37816
82.729	-0.48598	-0.3781
82.746	-0.48596	-0.37804
82.763	-0.48594	-0.37796
82.779	-0.48592	-0.37788
82.796	-0.4859	-0.37777
82.812	-0.48588	-0.37769
82.829	-0.48585	-0.37761
82.846	-0.48583	-0.37754
82.862	-0.48581	-0.37747
82.879	-0.48579	-0.37738
82.895	-0.48578	-0.37727
82.912	-0.48576	-0.37719
82.928	-0.48574	-0.3771
82.946	-0.48572	-0.37705
82.962	-0.4857	-0.377

82.98	-0.48568	-0.37694
82.996	-0.48566	-0.3769
83.013	-0.48564	-0.37686
83.029	-0.48562	-0.3768
83.046	-0.48559	-0.37672
83.063	-0.48557	-0.37663
83.08	-0.48554	-0.3765
83.097	-0.48552	-0.37639
83.112	-0.4855	-0.37629
83.13	-0.48548	-0.37616
83.147	-0.48546	-0.37604
83.162	-0.48545	-0.37596
83.179	-0.48543	-0.37591
83.196	-0.48541	-0.37587
83.213	-0.4854	-0.37584
83.229	-0.48537	-0.3758
83.246	-0.48535	-0.37577
83.263	-0.48532	-0.37574
83.28	-0.4853	-0.3757
83.297	-0.48527	-0.37565
83.314	-0.48524	-0.37557
83.33	-0.48522	-0.37545
83.347	-0.48519	-0.3753
83.364	-0.48517	-0.37516
83.38	-0.48515	-0.37501
83.397	-0.48512	-0.37487
83.414	-0.4851	-0.37478
83.43	-0.48508	-0.3747
83.448	-0.48506	-0.37466
83.463	-0.48504	-0.37459

83.479	-0.48502	-0.37453
83.498	-0.485	-0.37448
83.515	-0.48498	-0.37443
83.53	-0.48496	-0.37435
83.547	-0.48494	-0.37429
83.564	-0.48492	-0.37423
83.581	-0.48489	-0.37413
83.597	-0.48486	-0.37406
83.614	-0.48484	-0.37398
83.63	-0.48481	-0.3739
83.647	-0.48479	-0.37381
83.663	-0.48477	-0.37371
83.681	-0.48475	-0.3736
83.698	-0.48472	-0.3735
83.715	-0.4847	-0.37341
83.731	-0.48468	-0.37332
83.748	-0.48465	-0.37326
83.765	-0.48463	-0.37319
83.782	-0.48461	-0.37312
83.798	-0.48458	-0.37306
83.814	-0.48456	-0.37299
83.832	-0.48454	-0.37291
83.849	-0.48452	-0.37283
83.865	-0.4845	-0.37275
83.882	-0.48448	-0.3727
83.899	-0.48446	-0.37264
83.915	-0.48444	-0.37258
83.931	-0.48442	-0.37251
83.948	-0.48439	-0.37243
83.965	-0.48437	-0.37232

83.981	-0.48435	-0.37221
83.999	-0.48434	-0.37214
84.015	-0.48432	-0.37201
84.032	-0.48431	-0.37186
84.048	-0.4843	-0.37171
84.066	-0.48428	-0.37157
84.082	-0.48426	-0.37145
84.099	-0.48424	-0.37136
84.116	-0.48422	-0.37127
84.132	-0.4842	-0.3712
84.148	-0.48418	-0.37113
84.165	-0.48415	-0.37107
84.182	-0.48413	-0.37104
84.199	-0.4841	-0.37101
84.216	-0.48408	-0.37098
84.231	-0.48406	-0.37095
84.249	-0.48404	-0.37092
84.265	-0.48402	-0.37089
84.282	-0.484	-0.37086
84.299	-0.48398	-0.37083
84.316	-0.48395	-0.37079
84.333	-0.48393	-0.37072
84.349	-0.48391	-0.3706
84.366	-0.48389	-0.37039
84.383	-0.48387	-0.37016
84.4	-0.48385	-0.36999
84.416	-0.48384	-0.36986
84.433	-0.48382	-0.36976
84.449	-0.48381	-0.36968
84.465	-0.48379	-0.3696

84.483	-0.48378	-0.36955
84.499	-0.48375	-0.36951
84.516	-0.48374	-0.36947
84.533	-0.48372	-0.36942
84.549	-0.4837	-0.36938
84.566	-0.48368	-0.36932
84.583	-0.48367	-0.36928
84.6	-0.48365	-0.36923
84.617	-0.48363	-0.36919
84.633	-0.48362	-0.36915
84.65	-0.4836	-0.3691
84.666	-0.48358	-0.36903
84.683	-0.48356	-0.36895
84.699	-0.48354	-0.36886
84.716	-0.48351	-0.36878
84.734	-0.4835	-0.36873
84.75	-0.48347	-0.3687
84.767	-0.48346	-0.36865
84.783	-0.48344	-0.3686
84.801	-0.48342	-0.36856
84.817	-0.48341	-0.36851
84.834	-0.48339	-0.36846
84.85	-0.48338	-0.36841
84.867	-0.48336	-0.36836
84.884	-0.48334	-0.36829
84.902	-0.48332	-0.36822
84.917	-0.4833	-0.36812
84.935	-0.48328	-0.36804
84.951	-0.48326	-0.36798
84.968	-0.48324	-0.3679

84.983	-0.48322	-0.36785
85.002	-0.48321	-0.3678
85.018	-0.48319	-0.36775
85.036	-0.48317	-0.3677
85.051	-0.48316	-0.36767
85.068	-0.48314	-0.36764
85.085	-0.48312	-0.36761
85.101	-0.4831	-0.36756
85.118	-0.48308	-0.36747
85.135	-0.48307	-0.36738
85.152	-0.48305	-0.36727
85.168	-0.48303	-0.36718
85.185	-0.48301	-0.36704
85.202	-0.48299	-0.3669
85.218	-0.48297	-0.36674
85.235	-0.48295	-0.3666
85.252	-0.48293	-0.36647
85.269	-0.48291	-0.36636
85.286	-0.48289	-0.36627
85.302	-0.48288	-0.3661
85.318	-0.48286	-0.36593
85.336	-0.48284	-0.36584
85.352	-0.48282	-0.36578
85.37	-0.48281	-0.36573
85.386	-0.48279	-0.36569
85.403	-0.48277	-0.36566
85.42	-0.48275	-0.36562
85.436	-0.48273	-0.36557
85.453	-0.48271	-0.36551
85.47	-0.48269	-0.36545

85.487	-0.48267	-0.36536
85.503	-0.48265	-0.36526
85.519	-0.48264	-0.36517
85.537	-0.48262	-0.36509
85.554	-0.48261	-0.36501
85.57	-0.48259	-0.36492
85.586	-0.48257	-0.36484
85.604	-0.48256	-0.36476
85.619	-0.48254	-0.36468
85.636	-0.48252	-0.36457
85.653	-0.48251	-0.36445
85.67	-0.48249	-0.36437
85.687	-0.48247	-0.36428
85.703	-0.48245	-0.36423
85.72	-0.48243	-0.36418
85.737	-0.48241	-0.36415
85.753	-0.48239	-0.36412
85.771	-0.48238	-0.36404
85.787	-0.48236	-0.36396
85.803	-0.48235	-0.36389
85.82	-0.48233	-0.36379
85.837	-0.48232	-0.36363
85.853	-0.48231	-0.36348
85.87	-0.48229	-0.36333
85.887	-0.48227	-0.3632
85.903	-0.48226	-0.36312
85.92	-0.48224	-0.36308
85.937	-0.48222	-0.36302
85.954	-0.4822	-0.36296
85.97	-0.48218	-0.3629

85.987	-0.48217	-0.36285
86.005	-0.48215	-0.36281
86.021	-0.48214	-0.36277
86.038	-0.48212	-0.36273
86.054	-0.48211	-0.36267
86.07	-0.48209	-0.36258
86.087	-0.48208	-0.36249
86.104	-0.48206	-0.36241
86.121	-0.48205	-0.36234
86.138	-0.48203	-0.36224
86.154	-0.48202	-0.36215
86.171	-0.482	-0.36209
86.188	-0.48199	-0.36202
86.204	-0.48197	-0.36194
86.222	-0.48195	-0.36189
86.239	-0.48193	-0.36181
86.255	-0.48191	-0.36169
86.272	-0.48189	-0.36159
86.289	-0.48187	-0.36149
86.305	-0.48185	-0.3614
86.322	-0.48183	-0.36133
86.338	-0.48181	-0.36124
86.356	-0.48179	-0.36116
86.373	-0.48177	-0.3611
86.389	-0.48175	-0.36104
86.405	-0.48174	-0.36099
86.422	-0.48172	-0.36091
86.439	-0.48171	-0.3608
86.455	-0.48169	-0.36072
86.472	-0.48168	-0.36066

86.488	-0.48167	-0.36058
86.505	-0.48165	-0.36053
86.523	-0.48164	-0.36049
86.54	-0.48162	-0.36045
86.556	-0.48161	-0.3604
86.572	-0.4816	-0.36036
86.589	-0.48159	-0.36032
86.606	-0.48157	-0.36027
86.623	-0.48156	-0.36015
86.639	-0.48155	-0.35999
86.656	-0.48153	-0.35987
86.672	-0.48152	-0.35972
86.689	-0.48151	-0.35961
86.705	-0.48149	-0.35958
86.723	-0.48147	-0.35953
86.74	-0.48146	-0.35949
86.756	-0.48145	-0.35944
86.772	-0.48144	-0.35939
86.789	-0.48143	-0.35935
86.806	-0.48142	-0.3593
86.823	-0.48141	-0.35925
86.84	-0.48139	-0.35918
86.857	-0.48138	-0.35908
86.874	-0.48136	-0.35897
86.891	-0.48135	-0.3589
86.907	-0.48134	-0.35883
86.923	-0.48133	-0.35876
86.94	-0.48132	-0.35867
86.957	-0.48131	-0.35856
86.974	-0.4813	-0.35848

86.991	-0.48129	-0.35839
87.007	-0.48128	-0.35833
87.023	-0.48127	-0.35829
87.041	-0.48126	-0.35823
87.058	-0.48125	-0.35815
87.075	-0.48124	-0.35805
87.091	-0.48123	-0.35794
87.108	-0.48122	-0.35786
87.124	-0.48121	-0.35777
87.14	-0.4812	-0.35771
87.157	-0.48119	-0.35766
87.174	-0.48118	-0.35761
87.192	-0.48117	-0.35756
87.208	-0.48116	-0.35753
87.225	-0.48115	-0.3575
87.242	-0.48114	-0.35747
87.259	-0.48113	-0.35741
87.276	-0.48112	-0.35732
87.292	-0.48111	-0.35724
87.309	-0.48109	-0.35717
87.326	-0.48108	-0.35709
87.342	-0.48107	-0.35705
87.359	-0.48106	-0.35699
87.375	-0.48105	-0.35691
87.392	-0.48104	-0.35683
87.408	-0.48102	-0.35675
87.426	-0.48101	-0.35666
87.442	-0.481	-0.35653
87.458	-0.48099	-0.35638
87.476	-0.48099	-0.35623

87.492	-0.48098	-0.35609
87.509	-0.48097	-0.35594
87.526	-0.48095	-0.35582
87.543	-0.48094	-0.35573
87.559	-0.48092	-0.35561
87.576	-0.48091	-0.35554
87.593	-0.48089	-0.35547
87.61	-0.48088	-0.3554
87.626	-0.48086	-0.35534
87.643	-0.48085	-0.35526
87.659	-0.48083	-0.35518
87.676	-0.48082	-0.35511
87.692	-0.4808	-0.35503
87.71	-0.48079	-0.35495
87.726	-0.48078	-0.35489
87.743	-0.48076	-0.35483
87.76	-0.48074	-0.35478
87.777	-0.48073	-0.35472
87.794	-0.48071	-0.35466
87.81	-0.4807	-0.35459
87.826	-0.48069	-0.35448
87.843	-0.48067	-0.35437
87.86	-0.48066	-0.35426
87.877	-0.48065	-0.35415
87.893	-0.48064	-0.35405
87.91	-0.48063	-0.35396
87.926	-0.48062	-0.35388
87.942	-0.4806	-0.35381
87.96	-0.48059	-0.35373
87.977	-0.48057	-0.35366

87.994	-0.48056	-0.35357
88.011	-0.48055	-0.35346
88.027	-0.48054	-0.35337
88.044	-0.48053	-0.35327
88.06	-0.48052	-0.35314
88.078	-0.48051	-0.35298
88.094	-0.4805	-0.35284
88.11	-0.48048	-0.35275
88.128	-0.48047	-0.35268
88.143	-0.48046	-0.35261
88.161	-0.48044	-0.35253
88.177	-0.48043	-0.35246
88.194	-0.48041	-0.35239
88.211	-0.4804	-0.35236
88.227	-0.48038	-0.35232
88.244	-0.48037	-0.35226
88.262	-0.48036	-0.35219
88.277	-0.48034	-0.35212
88.295	-0.48033	-0.35205
88.311	-0.48031	-0.35199
88.328	-0.4803	-0.35193
88.345	-0.48028	-0.35184
88.361	-0.48027	-0.35175
88.378	-0.48025	-0.35169
88.395	-0.48024	-0.35163
88.412	-0.48022	-0.35158
88.427	-0.48021	-0.35152
88.444	-0.48019	-0.35147
88.462	-0.48018	-0.35138
88.479	-0.48017	-0.35127

88.495	-0.48016	-0.35115
88.512	-0.48015	-0.351
88.529	-0.48014	-0.35085
88.545	-0.48013	-0.35076
88.562	-0.48012	-0.35067
88.578	-0.48011	-0.35059
88.595	-0.48009	-0.35052
88.612	-0.48008	-0.35045
88.629	-0.48006	-0.35041
88.645	-0.48004	-0.35036
88.662	-0.48003	-0.35032
88.679	-0.48001	-0.35028
88.695	-0.47999	-0.35024
88.712	-0.47998	-0.35017
88.728	-0.47996	-0.35012
88.746	-0.47995	-0.35005
88.763	-0.47993	-0.34997
88.779	-0.47992	-0.3499
88.796	-0.47991	-0.34983
88.813	-0.4799	-0.34975
88.829	-0.4799	-0.34968
88.846	-0.47989	-0.34962
88.863	-0.47988	-0.34957
88.879	-0.47986	-0.3495
88.895	-0.47985	-0.34942
88.913	-0.47983	-0.34933
88.93	-0.47982	-0.34921
88.946	-0.47981	-0.34908
88.963	-0.4798	-0.34897
88.98	-0.47978	-0.34885

88.997	-0.47978	-0.34869
89.013	-0.47977	-0.34856
89.03	-0.47976	-0.34848
89.046	-0.47975	-0.34844
89.063	-0.47974	-0.3484
89.079	-0.47974	-0.34837
89.096	-0.47973	-0.34829
89.113	-0.47972	-0.34826
89.13	-0.4797	-0.34823
89.146	-0.47969	-0.3482
89.163	-0.47968	-0.34815
89.181	-0.47967	-0.34805
89.196	-0.47966	-0.34793
89.214	-0.47965	-0.34783
89.23	-0.47964	-0.34772
89.246	-0.47963	-0.34759
89.264	-0.47961	-0.34751
89.28	-0.4796	-0.34743
89.298	-0.47959	-0.34736
89.313	-0.47958	-0.34729
89.331	-0.47957	-0.34724
89.348	-0.47956	-0.34716
89.364	-0.47955	-0.34709
89.38	-0.47954	-0.34702
89.396	-0.47953	-0.34698
89.414	-0.47952	-0.34694
89.43	-0.47952	-0.34692
89.448	-0.4795	-0.34689
89.464	-0.47949	-0.34684
89.481	-0.47948	-0.34679

89.498	-0.47948	-0.3467
89.515	-0.47947	-0.3466
89.531	-0.47946	-0.3464
89.548	-0.47946	-0.34625
89.564	-0.47945	-0.34615
89.581	-0.47944	-0.34606
89.598	-0.47943	-0.34597
89.615	-0.47942	-0.34591
89.631	-0.47941	-0.34585
89.648	-0.4794	-0.34582
89.665	-0.47939	-0.34578
89.682	-0.47938	-0.34573
89.699	-0.47936	-0.34565
89.715	-0.47935	-0.34555
89.732	-0.47934	-0.34547
89.749	-0.47933	-0.3454
89.765	-0.47932	-0.34535
89.781	-0.47931	-0.34527
89.799	-0.4793	-0.34517
89.816	-0.47929	-0.34507
89.832	-0.47928	-0.34497
89.849	-0.47927	-0.34485
89.865	-0.47926	-0.34477
89.882	-0.47925	-0.3447
89.898	-0.47924	-0.3446
89.915	-0.47923	-0.34454
89.932	-0.47922	-0.3445
89.949	-0.47921	-0.34447
89.966	-0.4792	-0.34442
89.982	-0.47919	-0.34438

90	-0.47918	-0.34433
90.016	-0.47917	-0.34425
90.033	-0.47916	-0.34414
90.05	-0.47915	-0.34403
90.066	-0.47915	-0.34395
90.083	-0.47914	-0.34383
90.1	-0.47913	-0.34368
90.116	-0.47912	-0.34358
90.134	-0.47912	-0.34349
90.15	-0.47911	-0.34341
90.166	-0.4791	-0.34332
90.183	-0.4791	-0.34326
90.199	-0.47908	-0.34317
90.217	-0.47907	-0.34308
90.233	-0.47906	-0.34301
90.25	-0.47905	-0.34297
90.266	-0.47904	-0.3429
90.283	-0.47902	-0.34282
90.3	-0.47901	-0.34274
90.317	-0.479	-0.34269
90.333	-0.47899	-0.34262
90.35	-0.47898	-0.34256
90.368	-0.47896	-0.34251
90.383	-0.47895	-0.34242
90.4	-0.47894	-0.34233
90.417	-0.47893	-0.34227
90.434	-0.47892	-0.3422
90.451	-0.47891	-0.34213
90.468	-0.4789	-0.34205
90.484	-0.47889	-0.34195

90.501	-0.47889	-0.34186
90.517	-0.47888	-0.34177
90.534	-0.47888	-0.34168
90.55	-0.47887	-0.3416
90.567	-0.47886	-0.34153
90.586	-0.47885	-0.34144
90.601	-0.47884	-0.34135
90.617	-0.47883	-0.34125
90.634	-0.47881	-0.34117
90.651	-0.4788	-0.3411
90.667	-0.47878	-0.34102
90.685	-0.47877	-0.34094
90.701	-0.47875	-0.34088
90.718	-0.47873	-0.34081
90.734	-0.47872	-0.34073
90.751	-0.47871	-0.34064
90.768	-0.4787	-0.34057
90.784	-0.4787	-0.34049
90.801	-0.47869	-0.34042
90.817	-0.47869	-0.34035
90.834	-0.47868	-0.3403
90.851	-0.47868	-0.34025
90.868	-0.47867	-0.3402
90.885	-0.47866	-0.34014
90.902	-0.47865	-0.3401
90.918	-0.47864	-0.34005
90.935	-0.47864	-0.33999
90.952	-0.47863	-0.33993
90.969	-0.47862	-0.33985
90.985	-0.47862	-0.33976

91.002	-0.47861	-0.33967
91.019	-0.47859	-0.33957
91.037	-0.47858	-0.33949
91.053	-0.47857	-0.33942
91.069	-0.47856	-0.33932
91.086	-0.47855	-0.33922
91.103	-0.47854	-0.33909
91.119	-0.47853	-0.33893
91.135	-0.47852	-0.33878
91.152	-0.47852	-0.33865
91.169	-0.47851	-0.33853
91.185	-0.4785	-0.33847
91.203	-0.47849	-0.33842
91.219	-0.47848	-0.33838
91.236	-0.47846	-0.33835
91.253	-0.47846	-0.33833
91.269	-0.47845	-0.3383
91.286	-0.47844	-0.33827
91.302	-0.47843	-0.33822
91.32	-0.47842	-0.33815
91.337	-0.47842	-0.33806
91.352	-0.47841	-0.33797
91.369	-0.4784	-0.33789
91.386	-0.47839	-0.33782
91.402	-0.47837	-0.33774
91.419	-0.47837	-0.33764
91.436	-0.47836	-0.33755
91.454	-0.47835	-0.33746
91.47	-0.47835	-0.33737
91.487	-0.47834	-0.33729

91.504	-0.47833	-0.33722
91.52	-0.47833	-0.33715
91.537	-0.47832	-0.33709
91.554	-0.47831	-0.33704
91.57	-0.47831	-0.33701
91.588	-0.4783	-0.33696
91.603	-0.47829	-0.33692
91.62	-0.47828	-0.33687
91.637	-0.47827	-0.33684
91.654	-0.47825	-0.33678
91.67	-0.47824	-0.3367
91.687	-0.47823	-0.33665
91.704	-0.47822	-0.33659
91.721	-0.47821	-0.3365
91.737	-0.47821	-0.33641
91.754	-0.4782	-0.33632
91.771	-0.4782	-0.33621
91.787	-0.4782	-0.33609
91.805	-0.4782	-0.33601
91.822	-0.4782	-0.33594
91.838	-0.47819	-0.33587
91.855	-0.47819	-0.33583
91.871	-0.47818	-0.33578
91.888	-0.47818	-0.33573
91.906	-0.47817	-0.33567
91.922	-0.47816	-0.33563
91.938	-0.47815	-0.33556
91.955	-0.47814	-0.33548
91.971	-0.47813	-0.33538
91.987	-0.47812	-0.33528

92.005	-0.47811	-0.33517
92.021	-0.47811	-0.33502
92.038	-0.4781	-0.33491
92.054	-0.47809	-0.3348
92.072	-0.47809	-0.33471
92.089	-0.47808	-0.3346
92.105	-0.47808	-0.33453
92.122	-0.47808	-0.33448
92.139	-0.47808	-0.33445
92.155	-0.47808	-0.33441
92.172	-0.47807	-0.33437
92.188	-0.47807	-0.33432
92.205	-0.47806	-0.33425
92.221	-0.47805	-0.33417
92.238	-0.47804	-0.33406
92.255	-0.47803	-0.33397
92.273	-0.47802	-0.33389
92.289	-0.47801	-0.3338
92.305	-0.478	-0.33373
92.323	-0.478	-0.33368
92.339	-0.478	-0.33363
92.355	-0.478	-0.33356
92.373	-0.478	-0.33351
92.389	-0.478	-0.33342
92.406	-0.47799	-0.33329
92.423	-0.47799	-0.33317
92.438	-0.47798	-0.33308
92.455	-0.47798	-0.33297
92.473	-0.47797	-0.33289
92.489	-0.47796	-0.33282

92.506	-0.47795	-0.33276
92.523	-0.47795	-0.33269
92.539	-0.47795	-0.33264
92.556	-0.47794	-0.33258
92.572	-0.47794	-0.33252
92.59	-0.47794	-0.33246
92.607	-0.47794	-0.33239
92.623	-0.47794	-0.33233
92.64	-0.47794	-0.33229
92.657	-0.47794	-0.33224
92.673	-0.47794	-0.33221
92.69	-0.47794	-0.33218
92.707	-0.47794	-0.33212
92.723	-0.47794	-0.33205
92.739	-0.47793	-0.33196
92.757	-0.47792	-0.33187
92.773	-0.47792	-0.33173
92.79	-0.47791	-0.3316
92.807	-0.4779	-0.33147
92.824	-0.4779	-0.33137
92.84	-0.47789	-0.33129
92.857	-0.47788	-0.33125
92.873	-0.47787	-0.33122
92.89	-0.47787	-0.33113
92.907	-0.47786	-0.33107
92.924	-0.47785	-0.33099
92.94	-0.47785	-0.33097
92.957	-0.47785	-0.33093
92.975	-0.47785	-0.33088
92.992	-0.47785	-0.33083

93.008	-0.47785	-0.33079
93.025	-0.47785	-0.3307
93.041	-0.47785	-0.33061
93.058	-0.47785	-0.33048
93.074	-0.47784	-0.33033
93.091	-0.47783	-0.33016
93.107	-0.47783	-0.32994
93.124	-0.47782	-0.32978
93.142	-0.47782	-0.32969
93.158	-0.47781	-0.32962
93.176	-0.4778	-0.32957
93.191	-0.4778	-0.32954
93.208	-0.47779	-0.32949
93.225	-0.47779	-0.32947
93.242	-0.47779	-0.32941
93.258	-0.47779	-0.32936
93.276	-0.47778	-0.3293
93.292	-0.47778	-0.32924
93.309	-0.47778	-0.32918
93.324	-0.47777	-0.32913
93.342	-0.47777	-0.32909
93.359	-0.47777	-0.32903
93.375	-0.47776	-0.32898
93.392	-0.47776	-0.32892
93.409	-0.47775	-0.32887
93.426	-0.47775	-0.32874
93.443	-0.47774	-0.32861
93.459	-0.47773	-0.32848
93.476	-0.47773	-0.32838
93.492	-0.47772	-0.32825

93.509	-0.47772	-0.32815
93.526	-0.47772	-0.32802
93.543	-0.47772	-0.32791
93.56	-0.47772	-0.32786
93.576	-0.47772	-0.32782
93.592	-0.47772	-0.32778
93.609	-0.47771	-0.32773
93.626	-0.47771	-0.32768
93.643	-0.4777	-0.32761
93.66	-0.4777	-0.32756
93.676	-0.47769	-0.32748
93.693	-0.47769	-0.32738
93.709	-0.47768	-0.32727
93.726	-0.47768	-0.32714
93.742	-0.47768	-0.327
93.759	-0.47767	-0.32687
93.776	-0.47767	-0.32674
93.792	-0.47766	-0.32666
93.81	-0.47766	-0.3266
93.827	-0.47765	-0.32654
93.842	-0.47765	-0.32648
93.86	-0.47764	-0.32644
93.876	-0.47764	-0.32641
93.893	-0.47764	-0.32637
93.91	-0.47764	-0.32633
93.926	-0.47764	-0.32628
93.944	-0.47763	-0.32621
93.961	-0.47763	-0.32615
93.977	-0.47763	-0.32608
93.994	-0.47763	-0.32603

94.011	-0.47763	-0.32598
94.027	-0.47762	-0.32591
94.043	-0.47762	-0.32585
94.06	-0.47762	-0.3258
94.077	-0.47762	-0.32575
94.093	-0.47762	-0.32566
94.11	-0.47762	-0.32556
94.127	-0.47762	-0.32542
94.143	-0.47762	-0.3253
94.16	-0.47761	-0.32521
94.178	-0.47761	-0.32515
94.194	-0.47761	-0.32511
94.21	-0.4776	-0.32507
94.227	-0.4776	-0.32503
94.245	-0.4776	-0.32499
94.261	-0.4776	-0.32495
94.278	-0.4776	-0.32488
94.293	-0.47761	-0.3248
94.311	-0.4776	-0.32469
94.327	-0.4776	-0.32458
94.344	-0.47759	-0.32448
94.36	-0.47759	-0.32441
94.378	-0.47759	-0.32435
94.394	-0.47758	-0.32432
94.411	-0.47758	-0.32429
94.428	-0.47758	-0.32424
94.444	-0.47758	-0.32418
94.461	-0.47758	-0.32412
94.477	-0.47758	-0.32407
94.494	-0.47758	-0.324

94.511	-0.47758	-0.32394
94.528	-0.47758	-0.32386
94.546	-0.47758	-0.3238
94.562	-0.47757	-0.32374
94.579	-0.47757	-0.32369
94.595	-0.47757	-0.32359
94.612	-0.47756	-0.32347
94.629	-0.47756	-0.32337
94.645	-0.47756	-0.32329
94.662	-0.47755	-0.32323
94.678	-0.47755	-0.32315
94.695	-0.47755	-0.32308
94.711	-0.47755	-0.32303
94.728	-0.47755	-0.323
94.745	-0.47755	-0.32297
94.762	-0.47755	-0.32292
94.778	-0.47756	-0.32289
94.795	-0.47756	-0.32284
94.812	-0.47756	-0.32276
94.829	-0.47757	-0.32269
94.845	-0.47756	-0.3226
94.861	-0.47756	-0.32252
94.878	-0.47756	-0.32246
94.895	-0.47755	-0.32239
94.912	-0.47755	-0.3223
94.929	-0.47755	-0.32222
94.945	-0.47755	-0.3221
94.962	-0.47755	-0.32201
94.979	-0.47755	-0.32192
94.996	-0.47754	-0.32184

95.012	-0.47755	-0.32177
95.03	-0.47754	-0.32171
95.046	-0.47754	-0.32164
95.062	-0.47755	-0.32159
95.079	-0.47755	-0.32153
95.096	-0.47755	-0.32145
95.113	-0.47755	-0.32136
95.13	-0.47755	-0.32128
95.146	-0.47755	-0.32121
95.163	-0.47755	-0.32113
95.18	-0.47755	-0.32106
95.196	-0.47755	-0.32096
95.213	-0.47755	-0.32087
95.229	-0.47755	-0.3208
95.247	-0.47755	-0.32073
95.263	-0.47755	-0.32068
95.281	-0.47754	-0.32064
95.297	-0.47754	-0.32059
95.313	-0.47754	-0.32055
95.33	-0.47754	-0.32049
95.348	-0.47754	-0.3204
95.364	-0.47754	-0.32028
95.38	-0.47755	-0.32014
95.396	-0.47755	-0.31997
95.413	-0.47755	-0.31981
95.43	-0.47755	-0.31968
95.446	-0.47756	-0.31954
95.462	-0.47756	-0.31947
95.48	-0.47756	-0.31942
95.497	-0.47756	-0.31936

95.514	-0.47756	-0.31928
95.53	-0.47756	-0.31921
95.547	-0.47756	-0.31916
95.564	-0.47756	-0.31911
95.58	-0.47756	-0.31904
95.597	-0.47756	-0.31897
95.614	-0.47757	-0.3189
95.631	-0.47757	-0.31879
95.647	-0.47757	-0.31871
95.664	-0.47757	-0.31863
95.68	-0.47756	-0.31855
95.697	-0.47756	-0.31847
95.714	-0.47755	-0.31842
95.731	-0.47755	-0.31838
95.747	-0.47755	-0.31835
95.763	-0.47756	-0.31832
95.781	-0.47757	-0.31829
95.799	-0.47757	-0.31824
95.815	-0.47758	-0.31818
95.831	-0.47759	-0.31813
95.848	-0.4776	-0.31804
95.865	-0.4776	-0.31791
95.881	-0.47761	-0.31778
95.898	-0.47761	-0.31767
95.915	-0.47761	-0.31758
95.931	-0.47761	-0.31747
95.948	-0.47761	-0.31741
95.965	-0.47762	-0.31736
95.981	-0.47762	-0.31729
95.997	-0.47762	-0.31718

96.015	-0.47762	-0.31711
96.032	-0.47762	-0.31705
96.048	-0.47762	-0.31699
96.065	-0.47762	-0.31694
96.08	-0.47763	-0.31687
96.098	-0.47763	-0.31683
96.114	-0.47764	-0.31676
96.131	-0.47764	-0.31668
96.148	-0.47766	-0.31664
96.165	-0.47767	-0.31661
96.181	-0.47768	-0.31658
96.198	-0.47769	-0.31652
96.216	-0.47769	-0.31646
96.232	-0.47769	-0.31639
96.249	-0.4777	-0.31634
96.266	-0.4777	-0.31629
96.281	-0.4777	-0.31624
96.298	-0.47771	-0.31616
96.315	-0.47771	-0.31608
96.331	-0.47772	-0.31604
96.349	-0.47773	-0.31599
96.366	-0.47773	-0.31596
96.382	-0.47774	-0.31592
96.399	-0.47774	-0.31589
96.416	-0.47775	-0.31584
96.433	-0.47776	-0.31578
96.45	-0.47777	-0.3157
96.466	-0.47778	-0.31561
96.483	-0.47779	-0.31552
96.499	-0.4778	-0.31544

96.515	-0.47781	-0.31536
96.532	-0.47781	-0.31525
96.549	-0.47782	-0.31515
96.566	-0.47783	-0.31505
96.583	-0.47783	-0.31498
96.599	-0.47783	-0.31487
96.616	-0.47783	-0.31482
96.632	-0.47783	-0.31476
96.649	-0.47784	-0.31468
96.666	-0.47784	-0.3146
96.683	-0.47785	-0.31454
96.701	-0.47785	-0.31449
96.716	-0.47786	-0.31443
96.733	-0.47786	-0.31438
96.75	-0.47787	-0.31435
96.766	-0.47787	-0.31431
96.784	-0.47788	-0.31427
96.801	-0.47789	-0.31423
96.816	-0.4779	-0.31418
96.833	-0.47791	-0.31415
96.85	-0.47792	-0.31412
96.867	-0.47792	-0.31406
96.884	-0.47793	-0.31399
96.9	-0.47794	-0.31393
96.918	-0.47794	-0.31387
96.934	-0.47795	-0.31382
96.95	-0.47795	-0.31377
96.967	-0.47795	-0.31372
96.984	-0.47796	-0.31365
97	-0.47797	-0.31358

97.017	-0.47798	-0.31348
97.033	-0.47799	-0.31341
97.05	-0.478	-0.31334
97.068	-0.47801	-0.31327
97.085	-0.47802	-0.31317
97.101	-0.47802	-0.3131
97.117	-0.47803	-0.31302
97.133	-0.47803	-0.31292
97.151	-0.47803	-0.31283
97.167	-0.47803	-0.31275
97.184	-0.47804	-0.31268
97.202	-0.47804	-0.3126
97.217	-0.47805	-0.3125
97.235	-0.47806	-0.31241
97.251	-0.47808	-0.31232
97.268	-0.47809	-0.31223
97.285	-0.4781	-0.31218
97.3	-0.47811	-0.31212
97.318	-0.47812	-0.31205
97.334	-0.47812	-0.31199
97.352	-0.47813	-0.31194
97.368	-0.47814	-0.31188
97.385	-0.47816	-0.31179
97.402	-0.47817	-0.31167
97.419	-0.47818	-0.31151
97.435	-0.47819	-0.31134
97.451	-0.4782	-0.31121
97.468	-0.4782	-0.31108
97.484	-0.4782	-0.31101
97.502	-0.47821	-0.31091

97.519	-0.47821	-0.31087
97.535	-0.47822	-0.31084
97.552	-0.47822	-0.31081
97.569	-0.47823	-0.31078
97.585	-0.47824	-0.31073
97.603	-0.47825	-0.31069
97.619	-0.47826	-0.31061
97.636	-0.47827	-0.31056
97.652	-0.47828	-0.3105
97.668	-0.4783	-0.31043
97.685	-0.47831	-0.31033
97.702	-0.47832	-0.31024
97.719	-0.47833	-0.31012
97.736	-0.47834	-0.31002
97.752	-0.47835	-0.30996
97.769	-0.47836	-0.30989
97.786	-0.47836	-0.30985
97.802	-0.47837	-0.30981
97.818	-0.47837	-0.30976
97.836	-0.47838	-0.3097
97.852	-0.47839	-0.30964
97.869	-0.47841	-0.30957
97.886	-0.47842	-0.3095
97.902	-0.47843	-0.30943
97.919	-0.47844	-0.30937
97.936	-0.47845	-0.30932
97.954	-0.47846	-0.30925
97.97	-0.47847	-0.30919
97.987	-0.47848	-0.30912
98.003	-0.47848	-0.30903

98.021	-0.47848	-0.30894
98.037	-0.47849	-0.30882
98.053	-0.4785	-0.30873
98.07	-0.47851	-0.30866
98.086	-0.47852	-0.30859
98.103	-0.47853	-0.3085
98.119	-0.47854	-0.30847
98.136	-0.47856	-0.30841
98.153	-0.47857	-0.30836
98.17	-0.47858	-0.30832
98.186	-0.47859	-0.30828
98.204	-0.4786	-0.30825
98.22	-0.47861	-0.30821
98.237	-0.47862	-0.30817
98.254	-0.47863	-0.30813
98.27	-0.47865	-0.30809
98.288	-0.47866	-0.30801
98.303	-0.47868	-0.30796
98.32	-0.47869	-0.30787
98.337	-0.4787	-0.30776
98.354	-0.47871	-0.30767
98.37	-0.47872	-0.30761
98.388	-0.47873	-0.30754
98.404	-0.47874	-0.30746
98.421	-0.47875	-0.3074
98.437	-0.47877	-0.30734
98.454	-0.47878	-0.30727
98.47	-0.47879	-0.30719
98.487	-0.47881	-0.3071
98.504	-0.47882	-0.30703

98.522	-0.47883	-0.30696
98.538	-0.47884	-0.30689
98.554	-0.47885	-0.30682
98.571	-0.47886	-0.30675
98.588	-0.47887	-0.30668
98.604	-0.47889	-0.30664
98.621	-0.47891	-0.3066
98.638	-0.47892	-0.30652
98.655	-0.47894	-0.30643
98.671	-0.47895	-0.30637
98.688	-0.47897	-0.30631
98.705	-0.47898	-0.30625
98.722	-0.47899	-0.30618
98.738	-0.479	-0.30609
98.756	-0.47901	-0.30604
98.771	-0.47902	-0.30601
98.789	-0.47903	-0.30597
98.804	-0.47905	-0.30593
98.822	-0.47906	-0.30587
98.839	-0.47908	-0.30578
98.855	-0.4791	-0.30571
98.871	-0.47911	-0.30561
98.889	-0.47913	-0.30551
98.905	-0.47915	-0.30541
98.922	-0.47917	-0.30533
98.938	-0.47919	-0.30527
98.955	-0.4792	-0.30522
98.972	-0.47922	-0.30518
98.989	-0.47923	-0.30515
99.006	-0.47925	-0.30511

99.022	-0.47926	-0.30507
99.039	-0.47927	-0.30498
99.056	-0.47928	-0.30489
99.073	-0.47929	-0.3048
99.089	-0.4793	-0.3047
99.106	-0.47932	-0.3046
99.122	-0.47934	-0.30452
99.139	-0.47936	-0.30443
99.156	-0.47937	-0.30435
99.172	-0.47939	-0.30428
99.189	-0.47941	-0.30422
99.206	-0.47942	-0.30417
99.222	-0.47944	-0.30412
99.239	-0.47946	-0.30408
99.256	-0.47947	-0.30402
99.273	-0.47949	-0.30397
99.289	-0.47951	-0.30392
99.307	-0.47953	-0.30388
99.323	-0.47955	-0.30384
99.339	-0.47957	-0.30377
99.357	-0.47958	-0.30369
99.373	-0.4796	-0.3036
99.389	-0.47962	-0.30352
99.406	-0.47963	-0.30344
99.423	-0.47965	-0.30336
99.44	-0.47966	-0.3033
99.456	-0.47968	-0.30324
99.474	-0.4797	-0.30318
99.491	-0.47971	-0.30314
99.506	-0.47973	-0.30311

99.525	-0.47974	-0.30307
99.54	-0.47976	-0.30304
99.557	-0.47978	-0.30296
99.574	-0.47979	-0.30287
99.59	-0.47981	-0.30275
99.607	-0.47983	-0.3026
99.623	-0.47985	-0.30249
99.64	-0.47986	-0.3024
99.657	-0.47989	-0.30228
99.673	-0.47991	-0.30221
99.691	-0.47993	-0.30217
99.707	-0.47995	-0.30213
99.723	-0.47997	-0.3021
99.741	-0.47999	-0.30207
99.757	-0.48001	-0.30203
99.774	-0.48003	-0.30199
99.791	-0.48005	-0.30195
99.808	-0.48007	-0.3019
99.825	-0.48009	-0.30185
99.842	-0.48012	-0.3018
99.858	-0.48014	-0.30176
99.875	-0.48016	-0.30171
99.89	-0.48018	-0.30164
99.908	-0.48019	-0.30156
99.925	-0.48021	-0.30147
99.94	-0.48022	-0.30138
99.957	-0.48024	-0.30129
99.974	-0.48025	-0.30118
99.991	-0.48027	-0.30108
100.007	-0.48029	-0.30099

100.025	-0.48032	-0.30091
100.042	-0.48035	-0.30083
100.058	-0.48037	-0.30077
100.075	-0.48039	-0.30069
100.091	-0.48041	-0.3006
100.109	-0.48044	-0.30049
100.124	-0.48046	-0.30041
100.142	-0.48048	-0.30032
100.159	-0.4805	-0.30022
100.175	-0.48052	-0.30016
100.192	-0.48055	-0.30008
100.209	-0.48057	-0.30001
100.226	-0.48059	-0.29994
100.242	-0.48062	-0.29988
100.259	-0.48064	-0.29983
100.275	-0.48066	-0.29977
100.293	-0.48068	-0.29972
100.308	-0.4807	-0.29968
100.326	-0.48073	-0.29962
100.343	-0.48075	-0.29956
100.359	-0.48077	-0.29951
100.377	-0.48079	-0.29943
100.393	-0.48081	-0.29936
100.41	-0.48083	-0.2993
100.426	-0.48085	-0.29924
100.443	-0.48087	-0.2992
100.46	-0.48089	-0.29916
100.476	-0.48092	-0.29913
100.493	-0.48094	-0.2991
100.51	-0.48096	-0.29905

100.527	-0.48099	-0.29898
100.543	-0.48101	-0.29888
100.56	-0.48103	-0.29876
100.576	-0.48106	-0.29858
100.592	-0.48108	-0.29843
100.609	-0.4811	-0.2983
100.626	-0.48113	-0.29823
100.643	-0.48115	-0.29818
100.66	-0.48118	-0.29813
100.677	-0.4812	-0.2981
100.692	-0.48123	-0.29807
100.71	-0.48126	-0.29803
100.726	-0.48128	-0.298
100.743	-0.48131	-0.29796
100.76	-0.48133	-0.2979
100.777	-0.48135	-0.29782
100.793	-0.48137	-0.29769
100.81	-0.4814	-0.29754
100.827	-0.48142	-0.29739
100.844	-0.48144	-0.29724
100.86	-0.48145	-0.29707
100.877	-0.48147	-0.29695
100.893	-0.4815	-0.29687
100.91	-0.48152	-0.29681
100.927	-0.48154	-0.29675
100.943	-0.48157	-0.29669
100.96	-0.48159	-0.29664
100.977	-0.48162	-0.29658
100.994	-0.48164	-0.29646
101.01	-0.48167	-0.29638

101.027	-0.48169	-0.2963
101.043	-0.48171	-0.29621
101.061	-0.48173	-0.29613
101.078	-0.48175	-0.29606
101.094	-0.48177	-0.29602
101.11	-0.48179	-0.29599
101.128	-0.48182	-0.29596
101.143	-0.48185	-0.29593
101.161	-0.48187	-0.29586
101.177	-0.48191	-0.29579
101.194	-0.48196	-0.29576
101.211	-0.48199	-0.29573
101.228	-0.48202	-0.29568
101.244	-0.48205	-0.2956
101.262	-0.48207	-0.2955
101.277	-0.48209	-0.29539
101.294	-0.48211	-0.29527
101.311	-0.48213	-0.29518
101.328	-0.48215	-0.29509
101.345	-0.48217	-0.295
101.36	-0.4822	-0.29496
101.377	-0.48222	-0.29493
101.394	-0.48225	-0.2949
101.412	-0.48229	-0.29486
101.429	-0.48237	-0.29483
101.446	-0.48242	-0.29478
101.462	-0.48246	-0.29473
101.478	-0.4825	-0.29468
101.496	-0.48254	-0.29461
101.512	-0.48258	-0.29453

101.528	-0.48262	-0.29444
101.544	-0.48265	-0.29436
101.562	-0.48268	-0.29429
101.578	-0.48271	-0.29423
101.595	-0.48274	-0.2942
101.612	-0.48277	-0.29416
101.628	-0.4828	-0.29412
101.645	-0.48283	-0.29408
101.662	-0.48285	-0.29403
101.678	-0.48288	-0.29399
101.695	-0.48291	-0.29396
101.712	-0.48293	-0.29391
101.728	-0.48296	-0.29384
101.746	-0.48299	-0.29374
101.762	-0.48302	-0.29359
101.779	-0.48305	-0.29342
101.795	-0.48308	-0.29332
101.812	-0.48313	-0.29318
101.829	-0.48317	-0.29308
101.845	-0.48321	-0.29297
101.862	-0.48325	-0.29285
101.879	-0.48328	-0.29279
101.895	-0.48331	-0.29275
101.913	-0.48334	-0.29272
101.929	-0.48337	-0.29269
101.946	-0.4834	-0.29266
101.963	-0.48343	-0.29262
101.979	-0.48346	-0.29259
101.997	-0.48349	-0.29256
102.013	-0.48353	-0.29253

102.03	-0.48356	-0.2925
102.046	-0.4836	-0.29246
102.063	-0.48363	-0.29242
102.08	-0.48366	-0.29235
102.096	-0.48369	-0.2923
102.114	-0.48372	-0.29221
102.13	-0.48375	-0.29214
102.146	-0.48379	-0.29208
102.163	-0.48382	-0.29204
102.179	-0.48385	-0.29198
102.197	-0.48388	-0.29192
102.213	-0.48392	-0.29189
102.229	-0.48395	-0.29184
102.246	-0.48397	-0.2918
102.264	-0.484	-0.29174
102.28	-0.48403	-0.2917
102.297	-0.48406	-0.29165
102.313	-0.48409	-0.2916
102.33	-0.48412	-0.29155
102.347	-0.48415	-0.2915
102.364	-0.48418	-0.29142
102.38	-0.48422	-0.29133
102.398	-0.48425	-0.29124
102.414	-0.48428	-0.29112
102.43	-0.48431	-0.29101
102.448	-0.48434	-0.29091
102.464	-0.48436	-0.29083
102.481	-0.4844	-0.29079
102.498	-0.48443	-0.29076
102.514	-0.48446	-0.29072

102.531	-0.4845	-0.29065
102.548	-0.48453	-0.29062
102.564	-0.48456	-0.29059
102.582	-0.48459	-0.29055
102.598	-0.48462	-0.29051
102.615	-0.48466	-0.29047
102.631	-0.48469	-0.29041
102.648	-0.48472	-0.29034
102.665	-0.48475	-0.29027
102.682	-0.48478	-0.29023
102.699	-0.48481	-0.29019
102.715	-0.48485	-0.29013
102.732	-0.48488	-0.29007
102.749	-0.48491	-0.29
102.765	-0.48495	-0.28989
102.782	-0.48501	-0.28977
102.798	-0.48507	-0.28966
102.815	-0.48511	-0.28957
102.831	-0.48514	-0.28949
102.848	-0.48518	-0.28939
102.865	-0.48521	-0.28933
102.881	-0.48525	-0.2893
102.898	-0.48528	-0.28926
102.916	-0.48531	-0.28921
102.932	-0.48534	-0.28918
102.949	-0.48538	-0.28913
102.965	-0.48541	-0.28908
102.982	-0.48545	-0.28902
103	-0.48549	-0.28895
103.016	-0.48555	-0.28888

103.032	-0.48562	-0.28882
103.05	-0.48565	-0.28876
103.066	-0.48569	-0.2887
103.083	-0.48573	-0.28862
103.1	-0.48576	-0.28855
103.116	-0.4858	-0.28848
103.133	-0.48583	-0.2884
103.148	-0.48587	-0.28831
103.166	-0.48591	-0.28823
103.183	-0.48595	-0.28814
103.199	-0.48598	-0.28806
103.215	-0.48604	-0.28799
103.232	-0.4861	-0.28794
103.25	-0.48615	-0.28789
103.266	-0.48619	-0.28785
103.282	-0.48623	-0.28781
103.3	-0.48629	-0.28778
103.315	-0.48634	-0.28775
103.333	-0.48638	-0.28771
103.349	-0.48642	-0.28768
103.367	-0.48646	-0.28762
103.382	-0.4865	-0.28756
103.4	-0.48653	-0.28748
103.416	-0.48656	-0.28739
103.433	-0.4866	-0.28728
103.449	-0.48663	-0.28717
103.465	-0.48667	-0.28704
103.483	-0.48671	-0.28694
103.5	-0.48675	-0.28688
103.516	-0.48679	-0.28685

103.534	-0.48684	-0.28681
103.549	-0.48691	-0.28677
103.566	-0.48696	-0.28673
103.583	-0.487	-0.28668
103.599	-0.48704	-0.28659
103.615	-0.48708	-0.28651
103.634	-0.48712	-0.28645
103.651	-0.48716	-0.28636
103.667	-0.48719	-0.28628
103.683	-0.48723	-0.28619
103.702	-0.48727	-0.28615
103.717	-0.48732	-0.28612
103.734	-0.48738	-0.28607
103.751	-0.48744	-0.28602
103.767	-0.48748	-0.28596
103.785	-0.48752	-0.2859
103.801	-0.48758	-0.28585
103.816	-0.48765	-0.28577
103.834	-0.4877	-0.28569
103.85	-0.48775	-0.28562
103.867	-0.4878	-0.28557
103.883	-0.48786	-0.28554
103.9	-0.48794	-0.28551
103.917	-0.48799	-0.28547
103.933	-0.48804	-0.28544
103.952	-0.48808	-0.2854
103.968	-0.48812	-0.28537
103.985	-0.48817	-0.28533
104.001	-0.48821	-0.28529
104.017	-0.48825	-0.28523

104.036	-0.48829	-0.28514
104.051	-0.48834	-0.28507
104.069	-0.48838	-0.28499
104.085	-0.48843	-0.28491
104.102	-0.48851	-0.28485
104.119	-0.48858	-0.2848
104.135	-0.48863	-0.28476
104.151	-0.48869	-0.28469
104.169	-0.48878	-0.28465
104.185	-0.48887	-0.28462
104.202	-0.48893	-0.28457
104.219	-0.489	-0.28454
104.235	-0.48904	-0.2845
104.252	-0.48909	-0.28443
104.269	-0.48913	-0.28436
104.285	-0.48917	-0.2843
104.302	-0.48922	-0.28427
104.317	-0.48926	-0.28423
104.335	-0.48931	-0.2842
104.352	-0.48936	-0.28416
104.368	-0.4894	-0.28412
104.386	-0.48945	-0.28407
104.402	-0.48949	-0.284
104.418	-0.48954	-0.28392
104.436	-0.48959	-0.28383
104.452	-0.48964	-0.28374
104.47	-0.48969	-0.28361
104.486	-0.48977	-0.28348
104.502	-0.48987	-0.2834
104.519	-0.48996	-0.28335

104.535	-0.49004	-0.28329
104.552	-0.49008	-0.28324
104.569	-0.49013	-0.2832
104.586	-0.49018	-0.28316
104.603	-0.49022	-0.28312
104.62	-0.49026	-0.28308
104.637	-0.49031	-0.28304
104.653	-0.49035	-0.28299
104.67	-0.4904	-0.28293
104.686	-0.49045	-0.28286
104.703	-0.4905	-0.28281
104.72	-0.49057	-0.28276
104.737	-0.49062	-0.28272
104.753	-0.49069	-0.28267
104.77	-0.49077	-0.28262
104.786	-0.49082	-0.28258
104.804	-0.4909	-0.28255
104.82	-0.49095	-0.28251
104.837	-0.49101	-0.28247
104.854	-0.49105	-0.28241
104.871	-0.4911	-0.28232
104.887	-0.49116	-0.28223
104.904	-0.49121	-0.28215
104.921	-0.49126	-0.28209
104.937	-0.49132	-0.28203
104.953	-0.49139	-0.28196
104.97	-0.49144	-0.28191
104.987	-0.49151	-0.28187
105.004	-0.49156	-0.28182
105.02	-0.49162	-0.28179

105.038	-0.49168	-0.28175
105.055	-0.49175	-0.2817
105.071	-0.49183	-0.28165
105.087	-0.4919	-0.2816
105.105	-0.49196	-0.28157
105.121	-0.49201	-0.28153
105.138	-0.49207	-0.28149
105.155	-0.49212	-0.28145
105.172	-0.49217	-0.28142
105.189	-0.49222	-0.28137
105.205	-0.49227	-0.2813
105.221	-0.49232	-0.28121
105.238	-0.49242	-0.28113
105.254	-0.49251	-0.28103
105.271	-0.49261	-0.28091
105.288	-0.49268	-0.28079
105.305	-0.49276	-0.28065
105.321	-0.49283	-0.28051
105.338	-0.49288	-0.28042
105.355	-0.49293	-0.28032
105.372	-0.49298	-0.28027
105.388	-0.49303	-0.28021
105.405	-0.49309	-0.28017
105.422	-0.49314	-0.28013
105.438	-0.49322	-0.2801
105.455	-0.49333	-0.28006
105.472	-0.49349	-0.28002
105.49	-0.49365	-0.27997
105.506	-0.49377	-0.27989
105.522	-0.49385	-0.2798

105.539	-0.4939	-0.27972
105.555	-0.49398	-0.27963
105.571	-0.49409	-0.27954
105.589	-0.49421	-0.27947
105.606	-0.49427	-0.27942
105.622	-0.49432	-0.27937
105.639	-0.49439	-0.27934
105.656	-0.4945	-0.2793
105.672	-0.49457	-0.27927
105.689	-0.49462	-0.27923
105.707	-0.49468	-0.27919
105.723	-0.49475	-0.27915
105.74	-0.49486	-0.2791
105.755	-0.49501	-0.27902
105.773	-0.49512	-0.27895
105.789	-0.49519	-0.27888
105.806	-0.49524	-0.27881
105.822	-0.49529	-0.27875
105.839	-0.49536	-0.27866
105.856	-0.49541	-0.27855
105.872	-0.49546	-0.27843
105.889	-0.4955	-0.27833
105.906	-0.49555	-0.27826
105.923	-0.4956	-0.27819
105.939	-0.49566	-0.27812
105.956	-0.49573	-0.27806
105.973	-0.49581	-0.27801
105.989	-0.49589	-0.27797
106.006	-0.49599	-0.27793
106.022	-0.49609	-0.27788

106.04	-0.4962	-0.27782
106.056	-0.49632	-0.27776
106.073	-0.49641	-0.27773
106.09	-0.49646	-0.2777
106.107	-0.49652	-0.27766
106.124	-0.4966	-0.27761
106.14	-0.49666	-0.27756
106.156	-0.49671	-0.27747
106.173	-0.49676	-0.27738
106.191	-0.49681	-0.27731
106.207	-0.49685	-0.27722
106.223	-0.4969	-0.27712
106.24	-0.49695	-0.27703
106.257	-0.497	-0.27693
106.273	-0.49706	-0.27685
106.29	-0.49713	-0.2768
106.307	-0.49723	-0.27676
106.323	-0.49737	-0.27671
106.339	-0.49749	-0.27663
106.357	-0.49761	-0.27658
106.373	-0.49775	-0.27654
106.391	-0.49792	-0.2765
106.407	-0.4981	-0.27644
106.424	-0.49823	-0.2764
106.441	-0.49831	-0.27635
106.457	-0.49837	-0.27631
106.474	-0.49843	-0.27627
106.49	-0.49851	-0.27624
106.506	-0.49861	-0.2762
106.525	-0.49872	-0.27617

106.541	-0.49882	-0.27613
106.556	-0.49889	-0.2761
106.574	-0.49899	-0.27603
106.591	-0.49909	-0.27595
106.607	-0.49919	-0.27587
106.624	-0.49925	-0.27579
106.64	-0.49932	-0.2757
106.657	-0.49941	-0.27561
106.674	-0.49955	-0.27554
106.691	-0.49971	-0.27544
106.707	-0.49982	-0.27536
106.724	-0.49993	-0.27528
106.741	-0.50004	-0.27524
106.757	-0.50012	-0.27521
106.774	-0.50017	-0.27517
106.791	-0.50021	-0.27514
106.807	-0.50026	-0.2751
106.825	-0.50034	-0.27507
106.841	-0.50042	-0.27503
106.858	-0.50051	-0.27499
106.874	-0.50066	-0.27496
106.891	-0.50077	-0.27492
106.908	-0.50088	-0.27489
106.924	-0.50102	-0.27485
106.941	-0.50116	-0.27481
106.957	-0.50126	-0.27478
106.974	-0.50136	-0.27474
106.992	-0.50143	-0.27468
107.008	-0.5015	-0.27463
107.024	-0.50161	-0.27455

107.041	-0.50176	-0.27448
107.058	-0.50195	-0.2744
107.074	-0.50211	-0.27431
107.092	-0.50226	-0.27423
107.108	-0.50238	-0.27416
107.124	-0.50251	-0.27411
107.141	-0.50262	-0.27407
107.158	-0.50273	-0.27403
107.176	-0.50285	-0.27399
107.192	-0.50297	-0.27395
107.209	-0.50312	-0.27391
107.225	-0.50327	-0.27388
107.242	-0.50345	-0.27384
107.26	-0.50367	-0.27381
107.275	-0.50387	-0.27377
107.292	-0.50404	-0.27372
107.309	-0.50419	-0.27365
107.326	-0.50434	-0.27359
107.343	-0.50441	-0.27353
107.359	-0.50453	-0.27346
107.376	-0.5046	-0.27339
107.392	-0.50471	-0.2733
107.408	-0.50483	-0.27324
107.426	-0.50495	-0.2732
107.441	-0.50507	-0.27316
107.459	-0.50519	-0.27311
107.475	-0.50534	-0.27308
107.492	-0.50549	-0.27303
107.509	-0.50561	-0.27298
107.526	-0.50571	-0.27293

107.543	-0.50581	-0.27286
107.559	-0.50592	-0.27282
107.575	-0.50604	-0.27278
107.591	-0.50615	-0.27274
107.609	-0.50626	-0.27271
107.625	-0.50638	-0.27267
107.641	-0.50654	-0.27264
107.66	-0.50674	-0.2726
107.676	-0.50694	-0.27256
107.692	-0.50714	-0.27253
107.709	-0.5073	-0.27246
107.726	-0.50742	-0.27235
107.744	-0.50755	-0.27224
107.76	-0.5077	-0.27213
107.776	-0.50781	-0.27202
107.793	-0.50796	-0.27195
107.81	-0.50808	-0.27186
107.826	-0.50821	-0.27179
107.843	-0.50837	-0.27172
107.859	-0.50855	-0.27166
107.876	-0.50877	-0.2716
107.892	-0.50899	-0.27155
107.91	-0.50915	-0.27146
107.926	-0.50925	-0.27139
107.944	-0.50937	-0.27133
107.959	-0.50948	-0.27129
107.977	-0.50958	-0.27126
107.994	-0.50966	-0.27122
108.01	-0.50972	-0.27119
108.027	-0.50982	-0.27115

108.043	-0.50996	-0.27112
108.06	-0.51011	-0.27107
108.077	-0.51028	-0.27104
108.092	-0.5105	-0.271
108.111	-0.5107	-0.27096
108.127	-0.51093	-0.27091
108.144	-0.5112	-0.27087
108.161	-0.51141	-0.27084
108.177	-0.51157	-0.2708
108.194	-0.51171	-0.27076
108.212	-0.51184	-0.27071
108.228	-0.51198	-0.27068
108.245	-0.51215	-0.27064
108.261	-0.51231	-0.27057
108.278	-0.51249	-0.2705
108.294	-0.51261	-0.27043
108.311	-0.51275	-0.27038
108.327	-0.51294	-0.27034
108.343	-0.5131	-0.27027
108.361	-0.51325	-0.2702
108.378	-0.51335	-0.27016
108.395	-0.51344	-0.27012
108.411	-0.51354	-0.27006
108.428	-0.51368	-0.27
108.444	-0.51379	-0.26995
108.461	-0.51391	-0.26989
108.478	-0.51398	-0.26983
108.494	-0.51405	-0.26978
108.512	-0.51415	-0.26973
108.527	-0.51428	-0.26969

108.544	-0.5144	-0.26964
108.562	-0.51452	-0.26958
108.578	-0.51465	-0.26953
108.594	-0.51483	-0.26947
108.612	-0.51502	-0.26942
108.629	-0.51526	-0.26938
108.645	-0.51554	-0.26934
108.662	-0.51578	-0.2693
108.678	-0.51597	-0.26925
108.695	-0.51617	-0.2692
108.712	-0.51639	-0.26917
108.728	-0.51662	-0.26913
108.745	-0.51677	-0.26909
108.762	-0.5169	-0.26905
108.778	-0.51702	-0.26901
108.795	-0.51715	-0.26897
108.812	-0.51734	-0.26893
108.827	-0.51747	-0.2689
108.845	-0.51761	-0.26886
108.861	-0.51776	-0.26881
108.878	-0.51791	-0.26876
108.895	-0.51808	-0.26872
108.911	-0.51826	-0.26868
108.928	-0.51845	-0.26863
108.946	-0.51862	-0.26858
108.962	-0.51874	-0.2685
108.979	-0.51887	-0.26843
108.995	-0.51901	-0.26835
109.012	-0.51916	-0.26829
109.029	-0.51928	-0.26822

109.045	-0.51944	-0.26815
109.062	-0.51963	-0.26806
109.078	-0.51978	-0.26801
109.096	-0.51995	-0.26797
109.112	-0.52014	-0.26792
109.128	-0.52032	-0.26786
109.145	-0.52049	-0.26781
109.162	-0.52069	-0.26775
109.179	-0.52083	-0.2677
109.196	-0.52097	-0.26766
109.213	-0.52108	-0.26762
109.229	-0.52117	-0.26758
109.246	-0.52128	-0.2675
109.262	-0.52138	-0.26744
109.279	-0.52151	-0.2674
109.296	-0.52172	-0.26736
109.311	-0.52199	-0.26733
109.329	-0.52229	-0.26729
109.346	-0.52267	-0.26725
109.362	-0.52299	-0.26721
109.38	-0.52322	-0.26717
109.396	-0.52337	-0.26713
109.412	-0.52346	-0.2671
109.429	-0.52354	-0.26706
109.445	-0.52358	-0.26702
109.462	-0.52364	-0.26698
109.48	-0.52372	-0.26694
109.496	-0.5238	-0.2669
109.513	-0.52395	-0.26686
109.529	-0.52415	-0.26682

109.547	-0.52435	-0.26678
109.563	-0.52451	-0.26674
109.58	-0.52465	-0.2667
109.597	-0.52479	-0.26666
109.614	-0.52492	-0.26662
109.63	-0.52504	-0.26658
109.647	-0.52518	-0.26653
109.663	-0.52531	-0.26648
109.68	-0.52547	-0.26644
109.697	-0.52568	-0.2664
109.712	-0.52587	-0.26636
109.73	-0.52599	-0.26631
109.746	-0.52611	-0.26627
109.764	-0.52623	-0.26623
109.78	-0.52636	-0.26619
109.797	-0.52649	-0.26615
109.813	-0.52666	-0.26609
109.83	-0.52681	-0.26603
109.847	-0.52702	-0.26597
109.864	-0.52727	-0.26591
109.88	-0.52753	-0.26583
109.898	-0.52772	-0.26574
109.914	-0.52789	-0.26564
109.931	-0.52802	-0.26557
109.946	-0.52815	-0.2655
109.963	-0.52828	-0.26545
109.98	-0.52841	-0.26541
109.996	-0.52856	-0.26537
110.014	-0.52871	-0.26533
110.031	-0.52891	-0.26529

110.047	-0.52909	-0.26525
110.064	-0.52927	-0.26521
110.08	-0.52946	-0.26515
110.097	-0.52965	-0.2651
110.114	-0.52982	-0.26506
110.131	-0.52996	-0.26499
110.146	-0.53011	-0.26494
110.164	-0.53021	-0.26489
110.181	-0.53034	-0.26485
110.198	-0.53048	-0.26481
110.214	-0.53066	-0.26477
110.231	-0.53083	-0.26473
110.247	-0.53099	-0.26469
110.264	-0.53114	-0.26465
110.281	-0.5313	-0.26461
110.297	-0.53151	-0.26457
110.313	-0.53181	-0.26453
110.331	-0.53209	-0.26447
110.347	-0.53231	-0.26441
110.365	-0.53252	-0.26432
110.381	-0.5327	-0.26428
110.397	-0.53292	-0.26423
110.414	-0.53313	-0.26418
110.431	-0.5334	-0.26414
110.447	-0.53361	-0.26409
110.465	-0.53382	-0.26405
110.481	-0.53408	-0.26401
110.498	-0.53435	-0.26397
110.515	-0.53461	-0.26393
110.531	-0.53481	-0.26389

110.548	-0.53496	-0.26381
110.565	-0.53509	-0.26373
110.581	-0.53523	-0.26368
110.597	-0.53537	-0.2636
110.615	-0.53555	-0.26354
110.631	-0.53575	-0.26346
110.647	-0.53595	-0.26341
110.665	-0.53615	-0.26335
110.681	-0.53639	-0.26331
110.698	-0.53667	-0.26327
110.714	-0.53699	-0.26323
110.731	-0.53723	-0.26319
110.749	-0.53742	-0.26315
110.766	-0.53759	-0.2631
110.781	-0.53773	-0.26306
110.798	-0.53788	-0.26302
110.814	-0.53804	-0.26298
110.831	-0.53819	-0.26294
110.848	-0.53832	-0.2629
110.864	-0.53853	-0.26286
110.882	-0.53881	-0.26281
110.898	-0.53908	-0.26278
110.915	-0.53937	-0.26274
110.931	-0.53967	-0.2627
110.948	-0.53993	-0.26266
110.966	-0.54017	-0.26262
110.982	-0.54038	-0.26258
110.998	-0.54054	-0.26254
111.015	-0.54066	-0.2625
111.032	-0.54075	-0.26242

111.049	-0.54086	-0.26237
111.065	-0.54096	-0.26233
111.082	-0.54105	-0.26229
111.098	-0.54118	-0.26225
111.114	-0.54135	-0.26221
111.133	-0.54157	-0.26217
111.149	-0.54185	-0.26213
111.165	-0.54226	-0.26209
111.182	-0.54261	-0.26205
111.199	-0.54295	-0.26201
111.215	-0.54325	-0.26197
111.233	-0.54365	-0.26193
111.25	-0.54394	-0.26188
111.265	-0.54412	-0.26182
111.283	-0.5443	-0.26177
111.298	-0.54446	-0.26173
111.315	-0.54461	-0.26169
111.332	-0.54477	-0.26164
111.349	-0.54501	-0.26158
111.366	-0.54522	-0.26152
111.383	-0.54545	-0.26148
111.4	-0.54567	-0.26144
111.417	-0.54594	-0.2614
111.433	-0.54617	-0.26136
111.45	-0.54635	-0.26132
111.465	-0.54651	-0.26127
111.482	-0.54666	-0.26123
111.499	-0.54683	-0.26119
111.515	-0.54701	-0.26115
111.533	-0.54725	-0.26111

111.549	-0.5475	-0.26107
111.566	-0.54782	-0.26103
111.584	-0.54817	-0.26099
111.6	-0.54854	-0.26095
111.616	-0.54882	-0.26091
111.634	-0.54904	-0.26088
111.65	-0.54926	-0.26084
111.667	-0.54944	-0.2608
111.684	-0.54962	-0.26075
111.7	-0.54984	-0.2607
111.717	-0.55005	-0.26065
111.733	-0.55024	-0.26059
111.75	-0.55043	-0.26051
111.767	-0.55064	-0.26044
111.783	-0.55092	-0.2604
111.8	-0.55122	-0.26035
111.817	-0.55148	-0.26031
111.833	-0.55179	-0.26027
111.85	-0.55199	-0.26022
111.868	-0.5522	-0.26018
111.883	-0.55247	-0.26014
111.901	-0.5527	-0.2601
111.917	-0.55292	-0.26006
111.933	-0.55316	-0.26001
111.95	-0.55343	-0.25996
111.967	-0.55368	-0.25992
111.983	-0.55398	-0.25988
112.001	-0.55431	-0.25984
112.017	-0.55459	-0.2598
112.034	-0.55483	-0.25976

112.05	-0.55507	-0.25971
112.067	-0.55535	-0.25967
112.084	-0.55564	-0.25963
112.1	-0.55588	-0.25957
112.116	-0.55613	-0.25953
112.133	-0.55639	-0.25948
112.15	-0.55671	-0.25944
112.168	-0.55699	-0.2594
112.184	-0.55731	-0.25936
112.2	-0.55754	-0.25932
112.218	-0.55773	-0.25928
112.233	-0.55786	-0.25924
112.25	-0.55803	-0.2592
112.268	-0.55823	-0.25916
112.284	-0.55846	-0.25912
112.301	-0.55879	-0.25908
112.317	-0.55911	-0.25903
112.334	-0.55943	-0.25899
112.35	-0.55973	-0.25894
112.367	-0.56003	-0.2589
112.385	-0.56031	-0.25885
112.401	-0.56054	-0.2588
112.419	-0.56071	-0.25875
112.434	-0.56088	-0.25871
112.45	-0.56106	-0.25867
112.468	-0.56124	-0.25863
112.484	-0.56145	-0.25859
112.501	-0.56164	-0.25854
112.518	-0.56179	-0.25851
112.534	-0.56193	-0.25847

112.551	-0.56205	-0.25843
112.569	-0.56217	-0.25839
112.586	-0.56236	-0.25835
112.602	-0.56257	-0.25831
112.618	-0.56282	-0.25827
112.635	-0.56309	-0.25823
112.652	-0.56337	-0.25819
112.668	-0.56363	-0.25815
112.685	-0.56387	-0.2581
112.701	-0.56419	-0.25806
112.718	-0.56448	-0.25803
112.734	-0.56476	-0.25798
112.752	-0.56498	-0.25794
112.769	-0.56519	-0.2579
112.785	-0.56542	-0.25786
112.802	-0.5657	-0.25782
112.819	-0.56598	-0.25778
112.836	-0.56621	-0.25774
112.852	-0.56639	-0.2577
112.869	-0.56659	-0.25766
112.885	-0.56685	-0.2576
112.901	-0.56721	-0.25754
112.918	-0.5675	-0.25748
112.935	-0.56776	-0.25743
112.952	-0.56795	-0.25738
112.968	-0.56818	-0.25734
112.985	-0.56843	-0.25731
113.002	-0.56876	-0.25727
113.019	-0.56902	-0.25723
113.036	-0.56922	-0.25719

113.053	-0.56939	-0.25715
113.069	-0.56961	-0.25711
113.086	-0.56989	-0.25707
113.103	-0.5702	-0.25702
113.119	-0.57046	-0.25696
113.137	-0.57075	-0.2569
113.152	-0.57097	-0.25684
113.169	-0.57125	-0.25679
113.187	-0.57159	-0.25676
113.203	-0.57193	-0.25672
113.219	-0.57226	-0.25668
113.235	-0.57248	-0.25664
113.253	-0.57265	-0.2566
113.27	-0.5728	-0.25656
113.286	-0.57295	-0.25652
113.303	-0.57307	-0.25647
113.319	-0.57324	-0.25641
113.336	-0.5734	-0.25633
113.354	-0.57357	-0.25628
113.369	-0.57382	-0.25624
113.386	-0.57426	-0.2562
113.402	-0.57466	-0.25616
113.419	-0.57512	-0.25612
113.436	-0.5755	-0.25608
113.452	-0.57584	-0.25604
113.469	-0.57615	-0.256
113.487	-0.57645	-0.25596
113.503	-0.57671	-0.25591
113.52	-0.57693	-0.25587
113.537	-0.57711	-0.25583

113.553	-0.57734	-0.25579
113.569	-0.57763	-0.25574
113.585	-0.57793	-0.25568
113.603	-0.57825	-0.2556
113.619	-0.57849	-0.25556
113.635	-0.5787	-0.25552
113.652	-0.57894	-0.25548
113.67	-0.57926	-0.25544
113.686	-0.5797	-0.2554
113.702	-0.57997	-0.25536
113.72	-0.5802	-0.25532
113.737	-0.58047	-0.25528
113.753	-0.58075	-0.25525
113.769	-0.58103	-0.25521
113.787	-0.58129	-0.25517
113.803	-0.5815	-0.25513
113.82	-0.58164	-0.2551
113.837	-0.58177	-0.25506
113.853	-0.58195	-0.25501
113.87	-0.58215	-0.25497
113.886	-0.5823	-0.25492
113.903	-0.58247	-0.25484
113.919	-0.58274	-0.25478
113.935	-0.58304	-0.25473
113.953	-0.58336	-0.25469
113.969	-0.58361	-0.25465
113.987	-0.58388	-0.2546
114.002	-0.58413	-0.25456
114.02	-0.58433	-0.25452
114.036	-0.58455	-0.25448

114.053	-0.5848	-0.25444
114.069	-0.58504	-0.25441
114.086	-0.58527	-0.25437
114.103	-0.58551	-0.25433
114.12	-0.58575	-0.25429
114.137	-0.586	-0.25426
114.153	-0.58621	-0.25422
114.17	-0.58645	-0.25417
114.186	-0.58672	-0.25413
114.203	-0.587	-0.25409
114.22	-0.58728	-0.25405
114.237	-0.58771	-0.25401
114.253	-0.58808	-0.25397
114.271	-0.58848	-0.25393
114.286	-0.58881	-0.2539
114.304	-0.58905	-0.25386
114.32	-0.58927	-0.25383
114.337	-0.58945	-0.25379
114.353	-0.58969	-0.25375
114.369	-0.58991	-0.25371
114.386	-0.59011	-0.25367
114.402	-0.59028	-0.25363
114.42	-0.59047	-0.25359
114.437	-0.59069	-0.25355
114.454	-0.59094	-0.25351
114.47	-0.59119	-0.25348
114.487	-0.59149	-0.25344
114.503	-0.59184	-0.25341
114.52	-0.59214	-0.25337
114.537	-0.59243	-0.25333

114.553	-0.59268	-0.25329
114.57	-0.59293	-0.25326
114.588	-0.59317	-0.25322
114.603	-0.59341	-0.25318
114.62	-0.5936	-0.25314
114.637	-0.59377	-0.2531
114.653	-0.59399	-0.25307
114.67	-0.5943	-0.25303
114.687	-0.59462	-0.253
114.703	-0.59496	-0.25296
114.72	-0.59524	-0.25292
114.737	-0.59547	-0.25289
114.754	-0.5957	-0.25285
114.77	-0.59588	-0.25282
114.787	-0.59601	-0.25278
114.804	-0.59613	-0.25274
114.821	-0.59623	-0.2527
114.837	-0.59637	-0.25266
114.854	-0.59661	-0.25263
114.871	-0.59688	-0.25259
114.888	-0.59719	-0.25255
114.904	-0.59749	-0.25251
114.92	-0.59784	-0.25246
114.937	-0.59813	-0.25242
114.954	-0.59836	-0.25238
114.969	-0.59855	-0.25234
114.987	-0.59875	-0.2523
115.004	-0.59892	-0.25227
115.021	-0.59906	-0.25223
115.038	-0.5992	-0.25219

115.054	-0.59937	-0.25215
115.071	-0.59951	-0.25212
115.088	-0.59961	-0.25208
115.104	-0.59976	-0.25205
115.121	-0.59989	-0.25201
115.139	-0.60002	-0.25198
115.154	-0.60025	-0.25194
115.172	-0.60058	-0.25191
115.188	-0.60102	-0.25187
115.205	-0.60144	-0.25184
115.221	-0.60183	-0.2518
115.238	-0.60218	-0.25177
115.253	-0.60248	-0.25173
115.271	-0.60274	-0.25169
115.288	-0.60296	-0.25166
115.305	-0.60316	-0.25162
115.32	-0.60333	-0.25159
115.338	-0.60352	-0.25155
115.355	-0.60374	-0.25152
115.371	-0.60388	-0.25148
115.387	-0.60402	-0.25145
115.405	-0.60418	-0.25142
115.421	-0.60433	-0.25138
115.438	-0.60449	-0.25135
115.455	-0.60465	-0.25132
115.472	-0.60485	-0.25128
115.488	-0.60501	-0.25125
115.504	-0.60517	-0.25122
115.522	-0.60531	-0.25118
115.539	-0.6055	-0.25115

115.555	-0.60569	-0.25112
115.572	-0.60583	-0.25108
115.589	-0.60598	-0.25105
115.605	-0.60617	-0.25101
115.622	-0.60637	-0.25098
115.638	-0.60655	-0.25094
115.655	-0.60681	-0.25091
115.672	-0.60708	-0.25087
115.688	-0.60735	-0.25083
115.705	-0.60752	-0.2508
115.722	-0.60769	-0.25076
115.739	-0.60781	-0.25073
115.755	-0.60795	-0.25069
115.773	-0.60809	-0.25066
115.788	-0.60827	-0.25062
115.805	-0.60846	-0.25059
115.822	-0.60862	-0.25056
115.839	-0.60884	-0.25052
115.856	-0.60902	-0.25049
115.872	-0.6092	-0.25045
115.889	-0.60942	-0.2504
115.906	-0.60959	-0.2503
115.923	-0.60971	-0.25022
115.939	-0.60984	-0.25015
115.956	-0.60998	-0.25009
115.972	-0.61013	-0.25
115.989	-0.61026	-0.24993
116.006	-0.61038	-0.24986
116.022	-0.6105	-0.24981
116.039	-0.61063	-0.24978

116.056	-0.61076	-0.24974
116.073	-0.6109	-0.2497
116.089	-0.61107	-0.24967
116.106	-0.61127	-0.24963
116.123	-0.61149	-0.2496
116.139	-0.61169	-0.24956
116.156	-0.61188	-0.24953
116.173	-0.61201	-0.24949
116.189	-0.61212	-0.24945
116.207	-0.61221	-0.24941
116.223	-0.61229	-0.24937
116.24	-0.61237	-0.24934
116.256	-0.61245	-0.2493
116.273	-0.61251	-0.24926
116.289	-0.61258	-0.24921
116.306	-0.61267	-0.24917
116.323	-0.61279	-0.24911
116.338	-0.61289	-0.24906
116.356	-0.61301	-0.24896
116.372	-0.61316	-0.24889
116.388	-0.61341	-0.24884
116.405	-0.61369	-0.2488
116.422	-0.61389	-0.24876
116.439	-0.6141	-0.24873
116.456	-0.6143	-0.24869
116.472	-0.61444	-0.24866
116.49	-0.61457	-0.24862
116.506	-0.61471	-0.24859
116.523	-0.61481	-0.24855
116.54	-0.61491	-0.24851

116.557	-0.61501	-0.24848
116.573	-0.61514	-0.24844
116.59	-0.61526	-0.2484
116.606	-0.61537	-0.24836
116.623	-0.61553	-0.24832
116.639	-0.61578	-0.24829
116.655	-0.61601	-0.24825
116.673	-0.6162	-0.24821
116.688	-0.61637	-0.24818
116.706	-0.61649	-0.24814
116.722	-0.61661	-0.2481
116.739	-0.61673	-0.24807
116.756	-0.61687	-0.24803
116.773	-0.61699	-0.24799
116.789	-0.6171	-0.24796
116.807	-0.61723	-0.24793
116.823	-0.61742	-0.24789
116.84	-0.61762	-0.24786
116.856	-0.61779	-0.24783
116.872	-0.618	-0.24779
116.89	-0.6182	-0.24776
116.907	-0.6184	-0.24772
116.923	-0.61861	-0.24769
116.94	-0.61883	-0.24765
116.957	-0.61899	-0.24761
116.974	-0.61914	-0.24758
116.991	-0.6193	-0.24755
117.007	-0.61944	-0.24752
117.024	-0.61958	-0.24749
117.04	-0.61972	-0.24745

117.057	-0.61983	-0.24743
117.073	-0.61997	-0.24739
117.09	-0.62012	-0.24736
117.107	-0.62031	-0.24733
117.124	-0.62053	-0.24729
117.141	-0.62077	-0.24726
117.157	-0.62103	-0.24722
117.174	-0.62135	-0.24719
117.192	-0.6217	-0.24716
117.207	-0.62207	-0.24713
117.225	-0.62241	-0.24709
117.241	-0.6227	-0.24706
117.258	-0.62296	-0.24703
117.275	-0.62319	-0.247
117.291	-0.62336	-0.24697
117.308	-0.62348	-0.24694
117.324	-0.62361	-0.24691
117.341	-0.62372	-0.24688
117.357	-0.62385	-0.24684
117.374	-0.62399	-0.24681
117.39	-0.62417	-0.24678
117.408	-0.62438	-0.24675
117.424	-0.62463	-0.24671
117.441	-0.62488	-0.24667
117.457	-0.62519	-0.24664
117.475	-0.62546	-0.2466
117.491	-0.62567	-0.24657
117.508	-0.62588	-0.24653
117.524	-0.62604	-0.2465
117.541	-0.62621	-0.24646

117.558	-0.6264	-0.24643
117.575	-0.62654	-0.2464
117.592	-0.62671	-0.24636
117.609	-0.62695	-0.24633
117.625	-0.62724	-0.2463
117.642	-0.62758	-0.24626
117.659	-0.62785	-0.24623
117.676	-0.62814	-0.2462
117.692	-0.62841	-0.24616
117.709	-0.62868	-0.24613
117.725	-0.62895	-0.24609
117.742	-0.62913	-0.24606
117.759	-0.62925	-0.24602
117.776	-0.62939	-0.24599
117.793	-0.62957	-0.24595
117.81	-0.62975	-0.24592
117.825	-0.62998	-0.24588
117.842	-0.63024	-0.24585
117.859	-0.63051	-0.2458
117.876	-0.6308	-0.24575
117.892	-0.63109	-0.24568
117.909	-0.63139	-0.24562
117.926	-0.63159	-0.24558
117.941	-0.63175	-0.24554
117.959	-0.63188	-0.24551
117.976	-0.63199	-0.24547
117.992	-0.63215	-0.24544
118.009	-0.63228	-0.24541
118.025	-0.63247	-0.24538
118.042	-0.63268	-0.24535

118.06	-0.63292	-0.24532
118.076	-0.63318	-0.24528
118.093	-0.63347	-0.24525
118.109	-0.63375	-0.24521
118.126	-0.63404	-0.24518
118.144	-0.63428	-0.24515
118.16	-0.63448	-0.24511
118.176	-0.63475	-0.24507
118.193	-0.63503	-0.24504
118.21	-0.63532	-0.245
118.226	-0.63564	-0.24497
118.243	-0.63594	-0.24493
118.26	-0.63622	-0.24489
118.276	-0.63645	-0.24486
118.293	-0.63672	-0.24482
118.309	-0.63699	-0.24479
118.327	-0.63728	-0.24475
118.343	-0.63757	-0.24472
118.359	-0.63792	-0.24469
118.376	-0.63831	-0.24465
118.393	-0.63873	-0.24462
118.41	-0.63916	-0.24459
118.426	-0.63963	-0.24455
118.443	-0.64008	-0.24451
118.46	-0.64043	-0.24447
118.476	-0.64075	-0.24442
118.492	-0.64108	-0.24436
118.51	-0.64139	-0.24432
118.526	-0.6417	-0.24426
118.543	-0.64203	-0.24422

118.559	-0.64241	-0.24419
118.576	-0.64277	-0.24415
118.592	-0.64312	-0.24412
118.609	-0.6436	-0.24409
118.626	-0.64405	-0.24406
118.643	-0.64438	-0.24403
118.66	-0.64469	-0.244
118.677	-0.64498	-0.24396
118.693	-0.64529	-0.24393
118.711	-0.6456	-0.24389
118.726	-0.64595	-0.24385
118.743	-0.64628	-0.2438
118.759	-0.64658	-0.24377
118.776	-0.64697	-0.24373
118.792	-0.64731	-0.2437
118.81	-0.64772	-0.24367
118.826	-0.64815	-0.24364
118.842	-0.64864	-0.2436
118.86	-0.64904	-0.24357
118.877	-0.64966	-0.24353
118.893	-0.65047	-0.2435
118.91	-0.65132	-0.24344
118.926	-0.65198	-0.24338
118.943	-0.65261	-0.24334
118.959	-0.65331	-0.2433
118.976	-0.65395	-0.24327
118.992	-0.65449	-0.24324
119.009	-0.65492	-0.24321
119.026	-0.65528	-0.24318
119.042	-0.65546	-0.24315

119.059	-0.65569	-0.24312
119.076	-0.65591	-0.24309
119.092	-0.65627	-0.24306
119.109	-0.6567	-0.24303
119.126	-0.65715	-0.24299
119.142	-0.65762	-0.24296
119.16	-0.65811	-0.24292
119.175	-0.65862	-0.24288
119.194	-0.65908	-0.24285
119.209	-0.65963	-0.24281
119.225	-0.66019	-0.24278
119.243	-0.66074	-0.24274
119.26	-0.66135	-0.24271
119.276	-0.6619	-0.24268
119.293	-0.66257	-0.24264
119.31	-0.66306	-0.24261
119.327	-0.66355	-0.24258
119.344	-0.66405	-0.24255
119.36	-0.66456	-0.24252
119.377	-0.66506	-0.24249
119.393	-0.6656	-0.24246
119.41	-0.66619	-0.24243
119.426	-0.66679	-0.2424
119.444	-0.66728	-0.24237
119.46	-0.66769	-0.24234
119.477	-0.66811	-0.24231
119.494	-0.66855	-0.24228
119.511	-0.66913	-0.24225
119.527	-0.66951	-0.24222
119.543	-0.66999	-0.24219

119.56	-0.67051	-0.24216
119.577	-0.67104	-0.24213
119.593	-0.67162	-0.2421
119.611	-0.67213	-0.24206
119.626	-0.6728	-0.24203
119.644	-0.67359	-0.242
119.66	-0.67428	-0.24197
119.676	-0.67488	-0.24193
119.693	-0.6756	-0.2419
119.71	-0.67614	-0.24187
119.727	-0.67675	-0.24184
119.744	-0.67733	-0.24181
119.76	-0.67788	-0.24178
119.777	-0.67824	-0.24174
119.794	-0.67854	-0.24171
119.811	-0.67895	-0.24168
119.827	-0.67946	-0.24165
119.845	-0.68009	-0.24162
119.861	-0.68068	-0.24159
119.878	-0.68137	-0.24156
119.894	-0.68206	-0.24153
119.912	-0.68293	-0.2415
119.928	-0.68372	-0.24147
119.944	-0.68451	-0.24144
119.961	-0.68498	-0.24141
119.977	-0.68533	-0.24138
119.994	-0.68574	-0.24135
120.01	-0.68614	-0.24131
120.027	-0.68662	-0.24128
120.043	-0.68734	-0.24125

120.061	-0.68777	-0.24122
120.077	-0.68848	-0.24119
120.095	-0.68911	-0.24116
120.111	-0.68983	-0.24113
120.127	-0.69057	-0.2411
120.145	-0.69119	-0.24107
120.161	-0.69188	-0.24104
120.178	-0.69258	-0.24101
120.194	-0.69324	-0.24098
120.211	-0.69397	-0.24095
120.227	-0.69459	-0.24091
120.244	-0.6949	-0.24088
120.26	-0.69566	-0.24085
120.277	-0.69624	-0.24082
120.295	-0.697	-0.24079
120.311	-0.69778	-0.24076
120.327	-0.69857	-0.24073
120.344	-0.69914	-0.2407
120.361	-0.69983	-0.24067
120.379	-0.70047	-0.24064
120.395	-0.70093	-0.24061
120.412	-0.7012	-0.24058
120.429	-0.70178	-0.24055
120.445	-0.70234	-0.24052
120.461	-0.70285	-0.24049
120.478	-0.70333	-0.24046
120.495	-0.70425	-0.24043
120.511	-0.7051	-0.2404
120.529	-0.70584	-0.24037
120.545	-0.70651	-0.24035

120.561	-0.70731	-0.24031
120.578	-0.70788	-0.24028
120.595	-0.70849	-0.24025
120.611	-0.70933	-0.24022
120.628	-0.71015	-0.24019
120.645	-0.71066	-0.24016
120.661	-0.71106	-0.24013
120.678	-0.71139	-0.24009
120.696	-0.71218	-0.24006
120.712	-0.71306	-0.24003
120.728	-0.71379	-0.24
120.745	-0.71458	-0.23996
120.762	-0.71529	-0.23993
120.78	-0.71602	-0.2399
120.795	-0.7167	-0.23988
120.811	-0.71732	-0.23985
120.828	-0.71794	-0.23982
120.845	-0.7185	-0.23979
120.862	-0.71891	-0.23976
120.878	-0.71937	-0.23973
120.895	-0.72002	-0.2397
120.911	-0.72076	-0.23967
120.928	-0.72165	-0.23964
120.946	-0.7222	-0.23961
120.962	-0.72304	-0.23958
120.978	-0.72372	-0.23956
120.995	-0.72453	-0.23953
121.013	-0.72523	-0.23951
121.029	-0.72601	-0.23948
121.046	-0.72692	-0.23945

121.062	-0.72755	-0.23942
121.078	-0.72825	-0.23939
121.095	-0.72913	-0.23936
121.113	-0.72995	-0.23933
121.129	-0.73056	-0.2393
121.146	-0.7312	-0.23927
121.162	-0.73195	-0.23924
121.179	-0.73284	-0.23922
121.195	-0.73351	-0.23919
121.212	-0.7341	-0.23916
121.229	-0.73486	-0.23913
121.246	-0.73574	-0.2391
121.263	-0.73653	-0.23908
121.279	-0.7371	-0.23905
121.296	-0.73774	-0.23902
121.313	-0.73823	-0.23899
121.329	-0.73882	-0.23896
121.346	-0.73969	-0.23893
121.363	-0.74053	-0.2389
121.38	-0.74142	-0.23887
121.396	-0.74195	-0.23884
121.413	-0.74268	-0.23881
121.429	-0.74366	-0.23878
121.446	-0.74449	-0.23876
121.463	-0.74548	-0.23873
121.48	-0.74586	-0.2387
121.495	-0.74667	-0.23868
121.513	-0.7474	-0.23865
121.529	-0.74806	-0.23863
121.546	-0.74865	-0.2386

121.563	-0.74895	-0.23857
121.58	-0.74962	-0.23854
121.597	-0.75051	-0.23852
121.614	-0.75137	-0.23849
121.63	-0.75188	-0.23846
121.646	-0.75238	-0.23843
121.663	-0.75292	-0.2384
121.68	-0.75362	-0.23837
121.696	-0.75455	-0.23835
121.713	-0.75535	-0.23832
121.73	-0.75604	-0.23829
121.746	-0.7567	-0.23826
121.763	-0.75778	-0.23823
121.779	-0.75884	-0.2382
121.797	-0.75985	-0.23818
121.813	-0.76083	-0.23815
121.83	-0.76119	-0.23812
121.847	-0.76179	-0.2381
121.863	-0.76241	-0.23807
121.88	-0.76328	-0.23805
121.897	-0.76398	-0.23802
121.913	-0.7651	-0.238
121.93	-0.76558	-0.23797
121.947	-0.76651	-0.23794
121.964	-0.76719	-0.23791
121.981	-0.76785	-0.23788
121.998	-0.76863	-0.23785
122.013	-0.76896	-0.23782
122.03	-0.76945	-0.23779
122.047	-0.7702	-0.23776

122.064	-0.77094	-0.23773
122.08	-0.77178	-0.23771
122.097	-0.77306	-0.23768
122.114	-0.77369	-0.23766
122.131	-0.77436	-0.23763
122.148	-0.77509	-0.23761
122.164	-0.77624	-0.23759
122.181	-0.77713	-0.23756
122.198	-0.77775	-0.23754
122.214	-0.77831	-0.23752
122.231	-0.77885	-0.23749
122.248	-0.77994	-0.23746
122.265	-0.78087	-0.23743
122.28	-0.78189	-0.2374
122.297	-0.78206	-0.23737
122.314	-0.78283	-0.23734
122.329	-0.78346	-0.23731
122.347	-0.78409	-0.23728
122.364	-0.785	-0.23725
122.381	-0.78551	-0.23722
122.398	-0.78615	-0.2372
122.415	-0.78663	-0.23717
122.43	-0.78772	-0.23715
122.447	-0.78883	-0.23712
122.463	-0.78982	-0.2371
122.481	-0.79043	-0.23707
122.498	-0.79121	-0.23705
122.515	-0.79192	-0.23702
122.532	-0.79265	-0.237
122.548	-0.79377	-0.23697

122.565	-0.79417	-0.23694
122.581	-0.79492	-0.23691
122.597	-0.79546	-0.23688
122.614	-0.79618	-0.23685
122.631	-0.79701	-0.23683
122.648	-0.79789	-0.2368
122.664	-0.79888	-0.23677
122.681	-0.79971	-0.23675
122.697	-0.80044	-0.23672
122.714	-0.80118	-0.2367
122.731	-0.80203	-0.23668
122.748	-0.80288	-0.23665
122.765	-0.80342	-0.23663
122.781	-0.80407	-0.23661
122.798	-0.80483	-0.23658
122.814	-0.80533	-0.23655
122.83	-0.80598	-0.23651
122.848	-0.80706	-0.23648
122.863	-0.80754	-0.23645
122.881	-0.80831	-0.23642
122.897	-0.80903	-0.2364
122.914	-0.80985	-0.23638
122.93	-0.81051	-0.23635
122.947	-0.81134	-0.23633
122.963	-0.81212	-0.23631
122.981	-0.81274	-0.23629
122.997	-0.8135	-0.23626
123.014	-0.8144	-0.23623
123.03	-0.81522	-0.23621
123.047	-0.81634	-0.23618

123.063	-0.81746	-0.23615
123.081	-0.81816	-0.23612
123.097	-0.81852	-0.23609
123.114	-0.8192	-0.23607
123.131	-0.82001	-0.23604
123.148	-0.82087	-0.23602
123.164	-0.82145	-0.23599
123.181	-0.8222	-0.23597
123.198	-0.82287	-0.23594
123.214	-0.82353	-0.23592
123.232	-0.82454	-0.23589
123.248	-0.82552	-0.23587
123.265	-0.8266	-0.23584
123.281	-0.827	-0.23582
123.299	-0.82756	-0.23579
123.314	-0.82816	-0.23577
123.332	-0.82881	-0.23575
123.348	-0.82947	-0.23573
123.364	-0.8303	-0.2357
123.381	-0.83115	-0.23568
123.397	-0.8318	-0.23565
123.414	-0.83271	-0.23563
123.431	-0.83376	-0.2356
123.448	-0.83471	-0.23557
123.465	-0.8355	-0.23554
123.481	-0.83647	-0.23552
123.497	-0.83701	-0.23549
123.513	-0.83737	-0.23547
123.531	-0.83762	-0.23545
123.548	-0.83838	-0.23543

123.565	-0.83944	-0.2354
123.581	-0.84006	-0.23538
123.598	-0.84069	-0.23535
123.615	-0.8416	-0.23533
123.631	-0.84273	-0.2353
123.648	-0.84372	-0.23528
123.665	-0.84432	-0.23526
123.681	-0.84512	-0.23524
123.698	-0.8459	-0.23522
123.715	-0.84674	-0.2352
123.732	-0.84752	-0.23518
123.747	-0.84837	-0.23516
123.765	-0.84918	-0.23514
123.782	-0.84976	-0.23512
123.798	-0.8507	-0.23509
123.815	-0.85162	-0.23507
123.832	-0.85244	-0.23504
123.847	-0.85277	-0.23501
123.865	-0.85337	-0.23499
123.882	-0.8542	-0.23496
123.898	-0.85486	-0.23494
123.915	-0.85567	-0.23492
123.931	-0.85674	-0.2349
123.948	-0.85787	-0.23488
123.965	-0.85834	-0.23486
123.982	-0.85919	-0.23484
123.999	-0.85997	-0.23482
124.015	-0.86072	-0.2348
124.032	-0.86152	-0.23478
124.048	-0.86221	-0.23476

124.065	-0.86304	-0.23474
124.081	-0.86408	-0.23471
124.099	-0.86468	-0.23469
124.115	-0.86509	-0.23467
124.131	-0.86602	-0.23465
124.148	-0.86685	-0.23463
124.165	-0.86744	-0.23461
124.182	-0.8681	-0.23459
124.199	-0.86865	-0.23456
124.214	-0.86932	-0.23454
124.231	-0.8701	-0.23452
124.248	-0.87072	-0.23449
124.265	-0.87165	-0.23447
124.283	-0.87242	-0.23445
124.298	-0.87288	-0.23442
124.315	-0.8733	-0.2344
124.331	-0.87433	-0.23438
124.349	-0.87527	-0.23436
124.366	-0.87599	-0.23434
124.382	-0.87734	-0.23433
124.399	-0.87841	-0.23431
124.415	-0.87932	-0.23429
124.431	-0.88004	-0.23427
124.449	-0.88088	-0.23425
124.465	-0.88206	-0.23423
124.482	-0.88244	-0.23421
124.499	-0.88299	-0.23418
124.515	-0.88362	-0.23417
124.532	-0.88441	-0.23414
124.548	-0.88526	-0.23413

124.566	-0.88599	-0.23411
124.582	-0.88644	-0.23409
124.599	-0.8873	-0.23407
124.616	-0.88778	-0.23405
124.632	-0.88852	-0.23403
124.649	-0.88967	-0.23401
124.666	-0.89061	-0.23399
124.683	-0.89136	-0.23396
124.7	-0.89192	-0.23394
124.716	-0.89281	-0.23392
124.732	-0.89398	-0.2339
124.749	-0.89495	-0.23388
124.765	-0.89574	-0.23386
124.782	-0.89657	-0.23385
124.799	-0.8971	-0.23383
124.815	-0.89764	-0.23381
124.833	-0.89863	-0.23379
124.849	-0.89958	-0.23377
124.866	-0.90025	-0.23376
124.883	-0.90082	-0.23374
124.9	-0.90175	-0.23372
124.916	-0.90279	-0.2337
124.933	-0.90339	-0.23368
124.95	-0.90443	-0.23367
124.966	-0.90535	-0.23364
124.983	-0.9059	-0.23362
124.998	-0.90633	-0.2336
125.017	-0.90708	-0.23358
125.032	-0.90802	-0.23356
125.05	-0.90863	-0.23354

125.066	-0.90944	-0.23352
125.082	-0.91026	-0.2335
125.099	-0.91117	-0.23348
125.115	-0.91209	-0.23346
125.133	-0.91329	-0.23345
125.15	-0.91414	-0.23343
125.167	-0.9149	-0.23342
125.183	-0.9154	-0.2334
125.201	-0.91605	-0.23338
125.216	-0.9169	-0.23336
125.234	-0.91739	-0.23334
125.25	-0.91822	-0.23332
125.267	-0.91894	-0.2333
125.283	-0.91938	-0.23328
125.299	-0.92045	-0.23327
125.317	-0.92111	-0.23325
125.333	-0.92205	-0.23323
125.349	-0.92307	-0.23321
125.366	-0.92383	-0.2332
125.383	-0.92449	-0.23318
125.4	-0.92514	-0.23316
125.416	-0.92605	-0.23314
125.434	-0.92667	-0.23313
125.449	-0.92748	-0.23311
125.466	-0.928	-0.23309
125.482	-0.92885	-0.23308
125.5	-0.92943	-0.23306
125.516	-0.93	-0.23305
125.533	-0.93093	-0.23303
125.55	-0.93192	-0.23301

125.568	-0.93307	-0.233
125.583	-0.93351	-0.23299
125.6	-0.93426	-0.23297
125.616	-0.93486	-0.23296
125.634	-0.93562	-0.23294
125.65	-0.93644	-0.23292
125.667	-0.93742	-0.2329
125.683	-0.9381	-0.23289
125.7	-0.9384	-0.23287
125.717	-0.93911	-0.23285
125.732	-0.93959	-0.23283
125.751	-0.94027	-0.23282
125.768	-0.94102	-0.2328
125.784	-0.94167	-0.23279
125.801	-0.94253	-0.23277
125.817	-0.943	-0.23276
125.834	-0.94388	-0.23274
125.851	-0.94463	-0.23272
125.867	-0.94532	-0.2327
125.884	-0.94596	-0.23268
125.9	-0.94666	-0.23266
125.917	-0.94716	-0.23264
125.934	-0.94791	-0.23262
125.95	-0.94866	-0.23261
125.966	-0.94924	-0.23259
125.983	-0.94966	-0.23258
126	-0.95035	-0.23256
126.017	-0.95085	-0.23255
126.034	-0.95152	-0.23254
126.051	-0.95232	-0.23253

126.066	-0.95289	-0.23252
126.083	-0.95355	-0.2325
126.101	-0.954	-0.23248
126.117	-0.95448	-0.23246
126.134	-0.95511	-0.23245
126.15	-0.95563	-0.23243
126.167	-0.95638	-0.23241
126.183	-0.95726	-0.23238
126.201	-0.95805	-0.23236
126.217	-0.95883	-0.23235
126.234	-0.95952	-0.23233
126.25	-0.96002	-0.23232
126.267	-0.96063	-0.2323
126.284	-0.96137	-0.23229
126.301	-0.96178	-0.23228
126.318	-0.96231	-0.23227
126.334	-0.96293	-0.23225
126.35	-0.9635	-0.23223
126.368	-0.96413	-0.23222
126.384	-0.96488	-0.2322
126.4	-0.96603	-0.23218
126.418	-0.9671	-0.23217
126.434	-0.96758	-0.23215
126.45	-0.96798	-0.23214
126.467	-0.96884	-0.23213
126.485	-0.9697	-0.23212
126.5	-0.97048	-0.2321
126.518	-0.97137	-0.23209
126.535	-0.97218	-0.23207
126.55	-0.97283	-0.23205

126.567	-0.97429	-0.23204
126.585	-0.97516	-0.23202
126.6	-0.97607	-0.232
126.619	-0.97659	-0.23199
126.635	-0.97736	-0.23198
126.652	-0.9785	-0.23197
126.667	-0.97886	-0.23196
126.684	-0.97933	-0.23195
126.701	-0.98012	-0.23194
126.718	-0.98084	-0.23192
126.734	-0.98125	-0.23191
126.751	-0.98213	-0.23189
126.768	-0.98303	-0.23188
126.785	-0.98345	-0.23186
126.801	-0.98384	-0.23184
126.818	-0.98398	-0.23183
126.834	-0.98458	-0.23181
126.85	-0.98489	-0.2318
126.868	-0.98572	-0.23179
126.884	-0.98659	-0.23178
126.901	-0.98762	-0.23177
126.918	-0.98858	-0.23176
126.934	-0.98957	-0.23174
126.951	-0.99073	-0.23173
126.967	-0.99165	-0.23171
126.984	-0.99211	-0.2317
127.001	-0.99249	-0.23169
127.018	-0.99308	-0.23168
127.034	-0.99321	-0.23167
127.05	-0.99337	-0.23166

127.068	-0.99375	-0.23165
127.085	-0.99451	-0.23164
127.1	-0.99595	-0.23162
127.117	-0.99693	-0.23161
127.134	-0.99777	-0.2316
127.151	-0.99831	-0.23159
127.167	-0.99894	-0.23158
127.184	-0.99967	-0.23156
127.201	-1.00039	-0.23155
127.218	-1.00099	-0.23154
127.234	-1.00174	-0.23152
127.251	-1.00211	-0.23151
127.267	-1.00255	-0.2315
127.285	-1.00332	-0.23148
127.301	-1.00404	-0.23147
127.317	-1.00448	-0.23146
127.334	-1.00449	-0.23145
127.351	-1.00477	-0.23144
127.368	-1.00537	-0.23143
127.384	-1.00604	-0.23142
127.401	-1.00688	-0.23141
127.418	-1.00781	-0.23139
127.434	-1.00866	-0.23138
127.451	-1.00933	-0.23137
127.468	-1.01005	-0.23136
127.484	-1.01057	-0.23135
127.501	-1.01114	-0.23134
127.518	-1.01128	-0.23133
127.534	-1.01154	-0.23132
127.551	-1.01203	-0.23131

127.567	-1.01257	-0.2313
127.585	-1.01321	-0.23129
127.602	-1.01409	-0.23128
127.618	-1.01458	-0.23127
127.635	-1.01547	-0.23126
127.651	-1.01625	-0.23126
127.669	-1.01699	-0.23125
127.684	-1.01767	-0.23124
127.702	-1.01802	-0.23123
127.718	-1.01863	-0.23122
127.735	-1.0192	-0.23121
127.751	-1.01988	-0.23121
127.768	-1.02059	-0.2312
127.784	-1.02127	-0.23119
127.801	-1.02192	-0.23117
127.818	-1.02261	-0.23116
127.834	-1.02344	-0.23115
127.851	-1.0243	-0.23113
127.868	-1.02509	-0.23112
127.884	-1.0256	-0.23111
127.9	-1.02632	-0.2311
127.917	-1.02686	-0.23109
127.934	-1.02749	-0.23108
127.951	-1.02807	-0.23107
127.967	-1.0285	-0.23106
127.984	-1.02899	-0.23105
128.002	-1.02958	-0.23104
128.018	-1.03028	-0.23104
128.034	-1.03079	-0.23103
128.051	-1.0312	-0.23102

128.068	-1.03167	-0.23101
128.085	-1.03226	-0.231
128.101	-1.03289	-0.231
128.119	-1.03366	-0.23099
128.134	-1.0345	-0.23098
128.152	-1.03512	-0.23097
128.168	-1.03582	-0.23096
128.185	-1.03677	-0.23095
128.201	-1.03741	-0.23094
128.217	-1.03809	-0.23093
128.234	-1.03869	-0.23093
128.252	-1.03912	-0.23092
128.267	-1.03971	-0.23091
128.285	-1.0403	-0.23091
128.302	-1.04095	-0.2309
128.317	-1.04147	-0.2309
128.334	-1.04227	-0.23089
128.351	-1.04325	-0.23088
128.367	-1.04411	-0.23087
128.385	-1.04501	-0.23086
128.401	-1.04545	-0.23085
128.418	-1.04597	-0.23084
128.434	-1.04652	-0.23083
128.452	-1.04713	-0.23082
128.468	-1.0478	-0.23081
128.484	-1.04859	-0.23081
128.501	-1.04921	-0.2308
128.519	-1.05018	-0.2308
128.535	-1.05126	-0.23079
128.552	-1.05205	-0.23078

128.569	-1.05291	-0.23077
128.584	-1.05349	-0.23077
128.601	-1.05396	-0.23076
128.617	-1.05445	-0.23075
128.634	-1.05505	-0.23074
128.651	-1.05545	-0.23074
128.668	-1.05601	-0.23073
128.685	-1.05637	-0.23073
128.701	-1.05686	-0.23072
128.718	-1.05764	-0.23071
128.734	-1.05818	-0.2307
128.751	-1.05854	-0.2307
128.768	-1.05898	-0.23069
128.784	-1.05969	-0.23069
128.801	-1.06054	-0.23068
128.818	-1.06142	-0.23068
128.836	-1.06238	-0.23067
128.852	-1.06322	-0.23067
128.869	-1.06429	-0.23066
128.885	-1.06481	-0.23066
128.902	-1.06553	-0.23065
128.918	-1.06598	-0.23065
128.934	-1.06633	-0.23064
128.952	-1.06693	-0.23064
128.968	-1.06771	-0.23063
128.985	-1.0686	-0.23063
129.002	-1.06936	-0.23063
129.018	-1.06988	-0.23062
129.035	-1.07056	-0.23062
129.051	-1.07125	-0.23061

129.069	-1.07236	-0.23061
129.085	-1.07306	-0.23061
129.101	-1.07361	-0.2306
129.118	-1.07423	-0.2306
129.135	-1.07519	-0.23059
129.151	-1.07614	-0.23059
129.168	-1.07665	-0.23058
129.185	-1.07759	-0.23057
129.202	-1.0783	-0.23057
129.219	-1.07899	-0.23057
129.236	-1.07955	-0.23056
129.251	-1.08022	-0.23056
129.268	-1.08047	-0.23055
129.285	-1.08109	-0.23055
129.302	-1.08198	-0.23055
129.318	-1.08254	-0.23054
129.335	-1.08321	-0.23054
129.352	-1.08365	-0.23053
129.368	-1.08449	-0.23053
129.385	-1.08544	-0.23052
129.401	-1.08644	-0.23052
129.418	-1.08733	-0.23051
129.435	-1.08833	-0.23051
129.452	-1.08868	-0.2305
129.468	-1.08929	-0.2305
129.485	-1.08987	-0.2305
129.502	-1.09048	-0.23049
129.518	-1.091	-0.23049
129.535	-1.09191	-0.23049
129.553	-1.093	-0.23048

129.569	-1.09373	-0.23048
129.585	-1.09491	-0.23048
129.601	-1.0957	-0.23047
129.618	-1.09669	-0.23047
129.635	-1.09756	-0.23047
129.651	-1.0985	-0.23046
129.669	-1.09931	-0.23046
129.685	-1.10039	-0.23046
129.701	-1.10149	-0.23046
129.719	-1.10231	-0.23046
129.735	-1.10367	-0.23046
129.752	-1.1047	-0.23046
129.768	-1.10588	-0.23046
129.786	-1.1071	-0.23045
129.802	-1.10781	-0.23045
129.819	-1.10852	-0.23044
129.835	-1.10902	-0.23044
129.852	-1.10963	-0.23043
129.869	-1.11045	-0.23043
129.886	-1.11118	-0.23042
129.902	-1.11144	-0.23042
129.919	-1.11203	-0.23042
129.935	-1.11292	-0.23042
129.952	-1.11365	-0.23042
129.969	-1.11477	-0.23042
129.986	-1.11552	-0.23042
130.002	-1.11661	-0.23041
130.019	-1.11735	-0.23041
130.035	-1.11827	-0.23041
130.051	-1.11892	-0.23041

130.07	-1.12002	-0.2304
130.087	-1.12069	-0.2304
130.103	-1.12155	-0.2304
130.119	-1.12218	-0.2304
130.135	-1.12306	-0.2304
130.152	-1.12321	-0.23039
130.169	-1.12391	-0.23039
130.185	-1.12483	-0.23039
130.202	-1.12533	-0.23039
130.219	-1.12618	-0.23038
130.235	-1.12677	-0.23038
130.252	-1.12744	-0.23038
130.269	-1.12844	-0.23038
130.284	-1.1289	-0.23038
130.301	-1.12964	-0.23038
130.319	-1.13068	-0.23037
130.335	-1.13178	-0.23037
130.351	-1.13265	-0.23037
130.369	-1.13337	-0.23037
130.385	-1.13367	-0.23036
130.402	-1.13465	-0.23036
130.417	-1.13567	-0.23036
130.435	-1.13653	-0.23035
130.452	-1.13715	-0.23034
130.468	-1.13753	-0.23034
130.485	-1.13806	-0.23034
130.502	-1.13841	-0.23034
130.518	-1.13925	-0.23033
130.534	-1.14038	-0.23033
130.552	-1.14149	-0.23033

130.568	-1.14189	-0.23033
130.585	-1.14304	-0.23033
130.601	-1.14415	-0.23033
130.618	-1.14499	-0.23033
130.634	-1.14572	-0.23033
130.651	-1.14707	-0.23033
130.668	-1.14825	-0.23033
130.685	-1.14917	-0.23033
130.702	-1.15022	-0.23033
130.718	-1.15145	-0.23033
130.735	-1.1522	-0.23034
130.751	-1.15284	-0.23033
130.768	-1.154	-0.23034
130.785	-1.15465	-0.23034
130.802	-1.15513	-0.23034
130.818	-1.15583	-0.23034
130.836	-1.15662	-0.23034
130.852	-1.15769	-0.23034
130.869	-1.15869	-0.23034
130.885	-1.15954	-0.23033
130.902	-1.16026	-0.23034
130.918	-1.16099	-0.23034
130.935	-1.16145	-0.23034
130.952	-1.16215	-0.23034
130.968	-1.16267	-0.23034
130.986	-1.16298	-0.23035
131.002	-1.16361	-0.23035
131.018	-1.16423	-0.23035
131.035	-1.16474	-0.23034
131.051	-1.1655	-0.23034

131.068	-1.16597	-0.23034
131.085	-1.16685	-0.23034
131.102	-1.1675	-0.23034
131.118	-1.16825	-0.23034
131.134	-1.16891	-0.23035
131.152	-1.16987	-0.23035
131.17	-1.17086	-0.23035
131.185	-1.17186	-0.23035
131.201	-1.17259	-0.23035
131.219	-1.17348	-0.23035
131.235	-1.17439	-0.23036
131.252	-1.17514	-0.23036
131.268	-1.17615	-0.23036
131.285	-1.1767	-0.23036
131.302	-1.17756	-0.23036
131.318	-1.17849	-0.23037
131.336	-1.17977	-0.23036
131.352	-1.18092	-0.23036
131.369	-1.18239	-0.23036
131.385	-1.18367	-0.23036
131.401	-1.18518	-0.23037
131.418	-1.187	-0.23037
131.435	-1.1884	-0.23037
131.452	-1.19002	-0.23037
131.468	-1.19153	-0.23038
131.485	-1.19319	-0.23038
131.502	-1.19458	-0.23038
131.518	-1.1965	-0.23038
131.535	-1.19761	-0.23039
131.552	-1.19889	-0.23039

131.568	-1.20011	-0.23039
131.585	-1.20171	-0.2304
131.602	-1.20358	-0.2304
131.618	-1.20475	-0.23041
131.635	-1.20645	-0.23042
131.653	-1.20802	-0.23042
131.669	-1.21007	-0.23042
131.686	-1.21137	-0.23042
131.702	-1.21332	-0.23043
131.718	-1.21513	-0.23043
131.736	-1.21674	-0.23043
131.752	-1.21865	-0.23043
131.768	-1.22046	-0.23043
131.785	-1.22261	-0.23044
131.803	-1.22445	-0.23044
131.818	-1.2264	-0.23045
131.836	-1.22836	-0.23046
131.851	-1.23021	-0.23046
131.869	-1.2319	-0.23046
131.885	-1.23293	-0.23046
131.902	-1.23365	-0.23046
131.918	-1.23444	-0.23046
131.936	-1.23505	-0.23046
131.951	-1.2355	-0.23046
131.968	-1.23591	-0.23046
131.985	-1.23607	-0.23046
132.001	-1.23608	-0.23046
132.018	-1.23623	-0.23046
132.034	-1.23626	-0.23046
132.051	-1.23686	-0.23046

132.068	-1.23716	-0.23046
132.084	-1.23741	-0.23046
132.101	-1.23749	-0.23046
132.118	-1.23804	-0.23046
132.134	-1.23841	-0.23046
132.151	-1.23904	-0.23046
132.168	-1.23933	-0.23047
132.184	-1.23941	-0.23047
132.2	-1.23941	-0.23047
132.217	-1.23965	-0.23047
132.234	-1.23992	-0.23047
132.25	-1.24014	-0.23047
132.268	-1.24062	-0.23047
132.285	-1.24113	-0.23048
132.302	-1.24169	-0.23048
132.318	-1.24254	-0.23048
132.334	-1.243	-0.23048
132.351	-1.24364	-0.23049
132.368	-1.24451	-0.23049
132.385	-1.24517	-0.2305
132.401	-1.24584	-0.2305
132.417	-1.24648	-0.23051
132.435	-1.2468	-0.23051
132.451	-1.24705	-0.23052
132.467	-1.24713	-0.23052
132.485	-1.24715	-0.23052
132.5	-1.24694	-0.23052
132.518	-1.24667	-0.23052
132.534	-1.24656	-0.23052
132.551	-1.24657	-0.23052

132.567	-1.24663	-0.23052
132.585	-1.24665	-0.23053
132.602	-1.24668	-0.23053
132.618	-1.24675	-0.23054
132.634	-1.24678	-0.23054
132.651	-1.24686	-0.23055
132.669	-1.24696	-0.23055
132.684	-1.247	-0.23055
132.701	-1.2471	-0.23055
132.718	-1.24726	-0.23056
132.733	-1.24752	-0.23056
132.751	-1.24783	-0.23056
132.768	-1.24812	-0.23057
132.785	-1.24836	-0.23057
132.801	-1.24852	-0.23057
132.818	-1.24862	-0.23058
132.835	-1.24867	-0.23058
132.852	-1.24866	-0.23058
132.868	-1.24863	-0.23058
132.885	-1.2486	-0.23058
132.901	-1.24803	-0.23058
132.917	-1.24759	-0.23059
132.934	-1.24734	-0.23059
132.951	-1.24718	-0.2306
132.968	-1.24703	-0.2306
132.985	-1.24688	-0.23061
133.001	-1.24673	-0.23061
133.018	-1.24665	-0.23061
133.035	-1.24664	-0.23061
133.051	-1.24676	-0.23062

133.069	-1.24678	-0.23062
133.084	-1.2468	-0.23062
133.101	-1.24684	-0.23062
133.118	-1.24695	-0.23062
133.135	-1.24709	-0.23063
133.152	-1.24728	-0.23063
133.168	-1.24745	-0.23063
133.185	-1.24766	-0.23064
133.202	-1.24796	-0.23064
133.218	-1.24842	-0.23065
133.234	-1.24902	-0.23065
133.252	-1.24955	-0.23066
133.268	-1.24998	-0.23066
133.285	-1.25042	-0.23067
133.302	-1.25089	-0.23067
133.318	-1.25134	-0.23067
133.335	-1.25168	-0.23067
133.351	-1.25191	-0.23068
133.368	-1.25203	-0.23068
133.385	-1.25208	-0.23068
133.402	-1.25208	-0.23068
133.418	-1.25194	-0.23068
133.435	-1.2518	-0.23068
133.451	-1.25153	-0.23069
133.468	-1.25106	-0.23069
133.485	-1.25051	-0.2307
133.501	-1.24971	-0.2307
133.518	-1.24881	-0.23071
133.535	-1.24789	-0.23072
133.551	-1.24675	-0.23072

133.569	-1.24543	-0.23073
133.586	-1.24354	-0.23073
133.602	-1.2413	-0.23073
133.619	-1.23888	-0.23074
133.635	-1.23666	-0.23074
133.651	-1.2342	-0.23074
133.668	-1.23238	-0.23074
133.685	-1.23017	-0.23075
133.702	-1.22839	-0.23075
133.719	-1.22707	-0.23076
133.735	-1.22544	-0.23077
133.752	-1.22376	-0.23077
133.768	-1.22177	-0.23077
133.785	-1.21998	-0.23077
133.801	-1.21825	-0.23077
133.817	-1.21644	-0.23077
133.835	-1.21463	-0.23077
133.852	-1.21289	-0.23077
133.868	-1.21095	-0.23077
133.885	-1.2094	-0.23078
133.902	-1.20763	-0.23078
133.919	-1.20588	-0.23079
133.935	-1.20391	-0.23079
133.952	-1.20181	-0.2308
133.969	-1.20003	-0.2308
133.986	-1.19815	-0.23081
134.003	-1.19604	-0.23081
134.019	-1.19408	-0.23081
134.036	-1.19193	-0.23082
134.052	-1.18935	-0.23082

134.068	-1.18727	-0.23082
134.086	-1.185	-0.23082
134.102	-1.18271	-0.23082
134.119	-1.18037	-0.23083
134.136	-1.17822	-0.23083
134.152	-1.17618	-0.23083
134.168	-1.17362	-0.23083
134.186	-1.17112	-0.23083
134.202	-1.16859	-0.23083
134.219	-1.16617	-0.23083
134.235	-1.16371	-0.23083
134.252	-1.16116	-0.23083
134.268	-1.15887	-0.23083
134.285	-1.15646	-0.23084
134.302	-1.15382	-0.23084
134.318	-1.15082	-0.23084
134.336	-1.14835	-0.23084
134.352	-1.1457	-0.23085
134.369	-1.14294	-0.23085
134.386	-1.14005	-0.23085
134.402	-1.13707	-0.23085
134.419	-1.13423	-0.23085
134.435	-1.13082	-0.23085
134.452	-1.12777	-0.23085
134.468	-1.12449	-0.23085
134.485	-1.12094	-0.23085
134.502	-1.11698	-0.23085
134.519	-1.11298	-0.23084
134.535	-1.10918	-0.23084
134.551	-1.10484	-0.23084

134.569	-1.10118	-0.23084
134.584	-1.09706	-0.23084
134.602	-1.09324	-0.23084
134.618	-1.08953	-0.23084
134.635	-1.08566	-0.23084
134.652	-1.08201	-0.23084
134.669	-1.0783	-0.23084
134.685	-1.07441	-0.23084
134.702	-1.07083	-0.23084
134.719	-1.06706	-0.23084
134.735	-1.06292	-0.23084
134.751	-1.05881	-0.23084
134.769	-1.05475	-0.23084
134.786	-1.05061	-0.23085
134.802	-1.04662	-0.23085
134.819	-1.04254	-0.23086
134.836	-1.03844	-0.23086
134.852	-1.03432	-0.23086
134.869	-1.03064	-0.23086
134.885	-1.02676	-0.23085
134.903	-1.02272	-0.23085
134.919	-1.01847	-0.23085
134.935	-1.01366	-0.23085
134.952	-1.00905	-0.23085
134.969	-1.00496	-0.23085
134.986	-1.00082	-0.23085
135.002	-0.99643	-0.23085
135.019	-0.99225	-0.23085
135.035	-0.98771	-0.23085
135.052	-0.98323	-0.23085

135.069	-0.97905	-0.23085
135.086	-0.97458	-0.23085
135.103	-0.97001	-0.23085
135.119	-0.96515	-0.23085
135.136	-0.96039	-0.23086
135.153	-0.95551	-0.23086
135.169	-0.95077	-0.23086
135.185	-0.94567	-0.23086
135.201	-0.94083	-0.23086
135.218	-0.93596	-0.23085
135.235	-0.9315	-0.23085
135.251	-0.92671	-0.23085
135.268	-0.92217	-0.23084
135.285	-0.91723	-0.23084
135.303	-0.91279	-0.23084
135.318	-0.90811	-0.23084
135.336	-0.90329	-0.23084
135.352	-0.89872	-0.23084
135.368	-0.89389	-0.23084
135.385	-0.88896	-0.23084
135.402	-0.88434	-0.23084
135.418	-0.88003	-0.23084
135.436	-0.87495	-0.23084
135.451	-0.87054	-0.23084
135.469	-0.86576	-0.23084
135.485	-0.86099	-0.23084
135.502	-0.8558	-0.23085
135.519	-0.85084	-0.23085
135.536	-0.84607	-0.23085
135.553	-0.84107	-0.23085

135.569	-0.83604	-0.23084
135.585	-0.83105	-0.23084
135.602	-0.82613	-0.23084
135.619	-0.82086	-0.23083
135.636	-0.81598	-0.23083
135.652	-0.81074	-0.23083
135.669	-0.8055	-0.23083
135.686	-0.79995	-0.23083
135.702	-0.79469	-0.23083
135.719	-0.78943	-0.23083
135.736	-0.78451	-0.23083
135.752	-0.77957	-0.23083
135.769	-0.77464	-0.23083
135.785	-0.76978	-0.23083
135.802	-0.76492	-0.23083
135.818	-0.75983	-0.23083
135.835	-0.75518	-0.23082
135.853	-0.74992	-0.23082
135.87	-0.74502	-0.23082
135.885	-0.74018	-0.23082
135.902	-0.73538	-0.23082
135.918	-0.73061	-0.23082
135.935	-0.72576	-0.23082
135.952	-0.72115	-0.23081
135.968	-0.71704	-0.23081
135.986	-0.7128	-0.23081
136.001	-0.70848	-0.2308
136.018	-0.70472	-0.2308
136.035	-0.70072	-0.2308
136.052	-0.69666	-0.2308

136.069	-0.69274	-0.2308
136.085	-0.68926	-0.2308
136.102	-0.68529	-0.2308
136.12	-0.68132	-0.2308
136.136	-0.67731	-0.2308
136.153	-0.67376	-0.2308
136.169	-0.66984	-0.2308
136.186	-0.66633	-0.2308
136.203	-0.66271	-0.2308
136.219	-0.65929	-0.2308
136.236	-0.65573	-0.23079
136.252	-0.6527	-0.23079
136.268	-0.64981	-0.23078
136.285	-0.64697	-0.23078
136.302	-0.64427	-0.23077
136.32	-0.64127	-0.23077
136.336	-0.63842	-0.23076
136.351	-0.63582	-0.23076
136.368	-0.63316	-0.23076
136.386	-0.63001	-0.23076
136.403	-0.62734	-0.23076
136.419	-0.62451	-0.23076
136.435	-0.62226	-0.23076
136.453	-0.62001	-0.23076
136.469	-0.61768	-0.23076
136.485	-0.6155	-0.23075
136.503	-0.61316	-0.23074
136.519	-0.61074	-0.23073
136.535	-0.60845	-0.23073
136.552	-0.60648	-0.23072

136.569	-0.6043	-0.23072
136.585	-0.60238	-0.23072
136.602	-0.60036	-0.23072
136.619	-0.59854	-0.23071
136.636	-0.59653	-0.23071
136.652	-0.59488	-0.23071
136.669	-0.59318	-0.2307
136.686	-0.59149	-0.2307
136.703	-0.58987	-0.23069
136.719	-0.5882	-0.23069
136.736	-0.5863	-0.23069
136.752	-0.5845	-0.23068
136.769	-0.58295	-0.23068
136.785	-0.58145	-0.23067
136.802	-0.58017	-0.23067
136.82	-0.57875	-0.23067
136.836	-0.57764	-0.23067
136.852	-0.57658	-0.23067
136.869	-0.57539	-0.23067
136.885	-0.57413	-0.23066
136.903	-0.57271	-0.23066
136.919	-0.57163	-0.23066
136.935	-0.57038	-0.23065
136.951	-0.56886	-0.23065
136.969	-0.56753	-0.23064
136.985	-0.5664	-0.23063
137.002	-0.56559	-0.23063
137.019	-0.56503	-0.23062
137.037	-0.56434	-0.23061
137.052	-0.5637	-0.23061

137.069	-0.56283	-0.2306
137.086	-0.56169	-0.23059
137.103	-0.56115	-0.23059
137.119	-0.55997	-0.23059
137.136	-0.5588	-0.23058
137.152	-0.55769	-0.23058
137.169	-0.55648	-0.23058
137.185	-0.55581	-0.23057
137.202	-0.55465	-0.23057
137.218	-0.55409	-0.23057
137.235	-0.55324	-0.23057
137.252	-0.55272	-0.23057
137.268	-0.55192	-0.23057
137.286	-0.55143	-0.23057
137.302	-0.55097	-0.23057
137.319	-0.55023	-0.23056
137.335	-0.54992	-0.23056
137.352	-0.5493	-0.23055
137.369	-0.54844	-0.23054
137.386	-0.54771	-0.23054
137.402	-0.54702	-0.23053
137.419	-0.54641	-0.23053
137.435	-0.54603	-0.23052
137.452	-0.54535	-0.23052
137.469	-0.54492	-0.23051
137.486	-0.54478	-0.23051
137.502	-0.54419	-0.23051
137.518	-0.54399	-0.23051
137.536	-0.54346	-0.23051
137.552	-0.54248	-0.23051

137.569	-0.54212	-0.23051
137.586	-0.54173	-0.2305
137.602	-0.54122	-0.2305
137.62	-0.54078	-0.2305
137.636	-0.54039	-0.23049
137.653	-0.53991	-0.23049
137.669	-0.5398	-0.23048
137.686	-0.53955	-0.23047
137.703	-0.53931	-0.23046
137.719	-0.53901	-0.23046
137.737	-0.53858	-0.23045
137.752	-0.5383	-0.23045
137.77	-0.53796	-0.23045
137.786	-0.5377	-0.23045
137.804	-0.53744	-0.23045
137.82	-0.53717	-0.23045
137.837	-0.53653	-0.23045
137.853	-0.53629	-0.23045
137.87	-0.53595	-0.23045
137.887	-0.53569	-0.23045
137.903	-0.53554	-0.23045
137.92	-0.53546	-0.23044
137.937	-0.53543	-0.23044
137.954	-0.53527	-0.23044
137.97	-0.53505	-0.23044
137.987	-0.53484	-0.23043
138.003	-0.53455	-0.23043
138.021	-0.53415	-0.23042
138.037	-0.53384	-0.23042
138.054	-0.53348	-0.23042

138.071	-0.53325	-0.23042
138.087	-0.533	-0.23041
138.104	-0.53283	-0.23041
138.121	-0.5327	-0.23041
138.137	-0.53251	-0.23041
138.154	-0.53231	-0.23041
138.17	-0.53212	-0.23041
138.187	-0.53195	-0.2304
138.204	-0.53183	-0.2304
138.221	-0.53165	-0.23039
138.237	-0.53146	-0.23038
138.253	-0.53131	-0.23037
138.27	-0.53118	-0.23037
138.286	-0.53103	-0.23036
138.303	-0.53091	-0.23035
138.321	-0.53075	-0.23034
138.337	-0.53061	-0.23034
138.354	-0.53052	-0.23034
138.371	-0.53045	-0.23034
138.388	-0.53032	-0.23034
138.405	-0.53021	-0.23034
138.421	-0.53009	-0.23034
138.438	-0.52997	-0.23033
138.454	-0.52984	-0.23032
138.472	-0.52971	-0.23032
138.488	-0.52951	-0.23031
138.504	-0.52935	-0.2303
138.521	-0.52914	-0.2303
138.537	-0.52899	-0.23029
138.554	-0.52884	-0.23028

138.571	-0.52866	-0.23027
138.588	-0.52857	-0.23026
138.604	-0.52851	-0.23026
138.621	-0.52841	-0.23025
138.638	-0.52831	-0.23025
138.655	-0.52823	-0.23024
138.671	-0.52814	-0.23023
138.688	-0.52808	-0.23023
138.705	-0.52803	-0.23022
138.721	-0.52796	-0.23022
138.737	-0.52787	-0.23021
138.754	-0.52777	-0.23021
138.771	-0.52769	-0.2302
138.787	-0.52763	-0.23019
138.804	-0.52758	-0.23018
138.821	-0.52751	-0.23018
138.837	-0.52745	-0.23017
138.854	-0.5274	-0.23016
138.871	-0.52735	-0.23015
138.887	-0.52731	-0.23014
138.904	-0.52726	-0.23013
138.921	-0.52718	-0.23013
138.938	-0.52709	-0.23012
138.954	-0.52699	-0.23011
138.97	-0.5269	-0.23011
138.987	-0.52683	-0.2301
139.005	-0.52675	-0.23009
139.021	-0.5267	-0.23008
139.038	-0.52668	-0.23007
139.054	-0.52666	-0.23006

139.071	-0.52663	-0.23005
139.088	-0.52661	-0.23004
139.104	-0.52659	-0.23004
139.121	-0.52658	-0.23003
139.137	-0.52655	-0.23002
139.154	-0.52653	-0.23002
139.171	-0.52651	-0.23001
139.187	-0.52649	-0.23001
139.204	-0.52648	-0.23
139.221	-0.52646	-0.22999
139.237	-0.52645	-0.22998
139.253	-0.52643	-0.22998
139.269	-0.52641	-0.22997
139.287	-0.52639	-0.22996
139.305	-0.52637	-0.22995
139.321	-0.52634	-0.22994
139.338	-0.52632	-0.22993
139.355	-0.52631	-0.22992
139.371	-0.52621	-0.22991
139.387	-0.52613	-0.2299
139.404	-0.52612	-0.22989
139.42	-0.52611	-0.22988
139.437	-0.52609	-0.22987
139.454	-0.52608	-0.22986
139.47	-0.52607	-0.22986
139.487	-0.52605	-0.22985
139.503	-0.52604	-0.22984
139.52	-0.52603	-0.22984
139.537	-0.52599	-0.22983
139.553	-0.52596	-0.22983

139.57	-0.52595	-0.22982
139.587	-0.52592	-0.22982
139.604	-0.52591	-0.22981
139.62	-0.5259	-0.2298
139.637	-0.52589	-0.22979
139.653	-0.52588	-0.22979
139.669	-0.52587	-0.22978
139.686	-0.52585	-0.22977
139.703	-0.52583	-0.22977
139.719	-0.52582	-0.22976
139.737	-0.52581	-0.22976
139.753	-0.52578	-0.22976
139.77	-0.52576	-0.22975
139.787	-0.52575	-0.22975
139.804	-0.52573	-0.22974
139.82	-0.52572	-0.22973
139.837	-0.52571	-0.22972
139.853	-0.5257	-0.22971
139.87	-0.52569	-0.2297
139.886	-0.52568	-0.2297
139.904	-0.52566	-0.22969
139.92	-0.52565	-0.22968
139.936	-0.52564	-0.22967
139.953	-0.52564	-0.22967
139.971	-0.52563	-0.22966
139.987	-0.52562	-0.22965
140.004	-0.52562	-0.22964
140.021	-0.52561	-0.22963
140.036	-0.5256	-0.22963
140.054	-0.5256	-0.22962

140.069	-0.52558	-0.22961
140.086	-0.52557	-0.22961
140.104	-0.52556	-0.22961
140.121	-0.52555	-0.22961
140.137	-0.52554	-0.22961
140.154	-0.52553	-0.22961
140.171	-0.52552	-0.22961
140.188	-0.52551	-0.2296
140.204	-0.5255	-0.2296
140.221	-0.52549	-0.2296
140.238	-0.52548	-0.22959
140.253	-0.52548	-0.22958
140.27	-0.52547	-0.22957
140.288	-0.52546	-0.22957
140.304	-0.52546	-0.22956
140.32	-0.52545	-0.22955
140.337	-0.52545	-0.22954
140.354	-0.52544	-0.22953
140.371	-0.52543	-0.22952
140.388	-0.52543	-0.22952
140.405	-0.52542	-0.22951
140.42	-0.52541	-0.22951
140.438	-0.5254	-0.2295
140.455	-0.52539	-0.2295
140.471	-0.52538	-0.2295
140.488	-0.52537	-0.2295
140.504	-0.52536	-0.2295
140.521	-0.52535	-0.2295
140.537	-0.52534	-0.22949
140.554	-0.52533	-0.22949

140.572	-0.52533	-0.22949
140.588	-0.52532	-0.22948
140.604	-0.52531	-0.22947
140.621	-0.52531	-0.22947
140.638	-0.5253	-0.22946
140.655	-0.52529	-0.22945
140.67	-0.52528	-0.22944
140.688	-0.52526	-0.22943
140.703	-0.52525	-0.22942
140.721	-0.52524	-0.22941
140.737	-0.52523	-0.2294
140.754	-0.52521	-0.2294
140.771	-0.52521	-0.22939
140.788	-0.52521	-0.22938
140.805	-0.5252	-0.22938
140.821	-0.5252	-0.22938
140.838	-0.52519	-0.22937
140.855	-0.52519	-0.22936
140.871	-0.52518	-0.22936
140.887	-0.52517	-0.22935
140.904	-0.52516	-0.22935
140.921	-0.52516	-0.22934
140.938	-0.52515	-0.22933
140.955	-0.52515	-0.22933
140.971	-0.52515	-0.22932
140.988	-0.52515	-0.22931
141.005	-0.52515	-0.2293
141.021	-0.52515	-0.22929
141.038	-0.52515	-0.22928
141.055	-0.52514	-0.22927

141.071	-0.52514	-0.22926
141.088	-0.52514	-0.22925
141.104	-0.52513	-0.22924
141.122	-0.52513	-0.22923
141.138	-0.52512	-0.22923
141.154	-0.52512	-0.22922
141.171	-0.52512	-0.22922
141.188	-0.52512	-0.22921
141.204	-0.52511	-0.22921
141.222	-0.52511	-0.2292
141.238	-0.5251	-0.22919
141.254	-0.5251	-0.22918
141.271	-0.5251	-0.22918
141.288	-0.5251	-0.22917
141.304	-0.52509	-0.22916
141.322	-0.52509	-0.22915
141.338	-0.52509	-0.22914
141.354	-0.52509	-0.22913
141.371	-0.5251	-0.22912
141.388	-0.5251	-0.22912
141.404	-0.5251	-0.22911
141.421	-0.5251	-0.2291
141.438	-0.52509	-0.2291
141.455	-0.52509	-0.22909
141.471	-0.52509	-0.22909
141.488	-0.5251	-0.22909
141.504	-0.5251	-0.22908
141.522	-0.52509	-0.22908
141.538	-0.52509	-0.22907
141.555	-0.52509	-0.22906

141.572	-0.52509	-0.22906
141.588	-0.52509	-0.22905
141.605	-0.52508	-0.22904
141.622	-0.52508	-0.22904
141.638	-0.52507	-0.22903
141.654	-0.52507	-0.22902
141.671	-0.52508	-0.22902
141.688	-0.52508	-0.22901
141.705	-0.52508	-0.229
141.721	-0.52508	-0.22899
141.737	-0.52509	-0.22899
141.755	-0.52509	-0.22898
141.771	-0.52509	-0.22897
141.788	-0.52509	-0.22897
141.804	-0.52509	-0.22897
141.822	-0.52509	-0.22896
141.839	-0.52509	-0.22896
141.855	-0.52509	-0.22895
141.872	-0.5251	-0.22894
141.889	-0.5251	-0.22893
141.905	-0.5251	-0.22892
141.921	-0.5251	-0.22891
141.938	-0.52511	-0.2289
141.955	-0.52511	-0.22889
141.971	-0.52511	-0.22888
141.988	-0.52511	-0.22887
142.004	-0.52511	-0.22887
142.022	-0.5251	-0.22886
142.038	-0.52511	-0.22886
142.055	-0.52511	-0.22886

142.072	-0.52511	-0.22885
142.089	-0.52511	-0.22885
142.105	-0.52512	-0.22884
142.122	-0.52512	-0.22884
142.139	-0.52512	-0.22883
142.155	-0.52513	-0.22882
142.171	-0.52513	-0.22881
142.189	-0.52514	-0.22881
142.206	-0.52514	-0.2288
142.222	-0.52515	-0.2288
142.239	-0.52515	-0.22879
142.255	-0.52515	-0.22879
142.272	-0.52516	-0.22879
142.289	-0.52516	-0.22878
142.306	-0.52516	-0.22877
142.323	-0.52517	-0.22876
142.339	-0.52518	-0.22875
142.355	-0.52519	-0.22874
142.371	-0.5252	-0.22873
142.389	-0.52522	-0.22872
142.405	-0.52524	-0.22871
142.423	-0.52526	-0.2287
142.438	-0.52528	-0.22869
142.455	-0.52529	-0.22868
142.471	-0.52531	-0.22868
142.489	-0.52532	-0.22867
142.506	-0.52535	-0.22867
142.522	-0.5254	-0.22866
142.538	-0.52546	-0.22866
142.556	-0.52555	-0.22865

142.572	-0.52568	-0.22864
142.589	-0.52576	-0.22863
142.605	-0.52587	-0.22863
142.622	-0.52599	-0.22862
142.64	-0.52609	-0.22861
142.656	-0.52617	-0.22861
142.672	-0.5262	-0.2286
142.689	-0.52623	-0.2286
142.705	-0.52625	-0.22859
142.722	-0.52627	-0.22858
142.74	-0.5263	-0.22858
142.756	-0.52634	-0.22857
142.773	-0.52637	-0.22856
142.789	-0.52642	-0.22855
142.806	-0.52648	-0.22854
142.822	-0.52652	-0.22853
142.839	-0.52656	-0.22852
142.855	-0.52659	-0.22852
142.872	-0.52661	-0.22851
142.889	-0.52663	-0.2285
142.904	-0.52665	-0.22849
142.922	-0.52667	-0.22849
142.939	-0.52668	-0.22848
142.956	-0.5267	-0.22847
142.972	-0.52672	-0.22846
142.989	-0.52674	-0.22844
143.006	-0.52676	-0.22843
143.022	-0.52678	-0.22842
143.039	-0.5268	-0.22842
143.055	-0.52682	-0.22841

143.072	-0.52684	-0.2284
143.09	-0.52686	-0.2284
143.106	-0.52688	-0.22839
143.122	-0.5269	-0.22839
143.139	-0.52692	-0.22839
143.155	-0.52693	-0.22838
143.173	-0.52695	-0.22837
143.189	-0.52697	-0.22837
143.205	-0.52699	-0.22836
143.222	-0.52701	-0.22835
143.239	-0.52702	-0.22834
143.256	-0.52704	-0.22833
143.271	-0.52706	-0.22832
143.289	-0.52707	-0.22831
143.305	-0.52709	-0.2283
143.323	-0.5271	-0.2283
143.338	-0.52711	-0.22829
143.356	-0.52713	-0.22828
143.372	-0.52714	-0.22828
143.389	-0.52716	-0.22828
143.406	-0.52717	-0.22827
143.422	-0.52718	-0.22827
143.438	-0.52719	-0.22826
143.456	-0.5272	-0.22825
143.473	-0.52721	-0.22824
143.49	-0.52721	-0.22823
143.506	-0.52722	-0.22823
143.522	-0.52724	-0.22822
143.539	-0.52725	-0.22822
143.556	-0.52726	-0.22821

143.573	-0.52727	-0.22821
143.589	-0.52728	-0.2282
143.606	-0.52729	-0.2282
143.623	-0.5273	-0.22819
143.639	-0.52731	-0.22818
143.656	-0.52731	-0.22817
143.674	-0.52732	-0.22816
143.69	-0.52733	-0.22816
143.707	-0.52733	-0.22815
143.723	-0.52734	-0.22814
143.739	-0.52734	-0.22813
143.756	-0.52735	-0.22813
143.773	-0.52736	-0.22812
143.79	-0.52736	-0.22811
143.806	-0.52737	-0.22811
143.823	-0.52737	-0.22811
143.84	-0.52738	-0.2281
143.857	-0.52738	-0.2281
143.873	-0.52739	-0.2281
143.891	-0.52739	-0.2281
143.906	-0.5274	-0.22809
143.923	-0.52741	-0.22809
143.94	-0.52742	-0.22808
143.956	-0.52743	-0.22807
143.973	-0.52743	-0.22806
143.989	-0.52744	-0.22806
144.006	-0.52745	-0.22805
144.023	-0.52745	-0.22804
144.039	-0.52746	-0.22804
144.057	-0.52747	-0.22803

144.073	-0.52747	-0.22803
144.089	-0.52748	-0.22802
144.106	-0.52748	-0.22802
144.123	-0.52748	-0.22801
144.14	-0.52749	-0.228
144.156	-0.52749	-0.22799
144.172	-0.5275	-0.22798
144.189	-0.5275	-0.22797
144.207	-0.52751	-0.22796
144.223	-0.52751	-0.22795
144.24	-0.52751	-0.22795
144.257	-0.52752	-0.22794
144.274	-0.52752	-0.22794
144.29	-0.52753	-0.22793
144.306	-0.52753	-0.22793
144.324	-0.52753	-0.22792
144.34	-0.52753	-0.22792
144.356	-0.52753	-0.22791
144.373	-0.52753	-0.2279
144.39	-0.52753	-0.2279
144.406	-0.52753	-0.22789
144.423	-0.52752	-0.22788
144.44	-0.52752	-0.22787
144.456	-0.52752	-0.22786
144.473	-0.52752	-0.22785
144.49	-0.52753	-0.22784
144.506	-0.52753	-0.22783
144.523	-0.52753	-0.22782
144.54	-0.52754	-0.22781
144.555	-0.52754	-0.2278

144.572	-0.52754	-0.2278
144.589	-0.52755	-0.22779
144.607	-0.52755	-0.22778
144.622	-0.52755	-0.22777
144.639	-0.52756	-0.22776
144.656	-0.52756	-0.22776
144.673	-0.52756	-0.22775
144.69	-0.52757	-0.22775
144.706	-0.52757	-0.22774
144.723	-0.52757	-0.22773
144.741	-0.52757	-0.22772
144.757	-0.52757	-0.22772
144.773	-0.52757	-0.22771
144.79	-0.52757	-0.22769
144.806	-0.52757	-0.22768
144.823	-0.52758	-0.22767
144.84	-0.52759	-0.22765
144.856	-0.52759	-0.22764
144.873	-0.5276	-0.22763
144.889	-0.5276	-0.22762
144.907	-0.52761	-0.22761
144.923	-0.52761	-0.2276
144.939	-0.52761	-0.22759
144.956	-0.52761	-0.22758
144.973	-0.52761	-0.22757
144.989	-0.52761	-0.22756
145.006	-0.52762	-0.22755
145.023	-0.52763	-0.22755
145.04	-0.52764	-0.22754
145.057	-0.52764	-0.22753

145.074	-0.52765	-0.22752
145.088	-0.52765	-0.22751
145.106	-0.52765	-0.2275
145.123	-0.52766	-0.2275
145.14	-0.52766	-0.22749
145.156	-0.52767	-0.22748
145.173	-0.52768	-0.22748
145.189	-0.52768	-0.22748
145.207	-0.52769	-0.22747
145.223	-0.5277	-0.22747
145.24	-0.52771	-0.22746
145.257	-0.52772	-0.22746
145.273	-0.52774	-0.22745
145.29	-0.52775	-0.22744
145.306	-0.52776	-0.22743
145.323	-0.52777	-0.22742
145.341	-0.52777	-0.22741
145.357	-0.52777	-0.2274
145.373	-0.52778	-0.22739
145.39	-0.52778	-0.22738
145.406	-0.52778	-0.22738
145.423	-0.52779	-0.22737
145.44	-0.5278	-0.22737
145.456	-0.52781	-0.22736
145.473	-0.52782	-0.22736
145.49	-0.52783	-0.22736
145.506	-0.52785	-0.22735
145.524	-0.52786	-0.22734
145.541	-0.52787	-0.22734
145.557	-0.52788	-0.22733

145.574	-0.5279	-0.22732
145.591	-0.52791	-0.22731
145.607	-0.52792	-0.2273
145.623	-0.52793	-0.2273
145.639	-0.52794	-0.22729
145.656	-0.52795	-0.22729
145.674	-0.52796	-0.22728
145.689	-0.52797	-0.22728
145.707	-0.52798	-0.22727
145.722	-0.52799	-0.22727
145.74	-0.528	-0.22726
145.757	-0.52802	-0.22726
145.774	-0.52803	-0.22725
145.79	-0.52804	-0.22724
145.807	-0.52805	-0.22724
145.824	-0.52806	-0.22723
145.84	-0.52807	-0.22722
145.858	-0.52808	-0.22721
145.874	-0.52809	-0.22721
145.89	-0.52809	-0.2272
145.907	-0.5281	-0.22719
145.923	-0.52811	-0.22719
145.94	-0.52812	-0.22718
145.957	-0.52813	-0.22717
145.973	-0.52815	-0.22716
145.99	-0.52816	-0.22716
146.007	-0.52817	-0.22715
146.023	-0.52817	-0.22714
146.04	-0.52819	-0.22714
146.057	-0.52819	-0.22713

146.074	-0.5282	-0.22713
146.091	-0.52821	-0.22712
146.108	-0.52822	-0.22711
146.123	-0.52823	-0.22711
146.14	-0.52824	-0.2271
146.157	-0.52825	-0.22709
146.174	-0.52826	-0.22709
146.19	-0.52827	-0.22708
146.207	-0.52828	-0.22708
146.224	-0.52829	-0.22707
146.241	-0.5283	-0.22706
146.257	-0.52831	-0.22705
146.274	-0.52832	-0.22704
146.29	-0.52833	-0.22704
146.307	-0.52834	-0.22703
146.324	-0.52836	-0.22702
146.341	-0.52838	-0.22701
146.356	-0.52839	-0.22701
146.374	-0.52841	-0.227
146.391	-0.52843	-0.22699
146.407	-0.52845	-0.22699
146.423	-0.52846	-0.22698
146.44	-0.52848	-0.22697
146.457	-0.52849	-0.22697
146.474	-0.5285	-0.22696
146.49	-0.52851	-0.22696
146.507	-0.52852	-0.22696
146.525	-0.52853	-0.22695
146.54	-0.52854	-0.22695
146.558	-0.52855	-0.22694

146.574	-0.52856	-0.22694
146.59	-0.52858	-0.22693
146.608	-0.52859	-0.22692
146.624	-0.5286	-0.22692
146.64	-0.52861	-0.22691
146.657	-0.52862	-0.2269
146.674	-0.52864	-0.2269
146.69	-0.52865	-0.22689
146.708	-0.52867	-0.22688
146.724	-0.52869	-0.22687
146.741	-0.52871	-0.22686
146.758	-0.52872	-0.22685
146.774	-0.52874	-0.22685
146.791	-0.52876	-0.22684
146.808	-0.52878	-0.22683
146.824	-0.52879	-0.22683
146.841	-0.5288	-0.22682
146.858	-0.52882	-0.22682
146.875	-0.52883	-0.22682
146.891	-0.52885	-0.22682
146.909	-0.52886	-0.22682
146.924	-0.52888	-0.22682
146.941	-0.52889	-0.22681
146.958	-0.5289	-0.2268
146.974	-0.52892	-0.2268
146.992	-0.52893	-0.22679
147.007	-0.52894	-0.22678
147.024	-0.52895	-0.22677
147.041	-0.52897	-0.22677
147.058	-0.52898	-0.22677

147.074	-0.52899	-0.22677
147.091	-0.52901	-0.22676
147.108	-0.52902	-0.22676
147.124	-0.52903	-0.22675
147.142	-0.52905	-0.22675
147.158	-0.52907	-0.22674
147.176	-0.52908	-0.22674
147.192	-0.52909	-0.22673
147.209	-0.5291	-0.22672
147.225	-0.52911	-0.22671
147.242	-0.52913	-0.2267
147.258	-0.52914	-0.22669
147.275	-0.52915	-0.22669
147.292	-0.52917	-0.22668
147.308	-0.52918	-0.22668
147.325	-0.52919	-0.22667
147.342	-0.5292	-0.22667
147.359	-0.52921	-0.22666
147.374	-0.52923	-0.22666
147.392	-0.52924	-0.22665
147.408	-0.52926	-0.22664
147.425	-0.52927	-0.22664
147.443	-0.52929	-0.22663
147.459	-0.52931	-0.22663
147.476	-0.52933	-0.22662
147.492	-0.52934	-0.22662
147.509	-0.52935	-0.22661
147.526	-0.52936	-0.2266
147.542	-0.52937	-0.2266
147.559	-0.52938	-0.22659

147.575	-0.52939	-0.22658
147.592	-0.5294	-0.22656
147.608	-0.52941	-0.22656
147.625	-0.52943	-0.22655
147.642	-0.52944	-0.22654
147.658	-0.52945	-0.22654
147.675	-0.52946	-0.22654
147.691	-0.52946	-0.22653
147.708	-0.52946	-0.22652
147.725	-0.52947	-0.22652
147.742	-0.52947	-0.22651
147.758	-0.52948	-0.2265
147.775	-0.52949	-0.22649
147.792	-0.52951	-0.22649
147.809	-0.52952	-0.22648
147.825	-0.52954	-0.22647
147.842	-0.52955	-0.22647
147.859	-0.52957	-0.22646
147.875	-0.52958	-0.22646
147.893	-0.52959	-0.22645
147.909	-0.52961	-0.22645
147.925	-0.52962	-0.22644
147.941	-0.52963	-0.22643
147.959	-0.52964	-0.22643
147.975	-0.52965	-0.22642
147.992	-0.52966	-0.22641
148.008	-0.52967	-0.2264
148.025	-0.52968	-0.22639
148.042	-0.52969	-0.22638
148.058	-0.5297	-0.22637

148.075	-0.52971	-0.22636
148.09	-0.52972	-0.22635
148.108	-0.52973	-0.22634
148.126	-0.52973	-0.22634
148.141	-0.52974	-0.22633
148.158	-0.52975	-0.22632
148.174	-0.52976	-0.22631
148.192	-0.52977	-0.2263
148.208	-0.52978	-0.22629
148.225	-0.52979	-0.22629
148.241	-0.5298	-0.22628
148.259	-0.52981	-0.22627
148.275	-0.52982	-0.22627
148.292	-0.52983	-0.22626
148.308	-0.52984	-0.22625
148.325	-0.52984	-0.22625
148.341	-0.52985	-0.22624
148.358	-0.52985	-0.22623
148.375	-0.52987	-0.22622
148.391	-0.52987	-0.22622
148.408	-0.52989	-0.22621
148.424	-0.5299	-0.2262
148.441	-0.52991	-0.22619
148.458	-0.52993	-0.22618
148.474	-0.52995	-0.22618
148.49	-0.52996	-0.22617
148.507	-0.52997	-0.22617
148.524	-0.52999	-0.22617
148.541	-0.53	-0.22617
148.558	-0.53002	-0.22616

148.574	-0.53003	-0.22616
148.591	-0.53004	-0.22616
148.608	-0.53006	-0.22615
148.625	-0.53007	-0.22614
148.641	-0.53008	-0.22614
148.657	-0.53009	-0.22613
148.674	-0.53011	-0.22612
148.691	-0.53012	-0.22612
148.707	-0.53013	-0.22611
148.725	-0.53014	-0.2261
148.741	-0.53016	-0.22609
148.758	-0.53017	-0.22608
148.774	-0.53018	-0.22607
148.79	-0.5302	-0.22607
148.808	-0.53021	-0.22606
148.825	-0.53022	-0.22605
148.841	-0.53024	-0.22605
148.858	-0.53025	-0.22605
148.874	-0.53027	-0.22604
148.892	-0.53029	-0.22604
148.908	-0.5303	-0.22604
148.925	-0.53031	-0.22603
148.941	-0.53033	-0.22603
148.958	-0.53033	-0.22602
148.974	-0.53034	-0.22601
148.991	-0.53036	-0.226
149.008	-0.53037	-0.226
149.025	-0.53038	-0.22599
149.042	-0.5304	-0.22599
149.058	-0.53041	-0.22599

149.074	-0.53043	-0.22598
149.091	-0.53044	-0.22598
149.108	-0.53046	-0.22598
149.125	-0.53048	-0.22598
149.142	-0.53049	-0.22598
149.158	-0.53051	-0.22598
149.175	-0.53052	-0.22597
149.191	-0.53054	-0.22597
149.209	-0.53055	-0.22597
149.225	-0.53057	-0.22596
149.242	-0.53058	-0.22596
149.258	-0.5306	-0.22595
149.274	-0.53062	-0.22595
149.291	-0.53063	-0.22594
149.308	-0.53065	-0.22594
149.324	-0.53067	-0.22593
149.341	-0.53069	-0.22592
149.358	-0.53071	-0.22592
149.374	-0.53072	-0.22591
149.391	-0.53074	-0.22591
149.408	-0.53076	-0.2259
149.425	-0.53078	-0.22589
149.441	-0.53079	-0.22589
149.458	-0.53081	-0.22589
149.475	-0.53083	-0.22589
149.491	-0.53084	-0.22589
149.509	-0.53085	-0.22589
149.525	-0.53087	-0.22589
149.541	-0.53089	-0.22589
149.558	-0.5309	-0.22589

149.575	-0.53091	-0.22588
149.592	-0.53092	-0.22588
149.609	-0.53094	-0.22587
149.626	-0.53095	-0.22587
149.642	-0.53097	-0.22586
149.658	-0.53099	-0.22586
149.676	-0.531	-0.22585
149.693	-0.53102	-0.22584
149.709	-0.53103	-0.22584
149.726	-0.53105	-0.22584
149.742	-0.53107	-0.22583
149.759	-0.53109	-0.22583
149.775	-0.5311	-0.22582
149.792	-0.53112	-0.22581
149.809	-0.53114	-0.22581
149.826	-0.53115	-0.2258
149.842	-0.53117	-0.2258
149.858	-0.53118	-0.22579
149.875	-0.5312	-0.22579
149.891	-0.53121	-0.22578
149.908	-0.53122	-0.22578
149.925	-0.53124	-0.22577
149.941	-0.53126	-0.22577
149.958	-0.53127	-0.22577
149.976	-0.53129	-0.22577
149.992	-0.5313	-0.22577
150.009	-0.53131	-0.22577
150.026	-0.53133	-0.22576
150.042	-0.53135	-0.22576
150.058	-0.53137	-0.22575

150.075	-0.53139	-0.22574
150.092	-0.5314	-0.22574
150.109	-0.53142	-0.22573
150.124	-0.53143	-0.22572
150.142	-0.53144	-0.22571
150.159	-0.53146	-0.22571
150.175	-0.53147	-0.2257
150.193	-0.53148	-0.2257
150.209	-0.53149	-0.22569
150.225	-0.5315	-0.22569
150.242	-0.53151	-0.22569
150.259	-0.53152	-0.22569
150.275	-0.53153	-0.22569
150.293	-0.53155	-0.22568
150.309	-0.53156	-0.22568
150.325	-0.53158	-0.22568
150.342	-0.5316	-0.22567
150.359	-0.53161	-0.22567
150.375	-0.53163	-0.22566
150.392	-0.53165	-0.22566
150.408	-0.53167	-0.22565
150.425	-0.53169	-0.22565
150.442	-0.5317	-0.22564
150.458	-0.53172	-0.22564
150.476	-0.53173	-0.22563
150.492	-0.53174	-0.22563
150.508	-0.53175	-0.22563
150.525	-0.53176	-0.22562
150.541	-0.53178	-0.22562
150.559	-0.53179	-0.22561

150.576	-0.5318	-0.22561
150.593	-0.53182	-0.22561
150.609	-0.53183	-0.2256
150.625	-0.53185	-0.2256
150.642	-0.53187	-0.22559
150.659	-0.53189	-0.22559
150.675	-0.5319	-0.22558
150.691	-0.53192	-0.22558
150.709	-0.53193	-0.22557
150.724	-0.53194	-0.22557
150.741	-0.53195	-0.22556
150.759	-0.53197	-0.22556
150.776	-0.53198	-0.22555
150.792	-0.532	-0.22555
150.809	-0.53201	-0.22554
150.825	-0.53203	-0.22554
150.842	-0.53205	-0.22553
150.857	-0.53207	-0.22553
150.875	-0.53208	-0.22553
150.892	-0.5321	-0.22552
150.908	-0.53212	-0.22552
150.924	-0.53213	-0.22551
150.941	-0.53214	-0.22551
150.958	-0.53215	-0.2255
150.975	-0.53217	-0.2255
150.991	-0.53219	-0.22549
151.008	-0.5322	-0.22549
151.024	-0.53222	-0.22548
151.042	-0.53224	-0.22548
151.058	-0.53227	-0.22548

151.074	-0.53229	-0.22547
151.092	-0.53231	-0.22547
151.108	-0.53233	-0.22546
151.125	-0.53234	-0.22545
151.141	-0.53236	-0.22545
151.158	-0.53237	-0.22544
151.175	-0.53239	-0.22543
151.191	-0.5324	-0.22543
151.208	-0.53242	-0.22543
151.225	-0.53243	-0.22543
151.241	-0.53245	-0.22543
151.259	-0.53246	-0.22543
151.275	-0.53248	-0.22542
151.291	-0.53249	-0.22542
151.308	-0.53251	-0.22542
151.325	-0.53253	-0.22541
151.342	-0.53255	-0.22541
151.359	-0.53257	-0.2254
151.375	-0.53259	-0.2254
151.391	-0.53261	-0.22539
151.408	-0.53263	-0.22539
151.425	-0.53265	-0.22539
151.442	-0.53266	-0.22539
151.458	-0.53267	-0.22539
151.475	-0.53268	-0.22539
151.492	-0.5327	-0.22539
151.508	-0.53271	-0.22538
151.525	-0.53273	-0.22537
151.542	-0.53274	-0.22537
151.558	-0.53276	-0.22536

151.575	-0.53278	-0.22536
151.591	-0.53279	-0.22535
151.608	-0.53281	-0.22535
151.625	-0.53283	-0.22534
151.642	-0.53285	-0.22534
151.658	-0.53287	-0.22534
151.675	-0.53289	-0.22534
151.691	-0.53291	-0.22534
151.708	-0.53294	-0.22533
151.725	-0.53295	-0.22533
151.742	-0.53297	-0.22533
151.758	-0.53299	-0.22533
151.775	-0.53301	-0.22533
151.792	-0.53303	-0.22533
151.809	-0.53305	-0.22533
151.825	-0.53307	-0.22533
151.842	-0.53308	-0.22533
151.859	-0.5331	-0.22533
151.876	-0.53312	-0.22532
151.892	-0.53314	-0.22531
151.909	-0.53315	-0.22531
151.925	-0.53317	-0.2253
151.942	-0.53319	-0.2253
151.958	-0.53321	-0.2253
151.974	-0.53322	-0.22529
151.992	-0.53324	-0.22529
152.008	-0.53325	-0.22529
152.024	-0.53326	-0.22529
152.041	-0.53328	-0.22528
152.058	-0.53329	-0.22528

152.075	-0.53331	-0.22528
152.092	-0.53333	-0.22527
152.109	-0.53334	-0.22527
152.125	-0.53336	-0.22527
152.14	-0.53337	-0.22527
152.158	-0.53339	-0.22527
152.175	-0.53341	-0.22527
152.192	-0.53342	-0.22526
152.208	-0.53344	-0.22526
152.225	-0.53345	-0.22525
152.242	-0.53347	-0.22524
152.259	-0.53349	-0.22524
152.275	-0.53351	-0.22523
152.293	-0.53353	-0.22523
152.309	-0.53354	-0.22523
152.325	-0.53355	-0.22523
152.342	-0.53357	-0.22523
152.359	-0.53358	-0.22523
152.375	-0.5336	-0.22523
152.391	-0.53361	-0.22523
152.409	-0.53362	-0.22523
152.425	-0.53364	-0.22523
152.443	-0.53366	-0.22522
152.458	-0.53367	-0.22522
152.475	-0.53368	-0.22521
152.491	-0.5337	-0.22521
152.509	-0.53371	-0.22521
152.525	-0.53372	-0.22521
152.542	-0.53374	-0.22521
152.558	-0.53376	-0.2252

152.575	-0.53377	-0.2252
152.592	-0.53379	-0.2252
152.608	-0.53381	-0.2252
152.624	-0.53382	-0.22519
152.641	-0.53384	-0.22519
152.658	-0.53386	-0.22518
152.675	-0.53387	-0.22518
152.692	-0.53389	-0.22517
152.708	-0.5339	-0.22517
152.725	-0.53392	-0.22517
152.742	-0.53393	-0.22517
152.758	-0.53395	-0.22517
152.775	-0.53396	-0.22517
152.792	-0.53398	-0.22517
152.809	-0.534	-0.22517
152.825	-0.53402	-0.22516
152.841	-0.53403	-0.22516
152.858	-0.53405	-0.22516
152.875	-0.53406	-0.22515
152.891	-0.53408	-0.22515
152.908	-0.53409	-0.22515
152.925	-0.53409	-0.22514
152.942	-0.5341	-0.22514
152.959	-0.53411	-0.22514
152.974	-0.53412	-0.22514
152.992	-0.53413	-0.22514
153.008	-0.53414	-0.22514
153.025	-0.53416	-0.22514
153.042	-0.53417	-0.22514
153.059	-0.53419	-0.22515

153.076	-0.53421	-0.22514
153.093	-0.53423	-0.22514
153.109	-0.53425	-0.22514
153.125	-0.53427	-0.22513
153.143	-0.53429	-0.22513
153.159	-0.5343	-0.22512
153.176	-0.53432	-0.22512
153.192	-0.53434	-0.22511
153.209	-0.53436	-0.22511
153.225	-0.53437	-0.22511
153.241	-0.53438	-0.22511
153.258	-0.53439	-0.22511
153.276	-0.53441	-0.2251
153.292	-0.53442	-0.2251
153.309	-0.53444	-0.2251
153.326	-0.53445	-0.2251
153.341	-0.53446	-0.2251
153.359	-0.53448	-0.22509
153.375	-0.53449	-0.22509
153.391	-0.53451	-0.22508
153.408	-0.53452	-0.22508
153.425	-0.53453	-0.22508
153.442	-0.53454	-0.22508
153.458	-0.53455	-0.22508
153.475	-0.53456	-0.22508
153.492	-0.53457	-0.22509
153.509	-0.53458	-0.22509
153.526	-0.5346	-0.22509
153.543	-0.53462	-0.22509
153.559	-0.53464	-0.22509

153.576	-0.53466	-0.22508
153.592	-0.53468	-0.22508
153.609	-0.5347	-0.22508
153.626	-0.53472	-0.22508
153.642	-0.53474	-0.22508
153.658	-0.53476	-0.22508
153.675	-0.53477	-0.22508
153.692	-0.53479	-0.22508
153.709	-0.53481	-0.22508
153.726	-0.53483	-0.22509
153.742	-0.53485	-0.22509
153.759	-0.53486	-0.22509
153.776	-0.53488	-0.22509
153.793	-0.53489	-0.22509
153.809	-0.53491	-0.22509
153.826	-0.53492	-0.22509
153.842	-0.53494	-0.22509
153.859	-0.53496	-0.22508
153.876	-0.53498	-0.22508
153.893	-0.535	-0.22508
153.91	-0.53503	-0.22508
153.926	-0.53505	-0.22508
153.943	-0.53507	-0.22508
153.96	-0.53509	-0.22508
153.976	-0.53511	-0.22508
153.992	-0.53513	-0.22508
154.009	-0.53514	-0.22508
154.026	-0.53516	-0.22507
154.042	-0.53517	-0.22507
154.06	-0.53519	-0.22507

154.076	-0.53521	-0.22507
154.093	-0.53523	-0.22507
154.109	-0.53525	-0.22507
154.127	-0.53527	-0.22508
154.142	-0.53529	-0.22508
154.16	-0.53531	-0.22508
154.177	-0.53532	-0.22508
154.193	-0.53534	-0.22508
154.211	-0.53536	-0.22508
154.227	-0.53538	-0.22507
154.243	-0.53539	-0.22507
154.26	-0.53541	-0.22506
154.277	-0.53543	-0.22506
154.294	-0.53544	-0.22506
154.31	-0.53546	-0.22506
154.326	-0.53547	-0.22506
154.343	-0.53549	-0.22506
154.361	-0.5355	-0.22507
154.377	-0.53552	-0.22507
154.394	-0.53554	-0.22507
154.411	-0.53556	-0.22507
154.427	-0.53558	-0.22507
154.444	-0.53561	-0.22507
154.46	-0.53563	-0.22507
154.478	-0.53566	-0.22507
154.493	-0.53569	-0.22507
154.51	-0.53571	-0.22507
154.527	-0.53572	-0.22506
154.544	-0.53574	-0.22506
154.561	-0.53576	-0.22506

154.577	-0.53578	-0.22506
154.593	-0.53579	-0.22507
154.61	-0.53581	-0.22507
154.627	-0.53583	-0.22507
154.644	-0.53585	-0.22507
154.66	-0.53587	-0.22508
154.677	-0.53589	-0.22508
154.694	-0.53591	-0.22508
154.71	-0.53592	-0.22508
154.728	-0.53594	-0.22507
154.744	-0.53596	-0.22507
154.76	-0.53597	-0.22507
154.777	-0.53598	-0.22507
154.793	-0.53599	-0.22506
154.811	-0.536	-0.22506
154.827	-0.53602	-0.22505
154.844	-0.53603	-0.22505
154.86	-0.53605	-0.22504
154.876	-0.53607	-0.22504
154.894	-0.53609	-0.22504
154.911	-0.53611	-0.22504
154.927	-0.53612	-0.22504
154.944	-0.53614	-0.22504
154.96	-0.53615	-0.22504
154.977	-0.53617	-0.22504
154.993	-0.53618	-0.22504
155.01	-0.53619	-0.22503
155.027	-0.53621	-0.22503
155.045	-0.53622	-0.22503
155.06	-0.53623	-0.22502

155.078	-0.53624	-0.22502
155.094	-0.53626	-0.22501
155.11	-0.53628	-0.22501
155.127	-0.53629	-0.22501
155.144	-0.53631	-0.225
155.16	-0.53632	-0.225
155.177	-0.53634	-0.225
155.194	-0.53634	-0.225
155.211	-0.53635	-0.225
155.228	-0.53636	-0.225
155.244	-0.53637	-0.225
155.26	-0.53638	-0.225
155.277	-0.53638	-0.22499
155.294	-0.53639	-0.22499
155.311	-0.5364	-0.22498
155.328	-0.53642	-0.22498
155.344	-0.53643	-0.22497
155.36	-0.53645	-0.22497
155.376	-0.53646	-0.22496
155.393	-0.53647	-0.22496
155.41	-0.53649	-0.22495
155.427	-0.5365	-0.22495
155.444	-0.53652	-0.22494
155.46	-0.53653	-0.22494
155.476	-0.53654	-0.22494
155.493	-0.53656	-0.22494
155.51	-0.53657	-0.22494
155.527	-0.53658	-0.22494
155.543	-0.5366	-0.22494
155.56	-0.53661	-0.22494

155.576	-0.53662	-0.22494
155.594	-0.53664	-0.22494
155.61	-0.53665	-0.22494
155.627	-0.53666	-0.22494
155.643	-0.53667	-0.22493
155.66	-0.53669	-0.22493
155.676	-0.5367	-0.22492
155.694	-0.53672	-0.22492
155.71	-0.53673	-0.22491
155.727	-0.53674	-0.22491
155.744	-0.53675	-0.2249
155.761	-0.53676	-0.2249
155.777	-0.53678	-0.2249
155.794	-0.53679	-0.2249
155.81	-0.5368	-0.2249
155.827	-0.53682	-0.2249
155.843	-0.53683	-0.22489
155.861	-0.53685	-0.22489
155.878	-0.53687	-0.22489
155.894	-0.53689	-0.22489
155.911	-0.53691	-0.22489
155.928	-0.53693	-0.22488
155.944	-0.53694	-0.22488
155.96	-0.53695	-0.22488
155.976	-0.53697	-0.22488
155.994	-0.53698	-0.22488
156.01	-0.537	-0.22488
156.028	-0.53702	-0.22488
156.044	-0.53703	-0.22488
156.06	-0.53704	-0.22488

156.076	-0.53706	-0.22488
156.093	-0.53707	-0.22488
156.11	-0.53709	-0.22488
156.127	-0.5371	-0.22488
156.144	-0.53712	-0.22488
156.161	-0.53713	-0.22489
156.177	-0.53715	-0.22489
156.194	-0.53717	-0.22489
156.21	-0.53719	-0.22489
156.228	-0.53721	-0.2249
156.244	-0.53723	-0.2249
156.261	-0.53726	-0.22489
156.277	-0.53728	-0.22489
156.295	-0.5373	-0.22488
156.31	-0.53733	-0.22488
156.327	-0.53735	-0.22487
156.344	-0.53737	-0.22487
156.36	-0.53739	-0.22487
156.378	-0.53741	-0.22487
156.393	-0.53743	-0.22487
156.411	-0.53745	-0.22487
156.428	-0.53746	-0.22487
156.444	-0.53748	-0.22488
156.46	-0.5375	-0.22488
156.478	-0.53751	-0.22488
156.495	-0.53753	-0.22487
156.511	-0.53755	-0.22487
156.527	-0.53756	-0.22487
156.544	-0.53758	-0.22486
156.561	-0.5376	-0.22486

156.578	-0.53761	-0.22486
156.595	-0.53763	-0.22485
156.611	-0.53765	-0.22485
156.629	-0.53767	-0.22485
156.644	-0.53769	-0.22485
156.661	-0.53771	-0.22485
156.678	-0.53774	-0.22485
156.694	-0.53776	-0.22485
156.711	-0.53778	-0.22485
156.729	-0.5378	-0.22486
156.744	-0.53783	-0.22486
156.761	-0.53785	-0.22486
156.777	-0.53787	-0.22486
156.795	-0.53788	-0.22486
156.811	-0.53791	-0.22485
156.828	-0.53793	-0.22485
156.844	-0.53795	-0.22485
156.862	-0.53797	-0.22485
156.878	-0.53799	-0.22485
156.894	-0.53801	-0.22485
156.911	-0.53802	-0.22485
156.928	-0.53804	-0.22485
156.945	-0.53807	-0.22484
156.961	-0.53808	-0.22484
156.977	-0.5381	-0.22483
156.995	-0.53812	-0.22483
157.011	-0.53813	-0.22482
157.028	-0.53815	-0.22482
157.045	-0.53816	-0.22482
157.061	-0.53817	-0.22482

157.078	-0.53818	-0.22482
157.095	-0.53819	-0.22482
157.111	-0.53821	-0.22482
157.128	-0.53823	-0.22482
157.146	-0.53824	-0.22482
157.162	-0.53826	-0.22482
157.178	-0.53829	-0.22482
157.195	-0.5383	-0.22482
157.212	-0.53833	-0.22481
157.228	-0.53835	-0.22481
157.245	-0.53837	-0.22481
157.262	-0.53839	-0.22481
157.278	-0.53841	-0.2248
157.294	-0.53843	-0.2248
157.312	-0.53845	-0.2248
157.328	-0.53847	-0.2248
157.346	-0.53848	-0.2248
157.361	-0.5385	-0.2248
157.378	-0.53851	-0.2248
157.395	-0.53853	-0.22479
157.411	-0.53855	-0.22479
157.428	-0.53857	-0.22479
157.444	-0.53858	-0.22478
157.461	-0.5386	-0.22478
157.478	-0.53862	-0.22478
157.495	-0.53863	-0.22478
157.511	-0.53865	-0.22478
157.527	-0.53866	-0.22478
157.545	-0.53868	-0.22478
157.561	-0.53869	-0.22479

157.577	-0.53871	-0.22479
157.594	-0.53872	-0.22479
157.611	-0.53874	-0.22479
157.627	-0.53876	-0.22479
157.644	-0.53878	-0.22479
157.662	-0.53879	-0.22479
157.678	-0.53881	-0.22479
157.695	-0.53883	-0.22478
157.712	-0.53885	-0.22478
157.728	-0.53886	-0.22478
157.745	-0.53888	-0.22477
157.762	-0.53889	-0.22476
157.778	-0.5389	-0.22476
157.795	-0.53892	-0.22475
157.812	-0.53893	-0.22475
157.827	-0.53894	-0.22475
157.844	-0.53895	-0.22474
157.861	-0.53897	-0.22474
157.878	-0.53899	-0.22474
157.895	-0.53901	-0.22474
157.911	-0.53903	-0.22474
157.928	-0.53906	-0.22473
157.944	-0.53908	-0.22473
157.961	-0.53909	-0.22473
157.978	-0.53911	-0.22473
157.994	-0.53913	-0.22473
158.01	-0.53914	-0.22473
158.028	-0.53916	-0.22473
158.045	-0.53917	-0.22473
158.061	-0.53919	-0.22473

158.078	-0.53921	-0.22473
158.094	-0.53923	-0.22473
158.112	-0.53925	-0.22472
158.127	-0.53927	-0.22472
158.145	-0.53929	-0.22472
158.161	-0.53931	-0.22471
158.179	-0.53932	-0.22471
158.194	-0.53934	-0.22471
158.212	-0.53936	-0.2247
158.228	-0.53938	-0.2247
158.245	-0.53939	-0.2247
158.262	-0.53941	-0.2247
158.278	-0.53943	-0.2247
158.294	-0.53945	-0.2247
158.311	-0.53947	-0.2247
158.328	-0.5395	-0.2247
158.344	-0.53952	-0.2247
158.361	-0.53954	-0.2247
158.377	-0.53956	-0.2247
158.395	-0.53957	-0.2247
158.412	-0.53959	-0.22469
158.428	-0.53961	-0.22469
158.444	-0.53963	-0.22469
158.461	-0.53964	-0.22468
158.478	-0.53965	-0.22468
158.494	-0.53967	-0.22468
158.511	-0.53969	-0.22468
158.528	-0.53971	-0.22468
158.544	-0.53973	-0.22468
158.561	-0.53975	-0.22469

158.578	-0.53977	-0.22469
158.595	-0.53979	-0.22469
158.611	-0.53981	-0.22469
158.628	-0.53983	-0.22469
158.645	-0.53985	-0.22469
158.662	-0.53986	-0.22469
158.678	-0.53988	-0.22468
158.694	-0.5399	-0.22468
158.712	-0.53991	-0.22467
158.727	-0.53993	-0.22467
158.744	-0.53994	-0.22467
158.762	-0.53996	-0.22467
158.778	-0.53998	-0.22467
158.795	-0.53999	-0.22467
158.811	-0.54001	-0.22466
158.827	-0.54003	-0.22466
158.843	-0.54005	-0.22466
158.861	-0.54007	-0.22466
158.877	-0.54009	-0.22466
158.893	-0.54011	-0.22466
158.91	-0.54013	-0.22466
158.928	-0.54014	-0.22466
158.943	-0.54016	-0.22465
158.961	-0.54018	-0.22466
158.978	-0.5402	-0.22465
158.995	-0.54022	-0.22465
159.011	-0.54024	-0.22465
159.028	-0.54027	-0.22465
159.044	-0.54029	-0.22465
159.061	-0.54031	-0.22465

159.077	-0.54033	-0.22465
159.094	-0.54035	-0.22465
159.111	-0.54036	-0.22465
159.127	-0.54039	-0.22465
159.145	-0.54041	-0.22465
159.161	-0.54043	-0.22465
159.177	-0.54045	-0.22466
159.194	-0.54047	-0.22466
159.212	-0.54048	-0.22466
159.228	-0.54051	-0.22466
159.245	-0.54053	-0.22466
159.262	-0.54055	-0.22466
159.278	-0.54057	-0.22466
159.295	-0.5406	-0.22466
159.311	-0.54062	-0.22466
159.329	-0.54066	-0.22466
159.345	-0.5407	-0.22467
159.362	-0.54073	-0.22467
159.378	-0.54076	-0.22468
159.395	-0.54079	-0.22468
159.412	-0.54081	-0.22469
159.428	-0.54083	-0.22469
159.445	-0.54085	-0.22469
159.461	-0.54087	-0.22469
159.478	-0.5409	-0.22469
159.496	-0.54092	-0.22469
159.512	-0.54094	-0.22469
159.529	-0.54096	-0.22469
159.544	-0.54098	-0.22469
159.562	-0.541	-0.22469

159.578	-0.54103	-0.22469
159.595	-0.54105	-0.22469
159.612	-0.54108	-0.22469
159.628	-0.5411	-0.22469
159.645	-0.54112	-0.22469
159.662	-0.54115	-0.22469
159.678	-0.54117	-0.2247
159.695	-0.54119	-0.2247
159.713	-0.54122	-0.2247
159.73	-0.54124	-0.2247
159.746	-0.54127	-0.2247
159.763	-0.54129	-0.2247
159.779	-0.54131	-0.2247
159.795	-0.54134	-0.22469
159.812	-0.54136	-0.22469
159.829	-0.54138	-0.22469
159.846	-0.5414	-0.22469
159.862	-0.54142	-0.22469
159.879	-0.54144	-0.2247
159.896	-0.54146	-0.2247
159.913	-0.54148	-0.2247
159.929	-0.5415	-0.2247
159.945	-0.54152	-0.22471
159.962	-0.54154	-0.22471
159.979	-0.54156	-0.22471
159.996	-0.54159	-0.22471
160.013	-0.54161	-0.22471
160.029	-0.54163	-0.22471
160.045	-0.54165	-0.22471
160.062	-0.54167	-0.22471

160.079	-0.54169	-0.2247
160.096	-0.54171	-0.2247
160.113	-0.54173	-0.2247
160.128	-0.54176	-0.22471
160.147	-0.54178	-0.22471
160.163	-0.5418	-0.22472
160.179	-0.54181	-0.22472
160.197	-0.54183	-0.22473
160.213	-0.54185	-0.22473
160.229	-0.54187	-0.22473
160.246	-0.54189	-0.22473
160.263	-0.54191	-0.22473
160.279	-0.54193	-0.22473
160.296	-0.54195	-0.22473
160.313	-0.54197	-0.22473
160.329	-0.54199	-0.22472
160.346	-0.54201	-0.22472
160.363	-0.54203	-0.22472
160.38	-0.54205	-0.22472
160.396	-0.54207	-0.22472
160.413	-0.54209	-0.22472
160.429	-0.54211	-0.22472
160.446	-0.54212	-0.22472
160.463	-0.54214	-0.22472
160.48	-0.54216	-0.22472
160.496	-0.54217	-0.22472
160.513	-0.54219	-0.22472
160.53	-0.54221	-0.22472
160.546	-0.54223	-0.22472
160.563	-0.54225	-0.22472

160.58	-0.54227	-0.22471
160.595	-0.54229	-0.22471
160.613	-0.54231	-0.22471
160.63	-0.54232	-0.2247
160.646	-0.54234	-0.2247
160.662	-0.54236	-0.2247
160.68	-0.54238	-0.2247
160.697	-0.5424	-0.2247
160.712	-0.54242	-0.2247
160.729	-0.54244	-0.2247
160.746	-0.54246	-0.2247
160.763	-0.54248	-0.2247
160.78	-0.5425	-0.2247
160.796	-0.54251	-0.2247
160.813	-0.54253	-0.2247
160.83	-0.54255	-0.2247
160.846	-0.54256	-0.2247
160.863	-0.54258	-0.22469
160.879	-0.5426	-0.22469
160.896	-0.54262	-0.22469
160.913	-0.54264	-0.22469
160.93	-0.54266	-0.22469
160.946	-0.54267	-0.2247
160.963	-0.54269	-0.2247
160.979	-0.54271	-0.2247
160.996	-0.54273	-0.2247
161.013	-0.54275	-0.2247
161.029	-0.54277	-0.2247
161.046	-0.54279	-0.2247
161.063	-0.54281	-0.22469

161.079	-0.54283	-0.22469
161.096	-0.54284	-0.22469
161.113	-0.54286	-0.22469
161.129	-0.54287	-0.22469
161.146	-0.54289	-0.22469
161.162	-0.54291	-0.2247
161.18	-0.54292	-0.2247
161.196	-0.54295	-0.2247
161.213	-0.54297	-0.22471
161.23	-0.54299	-0.22471
161.246	-0.54302	-0.22471
161.263	-0.54304	-0.22471
161.28	-0.54306	-0.22471
161.297	-0.54309	-0.2247
161.312	-0.54311	-0.2247
161.33	-0.54313	-0.22469
161.346	-0.54315	-0.22469
161.362	-0.54317	-0.22469
161.379	-0.54319	-0.22469
161.396	-0.54321	-0.22469
161.413	-0.54322	-0.22469
161.429	-0.54324	-0.2247
161.446	-0.54325	-0.2247
161.463	-0.54327	-0.2247
161.48	-0.54329	-0.2247
161.497	-0.54331	-0.22471
161.512	-0.54333	-0.22471
161.529	-0.54335	-0.2247
161.546	-0.54337	-0.2247
161.563	-0.54338	-0.2247

161.58	-0.5434	-0.2247
161.596	-0.54342	-0.2247
161.613	-0.54343	-0.2247
161.63	-0.54345	-0.2247
161.646	-0.54346	-0.2247
161.663	-0.54348	-0.2247
161.679	-0.54349	-0.2247
161.696	-0.54351	-0.2247
161.713	-0.54353	-0.2247
161.73	-0.54355	-0.2247
161.746	-0.54357	-0.2247
161.763	-0.54359	-0.2247
161.78	-0.54361	-0.2247
161.796	-0.54363	-0.22469
161.813	-0.54364	-0.22469
161.83	-0.54366	-0.22469
161.846	-0.54367	-0.22469
161.863	-0.54368	-0.22469
161.88	-0.5437	-0.22468
161.896	-0.54371	-0.22468
161.912	-0.54373	-0.22469
161.929	-0.54375	-0.22469
161.946	-0.54377	-0.22469
161.962	-0.54379	-0.22469
161.979	-0.54381	-0.2247
161.996	-0.54383	-0.2247
162.013	-0.54385	-0.2247
162.029	-0.54387	-0.2247
162.046	-0.54388	-0.22469
162.062	-0.54391	-0.22469

162.079	-0.54393	-0.22469
162.096	-0.54394	-0.22469
162.112	-0.54396	-0.22468
162.129	-0.54398	-0.22468
162.146	-0.544	-0.22468
162.161	-0.54402	-0.22468
162.178	-0.54404	-0.22468
162.195	-0.54405	-0.22468
162.212	-0.54407	-0.22468
162.228	-0.54409	-0.22468
162.245	-0.54411	-0.22468
162.261	-0.54413	-0.22468
162.279	-0.54415	-0.22468
162.294	-0.54417	-0.22468
162.311	-0.5442	-0.22467
162.329	-0.54422	-0.22467
162.345	-0.54425	-0.22466
162.361	-0.54427	-0.22466
162.379	-0.54429	-0.22466
162.395	-0.54431	-0.22465
162.412	-0.54434	-0.22465
162.428	-0.54436	-0.22465
162.445	-0.54438	-0.22465
162.462	-0.54441	-0.22465
162.478	-0.54443	-0.22465
162.495	-0.54447	-0.22465
162.512	-0.5445	-0.22466
162.528	-0.54452	-0.22466
162.545	-0.54454	-0.22466
162.561	-0.54457	-0.22466

162.578	-0.5446	-0.22466
162.595	-0.54462	-0.22466
162.612	-0.54465	-0.22466
162.628	-0.54468	-0.22466
162.645	-0.5447	-0.22467
162.662	-0.54474	-0.22467
162.678	-0.54477	-0.22467
162.696	-0.5448	-0.22467
162.712	-0.54483	-0.22468
162.729	-0.54485	-0.22468
162.745	-0.54488	-0.22468
162.762	-0.54491	-0.22468
162.778	-0.54493	-0.22469
162.795	-0.54496	-0.22469
162.811	-0.54499	-0.22469
162.828	-0.54502	-0.22469
162.845	-0.54505	-0.2247
162.862	-0.54507	-0.2247
162.879	-0.5451	-0.22471
162.895	-0.54513	-0.22471
162.912	-0.54517	-0.22472
162.929	-0.54519	-0.22472
162.946	-0.54523	-0.22472
162.962	-0.54526	-0.22473
162.979	-0.54528	-0.22474
162.996	-0.54531	-0.22474
163.012	-0.54533	-0.22474
163.029	-0.54535	-0.22475
163.046	-0.54538	-0.22475
163.063	-0.5454	-0.22476

163.079	-0.54542	-0.22476
163.096	-0.54545	-0.22476
163.112	-0.54547	-0.22477
163.129	-0.5455	-0.22477
163.145	-0.54552	-0.22478
163.162	-0.54555	-0.22478
163.179	-0.54558	-0.22478
163.196	-0.54561	-0.22478
163.212	-0.54563	-0.22478
163.228	-0.54566	-0.22478
163.246	-0.54569	-0.22478
163.263	-0.54571	-0.22479
163.279	-0.54574	-0.22479
163.295	-0.54576	-0.2248
163.313	-0.54578	-0.2248
163.33	-0.5458	-0.22481
163.347	-0.54582	-0.22482
163.362	-0.54584	-0.22483
163.38	-0.54586	-0.22483
163.396	-0.54588	-0.22484
163.413	-0.5459	-0.22484
163.429	-0.54592	-0.22484
163.446	-0.54595	-0.22484
163.462	-0.54597	-0.22484
163.479	-0.546	-0.22484
163.496	-0.54602	-0.22484
163.513	-0.54604	-0.22484
163.53	-0.54607	-0.22485
163.546	-0.54609	-0.22485
163.564	-0.54611	-0.22486

163.58	-0.54613	-0.22486
163.597	-0.54615	-0.22487
163.613	-0.54617	-0.22487
163.63	-0.5462	-0.22487
163.647	-0.54622	-0.22487
163.663	-0.54625	-0.22487
163.68	-0.54627	-0.22487
163.697	-0.54629	-0.22488
163.713	-0.54631	-0.22488
163.729	-0.54633	-0.22488
163.746	-0.54635	-0.22489
163.763	-0.54637	-0.22489
163.779	-0.54639	-0.22489
163.797	-0.54641	-0.22488
163.812	-0.54643	-0.22488
163.829	-0.54645	-0.22488
163.847	-0.54647	-0.22487
163.863	-0.54649	-0.22487
163.879	-0.5465	-0.22486
163.896	-0.54651	-0.22486
163.912	-0.54652	-0.22486
163.929	-0.54654	-0.22486
163.945	-0.54655	-0.22485
163.962	-0.54656	-0.22485
163.978	-0.54658	-0.22486
163.995	-0.54659	-0.22486
164.012	-0.54661	-0.22486
164.03	-0.54663	-0.22486
164.046	-0.54665	-0.22486
164.062	-0.54667	-0.22486

164.079	-0.54669	-0.22486
164.095	-0.54671	-0.22486
164.112	-0.54673	-0.22486
164.13	-0.54675	-0.22486
164.146	-0.54676	-0.22486
164.162	-0.54678	-0.22486
164.179	-0.54679	-0.22486
164.196	-0.5468	-0.22486
164.212	-0.54682	-0.22486
164.228	-0.54683	-0.22486
164.245	-0.54685	-0.22485
164.262	-0.54686	-0.22485
164.279	-0.54688	-0.22485
164.295	-0.54689	-0.22485
164.312	-0.54691	-0.22486
164.329	-0.54693	-0.22486
164.346	-0.54695	-0.22486
164.363	-0.54697	-0.22486
164.379	-0.54699	-0.22487
164.395	-0.54701	-0.22487
164.412	-0.54703	-0.22487
164.429	-0.54705	-0.22488
164.445	-0.54706	-0.22487
164.462	-0.54708	-0.22488
164.479	-0.54709	-0.22488
164.496	-0.5471	-0.22488
164.513	-0.54711	-0.22488
164.528	-0.54713	-0.22488
164.546	-0.54714	-0.22488
164.563	-0.54716	-0.22489

164.579	-0.54718	-0.22489
164.595	-0.5472	-0.22489
164.612	-0.54722	-0.22488
164.629	-0.54724	-0.22488
164.646	-0.54726	-0.22488
164.663	-0.54728	-0.22488
164.679	-0.5473	-0.22488
164.696	-0.54732	-0.22487
164.713	-0.54734	-0.22488
164.729	-0.54736	-0.22488
164.746	-0.54737	-0.22488
164.762	-0.54739	-0.22489
164.779	-0.5474	-0.22489
164.796	-0.54742	-0.2249
164.812	-0.54743	-0.2249
164.829	-0.54745	-0.22491
164.845	-0.54747	-0.22491
164.862	-0.54749	-0.22492
164.879	-0.54751	-0.22492
164.896	-0.54753	-0.22492
164.913	-0.54756	-0.22493
164.929	-0.54757	-0.22493
164.946	-0.54759	-0.22493
164.962	-0.54761	-0.22493
164.98	-0.54763	-0.22493
164.996	-0.54765	-0.22493
165.012	-0.54766	-0.22493
165.028	-0.54768	-0.22494
165.046	-0.5477	-0.22494
165.062	-0.54771	-0.22495

165.08	-0.54773	-0.22495
165.096	-0.54775	-0.22495
165.113	-0.54777	-0.22496
165.129	-0.54778	-0.22496
165.146	-0.5478	-0.22496
165.163	-0.54781	-0.22496
165.179	-0.54783	-0.22496
165.197	-0.54784	-0.22497
165.212	-0.54786	-0.22497
165.229	-0.54787	-0.22497
165.246	-0.54789	-0.22497
165.263	-0.54791	-0.22497
165.279	-0.54793	-0.22498
165.296	-0.54795	-0.22498
165.312	-0.54796	-0.22498
165.328	-0.54798	-0.22498
165.346	-0.54799	-0.22498
165.362	-0.54801	-0.22498
165.38	-0.54802	-0.22498
165.396	-0.54804	-0.22498
165.411	-0.54805	-0.22498
165.429	-0.54806	-0.22498
165.445	-0.54807	-0.22498
165.461	-0.54809	-0.22497
165.478	-0.5481	-0.22497
165.496	-0.54812	-0.22498
165.512	-0.54813	-0.22498
165.529	-0.54815	-0.22498
165.545	-0.54816	-0.22499
165.562	-0.54818	-0.22499

165.579	-0.54819	-0.225
165.596	-0.54821	-0.225
165.612	-0.54822	-0.22501
165.629	-0.54823	-0.22501
165.646	-0.54824	-0.22501
165.662	-0.54824	-0.225
165.679	-0.54825	-0.225
165.695	-0.54826	-0.225
165.711	-0.54828	-0.22499
165.729	-0.54829	-0.22499
165.745	-0.54831	-0.22499
165.763	-0.54833	-0.22499
165.778	-0.54835	-0.225
165.795	-0.54836	-0.225
165.811	-0.54838	-0.22501
165.827	-0.54839	-0.22501
165.845	-0.54841	-0.22501
165.861	-0.54842	-0.22501
165.879	-0.54844	-0.22501
165.896	-0.54845	-0.22501
165.912	-0.54847	-0.225
165.928	-0.54848	-0.225
165.944	-0.5485	-0.22499
165.961	-0.54852	-0.22499
165.978	-0.54853	-0.225
165.994	-0.54855	-0.225
166.011	-0.54856	-0.22501
166.028	-0.54858	-0.22501
166.045	-0.54861	-0.22502
166.061	-0.54863	-0.22503

166.079	-0.54865	-0.22503
166.095	-0.54867	-0.22504
166.112	-0.54868	-0.22504
166.129	-0.5487	-0.22504
166.145	-0.54872	-0.22504
166.162	-0.54874	-0.22504
166.178	-0.54875	-0.22505
166.196	-0.54877	-0.22505
166.212	-0.54879	-0.22505
166.229	-0.54881	-0.22505
166.245	-0.54883	-0.22505
166.262	-0.54886	-0.22506
166.278	-0.54888	-0.22506
166.295	-0.54891	-0.22507
166.312	-0.54893	-0.22507
166.328	-0.54895	-0.22508
166.345	-0.54897	-0.22508
166.363	-0.54899	-0.22509
166.379	-0.549	-0.22509
166.395	-0.54902	-0.22509
166.411	-0.54904	-0.2251
166.429	-0.54906	-0.2251
166.445	-0.54908	-0.2251
166.462	-0.5491	-0.2251
166.478	-0.54912	-0.22511
166.496	-0.54914	-0.22511
166.512	-0.54916	-0.22512
166.529	-0.54918	-0.22512
166.546	-0.5492	-0.22512
166.562	-0.54922	-0.22512

166.579	-0.54924	-0.22512
166.595	-0.54927	-0.22512
166.612	-0.54929	-0.22513
166.629	-0.54931	-0.22513
166.645	-0.54933	-0.22513
166.662	-0.54936	-0.22514
166.679	-0.54938	-0.22514
166.696	-0.5494	-0.22515
166.712	-0.54943	-0.22516
166.729	-0.54945	-0.22516
166.746	-0.54947	-0.22517
166.763	-0.54948	-0.22517
166.779	-0.5495	-0.22517
166.795	-0.54952	-0.22518
166.812	-0.54954	-0.22518
166.83	-0.54955	-0.22518
166.846	-0.54957	-0.22518
166.862	-0.54959	-0.22519
166.879	-0.54961	-0.22519
166.897	-0.54964	-0.2252
166.912	-0.54966	-0.2252
166.929	-0.54968	-0.2252
166.946	-0.54971	-0.22521
166.962	-0.54973	-0.22521
166.98	-0.54975	-0.22521
166.997	-0.54977	-0.22521
167.013	-0.54979	-0.22521
167.03	-0.54981	-0.22522
167.046	-0.54983	-0.22522
167.063	-0.54985	-0.22522

167.08	-0.54986	-0.22522
167.096	-0.54988	-0.22523
167.113	-0.54989	-0.22524
167.129	-0.54991	-0.22524
167.146	-0.54992	-0.22525
167.163	-0.54994	-0.22525
167.18	-0.54995	-0.22525
167.197	-0.54997	-0.22526
167.212	-0.54998	-0.22526
167.23	-0.55 -0.22527	
167.246	-0.55001	-0.22527
167.263	-0.55002	-0.22527
167.28	-0.55004	-0.22527
167.296	-0.55005	-0.22527
167.312	-0.55006	-0.22527
167.33	-0.55007	-0.22526
167.345	-0.55008	-0.22526
167.363	-0.55009	-0.22526
167.38	-0.55011	-0.22526
167.396	-0.55012	-0.22526
167.413	-0.55014	-0.22527
167.43	-0.55015	-0.22527
167.447	-0.55017	-0.22527
167.462	-0.55018	-0.22527
167.48	-0.55019	-0.22527
167.497	-0.5502	-0.22527
167.514	-0.55021	-0.22527
167.53	-0.55022	-0.22527
167.546	-0.55023	-0.22528
167.562	-0.55024	-0.22528

167.58	-0.55026	-0.22527
167.596	-0.55027	-0.22527
167.613	-0.55029	-0.22528
167.629	-0.55031	-0.22528
167.646	-0.55032	-0.22529
167.662	-0.55034	-0.22529
167.68	-0.55035	-0.2253
167.697	-0.55036	-0.2253
167.713	-0.55037	-0.22531
167.73	-0.55038	-0.22531
167.747	-0.55039	-0.22531
167.763	-0.5504	-0.22531
167.779	-0.55041	-0.22531
167.797	-0.55042	-0.2253
167.814	-0.55043	-0.2253
167.83	-0.55045	-0.2253
167.847	-0.55046	-0.2253
167.863	-0.55048	-0.2253
167.88	-0.55049	-0.22531
167.896	-0.55051	-0.22531
167.913	-0.55052	-0.22531
167.93	-0.55054	-0.22531
167.945	-0.55055	-0.22531
167.963	-0.55057	-0.22532
167.98	-0.55058	-0.22532
167.996	-0.5506	-0.22532
168.013	-0.55062	-0.22532
168.029	-0.55064	-0.22532
168.046	-0.55066	-0.22532
168.063	-0.55068	-0.22532

168.079	-0.5507	-0.22532
168.096	-0.55071	-0.22532
168.113	-0.55073	-0.22532
168.129	-0.55074	-0.22532
168.147	-0.55075	-0.22532
168.163	-0.55076	-0.22532
168.179	-0.55077	-0.22533
168.196	-0.55078	-0.22533
168.213	-0.55079	-0.22533
168.23	-0.5508	-0.22534
168.247	-0.55081	-0.22534
168.263	-0.55083	-0.22534
168.279	-0.55084	-0.22534
168.296	-0.55086	-0.22535
168.313	-0.55087	-0.22535
168.329	-0.55089	-0.22535
168.346	-0.5509	-0.22535
168.363	-0.55092	-0.22535
168.38	-0.55094	-0.22535
168.396	-0.55096	-0.22535
168.413	-0.55097	-0.22535
168.43	-0.55099	-0.22535
168.446	-0.551	-0.22535
168.463	-0.55102	-0.22535
168.479	-0.55103	-0.22535
168.497	-0.55105	-0.22536
168.513	-0.55107	-0.22536
168.53	-0.55108	-0.22536
168.546	-0.5511	-0.22536
168.563	-0.55112	-0.22536

168.58	-0.55113	-0.22536
168.596	-0.55115	-0.22536
168.613	-0.55117	-0.22536
168.63	-0.55119	-0.22536
168.647	-0.55121	-0.22537
168.664	-0.55123	-0.22538
168.679	-0.55125	-0.22538
168.696	-0.55127	-0.22539
168.713	-0.55129	-0.22539
168.73	-0.55131	-0.2254
168.746	-0.55132	-0.2254
168.763	-0.55134	-0.2254
168.779	-0.55136	-0.2254
168.795	-0.55137	-0.2254
168.813	-0.55138	-0.2254
168.83	-0.5514	-0.2254
168.847	-0.55142	-0.22541
168.863	-0.55144	-0.22541
168.88	-0.55146	-0.22541
168.897	-0.55148	-0.22541
168.913	-0.5515	-0.22541
168.93	-0.55152	-0.22541
168.947	-0.55153	-0.22542
168.963	-0.55155	-0.22542
168.98	-0.55157	-0.22543
168.997	-0.55158	-0.22544
169.014	-0.55159	-0.22544
169.031	-0.55161	-0.22545
169.046	-0.55162	-0.22545
169.064	-0.55164	-0.22545

169.08	-0.55166	-0.22545
169.098	-0.55168	-0.22545
169.113	-0.55169	-0.22545
169.13	-0.55171	-0.22545
169.147	-0.55173	-0.22545
169.163	-0.55175	-0.22545
169.179	-0.55176	-0.22545
169.197	-0.55178	-0.22546
169.214	-0.5518	-0.22546
169.23	-0.55181	-0.22547
169.246	-0.55183	-0.22547
169.263	-0.55185	-0.22547
169.28	-0.55186	-0.22547
169.297	-0.55188	-0.22547
169.313	-0.55189	-0.22547
169.33	-0.5519	-0.22548
169.346	-0.55192	-0.22548
169.364	-0.55193	-0.22548
169.381	-0.55195	-0.22548
169.397	-0.55196	-0.22548
169.413	-0.55198	-0.22548
169.43	-0.552	-0.22548
169.446	-0.55201	-0.22548
169.463	-0.55203	-0.22548
169.48	-0.55205	-0.22548
169.496	-0.55206	-0.22548
169.512	-0.55208	-0.22549
169.53	-0.55209	-0.22549
169.547	-0.55211	-0.22549
169.564	-0.55213	-0.22549

169.58	-0.55215	-0.2255
169.597	-0.55217	-0.2255
169.613	-0.55218	-0.2255
169.63	-0.5522	-0.2255
169.646	-0.55222	-0.2255
169.663	-0.55223	-0.2255
169.679	-0.55225	-0.22549
169.696	-0.55227	-0.22549
169.713	-0.55228	-0.22549
169.73	-0.5523	-0.22549
169.747	-0.55232	-0.22549
169.763	-0.55234	-0.22549
169.781	-0.55236	-0.2255
169.797	-0.55237	-0.2255
169.813	-0.55239	-0.2255
169.83	-0.55241	-0.22551
169.847	-0.55243	-0.22551
169.864	-0.55244	-0.22551
169.88	-0.55246	-0.22551
169.896	-0.55247	-0.22551
169.913	-0.55249	-0.22551
169.93	-0.5525	-0.22551
169.947	-0.55251	-0.22551
169.963	-0.55252	-0.22551
169.98	-0.55253	-0.22552
169.997	-0.55255	-0.22552
170.013	-0.55257	-0.22551
170.03	-0.55258	-0.22551
170.046	-0.5526	-0.22551
170.064	-0.55262	-0.22551

170.08	-0.55264	-0.22551
170.096	-0.55266	-0.22551
170.111	-0.55267	-0.22551
170.129	-0.55269	-0.22551
170.146	-0.55271	-0.22551
170.163	-0.55272	-0.22552
170.18	-0.55274	-0.22552
170.197	-0.55275	-0.22553
170.213	-0.55276	-0.22553
170.23	-0.55278	-0.22554
170.246	-0.55279	-0.22554
170.263	-0.55281	-0.22554
170.28	-0.55283	-0.22554
170.296	-0.55285	-0.22554
170.314	-0.55288	-0.22554
170.33	-0.5529	-0.22554
170.346	-0.55292	-0.22554
170.363	-0.55293	-0.22555
170.379	-0.55294	-0.22555
170.396	-0.55296	-0.22555
170.412	-0.55297	-0.22555
170.43	-0.55299	-0.22556
170.446	-0.55301	-0.22556
170.463	-0.55303	-0.22556
170.48	-0.55306	-0.22556
170.497	-0.55308	-0.22556
170.513	-0.5531	-0.22556
170.529	-0.55313	-0.22557
170.546	-0.55315	-0.22557
170.563	-0.55317	-0.22557

170.58	-0.55319	-0.22557
170.597	-0.5532	-0.22557
170.613	-0.55322	-0.22558
170.63	-0.55323	-0.22558
170.647	-0.55324	-0.22558
170.663	-0.55326	-0.22559
170.68	-0.55327	-0.22559
170.697	-0.55329	-0.22559
170.713	-0.55331	-0.22559
170.73	-0.55333	-0.2256
170.746	-0.55334	-0.2256
170.763	-0.55337	-0.2256
170.779	-0.55338	-0.2256
170.797	-0.5534	-0.22561
170.814	-0.55342	-0.22561
170.83	-0.55344	-0.22561
170.847	-0.55346	-0.22561
170.863	-0.55348	-0.22561
170.879	-0.5535	-0.22562
170.896	-0.55352	-0.22562
170.914	-0.55355	-0.22562
170.93	-0.55357	-0.22562
170.947	-0.5536	-0.22563
170.963	-0.55363	-0.22563
170.981	-0.55365	-0.22563
170.997	-0.55367	-0.22563
171.013	-0.5537	-0.22563
171.03	-0.55372	-0.22563
171.047	-0.55374	-0.22563
171.064	-0.55376	-0.22563

171.08	-0.55377	-0.22563
171.097	-0.55379	-0.22563
171.113	-0.55381	-0.22563
171.13	-0.55382	-0.22564
171.147	-0.55384	-0.22564
171.163	-0.55386	-0.22564
171.18	-0.55388	-0.22564
171.196	-0.5539	-0.22564
171.213	-0.55393	-0.22565
171.23	-0.55395	-0.22565
171.246	-0.55397	-0.22565
171.264	-0.55399	-0.22566
171.28	-0.55401	-0.22566
171.297	-0.55403	-0.22566
171.314	-0.55405	-0.22566
171.329	-0.55408	-0.22567
171.347	-0.5541	-0.22567
171.364	-0.55412	-0.22567
171.38	-0.55414	-0.22567
171.397	-0.55416	-0.22567
171.413	-0.55418	-0.22567
171.43	-0.55421	-0.22568
171.447	-0.55423	-0.22568
171.463	-0.55425	-0.22568
171.48	-0.55427	-0.22568
171.497	-0.55429	-0.22568
171.513	-0.55431	-0.22568
171.531	-0.55433	-0.22568
171.546	-0.55435	-0.22568
171.563	-0.55437	-0.22568

171.58	-0.55439	-0.22568
171.597	-0.55441	-0.22568
171.614	-0.55443	-0.22568
171.63	-0.55444	-0.22568
171.647	-0.55446	-0.22568
171.663	-0.55448	-0.22568
171.68	-0.55449	-0.22569
171.697	-0.5545	-0.22569
171.713	-0.55452	-0.2257
171.73	-0.55453	-0.2257
171.747	-0.55454	-0.2257
171.763	-0.55455	-0.22571
171.781	-0.55457	-0.22571
171.798	-0.55458	-0.22572
171.813	-0.5546	-0.22572
171.831	-0.55462	-0.22572
171.847	-0.55464	-0.22572
171.864	-0.55466	-0.22572
171.88	-0.55468	-0.22572
171.897	-0.55469	-0.22572
171.913	-0.5547	-0.22573
171.931	-0.55472	-0.22573
171.946	-0.55473	-0.22572
171.964	-0.55475	-0.22572
171.981	-0.55476	-0.22573
171.997	-0.55478	-0.22573
172.014	-0.55479	-0.22573
172.031	-0.55481	-0.22573
172.047	-0.55483	-0.22574
172.064	-0.55485	-0.22574

172.081	-0.55487	-0.22574
172.097	-0.55488	-0.22574
172.114	-0.5549	-0.22574
172.13	-0.55491	-0.22574
172.147	-0.55493	-0.22574
172.164	-0.55494	-0.22574
172.18	-0.55495	-0.22574
172.197	-0.55497	-0.22574
172.213	-0.55499	-0.22574
172.23	-0.555	-0.22574
172.247	-0.55502	-0.22574
172.264	-0.55503	-0.22574
172.28	-0.55505	-0.22574
172.296	-0.55506	-0.22574
172.313	-0.55508	-0.22574
172.33	-0.55509	-0.22574
172.347	-0.55511	-0.22574
172.363	-0.55513	-0.22574
172.38	-0.55515	-0.22574
172.397	-0.55516	-0.22575
172.413	-0.55518	-0.22575
172.43	-0.5552	-0.22575
172.447	-0.55522	-0.22575
172.463	-0.55523	-0.22575
172.48	-0.55525	-0.22575
172.496	-0.55527	-0.22575
172.513	-0.55528	-0.22575
172.53	-0.5553	-0.22574
172.546	-0.55532	-0.22574
172.564	-0.55533	-0.22574

172.58	-0.55535	-0.22574
172.597	-0.55537	-0.22575
172.613	-0.55538	-0.22575
172.63	-0.55541	-0.22575
172.646	-0.55543	-0.22575
172.663	-0.55545	-0.22576
172.68	-0.55547	-0.22576
172.697	-0.55549	-0.22576
172.714	-0.55551	-0.22576
172.731	-0.55552	-0.22576
172.747	-0.55554	-0.22577
172.764	-0.55556	-0.22577
172.78	-0.55557	-0.22577
172.797	-0.55559	-0.22577
172.814	-0.55561	-0.22577
172.831	-0.55563	-0.22578
172.848	-0.55565	-0.22578
172.864	-0.55567	-0.22579
172.881	-0.5557	-0.22579
172.898	-0.55572	-0.2258
172.914	-0.55574	-0.2258
172.931	-0.55576	-0.22581
172.947	-0.55578	-0.22581
172.964	-0.5558	-0.22582
172.98	-0.55583	-0.22582
172.997	-0.55585	-0.22583
173.014	-0.55587	-0.22583
173.031	-0.55588	-0.22583
173.047	-0.55591	-0.22584
173.064	-0.55593	-0.22584

173.081	-0.55595	-0.22585
173.097	-0.55597	-0.22585
173.115	-0.55599	-0.22586
173.132	-0.55602	-0.22586
173.148	-0.55605	-0.22586
173.165	-0.55608	-0.22587
173.181	-0.55611	-0.22587
173.198	-0.55614	-0.22587
173.214	-0.55616	-0.22587
173.231	-0.55618	-0.22587
173.248	-0.55621	-0.22588
173.265	-0.55624	-0.22588
173.282	-0.55626	-0.22588
173.298	-0.55629	-0.22589
173.315	-0.55632	-0.2259
173.331	-0.55634	-0.22591
173.348	-0.55637	-0.22592
173.364	-0.5564	-0.22592
173.38	-0.55642	-0.22593
173.398	-0.55644	-0.22594
173.415	-0.55647	-0.22595
173.431	-0.55649	-0.22595
173.448	-0.55652	-0.22596
173.464	-0.55654	-0.22597
173.482	-0.55656	-0.22597
173.498	-0.55659	-0.22598
173.515	-0.5566	-0.22599
173.531	-0.55662	-0.22599
173.548	-0.55664	-0.226
173.565	-0.55667	-0.226

173.582	-0.55669	-0.22601
173.598	-0.55671	-0.22601
173.615	-0.55674	-0.22601
173.631	-0.55676	-0.22601
173.649	-0.55679	-0.22601
173.665	-0.55682	-0.22602
173.682	-0.55685	-0.22602
173.699	-0.55687	-0.22602
173.715	-0.55689	-0.22603
173.731	-0.55691	-0.22603
173.749	-0.55693	-0.22604
173.765	-0.55695	-0.22604
173.781	-0.55697	-0.22604
173.799	-0.55699	-0.22605
173.815	-0.55701	-0.22605
173.832	-0.55703	-0.22606
173.849	-0.55706	-0.22606
173.865	-0.55708	-0.22606
173.882	-0.5571	-0.22606
173.898	-0.55712	-0.22606
173.915	-0.55713	-0.22606
173.932	-0.55714	-0.22606
173.949	-0.55716	-0.22606
173.965	-0.55718	-0.22606
173.981	-0.5572	-0.22606
173.998	-0.55722	-0.22606
174.014	-0.55725	-0.22606
174.032	-0.55727	-0.22606
174.048	-0.5573	-0.22607
174.065	-0.55732	-0.22607

174.082	-0.55734	-0.22607
174.098	-0.55737	-0.22607
174.115	-0.55739	-0.22607
174.131	-0.5574	-0.22607
174.15	-0.55742	-0.22607
174.166	-0.55744	-0.22608
174.182	-0.55746	-0.22608
174.199	-0.55748	-0.22608
174.215	-0.55749	-0.22608
174.232	-0.55751	-0.22609
174.249	-0.55753	-0.22609
174.266	-0.55754	-0.22609
174.283	-0.55756	-0.22609
174.299	-0.55758	-0.22609
174.315	-0.5576	-0.22608
174.332	-0.55761	-0.22608
174.349	-0.55763	-0.22608
174.366	-0.55765	-0.22607
174.382	-0.55767	-0.22607
174.398	-0.5577	-0.22607
174.415	-0.55772	-0.22606
174.432	-0.55774	-0.22606
174.449	-0.55776	-0.22607
174.465	-0.55778	-0.22607
174.482	-0.5578	-0.22607
174.499	-0.55781	-0.22607
174.514	-0.55783	-0.22607
174.531	-0.55785	-0.22607
174.549	-0.55787	-0.22608
174.565	-0.55789	-0.22608

174.582	-0.5579	-0.22608
174.598	-0.55792	-0.22608
174.616	-0.55794	-0.22608
174.632	-0.55796	-0.22608
174.649	-0.55798	-0.22608
174.665	-0.558	-0.22608
174.682	-0.55801	-0.22608
174.698	-0.55803	-0.22607
174.715	-0.55805	-0.22607
174.732	-0.55807	-0.22607
174.748	-0.55808	-0.22607
174.764	-0.55811	-0.22607
174.781	-0.55813	-0.22608
174.798	-0.55815	-0.22608
174.815	-0.55817	-0.22608
174.833	-0.55819	-0.22608
174.849	-0.55821	-0.22608
174.866	-0.55822	-0.22608
174.882	-0.55824	-0.22608
174.898	-0.55825	-0.22608
174.916	-0.55827	-0.22608
174.931	-0.55829	-0.22608
174.948	-0.5583	-0.22608
174.964	-0.55831	-0.22608
174.982	-0.55833	-0.22608
174.998	-0.55835	-0.22609
175.015	-0.55837	-0.22609
175.031	-0.55839	-0.22609
175.048	-0.55841	-0.22609
175.065	-0.55843	-0.22609

175.081	-0.55845	-0.22609
175.099	-0.55848	-0.22609
175.115	-0.5585	-0.22608
175.131	-0.55852	-0.22608
175.148	-0.55853	-0.22608
175.165	-0.55855	-0.22609
175.182	-0.55857	-0.22609
175.198	-0.55859	-0.22609
175.215	-0.55861	-0.2261
175.231	-0.55863	-0.2261
175.248	-0.55864	-0.22611
175.265	-0.55866	-0.22611
175.282	-0.55868	-0.22611
175.298	-0.5587	-0.2261
175.315	-0.55872	-0.2261
175.332	-0.55874	-0.2261
175.348	-0.55876	-0.2261
175.364	-0.55878	-0.2261
175.381	-0.5588	-0.2261
175.398	-0.55882	-0.22611
175.414	-0.55884	-0.22611
175.431	-0.55885	-0.22611
175.447	-0.55887	-0.22611
175.465	-0.55888	-0.22611
175.482	-0.5589	-0.22611
175.498	-0.55891	-0.22612
175.515	-0.55893	-0.22612
175.532	-0.55895	-0.22612
175.549	-0.55896	-0.22612
175.566	-0.55898	-0.22612

175.581	-0.55899	-0.22612
175.599	-0.55901	-0.22612
175.615	-0.55902	-0.22613
175.632	-0.55903	-0.22613
175.649	-0.55905	-0.22613
175.665	-0.55907	-0.22613
175.681	-0.55908	-0.22613
175.698	-0.5591	-0.22614
175.714	-0.55912	-0.22614
175.731	-0.55914	-0.22614
175.748	-0.55916	-0.22614
175.764	-0.55918	-0.22614
175.781	-0.55919	-0.22615
175.798	-0.55921	-0.22615
175.814	-0.55922	-0.22614
175.831	-0.55923	-0.22614
175.847	-0.55925	-0.22614
175.864	-0.55926	-0.22614
175.881	-0.55928	-0.22614
175.897	-0.55929	-0.22615
175.914	-0.55931	-0.22615
175.931	-0.55932	-0.22616
175.948	-0.55934	-0.22616
175.965	-0.55936	-0.22616
175.981	-0.55938	-0.22616
175.998	-0.5594	-0.22617
176.014	-0.55941	-0.22617
176.032	-0.55943	-0.22617
176.048	-0.55945	-0.22617
176.065	-0.55947	-0.22618

176.081	-0.55949	-0.22618
176.098	-0.55952	-0.22618
176.115	-0.55954	-0.22618
176.131	-0.55956	-0.22619
176.147	-0.55958	-0.22619
176.164	-0.55961	-0.22619
176.181	-0.55963	-0.22619
176.198	-0.55965	-0.22619
176.214	-0.55967	-0.22619
176.231	-0.55969	-0.22619
176.248	-0.55971	-0.2262
176.265	-0.55972	-0.2262
176.281	-0.55974	-0.2262
176.298	-0.55976	-0.22621
176.315	-0.55978	-0.22621
176.332	-0.55981	-0.22621
176.348	-0.55983	-0.22621
176.365	-0.55985	-0.22622
176.381	-0.55988	-0.22622
176.399	-0.5599	-0.22622
176.415	-0.55993	-0.22623
176.432	-0.55995	-0.22623
176.447	-0.55998	-0.22624
176.465	-0.56	-0.22624
176.481	-0.56003	-0.22625
176.498	-0.56005	-0.22626
176.514	-0.56008	-0.22627
176.531	-0.5601	-0.22627
176.548	-0.56013	-0.22627
176.566	-0.56016	-0.22628

176.582	-0.56019	-0.22629
176.599	-0.56021	-0.22629
176.615	-0.56024	-0.2263
176.632	-0.56026	-0.2263
176.648	-0.56029	-0.2263
176.665	-0.56032	-0.22631
176.681	-0.56034	-0.22631
176.699	-0.56037	-0.22631
176.715	-0.56039	-0.22631
176.732	-0.56042	-0.22631
176.748	-0.56044	-0.22632
176.766	-0.56046	-0.22632
176.781	-0.56049	-0.22632
176.799	-0.56051	-0.22632
176.815	-0.56053	-0.22633
176.832	-0.56056	-0.22633
176.849	-0.56058	-0.22634
176.866	-0.56061	-0.22634
176.881	-0.56063	-0.22634
176.898	-0.56066	-0.22635
176.915	-0.56069	-0.22635
176.932	-0.56072	-0.22635
176.949	-0.56075	-0.22635
176.965	-0.56077	-0.22635
176.984	-0.5608	-0.22635
177	-0.56082	-0.22636
177.016	-0.56084	-0.22636
177.033	-0.56085	-0.22637
177.049	-0.56087	-0.22637
177.066	-0.56089	-0.22637

177.083	-0.5609	-0.22638
177.099	-0.56092	-0.22638
177.116	-0.56094	-0.22639
177.132	-0.56095	-0.22639
177.15	-0.56097	-0.22639
177.166	-0.56099	-0.2264
177.184	-0.56101	-0.2264
177.199	-0.56102	-0.2264
177.216	-0.56104	-0.2264
177.233	-0.56105	-0.2264
177.249	-0.56106	-0.2264
177.266	-0.56107	-0.2264
177.282	-0.56109	-0.2264
177.299	-0.5611	-0.2264
177.316	-0.56112	-0.2264
177.333	-0.56113	-0.2264
177.35	-0.56115	-0.22641
177.366	-0.56116	-0.22641
177.382	-0.56118	-0.22641
177.399	-0.56119	-0.22641
177.416	-0.5612	-0.22642
177.432	-0.56122	-0.22642
177.45	-0.56124	-0.22643
177.467	-0.56125	-0.22643
177.482	-0.56126	-0.22643
177.5	-0.56128	-0.22643
177.517	-0.5613	-0.22643
177.533	-0.56131	-0.22643
177.549	-0.56132	-0.22643
177.566	-0.56133	-0.22643

177.583	-0.56134	-0.22643
177.599	-0.56134	-0.22643
177.616	-0.56134	-0.22643
177.632	-0.56135	-0.22644
177.649	-0.56135	-0.22644
177.666	-0.56136	-0.22644
177.683	-0.56137	-0.22644
177.699	-0.56138	-0.22644
177.715	-0.56139	-0.22644
177.733	-0.56139	-0.22644
177.749	-0.5614	-0.22645
177.766	-0.56141	-0.22644
177.783	-0.56142	-0.22644
177.799	-0.56143	-0.22644
177.816	-0.56144	-0.22644
177.832	-0.56144	-0.22644
177.85	-0.56145	-0.22644
177.866	-0.56146	-0.22644
177.882	-0.56147	-0.22645
177.899	-0.56147	-0.22645
177.916	-0.56148	-0.22645
177.933	-0.56149	-0.22646
177.949	-0.56149	-0.22647
177.966	-0.5615	-0.22647
177.983	-0.5615	-0.22647
177.999	-0.56151	-0.22647
178.016	-0.56151	-0.22647
178.032	-0.56152	-0.22647
178.049	-0.56153	-0.22647
178.067	-0.56154	-0.22647

178.082	-0.56155	-0.22647
178.1	-0.56155	-0.22648
178.116	-0.56156	-0.22648
178.133	-0.56157	-0.22648
178.149	-0.56158	-0.22648
178.166	-0.56158	-0.22648
178.182	-0.56159	-0.22648
178.199	-0.5616	-0.22649
178.216	-0.56161	-0.22649
178.232	-0.56162	-0.22649
178.249	-0.56163	-0.22649
178.266	-0.56164	-0.22649
178.283	-0.56165	-0.2265
178.299	-0.56166	-0.2265
178.316	-0.56166	-0.2265
178.332	-0.56167	-0.2265
178.349	-0.56167	-0.2265
178.366	-0.56168	-0.22651
178.383	-0.56168	-0.22651
178.399	-0.56169	-0.22651
178.416	-0.56169	-0.22652
178.432	-0.5617	-0.22652
178.448	-0.56171	-0.22652
178.465	-0.56172	-0.22652
178.482	-0.56173	-0.22652
178.5	-0.56174	-0.22653
178.516	-0.56176	-0.22653
178.532	-0.56177	-0.22653
178.549	-0.56178	-0.22654
178.566	-0.56179	-0.22654

178.582	-0.5618	-0.22655
178.6	-0.5618	-0.22655
178.615	-0.56181	-0.22655
178.633	-0.56181	-0.22656
178.649	-0.56182	-0.22655
178.666	-0.56183	-0.22655
178.682	-0.56184	-0.22655
178.7	-0.56185	-0.22655
178.716	-0.56187	-0.22655
178.733	-0.56188	-0.22655
178.75	-0.56189	-0.22656
178.766	-0.5619	-0.22656
178.783	-0.56191	-0.22656
178.8	-0.56192	-0.22657
178.816	-0.56193	-0.22657
178.833	-0.56193	-0.22658
178.849	-0.56194	-0.22658
178.866	-0.56194	-0.22658
178.883	-0.56195	-0.22658
178.9	-0.56195	-0.22658
178.916	-0.56196	-0.22658
178.933	-0.56197	-0.22658
178.949	-0.56198	-0.22658
178.967	-0.56199	-0.22659
178.983	-0.56201	-0.22659
178.999	-0.56202	-0.22659
179.015	-0.56203	-0.2266
179.032	-0.56204	-0.2266
179.049	-0.56205	-0.2266
179.066	-0.56205	-0.2266

179.083	-0.56205	-0.2266
179.1	-0.56205	-0.2266
179.117	-0.56205	-0.2266
179.133	-0.56205	-0.2266
179.149	-0.56205	-0.2266
179.166	-0.56206	-0.2266
179.182	-0.56206	-0.2266
179.199	-0.56207	-0.2266
179.216	-0.56208	-0.2266
179.232	-0.56209	-0.2266
179.249	-0.5621	-0.22661
179.265	-0.56211	-0.22661
179.282	-0.56212	-0.22661
179.298	-0.56213	-0.22661
179.315	-0.56213	-0.22661
179.332	-0.56214	-0.22661
179.349	-0.56214	-0.22661
179.365	-0.56215	-0.22661
179.382	-0.56215	-0.22661
179.399	-0.56215	-0.22662
179.416	-0.56215	-0.22662
179.432	-0.56215	-0.22662
179.448	-0.56215	-0.22662
179.466	-0.56215	-0.22662
179.482	-0.56216	-0.22662
179.499	-0.56217	-0.22662
179.516	-0.56217	-0.22662
179.532	-0.56218	-0.22662
179.549	-0.56219	-0.22662
179.565	-0.5622	-0.22662

179.582	-0.56221	-0.22662
179.598	-0.56222	-0.22662
179.616	-0.56223	-0.22662
179.632	-0.56224	-0.22663
179.649	-0.56225	-0.22663
179.666	-0.56226	-0.22663
179.682	-0.56227	-0.22664
179.698	-0.56227	-0.22664
179.715	-0.56228	-0.22665
179.731	-0.56229	-0.22665
179.749	-0.56229	-0.22665
179.765	-0.5623	-0.22665
179.783	-0.56231	-0.22665
179.798	-0.56231	-0.22666
179.815	-0.56232	-0.22666
179.832	-0.56233	-0.22666
179.848	-0.56234	-0.22666
179.865	-0.56235	-0.22667
179.881	-0.56236	-0.22667
179.899	-0.56237	-0.22668
179.916	-0.56238	-0.22668
179.932	-0.56239	-0.22668
179.949	-0.5624	-0.22668
179.965	-0.56241	-0.22669
179.982	-0.56242	-0.22669
180	-0.56244	-0.22669
180.016	-0.56245	-0.2267
180.033	-0.56246	-0.2267
180.05	-0.56247	-0.2267
180.065	-0.56248	-0.22671

180.082	-0.56249	-0.22671
180.099	-0.5625	-0.22672
180.116	-0.5625	-0.22672
180.132	-0.56251	-0.22673
180.149	-0.56252	-0.22674
180.166	-0.56253	-0.22674
180.183	-0.56254	-0.22675
180.199	-0.56255	-0.22676
180.216	-0.56256	-0.22676
180.232	-0.56258	-0.22677
180.248	-0.56259	-0.22678
180.266	-0.5626	-0.22678
180.282	-0.56261	-0.22679
180.299	-0.56261	-0.2268
180.315	-0.56262	-0.2268
180.333	-0.56263	-0.2268
180.349	-0.56264	-0.2268
180.366	-0.56266	-0.2268
180.383	-0.56267	-0.2268
180.399	-0.56268	-0.22681
180.416	-0.5627	-0.22681
180.433	-0.56271	-0.22681
180.449	-0.56272	-0.22682
180.466	-0.56273	-0.22683
180.483	-0.56274	-0.22683
180.5	-0.56275	-0.22684
180.517	-0.56276	-0.22685
180.532	-0.56277	-0.22685
180.55	-0.56278	-0.22686
180.566	-0.56278	-0.22686

180.583	-0.56279	-0.22686
180.6	-0.56281	-0.22687
180.616	-0.56282	-0.22687
180.633	-0.56283	-0.22688
180.65	-0.56284	-0.22688
180.667	-0.56285	-0.22688
180.684	-0.56286	-0.22689
180.7	-0.56287	-0.22689
180.716	-0.56288	-0.22689
180.733	-0.56289	-0.22689
180.75	-0.5629	-0.2269
180.766	-0.56291	-0.2269
180.783	-0.56292	-0.2269
180.8	-0.56293	-0.22691
180.816	-0.56294	-0.22691
180.833	-0.56295	-0.22692
180.849	-0.56296	-0.22692
180.867	-0.56296	-0.22693
180.882	-0.56297	-0.22694
180.9	-0.56298	-0.22694
180.916	-0.56299	-0.22695
180.933	-0.56299	-0.22695
180.949	-0.563	-0.22696
180.967	-0.56301	-0.22696
180.983	-0.56301	-0.22697
180.999	-0.56302	-0.22697
181.017	-0.56302	-0.22697
181.034	-0.56303	-0.22698
181.051	-0.56304	-0.22698
181.068	-0.56305	-0.22698

181.084	-0.56305	-0.22699
181.1	-0.56306	-0.22699
181.117	-0.56306	-0.22699
181.133	-0.56307	-0.22699
181.149	-0.56307	-0.22699
181.166	-0.56307	-0.22699
181.183	-0.56307	-0.22699
181.2	-0.56307	-0.22699
181.216	-0.56308	-0.227
181.233	-0.56308	-0.227
181.25	-0.56309	-0.227
181.266	-0.56309	-0.22701
181.283	-0.56309	-0.22701
181.299	-0.5631	-0.22701
181.316	-0.5631	-0.22701
181.333	-0.5631	-0.22701
181.349	-0.56311	-0.227
181.366	-0.56311	-0.227
181.382	-0.56312	-0.227
181.398	-0.56312	-0.227
181.415	-0.56312	-0.227
181.432	-0.56312	-0.22701
181.45	-0.56312	-0.22701
181.466	-0.56311	-0.22701
181.482	-0.56311	-0.22702
181.499	-0.56311	-0.22702
181.516	-0.56311	-0.22702
181.533	-0.56311	-0.22703
181.55	-0.56311	-0.22703
181.566	-0.56311	-0.22703

181.582	-0.56311	-0.22703
181.599	-0.56311	-0.22703
181.617	-0.56311	-0.22703
181.633	-0.5631	-0.22703
181.65	-0.5631	-0.22703
181.664	-0.5631	-0.22703
181.683	-0.5631	-0.22704
181.698	-0.5631	-0.22704
181.715	-0.56311	-0.22704
181.732	-0.56311	-0.22704
181.748	-0.56312	-0.22705
181.765	-0.56312	-0.22705
181.782	-0.56312	-0.22705
181.799	-0.56313	-0.22705
181.815	-0.56313	-0.22706
181.831	-0.56313	-0.22706
181.848	-0.56314	-0.22706
181.865	-0.56315	-0.22706
181.882	-0.56315	-0.22707
181.9	-0.56316	-0.22707
181.916	-0.56316	-0.22708
181.933	-0.56317	-0.22708
181.949	-0.56317	-0.22709
181.966	-0.56318	-0.22709
181.982	-0.56318	-0.2271
182	-0.56319	-0.2271
182.015	-0.56319	-0.2271
182.032	-0.5632	-0.2271
182.048	-0.56321	-0.22711
182.065	-0.56322	-0.22711

182.082	-0.56322	-0.22712
182.099	-0.56323	-0.22712
182.116	-0.56324	-0.22713
182.132	-0.56324	-0.22713
182.15	-0.56325	-0.22714
182.166	-0.56326	-0.22715
182.182	-0.56326	-0.22715
182.199	-0.56327	-0.22715
182.215	-0.56328	-0.22716
182.233	-0.56329	-0.22716
182.249	-0.56329	-0.22716
182.266	-0.5633	-0.22717
182.283	-0.5633	-0.22717
182.3	-0.56331	-0.22717
182.316	-0.56332	-0.22717
182.333	-0.56332	-0.22718
182.349	-0.56333	-0.22718
182.366	-0.56334	-0.22719
182.383	-0.56336	-0.22719
182.399	-0.56336	-0.2272
182.417	-0.56337	-0.22721
182.433	-0.56337	-0.22721
182.45	-0.56337	-0.22721
182.466	-0.56337	-0.22722
182.484	-0.56337	-0.22723
182.5	-0.56337	-0.22723
182.517	-0.56337	-0.22724
182.534	-0.56338	-0.22724
182.55	-0.56338	-0.22725
182.566	-0.56339	-0.22725

182.584	-0.5634	-0.22726
182.599	-0.56341	-0.22726
182.616	-0.56341	-0.22727
182.632	-0.56342	-0.22727
182.649	-0.56342	-0.22727
182.667	-0.56343	-0.22728
182.683	-0.56344	-0.22728
182.7	-0.56345	-0.22729
182.716	-0.56346	-0.22729
182.732	-0.56347	-0.2273
182.75	-0.56348	-0.2273
182.767	-0.56349	-0.22731
182.783	-0.5635	-0.22731
182.8	-0.56351	-0.22731
182.816	-0.56352	-0.22732
182.832	-0.56353	-0.22732
182.85	-0.56353	-0.22732
182.866	-0.56354	-0.22733
182.883	-0.56354	-0.22733
182.9	-0.56355	-0.22733
182.916	-0.56355	-0.22733
182.933	-0.56356	-0.22734
182.949	-0.56356	-0.22734
182.967	-0.56356	-0.22734
182.982	-0.56357	-0.22734
183	-0.56357	-0.22735
183.016	-0.56358	-0.22735
183.033	-0.56358	-0.22735
183.049	-0.56359	-0.22736
183.067	-0.56359	-0.22736

183.083	-0.5636	-0.22737
183.099	-0.56361	-0.22737
183.117	-0.56361	-0.22737
183.133	-0.56362	-0.22737
183.149	-0.56363	-0.22737
183.166	-0.56363	-0.22738
183.184	-0.56364	-0.22738
183.2	-0.56364	-0.22739
183.216	-0.56364	-0.22739
183.234	-0.56365	-0.2274
183.25	-0.56365	-0.2274
183.267	-0.56366	-0.2274
183.284	-0.56366	-0.2274
183.301	-0.56366	-0.2274
183.317	-0.56367	-0.2274
183.333	-0.56368	-0.2274
183.35	-0.56368	-0.2274
183.366	-0.56369	-0.22741
183.384	-0.5637	-0.22741
183.399	-0.56371	-0.22742
183.416	-0.56372	-0.22743
183.433	-0.56373	-0.22743
183.449	-0.56374	-0.22744
183.466	-0.56375	-0.22745
183.483	-0.56376	-0.22745
183.5	-0.56377	-0.22745
183.517	-0.56378	-0.22745
183.533	-0.56379	-0.22745
183.55	-0.56379	-0.22746
183.567	-0.5638	-0.22746

183.583	-0.56381	-0.22746
183.6	-0.56382	-0.22746
183.617	-0.56382	-0.22746
183.632	-0.56383	-0.22746
183.65	-0.56383	-0.22746
183.667	-0.56384	-0.22747
183.683	-0.56384	-0.22747
183.7	-0.56385	-0.22747
183.717	-0.56385	-0.22747
183.733	-0.56385	-0.22748
183.75	-0.56386	-0.22748
183.766	-0.56386	-0.22749
183.783	-0.56388	-0.22749
183.8	-0.56389	-0.22749
183.816	-0.5639	-0.2275
183.833	-0.5639	-0.2275
183.85	-0.56391	-0.22751
183.867	-0.56392	-0.22751
183.883	-0.56392	-0.22752
183.9	-0.56393	-0.22752
183.917	-0.56394	-0.22753
183.933	-0.56394	-0.22753
183.951	-0.56394	-0.22754
183.967	-0.56395	-0.22754
183.983	-0.56395	-0.22755
184	-0.56396	-0.22755
184.016	-0.56396	-0.22755
184.033	-0.56397	-0.22756
184.049	-0.56398	-0.22756
184.066	-0.56398	-0.22756

184.083	-0.56399	-0.22756
184.1	-0.564	-0.22757
184.117	-0.564	-0.22757
184.133	-0.56401	-0.22758
184.151	-0.56402	-0.22758
184.166	-0.56402	-0.22758
184.183	-0.56403	-0.22758
184.199	-0.56403	-0.22758
184.217	-0.56403	-0.22758
184.233	-0.56404	-0.22759
184.249	-0.56405	-0.22759
184.266	-0.56405	-0.2276
184.282	-0.56406	-0.2276
184.299	-0.56407	-0.22761
184.316	-0.56407	-0.22762
184.332	-0.56407	-0.22762
184.35	-0.56407	-0.22762
184.366	-0.56408	-0.22762
184.383	-0.56408	-0.22762
184.399	-0.56408	-0.22762
184.416	-0.56408	-0.22762
184.433	-0.56408	-0.22763
184.449	-0.56409	-0.22763
184.465	-0.56409	-0.22764
184.483	-0.5641	-0.22765
184.499	-0.5641	-0.22765
184.515	-0.5641	-0.22766
184.532	-0.56411	-0.22766
184.549	-0.56411	-0.22767
184.566	-0.56412	-0.22767

184.583	-0.56412	-0.22768
184.6	-0.56412	-0.22768
184.616	-0.56413	-0.22768
184.632	-0.56413	-0.22768
184.649	-0.56414	-0.22769
184.666	-0.56414	-0.22769
184.683	-0.56415	-0.22769
184.7	-0.56415	-0.22769
184.716	-0.56416	-0.22769
184.733	-0.56417	-0.22769
184.749	-0.56417	-0.2277
184.766	-0.56418	-0.2277
184.782	-0.56419	-0.22769
184.8	-0.56419	-0.22769
184.816	-0.5642	-0.2277
184.833	-0.56421	-0.2277
184.85	-0.56421	-0.22771
184.866	-0.56422	-0.22771
184.883	-0.56423	-0.22771
184.9	-0.56423	-0.22771
184.915	-0.56424	-0.22771
184.933	-0.56424	-0.22772
184.949	-0.56425	-0.22772
184.966	-0.56425	-0.22772
184.983	-0.56426	-0.22772
184.999	-0.56426	-0.22772
185.015	-0.56427	-0.22773
185.032	-0.56427	-0.22773
185.049	-0.56428	-0.22773
185.066	-0.56429	-0.22774

185.083	-0.56429	-0.22774
185.098	-0.5643	-0.22774
185.116	-0.56431	-0.22775
185.133	-0.56432	-0.22775
185.149	-0.56433	-0.22775
185.166	-0.56434	-0.22776
185.182	-0.56436	-0.22776
185.2	-0.56437	-0.22777
185.216	-0.56437	-0.22777
185.233	-0.56438	-0.22778
185.248	-0.56439	-0.22778
185.266	-0.56439	-0.22778
185.282	-0.5644	-0.22779
185.299	-0.56441	-0.22779
185.316	-0.56442	-0.22779
185.332	-0.56443	-0.2278
185.349	-0.56444	-0.22781
185.365	-0.56445	-0.22781
185.383	-0.56447	-0.22782
185.399	-0.56448	-0.22783
185.415	-0.56449	-0.22784
185.432	-0.5645	-0.22785
185.45	-0.56451	-0.22785
185.465	-0.56452	-0.22786
185.481	-0.56453	-0.22786
185.499	-0.56454	-0.22786
185.516	-0.56455	-0.22786
185.533	-0.56456	-0.22787
185.55	-0.56457	-0.22787
185.566	-0.56458	-0.22788

185.583	-0.56459	-0.22788
185.6	-0.5646	-0.22789
185.616	-0.56461	-0.2279
185.633	-0.56462	-0.2279
185.649	-0.56463	-0.22791
185.666	-0.56464	-0.22792
185.682	-0.56466	-0.22792
185.699	-0.56467	-0.22793
185.716	-0.56469	-0.22793
185.732	-0.5647	-0.22794
185.75	-0.56472	-0.22794
185.766	-0.56473	-0.22795
185.783	-0.56475	-0.22795
185.799	-0.56476	-0.22796
185.816	-0.56477	-0.22796
185.833	-0.56478	-0.22796
185.85	-0.56479	-0.22797
185.866	-0.5648	-0.22797
185.883	-0.56481	-0.22798
185.9	-0.56481	-0.22799
185.916	-0.56483	-0.228
185.933	-0.56484	-0.22801
185.95	-0.56485	-0.22802
185.967	-0.56487	-0.22802
185.983	-0.56488	-0.22803
186	-0.56489	-0.22804
186.017	-0.5649	-0.22804
186.034	-0.5649	-0.22805
186.05	-0.56491	-0.22805
186.067	-0.56491	-0.22806

186.084	-0.56491	-0.22807
186.1	-0.56492	-0.22808
186.117	-0.56493	-0.22808
186.133	-0.56494	-0.22809
186.15	-0.56494	-0.2281
186.166	-0.56495	-0.2281
186.184	-0.56496	-0.22811
186.2	-0.56497	-0.22811
186.217	-0.56498	-0.22812
186.234	-0.56499	-0.22812
186.251	-0.565	-0.22812
186.268	-0.56501	-0.22813
186.285	-0.56502	-0.22813
186.3	-0.56503	-0.22813
186.317	-0.56504	-0.22814
186.334	-0.56505	-0.22814
186.351	-0.56505	-0.22815
186.367	-0.56506	-0.22816
186.384	-0.56507	-0.22817
186.4	-0.56507	-0.22817
186.418	-0.56508	-0.22817
186.434	-0.56508	-0.22818
186.451	-0.56509	-0.22818
186.467	-0.56509	-0.22818
186.484	-0.5651	-0.22818
186.5	-0.5651	-0.22818
186.517	-0.5651	-0.22818
186.534	-0.5651	-0.22819
186.551	-0.56511	-0.22819
186.568	-0.56511	-0.2282

186.584	-0.56512	-0.2282
186.601	-0.56512	-0.22821
186.618	-0.56513	-0.22821
186.634	-0.56514	-0.22821
186.651	-0.56514	-0.22821
186.666	-0.56515	-0.22821
186.684	-0.56515	-0.22821
186.699	-0.56516	-0.22821
186.717	-0.56517	-0.22821
186.734	-0.56517	-0.22821
186.75	-0.56518	-0.22821
186.767	-0.56518	-0.22822
186.784	-0.56519	-0.22822
186.8	-0.5652	-0.22822
186.817	-0.5652	-0.22822
186.833	-0.56521	-0.22823
186.85	-0.56522	-0.22823
186.867	-0.56522	-0.22823
186.884	-0.56523	-0.22823
186.901	-0.56523	-0.22824
186.917	-0.56524	-0.22824
186.934	-0.56524	-0.22825
186.951	-0.56525	-0.22825
186.967	-0.56525	-0.22826
186.983	-0.56526	-0.22826
187	-0.56527	-0.22826
187.016	-0.56528	-0.22826
187.033	-0.56528	-0.22826
187.05	-0.56529	-0.22827
187.066	-0.56529	-0.22827

187.084	-0.5653	-0.22828
187.1	-0.56531	-0.22828
187.117	-0.56531	-0.22829
187.133	-0.56532	-0.22829
187.15	-0.56532	-0.2283
187.167	-0.56532	-0.2283
187.183	-0.56532	-0.22831
187.2	-0.56533	-0.22831
187.216	-0.56534	-0.22832
187.233	-0.56534	-0.22832
187.25	-0.56535	-0.22832
187.266	-0.56536	-0.22832
187.283	-0.56537	-0.22833
187.3	-0.56538	-0.22833
187.316	-0.56539	-0.22833
187.334	-0.5654	-0.22834
187.349	-0.56541	-0.22834
187.366	-0.56542	-0.22835
187.384	-0.56543	-0.22835
187.4	-0.56545	-0.22835
187.417	-0.56546	-0.22835
187.433	-0.56547	-0.22835
187.45	-0.56549	-0.22835
187.466	-0.5655	-0.22835
187.483	-0.56551	-0.22835
187.5	-0.56552	-0.22835
187.516	-0.56553	-0.22835
187.532	-0.56555	-0.22836
187.55	-0.56556	-0.22836
187.566	-0.56557	-0.22836

187.583	-0.56558	-0.22837
187.599	-0.5656	-0.22837
187.617	-0.56561	-0.22837
187.633	-0.56562	-0.22838
187.65	-0.56564	-0.22838
187.667	-0.56565	-0.22839
187.683	-0.56566	-0.2284
187.699	-0.56567	-0.22841
187.716	-0.56568	-0.22842
187.733	-0.56569	-0.22843
187.749	-0.5657	-0.22844
187.767	-0.56571	-0.22844
187.784	-0.56572	-0.22845
187.8	-0.56573	-0.22845
187.817	-0.56574	-0.22845
187.833	-0.56575	-0.22846
187.851	-0.56576	-0.22846
187.867	-0.56577	-0.22847
187.884	-0.56578	-0.22847
187.901	-0.56578	-0.22848
187.917	-0.56579	-0.22848
187.934	-0.5658	-0.22849
187.95	-0.56581	-0.22849
187.967	-0.56581	-0.2285
187.984	-0.56582	-0.22851
188.001	-0.56583	-0.22851
188.018	-0.56583	-0.22852
188.034	-0.56584	-0.22853
188.051	-0.56585	-0.22854
188.068	-0.56586	-0.22854

188.085	-0.56586	-0.22855
188.101	-0.56587	-0.22856
188.118	-0.56588	-0.22856
188.134	-0.56588	-0.22857
188.151	-0.56589	-0.22857
188.168	-0.5659	-0.22857
188.185	-0.56591	-0.22857
188.201	-0.56592	-0.22857
188.217	-0.56593	-0.22858
188.234	-0.56594	-0.22858
188.252	-0.56595	-0.22858
188.268	-0.56596	-0.22859
188.284	-0.56597	-0.22859
188.301	-0.56598	-0.22859
188.317	-0.56598	-0.2286
188.334	-0.56599	-0.2286
188.351	-0.566	-0.2286
188.368	-0.566	-0.2286
188.384	-0.56601	-0.2286
188.4	-0.56602	-0.22861
188.417	-0.56602	-0.22861
188.435	-0.56603	-0.22862
188.451	-0.56604	-0.22862
188.468	-0.56605	-0.22862
188.483	-0.56605	-0.22863
188.5	-0.56606	-0.22863
188.517	-0.56607	-0.22863
188.534	-0.56607	-0.22863
188.55	-0.56607	-0.22863
188.567	-0.56608	-0.22864

188.584	-0.56608	-0.22864
188.6	-0.56609	-0.22864
188.617	-0.56609	-0.22864
188.633	-0.5661	-0.22865
188.651	-0.5661	-0.22865
188.667	-0.56612	-0.22865
188.684	-0.56612	-0.22865
188.7	-0.56613	-0.22866
188.717	-0.56614	-0.22866
188.733	-0.56615	-0.22866
188.751	-0.56616	-0.22866
188.767	-0.56618	-0.22867
188.784	-0.56619	-0.22867
188.801	-0.5662	-0.22867
188.818	-0.56622	-0.22868
188.834	-0.56623	-0.22867
188.85	-0.56624	-0.22867
188.868	-0.56625	-0.22867
188.883	-0.56625	-0.22867
188.9	-0.56626	-0.22867
188.916	-0.56627	-0.22867
188.934	-0.56628	-0.22867
188.951	-0.56629	-0.22867
188.967	-0.5663	-0.22868
188.984	-0.56632	-0.22869
189.001	-0.56634	-0.22869
189.017	-0.56636	-0.2287
189.034	-0.56639	-0.2287
189.051	-0.5664	-0.2287
189.067	-0.56642	-0.2287

189.084	-0.56643	-0.2287
189.099	-0.56644	-0.2287
189.117	-0.56646	-0.2287
189.133	-0.56647	-0.2287
189.15	-0.56648	-0.22871
189.166	-0.56649	-0.22871
189.183	-0.5665	-0.22871
189.201	-0.56652	-0.22872
189.217	-0.56653	-0.22872
189.234	-0.56654	-0.22873
189.25	-0.56656	-0.22873
189.267	-0.56657	-0.22874
189.284	-0.56658	-0.22874
189.301	-0.56659	-0.22874
189.318	-0.56661	-0.22874
189.334	-0.56662	-0.22874
189.352	-0.56663	-0.22874
189.368	-0.56665	-0.22874
189.384	-0.56667	-0.22875
189.4	-0.56668	-0.22875
189.417	-0.5667	-0.22876
189.434	-0.56671	-0.22877
189.451	-0.56673	-0.22877
189.467	-0.56673	-0.22877
189.482	-0.56674	-0.22878
189.501	-0.56675	-0.22878
189.518	-0.56676	-0.22878
189.534	-0.56677	-0.22878
189.55	-0.56678	-0.22878
189.567	-0.5668	-0.22878

189.583	-0.56681	-0.22879
189.599	-0.56683	-0.22879
189.616	-0.56685	-0.22879
189.633	-0.56686	-0.22879
189.649	-0.56688	-0.22879
189.666	-0.56689	-0.22879
189.682	-0.5669	-0.22879
189.699	-0.56691	-0.22879
189.716	-0.56691	-0.2288
189.733	-0.56692	-0.2288
189.749	-0.56692	-0.22881
189.766	-0.56692	-0.22881
189.783	-0.56693	-0.22882
189.8	-0.56694	-0.22882
189.816	-0.56694	-0.22883
189.833	-0.56695	-0.22883
189.85	-0.56695	-0.22883
189.866	-0.56696	-0.22883
189.883	-0.56697	-0.22884
189.899	-0.56697	-0.22884
189.916	-0.56698	-0.22884
189.933	-0.56699	-0.22884
189.95	-0.567	-0.22884
189.966	-0.56701	-0.22884
189.981	-0.56702	-0.22884
189.999	-0.56703	-0.22884
190.016	-0.56704	-0.22885
190.032	-0.56705	-0.22885
190.05	-0.56706	-0.22885
190.066	-0.56708	-0.22886

190.083	-0.56709	-0.22886
190.099	-0.5671	-0.22887
190.116	-0.56712	-0.22887
190.133	-0.56713	-0.22888
190.149	-0.56715	-0.22888
190.166	-0.56716	-0.22889
190.183	-0.56717	-0.22889
190.199	-0.56718	-0.2289
190.216	-0.56719	-0.2289
190.233	-0.56721	-0.22891
190.249	-0.56722	-0.22892
190.266	-0.56723	-0.22892
190.283	-0.56725	-0.22892
190.299	-0.56726	-0.22892
190.316	-0.56728	-0.22893
190.332	-0.5673	-0.22893
190.35	-0.56732	-0.22893
190.366	-0.56734	-0.22894
190.382	-0.56736	-0.22894
190.399	-0.56737	-0.22895
190.416	-0.56739	-0.22895
190.433	-0.56741	-0.22896
190.449	-0.56742	-0.22896
190.466	-0.56744	-0.22897
190.483	-0.56746	-0.22898
190.499	-0.56748	-0.22899
190.517	-0.56751	-0.229
190.533	-0.56753	-0.22901
190.55	-0.56755	-0.22901
190.566	-0.56757	-0.22902

190.583	-0.5676	-0.22902
190.6	-0.56762	-0.22903
190.617	-0.56764	-0.22903
190.634	-0.56767	-0.22903
190.65	-0.56769	-0.22904
190.667	-0.56771	-0.22905
190.683	-0.56773	-0.22906
190.7	-0.56776	-0.22907
190.717	-0.56778	-0.22908
190.734	-0.5678	-0.22908
190.751	-0.56782	-0.22909
190.766	-0.56784	-0.2291
190.784	-0.56786	-0.2291
190.8	-0.56788	-0.22911
190.817	-0.5679	-0.22912
190.834	-0.56793	-0.22912
190.85	-0.56795	-0.22913
190.867	-0.56796	-0.22914
190.884	-0.56798	-0.22915
190.901	-0.568	-0.22915
190.918	-0.56801	-0.22916
190.934	-0.56802	-0.22916
190.951	-0.56804	-0.22917
190.968	-0.56806	-0.22918
190.984	-0.56807	-0.22919
191.001	-0.56809	-0.2292
191.018	-0.56812	-0.22921
191.035	-0.56815	-0.22922
191.052	-0.56819	-0.22923
191.068	-0.56827	-0.22924

191.084	-0.56836	-0.22924
191.102	-0.56851	-0.22925
191.118	-0.56868	-0.22926
191.135	-0.56891	-0.22926
191.152	-0.56916	-0.22927
191.168	-0.56941	-0.22927
191.184	-0.56965	-0.22927
191.201	-0.56989	-0.22927
191.217	-0.57005	-0.22928
191.235	-0.57014	-0.22928
191.252	-0.5702	-0.22929
191.268	-0.57025	-0.22929
191.285	-0.57028	-0.2293
191.302	-0.5703	-0.22931
191.318	-0.57033	-0.22932
191.336	-0.57035	-0.22933
191.352	-0.57036	-0.22933
191.369	-0.57038	-0.22934
191.385	-0.5704	-0.22934
191.401	-0.57042	-0.22935
191.418	-0.57043	-0.22936
191.435	-0.57044	-0.22936
191.452	-0.57046	-0.22936
191.468	-0.57047	-0.22937
191.485	-0.57048	-0.22937
191.502	-0.57048	-0.22938
191.519	-0.57049	-0.22939
191.535	-0.57049	-0.22939
191.552	-0.5705	-0.2294
191.569	-0.5705	-0.2294

191.585	-0.5705	-0.2294
191.603	-0.57051	-0.22941
191.618	-0.57051	-0.22941
191.636	-0.57052	-0.22941
191.653	-0.57052	-0.22942
191.668	-0.57052	-0.22942
191.685	-0.57052	-0.22942
191.702	-0.57052	-0.22943
191.719	-0.57052	-0.22943
191.735	-0.57052	-0.22943
191.752	-0.57052	-0.22944
191.768	-0.57051	-0.22944
191.785	-0.57051	-0.22944
191.802	-0.5705	-0.22944
191.819	-0.5705	-0.22944
191.835	-0.57049	-0.22944
191.852	-0.57049	-0.22945
191.869	-0.57049	-0.22945
191.885	-0.57049	-0.22945
191.902	-0.57048	-0.22946
191.919	-0.57048	-0.22946
191.935	-0.57047	-0.22947
191.952	-0.57047	-0.22947
191.968	-0.57048	-0.22947
191.986	-0.57048	-0.22948
192.002	-0.57049	-0.22948
192.018	-0.5705	-0.22948
192.036	-0.57053	-0.22949
192.052	-0.57063	-0.22949
192.069	-0.57076	-0.22949

192.084	-0.57091	-0.22949
192.102	-0.57104	-0.22949
192.118	-0.57111	-0.22948
192.135	-0.57115	-0.22948
192.152	-0.57116	-0.22948
192.168	-0.57117	-0.22948
192.185	-0.57118	-0.22948
192.202	-0.57118	-0.22948
192.219	-0.57118	-0.22949
192.235	-0.57118	-0.22949
192.252	-0.57119	-0.22949
192.268	-0.57119	-0.2295
192.285	-0.57119	-0.2295
192.302	-0.57118	-0.2295
192.319	-0.57118	-0.2295
192.335	-0.57118	-0.2295
192.351	-0.57117	-0.2295
192.368	-0.57116	-0.2295
192.384	-0.57116	-0.2295
192.402	-0.57115	-0.22951
192.419	-0.57114	-0.22951
192.435	-0.57113	-0.22951
192.452	-0.57111	-0.22951
192.468	-0.5711	-0.22952
192.484	-0.57109	-0.22952
192.502	-0.57108	-0.22952
192.519	-0.57108	-0.22952
192.535	-0.57107	-0.22952
192.552	-0.57107	-0.22952
192.569	-0.57106	-0.22952

192.585	-0.57106	-0.22952
192.602	-0.57105	-0.22952
192.618	-0.57105	-0.22952
192.635	-0.57104	-0.22952
192.651	-0.57103	-0.22952
192.667	-0.57103	-0.22952
192.684	-0.57102	-0.22952
192.701	-0.57102	-0.22952
192.719	-0.57101	-0.22952
192.735	-0.57101	-0.22953
192.75	-0.571	-0.22953
192.768	-0.571	-0.22953
192.784	-0.571	-0.22953
192.801	-0.57099	-0.22954
192.818	-0.57099	-0.22954
192.835	-0.571	-0.22954
192.852	-0.57102	-0.22954
192.868	-0.57108	-0.22954
192.885	-0.5712	-0.22954
192.902	-0.57145	-0.22954
192.918	-0.57171	-0.22955
192.935	-0.57198	-0.22955
192.951	-0.57216	-0.22956
192.968	-0.57227	-0.22956
192.985	-0.57235	-0.22957
193.001	-0.57239	-0.22957
193.018	-0.57242	-0.22958
193.035	-0.57243	-0.22958
193.051	-0.57244	-0.22958
193.068	-0.57245	-0.22958

193.085	-0.57246	-0.22959
193.102	-0.57246	-0.22959
193.118	-0.57246	-0.22959
193.135	-0.57246	-0.22959
193.151	-0.57246	-0.2296
193.168	-0.57246	-0.2296
193.185	-0.57246	-0.2296
193.201	-0.57247	-0.2296
193.219	-0.57247	-0.2296
193.234	-0.57246	-0.2296
193.252	-0.57246	-0.22961
193.268	-0.57246	-0.22961
193.285	-0.57245	-0.22962
193.303	-0.57244	-0.22963
193.319	-0.57244	-0.22963
193.335	-0.57243	-0.22964
193.352	-0.57242	-0.22964
193.368	-0.57242	-0.22964
193.384	-0.57241	-0.22964
193.402	-0.5724	-0.22964
193.417	-0.57239	-0.22965
193.434	-0.57239	-0.22965
193.451	-0.57238	-0.22965
193.468	-0.57237	-0.22966
193.485	-0.57236	-0.22967
193.502	-0.57234	-0.22968
193.519	-0.57233	-0.22969
193.535	-0.57232	-0.22969
193.551	-0.57231	-0.2297
193.568	-0.5723	-0.2297

193.584	-0.57229	-0.22971
193.602	-0.57228	-0.22971
193.618	-0.57226	-0.22971
193.635	-0.57225	-0.22972
193.652	-0.57224	-0.22972
193.669	-0.57223	-0.22972
193.685	-0.57223	-0.22973
193.702	-0.57222	-0.22973
193.719	-0.57221	-0.22974
193.735	-0.5722	-0.22974
193.751	-0.5722	-0.22974
193.768	-0.5722	-0.22974
193.785	-0.5722	-0.22974
193.801	-0.5722	-0.22975
193.818	-0.57222	-0.22975
193.834	-0.57225	-0.22976
193.851	-0.57232	-0.22977
193.868	-0.5724	-0.22978
193.885	-0.57248	-0.22978
193.902	-0.57253	-0.22979
193.918	-0.57257	-0.22979
193.935	-0.57259	-0.22979
193.951	-0.5726	-0.22979
193.969	-0.57261	-0.22979
193.984	-0.57261	-0.22979
194.001	-0.57262	-0.22979
194.018	-0.57262	-0.22979
194.035	-0.57261	-0.2298
194.052	-0.57261	-0.2298
194.068	-0.5726	-0.22981

194.085	-0.57259	-0.22981
194.101	-0.57258	-0.22982
194.118	-0.57257	-0.22982
194.134	-0.57256	-0.22983
194.151	-0.57256	-0.22983
194.168	-0.57256	-0.22984
194.184	-0.57255	-0.22984
194.201	-0.57255	-0.22984
194.218	-0.57254	-0.22985
194.234	-0.57253	-0.22985
194.251	-0.57252	-0.22985
194.268	-0.57251	-0.22985
194.285	-0.5725	-0.22985
194.301	-0.57249	-0.22985
194.317	-0.57247	-0.22986
194.335	-0.57246	-0.22986
194.35	-0.57245	-0.22986
194.367	-0.57244	-0.22986
194.384	-0.57243	-0.22986
194.4	-0.57242	-0.22987
194.417	-0.57241	-0.22987
194.434	-0.5724	-0.22988
194.45	-0.57239	-0.22989
194.467	-0.57237	-0.2299
194.483	-0.57236	-0.2299
194.501	-0.57235	-0.22991
194.518	-0.57234	-0.22992
194.535	-0.57234	-0.22993
194.551	-0.57233	-0.22993
194.568	-0.57232	-0.22993

194.585	-0.57231	-0.22993
194.601	-0.5723	-0.22993
194.617	-0.57228	-0.22993
194.635	-0.57227	-0.22994
194.651	-0.57227	-0.22994
194.668	-0.57227	-0.22994
194.684	-0.57227	-0.22995
194.701	-0.57228	-0.22996
194.718	-0.57234	-0.22997
194.735	-0.57245	-0.22997
194.751	-0.57264	-0.22998
194.768	-0.57278	-0.22998
194.784	-0.57295	-0.22999
194.802	-0.57306	-0.22999
194.818	-0.57312	-0.22999
194.835	-0.57315	-0.23
194.852	-0.57315	-0.23
194.869	-0.57316	-0.23001
194.885	-0.57316	-0.23002
194.901	-0.57317	-0.23002
194.918	-0.57317	-0.23003
194.935	-0.57317	-0.23004
194.952	-0.57318	-0.23005
194.968	-0.57319	-0.23006
194.985	-0.57319	-0.23007
195.002	-0.5732	-0.23008
195.019	-0.5732	-0.23009
195.036	-0.5732	-0.2301
195.052	-0.57319	-0.2301
195.068	-0.57319	-0.23011

195.085	-0.57318	-0.23011
195.102	-0.57317	-0.23012
195.119	-0.57317	-0.23012
195.135	-0.57316	-0.23013
195.153	-0.57315	-0.23013
195.167	-0.57314	-0.23014
195.185	-0.57314	-0.23015
195.202	-0.57313	-0.23016
195.22	-0.57313	-0.23017
195.235	-0.57313	-0.23018
195.253	-0.57312	-0.23019
195.269	-0.57312	-0.2302
195.286	-0.57312	-0.2302
195.302	-0.57311	-0.23021
195.319	-0.5731	-0.23021
195.335	-0.57309	-0.23022
195.352	-0.57308	-0.23022
195.369	-0.57307	-0.23023
195.386	-0.57306	-0.23023
195.403	-0.57305	-0.23024
195.419	-0.57304	-0.23025
195.436	-0.57303	-0.23026
195.453	-0.57302	-0.23027
195.469	-0.57302	-0.23027
195.486	-0.57301	-0.23028
195.503	-0.573	-0.23029
195.519	-0.573	-0.2303
195.535	-0.573	-0.2303
195.553	-0.573	-0.23031
195.569	-0.573	-0.23032

195.586	-0.57303	-0.23032
195.602	-0.57309	-0.23033
195.619	-0.57319	-0.23033
195.636	-0.57334	-0.23034
195.653	-0.57352	-0.23034
195.67	-0.57366	-0.23035
195.686	-0.57371	-0.23035
195.703	-0.57372	-0.23036
195.72	-0.57373	-0.23037
195.736	-0.57374	-0.23037
195.754	-0.57374	-0.23037
195.77	-0.57373	-0.23038
195.786	-0.57373	-0.23038
195.802	-0.57373	-0.23039
195.819	-0.57372	-0.23039
195.836	-0.57372	-0.23039
195.852	-0.57371	-0.23039
195.869	-0.5737	-0.2304
195.886	-0.57369	-0.2304
195.903	-0.57369	-0.23041
195.92	-0.57368	-0.23041
195.937	-0.57367	-0.23042
195.952	-0.57366	-0.23043
195.97	-0.57366	-0.23043
195.986	-0.57365	-0.23044
196.003	-0.57364	-0.23044
196.019	-0.57363	-0.23045
196.036	-0.57362	-0.23046
196.052	-0.57361	-0.23046
196.07	-0.5736	-0.23047

196.086	-0.57359	-0.23047
196.102	-0.57357	-0.23047
196.12	-0.57355	-0.23048
196.137	-0.57353	-0.23048
196.153	-0.57351	-0.23048
196.169	-0.5735	-0.23048
196.186	-0.57348	-0.23049
196.202	-0.57346	-0.23049
196.219	-0.57341	-0.2305
196.235	-0.57337	-0.23051
196.252	-0.57335	-0.23051
196.268	-0.57333	-0.23052
196.286	-0.57331	-0.23053
196.303	-0.57329	-0.23053
196.319	-0.57328	-0.23054
196.336	-0.57327	-0.23054
196.352	-0.57327	-0.23054
196.368	-0.57327	-0.23055
196.387	-0.57329	-0.23055
196.402	-0.57331	-0.23055
196.419	-0.57334	-0.23055
196.435	-0.57344	-0.23055
196.453	-0.57355	-0.23056
196.47	-0.57368	-0.23056
196.486	-0.57374	-0.23056
196.503	-0.57376	-0.23057
196.519	-0.57377	-0.23057
196.536	-0.57378	-0.23058
196.552	-0.57379	-0.23058
196.568	-0.5738	-0.23058

196.586	-0.5738	-0.23059
196.603	-0.57381	-0.23059
196.619	-0.57381	-0.23059
196.636	-0.57381	-0.2306
196.652	-0.57382	-0.2306
196.668	-0.57382	-0.23061
196.685	-0.57382	-0.23061
196.703	-0.57381	-0.23062
196.719	-0.57381	-0.23062
196.736	-0.5738	-0.23063
196.752	-0.5738	-0.23063
196.769	-0.57379	-0.23063
196.785	-0.57378	-0.23063
196.802	-0.57377	-0.23063
196.818	-0.57376	-0.23063
196.835	-0.57375	-0.23064
196.851	-0.57374	-0.23064
196.868	-0.57373	-0.23064
196.885	-0.57372	-0.23065
196.901	-0.57371	-0.23065
196.919	-0.5737	-0.23066
196.935	-0.57368	-0.23066
196.952	-0.57366	-0.23067
196.969	-0.57365	-0.23068
196.986	-0.57364	-0.23069
197.002	-0.57362	-0.2307
197.018	-0.57361	-0.23071
197.035	-0.57361	-0.23072
197.052	-0.5736	-0.23072
197.069	-0.57359	-0.23073

197.084	-0.57358	-0.23074
197.102	-0.57357	-0.23075
197.118	-0.57356	-0.23075
197.135	-0.57355	-0.23076
197.151	-0.57354	-0.23076
197.169	-0.57354	-0.23076
197.184	-0.57354	-0.23077
197.201	-0.57353	-0.23077
197.218	-0.57353	-0.23078
197.235	-0.57354	-0.23078
197.25	-0.57357	-0.23078
197.268	-0.57362	-0.23079
197.284	-0.57371	-0.23079
197.301	-0.57382	-0.23079
197.318	-0.57392	-0.2308
197.335	-0.57401	-0.23081
197.352	-0.57406	-0.23081
197.368	-0.5741	-0.23082
197.386	-0.57412	-0.23083
197.402	-0.57413	-0.23084
197.418	-0.57414	-0.23084
197.434	-0.57415	-0.23085
197.451	-0.57415	-0.23085
197.468	-0.57415	-0.23086
197.485	-0.57415	-0.23087
197.501	-0.57416	-0.23087
197.518	-0.57416	-0.23088
197.534	-0.57416	-0.23089
197.551	-0.57415	-0.2309
197.568	-0.57415	-0.23091

197.585	-0.57415	-0.23091
197.602	-0.57415	-0.23092
197.618	-0.57414	-0.23093
197.634	-0.57414	-0.23094
197.651	-0.57413	-0.23095
197.668	-0.57413	-0.23095
197.685	-0.57412	-0.23096
197.701	-0.57411	-0.23097
197.718	-0.5741	-0.23097
197.735	-0.5741	-0.23097
197.751	-0.57409	-0.23098
197.768	-0.57408	-0.23098
197.785	-0.57407	-0.23099
197.801	-0.57407	-0.23099
197.817	-0.57406	-0.231
197.835	-0.57405	-0.23101
197.851	-0.57405	-0.23102
197.869	-0.57403	-0.23104
197.885	-0.57402	-0.23105
197.903	-0.57401	-0.23106
197.918	-0.57401	-0.23107
197.935	-0.574	-0.23108
197.952	-0.574	-0.23109
197.969	-0.574	-0.2311
197.985	-0.574	-0.2311
198.002	-0.57402	-0.23111
198.018	-0.57407	-0.23111
198.036	-0.57412	-0.23112
198.051	-0.57419	-0.23112
198.068	-0.57427	-0.23113

198.085	-0.57434	-0.23113
198.102	-0.57438	-0.23114
198.118	-0.57442	-0.23114
198.136	-0.57446	-0.23115
198.152	-0.57448	-0.23116
198.168	-0.57449	-0.23117
198.185	-0.5745	-0.23118
198.202	-0.57451	-0.23119
198.219	-0.57451	-0.2312
198.236	-0.57452	-0.23121
198.253	-0.57452	-0.23122
198.268	-0.57453	-0.23123
198.286	-0.57452	-0.23123
198.301	-0.57452	-0.23124
198.319	-0.57452	-0.23124
198.335	-0.57451	-0.23124
198.351	-0.57449	-0.23125
198.369	-0.57447	-0.23126
198.385	-0.57445	-0.23126
198.402	-0.57444	-0.23127
198.419	-0.57444	-0.23127
198.435	-0.57443	-0.23128
198.452	-0.57443	-0.23129
198.468	-0.57442	-0.23129
198.485	-0.57442	-0.2313
198.502	-0.57441	-0.23131
198.519	-0.57441	-0.23132
198.535	-0.5744	-0.23132
198.552	-0.57439	-0.23133
198.569	-0.57438	-0.23133

198.585	-0.57437	-0.23134
198.601	-0.57436	-0.23135
198.617	-0.57435	-0.23135
198.636	-0.57434	-0.23136
198.651	-0.57434	-0.23137
198.667	-0.57435	-0.23137
198.684	-0.57437	-0.23138
198.701	-0.57445	-0.23139
198.718	-0.57459	-0.2314
198.734	-0.57472	-0.23141
198.752	-0.57479	-0.23142
198.768	-0.57485	-0.23142
198.785	-0.57487	-0.23143
198.801	-0.57488	-0.23144
198.817	-0.57489	-0.23145
198.834	-0.57489	-0.23146
198.852	-0.5749	-0.23147
198.868	-0.5749	-0.23147
198.885	-0.5749	-0.23148
198.901	-0.5749	-0.23149
198.918	-0.5749	-0.23149
198.934	-0.5749	-0.2315
198.952	-0.57489	-0.23151
198.967	-0.5749	-0.23151
198.985	-0.5749	-0.23152
199.001	-0.5749	-0.23153
199.018	-0.5749	-0.23154
199.035	-0.5749	-0.23154
199.051	-0.5749	-0.23155
199.068	-0.57489	-0.23156

199.084	-0.57489	-0.23156
199.102	-0.57488	-0.23157
199.118	-0.57487	-0.23158
199.134	-0.57486	-0.23158
199.151	-0.57485	-0.23159
199.168	-0.57484	-0.2316
199.184	-0.57483	-0.2316
199.201	-0.57483	-0.23161
199.218	-0.57482	-0.23162
199.236	-0.57481	-0.23163
199.251	-0.5748	-0.23163
199.269	-0.57479	-0.23164
199.285	-0.57479	-0.23165
199.302	-0.57479	-0.23166
199.318	-0.5748	-0.23167
199.335	-0.57481	-0.23168
199.351	-0.57487	-0.23169
199.367	-0.57494	-0.2317
199.384	-0.57503	-0.23171
199.401	-0.57513	-0.23172
199.418	-0.57523	-0.23173
199.434	-0.57529	-0.23174
199.451	-0.57531	-0.23174
199.468	-0.57532	-0.23175
199.484	-0.57533	-0.23176
199.501	-0.57534	-0.23177
199.518	-0.57534	-0.23178
199.534	-0.57534	-0.2318
199.552	-0.57534	-0.2318
199.568	-0.57533	-0.23181

199.585	-0.57533	-0.23182
199.601	-0.57533	-0.23183
199.618	-0.57533	-0.23183
199.634	-0.57533	-0.23184
199.651	-0.57533	-0.23185
199.668	-0.57533	-0.23185
199.685	-0.57533	-0.23186
199.701	-0.57533	-0.23187
199.718	-0.57533	-0.23188
199.734	-0.57533	-0.23189
199.751	-0.57532	-0.23191
199.768	-0.57532	-0.23192
199.785	-0.57531	-0.23193
199.801	-0.57531	-0.23193
199.818	-0.5753	-0.23194
199.835	-0.5753	-0.23195
199.85	-0.57529	-0.23196
199.868	-0.57529	-0.23197
199.884	-0.57529	-0.23198
199.901	-0.5753	-0.23199
199.918	-0.57532	-0.232
199.935	-0.57538	-0.23201
199.952	-0.57556	-0.23203
199.968	-0.57579	-0.23204
199.985	-0.57602	-0.23205
200.001	-0.57622	-0.23206
200.018	-0.57632	-0.23206
200.035	-0.57637	-0.23207
200.052	-0.5764	-0.23208
200.068	-0.57642	-0.23209

200.084	-0.57645	-0.2321
200.102	-0.57646	-0.23211
200.118	-0.57647	-0.23212
200.134	-0.57648	-0.23214
200.151	-0.57649	-0.23215
200.168	-0.57649	-0.23216
200.185	-0.57649	-0.23218
200.202	-0.5765	-0.23219
200.218	-0.57649	-0.2322
200.234	-0.57649	-0.23221
200.251	-0.57648	-0.23222
200.267	-0.57648	-0.23223
200.284	-0.57647	-0.23224
200.301	-0.57647	-0.23225
200.318	-0.57647	-0.23226
200.335	-0.57646	-0.23227
200.352	-0.57646	-0.23229
200.368	-0.57645	-0.2323
200.384	-0.57644	-0.23232
200.401	-0.57643	-0.23233
200.417	-0.57641	-0.23235
200.435	-0.57638	-0.23236
200.452	-0.57636	-0.23237
200.468	-0.57635	-0.23238
200.485	-0.57634	-0.23239
200.502	-0.57633	-0.2324
200.518	-0.57632	-0.23241
200.535	-0.57632	-0.23242
200.552	-0.57632	-0.23243
200.569	-0.57633	-0.23245

200.585	-0.57636	-0.23246
200.601	-0.57643	-0.23247
200.618	-0.57652	-0.23248
200.635	-0.57663	-0.23249
200.651	-0.57674	-0.2325
200.669	-0.57683	-0.23252
200.685	-0.57689	-0.23253
200.702	-0.57692	-0.23255
200.718	-0.57693	-0.23256
200.735	-0.57694	-0.23258
200.752	-0.57694	-0.23259
200.768	-0.57694	-0.23261
200.786	-0.57695	-0.23262
200.801	-0.57695	-0.23263
200.818	-0.57696	-0.23264
200.835	-0.57696	-0.23265
200.852	-0.57696	-0.23266
200.868	-0.57696	-0.23267
200.885	-0.57696	-0.23269
200.902	-0.57695	-0.2327
200.919	-0.57695	-0.23271
200.935	-0.57694	-0.23272
200.952	-0.57693	-0.23273
200.968	-0.57692	-0.23274
200.985	-0.57691	-0.23276
201.001	-0.5769	-0.23277
201.018	-0.57689	-0.23278
201.035	-0.57689	-0.2328
201.052	-0.57688	-0.23281
201.068	-0.57687	-0.23283

201.085	-0.57686	-0.23284
201.103	-0.57685	-0.23286
201.119	-0.57684	-0.23287
201.136	-0.57683	-0.23288
201.152	-0.57683	-0.23289
201.168	-0.57683	-0.23289
201.185	-0.57683	-0.2329
201.201	-0.57683	-0.23291
201.218	-0.57684	-0.23292
201.235	-0.57687	-0.23293
201.252	-0.57691	-0.23294
201.269	-0.57697	-0.23296
201.285	-0.57699	-0.23297
201.301	-0.577	-0.23299
201.319	-0.57701	-0.23301
201.335	-0.57701	-0.23303
201.352	-0.57702	-0.23304
201.368	-0.57702	-0.23305
201.385	-0.57702	-0.23307
201.402	-0.57702	-0.23308
201.418	-0.57702	-0.23309
201.435	-0.57702	-0.2331
201.451	-0.57702	-0.23311
201.468	-0.57702	-0.23312
201.486	-0.57702	-0.23312
201.501	-0.57701	-0.23313
201.518	-0.577	-0.23314
201.535	-0.577	-0.23315
201.551	-0.57699	-0.23316
201.568	-0.57698	-0.23317

201.585	-0.57697	-0.23319
201.602	-0.57696	-0.2332
201.618	-0.57696	-0.23322
201.635	-0.57695	-0.23323
201.652	-0.57695	-0.23325
201.668	-0.57694	-0.23326
201.685	-0.57694	-0.23327
201.701	-0.57694	-0.23329
201.718	-0.57694	-0.2333
201.734	-0.57695	-0.23331
201.751	-0.57696	-0.23333
201.768	-0.57699	-0.23334
201.785	-0.57704	-0.23335
201.802	-0.57707	-0.23336
201.817	-0.57709	-0.23337
201.835	-0.5771	-0.23338
201.851	-0.57712	-0.23339
201.868	-0.57712	-0.2334
201.884	-0.57713	-0.23342
201.901	-0.57713	-0.23343
201.918	-0.57713	-0.23345
201.935	-0.57713	-0.23347
201.952	-0.57713	-0.23349
201.969	-0.57713	-0.23351
201.985	-0.57713	-0.23352
202.001	-0.57712	-0.23354
202.017	-0.57712	-0.23355
202.034	-0.57711	-0.23357
202.05	-0.57711	-0.23358
202.068	-0.5771	-0.23359

202.084	-0.5771	-0.2336
202.101	-0.57709	-0.23362
202.118	-0.57709	-0.23363
202.134	-0.57709	-0.23365
202.152	-0.5771	-0.23366
202.168	-0.57711	-0.23367
202.185	-0.57712	-0.23369
202.201	-0.57714	-0.2337
202.218	-0.5772	-0.23371
202.234	-0.57727	-0.23372
202.251	-0.57734	-0.23374
202.268	-0.57738	-0.23375
202.284	-0.57739	-0.23377
202.301	-0.5774	-0.23379
202.318	-0.57741	-0.2338
202.334	-0.57742	-0.23382
202.351	-0.57742	-0.23384
202.368	-0.57742	-0.23385
202.384	-0.57742	-0.23387
202.401	-0.57742	-0.23388
202.417	-0.57742	-0.2339
202.434	-0.57742	-0.23391
202.45	-0.57741	-0.23392
202.467	-0.5774	-0.23394
202.484	-0.5774	-0.23396
202.501	-0.57739	-0.23397
202.517	-0.57739	-0.23399
202.534	-0.57739	-0.23401
202.551	-0.57739	-0.23403
202.567	-0.57738	-0.23406

202.584	-0.57739	-0.23408
202.6	-0.57739	-0.2341
202.618	-0.57739	-0.23412
202.634	-0.57739	-0.23413
202.651	-0.5774	-0.23415
202.667	-0.57742	-0.23416
202.684	-0.57745	-0.23418
202.701	-0.5775	-0.2342
202.718	-0.57754	-0.23421
202.734	-0.57759	-0.23423
202.751	-0.57763	-0.23424
202.767	-0.57766	-0.23426
202.784	-0.57767	-0.23428
202.8	-0.57768	-0.2343
202.817	-0.57769	-0.23431
202.834	-0.5777	-0.23433
202.851	-0.5777	-0.23435
202.868	-0.57771	-0.23437
202.884	-0.57771	-0.23439
202.901	-0.57771	-0.2344
202.917	-0.5777	-0.23442
202.934	-0.5777	-0.23444
202.95	-0.5777	-0.23446
202.967	-0.57769	-0.23448
202.983	-0.57769	-0.2345
203	-0.57769	-0.23452
203.017	-0.57768	-0.23454
203.034	-0.57766	-0.23455
203.051	-0.57765	-0.23457
203.067	-0.57763	-0.23459

203.084	-0.57762	-0.23461
203.101	-0.57761	-0.23463
203.118	-0.57761	-0.23465
203.135	-0.57762	-0.23467
203.151	-0.57763	-0.23469
203.168	-0.57767	-0.23471
203.184	-0.57774	-0.23473
203.201	-0.57783	-0.23474
203.218	-0.5779	-0.23476
203.234	-0.57795	-0.23478
203.252	-0.57798	-0.2348
203.267	-0.57799	-0.23482
203.284	-0.578	-0.23484
203.301	-0.578	-0.23486
203.318	-0.57801	-0.23488
203.334	-0.57801	-0.2349
203.35	-0.57801	-0.23492
203.368	-0.57801	-0.23494
203.384	-0.57801	-0.23496
203.402	-0.57801	-0.23498
203.418	-0.57801	-0.235
203.434	-0.578	-0.23502
203.45	-0.578	-0.23504
203.467	-0.57801	-0.23507
203.483	-0.57801	-0.2351
203.5	-0.57801	-0.23513
203.518	-0.578	-0.23515
203.534	-0.578	-0.23517
203.551	-0.578	-0.23519
203.566	-0.57799	-0.23521

203.584	-0.57799	-0.23523
203.601	-0.57799	-0.23525
203.617	-0.578	-0.23526
203.634	-0.57801	-0.23528
203.651	-0.57803	-0.2353
203.667	-0.57808	-0.23532
203.684	-0.57812	-0.23534
203.701	-0.57817	-0.23536
203.717	-0.57818	-0.23538
203.734	-0.5782	-0.2354
203.751	-0.5782	-0.23542
203.767	-0.57821	-0.23544
203.784	-0.57822	-0.23548
203.801	-0.57822	-0.2355
203.817	-0.57822	-0.23552
203.834	-0.57822	-0.23554
203.85	-0.57822	-0.23556
203.868	-0.57822	-0.23558
203.885	-0.57822	-0.23561
203.901	-0.57822	-0.23563
203.918	-0.57822	-0.23565
203.934	-0.57821	-0.23567
203.951	-0.57821	-0.23569
203.967	-0.57821	-0.23571
203.984	-0.5782	-0.23573
204	-0.57819	-0.23575
204.017	-0.57818	-0.23576
204.034	-0.57818	-0.23578
204.05	-0.57818	-0.23581
204.068	-0.57818	-0.23583

204.084	-0.57818	-0.23586
204.1	-0.57818	-0.2359
204.117	-0.57819	-0.23592
204.134	-0.5782	-0.23595
204.151	-0.57823	-0.23598
204.168	-0.57826	-0.236
204.184	-0.57829	-0.23602
204.201	-0.57831	-0.23604
204.217	-0.57833	-0.23606
204.234	-0.57834	-0.23608
204.25	-0.57835	-0.2361
204.267	-0.57835	-0.23612
204.284	-0.57835	-0.23614
204.301	-0.57835	-0.23616
204.318	-0.57834	-0.23618
204.334	-0.57834	-0.2362
204.349	-0.57833	-0.23622
204.367	-0.57833	-0.23625
204.383	-0.57833	-0.23627
204.4	-0.57833	-0.23629
204.417	-0.57832	-0.23631
204.433	-0.57832	-0.23633
204.449	-0.57831	-0.23636
204.466	-0.57829	-0.23638
204.483	-0.57828	-0.2364
204.499	-0.57827	-0.23643
204.516	-0.57827	-0.23645
204.533	-0.57827	-0.23648
204.55	-0.57827	-0.23653
204.567	-0.57829	-0.23656

204.583	-0.57834	-0.23658
204.6	-0.57839	-0.23661
204.616	-0.57845	-0.23663
204.633	-0.5785	-0.23666
204.649	-0.57852	-0.23668
204.666	-0.57854	-0.2367
204.683	-0.57855	-0.23672
204.699	-0.57855	-0.23674
204.716	-0.57856	-0.23676
204.733	-0.57857	-0.23679
204.749	-0.57858	-0.23681
204.766	-0.57859	-0.23683
204.783	-0.57859	-0.23686
204.8	-0.5786	-0.2369
204.817	-0.5786	-0.23693
204.833	-0.5786	-0.23696
204.85	-0.57861	-0.23699
204.867	-0.57861	-0.23702
204.883	-0.57861	-0.23705
204.899	-0.57861	-0.23707
204.916	-0.57861	-0.23711
204.933	-0.57862	-0.23715
204.949	-0.57863	-0.23718
204.966	-0.57864	-0.23721
204.983	-0.57866	-0.23723
205	-0.5787	-0.23726
205.016	-0.57874	-0.23729
205.032	-0.57879	-0.23732
205.049	-0.57882	-0.23735
205.066	-0.57883	-0.23738

205.083	-0.57885	-0.23742
205.1	-0.57886	-0.23748
205.116	-0.57887	-0.23757
205.133	-0.57888	-0.23766
205.15	-0.57888	-0.23775
205.166	-0.57889	-0.23784
205.183	-0.57889	-0.2379
205.2	-0.5789	-0.23794
205.216	-0.5789	-0.23797
205.233	-0.57891	-0.238
205.25	-0.57892	-0.23803
205.266	-0.57894	-0.23806
205.283	-0.57901	-0.23808
205.3	-0.57911	-0.23811
205.317	-0.5792	-0.23814
205.333	-0.57926	-0.23817
205.349	-0.57933	-0.2382
205.366	-0.57939	-0.23823
205.383	-0.57941	-0.23826
205.399	-0.57943	-0.23829
205.417	-0.57944	-0.23832
205.433	-0.57946	-0.23835
205.45	-0.57947	-0.23839
205.467	-0.57948	-0.23842
205.483	-0.57949	-0.23845
205.5	-0.57951	-0.23848
205.517	-0.57951	-0.23851
205.533	-0.57952	-0.23854
205.551	-0.57952	-0.23857
205.567	-0.57953	-0.23862

205.584	-0.57953	-0.23869
205.601	-0.57953	-0.23875
205.618	-0.57953	-0.23879
205.633	-0.57953	-0.23883
205.65	-0.57954	-0.23886
205.666	-0.57955	-0.23889
205.684	-0.57956	-0.23893
205.7	-0.57957	-0.23896
205.717	-0.57959	-0.23899
205.733	-0.57962	-0.23902
205.75	-0.57965	-0.23905
205.767	-0.57969	-0.23909
205.783	-0.5797	-0.23912
205.8	-0.57972	-0.23915
205.817	-0.57973	-0.23919
205.833	-0.57974	-0.23923
205.85	-0.57975	-0.23927
205.867	-0.57975	-0.23931
205.883	-0.57976	-0.23936
205.9	-0.57977	-0.2394
205.917	-0.57977	-0.23943
205.933	-0.57978	-0.23947
205.95	-0.57978	-0.23951
205.966	-0.57979	-0.23955
205.982	-0.57979	-0.23958
206	-0.57979	-0.23962
206.016	-0.57979	-0.23965
206.033	-0.57979	-0.23969
206.05	-0.5798	-0.23972
206.066	-0.57982	-0.23975

206.083	-0.57983	-0.23978
206.1	-0.57985	-0.23982
206.116	-0.57986	-0.23985
206.132	-0.57988	-0.23988
206.149	-0.5799	-0.23993
206.166	-0.57991	-0.23998
206.183	-0.57992	-0.24004
206.199	-0.57993	-0.24013
206.217	-0.57993	-0.24021
206.234	-0.57994	-0.24028
206.25	-0.57994	-0.24032
206.267	-0.57995	-0.24036
206.282	-0.57995	-0.2404
206.299	-0.57995	-0.24044
206.316	-0.57995	-0.24048
206.332	-0.57995	-0.24053
206.349	-0.57995	-0.24058
206.366	-0.57995	-0.24061
206.383	-0.57996	-0.24065
206.399	-0.57996	-0.24068
206.416	-0.57998	-0.24072
206.433	-0.57999	-0.24077
206.449	-0.58001	-0.24081
206.466	-0.58003	-0.24085
206.482	-0.58007	-0.24088
206.5	-0.58009	-0.24093
206.516	-0.5801	-0.24099
206.533	-0.58012	-0.24104
206.548	-0.58013	-0.2411
206.566	-0.58014	-0.24113

206.583	-0.58015	-0.24117
206.598	-0.58015	-0.2412
206.615	-0.58016	-0.24124
206.632	-0.58017	-0.24128
206.648	-0.58017	-0.24134
206.666	-0.58017	-0.24141
206.682	-0.58017	-0.24151
206.699	-0.58015	-0.24162
206.715	-0.58013	-0.24171
206.731	-0.58012	-0.24185
206.749	-0.58011	-0.24192
206.766	-0.58011	-0.24201
206.782	-0.58011	-0.24209
206.798	-0.58012	-0.24215
206.815	-0.58013	-0.24218
206.831	-0.58018	-0.24222
206.848	-0.58023	-0.24225
206.864	-0.5803	-0.24229
206.882	-0.58034	-0.24235
206.898	-0.58036	-0.24241
206.915	-0.58037	-0.24249
206.932	-0.58038	-0.24255
206.948	-0.58038	-0.24264
206.966	-0.58038	-0.24273
206.981	-0.58038	-0.24284
206.998	-0.58038	-0.24293
207.015	-0.58039	-0.24301
207.032	-0.58039	-0.24309
207.048	-0.58039	-0.24315
207.065	-0.58039	-0.24324

207.082	-0.58038	-0.24333
207.097	-0.58038	-0.24346
207.115	-0.58038	-0.24354
207.131	-0.58039	-0.24363
207.148	-0.58039	-0.24371
207.165	-0.5804	-0.24376
207.181	-0.58041	-0.24384
207.198	-0.58043	-0.24392
207.215	-0.58044	-0.24397
207.23	-0.58047	-0.24401
207.247	-0.58049	-0.24407
207.264	-0.5805	-0.24416
207.281	-0.58051	-0.24425
207.298	-0.58052	-0.24433
207.314	-0.58053	-0.2444
207.331	-0.58054	-0.24448
207.347	-0.58054	-0.24453
207.365	-0.58055	-0.24458
207.381	-0.58056	-0.24463
207.398	-0.58057	-0.24469
207.414	-0.58059	-0.24475
207.43	-0.58062	-0.24483
207.447	-0.58068	-0.24494
207.463	-0.5808	-0.24516
207.48	-0.58093	-0.24533
207.497	-0.58104	-0.2455
207.514	-0.58108	-0.24565
207.53	-0.5811	-0.24582
207.547	-0.58111	-0.24592
207.564	-0.58112	-0.24602

207.58	-0.58112	-0.24608
207.597	-0.58112	-0.24614
207.614	-0.58113	-0.24624
207.63	-0.58113	-0.24633
207.647	-0.58113	-0.24644
207.663	-0.58113	-0.24655
207.68	-0.58113	-0.24669
207.696	-0.58113	-0.24683
207.713	-0.58113	-0.24699
207.73	-0.58113	-0.24719
207.747	-0.58113	-0.24736
207.763	-0.58113	-0.24747
207.781	-0.58114	-0.24753
207.797	-0.58115	-0.24758
207.813	-0.58119	-0.24762
207.83	-0.58127	-0.24765
207.846	-0.58133	-0.24771
207.863	-0.58137	-0.2478
207.879	-0.5814	-0.24789
207.896	-0.58142	-0.24801
207.912	-0.58142	-0.24823
207.93	-0.58143	-0.24849
207.946	-0.58143	-0.24874
207.963	-0.58143	-0.24893
207.979	-0.58143	-0.24909
207.997	-0.58143	-0.24927
208.013	-0.58143	-0.24939
208.03	-0.58142	-0.24953
208.046	-0.58142	-0.24964
208.063	-0.58142	-0.24973

208.08	-0.58143	-0.2498
208.096	-0.58144	-0.24987
208.113	-0.58144	-0.24994
208.129	-0.58146	-0.25
208.145	-0.58148	-0.25004
208.163	-0.58151	-0.25012
208.179	-0.58153	-0.25024
208.196	-0.58155	-0.25039
208.212	-0.58157	-0.25055
208.229	-0.58158	-0.2507
208.246	-0.58159	-0.25087
208.262	-0.5816	-0.25108
208.279	-0.58161	-0.25127
208.295	-0.58162	-0.25144
208.312	-0.58162	-0.25162
208.328	-0.58162	-0.25181
208.345	-0.58163	-0.25197
208.361	-0.58164	-0.25212
208.378	-0.58164	-0.25223
208.395	-0.58166	-0.25236
208.412	-0.58167	-0.25246
208.428	-0.5817	-0.25255
208.444	-0.58175	-0.25265
208.461	-0.5818	-0.25278
208.478	-0.58182	-0.25286
208.494	-0.58183	-0.253
208.512	-0.58184	-0.25318
208.529	-0.58186	-0.25335
208.545	-0.58186	-0.25349
208.561	-0.58187	-0.2536

208.578	-0.58187	-0.25372
208.595	-0.58188	-0.25383
208.611	-0.5819	-0.25393
208.627	-0.58191	-0.25404
208.644	-0.58192	-0.25418
208.66	-0.58193	-0.25436
208.678	-0.58194	-0.2546
208.694	-0.58195	-0.2548
208.711	-0.58197	-0.25498
208.727	-0.58199	-0.25516
208.745	-0.582	-0.25537
208.761	-0.58204	-0.25557
208.777	-0.58208	-0.25582
208.794	-0.58212	-0.25602
208.811	-0.58215	-0.2562
208.827	-0.58217	-0.25643
208.845	-0.58219	-0.25665
208.861	-0.58221	-0.25691
208.878	-0.58223	-0.25715
208.894	-0.58224	-0.25734
208.911	-0.58225	-0.25746
208.928	-0.58226	-0.25761
208.944	-0.58226	-0.25776
208.961	-0.58227	-0.25789
208.978	-0.58227	-0.25801
208.993	-0.58227	-0.25813
209.011	-0.58228	-0.25826
209.027	-0.58229	-0.25838
209.045	-0.5823	-0.25855
209.061	-0.58232	-0.25871

209.077	-0.58234	-0.25889
209.094	-0.58237	-0.25908
209.11	-0.5824	-0.2593
209.128	-0.58242	-0.25947
209.144	-0.58244	-0.25962
209.16	-0.58245	-0.25974
209.177	-0.58247	-0.25986
209.194	-0.58247	-0.26
209.21	-0.58247	-0.26015
209.227	-0.58247	-0.26031
209.244	-0.58248	-0.2605
209.26	-0.58249	-0.26068
209.277	-0.5825	-0.26083
209.294	-0.58251	-0.26095
209.31	-0.58253	-0.26112
209.326	-0.58255	-0.26127
209.343	-0.58256	-0.26142
209.361	-0.58258	-0.26155
209.377	-0.58259	-0.26167
209.394	-0.5826	-0.26184
209.41	-0.58261	-0.26202
209.427	-0.58262	-0.2622
209.443	-0.58263	-0.26235
209.46	-0.58264	-0.26251
209.476	-0.58265	-0.26265
209.493	-0.58266	-0.26282
209.51	-0.58268	-0.26301
209.526	-0.5827	-0.26318
209.543	-0.58274	-0.26336
209.559	-0.58278	-0.26353

209.577	-0.58283	-0.26368
209.593	-0.58285	-0.26382
209.609	-0.58287	-0.26394
209.626	-0.58288	-0.26405
209.644	-0.5829	-0.2642
209.66	-0.58291	-0.26434
209.676	-0.58291	-0.26453
209.693	-0.58292	-0.26475
209.71	-0.58292	-0.26498
209.726	-0.58292	-0.26519
209.743	-0.58292	-0.26538
209.759	-0.58292	-0.26552
209.776	-0.58292	-0.26563
209.793	-0.58292	-0.26572
209.809	-0.58292	-0.26581
209.826	-0.58292	-0.26592
209.843	-0.58293	-0.26605
209.858	-0.58294	-0.26624
209.875	-0.58295	-0.26645
209.891	-0.58298	-0.26669
209.908	-0.583	-0.26694
209.925	-0.58302	-0.26716
209.942	-0.58303	-0.26735
209.959	-0.58304	-0.26752
209.976	-0.58305	-0.26767
209.992	-0.58305	-0.26783
210.008	-0.58306	-0.26802
210.025	-0.58306	-0.26821
210.042	-0.58307	-0.26837
210.058	-0.58307	-0.26851

210.075	-0.58308	-0.26864
210.091	-0.58309	-0.26876
210.109	-0.58311	-0.26886
210.125	-0.58312	-0.26894
210.142	-0.58314	-0.26904
210.158	-0.58315	-0.26914
210.175	-0.58317	-0.26927
210.191	-0.58317	-0.26943
210.208	-0.58318	-0.26961
210.224	-0.58318	-0.26976
210.24	-0.58318	-0.26995
210.257	-0.58318	-0.27014
210.273	-0.58317	-0.27031
210.29	-0.58317	-0.27049
210.308	-0.58317	-0.27065
210.324	-0.58318	-0.27078
210.341	-0.58318	-0.2709
210.357	-0.58319	-0.27103
210.373	-0.5832	-0.27118
210.39	-0.5832	-0.27135
210.407	-0.58321	-0.27151
210.423	-0.58322	-0.27164
210.44	-0.58322	-0.27181
210.457	-0.58322	-0.27199
210.473	-0.58322	-0.27219
210.489	-0.58321	-0.27239
210.505	-0.58321	-0.27261
210.523	-0.5832	-0.27277
210.538	-0.5832	-0.27294
210.556	-0.5832	-0.27315

210.572	-0.5832	-0.27338
210.589	-0.58321	-0.27361
210.606	-0.58321	-0.27381
210.622	-0.58322	-0.27402
210.638	-0.58324	-0.27423
210.655	-0.58326	-0.27438
210.672	-0.58328	-0.27452
210.689	-0.58329	-0.27469
210.705	-0.5833	-0.27482
210.722	-0.58331	-0.275
210.739	-0.58331	-0.27516
210.755	-0.58331	-0.27537
210.772	-0.58331	-0.27556
210.787	-0.5833	-0.27573
210.804	-0.5833	-0.27589
210.821	-0.5833	-0.27608
210.837	-0.5833	-0.27627
210.853	-0.58331	-0.27646
210.87	-0.58332	-0.27666
210.887	-0.58333	-0.27685
210.904	-0.58334	-0.27707
210.921	-0.58335	-0.27735
210.936	-0.58337	-0.27768
210.954	-0.58338	-0.27797
210.97	-0.58339	-0.27815
210.986	-0.5834	-0.27831
211.004	-0.5834	-0.27847
211.02	-0.58341	-0.27867
211.036	-0.58342	-0.27888
211.052	-0.58342	-0.27906

211.07	-0.58344	-0.27924
211.086	-0.58345	-0.27941
211.103	-0.58347	-0.27961
211.12	-0.5835	-0.27983
211.136	-0.58352	-0.28002
211.152	-0.58356	-0.28017
211.169	-0.58359	-0.28035
211.186	-0.5836	-0.28054
211.202	-0.58362	-0.28075
211.219	-0.58362	-0.28098
211.235	-0.58363	-0.28123
211.252	-0.58363	-0.28148
211.268	-0.58363	-0.28169
211.285	-0.58363	-0.28189
211.302	-0.58364	-0.28208
211.318	-0.58364	-0.2822
211.335	-0.58365	-0.28232
211.351	-0.58366	-0.28247
211.368	-0.58367	-0.28262
211.385	-0.58369	-0.28282
211.401	-0.58371	-0.28309
211.418	-0.58372	-0.28337
211.434	-0.58374	-0.28363
211.451	-0.58376	-0.28392
211.467	-0.58377	-0.28418
211.484	-0.58379	-0.28447
211.5	-0.5838	-0.28472
211.517	-0.58381	-0.28492
211.534	-0.58382	-0.28506
211.55	-0.58383	-0.2852

211.567	-0.58384	-0.28533
211.583	-0.58385	-0.28546
211.6	-0.58386	-0.28557
211.617	-0.58387	-0.28568
211.634	-0.58388	-0.28578
211.65	-0.58389	-0.28588
211.666	-0.5839	-0.28606
211.683	-0.58391	-0.28625
211.7	-0.58392	-0.28649
211.716	-0.58393	-0.2868
211.733	-0.58395	-0.2871
211.75	-0.58396	-0.28734
211.766	-0.58398	-0.28753
211.783	-0.58401	-0.28766
211.799	-0.58404	-0.2878
211.816	-0.58408	-0.2879
211.833	-0.58412	-0.28798
211.85	-0.58415	-0.28808
211.865	-0.58417	-0.28816
211.883	-0.58418	-0.28829
211.899	-0.5842	-0.28848
211.915	-0.5842	-0.28868
211.932	-0.58421	-0.28889
211.949	-0.58422	-0.28916
211.966	-0.58422	-0.28939
211.983	-0.58423	-0.28965
211.998	-0.58423	-0.28989
212.015	-0.58423	-0.29013
212.032	-0.58424	-0.29036
212.048	-0.58425	-0.29051

212.064	-0.58426	-0.2907
212.081	-0.58427	-0.29087
212.098	-0.58428	-0.29103
212.115	-0.5843	-0.29116
212.131	-0.58431	-0.29131
212.148	-0.58432	-0.29143
212.164	-0.58433	-0.29157
212.181	-0.58434	-0.29169
212.198	-0.58435	-0.29183
212.214	-0.58437	-0.29199
212.231	-0.58438	-0.29218
212.248	-0.5844	-0.29239
212.264	-0.58442	-0.29255
212.281	-0.58443	-0.2927
212.297	-0.58445	-0.29283
212.314	-0.58447	-0.29299
212.331	-0.58449	-0.29314
212.347	-0.58451	-0.29329
212.364	-0.58452	-0.29345
212.381	-0.58453	-0.29357
212.397	-0.58454	-0.29366
212.413	-0.58454	-0.29375
212.43	-0.58455	-0.29386
212.446	-0.58454	-0.29394
212.463	-0.58453	-0.29404
212.48	-0.58453	-0.29415
212.496	-0.58453	-0.29433
212.513	-0.58454	-0.29452
212.53	-0.58456	-0.29477
212.545	-0.58457	-0.295

212.562	-0.58459	-0.29525
212.579	-0.58461	-0.29547
212.595	-0.58462	-0.29567
212.613	-0.58464	-0.29583
212.628	-0.58465	-0.29598
212.645	-0.58465	-0.29611
212.662	-0.58465	-0.29623
212.678	-0.58465	-0.29633
212.695	-0.58465	-0.29643
212.712	-0.58466	-0.29662
212.728	-0.58466	-0.29682
212.745	-0.58467	-0.29702
212.762	-0.58468	-0.29717
212.778	-0.5847	-0.2973
212.795	-0.58472	-0.29742
212.811	-0.58474	-0.29753
212.828	-0.58476	-0.29759
212.844	-0.58477	-0.29764
212.861	-0.58479	-0.29769
212.877	-0.58479	-0.29777
212.895	-0.5848	-0.29788
212.91	-0.58481	-0.29803
212.928	-0.58482	-0.2982
212.944	-0.58483	-0.29837
212.961	-0.58485	-0.29855
212.978	-0.58488	-0.29873
212.994	-0.58492	-0.29887
213.01	-0.58496	-0.29897
213.027	-0.58498	-0.29907
213.043	-0.585	-0.29916

213.059	-0.58501	-0.29926
213.077	-0.58503	-0.29937
213.094	-0.58503	-0.29949
213.11	-0.58503	-0.2996
213.126	-0.58503	-0.29971
213.144	-0.58504	-0.29989
213.159	-0.58505	-0.30005
213.177	-0.58506	-0.30019
213.193	-0.58508	-0.30032
213.21	-0.58509	-0.30045
213.227	-0.58511	-0.30056
213.243	-0.58514	-0.30068
213.259	-0.58517	-0.30081
213.276	-0.58519	-0.30093
213.292	-0.5852	-0.30102
213.309	-0.58521	-0.30111
213.326	-0.58523	-0.30122
213.342	-0.58524	-0.30133
213.359	-0.58525	-0.30148
213.375	-0.58527	-0.30163
213.392	-0.58528	-0.30176
213.409	-0.5853	-0.3019
213.426	-0.58532	-0.30202
213.441	-0.58534	-0.30211
213.459	-0.58535	-0.3022
213.476	-0.58535	-0.3023
213.492	-0.58536	-0.3024
213.508	-0.58537	-0.30251
213.525	-0.58537	-0.30262
213.542	-0.58538	-0.30272

213.558	-0.5854	-0.30281
213.575	-0.58541	-0.30288
213.592	-0.58543	-0.30295
213.609	-0.58546	-0.30303
213.625	-0.5855	-0.30314
213.642	-0.58554	-0.30323
213.659	-0.58557	-0.30331
213.676	-0.58559	-0.30337
213.692	-0.58561	-0.30343
213.708	-0.58563	-0.30351
213.725	-0.58564	-0.3036
213.742	-0.58565	-0.30368
213.758	-0.58566	-0.30377
213.775	-0.58567	-0.30386
213.792	-0.58568	-0.30394
213.808	-0.58569	-0.30403
213.826	-0.58571	-0.30411
213.842	-0.58573	-0.30419
213.858	-0.58575	-0.30425
213.875	-0.58576	-0.3043
213.892	-0.58577	-0.30435
213.908	-0.58578	-0.30441
213.925	-0.58579	-0.30446
213.941	-0.58579	-0.30452
213.959	-0.58579	-0.30461
213.975	-0.58579	-0.30467
213.992	-0.58579	-0.30472
214.008	-0.58578	-0.30476
214.025	-0.58578	-0.30479
214.041	-0.58578	-0.30485

214.058	-0.58578	-0.30492
214.075	-0.58579	-0.305
214.091	-0.58579	-0.30509
214.108	-0.5858	-0.30517
214.126	-0.58581	-0.30522
214.141	-0.58582	-0.30528
214.158	-0.58583	-0.30533
214.174	-0.58584	-0.30537
214.191	-0.58585	-0.30541
214.208	-0.58586	-0.30544
214.224	-0.58586	-0.30547
214.241	-0.58587	-0.3055
214.257	-0.58588	-0.30553
214.274	-0.58589	-0.30557
214.291	-0.5859	-0.30561
214.307	-0.58591	-0.30565
214.324	-0.58592	-0.30569
214.341	-0.58593	-0.30573
214.358	-0.58594	-0.30578
214.374	-0.58595	-0.3058
214.39	-0.58596	-0.30584
214.407	-0.58596	-0.30586
214.423	-0.58596	-0.30589
214.441	-0.58597	-0.30592
214.458	-0.58598	-0.30595
214.474	-0.58598	-0.30598
214.491	-0.586	-0.30602
214.507	-0.58601	-0.30605
214.524	-0.58603	-0.30609
214.54	-0.58606	-0.30612

214.556	-0.58609	-0.30615
214.573	-0.58611	-0.30617
214.59	-0.58612	-0.30619
214.607	-0.58614	-0.30621
214.624	-0.58615	-0.30623
214.64	-0.58616	-0.30625
214.657	-0.58616	-0.30627
214.674	-0.58616	-0.30629
214.69	-0.58615	-0.30631
214.707	-0.58615	-0.30633
214.724	-0.58615	-0.30635
214.74	-0.58615	-0.30637
214.756	-0.58616	-0.3064
214.773	-0.58617	-0.30643
214.791	-0.58619	-0.30645
214.806	-0.58622	-0.30649
214.824	-0.58626	-0.30652
214.84	-0.58628	-0.30655
214.857	-0.58629	-0.30657
214.874	-0.5863	-0.3066
214.89	-0.58631	-0.30662
214.906	-0.58632	-0.30666
214.923	-0.58633	-0.30669
214.94	-0.58633	-0.30671
214.957	-0.58634	-0.30673
214.973	-0.58634	-0.30675
214.99	-0.58635	-0.30678
215.007	-0.58637	-0.3068
215.022	-0.58638	-0.30682
215.039	-0.5864	-0.30685

215.056	-0.58641	-0.30687
215.074	-0.58643	-0.30689
215.09	-0.58645	-0.30692
215.106	-0.58646	-0.30694
215.124	-0.58647	-0.30697
215.14	-0.58648	-0.30699
215.157	-0.58648	-0.30701
215.173	-0.58649	-0.30704
215.19	-0.58649	-0.30707
215.206	-0.58649	-0.30709
215.223	-0.58648	-0.30711
215.24	-0.58648	-0.30713
215.255	-0.58648	-0.30715
215.273	-0.58648	-0.30717
215.29	-0.58649	-0.30719
215.306	-0.5865	-0.30721
215.323	-0.58651	-0.30724
215.339	-0.58652	-0.30726
215.356	-0.58654	-0.30729
215.373	-0.58655	-0.30731
215.39	-0.58656	-0.30734
215.406	-0.58656	-0.30736
215.423	-0.58657	-0.30738
215.439	-0.58657	-0.3074
215.456	-0.58658	-0.30742
215.473	-0.58658	-0.30745
215.489	-0.58659	-0.30747
215.506	-0.5866	-0.30749
215.522	-0.58661	-0.30751
215.54	-0.58662	-0.30753

215.556	-0.58664	-0.30755
215.573	-0.58665	-0.30757
215.59	-0.58666	-0.30759
215.606	-0.58668	-0.30762
215.622	-0.58669	-0.30764
215.639	-0.5867	-0.30766
215.656	-0.58671	-0.30768
215.672	-0.58672	-0.3077
215.689	-0.58673	-0.30772
215.706	-0.58676	-0.30775
215.722	-0.58681	-0.30777
215.739	-0.58685	-0.30779
215.755	-0.58688	-0.30781
215.773	-0.5869	-0.30783
215.79	-0.58692	-0.30786
215.806	-0.58693	-0.30788
215.823	-0.58694	-0.30791
215.839	-0.58694	-0.30793
215.856	-0.58694	-0.30795
215.872	-0.58694	-0.30797
215.889	-0.58695	-0.308
215.907	-0.58695	-0.30802
215.923	-0.58696	-0.30805
215.939	-0.58698	-0.30807
215.956	-0.587	-0.30809
215.973	-0.58702	-0.30812
215.989	-0.58706	-0.30814
216.006	-0.58709	-0.30816
216.022	-0.58711	-0.30818
216.039	-0.58713	-0.3082

216.056	-0.58714	-0.30822
216.072	-0.58715	-0.30825
216.089	-0.58717	-0.30827
216.106	-0.58718	-0.30829
216.123	-0.5872	-0.30831
216.14	-0.58723	-0.30834
216.156	-0.58728	-0.30836
216.173	-0.58734	-0.30839
216.191	-0.58738	-0.30841
216.206	-0.5874	-0.30843
216.223	-0.58741	-0.30846
216.24	-0.58742	-0.30848
216.256	-0.58743	-0.30851
216.273	-0.58743	-0.30854
216.29	-0.58743	-0.30856
216.306	-0.58744	-0.30859
216.322	-0.58744	-0.30861
216.339	-0.58745	-0.30864
216.356	-0.58746	-0.30866
216.372	-0.58747	-0.30868
216.39	-0.58748	-0.3087
216.406	-0.5875	-0.30872
216.422	-0.58751	-0.30875
216.439	-0.58753	-0.30877
216.456	-0.58754	-0.3088
216.474	-0.58756	-0.30882
216.49	-0.58757	-0.30885
216.507	-0.58758	-0.30887
216.524	-0.5876	-0.3089
216.54	-0.58762	-0.30892

216.558	-0.58763	-0.30894
216.573	-0.58765	-0.30897
216.589	-0.58766	-0.30899
216.607	-0.58768	-0.30901
216.623	-0.5877	-0.30903
216.64	-0.58772	-0.30905
216.656	-0.58773	-0.30907
216.673	-0.58774	-0.30909
216.689	-0.58776	-0.30911
216.707	-0.58777	-0.30913
216.723	-0.58778	-0.30915
216.74	-0.58779	-0.30917
216.756	-0.58781	-0.3092
216.774	-0.58782	-0.30922
216.79	-0.58784	-0.30924
216.807	-0.58786	-0.30927
216.823	-0.58787	-0.30929
216.84	-0.58789	-0.30931
216.856	-0.58791	-0.30933
216.874	-0.58793	-0.30935
216.89	-0.58794	-0.30937
216.906	-0.58795	-0.30939
216.923	-0.58796	-0.30941
216.94	-0.58796	-0.30943
216.956	-0.58797	-0.30945
216.972	-0.58798	-0.30946
216.989	-0.58799	-0.30948
217.006	-0.588	-0.3095
217.022	-0.58801	-0.30952
217.038	-0.58803	-0.30954

217.057	-0.58804	-0.30956
217.073	-0.58805	-0.30959
217.089	-0.58806	-0.30961
217.106	-0.58808	-0.30963
217.123	-0.58809	-0.30965
217.138	-0.5881	-0.30966
217.156	-0.58811	-0.30968
217.172	-0.58813	-0.30971
217.189	-0.58814	-0.30973
217.206	-0.58816	-0.30975
217.223	-0.58818	-0.30977
217.24	-0.58819	-0.30979
217.257	-0.5882	-0.30981
217.274	-0.58821	-0.30983
217.29	-0.58822	-0.30984
217.307	-0.58822	-0.30986
217.322	-0.58822	-0.30987
217.339	-0.58823	-0.30989
217.355	-0.58823	-0.30991
217.373	-0.58824	-0.30993
217.389	-0.58825	-0.30995
217.407	-0.58827	-0.30997
217.422	-0.58828	-0.30999
217.44	-0.58829	-0.31002
217.455	-0.5883	-0.31004
217.473	-0.58831	-0.31006
217.489	-0.58831	-0.31008
217.506	-0.58832	-0.3101
217.522	-0.58832	-0.31013
217.539	-0.58833	-0.31015

217.556	-0.58834	-0.31017
217.572	-0.58835	-0.31019
217.589	-0.58836	-0.31021
217.605	-0.58837	-0.31023
217.622	-0.58838	-0.31025
217.639	-0.58839	-0.31028
217.656	-0.58839	-0.3103
217.673	-0.5884	-0.31033
217.689	-0.58841	-0.31036
217.706	-0.58842	-0.31038
217.722	-0.58842	-0.3104
217.738	-0.58843	-0.31043
217.755	-0.58844	-0.31045
217.772	-0.58844	-0.31048
217.789	-0.58845	-0.3105
217.806	-0.58846	-0.31053
217.822	-0.58847	-0.31055
217.839	-0.58848	-0.31058
217.855	-0.58849	-0.3106
217.871	-0.5885	-0.31063
217.888	-0.58852	-0.31065
217.905	-0.58853	-0.31068
217.922	-0.58855	-0.3107
217.938	-0.58858	-0.31073
217.955	-0.5886	-0.31076
217.971	-0.58861	-0.31078
217.988	-0.58863	-0.31082
218.005	-0.58864	-0.31086
218.022	-0.58864	-0.31089
218.039	-0.58865	-0.31092

218.055	-0.58866	-0.31094
218.071	-0.58866	-0.31097
218.088	-0.58867	-0.311
218.104	-0.58869	-0.31102
218.121	-0.5887	-0.31105
218.137	-0.58872	-0.31108
218.154	-0.58874	-0.31111
218.171	-0.58878	-0.31114
218.187	-0.5888	-0.31119
218.204	-0.58882	-0.31123
218.221	-0.58884	-0.31128
218.238	-0.58885	-0.31134
218.254	-0.58886	-0.3114
218.271	-0.58887	-0.31148
218.289	-0.58889	-0.31155
218.305	-0.5889	-0.31161
218.321	-0.58891	-0.3117
218.338	-0.58892	-0.31178
218.355	-0.58894	-0.31183
218.371	-0.58895	-0.31186
218.387	-0.58897	-0.31189
218.404	-0.58898	-0.31192
218.421	-0.589	-0.31195
218.438	-0.58902	-0.31198
218.454	-0.58905	-0.31202
218.471	-0.58908	-0.31205
218.487	-0.58911	-0.31208
218.504	-0.58913	-0.31213
218.521	-0.58915	-0.31217
218.537	-0.58917	-0.31221

218.554	-0.58919	-0.31225
218.571	-0.5892	-0.31231
218.587	-0.58922	-0.31235
218.604	-0.58923	-0.31238
218.621	-0.58925	-0.31241
218.637	-0.58926	-0.31244
218.654	-0.58928	-0.31247
218.67	-0.58929	-0.31251
218.687	-0.58931	-0.31256
218.704	-0.58932	-0.31261
218.72	-0.58933	-0.31267
218.737	-0.58934	-0.31272
218.754	-0.58936	-0.31282
218.771	-0.58937	-0.3129
218.788	-0.5894	-0.31294
218.804	-0.58942	-0.31298
218.821	-0.58946	-0.31301
218.838	-0.58949	-0.31305
218.854	-0.58953	-0.31308
218.871	-0.58958	-0.31311
218.887	-0.5896	-0.31315
218.903	-0.58961	-0.31318
218.921	-0.58962	-0.31321
218.938	-0.58963	-0.31324
218.954	-0.58964	-0.31327
218.971	-0.58965	-0.31331
218.987	-0.58966	-0.31334
219.004	-0.58968	-0.31337
219.02	-0.58969	-0.31344
219.037	-0.58971	-0.31352

219.053	-0.58973	-0.31361
219.07	-0.58975	-0.31367
219.088	-0.58977	-0.31372
219.104	-0.58979	-0.31376
219.121	-0.58981	-0.31379
219.137	-0.58983	-0.31383
219.153	-0.58985	-0.31386
219.17	-0.58987	-0.31389
219.187	-0.58989	-0.31393
219.203	-0.5899	-0.31399
219.22	-0.58992	-0.31406
219.237	-0.58993	-0.31413
219.254	-0.58994	-0.31417
219.27	-0.58996	-0.3142
219.288	-0.58997	-0.31424
219.304	-0.58998	-0.31427
219.321	-0.58999	-0.3143
219.337	-0.59001	-0.31433
219.354	-0.59003	-0.31437
219.37	-0.59004	-0.3144
219.386	-0.59006	-0.31445
219.403	-0.59008	-0.31451
219.42	-0.59009	-0.31458
219.436	-0.5901	-0.31461
219.453	-0.59012	-0.31465
219.47	-0.59013	-0.31468
219.487	-0.59015	-0.31472
219.504	-0.59017	-0.31475
219.52	-0.59019	-0.3148
219.536	-0.59021	-0.31484

219.554	-0.59023	-0.31488
219.57	-0.59025	-0.31492
219.586	-0.59027	-0.31499
219.603	-0.59029	-0.31508
219.62	-0.5903	-0.31515
219.636	-0.59032	-0.3152
219.653	-0.59033	-0.31527
219.669	-0.59035	-0.31533
219.687	-0.59036	-0.31538
219.703	-0.59037	-0.31542
219.72	-0.59037	-0.31545
219.737	-0.59037	-0.31549
219.753	-0.59038	-0.31552
219.769	-0.59038	-0.31557
219.786	-0.59038	-0.31561
219.803	-0.59038	-0.31566
219.82	-0.59039	-0.31571
219.836	-0.5904	-0.31575
219.853	-0.59041	-0.31578
219.87	-0.59042	-0.31583
219.886	-0.59044	-0.31587
219.904	-0.59045	-0.3159
219.92	-0.59047	-0.31594
219.936	-0.59048	-0.31599
219.953	-0.59048	-0.31604
219.97	-0.59049	-0.31608
219.987	-0.5905	-0.31612
220.003	-0.59051	-0.31615
220.02	-0.59052	-0.31619
220.036	-0.59054	-0.31622

220.053	-0.59056	-0.31626
220.069	-0.59058	-0.31629
220.086	-0.59061	-0.31633
220.102	-0.59063	-0.31637
220.119	-0.59065	-0.31646
220.136	-0.59066	-0.31661
220.153	-0.59068	-0.31673
220.17	-0.59069	-0.31684
220.186	-0.59071	-0.31693
220.203	-0.59073	-0.317
220.22	-0.59075	-0.31708
220.237	-0.59077	-0.31713
220.253	-0.59079	-0.31716
220.27	-0.59081	-0.3172
220.287	-0.59083	-0.31723
220.303	-0.59085	-0.31726
220.32	-0.59087	-0.3173
220.336	-0.59088	-0.31733
220.353	-0.59089	-0.31737
220.37	-0.59091	-0.3174
220.387	-0.59091	-0.31744
220.403	-0.59092	-0.31748
220.42	-0.59093	-0.31752
220.437	-0.59094	-0.31756
220.453	-0.59095	-0.3176
220.47	-0.59096	-0.31763
220.487	-0.59098	-0.31767
220.503	-0.59099	-0.31773
220.52	-0.591	-0.31781
220.536	-0.59101	-0.31791

220.552	-0.59102	-0.31797
220.57	-0.59103	-0.31802
220.586	-0.59104	-0.31806
220.603	-0.59106	-0.3181
220.62	-0.59107	-0.31814
220.636	-0.59109	-0.31817
220.652	-0.59111	-0.31823
220.67	-0.59113	-0.31826
220.686	-0.59115	-0.3183
220.703	-0.59117	-0.31833
220.718	-0.59118	-0.31837
220.736	-0.5912	-0.31842
220.752	-0.59122	-0.31847
220.769	-0.59125	-0.31852
220.786	-0.59128	-0.31856
220.803	-0.59134	-0.3186
220.82	-0.59139	-0.31865
220.836	-0.59141	-0.31869
220.853	-0.59143	-0.31874
220.87	-0.59145	-0.31877
220.886	-0.59146	-0.31882
220.903	-0.59147	-0.31887
220.92	-0.59148	-0.3189
220.936	-0.5915	-0.31894
220.953	-0.59151	-0.31897
220.969	-0.59153	-0.31901
220.986	-0.59155	-0.31905
221.003	-0.59157	-0.31909
221.019	-0.59158	-0.31913
221.036	-0.5916	-0.31917

221.053	-0.59162	-0.31921
221.069	-0.59164	-0.31924
221.086	-0.59166	-0.31928
221.102	-0.59168	-0.31931
221.119	-0.5917	-0.31935
221.136	-0.59172	-0.31939
221.153	-0.59174	-0.31944
221.169	-0.59176	-0.31951
221.186	-0.59177	-0.31959
221.202	-0.59178	-0.31966
221.219	-0.5918	-0.3197
221.236	-0.59181	-0.31974
221.252	-0.59183	-0.31978
221.269	-0.59185	-0.31982
221.286	-0.59187	-0.31986
221.302	-0.59189	-0.31989
221.319	-0.59191	-0.31993
221.336	-0.59192	-0.31997
221.353	-0.59194	-0.32001
221.37	-0.59195	-0.32005
221.385	-0.59197	-0.32012
221.403	-0.59198	-0.32017
221.419	-0.592	-0.32022
221.436	-0.59202	-0.32027
221.453	-0.59204	-0.32032
221.469	-0.59206	-0.32038
221.486	-0.59208	-0.32042
221.504	-0.5921	-0.32046
221.52	-0.59213	-0.32052
221.537	-0.59215	-0.32056

221.553	-0.59217	-0.3206
221.569	-0.59219	-0.32064
221.587	-0.59221	-0.32067
221.603	-0.59223	-0.32071
221.62	-0.59225	-0.32075
221.637	-0.59226	-0.32079
221.653	-0.59228	-0.32083
221.67	-0.5923	-0.32087
221.686	-0.59232	-0.32092
221.704	-0.59234	-0.32097
221.72	-0.59237	-0.32102
221.737	-0.59239	-0.32108
221.754	-0.59241	-0.32114
221.769	-0.59243	-0.32117
221.786	-0.59245	-0.32121
221.802	-0.59247	-0.32126
221.82	-0.59249	-0.3213
221.836	-0.5925	-0.32134
221.852	-0.59252	-0.32137
221.869	-0.59254	-0.3214
221.885	-0.59256	-0.32144
221.902	-0.59257	-0.32147
221.919	-0.59259	-0.32151
221.936	-0.5926	-0.32155
221.952	-0.59262	-0.32159
221.968	-0.59264	-0.32163
221.985	-0.59265	-0.32168
222.002	-0.59267	-0.32173
222.019	-0.59269	-0.32178
222.035	-0.59271	-0.32183

222.053	-0.59273	-0.32187
222.069	-0.59275	-0.32191
222.085	-0.59277	-0.32194
222.102	-0.59279	-0.32198
222.118	-0.59281	-0.32201
222.135	-0.59283	-0.32205
222.151	-0.59285	-0.32208
222.168	-0.59288	-0.32212
222.185	-0.5929	-0.32215
222.202	-0.59292	-0.32219
222.218	-0.59294	-0.32223
222.235	-0.59296	-0.32227
222.251	-0.59298	-0.32231
222.268	-0.593	-0.32235
222.285	-0.59301	-0.32239
222.302	-0.59303	-0.32243
222.319	-0.59305	-0.32247
222.335	-0.59307	-0.3225
222.352	-0.59308	-0.32254
222.368	-0.59309	-0.32258
222.384	-0.59311	-0.32262
222.401	-0.59312	-0.32267
222.418	-0.59313	-0.32271
222.435	-0.59315	-0.32275
222.452	-0.59316	-0.32279
222.467	-0.59318	-0.32283
222.485	-0.5932	-0.32288
222.501	-0.59322	-0.32292
222.517	-0.59324	-0.32296
222.535	-0.59325	-0.323

222.551	-0.59327	-0.32304
222.568	-0.5933	-0.32307
222.584	-0.59332	-0.32311
222.601	-0.59334	-0.32315
222.618	-0.59336	-0.32319
222.634	-0.59338	-0.32323
222.651	-0.5934	-0.32327
222.668	-0.59342	-0.32331
222.685	-0.59344	-0.32335
222.701	-0.59346	-0.32338
222.718	-0.59347	-0.32342
222.734	-0.5935	-0.32345
222.751	-0.59352	-0.32349
222.767	-0.59354	-0.32352
222.784	-0.59356	-0.32356
222.801	-0.59359	-0.3236
222.817	-0.59361	-0.32364
222.835	-0.59362	-0.32367
222.851	-0.59364	-0.32371
222.867	-0.59365	-0.32375
222.885	-0.59366	-0.32379
222.901	-0.59367	-0.32382
222.917	-0.59368	-0.32386
222.934	-0.59369	-0.3239
222.95	-0.5937	-0.32394
222.967	-0.59371	-0.32397
222.984	-0.59372	-0.32401
223.001	-0.59373	-0.32405
223.017	-0.59374	-0.32409
223.034	-0.59374	-0.32413

223.05	-0.59375	-0.32418
223.067	-0.59376	-0.32422
223.084	-0.59378	-0.32426
223.101	-0.59379	-0.3243
223.117	-0.59381	-0.32434
223.135	-0.59383	-0.32437
223.149	-0.59384	-0.32441
223.166	-0.59386	-0.32445
223.184	-0.59388	-0.32449
223.2	-0.5939	-0.32452
223.217	-0.59392	-0.32456
223.234	-0.59396	-0.3246
223.251	-0.59402	-0.32464
223.267	-0.59407	-0.32468
223.284	-0.59413	-0.32472
223.3	-0.59418	-0.32475
223.317	-0.59423	-0.32479
223.334	-0.59426	-0.32483
223.35	-0.59428	-0.32487
223.368	-0.5943	-0.3249
223.384	-0.59432	-0.32494
223.4	-0.59433	-0.32498
223.417	-0.59434	-0.32502
223.434	-0.59436	-0.32506
223.45	-0.59438	-0.32509
223.468	-0.5944	-0.32513
223.483	-0.59442	-0.32517
223.5	-0.59444	-0.32521
223.518	-0.59446	-0.32525
223.534	-0.59448	-0.32529

223.551	-0.5945	-0.32533
223.567	-0.59452	-0.32537
223.584	-0.59454	-0.32541
223.601	-0.59456	-0.32546
223.617	-0.59459	-0.32549
223.634	-0.59461	-0.32553
223.65	-0.59463	-0.32557
223.667	-0.59465	-0.32561
223.684	-0.59467	-0.32564
223.701	-0.59469	-0.32568
223.718	-0.59471	-0.32571
223.734	-0.59472	-0.32574
223.751	-0.59474	-0.32578
223.768	-0.59476	-0.32582
223.785	-0.59478	-0.32585
223.8	-0.5948	-0.32589
223.817	-0.59481	-0.32597
223.834	-0.59483	-0.32605
223.85	-0.59485	-0.32613
223.867	-0.59487	-0.32617
223.884	-0.59489	-0.32621
223.9	-0.59491	-0.32625
223.917	-0.59493	-0.32629
223.934	-0.59495	-0.32633
223.951	-0.59497	-0.32636
223.968	-0.59499	-0.3264
223.984	-0.59501	-0.32644
224.001	-0.59503	-0.32647
224.017	-0.59505	-0.32651
224.035	-0.59508	-0.32655

224.051	-0.5951	-0.32659
224.068	-0.59513	-0.32662
224.084	-0.59515	-0.32666
224.101	-0.59518	-0.3267
224.117	-0.5952	-0.32673
224.134	-0.59522	-0.32677
224.151	-0.59525	-0.3268
224.168	-0.59527	-0.32684
224.184	-0.59529	-0.32687
224.201	-0.59531	-0.32691
224.217	-0.59534	-0.32694
224.234	-0.59536	-0.32698
224.251	-0.59539	-0.32701
224.268	-0.59542	-0.32705
224.284	-0.59544	-0.32708
224.301	-0.59547	-0.32711
224.318	-0.59549	-0.32715
224.334	-0.59551	-0.32718
224.351	-0.59553	-0.32722
224.368	-0.59555	-0.32725
224.384	-0.59557	-0.32728
224.4	-0.5956	-0.32732
224.418	-0.59563	-0.32735
224.434	-0.59565	-0.32738
224.451	-0.59568	-0.32742
224.468	-0.5957	-0.32745
224.484	-0.59572	-0.32749
224.501	-0.59574	-0.32752
224.517	-0.59577	-0.32755
224.535	-0.59579	-0.32758

224.551	-0.59581	-0.32761
224.568	-0.59583	-0.32765
224.584	-0.59586	-0.32768
224.601	-0.59588	-0.32771
224.618	-0.5959	-0.32774
224.634	-0.59592	-0.32777
224.651	-0.59594	-0.32781
224.667	-0.59596	-0.32784
224.685	-0.59598	-0.32787
224.701	-0.59599	-0.3279
224.718	-0.596	-0.32793
224.735	-0.59602	-0.32797
224.751	-0.59603	-0.32799
224.767	-0.59605	-0.32802
224.784	-0.59607	-0.32805
224.802	-0.59608	-0.32808
224.818	-0.59609	-0.32811
224.835	-0.59611	-0.32814
224.852	-0.59612	-0.32817
224.868	-0.59614	-0.3282
224.884	-0.59615	-0.32823
224.901	-0.59618	-0.32826
224.918	-0.5962	-0.32829
224.934	-0.59623	-0.32832
224.951	-0.59628	-0.32835
224.967	-0.59631	-0.32838
224.984	-0.59635	-0.32841
225	-0.59638	-0.32843
225.017	-0.5964	-0.32846
225.033	-0.59642	-0.32849

225.051	-0.59644	-0.32852
225.068	-0.59645	-0.32855
225.084	-0.59646	-0.32858
225.101	-0.59647	-0.32861
225.118	-0.59649	-0.32864
225.133	-0.5965	-0.32867
225.151	-0.59651	-0.3287
225.167	-0.59653	-0.32872
225.184	-0.59655	-0.32875
225.201	-0.59657	-0.32877
225.217	-0.59659	-0.32879
225.234	-0.59661	-0.32881
225.251	-0.59663	-0.32884
225.267	-0.59666	-0.32886
225.285	-0.59668	-0.32889
225.301	-0.59671	-0.32891
225.318	-0.59674	-0.32894
225.334	-0.59676	-0.32897
225.351	-0.59679	-0.32899
225.368	-0.59681	-0.32902
225.384	-0.59684	-0.32905
225.4	-0.59686	-0.32908
225.418	-0.59688	-0.32911
225.433	-0.5969	-0.32913
225.451	-0.59693	-0.32916
225.468	-0.59695	-0.32918
225.485	-0.59698	-0.32921
225.501	-0.59702	-0.32923
225.517	-0.59705	-0.32926
225.535	-0.59707	-0.32929

225.551	-0.5971	-0.32932
225.568	-0.59714	-0.32934
225.585	-0.59716	-0.32937
225.6	-0.59719	-0.32939
225.617	-0.59721	-0.32942
225.634	-0.59723	-0.32944
225.651	-0.59725	-0.32946
225.667	-0.59727	-0.32948
225.684	-0.59729	-0.32951
225.701	-0.5973	-0.32953
225.717	-0.59732	-0.32955
225.734	-0.59735	-0.32957
225.751	-0.59737	-0.32959
225.768	-0.5974	-0.32962
225.783	-0.59742	-0.32964
225.8	-0.59744	-0.32967
225.818	-0.59746	-0.32969
225.834	-0.59748	-0.32971
225.852	-0.59749	-0.32973
225.867	-0.59751	-0.32975
225.884	-0.59752	-0.32977
225.901	-0.59754	-0.32979
225.917	-0.59756	-0.32981
225.934	-0.59758	-0.32983
225.95	-0.5976	-0.32985
225.967	-0.59763	-0.32988
225.984	-0.59765	-0.3299
226	-0.59767	-0.32992
226.018	-0.59769	-0.32994
226.034	-0.59771	-0.32996

226.051	-0.59773	-0.32998
226.067	-0.59775	-0.33
226.084	-0.59777	-0.33002
226.101	-0.59779	-0.33004
226.117	-0.59781	-0.33006
226.135	-0.59783	-0.33007
226.151	-0.59785	-0.33009
226.167	-0.59786	-0.3301
226.184	-0.59787	-0.33012
226.2	-0.59789	-0.33014
226.217	-0.5979	-0.33015
226.234	-0.59792	-0.33017
226.25	-0.59793	-0.33018
226.266	-0.59795	-0.3302
226.284	-0.59797	-0.33021
226.3	-0.59799	-0.33022
226.317	-0.59801	-0.33023
226.334	-0.59803	-0.33025
226.35	-0.59804	-0.33026
226.367	-0.59806	-0.33027
226.383	-0.59808	-0.33028
226.4	-0.59809	-0.3303
226.417	-0.59811	-0.33031
226.433	-0.59813	-0.33032
226.449	-0.59814	-0.33033
226.466	-0.59816	-0.33034
226.484	-0.59818	-0.33035
226.5	-0.5982	-0.33036
226.517	-0.59823	-0.33037
226.534	-0.59825	-0.33039

226.551	-0.59827	-0.3304
226.567	-0.59829	-0.3304
226.583	-0.59831	-0.33041
226.6	-0.59833	-0.33042
226.617	-0.59835	-0.33043
226.633	-0.59836	-0.33044
226.65	-0.59838	-0.33045
226.667	-0.59841	-0.33046
226.684	-0.59843	-0.33047
226.701	-0.59845	-0.33048
226.717	-0.59848	-0.33049
226.734	-0.5985	-0.3305
226.75	-0.59853	-0.33051
226.767	-0.59856	-0.33052
226.783	-0.59859	-0.33052
226.8	-0.59861	-0.33053
226.818	-0.59864	-0.33054
226.833	-0.59866	-0.33054
226.85	-0.59869	-0.33054
226.867	-0.59871	-0.33055
226.884	-0.59874	-0.33054
226.9	-0.59876	-0.33054
226.917	-0.59878	-0.33055
226.934	-0.5988	-0.33055
226.95	-0.59883	-0.33055
226.967	-0.59885	-0.33056
226.984	-0.59887	-0.33056
227	-0.5989	-0.33057
227.017	-0.59893	-0.33057
227.034	-0.59895	-0.33058

227.05	-0.59899	-0.33059
227.067	-0.59902	-0.3306
227.084	-0.59906	-0.3306
227.101	-0.5991	-0.33061
227.118	-0.59914	-0.33062
227.135	-0.59917	-0.33062
227.152	-0.59922	-0.33062
227.168	-0.59925	-0.33062
227.185	-0.59928	-0.33062
227.201	-0.59931	-0.33062
227.218	-0.59934	-0.33061
227.235	-0.59937	-0.33061
227.251	-0.59939	-0.33059
227.268	-0.59942	-0.33058
227.285	-0.59944	-0.33057
227.301	-0.59947	-0.33057
227.318	-0.59949	-0.33057
227.335	-0.59951	-0.33057
227.352	-0.59954	-0.33057
227.368	-0.59957	-0.33057
227.385	-0.5996	-0.33057
227.402	-0.59963	-0.33057
227.419	-0.59965	-0.33056
227.435	-0.59967	-0.33055
227.451	-0.59969	-0.33052
227.469	-0.59972	-0.33049
227.486	-0.59974	-0.33047
227.503	-0.59976	-0.33044
227.519	-0.59979	-0.33043
227.536	-0.59981	-0.33041

227.553	-0.59983	-0.3304
227.57	-0.59985	-0.33038
227.587	-0.59987	-0.33037
227.603	-0.59989	-0.33035
227.619	-0.59991	-0.33032
227.636	-0.59992	-0.33028
227.653	-0.59994	-0.33025
227.67	-0.59997	-0.33022
227.687	-0.59999	-0.33017
227.703	-0.60001	-0.33013
227.719	-0.60003	-0.33008
227.737	-0.60005	-0.33005
227.754	-0.60007	-0.33
227.77	-0.60008	-0.32994
227.786	-0.6001	-0.32985
227.804	-0.60011	-0.32975
227.82	-0.60012	-0.32964
227.836	-0.60013	-0.32951
227.853	-0.60015	-0.3294
227.87	-0.60017	-0.32928
227.887	-0.60019	-0.32919
227.904	-0.60021	-0.32913
227.92	-0.60023	-0.32909
227.936	-0.60026	-0.32906
227.953	-0.60028	-0.32899
227.971	-0.60029	-0.32889
227.987	-0.60031	-0.32879
228.003	-0.60033	-0.32867
228.02	-0.60035	-0.3285
228.037	-0.60037	-0.32831

228.053	-0.60039	-0.32807
228.071	-0.60042	-0.32784
228.087	-0.60044	-0.32765
228.104	-0.60047	-0.32748
228.121	-0.60049	-0.32732
228.138	-0.60052	-0.32713
228.155	-0.60054	-0.32684

Figure 11

2 Theta	SA	L-SA
5	0.10667	0.89
5.02	0.10667	0.89
5.04	0.10667	0.89
5.06	0.10667	0.44
5.08	0.10667	0
5.1	0.12667	0
5.12	0.15333	0.28
5.14	0.17333	0.3
5.16	0.16	0.27
5.18	0.13333	0.18
5.2	0.1	0.06
5.22	0.06	0
5.24	0.03333	0
5.26	0.02	0.02
5.28	0.00667	0.02
5.3	0.00667	0
5.32	0	0.04
5.34	0.01333	0.16
5.36	0.04	0.26
5.38	0.06	0.28

5.4	0.08	0.27
5.42	0.08667	0.23
5.44	0.07333	0.17
5.46	0.05333	0.04
5.48	0.02667	0.09
5.5	0.00667	0.11
5.52	0	0.08
5.54	0.01333	0.03
5.56	0.04667	0
5.58	0.08	0
5.6	0.11333	0
5.62	0.12667	0.1
5.64	0.11333	0.18
5.66	0.08	0.23
5.68	0.04667	0.2
5.7	0.02	0.18
5.72	0	0.16
5.74	0	0.14
5.76	0	0.15
5.78	0	0.22
5.8	0	0.2
5.82	0	0.14
5.84	0	0.04
5.86	0	0
5.88	0.00667	0
5.9	0.02	0.02
5.92	0.04	0.06
5.94	0.05333	0.08
5.96	0.06	0.09
5.98	0.06667	0.08

6	0.05333	0.01
6.02	0.02667	0.05
6.04	0.00667	0.03
6.06	0.00667	0.1
6.08	0.04667	0.24
6.1	0.12	0.31
6.12	0.20667	0.29
6.14	0.28667	0.24
6.16	0.30667	0.25
6.18	0.26667	0.29
6.2	0.18	0.23
6.22	0.09333	0.21
6.24	0.03333	0.21
6.26	0.01333	0.11
6.28	0.02667	0.08
6.3	0.06667	0.08
6.32	0.09333	0.16
6.34	0.12667	0.25
6.36	0.15333	0.34
6.38	0.17333	0.34
6.4	0.18667	0.27
6.42	0.17333	0.17
6.44	0.12667	0.13
6.46	0.08667	0.04
6.48	0.04	0
6.5	0.02	0.01
6.52	0.02667	0
6.54	0.05333	0.1
6.56	0.08	0.14
6.58	0.09333	0.21

6.6	0.10667	0.23
6.62	0.12	0.12
6.64	0.12667	0.24
6.66	0.16	0.27
6.68	0.18667	0.24
6.7	0.24	0.35
6.72	0.27333	0.35
6.74	0.3	0.34
6.76	0.36	0.18
6.78	0.35333	0.07
6.8	0.34	0.08
6.82	0.33333	0.19
6.84	0.3	0.32
6.86	0.29333	0.41
6.88	0.30667	0.41
6.9	0.36667	0.44
6.92	0.48667	0.44
6.94	0.55333	0.48
6.96	0.62	0.23
6.98	0.62	0.44
7	0.56	0.61
7.02	0.45333	0.62
7.04	0.32667	0.56
7.06	0.22	0.51
7.08	0.16	0.49
7.1	0.09333	0.37
7.12	0.09333	0.12
7.14	0.12667	0.37
7.16	0.19333	0.49
7.18	0.29333	0.48

7.2	0.40667	0.58
7.22	0.49333	0.64
7.24	0.55333	0.68
7.26	0.58667	0.76
7.28	0.62667	0.64
7.3	0.66	0.73
7.32	0.67333	0.67
7.34	0.66667	0.42
7.36	0.62667	0.47
7.38	0.55333	0.51
7.4	0.46667	0.44
7.42	0.38	0.67
7.44	0.33333	0.51
7.46	0.32	0.64
7.48	0.36667	0.84
7.5	0.46	0.64
7.52	0.54	0.85
7.54	0.62	0.72
7.56	0.64	0.64
7.58	0.59333	0.78
7.6	0.54667	0.62
7.62	0.54	0.8
7.64	0.59333	1.14
7.66	0.69333	0.93
7.68	0.73333	1.24
7.7	0.72	1.29
7.72	0.59333	1.41
7.74	0.45333	1.55
7.76	0.35333	1.65
7.78	0.32667	1.69

7.8	0.38667	1.94
7.82	0.50667	1.98
7.84	0.6	2.22
7.86	0.61333	2.45
7.88	0.56667	2.27
7.9	0.54667	2.6
7.92	0.46667	2.74
7.94	0.42667	2.9
7.96	0.42	3.39
7.98	0.47333	3.78
8	0.54	4.1
8.02	0.58	4.61
8.04	0.60667	4.67
8.06	0.66	5.09
8.08	0.64667	5.51
8.1	0.68	5.88
8.12	0.72	6.25
8.14	0.76	6.38
8.16	0.79333	6.42
8.18	0.77333	6.64
8.2	0.7	6.87
8.22	0.62667	6.77
8.24	0.53333	6.99
8.26	0.47333	7.01
8.28	0.47333	6.99
8.3	0.47333	7.02
8.32	0.52	6.82
8.34	0.61333	6.78
8.36	0.7	6.96
8.38	0.78667	6.5

8.4	0.81333	5.95
8.42	0.82	5.51
8.44	0.81333	4.96
8.46	0.72667	4.76
8.48	0.61333	4.3
8.5	0.54667	4.09
8.52	0.47333	3.96
8.54	0.44	3.74
8.56	0.43333	3.37
8.58	0.51333	3.26
8.6	0.62667	3.01
8.62	0.7	2.79
8.64	0.71333	2.42
8.66	0.7	2.16
8.68	0.61333	2.05
8.7	0.52	1.92
8.72	0.42667	1.86
8.74	0.37333	1.92
8.76	0.35333	1.83
8.78	0.35333	1.72
8.8	0.37333	1.64
8.82	0.41333	1.32
8.84	0.47333	1.02
8.86	0.57333	0.73
8.88	0.66	0.6
8.9	0.7	0.48
8.92	0.67333	0.55
8.94	0.60667	0.55
8.96	0.48	0.62
8.98	0.34667	0.58

9	0.23333	0.58
9.02	0.17333	0.58
9.04	0.18	0.53
9.06	0.23333	0.58
9.08	0.30667	0.76
9.1	0.40667	0.58
9.12	0.48667	0.6
9.14	0.54	0.59
9.16	0.51333	0.57
9.18	0.45333	0.36
9.2	0.38	0.34
9.22	0.32	0.4
9.24	0.26	0.42
9.26	0.24	0.23
9.28	0.22667	0.23
9.3	0.20667	0.19
9.32	0.17333	0.25
9.34	0.14667	0.4
9.36	0.12	0.56
9.38	0.14	0.61
9.4	0.16	0.55
9.42	0.22	0.37
9.44	0.29333	0.2
9.46	0.34	0.03
9.48	0.38	0
9.5	0.38667	0.04
9.52	0.36	0.06
9.54	0.34	0.13
9.56	0.28	0.18
9.58	0.22667	0.31

9.6	0.18667	0.39
9.62	0.14667	0.4
9.64	0.10667	0.5
9.66	0.1	0.45
9.68	0.11333	0.44
9.7	0.16	0.4
9.72	0.19333	0.22
9.74	0.24667	0.22
9.76	0.26667	0.2
9.78	0.27333	0.21
9.8	0.26	0.18
9.82	0.24667	0.25
9.84	0.23333	0.3
9.86	0.23333	0.41
9.88	0.26	0.44
9.9	0.30667	0.53
9.92	0.31333	0.52
9.94	0.32667	0.39
9.96	0.32667	0.18
9.98	0.26667	0.2
10	0.21333	0.09
10.02	0.17333	0.04
10.04	0.14	0.15
10.06	0.11333	0.32
10.08	0.10667	0.48
10.1	0.12	0.51
10.12	0.14667	0.5
10.14	0.18	0.46
10.16	0.25333	0.28
10.18	0.31333	0

10.2	0.36667	0
10.22	0.34667	0
10.24	0.28667	0.14
10.26	0.22667	0.22
10.28	0.21333	0.26
10.3	0.22667	0.2
10.32	0.28667	0.25
10.34	0.35333	0.24
10.36	0.39333	0.21
10.38	0.35333	0.1
10.4	0.30667	0.18
10.42	0.27333	0.11
10.44	0.27333	0.1
10.46	0.24667	0.03
10.48	0.23333	0.15
10.5	0.20667	0.17
10.52	0.16	0.16
10.54	0.12	0.11
10.56	0.10667	0.08
10.58	0.10667	0
10.6	0.11333	0.01
10.62	0.15333	0.03
10.64	0.2	0.09
10.66	0.24667	0.09
10.68	0.3	0.11
10.7	0.35333	0.11
10.72	0.34667	0.09
10.74	0.26667	0
10.76	0.19333	0.05
10.78	0.16667	0.13

10.8	0.15333	0.16
10.82	0.18	0.12
10.84	0.25333	0.18
10.86	0.31333	0.13
10.88	0.36667	0.17
10.9	0.36	0.13
10.92	0.36667	0.18
10.94	0.41333	0.24
10.96	0.42	0.29
10.98	0.44667	0.24
11	0.48	0.22
11.02	0.5	0.09
11.04	0.51333	0.29
11.06	0.48	0.21
11.08	0.46	0.36
11.1	0.45333	0.59
11.12	0.43333	0.81
11.14	0.44	0.89
11.16	0.44667	0.85
11.18	0.46667	0.78
11.2	0.44	1.02
11.22	0.36667	1.09
11.24	0.32	1.33
11.26	0.31333	1.41
11.28	0.35333	1.56
11.3	0.44667	1.81
11.32	0.54	1.95
11.34	0.62667	2.11
11.36	0.64	2.29
11.38	0.57333	2.55

11.4	0.50667	2.79
11.42	0.44	2.71
11.44	0.44667	2.9
11.46	0.46667	3.15
11.48	0.5	3.07
11.5	0.52667	3.04
11.52	0.55333	3.04
11.54	0.54	3.08
11.56	0.50667	3.13
11.58	0.46667	3.08
11.6	0.47333	3.2
11.62	0.44667	3.23
11.64	0.44	3.13
11.66	0.45333	3.14
11.68	0.49333	3.21
11.7	0.54667	2.98
11.72	0.56667	2.82
11.74	0.56667	2.72
11.76	0.56667	2.53
11.78	0.50667	2.47
11.8	0.45333	2.46
11.82	0.40667	2.16
11.84	0.4	2
11.86	0.45333	1.93
11.88	0.48	1.81
11.9	0.54	1.76
11.92	0.6	1.73
11.94	0.60667	1.95
11.96	0.58	2.2
11.98	0.52667	2.14

12	0.49333	2.01
12.02	0.48667	1.96
12.04	0.46	1.82
12.06	0.48	1.57
12.08	0.49333	1.42
12.1	0.54	1.53
12.12	0.6	1.58
12.14	0.67333	1.77
12.16	0.77333	1.67
12.18	0.86	1.64
12.2	0.87333	1.8
12.22	0.81333	1.71
12.24	0.72667	1.6
12.26	0.64667	1.66
12.28	0.56667	1.62
12.3	0.52667	1.64
12.32	0.57333	1.46
12.34	0.63333	1.44
12.36	0.69333	1.36
12.38	0.74	1.24
12.4	0.82667	1.17
12.42	0.88667	1.26
12.44	0.88667	1.29
12.46	0.86	1.18
12.48	0.86	1.13
12.5	0.80667	1.27
12.52	0.72667	1.24
12.54	0.65333	1.41
12.56	0.66667	1.57
12.58	0.67333	1.53

12.6	0.65333	1.46
12.62	0.66667	1.35
12.64	0.72667	1.2
12.66	0.76667	1.12
12.68	0.78667	0.97
12.7	0.78	0.87
12.72	0.82	0.8
12.74	0.83333	0.7
12.76	0.82	0.71
12.78	0.80667	0.65
12.8	0.81333	0.61
12.82	0.84667	0.7
12.84	0.85333	0.71
12.86	0.81333	0.75
12.88	0.78	0.71
12.9	0.75333	0.65
12.92	0.7	0.57
12.94	0.64	0.5
12.96	0.63333	0.48
12.98	0.69333	0.55
13	0.77333	0.56
13.02	0.84667	0.57
13.04	0.90667	0.34
13.06	0.95333	0.22
13.08	0.94	0.18
13.1	0.88	0.27
13.12	0.83333	0.24
13.14	0.78	0.29
13.16	0.75333	0.27
13.18	0.71333	0.31

13.2	0.68	0.22
13.22	0.68	0.2
13.24	0.68	0.23
13.26	0.73333	0.26
13.28	0.80667	0.22
13.3	0.88667	0.29
13.32	0.98667	0.23
13.34	1.04	0.24
13.36	1.07333	0.14
13.38	1.07333	0.07
13.4	1.00667	0.03
13.42	0.93333	0.01
13.44	0.82667	0
13.46	0.69333	0
13.48	0.63333	0
13.5	0.62	0
13.52	0.68667	0.1
13.54	0.8	0.13
13.56	0.90667	0.26
13.58	1.04	0.34
13.6	1.11333	0.4
13.62	1.10667	0.39
13.64	1.09333	0.27
13.66	1.07333	0.2
13.68	1.06	0.19
13.7	1.02667	0.12
13.72	1.00667	0.14
13.74	1.04667	0.12
13.76	1.09333	0.08
13.78	1.12	0.1

13.8	1.12667	0.07
13.82	1.12	0.04
13.84	1.06667	0.08
13.86	0.92	0.08
13.88	0.78667	0.06
13.9	0.71333	0.14
13.92	0.73333	0.29
13.94	0.80667	0.43
13.96	0.91333	0.4
13.98	1.00667	0.37
14	1.05333	0.39
14.02	1.04667	0.34
14.04	1.04667	0.26
14.06	1.03333	0.31
14.08	1.07333	0.35
14.1	1.13333	0.29
14.12	1.15333	0.17
14.14	1.12	0.08
14.16	1.02667	0.06
14.18	0.96667	0.13
14.2	0.98	0.24
14.22	0.97333	0.37
14.24	0.98	0.45
14.26	1.01333	0.47
14.28	0.99333	0.36
14.3	0.93333	0.25
14.32	0.84	0.17
14.34	0.79333	0.07
14.36	0.80667	0.12
14.38	0.78667	0.16

14.4	0.74667	0.21
14.42	0.72667	0.18
14.44	0.66667	0.08
14.46	0.60667	0.17
14.48	0.58	0.23
14.5	0.6	0.24
14.52	0.68667	0.29
14.54	0.76667	0.35
14.56	0.83333	0.33
14.58	0.84667	0.24
14.6	0.76667	0.13
14.62	0.62667	0.2
14.64	0.50667	0.27
14.66	0.44	0.24
14.68	0.47333	0.22
14.7	0.53333	0.24
14.72	0.6	0.22
14.74	0.64667	0.22
14.76	0.66	0.21
14.78	0.62667	0.25
14.8	0.58	0.22
14.82	0.54	0.12
14.84	0.51333	0.08
14.86	0.44667	0.02
14.88	0.36667	0.04
14.9	0.33333	0.11
14.92	0.30667	0.16
14.94	0.30667	0.08
14.96	0.34667	0.19
14.98	0.38667	0.25

15	0.40667	0.25
15.02	0.38	0.21
15.04	0.36667	0.31
15.06	0.38667	0.41
15.08	0.38667	0.37
15.1	0.41333	0.32
15.12	0.45333	0.49
15.14	0.43333	0.5
15.16	0.35333	0.39
15.18	0.24667	0.39
15.2	0.18667	0.51
15.22	0.16667	0.52
15.24	0.18667	0.51
15.26	0.26	0.52
15.28	0.31333	0.48
15.3	0.30667	0.37
15.32	0.26	0.43
15.34	0.22	0.57
15.36	0.21333	0.62
15.38	0.20667	0.5
15.4	0.22	0.48
15.42	0.24	0.49
15.44	0.20667	0.41
15.46	0.16667	0.27
15.48	0.14	0.33
15.5	0.13333	0.4
15.52	0.14	0.34
15.54	0.14	0.3
15.56	0.14	0.48
15.58	0.12	0.66

15.6	0.07333	0.69
15.62	0.04667	0.62
15.64	0.04	0.54
15.66	0.06	0.57
15.68	0.09333	0.44
15.7	0.14667	0.45
15.72	0.18	0.49
15.74	0.18	0.54
15.76	0.14	0.75
15.78	0.11333	0.83
15.8	0.11333	0.76
15.82	0.12	0.87
15.84	0.12	0.87
15.86	0.13333	0.76
15.88	0.13333	0.72
15.9	0.11333	0.79
15.92	0.10667	0.82
15.94	0.14	0.78
15.96	0.19333	0.83
15.98	0.22667	0.93
16	0.22667	0.94
16.02	0.22667	0.96
16.04	0.17333	1.08
16.06	0.12667	1.02
16.08	0.11333	0.88
16.1	0.12	0.95
16.12	0.12667	1.06
16.14	0.13333	1.09
16.16	0.14	1.11
16.18	0.14	1.21

16.2	0.11333	1.29
16.22	0.09333	1.45
16.24	0.08667	1.49
16.26	0.08	1.45
16.28	0.09333	1.57
16.3	0.11333	1.7
16.32	0.13333	1.63
16.34	0.12667	1.61
16.36	0.09333	1.82
16.38	0.06667	2.1
16.4	0.06	2.25
16.42	0.07333	2.23
16.44	0.12	2.4
16.46	0.18667	2.47
16.48	0.24667	2.19
16.5	0.25333	2.08
16.52	0.20667	2.13
16.54	0.14	2.07
16.56	0.08667	2.3
16.58	0.04667	2.45
16.6	0.04667	2.51
16.62	0.07333	2.56
16.64	0.11333	2.44
16.66	0.14	2.4
16.68	0.14	2.35
16.7	0.12	2.37
16.72	0.10667	2.63
16.74	0.1	2.87
16.76	0.10667	2.86
16.78	0.10667	2.88

16.8	0.10667	2.94
16.82	0.13333	3.12
16.84	0.16667	3.25
16.86	0.16667	3.47
16.88	0.18	3.62
16.9	0.2	3.68
16.92	0.17333	3.62
16.94	0.14	3.58
16.96	0.12667	3.57
16.98	0.14	3.66
17	0.18667	3.7
17.02	0.19333	3.72
17.04	0.2	3.68
17.06	0.20667	3.76
17.08	0.15333	3.99
17.1	0.11333	4.21
17.12	0.09333	4.33
17.14	0.12	4.27
17.16	0.16	4.2
17.18	0.16	4.18
17.2	0.17333	4.08
17.22	0.18667	4.2
17.24	0.16667	4.45
17.26	0.16667	4.52
17.28	0.18667	4.6
17.3	0.22667	4.64
17.32	0.24667	4.76
17.34	0.24	4.88
17.36	0.24667	4.99
17.38	0.24667	5.28

17.4	0.22667	5.19
17.42	0.22	4.97
17.44	0.23333	5.12
17.46	0.21333	5.33
17.48	0.16	5.4
17.5	0.10667	5.14
17.52	0.08667	5.02
17.54	0.09333	5.11
17.56	0.12	4.98
17.58	0.17333	5.13
17.6	0.21333	5.58
17.62	0.21333	6.02
17.64	0.16	6.24
17.66	0.10667	6.13
17.68	0.08	5.97
17.7	0.09333	5.96
17.72	0.14	5.95
17.74	0.21333	6.01
17.76	0.28667	6.08
17.78	0.34667	6.24
17.8	0.34	6.27
17.82	0.3	6.01
17.84	0.25333	5.95
17.86	0.22	6.18
17.88	0.18667	6.29
17.9	0.15333	6.21
17.92	0.15333	6.27
17.94	0.20667	6.32
17.96	0.26667	6.45
17.98	0.33333	6.34

18	0.39333	6.53
18.02	0.46	6.76
18.04	0.5	6.71
18.06	0.48667	6.57
18.08	0.46	6.63
18.1	0.43333	6.84
18.12	0.38	7.26
18.14	0.31333	7.22
18.16	0.25333	7.09
18.18	0.22667	7
18.2	0.22667	6.85
18.22	0.24667	6.81
18.24	0.26667	6.72
18.26	0.26667	6.83
18.28	0.24667	6.97
18.3	0.24667	6.96
18.32	0.26	6.89
18.34	0.27333	6.94
18.36	0.31333	6.99
18.38	0.35333	7.08
18.4	0.36	7.09
18.42	0.33333	7.26
18.44	0.30667	7.43
18.46	0.29333	7.74
18.48	0.26	7.78
18.5	0.22667	7.55
18.52	0.22667	7.26
18.54	0.24	6.97
18.56	0.24	6.8
18.58	0.24	6.7

18.6	0.26667	6.77
18.62	0.29333	7.01
18.64	0.31333	7.24
18.66	0.35333	7.26
18.68	0.40667	7.12
18.7	0.45333	7.13
18.72	0.44667	7.29
18.74	0.42667	7.3
18.76	0.42	7.18
18.78	0.38	7.2
18.8	0.34667	7.29
18.82	0.32667	7.07
18.84	0.30667	6.8
18.86	0.27333	6.82
18.88	0.23333	6.92
18.9	0.21333	6.79
18.92	0.21333	6.57
18.94	0.22	6.58
18.96	0.25333	6.63
18.98	0.29333	6.69
19	0.31333	6.87
19.02	0.32	6.99
19.04	0.32	7.17
19.06	0.32667	7.32
19.08	0.34667	7.26
19.1	0.38	7.08
19.12	0.41333	6.88
19.14	0.38667	6.73
19.16	0.32	6.53
19.18	0.21333	6.23

19.2	0.12667	6.26
19.22	0.07333	6.49
19.24	0.09333	6.62
19.26	0.14667	6.64
19.28	0.22	6.53
19.3	0.26667	6.62
19.32	0.28667	6.71
19.34	0.26667	6.78
19.36	0.25333	6.85
19.38	0.26	6.84
19.4	0.28667	6.79
19.42	0.29333	6.69
19.44	0.28667	6.51
19.46	0.24667	6.54
19.48	0.19333	6.72
19.5	0.13333	6.97
19.52	0.10667	7.07
19.54	0.11333	6.94
19.56	0.14	6.84
19.58	0.16667	6.85
19.6	0.19333	6.71
19.62	0.19333	6.7
19.64	0.18	6.88
19.66	0.16667	7.03
19.68	0.15333	7.1
19.7	0.14667	7.05
19.72	0.15333	7.01
19.74	0.18667	6.9
19.76	0.22	6.68
19.78	0.24	6.69

19.8	0.25333	6.78
19.82	0.26	6.72
19.84	0.25333	6.67
19.86	0.24667	6.79
19.88	0.24	6.95
19.9	0.24	6.89
19.92	0.22	6.69
19.94	0.20667	6.68
19.96	0.22667	6.71
19.98	0.25333	6.61
20	0.28667	6.73
20.02	0.29333	6.92
20.04	0.27333	6.99
20.06	0.22	6.83
20.08	0.14667	6.71
20.1	0.09333	6.78
20.12	0.08	6.79
20.14	0.07333	6.93
20.16	0.06667	7.05
20.18	0.06	6.98
20.2	0.06667	6.78
20.22	0.09333	6.71
20.24	0.11333	6.94
20.26	0.13333	7.09
20.28	0.16667	7.16
20.3	0.18	7.13
20.32	0.17333	6.99
20.34	0.17333	6.91
20.36	0.2	7.05
20.38	0.22667	7.21

20.4	0.22667	7.49
20.42	0.20667	7.53
20.44	0.19333	7.56
20.46	0.17333	7.24
20.48	0.15333	7.16
20.5	0.12667	7.29
20.52	0.13333	7.51
20.54	0.16	7.55
20.56	0.16	7.67
20.58	0.16667	7.9
20.6	0.15333	8.02
20.62	0.13333	7.92
20.64	0.09333	7.88
20.66	0.05333	7.89
20.68	0.02667	8.01
20.7	0.03333	8.22
20.72	0.03333	8.36
20.74	0.06	8.59
20.76	0.08667	8.81
20.78	0.12	8.91
20.8	0.13333	9.01
20.82	0.14667	9.07
20.84	0.14	9.29
20.86	0.14	9.53
20.88	0.12	9.6
20.9	0.12667	9.6
20.92	0.13333	9.72
20.94	0.15333	9.85
20.96	0.16667	10.14
20.98	0.18	10.13

21	0.16	10.19
21.02	0.14	10.11
21.04	0.1	9.95
21.06	0.06	9.81
21.08	0.03333	9.77
21.1	0.03333	9.99
21.12	0.06	10.24
21.14	0.10667	10.22
21.16	0.15333	10.18
21.18	0.19333	10.09
21.2	0.18667	9.92
21.22	0.14	9.85
21.24	0.08667	9.95
21.26	0.06	10.14
21.28	0.04667	10.25
21.3	0.06667	10.39
21.32	0.11333	10.24
21.34	0.16	10.5
21.36	0.16	10.32
21.38	0.12667	10.16
21.4	0.08667	10.04
21.42	0.05333	9.96
21.44	0.01333	10.07
21.46	0	10.11
21.48	0.00667	9.9
21.5	0.04	10.19
21.52	0.06667	10.11
21.54	0.08667	10.06
21.56	0.11333	10.04
21.58	0.14	10.17

21.6	0.13333	10.33
21.62	0.12	10.4
21.64	0.12	10.28
21.66	0.13333	10.17
21.68	0.14667	9.95
21.7	0.15333	9.65
21.72	0.16667	9.4
21.74	0.16	9.3
21.76	0.12667	9.2
21.78	0.08667	9.32
21.8	0.06667	9.27
21.82	0.07333	9.13
21.84	0.08667	8.86
21.86	0.10667	8.61
21.88	0.11333	8.37
21.9	0.1	8.25
21.92	0.08	8.11
21.94	0.06667	8.19
21.96	0.08	8.12
21.98	0.1	8.12
22	0.14	7.99
22.02	0.16667	7.9
22.04	0.16	7.8
22.06	0.13333	7.67
22.08	0.10667	7.7
22.1	0.08667	7.93
22.12	0.06667	7.69
22.14	0.05333	7.58
22.16	0.06667	7.18
22.18	0.06667	6.95

22.2	0.08	6.76
22.22	0.1	6.63
22.24	0.13333	6.68
22.26	0.16667	6.75
22.28	0.16	6.51
22.3	0.13333	6.32
22.32	0.1	6.15
22.34	0.06667	6.32
22.36	0.03333	6.29
22.38	0.01333	6.23
22.4	0.00667	6.14
22.42	0.01333	6
22.44	0.00667	5.79
22.46	0	5.7
22.48	0	5.84
22.5	0	5.91
22.52	0.00667	5.76
22.54	0.02	5.75
22.56	0.04	5.75
22.58	0.06	5.59
22.6	0.08667	5.46
22.62	0.09333	5.46
22.64	0.07333	5.48
22.66	0.06	5.47
22.68	0.05333	5.39
22.7	0.06	5.23
22.72	0.07333	5.09
22.74	0.09333	4.99
22.76	0.10667	5.18
22.78	0.08667	5.38

22.8	0.06	5.46
22.82	0.05333	5.34
22.84	0.06667	5.07
22.86	0.08	4.59
22.88	0.10667	4.51
22.9	0.12667	4.56
22.92	0.13333	4.85
22.94	0.11333	4.81
22.96	0.08667	4.8
22.98	0.07333	4.58
23	0.08	4.57
23.02	0.06	4.59
23.04	0.05333	4.78
23.06	0.07333	4.77
23.08	0.10667	4.57
23.1	0.14	4.14
23.12	0.16667	4
23.14	0.18	4.03
23.16	0.18667	4.47
23.18	0.14	4.71
23.2	0.08667	4.78
23.22	0.03333	4.55
23.24	0.02667	4.19
23.26	0.07333	3.89
23.28	0.16	3.91
23.3	0.26	4.06
23.32	0.34667	4.41
23.34	0.37333	4.41
23.36	0.33333	4.34
23.38	0.24667	4

23.4	0.16	3.95
23.42	0.10667	3.99
23.44	0.08667	4.15
23.46	0.08667	4.15
23.48	0.12	4.08
23.5	0.14	3.96
23.52	0.15333	3.89
23.54	0.13333	3.88
23.56	0.11333	4.06
23.58	0.1	4.21
23.6	0.1	4.13
23.62	0.10667	4.01
23.64	0.14	3.72
23.66	0.14667	3.63
23.68	0.14667	3.48
23.7	0.11333	3.5
23.72	0.06667	3.49
23.74	0.02667	3.55
23.76	0.00667	3.41
23.78	0.01333	3.55
23.8	0.03333	3.61
23.82	0.05333	3.8
23.84	0.08667	3.8
23.86	0.11333	3.73
23.88	0.12667	3.61
23.9	0.13333	3.57
23.92	0.12667	3.5
23.94	0.1	3.53
23.96	0.06667	3.37
23.98	0.06667	3.24

24	0.09333	3.27
24.02	0.14	3.25
24.04	0.19333	3.42
24.06	0.21333	3.55
24.08	0.17333	3.58
24.1	0.12	3.63
24.12	0.08	3.57
24.14	0.08667	3.57
24.16	0.11333	3.6
24.18	0.16667	3.55
24.2	0.22	3.47
24.22	0.24	3.24
24.24	0.22	3.08
24.26	0.21333	3.22
24.28	0.22	3.44
24.3	0.26	3.61
24.32	0.26667	3.61
24.34	0.28667	3.47
24.36	0.28667	3.25
24.38	0.26667	3.13
24.4	0.22667	3.04
24.42	0.18	3.27
24.44	0.12667	3.31
24.46	0.1	3.4
24.48	0.07333	3.39
24.5	0.08667	3.4
24.52	0.10667	3.45
24.54	0.11333	3.44
24.56	0.11333	3.48
24.58	0.12667	3.69

24.6	0.14667	3.67
24.62	0.18	3.9
24.64	0.21333	3.68
24.66	0.24667	3.57
24.68	0.24667	3.18
24.7	0.2	3.07
24.72	0.14667	3.09
24.74	0.11333	3.36
24.76	0.11333	3.66
24.78	0.10667	3.82
24.8	0.10667	3.7
24.82	0.11333	3.57
24.84	0.09333	3.11
24.86	0.07333	2.98
24.88	0.08	2.77
24.9	0.12	2.8
24.92	0.17333	2.91
24.94	0.2	3.13
24.96	0.21333	3.44
24.98	0.20667	3.68
25	0.15333	3.69
25.02	0.10667	3.66
25.04	0.06667	3.59
25.06	0.06667	3.43
25.08	0.08667	3.27
25.1	0.12667	3.24
25.12	0.17333	3.26
25.14	0.22	3.22
25.16	0.24667	3.18
25.18	0.24	3.13

25.2	0.20667	3.1
25.22	0.18	2.99
25.24	0.17333	2.93
25.26	0.19333	2.88
25.28	0.2	2.97
25.3	0.23333	3.04
25.32	0.26667	3.29
25.34	0.25333	3.34
25.36	0.20667	3.3
25.38	0.17333	3.05
25.4	0.16667	2.91
25.42	0.18	2.77
25.44	0.19333	2.77
25.46	0.21333	2.8
25.48	0.22667	2.86
25.5	0.19333	2.87
25.52	0.14	2.91
25.54	0.12	2.91
25.56	0.12	3.07
25.58	0.15333	3.26
25.6	0.19333	3.21
25.62	0.22667	3.08
25.64	0.26667	2.75
25.66	0.3	2.59
25.68	0.31333	2.6
25.7	0.31333	2.75
25.72	0.28	2.84
25.74	0.23333	2.85
25.76	0.19333	2.63
25.78	0.18667	2.43

25.8	0.23333	2.45
25.82	0.32	2.57
25.84	0.37333	2.8
25.86	0.38	3.01
25.88	0.32667	3.06
25.9	0.24	3.03
25.92	0.15333	2.91
25.94	0.1	2.6
25.96	0.08667	2.68
25.98	0.12	2.54
26	0.14667	2.6
26.02	0.2	2.64
26.04	0.25333	2.73
26.06	0.3	2.82
26.08	0.32	2.7
26.1	0.3	2.5
26.12	0.24667	2.4
26.14	0.2	2.4
26.16	0.16	2.5
26.18	0.14	2.6
26.2	0.11333	2.68
26.22	0.1	2.69
26.24	0.07333	2.57
26.26	0.04	2.4
26.28	0.03333	2.34
26.3	0.05333	2.51
26.32	0.09333	2.61
26.34	0.13333	2.75
26.36	0.14667	2.71
26.38	0.14667	2.61

26.4	0.12667	2.42
26.42	0.12	2.27
26.44	0.13333	2.27
26.46	0.16	2.31
26.48	0.19333	2.29
26.5	0.21333	2.28
26.52	0.21333	2.29
26.54	0.2	2.41
26.56	0.18	2.38
26.58	0.18	2.42
26.6	0.19333	2.43
26.62	0.21333	2.36
26.64	0.25333	2.24
26.66	0.26	2.21
26.68	0.25333	2.2
26.7	0.22	2.35
26.72	0.16667	2.37
26.74	0.12	2.21
26.76	0.08667	2.1
26.78	0.06	2.05
26.8	0.06	2.05
26.82	0.05333	2.21
26.84	0.05333	2.22
26.86	0.05333	2.16
26.88	0.06	2.2
26.9	0.06	1.98
26.92	0.05333	1.92
26.94	0.04	1.88
26.96	0.02667	1.97
26.98	0.01333	2

27	0	1.96
27.02	0.00667	1.91
27.04	0.02667	2
27.06	0.06	2.09
27.08	0.09333	2.16
27.1	0.12667	2.11
27.12	0.14667	2.03
27.14	0.14	1.78
27.16	0.10667	1.67
27.18	0.06	1.72
27.2	0.02667	1.68
27.22	0.00667	1.79
27.24	0.01333	1.77
27.26	0.04667	1.73
27.28	0.1	1.75
27.3	0.15333	1.83
27.32	0.19333	2.06
27.34	0.20667	2.28
27.36	0.19333	2.23
27.38	0.16	2.02
27.4	0.12	1.81
27.42	0.10667	1.61
27.44	0.11333	1.5
27.46	0.13333	1.39
27.48	0.16	1.51
27.5	0.15333	1.58
27.52	0.12667	1.71
27.54	0.08	1.63
27.56	0.04	1.76
27.58	0.02	1.66

27.6	0.00667	1.5
27.62	0.02	1.33
27.64	0.04	1.32
27.66	0.06	1.37
27.68	0.07333	1.54
27.7	0.07333	1.7
27.72	0.06667	1.74
27.74	0.06	1.63
27.76	0.05333	1.53
27.78	0.05333	1.55
27.8	0.07333	1.72
27.82	0.11333	1.78
27.84	0.14	1.77
27.86	0.14667	1.52
27.88	0.14	1.2
27.9	0.14	1.1
27.92	0.12667	1.1
27.94	0.13333	1.28
27.96	0.16667	1.55
27.98	0.19333	1.66
28	0.2	1.64
28.02	0.16667	1.34
28.04	0.12	1.24
28.06	0.09333	1.28
28.08	0.08	1.42
28.1	0.08667	1.47
28.12	0.1	1.46
28.14	0.11333	1.46
28.16	0.13333	1.41
28.18	0.13333	1.45

28.2	0.13333	1.55
28.22	0.14667	1.69
28.24	0.16667	1.74
28.26	0.16	1.58
28.28	0.14	1.33
28.3	0.12	1.22
28.32	0.12	1.12
28.34	0.12667	1.33
28.36	0.14667	1.47
28.38	0.15333	1.47
28.4	0.15333	1.45
28.42	0.13333	1.42
28.44	0.09333	1.41
28.46	0.06	1.36
28.48	0.04	1.34
28.5	0.03333	1.52
28.52	0.03333	1.37
28.54	0.04	1.27
28.56	0.05333	1.19
28.58	0.06	1.21
28.6	0.07333	1.34
28.62	0.06	1.4
28.64	0.05333	1.67
28.66	0.04667	1.81
28.68	0.04667	1.65
28.7	0.05333	1.62
28.72	0.06	1.44
28.74	0.08	1.4
28.76	0.11333	1.37
28.78	0.11333	1.44

28.8	0.12	1.47
28.82	0.1	1.31
28.84	0.08667	1.09
28.86	0.06	0.98
28.88	0.04667	0.88
28.9	0.06	1.05
28.92	0.08	1.32
28.94	0.1	1.46
28.96	0.10667	1.46
28.98	0.08667	1.4
29	0.06	1.21
29.02	0.04	1.13
29.04	0.02667	1.09
29.06	0.03333	1.25
29.08	0.06	1.4
29.1	0.10667	1.3
29.12	0.14667	1.23
29.14	0.17333	1.27
29.16	0.19333	1.34
29.18	0.18	1.54
29.2	0.14667	1.75
29.22	0.10667	1.85
29.24	0.08667	1.74
29.26	0.09333	1.48
29.28	0.12	1.24
29.3	0.15333	1.12
29.32	0.17333	1.16
29.34	0.15333	1.22
29.36	0.11333	1.36
29.38	0.07333	1.46

29.4	0.04	1.4
29.42	0.02	1.41
29.44	0.02	1.25
29.46	0.02667	1.09
29.48	0.05333	1.08
29.5	0.09333	1
29.52	0.14	0.98
29.54	0.18667	1.01
29.56	0.20667	1.01
29.58	0.2	1.03
29.6	0.16	1.02
29.62	0.1	1.05
29.64	0.04667	0.94
29.66	0.01333	0.73
29.68	0.02	0.55
29.7	0.06	0.52
29.72	0.10667	0.53
29.74	0.16	0.58
29.76	0.18667	0.83
29.78	0.19333	0.91
29.8	0.18	0.87
29.82	0.18	0.75
29.84	0.18667	0.54
29.86	0.18	0.47
29.88	0.18667	0.46
29.9	0.19333	0.49
29.92	0.17333	0.7
29.94	0.16	0.72
29.96	0.15333	0.8
29.98	0.16	0.75

30	0.18	0.49
30.02	0.21333	0.29
30.04	0.26	0.26
30.06	0.29333	0.41
30.08	0.28	0.71
30.1	0.24	0.87
30.12	0.16667	0.92
30.14	0.09333	0.82
30.16	0.04	0.61
30.18	0.03333	0.43
30.2	0.05333	0.37
30.22	0.08667	0.36
30.24	0.11333	0.35
30.26	0.11333	0.27
30.28	0.10667	0.07
30.3	0.12	0.04
30.32	0.14	0.13
30.34	0.18	0.25
30.36	0.19333	0.39
30.38	0.19333	0.5
30.4	0.18667	0.52
30.42	0.16	0.44
30.44	0.17333	0.29
30.46	0.24	0.21
30.48	0.32	0.25
30.5	0.4	0.22
30.52	0.42	0.18
30.54	0.37333	0.23
30.56	0.28	0.18
30.58	0.14667	0.21

30.6	0.06667	0.34
30.62	0.05333	0.45
30.64	0.1	0.62
30.66	0.20667	0.63
30.68	0.3	0.53
30.7	0.34667	0.44
30.72	0.32	0.19
30.74	0.24	0.11
30.76	0.18667	0.1
30.78	0.14667	0.1
30.8	0.16667	0.09
30.82	0.23333	0.13
30.84	0.30667	0.2
30.86	0.36	0.22
30.88	0.37333	0.18
30.9	0.35333	0.15
30.92	0.32667	0.09
30.94	0.24667	0.03
30.96	0.18	0.11
30.98	0.10667	0.24
31	0.04667	0.4
31.02	0.03333	0.45
31.04	0.06667	0.42
31.06	0.15333	0.27
31.08	0.25333	0.18
31.1	0.34	0.05
31.12	0.42	0.05
31.14	0.42667	0.06
31.16	0.41333	0.1
31.18	0.40667	0.09

31.2	0.38	0.07
31.22	0.38	0
31.24	0.36	0.01
31.26	0.34	0.12
31.28	0.33333	0.14
31.3	0.28	0.25
31.32	0.24	0.29
31.34	0.21333	0.27
31.36	0.16	0.21
31.38	0.12667	0.1
31.4	0.1	0.04
31.42	0.09333	0.05
31.44	0.10667	0
31.46	0.11333	0.02
31.48	0.15333	0.02
31.5	0.20667	0.01
31.52	0.22667	0.01
31.54	0.26	0
31.56	0.27333	--
31.58	0.27333	0.04
31.6	0.26667	0.07
31.62	0.27333	0.09
31.64	0.31333	0.06
31.66	0.35333	0.09
31.68	0.36667	0.11
31.7	0.35333	0.1
31.72	0.29333	0.06
31.74	0.23333	0.09
31.76	0.17333	0.07
31.78	0.15333	0.03

31.8	0.17333	0
31.82	0.2	0
31.84	0.20667	0.02
31.86	0.20667	0.04
31.88	0.18667	0.05
31.9	0.19333	--
31.92	0.21333	0.05
31.94	0.25333	0.05
31.96	0.31333	0.03
31.98	0.35333	0
32	0.36	0
32.02	0.36667	0.01
32.04	0.35333	0.02
32.06	0.36	0.02
32.08	0.36	--
32.1	0.37333	0.03
32.12	0.37333	0.01
32.14	0.32667	0.02
32.16	0.28	0.03
32.18	0.21333	0.02
32.2	0.16667	0.03
32.22	0.15333	0.03
32.24	0.17333	0.02
32.26	0.22	--
32.28	0.24667	0
32.3	0.23333	0
32.32	0.22	0.06
32.34	0.17333	0.1
32.36	0.14667	0.13
32.38	0.16	0.13

32.4	0.18	0.11
32.42	0.22667	0.05
32.44	0.24667	0.01
32.46	0.24	0
32.48	0.22667	0.04
32.5	0.20667	0.04
32.52	0.19333	0.05
32.54	0.18	0.05
32.56	0.17333	0.03
32.58	0.17333	0
32.6	0.15333	--
32.62	0.13333	0
32.64	0.12	0.05
32.66	0.13333	0.11
32.68	0.15333	0.14
32.7	0.18667	0.16
32.72	0.22	0.15
32.74	0.26	0.1
32.76	0.26	0.03
32.78	0.26	0
32.8	0.26	0
32.82	0.28	0
32.84	0.32667	0.01
32.86	0.36667	0.04
32.88	0.39333	0.13
32.9	0.41333	0.16
32.92	0.36	0.18
32.94	0.26667	0.23
32.96	0.17333	0.22
32.98	0.11333	0.16

33	0.09333	0.13
33.02	0.12	0.07
33.04	0.18	0.06
33.06	0.26667	0
33.08	0.29333	0
33.1	0.28	0
33.12	0.24	0.05
33.14	0.2	0.1
33.16	0.16667	0.13
33.18	0.15333	0.13
33.2	0.14667	0.1
33.22	0.14667	0.08
33.24	0.10667	0.01
33.26	0.06667	0
33.28	0.04667	0.09
33.3	0.04	0.12
33.32	0.05333	0.14
33.34	0.08667	0.13
33.36	0.14	0.1
33.38	0.16667	0.09
33.4	0.16	0.03
33.42	0.15333	0.02
33.44	0.14	0
33.46	0.1	0.02
33.48	0.06667	0.07
33.5	0.04667	0.08
33.52	0.03333	0.12
33.54	0.02667	0.12
33.56	0.03333	0.14
33.58	0.06	0.12

33.6	0.10667	0
33.62	0.15333	0.09
33.64	0.19333	0.2
33.66	0.2	0.25
33.68	0.18	0.28
33.7	0.14	0.24
33.72	0.11333	0.17
33.74	0.10667	0.09
33.76	0.13333	0.01
33.78	0.16667	0.06
33.8	0.18	0.1
33.82	0.15333	0.12
33.84	0.11333	0.13
33.86	0.09333	0.1
33.88	0.09333	0.04
33.9	0.11333	0.02
33.92	0.14	0.08
33.94	0.16	0.09
33.96	0.14667	0.07
33.98	0.11333	0.11
34	0.08667	0.15
34.02	0.08	0.16
34.04	0.08	0.12
34.06	0.08	0.08
34.08	0.08	0.08
34.1	0.07333	0.02
34.12	0.06	0
34.14	0.06	0.03
34.16	0.06667	0.12
34.18	0.08	0.14

34.2	0.07333	0.21
34.22	0.05333	0.25
34.24	0.04	0.24
34.26	0.04667	0.17
34.28	0.08	0.11
34.3	0.15333	0.05
34.32	0.22667	0.06
34.34	0.28	0.04
34.36	0.29333	0.03
34.38	0.25333	0.14
34.4	0.18667	0.23
34.42	0.12667	0.24
34.44	0.09333	0.26
34.46	0.07333	0.25
34.48	0.06	0.25
34.5	0.05333	0.19
34.52	0.05333	0.08
34.54	0.05333	0.12
34.56	0.06	0.11
34.58	0.07333	0.07
34.6	0.07333	0.14
34.62	0.05333	0.18
34.64	0.03333	0.16
34.66	0.03333	0.13
34.68	0.06667	0.07
34.7	0.12	0.13
34.72	0.18	0.15
34.74	0.21333	0.24
34.76	0.2	0.31
34.78	0.13333	0.32

34.8	0.07333	0.28
34.82	0.04667	0.19
34.84	0.06	0.08
34.86	0.11333	0.06
34.88	0.18667	0.01
34.9	0.24667	0.01
34.92	0.25333	0
34.94	0.21333	0.09
34.96	0.14667	0.24
34.98	0.09333	0.31
35	0.06667	0.35
35.02	0.06667	0.32
35.04	0.10667	0.28
35.06	0.15333	0.19
35.08	0.17333	0.16
35.1	0.19333	0.28
35.12	0.2	0.36
35.14	0.21333	0.35
35.16	0.21333	0.38
35.18	0.2	0.29
35.2	0.19333	0.29
35.22	0.17333	0.29
35.24	0.14	0.3
35.26	0.12	0.36
35.28	0.09333	0.3
35.3	0.10667	0.32
35.32	0.12	0.43
35.34	0.13333	0.41
35.36	0.15333	0.45
35.38	0.16667	0.5

35.4	0.17333	0.47
35.42	0.18	0.47
35.44	0.18	0.31
35.46	0.2	0.23
35.48	0.2	0.23
35.5	0.19333	0.25
35.52	0.18667	0.3
35.54	0.18	0.44
35.56	0.17333	0.63
35.58	0.17333	0.73
35.6	0.18667	0.69
35.62	0.20667	0.52
35.64	0.21333	0.39
35.66	0.21333	0.27
35.68	0.20667	0.16
35.7	0.20667	0.2
35.72	0.20667	0.39
35.74	0.22667	0.45
35.76	0.26	0.51
35.78	0.29333	0.43
35.8	0.29333	0.47
35.82	0.28667	0.38
35.84	0.25333	0.36
35.86	0.22667	0.35
35.88	0.20667	0.46
35.9	0.22	0.41
35.92	0.25333	0.4
35.94	0.28	0.4
35.96	0.26667	0.54
35.98	0.23333	0.5

36	0.18	0.53
36.02	0.12667	0.46
36.04	0.1	0.44
36.06	0.1	0.3
36.08	0.12	0.18
36.1	0.13333	0.19
36.12	0.14	0.24
36.14	0.12667	0.23
36.16	0.11333	0.26
36.18	0.11333	0.23
36.2	0.12667	0.15
36.22	0.14667	0.11
36.24	0.16	0.21
36.26	0.16	0.34
36.28	0.13333	0.36
36.3	0.08667	0.42
36.32	0.04667	0.41
36.34	0.01333	0.31
36.36	0.00667	0.08
36.38	0.01333	0.03
36.4	0.03333	0.14
36.42	0.06667	0.19
36.44	0.1	0.16
36.46	0.12667	0.19
36.48	0.13333	0.26
36.5	0.13333	0.27
36.52	0.12667	0.24
36.54	0.11333	0.29
36.56	0.12	0.39
36.58	0.14667	0.44

36.6	0.18	0.36
36.62	0.2	0.29
36.64	0.19333	0.28
36.66	0.19333	0.18
36.68	0.17333	0.17
36.7	0.16667	0.21
36.72	0.18667	0.32
36.74	0.2	0.31
36.76	0.18667	0.21
36.78	0.15333	0.22
36.8	0.1	0.16
36.82	0.05333	0.15
36.84	0.02	0.18
36.86	0.02	0.2
36.88	0.06	0.24
36.9	0.1	0.21
36.92	0.13333	0.13
36.94	0.16	0.12
36.96	0.14667	0.04
36.98	0.11333	0.08
37	0.06667	0.04
37.02	0.03333	0
37.04	0.02667	0.1
37.06	0.06	0.18
37.08	0.11333	0.29
37.1	0.18	0.27
37.12	0.21333	0.31
37.14	0.21333	0.35
37.16	0.16667	0.32
37.18	0.10667	0.2

37.2	0.04667	0.19
37.22	0.02	0.16
37.24	0.02667	0.16
37.26	0.06	0.1
37.28	0.11333	0.12
37.3	0.17333	0.12
37.32	0.20667	0.1
37.34	0.21333	0
37.36	0.18667	0
37.38	0.16	0.09
37.4	0.11333	0.19
37.42	0.08	0.22
37.44	0.07333	0.28
37.46	0.08	0.28
37.48	0.08	0.23
37.5	0.08667	0.12
37.52	0.08	0.07
37.54	0.06667	0.08
37.56	0.05333	0.06
37.58	0.06667	0.01
37.6	0.08	0
37.62	0.10667	0.03
37.64	0.12	0.12
37.66	0.13333	0.19
37.68	0.12	0.19
37.7	0.11333	0.2
37.72	0.12	0.2
37.74	0.13333	0.15
37.76	0.14	0.05
37.78	0.14	0.03

37.8	0.14	0.04
37.82	0.12	0.02
37.84	0.1	0.01
37.86	0.08667	0.03
37.88	0.09333	0.04
37.9	0.10667	0.11
37.92	0.11333	0.1
37.94	0.12667	0.16
37.96	0.14	0.2
37.98	0.14667	0.18
38	0.15333	0.16
38.02	0.18	0.09
38.04	0.21333	0.01
38.06	0.24	0.04
38.08	0.24	0
38.1	0.23333	0.09
38.12	0.21333	0.17
38.14	0.16667	0.22
38.16	0.13333	0.22
38.18	0.13333	0.18
38.2	0.16	0.08
38.22	0.19333	0.06
38.24	0.22	0
38.26	0.22667	0.06
38.28	0.22	0.1
38.3	0.19333	0.11
38.32	0.18667	0.1
38.34	0.19333	0.07
38.36	0.21333	0.02
38.38	0.20667	0

38.4	0.17333	0
38.42	0.11333	0.03
38.44	0.06	0.04
38.46	0.02667	0.05
38.48	0.02667	0.05
38.5	0.06	0.04
38.52	0.10667	0.02
38.54	0.14667	--
38.56	0.16	0
38.58	0.15333	0
38.6	0.14	0
38.62	0.12667	0.05
38.64	0.1	0.11
38.66	0.08	0.14
38.68	0.06667	0.15
38.7	0.06	0.12
38.72	0.06	0.09
38.74	0.05333	0.02
38.76	0.06667	0.01
38.78	0.06667	0.01
38.8	0.06	0
38.82	0.07333	0.03
38.84	0.1	0.03
38.86	0.13333	0.09
38.88	0.17333	--
38.9	0.20667	0.14
38.92	0.24	0.16
38.94	0.23333	0.11
38.96	0.2	0.12
38.98	0.15333	0.08

39	0.11333	0.1
39.02	0.06667	0.06
39.04	0.04667	0.08
39.06	0.04667	0.06
39.08	0.07333	0.03
39.1	0.1	0
39.12	0.12667	0.01
39.14	0.13333	0.01
39.16	0.13333	0
39.18	0.10667	0.03
39.2	0.08	0.04
39.22	0.06667	--
39.24	0.07333	0.04
39.26	0.09333	0.03
39.28	0.11333	0.07
39.3	0.13333	0.09
39.32	0.15333	0.09
39.34	0.15333	0.06
39.36	0.15333	0.08
39.38	0.16667	0.07
39.4	0.2	--
39.42	0.22	0
39.44	0.22667	0.04
39.46	0.24	0.15
39.48	0.22	0.18
39.5	0.20667	0.21
39.52	0.2	0.22
39.54	0.21333	0.19
39.56	0.22667	0.12
39.58	0.22	0

39.6	0.21333	0.06
39.62	0.19333	0.06
39.64	0.15333	0.04
39.66	0.14	0.07
39.68	0.14667	0.07
39.7	0.16	0.06
39.72	0.18	0.05
39.74	0.2	--
39.76	0.20667	0.02
39.78	0.18	0
39.8	0.12667	0
39.82	0.07333	0
39.84	0.03333	0.1
39.86	0.01333	0.19
39.88	0.03333	0.24
39.9	0.1	0.25
39.92	0.17333	0.21
39.94	0.23333	0.14
39.96	0.26667	0.03
39.98	0.24667	0
40	0.22667	0.08
40.02	0.21333	0.12
40.04	0.24667	0.2
40.06	0.30667	0.29
40.08	0.37333	0.31
40.1	0.41333	0.26
40.12	0.40667	0.23
40.14	0.34	0.16
40.16	0.24667	0.14
40.18	0.14	0.1

40.2	0.06667	0.12
40.22	0.02667	0.12
40.24	0.01333	0.08
40.26	0.03333	0.02
40.28	0.06	0.02
40.3	0.07333	0
40.32	0.08667	0.01
40.34	0.1	0.13
40.36	0.1	0.21
40.38	0.09333	0.21
40.4	0.06667	0.24
40.42	0.04667	0.23
40.44	0.02667	0.15
40.46	0.02667	0.1
40.48	0.06667	0.09
40.5	0.13333	0.08
40.52	0.2	0.08
40.54	0.24	0.06
40.56	0.24667	0.09
40.58	0.23333	0.2
40.6	0.20667	0.22
40.62	0.2	0.26
40.64	0.23333	0.25
40.66	0.26	0.2
40.68	0.28	0.3
40.7	0.27333	0.31
40.72	0.22667	0.27
40.74	0.16	0.26
40.76	0.09333	0.19
40.78	0.05333	0.15

40.8	0.05333	0.08
40.82	0.08667	0.04
40.84	0.14	0.09
40.86	0.18667	0.07
40.88	0.22	0.03
40.9	0.20667	0
40.92	0.16	0
40.94	0.09333	0
40.96	0.04	0
40.98	0.02	0.05
41	0.02667	0.16
41.02	0.07333	0.19
41.04	0.14667	0.25
41.06	0.21333	0.3
41.08	0.26	0.3
41.1	0.29333	0.21
41.12	0.30667	0.19
41.14	0.28667	0.15
41.16	0.24667	0.17
41.18	0.2	0.12
41.2	0.16667	0.15
41.22	0.12667	0.17
41.24	0.09333	0.18
41.26	0.1	0.16
41.28	0.11333	0.13
41.3	0.12	0.12
41.32	0.12	0.1
41.34	0.13333	0.09
41.36	0.15333	0.13
41.38	0.16667	0.16

41.4 0.19333 0.23

41.42 0.24 0.23

41.44 0.25333 0.21

41.46 0.24 0.21

41.48 0.18667 0.13

41.5 0.13333 0.07

41.52 0.08 0.04

41.54 0.04667 0

41.56 0.04667 0

41.58 0.06 0.01

41.6 0.07333 0.04

41.62 0.08667 0.07

41.64 0.08667 0.07

41.66 0.09333 0.18

41.68 0.1 0.26

41.7 0.11333 0.26

41.72 0.12667 0.29

41.74 0.13333 0.29

41.76 0.12 0.19

41.78 0.11333 0.11

41.8 0.11333 0.08

41.82 0.14 0.15

41.84 0.18 0.15

41.86 0.21333 0.11

41.88 0.25333 0.12

41.9 0.26667 0.08

41.92 0.24 0.01

41.94 0.2 0

41.96 0.17333 0

41.98 0.16 0.01

42	0.17333	0
42.02	0.19333	0.03
42.04	0.23333	0.08
42.06	0.24	0.09
42.08	0.19333	0.08
42.1	0.14667	0.11
42.12	0.10667	0.11
42.14	0.07333	0.11
42.16	0.08	0.04
42.18	0.13333	0.09
42.2	0.21333	0.09
42.22	0.28	0.03
42.24	0.32667	0.04
42.26	0.32	0.12
42.28	0.27333	0.16
42.3	0.18	0.2
42.32	0.1	0.21
42.34	0.07333	0.23
42.36	0.08	0.16
42.38	0.13333	0.04
42.4	0.19333	0.1
42.42	0.23333	0.13
42.44	0.25333	0.12
42.46	0.23333	0.16
42.48	0.22	0.17
42.5	0.22	0.14
42.52	0.22667	0.06
42.54	0.23333	0.01
42.56	0.22	0.06
42.58	0.19333	0.01

42.6	0.15333	0.09
42.62	0.10667	0.23
42.64	0.08667	0.3
42.66	0.08667	0.35
42.68	0.12	0.38
42.7	0.16667	0.4
42.72	0.2	0.32
42.74	0.22667	0.21
42.76	0.23333	0.19
42.78	0.22667	0.14
42.8	0.2	0.07
42.82	0.16667	0.1
42.84	0.13333	0.09
42.86	0.12	0.18
42.88	0.1	0.23
42.9	0.10667	0.31
42.92	0.12	0.3
42.94	0.14667	0.25
42.96	0.15333	0.19
42.98	0.15333	0.15
43	0.15333	0.1
43.02	0.14667	0.11
43.04	0.11333	0.12
43.06	0.09333	0.24
43.08	0.09333	0.3
43.1	0.08	0.43
43.12	0.06	0.47
43.14	0.05333	0.43
43.16	0.04667	0.35
43.18	0.03333	0.22

43.2	0.02667	0.11
43.22	0.05333	0.06
43.24	0.11333	0.12
43.26	0.17333	0.26
43.28	0.24	0.27
43.3	0.29333	0.26
43.32	0.30667	0.19
43.34	0.28667	0.14
43.36	0.23333	0.06
43.38	0.18667	0.02
43.4	0.18	0.07
43.42	0.18667	0.08
43.44	0.24	0.14
43.46	0.29333	0.28
43.48	0.33333	0.34
43.5	0.33333	0.36
43.52	0.28667	0.4
43.54	0.22667	0.38
43.56	0.18	0.31
43.58	0.15333	0.19
43.6	0.17333	0.19
43.62	0.20667	0.19
43.64	0.22667	0.16
43.66	0.23333	0.11
43.68	0.21333	0.18
43.7	0.17333	0.19
43.72	0.11333	0.18
43.74	0.06667	0.11
43.76	0.04667	0.09
43.78	0.03333	0.08

43.8	0.04	0.07
43.82	0.07333	0.03
43.84	0.12667	0.08
43.86	0.17333	0.15
43.88	0.2	0.21
43.9	0.21333	0.22
43.92	0.19333	0.17
43.94	0.14	0.23
43.96	0.07333	0.26
43.98	0.04	0.25
44	0.03333	0.2
44.02	0.06	0.32
44.04	0.10667	0.41
44.06	0.17333	0.39
44.08	0.22	0.37
44.1	0.22667	0.39
44.12	0.20667	0.34
44.14	0.16667	0.21
44.16	0.12	0.05
44.18	0.08	0.07
44.2	0.05333	0.04
44.22	0.06667	0.03
44.24	0.1	0.13
44.26	0.15333	0.23
44.28	0.2	0.36
44.3	0.22	0.42
44.32	0.21333	0.37
44.34	0.2	0.33
44.36	0.16667	0.2
44.38	0.16	0.07

44.4	0.18	0.02
44.42	0.2	0.05
44.44	0.22667	0.13
44.46	0.23333	0.14
44.48	0.20667	0.12
44.5	0.17333	0.1
44.52	0.13333	0.02
44.54	0.10667	0.01
44.56	0.06667	0.01
44.58	0.04	0.09
44.6	0.04	0.13
44.62	0.06667	0.22
44.64	0.10667	0.23
44.66	0.17333	0.23
44.68	0.25333	0.21
44.7	0.31333	0.22
44.72	0.30667	0.21
44.74	0.26667	0.26
44.76	0.20667	0.26
44.78	0.14	0.36
44.8	0.07333	0.39
44.82	0.04	0.45
44.84	0.03333	0.45
44.86	0.04667	0.39
44.88	0.07333	0.34
44.9	0.1	0.22
44.92	0.12	0.12
44.94	0.13333	0.09
44.96	0.12667	0.17
44.98	0.12	0.24

45	0.10667	0.29
45.02	0.08	0.33
45.04	0.08	0.37
45.06	0.08	0.31
45.08	0.09333	0.24
45.1	0.12	0.15
45.12	0.14	0.18
45.14	0.16	0.18
45.16	0.18	0.18
45.18	0.18	0.22
45.2	0.18667	0.25
45.22	0.18	0.27
45.24	0.16	0.24
45.26	0.14	0.23
45.28	0.11333	0.27
45.3	0.1	0.27
45.32	0.10667	0.22
45.34	0.13333	0.18
45.36	0.17333	0.07
45.38	0.21333	0.07
45.4	0.24	0.09
45.42	0.23333	0.18
45.44	0.21333	0.28
45.46	0.16667	0.4
45.48	0.11333	0.44
45.5	0.08667	0.37
45.52	0.08	0.26
45.54	0.08667	0.22
45.56	0.11333	0.13
45.58	0.14	0.09

45.6	0.15333	0.09
45.62	0.13333	0.22
45.64	0.09333	0.29
45.66	0.06	0.29
45.68	0.04	0.34
45.7	0.04	0.31
45.72	0.06	0.21
45.74	0.08667	0.1
45.76	0.11333	0.03
45.78	0.10667	0.09
45.8	0.08	0.14
45.82	0.04667	0.13
45.84	0.02	0.19
45.86	0.00667	0.18
45.88	0.01333	0.17
45.9	0.03333	0.07
45.92	0.06667	0.08
45.94	0.1	0.24
45.96	0.13333	0.3
45.98	0.14	0.31
46	0.14	0.32
46.02	0.13333	0.32
46.04	0.12	0.35
46.06	0.10667	0.29
46.08	0.1	0.27
46.1	0.1	0.29
46.12	0.10667	0.19
46.14	0.12	0.09
46.16	0.13333	0
46.18	0.17333	0

46.2	0.21333	0.02
46.22	0.25333	0.04
46.24	0.28667	0.01
46.26	0.28	0.06
46.28	0.25333	0.11
46.3	0.21333	0.15
46.32	0.17333	0.22
46.34	0.16	0.28
46.36	0.16	0.3
46.38	0.19333	0.23
46.4	0.23333	0.16
46.42	0.26	0.15
46.44	0.26667	0.15
46.46	0.24667	0.18
46.48	0.2	0.22
46.5	0.14	0.21
46.52	0.08	0.27
46.54	0.03333	0.25
46.56	0.02	0.22
46.58	0.02667	0.17
46.6	0.08	0.15
46.62	0.13333	0.19
46.64	0.19333	0.18
46.66	0.24	0.13
46.68	0.24667	0.17
46.7	0.22	0.23
46.72	0.16667	0.21
46.74	0.12	0.2
46.76	0.1	0.16
46.78	0.10667	0.27

46.8	0.14667	0.28
46.82	0.2	0.23
46.84	0.22667	0.24
46.86	0.22	0.27
46.88	0.17333	0.33
46.9	0.12	0.31
46.92	0.05333	0.29
46.94	0.02	0.42
46.96	0.02667	0.42
46.98	0.08667	0.38
47	0.18	0.29
47.02	0.26667	0.21
47.04	0.35333	0.21
47.06	0.40667	0.15
47.08	0.40667	0.2
47.1	0.38	0.25
47.12	0.30667	0.25
47.14	0.24	0.22
47.16	0.17333	0.16
47.18	0.11333	0.12
47.2	0.09333	0.02
47.22	0.1	0.01
47.24	0.14667	0.09
47.26	0.21333	0.22
47.28	0.27333	0.28
47.3	0.32667	0.38
47.32	0.35333	0.45
47.34	0.34667	0.41
47.36	0.3	0.34
47.38	0.22667	0.29

47.4	0.16	0.29
47.42	0.11333	0.31
47.44	0.1	0.21
47.46	0.12667	0.22
47.48	0.18	0.2
47.5	0.24	0.15
47.52	0.28667	0.14
47.54	0.28	0.12
47.56	0.26	0.14
47.58	0.24667	0.1
47.6	0.24	0.06
47.62	0.22667	0.11
47.64	0.21333	0.13
47.66	0.2	0.13
47.68	0.19333	0.2
47.7	0.16	0.21
47.72	0.14667	0.2
47.74	0.16	0.14
47.76	0.16	0.08
47.78	0.16667	0.1
47.8	0.14667	0.06
47.82	0.12667	0.01
47.84	0.11333	0.09
47.86	0.09333	0.15
47.88	0.08	0.17
47.9	0.08	0.17
47.92	0.08667	0.12
47.94	0.10667	0.12
47.96	0.12667	0.11
47.98	0.16	0.08

48	0.19333	0.16
48.02	0.22	0.17
48.04	0.22	0.2
48.06	0.18667	0.25
48.08	0.14	0.23
48.1	0.09333	0.19
48.12	0.07333	0.13
48.14	0.06667	0.14
48.16	0.08667	0.15
48.18	0.12667	0.09
48.2	0.16667	0.09
48.22	0.18667	0.09
48.24	0.2	0.05
48.26	0.2	0.03
48.28	0.2	0
48.3	0.17333	0.16
48.32	0.14	0.25
48.34	0.12667	0.27
48.36	0.12	0.27
48.38	0.12	0.22
48.4	0.12667	0.13
48.42	0.15333	0.09
48.44	0.16667	0.01
48.46	0.17333	0.15
48.48	0.18	0.22
48.5	0.19333	0.22
48.52	0.19333	0.21
48.54	0.16	0.22
48.56	0.11333	0.21
48.58	0.07333	0.17

48.6	0.04667	0.14
48.62	0.04	0.21
48.64	0.07333	0.21
48.66	0.14	0.13
48.68	0.22	0.07
48.7	0.28	0.08
48.72	0.29333	0.09
48.74	0.28667	0.04
48.76	0.24	0.04
48.78	0.18	0.11
48.8	0.13333	0.21
48.82	0.12	0.24
48.84	0.12	0.24
48.86	0.14667	0.25
48.88	0.15333	0.24
48.9	0.16667	0.17
48.92	0.15333	0.13
48.94	0.14667	0.13
48.96	0.14	0.17
48.98	0.14	0.13
49	0.14	0.08
49.02	0.14	0.05
49.04	0.14667	0.09
49.06	0.14667	0.13
49.08	0.14	0.14
49.1	0.14	0.11
49.12	0.15333	0.17
49.14	0.16667	0.27
49.16	0.18	0.28
49.18	0.18	0.28

49.2	0.17333	0.37
49.22	0.14	0.37
49.24	0.1	0.3
49.26	0.05333	0.18
49.28	0.02667	0.17
49.3	0.02	0.12
49.32	0.02	0.05
49.34	0.02667	0.06
49.36	0.04667	0.14
49.38	0.06	0.22
49.4	0.08	0.26
49.42	0.09333	0.23
49.44	0.10667	0.21
49.46	0.10667	0.14
49.48	0.08667	0.12
49.5	0.06	0.13
49.52	0.03333	0.14
49.54	0.00667	0.23
49.56	0	0.24
49.58	0.02	0.24
49.6	0.05333	0.22
49.62	0.1	0.16
49.64	0.13333	0.14
49.66	0.15333	0.19
49.68	0.13333	0.12
49.7	0.1	0.19
49.72	0.05333	0.23
49.74	0.02667	0.24
49.76	0.01333	0.23
49.78	0.02667	0.19

49.8	0.06667	0.23
49.82	0.12	0.2
49.84	0.16	0.13
49.86	0.19333	0.19
49.88	0.19333	0.21
49.9	0.18667	0.16
49.92	0.16	0.14
49.94	0.13333	0.11
49.96	0.11333	0.09
49.98	0.09333	0
50	0.08667	0
50.02	0.07333	0.08
50.04	0.04667	0.17
50.06	0.04	0.24
50.08	0.04667	0.25
50.1	0.08	0.24
50.12	0.11333	0.22
50.14	0.14	0.15
50.16	0.16	0.12
50.18	0.15333	0.12
50.2	0.13333	0.13
50.22	0.13333	0.13
50.24	0.14667	0.08
50.26	0.18	0.11
50.28	0.2	0.14
50.3	0.21333	0.17
50.32	0.19333	0.19
50.34	0.15333	0.2
50.36	0.11333	0.23
50.38	0.10667	0.2

50.4	0.11333	0.19
50.42	0.13333	0.25
50.44	0.14667	0.2
50.46	0.16667	0.23
50.48	0.15333	0.3
50.5	0.11333	0.28
50.52	0.07333	0.32
50.54	0.04	0.28
50.56	0.02	0.28
50.58	0.01333	0.26
50.6	0.02667	0.11
50.62	0.04667	0.09
50.64	0.06667	0.13
50.66	0.08	0.1
50.68	0.08667	0.17
50.7	0.1	0.16
50.72	0.11333	0.17
50.74	0.11333	0.21
50.76	0.12667	0.18
50.78	0.12667	0.18
50.8	0.13333	0.18
50.82	0.13333	0.11
50.84	0.12	0.1
50.86	0.11333	0.06
50.88	0.09333	0.05
50.9	0.06667	0.12
50.92	0.04667	0.16
50.94	0.02	0.23
50.96	0.00667	0.31
50.98	0.00667	0.33

51	0.01333	0.34
51.02	0.02667	0.38
51.04	0.04	0.3
51.06	0.06	0.25
51.08	0.08	0.2
51.1	0.08667	0.17
51.12	0.09333	0.13
51.14	0.09333	0.1
51.16	0.07333	0.08
51.18	0.04667	0.16
51.2	0.02667	0.18
51.22	0.01333	0.2
51.24	0.02	0.25
51.26	0.04	0.24
51.28	0.08	0.18
51.3	0.12667	0.12
51.32	0.16	0.09
51.34	0.16	0.14
51.36	0.15333	0.14
51.38	0.13333	0.09
51.4	0.10667	0.17
51.42	0.08667	0.23
51.44	0.06	0.28
51.46	0.04667	0.26
51.48	0.04667	0.22
51.5	0.03333	0.26
51.52	0.03333	0.25
51.54	0.02667	0.24
51.56	0.03333	0.23
51.58	0.03333	0.29

51.6	0.02667	0.35
51.62	0.02667	0.32
51.64	0.02667	0.3
51.66	0.02	0.3
51.68	0.03333	0.31
51.7	0.05333	0.31
51.72	0.06667	0.25
51.74	0.08667	0.23
51.76	0.10667	0.23
51.78	0.14	0.2
51.8	0.14667	0.18
51.82	0.14667	0.18
51.84	0.14667	0.17
51.86	0.12	0.17
51.88	0.08667	0.12
51.9	0.06	0.03
51.92	0.04667	0.04
51.94	0.04	0.09
51.96	0.04	0.14
51.98	0.03333	0.15
52	0.02667	0.2
52.02	0.02	0.25
52.04	0.01333	0.24
52.06	0.01333	0.17
52.08	0.02	0.15
52.1	0.02667	0.16
52.12	0.02667	0.16
52.14	0.02667	0.08
52.16	0.03333	0.11
52.18	0.04	0.12

52.2	0.04	0.14
52.22	0.04	0.18
52.24	0.04	0.18
52.26	0.04	0.32
52.28	0.04	0.34
52.3	0.04667	0.35
52.32	0.06	0.31
52.34	0.05333	0.24
52.36	0.04667	0.16
52.38	0.03333	0.13
52.4	0.01333	0.13
52.42	0.00667	0.16
52.44	0.02	0.06
52.46	0.04667	0.14
52.48	0.07333	0.22
52.5	0.1	0.31
52.52	0.11333	0.3
52.54	0.12667	0.31
52.56	0.12	0.27
52.58	0.10667	0.15
52.6	0.09333	0
52.62	0.08667	0
52.64	0.06667	0.01
52.66	0.04667	0.06
52.68	0.02667	0.11
52.7	0.01333	0.14
52.72	0.00667	0.16
52.74	0.01333	0.13
52.76	0.03333	0.09
52.78	0.06	0.03

52.8	0.08667	0.07
52.82	0.11333	0.04
52.84	0.13333	0.07
52.86	0.14	0.08
52.88	0.13333	0.08
52.9	0.11333	0.17
52.92	0.09333	0.27
52.94	0.06667	0.3
52.96	0.04	0.34
52.98	0.02667	0.31
53	0.02	0.27
53.02	0.02	0.18
53.04	0.01333	0.07
53.06	0.02	0.08
53.08	0.04	0.1
53.1	0.06667	0.08
53.12	0.08667	0.12
53.14	0.12	0.13
53.16	0.15333	0.13
53.18	0.17333	0.2
53.2	0.15333	0.23
53.22	0.14	0.23
53.24	0.12667	0.27
53.26	0.11333	0.25
53.28	0.1	0.2
53.3	0.10667	0.16
53.32	0.12	0.09
53.34	0.14	0.07
53.36	0.14	0.11
53.38	0.13333	0.07

53.4	0.13333	0.14
53.42	0.14667	0.17
53.44	0.15333	0.2
53.46	0.14667	0.18
53.48	0.13333	0.14
53.5	0.12667	0.09
53.52	0.10667	0.17
53.54	0.1	0.16
53.56	0.11333	0.17
53.58	0.13333	0.16
53.6	0.14	0.18
53.62	0.12667	0.13
53.64	0.1	0.22
53.66	0.08	0.29
53.68	0.06	0.41
53.7	0.06667	0.47
53.72	0.09333	0.43
53.74	0.12667	0.36
53.76	0.14667	0.28
53.78	0.16	0.15
53.8	0.15333	0.08
53.82	0.12667	0.04
53.84	0.08667	0.03
53.86	0.04667	0.03
53.88	0.02	0.06
53.9	0	0.09
53.92	0	0.08
53.94	0.00667	0.11
53.96	0.02667	0.11
53.98	0.04	0.12

54	0.05333	0.12
54.02	0.06	0.08
54.04	0.06	0.08
54.06	0.06667	0.05
54.08	0.08	0
54.1	0.09333	0
54.12	0.12	0.04
54.14	0.12	0.09
54.16	0.11333	0.1
54.18	0.1	0.13
54.2	0.07333	0.17
54.22	0.04667	0.3
54.24	0.03333	0.47
54.26	0.02	0.52
54.28	0.02	0.53
54.3	0.04	0.48
54.32	0.07333	0.32
54.34	0.12	0.18
54.36	0.14667	0.07
54.38	0.16667	0.06
54.4	0.18	0.11
54.42	0.16667	0.09
54.44	0.15333	0.06
54.46	0.14667	0.07
54.48	0.12667	0.06
54.5	0.10667	0.09
54.52	0.08	0.1
54.54	0.05333	0.09
54.56	0.04667	0.2
54.58	0.04667	0.27

54.6	0.04	0.29
54.62	0.04	0.26
54.64	0.02667	0.19
54.66	0.02	0.14
54.68	0.01333	0.09
54.7	0.00667	0.04
54.72	0.01333	0.08
54.74	0.04667	0.06
54.76	0.09333	0.08
54.78	0.14667	0.07
54.8	0.19333	0.12
54.82	0.22667	0.16
54.84	0.22	0.24
54.86	0.18	0.31
54.88	0.13333	0.3
54.9	0.08667	0.24
54.92	0.06	0.2
54.94	0.04667	0.09
54.96	0.04	0.08
54.98	0.04	0.06
55	0.04	0.09
55.02	0.04	0.11
55.04	0.05333	0.12
55.06	0.06	0.12
55.08	0.07333	0.14
55.1	0.1	0.13
55.12	0.13333	0.2
55.14	0.17333	0.21
55.16	0.20667	0.2
55.18	0.22667	0.13

55.2	0.21333	0.12
55.22	0.16667	0.12
55.24	0.11333	0.16
55.26	0.08	0.15
55.28	0.06667	0.2
55.3	0.08	0.18
55.32	0.1	0.15
55.34	0.1	0.13
55.36	0.08	0.24
55.38	0.05333	0.25
55.4	0.02667	0.29
55.42	0	0.28
55.44	0	0.34
55.46	0	0.34
55.48	0.00667	0.32
55.5	0.00667	0.3
55.52	0.01333	0.29
55.54	0.03333	0.22
55.56	0.05333	0.19
55.58	0.07333	0.13
55.6	0.1	0.13
55.62	0.12667	0.12
55.64	0.14667	0.06
55.66	0.14	0.07
55.68	0.12667	0.04
55.7	0.10667	0.13
55.72	0.08667	0.28
55.74	0.06667	0.39
55.76	0.06	0.48
55.78	0.06	0.47

55.8	0.06667	0.34
55.82	0.08	0.26
55.84	0.10667	0.12
55.86	0.12667	0.06
55.88	0.15333	0.08
55.9	0.18667	0.04
55.92	0.22667	0.08
55.94	0.26667	0.08
55.96	0.30667	0.07
55.98	0.35333	0.15
56	0.38	0.25
56.02	0.34667	0.32
56.04	0.30667	0.29
56.06	0.24	0.25
56.08	0.17333	0.2
56.1	0.12	0.18
56.12	0.08667	0.14
56.14	0.08	0.2
56.16	0.09333	0.27
56.18	0.10667	0.36
56.2	0.14	0.42
56.22	0.18667	0.45
56.24	0.22	0.45
56.26	0.24667	0.48
56.28	0.25333	0.39
56.3	0.23333	0.31
56.32	0.2	0.21
56.34	0.17333	0.14
56.36	0.16	0.14
56.38	0.16	0.1

56.4	0.16667	0.14
56.42	0.16	0.15
56.44	0.14	0.11
56.46	0.10667	0.1
56.48	0.07333	0.07
56.5	0.05333	0.05
56.52	0.04667	0.08
56.54	0.04	0.07
56.56	0.03333	0.09
56.58	0.02	0.08
56.6	0.00667	0.11
56.62	0	0.14
56.64	0.00667	0.18
56.66	0.04	0.16
56.68	0.08667	0.25
56.7	0.14	0.26
56.72	0.19333	0.25
56.74	0.22	0.25
56.76	0.21333	0.28
56.78	0.17333	0.23
56.8	0.13333	0.22
56.82	0.08667	0.18
56.84	0.04667	0.25
56.86	0.02	0.22
56.88	0.01333	0.21
56.9	0.00667	0.26
56.92	0.02	0.26
56.94	0.04667	0.24
56.96	0.08667	0.2
56.98	0.13333	0.17

57	0.17333	0.17
57.02	0.2	0.15
57.04	0.20667	0.15
57.06	0.18667	0.1
57.08	0.16	0.12
57.1	0.11333	0.14
57.12	0.06667	0.15
57.14	0.03333	0.21
57.16	0.02667	0.29
57.18	0.03333	0.28
57.2	0.05333	0.2
57.22	0.08	0.17
57.24	0.1	0.17
57.26	0.1	0.14
57.28	0.1	0.18
57.3	0.1	0.19
57.32	0.10667	0.15
57.34	0.10667	0.19
57.36	0.11333	0.23
57.38	0.11333	0.3
57.4	0.1	0.34
57.42	0.07333	0.36
57.44	0.06667	0.33
57.46	0.08	0.27
57.48	0.09333	0.23
57.5	0.10667	0.25
57.52	0.10667	0.22
57.54	0.1	0.18
57.56	0.08	0.23
57.58	0.06	0.18

57.6	0.05333	0.18
57.62	0.06667	0.23
57.64	0.08667	0.29
57.66	0.12	0.28
57.68	0.14667	0.3
57.7	0.16	0.28
57.72	0.15333	0.28
57.74	0.14	0.19
57.76	0.11333	0.15
57.78	0.1	0.12
57.8	0.08667	0.08
57.82	0.1	0.03
57.84	0.10667	0.02
57.86	0.11333	0.03
57.88	0.10667	0.05
57.9	0.09333	0.12
57.92	0.08	0.21
57.94	0.06667	0.3
57.96	0.06	0.39
57.98	0.06	0.42
58	0.07333	0.37
58.02	0.08	0.29
58.04	0.08	0.21
58.06	0.06667	0.19
58.08	0.06667	0.16
58.1	0.07333	0.11
58.12	0.07333	0.09
58.14	0.08667	0.07
58.16	0.1	0.07
58.18	0.10667	0.09

58.2	0.09333	0.11

58.22	0.08	0.13

58.24	0.08667	0.13

58.26	0.09333	0.18

58.28	0.1	0.21

58.3	0.10667	0.2

58.32	0.12	0.22

58.34	0.13333	0.26

58.36	0.12667	0.21

58.38	0.10667	0.16

58.4	0.1	0.16

58.42	0.08667	0.22

58.44	0.08667	0.22

58.46	0.09333	0.14

58.48	0.12	0.11

58.5	0.15333	0.12

58.52	0.17333	0.06

58.54	0.16667	0.02

58.56	0.16	0.01

58.58	0.14	0.02

58.6	0.12	0.01

58.62	0.1	0

58.64	0.09333	0.02

58.66	0.1	0.06

58.68	0.10667	0.07

58.7	0.11333	0.06

58.72	0.10667	0.09

58.74	0.1	0.09

58.76	0.09333	0.1

58.78	0.08667	0.09

58.8	0.08667	0.12
58.82	0.08667	0.14
58.84	0.08	0.1
58.86	0.05333	0.08
58.88	0.03333	0.1
58.9	0.02	0.05
58.92	0.02	0.03
58.94	0.04	0.04
58.96	0.06667	0.1
58.98	0.10667	0.13
59	0.13333	0.12
59.02	0.14	0.14
59.04	0.14	0.1
59.06	0.14667	0.09
59.08	0.15333	0.12
59.1	0.15333	0.17
59.12	0.15333	0.2
59.14	0.16	0.19
59.16	0.14667	0.15
59.18	0.12667	0.08
59.2	0.1	0.03
59.22	0.07333	0.02
59.24	0.05333	0.03
59.26	0.03333	0.04
59.28	0.02667	0.11
59.3	0.02	0.17
59.32	0.02	0.18
59.34	0.02667	0.13
59.36	0.04	0.14
59.38	0.06	0.14

59.4	0.08	0.09
59.42	0.1	0.09
59.44	0.12	0.13
59.46	0.12	0.09
59.48	0.11333	0.09
59.5	0.10667	0.05
59.52	0.10667	0.04
59.54	0.10667	0.06
59.56	0.12667	0.05
59.58	0.15333	0.11
59.6	0.17333	0.17
59.62	0.19333	0.17
59.64	0.21333	0.16
59.66	0.18667	0.19
59.68	0.15333	0.2
59.7	0.10667	0.18
59.72	0.06667	0.14
59.74	0.04	0.14
59.76	0.02	0.1
59.78	0.03333	0.1
59.8	0.05333	0.07
59.82	0.07333	0.11
59.84	0.09333	0.13
59.86	0.1	0.11
59.88	0.09333	0.05
59.9	0.08	0.02
59.92	0.05333	0
59.94	0.04	0.04
59.96	0.03333	0.05
59.98	0.04667	0.09

60 0.06 0.18

Figure S19

For the secretion expression of TNF-α:

Control LPFEG DPFEG LPS LPS+LPFEG LPS+DPFEG LPS+L-SA LPS+D-SALPS+SA

0.20600000 0.20600000 0.093000 103.991000 106.1480000 117.5620000 85.25300000

67.68400000 66.542000

0.09300000 0.14800000 0.042000 100.767000 105.2030000 116.1950000 88.27500000

68.71400000 59.632000

0.14907370 0.17661630 0.067087 102.379300 105.6754000 116.8786000 86.76392000

68.19880000 62.323000

For the secretion expression of IL-1β:

Control LPFEG DPFEG LPS LPS+LPFEG LPS+DPFEG LPS+L-SA LPS+D-SALPS+SA

3.7360000 3.069000 5.392000 303.0220000 225.4970000 293.3190000 28.9480000

31.8120000 32.362000

3.4030000 3.403000 4.731000 316.3540000 230.3760000 284.5200000 37.2070000

27.3540000 26.342000

3.5694020 3.236133 5.061568 309.6880000 227.9363000 288.9195000 33.0771900

29.5828600 25.189000

Figure S23

For 1 h

Control SD 5% L SD 5% D SD

4.28449 0.245 3.54359 0.304 3.7037 0.259

For 6 h

Control SD 5% L SD 5% D SD

4.13223 0.215 2.22025 0.22 2.62055 0.377

Figure S24

For 1 h

5% L	5% D
0.482	0.491
0.497	0.534
0.461	0.445
0.494	0.449
0.441	0.495

5% L	SD	5% D	SD
0.475	0.0237	0.4828	0.037

For 6 h

5% L	5% D
0.345	0.412
0.357	0.408
0.36	0.415
0.361	0.419
0.364	0.411

5% L	SD	5% D	SD
0.3574	0.00737	0.413	0.00418

Figure S25

Temperature	NP	L	L-NP	D	D-NP
20.20678	-0.59968	-0.90663	-0.79401	-1.79429	-0.85682
20.20586	-0.59964	-0.90633	-0.79421	-1.78902	-0.85566
20.20437	-0.5996	-0.90587	-0.79471	-1.78332	-0.85425
20.20487	-0.59956	-0.90536	-0.79491	-1.77815	-0.85195
20.20397	-0.59952	-0.90496	-0.79514	-1.77325	-0.8485
20.20408	-0.59946	-0.90472	-0.79547	-1.76884	-0.84524
20.20384	-0.59944	-0.90452	-0.7959	-1.76513	-0.84333
20.2044	-0.59942	-0.90435	-0.79653	-1.7629	-0.84256
20.20264	-0.59942	-0.90422	-0.79817	-1.76226	-0.84173
20.20421	-0.59948	-0.90414	-0.80219	-1.76237	-0.84123

20.20478	-0.59968	-0.90408	-0.80931	-1.76278	-0.841
20.205	-0.60034	-0.90403	-0.8185	-1.76308	-0.84084
20.2068	-0.60152	-0.90392	-0.82769	-1.76333	-0.84073
20.20954	-0.60284	-0.90366	-0.8337	-1.76359	-0.84064
20.21262	-0.60382	-0.90213	-0.83655	-1.76365	-0.84057
20.21763	-0.60414	-0.89487	-0.83762	-1.7633	-0.84052
20.22253	-0.60418	-0.872	-0.83769	-1.76082	-0.84052
20.22983	-0.6041	-0.83035	-0.83714	-1.75121	-0.84055
20.23592	-0.60252	-0.77854	-0.83276	-1.72769	-0.84062
20.24468	-0.59414	-0.73575	-0.80774	-1.6851	-0.84069
20.2537	-0.5744	-0.68959	-0.754	-1.64239	-0.84074
20.26422	-0.5541	-0.63799	-0.70352	-1.60534	-0.84077
20.27636	-0.5373	-0.58074	-0.66532	-1.56255	-0.8408
20.28926	-0.51798	-0.52307	-0.62268	-1.51546	-0.84083
20.30285	-0.4963	-0.45823	-0.57667	-1.46852	-0.84085
20.31868	-0.47306	-0.39327	-0.52817	-1.41547	-0.84088
20.33426	-0.44924	-0.32367	-0.47643	-1.36126	-0.84093
20.35002	-0.42546	-0.25318	-0.42319	-1.30681	-0.841
20.36821	-0.40152	-0.17931	-0.37117	-1.25098	-0.84109
20.38559	-0.37674	-0.10835	-0.32175	-1.19623	-0.84127
20.40594	-0.35174	-0.03799	-0.27258	-1.143	-0.84163
20.42562	-0.32796	0.02961	-0.22385	-1.09018	-0.84236
20.44392	-0.30466	0.09485	-0.17866	-1.03756	-0.84367
20.46489	-0.28186	0.16004	-0.13577	-0.98794	-0.84576
20.48582	-0.2608	0.22143	-0.09422	-0.94434	-0.84965
20.50639	-0.24114	0.27804	-0.05889	-0.90248	-0.85658
20.52866	-0.22304	0.33012	-0.02818	-0.86427	-0.86646
20.55018	-0.2067	0.37822	-1.94502E-4	-0.82962	-0.87897
20.57101	-0.19094	0.42231	0.02515	-0.79315	-0.89516
20.5934	-0.1771	0.46334	0.04558	-0.76517	-0.91182

20.61566	-0.16492	0.50149	0.06235	-0.74056	-0.92737
20.63934	-0.15566	0.53437	0.07468	-0.71743	-0.94096
20.66121	-0.1481	0.56536	0.08532	-0.69977	-0.95505
20.68454	-0.141	0.59239	0.08994	-0.68106	-0.97065
20.70784	-0.13418	0.61536	0.09486	-0.66614	-0.98752
20.73058	-0.1284	0.63767	0.09917	-0.65295	-1.00191
20.75366	-0.12472	0.66019	0.10085	-0.64229	-1.0167
20.77756	-0.12248	0.68136	0.09841	-0.6355	-1.02979
20.80042	-0.1212	0.69811	0.09524	-0.62768	-1.04318
20.82405	-0.1205	0.71408	0.08941	-0.61681	-1.05999
20.84742	-0.12002	0.72572	0.08245	-0.60926	-1.07288
20.87054	-0.12068	0.73619	0.0744	-0.60347	-1.08729
20.89491	-0.12176	0.7468	0.06231	-0.60167	-1.10017
20.92008	-0.12174	0.75896	0.05224	-0.60023	-1.11192
20.94443	-0.1227	0.77006	0.04163	-0.59845	-1.12819
20.96854	-0.1241	0.77944	0.03108	-0.5952	-1.14391
20.9942	-0.12674	0.78885	0.0181	-0.59277	-1.15655
21.01898	-0.12942	0.79345	0.00697	-0.59179	-1.17023
21.04472	-0.13338	0.79794	-0.00556	-0.59122	-1.18317
21.07002	-0.13808	0.80533	-0.02054	-0.5934	-1.1965
21.09526	-0.141	0.8108	-0.03599	-0.5941	-1.2101
21.12123	-0.1446	0.81709	-0.05355	-0.59395	-1.22584
21.14761	-0.14896	0.82444	-0.073	-0.59367	-1.24115
21.17384	-0.15388	0.82925	-0.08935	-0.59472	-1.25638
21.20096	-0.16026	0.83228	-0.10722	-0.60111	-1.26933
21.2271	-0.16614	0.83129	-0.12783	-0.60626	-1.28072
21.25339	-0.1705	0.8304	-0.14693	-0.60786	-1.29124
21.2796	-0.175	0.83198	-0.16705	-0.61044	-1.30205
21.30606	-0.18124	0.8337	-0.18813	-0.61558	-1.31471
21.33357	-0.18798	0.833	-0.20942	-0.6206	-1.32807

21.36142	-0.19482	0.83353	-0.22992	-0.62639	-1.3443
21.38822	-0.20176	0.83519	-0.25384	-0.63137	-1.35786
21.4157	-0.20822	0.83445	-0.27833	-0.63511	-1.36806
21.44292	-0.2143	0.83288	-0.30051	-0.63952	-1.37829
21.46993	-0.22092	0.82603	-0.32475	-0.64618	-1.38679
21.49797	-0.22876	0.81793	-0.34769	-0.6545	-1.39761
21.52653	-0.23558	0.81311	-0.36715	-0.66292	-1.4094
21.5524	-0.24342	0.80652	-0.39105	-0.66939	-1.41992
21.58152	-0.25132	0.8004	-0.41491	-0.67585	-1.42857
21.60922	-0.259	0.79815	-0.44011	-0.68291	-1.4411
21.63878	-0.2674	0.79553	-0.46693	-0.69069	-1.45358
21.66574	-0.27562	0.79062	-0.48918	-0.69936	-1.46657
21.694	-0.28422	0.78602	-0.51414	-0.70599	-1.4744
21.72162	-0.29264	0.7792	-0.53792	-0.71205	-1.48416
21.75057	-0.30082	0.77275	-0.5625	-0.7193	-1.49135
21.77898	-0.30888	0.76734	-0.58627	-0.72548	-1.49936
21.8072	-0.31764	0.76036	-0.60869	-0.73341	-1.50678
21.83515	-0.32592	0.75295	-0.63069	-0.74176	-1.516
21.86278	-0.33444	0.74704	-0.654	-0.75008	-1.52376
21.89284	-0.34208	0.74084	-0.67713	-0.76038	-1.53135
21.922	-0.3488	0.73573	-0.70402	-0.77082	-1.53928
21.95076	-0.35622	0.73203	-0.72816	-0.77795	-1.54978
21.9789	-0.365	0.72894	-0.75336	-0.78468	-1.55771
22.00754	-0.37354	0.72562	-0.77547	-0.79086	-1.5669
22.03699	-0.38184	0.72017	-0.79606	-0.79605	-1.57146
22.06594	-0.38872	0.71352	-0.81839	-0.80383	-1.57923
22.09476	-0.3962	0.70738	-0.84199	-0.81053	-1.58466
22.12424	-0.40298	0.7009	-0.86481	-0.81487	-1.59152
22.15277	-0.41122	0.69464	-0.88569	-0.81892	-1.59673
22.18355	-0.41884	0.69085	-0.90727	-0.82372	-1.60343

22.21242	-0.42672	0.68707	-0.92975	-0.83206	-1.60931
22.24241	-0.43444	0.68413	-0.95282	-0.84252	-1.61409
22.27176	-0.44156	0.68023	-0.97685	-0.84894	-1.61716
22.30159	-0.44906	0.676-0.99928	-0.85311	-1.62013	
22.33037	-0.45562	0.67088	-1.01919	-0.85565	-1.62371
22.36116	-0.4618	0.66351	-1.03993	-0.85896	-1.62881
22.39111	-0.46802	0.6557	-1.05982	-0.86513	-1.63382
22.42024	-0.47526	0.65049	-1.07859	-0.87056	-1.63858
22.44999	-0.48222	0.64588	-1.10129	-0.87632	-1.64275
22.47991	-0.48958	0.64344	-1.12129	-0.87955	-1.6467
22.50928	-0.49612	0.64221	-1.14177	-0.88096	-1.6526
22.53906	-0.50134	0.63919	-1.15769	-0.88341	-1.65781
22.5684	-0.50884	0.63258	-1.17471	-0.88946	-1.66075
22.59834	-0.51492	0.6248	-1.19163	-0.89601	-1.66212
22.62962	-0.52078	0.61975	-1.21369	-0.90143	-1.66335
22.6593	-0.52618	0.61603	-1.2386	-0.90551	-1.66617
22.68956	-0.53192	0.61294	-1.25735	-0.90754	-1.66911
22.71964	-0.53846	0.60955	-1.27234	-0.90877	-1.67134
22.74882	-0.54526	0.6048	-1.28709	-0.91318	-1.67328
22.7795	-0.55126	0.60139	-1.30383	-0.91929	-1.6742
22.81146	-0.55742	0.59897	-1.32142	-0.92325	-1.67591
22.84149	-0.56292	0.59528	-1.3412	-0.92723	-1.67764
22.87125	-0.56846	0.59151	-1.3567	-0.92976	-1.67928
22.90139	-0.5748	0.58947	-1.37392	-0.93005	-1.68176
22.93148	-0.58012	0.58784	-1.39078	-0.93062	-1.68454
22.96192	-0.5842	0.58623	-1.40695	-0.93284	-1.68722
22.99225	-0.58852	0.58401	-1.42483	-0.93481	-1.6897
23.02375	-0.59344	0.57947	-1.44185	-0.93766	-1.69104
23.05357	-0.59928	0.57427	-1.45481	-0.94008	-1.69173
23.0847	-0.60418	0.57035	-1.46694	-0.94108	-1.69245

23.11543	-0.60934	0.56609	-1.47743	-0.94183	-1.69287
23.1465	-0.6133	0.56322	-1.48991	-0.94263	-1.693
23.17624	-0.61716	0.56177	-1.5044	-0.94331	-1.6931
23.20674	-0.62128	0.56057	-1.52205	-0.94402	-1.69327
23.23847	-0.62534	0.56003	-1.53594	-0.94465	-1.69364
23.26904	-0.62884	0.55897	-1.55208	-0.94542	-1.69413
23.29946	-0.6327	0.5565	-1.56559	-0.94597	-1.6945
23.32996	-0.63742	0.55438	-1.58001	-0.94624	-1.69472
23.36104	-0.64326	0.5528	-1.59243	-0.94656	-1.69485
23.3919	-0.6476	0.55147	-1.60293	-0.94694	-1.69501
23.4237	-0.65148	0.55003	-1.6148	-0.94734	-1.69511
23.45412	-0.65494	0.54856	-1.62625	-0.94774	-1.69525
23.48455	-0.65808	0.54694	-1.63775	-0.94791	-1.69543
23.51653	-0.66092	0.54583	-1.65067	-0.94816	-1.69553
23.54622	-0.66624	0.54527	-1.66412	-0.94822	-1.69568
23.5778	-0.66952	0.54466	-1.6765	-0.94839	-1.69567
23.6087	-0.67282	0.54408	-1.68551	-0.94856	-1.69565
23.63998	-0.67664	0.54289	-1.69391	-0.94863	-1.69566
23.67105	-0.6792	0.54148	-1.70613	-0.94882	-1.6957
23.70239	-0.68236	0.54081	-1.71569	-0.94888	-1.69575
23.73458	-0.6863	0.54044	-1.72565	-0.94894	-1.69564
23.76618	-0.68892	0.53986	-1.73647	-0.94893	-1.69535
23.79576	-0.69254	0.53961	-1.74644	-0.94889	-1.69493
23.82716	-0.6951	0.53905	-1.7591	-0.94883	-1.69448
23.85865	-0.69808	0.53862	-1.77246	-0.94871	-1.69419
23.89008	-0.70096	0.53836	-1.77781	-0.94855	-1.69406
23.92119	-0.70282	0.53815	-1.78449	-0.94827	-1.69397
23.95257	-0.70558	0.53797	-1.79083	-0.94795	-1.69389
23.98409	-0.70764	0.53768	-1.79859	-0.9474	-1.69362
24.01563	-0.7088	0.53744	-1.81105	-0.94657	-1.69315

24.04657	-0.71106	0.53734	-1.82186	-0.94584	-1.69247
24.07763	-0.71444	0.53734	-1.83104	-0.94548	-1.69194
24.1091	-0.71764	0.53729	-1.84062	-0.9452	-1.69155
24.14043	-0.72056	0.53721	-1.84438	-0.945	-1.69127
24.17214	-0.72266	0.53719	-1.85276	-0.94473	-1.69082
24.20366	-0.72348	0.53722	-1.86224	-0.94409	-1.68979
24.23525	-0.72396	0.53735	-1.86799	-0.94265	-1.68838
24.26568	-0.7247	0.53745	-1.87625	-0.94092	-1.68655
24.29702	-0.72606	0.53749	-1.88049	-0.93948	-1.685
24.32841	-0.72788	0.53753	-1.88434	-0.93755	-1.68378
24.36121	-0.72998	0.53759	-1.89226	-0.93512	-1.68233
24.39174	-0.73166	0.53778	-1.90069	-0.93175	-1.68082
24.42341	-0.73246	0.53841	-1.90962	-0.92674	-1.67876
24.45555	-0.73334	0.53903	-1.91737	-0.92341	-1.67641
24.48658	-0.7347	0.53956	-1.9262	-0.92188	-1.67352
24.51802	-0.73724	0.53981	-1.93009	-0.92064	-1.67096
24.54924	-0.73932	0.53992	-1.93348	-0.919	-1.66886
24.58111	-0.74092	0.54006	-1.93583	-0.91522	-1.66697
24.61261	-0.74174	0.54038	-1.94208	-0.90939	-1.66462
24.64421	-0.74258	0.54146	-1.95039	-0.90363	-1.66222
24.67648	-0.7435	0.54324	-1.95913	-0.89757	-1.65904
24.70803	-0.7447	0.54438	-1.96778	-0.8925	-1.65638
24.73938	-0.74564	0.54522	-1.97161	-0.88681	-1.65408
24.77037	-0.74638	0.54595	-1.97391	-0.87868	-1.65173
24.80287	-0.74704	0.54734	-1.97687	-0.87374	-1.64856
24.83394	-0.74766	0.5497	-1.97974	-0.87056	-1.64521
24.86633	-0.74844	0.55147	-1.9835	-0.86845	-1.64023
24.89668	-0.7492	0.55329	-1.98718	-0.86563	-1.6346
24.92991	-0.74982	0.55543	-1.99201	-0.86217	-1.63021
24.9615	-0.75052	0.55754	-1.99741	-0.85709	-1.62662

24.99354	-0.75108	0.56036	-2.0016	-0.85205	-1.62371
25.02505	-0.75146	0.56413	-2.00592	-0.84664	-1.62167
25.05641	-0.75206	0.5675	-2.00982	-0.84115	-1.61848
25.08806	-0.75266	0.57039	-2.01246	-0.83583	-1.61602
25.12048	-0.7533	0.57139	-2.01557	-0.83058	-1.61293
25.15238	-0.75396	0.57199	-2.01842	-0.82573	-1.60894
25.18531	-0.75438	0.57247	-2.02158	-0.82175	-1.60562
25.21627	-0.75462	0.57401	-2.02457	-0.81684	-1.60129
25.24694	-0.7548	0.57818	-2.02669	-0.81064	-1.59772
25.27933	-0.75506	0.58321	-2.02812	-0.80595	-1.59396
25.31116	-0.7552	0.58595	-2.02956	-0.80183	-1.58924
25.34343	-0.75534	0.58775	-2.03122	-0.79939	-1.58481
25.37495	-0.75546	0.58914	-2.03384	-0.7972	-1.5825
25.40703	-0.75556	0.5912	-2.03838	-0.79255	-1.58096
25.4396	-0.75562	0.59462	-2.04393	-0.78717	-1.57913
25.47078	-0.7557	0.59843	-2.04839	-0.78235	-1.57604
25.50271	-0.75582	0.60193	-2.05182	-0.77796	-1.57266
25.53465	-0.75596	0.60533	-2.05392	-0.77547	-1.5677
25.56594	-0.7561	0.60923	-2.05531	-0.77171	-1.56145
25.59921	-0.75638	0.61408	-2.05638	-0.76629	-1.55533
25.6308	-0.75652	0.61891	-2.05721	-0.7604	-1.55165
25.66288	-0.75662	0.62173	-2.05787	-0.75269	-1.55022
25.69502	-0.75674	0.62397	-2.05921	-0.74481	-1.54955
25.72662	-0.75678	0.62609	-2.06172	-0.74015	-1.54823
25.75821	-0.75684	0.62953	-2.06598	-0.73567	-1.54547
25.79056	-0.7569	0.63379	-2.06965	-0.73197	-1.54092
25.82214	-0.75702	0.6379	-2.07201	-0.72854	-1.53606
25.85424	-0.75712	0.64183	-2.07329	-0.72387	-1.53136
25.8872	-0.75718	0.64483	-2.07373	-0.71826	-1.52731
25.91814	-0.75722	0.6486	-2.07415	-0.71238	-1.52364

25.95052	-0.75726	0.65379	-2.07446	-0.7047	-1.52151
25.98245	-0.75726	0.65762	-2.0746	-0.7012	-1.51985
26.01462	-0.75724	0.66093	-2.07494	-0.69793	-1.51695
26.04727	-0.75722	0.66452	-2.07643	-0.69405	-1.51281
26.07867	-0.7572	0.66795	-2.07934	-0.68918	-1.50937
26.11078	-0.75712	0.67279	-2.08192	-0.68519	-1.50602
26.1436	-0.75702	0.67746	-2.08319	-0.67934	-1.50254
26.17533	-0.75694	0.68169	-2.08357	-0.67383	-1.49926
26.20694	-0.75684	0.68508	-2.08414	-0.66712	-1.495
26.23983	-0.75676	0.68735	-2.08439	-0.66005	-1.49068
26.27162	-0.7566	0.68924	-2.08455	-0.65493	-1.48832
26.30336	-0.75636	0.69175	-2.0847	-0.65073	-1.48572
26.33538	-0.75608	0.69634	-2.08528	-0.64786	-1.48297
26.36774	-0.75592	0.70284	-2.08607	-0.64471	-1.48008
26.40049	-0.75584	0.70743	-2.08676	-0.63962	-1.47719
26.43199	-0.75572	0.70975	-2.08712	-0.63383	-1.47358
26.46449	-0.75544	0.71113	-2.08734	-0.62727	-1.46874
26.49652	-0.75496	0.71459	-2.08751	-0.62106	-1.46223
26.52836	-0.75448	0.72013	-2.08765	-0.61482	-1.45481
26.56047	-0.75394	0.72637	-2.08777	-0.61026	-1.4491
26.59233	-0.7535	0.73209	-2.08797	-0.6065	-1.44601
26.62497	-0.75322	0.73437	-2.08826	-0.60192	-1.44357
26.65753	-0.75304	0.73524	-2.08853	-0.59774	-1.44241
26.68936	-0.75286	0.73718	-2.08879	-0.5906	-1.4416
26.72191	-0.75248	0.7399	-2.08898	-0.58336	-1.43948
26.75319	-0.75158	0.74535	-2.0894	-0.57723	-1.43744
26.78654	-0.7501	0.75312	-2.0897	-0.57027	-1.43509
26.81744	-0.74792	0.75952	-2.0899	-0.56665	-1.4319
26.85012	-0.74678	0.76328	-2.09022	-0.56129	-1.42731
26.88288	-0.74608	0.76544	-2.09038	-0.55551	-1.4224

26.91326	-0.7456	0.76678	-2.09057	-0.54996	-1.41737
26.94561	-0.74498	0.76763	-2.09073	-0.54598	-1.41391
26.97826	-0.7442	0.76986	-2.0909	-0.5426	-1.41134
27.01072	-0.7431	0.77461	-2.09111	-0.53902	-1.40846
27.04368	-0.74242	0.78089	-2.09131	-0.53242	-1.404
27.07534	-0.74168	0.78618	-2.09146	-0.52484	-1.40051
27.10752	-0.74056	0.78971	-2.09153	-0.51724	-1.39778
27.14045	-0.73882	0.79229	-2.09154	-0.511	-1.39537
27.17182	-0.73738	0.79565	-2.09154	-0.50624	-1.3928
27.20506	-0.73638	0.79982	-2.09152	-0.50151	-1.39021
27.2367	-0.73584	0.80283	-2.09156	-0.49703	-1.38743
27.26925	-0.73546	0.80525	-2.09163	-0.49268	-1.3847
27.30242	-0.73504	0.80762	-2.09169	-0.48617	-1.38048
27.3343	-0.73418	0.8124	-2.09172	-0.47997	-1.37557
27.36503	-0.73258	0.81951	-2.09171	-0.47255	-1.36989
27.39868	-0.7303	0.82677	-2.0917	-0.46526	-1.36656
27.43018	-0.72806	0.83159	-2.09168	-0.46076	-1.36492
27.46338	-0.72662	0.83538	-2.09167	-0.45775	-1.36393
27.49508	-0.72568	0.83911	-2.09165	-0.45494	-1.36202
27.52711	-0.72472	0.84293	-2.09163	-0.45118	-1.35828
27.5594	-0.72364	0.84656	-2.0916	-0.4455	-1.35288
27.59278	-0.72222	0.85125	-2.09154	-0.44006	-1.34796
27.62452	-0.7211	0.85564	-2.09143	-0.43473	-1.34414
27.65671	-0.72006	0.85814	-2.0912	-0.42851	-1.34124
27.68947	-0.71874	0.8598	-2.09095	-0.42323	-1.33691
27.721	-0.71728	0.8612	-2.09082	-0.41648	-1.33094
27.75381	-0.7157	0.86484	-2.09069	-0.40864	-1.32559
27.78638	-0.71416	0.87044	-2.09059	-0.40307	-1.32199
27.8175	-0.71298	0.87616	-2.0905	-0.39982	-1.32056
27.85047	-0.71144	0.88277	-2.09036	-0.39632	-1.3196

27.88358	-0.70962	0.88795	-2.09019	-0.39244	-1.31734
27.91555	-0.70796	0.89228	-2.08985	-0.3875	-1.31353
27.94754	-0.70656	0.89625	-2.08957	-0.38088	-1.31007
27.97915	-0.70532	0.89881	-2.08939	-0.37338	-1.30702
28.01106	-0.70444	0.90004	-2.08912	-0.36732	-1.30471
28.04466	-0.70348	0.90122	-2.08865	-0.36274	-1.30308
28.07695	-0.70224	0.90245	-2.08756	-0.35899	-1.30041
28.10938	-0.70006	0.90457	-2.08647	-0.3561	-1.29537
28.14122	-0.6971	0.90993	-2.08578	-0.35466	-1.28997
28.1745	-0.6936	0.9177	-2.08546	-0.35003	-1.28534
28.20728	-0.69184	0.92483	-2.08518	-0.34081	-1.28279
28.23904	-0.69136	0.93014	-2.08488	-0.33181	-1.28157
28.27217	-0.69092	0.93289	-2.08455	-0.3249	-1.27975
28.30356	-0.6905	0.93448	-2.08372	-0.3208	-1.27765
28.33569	-0.69002	0.9357	-2.08206	-0.3173	-1.27483
28.36758	-0.68914	0.93685	-2.07977	-0.31265	-1.27066
28.39976	-0.68758	0.93784	-2.07749	-0.3058	-1.26633
28.43287	-0.686	0.94064	-2.07594	-0.29668	-1.26198
28.46552	-0.68432	0.94441	-2.07506	-0.29239	-1.25646
28.49787	-0.6833	0.94982	-2.0745	-0.28889	-1.25309
28.5321	-0.68304	0.95849	-2.07416	-0.28594	-1.25013
28.56394	-0.6827	0.96475	-2.07352	-0.2829	-1.24768
28.59485	-0.68192	0.97032	-2.07285	-0.27795	-1.24574
28.6278	-0.68036	0.97338	-2.07244	-0.2693	-1.24364
28.66026	-0.67798	0.97537	-2.07181	-0.26281	-1.24117
28.69348	-0.67542	0.97821	-2.0704	-0.25941	-1.23907
28.72608	-0.67382	0.98225	-2.06798	-0.25504	-1.23621
28.75738	-0.6728	0.98558	-2.06506	-0.2525	-1.23176
28.79029	-0.67162	0.98818	-2.06331	-0.24831	-1.2267
28.82263	-0.66998	0.99004	-2.0627	-0.23959	-1.22251

28.85558	-0.66774	0.99349	-2.06228	-0.23125	-1.22014
28.88703	-0.66608	0.99948	-2.06158	-0.22358	-1.21746
28.91927	-0.66512	1.00651	-2.06006	-0.21848	-1.21367
28.95203	-0.6649	1.01264	-2.05724	-0.21589	-1.2099
28.98386	-0.6645	1.01713	-2.05306	-0.21316	-1.20548
29.01761	-0.66338	1.02129	-2.05114	-0.21101	-1.20278
29.04972	-0.6614	1.02532	-2.05027	-0.20824	-1.20203
29.08168	-0.6586	1.02763	-2.0494	-0.20424	-1.20105
29.11415	-0.65606	1.03056	-2.04802	-0.19698	-1.19997
29.14736	-0.65478	1.03352	-2.04593	-0.18991	-1.19807
29.17915	-0.65364	1.03819	-2.04387	-0.18394	-1.19473
29.21232	-0.65252	1.0439	-2.04257	-0.17969	-1.19135
29.24488	-0.65156	1.04864	-2.04187	-0.17574	-1.18803
29.27722	-0.6499	1.05134	-2.04106	-0.17	-1.18359
29.30977	-0.6483	1.05314	-2.03906	-0.16103	-1.17898
29.34183	-0.64724	1.05485	-2.03547	-0.1507	-1.1737
29.37462	-0.64626	1.0577	-2.03245	-0.14596	-1.1695
29.40639	-0.64496	1.06194	-2.03035	-0.1457	-1.16707
29.43909	-0.64346	1.06855	-2.02912	-0.14608	-1.16554
29.47159	-0.64224	1.07494	-2.02858	-0.14412	-1.16413
29.50475	-0.64128	1.07829	-2.02786	-0.13943	-1.16241
29.53646	-0.64044	1.08072	-2.02663	-0.1329	-1.15999
29.56956	-0.63936	1.08481	-2.02497	-0.12617	-1.15615
29.60205	-0.63744	1.08701	-2.02277	-0.11926	-1.1518
29.63448	-0.63474	1.08886	-2.02014	-0.11134	-1.14762
29.66682	-0.63238	1.09041	-2.01814	-0.10236	-1.14507
29.69874	-0.62994	1.09179	-2.01556	-0.09666	-1.14264
29.73166	-0.62804	1.09492	-2.01287	-0.09258	-1.14056
29.76348	-0.62666	1.09796	-2.00966	-0.08876	-1.13796
29.796	-0.62556	1.09996	-2.00594	-0.08529	-1.1349

29.82869	-0.62506	1.10244	-2.00327	-0.08136	-1.13144
29.86177	-0.62458	1.10703	-2.00154	-0.07911	-1.128
29.89341	-0.6235	1.11457	-2.00054	-0.07816	-1.12444
29.92715	-0.62178	1.12352	-1.99974	-0.07622	-1.12161
29.95951	-0.62004	1.12844	-1.99881	-0.07324	-1.12017
29.9915	-0.6185	1.13035	-1.99757	-0.06598	-1.11847
30.0244	-0.61768	1.13225	-1.99612	-0.05476	-1.11591
30.0572	-0.61728	1.13408	-1.99445	-0.04923	-1.11193
30.08985	-0.61666	1.13579	-1.99364	-0.04503	-1.1067
30.12222	-0.61544	1.13825	-1.99293	-0.04021	-1.10207
30.15501	-0.61414	1.14013	-1.99241	-0.03554	-1.09984
30.18756	-0.61218	1.1429	-1.99187	-0.03033	-1.09829
30.22062	-0.61008	1.14807	-1.9909	-0.0239	-1.09662
30.25261	-0.60744	1.1542	-1.98886	-0.0185	-1.09418
30.2854	-0.60582	1.16003	-1.98575	-0.01355	-1.09116
30.31745	-0.60504	1.1648	-1.98087	-0.01044	-1.08803
30.35004	-0.60464	1.16958	-1.97625	-0.00735	-1.08546
30.38224	-0.604	1.17443	-1.97367	-0.00467	-1.084
30.4156	-0.60282	1.18064	-1.97227	-0.00219	-1.0822
30.44801	-0.60132	1.18434	-1.97161	0.00138	-1.07961
30.48056	-0.5991	1.18769	-1.9711	0.00799	-1.07647
30.5125	-0.59708	1.19066	-1.97072	0.0179	-1.07401
30.5455	-0.59598	1.19364	-1.97027	0.02614	-1.07194
30.57846	-0.59506	1.19633	-1.96923	0.02888	-1.06961
30.61074	-0.59434	1.19911	-1.96741	0.02876	-1.06759
30.64363	-0.59376	1.20091	-1.96457	0.02971	-1.06511
30.67628	-0.5923	1.2034	-1.96155	0.03281	-1.06144
30.70824	-0.59038	1.20565	-1.95963	0.04052	-1.05897
30.74089	-0.58914	1.2075	-1.95767	0.047	-1.05619
30.7729	-0.5877	1.2099	-1.95555	0.05025	-1.05303

30.80587	-0.58656	1.21229	-1.95401	0.05304	-1.04954
30.83835	-0.58534	1.21535	-1.95306	0.05593	-1.04577
30.87131	-0.5832	1.22113	-1.95229	0.06006	-1.04142
30.90469	-0.58134	1.2273	-1.95114	0.06551	-1.03785
30.93734	-0.57984	1.23249	-1.94868	0.07247	-1.03476
30.96996	-0.57768	1.23632	-1.94369	0.0788	-1.0319
31.00162	-0.5761	1.23782	-1.93778	0.08265	-1.0295
31.03482	-0.57494	1.23994	-1.933	0.08534	-1.02793
31.06743	-0.57398	1.24246	-1.93176	0.09122	-1.02628
31.09947	-0.57298	1.24552	-1.93127	0.09647	-1.02432
31.13228	-0.572	1.24929	-1.93091	0.10288	-1.02266
31.16506	-0.57026	1.25215	-1.93037	0.10877	-1.0203
31.19797	-0.5681	1.25472	-1.92965	0.11065	-1.01772
31.23034	-0.56656	1.25804	-1.92845	0.11208	-1.01517
31.263	-0.56528	1.26065	-1.92703	0.11542	-1.01211
31.2972	-0.56408	1.2634	-1.92472	0.12165	-1.0082
31.32944	-0.56338	1.26617	-1.91976	0.13025	-1.00435
31.36199	-0.56236	1.26836	-1.9159	0.13795	-1.00135
31.3933	-0.56022	1.27162	-1.91358	0.14087	-0.99961
31.42732	-0.55812	1.2765	-1.91237	0.14237	-0.99867
31.4601	-0.55598	1.28032	-1.91168	0.14534	-0.99799
31.49256	-0.55442	1.28422	-1.91101	0.14925	-0.9954
31.52463	-0.55402	1.28883	-1.9105	0.15392	-0.99328
31.55665	-0.55386	1.29154	-1.90945	0.15674	-0.99027
31.58998	-0.55302	1.29296	-1.90718	0.15908	-0.98679
31.62284	-0.55104	1.29411	-1.90399	0.16275	-0.98406
31.65624	-0.5487	1.29573	-1.90217	0.16707	-0.98158
31.6872	-0.54704	1.29859	-1.9015	0.17252	-0.97942
31.71969	-0.5458	1.30219	-1.90076	0.17594	-0.97854
31.75256	-0.54564	1.30535	-1.89945	0.17867	-0.97757

31.78517	-0.5456	1.30679	-1.89804	0.18164	-0.97656
31.818	-0.54542	1.30814	-1.89648	0.18444	-0.97525
31.85047	-0.54526	1.31076	-1.89487	0.18806	-0.97252
31.88265	-0.54466	1.31558	-1.8934	0.1941	-0.96709
31.91467	-0.54334	1.32046	-1.89146	0.20137	-0.96197
31.94863	-0.54192	1.32561	-1.88955	0.20646	-0.95704
31.982	-0.54056	1.32912	-1.88842	0.21143	-0.95273
32.01382	-0.5394	1.33121	-1.88712	0.21443	-0.95031
32.04698	-0.53864	1.33443	-1.88565	0.21756	-0.94784
32.07874	-0.53752	1.33745	-1.88337	0.22335	-0.94609
32.11122	-0.53566	1.34106	-1.88096	0.22928	-0.94563
32.1439	-0.53424	1.34513	-1.87929	0.23419	-0.94539
32.17653	-0.53314	1.34933	-1.87807	0.2376	-0.94495
32.21047	-0.5321	1.35194	-1.87663	0.23957	-0.94357
32.24253	-0.53108	1.35425	-1.87437	0.24267	-0.94112
32.27496	-0.52888	1.35657	-1.87218	0.24836	-0.93698
32.30782	-0.52636	1.35865	-1.87078	0.25671	-0.93247
32.34072	-0.52432	1.3626	-1.86915	0.26434	-0.9292
32.37322	-0.5225	1.36921	-1.86714	0.26894	-0.92621
32.4062	-0.52156	1.37447	-1.86419	0.27166	-0.92261
32.43842	-0.52052	1.37894	-1.86083	0.27412	-0.92028
32.47131	-0.51914	1.38291	-1.85903	0.2784	-0.91876
32.50398	-0.51752	1.386-1.8579	0.28283	-0.91808	
32.53624	-0.51538	1.38774	-1.85724	0.28864	-0.91775
32.56926	-0.51388	1.3904	-1.85663	0.29154	-0.91695
32.60187	-0.5131	1.3938	-1.85538	0.29334	-0.91429
32.6351	-0.5125	1.3974	-1.85195	0.29542	-0.90924
32.66693	-0.51166	1.40374	-1.84735	0.29778	-0.90346
32.69958	-0.51028	1.40765	-1.84504	0.30199	-0.89958
32.73406	-0.5087	1.40789	-1.84433	0.30739	-0.89798

32.76518	-0.50748	1.40846	-1.8437	0.31147	-0.8974
32.79838	-0.5066	1.40995	-1.84307	0.31778	-0.89726
32.8309	-0.50552	1.4138	-1.84113	0.32137	-0.89702
32.86328	-0.50402	1.42084	-1.83732	0.32391	-0.89633
32.89632	-0.50226	1.42553	-1.83393	0.32665	-0.8947
32.92874	-0.50098	1.42833	-1.83239	0.32967	-0.89121
32.96142	-0.50046	1.43131	-1.83146	0.33373	-0.88674
32.99535	-0.50022	1.43537	-1.83092	0.33859	-0.88152
33.02776	-0.49968	1.4393	-1.8303	0.34182	-0.87708
33.06057	-0.4981	1.44228	-1.82961	0.3452	-0.87458
33.09388	-0.49534	1.44402	-1.82905	0.34954	-0.87336
33.12512	-0.49238	1.44733	-1.82862	0.35545	-0.87263
33.15844	-0.4906	1.45088	-1.82789	0.35837	-0.87207
33.19086	-0.48914	1.45323	-1.82658	0.36026	-0.87079
33.22419	-0.4876	1.45555	-1.82483	0.36235	-0.86875
33.25704	-0.48654	1.45653	-1.82359	0.36555	-0.86641
33.28884	-0.48564	1.45814	-1.82284	0.37194	-0.86517
33.32214	-0.48502	1.46127	-1.82183	0.37915	-0.86377
33.35467	-0.48448	1.46396	-1.8203	0.38459	-0.86286
33.38762	-0.48384	1.46562	-1.81887	0.38897	-0.86112
33.42019	-0.48302	1.46636	-1.81666	0.39316	-0.8583
33.45372	-0.48224	1.4672	-1.81545	0.39643	-0.85521
33.48532	-0.48166	1.47043	-1.81445	0.39764	-0.8527
33.51846	-0.48092	1.47731	-1.81359	0.39857	-0.85081
33.55082	-0.4799	1.4845	-1.81297	0.40088	-0.84923
33.584	-0.47878	1.48939	-1.81214	0.4043	-0.84806
33.61755	-0.4773	1.49087	-1.81097	0.40919	-0.84661
33.64924	-0.47562	1.49121	-1.8095	0.41416	-0.84431
33.68328	-0.4743	1.49291	-1.80786	0.41726	-0.84194
33.71524	-0.4729	1.49592	-1.80583	0.42	-0.83986

33.74774	-0.47174	1.49962	-1.80352	0.42328	-0.83763
33.78002	-0.47082	1.50217	-1.80214	0.42653	-0.83554
33.81274	-0.46986	1.5031	-1.80079	0.42974	-0.83359
33.84496	-0.46862	1.50409	-1.79928	0.43262	-0.83249
33.87847	-0.4677	1.50709	-1.79627	0.43507	-0.83163
33.9117	-0.46702	1.51447	-1.79192	0.43888	-0.83035
33.943	-0.46598	1.5256	-1.78755	0.44485	-0.8285
33.97687	-0.46498	1.53428	-1.78414	0.45136	-0.82511
34.00894	-0.46366	1.53774	-1.78289	0.45625	-0.82285
34.04282	-0.4626	1.53797	-1.78233	0.45871	-0.8213
34.07451	-0.46216	1.53751	-1.78196	0.46031	-0.81995
34.10744	-0.46168	1.53718	-1.78152	0.46331	-0.81759
34.1406	-0.46004	1.53742	-1.77941	0.46958	-0.81504
34.17352	-0.45772	1.53823	-1.77571	0.47557	-0.81291
34.20687	-0.4553	1.54018	-1.77301	0.48028	-0.81048
34.2394	-0.45354	1.54164	-1.77197	0.48207	-0.80899
34.27131	-0.45278	1.54301	-1.77134	0.4836	-0.80693
34.30405	-0.45234	1.54565	-1.7707	0.48604	-0.80366
34.33692	-0.45148	1.54989	-1.77013	0.48993	-0.80054
34.36993	-0.45108	1.55271	-1.76929	0.49475	-0.79796
34.40324	-0.4507	1.55507	-1.76837	0.49796	-0.79683
34.43551	-0.45026	1.55692	-1.76775	0.50029	-0.79625
34.46862	-0.44976	1.55832	-1.76725	0.50356	-0.79443
34.50148	-0.44936	1.56021	-1.76654	0.50734	-0.79109
34.5355	-0.44846	1.56241	-1.76595	0.51203	-0.78693
34.5685	-0.44718	1.56567	-1.76463	0.51723	-0.78425
34.60022	-0.44542	1.5697	-1.76248	0.52001	-0.78275
34.63325	-0.44356	1.57271	-1.76142	0.52274	-0.78175
34.66533	-0.44254	1.57417	-1.76095	0.52592	-0.78089
34.69842	-0.44186	1.57487	-1.76062	0.52932	-0.77981

34.7314	-0.44092	1.57549	-1.76014	0.5322	-0.77828
34.76404	-0.43982	1.5772	-1.75934	0.53561	-0.77572
34.79756	-0.43862	1.57984	-1.75757	0.53866	-0.77255
34.82983	-0.4378	1.58407	-1.75622	0.54075	-0.77088
34.86208	-0.43758	1.58857	-1.75414	0.5425	-0.77021
34.89545	-0.43742	1.59274	-1.75126	0.54426	-0.76981
34.92848	-0.43696	1.59502	-1.74955	0.54651	-0.76905
34.96072	-0.43612	1.59673	-1.7482	0.55023	-0.76777
34.99385	-0.4351	1.59933	-1.74715	0.55433	-0.76581
35.02644	-0.43374	1.60267	-1.74624	0.55754	-0.76326
35.05936	-0.43246	1.60598	-1.74526	0.55985	-0.76024
35.09246	-0.43098	1.60903	-1.74393	0.5621	-0.75777
35.12565	-0.42934	1.61019	-1.74231	0.56457	-0.75601
35.15792	-0.4278	1.61052	-1.74041	0.56621	-0.75499
35.19084	-0.42708	1.61083	-1.73784	0.56982	-0.75425
35.22292	-0.42642	1.61192	-1.73587	0.57437	-0.75339
35.25613	-0.42526	1.61468	-1.73461	0.57879	-0.75163
35.2888	-0.42322	1.61948	-1.73334	0.58319	-0.74863
35.32175	-0.42068	1.62754	-1.73273	0.58716	-0.74565
35.35547	-0.41886	1.63562	-1.73215	0.59008	-0.74405
35.38791	-0.418	1.64166	-1.73153	0.59257	-0.7435
35.42054	-0.4177	1.64577	-1.73078	0.59523	-0.74297
35.45333	-0.41758	1.64752	-1.72975	0.59797	-0.7418
35.48556	-0.41734	1.64781	-1.72831	0.60049	-0.73958
35.51904	-0.4167	1.64823	-1.7275	0.60322	-0.73667
35.55139	-0.41558	1.64842	-1.72702	0.60781	-0.73452
35.58409	-0.41374	1.64901	-1.72638	0.61318	-0.73325
35.61822	-0.41218	1.65042	-1.72572	0.61979	-0.73131
35.65032	-0.41138	1.65302	-1.7252	0.62301	-0.72936
35.68272	-0.41078	1.6565	-1.7245	0.62383	-0.72784

35.71593	-0.4103	1.65966	-1.72233	0.62419	-0.72627
35.749	-0.40982	1.66217	-1.71915	0.62528	-0.72541
35.7818	-0.40902	1.66451	-1.71701	0.62874	-0.72444
35.81442	-0.40866	1.66649	-1.71621	0.63463	-0.72246
35.84739	-0.40834	1.66842	-1.71527	0.64064	-0.72012
35.88062	-0.40792	1.6704	-1.71463	0.64432	-0.71772
35.91332	-0.4074	1.67273	-1.71405	0.64778	-0.71647
35.94595	-0.40596	1.67517	-1.71268	0.65051	-0.71586
35.97918	-0.40372	1.67713	-1.71131	0.65284	-0.71543
36.01125	-0.40234	1.67863	-1.71021	0.6552	-0.7149
36.04522	-0.40112	1.67978	-1.70895	0.65733	-0.71362
36.07732	-0.40034	1.68117	-1.70809	0.65894	-0.71154
36.11002	-0.39966	1.68209	-1.70713	0.6605	-0.7097
36.1427	-0.39866	1.68347	-1.70593	0.66162	-0.70788
36.1759	-0.39788	1.68506	-1.70399	0.66262	-0.70523
36.20852	-0.3971	1.68628	-1.7027	0.66493	-0.7026
36.24214	-0.39612	1.6883	-1.70218	0.66894	-0.69992
36.27456	-0.39488	1.69076	-1.70157	0.6736	-0.6982
36.30726	-0.3938	1.69405	-1.70091	0.67851	-0.6971
36.34101	-0.39296	1.69762	-1.69954	0.68147	-0.69645
36.37368	-0.3924	1.70029	-1.69755	0.68339	-0.69558
36.40682	-0.39182	1.70318	-1.69553	0.68691	-0.69352
36.4406	-0.39116	1.70564	-1.6928	0.69148	-0.6914
36.47138	-0.3901	1.70713	-1.69023	0.69469	-0.68904
36.50472	-0.3887	1.7077	-1.68923	0.70071	-0.68768
36.53762	-0.3877	1.70825	-1.68871	0.70482	-0.68665
36.57038	-0.3869	1.70956	-1.68827	0.70805	-0.68603
36.60319	-0.38656	1.71313	-1.68802	0.70973	-0.68518
36.63712	-0.3862	1.71969	-1.68762	0.71026	-0.68196
36.66976	-0.38574	1.72447	-1.68701	0.71036	-0.67604

36.70337	-0.38518	1.72639	-1.68609	0.71128	-0.67289
36.73589	-0.3843	1.72756	-1.68504	0.71339	-0.67217
36.76719	-0.38338	1.729	-1.68414	0.71627	-0.67176
36.80108	-0.38208	1.73122	-1.68277	0.71893	-0.67154
36.83418	-0.38068	1.73448	-1.68083	0.72079	-0.67114
36.8674	-0.37954	1.73798	-1.67995	0.72175	-0.66969
36.89934	-0.37888	1.74131	-1.67949	0.72443	-0.66728
36.93191	-0.3784	1.74344	-1.67899	0.72717	-0.66428
36.9654	-0.37796	1.74538	-1.67848	0.73055	-0.66254
36.99895	-0.37766	1.74799	-1.67768	0.73322	-0.66191
37.03112	-0.3772	1.75086	-1.67634	0.73554	-0.66129
37.06396	-0.37592	1.75386	-1.67514	0.7386	-0.66087
37.09696	-0.37414	1.75615	-1.67349	0.74173	-0.66047
37.13014	-0.37252	1.75733	-1.67208	0.74537	-0.65933
37.16318	-0.3717	1.75919	-1.67142	0.748	-0.65818
37.1952	-0.37146	1.76217	-1.67073	0.74966	-0.65719
37.22787	-0.37134	1.76615	-1.67011	0.75184	-0.65643
37.26091	-0.37082	1.77114	-1.66955	0.75559	-0.65467
37.29321	-0.3698	1.77303	-1.66875	0.75918	-0.6517
37.3267	-0.36838	1.77357	-1.66776	0.7629	-0.64713
37.35919	-0.36694	1.77406	-1.66734	0.76509	-0.64297
37.3928	-0.366	1.77531	-1.66694	0.76579	-0.64081
37.426	-0.36516	1.77984	-1.66656	0.76616	-0.63926
37.45886	-0.364	1.78548	-1.66607	0.76726	-0.63865
37.49266	-0.36322	1.78866	-1.66469	0.76901	-0.63822
37.52495	-0.36272	1.78964	-1.66314	0.77387	-0.63745
37.5579	-0.36252	1.7901	-1.66212	0.78176	-0.63693
37.5901	-0.36234	1.79153	-1.66142	0.78767	-0.63647
37.62276	-0.36174	1.7939	-1.66073	0.79067	-0.63623
37.65721	-0.3602	1.79669	-1.66031	0.79208	-0.63538

37.68976	-0.35878	1.79881	-1.65986	0.79314	-0.63398
37.72307	-0.35786	1.7999	-1.65924	0.79483	-0.63139
37.75531	-0.35732	1.80109	-1.65826	0.79727	-0.62748
37.78794	-0.3571	1.80393	-1.65679	0.80026	-0.6243
37.8214	-0.35686	1.80843	-1.65461	0.80254	-0.62193
37.85394	-0.35678	1.81359	-1.65259	0.8069	-0.62059
37.88675	-0.35668	1.81638	-1.65082	0.80989	-0.6199
37.91938	-0.35624	1.81788	-1.64964	0.8108	-0.61899
37.95166	-0.35468	1.81804	-1.64913	0.81117	-0.61826
37.98586	-0.3527	1.81794	-1.64864	0.81144	-0.61777
38.01776	-0.35096	1.81812	-1.64803	0.81249	-0.61723
38.05136	-0.35026	1.81888	-1.64756	0.81558	-0.61681
38.08418	-0.34996	1.82052	-1.64714	0.81739	-0.61655
38.11746	-0.34956	1.82287	-1.64656	0.81866	-0.61626
38.14955	-0.34898	1.8247	-1.6458	0.82012	-0.61575
38.18365	-0.34828	1.82589	-1.64477	0.82155	-0.61447
38.21622	-0.34714	1.82734	-1.64352	0.82557	-0.61308
38.2481	-0.34606	1.82871	-1.64234	0.83112	-0.61142
38.28178	-0.3446	1.82954	-1.64155	0.83404	-0.60961
38.31422	-0.34396	1.83018	-1.64095	0.83664	-0.60887
38.34652	-0.34366	1.831-1.64037	0.83872	-0.60821	
38.38068	-0.3434	1.83282	-1.63932	0.84007	-0.60794
38.41358	-0.34294	1.83616	-1.63824	0.84196	-0.6072
38.44622	-0.3423	1.83984	-1.63755	0.84536	-0.60534
38.47836	-0.34154	1.84264	-1.63711	0.84778	-0.60278
38.51153	-0.34074	1.84428	-1.63647	0.85065	-0.60102
38.54434	-0.3398	1.84571	-1.63604	0.85408	-0.60006
38.57837	-0.33914	1.84834	-1.63536	0.85581	-0.59915
38.61179	-0.33836	1.85193	-1.63377	0.85711	-0.59744
38.64488	-0.33752	1.85504	-1.63213	0.85792	-0.59618

38.6782	-0.33708	1.85644	-1.63118	0.85865	-0.59539
38.7095	-0.33658	1.85747	-1.6307	0.85945	-0.59506
38.74175	-0.33612	1.8587	-1.63018	0.8619	-0.5948
38.77514	-0.33578	1.8608	-1.62899	0.86523	-0.59394
38.80828	-0.33504	1.86458	-1.6276	0.86876	-0.59103
38.84155	-0.3342	1.86875	-1.62635	0.8713	-0.58845
38.874	-0.33294	1.87168	-1.62563	0.87254	-0.58735
38.90708	-0.33118	1.87329	-1.62518	0.87315	-0.58674
38.94009	-0.32984	1.87431	-1.62473	0.87365	-0.58584
38.9731	-0.32918	1.87467	-1.62425	0.87432	-0.58415
39.00649	-0.3289	1.87499	-1.62366	0.87557	-0.58242
39.03905	-0.32864	1.87572	-1.62282	0.87706	-0.58169
39.07172	-0.32824	1.87727	-1.62192	0.87984	-0.58118
39.10467	-0.3278	1.88031	-1.62128	0.88234	-0.5808
39.13753	-0.32748	1.88473	-1.62084	0.88394	-0.58037
39.1709	-0.32712	1.88821	-1.62041	0.88503	-0.5794
39.20394	-0.32686	1.88953	-1.61995	0.88597	-0.57796
39.2362	-0.32668	1.89069	-1.61938	0.88847	-0.5763
39.27039	-0.32638	1.89205	-1.61824	0.89255	-0.57461
39.30307	-0.32542	1.89452	-1.61701	0.89869	-0.57336
39.33582	-0.32408	1.8973	-1.61615	0.90418	-0.57254
39.36829	-0.3228	1.8992	-1.61547	0.90744	-0.57187
39.4021	-0.32162	1.90028	-1.61479	0.90924	-0.57077
39.4343	-0.32074	1.90241	-1.6138	0.91064	-0.56973
39.4675	-0.31994	1.90535	-1.61272	0.91108	-0.56798
39.49976	-0.31934	1.9091	-1.612	0.91146	-0.56648
39.53421	-0.31836	1.91179	-1.61156	0.91175	-0.56579
39.56594	-0.31754	1.91296	-1.61111	0.9121	-0.5651
39.59878	-0.3169	1.91352	-1.61057	0.91358	-0.56461
39.63204	-0.31648	1.91396	-1.60997	0.91551	-0.56419

39.66481	-0.31616	1.91447	-1.60944	0.91814	-0.5626
39.69838	-0.31578	1.91562	-1.60903	0.9205	-0.55942
39.7315	-0.31524	1.91742	-1.60867	0.9228	-0.5569
39.76392	-0.31476	1.91963	-1.60828	0.92629	-0.55619
39.7978	-0.31442	1.92101	-1.6079	0.9295	-0.55578
39.829	-0.31426	1.92196	-1.60746	0.931	-0.55512
39.86302	-0.31406	1.92314	-1.60658	0.93227	-0.55469
39.8949	-0.31372	1.92411	-1.60593	0.93264	-0.55315
39.92858	-0.31348	1.92546	-1.60533	0.93299	-0.55146
39.96106	-0.31326	1.92795	-1.60489	0.9334	-0.54993
39.99502	-0.31296	1.9307	-1.60449	0.93369	-0.54932
40.02767	-0.31256	1.93445	-1.60387	0.93495	-0.54886
40.06122	-0.31156	1.93728	-1.6026	0.93743	-0.54864
40.0933	-0.31048	1.93826	-1.60091	0.93973	-0.54837
40.12558	-0.31006	1.93883	-1.60023	0.94149	-0.54803
40.15868	-0.30978	1.93936	-1.5998	0.94266	-0.54687
40.19168	-0.3094	1.94017	-1.59938	0.94432	-0.54486
40.2258	-0.30926	1.94225	-1.59895	0.94702	-0.54321
40.25865	-0.30906	1.94619	-1.59845	0.94933	-0.54271
40.29106	-0.30872	1.94991	-1.59794	0.95008	-0.54227
40.32397	-0.30822	1.95348	-1.59751	0.95046	-0.54187
40.35758	-0.30748	1.95701	-1.59707	0.95125	-0.54154
40.39012	-0.30658	1.95904	-1.59662	0.95463	-0.54045
40.42243	-0.30554	1.9604	-1.59616	0.95926	-0.53856
40.45516	-0.30474	1.96128	-1.59551	0.96357	-0.53724
40.48956	-0.30426	1.96158	-1.59468	0.965	-0.53628
40.52141	-0.30384	1.96201	-1.59398	0.96579	-0.53548
40.55619	-0.30338	1.96234	-1.5935	0.96719	-0.53507
40.58762	-0.3027	1.96288	-1.59306	0.96796	-0.53468
40.62022	-0.302	1.96541	-1.59268	0.96841	-0.53412

40.65305	-0.30124	1.96753	-1.59226	0.96917	-0.53319
40.68639	-0.30052	1.96968	-1.5918	0.9704	-0.53267
40.72064	-0.29968	1.97219	-1.59117	0.97299	-0.53233
40.75284	-0.29912	1.97348	-1.59061	0.97504	-0.53195
40.78666	-0.2989	1.97417	-1.59017	0.97652	-0.53163
40.81906	-0.29876	1.97497	-1.58975	0.97805	-0.53121
40.8516	-0.29842	1.97577	-1.58919	0.97905	-0.53013
40.8842	-0.29816	1.97668	-1.58857	0.97998	-0.52859
40.91771	-0.29788	1.97824	-1.58764	0.98049	-0.52695
40.95074	-0.2976	1.98017	-1.58715	0.98091	-0.5259
40.9835	-0.29742	1.98147	-1.58677	0.98212	-0.52538
41.01652	-0.29698	1.98325	-1.58638	0.98469	-0.52466
41.04938	-0.296	1.98562	-1.58594	0.98668	-0.52366
41.08295	-0.29472	1.98754	-1.58554	0.98845	-0.523
41.11508	-0.2939	1.98867	-1.58517	0.99076	-0.52246
41.14883	-0.29366	1.98994	-1.5848	0.99352	-0.52196
41.18166	-0.29354	1.99169	-1.58438	0.99535	-0.52141
41.2143	-0.29334	1.9952	-1.58366	0.99614	-0.52087
41.24655	-0.2927	2.00129	-1.5831	0.99681	-0.51995
41.27988	-0.29206	2.00602	-1.58268	0.99748	-0.51933
41.31262	-0.2918	2.00748	-1.58226	0.99845	-0.51888
41.34589	-0.29158	2.00814	-1.58183	0.99959	-0.51851
41.37886	-0.29142	2.00891	-1.58132	1.00025	-0.51814
41.41236	-0.29112	2.0094	-1.58071	1.00062	-0.51783
41.44549	-0.29062	2.0103	-1.58027	1.00116	-0.51748
41.47924	-0.29012	2.01134	-1.57989	1.00258	-0.51706
41.51212	-0.28978	2.01211	-1.57953	1.0049	-0.51627
41.543	-0.28956	2.01286	-1.57917	1.00657	-0.51469
41.57743	-0.28944	2.01322	-1.5788	1.00854	-0.51361
41.61102	-0.2893	2.01369	-1.5784	1.01159	-0.51292

41.64352	-0.28906	2.01431	-1.57798	1.01488	-0.51195
41.67603	-0.2888	2.01516	-1.57753	1.019	-0.51152
41.7086	-0.2884	2.01772	-1.57701	1.02076	-0.51111
41.74229	-0.288	2.02125	-1.57652	1.02127	-0.51069
41.77654	-0.2876	2.02437	-1.57609	1.02208	-0.51034
41.80948	-0.28708	2.02669	-1.57563	1.0224	-0.50992
41.84178	-0.28634	2.02792	-1.57518	1.02328	-0.50914
41.8741	-0.28584	2.02873	-1.57464	1.02494	-0.50857
41.9071	-0.2855	2.02924	-1.57421	1.02714	-0.5081
41.94011	-0.28526	2.02958	-1.5739	1.02956	-0.50764
41.97332	-0.28504	2.0307	-1.57362	1.03115	-0.50724
42.00672	-0.28464	2.03311	-1.57327	1.03244	-0.50661
42.04016	-0.28428	2.03586	-1.5727	1.0335	-0.50589
42.07203	-0.2838	2.03778	-1.57146	1.03421	-0.50544
42.10488	-0.28274	2.03923	-1.57052	1.03507	-0.50483
42.13796	-0.28156	2.04013	-1.5699	1.03585	-0.50388
42.17136	-0.2806	2.04148	-1.56944	1.03662	-0.5032
42.20282	-0.28006	2.04411	-1.56898	1.03759	-0.50214
42.23711	-0.27986	2.04812	-1.56853	1.03848	-0.50103
42.26947	-0.27966	2.05305	-1.56814	1.03964	-0.5003
42.30265	-0.27946	2.05608	-1.56779	1.04159	-0.49965
42.33692	-0.2793	2.05821	-1.56745	1.04396	-0.49915
42.36926	-0.2792	2.0596	-1.56705	1.04601	-0.49873
42.40175	-0.27908	2.0603	-1.56665	1.04702	-0.49836
42.43528	-0.2789	2.06093	-1.56626	1.04776	-0.498
42.46798	-0.2786	2.06154	-1.56589	1.04851	-0.49765
42.50152	-0.27804	2.06219	-1.56556	1.04976	-0.49729
42.53421	-0.2771	2.06306	-1.56521	1.05098	-0.49685
42.56661	-0.27622	2.06372	-1.56482	1.05242	-0.49574
42.60097	-0.27572	2.06412	-1.56439	1.0553	-0.49406

42.63355	-0.27536	2.06465	-1.56396	1.05851	-0.49229
42.66603	-0.27518	2.06515	-1.5636	1.0616	-0.49102
42.69954	-0.2751	2.06566	-1.56328	1.06328	-0.4899
42.73248	-0.27496	2.06704	-1.56295	1.06407	-0.48949
42.76544	-0.27482	2.0684	-1.5626	1.06481	-0.48916
42.79912	-0.27452	2.06999	-1.56215	1.06639	-0.48887
42.83164	-0.27418	2.07063	-1.56131	1.06878	-0.48851
42.86494	-0.27384	2.07117	-1.5608	1.07151	-0.48766
42.89796	-0.2731	2.07184	-1.56038	1.07458	-0.48638
42.93092	-0.272	2.07234	-1.55999	1.07748	-0.48558
42.96379	-0.27096	2.07379	-1.55974	1.08107	-0.48517
42.99631	-0.27028	2.07682	-1.55953	1.08536	-0.48477
43.03011	-0.26994	2.07837	-1.55931	1.08674	-0.48443
43.06378	-0.26966	2.07905	-1.559	1.08723	-0.48402
43.09617	-0.26954	2.07936	-1.55841	1.08807	-0.4835
43.12929	-0.26928	2.08015	-1.55712	1.08946	-0.4831
43.16312	-0.2682	2.08279	-1.55648	1.09343	-0.48268
43.19574	-0.26648	2.08729	-1.55608	1.09797	-0.48227
43.22793	-0.26556	2.08992	-1.55572	1.10037	-0.48191
43.26191	-0.2651	2.09088	-1.55533	1.10162	-0.48152
43.29472	-0.2646	2.09184	-1.55496	1.1028	-0.48094
43.32784	-0.2643	2.09266	-1.55458	1.10357	-0.47998
43.3595	-0.26396	2.09523	-1.55421	1.1041	-0.47865
43.39295	-0.26366	2.09932	-1.55384	1.10467	-0.47697
43.42626	-0.2636	2.10247	-1.55353	1.1058	-0.47628
43.46022	-0.26344	2.10407	-1.55319	1.10748	-0.47591
43.4937	-0.26314	2.10492	-1.55279	1.1097	-0.47562
43.52675	-0.26264	2.10576	-1.55219	1.11071	-0.4753
43.55854	-0.262	2.10634	-1.55158	1.11117	-0.47495
43.59216	-0.26176	2.10663	-1.55117	1.11216	-0.47455

43.62562	-0.26164	2.10709	-1.55083	1.11502	-0.47412
43.6583	-0.2615	2.10755	-1.55051	1.11831	-0.47372
43.69204	-0.2613	2.10909	-1.55007	1.12301	-0.47333
43.7252	-0.26112	2.11091	-1.5493	1.1266	-0.47287
43.75859	-0.26076	2.11184	-1.5484	1.12894	-0.47179
43.79052	-0.2606	2.11253	-1.54785	1.13071	-0.47076
43.82422	-0.26038	2.11323	-1.54756	1.13234	-0.47032
43.85644	-0.25992	2.11408	-1.54733	1.13347	-0.46996
43.89019	-0.2596	2.11551	-1.54708	1.13484	-0.46959
43.92208	-0.25936	2.11629	-1.54679	1.1375	-0.46924
43.95649	-0.25904	2.11709	-1.54643	1.13924	-0.4688
43.98884	-0.25876	2.11766	-1.54605	1.14068	-0.46836
44.0218	-0.25818	2.11802	-1.54566	1.14169	-0.46796
44.05498	-0.2575	2.11861	-1.54525	1.1424	-0.46757
44.08836	-0.25724	2.12039	-1.54484	1.14401	-0.46719
44.12176	-0.2571	2.12254	-1.54446	1.14695	-0.46685
44.15519	-0.25696	2.1249	-1.54414	1.14905	-0.4665
44.1883	-0.25654	2.12776	-1.54383	1.15009	-0.46615
44.22138	-0.25552	2.13023	-1.54351	1.15054	-0.46562
44.2533	-0.2545	2.13186	-1.54322	1.15159	-0.46486
44.28686	-0.25412	2.13291	-1.54298	1.1555	-0.46354
44.32027	-0.25382	2.13434	-1.5427	1.16175	-0.46283
44.35292	-0.25366	2.13557	-1.54242	1.1651	-0.46242
44.38602	-0.2534	2.13671	-1.5421	1.16668	-0.46204
44.41985	-0.25324	2.13796	-1.54177	1.16748	-0.46167
44.45246	-0.25304	2.13868	-1.54136	1.16779	-0.46137
44.48523	-0.25292	2.13932	-1.54095	1.16887	-0.46105
44.5189	-0.25284	2.14063	-1.54059	1.17136	-0.46069
44.55166	-0.25272	2.14251	-1.54024	1.17402	-0.46031
44.58466	-0.25258	2.14474	-1.53994	1.17655	-0.45993

44.61806	-0.25242	2.14671	-1.53962	1.17823	-0.45955
44.65102	-0.25224	2.14841	-1.53929	1.17996	-0.45916
44.68402	-0.25204	2.14952	-1.53896	1.18237	-0.45874
44.71735	-0.25182	2.15036	-1.53865	1.18339	-0.45816
44.75063	-0.25164	2.15101	-1.53838	1.18377	-0.45776
44.78391	-0.25144	2.15198	-1.53808	1.18429	-0.45748
44.81685	-0.2513	2.15259	-1.53777	1.18528	-0.45726
44.84984	-0.25118	2.15305	-1.53744	1.18906	-0.45698
44.88353	-0.25104	2.15355	-1.53716	1.19247	-0.45663
44.9161	-0.2509	2.15398	-1.53686	1.19461	-0.45626
44.94922	-0.25076	2.15537	-1.53657	1.19544	-0.45586
44.98212	-0.2506	2.15787	-1.53627	1.1963	-0.45547
45.01512	-0.25048	2.15993	-1.53592	1.19662	-0.45503
45.04929	-0.25034	2.16124	-1.53554	1.19696	-0.45423
45.08238	-0.2502	2.16243	-1.53519	1.1982	-0.4537
45.11528	-0.25004	2.16359	-1.53488	1.20007	-0.45336
45.14814	-0.24988	2.16413	-1.53468	1.20192	-0.45306
45.18146	-0.24974	2.16452	-1.53452	1.20322	-0.45274
45.21416	-0.24962	2.16503	-1.5343	1.20433	-0.45234
45.24766	-0.24952	2.16529	-1.53402	1.20559	-0.45175
45.28019	-0.24938	2.16571	-1.53372	1.20735	-0.45109
45.31284	-0.24922	2.16699	-1.53342	1.20882	-0.45053
45.34682	-0.24892	2.16868	-1.53314	1.20964	-0.45016
45.37877	-0.2476	2.16942	-1.5329	1.21051	-0.44991
45.41328	-0.24626	2.17012	-1.53265	1.21114	-0.44969
45.44626	-0.24586	2.17103	-1.53236	1.21143	-0.44942
45.47913	-0.24568	2.17333	-1.53204	1.21214	-0.44896
45.51195	-0.24558	2.17484	-1.53173	1.21364	-0.44808
45.54567	-0.24548	2.17553	-1.53154	1.21519	-0.44722
45.57817	-0.2453	2.17588	-1.53137	1.21731	-0.44687

45.61183	-0.24496	2.17626	-1.53119	1.21901	-0.44663
45.64416	-0.2447	2.17717	-1.53102	1.21992	-0.44636
45.67848	-0.24438	2.1802	-1.53081	1.22146	-0.44605
45.71081	-0.24406	2.18413	-1.53054	1.22452	-0.44571
45.74409	-0.2438	2.18746	-1.53026	1.22637	-0.44533
45.77628	-0.24364	2.18961	-1.52995	1.22831	-0.44498
45.80994	-0.24348	2.19058	-1.52965	1.23082	-0.44465
45.84316	-0.24334	2.19092	-1.52941	1.23261	-0.4443
45.87596	-0.24316	2.19138	-1.52914	1.23349	-0.44396
45.90976	-0.24302	2.19339	-1.5287	1.23393	-0.44373
45.94296	-0.24288	2.19739	-1.52835	1.2342	-0.44354
45.97568	-0.24268	2.20095	-1.52807	1.23474	-0.44334
46.00922	-0.24238	2.20301	-1.52784	1.2367	-0.44288
46.04206	-0.24208	2.20399	-1.52764	1.23995	-0.44163
46.0759	-0.24186	2.20459	-1.52738	1.24205	-0.44009
46.10764	-0.2417	2.20503	-1.52705	1.24345	-0.43962
46.14084	-0.24156	2.20552	-1.52676	1.24446	-0.43933
46.17456	-0.24138	2.20644	-1.52646	1.24605	-0.43911
46.20862	-0.24116	2.20823	-1.52614	1.24839	-0.4389
46.24111	-0.241	2.21031	-1.52568	1.24934	-0.43861
46.27392	-0.24082	2.21106	-1.52493	1.24976	-0.43826
46.3075	-0.2406	2.21156	-1.52463	1.25024	-0.43788
46.34112	-0.24046	2.21253	-1.52434	1.25088	-0.43761
46.37264	-0.24034	2.21343	-1.524	1.25258	-0.43738
46.4062	-0.24024	2.21458	-1.52371	1.25518	-0.43715
46.4393	-0.24014	2.21592	-1.5234	1.2563	-0.43687
46.47293	-0.24004	2.21677	-1.52315	1.25683	-0.43657
46.50634	-0.23944	2.21737	-1.52302	1.25772	-0.43627
46.53859	-0.23836	2.21767	-1.52288	1.25917	-0.436
46.57205	-0.23738	2.21801	-1.52264	1.26051	-0.43567

46.60531	-0.23708	2.21859	-1.52238	1.26169	-0.4353
46.63904	-0.23698	2.2195	-1.52206	1.26222	-0.43502
46.67164	-0.2369	2.22097	-1.52178	1.26291	-0.43471
46.7051	-0.2368	2.22253	-1.52159	1.26388	-0.43429
46.73814	-0.23668	2.22388	-1.5214	1.26513	-0.43394
46.77114	-0.23652	2.22473	-1.52115	1.26688	-0.43359
46.80404	-0.23638	2.22603	-1.52084	1.26848	-0.43322
46.83686	-0.2362	2.22777	-1.52052	1.27009	-0.43292
46.86935	-0.236	2.22961	-1.52024	1.27085	-0.43258
46.90194	-0.23574	2.23088	-1.52007	1.2714	-0.43225
46.93606	-0.2356	2.23133	-1.51991	1.27206	-0.43197
46.96992	-0.2355	2.23188	-1.51972	1.27247	-0.43164
47.00258	-0.2354	2.23234	-1.51947	1.2735	-0.43129
47.03612	-0.23526	2.23305	-1.51918	1.27591	-0.43097
47.06948	-0.23502	2.23438	-1.51896	1.2777	-0.43063
47.10278	-0.23458	2.23511	-1.51879	1.27937	-0.43033
47.13604	-0.23442	2.23576	-1.51864	1.28056	-0.43004
47.16809	-0.2343	2.23639	-1.51844	1.28167	-0.42962
47.20176	-0.2342	2.23748	-1.51803	1.28338	-0.42919
47.23556	-0.2341	2.24018	-1.51757	1.28585	-0.42881
47.26817	-0.23394	2.24187	-1.5172	1.28793	-0.42847
47.30123	-0.23374	2.24235	-1.5169	1.28973	-0.42815
47.3341	-0.23352	2.24274	-1.51667	1.29093	-0.42785
47.36757	-0.23332	2.24311	-1.51645	1.29215	-0.42752
47.40053	-0.23318	2.24374	-1.51622	1.29296	-0.42714
47.43378	-0.23306	2.24495	-1.51596	1.29358	-0.42682
47.46723	-0.23292	2.24648	-1.51572	1.29416	-0.42651
47.50019	-0.23282	2.24739	-1.51542	1.29466	-0.42618
47.53365	-0.23272	2.24792	-1.51518	1.29571	-0.42583
47.56799	-0.2326	2.24833	-1.51495	1.29723	-0.42552

47.60041	-0.2325	2.24872	-1.51471	1.29915	-0.42526
47.63264	-0.2324	2.25018	-1.51446	1.29994	-0.4251
47.66496	-0.2323	2.25348	-1.51424	1.30027	-0.42495
47.69874	-0.23222	2.2564	-1.51405	1.30066	-0.42471
47.732	-0.23212	2.25851	-1.51388	1.30111	-0.42428
47.76538	-0.23198	2.25925	-1.51366	1.30217	-0.42317
47.7988	-0.23182	2.25978	-1.51348	1.30374	-0.42215
47.83131	-0.23158	2.26044	-1.51324	1.30591	-0.42183
47.86457	-0.23126	2.26086	-1.51294	1.30851	-0.42154
47.89809	-0.23108	2.26143	-1.51265	1.30946	-0.42128
47.93046	-0.23094	2.26285	-1.51242	1.3101	-0.42105
47.96394	-0.23082	2.26557	-1.51223	1.31049	-0.42086
47.9968	-0.23072	2.26785	-1.51204	1.31106	-0.42068
48.02954	-0.23062	2.2692	-1.5118	1.31259	-0.42043
48.06314	-0.2305	2.26979	-1.51154	1.31367	-0.4202
48.0971	-0.23036	2.27056	-1.51129	1.3145	-0.41998
48.1301	-0.23022	2.27134	-1.51107	1.31523	-0.41974
48.16272	-0.23006	2.27203	-1.5108	1.31621	-0.41946
48.19639	-0.2299	2.27285	-1.5105	1.31755	-0.41846
48.22996	-0.2298	2.27331	-1.51026	1.31922	-0.4174
48.26276	-0.2297	2.27397	-1.51014	1.3207	-0.41707
48.29609	-0.22962	2.27441	-1.51007	1.32253	-0.41688
48.32794	-0.22954	2.27502	-1.50999	1.32453	-0.41677
48.36171	-0.22942	2.27623	-1.50982	1.32738	-0.41668
48.39453	-0.22912	2.27887	-1.50957	1.32863	-0.4165
48.42828	-0.22884	2.28075	-1.50937	1.3293	-0.41626
48.46172	-0.2287	2.28179	-1.50918	1.33014	-0.41601
48.49504	-0.22858	2.28237	-1.50906	1.33095	-0.41574
48.52696	-0.22846	2.28296	-1.50897	1.33155	-0.41547
48.56124	-0.22836	2.28336	-1.5088	1.33211	-0.41517

48.59351	-0.22822	2.28372	-1.50851	1.33267	-0.41459
48.62688	-0.22808	2.28435	-1.50761	1.3338	-0.41414
48.66054	-0.22778	2.28587	-1.50691	1.33612	-0.41386
48.6934	-0.22752	2.28706	-1.50666	1.3377	-0.41362
48.72597	-0.22742	2.28882	-1.50651	1.33836	-0.41343
48.75986	-0.22732	2.28957	-1.50642	1.33926	-0.41326
48.7918	-0.22724	2.29008	-1.50628	1.34028	-0.41305
48.82528	-0.22712	2.29072	-1.50612	1.3426	-0.41285
48.85926	-0.227	2.29154	-1.50594	1.34476	-0.4126
48.89131	-0.22688	2.29318	-1.50578	1.34556	-0.41231
48.92539	-0.22678	2.29497	-1.50569	1.34636	-0.41185
48.9588	-0.22672	2.29593	-1.50561	1.34688	-0.41119
48.99288	-0.22666	2.29651	-1.50548	1.3481	-0.41059
49.02489	-0.2266	2.2969	-1.50535	1.34969	-0.41034
49.05769	-0.22652	2.29758	-1.50514	1.35014	-0.41023
49.09146	-0.2264	2.29853	-1.50492	1.3506	-0.41024
49.12467	-0.22626	2.29929	-1.50475	1.35125	-0.41022
49.1577	-0.22612	2.3003	-1.50451	1.3519	-0.41
49.19033	-0.22602	2.3012	-1.50427	1.35427	-0.40891
49.22346	-0.22592	2.30216	-1.50406	1.35657	-0.40671
49.25696	-0.22584	2.30413	-1.50393	1.35885	-0.40541
49.28932	-0.22578	2.30603	-1.50391	1.36077	-0.40502
49.32328	-0.22572	2.30759	-1.50393	1.36164	-0.40487
49.35619	-0.2256	2.30895	-1.50386	1.36216	-0.40474
49.3898	-0.22536	2.30956	-1.50366	1.36271	-0.40455
49.42242	-0.22482	2.31027	-1.50346	1.36307	-0.40431
49.45696	-0.22448	2.31073	-1.50335	1.36344	-0.4041
49.48878	-0.22438	2.31124	-1.50331	1.36379	-0.40387
49.52228	-0.22428	2.31203	-1.50322	1.36424	-0.40362
49.55517	-0.22422	2.31321	-1.50301	1.36511	-0.40343

49.58878	-0.22416	2.3143	-1.5025	1.36677	-0.4033
49.62187	-0.22404	2.31497	-1.50176	1.36776	-0.40318
49.65533	-0.22374	2.31545	-1.50163	1.36845	-0.40306
49.68894	-0.22356	2.31582	-1.50156	1.36919	-0.40282
49.72214	-0.22346	2.31617	-1.5015	1.36961	-0.4025
49.75559	-0.2234	2.31668	-1.50147	1.36996	-0.40227
49.78852	-0.22336	2.31775	-1.50142	1.37059	-0.4021
49.82082	-0.22332	2.31886	-1.50127	1.3715	-0.402
49.85376	-0.22326	2.31986	-1.5011	1.37272	-0.40196
49.88687	-0.2232	2.32039	-1.50068	1.37345	-0.40187
49.92061	-0.22314	2.32118	-1.50034	1.37477	-0.4017
49.954	-0.22306	2.32304	-1.5002	1.37602	-0.40146
49.98678	-0.22298	2.32605	-1.50007	1.37717	-0.40079
50.01992	-0.22286	2.33079	-1.49998	1.37778	-0.40043
50.05328	-0.22276	2.33315	-1.49988	1.37812	-0.40026
50.08668	-0.22268	2.33371	-1.49973	1.37861	-0.40011
50.11972	-0.22262	2.33411	-1.49956	1.37899	-0.4
50.1526	-0.22256	2.33458	-1.49932	1.37937	-0.39991
50.1863	-0.22252	2.33494	-1.49898	1.37992	-0.39982
50.21907	-0.22246	2.33536	-1.4988	1.38053	-0.39972
50.25336	-0.22242	2.3357	-1.49871	1.3814	-0.39959
50.2866	-0.22238	2.33619	-1.49862	1.38291	-0.39942
50.31928	-0.22234	2.33718	-1.49852	1.38423	-0.39921
50.3519	-0.22232	2.33843	-1.49836	1.38512	-0.399
50.38589	-0.22226	2.33998	-1.49813	1.38598	-0.3988
50.41754	-0.22222	2.34107	-1.49801	1.3868	-0.39866
50.45152	-0.22218	2.34156	-1.498	1.38732	-0.39858
50.4851	-0.22212	2.34202	-1.49802	1.38773	-0.39854
50.51772	-0.22204	2.34239	-1.49804	1.38827	-0.39848
50.55116	-0.222	2.3428	-1.49804	1.3891	-0.39836

50.58367	-0.22196	2.34341	-1.49804	1.3904	-0.39818
50.61764	-0.22192	2.34519	-1.49812	1.39211	-0.39796
50.65098	-0.22188	2.34813	-1.49812	1.39305	-0.39775
50.6839	-0.22184	2.35058	-1.49802	1.39381	-0.39759
50.71713	-0.22182	2.35214	-1.49781	1.39466	-0.39753
50.75024	-0.2218	2.35285	-1.49724	1.39525	-0.39752
50.78244	-0.2218	2.35347	-1.497	1.39581	-0.39752
50.81562	-0.22178	2.35428	-1.49686	1.39628	-0.39744
50.84896	-0.22174	2.35526	-1.49669	1.39672	-0.39734
50.88239	-0.22172	2.35574	-1.49658	1.39716	-0.39723
50.91493	-0.2217	2.35621	-1.49651	1.39762	-0.39714
50.94968	-0.22168	2.35664	-1.49645	1.39811	-0.39708
50.98242	-0.22168	2.35705	-1.49654	1.39893	-0.39702
51.01478	-0.22166	2.3575	-1.49667	1.4001	-0.39694
51.04825	-0.22164	2.35868	-1.49663	1.40131	-0.39687
51.08159	-0.22162	2.35984	-1.49625	1.4021	-0.39682
51.11512	-0.22158	2.36073	-1.49447	1.4035	-0.3968
51.14771	-0.22152	2.36123	-1.49297	1.40652	-0.39683
51.18096	-0.22148	2.36178	-1.49276	1.40903	-0.39682
51.21399	-0.22142	2.36266	-1.49276	1.4099	-0.3967
51.2481	-0.22138	2.36375	-1.49285	1.41037	-0.39625
51.2806	-0.22134	2.36418	-1.49286	1.41079	-0.3956
51.3136	-0.22132	2.3646	-1.49281	1.41161	-0.39538
51.34696	-0.22128	2.36569	-1.49278	1.41345	-0.39536
51.38063	-0.22126	2.36774	-1.49285	1.41598	-0.39543
51.41336	-0.22124	2.36843	-1.4929	1.41697	-0.39549
51.44668	-0.2212	2.36877	-1.49285	1.41733	-0.39559
51.47957	-0.22118	2.36907	-1.49276	1.41772	-0.39564
51.51209	-0.2212	2.3694	-1.49267	1.41812	-0.39566
51.54512	-0.22122	2.37021	-1.49258	1.41917	-0.39561

51.57919	-0.2212	2.37161	-1.49255	1.4214	-0.3955
51.61254	-0.22116	2.37201	-1.49249	1.42278	-0.39535
51.64626	-0.2211	2.37242	-1.4924	1.4233	-0.39524
51.67922	-0.22102	2.37275	-1.49229	1.4237	-0.39516
51.71179	-0.221	2.37315	-1.49229	1.42434	-0.39518
51.74512	-0.221	2.37398	-1.49247	1.42516	-0.39522
51.77759	-0.22102	2.37498	-1.49276	1.42616	-0.39529
51.81156	-0.22106	2.37626	-1.49325	1.42669	-0.39536
51.84431	-0.22108	2.37779	-1.49343	1.42719	-0.39538
51.87782	-0.2211	2.37947	-1.49344	1.42794	-0.3954
51.9109	-0.22112	2.38101	-1.49336	1.4284	-0.3954
51.944	-0.22112	2.382	-1.49292	1.42884	-0.39537
51.97688	-0.22114	2.3825	-1.4922	1.4293	-0.3954
52.01006	-0.22114	2.38293	-1.49204	1.42976	-0.39551
52.0435	-0.22118	2.38365	-1.49203	1.43059	-0.3956
52.07666	-0.22118	2.38405	-1.4921	1.43142	-0.39568
52.11006	-0.2212	2.38446	-1.49217	1.43223	-0.39569
52.14385	-0.22124	2.38514	-1.4922	1.43305	-0.39565
52.17672	-0.22132	2.38604	-1.49215	1.43401	-0.39566
52.20954	-0.2214	2.3872	-1.49204	1.435	-0.39568
52.2427	-0.22146	2.38894	-1.49195	1.43557	-0.3957
52.27582	-0.22148	2.39039	-1.49187	1.43604	-0.3957
52.30917	-0.22152	2.39137	-1.49184	1.43663	-0.39566
52.34326	-0.22158	2.39207	-1.4919	1.43817	-0.39563
52.37617	-0.22162	2.39256	-1.49202	1.44027	-0.39565
52.409	-0.22164	2.39316	-1.49209	1.44147	-0.39568
52.44226	-0.22162	2.39362	-1.49207	1.44218	-0.39579
52.47534	-0.22162	2.39406	-1.49198	1.44285	-0.39591
52.50851	-0.22164	2.39461	-1.49185	1.44339	-0.39599
52.54033	-0.2217	2.39493	-1.49177	1.44383	-0.39603

52.57436	-0.22178	2.39532	-1.4917	1.44428	-0.39601
52.6075	-0.22188	2.39577	-1.49164	1.44473	-0.39597
52.64129	-0.22198	2.39622	-1.4916	1.44522	-0.39599
52.6745	-0.22204	2.39705	-1.49158	1.44571	-0.39611
52.70786	-0.22208	2.39766	-1.49161	1.44616	-0.39626
52.74051	-0.2221	2.39811	-1.49168	1.44654	-0.3964
52.77403	-0.22212	2.3989	-1.49164	1.44687	-0.39653
52.8073	-0.22212	2.40102	-1.49158	1.44729	-0.39659
52.8403	-0.22216	2.40242	-1.49146	1.44863	-0.39667
52.8737	-0.22222	2.40387	-1.49139	1.44999	-0.39706
52.90637	-0.22228	2.40446	-1.4914	1.4519	-0.3977
52.93974	-0.22232	2.40488	-1.49139	1.45269	-0.39867
52.97329	-0.22232	2.40536	-1.4914	1.45321	-0.3993
53.00591	-0.22234	2.40681	-1.49138	1.45367	-0.39939
53.03895	-0.22244	2.40928	-1.49132	1.45428	-0.39944
53.07254	-0.22256	2.41112	-1.49132	1.455-0.39952	
53.10608	-0.2227	2.41199	-1.49128	1.45546	-0.39956
53.13899	-0.22278	2.41247	-1.49119	1.45603	-0.39963
53.1723	-0.22284	2.41288	-1.49117	1.4569	-0.39975
53.20532	-0.22292	2.41336	-1.4911	1.45763	-0.39986
53.23838	-0.22322	2.41386	-1.4911	1.4588	-0.39998
53.27266	-0.22342	2.41486	-1.4911	1.45951	-0.40014
53.3045	-0.22364	2.41535	-1.49105	1.45991	-0.40021
53.33774	-0.22376	2.4159	-1.49099	1.46068	-0.40033
53.37104	-0.22384	2.41658	-1.49099	1.46275	-0.40077
53.40482	-0.22394	2.41774	-1.49103	1.46493	-0.40172
53.43842	-0.22422	2.41871	-1.49111	1.46628	-0.40327
53.47118	-0.2244	2.41924	-1.49117	1.46688	-0.40468
53.50455	-0.22462	2.41966	-1.49118	1.46724	-0.40515
53.53732	-0.22478	2.42002	-1.49116	1.46766	-0.4054

53.5711	-0.2249	2.42036	-1.49113	1.46813	-0.40566
53.60372	-0.22498	2.42075	-1.49111	1.4686	-0.40589
53.63662	-0.22508	2.42121	-1.49104	1.4691	-0.40604
53.67054	-0.2252	2.42268	-1.49092	1.46953	-0.40614
53.7034	-0.22536	2.42464	-1.4909	1.46999	-0.40622
53.73696	-0.22574	2.42624	-1.49087	1.47044	-0.40639
53.77001	-0.2263	2.42722	-1.49082	1.4709	-0.4066
53.8046	-0.2265	2.42773	-1.49072	1.47151	-0.40683
53.83621	-0.22666	2.42822	-1.49056	1.47209	-0.40705
53.86894	-0.22678	2.42868	-1.49049	1.47279	-0.40722
53.90297	-0.22692	2.42904	-1.49056	1.47371	-0.40736
53.9354	-0.22708	2.42934	-1.49061	1.47443	-0.40754
53.9697	-0.2272	2.42965	-1.49059	1.47478	-0.40775
54.00274	-0.22734	2.43004	-1.49051	1.47512	-0.40793
54.03545	-0.22746	2.43107	-1.49037	1.47551	-0.4081
54.06981	-0.22762	2.43275	-1.49027	1.47594	-0.40828
54.102	-0.22806	2.43438	-1.49025	1.4774	-0.40847
54.13591	-0.22834	2.43535	-1.49021	1.47868	-0.40873
54.1684	-0.22848	2.4358	-1.49014	1.47917	-0.40922
54.2021	-0.22862	2.43621	-1.49004	1.4796	-0.41012
54.23586	-0.22874	2.43667	-1.48992	1.48002	-0.41049
54.26807	-0.22886	2.43704	-1.48987	1.48044	-0.41075
54.30094	-0.22904	2.43742	-1.48994	1.48093	-0.41104
54.3335	-0.22936	2.43782	-1.49004	1.48178	-0.41134
54.36811	-0.22986	2.4382	-1.49024	1.48334	-0.41165
54.40118	-0.2302	2.43872	-1.49026	1.4851	-0.41191
54.43554	-0.23058	2.43925	-1.49015	1.486-0.41213	
54.46739	-0.23106	2.44004	-1.49001	1.48645	-0.41236
54.50082	-0.23174	2.44076	-1.48993	1.48687	-0.41264
54.5337	-0.23248	2.44128	-1.48989	1.48726	-0.41291

54.56646	-0.23274	2.44174	-1.48992	1.48769	-0.4132
54.60058	-0.2329	2.44218	-1.48996	1.48815	-0.41345
54.63384	-0.2332	2.44291	-1.48991	1.48861	-0.41368
54.66687	-0.23346	2.44406	-1.48987	1.48915	-0.41395
54.70079	-0.23376	2.44472	-1.48981	1.49001	-0.41427
54.73277	-0.23392	2.44517	-1.48967	1.49084	-0.41457
54.76586	-0.23408	2.44574	-1.48955	1.49129	-0.41488
54.7993	-0.23422	2.4462	-1.48947	1.49172	-0.41509
54.83191	-0.23442	2.44664	-1.48937	1.4921	-0.41528
54.86542	-0.23458	2.44705	-1.48937	1.49251	-0.41553
54.8999	-0.23486	2.44745	-1.48948	1.49303	-0.41582
54.93272	-0.23556	2.448	-1.48953	1.49354	-0.41614
54.96575	-0.23632	2.44947	-1.4896	1.49401	-0.41644
54.99878	-0.23666	2.45076	-1.48968	1.49455	-0.41666
55.03243	-0.23688	2.45132	-1.48967	1.495	-0.41687
55.06443	-0.2373	2.45192	-1.48966	1.49585	-0.41702
55.09824	-0.23824	2.45272	-1.48964	1.49671	-0.41719
55.1312	-0.23926	2.45419	-1.48947	1.49757	-0.41741
55.16487	-0.24014	2.45629	-1.48921	1.49814	-0.41771
55.19868	-0.241	2.45728	-1.4891	1.49862	-0.41877
55.23196	-0.24188	2.45811	-1.48898	1.49906	-0.42129
55.26489	-0.2432	2.45872	-1.48902	1.49948	-0.42348
55.29831	-0.24404	2.45925	-1.48908	1.49991	-0.42537
55.33063	-0.24436	2.45965	-1.48915	1.50039	-0.42582
55.36526	-0.24478	2.46007	-1.48921	1.50085	-0.42617
55.39704	-0.24502	2.46065	-1.48927	1.50136	-0.42684
55.43116	-0.2453	2.4618	-1.4894	1.50218	-0.42748
55.46507	-0.24548	2.46283	-1.48952	1.5027	-0.42793
55.49696	-0.246	2.46352	-1.48955	1.50333	-0.42832
55.53	-0.2471	2.46399	-1.48948	1.50383	-0.42878

55.56442	-0.24854	2.46437	-1.48939	1.5043	-0.43017
55.59668	-0.25024	2.46479	-1.48934	1.50478	-0.43121
55.63064	-0.25134	2.46525	-1.4893	1.50528	-0.43168
55.66305	-0.25218	2.46607	-1.4893	1.50577	-0.43191
55.69638	-0.2528	2.46664	-1.48926	1.50644	-0.43212
55.72991	-0.25308	2.46702	-1.48927	1.50707	-0.43233
55.76262	-0.25362	2.46743	-1.48932	1.5076	-0.43255
55.79604	-0.25418	2.46801	-1.48936	1.50813	-0.43285
55.8288	-0.25498	2.46976	-1.48939	1.50864	-0.43321
55.86222	-0.2561	2.47294	-1.48946	1.50914	-0.43381
55.89586	-0.25676	2.47485	-1.48956	1.50955	-0.43439
55.9292	-0.25724	2.47656	-1.48967	1.50994	-0.43465
55.96196	-0.25782	2.4774	-1.48976	1.51035	-0.43483
55.99524	-0.25854	2.47786	-1.48977	1.5108	-0.43504
56.0287	-0.2596	2.47835	-1.48974	1.51151	-0.43529
56.0618	-0.26082	2.47888	-1.48975	1.51301	-0.43556
56.09472	-0.26194	2.47931	-1.48976	1.51534	-0.43577
56.12788	-0.26264	2.47977	-1.48982	1.51787	-0.43589
56.16137	-0.26296	2.48023	-1.48991	1.51927	-0.43596
56.19558	-0.26356	2.48065	-1.48994	1.51986	-0.43606
56.22921	-0.26412	2.48112	-1.48993	1.52024	-0.4362
56.26235	-0.26514	2.48181	-1.4899	1.52067	-0.43636
56.29528	-0.26648	2.48228	-1.48982	1.52143	-0.43656
56.32763	-0.2679	2.48272	-1.48982	1.52201	-0.43678
56.36134	-0.26906	2.48317	-1.48991	1.52247	-0.43698
56.39444	-0.27	2.48358	-1.49002	1.52303	-0.43711
56.42706	-0.27046	2.48395	-1.49015	1.52354	-0.43717
56.46106	-0.27098	2.48434	-1.49028	1.52413	-0.43716
56.49304	-0.27152	2.48477	-1.49032	1.52459	-0.43681
56.52688	-0.2722	2.48528	-1.49028	1.52496	-0.43617

56.55988	-0.2727	2.48581	-1.49024	1.52532	-0.43574
56.5934	-0.27314	2.4865	-1.49012	1.52567	-0.43567
56.62651	-0.27402	2.48709	-1.49007	1.52601	-0.43571
56.6594	-0.27492	2.48817	-1.49014	1.52635	-0.43577
56.69304	-0.27612	2.48938	-1.4902	1.52673	-0.43563
56.72625	-0.27694	2.48994	-1.4902	1.52714	-0.43475
56.75966	-0.27786	2.49033	-1.49022	1.52757	-0.43339
56.79187	-0.27908	2.49071	-1.49027	1.52805	-0.43272
56.82582	-0.28086	2.4911	-1.49048	1.52849	-0.43262
56.8588	-0.28196	2.49156	-1.49092	1.52911	-0.43268
56.89271	-0.28252	2.49202	-1.49114	1.53105	-0.4326
56.9256	-0.28286	2.49248	-1.49116	1.53355	-0.43215
56.95862	-0.28338	2.49298	-1.49119	1.53632	-0.43169
56.99172	-0.28498	2.49379	-1.49127	1.53716	-0.43112
57.02462	-0.2872	2.49582	-1.49138	1.53747	-0.43105
57.05863	-0.28904	2.49741	-1.49157	1.53777	-0.43105
57.09253	-0.29028	2.498-1.49168	1.53808	-0.43099	
57.12641	-0.29102	2.49844	-1.49169	1.53896	-0.43083
57.15868	-0.29154	2.49883	-1.49165	1.54115	-0.43054
57.19201	-0.2924	2.49929	-1.49166	1.54269	-0.43025
57.2253	-0.29402	2.49981	-1.49172	1.54429	-0.43017
57.25794	-0.29658	2.50031	-1.49186	1.54505	-0.43011
57.29229	-0.29878	2.501-1.492	1.54541	-0.42987	
57.32559	-0.2995	2.50169	-1.49206	1.54578	-0.42938
57.35858	-0.29988	2.50221	-1.49205	1.54615	-0.42889
57.39162	-0.30042	2.5027	-1.49202	1.54645	-0.42865
57.42548	-0.3014	2.50331	-1.492	1.54673	-0.42857
57.45812	-0.3032	2.50387	-1.49201	1.54699	-0.42852
57.49148	-0.30452	2.50435	-1.49205	1.54739	-0.42839
57.52388	-0.30508	2.50485	-1.49218	1.54823	-0.42824

57.55735	-0.30546	2.50528	-1.4923	1.54906	-0.4281
57.59115	-0.30598	2.50564	-1.49238	1.54971	-0.42791
57.62383	-0.30706	2.50604	-1.49249	1.55002	-0.42773
57.65744	-0.3091	2.50643	-1.4926	1.55024	-0.42763
57.68964	-0.3107	2.50706	-1.49273	1.55044	-0.42751
57.72364	-0.31154	2.50932	-1.49286	1.5507	-0.42742
57.75732	-0.312	2.51215	-1.49297	1.55103	-0.42736
57.78984	-0.3126	2.51416	-1.49305	1.5514	-0.42719
57.82343	-0.31374	2.51477	-1.49311	1.55176	-0.42703
57.85679	-0.3154	2.51503	-1.49314	1.55213	-0.42692
57.8898	-0.31744	2.51537	-1.49317	1.55252	-0.42676
57.9233	-0.32006	2.51583	-1.49325	1.55306	-0.42659
57.95658	-0.32278	2.51692	-1.49335	1.5538	-0.42644
57.98884	-0.32478	2.51907	-1.49347	1.5542	-0.42628
58.02241	-0.32606	2.51995	-1.49357	1.55455	-0.42616
58.05584	-0.32694	2.52038	-1.49367	1.55493	-0.42607
58.0891	-0.32812	2.52081	-1.49392	1.5553	-0.42596
58.12241	-0.32932	2.52123	-1.49438	1.55563	-0.42582
58.1551	-0.32994	2.52156	-1.495	1.55598	-0.42565
58.18903	-0.33022	2.52191	-1.49524	1.5565	-0.42553
58.2221	-0.33092	2.52226	-1.49538	1.55774	-0.42542
58.25504	-0.33172	2.52259	-1.49537	1.55919	-0.4253
58.28874	-0.3332	2.52292	-1.49531	1.55988	-0.42517
58.32227	-0.33492	2.52334	-1.4953	1.56025	-0.42494
58.35543	-0.33584	2.52407	-1.49529	1.56056	-0.42476
58.3888	-0.33664	2.52504	-1.49535	1.56093	-0.42462
58.42202	-0.3378	2.52547	-1.49543	1.56208	-0.4246
58.455	-0.33902	2.52585	-1.49554	1.56413	-0.42456
58.48851	-0.34124	2.52622	-1.49569	1.5656	-0.42446
58.52058	-0.3436	2.52737	-1.49586	1.56633	-0.42433

58.55519	-0.34552	2.52994	-1.49601	1.56662	-0.42411
58.58807	-0.34784	2.53172	-1.49612	1.56681	-0.42396
58.621	-0.34958	2.53209	-1.49621	1.56701	-0.42387
58.65447	-0.35098	2.53239	-1.49629	1.56723	-0.42381
58.6888	-0.352	2.5326	-1.49641	1.56749	-0.42381
58.7216	-0.35258	2.53286	-1.49649	1.56777	-0.42377
58.75399	-0.35282	2.53318	-1.49655	1.56808	-0.42363
58.78798	-0.35324	2.53355	-1.49658	1.5684	-0.42349
58.82093	-0.35422	2.53406	-1.49665	1.56875	-0.4233
58.85382	-0.3563	2.5348	-1.49674	1.56912	-0.42319
58.88744	-0.35806	2.53555	-1.49681	1.56948	-0.42313
58.92011	-0.35942	2.53607	-1.49681	1.56982	-0.42302
58.95404	-0.35988	2.5365	-1.4968	1.57017	-0.42291
58.98666	-0.36048	2.53722	-1.49676	1.5705	-0.4228
59.01982	-0.36268	2.53838	-1.49674	1.57087	-0.42271
59.0533	-0.3659	2.54075	-1.49681	1.57125	-0.42268
59.08626	-0.3684	2.54254	-1.49691	1.57177	-0.42264
59.11934	-0.37064	2.54328	-1.49719	1.57217	-0.42258
59.1531	-0.37132	2.5437	-1.49796	1.57255	-0.42253
59.18676	-0.37138	2.54407	-1.49832	1.57289	-0.42253
59.2197	-0.37152	2.54441	-1.49843	1.57321	-0.42255
59.25334	-0.37154	2.54475	-1.49843	1.5735	-0.42255
59.28545	-0.37182	2.54505	-1.49838	1.57379	-0.42242
59.31926	-0.37258	2.54536	-1.49842	1.57412	-0.42215
59.35341	-0.37304	2.54566	-1.49852	1.57459	-0.42184
59.38725	-0.37362	2.54601	-1.49862	1.57549	-0.4217
59.41949	-0.37426	2.54675	-1.49875	1.57691	-0.42164
59.45276	-0.37496	2.54777	-1.49887	1.57785	-0.42169
59.48523	-0.37612	2.54853	-1.49902	1.57818	-0.42174
59.51914	-0.37698	2.54906	-1.49921	1.57847	-0.42178

59.55249	-0.37752	2.54937	-1.49945	1.57882	-0.42176
59.58611	-0.37778	2.54967	-1.49977	1.57924	-0.42169
59.61808	-0.3779	2.55	-1.49999	1.5798	-0.4216
59.6514	-0.37806	2.55084	-1.5002	1.58015	-0.4216
59.68584	-0.37852	2.55249	-1.50038	1.58041	-0.42165
59.71913	-0.3796	2.5533	-1.50049	1.58072	-0.42172
59.75222	-0.38152	2.55366	-1.50061	1.58116	-0.42176
59.7853	-0.38304	2.55393	-1.50073	1.58221	-0.42168
59.81839	-0.38338	2.55414	-1.5008	1.58279	-0.42154
59.85112	-0.38344	2.55446	-1.5009	1.58307	-0.42144
59.88476	-0.3835	2.55506	-1.50107	1.58329	-0.42138
59.91831	-0.38366	2.5559	-1.50117	1.58346	-0.42143
59.9514	-0.3843	2.55699	-1.50129	1.58367	-0.42149
59.98401	-0.38544	2.55766	-1.50137	1.58397	-0.42146
60.0173	-0.38624	2.55799	-1.50143	1.5843	-0.42135
60.05066	-0.38656	2.55832	-1.50156	1.58467	-0.42119
60.08441	-0.38696	2.55871	-1.50172	1.58525	-0.42102
60.11652	-0.38746	2.55947	-1.50185	1.58594	-0.42093
60.15016	-0.38812	2.56032	-1.5019	1.58679	-0.4209
60.18402	-0.38964	2.56077	-1.50195	1.58724	-0.42094
60.21804	-0.3903	2.5611	-1.5021	1.58765	-0.42098
60.2507	-0.3904	2.56159	-1.50254	1.58794	-0.42103
60.28436	-0.3905	2.562	-1.5034	1.58829	-0.42105
60.31692	-0.39062	2.56242	-1.50371	1.5887	-0.42107
60.34985	-0.39082	2.56278	-1.50384	1.5891	-0.42115
60.3832	-0.39122	2.56308	-1.50392	1.58956	-0.42118
60.4165	-0.3917	2.56346	-1.50403	1.58992	-0.42117
60.44949	-0.39226	2.5639	-1.50415	1.59017	-0.42104
60.48322	-0.39246	2.56428	-1.50433	1.59043	-0.4205
60.51688	-0.39266	2.56469	-1.50451	1.59074	-0.42037

60.5501	-0.39288	2.56504	-1.50469	1.59116	-0.4203
60.5831	-0.39316	2.5654	-1.50489	1.59231	-0.42025
60.61584	-0.39352	2.56597	-1.50508	1.59437	-0.42028
60.64938	-0.3937	2.5665	-1.50528	1.59486	-0.42028
60.68316	-0.39392	2.56709	-1.50546	1.59521	-0.42025
60.71555	-0.39404	2.56756	-1.50564	1.59555	-0.42026
60.74915	-0.39418	2.56798	-1.50585	1.59588	-0.42025
60.78276	-0.39448	2.5684	-1.50608	1.59698	-0.42022
60.81629	-0.39486	2.56929	-1.50631	1.5977	-0.4202
60.84872	-0.39516	2.5705	-1.50652	1.59795	-0.42017
60.88218	-0.39556	2.57129	-1.50667	1.59814	-0.4201
60.91479	-0.39588	2.57179	-1.50679	1.59828	-0.42004
60.94861	-0.39628	2.57217	-1.50689	1.59841	-0.42
60.98189	-0.39668	2.57262	-1.50702	1.59869	-0.41999
61.01526	-0.39686	2.5734	-1.50721	1.59956	-0.42003
61.04812	-0.397	2.57448	-1.50748	1.60118	-0.4202
61.08196	-0.39716	2.5756	-1.50773	1.60326	-0.42031
61.11555	-0.3974	2.57599	-1.50788	1.60437	-0.42038
61.14855	-0.39808	2.57631	-1.50798	1.60465	-0.42042
61.18166	-0.39906	2.57667	-1.50806	1.60481	-0.42045
61.21528	-0.3997	2.57707	-1.5082	1.60495	-0.42053
61.2482	-0.39988	2.5775	-1.50835	1.60514	-0.42063
61.2825	-0.4001	2.57801	-1.50853	1.60555	-0.42072
61.31414	-0.40046	2.57842	-1.50876	1.60669	-0.42079
61.34786	-0.40108	2.57884	-1.50918	1.60792	-0.42079
61.38103	-0.40236	2.57924	-1.50952	1.60844	-0.42073
61.41454	-0.40342	2.57959	-1.50972	1.60865	-0.4207
61.44843	-0.40468	2.57991	-1.50991	1.60881	-0.42078
61.4814	-0.40572	2.58022	-1.51015	1.60895	-0.42104
61.51492	-0.40612	2.58077	-1.51062	1.60914	-0.42172

61.54752	-0.4064	2.58183	-1.51147	1.60943	-0.42194
61.5803	-0.4067	2.58273	-1.51181	1.60969	-0.42193
61.6143	-0.40734	2.58332	-1.51206	1.60991	-0.42192
61.64792	-0.40898	2.58383	-1.51229	1.6101	-0.4219
61.68072	-0.4105	2.58456	-1.51253	1.6103	-0.42191
61.7141	-0.4114	2.5859	-1.51275	1.61063	-0.42204
61.74743	-0.41222	2.58806	-1.51299	1.61096	-0.42214
61.78062	-0.41288	2.58943	-1.51327	1.61118	-0.42226
61.81348	-0.41404	2.59056	-1.51367	1.61138	-0.42239
61.84749	-0.41616	2.59131	-1.51412	1.61157	-0.42246
61.88044	-0.41782	2.59167	-1.51438	1.61183	-0.42254
61.91372	-0.41908	2.59192	-1.51454	1.61228	-0.42259
61.94712	-0.42038	2.59221	-1.51473	1.61269	-0.42254
61.98042	-0.42168	2.59261	-1.51492	1.61295	-0.42247
62.01387	-0.42398	2.59342	-1.51515	1.61319	-0.42239
62.04639	-0.4257	2.59402	-1.51552	1.61338	-0.42237
62.07966	-0.4265	2.59442	-1.51604	1.61359	-0.42245
62.11365	-0.42704	2.59475	-1.51639	1.61393	-0.42255
62.1466	-0.42758	2.59522	-1.5167	1.61449	-0.42264
62.1805	-0.42866	2.59677	-1.51693	1.61495	-0.42269
62.21332	-0.43062	2.59864	-1.51713	1.61524	-0.4227
62.24621	-0.43384	2.60059	-1.51731	1.61545	-0.42273
62.27978	-0.43824	2.6012	-1.5175	1.61562	-0.42279
62.31274	-0.44214	2.60148	-1.51771	1.61581	-0.42289
62.34618	-0.44428	2.60173	-1.518	1.61602	-0.42301
62.37974	-0.44514	2.60198	-1.51854	1.61638	-0.42324
62.41261	-0.44542	2.60227	-1.51918	1.61706	-0.42354
62.44581	-0.44556	2.60259	-1.51948	1.61736	-0.42371
62.47968	-0.4459	2.60294	-1.51973	1.61761	-0.42379
62.51242	-0.44628	2.60332	-1.51999	1.61782	-0.42385

62.54522	-0.44698	2.60366	-1.52031	1.61803	-0.42395
62.57812	-0.44784	2.60394	-1.52081	1.61829	-0.42407
62.61258	-0.44858	2.60415	-1.52142	1.61859	-0.42421
62.64532	-0.44982	2.60435	-1.52247	1.61891	-0.4243
62.67854	-0.45086	2.60462	-1.5231	1.61917	-0.42435
62.7112	-0.45166	2.60493	-1.52345	1.61943	-0.42447
62.7439	-0.45226	2.60527	-1.52379	1.61962	-0.42471
62.77757	-0.4532	2.60565	-1.52404	1.61976	-0.4251
62.811	-0.45528	2.60639	-1.5243	1.61993	-0.42537
62.8434	-0.4579	2.60738	-1.5246	1.62021	-0.42559
62.87705	-0.46066	2.60793	-1.52494	1.62053	-0.42579
62.91165	-0.46192	2.60857	-1.52555	1.62084	-0.42599
62.9438	-0.46278	2.60973	-1.52666	1.62112	-0.42615
62.97676	-0.46452	2.61057	-1.52737	1.62139	-0.42629
63.0098	-0.46676	2.61108	-1.52775	1.62168	-0.42642
63.04401	-0.46934	2.6114	-1.52803	1.62213	-0.42657
63.077	-0.47204	2.61165	-1.52827	1.62263	-0.42675
63.11078	-0.47422	2.61192	-1.52852	1.62319	-0.42698
63.14389	-0.47746	2.61226	-1.52876	1.62349	-0.42744
63.17715	-0.47984	2.61332	-1.52903	1.62378	-0.42777
63.2101	-0.4804	2.61513	-1.52933	1.62405	-0.42796
63.24306	-0.48092	2.61728	-1.52965	1.62436	-0.42804
63.27664	-0.48148	2.61829	-1.52993	1.62479	-0.42809
63.30972	-0.48322	2.61862	-1.53017	1.62521	-0.42826
63.34325	-0.48678	2.61884	-1.53044	1.6255	-0.42888
63.377	-0.4897	2.61909	-1.53075	1.62582	-0.42916
63.41024	-0.49128	2.61936	-1.53107	1.62616	-0.42935
63.44316	-0.49378	2.61969	-1.5314	1.62649	-0.42949
63.47613	-0.49662	2.61998	-1.53173	1.62676	-0.42962
63.50961	-0.50022	2.62031	-1.53218	1.62694	-0.43007

63.5425	-0.5038	2.62067	-1.53287	1.62709	-0.43097
63.577	-0.50522	2.62101	-1.53364	1.62727	-0.4315
63.61016	-0.50612	2.62134	-1.53459	1.62747	-0.43193
63.64316	-0.50658	2.62162	-1.53509	1.62774	-0.43218
63.67662	-0.50824	2.62187	-1.53564	1.62803	-0.43229
63.71008	-0.511	2.62211	-1.53652	1.62831	-0.43247
63.74278	-0.51326	2.62234	-1.5372	1.62859	-0.4327
63.77634	-0.51518	2.6225	-1.53886	1.62877	-0.43308
63.80918	-0.51604	2.62271	-1.54067	1.62893	-0.43371
63.84256	-0.5175	2.62302	-1.54163	1.62925	-0.43431
63.87582	-0.51952	2.62356	-1.54212	1.62956	-0.43478
63.90882	-0.52198	2.6248	-1.54267	1.62985	-0.43513
63.94196	-0.5246	2.62564	-1.54337	1.63014	-0.43541
63.97568	-0.52722	2.62633	-1.54412	1.63038	-0.43564
64.0089	-0.5302	2.627	-1.54455	1.63064	-0.43589
64.04274	-0.53246	2.62739	-1.54502	1.63093	-0.43627
64.07608	-0.53498	2.62773	-1.54533	1.63113	-0.43659
64.10916	-0.53748	2.62796	-1.54561	1.63124	-0.43689
64.14224	-0.5394	2.6281	-1.54598	1.63141	-0.43718
64.17474	-0.54182	2.62826	-1.54634	1.63168	-0.43749
64.2082	-0.54432	2.6286	-1.54686	1.6322	-0.4378
64.24129	-0.54616	2.62965	-1.54811	1.63303	-0.43812
64.27508	-0.5497	2.631	-1.54861	1.63337	-0.43838
64.30873	-0.5531	2.63173	-1.5491	1.63351	-0.4387
64.3414	-0.55558	2.63199	-1.5496	1.63361	-0.43949
64.3746	-0.5594	2.63224	-1.5501	1.63373	-0.44121
64.4085	-0.56226	2.63254	-1.55165	1.63395	-0.44269
64.442	-0.5641	2.63286	-1.55388	1.63456	-0.44353
64.47402	-0.56458	2.63323	-1.55616	1.63509	-0.44383
64.50705	-0.56458	2.63356	-1.55678	1.63537	-0.4441

64.54088	-0.56468	2.63383	-1.55711	1.63558	-0.44436
64.5741	-0.56636	2.6341	-1.55741	1.63569	-0.4446
64.60761	-0.57192	2.63435	-1.5578	1.63584	-0.44487
64.64174	-0.5781	2.63455	-1.55828	1.63605	-0.44534
64.67514	-0.58294	2.63478	-1.55928	1.6363	-0.44693
64.70789	-0.5855	2.63505	-1.56011	1.63697	-0.44898
64.74127	-0.5864	2.63541	-1.56091	1.63737	-0.44974
64.7746	-0.58734	2.63582	-1.56214	1.63786	-0.45005
64.807	-0.58876	2.63617	-1.56341	1.63869	-0.45034
64.84074	-0.59096	2.63647	-1.56462	1.63927	-0.45081
64.87418	-0.59406	2.63667	-1.56589	1.63956	-0.45213
64.90761	-0.5983	2.63688	-1.56636	1.63976	-0.4536
64.9408	-0.60092	2.63713	-1.56668	1.63985	-0.45399
64.97316	-0.60486	2.63748	-1.56698	1.63994	-0.45436
65.00674	-0.60834	2.63919	-1.56735	1.64007	-0.4547
65.04043	-0.6111	2.64076	-1.5683	1.64019	-0.45502
65.07426	-0.61436	2.64152	-1.5719	1.64033	-0.45573
65.10742	-0.61676	2.64219	-1.57617	1.64041	-0.45716
65.14037	-0.6209	2.64253	-1.57989	1.64055	-0.45929
65.17407	-0.62362	2.64286	-1.58153	1.64077	-0.46113
65.20682	-0.62574	2.64318	-1.58208	1.64096	-0.46299
65.23948	-0.62778	2.64345	-1.58306	1.64113	-0.46397
65.27247	-0.63054	2.6437	-1.58602	1.64126	-0.46451
65.3056	-0.63504	2.64393	-1.59087	1.64134	-0.46482
65.33963	-0.64226	2.64422	-1.59381	1.64152	-0.46515
65.37268	-0.64658	2.64459	-1.59536	1.64171	-0.46637
65.40569	-0.64962	2.64535	-1.59705	1.64191	-0.46985
65.43994	-0.6516	2.64604	-1.59861	1.64216	-0.47601
65.47217	-0.65392	2.64677	-1.60131	1.64237	-0.47834
65.50534	-0.65538	2.64744	-1.60479	1.64256	-0.47901

65.53888	-0.65828	2.64788	-1.60602	1.64278	-0.47961
65.57161	-0.6594	2.64822	-1.60661	1.64297	-0.48016
65.60576	-0.66128	2.64849	-1.60798	1.64321	-0.4809
65.63922	-0.66678	2.64875	-1.61019	1.64353	-0.4814
65.6725	-0.6711	2.64904	-1.61404	1.64374	-0.48197
65.70564	-0.67702	2.64937	-1.61778	1.64392	-0.48276
65.73974	-0.68102	2.64977	-1.61944	1.64409	-0.48396
65.77284	-0.68502	2.65012	-1.62058	1.64426	-0.48522
65.80576	-0.68886	2.65048	-1.62168	1.64448	-0.4874
65.83853	-0.69224	2.65086	-1.62365	1.6448	-0.49101
65.87122	-0.6927	2.65114	-1.62755	1.64508	-0.49413
65.90534	-0.69432	2.65143	-1.63177	1.64529	-0.49562
65.93855	-0.6991	2.65175	-1.63514	1.64545	-0.4966
65.97156	-0.70596	2.65204	-1.63732	1.6455	-0.49751
66.00532	-0.71252	2.65232	-1.63899	1.64564	-0.49943
66.0384	-0.71578	2.65255	-1.64169	1.6459	-0.50168
66.07164	-0.71676	2.65278	-1.64736	1.64617	-0.50294
66.10386	-0.71894	2.65309	-1.65392	1.64642	-0.50418
66.1375	-0.72196	2.6535	-1.65982	1.64659	-0.50593
66.17157	-0.72624	2.65389	-1.66325	1.64668	-0.50867
66.20497	-0.7323	2.65417	-1.66449	1.64675	-0.51054
66.23759	-0.73648	2.65437	-1.66526	1.64685	-0.51192
66.2716	-0.73874	2.65459	-1.6669	1.6471	-0.51344
66.30411	-0.74086	2.65487	-1.66979	1.64758	-0.51575
66.3375	-0.74262	2.65516	-1.67586	1.64803	-0.51801
66.37076	-0.74612	2.65541	-1.68355	1.6483	-0.51981
66.40424	-0.7511	2.65569	-1.68818	1.64839	-0.52147
66.43778	-0.7579	2.65605	-1.69102	1.6484	-0.52363
66.47081	-0.76348	2.65674	-1.69249	1.64848	-0.52762
66.5039	-0.7687	2.65835	-1.69481	1.64866	-0.53091

66.53728	-0.7746	2.659	-1.69853	1.64923	-0.53316
66.57007	-0.77724	2.65926	-1.70169	1.6497	-0.5357
66.60269	-0.78062	2.65944	-1.70565	1.64992	-0.5374
66.63745	-0.78234	2.65961	-1.71024	1.65008	-0.53943
66.67031	-0.7852	2.65982	-1.71418	1.65023	-0.54247
66.70369	-0.78852	2.66073	-1.71899	1.65043	-0.54505
66.73618	-0.79096	2.66298	-1.72271	1.65101	-0.5467
66.7701	-0.7951	2.66596	-1.72462	1.65138	-0.54766
66.8031	-0.8002	2.6679	-1.72723	1.65154	-0.54935
66.83626	-0.8041	2.66833	-1.73049	1.6516	-0.5521
66.87062	-0.80996	2.66852	-1.73503	1.6516	-0.55671
66.90296	-0.8161	2.66863	-1.7377	1.65162	-0.56257
66.93681	-0.81938	2.66874	-1.73908	1.65171	-0.56645
66.97037	-0.82268	2.66891	-1.74072	1.65187	-0.56873
67.00332	-0.82752	2.66914	-1.74219	1.65203	-0.57216
67.03676	-0.82892	2.66981	-1.74591	1.65222	-0.57609
67.06975	-0.83316	2.6707	-1.75421	1.65241	-0.57855
67.1028	-0.83768	2.67103	-1.76211	1.65257	-0.58012
67.1353	-0.84184	2.67131	-1.76823	1.65273	-0.58171
67.16928	-0.84554	2.67153	-1.77096	1.65288	-0.58335
67.20278	-0.85056	2.67175	-1.77166	1.65305	-0.58637
67.2365	-0.85682	2.67199	-1.77252	1.65323	-0.58948
67.26946	-0.8624	2.67221	-1.77481	1.65344	-0.59185
67.3027	-0.86676	2.67246	-1.78173	1.65363	-0.59455
67.33563	-0.8708	2.67268	-1.79245	1.65376	-0.59707
67.36868	-0.87466	2.67299	-1.80014	1.65385	-0.59958
67.40154	-0.87828	2.67335	-1.80452	1.65394	-0.60257
67.43516	-0.8815	2.67371	-1.80602	1.65402	-0.60456
67.46864	-0.88524	2.67415	-1.80862	1.65416	-0.60804
67.50099	-0.88948	2.67455	-1.81315	1.65433	-0.61246

67.53519	-0.89644	2.67482	-1.81968	1.65441	-0.61572
67.56824	-0.90278	2.67514	-1.82568	1.65444	-0.61943
67.60111	-0.90688	2.67547	-1.83173	1.6545	-0.62163
67.63504	-0.91094	2.67586	-1.83527	1.65454	-0.62478
67.6683	-0.9151	2.67615	-1.83931	1.65463	-0.62973
67.70172	-0.91834	2.67639	-1.84325	1.65474	-0.63485
67.73589	-0.92302	2.67662	-1.84749	1.65486	-0.64059
67.76856	-0.92808	2.67685	-1.85406	1.655	-0.64518
67.80098	-0.93274	2.67711	-1.85901	1.65517	-0.64847
67.83386	-0.93786	2.67744	-1.86157	1.65534	-0.65134
67.8674	-0.94236	2.67787	-1.86698	1.65545	-0.65461
67.90106	-0.94678	2.67835	-1.87298	1.65557	-0.65892
67.93366	-0.95064	2.67884	-1.87944	1.65573	-0.66289
67.96787	-0.95398	2.67918	-1.88304	1.65583	-0.66871
68.00078	-0.95936	2.67945	-1.88593	1.65596	-0.67526
68.03422	-0.96466	2.67961	-1.88907	1.65609	-0.68104
68.06695	-0.9677	2.67979	-1.89715	1.65626	-0.68542
68.10105	-0.9725	2.68004	-1.90978	1.65657	-0.68923
68.13362	-0.97612	2.68044	-1.92106	1.65682	-0.69247
68.16699	-0.98066	2.6818	-1.92564	1.65698	-0.69594
68.20015	-0.9883	2.68292	-1.93019	1.6571	-0.70052
68.23231	-0.99558	2.68339	-1.93302	1.65713	-0.70368
68.2665	-1.00044	2.68379	-1.93717	1.65721	-0.70681
68.29976	-1.00288	2.68412	-1.94927	1.65729	-0.71233
68.33298	-1.00444	2.68444	-1.96185	1.65733	-0.71882
68.36691	-1.00838	2.68471	-1.96899	1.65734	-0.72498
68.39926	-1.01492	2.68493	-1.97283	1.65738	-0.72956
68.43255	-1.02376	2.68522	-1.97619	1.6576	-0.73248
68.46625	-1.02766	2.6855	-1.97908	1.65808	-0.73595
68.49951	-1.03036	2.68575	-1.98717	1.6586	-0.74162

68.5331	-1.03608	2.68596	-1.99606	1.65879	-0.74899
68.56641	-1.04224	2.68613	-2.00338	1.65892	-0.7566
68.59918	-1.04854	2.68628	-2.01283	1.65912	-0.76214
68.6323	-1.05504	2.68648	-2.02248	1.65931	-0.76507
68.66596	-1.05806	2.68673	-2.02848	1.65935	-0.76866
68.70007	-1.06408	2.68701	-2.03518	1.65931	-0.77606
68.73222	-1.071	2.68734	-2.0421	1.65918	-0.78771
68.76614	-1.07432	2.688	-2.05193	1.65914	-0.79991
68.79895	-1.07724	2.68844	-2.06078	1.65921	-0.80883
68.83276	-1.08214	2.68876	-2.07064	1.65938	-0.81326
68.86536	-1.087	2.689	-2.0763	1.65954	-0.81648
68.89859	-1.0957	2.68924	-2.08551	1.65978	-0.81685
68.9314	-1.10176	2.68944	-2.10088	1.65995	-0.81794
68.96509	-1.10556	2.68971	-2.11019	1.66005	-0.82176
68.99874	-1.1099	2.69006	-2.11772	1.6601	-0.82847
69.03147	-1.1127	2.69039	-2.12858	1.66019	-0.83999
69.065	-1.11718	2.69079	-2.13987	1.66017	-0.85127
69.09802	-1.12384	2.69126	-2.15816	1.66018	-0.86274
69.13228	-1.131	2.69183	-2.16783	1.6602	-0.87183
69.16519	-1.13812	2.69221	-2.17019	1.66028	-0.87901
69.19848	-1.14494	2.69253	-2.1778	1.66043	-0.88467
69.2316	-1.14812	2.69284	-2.18898	1.66058	-0.88641
69.26495	-1.1547	2.69314	-2.19672	1.66071	-0.89225
69.29702	-1.1582	2.69339	-2.20881	1.66081	-0.90355
69.33074	-1.1608	2.69367	-2.21891	1.66089	-0.91733
69.36494	-1.1668	2.69421	-2.23209	1.6609	-0.92575
69.39735	-1.17174	2.69617	-2.24665	1.66083	-0.93204
69.43092	-1.1796	2.69996	-2.26062	1.66069	-0.9358
69.46368	-1.18884	2.70251	-2.27503	1.66067	-0.93973
69.49732	-1.19622	2.70309	-2.28807	1.6607	-0.95401

69.53098	-1.20128	2.70327	-2.30114	1.66081	-0.96932
69.56449	-1.20802	2.70344	-2.31474	1.66099	-0.98039
69.59741	-1.214	2.70367	-2.32258	1.66122	-0.98655
69.63142	-1.21964	2.70399	-2.33335	1.66141	-0.99206
69.66388	-1.22306	2.70457	-2.34489	1.66156	-1.00242
69.69811	-1.22782	2.70523	-2.35896	1.66166	-1.00783
69.73036	-1.23442	2.70579	-2.37704	1.66165	-1.01619
69.76416	-1.24132	2.70642	-2.39371	1.66164	-1.02242
69.79619	-1.24994	2.70684	-2.41442	1.66167	-1.03582
69.83028	-1.2569	2.7071	-2.43071	1.66173	-1.05438
69.86324	-1.2628	2.7073	-2.44116	1.66179	-1.06878
69.8975	-1.26804	2.70749	-2.45531	1.66181	-1.07787
69.93072	-1.2742	2.70765	-2.46829	1.6618	-1.08488
69.96352	-1.28142	2.70781	-2.47831	1.66181	-1.09253
69.9975	-1.28784	2.70793	-2.50331	1.66181	-1.10699
70.03018	-1.2955	2.70804	-2.5204	1.66183	-1.12319
70.06392	-1.30334	2.70826	-2.54053	1.66179	-1.13246
70.09722	-1.30892	2.70861	-2.5611	1.66179	-1.14204
70.12922	-1.3119	2.70928	-2.57673	1.66177	-1.15638
70.16311	-1.3182	2.70994	-2.58599	1.66169	-1.16926
70.19751	-1.32444	2.71023	-2.60179	1.6616	-1.18305
70.2292	-1.33262	2.71048	-2.62012	1.6616	-1.19515
70.26282	-1.34294	2.7107	-2.64084	1.66161	-1.20806
70.29605	-1.35044	2.71102	-2.6637	1.66166	-1.22061
70.32868	-1.35626	2.71242	-2.68743	1.66167	-1.23356
70.36304	-1.3637	2.71447	-2.70231	1.66164	-1.24777
70.39606	-1.37044	2.71549	-2.71694	1.66159	-1.26665
70.42875	-1.37708	2.7158	-2.7407	1.66159	-1.27799
70.46312	-1.38572	2.716	-2.75945	1.66157	-1.28957
70.49563	-1.39338	2.71611	-2.78096	1.66152	-1.30286

70.52957	-1.4004	2.71622	-2.80693	1.66131	-1.31605
70.56255	-1.40548	2.71632	-2.82508	1.66122	-1.33554
70.59622	-1.41162	2.71634	-2.84911	1.66131	-1.35823
70.62839	-1.41838	2.71642	-2.87258	1.66199	-1.37297
70.6613	-1.42782	2.71655	-2.89335	1.66251	-1.38618
70.69556	-1.43716	2.71673	-2.91523	1.66271	-1.40245
70.72881	-1.44556	2.71697	-2.93599	1.66266	-1.41957
70.76232	-1.45374	2.7172	-2.95955	1.66232	-1.43148
70.79486	-1.461	2.71737	-2.98659	1.66212	-1.45099
70.82838	-1.46876	2.71749	-3.01818	1.66201	-1.47504
70.86194	-1.47778	2.7176	-3.04641	1.66195	-1.49954
70.89481	-1.4849	2.7177	-3.06634	1.66203	-1.52104
70.92851	-1.4924	2.71779	-3.08494	1.66209	-1.5373
70.96094	-1.49882	2.71789	-3.10917	1.66213	-1.54621
70.99488	-1.50562	2.71801	-3.13792	1.66209	-1.56417
71.02772	-1.51336	2.71813	-3.16207	1.662-1.58686	
71.06128	-1.52042	2.71829	-3.18846	1.66195	-1.61096
71.0943	-1.52602	2.71845	-3.21533	1.66193	-1.63019
71.12739	-1.53482	2.71863	-3.24085	1.66192	-1.64766
71.16119	-1.54606	2.7189	-3.27609	1.66192	-1.67388
71.19475	-1.55626	2.71965	-3.3072	1.66182	-1.70043
71.22803	-1.56562	2.72034	-3.33512	1.6617	-1.72957
71.26072	-1.57106	2.72079	-3.35658	1.66157	-1.75316
71.29389	-1.57758	2.72097	-3.37945	1.6615	-1.76846
71.32728	-1.58628	2.72111	-3.4048	1.66149	-1.78909
71.36113	-1.59596	2.72127	-3.43275	1.66146	-1.816
71.39442	-1.60668	2.72149	-3.4651	1.66138	-1.83667
71.42788	-1.61564	2.72198	-3.49605	1.66135	-1.8599
71.46148	-1.62402	2.72241	-3.52791	1.66133	-1.88848
71.49371	-1.63352	2.72273	-3.5525	1.66135	-1.91509

71.52797	-1.64238	2.72302	-3.58322	1.66141	-1.94402
71.5605	-1.64912	2.72325	-3.61239	1.66144	-1.97409
71.59418	-1.65724	2.72351	-3.64786	1.66137	-1.99615
71.627	-1.66776	2.72414	-3.6904	1.66127	-2.01789
71.66022	-1.67672	2.72465	-3.72497	1.66117	-2.04578
71.69361	-1.68718	2.72512	-3.75002	1.66096	-2.07659
71.7266	-1.69458	2.72556	-3.77312	1.66085	-2.10574
71.76013	-1.7021	2.72588	-3.79835	1.66074	-2.13392
71.79318	-1.7114	2.72624	-3.83594	1.66047	-2.16918
71.82691	-1.72122	2.72683	-3.87603	1.66032	-2.20054
71.86038	-1.7286	2.72746	-3.91261	1.66026	-2.23306
71.8934	-1.73924	2.72795	-3.94949	1.66019	-2.26712
71.92618	-1.75064	2.72831	-3.98147	1.66022	-2.29106
71.95947	-1.76144	2.72855	-4.01754	1.66022	-2.31481
71.9925	-1.77362	2.7287	-4.04909	1.66012	-2.34643
72.02628	-1.78262	2.72884	-4.07777	1.66003	-2.38035
72.06	-1.79136	2.72903	-4.10963	1.65989	-2.42178
72.09284	-1.8013	2.72959	-4.13958	1.65971	-2.45924
72.12585	-1.8111	2.73057	-4.17514	1.65956	-2.49327
72.15884	-1.82162	2.73186	-4.20999	1.65944	-2.52638
72.19231	-1.83362	2.73274	-4.2445	1.65937	-2.55401
72.22554	-1.84432	2.73321	-4.29275	1.65939	-2.58554
72.2594	-1.8551	2.73376	-4.33504	1.65942	-2.62098
72.29205	-1.866	2.73425	-4.37034	1.65941	-2.65277
72.3253	-1.87726	2.73457	-4.40303	1.65922	-2.69711
72.35791	-1.88854	2.73487	-4.43494	1.65884	-2.7385
72.39234	-1.89982	2.73513	-4.46923	1.65841	-2.77922
72.42608	-1.90984	2.73542	-4.50948	1.65809	-2.81324
72.45969	-1.92046	2.73573	-4.54882	1.65793	-2.84184
72.49257	-1.9325	2.73602	-4.59126	1.65783	-2.87742

72.52551	-1.94396	2.73629	-4.63016	1.65775	-2.91626
72.55803	-1.9563	2.73654	-4.67033	1.65774	-2.95885
72.59144	-1.97068	2.73682	-4.71021	1.65768	-3.00272
72.62475	-1.98202	2.7371	-4.74648	1.65756	-3.04119
72.65749	-1.99446	2.73742	-4.78981	1.65739	-3.08143
72.6905	-2.0066	2.7377	-4.83124	1.65719	-3.12186
72.72426	-2.0165	2.73805	-4.87589	1.65701	-3.16175
72.75778	-2.02988	2.73909	-4.91951	1.65688	-3.20208
72.7918	-2.04128	2.74001	-4.9578	1.65678	-3.23993
72.82496	-2.05328	2.7407	-4.99414	1.65667	-3.28062
72.85774	-2.0653	2.74154	-5.03353	1.65655	-3.3253
72.89108	-2.0772	2.74181	-5.07491	1.6564	-3.36768
72.92408	-2.09288	2.742	-5.11916	1.6563	-3.40869
72.95746	-2.10812	2.74223	-5.16214	1.65621	-3.45111
72.99081	-2.12378	2.74243	-5.20146	1.65607	-3.49032
73.02455	-2.13638	2.74269	-5.23818	1.65595	-3.53373
73.05798	-2.14882	2.74301	-5.2849	1.65576	-3.57945
73.09126	-2.1619	2.74332	-5.33337	1.65552	-3.6247
73.12375	-2.17728	2.74364	-5.38141	1.65536	-3.67212
73.15634	-2.19242	2.74395	-5.42624	1.65518	-3.71452
73.18995	-2.20602	2.74419	-5.46488	1.65498	-3.75678
73.22321	-2.21962	2.74439	-5.50361	1.65481	-3.79658
73.25766	-2.23418	2.74455	-5.55048	1.65461	-3.83354
73.29074	-2.24838	2.74472	-5.60063	1.65452	-3.87316
73.32324	-2.26386	2.7449	-5.64647	1.65447	-3.92
73.3559	-2.27702	2.74506	-5.69185	1.65424	-3.96601
73.38847	-2.29228	2.74522	-5.73704	1.65357	-4.01525
73.42268	-2.30794	2.74549	-5.77798	1.65148	-4.0606
73.45615	-2.32434	2.74611	-5.82279	1.65005	-4.09965
73.48993	-2.34166	2.74672	-5.86508	1.64983	-4.14227

73.52359	-2.3565	2.74709	-5.91072	1.64967	-4.18335
73.5564	-2.37194	2.74732	-5.95871	1.64941	-4.21959
73.58965	-2.38682	2.74751	-6.00406	1.64846	-4.26076
73.62231	-2.40534	2.74773	-6.04603	1.64778	-4.29458
73.65509	-2.42346	2.7479	-6.09283	1.64753	-4.31978
73.6885	-2.44194	2.74808	-6.14315	1.64739	-4.35797
73.72324	-2.45714	2.74826	-6.18759	1.64733	-4.39669
73.75593	-2.47042	2.7485	-6.23313	1.64728	-4.4358
73.7897	-2.48566	2.74989	-6.2754	1.64719	-4.47694
73.82261	-2.5032	2.7529	-6.31792	1.64707	-4.50859
73.8559	-2.5206	2.75468	-6.36322	1.64684	-4.53418
73.88882	-2.54052	2.75513	-6.40792	1.64663	-4.55728
73.9225	-2.557	2.75524	-6.44497	1.64641	-4.58793
73.95447	-2.57578	2.75537	-6.48623	1.64619	-4.62275
73.98824	-2.59378	2.75551	-6.5309	1.64595	-4.65205
74.02211	-2.60944	2.75571	-6.57291	1.64562	-4.67431
74.0555	-2.62626	2.75596	-6.61849	1.64531	-4.69733
74.08882	-2.64592	2.75622	-6.66351	1.64506	-4.71675
74.12152	-2.66688	2.75677	-6.70838	1.64483	-4.74348
74.15439	-2.68904	2.75827	-6.75314	1.64455	-4.7585
74.18818	-2.70776	2.75963	-6.79452	1.64346	-4.78246
74.22134	-2.72558	2.75997	-6.82999	1.64106	-4.80237
74.25401	-2.74272	2.76007	-6.86982	1.63867	-4.8195
74.2881	-2.76078	2.76008	-6.91354	1.63784	-4.84265
74.32176	-2.78154	2.76013	-6.95625	1.63758	-4.85587
74.35428	-2.80258	2.76031	-6.99864	1.63743	-4.86366
74.38816	-2.8235	2.76088	-7.03951	1.6373	-4.8703
74.42085	-2.84446	2.76181	-7.07149	1.63693	-4.87637
74.45382	-2.86546	2.76282	-7.10395	1.63508	-4.88787
74.48671	-2.88512	2.76351	-7.14159	1.6332	-4.90034

74.5203	-2.90496	2.76391	-7.17695	1.63201	-4.90313
74.55316	-2.92664	2.76415	-7.21295	1.63133	-4.9056
74.58628	-2.94922	2.76438	-7.25259	1.63099	-4.90524
74.61948	-2.97234	2.76456	-7.28573	1.63065	-4.90944
74.65362	-2.99574	2.76472	-7.31767	1.63052	-4.91486
74.68701	-3.01618	2.76482	-7.34737	1.63041	-4.92136
74.72016	-3.03722	2.76485	-7.37836	1.63023	-4.91764
74.75232	-3.05938	2.76495	-7.40866	1.62962	-4.91811
74.78616	-3.0825	2.76519	-7.44211	1.62827	-4.92371
74.8191	-3.10454	2.76555	-7.47669	1.62736	-4.93053
74.85376	-3.12716	2.76603	-7.50173	1.6267	-4.93489
74.88635	-3.14744	2.76626	-7.52887	1.6264	-4.93911
74.921	-3.16936	2.76645	-7.55793	1.62617	-4.93652
74.95245	-3.19408	2.76664	-7.59435	1.62602	-4.92732
74.98596	-3.22006	2.76687	-7.62479	1.62593	-4.92149
75.01995	-3.24632	2.76713	-7.65438	1.62577	-4.91946
75.0528	-3.27292	2.76742	-7.67396	1.62556	-4.91777
75.08658	-3.29692	2.76782	-7.68802	1.62535	-4.91876
75.11938	-3.31794	2.76833	-7.70861	1.62515	-4.91998
75.15278	-3.34124	2.76885	-7.72945	1.62493	-4.91554
75.18593	-3.36398	2.76921	-7.7537	1.62468	-4.91057
75.21839	-3.38924	2.7695	-7.77974	1.62444	-4.90882
75.25312	-3.41834	2.76973	-7.80586	1.62423	-4.90501
75.28584	-3.44652	2.76991	-7.82269	1.62401	-4.9028
75.31868	-3.47204	2.77011	-7.84735	1.6238	-4.89453
75.35142	-3.49958	2.77033	-7.86039	1.62355	-4.87769
75.3848	-3.52568	2.77059	-7.86716	1.62316	-4.85684
75.41858	-3.5512	2.7711	-7.87406	1.62275	-4.84169
75.45169	-3.57826	2.77183	-7.88284	1.62242	-4.82844
75.48375	-3.60168	2.77247	-7.89639	1.62216	-4.81404

75.51781	-3.6251	2.77293	-7.91238	1.62198	-4.79878
75.55103	-3.65348	2.77336	-7.92673	1.62181	-4.78228
75.58503	-3.68294	2.77387	-7.93476	1.62163	-4.76112
75.61759	-3.71276	2.77443	-7.94677	1.62148	-4.74113
75.65082	-3.74092	2.77479	-7.95432	1.62133	-4.71318
75.68429	-3.76694	2.77507	-7.95388	1.62125	-4.67964
75.71759	-3.7932	2.77529	-7.95185	1.62124	-4.64531
75.75084	-3.82114	2.77547	-7.9578	1.62132	-4.61128
75.78282	-3.85038	2.77575	-7.96578	1.62138	-4.57528
75.81604	-3.88032	2.77634	-7.97134	1.62128	-4.54121
75.85107	-3.91354	2.77736	-7.9726	1.62116	-4.5013
75.88464	-3.9449	2.77858	-7.96518	1.62114	-4.45334
75.91763	-3.97266	2.77932	-7.9585	1.62139	-4.40477
75.95022	-3.9994	2.77968	-7.95516	1.62201	-4.34993
75.98402	-4.02626	2.78004	-7.94888	1.62242	-4.29392
76.01726	-4.05504	2.78038	-7.94233	1.62249	-4.24199
76.05072	-4.08464	2.78066	-7.9391	1.62246	-4.18076
76.0845	-4.11398	2.78089	-7.92693	1.62244	-4.11483
76.11756	-4.14194	2.78106	-7.91706	1.62244	-4.04734
76.15092	-4.17054	2.78127	-7.90916	1.62263	-3.97242
76.1847	-4.20232	2.78152	-7.90421	1.62286	-3.89736
76.2183	-4.23328	2.78181	-7.89164	1.62323	-3.82347
76.25074	-4.26862	2.78213	-7.87913	1.62346	-3.75536
76.28362	-4.30504	2.7824	-7.8603	1.62353	-3.69421
76.3179	-4.34062	2.78265	-7.84201	1.62363	-3.62841
76.35202	-4.37474	2.78293	-7.82327	1.62374	-3.56335
76.3847	-4.40742	2.78322	-7.80595	1.62377	-3.48763
76.41801	-4.43786	2.78349	-7.78572	1.62372	-3.41723
76.45071	-4.46592	2.78376	-7.76533	1.62356	-3.35081
76.48275	-4.49486	2.78403	-7.74268	1.62347	-3.29425

76.51661	-4.52198	2.78429	-7.72193	1.62344	-3.23267
76.55032	-4.55238	2.78452	-7.69613	1.62352	-3.17155
76.5834	-4.58594	2.78468	-7.67653	1.62358	-3.10861
76.61651	-4.62048	2.78479	-7.65052	1.62354	-3.05202
76.64985	-4.65626	2.78503	-7.63364	1.62344	-2.9978
76.6836	-4.69074	2.7861	-7.61479	1.62328	-2.94545
76.71705	-4.72322	2.78852	-7.58498	1.62322	-2.88639
76.75098	-4.75756	2.79041	-7.55271	1.62327	-2.83089
76.78406	-4.79222	2.79102	-7.5164	1.62344	-2.77919
76.8166	-4.82696	2.79117	-7.47665	1.62352	-2.72733
76.84888	-4.86412	2.79133	-7.44327	1.62357	-2.67356
76.88226	-4.9006	2.79149	-7.40818	1.62365	-2.62154
76.91745	-4.93502	2.79165	-7.36802	1.62376	-2.57143
76.95018	-4.96956	2.79188	-7.32696	1.6239	-2.52451
76.98337	-5.00318	2.79212	-7.28534	1.6241	-2.47802
77.01712	-5.03736	2.7924	-7.2435	1.62419	-2.43295
77.04971	-5.07228	2.79269	-7.20252	1.62415	-2.39457
77.08338	-5.1089	2.79293	-7.15956	1.62412	-2.35704
77.11688	-5.14648	2.7932	-7.11078	1.62412	-2.31968
77.1495	-5.18258	2.79353	-7.05908	1.62416	-2.27673
77.18262	-5.21788	2.79385	-7.01141	1.62433	-2.23083
77.21731	-5.25208	2.79415	-6.96744	1.62451	-2.1838
77.24981	-5.2885	2.79461	-6.92279	1.62464	-2.14828
77.28344	-5.32576	2.7955	-6.87792	1.6248	-2.11689
77.31719	-5.36496	2.79751	-6.83374	1.62495	-2.09261
77.34988	-5.40164	2.79905	-6.78422	1.62503	-2.06396
77.3833	-5.43744	2.79946	-6.73302	1.62507	-2.03044
77.41619	-5.47464	2.79961	-6.68483	1.62512	-1.99739
77.45045	-5.5139	2.7998	-6.62973	1.62516	-1.96137
77.48243	-5.55286	2.80003	-6.5795	1.62523	-1.93494

77.51625	-5.58998	2.80028	-6.53172	1.62529	-1.91283
77.55006	-5.62666	2.80056	-6.47625	1.62525	-1.89359
77.58276	-5.6624	2.8008	-6.42459	1.62516	-1.86818
77.6155	-5.6997	2.80104	-6.37583	1.62502	-1.83531
77.6495	-5.7402	2.8014	-6.32329	1.62494	-1.80532
77.68276	-5.78068	2.80181	-6.27138	1.62495	-1.7776
77.71606	-5.8169	2.8021	-6.2123	1.62496	-1.75273
77.74926	-5.8521	2.80237	-6.14384	1.62497	-1.73096
77.7822	-5.88886	2.80258	-6.08008	1.62498	-1.70106
77.81618	-5.9274	2.80283	-6.01599	1.62494	-1.67245
77.84889	-5.9672	2.80313	-5.95077	1.62491	-1.65171
77.8825	-6.0092	2.80343	-5.8834	1.62494	-1.62969
77.91574	-6.04692	2.8037	-5.81588	1.62505	-1.60979
77.94884	-6.08288	2.80385	-5.74579	1.6252	-1.58182
77.98162	-6.12078	2.80396	-5.6733	1.62535	-1.55152
78.01593	-6.1573	2.80413	-5.59858	1.62542	-1.52052
78.04961	-6.19632	2.80438	-5.52589	1.62544	-1.49415
78.08272	-6.23798	2.80478	-5.45735	1.6255	-1.47102
78.11563	-6.27818	2.80574	-5.39598	1.6256	-1.45267
78.14952	-6.319	2.80647	-5.3273	1.62568	-1.42879
78.18216	-6.36106	2.80677	-5.26046	1.6257	-1.40803
78.21603	-6.40046	2.80702	-5.20215	1.62575	-1.38556
78.24918	-6.43936	2.80726	-5.14275	1.62579	-1.35831
78.28235	-6.4788	2.80756	-5.0884	1.62584	-1.33045
78.31562	-6.51986	2.80789	-5.03068	1.62593	-1.29707
78.34876	-6.5581	2.80818	-4.96923	1.62599	-1.26399
78.38204	-6.5975	2.80838	-4.91533	1.626-1.23452	
78.4156	-6.63458	2.80849	-4.86656	1.62602	-1.20842
78.44916	-6.67262	2.80863	-4.82224	1.62598	-1.18719
78.48232	-6.71232	2.80886	-4.77618	1.62594	-1.16926

78.51619	-6.75422	2.80928	-4.72602	1.62593	-1.14788
78.54982	-6.79432	2.81044	-4.67577	1.62595	-1.12278
78.5825	-6.83596	2.81085	-4.63098	1.62616	-1.1006
78.61549	-6.87668	2.81109	-4.59027	1.62664	-1.08138
78.64874	-6.9193	2.81135	-4.55616	1.62675	-1.06127
78.68172	-6.9583	2.81162	-4.51678	1.62677	-1.03984
78.71578	-6.99834	2.81231	-4.4739	1.62674	-1.01737
78.74798	-7.0386	2.81287	-4.43001	1.62673	-0.9995
78.78112	-7.07946	2.8131	-4.39151	1.62672	-0.98223
78.8146	-7.12154	2.81334	-4.34522	1.6267	-0.97579
78.84815	-7.16118	2.81361	-4.30229	1.62664	-0.967
78.88122	-7.20022	2.81403	-4.25816	1.6266	-0.95748
78.91614	-7.2406	2.8145	-4.21954	1.62659	-0.93988
78.94844	-7.28256	2.81468	-4.18352	1.62662	-0.91799
78.98157	-7.32388	2.81471	-4.1449	1.62664	-0.89803
79.01478	-7.36688	2.81477	-4.10648	1.62663	-0.88474
79.04824	-7.40664	2.81492	-4.0681	1.62657	-0.87608
79.081	-7.44546	2.81536	-4.03042	1.62657	-0.86401
79.11482	-7.4857	2.81635	-3.9941	1.62661	-0.85146
79.14789	-7.52854	2.81685	-3.95152	1.62668	-0.84472
79.18168	-7.572	2.81711	-3.90342	1.62676	-0.83897
79.2151	-7.6139	2.8173	-3.86054	1.62682	-0.83384
79.24884	-7.6525	2.81745	-3.82221	1.62691	-0.82168
79.28125	-7.6901	2.81758	-3.78342	1.62743	-0.81068
79.31468	-7.73044	2.81775	-3.74622	1.62779	-0.80198
79.34798	-7.77206	2.81797	-3.70509	1.62789	-0.79766
79.38194	-7.81402	2.81818	-3.66834	1.62795	-0.78465
79.41582	-7.85548	2.81832	-3.6355	1.62791	-0.76963
79.44806	-7.89852	2.81847	-3.60456	1.62792	-0.75785
79.4818	-7.94006	2.81869	-3.56983	1.62797	-0.75119

79.51534	-7.97974	2.81896	-3.53667	1.62807	-0.74996
79.54884	-8.01956	2.81921	-3.50085	1.62815	-0.75018
79.58208	-8.06074	2.81941	-3.46169	1.62821	-0.74299
79.61426	-8.10428	2.81958	-3.43191	1.62822	-0.73546
79.64843	-8.14688	2.81979	-3.40436	1.62817	-0.728
79.68025	-8.1879	2.82035	-3.37445	1.62812	-0.72549
79.71444	-8.22684	2.82103	-3.34758	1.62815	-0.72382
79.74781	-8.26786	2.82129	-3.31697	1.62817	-0.72688
79.78092	-8.30938	2.82155	-3.28052	1.6282	-0.7242
79.81455	-8.35056	2.82179	-3.24933	1.62822	-0.71404
79.84801	-8.39202	2.82202	-3.22271	1.62822	-0.70079
79.88182	-8.43168	2.82225	-3.1971	1.62821	-0.68855
79.91434	-8.47154	2.8225	-3.17608	1.62816	-0.68191
79.94729	-8.51172	2.82276	-3.15541	1.62808	-0.67944
79.98056	-8.55048	2.823-3.13646	1.6281	-0.67426	
80.01425	-8.59084	2.82324	-3.1151	1.62831	-0.66985
80.04674	-8.63054	2.82348	-3.08811	1.62854	-0.66905
80.08106	-8.67014	2.82369	-3.05873	1.62865	-0.66446
80.11455	-8.7078	2.82387	-3.03179	1.62866	-0.66382
80.1473	-8.7456	2.824-3.0056	1.62861	-0.66455	
80.18079	-8.78566	2.82415	-2.98803	1.62859	-0.66431
80.21451	-8.825	2.82443	-2.97179	1.6286	-0.66153
80.2474	-8.86364	2.82476	-2.9583	1.6286	-0.65467
80.28156	-8.90376	2.82503	-2.94778	1.62862	-0.64451
80.31329	-8.94252	2.82525	-2.92521	1.62865	-0.63829
80.34632	-8.98276	2.82543	-2.89759	1.62865	-0.63786
80.37947	-9.02294	2.82565	-2.87022	1.62869	-0.6395
80.41382	-9.06144	2.82601	-2.84451	1.62874	-0.64035
80.4469	-9.10008	2.82626	-2.82852	1.6288	-0.64071
80.48026	-9.13946	2.82647	-2.81385	1.62886	-0.63949

80.51478	-9.17986	2.82671	-2.795	1.62889	-0.63599
80.54676	-9.21898	2.82716	-2.77648	1.62893	-0.63222
80.5809	-9.25688	2.8285	-2.76308	1.6291	-0.62714
80.61376	-9.29742	2.83027	-2.74783	1.62981	-0.62166
80.64694	-9.3377	2.83081	-2.73151	1.63026	-0.61914
80.68062	-9.37696	2.83107	-2.71136	1.63039	-0.61628
80.71359	-9.41632	2.83125	-2.68884	1.63041	-0.61512
80.74731	-9.4564	2.83139	-2.67618	1.63037	-0.61452
80.78045	-9.49632	2.83156	-2.6658	1.63042	-0.61437
80.81328	-9.53654	2.83171	-2.64853	1.63051	-0.61437
80.84688	-9.57594	2.83187	-2.63335	1.63056	-0.6142
80.8797	-9.61316	2.83201	-2.61165	1.63054	-0.61251
80.91378	-9.65252	2.83221	-2.59792	1.63043	-0.6102
80.9474	-9.69318	2.83241	-2.5932	1.63038	-0.60807
80.9799	-9.73358	2.83262	-2.58096	1.63044	-0.60579
81.01362	-9.77502	2.83323	-2.56947	1.63053	-0.60409
81.04664	-9.8154	2.83383	-2.55366	1.63058	-0.6032
81.07949	-9.85646	2.83443	-2.53179	1.63059	-0.60228
81.11342	-9.89798	2.83489	-2.51729	1.63053	-0.60155
81.14668	-9.94114	2.83511	-2.50686	1.63051	-0.60035
81.18034	-9.98204	2.8353	-2.4899	1.63052	-0.59893
81.21351	-10.02324	2.83546	-2.47707	1.6305	-0.59822
81.24616	-10.06308	2.83568	-2.45741	1.63048	-0.59784
81.279	-10.10322	2.83611	-2.44088	1.63044	-0.59767
81.3131	-10.14682	2.8367	-2.42982	1.63043	-0.59718
81.34644	-10.18848	2.83706	-2.4201	1.63056	-0.59614
81.38006	-10.23092	2.83729	-2.41576	1.63065	-0.59508
81.41345	-10.2743	2.83754	-2.41256	1.63073	-0.59464
81.44655	-10.3153	2.83784	-2.40352	1.63078	-0.59438
81.48007	-10.356	2.83873	-2.3917	1.63075	-0.59444

81.51284	-10.39778	2.83931	-2.37684	1.63069	-0.59422
81.54666	-10.44228	2.83962	-2.36005	1.63072	-0.59355
81.57931	-10.48884	2.83991	-2.34851	1.6308	-0.59169
81.61287	-10.53332	2.84015	-2.33872	1.63096	-0.59028
81.64636	-10.5774	2.84033	-2.3284	1.63137	-0.58936
81.6798	-10.62062	2.8405	-2.316	1.63158	-0.58862
81.71234	-10.66002	2.84065	-2.30963	1.63166	-0.58833
81.74598	-10.70204	2.84088	-2.29894	1.63162	-0.58756
81.77972	-10.74184	2.84137	-2.29518	1.63117	-0.58612
81.81296	-10.77914	2.84203	-2.28607	1.63074	-0.58557
81.84584	-10.82128	2.84244	-2.2743	1.63066	-0.58502
81.87976	-10.86276	2.84266	-2.26427	1.63072	-0.58447
81.91206	-10.90338	2.84277	-2.25533	1.63092	-0.58427
81.94508	-10.94668	2.84286	-2.24303	1.63102	-0.58398
81.97922	-10.9885	2.84294	-2.23583	1.63107	-0.5838
82.01264	-11.03058	2.843-2.23248	1.63101	-0.58346	
82.04601	-11.07574	2.84309	-2.22624	1.63096	-0.58251
82.07993	-11.11828	2.84318	-2.21871	1.63093	-0.58134
82.11218	-11.16024	2.8433	-2.2096	1.63087	-0.58083
82.14545	-11.20394	2.84345	-2.19526	1.63081	-0.58056
82.1789	-11.24764	2.84363	-2.183	1.63081	-0.58036
82.2124	-11.29404	2.84379	-2.17869	1.6308	-0.58001
82.24618	-11.3424	2.84402	-2.17509	1.63088	-0.57982
82.27961	-11.3908	2.84427	-2.17324	1.63091	-0.57966
82.3121	-11.44024	2.84446	-2.16711	1.63091	-0.57958
82.34549	-11.49016	2.84458	-2.15759	1.63094	-0.5795
82.3796	-11.54088	2.84471	-2.13998	1.63104	-0.57936
82.41272	-11.59666	2.8449	-2.13055	1.63119	-0.57921
82.44522	-11.6552	2.84514	-2.12753	1.63126	-0.57871
82.47836	-11.71396	2.8454	-2.12526	1.63122	-0.57828

82.51215	-11.77262	2.84571	-2.11806	1.63108	-0.5781
82.54582	-11.83586	2.84633	-2.11416	1.63101	-0.57803
82.57868	-11.89996	2.84672	-2.10555	1.631	-0.57794
82.61152	-11.96558	2.847	-2.09783	1.63106	-0.57784
82.64442	-12.03262	2.84721	-2.09373	1.63114	-0.5777
82.67811	-12.09648	2.84743	-2.09014	1.63112	-0.57749
82.71184	-12.15462	2.84778	-2.08478	1.63105	-0.57731
82.74518	-12.21134	2.84877	-2.0827	1.63101	-0.57719
82.77851	-12.2602	2.84943	-2.07832	1.63095	-0.57708
82.81238	-12.30788	2.84983	-2.06966	1.63096	-0.57697
82.84532	-12.35526	2.85015	-2.06139	1.63098	-0.57688
82.87801	-12.40148	2.85041	-2.05069	1.63092	-0.57687
82.91268	-12.4446	2.85065	-2.04387	1.63084	-0.57686
82.94512	-12.48248	2.85085	-2.04229	1.63076	-0.57683
82.97819	-12.51664	2.851	-2.03987	1.63072	-0.5768
83.01132	-12.55128	2.85121	-2.03758	1.63074	-0.57674
83.04451	-12.58374	2.8514	-2.03423	1.63076	-0.57664
83.0777	-12.61738	2.85154	-2.02936	1.63078	-0.57654
83.11212	-12.648	2.85165	-2.02245	1.63075	-0.57642
83.14438	-12.67784	2.85174	-2.01721	1.63068	-0.57626
83.17863	-12.70832	2.85189	-2.0118	1.63069	-0.57608
83.21134	-12.7369	2.85209	-2.00336	1.63074	-0.57597
83.245	-12.76366	2.85229	-1.99944	1.6308	-0.57587
83.27811	-12.78696	2.85247	-1.99747	1.63086	-0.5758
83.31157	-12.81106	2.85264	-1.99595	1.63083	-0.57571
83.34445	-12.83426	2.8528	-1.99618	1.63073	-0.57563
83.3776	-12.85746	2.853	-1.9937	1.6306	-0.57559
83.41124	-12.88066	2.85317	-1.9866	1.63049	-0.57554
83.44351	-12.90674	2.85338	-1.97851	1.63045	-0.57552
83.47722	-12.93438	2.85367	-1.96977	1.63051	-0.57543

83.51061	-12.963	2.85451	-1.96612	1.63063	-0.57533
83.54394	-12.99248	2.85687	-1.96522	1.63071	-0.57529
83.57736	-13.0203	2.85928	-1.96391	1.6307	-0.57528
83.61118	-13.04762	2.85991	-1.96191	1.6306	-0.57527
83.64426	-13.0794	2.86004	-1.95908	1.63049	-0.57526
83.67649	-13.11152	2.86005	-1.95508	1.63043	-0.57516
83.71069	-13.14666	2.86008	-1.94993	1.63037	-0.57508
83.74441	-13.18258	2.86018	-1.94715	1.63032	-0.57511
83.7775	-13.2186	2.8604	-1.94402	1.63023	-0.57522
83.81005	-13.25712	2.86127	-1.93967	1.6301	-0.57531
83.84444	-13.29524	2.86287	-1.93791	1.63002	-0.57534
83.87694	-13.33548	2.86453	-1.93601	1.62999	-0.57529
83.91078	-13.38006	2.86527	-1.93372	1.63	-0.57509
83.944	-13.42932	2.86545	-1.9293	1.63005	-0.57502
83.97609	-13.482	2.86552	-1.92367	1.63009	-0.57502
84.01012	-13.53358	2.86565	-1.91798	1.63008	-0.57514
84.0436	-13.58046	2.8658	-1.91567	1.63002	-0.57548
84.07653	-13.61892	2.86597	-1.91393	1.62996	-0.5757
84.11081	-13.65222	2.86614	-1.91297	1.62987	-0.57575
84.14309	-13.6836	2.8663	-1.91194	1.62986	-0.57576
84.1768	-13.7141	2.86646	-1.9107	1.62986	-0.57573
84.20984	-13.73788	2.86669	-1.90903	1.62979	-0.57577
84.24238	-13.75868	2.86695	-1.90744	1.62972	-0.57581
84.27587	-13.77594	2.86718	-1.90476	1.62962	-0.57589
84.30968	-13.78956	2.8674	-1.90236	1.62959	-0.57598
84.3421	-13.80466	2.86765	-1.90071	1.62957	-0.57606
84.3766	-13.81792	2.86782	-1.89673	1.62952	-0.57616
84.40958	-13.8287	2.86795	-1.89369	1.62945	-0.57624
84.44238	-13.83834	2.86807	-1.89087	1.62934	-0.5763
84.47626	-13.8518	2.86814	-1.88788	1.62922	-0.5764

84.50922	-13.86592	2.86821	-1.88539	1.62913	-0.57652
84.54364	-13.8785	2.86839	-1.88285	1.62902	-0.5769
84.57625	-13.89232	2.86865	-1.88112	1.62896	-0.57726
84.60949	-13.9053	2.86894	-1.88	1.62896	-0.57735
84.64236	-13.91774	2.86926	-1.87818	1.62899	-0.57737
84.67538	-13.93168	2.8695	-1.87644	1.62901	-0.57737
84.70874	-13.94842	2.86965	-1.87377	1.62894	-0.57739
84.74214	-13.96578	2.86979	-1.87184	1.62879	-0.57756
84.7765	-13.98196	2.86994	-1.87063	1.62863	-0.57785
84.80924	-13.99566	2.87011	-1.8698	1.62851	-0.57824
84.84275	-14.00582	2.87034	-1.86916	1.62845	-0.5786
84.87609	-14.01514	2.8707	-1.8683	1.62841	-0.57889
84.90889	-14.0208	2.87101	-1.86703	1.62833	-0.5791
84.94204	-14.02674	2.87151	-1.86492	1.62816	-0.5793
84.97595	-14.0312	2.87175	-1.86276	1.6279	-0.57938
85.00931	-14.0332	2.87186	-1.86175	1.62767	-0.57943
85.04172	-14.03816	2.87193	-1.86125	1.62752	-0.57948
85.07516	-14.03992	2.872	-1.86097	1.62742	-0.57954
85.10841	-14.04228	2.87211	-1.86026	1.62733	-0.57987
85.14228	-14.04668	2.87229	-1.85943	1.62722	-0.58078
85.17492	-14.0454	2.87246	-1.85846	1.62708	-0.58153
85.20944	-14.04	2.87256	-1.85627	1.62698	-0.58188
85.24238	-14.03112	2.87267	-1.85108	1.62693	-0.58217
85.27531	-14.01656	2.8728	-1.84666	1.62684	-0.5823
85.30789	-14.00352	2.87296	-1.84375	1.62672	-0.58244
85.34165	-13.99266	2.87334	-1.84223	1.62657	-0.58264
85.37446	-13.9795	2.87378	-1.84174	1.62632	-0.58283
85.40797	-13.9637	2.87401	-1.84143	1.62608	-0.58322
85.44132	-13.94454	2.87423	-1.84116	1.62594	-0.58359
85.47402	-13.9261	2.87448	-1.84095	1.62585	-0.58385

85.50762	-13.9074	2.87468	-1.84076	1.62585	-0.58406
85.54088	-13.88902	2.87488	-1.84028	1.62594	-0.58425
85.57374	-13.86796	2.87504	-1.83954	1.62594	-0.58443
85.60801	-13.84706	2.87517	-1.83878	1.62586	-0.58466
85.64161	-13.82412	2.87529	-1.83784	1.6253	-0.58492
85.67397	-13.80042	2.87544	-1.83659	1.62465	-0.5852
85.70791	-13.77892	2.87564	-1.83609	1.62436	-0.58543
85.74126	-13.75658	2.87589	-1.83544	1.62418	-0.58564
85.77392	-13.73574	2.87617	-1.83501	1.624	-0.58587
85.80725	-13.7198	2.87646	-1.83461	1.62366	-0.58612
85.83988	-13.7087	2.8767	-1.8337	1.62322	-0.5864
85.87401	-13.69948	2.87688	-1.83221	1.62304	-0.5868
85.90793	-13.68584	2.87706	-1.83089	1.62302	-0.58728
85.93964	-13.66318	2.87724	-1.8298	1.62309	-0.58808
85.97387	-13.62798	2.87747	-1.82924	1.6232	-0.58887
86.00622	-13.58526	2.87773	-1.82865	1.62314	-0.58916
86.03984	-13.53746	2.87796	-1.82849	1.62284	-0.5894
86.07345	-13.48582	2.8781	-1.82808	1.62216	-0.58964
86.10708	-13.4303	2.87815	-1.82777	1.62156	-0.58991
86.14044	-13.37384	2.87813	-1.82756	1.6213	-0.59023
86.17449	-13.31384	2.87817	-1.82729	1.62112	-0.59052
86.20762	-13.2509	2.87825	-1.82703	1.62091	-0.59082
86.24004	-13.18612	2.87832	-1.82686	1.62071	-0.59116
86.27341	-13.1145	2.87836	-1.82667	1.6205	-0.59194
86.30662	-13.0435	2.87842	-1.82647	1.6204	-0.59308
86.33968	-12.9706	2.87856	-1.82625	1.62038	-0.59356
86.37211	-12.89244	2.87884	-1.82602	1.62026	-0.59378
86.40547	-12.81178	2.87939	-1.82579	1.62008	-0.594
86.43997	-12.73044	2.87979	-1.8256	1.61987	-0.59423
86.47391	-12.6467	2.88001	-1.82541	1.61963	-0.59452

86.50675	-12.56418	2.8802	-1.82512	1.61943	-0.5953
86.54012	-12.47514	2.88042	-1.82488	1.61922	-0.59639
86.57344	-12.3831	2.88061	-1.82464	1.61885	-0.5971
86.60638	-12.2916	2.88083	-1.82439	1.61848	-0.59803
86.63892	-12.20046	2.88105	-1.82417	1.61824	-0.59934
86.67319	-12.10966	2.88121	-1.82389	1.61807	-0.6001
86.70626	-12.01446	2.88134	-1.82333	1.61793	-0.6005
86.73966	-11.91654	2.88145	-1.82283	1.61783	-0.60076
86.77275	-11.81834	2.88158	-1.82254	1.61775	-0.60089
86.80574	-11.71966	2.88178	-1.82231	1.6176	-0.60104
86.83884	-11.62028	2.88195	-1.82212	1.6174	-0.60127
86.87211	-11.51788	2.88212	-1.82193	1.61684	-0.60197
86.90606	-11.40964	2.88225	-1.82171	1.616	-0.6046
86.93781	-11.30358	2.88234	-1.82144	1.61525	-0.60732
86.97156	-11.195	2.88241	-1.82115	1.61478	-0.61038
87.00445	-11.08482	2.88246	-1.82093	1.61451	-0.61154
87.03816	-10.97732	2.88246	-1.82071	1.61422	-0.61182
87.07205	-10.86818	2.88253	-1.82049	1.61392	-0.61196
87.10582	-10.75614	2.88259	-1.82019	1.61365	-0.61207
87.13832	-10.63866	2.88276	-1.81961	1.61342	-0.61227
87.17264	-10.52012	2.88296	-1.81855	1.61321	-0.61265
87.20531	-10.40096	2.88323	-1.81788	1.613	-0.61343
87.23831	-10.28366	2.88458	-1.81762	1.61277	-0.61404
87.27188	-10.1653	2.88707	-1.81752	1.61254	-0.61436
87.30484	-10.04408	2.88871	-1.81763	1.61234	-0.61464
87.3382	-9.92028	2.88941	-1.8176	1.61213	-0.61532
87.37074	-9.79608	2.88943	-1.81716	1.61184	-0.61684
87.40454	-9.67226	2.88936	-1.81602	1.61155	-0.61833
87.43843	-9.54734	2.88944	-1.81555	1.61139	-0.61888
87.4707	-9.41964	2.88958	-1.81547	1.61131	-0.61913

87.50458	-9.29252	2.88982	-1.81559	1.61123	-0.61931
87.53809	-9.16174	2.88999	-1.81565	1.611	-0.61953
87.57075	-9.03128	2.89009	-1.8155	1.60924	-0.61983
87.6045	-8.90184	2.89021	-1.81527	1.60737	-0.6207
87.63791	-8.76776	2.8904	-1.81503	1.6068	-0.62195
87.67113	-8.63252	2.89058	-1.81484	1.60654	-0.62237
87.70441	-8.49726	2.89076	-1.81462	1.60628	-0.62266
87.73751	-8.35998	2.89099	-1.8144	1.60594	-0.62303
87.77042	-8.22434	2.8912	-1.8142	1.60558	-0.62368
87.80372	-8.09092	2.89139	-1.81405	1.60532	-0.62418
87.83738	-7.95326	2.89156	-1.81392	1.60513	-0.62449
87.87114	-7.81642	2.89162	-1.81379	1.60492	-0.62465
87.90472	-7.67854	2.89165	-1.81363	1.60471	-0.62476
87.93731	-7.53932	2.89171	-1.81343	1.60448	-0.62498
87.9692	-7.39896	2.89175	-1.81326	1.60426	-0.62587
88.00341	-7.26104	2.89185	-1.81317	1.60402	-0.62796
88.03625	-7.12474	2.89194	-1.81317	1.60371	-0.63044
88.06975	-6.98734	2.89198	-1.81312	1.60339	-0.63128
88.10269	-6.85006	2.89195	-1.81305	1.60302	-0.63193
88.13618	-6.71162	2.89202	-1.81291	1.60278	-0.63283
88.16918	-6.57312	2.89224	-1.81277	1.60254	-0.6334
88.20321	-6.43844	2.8932	-1.81265	1.60228	-0.63382
88.23663	-6.3084	2.89366	-1.81255	1.60198	-0.63409
88.27019	-6.17856	2.89378	-1.81241	1.6017	-0.63435
88.30258	-6.05008	2.89373	-1.8123	1.60147	-0.63461
88.33621	-5.92618	2.89369	-1.81222	1.60121	-0.63493
88.36864	-5.80624	2.89367	-1.81212	1.60052	-0.63525
88.40306	-5.68794	2.89367	-1.812	1.59938	-0.63556
88.43546	-5.57222	2.89377	-1.81187	1.599	-0.6361
88.46866	-5.4587	2.8939	-1.81173	1.59874	-0.63729

88.50294	-5.3473	2.89436	-1.81165	1.59857	-0.63819
88.5358	-5.24126	2.89471	-1.81164	1.59846	-0.63863
88.56962	-5.14038	2.89489	-1.81166	1.59827	-0.63887
88.60245	-5.04252	2.89499	-1.81163	1.59796	-0.63912
88.636	-4.94796	2.89506	-1.81164	1.59706	-0.63958
88.66965	-4.85702	2.8951	-1.81164	1.59534	-0.64043
88.70226	-4.76546	2.89523	-1.81169	1.59449	-0.6416
88.73489	-4.67526	2.89544	-1.81185	1.59427	-0.64249
88.7702	-4.59024	2.89574	-1.81192	1.5941	-0.64298
88.80288	-4.50808	2.896	-1.81201	1.59391	-0.64441
88.83605	-4.42944	2.89615	-1.81206	1.59364	-0.64552
88.8684	-4.35124	2.89625	-1.81197	1.5924	-0.64636
88.90165	-4.2752	2.89632	-1.81166	1.59183	-0.64695
88.93484	-4.2008	2.89647	-1.81137	1.59159	-0.64726
88.96796	-4.12972	2.89671	-1.81126	1.59135	-0.64752
89.00225	-4.06254	2.89718	-1.81118	1.59114	-0.64768
89.03453	-3.99456	2.89791	-1.81113	1.59089	-0.64783
89.06708	-3.92986	2.89826	-1.81112	1.59056	-0.64808
89.10074	-3.86548	2.89847	-1.81111	1.59028	-0.64848
89.13418	-3.80476	2.89875	-1.81118	1.58988	-0.65007
89.16712	-3.74658	2.89901	-1.81128	1.5895	-0.65206
89.20062	-3.6875	2.89935	-1.81139	1.58909	-0.65303
89.2347	-3.62972	2.89961	-1.81158	1.58853	-0.65342
89.26832	-3.57298	2.89981	-1.81167	1.58757	-0.65375
89.30094	-3.51622	2.89996	-1.8117	1.58631	-0.65408
89.33381	-3.46266	2.90008	-1.81173	1.58553	-0.65442
89.36668	-3.4085	2.90021	-1.81175	1.58531	-0.65477
89.40089	-3.35668	2.90039	-1.8118	1.58518	-0.65509
89.43319	-3.30826	2.90052	-1.81185	1.58507	-0.65539
89.46629	-3.26016	2.90065	-1.81189	1.58489	-0.65566

89.49959	-3.21084	2.90078	-1.81196	1.58461	-0.65602
89.53362	-3.16206	2.90095	-1.8121	1.58432	-0.65695
89.56793	-3.1143	2.90115	-1.81226	1.58404	-0.65874
89.60078	-3.07072	2.90137	-1.81239	1.58375	-0.66034
89.63382	-3.02876	2.90154	-1.81249	1.58346	-0.66098
89.66666	-2.98464	2.90165	-1.81251	1.58317	-0.66145
89.70024	-2.93948	2.90174	-1.81257	1.58291	-0.66186
89.7328	-2.89512	2.90182	-1.81286	1.5827	-0.66221
89.76632	-2.85366	2.90194	-1.8134	1.58247	-0.66282
89.79941	-2.8111	2.90214	-1.81433	1.58223	-0.66394
89.83359	-2.77188	2.90234	-1.81495	1.58192	-0.66567
89.86595	-2.73506	2.90252	-1.81509	1.58157	-0.66686
89.90004	-2.69638	2.90259	-1.81522	1.581-0.66858	
89.93286	-2.6557	2.90247	-1.81538	1.58048	-0.6701
89.96694	-2.61556	2.9025	-1.81551	1.57974	-0.67134
90.00028	-2.57572	2.90266	-1.81565	1.57901	-0.67196
90.03312	-2.5379	2.90286	-1.81576	1.57849	-0.67243
90.06566	-2.50166	2.90308	-1.8158	1.57782	-0.67295
90.09901	-2.46802	2.90324	-1.81586	1.57671	-0.67333
90.13228	-2.43506	2.9034	-1.81605	1.5761	-0.67368
90.16576	-2.40482	2.90394	-1.81641	1.57579	-0.67442
90.19965	-2.3734	2.90518	-1.81682	1.5755	-0.67578
90.23272	-2.33748	2.90577	-1.81704	1.57522	-0.67948
90.26563	-2.29806	2.9062	-1.81715	1.5747	-0.68162
90.29876	-2.2594	2.90642	-1.8173	1.57381	-0.68215
90.33312	-2.22438	2.90665	-1.81765	1.57347	-0.68262
90.36622	-2.19696	2.9069	-1.8181	1.57314	-0.68297
90.39949	-2.17392	2.90698	-1.81839	1.57269	-0.68374
90.43199	-2.1523	2.90705	-1.81863	1.57193	-0.68445
90.46598	-2.1258	2.90712	-1.81886	1.57078	-0.68485

90.49746	-2.09196	2.90716	-1.81908	1.57037	-0.6851
90.53172	-2.05422	2.90732	-1.81936	1.57008	-0.68535
90.56531	-2.0219	2.90756	-1.81979	1.56976	-0.68578
90.59863	-1.99552	2.90786	-1.82006	1.56943	-0.68686
90.63235	-1.97138	2.90847	-1.82053	1.56861	-0.6882
90.66412	-1.9488	2.90884	-1.82077	1.5682	-0.68993
90.69738	-1.92096	2.90896	-1.82097	1.56789	-0.69093
90.73093	-1.89202	2.90903	-1.82145	1.5676	-0.69226
90.76488	-1.86536	2.90914	-1.82256	1.56731	-0.69297
90.79843	-1.84016	2.90933	-1.82352	1.56677	-0.69348
90.83071	-1.81618	2.90957	-1.82397	1.56537	-0.6938
90.86444	-1.79466	2.90978	-1.8242	1.5637	-0.69411
90.89781	-1.77154	2.90997	-1.8244	1.56283	-0.69445
90.93059	-1.74868	2.91017	-1.82466	1.56236	-0.69543
90.96418	-1.72458	2.91034	-1.82497	1.56203	-0.6969
90.99749	-1.69978	2.91052	-1.82534	1.5617	-0.69811
91.03108	-1.67718	2.91076	-1.82593	1.56133	-0.69864
91.06441	-1.65276	2.91094	-1.8262	1.56096	-0.69901
91.09726	-1.63122	2.91106	-1.8264	1.56059	-0.69932
91.13097	-1.61056	2.91117	-1.82662	1.56021	-0.69966
91.16428	-1.5893	2.91126	-1.82689	1.55976	-0.70002
91.19715	-1.56754	2.91142	-1.82729	1.55863	-0.70074
91.22999	-1.5466	2.91171	-1.82774	1.55808	-0.70246
91.26356	-1.52522	2.91202	-1.82807	1.5577	-0.70564
91.29744	-1.50612	2.91226	-1.8284	1.5574	-0.70836
91.33156	-1.48946	2.91246	-1.82896	1.5571	-0.70945
91.36388	-1.47336	2.91266	-1.82957	1.55681	-0.70995
91.39738	-1.45582	2.91279	-1.83044	1.55645	-0.71041
91.43019	-1.43608	2.91286	-1.83111	1.55605	-0.71073
91.46303	-1.41504	2.91287	-1.83158	1.55565	-0.71107

91.49656	-1.39664	2.9128	-1.83208	1.55503	-0.7114
91.5298	-1.37926	2.91276	-1.83272	1.55454	-0.7118
91.56288	-1.36242	2.91274	-1.83309	1.55414	-0.71266
91.59652	-1.34486	2.91278	-1.83348	1.55348	-0.71347
91.62981	-1.32584	2.91293	-1.83386	1.55306	-0.71413
91.66311	-1.3093	2.91324	-1.83421	1.55273	-0.71503
91.696	-1.29428	2.91351	-1.83457	1.55244	-0.71671
91.7282	-1.27772	2.91399	-1.83492	1.55212	-0.71904
91.76186	-1.26182	2.91426	-1.83525	1.5514	-0.72069
91.79624	-1.24402	2.91444	-1.83555	1.54871	-0.7214
91.82982	-1.22946	2.91456	-1.83589	1.54572	-0.72167
91.86274	-1.21434	2.91469	-1.83633	1.54372	-0.72189
91.89543	-1.20066	2.91481	-1.8372	1.54283	-0.72214
91.92936	-1.18586	2.91502	-1.83818	1.54236	-0.72248
91.96161	-1.17352	2.91548	-1.83888	1.54199	-0.72305
91.99594	-1.15918	2.91577	-1.83965	1.54145	-0.72407
92.02905	-1.14692	2.91595	-1.84054	1.541-0.72692	
92.06212	-1.1327	2.91603	-1.84123	1.54063	-0.72958
92.09546	-1.11794	2.91605	-1.84162	1.54035	-0.73118
92.12964	-1.10374	2.9161	-1.84191	1.54011	-0.73195
92.16205	-1.08936	2.91621	-1.84225	1.53983	-0.7325
92.19551	-1.07782	2.91638	-1.84289	1.53921	-0.73345
92.22847	-1.0638	2.91656	-1.84565	1.5378	-0.735
92.2615	-1.05082	2.91673	-1.84864	1.53626	-0.73644
92.29449	-1.0402	2.91693	-1.85005	1.53525	-0.7373
92.32852	-1.02986	2.91715	-1.85043	1.53458	-0.738
92.36155	-1.02018	2.91733	-1.85074	1.53415	-0.7385
92.39491	-1.0076	2.91746	-1.85098	1.53361	-0.73887
92.42869	-0.99312	2.91753	-1.85129	1.53321	-0.73925
92.4617	-0.98212	2.91758	-1.85164	1.5328	-0.73994

92.49452	-0.97276	2.91776	-1.85249	1.53244	-0.74109
92.52778	-0.96498	2.91812	-1.85467	1.53209	-0.74249
92.56011	-0.9556	2.91877	-1.85656	1.53167	-0.74371
92.59441	-0.9462	2.91901	-1.85708	1.53114	-0.74418
92.62728	-0.9343	2.91918	-1.85744	1.53059	-0.74487
92.66106	-0.92166	2.91926	-1.85775	1.53002	-0.74603
92.69388	-0.91084	2.91934	-1.85853	1.52953	-0.74754
92.72722	-0.89778	2.9195	-1.85934	1.52808	-0.74831
92.76026	-0.88664	2.91968	-1.85997	1.52584	-0.74875
92.7937	-0.87826	2.9201	-1.86079	1.52339	-0.74901
92.82716	-0.87278	2.92064	-1.86116	1.52236	-0.75008
92.86006	-0.86502	2.9208	-1.86159	1.52187	-0.75245
92.89382	-0.85746	2.92089	-1.862	1.5216	-0.75583
92.92695	-0.84706	2.92099	-1.86241	1.52137	-0.75822
92.96015	-0.83594	2.92106	-1.86295	1.52112	-0.75917
92.99328	-0.82654	2.92115	-1.86393	1.52083	-0.75973
93.02634	-0.81806	2.92127	-1.86508	1.52025	-0.76009
93.06018	-0.8093	2.92131	-1.8656	1.51819	-0.76045
93.09295	-0.80296	2.92132	-1.86643	1.51492	-0.76093
93.12628	-0.79436	2.92136	-1.86771	1.513	-0.76154
93.15959	-0.78546	2.92145	-1.86834	1.51256	-0.76229
93.19238	-0.77846	2.92154	-1.86898	1.51232	-0.7641
93.22661	-0.77216	2.92167	-1.86949	1.51215	-0.76657
93.25978	-0.7674	2.92174	-1.86994	1.51192	-0.76913
93.29256	-0.75864	2.92176	-1.87098	1.51145	-0.77078
93.32599	-0.75006	2.92182	-1.87234	1.51045	-0.7712
93.35929	-0.74074	2.9219	-1.873	1.50917	-0.77148
93.39238	-0.73122	2.92204	-1.8734	1.50784	-0.77166
93.42547	-0.72628	2.9224	-1.87375	1.50736	-0.77188
93.45899	-0.7205	2.923	-1.87418	1.507	-0.77224

93.49244	-0.71438	2.92319	-1.8767	1.50666	-0.77341
93.52558	-0.7097	2.92331	-1.88233	1.5063	-0.7762
93.5588	-0.70254	2.9234	-1.88684	1.50596	-0.78032
93.59118	-0.69664	2.92352	-1.88862	1.5056	-0.78394
93.62599	-0.69062	2.92379	-1.88946	1.50521	-0.78607
93.65851	-0.68526	2.9241	-1.88981	1.50398	-0.78765
93.69201	-0.67922	2.92427	-1.89009	1.50262	-0.78946
93.72528	-0.67184	2.9244	-1.89029	1.50138	-0.79064
93.75918	-0.66372	2.92448	-1.89049	1.5004	-0.79129
93.79164	-0.6583	2.9246	-1.89084	1.49964	-0.79178
93.82445	-0.6531	2.92475	-1.89343	1.4989	-0.7922
93.85743	-0.6469	2.9249	-1.89574	1.49784	-0.79248
93.89063	-0.6426	2.92504	-1.89656	1.49625	-0.79306
93.92352	-0.63772	2.92514	-1.89686	1.49545	-0.79371
93.95708	-0.63048	2.92519	-1.89711	1.49481	-0.79482
93.99086	-0.62644	2.92527	-1.89844	1.49413	-0.79663
94.02432	-0.62022	2.9254	-1.90372	1.49355	-0.79855
94.05781	-0.61602	2.92554	-1.90799	1.49298	-0.80013
94.0909	-0.61168	2.9257	-1.90959	1.49223	-0.80077
94.12406	-0.60686	2.92589	-1.90989	1.49175	-0.80116
94.15742	-0.60174	2.92606	-1.90985	1.49136	-0.80153
94.19132	-0.59786	2.92621	-1.90993	1.49089	-0.80268
94.22379	-0.59352	2.92626	-1.91041	1.49016	-0.80471
94.25641	-0.58864	2.92626	-1.9118	1.48975	-0.80802
94.29044	-0.5823	2.92628	-1.91351	1.48926	-0.81106
94.3222	-0.57484	2.92632	-1.91484	1.48867	-0.81408
94.35644	-0.57236	2.9264	-1.91523	1.4874	-0.81513
94.3909	-0.5696	2.92651	-1.91552	1.48526	-0.81591
94.42336	-0.56658	2.92664	-1.91587	1.48327	-0.81641
94.45714	-0.56338	2.92686	-1.9161	1.48288	-0.81668

94.49028	-0.5569	2.92708	-1.91679	1.48257	-0.81716
94.52262	-0.54914	2.92726	-1.91851	1.48229	-0.81803
94.55561	-0.54532	2.92745	-1.92062	1.48194	-0.81898
94.58925	-0.54034	2.92762	-1.9214	1.48112	-0.82074
94.62266	-0.53934	2.92772	-1.92179	1.4787	-0.82327
94.65576	-0.53762	2.92775	-1.92199	1.47699	-0.82495
94.68881	-0.5345	2.92766	-1.92213	1.47585	-0.82586
94.72249	-0.53064	2.92764	-1.92232	1.47429	-0.82642
94.75574	-0.52614	2.92792	-1.92255	1.47266	-0.82709
94.78832	-0.52128	2.92847	-1.92346	1.47068	-0.82751
94.8222	-0.51672	2.92879	-1.92577	1.468	-0.82825
94.8558	-0.51242	2.92897	-1.92795	1.46702	-0.82996
94.88984	-0.50782	2.92902	-1.93099	1.46659	-0.83233
94.92218	-0.506	2.92903	-1.93222	1.46589	-0.83428
94.95509	-0.50518	2.92909	-1.93281	1.46548	-0.83518
94.98836	-0.5038	2.92914	-1.93438	1.46514	-0.83589
95.02149	-0.5022	2.92921	-1.93735	1.46476	-0.83628
95.055	-0.4961	2.92933	-1.93953	1.46437	-0.8368
95.08878	-0.4902	2.92943	-1.9409	1.46344	-0.83792
95.12247	-0.48658	2.92956	-1.94127	1.46153	-0.83902
95.15539	-0.48434	2.92966	-1.94159	1.46005	-0.84017
95.18819	-0.48342	2.9297	-1.94231	1.45919	-0.84187
95.22089	-0.4829	2.9298	-1.94301	1.45817	-0.84528
95.25436	-0.48022	2.92988	-1.94358	1.45738	-0.85109
95.28742	-0.47848	2.92995	-1.944	1.456	-0.85451
95.32043	-0.47702	2.92998	-1.94451	1.45146	-0.85638
95.35455	-0.47448	2.92996	-1.94501	1.44667	-0.85737
95.38812	-0.47058	2.92992	-1.94616	1.44327	-0.85786
95.4217	-0.46644	2.92987	-1.94736	1.44127	-0.85832
95.45431	-0.46212	2.92987	-1.94889	1.44051	-0.85888

95.48784	-0.45936	2.92991	-1.94958	1.43976	-0.85951
95.5204	-0.45646	2.92996	-1.95004	1.4389	-0.8607
95.55392	-0.45374	2.93012	-1.95041	1.43835	-0.86226
95.58761	-0.45284	2.93024	-1.95084	1.43786	-0.86362
95.61981	-0.45204	2.93033	-1.95149	1.43758	-0.86534
95.65348	-0.44968	2.93044	-1.95211	1.43722	-0.86721
95.68659	-0.44592	2.9307	-1.95464	1.43601	-0.86925
95.71978	-0.44182	2.93094	-1.958	1.43486	-0.87039
95.75322	-0.43994	2.93115	-1.96134	1.43404	-0.87107
95.78712	-0.43944	2.93123	-1.96266	1.43263	-0.87193
95.81991	-0.43898	2.93118	-1.96319	1.43135	-0.87276
95.85188	-0.43808	2.93115	-1.96359	1.43026	-0.87386
95.88678	-0.43614	2.9312	-1.96428	1.42933	-0.8748
95.91995	-0.43416	2.93128	-1.96485	1.42822	-0.87573
95.95163	-0.43154	2.93134	-1.96531	1.42678	-0.87613
95.98591	-0.4296	2.93139	-1.96592	1.42462	-0.87669
96.01913	-0.42812	2.93144	-1.96629	1.42356	-0.87795
96.05216	-0.4267	2.93143	-1.96713	1.42277	-0.88079
96.0857	-0.42534	2.93138	-1.96997	1.42222	-0.88496
96.11879	-0.42286	2.93134	-1.97344	1.42154	-0.88798
96.15268	-0.4203	2.93132	-1.97564	1.42047	-0.88915
96.18524	-0.41652	2.93134	-1.97656	1.41925	-0.88955
96.21936	-0.41354	2.93135	-1.97704	1.41733	-0.89031
96.25201	-0.41262	2.93134	-1.97789	1.41417	-0.89382
96.28582	-0.41114	2.93137	-1.97913	1.41143	-0.89948
96.319	-0.41022	2.93139	-1.98092	1.40919	-0.90435
96.35238	-0.40932	2.93144	-1.98201	1.40774	-0.90651
96.38634	-0.40778	2.9315	-1.983	1.40677	-0.90723
96.41841	-0.4069	2.93152	-1.98335	1.40594	-0.90821
96.45112	-0.40628	2.93156	-1.98393	1.40548	-0.90903

96.48492	-0.4039	2.93163	-1.98463	1.4051	-0.90949
96.51809	-0.4012	2.93174	-1.98541	1.40427	-0.91003
96.55055	-0.39788	2.932	-1.98668	1.40184	-0.91059
96.58465	-0.39692	2.93219	-1.98765	1.39887	-0.91136
96.61841	-0.3964	2.93233	-1.98822	1.39598	-0.91383
96.65112	-0.39562	2.93243	-1.98892	1.39483	-0.91641
96.68303	-0.39412	2.93247	-1.99018	1.39436	-0.91762
96.71705	-0.39128	2.9325	-1.99145	1.39373	-0.9191
96.75014	-0.39006	2.93252	-1.99295	1.39159	-0.92088
96.78363	-0.39004	2.93254	-1.99448	1.38906	-0.92203
96.81734	-0.3898	2.93254	-1.99546	1.38615	-0.92465
96.85089	-0.38882	2.93253	-1.99677	1.38456	-0.92682
96.88321	-0.38702	2.93249	-1.99809	1.38347	-0.92786
96.91541	-0.38506	2.9325	-1.99889	1.38298	-0.9284
96.94922	-0.38346	2.9325	-1.99926	1.38229	-0.92914
96.98343	-0.38224	2.93249	-2.00018	1.38196	-0.93022
97.01595	-0.3821	2.93241	-2.00301	1.38152	-0.93202
97.04999	-0.38178	2.93236	-2.00992	1.37942	-0.93366
97.08321	-0.38128	2.93236	-2.01586	1.37607	-0.93472
97.11535	-0.3804	2.93236	-2.01803	1.37465	-0.93508
97.14959	-0.37836	2.93237	-2.0192	1.37431	-0.93568
97.1827	-0.37654	2.93238	-2.02014	1.374	-0.93711
97.21618	-0.37594	2.9324	-2.02083	1.37336	-0.9406
97.24949	-0.37564	2.93242	-2.02184	1.37226	-0.94412
97.28242	-0.37518	2.93243	-2.02364	1.36912	-0.94763
97.31465	-0.37468	2.93242	-2.02576	1.36721	-0.94858
97.34903	-0.3739	2.93243	-2.02765	1.36671	-0.94907
97.38109	-0.37278	2.93241	-2.03004	1.36573	-0.95048
97.41468	-0.37182	2.93244	-2.03149	1.36435	-0.95351
97.44832	-0.37086	2.93245	-2.03263	1.36214	-0.95796

97.48093	-0.36974	2.93244	-2.03358	1.35875	-0.96172
97.51468	-0.36886	2.93248	-2.03416	1.35722	-0.96344
97.54786	-0.36768	2.9325	-2.03475	1.35614	-0.96443
97.58101	-0.36536	2.93271	-2.03553	1.35436	-0.96538
97.61425	-0.36356	2.9329	-2.03594	1.35177	-0.96694
97.64806	-0.36236	2.93302	-2.03668	1.34954	-0.96849
97.68121	-0.36202	2.93308	-2.03744	1.34885	-0.96951
97.71449	-0.36176	2.93311	-2.03822	1.34814	-0.97024
97.74753	-0.36142	2.93311	-2.03964	1.34763	-0.9712
97.78137	-0.3609	2.93307	-2.04093	1.34695	-0.97252
97.81446	-0.3605	2.93301	-2.04182	1.34511	-0.97591
97.84649	-0.36032	2.93295	-2.04277	1.34366	-0.97949
97.88046	-0.36014	2.93291	-2.04322	1.34294	-0.98163
97.91371	-0.3598	2.93297	-2.04429	1.34248	-0.98251
97.94725	-0.3591	2.93299	-2.04593	1.3419	-0.98331
97.97996	-0.35842	2.93295	-2.04741	1.34006	-0.9836
98.01399	-0.35756	2.93289	-2.05043	1.33701	-0.98427
98.04626	-0.35652	2.93286	-2.05436	1.33298	-0.98526
98.07976	-0.35562	2.9328	-2.05738	1.32809	-0.98802
98.11251	-0.35508	2.93273	-2.06037	1.32417	-0.99034
98.14602	-0.35468	2.93264	-2.0619	1.32165	-0.992
98.18	-0.3543	2.93251	-2.06252	1.31978	-0.99438
98.21295	-0.35356	2.93243	-2.06303	1.31812	-0.99664
98.24543	-0.35236	2.93239	-2.06357	1.3172	-0.99888
98.27878	-0.35084	2.93237	-2.06422	1.31588	-1.00382
98.31129	-0.3489	2.93235	-2.06535	1.31435	-1.00939
98.34509	-0.34738	2.93236	-2.06687	1.31348	-1.01356
98.37899	-0.34678	2.93224	-2.06814	1.31263	-1.01548
98.41197	-0.3466	2.93149	-2.0697	1.31161	-1.01604
98.44494	-0.3464	2.93093	-2.07153	1.31022	-1.01672

98.47821	-0.34614	2.93053	-2.07343	1.30735	-1.01753
98.51149	-0.3458	2.93038	-2.0758	1.30411	-1.01845
98.54528	-0.34562	2.93041	-2.07914	1.30234	-1.01944
98.57808	-0.34548	2.93042	-2.08272	1.30144	-1.01998
98.61	-0.3455	2.93044	-2.08618	1.30113	-1.02214
98.64382	-0.34548	2.93057	-2.09042	1.30059	-1.02675
98.67706	-0.3454	2.93067	-2.09482	1.2994	-1.03082
98.71031	-0.34504	2.9307	-2.09675	1.29805	-1.03413
98.74413	-0.34472	2.9306	-2.09764	1.29723	-1.03521
98.77715	-0.34444	2.93002	-2.09848	1.29662	-1.03577
98.81079	-0.3441	2.92948	-2.09991	1.29556	-1.03643
98.84425	-0.3439	2.92934	-2.10318	1.29294	-1.03786
98.87651	-0.34356	2.92925	-2.10777	1.28952	-1.04148
98.90972	-0.34316	2.92922	-2.10986	1.28559	-1.04745
98.94196	-0.34294	2.92916	-2.11069	1.28303	-1.05114
98.97632	-0.34236	2.92911	-2.112	1.28167	-1.05291
99.00929	-0.34178	2.92907	-2.11359	1.28028	-1.05368
99.04306	-0.3414	2.92902	-2.11739	1.27881	-1.05427
99.07599	-0.34106	2.92895	-2.12093	1.27752	-1.05534
99.10894	-0.34082	2.92882	-2.12288	1.27646	-1.05834
99.14122	-0.3406	2.92859	-2.12423	1.27542	-1.06405
99.17522	-0.3403	2.92844	-2.12525	1.27469	-1.06904
99.20849	-0.33982	2.92835	-2.12685	1.27234	-1.07206
99.24176	-0.3394	2.92833	-2.12979	1.26786	-1.07369
99.27501	-0.339	2.92834	-2.13161	1.26381	-1.07407
99.30856	-0.33872	2.92835	-2.13231	1.26011	-1.07465
99.3415	-0.33856	2.92834	-2.13288	1.25811	-1.0763
99.37509	-0.33846	2.92824	-2.13367	1.25767	-1.07715
99.40769	-0.33832	2.92807	-2.13539	1.25704	-1.07791
99.4408	-0.33802	2.92786	-2.13834	1.25621	-1.0784

99.47449	-0.33688	2.9277	-2.1406	1.25578	-1.07889
99.50711	-0.336	2.92761	-2.14306	1.25504	-1.07956
99.53991	-0.33542	2.9276	-2.1465	1.25299	-1.08266
99.57418	-0.33502	2.92758	-2.14906	1.24909	-1.08809
99.60712	-0.33482	2.92758	-2.152	1.2455	-1.09499
99.6407	-0.33438	2.92753	-2.15431	1.24427	-1.09968
99.67422	-0.33386	2.92739	-2.15495	1.24347	-1.10266
99.70675	-0.33364	2.9272	-2.15609	1.24149	-1.10534
99.73999	-0.33342	2.92702	-2.1576	1.236-1.10734	
99.77375	-0.33336	2.92682	-2.15988	1.23106	-1.10894
99.8068	-0.33328	2.9267	-2.1642	1.22877	-1.1105
99.83972	-0.33316	2.92664	-2.16863	1.22818	-1.11104
99.87326	-0.33288	2.92667	-2.17305	1.2276	-1.11166
99.90561	-0.3324	2.92671	-2.17751	1.22668	-1.11236
99.93889	-0.33172	2.92673	-2.17921	1.22358	-1.11319
99.97206	-0.33136	2.92672	-2.17997	1.21945	-1.11403
100.0057	-0.3312	2.92667	-2.18068	1.21443	-1.11548
100.0391	-0.33108	2.92658	-2.18117	1.2111	-1.11681
100.072	-0.33098	2.92649	-2.18234	1.20867	-1.11893
100.1057	-0.33082	2.92604	-2.18609	1.20779	-1.12172
100.1392	-0.3307	2.92498	-2.19093	1.20735	-1.12502
100.1719	-0.33058	2.92418	-2.19544	1.20696	-1.12826
100.2045	-0.33046	2.92383	-2.19763	1.20666	-1.13291
100.2371	-0.33036	2.9236	-2.19909	1.20597	-1.13929
100.2705	-0.33028	2.92343	-2.20115	1.20388	-1.14425
100.3037	-0.33016	2.9233	-2.20315	1.19928	-1.14762
100.3375	-0.33002	2.92324	-2.20555	1.19404	-1.14971
100.3709	-0.32976	2.92319	-2.20838	1.18896	-1.15116
100.4046	-0.32954	2.92317	-2.21007	1.18503	-1.15286
100.4373	-0.32942	2.9231	-2.21121	1.18252	-1.15508

100.4694	-0.3293	2.92298	-2.21309	1.18112	-1.15627
100.504	-0.32916	2.9229	-2.21449	1.18002	-1.15702
100.536	-0.32906	2.92287	-2.21708	1.1796	-1.15786
100.569	-0.32896	2.92277	-2.22044	1.17936	-1.15932
100.6033	-0.32882	2.92257	-2.22345	1.17915	-1.16223
100.6363	-0.32866	2.92228	-2.22577	1.17872	-1.16603
100.6701	-0.3283	2.92206	-2.22701	1.17776	-1.16948
100.7026	-0.32782	2.9219	-2.22785	1.17673	-1.17255
100.7356	-0.32752	2.92184	-2.22978	1.17523	-1.17429
100.7688	-0.32706	2.92175	-2.23287	1.17271	-1.17647
100.8025	-0.32668	2.92162	-2.23853	1.16986	-1.17927
100.8354	-0.32632	2.92142	-2.24342	1.16482	-1.1829
100.8688	-0.32616	2.92089	-2.24562	1.15911	-1.18731
100.9017	-0.32602	2.92055	-2.24645	1.15629	-1.18905
100.9352	-0.3259	2.92041	-2.24756	1.15501	-1.18969
100.9687	-0.3258	2.92032	-2.24932	1.15391	-1.19009
101.0018	-0.3257	2.92019	-2.25104	1.15301	-1.19108
101.0338	-0.32558	2.91999	-2.2544	1.15183	-1.1945
101.0666	-0.32542	2.91927	-2.25772	1.14996	-1.19984
101.1	-0.32526	2.91793	-2.26163	1.14719	-1.20304
101.134	-0.32512	2.91725	-2.26669	1.14256	-1.20563
101.1676	-0.32502	2.9168	-2.2709	1.1355	-1.20684
101.201	-0.32492	2.91639	-2.27319	1.13077	-1.20796
101.2332	-0.32482	2.91608	-2.27603	1.12879	-1.21125
101.2666	-0.32456	2.91545	-2.27859	1.12767	-1.21743
101.2994	-0.32418	2.91465	-2.28077	1.12678	-1.22465
101.334	-0.32394	2.91432	-2.28318	1.12547	-1.22956
101.3653	-0.3238	2.9141	-2.2862	1.1243	-1.23073
101.3984	-0.3237	2.91388	-2.2883	1.1224	-1.23144
101.4317	-0.3236	2.91362	-2.29344	1.12014	-1.23286

101.4657	-0.32352	2.91301	-2.29978	1.11714	-1.23701
101.4989	-0.32342	2.91253	-2.30261	1.1149	-1.24264
101.5317	-0.3233	2.91236	-2.30384	1.11393	-1.24694
101.5652	-0.32316	2.9122	-2.30434	1.11328	-1.25005
101.5975	-0.32302	2.91201	-2.30469	1.11169	-1.25141
101.6321	-0.3229	2.9118	-2.30519	1.10879	-1.25264
101.6653	-0.32276	2.91157	-2.30668	1.10498	-1.25367
101.6978	-0.32266	2.91138	-2.30922	1.10247	-1.25465
101.73	-0.32254	2.91125	-2.31306	1.10086	-1.25685
101.7645	-0.32236	2.91111	-2.32116	1.09912	-1.2596
101.7973	-0.3222	2.91096	-2.33018	1.0962	-1.26249
101.8306	-0.32194	2.91073	-2.33424	1.09163	-1.26589
101.8641	-0.32162	2.91041	-2.33739	1.08697	-1.27112
101.897	-0.3215	2.9099	-2.33941	1.08265	-1.27561
101.9297	-0.3214	2.90964	-2.34108	1.0796	-1.28051
101.9623	-0.32134	2.90949	-2.34263	1.07778	-1.28402
101.9957	-0.32126	2.90933	-2.3442	1.07622	-1.28536
102.0292	-0.32114	2.90901	-2.34622	1.07442	-1.28567
102.0627	-0.321	2.90864	-2.34851	1.07308	-1.28644
102.0958	-0.32088	2.90814	-2.35113	1.07054	-1.29004
102.129	-0.32074	2.90775	-2.35534	1.06538	-1.29647
102.162	-0.32062	2.90749	-2.35875	1.06023	-1.3043
102.1952	-0.32054	2.90719	-2.3622	1.05702	-1.30877
102.2283	-0.32044	2.90637	-2.36503	1.05504	-1.31158
102.2608	-0.32036	2.90594	-2.36798	1.05414	-1.31498
102.2946	-0.32022	2.90567	-2.37206	1.05272	-1.3173
102.3274	-0.31958	2.90541	-2.37735	1.05066	-1.31914
102.3615	-0.31886	2.90521	-2.38345	1.04823	-1.32022
102.3931	-0.31852	2.90504	-2.38834	1.04527	-1.32096
102.4269	-0.31842	2.90483	-2.39111	1.04212	-1.32234

102.4599	-0.31832	2.9046	-2.3929	1.03833	-1.32397
102.4935	-0.31822	2.90435	-2.39506	1.03533	-1.3262
102.5268	-0.3181	2.90386	-2.3969	1.03292	-1.33056
102.5604	-0.318	2.90309	-2.40133	1.03039	-1.33529
102.593	-0.31794	2.90264	-2.40388	1.02838	-1.33881
102.6258	-0.31788	2.90196	-2.40558	1.02497	-1.34091
102.6592	-0.31784	2.90158	-2.40922	1.02237	-1.34234
102.6923	-0.31774	2.9013	-2.41504	1.02127	-1.34425
102.7242	-0.31766	2.90106	-2.42012	1.01949	-1.34727
102.758	-0.31758	2.90082	-2.42561	1.01631	-1.3502
102.7923	-0.31748	2.90029	-2.42669	1.01174	-1.35251
102.825	-0.31738	2.89953	-2.42706	1.00436	-1.35613
102.858	-0.31726	2.89889	-2.42913	1.00097	-1.36475
102.8907	-0.31716	2.89851	-2.43235	0.99957	-1.37145
102.9232	-0.3171	2.89815	-2.4341	0.99892	-1.37487
102.9574	-0.31704	2.89776	-2.43608	0.99788	-1.37692
102.9904	-0.31692	2.89735	-2.43816	0.9965	-1.37761
103.0235	-0.31678	2.89704	-2.44181	0.99345	-1.37832
103.0558	-0.3166	2.89678	-2.45223	0.98833	-1.38053
103.0898	-0.31644	2.89651	-2.46195	0.98341	-1.3823
103.1239	-0.3163	2.89603	-2.46446	0.9807	-1.38522
103.1566	-0.3162	2.89432	-2.46551	0.97842	-1.39008
103.1893	-0.31608	2.89252	-2.46615	0.97691	-1.39459
103.2227	-0.31596	2.89191	-2.46897	0.97458	-1.39836
103.2564	-0.31584	2.89161	-2.47453	0.97191	-1.40272
103.2888	-0.31574	2.89135	-2.48092	0.96927	-1.40685
103.3216	-0.31568	2.89109	-2.48557	0.96693	-1.41068
103.3541	-0.31562	2.89077	-2.48921	0.96456	-1.41335
103.388	-0.31554	2.89041	-2.49481	0.96189	-1.41508
103.4214	-0.31544	2.89008	-2.50009	0.95833	-1.41811

103.4552	-0.31536	2.88969	-2.50289	0.95464	-1.42202
103.4878	-0.31522	2.88925	-2.5046	0.95054	-1.42606
103.5209	-0.3151	2.88866	-2.50522	0.94571	-1.42907
103.554	-0.31498	2.88773	-2.50584	0.94016	-1.4316
103.5868	-0.31486	2.88668	-2.50872	0.93747	-1.43375
103.6202	-0.31478	2.88627	-2.5153	0.93559	-1.43815
103.6534	-0.31472	2.88592	-2.52262	0.93384	-1.44374
103.6864	-0.31466	2.88561	-2.52891	0.93234	-1.44925
103.7195	-0.31464	2.8853	-2.53373	0.93004	-1.45554
103.752	-0.31458	2.88492	-2.5386	0.92662	-1.4589
103.7852	-0.3145	2.88451	-2.54673	0.92463	-1.46107
103.819	-0.31438	2.88401	-2.55533	0.92292	-1.46253
103.8516	-0.31414	2.88359	-2.55908	0.9191	-1.464
103.8846	-0.31402	2.88302	-2.55971	0.91213	-1.46628
103.9173	-0.31396	2.88245	-2.55929	0.9064	-1.46944
103.9517	-0.31392	2.8818	-2.55976	0.9023	-1.4716
103.985	-0.31388	2.88083	-2.56112	0.90098	-1.47371
104.0175	-0.31382	2.87998	-2.56472	0.90039	-1.47519
104.0497	-0.31376	2.87954	-2.57084	0.89828	-1.47728
104.0831	-0.31366	2.87889	-2.57774	0.89427	-1.48319
104.1165	-0.31358	2.87843	-2.58418	0.88898	-1.48915
104.15	-0.31348	2.87804	-2.58838	0.88274	-1.49426
104.1834	-0.31338	2.8775	-2.59235	0.8811	-1.49834
104.2166	-0.31334	2.877-2.59526	0.88059	-1.50168	
104.2488	-0.3133	2.8765	-2.59935	0.88035	-1.50419
104.2825	-0.31328	2.87592	-2.60545	0.87929	-1.50727
104.3148	-0.31324	2.87541	-2.60995	0.87478	-1.50971
104.349	-0.3132	2.87503	-2.61161	0.86697	-1.51203
104.3814	-0.31316	2.87466	-2.61408	0.86014	-1.5136
104.4147	-0.31312	2.8743	-2.61982	0.85767	-1.51499

104.4478	-0.31308	2.87392	-2.63143	0.85589	-1.51685
104.4813	-0.31304	2.8734	-2.64166	0.85427	-1.51973
104.5141	-0.31298	2.87272	-2.64615	0.85297	-1.52493
104.5485	-0.31292	2.87181	-2.64821	0.85164	-1.53314
104.5804	-0.31284	2.87053	-2.64917	0.8491	-1.53953
104.6138	-0.31276	2.86949	-2.65109	0.846-1.54397	
104.648	-0.31262	2.86903	-2.65259	0.84254	-1.54777
104.6798	-0.31256	2.86863	-2.65541	0.84005	-1.55077
104.7123	-0.31254	2.86826	-2.66148	0.83874	-1.55472
104.7455	-0.3125	2.86786	-2.66829	0.83785	-1.55795
104.7787	-0.31244	2.86717	-2.67538	0.83441	-1.55895
104.8119	-0.31236	2.86603	-2.68003	0.82759	-1.55945
104.8459	-0.31228	2.86513	-2.68157	0.81935	-1.55954
104.879	-0.31222	2.86398	-2.68504	0.81214	-1.56001
104.9123	-0.31218	2.86328	-2.69009	0.80903	-1.56202
104.946	-0.31214	2.86283	-2.69523	0.80824	-1.56489
104.978	-0.31206	2.86238	-2.70173	0.80768	-1.56879
105.0111	-0.312	2.86192	-2.70787	0.80652	-1.57312
105.0443	-0.31192	2.86148	-2.71343	0.80392	-1.57613
105.077	-0.31188	2.86105	-2.71914	0.7986	-1.57956
105.1106	-0.31184	2.8606	-2.72662	0.7937	-1.58426
105.1442	-0.31176	2.85905	-2.73699	0.79045	-1.58714
105.177	-0.31168	2.85548	-2.73789	0.78834	-1.58927
105.2098	-0.3116	2.85021	-2.73688	0.78702	-1.59102
105.2434	-0.31152	2.84761	-2.7358	0.78479	-1.59344
105.2761	-0.31146	2.84659	-2.73595	0.78122	-1.59581
105.3094	-0.31144	2.84614	-2.74402	0.77865	-1.59783
105.3419	-0.31142	2.84545	-2.76207	0.77715	-1.59886
105.3754	-0.31138	2.84444	-2.77622	0.77636	-1.60123
105.4077	-0.31134	2.8438	-2.7839	0.77402	-1.60653

105.4406	-0.3113	2.84335	-2.78473	0.76498	-1.61333
105.474	-0.31128	2.84288	-2.78453	0.75359	-1.61723
105.5078	-0.31126	2.84245	-2.78553	0.74743	-1.61893
105.541	-0.31124	2.84132	-2.78627	0.74526	-1.62061
105.5745	-0.31122	2.84009	-2.78979	0.74465	-1.62183
105.6073	-0.31118	2.83856	-2.79738	0.74419	-1.62334
105.6406	-0.31114	2.83796	-2.80643	0.74267	-1.62424
105.6728	-0.3111	2.83752	-2.81481	0.74108	-1.62531
105.7044	-0.31108	2.83728	-2.81935	0.73939	-1.6272
105.7384	-0.31104	2.83707	-2.82206	0.73594	-1.62947
105.7724	-0.31098	2.83675	-2.82588	0.73261	-1.63189
105.8052	-0.31094	2.83599	-2.83273	0.72941	-1.63414
105.8387	-0.31092	2.83499	-2.83919	0.72414	-1.63537
105.8717	-0.31094	2.83329	-2.84539	0.71952	-1.63711
105.905	-0.31094	2.83219	-2.84976	0.7169	-1.63882
105.9384	-0.3109	2.83135	-2.85213	0.71525	-1.64117
105.9715	-0.31086	2.8309	-2.85704	0.71441	-1.64316
106.005	-0.31084	2.83047	-2.86155	0.71374	-1.64406
106.0363	-0.3109	2.83009	-2.86779	0.71211	-1.64514
106.0702	-0.311	2.8297	-2.87472	0.70847	-1.64624
106.1039	-0.31114	2.82915	-2.87819	0.70293	-1.64798
106.1363	-0.31118	2.82868	-2.8827	0.69678	-1.64994
106.1706	-0.3112	2.82815	-2.88925	0.6911	-1.6515
106.2031	-0.31122	2.82722	-2.89664	0.68777	-1.65489
106.2362	-0.31126	2.82646	-2.90478	0.68525	-1.65992
106.2688	-0.31128	2.82595	-2.91044	0.68026	-1.66589
106.3034	-0.3113	2.82514	-2.91463	0.67478	-1.66898
106.3371	-0.31132	2.82315	-2.9174	0.67155	-1.66939
106.3686	-0.31132	2.81721	-2.92197	0.66918	-1.66944
106.4011	-0.31136	2.81081	-2.92979	0.66748	-1.66955

106.4352	-0.31138	2.8071	-2.93598	0.6649	-1.66975
106.4677	-0.31136	2.80589	-2.94292	0.66221	-1.67117
106.5011	-0.31136	2.80548	-2.95158	0.65921	-1.67276
106.5348	-0.3114	2.80497	-2.96053	0.65686	-1.67379
106.5663	-0.31144	2.80447	-2.96848	0.65377	-1.67433
106.6001	-0.31146	2.80416	-2.97269	0.64956	-1.67443
106.6334	-0.3115	2.80378	-2.97693	0.64393	-1.67445
106.6666	-0.3115	2.80294	-2.98209	0.63895	-1.67458
106.6997	-0.3115	2.80169	-2.99033	0.63479	-1.67537
106.7328	-0.31152	2.80126	-2.9975	0.63121	-1.67757
106.7662	-0.31156	2.80089	-2.99956	0.62827	-1.67884
106.7991	-0.31156	2.80025	-3.00006	0.62561	-1.67928
106.8326	-0.31156	2.7977	-3.00319	0.62232	-1.67997
106.8643	-0.31152	2.79361	-3.01207	0.61613	-1.68039
106.8969	-0.3115	2.79019	-3.0236	0.60824	-1.68092
106.93	-0.31146	2.78838	-3.03403	0.60368	-1.68132
106.9644	-0.31146	2.7876	-3.04181	0.60121	-1.68164
106.9976	-0.31146	2.78709	-3.04534	0.60032	-1.68185
107.0312	-0.31146	2.78668	-3.0501	0.59925	-1.68201
107.064	-0.31148	2.78633	-3.05453	0.59815	-1.6823
107.0969	-0.31148	2.78576	-3.06408	0.5974	-1.68239
107.1298	-0.31148	2.78525	-3.0756	0.59618	-1.68245
107.1628	-0.3115	2.78469	-3.08194	0.59437	-1.6825
107.1955	-0.3115	2.78401	-3.08825	0.5891	-1.68255
107.2288	-0.3115	2.78325	-3.09015	0.5829	-1.68267
107.2622	-0.31154	2.78262	-3.08996	0.57857	-1.68279
107.2948	-0.3116	2.78225	-3.09586	0.5757	-1.68301
107.3291	-0.31172	2.7819	-3.10474	0.57362	-1.6834
107.3613	-0.31192	2.78145	-3.11538	0.57134	-1.68367
107.395	-0.312	2.78039	-3.12526	0.56714	-1.68404

107.4277	-0.31202	2.77832	-3.13171	0.56289	-1.68433
107.4607	-0.31202	2.77521	-3.13384	0.55939	-1.68466
107.4943	-0.31202	2.77338	-3.13576	0.55571	-1.6848
107.5274	-0.312	2.77255	-3.14044	0.55157	-1.68484
107.5596	-0.312	2.77186	-3.15031	0.54613	-1.68484
107.5935	-0.31198	2.7714	-3.15991	0.5417	-1.68485
107.6262	-0.31196	2.7709	-3.16851	0.53866	-1.68497
107.6592	-0.31196	2.77043	-3.17811	0.53689	-1.68517
107.6928	-0.312	2.77	-3.18426	0.53573	-1.68525
107.7254	-0.312	2.76937	-3.19536	0.53154	-1.68521
107.7582	-0.31196	2.7682	-3.20632	0.52606	-1.68488
107.7922	-0.31192	2.7675	-3.20808	0.52054	-1.68453
107.8243	-0.3119	2.76692	-3.20906	0.51517	-1.68452
107.8583	-0.31194	2.76647	-3.21292	0.51204	-1.68489
107.8912	-0.31196	2.76598	-3.21786	0.51107	-1.68553
107.9231	-0.31196	2.76554	-3.22283	0.5095	-1.68646
107.9565	-0.31196	2.76446	-3.23144	0.50876	-1.68685
107.9904	-0.31196	2.76312	-3.23805	0.5069	-1.68698
108.0232	-0.31196	2.76113	-3.24732	0.50198	-1.68695
108.0565	-0.31196	2.75916	-3.26186	0.49773	-1.68685
108.0893	-0.31192	2.75627	-3.26909	0.493	-1.68675
108.1219	-0.31192	2.75277	-3.27702	0.4893	-1.68668
108.1554	-0.31194	2.74813	-3.28239	0.48758	-1.6867
108.1884	-0.31194	2.74517	-3.28611	0.48689	-1.68667
108.2211	-0.31194	2.74313	-3.29321	0.48565	-1.68652
108.2547	-0.3119	2.74232	-3.29818	0.4839	-1.68567
108.2881	-0.31188	2.74155	-3.30654	0.47961	-1.68443
108.3214	-0.31188	2.74108	-3.31773	0.47287	-1.68428
108.3547	-0.3119	2.74031	-3.32474	0.46695	-1.68423
108.3868	-0.31192	2.73764	-3.33098	0.46334	-1.68424

108.4208	-0.3119	2.73285	-3.33665	0.4607	-1.68425
108.453	-0.3119	2.72744	-3.34169	0.45893	-1.68404
108.4866	-0.31192	2.7227	-3.3527	0.45813	-1.68379
108.5194	-0.31194	2.72015	-3.36422	0.45693	-1.68348
108.5526	-0.31198	2.71949	-3.37295	0.45554	-1.68316
108.5856	-0.31198	2.71866	-3.37936	0.45305	-1.68297
108.6191	-0.31198	2.71748	-3.3863	0.45015	-1.68277
108.6529	-0.31194	2.7158	-3.39279	0.44844	-1.68263
108.6857	-0.3119	2.71378	-3.40361	0.44757	-1.68257
108.7181	-0.3119	2.71269	-3.41234	0.4459	-1.68254
108.7518	-0.31192	2.7121	-3.41615	0.44303	-1.68253
108.7844	-0.31198	2.71097	-3.42229	0.43761	-1.6825
108.8185	-0.31204	2.70911	-3.42808	0.43231	-1.68228
108.8515	-0.31212	2.70637	-3.43473	0.43034	-1.68184
108.8845	-0.31218	2.7043	-3.44679	0.43013	-1.68152
108.9176	-0.31218	2.70315	-3.45854	0.43021	-1.68108
108.9508	-0.31216	2.70258	-3.46329	0.42979	-1.68076
108.9838	-0.31212	2.70213	-3.46519	0.4285	-1.68025
109.0161	-0.31212	2.70172	-3.46992	0.42575	-1.67927
109.0496	-0.31218	2.70124	-3.47943	0.41975	-1.67896
109.0824	-0.31224	2.70065	-3.49019	0.41276	-1.67877
109.1166	-0.31226	2.6999	-3.49879	0.40633	-1.67857
109.1497	-0.31224	2.69788	-3.51017	0.40345	-1.67835
109.1832	-0.31222	2.69518	-3.51786	0.40338	-1.67806
109.2152	-0.3122	2.69257	-3.52386	0.40461	-1.67764
109.2484	-0.3122	2.69099	-3.53383	0.40548	-1.67717
109.2823	-0.31222	2.69035	-3.53959	0.40536	-1.67686
109.315	-0.31222	2.68884	-3.54489	0.40274	-1.67663
109.3484	-0.31222	2.68669	-3.55136	0.39875	-1.6764
109.3815	-0.31222	2.68511	-3.5553	0.39672	-1.67624

109.4141	-0.31222	2.68412	-3.56231	0.39706	-1.67603
109.4463	-0.31222	2.68353	-3.56735	0.3976	-1.67576
109.48	-0.31222	2.68284	-3.58168	0.3979	-1.67549
109.5144	-0.3122	2.6819	-3.59807	0.39723	-1.67525
109.5475	-0.31222	2.67989	-3.60541	0.39471	-1.67501
109.5808	-0.3122	2.67704	-3.61574	0.39105	-1.67482
109.6142	-0.31208	2.67509	-3.61944	0.38914	-1.6746
109.6474	-0.312	2.67366	-3.62518	0.38824	-1.67432
109.6804	-0.31194	2.67291	-3.64123	0.38807	-1.67404
109.7132	-0.3119	2.67242	-3.65174	0.38827	-1.67372
109.7459	-0.31188	2.67081	-3.65601	0.38845	-1.67346
109.7794	-0.31186	2.66713	-3.66065	0.3886	-1.6733
109.812	-0.31186	2.66335	-3.66619	0.38848	-1.67321
109.8457	-0.31188	2.66071	-3.67521	0.38669	-1.67316
109.8788	-0.31192	2.65998	-3.68804	0.3856	-1.67311
109.9134	-0.31192	2.65919	-3.69649	0.38523	-1.67293
109.9466	-0.31192	2.65833	-3.70251	0.38485	-1.67266
109.9804	-0.3119	2.65685	-3.70954	0.38489	-1.67237
110.0136	-0.31188	2.65307	-3.72043	0.38503	-1.67217
110.0458	-0.31188	2.65056	-3.73009	0.38512	-1.67201
110.0778	-0.3119	2.64906	-3.74267	0.38527	-1.6719
110.1109	-0.3119	2.6478	-3.75039	0.38528	-1.67178
110.1444	-0.31192	2.64653	-3.75721	0.38517	-1.67167
110.179	-0.31192	2.64549	-3.76168	0.38515	-1.67163
110.2121	-0.31188	2.6435	-3.76763	0.38511	-1.6717
110.2449	-0.31186	2.64196	-3.77882	0.38502	-1.67189
110.2784	-0.31184	2.64011	-3.79749	0.38494	-1.67228
110.3118	-0.31184	2.6375	-3.80979	0.38486	-1.67244
110.3451	-0.31184	2.63504	-3.81399	0.38479	-1.67248
110.3785	-0.31188	2.6332	-3.81772	0.3848	-1.6724

110.4117	-0.3119	2.63095	-3.82256	0.38485	-1.67227
110.4448	-0.31194	2.62937	-3.83677	0.38503	-1.67213
110.4777	-0.31196	2.62819	-3.84951	0.38566	-1.67208
110.5115	-0.31196	2.62741	-3.85286	0.38693	-1.67212
110.5443	-0.31196	2.62641	-3.85687	0.38743	-1.67236
110.5777	-0.31192	2.62365	-3.86775	0.38767	-1.67347
110.6111	-0.31188	2.61953	-3.87771	0.38771	-1.67513
110.6452	-0.31186	2.61473	-3.89498	0.38771	-1.6774
110.6777	-0.31184	2.61157	-3.90467	0.38776	-1.67934
110.7116	-0.31186	2.60935	-3.9035	0.38806	-1.67995
110.7444	-0.31188	2.60722	-3.90878	0.38946	-1.68012
110.7778	-0.3119	2.60553	-3.92114	0.39194	-1.68034
110.8108	-0.31192	2.60439	-3.9289	0.39559	-1.68053
110.844	-0.31194	2.60242	-3.94099	0.39831	-1.68077
110.8774	-0.31192	2.60139	-3.95068	0.40019	-1.68128
110.9107	-0.3119	2.60056	-3.95608	0.40068	-1.68199
110.9443	-0.3119	2.5994	-3.97128	0.40073	-1.68342
110.9783	-0.31188	2.59811	-3.98252	0.40078	-1.68548
111.0123	-0.31188	2.59599	-3.98735	0.40111	-1.68707
111.0451	-0.31186	2.59322	-4.00089	0.40418	-1.68796
111.0787	-0.31184	2.59149	-4.00579	0.41051	-1.6887
111.1117	-0.31184	2.58765	-4.01553	0.41555	-1.68997
111.1445	-0.31186	2.58378	-4.02679	0.41704	-1.69132
111.1784	-0.31188	2.5811	-4.03355	0.41759	-1.69223
111.2113	-0.3119	2.57951	-4.04158	0.41895	-1.69286
111.2444	-0.31192	2.57875	-4.05191	0.42289	-1.69403
111.2776	-0.3119	2.57591	-4.05935	0.4284	-1.6961
111.3119	-0.3119	2.57007	-4.07218	0.43199	-1.69951
111.3455	-0.31188	2.56277	-4.08206	0.4325	-1.70238
111.379	-0.31188	2.55818	-4.09486	0.43279	-1.70461

111.412	-0.31186	2.55652	-4.10913	0.43333	-1.70764
111.4452	-0.31184	2.55576	-4.12011	0.43575	-1.71106
111.4788	-0.3118	2.55435	-4.12075	0.43929	-1.71339
111.5122	-0.31182	2.55124	-4.12675	0.44248	-1.71506
111.545	-0.31182	2.54778	-4.13681	0.44471	-1.7176
111.5786	-0.31182	2.5458	-4.15203	0.44564	-1.7217
111.6125	-0.3118	2.54508	-4.16129	0.4475	-1.7273
111.6463	-0.3118	2.54447	-4.17332	0.44907	-1.73384
111.6793	-0.3118	2.54349	-4.17427	0.45029	-1.73905
111.7134	-0.31184	2.54072	-4.17754	0.45159	-1.74317
111.7464	-0.31186	2.53609	-4.19314	0.45268	-1.74737
111.7799	-0.31186	2.53049	-4.20453	0.45416	-1.7493
111.8133	-0.31188	2.52478	-4.21886	0.45568	-1.75034
111.8473	-0.31188	2.51931	-4.23406	0.45758	-1.75096
111.8814	-0.31192	2.51762	-4.23672	0.46065	-1.75197
111.9148	-0.31196	2.51722	-4.24312	0.46548	-1.75401
111.947	-0.31196	2.51708	-4.2474	0.46984	-1.75732
111.981	-0.31198	2.51702	-4.2542	0.47275	-1.76153
112.0141	-0.31196	2.51635	-4.26938	0.47472	-1.76484
112.0474	-0.31194	2.51447	-4.28546	0.47625	-1.76848
112.0808	-0.31192	2.51116	-4.29617	0.47777	-1.77213
112.1141	-0.31186	2.5087	-4.30606	0.4791	-1.77813
112.1472	-0.31182	2.50617	-4.30977	0.48054	-1.785
112.1814	-0.31182	2.50538	-4.31907	0.4824	-1.78818
112.2151	-0.31182	2.50506	-4.33312	0.48454	-1.78927
112.2486	-0.31182	2.50487	-4.34711	0.48675	-1.79004
112.2813	-0.31184	2.50444	-4.36263	0.48795	-1.79029
112.3141	-0.3119	2.50213	-4.37719	0.48909	-1.79192
112.3484	-0.31202	2.49685	-4.38218	0.48977	-1.79631
112.381	-0.31206	2.49086	-4.38136	0.49126	-1.80303

112.4156	-0.31208	2.48666	-4.38786	0.49338	-1.8103
112.4482	-0.31212	2.48373	-4.39682	0.49548	-1.81457
112.4821	-0.31216	2.48182	-4.40882	0.49732	-1.81728
112.516	-0.3122	2.47929	-4.4273	0.49814	-1.8185
112.5486	-0.31224	2.47763	-4.43989	0.50031	-1.82045
112.5826	-0.31222	2.47706	-4.44601	0.5027	-1.82546
112.6156	-0.3122	2.47687	-4.45263	0.5046	-1.82934
112.6498	-0.31216	2.47672	-4.46136	0.50637	-1.83337
112.6828	-0.31214	2.47628	-4.4674	0.50762	-1.83751
112.7164	-0.31216	2.47451	-4.48499	0.50897	-1.83965
112.7496	-0.31228	2.47194	-4.49458	0.515-1.84445	
112.7833	-0.31256	2.46827	-4.5075	0.52058	-1.84943
112.8172	-0.31262	2.46476	-4.51905	0.52299	-1.85185
112.8503	-0.31262	2.4617	-4.52681	0.52463	-1.85481
112.8839	-0.3126	2.4579	-4.5418	0.5258	-1.8622
112.9169	-0.31252	2.455-4.55192	0.52675	-1.86927	
112.9502	-0.3125	2.45224	-4.56146	0.52781	-1.87659
112.9837	-0.31248	2.45106	-4.56532	0.52872	-1.88012
113.0176	-0.31246	2.45037	-4.57259	0.52919	-1.87987
113.0506	-0.31246	2.44867	-4.58347	0.52951	-1.87935
113.0844	-0.31246	2.44602	-4.59115	0.52979	-1.87988
113.1186	-0.31246	2.44269	-4.60595	0.53008	-1.88298
113.1511	-0.31248	2.43608	-4.62128	0.53031	-1.89084
113.1847	-0.31248	2.43212	-4.63095	0.53057	-1.90205
113.2182	-0.31248	2.42891	-4.64318	0.53128	-1.90835
113.2508	-0.31248	2.42598	-4.64961	0.53173	-1.91229
113.2842	-0.31248	2.42503	-4.65767	0.53182	-1.91584
113.318	-0.3125	2.42435	-4.67468	0.53173	-1.91937
113.3514	-0.31252	2.42256	-4.69098	0.5315	-1.92189
113.3846	-0.31254	2.41833	-4.69765	0.53142	-1.9244

113.4185	-0.31254	2.41172	-4.70459	0.53147	-1.92684
113.4518	-0.31256	2.4071	-4.71125	0.53156	-1.92805
113.486	-0.3126	2.40326	-4.72052	0.53172	-1.92889
113.5195	-0.31262	2.40202	-4.73333	0.53186	-1.93138
113.5523	-0.31266	2.40149	-4.7502	0.53195	-1.9381
113.586	-0.31266	2.40103	-4.76223	0.53203	-1.95158
113.619	-0.31266	2.40044	-4.77065	0.53204	-1.95951
113.6509	-0.31266	2.39959	-4.78511	0.53197	-1.96365
113.6851	-0.31266	2.39764	-4.79107	0.53177	-1.96533
113.7186	-0.3127	2.39419	-4.79793	0.53107	-1.96815
113.7521	-0.31272	2.38987	-4.80961	0.53041	-1.97084
113.7847	-0.31272	2.38493	-4.81414	0.52993	-1.97486
113.8185	-0.3127	2.38003	-4.82556	0.52965	-1.97798
113.8521	-0.31272	2.37589	-4.84228	0.52941	-1.98021
113.8845	-0.31274	2.37358	-4.85187	0.52854	-1.98293
113.9189	-0.31276	2.37291	-4.86482	0.52751	-1.98682
113.9518	-0.31276	2.37216	-4.87168	0.5271	-1.9921
113.9853	-0.31278	2.37131	-4.87852	0.52648	-1.99949
114.0175	-0.31278	2.37014	-4.89034	0.52534	-2.00464
114.0511	-0.3128	2.36765	-4.90391	0.52298	-2.00741
114.0847	-0.31284	2.36409	-4.91479	0.52005	-2.00943
114.1178	-0.31288	2.36021	-4.9274	0.51848	-2.01119
114.1521	-0.31292	2.35484	-4.93492	0.51835	-2.01398
114.1848	-0.31294	2.35099	-4.94843	0.51828	-2.01822
114.2175	-0.31298	2.34864	-4.96087	0.51779	-2.02407
114.2507	-0.31302	2.34692	-4.9664	0.51435	-2.03373
114.2838	-0.31304	2.34313	-4.97717	0.50859	-2.0433
114.3174	-0.31306	2.34003	-4.99478	0.5058	-2.04676
114.3512	-0.31306	2.3383	-5.00899	0.50499	-2.0478
114.3832	-0.3131	2.33637	-5.02484	0.50432	-2.04874

114.417	-0.31316	2.33418	-5.03806	0.50339	-2.05134
114.4504	-0.31322	2.33107	-5.04275	0.50036	-2.0565
114.4839	-0.31324	2.32546	-5.05188	0.49641	-2.06396
114.5173	-0.31324	2.3223	-5.0619	0.49407	-2.07106
114.5505	-0.31324	2.32046	-5.07052	0.49272	-2.07626
114.5839	-0.31324	2.31754	-5.08111	0.49173	-2.08009
114.6174	-0.3133	2.31306	-5.09336	0.49103	-2.08406
114.6503	-0.31334	2.30785	-5.10506	0.4893	-2.09067
114.6832	-0.31336	2.30268	-5.1209	0.48244	-2.09845
114.7167	-0.31334	2.30045	-5.13223	0.47498	-2.10156
114.7503	-0.31332	2.2993	-5.14005	0.46867	-2.10278
114.7831	-0.31326	2.29838	-5.14669	0.46193	-2.10303
114.8153	-0.31328	2.29787	-5.15532	0.45831	-2.10549
114.8486	-0.31332	2.29721	-5.16865	0.45481	-2.11179
114.8819	-0.31338	2.29659	-5.18325	0.44832	-2.12326
114.9157	-0.31344	2.2951	-5.19766	0.44055	-2.13317
114.9484	-0.31348	2.29123	-5.2086	0.43124	-2.141
114.9811	-0.31348	2.28457	-5.21983	0.42484	-2.14404
115.015	-0.31348	2.27818	-5.22314	0.42183	-2.14579
115.0477	-0.31344	2.27353	-5.23383	0.41896	-2.14661
115.0814	-0.31342	2.26982	-5.24948	0.41449	-2.14837
115.1144	-0.31342	2.26633	-5.26079	0.40528	-2.15204
115.147	-0.31344	2.26342	-5.27899	0.39536	-2.15697
115.1803	-0.31346	2.2612	-5.29094	0.39168	-2.1627
115.2129	-0.31352	2.2603	-5.30127	0.39066	-2.16859
115.2452	-0.31356	2.25958	-5.31499	0.3902	-2.1758
115.2798	-0.3136	2.25662	-5.33156	0.38758	-2.18284
115.3122	-0.3136	2.25395	-5.33791	0.38209	-2.18729
115.3459	-0.3136	2.25264	-5.35117	0.3754	-2.1883
115.3788	-0.3136	2.25174	-5.36066	0.37038	-2.18953

115.4127	-0.31364	2.25069	-5.37237	0.36441	-2.19252
115.4448	-0.3137	2.24923	-5.38385	0.36093	-2.19826
115.4787	-0.31374	2.24649	-5.39506	0.35744	-2.20749
115.5117	-0.31376	2.24417	-5.40054	0.35615	-2.21696
115.5451	-0.31372	2.24204	-5.40949	0.356-2.2241	
115.5771	-0.31368	2.23961	-5.4156	0.35571	-2.23093
115.6105	-0.31368	2.23625	-5.43348	0.35459	-2.23853
115.6444	-0.3137	2.23447	-5.44827	0.35289	-2.24464
115.6778	-0.31374	2.23341	-5.46299	0.34795	-2.24802
115.7115	-0.31376	2.23162	-5.4776	0.34149	-2.25048
115.7438	-0.31376	2.22919	-5.48671	0.3363	-2.25022
115.7776	-0.3138	2.22406	-5.49799	0.33335	-2.25032
115.8099	-0.31384	2.2161	-5.51138	0.33295	-2.25252
115.8432	-0.3139	2.21111	-5.51793	0.33314	-2.25963
115.8763	-0.31392	2.20808	-5.52965	0.33259	-2.27022
115.9097	-0.31386	2.20562	-5.54257	0.32925	-2.27948
115.9425	-0.31378	2.20518	-5.56061	0.31992	-2.2842
115.9762	-0.31376	2.20494	-5.58403	0.30871	-2.28859
116.0082	-0.31378	2.20478	-5.59864	0.30323	-2.29431
116.0412	-0.31382	2.20452	-5.60667	0.29933	-2.2999
116.0753	-0.31384	2.20078	-5.60567	0.29634	-2.304
116.1078	-0.31386	2.19313	-5.60534	0.29252	-2.31166
116.1403	-0.31386	2.18482	-5.61482	0.28798	-2.31801
116.1743	-0.3139	2.17838	-5.63317	0.28131	-2.32279
116.207	-0.31394	2.17654	-5.65028	0.27527	-2.32711
116.24	-0.31398	2.17612	-5.66932	0.27157	-2.32793
116.2727	-0.31404	2.1754	-5.68324	0.26726	-2.32859
116.306	-0.3141	2.17454	-5.69461	0.2638	-2.33308
116.3395	-0.31412	2.17327	-5.70078	0.26072	-2.33951
116.3718	-0.31412	2.17162	-5.70939	0.25544	-2.34844

116.4055	-0.31402	2.16914	-5.71306	0.25164	-2.35929
116.4374	-0.31388	2.16453	-5.72825	0.25017	-2.36943
116.4716	-0.31382	2.15624	-5.74606	0.24893	-2.3744
116.5054	-0.3138	2.14894	-5.76506	0.24691	-2.37674
116.5368	-0.31382	2.14529	-5.78393	0.24335	-2.37932
116.5698	-0.31388	2.14395	-5.79238	0.24172	-2.38288
116.6036	-0.31396	2.14375	-5.79864	0.24113	-2.38879
116.6367	-0.314	2.14283	-5.81034	0.24173	-2.39383
116.6696	-0.31404	2.14016	-5.81973	0.243-2.39715	
116.7023	-0.31404	2.13478	-5.8363	0.24117	-2.4
116.7358	-0.31402	2.13059	-5.8515	0.23878	-2.40521
116.7694	-0.31404	2.12882	-5.86228	0.2372	-2.41368
116.8014	-0.31404	2.12766	-5.87357	0.23589	-2.4228
116.8349	-0.31416	2.12432	-5.88671	0.23534	-2.42723
116.8678	-0.3145	2.11729	-5.89335	0.23448	-2.43012
116.9012	-0.31478	2.10786	-5.90353	0.23266	-2.4352
116.9344	-0.31488	2.10292	-5.91428	0.2306	-2.43898
116.9674	-0.3149	2.10105	-5.92768	0.22861	-2.44107
117.0005	-0.31488	2.09999	-5.94525	0.22644	-2.44514
117.034	-0.31482	2.09958	-5.96176	0.22396	-2.45055
117.0667	-0.31478	2.09797	-5.97043	0.22167	-2.45947
117.1003	-0.31478	2.09221	-5.97415	0.22078	-2.47421
117.1337	-0.31476	2.08579	-5.97731	0.22052	-2.47946
117.1657	-0.31476	2.07787	-5.98726	0.22015	-2.48307
117.1988	-0.31478	2.07104	-6.00998	0.21974	-2.48949
117.2324	-0.31488	2.06773	-6.03064	0.21859	-2.49382
117.2662	-0.31524	2.06566	-6.05331	0.21625	-2.49666
117.299	-0.31562	2.06358	-6.06218	0.21248	-2.50065
117.3318	-0.31568	2.06161	-6.06261	0.20754	-2.50331
117.3668	-0.3157	2.05948	-6.07068	0.20299	-2.50745

117.3994	-0.31574	2.05802	-6.08336	0.19966	-2.51714
117.4325	-0.3158	2.05678	-6.10298	0.19875	-2.52716
117.465	-0.31588	2.05399	-6.12807	0.19828	-2.53699
117.4985	-0.31594	2.04924	-6.14273	0.19778	-2.54307
117.5307	-0.31596	2.04481	-6.15318	0.19726	-2.54347
117.5645	-0.31594	2.04217	-6.15777	0.19601	-2.54508
117.5974	-0.31584	2.04002	-6.16568	0.19443	-2.54665
117.6312	-0.31578	2.03763	-6.17212	0.19158	-2.54817
117.6642	-0.31578	2.03428	-6.18947	0.18945	-2.55195
117.6977	-0.3158	2.02868	-6.19995	0.18809	-2.55882
117.7304	-0.31584	2.02442	-6.21348	0.18749	-2.56905
117.7633	-0.31588	2.02094	-6.22948	0.18697	-2.58261
117.7965	-0.31586	2.01746	-6.24233	0.1865	-2.59385
117.8298	-0.31584	2.0153	-6.25088	0.18556	-2.60005
117.863	-0.31582	2.01371	-6.26905	0.18313	-2.60281
117.8964	-0.3158	2.01211	-6.28257	0.17972	-2.60424
117.93	-0.3158	2.00866	-6.29482	0.17389	-2.60569
117.9633	-0.31578	2.00544	-6.3053	0.16921	-2.61165
117.997	-0.31578	2.00092	-6.31547	0.16742	-2.61956
118.0298	-0.3158	1.99486	-6.32688	0.16654	-2.62887
118.0619	-0.31586	1.98858	-6.33967	0.16596	-2.63656
118.0956	-0.31604	1.98379	-6.35541	0.16549	-2.64082
118.1281	-0.31642	1.97969	-6.3673	0.16501	-2.64685
118.1613	-0.31656	1.97934	-6.38064	0.16371	-2.65072
118.1941	-0.31656	1.97857	-6.39244	0.1607	-2.65491
118.2285	-0.31654	1.97568	-6.40286	0.15697	-2.65842
118.2622	-0.31652	1.96755	-6.416	0.15315	-2.66059
118.2952	-0.3165	1.95678	-6.42834	0.15007	-2.66849
118.3293	-0.3165	1.9517	-6.44045	0.14722	-2.67797
118.3615	-0.3165	1.95105	-6.45034	0.14557	-2.68896

118.3944	-0.3165	1.95077	-6.45726	0.14479	-2.69891
118.4271	-0.3165	1.94936	-6.46589	0.1445	-2.70329
118.4604	-0.31654	1.94483	-6.47926	0.14383	-2.7054
118.4941	-0.31658	1.93825	-6.49622	0.14107	-2.70985
118.5271	-0.31664	1.93285	-6.5156	0.13748	-2.71429
118.5596	-0.31668	1.92833	-6.53015	0.13323	-2.72424
118.5939	-0.31672	1.9271	-6.5392	0.12962	-2.73405
118.6273	-0.31672	1.92725	-6.54771	0.12764	-2.74004
118.6598	-0.31672	1.92737	-6.56584	0.12552	-2.7457
118.6932	-0.31672	1.92704	-6.58298	0.12398	-2.74772
118.7257	-0.31676	1.92469	-6.59601	0.1225	-2.74835
118.7588	-0.31678	1.91894	-6.60917	0.12064	-2.75398
118.7924	-0.3168	1.91136	-6.61574	0.11809	-2.76592
118.8251	-0.31684	1.90494	-6.62476	0.11393	-2.77821
118.8584	-0.31684	1.89877	-6.64119	0.1086	-2.7885
118.8915	-0.31688	1.89269	-6.64827	0.10221	-2.79507
118.9248	-0.31692	1.8895	-6.65674	0.09669	-2.79797
118.9581	-0.31694	1.88789	-6.66912	0.09385	-2.80547
118.9919	-0.31698	1.88442	-6.68238	0.09235	-2.81569
119.0241	-0.31704	1.87955	-6.7029	0.09031	-2.82773
119.0568	-0.31706	1.87299	-6.72109	0.08941	-2.83211
119.091	-0.31706	1.86701	-6.73215	0.08877	-2.83497
119.1239	-0.31706	1.86265	-6.74092	0.0882	-2.8374
119.157	-0.31704	1.86008	-6.74878	0.08784	-2.83861
119.1896	-0.31706	1.85599	-6.75778	0.08705	-2.84466
119.2228	-0.31708	1.85263	-6.77107	0.08456	-2.85446
119.2563	-0.31706	1.84866	-6.78666	0.07608	-2.8667
119.2901	-0.31706	1.84169	-6.8003	0.06772	-2.87684
119.323	-0.31708	1.83539	-6.8192	0.0626	-2.87935
119.3562	-0.3172	1.82967	-6.83068	0.06104	-2.88082

119.3891	-0.31744	1.82488	-6.83836	0.06106	-2.88478
119.4225	-0.31776	1.82006	-6.84827	0.06093	-2.89275
119.4558	-0.31786	1.81488	-6.8541	0.05909	-2.90376
119.489	-0.3179	1.80926	-6.86293	0.05598	-2.91499
119.5222	-0.31788	1.80576	-6.8815	0.05203	-2.92326
119.5549	-0.31788	1.80324	-6.90131	0.04887	-2.93371
119.5881	-0.31786	1.80155	-6.91913	0.04265	-2.94602
119.6209	-0.3179	1.8001	-6.93818	0.03672	-2.95002
119.6542	-0.31792	1.79678	-6.9435	0.03066	-2.95228
119.6876	-0.318	1.7915	-6.94618	0.02537	-2.95597
119.7206	-0.31804	1.7841	-6.95626	0.02364	-2.95789
119.7541	-0.3181	1.77875	-6.97048	0.02299	-2.96779
119.7871	-0.31818	1.77537	-6.98835	0.02165	-2.98051
119.8204	-0.31828	1.77303	-7.00903	0.01817	-2.98834
119.8538	-0.31834	1.77023	-7.02128	0.01136	-2.99552
119.887	-0.3184	1.76677	-7.03537	9.8758E-4	-3.00373
119.92	-0.31844	1.76299	-7.04554	-0.00508	-3.01264
119.9532	-0.31844	1.75976	-7.0568	-0.00736	-3.03045
119.9862	-0.31844	1.75636	-7.0689	-0.00885	-3.04132
120.0186	-0.31846	1.75314	-7.07978	-0.0105	-3.05088
120.0516	-0.3185	1.74811	-7.09329	-0.01291	-3.04766
120.0846	-0.31858	1.74434	-7.10661	-0.01493	-3.05379
120.1179	-0.31866	1.73973	-7.11916	-0.01619	-3.06081
120.152	-0.31868	1.73361	-7.12985	-0.02018	-3.06465
120.1849	-0.3187	1.73059	-7.14323	-0.03013	-3.07563
120.2166	-0.3187	1.72828	-7.16203	-0.04045	-3.08526
120.2502	-0.3187	1.72531	-7.17441	-0.04372	-3.09911
120.2841	-0.31872	1.72346	-7.18465	-0.04445	-3.11217
120.3167	-0.31874	1.71871	-7.1938	-0.04476	-3.11871
120.3503	-0.31876	1.71198	-7.20208	-0.04743	-3.12082

120.3827	-0.31876	1.70488	-7.21759	-0.05439	-3.12511
120.4159	-0.31874	1.69804	-7.23524	-0.06002	-3.14252
120.4491	-0.31874	1.69451	-7.24305	-0.06401	-3.15693
120.4826	-0.31878	1.69298	-7.25471	-0.06547	-3.16651
120.5153	-0.3189	1.68933	-7.27232	-0.06813	-3.17316
120.5487	-0.31902	1.68318	-7.28528	-0.07414	-3.17404
120.5816	-0.31904	1.67341	-7.29773	-0.0823	-3.17842
120.6152	-0.31908	1.6676	-7.31489	-0.08926	-3.18988
120.648	-0.3191	1.66395	-7.328	-0.09455	-3.19958
120.6809	-0.31912	1.66187	-7.34068	-0.09618	-3.20924
120.7142	-0.31916	1.65808	-7.3512	-0.09873	-3.22191
120.7464	-0.31918	1.64948	-7.36348	-0.10146	-3.23493
120.7796	-0.31914	1.64267	-7.3839	-0.10436	-3.24919
120.8132	-0.31912	1.63986	-7.39891	-0.10905	-3.26072
120.8461	-0.31912	1.63789	-7.41298	-0.11413	-3.27257
120.8789	-0.3192	1.63775	-7.42483	-0.12188	-3.28315
120.9128	-0.31934	1.637	-7.43392	-0.13141	-3.2959
120.9457	-0.31952	1.63464	-7.44562	-0.13776	-3.30566
120.9779	-0.31956	1.62972	-7.46179	-0.14455	-3.31388
121.011	-0.31954	1.62232	-7.47891	-0.15276	-3.31984
121.0439	-0.31952	1.61381	-7.49457	-0.15643	-3.33162
121.0774	-0.31948	1.6061	-7.50867	-0.16258	-3.34378
121.11	-0.31944	1.59916	-7.52129	-0.1675	-3.35437
121.1433	-0.31944	1.58742	-7.53317	-0.17148	-3.363
121.1768	-0.3195	1.5749	-7.54568	-0.17387	-3.37079
121.2091	-0.31958	1.56773	-7.55957	-0.17569	-3.38301
121.242	-0.31964	1.56534	-7.57696	-0.17979	-3.396
121.2756	-0.31972	1.56671	-7.59126	-0.18671	-3.40674
121.307	-0.31976	1.56851	-7.6086	-0.19417	-3.41468
121.3406	-0.3198	1.56835	-7.62452	-0.20127	-3.41825

121.3748	-0.31984	1.56471	-7.63777	-0.20524	-3.42631
121.4076	-0.31984	1.555	-7.64426	-0.2084	-3.42747
121.4411	-0.31984	1.5355	-7.65218	-0.21408	-3.43761
121.4734	-0.31986	1.52356	-7.66989	-0.21888	-3.4529
121.5073	-0.3199	1.52233	-7.68957	-0.22431	-3.46752
121.5399	-0.31994	1.52328	-7.70396	-0.23074	-3.48265
121.5728	-0.31998	1.52407	-7.71918	-0.23773	-3.48787
121.6051	-0.32	1.524	-7.73043	-0.24559	-3.49187
121.6386	-0.32002	1.52095	-7.74146	-0.25336	-3.50753
121.6714	-0.32004	1.5179	-7.75479	-0.25617	-3.51853
121.7052	-0.32004	1.51402	-7.76536	-0.26075	-3.534
121.7374	-0.32006	1.50756	-7.77984	-0.26706	-3.54346
121.7709	-0.3201	1.49851	-7.79804	-0.27443	-3.54599
121.803	-0.32014	1.49156	-7.81854	-0.28431	-3.55578
121.8369	-0.32018	1.48397	-7.841	-0.29158	-3.56872
121.8699	-0.32022	1.47573	-7.8558	-0.29554	-3.58321
121.9032	-0.32026	1.47076	-7.86733	-0.29829	-3.6022
121.9366	-0.32032	1.46932	-7.87537	-0.30273	-3.62379
121.9693	-0.32034	1.46678	-7.88785	-0.30744	-3.63591
122.0024	-0.32036	1.46358	-7.90562	-0.31622	-3.64989
122.0347	-0.3204	1.45922	-7.92253	-0.321	-3.66456
122.0682	-0.32042	1.45206	-7.94402	-0.32367	-3.67727
122.1007	-0.32044	1.44364	-7.96173	-0.32763	-3.69805
122.1333	-0.32044	1.43457	-7.97526	-0.33379	-3.72104
122.1668	-0.32046	1.42426	-7.98948	-0.34478	-3.74574
122.2	-0.3205	1.41737	-7.99791	-0.35838	-3.77366
122.2328	-0.32058	1.41186	-8.0107	-0.36421	-3.79442
122.2655	-0.3208	1.4076	-8.02973	-0.37059	-3.81323
122.2982	-0.32096	1.40449	-8.04795	-0.37793	-3.82913
122.3314	-0.32102	1.39936	-8.07225	-0.38103	-3.84132

122.3646	-0.32106	1.39457	-8.08913	-0.38917	-3.85537
122.3975	-0.32108	1.39095	-8.10221	-0.3979	-3.87196
122.4302	-0.3211	1.38413	-8.11948	-0.40934	-3.88269
122.4623	-0.32112	1.37655	-8.14026	-0.42026	-3.88921
122.496	-0.32114	1.37008	-8.15664	-0.42861	-3.89662
122.5292	-0.32118	1.36647	-8.17394	-0.43789	-3.89738
122.5628	-0.3213	1.36461	-8.18314	-0.4488	-3.9003
122.595	-0.32166	1.36124	-8.19682	-0.4618	-3.89742
122.6276	-0.32194	1.35484	-8.22211	-0.48072	-3.892
122.66	-0.32196	1.34714	-8.24382	-0.4978	-3.89261
122.693	-0.32198	1.34081	-8.25997	-0.51095	-3.89451
122.7262	-0.32202	1.33775	-8.27506	-0.52131	-3.89313
122.759	-0.32206	1.33698	-8.28783	-0.52975	-3.89196
122.7916	-0.3221	1.33501	-8.30642	-0.53674	-3.887
122.8246	-0.32212	1.33081	-8.32846	-0.54557	-3.88248
122.857	-0.32212	1.32317	-8.34736	-0.55011	-3.88877
122.8904	-0.32212	1.31214	-8.36309	-0.54738	-3.89296
122.924	-0.32218	1.30444	-8.37907	-0.54333	-3.89133
122.9565	-0.32222	1.29997	-8.39702	-0.54488	-3.88473
122.9899	-0.32224	1.29647	-8.41435	-0.55791	-3.88556
123.0217	-0.32226	1.29496	-8.43259	-0.56849	-3.8886
123.0547	-0.32224	1.29083	-8.44875	-0.57853	-3.89397
123.0869	-0.32228	1.28539	-8.46841	-0.58503	-3.89971
123.1199	-0.32234	1.27979	-8.49008	-0.58945	-3.90172
123.1532	-0.32236	1.27363	-8.5097	-0.59868	-3.90206
123.186	-0.32236	1.26966	-8.53051	-0.60836	-3.90393
123.2196	-0.32236	1.26447	-8.54933	-0.6158	-3.90896
123.2515	-0.32238	1.26129	-8.56763	-0.62406	-3.9178
123.2848	-0.32252	1.25757	-8.59369	-0.62957	-3.92756
123.3179	-0.32274	1.25386	-8.6186	-0.6355	-3.93364

123.35	-0.32284	1.2496	-8.63785	-0.64205	-3.93871
123.3838	-0.3229	1.24622	-8.66166	-0.64507	-3.94692
123.4165	-0.32298	1.23666	-8.68098	-0.65275	-3.95106
123.4498	-0.32304	1.22694	-8.69584	-0.6639	-3.95644
123.481	-0.32306	1.22062	-8.71237	-0.67505	-3.96725
123.5142	-0.32306	1.21646	-8.72868	-0.6867	-3.9827
123.548	-0.32308	1.21184	-8.74939	-0.70186	-4.00272
123.58	-0.32312	1.20964	-8.77566	-0.70945	-4.02386
123.6133	-0.3232	1.20715	-8.80212	-0.72362	-4.03457
123.6454	-0.32328	1.2046	-8.81824	-0.73646	-4.04422
123.6791	-0.32328	1.20199	-8.83456	-0.74307	-4.04795
123.7123	-0.3233	1.1981	-8.85635	-0.74948	-4.0515
123.7435	-0.32332	1.1902	-8.8746	-0.75676	-4.05514
123.7769	-0.32336	1.18069	-8.89274	-0.76243	-4.05785
123.8106	-0.32346	1.17342	-8.91315	-0.77161	-4.06823
123.8426	-0.32356	1.17086	-8.9309	-0.78051	-4.0759
123.8758	-0.3236	1.1683	-8.95082	-0.79161	-4.07878
123.9077	-0.32366	1.16659	-8.96947	-0.80647	-4.09213
123.9409	-0.32372	1.16121	-8.9797	-0.82011	-4.10636
123.9744	-0.32378	1.15463	-9.00466	-0.83633	-4.12435
124.0056	-0.32384	1.15207	-9.03149	-0.84561	-4.14209
124.039	-0.32386	1.15196	-9.0549	-0.85496	-4.15376
124.0715	-0.32388	1.15273	-9.07796	-0.86263	-4.17943
124.105	-0.32394	1.15232	-9.09332	-0.87392	-4.21028
124.1381	-0.32402	1.14811	-9.10627	-0.88415	-4.23822
124.1698	-0.32408	1.14233	-9.12687	-0.89839	-4.2593
124.2035	-0.32414	1.13641	-9.15235	-0.91221	-4.27318
124.2362	-0.32418	1.13335	-9.17333	-0.92212	-4.28703
124.2682	-0.32424	1.13328	-9.19986	-0.93339	-4.29801
124.3016	-0.32428	1.13054	-9.22071	-0.9425	-4.30873

124.3339	-0.32436	1.1286	-9.24348	-0.94708	-4.30954
124.3657	-0.3244	1.12747	-9.27638	-0.95706	-4.30363
124.3994	-0.32442	1.12577	-9.3023	-0.97232	-4.29546
124.432	-0.32446	1.12565	-9.32624	-0.98819	-4.28964
124.4649	-0.32454	1.12468	-9.35012	-1.0019	-4.28626
124.4974	-0.32462	1.12217	-9.37274	-1.01376	-4.29019
124.5298	-0.32474	1.12087	-9.39807	-1.03172	-4.30032
124.5616	-0.32482	1.11917	-9.42768	-1.04572	-4.31215
124.5946	-0.32492	1.11783	-9.45096	-1.0655	-4.32617
124.6283	-0.32498	1.11692	-9.46798	-1.08077	-4.34459
124.6607	-0.325	1.11571	-9.47996	-1.09156	-4.36283
124.6932	-0.325	1.11476	-9.4909	-1.10908	-4.38506
124.7267	-0.325	1.11434	-9.50368	-1.1256	-4.41707
124.7586	-0.32502	1.11399	-9.52222	-1.1409	-4.43555
124.7913	-0.32506	1.11406	-9.54001	-1.1591	-4.45558
124.8234	-0.32514	1.1142	-9.56036	-1.16491	-4.47087
124.8564	-0.32528	1.11413	-9.58318	-1.18068	-4.47851
124.8893	-0.32552	1.11373	-9.60615	-1.19999	-4.48174
124.9217	-0.32564	1.11263	-9.6326	-1.21985	-4.47499
124.9539	-0.32572	1.11151	-9.65568	-1.24168	-4.46486
124.987	-0.32576	1.11009	-9.67791	-1.26198	-4.43943
125.0196	-0.3258	1.10956	-9.69686	-1.27855	-4.41148
125.0514	-0.32586	1.10949	-9.71838	-1.29196	-4.39768
125.0832	-0.32592	1.10963	-9.74569	-1.30651	-4.37365
125.1161	-0.32598	1.11025	-9.77708	-1.32597	-4.3455
125.1489	-0.32604	1.11112	-9.81185	-1.3529	-4.32066
125.1816	-0.32612	1.11138	-9.84172	-1.37552	-4.29183
125.2138	-0.3262	1.1115	-9.87022	-1.39811	-4.27095
125.246	-0.3263	1.11201	-9.8965	-1.41211	-4.24614
125.2782	-0.3264	1.11307	-9.92968	-1.42877	-4.22237

125.3108	-0.32644	1.11544	-9.96818	-1.44569	-4.19772
125.3431	-0.3265	1.12009	-10.00017	-1.47134	-4.17707
125.3755	-0.32654	1.12462	-10.02796	-1.4949	-4.1533
125.4074	-0.32656	1.12667	-10.05593	-1.51969	-4.13634
125.4396	-0.32662	1.12969	-10.07213	-1.54133	-4.1175
125.4717	-0.32668	1.13227	-10.08399	-1.56618	-4.10141
125.5039	-0.32674	1.13614	-10.09289	-1.58602	-4.09399
125.5364	-0.32698	1.14635	-10.09654	-1.61481	-4.08314
125.5677	-0.32728	1.16005	-10.10312	-1.64346	-4.07599
125.6011	-0.3274	1.1733	-10.11919	-1.66507	-4.06717
125.6331	-0.32744	1.18484	-10.14847	-1.67428	-4.04351
125.6654	-0.32744	1.1926	-10.1869	-1.68074	-4.01802
125.6974	-0.32742	1.1982	-10.22421	-1.68479	-3.99376
125.7293	-0.32746	1.20441	-10.25401	-1.70073	-3.96099
125.7623	-0.32754	1.2088	-10.27662	-1.72119	-3.92992
125.7952	-0.32762	1.21164	-10.28877	-1.73445	-3.90246
125.8264	-0.32774	1.21489	-10.30216	-1.73956	-3.87575
125.859	-0.32788	1.21849	-10.31152	-1.7434	-3.85813
125.8917	-0.32806	1.22231	-10.31955	-1.74942	-3.84431
125.9238	-0.32834	1.22639	-10.32759	-1.75458	-3.83484
125.9561	-0.32852	1.23185	-10.33353	-1.76143	-3.82899
125.9892	-0.32862	1.24132	-10.33455	-1.76134	-3.81862
126.0212	-0.3287	1.24935	-10.33585	-1.75608	-3.81261
126.0532	-0.32874	1.25748	-10.33036	-1.76129	-3.803
126.0859	-0.3288	1.26576	-10.32072	-1.76538	-3.79084
126.1181	-0.32882	1.27625	-10.30688	-1.77291	-3.78129
126.1515	-0.32886	1.28751	-10.30252	-1.78145	-3.7774
126.1841	-0.32892	1.30122	-10.29974	-1.78783	-3.77123
126.2163	-0.32906	1.31853	-10.29937	-1.79442	-3.76186
126.2496	-0.32934	1.33735	-10.29462	-1.79686	-3.75074

126.2818	-0.32988	1.3555	-10.28361	-1.79688	-3.74329
126.3137	-0.33014	1.37113	-10.2785	-1.79885	-3.74059
126.3471	-0.33022	1.38475	-10.27232	-1.79759	-3.73949
126.379	-0.3303	1.4008	-10.26401	-1.7972	-3.73974
126.4125	-0.33036	1.41435	-10.2405	-1.79807	-3.73774
126.4456	-0.33042	1.43112	-10.21293	-1.79712	-3.73711
126.4778	-0.33048	1.44808	-10.18332	-1.78909	-3.73569
126.5111	-0.33052	1.46688	-10.15092	-1.78563	-3.72551
126.5434	-0.33058	1.49094	-10.11356	-1.78056	-3.7122
126.5762	-0.33068	1.50508	-10.06385	-1.77585	-3.6959
126.6081	-0.33098	1.5136	-10.00389	-1.77395	-3.68022
126.6421	-0.33118	1.52343	-9.94695	-1.77126	-3.66472
126.6753	-0.33128	1.5391	-9.8792	-1.76519	-3.64614
126.7075	-0.33136	1.56331	-9.81679	-1.75445	-3.62911
126.7414	-0.3314	1.58804	-9.75703	-1.73583	-3.61514
126.7742	-0.33144	1.59961	-9.69417	-1.71246	-3.60597
126.8079	-0.33152	1.60861	-9.63287	-1.68813	-3.59159
126.8412	-0.33162	1.62109	-9.572	-1.65898	-3.57708
126.8736	-0.33186	1.63671	-9.50244	-1.63528	-3.56592
126.9079	-0.332	1.66929	-9.43702	-1.61398	-3.55173
126.9415	-0.33206	1.69632	-9.36813	-1.59748	-3.53943
126.9744	-0.3321	1.71561	-9.29648	-1.57754	-3.52473
127.0081	-0.33218	1.72829	-9.22304	-1.55513	-3.50884
127.0412	-0.33226	1.73863	-9.14697	-1.53073	-3.48272
127.0752	-0.33244	1.75069	-9.06916	-1.50314	-3.44754
127.1086	-0.33254	1.77347	-8.99831	-1.48612	-3.41532
127.143	-0.33262	1.79366	-8.91979	-1.46858	-3.38056
127.1766	-0.33268	1.81479	-8.83298	-1.45053	-3.34526
127.2109	-0.33278	1.83621	-8.73613	-1.42736	-3.30314
127.2442	-0.333	1.85719	-8.62293	-1.40966	-3.2444

127.2765	-0.33346	1.88113	-8.51195	-1.3898	-3.17344
127.3104	-0.3338	1.90272	-8.40301	-1.37232	-3.09687
127.3448	-0.334	1.92142	-8.29271	-1.35338	-3.02421
127.3789	-0.33408	1.94263	-8.17252	-1.32846	-2.95304
127.4129	-0.33416	1.96303	-8.05231	-1.30369	-2.89247
127.4477	-0.33424	1.98967	-7.9288	-1.28654	-2.85163
127.4804	-0.33428	2.01646	-7.80455	-1.27116	-2.80927
127.5152	-0.33434	2.04713	-7.68516	-1.25839	-2.75918
127.5501	-0.33444	2.06676	-7.55286	-1.23808	-2.70161
127.5834	-0.33476	2.08458	-7.42054	-1.20631	-2.63333
127.6175	-0.33498	2.10793	-7.29488	-1.16538	-2.56882
127.6516	-0.33508	2.1283	-7.16696	-1.12191	-2.51294
127.6856	-0.33516	2.15495	-7.04437	-1.08484	-2.4604
127.7204	-0.33528	2.18128	-6.91312	-1.05586	-2.40384
127.7538	-0.33538	2.20777	-6.77743	-1.02612	-2.35471
127.7888	-0.33554	2.2332	-6.64362	-0.99492	-2.30304
127.823	-0.33566	2.25978	-6.50718	-0.95168	-2.25726
127.8586	-0.3358	2.29131	-6.38286	-0.90407	-2.21485
127.8922	-0.3363	2.31752	-6.25824	-0.85538	-2.17436
127.9257	-0.33678	2.33854	-6.13697	-0.82967	-2.13447
127.9609	-0.33702	2.36568	-6.02968	-0.79861	-2.0923
127.9952	-0.33714	2.39148	-5.92462	-0.76593	-2.0527
128.0306	-0.33722	2.42142	-5.82745	-0.73029	-2.01165
128.0645	-0.33726	2.45327	-5.73851	-0.6886	-1.97437
128.1	-0.33736	2.47834	-5.64839	-0.65653	-1.93954
128.1352	-0.3375	2.50721	-5.56285	-0.62579	-1.91303
128.1692	-0.33764	2.5287	-5.48383	-0.58607	-1.88677
128.2039	-0.3379	2.54984	-5.39872	-0.55252	-1.8573
128.2391	-0.3381	2.5715	-5.32234	-0.5212	-1.83363
128.2747	-0.33822	2.59073	-5.25421	-0.5004	-1.81189

128.3094	-0.33834	2.61005	-5.18604	-0.48592	-1.79022
128.3448	-0.33844	2.62878	-5.1224	-0.46267	-1.77005
128.3794	-0.33854	2.64669	-5.05536	-0.43593	-1.74169
128.4135	-0.33866	2.67137	-4.98831	-0.41723	-1.71553
128.4476	-0.33876	2.69244	-4.92636	-0.39673	-1.69584
128.4831	-0.3389	2.71065	-4.87206	-0.3756	-1.67452
128.5181	-0.33904	2.72291	-4.81602	-0.35433	-1.65534
128.553	-0.33916	2.7354	-4.76093	-0.32531	-1.63313
128.5874	-0.33926	2.74817	-4.70968	-0.28579	-1.60799
128.6226	-0.33934	2.76694	-4.66036	-0.24626	-1.58802
128.6565	-0.33946	2.78211	-4.61303	-0.21118	-1.56917
128.692	-0.3396	2.79832	-4.56791	-0.1706	-1.55108
128.7275	-0.33982	2.81596	-4.51856	-0.13718	-1.53556
128.7619	-0.34004	2.83374	-4.47772	-0.10312	-1.51714
128.7971	-0.34016	2.83731	-4.43829	-0.06701	-1.49813
128.8322	-0.34026	2.84563	-4.40554	-0.02384	-1.48319
128.8674	-0.34034	2.85009	-4.36573	0.02047	-1.46583
128.9032	-0.34044	2.85655	-4.32505	0.06543	-1.44897
128.9374	-0.34056	2.86823	-4.27879	0.11035	-1.43203
128.9734	-0.3407	2.88274	-4.24059	0.14732	-1.42212
129.0079	-0.34084	2.88867	-4.20878	0.17632	-1.41413
129.0436	-0.341	2.89662	-4.17634	0.20795	-1.41132
129.0782	-0.3412	2.90641	-4.14682	0.23808	-1.39868
129.1136	-0.34136	2.91003	-4.11193	0.26365	-1.38111
129.1494	-0.3415	2.92403	-4.0785	0.28622	-1.36277
129.1838	-0.34164	2.93462	-4.04983	0.29717	-1.34572
129.2197	-0.34176	2.94556	-4.01924	0.29282	-1.33635
129.2539	-0.34188	2.95399	-3.98685	0.27798	-1.33362
129.289	-0.342	2.9625	-3.96154	0.25205	-1.33042
129.3233	-0.3421	2.96578	-3.9321	0.23166	-1.33353

129.3586	-0.3422	2.97232	-3.90284	0.2213	-1.33975
129.3923	-0.3424	2.97676	-3.87602	0.22587	-1.33576
129.4278	-0.34296	2.9794	-3.84438	0.24199	-1.32651
129.4626	-0.34362	2.98421	-3.81641	0.25852	-1.31949
129.4967	-0.34386	2.98947	-3.78797	0.26027	-1.30934
129.5306	-0.34398	2.99372	-3.76022	0.26693	-1.30112
129.5655	-0.34406	3.0012	-3.72967	0.27351	-1.29433
129.6004	-0.34414	3.00683	-3.70791	0.28546	-1.28318
129.6354	-0.34424	3.01009	-3.68601	0.3093	-1.27478
129.6691	-0.34436	3.01091	-3.66325	0.33332	-1.26988
129.704	-0.34448	3.01567	-3.63777	0.34524	-1.26604
129.738	-0.3446	3.02344	-3.61132	0.3488	-1.26402
129.7715	-0.34474	3.03254	-3.5834	0.3535	-1.26212
129.8062	-0.34488	3.03947	-3.56613	0.36619	-1.25839
129.8418	-0.34498	3.04363	-3.54766	0.3758	-1.25729
129.876	-0.34506	3.04369	-3.52241	0.38956	-1.25508
129.9098	-0.34512	3.04875	-3.50362	0.3969	-1.25304
129.9442	-0.34516	3.05291	-3.48549	0.39588	-1.24962
129.9784	-0.3452	3.05795	-3.45984	0.39483	-1.24161
130.0127	-0.34524	3.06269	-3.43463	0.40244	-1.23221
130.0476	-0.34526	3.06802	-3.41365	0.4165	-1.23273
130.0817	-0.3453	3.0713	-3.39559	0.43344	-1.25533
130.1152	-0.34534	3.07424	-3.38185	0.45016	-1.26799
130.1506	-0.3454	3.07585	-3.36408	0.45344	-1.27192
130.1839	-0.34544	3.07906	-3.33951	0.4491	-1.26862
130.2182	-0.34546	3.08056	-3.31815	0.45215	-1.26551
130.2528	-0.34552	3.08405	-3.30391	0.45944	-1.26115
130.2869	-0.34562	3.08761	-3.29094	0.47859	-1.25866
130.321	-0.34574	3.09221	-3.27612	0.49122	-1.25728
130.3546	-0.34584	3.10172	-3.25254	0.49259	-1.25487

130.3882	-0.34588	3.10825	-3.23107	0.49781	-1.25145
130.4231	-0.34592	3.10999	-3.20769	0.49934	-1.24636
130.4569	-0.346	3.1115	-3.19276	0.49771	-1.23909
130.4914	-0.34608	3.11247	-3.1809	0.49715	-1.23399
130.5249	-0.34612	3.11334	-3.16963	0.49404	-1.23211
130.5596	-0.34616	3.11736	-3.15715	0.48523	-1.23084
130.594	-0.3462	3.12018	-3.1384	0.4817	-1.23049
130.6273	-0.34626	3.12131	-3.11995	0.49106	-1.23008
130.6622	-0.3464	3.12285	-3.10407	0.50166	-1.22862
130.6946	-0.34666	3.12388	-3.08992	0.50412	-1.22716
130.7295	-0.3468	3.12447	-3.0802	0.49784	-1.2259
130.7621	-0.34696	3.12558	-3.06612	0.4877	-1.22347
130.7968	-0.34712	3.12832	-3.04748	0.48792	-1.21905
130.8309	-0.3474	3.13389	-3.03505	0.49262	-1.21251
130.8642	-0.34862	3.1409	-3.02658	0.50134	-1.20568
130.898	-0.35026	3.14451	-3.01752	0.50586	-1.20357
130.9318	-0.35138	3.14576	-3.00766	0.50816	-1.20278
130.966	-0.3525	3.14697	-2.98985	0.51042	-1.20122
130.9986	-0.35362	3.1476	-2.97309	0.51364	-1.19821
131.0322	-0.3548	3.14914	-2.95901	0.51669	-1.19535
131.0671	-0.3566	3.15067	-2.94882	0.52002	-1.19257
131.1	-0.36172	3.15155	-2.93924	0.52155	-1.18988
131.1344	-0.37086	3.15242	-2.92999	0.52201	-1.18576
131.1677	-0.38508	3.15272	-2.91861	0.52316	-1.17866
131.2014	-0.39552	3.15294	-2.90626	0.52636	-1.17261
131.2365	-0.39892	3.15473	-2.89233	0.53046	-1.16865
131.2684	-0.39856	3.15939	-2.88079	0.5333	-1.16612
131.3028	-0.39992	3.16524	-2.87078	0.53444	-1.16517
131.3361	-0.40492	3.16834	-2.86475	0.53411	-1.16454
131.3696	-0.41362	3.16887	-2.85589	0.53326	-1.16344

131.4033	-0.42132	3.16858	-2.84106	0.53337	-1.16287
131.4375	-0.42642	3.16887	-2.82502	0.53318	-1.16129
131.4705	-0.43138	3.1699	-2.81532	0.53325	-1.1583
131.504	-0.4356	3.17196	-2.8055	0.53334	-1.15538
131.5374	-0.44016	3.17538	-2.79959	0.53293	-1.15108
131.5722	-0.44236	3.17679	-2.79616	0.53226	-1.14775
131.605	-0.44312	3.17753	-2.78985	0.5303	-1.14668
131.638	-0.44328	3.17817	-2.7877	0.52831	-1.14674
131.6725	-0.44462	3.17874	-2.77634	0.52588	-1.14761
131.7059	-0.44436	3.17927	-2.76573	0.52187	-1.14876
131.7388	-0.44342	3.1796	-2.75424	0.51457	-1.15154
131.7728	-0.44246	3.1803	-2.7371	0.50639	-1.15551
131.8062	-0.44182	3.18161	-2.73784	0.50195	-1.16152
131.8398	-0.44148	3.18488	-2.73536	0.50147	-1.16891
131.873	-0.44138	3.18769	-2.72604	0.50204	-1.17194
131.9068	-0.43966	3.19035	-2.70999	0.50681	-1.17231
131.94	-0.43798	3.19177	-2.7023	0.51785	-1.17239
131.9729	-0.437	3.19217	-2.69749	0.53325	-1.17232
132.0071	-0.43612	3.1927	-2.69647	0.53334	-1.17223
132.041	-0.43548	3.19308	-2.69115	0.53293	-1.17219
132.0745	-0.43494	3.19396	-2.67538	0.53226	-1.17219
132.1083	-0.43432	3.19517	-2.66413	0.5303	-1.17212
132.1417	-0.43366	3.19546	-2.66077	0.52831	-1.17199
132.1745	-0.43308	3.19608	-2.65987	0.52588	-1.17141
132.2079	-0.43228	3.19762	-2.65682	0.52187	-1.16982
132.2411	-0.43152	3.20038	-2.65027	0.51457	-1.1685
132.2759	-0.43058	3.20392	-2.6419	0.50639	-1.16779
132.3101	-0.42978	3.20586	-2.63714	0.50195	-1.16687
132.3423	-0.42874	3.2067	-2.63461	0.50147	-1.16607
132.3762	-0.4271	3.20724	-2.62934	0.50204	-1.16437

132.4096	-0.425	3.20775	-2.6208	0.50681	-1.16242
132.4427	-0.4226	3.20796	-2.61411	0.51785	-1.16044
132.4758	-0.4215	3.20932	-2.61151	0.52187	-1.15912
132.5095	-0.42094	3.21039	-2.60977	0.53404	-1.15772
132.5419	-0.4206	3.21086	-2.60408	0.53404	-1.15623
132.5754	-0.42026	3.21129	-2.59044	0.5303	-1.15572
132.6089	-0.41984	3.21141	-2.5774	0.49122	-1.15542
132.6432	-0.41884	3.21138	-2.57002	0.49259	-1.15537
132.6758	-0.41608	3.21113	-2.56917	0.49781	-1.15518
132.7088	-0.41304	3.20396	-2.5645	0.49934	-1.15455
132.7412	-0.41176	3.13482	-2.56126	0.49771	-1.15363
132.7711	-0.41152	3.03776	-2.56176	0.49715	-1.15325
132.7975	-0.41154	2.93915	-2.5625	0.49404	-1.15311
132.8157	-0.4116	2.83989	-2.56336	0.49362	-1.15308
132.8263	-0.41166	2.74876	-2.56323	0.49266	-1.15302
132.8412	-0.4117	2.67172	-2.55964	0.49306	-1.15292
132.8636	-0.41172	2.61775	-2.55375	0.49936	-1.15255
132.8939	-0.41178	2.58893	-2.54596	0.50706	-1.15157
132.9264	-0.41182	2.57024	-2.53422	0.50851	-1.15082
132.9591	-0.41184	2.56048	-2.52408	0.50856	-1.15039
132.9931	-0.41186	2.55561	-2.51948	0.49122	-1.15027
133.0276	-0.41188	2.55994	-2.51939	0.49259	-1.1502
133.0633	-0.4119	2.57905	-2.51892	0.49781	-1.15011
133.0976	-0.41198	2.59504	-2.51893	0.49934	-1.15001
133.133	-0.41206	2.60954	-2.51636	0.49771	-1.14989
133.1674	-0.41216	2.61793	-2.51262	0.49715	-1.14973
133.2034	-0.41222	2.63022	-2.50567	0.49404	-1.14954
133.2385	-0.41226	2.65581	-2.49979	0.49936	-1.14938
133.274	-0.41232	2.67879	-2.495	0.50706	-1.14918
133.3098	-0.41238	2.69672	-2.49243	0.50851	-1.14902

133.344	-0.41246	2.71355	-2.49105	0.50856	-1.14888
133.3792	-0.41254	2.72214	-2.49026	0.50972	-1.14879
133.4137	-0.41258	2.73988	-2.48799	0.49122	-1.14871
133.449	-0.41266	2.76343	-2.48629	0.49259	-1.14865
133.4839	-0.41274	2.78055	-2.48576	0.49781	-1.14853
133.5195	-0.41282	2.79064	-2.4846	0.49934	-1.1484
133.5551	-0.41288	2.7974	-2.48419	0.49771	-1.1483
133.5898	-0.41296	2.80394	-2.48319	0.49715	-1.14819
133.6256	-0.413	2.82046	-2.4804	0.36259	-1.14805
133.66	-0.41308	2.83989	-2.47773	0.37747	-1.14794
133.6953	-0.41316	2.85394	-2.4752	0.38834	-1.14784
133.7302	-0.41322	2.8658	-2.4712	0.38307	-1.14776
133.7656	-0.41328	2.87796	-2.46775	0.38532	-1.14768
133.8005	-0.41338	2.89154	-2.46412	0.39127	-1.14753
133.8336	-0.41354	2.90141	-2.46124	0.40208	-1.14739
133.8684	-0.4139	2.91069	-2.45745	0.41882	-1.14722
133.9028	-0.41418	2.91016	-2.45557	0.42919	-1.14705
133.9378	-0.41436	2.91623	-2.45493	0.42695	-1.14688
133.9732	-0.41454	2.93076	-2.4543	0.42477	-1.14673
134.0089	-0.41488	2.93618	-2.45329	0.42488	-1.14663
134.0419	-0.41538	2.93969	-2.45088	0.42944	-1.1466
134.0766	-0.41596	2.94153	-2.44827	0.43757	-1.14666
134.1111	-0.41656	2.94601	-2.44637	0.44607	-1.14669
134.1451	-0.4178	2.95751	-2.44563	0.45405	-1.14668
134.1793	-0.4191	2.96773	-2.44494	0.4601	-1.14661
134.2124	-0.42052	2.97474	-2.44372	0.45684	-1.14648
134.2466	-0.42182	2.98145	-2.44164	0.45812	-1.14634
134.2816	-0.42298	2.98234	-2.43892	0.46009	-1.14629
134.3164	-0.42374	2.98539	-2.436	0.46539	-1.1463
134.3516	-0.4243	2.98627	-2.43405	0.47276	-1.14634

134.3853	-0.42484	2.98879	-2.43178	0.47552	-1.14635
134.4191	-0.42572	2.99233	-2.43015	0.47291	-1.14627
134.4533	-0.42698	2.99919	-2.42777	0.47829	-1.14611
134.4877	-0.43004	3.00382	-2.42566	0.48739	-1.14597
134.5214	-0.43304	3.00729	-2.42464	0.493-1.14585	
134.5549	-0.43514	3.01679	-2.42398	0.49362	-1.14578
134.5889	-0.43724	3.02497	-2.42317	0.49266	-1.14574
134.6234	-0.43864	3.02882	-2.42188	0.49306	-1.14572
134.6582	-0.43934	3.03415	-2.41913	0.49936	-1.14574
134.6925	-0.44004	3.03562	-2.41657	0.50706	-1.14576
134.7259	-0.4408	3.03634	-2.41443	0.50851	-1.14575
134.7596	-0.44212	3.04112	-2.41216	0.50856	-1.14569
134.7934	-0.44386	3.04749	-2.4113	0.50972	-1.14556
134.8272	-0.44564	3.05498	-2.41026	0.51119	-1.14549
134.8606	-0.4468	3.05974	-2.40918	0.51507	-1.14549
134.895	-0.44774	3.05976	-2.4085	0.51831	-1.1455
134.9288	-0.4481	3.05909	-2.40806	0.51969	-1.14548
134.963	-0.4484	3.06016	-2.40788	0.52139	-1.14543
134.9961	-0.44862	3.06233	-2.40777	0.52248	-1.14541
135.0309	-0.44922	3.06619	-2.40734	0.52296	-1.14569
135.064	-0.45122	3.06836	-2.40661	0.52427	-1.14623
135.0989	-0.45338	3.06916	-2.40457	0.52705	-1.14656
135.1331	-0.45552	3.07223	-2.40091	0.52889	-1.14672
135.1658	-0.4574	3.07974	-2.39831	0.53015	-1.14673
135.1994	-0.45892	3.08773	-2.39746	0.53075	-1.14667
135.2325	-0.46004	3.09513	-2.39629	0.53142	-1.1466
135.2674	-0.46164	3.09935	-2.39513	0.53332	-1.14652
135.3007	-0.46388	3.10094	-2.39422	0.53774	-1.14645
135.3348	-0.46594	3.10285	-2.39315	0.54147	-1.14644
135.3685	-0.4692	3.10497	-2.39252	0.54443	-1.14649

135.4014	-0.47166	3.10652	-2.39201	0.54481	-1.14654
135.4348	-0.47302	3.10775	-2.39071	0.54438	-1.14655
135.4684	-0.4737	3.10843	-2.38906	0.54317	-1.14655
135.5033	-0.47446	3.1098	-2.38815	0.54431	-1.14653
135.5377	-0.47578	3.11249	-2.38759	0.54735	-1.14653
135.5711	-0.47924	3.11654	-2.38717	0.55241	-1.14663
135.6042	-0.4827	3.12155	-2.38695	0.55768	-1.14672
135.6376	-0.4863	3.12421	-2.38665	0.56098	-1.14678
135.6709	-0.48918	3.1275	-2.38638	0.56223	-1.14686
135.7052	-0.49022	3.12948	-2.38613	0.56232	-1.14699
135.739	-0.49064	3.1319	-2.38583	0.55785	-1.14716
135.7716	-0.49086	3.13538	-2.38548	0.54769	-1.14754
135.8054	-0.49092	3.13833	-2.38442	0.53769	-1.14777
135.8396	-0.49206	3.14044	-2.38404	0.53436	-1.14789
135.8728	-0.49426	3.14359	-2.38383	0.53497	-1.14796
135.9071	-0.49718	3.14638	-2.38363	0.54089	-1.14801
135.9401	-0.50078	3.14858	-2.38301	0.55088	-1.14797
135.9739	-0.50382	3.1503	-2.38151	0.56062	-1.1479
136.0074	-0.50626	3.15114	-2.37996	0.56706	-1.14793
136.0404	-0.50706	3.15129	-2.3795	0.57011	-1.14805
136.0742	-0.5067	3.15161	-2.37898	0.57285	-1.14831
136.1079	-0.50504	3.15254	-2.37874	0.57518	-1.14868
136.142	-0.50372	3.15472	-2.37838	0.57709	-1.1489
136.1748	-0.50272	3.15966	-2.37804	0.58043	-1.14896
136.2086	-0.50204	3.16544	-2.37751	0.58284	-1.14904
136.2416	-0.50156	3.17098	-2.37708	0.58416	-1.14909
136.2753	-0.50112	3.17427	-2.3768	0.58479	-1.14914
136.309	-0.50002	3.17569	-2.37655	0.58495	-1.14926
136.343	-0.49906	3.17673	-2.37627	0.58508	-1.14939
136.3755	-0.49844	3.1792	-2.37598	0.58549	-1.14945

136.4086	-0.49784	3.18249	-2.37573	0.58576	-1.14948
136.4427	-0.4974	3.18598	-2.37502	0.58602	-1.14956
136.4764	-0.49706	3.18812	-2.37394	0.58637	-1.14966
136.5112	-0.4966	3.18944	-2.37283	0.58683	-1.14979
136.544	-0.49636	3.1905	-2.37221	0.58787	-1.14994
136.5772	-0.49618	3.19268	-2.37185	0.58912	-1.15011
136.611	-0.4961	3.19755	-2.37147	0.59009	-1.15037
136.6441	-0.49606	3.20154	-2.37114	0.59173	-1.15072
136.6779	-0.49604	3.20459	-2.37084	0.59273	-1.15091
136.711	-0.49606	3.20575	-2.37057	0.59366	-1.15098
136.7447	-0.4961	3.20613	-2.37033	0.59496	-1.15102
136.7781	-0.49618	3.20638	-2.37016	0.59533	-1.15118
136.8111	-0.49658	3.20641	-2.37001	0.59572	-1.15138
136.8448	-0.49744	3.20674	-2.36988	0.59596	-1.15149
136.8789	-0.4988	3.20909	-2.36973	0.5963	-1.15154
136.9116	-0.50134	3.2117	-2.36951	0.59778	-1.15159
136.9453	-0.5076	3.21361	-2.36925	0.59946	-1.15168
136.9777	-0.51532	3.21407	-2.36873	0.60008	-1.15187
137.0116	-0.52096	3.21429	-2.36758	0.60034	-1.15204
137.0452	-0.52698	3.21455	-2.36678	0.60054	-1.15213
137.0783	-0.53076	3.21496	-2.3665	0.60076	-1.1522
137.1126	-0.53554	3.21545	-2.36628	0.60106	-1.15231
137.1464	-0.52432	3.21571	-2.36605	0.60148	-1.15248
137.1792	-0.5299	3.2158	-2.3658	0.60207	-1.15265
137.2125	-0.53676	3.21623	-2.36553	0.60253	-1.15279
137.2458	-0.53652	3.21697	-2.36533	0.60282	-1.15291
137.2781	-0.53046	3.21749	-2.36514	0.60315	-1.15305
137.313	-0.5379	3.21785	-2.36499	0.6033	-1.15322
137.3466	-0.54024	3.21807	-2.36482	0.60383	-1.15344
137.3797	-0.53992	3.21843	-2.36468	0.60551	-1.15372

137.4129	-0.53956	3.21892	-2.36457	0.60872	-1.15413
137.4462	-0.54038	3.21922	-2.36439	0.60972	-1.15435
137.4795	-0.54204	3.21939	-2.36416	0.61008	-1.15454
137.5137	-0.54024	3.21953	-2.36387	0.61039	-1.15474
137.5475	-0.53992	3.22006	-2.36357	0.61074	-1.15516
137.581	-0.53956	3.2219	-2.36334	0.61108	-1.16132
137.6139	-0.54038	3.2251	-2.3631	0.6117	-1.19527
137.6476	-0.54024	3.22748	-2.36283	0.61233	-1.23814
137.6813	-0.53992	3.22823	-2.3626	0.61275	-1.25031
137.7145	-0.53956	3.22858	-2.36241	0.61304	-1.247
137.7476	-0.54038	3.22895	-2.36225	0.61321	-1.24167
137.7808	-0.53676	3.23021	-2.362	0.61329	-1.2379
137.8151	-0.53652	3.23186	-2.36173	0.61339	-1.23629
137.8488	-0.53046	3.2333	-2.36149	0.61357	-1.2353
137.8807	-0.5379	3.23391	-2.3613	0.61408	-1.23465
137.9134	-0.54038	3.23433	-2.36119	0.6144	-1.23408
137.9468	-0.54024	3.23476	-2.36105	0.61464	-1.23347
137.9816	-0.53992	3.23562	-2.3608	0.61485	-1.2327
138.0154	-0.53956	3.23649	-2.36053	0.61508	-1.23209
138.0484	-0.54038	3.23709	-2.36029	0.61531	-1.23148
138.0815	-0.5799	3.23752	-2.36005	0.61558	-1.23088
138.1152	-0.5783	3.23789	-2.35983	0.61582	-1.23026
138.1477	-0.57784	3.23842	-2.35958	0.61598	-1.22941
138.1814	-0.57872	3.23975	-2.35938	0.61613	-1.22849
138.2145	-0.58078	3.24118	-2.35916	0.61625	-1.22776
138.2469	-0.58098	3.24193	-2.35894	0.61637	-1.22722
138.2813	-0.5807	3.24263	-2.35873	0.6166	-1.22673
138.3139	-0.58032	3.24326	-2.35854	0.61677	-1.22625
138.3484	-0.58012	3.24505	-2.35837	0.61699	-1.22579
138.3814	-0.58018	3.24649	-2.35823	0.61723	-1.22527

138.4139	-0.58046	3.24819	-2.35806	0.61745	-1.22474
138.4479	-0.58078	3.24935	-2.35778	0.61768	-1.22423
138.4816	-0.60836	3.25	-2.35752	0.61789	-1.22368
138.5152	-0.61754	3.25027	-2.3573	0.61807	-1.22313
138.5486	-0.61454	3.25104	-2.3571	0.61827	-1.22259
138.5822	-0.6147	3.25363	-2.35691	0.61848	-1.22207
138.6154	-0.61638	3.2579	-2.35674	0.61871	-1.22163
138.6474	-0.6243	3.26268	-2.35657	0.61892	-1.22121
138.6819	-0.63376	3.26564	-2.35644	0.6191	-1.22081
138.7149	-0.62606	3.26681	-2.35635	0.61927	-1.22046
138.7484	-0.65882	3.26762	-2.3562	0.61949	-1.2201
138.7824	-0.65122	3.2689	-2.35604	0.6197	-1.21966
138.8159	-0.64688	3.26994	-2.35583	0.61994	-1.2193
138.8491	-0.64384	3.27181	-2.35569	0.62036	-1.21885
138.8822	-0.64064	3.27434	-2.35559	0.62087	-1.21837
138.915	-0.63712	3.27588	-2.35547	0.62137	-1.21799
138.9473	-0.62908	3.2764	-2.35541	0.62233	-1.21767
138.9819	-0.62294	3.27683	-2.35538	0.62271	-1.21741
139.0142	-0.62008	3.27769	-2.35535	0.62298	-1.21714
139.0489	-0.61926	3.27922	-2.35542	0.62328	-1.21679
139.0818	-0.61786	3.28098	-2.35537	0.62352	-1.21647
139.1156	-0.6156	3.28197	-2.35521	0.62374	-1.21618
139.1486	-0.6106	3.28267	-2.35503	0.62398	-1.21592
139.1815	-0.60694	3.28315	-2.35484	0.62417	-1.21569
139.2155	-0.60608	3.28391	-2.35465	0.6243	-1.21545
139.2486	-0.60534	3.28461	-2.35457	0.62451	-1.21521
139.281	-0.6032	3.28489	-2.35452	0.62472	-1.21492
139.3146	-0.59842	3.2852	-2.35442	0.62514	-1.21467
139.3478	-0.5971	3.2855	-2.35435	0.62631	-1.21449
139.3816	-0.5965	3.28579	-2.3542	0.6274	-1.21429

139.4142	-0.59738	3.28605	-2.35398	0.62776	-1.21408
139.4484	-0.59924	3.2862	-2.35383	0.62799	-1.21393
139.4813	-0.59794	3.28632	-2.35378	0.62817	-1.21381
139.515	-0.5967	3.28645	-2.35386	0.62838	-1.21366
139.548	-0.59536	3.28661	-2.35389	0.62858	-1.21348
139.5809	-0.59276	3.28675	-2.35379	0.62879	-1.21324
139.6142	-0.59128	3.28688	-2.35347	0.629-1.21273	
139.648	-0.58756	3.28696	-2.35313	0.62923	-1.21245
139.6809	-0.58472	3.28705	-2.35298	0.62964	-1.21226
139.714	-0.5829	3.28721	-2.35293	0.63035	-1.21212
139.7483	-0.58206	3.28736	-2.35286	0.63117	-1.21202
139.7822	-0.58224	3.2875	-2.35279	0.63152	-1.21193
139.8153	-0.58288	3.28768	-2.35266	0.63177	-1.21182
139.8488	-0.5833	3.28784	-2.35249	0.63196	-1.21176
139.8822	-0.5836	3.28799	-2.35231	0.63208	-1.21174
139.9141	-0.58338	3.28814	-2.35215	0.63226	-1.21167
139.9483	-0.58218	3.28824	-2.3521	0.63251	-1.21151
139.9814	-0.5799	3.28832	-2.35216	0.63275	-1.2113
140.0156	-0.5783	3.28848	-2.35246	0.63299	-1.21095
140.0488	-0.57784	3.28875	-2.35272	0.63331	-1.21071
140.0823	-0.57872	3.28899	-2.3528	0.63393	-1.21055
140.1149	-0.58078	3.28919	-2.35269	0.63446	-1.21038
140.1481	-0.58098	3.28929	-2.35258	0.63474	-1.21028
140.1822	-0.5807	3.28931	-2.35247	0.63488	-1.21024
140.2158	-0.58032	3.28938	-2.35242	0.635-1.21024	
140.2489	-0.58012	3.28957	-2.35247	0.63516	-1.2103
140.2822	-0.58018	3.28976	-2.35273	0.63545	-1.21026
140.3154	-0.58046	3.28991	-2.35291	0.63576	-1.21011
140.3495	-0.58078	3.29008	-2.35301	0.63603	-1.20993
140.3828	-0.58144	3.29025	-2.35305	0.6364	-1.2098

140.4164	-0.58264	3.29066	-2.35307	0.63673	-1.20983
140.4487	-0.5849	3.29134	-2.35311	0.63702	-1.20988
140.4821	-0.58712	3.29157	-2.35324	0.63732	-1.20989
140.5163	-0.58804	3.29175	-2.35329	0.63757	-1.20983
140.549	-0.58876	3.29196	-2.35308	0.63781	-1.20958
140.5829	-0.59022	3.29212	-2.35264	0.63804	-1.2094
140.6159	-0.59422	3.29232	-2.35238	0.63831	-1.20935
140.6492	-0.60284	3.29253	-2.35232	0.63855	-1.20945
140.6826	-0.6117	3.29271	-2.35237	0.63883	-1.20991
140.7162	-0.61606	3.29302	-2.3524	0.63919	-1.21022
140.7494	-0.61892	3.29328	-2.35244	0.63968	-1.21021
140.7821	-0.6238	3.29354	-2.35251	0.64	-1.21012
140.8151	-0.63078	3.29413	-2.35261	0.64031	-1.21002
140.8493	-0.64228	3.29522	-2.35271	0.64055	-1.21001
140.882	-0.65398	3.29616	-2.35271	0.64071	-1.21003
140.9155	-0.64228	3.29711	-2.35269	0.64091	-1.21005
140.9496	-0.65398	3.29753	-2.35264	0.64112	-1.21002
140.9826	-0.65706	3.2984	-2.35256	0.64149	-1.20994
141.0162	-0.65694	3.30142	-2.3525	0.64246	-1.20991
141.0493	-0.65944	3.3057	-2.35243	0.64298	-1.20995
141.0832	-0.6515	3.30928	-2.35246	0.64328	-1.21002
141.1158	-0.6497	3.31035	-2.35254	0.64349	-1.21004
141.1494	-0.63636	3.31071	-2.35264	0.64367	-1.21002
141.1832	-0.63692	3.31097	-2.35274	0.64391	-1.20998
141.2158	-0.62438	3.31115	-2.35281	0.64416	-1.20994
141.2492	-0.63794	3.31151	-2.35289	0.64434	-1.20995
141.2824	-0.64228	3.31212	-2.35298	0.64446	-1.20998
141.3161	-0.65398	3.31275	-2.35307	0.64457	-1.21004
141.35	-0.65706	3.31321	-2.35309	0.64469	-1.21009
141.3827	-0.65694	3.31347	-2.35301	0.64489	-1.21019

141.4158	-0.65944	3.31366	-2.35297	0.64537	-1.21063
141.449	-0.6515	3.3138	-2.35302	0.6457	-1.2111
141.483	-0.6497	3.31389	-2.35312	0.64598	-1.21164
141.5166	-0.63636	3.31399	-2.35322	0.64621	-1.2119
141.5488	-0.63692	3.31409	-2.35322	0.6464	-1.212
141.5829	-0.62438	3.31417	-2.35316	0.64671	-1.21207
141.6154	-0.63794	3.31433	-2.3532	0.64706	-1.21212
141.6494	-0.65882	3.31451	-2.35332	0.64741	-1.21222
141.6829	-0.65122	3.31464	-2.35351	0.64765	-1.2124
141.7164	-0.65694	3.31477	-2.35379	0.64778	-1.21259
141.7487	-0.65944	3.31481	-2.35392	0.64787	-1.21273
141.7816	-0.66116	3.31491	-2.35397	0.64793	-1.21282
141.8161	-0.66156	3.31509	-2.35396	0.64804	-1.21286
141.8496	-0.68656	3.31526	-2.35393	0.64823	-1.21296
141.8822	-0.68042	3.31539	-2.35392	0.64842	-1.21311
141.916	-0.68612	3.31539	-2.35397	0.64875	-1.21344
141.95	-0.68588	3.31531	-2.35406	0.64906	-1.21364
141.9822	-0.69104	3.31532	-2.35417	0.64926	-1.21395
142.0158	-0.68238	3.31537	-2.35426	0.64942	-1.21415
142.0488	-0.69862	3.31545	-2.35429	0.64952	-1.21428
142.0825	-0.69884	3.31552	-2.35429	0.64958	-1.21438
142.1148	-0.70618	3.31554	-2.35431	0.64968	-1.21455
142.149	-0.72566	3.31553	-2.35439	0.64988	-1.21515
142.1826	-0.73056	3.31558	-2.35475	0.6502	-1.21746
142.216	-0.72172	3.31569	-2.35506	0.65046	-1.21916
142.2488	-0.7219	3.31606	-2.3552	0.65071	-1.21966
142.282	-0.73986	3.31741	-2.35525	0.65085	-1.21989
142.3147	-0.74034	3.31931	-2.35521	0.65091	-1.22002
142.3483	-0.74068	3.32063	-2.35519	0.65094	-1.22013
142.3818	-0.7406	3.32179	-2.35522	0.651-1.22022	

142.4147	-0.74042	3.32241	-2.3553	0.65112	-1.2203
142.4482	-0.73992	3.32305	-2.35544	0.65139	-1.22038
142.4825	-0.73944	3.32523	-2.35554	0.65165	-1.22054
142.5152	-0.7389	3.32802	-2.35563	0.65184	-1.22076
142.5487	-0.73748	3.33066	-2.3557	0.65193	-1.22104
142.5823	-0.73432	3.33331	-2.35583	0.65203	-1.22126
142.6146	-0.7315	3.33521	-2.35598	0.65211	-1.2214
142.6479	-0.7287	3.33619	-2.35613	0.6522	-1.22149
142.6824	-0.74036	3.33722	-2.35621	0.65232	-1.22159
142.7149	-0.74166	3.33778	-2.35623	0.65247	-1.22168
142.7482	-0.74492	3.33846	-2.35625	0.65262	-1.22187
142.782	-0.74726	3.33963	-2.35627	0.65276	-1.22204
142.815	-0.75002	3.34067	-2.35628	0.65287	-1.22214
142.8487	-0.7521	3.34127	-2.3563	0.65297	-1.22222
142.8826	-0.75276	3.34191	-2.35639	0.65316	-1.22231
142.9155	-0.75382	3.34213	-2.35654	0.65337	-1.2224
142.9478	-0.75592	3.34218	-2.35675	0.65349	-1.22267
142.9817	-0.75608	3.34223	-2.35694	0.6535	-1.22301
143.0153	-0.75652	3.34229	-2.35711	0.65337	-1.22323
143.049	-0.76106	3.34236	-2.35724	0.65333	-1.22385
143.0822	-0.7971	3.34249	-2.35739	0.6534	-1.22472
143.1157	-0.79372	3.34251	-2.35746	0.65355	-1.22535
143.1485	-0.79112	3.34244	-2.35753	0.65372	-1.22577
143.1814	-0.78906	3.34231	-2.35757	0.6539	-1.22594
143.2154	-0.78782	3.34224	-2.35768	0.65407	-1.22603
143.2488	-0.78572	3.34221	-2.35804	0.65424	-1.22614
143.2822	-0.78212	3.34227	-2.35848	0.65438	-1.22627
143.3149	-0.81818	3.34234	-2.35868	0.65449	-1.22646
143.3482	-0.80576	3.3423	-2.35885	0.65456	-1.22672
143.3809	-0.79472	3.34226	-2.35893	0.65462	-1.22698

143.4161	-0.78554	3.34222	-2.35907	0.65468	-1.22722
143.4489	-0.77342	3.34212	-2.35923	0.65469	-1.22739
143.4827	-0.76384	3.34205	-2.35935	0.65468	-1.22748
143.5151	-0.77754	3.34204	-2.35953	0.6547	-1.22755
143.5488	-0.78052	3.34205	-2.35969	0.65476	-1.22764
143.5823	-0.78292	3.34213	-2.35985	0.65488	-1.22774
143.6157	-0.7915	3.34227	-2.35998	0.65502	-1.22789
143.6489	-0.80004	3.34237	-2.36 0.65512	-1.22804	
143.683	-0.8091	3.3424	-2.36009	0.65525	-1.2282
143.7151	-0.80244	3.34235	-2.3603	0.6554	-1.22841
143.7469	-0.8141	3.34233	-2.36099	0.65552	-1.22913
143.7811	-0.82738	3.3424	-2.36158	0.65559	-1.23066
143.8152	-0.84048	3.34252	-2.36177	0.65562	-1.23168
143.8485	-0.83424	3.34265	-2.36185	0.65569	-1.23189
143.882	-0.82858	3.34274	-2.36196	0.65582	-1.23202
143.9152	-0.841	3.34274	-2.36203	0.65616	-1.23207
143.9483	-0.85168	3.34281	-2.36212	0.65649	-1.23214
143.9803	-0.84494	3.34294	-2.36227	0.65668	-1.23231
144.0151	-0.84198	3.34314	-2.36243	0.65677	-1.23253
144.0485	-0.83676	3.34372	-2.36269	0.6568	-1.23277
144.0808	-0.83174	3.34522	-2.36306	0.65681	-1.23296
144.1156	-0.8268	3.34762	-2.36317	0.65692	-1.23309
144.1486	-0.8215	3.35101	-2.36323	0.65712	-1.23326
144.1819	-0.81976	3.35189	-2.36325	0.65741	-1.23347
144.2145	-0.81592	3.35209	-2.36332	0.65764	-1.23387
144.2477	-0.81238	3.35238	-2.36347	0.65781	-1.23469
144.2816	-0.80922	3.35325	-2.36368	0.6579	-1.23539
144.3148	-0.80226	3.35458	-2.36399	0.65799	-1.23585
144.3484	-0.7971	3.35538	-2.36424	0.65811	-1.23642
144.3811	-0.79372	3.35572	-2.36436	0.65824	-1.23667

144.4145	-0.79112	3.356	-2.36444	0.65833	-1.23689
144.4473	-0.78906	3.35636	-2.36453	0.65834	-1.23709
144.482	-0.78782	3.35689	-2.36463	0.65824	-1.23723
144.5148	-0.78572	3.35723	-2.36478	0.65826	-1.23735
144.5488	-0.78212	3.3583	-2.36486	0.65843	-1.23747
144.5813	-0.78124	3.35967	-2.36489	0.66022	-1.23763
144.6145	-0.77954	3.36026	-2.36492	0.66236	-1.23785
144.6482	-0.776	3.36059	-2.36502	0.66288	-1.23812
144.6817	-0.77056	3.36073	-2.36553	0.66302	-1.23837
144.7146	-0.76916	3.36088	-2.36617	0.66301	-1.23859
144.7476	-0.76824	3.36113	-2.36664	0.66292	-1.2388
144.7814	-0.76538	3.36149	-2.36683	0.66298	-1.23905
144.815	-0.76378	3.36171	-2.3669	0.66303	-1.23947
144.8486	-0.76106	3.36183	-2.36697	0.66308	-1.24004
144.8817	-0.75652	3.36188	-2.36711	0.66307	-1.24038
144.9149	-0.75608	3.36187	-2.36743	0.66294	-1.24062
144.9484	-0.75592	3.36177	-2.3684	0.6628	-1.24085
144.9817	-0.75382	3.36171	-2.36869	0.66278	-1.24103
145.0155	-0.75276	3.36171	-2.36889	0.6629	-1.24121
145.0489	-0.7521	3.36182	-2.36903	0.66325	-1.24139
145.0818	-0.75002	3.36197	-2.36912	0.66345	-1.24154
145.1154	-0.74726	3.36207	-2.36916	0.66355	-1.24174
145.1489	-0.74492	3.36211	-2.36922	0.6636	-1.24198
145.1816	-0.74166	3.36212	-2.36929	0.66362	-1.2422
145.2158	-0.74036	3.36209	-2.36948	0.66373	-1.24242
145.249	-0.73986	3.36216	-2.36993	0.6639	-1.24262
145.2827	-0.74034	3.36215	-2.37014	0.66394	-1.24282
145.3154	-0.74068	3.36205	-2.37018	0.66397	-1.24303
145.3482	-0.7406	3.36201	-2.3702	0.66399	-1.24325
145.3815	-0.74042	3.362	-2.37031	0.66402	-1.2435

145.4147	-0.73992	3.36204	-2.37066	0.66412	-1.24375
145.4475	-0.73944	3.36211	-2.37224	0.66419	-1.244
145.4816	-0.7389	3.36212	-2.37384	0.66429	-1.24424
145.5152	-0.73748	3.36208	-2.37466	0.6644	-1.2445
145.5484	-0.73432	3.36211	-2.37498	0.66449	-1.24477
145.5814	-0.7315	3.36212	-2.37515	0.66459	-1.24526
145.615	-0.7287	3.36211	-2.3753	0.6646	-1.24568
145.6482	-0.72708	3.36209	-2.3755	0.6646	-1.24599
145.6817	-0.7262	3.36211	-2.37568	0.66463	-1.24629
145.7146	-0.72446	3.36219	-2.37586	0.66466	-1.24651
145.7473	-0.72302	3.36231	-2.37596	0.66467	-1.24672
145.7811	-0.72188	3.36236	-2.37603	0.66468	-1.24699
145.8146	-0.72012	3.36239	-2.37611	0.66462	-1.24742
145.8483	-0.71958	3.36248	-2.37621	0.66457	-1.24822
145.8816	-0.71908	3.36264	-2.37637	0.6646	-1.24882
145.9147	-0.71816	3.36325	-2.37655	0.66469	-1.24915
145.9477	-0.7174	3.36537	-2.37671	0.6648	-1.24942
145.981	-0.71708	3.369	-2.3769	0.66491	-1.24964
146.0144	-0.7169	3.37411	-2.37712	0.665	-1.24984
146.0483	-0.71672	3.37834	-2.37729	0.66509	-1.25004
146.0812	-0.71608	3.37919	-2.37743	0.66528	-1.25029
146.1146	-0.7148	3.37934	-2.37749	0.66538	-1.25083
146.1478	-0.71338	3.37945	-2.37757	0.66537	-1.25154
146.1807	-0.71216	3.37965	-2.37775	0.66536	-1.25189
146.2142	-0.7111	3.38081	-2.37798	0.66541	-1.25214
146.2482	-0.71022	3.38255	-2.37818	0.66558	-1.25235
146.2812	-0.70824	3.38345	-2.37833	0.66603	-1.25261
146.3148	-0.70484	3.38395	-2.37845	0.6663	-1.25289
146.3479	-0.70174	3.38447	-2.37856	0.66639	-1.25315
146.3816	-0.69906	3.3856	-2.37873	0.66639	-1.25334

146.4156	-0.69768	3.38723	-2.37889	0.66631	-1.25352
146.4482	-0.6977	3.38887	-2.37904	0.66608	-1.25378
146.4816	-0.69772	3.39033	-2.3792	0.6661	-1.25415
146.5145	-0.6976	3.39101	-2.37936	0.66622	-1.2545
146.5474	-0.69768	3.39133	-2.37954	0.66641	-1.25481
146.5812	-0.69766	3.39164	-2.37986	0.66657	-1.25507
146.6155	-0.69764	3.39186	-2.38013	0.66668	-1.25533
146.6486	-0.69758	3.39222	-2.38042	0.66677	-1.25557
146.682	-0.69726	3.3931	-2.38063	0.6669	-1.25585
146.7146	-0.69688	3.39448	-2.38079	0.66706	-1.25616
146.7477	-0.69676	3.39546	-2.38091	0.66722	-1.25644
146.7808	-0.69688	3.39627	-2.38102	0.66731	-1.25675
146.8144	-0.69692	3.39676	-2.38114	0.66736	-1.25712
146.8479	-0.69652	3.39702	-2.38124	0.66739	-1.25744
146.8814	-0.69556	3.39716	-2.38136	0.66739	-1.2577
146.9149	-0.6933	3.39738	-2.38156	0.66739	-1.25796
146.9479	-0.69184	3.39761	-2.38175	0.66741	-1.25816
146.9805	-0.6915	3.39782	-2.38192	0.6674	-1.25845
147.0146	-0.69134	3.39793	-2.38211	0.66738	-1.25875
147.0479	-0.69124	3.39791	-2.38233	0.66743	-1.25903
147.081	-0.69136	3.39791	-2.38257	0.66748	-1.25933
147.1142	-0.69146	3.39795	-2.3828	0.66754	-1.25966
147.1478	-0.69146	3.39794	-2.38296	0.66773	-1.26008
147.1806	-0.69134	3.39792	-2.38302	0.6679	-1.26051
147.2141	-0.6909	3.39785	-2.38307	0.66801	-1.26082
147.2474	-0.69026	3.39766	-2.38314	0.66814	-1.26107
147.2815	-0.69	3.39757	-2.38322	0.66839	-1.26131
147.3143	-0.68984	3.39764	-2.38335	0.66857	-1.2616
147.3482	-0.68942	3.39776	-2.38349	0.66871	-1.26224
147.3811	-0.68902	3.39792	-2.3837	0.66875	-1.26293

147.4145	-0.68876	3.39803	-2.38456	0.66876	-1.26356
147.4475	-0.6887	3.39801	-2.38517	0.66887	-1.2639
147.4813	-0.68862	3.39786	-2.38545	0.66918	-1.26413
147.5146	-0.68852	3.39775	-2.38565	0.6693	-1.26439
147.5475	-0.6884	3.39773	-2.3858	0.66937	-1.2647
147.581	-0.6881	3.39774	-2.38591	0.66947	-1.26524
147.6144	-0.68776	3.39774	-2.38606	0.66958	-1.26587
147.648	-0.68768	3.39779	-2.38619	0.6697	-1.26621
147.6806	-0.68756	3.3978	-2.38631	0.66976	-1.26653
147.7149	-0.68736	3.39782	-2.38645	0.66975	-1.26688
147.7476	-0.68708	3.39786	-2.38667	0.66974	-1.2673
147.7806	-0.68672	3.39784	-2.38709	0.66977	-1.26791
147.815	-0.6865	3.39777	-2.3874	0.66978	-1.26835
147.8476	-0.68636	3.39771	-2.38754	0.66985	-1.26863
147.8812	-0.68626	3.39765	-2.38767	0.66999	-1.26884
147.9146	-0.68612	3.39755	-2.38781	0.67017	-1.26907
147.9481	-0.68604	3.39744	-2.38794	0.67028	-1.26933
147.9807	-0.68594	3.39737	-2.38816	0.67037	-1.2697
148.015	-0.68586	3.39731	-2.38844	0.67043	-1.2703
148.0478	-0.68556	3.39732	-2.38881	0.67055	-1.27067
148.0807	-0.68518	3.39735	-2.38916	0.67065	-1.27099
148.1143	-0.68504	3.39727	-2.38942	0.67077	-1.27128
148.1481	-0.68496	3.39715	-2.38957	0.67083	-1.27163
148.1812	-0.68492	3.39706	-2.38968	0.67087	-1.27229
148.2146	-0.68486	3.39704	-2.38979	0.67099	-1.27272
148.2469	-0.6848	3.39741	-2.38997	0.67104	-1.273
148.2814	-0.6847	3.39874	-2.39013	0.67113	-1.2732
148.3148	-0.68454	3.40059	-2.39028	0.67134	-1.27342
148.3468	-0.6843	3.40119	-2.39042	0.67171	-1.27367
148.381	-0.68408	3.40124	-2.39056	0.67205	-1.27399

148.4137	-0.68394	3.40126	-2.39074	0.67235	-1.27433
148.4478	-0.68384	3.40134	-2.39096	0.67248	-1.27462
148.481	-0.6837	3.40206	-2.39113	0.67262	-1.27491
148.5142	-0.68352	3.40517	-2.39136	0.67279	-1.27516
148.5474	-0.68328	3.40804	-2.39168	0.67299	-1.2754
148.581	-0.68314	3.4091	-2.39201	0.67315	-1.2757
148.6139	-0.683	3.40968	-2.39247	0.6733	-1.27601
148.6482	-0.68294	3.41008	-2.3926	0.67355	-1.27633
148.6813	-0.68286	3.41026	-2.39259	0.67471	-1.27664
148.7141	-0.68278	3.41071	-2.3927	0.67643	-1.27703
148.7473	-0.68268	3.41227	-2.39288	0.67721	-1.27833
148.7804	-0.68248	3.41394	-2.39342	0.67737	-1.28019
148.8137	-0.68232	3.41568	-2.39383	0.67742	-1.28097
148.8473	-0.68222	3.41679	-2.39393	0.6774	-1.28124
148.8814	-0.68216	3.4171	-2.39394	0.67743	-1.28138
148.9144	-0.68212	3.41725	-2.39403	0.67755	-1.2815
148.9475	-0.68208	3.4175	-2.39414	0.67776	-1.28169
148.9809	-0.682	3.41851	-2.39474	0.678-1.28206	
149.0141	-0.6819	3.42072	-2.39516	0.67827	-1.28249
149.0472	-0.68174	3.42201	-2.39539	0.67852	-1.28279
149.0811	-0.6815	3.42233	-2.39559	0.67872	-1.28302
149.115	-0.68138	3.42264	-2.39568	0.67889	-1.28327
149.1486	-0.68128	3.42301	-2.39577	0.679-1.28356	
149.1818	-0.68118	3.42354	-2.39587	0.67907	-1.28387
149.2142	-0.68114	3.42476	-2.39598	0.67917	-1.28424
149.2476	-0.6811	3.42534	-2.39619	0.67929	-1.28455
149.2805	-0.68104	3.42557	-2.39653	0.67944	-1.28482
149.3136	-0.68094	3.42572	-2.39709	0.67955	-1.28507
149.3478	-0.68078	3.42608	-2.39745	0.67966	-1.28527
149.3813	-0.6806	3.42733	-2.3976	0.67983	-1.28544

149.4147	-0.68042	3.4292	-2.39766	0.67999	-1.28568
149.4485	-0.6803	3.42984	-2.39769	0.68012	-1.28608
149.4811	-0.68018	3.42999	-2.39779	0.68021	-1.28711
149.5145	-0.6801	3.42979	-2.39792	0.68031	-1.28795
149.5475	-0.68006	3.42946	-2.39803	0.68043	-1.28887
149.581	-0.68002	3.42942	-2.39812	0.68059	-1.28916
149.6141	-0.67996	3.4293	-2.39826	0.68073	-1.28938
149.6471	-0.6799	3.42877	-2.39845	0.68085	-1.28957
149.6804	-0.67982	3.4285	-2.39899	0.68109	-1.28983
149.714	-0.6797	3.4284	-2.39934	0.68132	-1.29018
149.7478	-0.67958	3.42834	-2.39955	0.68153	-1.29067
149.7809	-0.67944	3.42832	-2.39965	0.68168	-1.29101
149.8144	-0.67934	3.42827	-2.39974	0.68165	-1.29126
149.8476	-0.67926	3.42822	-2.39984	0.68163	-1.29152
149.8802	-0.67922	3.42819	-2.39992	0.68169	-1.29184
149.9148	-0.67914	3.42816	-2.40003	0.68184	-1.29217
149.9477	-0.67908	3.42815	-2.40016	0.6822	-1.29246
149.9803	-0.67898	3.42813	-2.40028	0.68245	-1.29267
150.0145	-0.6789	3.42807	-2.40048	0.68257	-1.29282
150.0476	-0.67882	3.42801	-2.40088	0.68263	-1.29303
150.0811	-0.67874	3.42793	-2.40128	0.68262	-1.29327
150.1155	-0.67866	3.42785	-2.40202	0.68267	-1.29357
150.1475	-0.6786	3.42778	-2.40242	0.68277	-1.29385
150.1809	-0.67854	3.42773	-2.40262	0.68295	-1.29409
150.2147	-0.67852	3.42768	-2.40272	0.68313	-1.29449
150.2478	-0.67848	3.4277	-2.40284	0.68325	-1.29526
150.2806	-0.6784	3.42768	-2.40298	0.68333	-1.29594
150.3142	-0.6783	3.42766	-2.40316	0.68335	-1.2963
150.3471	-0.67822	3.42758	-2.40337	0.6834	-1.29659
150.3817	-0.67814	3.42745	-2.40352	0.6835	-1.29682

150.4148	-0.67812	3.42737	-2.4036	0.68357	-1.29712
150.4476	-0.67812	3.42741	-2.40366	0.68367	-1.29742
150.4814	-0.67808	3.42774	-2.40372	0.68379	-1.29772
150.5153	-0.67804	3.42811	-2.40384	0.68391	-1.298
150.5471	-0.678	3.42828	-2.40404	0.68412	-1.2983
150.581	-0.67792	3.42837	-2.40424	0.68438	-1.29863
150.614	-0.67788	3.42837	-2.40444	0.68457	-1.29896
150.6474	-0.67786	3.42835	-2.40458	0.68473	-1.29935
150.6814	-0.67782	3.42857	-2.4047	0.68483	-1.29977
150.7136	-0.6778	3.42946	-2.4049	0.6849	-1.3001
150.7478	-0.67776	3.43095	-2.40521	0.68503	-1.30073
150.7816	-0.67772	3.43186	-2.40562	0.68522	-1.3017
150.8139	-0.67768	3.43221	-2.40583	0.68552	-1.30284
150.8475	-0.67766	3.43229	-2.406	0.68575	-1.30374
150.8809	-0.67762	3.43267	-2.40617	0.68584	-1.30415
150.9136	-0.6776	3.43366	-2.40637	0.6859	-1.30441
150.9471	-0.67758	3.43438	-2.40669	0.68598	-1.30469
150.9807	-0.67754	3.43472	-2.40699	0.68604	-1.30499
151.0148	-0.67752	3.43491	-2.40722	0.68613	-1.30533
151.0476	-0.67752	3.43504	-2.40745	0.6862	-1.30564
151.0814	-0.67752	3.43532	-2.40761	0.68623	-1.30642
151.1144	-0.67752	3.43573	-2.40767	0.68624	-1.30787
151.148	-0.6775	3.43636	-2.40776	0.68631	-1.31007
151.1806	-0.67746	3.43748	-2.40786	0.68644	-1.31225
151.2141	-0.67734	3.43974	-2.40797	0.68663	-1.31396
151.2477	-0.67706	3.44176	-2.40809	0.68702	-1.31524
151.2814	-0.67696	3.44271	-2.40823	0.68737	-1.31772
151.3147	-0.6769	3.44301	-2.4084	0.68753	-1.32051
151.3468	-0.67688	3.4433	-2.40868	0.68771	-1.32616
151.3801	-0.67688	3.4437	-2.40909	0.68804	-1.33848

151.4138	-0.67688	3.44438	-2.40941	0.68822	-1.35474
151.4474	-0.67688	3.44542	-2.40962	0.68835	-1.36782
151.48	-0.67688	3.44591	-2.4098	0.68847	-1.37429
151.5142	-0.6769	3.44616	-2.40999	0.68856	-1.37936
151.5468	-0.6769	3.44628	-2.41027	0.68868	-1.38449
151.5812	-0.67688	3.44631	-2.41079	0.68879	-1.38793
151.6139	-0.67688	3.44639	-2.41135	0.68891	-1.38987
151.6477	-0.67688	3.44657	-2.41153	0.68897	-1.39115
151.6814	-0.6769	3.44678	-2.41159	0.68902	-1.39285
151.7142	-0.67694	3.44704	-2.41165	0.68916	-1.39954
151.7468	-0.67694	3.4472	-2.41178	0.6893	-1.41152
151.78	-0.67698	3.44725	-2.41253	0.68938	-1.42211
151.8139	-0.67704	3.44702	-2.4141	0.68951	-1.42497
151.8479	-0.67708	3.44617	-2.41546	0.68958	-1.4243
151.8811	-0.67706	3.44566	-2.41594	0.68971	-1.42176
151.9144	-0.67704	3.44561	-2.41613	0.68986	-1.42019
151.9471	-0.67704	3.44569	-2.41624	0.69004	-1.41932
151.9809	-0.67704	3.44581	-2.41634	0.69022	-1.41868
152.0144	-0.67706	3.44585	-2.41643	0.69042	-1.41801
152.0479	-0.6771	3.44587	-2.41657	0.69058	-1.41731
152.0809	-0.6771	3.44588	-2.41672	0.69069	-1.41659
152.1147	-0.67712	3.4459	-2.41692	0.69074	-1.41636
152.1483	-0.67714	3.44588	-2.4172	0.69074	-1.4162
152.1811	-0.67716	3.44585	-2.41765	0.69075	-1.41614
152.2146	-0.67714	3.44584	-2.4179	0.69089	-1.41605
152.2475	-0.67714	3.44586	-2.41818	0.69112	-1.41587
152.2804	-0.67714	3.44591	-2.4184	0.69154	-1.41556
152.3136	-0.67712	3.44594	-2.4186	0.6919	-1.41548
152.347	-0.67716	3.44589	-2.41881	0.69197	-1.4153
152.3805	-0.67722	3.44586	-2.41892	0.69184	-1.41517

152.4136	-0.67726	3.44583	-2.41895	0.69156	-1.41504
152.4475	-0.67738	3.44579	-2.41897	0.69145	-1.4149
152.4811	-0.6774	3.4458	-2.41899	0.6915	-1.41476
152.514	-0.67742	3.44586	-2.41906	0.69174	-1.41465
152.5476	-0.67744	3.44594	-2.41948	0.69189	-1.41456
152.5806	-0.67746	3.44607	-2.42073	0.69203	-1.41449
152.6142	-0.67748	3.44608	-2.42205	0.69218	-1.41449
152.6472	-0.6775	3.44586	-2.42251	0.69243	-1.41471
152.6796	-0.67752	3.44569	-2.42265	0.69269	-1.41477
152.7136	-0.67754	3.44566	-2.42269	0.69291	-1.41493
152.7457	-0.67754	3.44585	-2.42273	0.69297	-1.41555
152.7799	-0.67758	3.44712	-2.42278	0.69294	-1.41616
152.8131	-0.67758	3.44784	-2.42291	0.69289	-1.41656
152.8473	-0.6776	3.44832	-2.42311	0.69287	-1.41697
152.8804	-0.67762	3.44902	-2.42332	0.69289	-1.41709
152.914	-0.67764	3.45065	-2.42352	0.69293	-1.41718
152.9472	-0.67764	3.45414	-2.42376	0.69307	-1.41726
152.9806	-0.67768	3.45763	-2.42398	0.69326	-1.41733
153.0128	-0.67772	3.45832	-2.42417	0.69338	-1.41738
153.0466	-0.67782	3.45859	-2.42435	0.69345	-1.41741
153.0802	-0.67786	3.45881	-2.42448	0.69342	-1.41742
153.1136	-0.6779	3.45915	-2.42466	0.69336	-1.41745
153.1471	-0.67794	3.46113	-2.42485	0.69336	-1.41743
153.1802	-0.67796	3.46281	-2.42507	0.69344	-1.41745
153.2138	-0.67798	3.46359	-2.4253	0.6936	-1.41754
153.2469	-0.67798	3.4639	-2.42548	0.69372	-1.41764
153.2806	-0.67804	3.46401	-2.42569	0.69378	-1.41774
153.3138	-0.67812	3.46431	-2.42586	0.69386	-1.41772
153.3472	-0.6783	3.46636	-2.42597	0.69391	-1.4175
153.3799	-0.6784	3.46954	-2.42612	0.694-1.41731	

153.4143	-0.67846	3.47285	-2.42632	0.69409	-1.41733
153.447	-0.67846	3.47404	-2.42647	0.6941	-1.41748
153.4808	-0.67846	3.47433	-2.42661	0.69412	-1.41819
153.5142	-0.67842	3.47471	-2.42664	0.69425	-1.41876
153.5469	-0.67842	3.4751	-2.42664	0.69437	-1.41906
153.5804	-0.67842	3.47535	-2.42672	0.69448	-1.41928
153.6136	-0.67844	3.47564	-2.42705	0.69452	-1.41944
153.6472	-0.6785	3.47591	-2.42786	0.69456	-1.41953
153.6804	-0.67858	3.47608	-2.4286	0.69459	-1.41957
153.7139	-0.67866	3.47626	-2.42896	0.6946	-1.41962
153.7468	-0.6787	3.47633	-2.42919	0.69458	-1.41978
153.78	-0.67872	3.47632	-2.42941	0.69453	-1.42015
153.8138	-0.67876	3.47633	-2.42963	0.69456	-1.42044
153.8471	-0.67882	3.47617	-2.42985	0.69458	-1.42062
153.88	-0.67888	3.47585	-2.43003	0.69469	-1.42071
153.9136	-0.6789	3.47556	-2.43017	0.69478	-1.42065
153.9472	-0.67892	3.47547	-2.43033	0.69485	-1.4205
153.9812	-0.67896	3.47549	-2.43045	0.69494	-1.42048
154.0146	-0.67904	3.47549	-2.43054	0.69508	-1.42058
154.0476	-0.67912	3.47543	-2.43065	0.69537	-1.42099
154.0799	-0.67918	3.47529	-2.43075	0.69583	-1.42166
154.1134	-0.67924	3.47487	-2.43093	0.69627	-1.422
154.1471	-0.67928	3.47469	-2.43112	0.69653	-1.42214
154.1797	-0.67932	3.47434	-2.43128	0.69668	-1.4222
154.2133	-0.67936	3.47404	-2.43144	0.69674	-1.42228
154.2467	-0.67938	3.47391	-2.43159	0.69676	-1.42236
154.2801	-0.67942	3.47383	-2.43174	0.69656	-1.42248
154.3139	-0.6795	3.4737	-2.43215	0.69644	-1.42271
154.3474	-0.67962	3.47358	-2.43249	0.69653	-1.42295
154.38	-0.6797	3.47347	-2.43285	0.69666	-1.42316

154.4132	-0.6797	3.47336	-2.43316	0.69674	-1.42329
154.4472	-0.6797	3.47327	-2.43334	0.6968	-1.42332
154.4803	-0.67968	3.47323	-2.43341	0.69665	-1.42333
154.5137	-0.67968	3.47314	-2.43344	0.69654	-1.42337
154.5466	-0.67972	3.47304	-2.43352	0.69655	-1.4235
154.5807	-0.67976	3.47297	-2.43365	0.69662	-1.42361
154.6139	-0.67982	3.47297	-2.43383	0.69673	-1.42367
154.6474	-0.67996	3.47303	-2.43407	0.69683	-1.4237
154.6804	-0.68004	3.47324	-2.43434	0.69694	-1.42371
154.7132	-0.68008	3.47347	-2.43463	0.69695	-1.42373
154.746	-0.68012	3.47366	-2.43484	0.6969	-1.42388
154.7806	-0.68012	3.47379	-2.435	0.69684	-1.42411
154.8132	-0.68018	3.4739	-2.43511	0.69682	-1.42449
154.847	-0.68026	3.47391	-2.43524	0.69683	-1.42501
154.8796	-0.68038	3.47382	-2.43537	0.69687	-1.42526
154.9138	-0.68058	3.47372	-2.4355	0.69692	-1.42532
154.9466	-0.68082	3.47364	-2.43557	0.69695	-1.4253
154.9806	-0.68098	3.47363	-2.43568	0.69699	-1.42528
155.0127	-0.6811	3.47372	-2.43584	0.69703	-1.42527
155.0463	-0.68116	3.47401	-2.43615	0.697-1.42539	
155.0793	-0.68122	3.47442	-2.4368	0.69686	-1.42557
155.1134	-0.6813	3.47514	-2.43721	0.69676	-1.42588
155.1458	-0.68136	3.4761	-2.43745	0.69673	-1.42622
155.1798	-0.68144	3.47708	-2.43765	0.6967	-1.42642
155.2124	-0.68146	3.47786	-2.43777	0.69669	-1.42655
155.2464	-0.68146	3.47834	-2.43788	0.69665	-1.42665
155.2793	-0.68146	3.47897	-2.438	0.69665	-1.4267
155.3126	-0.68148	3.47953	-2.43817	0.69668	-1.42675
155.347	-0.68154	3.47978	-2.43839	0.69673	-1.42678
155.3798	-0.68162	3.48012	-2.43863	0.69681	-1.4268

155.4126	-0.6818	3.48089	-2.43882	0.69687	-1.42688
155.4455	-0.6819	3.48216	-2.43897	0.69705	-1.42706
155.4786	-0.68196	3.48351	-2.43904	0.69733	-1.42732
155.512	-0.68202	3.48422	-2.43912	0.69742	-1.42752
155.5461	-0.68204	3.48486	-2.43923	0.69737	-1.42772
155.5798	-0.68208	3.48594	-2.43939	0.69694	-1.42794
155.6132	-0.68214	3.48855	-2.43964	0.69607	-1.42814
155.6473	-0.68218	3.49009	-2.43993	0.69579	-1.4283
155.6799	-0.68224	3.49037	-2.44012	0.69583	-1.42843
155.7123	-0.6823	3.49059	-2.44019	0.69604	-1.42855
155.7454	-0.68238	3.49103	-2.44022	0.6963	-1.42867
155.7789	-0.68248	3.49231	-2.44031	0.6964	-1.42896
155.8124	-0.68256	3.4936	-2.44047	0.69637	-1.42925
155.8457	-0.68262	3.49435	-2.44074	0.69634	-1.42961
155.8797	-0.68266	3.49456	-2.44093	0.69627	-1.42996
155.9138	-0.68272	3.49473	-2.44106	0.69625	-1.43012
155.9465	-0.68278	3.49492	-2.44117	0.69622	-1.43022
155.9792	-0.68282	3.49514	-2.44128	0.69622	-1.43036
156.0122	-0.68286	3.49537	-2.4414	0.69624	-1.43053
156.0456	-0.68288	3.4958	-2.44157	0.69623	-1.43074
156.0794	-0.6829	3.4961	-2.44177	0.69614	-1.43086
156.113	-0.68296	3.49628	-2.44206	0.69604	-1.43093
156.1464	-0.68318	3.49638	-2.44232	0.69598	-1.431
156.18	-0.68342	3.49612	-2.44264	0.69602	-1.43115
156.2129	-0.68352	3.49588	-2.44298	0.69624	-1.43161
156.2462	-0.6836	3.49584	-2.44351	0.69656	-1.43228
156.2796	-0.68362	3.4958	-2.44419	0.69667	-1.43268
156.3128	-0.68364	3.49574	-2.44442	0.69667	-1.43281
156.346	-0.68366	3.4955	-2.44459	0.69662	-1.43288
156.3802	-0.68372	3.49536	-2.44472	0.69636	-1.43295

156.4133	-0.6838	3.49536	-2.44491	0.6964	-1.43304
156.4458	-0.68388	3.49543	-2.44527	0.69666	-1.43315
156.4788	-0.68396	3.49555	-2.4458	0.69679	-1.4333
156.5135	-0.68402	3.49562	-2.44613	0.69683	-1.43344
156.5465	-0.68406	3.49556	-2.44635	0.69673	-1.43374
156.58	-0.68408	3.49541	-2.44646	0.69634	-1.43431
156.6127	-0.68412	3.49524	-2.44652	0.6963	-1.43542
156.6462	-0.68416	3.49518	-2.44663	0.69631	-1.43605
156.6799	-0.68422	3.49523	-2.44678	0.69638	-1.43622
156.7121	-0.68428	3.49528	-2.44695	0.69646	-1.43634
156.7455	-0.68436	3.49532	-2.44717	0.69646	-1.43648
156.7803	-0.68446	3.49532	-2.44738	0.6964	-1.43662
156.8124	-0.68452	3.49533	-2.44752	0.69627	-1.43684
156.8472	-0.68458	3.49539	-2.44759	0.69625	-1.43715
156.8798	-0.68462	3.49541	-2.44769	0.69643	-1.43739
156.912	-0.68464	3.49539	-2.44788	0.69658	-1.43762
156.9454	-0.68468	3.49537	-2.44827	0.69664	-1.43784
156.9787	-0.68478	3.49531	-2.44923	0.69647	-1.438
157.0111	-0.68496	3.49534	-2.44985	0.69597	-1.43811
157.0446	-0.68532	3.49542	-2.45051	0.69552	-1.4382
157.079	-0.68546	3.49546	-2.45147	0.69515	-1.43833
157.113	-0.68554	3.49553	-2.45172	0.69501	-1.43854
157.1461	-0.68556	3.49563	-2.45191	0.69501	-1.43894
157.1798	-0.68554	3.49574	-2.45204	0.69508	-1.4392
157.2111	-0.68556	3.496-2.45211	0.69517	-1.43942	
157.2446	-0.6856	3.49665	-2.45226	0.69515	-1.43961
157.2795	-0.68562	3.49743	-2.45242	0.69492	-1.4398
157.3118	-0.68568	3.49831	-2.45255	0.69433	-1.43992
157.3466	-0.68574	3.49992	-2.45274	0.69412	-1.44005
157.3786	-0.6859	3.50097	-2.45293	0.69402	-1.44017

157.4124	-0.68628	3.50258	-2.45311	0.69397	-1.44033
157.4454	-0.68646	3.5057	-2.45334	0.69394	-1.44066
157.4788	-0.68648	3.51061	-2.45362	0.69386	-1.44106
157.511	-0.68644	3.51475	-2.45383	0.69381	-1.44129
157.5451	-0.68638	3.51634	-2.45399	0.69378	-1.44164
157.5775	-0.68636	3.51708	-2.45406	0.69371	-1.44193
157.6121	-0.68638	3.51743	-2.45409	0.69362	-1.44215
157.646	-0.68644	3.51767	-2.4541	0.6933	-1.4424
157.679	-0.6865	3.51813	-2.45422	0.69307	-1.4426
157.7121	-0.68656	3.51858	-2.45441	0.69298	-1.44283
157.7452	-0.68658	3.51916	-2.45476	0.69292	-1.4431
157.7788	-0.68662	3.51971	-2.45542	0.69292	-1.44331
157.8125	-0.68666	3.51993	-2.45561	0.69295	-1.4435
157.8464	-0.68686	3.52008	-2.45576	0.69292	-1.44367
157.8784	-0.68732	3.52026	-2.45587	0.69278	-1.44383
157.9111	-0.6876	3.52048	-2.45598	0.69221	-1.44413
157.9452	-0.68778	3.52081	-2.45617	0.69093	-1.4446
157.979	-0.68784	3.52119	-2.45635	0.69027	-1.44483
158.0119	-0.68786	3.52139	-2.45647	0.69004	-1.44508
158.0449	-0.6879	3.52156	-2.45658	0.68998	-1.44533
158.0782	-0.68796	3.52167	-2.4567	0.68999	-1.44552
158.112	-0.68804	3.52169	-2.45686	0.69001	-1.44577
158.1453	-0.68814	3.52168	-2.45704	0.69014	-1.44598
158.1787	-0.6882	3.52154	-2.4573	0.69021	-1.44614
158.2117	-0.68826	3.52149	-2.45756	0.69024	-1.44635
158.2444	-0.68824	3.5215	-2.45775	0.69028	-1.44661
158.2779	-0.68814	3.52155	-2.45795	0.69026	-1.44691
158.3109	-0.68812	3.52155	-2.45817	0.6902	-1.44726
158.3453	-0.68818	3.52136	-2.45836	0.69008	-1.44749
158.3783	-0.68872	3.5209	-2.45856	0.68972	-1.44764

158.4112	-0.68932	3.52053	-2.45878	0.68964	-1.44781
158.4456	-0.68948	3.52017	-2.45901	0.6896	-1.44794
158.4782	-0.6895	3.52	-2.45934	0.68956	-1.44807
158.5126	-0.6895	3.51991	-2.45965	0.68952	-1.44822
158.5461	-0.68954	3.51989	-2.4599	0.68947	-1.4484
158.5787	-0.68962	3.51988	-2.46006	0.68949	-1.44875
158.6123	-0.6897	3.51986	-2.46022	0.68957	-1.44949
158.6456	-0.68974	3.51973	-2.46034	0.68964	-1.44978
158.6787	-0.68978	3.51958	-2.46047	0.6896	-1.44997
158.7111	-0.68984	3.51953	-2.4606	0.68949	-1.45006
158.7446	-0.6899	3.51959	-2.46075	0.68927	-1.45017
158.778	-0.68994	3.51979	-2.46091	0.68916	-1.45047
158.8117	-0.68998	3.51991	-2.4611	0.68915	-1.45097
158.8466	-0.69006	3.51994	-2.46126	0.68912	-1.45133
158.8784	-0.69016	3.51988	-2.46143	0.68907	-1.45167
158.9117	-0.69032	3.51977	-2.4616	0.68906	-1.45194
158.9448	-0.69038	3.51954	-2.46176	0.68909	-1.4522
158.9783	-0.69042	3.5194	-2.46194	0.68915	-1.4525
159.0123	-0.69046	3.51929	-2.46215	0.68921	-1.45275
159.045	-0.6905	3.51921	-2.46236	0.68923	-1.45303
159.0782	-0.69054	3.51917	-2.46256	0.68921	-1.45335
159.1114	-0.69058	3.51917	-2.46275	0.6892	-1.45368
159.1454	-0.69066	3.51923	-2.46291	0.68924	-1.45405
159.1784	-0.69076	3.51934	-2.46308	0.68926	-1.45438
159.2116	-0.69084	3.51974	-2.4633	0.68929	-1.45465
159.2442	-0.6909	3.52043	-2.46347	0.68935	-1.45489
159.2783	-0.69094	3.52117	-2.46356	0.68944	-1.45516
159.3124	-0.69096	3.52199	-2.46368	0.68963	-1.45553
159.3454	-0.69098	3.52234	-2.46373	0.68982	-1.45604
159.3795	-0.691	3.52249	-2.46388	0.69009	-1.45673

159.4111	-0.69104	3.52289	-2.46412	0.69015	-1.45702
159.4449	-0.6911	3.52377	-2.46437	0.69014	-1.45723
159.4789	-0.69116	3.52461	-2.46459	0.69013	-1.45746
159.5119	-0.69124	3.52609	-2.46479	0.69015	-1.45772
159.545	-0.6913	3.52773	-2.46489	0.69018	-1.45802
159.5779	-0.6914	3.52833	-2.46498	0.69014	-1.45832
159.6119	-0.69144	3.52859	-2.46511	0.69	-1.4585
159.6456	-0.69148	3.52881	-2.46528	0.68991	-1.45863
159.6782	-0.6915	3.52891	-2.46563	0.68985	-1.4588
159.7111	-0.69154	3.52904	-2.46612	0.6899	-1.45954
159.7449	-0.69162	3.52933	-2.46639	0.69	-1.46103
159.7781	-0.69172	3.52978	-2.46658	0.69018	-1.46232
159.8115	-0.69182	3.53049	-2.46673	0.69039	-1.46282
159.8452	-0.69188	3.53146	-2.46691	0.69045	-1.46303
159.8791	-0.69192	3.53177	-2.46713	0.69031	-1.46341
159.9122	-0.69198	3.53199	-2.46738	0.68983	-1.4646
159.9454	-0.69202	3.53218	-2.46761	0.68968	-1.46499
159.9783	-0.69208	3.53232	-2.46784	0.68974	-1.46524
160.0108	-0.69216	3.53247	-2.46809	0.68987	-1.46543
160.0456	-0.6922	3.53265	-2.46837	0.68999	-1.46561
160.0773	-0.69224	3.53279	-2.46862	0.69001	-1.46592
160.1113	-0.6923	3.53269	-2.46881	0.68976	-1.46651
160.1454	-0.69236	3.53193	-2.46896	0.68895	-1.46706
160.1786	-0.69242	3.53141	-2.46909	0.68847	-1.46736
160.2118	-0.69248	3.53137	-2.46927	0.68833	-1.46763
160.2458	-0.69252	3.53151	-2.46951	0.68829	-1.46789
160.2778	-0.69262	3.53183	-2.46977	0.68827	-1.46818
160.3112	-0.69286	3.532	-2.47008	0.68831	-1.46846
160.3451	-0.6931	3.53211	-2.47036	0.68828	-1.46869
160.3784	-0.69318	3.5321	-2.47071	0.68821	-1.46886

160.4109	-0.69324	3.53202	-2.47093	0.68802	-1.46898
160.4449	-0.69332	3.53202	-2.47108	0.68708	-1.46918
160.4786	-0.6934	3.53205	-2.47119	0.68562	-1.46948
160.5109	-0.69344	3.53216	-2.4713	0.68503	-1.46989
160.5444	-0.69346	3.53228	-2.47147	0.6847	-1.47037
160.5779	-0.69348	3.53228	-2.4717	0.68415	-1.47068
160.6116	-0.69352	3.53217	-2.47193	0.684-1.47097	
160.6447	-0.69358	3.53208	-2.4721	0.68395	-1.47126
160.6778	-0.69368	3.53195	-2.47226	0.68408	-1.47144
160.7112	-0.6937	3.53187	-2.47243	0.68431	-1.47164
160.7447	-0.69372	3.53188	-2.47261	0.68435	-1.47188
160.7778	-0.69376	3.5319	-2.47281	0.68424	-1.47231
160.8114	-0.69382	3.53194	-2.47303	0.6841	-1.47271
160.8451	-0.6939	3.532-2.4733	0.684-1.473		
160.8779	-0.69396	3.53197	-2.47355	0.68399	-1.47314
160.9115	-0.69398	3.53185	-2.47374	0.68411	-1.47329
160.9456	-0.694	3.53184	-2.47383	0.68425	-1.47371
160.9786	-0.69404	3.53192	-2.47396	0.68438	-1.47526
161.0112	-0.6941	3.53221	-2.47417	0.68457	-1.47665
161.0449	-0.69418	3.53278	-2.47445	0.68467	-1.47706
161.078	-0.69426	3.53305	-2.47486	0.68456	-1.47728
161.1116	-0.69434	3.5332	-2.47512	0.68407	-1.47747
161.1464	-0.69438	3.53375	-2.47531	0.68358	-1.4777
161.1784	-0.69442	3.53486	-2.47557	0.68348	-1.47797
161.2113	-0.69448	3.53597	-2.47588	0.6836	-1.47827
161.2444	-0.6946	3.53734	-2.47612	0.68381	-1.47857
161.2782	-0.69496	3.5386	-2.47637	0.6838	-1.47884
161.3115	-0.69508	3.53991	-2.47655	0.68375	-1.4791
161.3454	-0.69514	3.54122	-2.47679	0.68362	-1.47935
161.378	-0.69518	3.54189	-2.47708	0.68357	-1.47963

161.4122	-0.69522	3.5423	-2.47735	0.68358	-1.4801
161.4445	-0.6953	3.54282	-2.47767	0.68355	-1.48073
161.4785	-0.69536	3.54463	-2.478	0.68357	-1.48129
161.5118	-0.6954	3.54735	-2.47829	0.68366	-1.48199
161.5446	-0.69542	3.54943	-2.47856	0.68375	-1.48249
161.5781	-0.69544	3.54997	-2.47883	0.6838	-1.4829
161.6124	-0.69548	3.5501	-2.47902	0.68379	-1.48312
161.6447	-0.69558	3.5502	-2.47919	0.68332	-1.48329
161.6782	-0.6958	3.55049	-2.47941	0.68306	-1.48351
161.7112	-0.69602	3.55174	-2.47958	0.6831	-1.48379
161.7441	-0.69614	3.55547	-2.47981	0.68322	-1.48405
161.7781	-0.69616	3.55741	-2.48005	0.68342	-1.48428
161.8113	-0.69616	3.55782	-2.4802	0.68352	-1.48441
161.8447	-0.69618	3.55791	-2.48032	0.68343	-1.48458
161.8777	-0.69622	3.55794	-2.4805	0.68333	-1.48487
161.9115	-0.69638	3.55796	-2.48081	0.68326	-1.48516
161.9449	-0.69658	3.55803	-2.48173	0.68323	-1.4855
161.9781	-0.69666	3.5581	-2.48235	0.68335	-1.48582
162.0114	-0.69672	3.55817	-2.48263	0.68364	-1.48612
162.0434	-0.69676	3.55825	-2.48283	0.68378	-1.48652
162.0772	-0.6968	3.55834	-2.48297	0.68388	-1.48691
162.1108	-0.69694	3.55844	-2.48306	0.68386	-1.48724
162.144	-0.6971	3.55848	-2.48314	0.68381	-1.48756
162.1767	-0.69718	3.5585	-2.48327	0.68375	-1.48783
162.2106	-0.6972	3.55847	-2.48349	0.6837	-1.48815
162.2437	-0.69718	3.55831	-2.48387	0.6837	-1.48845
162.2783	-0.6972	3.55802	-2.48479	0.6837	-1.48872
162.3116	-0.69724	3.55784	-2.48546	0.68375	-1.48903
162.3444	-0.69734	3.55777	-2.48578	0.68392	-1.4893
162.3776	-0.69742	3.55783	-2.48604	0.68394	-1.48951

162.4113	-0.69748	3.5579	-2.48626	0.68396	-1.48978
162.4446	-0.69752	3.55785	-2.48643	0.68403	-1.49002
162.4783	-0.69756	3.55755	-2.48659	0.68403	-1.49025
162.512	-0.69758	3.55738	-2.48676	0.6841	-1.4905
162.5452	-0.69756	3.55735	-2.48698	0.68418	-1.49071
162.5783	-0.69754	3.5573	-2.48721	0.68421	-1.49093
162.6122	-0.69756	3.55721	-2.4876	0.68426	-1.49114
162.645	-0.69762	3.55712	-2.48861	0.6843	-1.49128
162.6778	-0.69774	3.55704	-2.48915	0.68435	-1.49149
162.7118	-0.69782	3.55706	-2.48945	0.68456	-1.49172
162.7449	-0.6979	3.55712	-2.48973	0.68468	-1.49211
162.7785	-0.698	3.55719	-2.48997	0.68476	-1.49266
162.812	-0.69812	3.55722	-2.49024	0.68482	-1.4929
162.8456	-0.69824	3.55726	-2.49053	0.68486	-1.49307
162.8777	-0.69842	3.55743	-2.49071	0.68498	-1.49317
162.9121	-0.69858	3.55785	-2.49084	0.68527	-1.49329
162.9456	-0.69866	3.55864	-2.49099	0.68541	-1.49343
162.978	-0.69872	3.56014	-2.49127	0.6855	-1.49361
163.0119	-0.69878	3.56209	-2.49258	0.68557	-1.49382
163.0449	-0.69882	3.56372	-2.49469	0.68564	-1.49403
163.0786	-0.69886	3.56491	-2.49664	0.68571	-1.49426
163.1118	-0.69892	3.56522	-2.49699	0.68584	-1.49453
163.1454	-0.69896	3.56543	-2.4971	0.68596	-1.49479
163.1774	-0.69902	3.56567	-2.49715	0.68604	-1.49507
163.211	-0.6991	3.56609	-2.49725	0.68613	-1.49533
163.2449	-0.69918	3.56626	-2.49736	0.68624	-1.49556
163.2783	-0.69928	3.56634	-2.49761	0.68636	-1.49569
163.3121	-0.69938	3.56637	-2.49788	0.68646	-1.49579
163.3453	-0.69946	3.56645	-2.4983	0.68651	-1.49585
163.3785	-0.69952	3.56656	-2.49854	0.6865	-1.49598

163.4121	-0.69958	3.56668	-2.49877	0.68652	-1.49623
163.4453	-0.69962	3.56678	-2.49896	0.68664	-1.49655
163.4783	-0.69974	3.56683	-2.49919	0.6868	-1.49692
163.5121	-0.69982	3.56687	-2.49946	0.68696	-1.49727
163.5449	-0.69992	3.56688	-2.49971	0.68708	-1.49753
163.5779	-0.7	3.56689	-2.49996	0.68711	-1.49777
163.6115	-0.70002	3.56686	-2.50021	0.6871	-1.49795
163.6453	-0.70004	3.56687	-2.50058	0.6872	-1.49809
163.6781	-0.70006	3.56689	-2.50136	0.68793	-1.49827
163.7118	-0.7001	3.56687	-2.50178	0.68881	-1.49847
163.7446	-0.7002	3.56683	-2.50207	0.68906	-1.49869
163.7785	-0.70032	3.56677	-2.50227	0.6891	-1.49891
163.8108	-0.70036	3.56669	-2.50243	0.68903	-1.49908
163.8447	-0.70036	3.56663	-2.50262	0.68903	-1.49926
163.878	-0.7004	3.56658	-2.50287	0.6891	-1.49944
163.911	-0.70046	3.56648	-2.50323	0.68917	-1.49965
163.9451	-0.7007	3.56644	-2.50397	0.68928	-1.49983
163.9781	-0.70094	3.56642	-2.50449	0.68938	-1.49998
164.0116	-0.70104	3.56639	-2.50479	0.68961	-1.50012
164.0443	-0.7011	3.56647	-2.50505	0.68977	-1.50032
164.0774	-0.70114	3.56676	-2.50534	0.68992	-1.50049
164.1115	-0.70116	3.5676	-2.50562	0.69007	-1.50061
164.1446	-0.7012	3.56889	-2.50592	0.69019	-1.50072
164.1782	-0.70124	3.56948	-2.50623	0.69028	-1.50087
164.2115	-0.70132	3.57013	-2.50657	0.69037	-1.50168
164.2443	-0.70136	3.57091	-2.50692	0.69045	-1.50397
164.2785	-0.70138	3.5718	-2.50724	0.69059	-1.50546
164.3118	-0.70142	3.57256	-2.50752	0.69073	-1.50576
164.3459	-0.70148	3.57304	-2.50776	0.6908	-1.50591
164.3781	-0.70156	3.57341	-2.50802	0.69081	-1.50601

164.4114	-0.7017	3.57366	-2.50843	0.69085	-1.50615
164.4451	-0.70178	3.5739	-2.50886	0.69097	-1.50633
164.4781	-0.70186	3.57411	-2.50915	0.69123	-1.50652
164.5116	-0.7019	3.57431	-2.50943	0.69148	-1.50668
164.5451	-0.70196	3.5745	-2.50961	0.69165	-1.50683
164.5782	-0.702	3.57466	-2.50974	0.69175	-1.50695
164.6119	-0.70204	3.57475	-2.50987	0.69177	-1.50707
164.6444	-0.7021	3.57484	-2.51008	0.69176	-1.50717
164.6784	-0.70216	3.57489	-2.51034	0.69179	-1.5073
164.7109	-0.7022	3.57492	-2.51059	0.69186	-1.50746
164.7448	-0.70222	3.57494	-2.51081	0.69199	-1.50764
164.778	-0.70226	3.57494	-2.51103	0.69209	-1.50786
164.8117	-0.70232	3.57492	-2.51129	0.69219	-1.50808
164.8455	-0.7024	3.57495	-2.51159	0.69228	-1.50827
164.8785	-0.70252	3.57499	-2.51182	0.69238	-1.50839
164.9113	-0.70262	3.57508	-2.51202	0.69242	-1.50844
164.9446	-0.70272	3.57514	-2.51221	0.69245	-1.50856
164.9782	-0.7028	3.57523	-2.51252	0.69253	-1.50892
165.0114	-0.70288	3.57532	-2.51308	0.69268	-1.50949
165.0448	-0.70298	3.57538	-2.51427	0.69293	-1.51019
165.0784	-0.7031	3.57547	-2.51724	0.69315	-1.51055
165.1125	-0.70318	3.57555	-2.51983	0.69331	-1.51067
165.145	-0.70324	3.57564	-2.5207	0.69349	-1.51081
165.1774	-0.70328	3.57612	-2.52099	0.69368	-1.51106
165.2111	-0.70334	3.57743	-2.52115	0.69383	-1.51163
165.2451	-0.70344	3.57841	-2.52131	0.69389	-1.51234
165.2785	-0.70352	3.5795	-2.52153	0.69389	-1.51254
165.312	-0.70358	3.5801	-2.52179	0.6939	-1.5127
165.345	-0.70366	3.58033	-2.522	0.69395	-1.51287
165.3782	-0.70374	3.58103	-2.52221	0.69396	-1.51305

165.412	-0.70382	3.58201	-2.52252	0.69398	-1.51325
165.4449	-0.7039	3.58326	-2.52278	0.69407	-1.51342
165.4784	-0.70396	3.58495	-2.52311	0.69434	-1.51356
165.5114	-0.70402	3.58603	-2.52342	0.69527	-1.51371
165.5452	-0.70412	3.58636	-2.52363	0.69568	-1.51391
165.5781	-0.70422	3.58657	-2.52387	0.69576	-1.51412
165.612	-0.7043	3.58667	-2.52424	0.69573	-1.51433
165.645	-0.70438	3.58678	-2.5253	0.69574	-1.51454
165.6771	-0.70444	3.58682	-2.52743	0.69594	-1.5148
165.7123	-0.7045	3.58679	-2.52836	0.69673	-1.51504
165.7442	-0.70456	3.58673	-2.52868	0.69698	-1.51529
165.7775	-0.70462	3.58676	-2.52886	0.69709	-1.51552
165.8112	-0.70466	3.58702	-2.52916	0.69707	-1.51568
165.8439	-0.70474	3.58766	-2.5298	0.69705	-1.51582
165.8782	-0.70478	3.58783	-2.53071	0.69703	-1.51595
165.9119	-0.7048	3.58786	-2.53099	0.69707	-1.51612
165.9449	-0.7048	3.58778	-2.5311	0.69714	-1.51633
165.9784	-0.70478	3.58766	-2.53115	0.69721	-1.51648
166.0114	-0.70478	3.58767	-2.53133	0.69732	-1.5166
166.0444	-0.70482	3.58771	-2.53184	0.69751	-1.51667
166.0778	-0.70488	3.5877	-2.53298	0.69762	-1.51676
166.111	-0.705	3.58767	-2.53327	0.6977	-1.51696
166.1446	-0.70508	3.58768	-2.53346	0.69773	-1.51723
166.1779	-0.70518	3.58767	-2.53367	0.69774	-1.51744
166.211	-0.70524	3.58768	-2.5339	0.69779	-1.51764
166.2448	-0.70526	3.58769	-2.53421	0.69786	-1.51779
166.2779	-0.70528	3.58769	-2.53454	0.69785	-1.51795
166.3126	-0.70528	3.58777	-2.5349	0.69788	-1.51815
166.3455	-0.70528	3.58791	-2.53527	0.69796	-1.51828
166.3785	-0.70534	3.58796	-2.53608	0.69812	-1.51836

166.4111	-0.70558	3.58797	-2.5366	0.69844	-1.51842
166.4441	-0.7058	3.58796	-2.53692	0.69858	-1.51845
166.4783	-0.7059	3.58804	-2.53728	0.69867	-1.51859
166.5121	-0.70592	3.58889	-2.53759	0.69876	-1.51895
166.5455	-0.70592	3.59157	-2.53789	0.69887	-1.51938
166.5783	-0.70594	3.59413	-2.53818	0.69902	-1.51968
166.6117	-0.70598	3.59504	-2.53838	0.69918	-1.51983
166.6457	-0.70602	3.59552	-2.5386	0.69931	-1.51984
166.6787	-0.70604	3.59569	-2.53896	0.69941	-1.51998
166.7118	-0.70606	3.59575	-2.53988	0.69954	-1.5202
166.7441	-0.70612	3.59581	-2.54071	0.69971	-1.52042
166.7778	-0.7062	3.59591	-2.54109	0.69984	-1.52068
166.8112	-0.70636	3.59679	-2.5414	0.69992	-1.52083
166.8456	-0.7067	3.59835	-2.54167	0.69998	-1.5209
166.8786	-0.70704	3.59902	-2.54205	0.70011	-1.521
166.9115	-0.70716	3.59912	-2.54254	0.70027	-1.52112
166.9452	-0.7072	3.59916	-2.5429	0.70045	-1.52129
166.9782	-0.7072	3.59936	-2.54335	0.70059	-1.52156
167.0112	-0.70712	3.5999	-2.54364	0.70069	-1.5218
167.0444	-0.70702	3.60083	-2.54385	0.70078	-1.52205
167.0775	-0.70702	3.60116	-2.54401	0.7009	-1.52226
167.1117	-0.70706	3.60129	-2.54414	0.701-1.52246	
167.1452	-0.70724	3.60144	-2.54434	0.70111	-1.52267
167.178	-0.70738	3.60162	-2.54463	0.70118	-1.52285
167.2113	-0.70742	3.60176	-2.545	0.70127	-1.523
167.244	-0.70744	3.60181	-2.54534	0.70135	-1.52313
167.2784	-0.70744	3.60173	-2.54558	0.70139	-1.52323
167.3126	-0.70742	3.60156	-2.54583	0.70141	-1.52329
167.3457	-0.70744	3.60151	-2.54613	0.70141	-1.52332
167.3789	-0.7075	3.60155	-2.54638	0.70147	-1.5234

167.4126	-0.70766	3.60159	-2.54659	0.70164	-1.52356
167.4454	-0.70794	3.60164	-2.54675	0.70209	-1.52368
167.4786	-0.70798	3.6017	-2.54691	0.70234	-1.52376
167.5118	-0.708	3.60175	-2.54715	0.70259	-1.52382
167.5454	-0.70798	3.60184	-2.54746	0.70271	-1.52393
167.5782	-0.708	3.60192	-2.54786	0.70281	-1.52441
167.6122	-0.70802	3.60199	-2.54818	0.70292	-1.52529
167.6456	-0.70804	3.60204	-2.54849	0.70304	-1.52595
167.6791	-0.70808	3.60204	-2.5489	0.70315	-1.52621
167.7126	-0.7081	3.602-2.54935	0.7033	-1.52642	
167.7449	-0.70812	3.60192	-2.54978	0.70343	-1.52655
167.7781	-0.70814	3.60182	-2.55052	0.70348	-1.52666
167.8116	-0.70812	3.60179	-2.55101	0.70353	-1.52684
167.8451	-0.7081	3.60189	-2.55132	0.70361	-1.52706
167.8794	-0.70812	3.60239	-2.55166	0.70375	-1.52728
167.9124	-0.70818	3.60316	-2.55218	0.70398	-1.5275
167.946	-0.70824	3.60454	-2.55285	0.70416	-1.52764
167.9788	-0.7084	3.60499	-2.55472	0.70428	-1.52775
168.0116	-0.70846	3.60515	-2.55638	0.70438	-1.52788
168.0451	-0.7085	3.60528	-2.55681	0.70442	-1.52802
168.0786	-0.70856	3.60539	-2.5571	0.70444	-1.52815
168.1116	-0.70858	3.6056	-2.55735	0.70443	-1.52842
168.1454	-0.7086	3.60612	-2.55761	0.70445	-1.52879
168.1778	-0.70862	3.60661	-2.55792	0.7045	-1.52927
168.212	-0.70864	3.60753	-2.5583	0.70457	-1.52968
168.2456	-0.70866	3.60838	-2.55873	0.70467	-1.52987
168.2785	-0.70868	3.60936	-2.5591	0.70479	-1.53
168.3121	-0.70874	3.6111	-2.5594	0.70504	-1.53016
168.3453	-0.70882	3.61362	-2.55971	0.70522	-1.53027
168.3795	-0.7089	3.61451	-2.56006	0.70533	-1.53036

168.4122	-0.70894	3.61491	-2.5604	0.70545	-1.53047
168.4454	-0.70894	3.61505	-2.56071	0.7055	-1.5306
168.4775	-0.70892	3.61512	-2.56099	0.70558	-1.5308
168.5111	-0.70892	3.61524	-2.56137	0.70572	-1.53122
168.5444	-0.70892	3.61539	-2.56195	0.70582	-1.53174
168.5785	-0.70894	3.61556	-2.56223	0.7059	-1.53201
168.6124	-0.70894	3.6157	-2.56243	0.70591	-1.53224
168.6449	-0.70894	3.61583	-2.56257	0.70586	-1.53247
168.6789	-0.70896	3.61591	-2.56271	0.70584	-1.53262
168.712	-0.709	3.61595	-2.56296	0.70593	-1.53273
168.7442	-0.70904	3.61595	-2.56364	0.70612	-1.53284
168.7779	-0.70906	3.61597	-2.56558	0.70631	-1.53296
168.8118	-0.7091	3.61596	-2.56742	0.70646	-1.53313
168.8458	-0.70912	3.61598	-2.56827	0.70653	-1.5333
168.8784	-0.70916	3.61599	-2.56868	0.70653	-1.53343
168.9118	-0.70922	3.61597	-2.56891	0.70653	-1.53355
168.945	-0.7093	3.61593	-2.56914	0.70662	-1.53376
168.9778	-0.70936	3.61593	-2.56941	0.70671	-1.53409
169.0119	-0.70942	3.61593	-2.56966	0.7068	-1.53457
169.045	-0.70948	3.61598	-2.56996	0.70686	-1.53483
169.0777	-0.70952	3.61624	-2.57023	0.70693	-1.53501
169.1121	-0.70954	3.61656	-2.57056	0.707-1.53515	
169.1451	-0.70956	3.61677	-2.57118	0.7071	-1.53535
169.1795	-0.70958	3.6169	-2.57158	0.70722	-1.53551
169.2118	-0.7096	3.61691	-2.57189	0.70732	-1.53564
169.246	-0.70968	3.61696	-2.57208	0.70738	-1.53583
169.2789	-0.70988	3.61715	-2.5722	0.70748	-1.53605
169.3117	-0.70998	3.6187	-2.57235	0.70753	-1.53633
169.3446	-0.71004	3.62137	-2.57256	0.70761	-1.53676
169.3781	-0.71006	3.6239	-2.57296	0.70768	-1.53713

169.4118	-0.7101	3.62542	-2.57376	0.70777	-1.53749
169.4446	-0.71014	3.62589	-2.57498	0.7079	-1.53777
169.4777	-0.7102	3.62606	-2.57619	0.70805	-1.53795
169.5114	-0.71028	3.62621	-2.57707	0.70822	-1.53814
169.5444	-0.71036	3.62631	-2.57759	0.70833	-1.5383
169.5781	-0.71042	3.62643	-2.57802	0.70843	-1.53849
169.6118	-0.71044	3.62662	-2.57864	0.70848	-1.5387
169.6446	-0.71048	3.62676	-2.57961	0.70853	-1.53883
169.678	-0.71052	3.6269	-2.58027	0.70864	-1.53887
169.7113	-0.71058	3.62692	-2.58066	0.7088	-1.53893
169.7453	-0.71066	3.62676	-2.58102	0.70896	-1.53902
169.7784	-0.71074	3.62659	-2.58139	0.70915	-1.53925
169.8115	-0.7108	3.62647	-2.58168	0.70925	-1.53981
169.8458	-0.71084	3.62638	-2.58188	0.7093	-1.54015
169.8785	-0.71086	3.62637	-2.58203	0.70931	-1.54039
169.9115	-0.71086	3.62636	-2.58216	0.70932	-1.54054
169.9446	-0.71092	3.62638	-2.58236	0.70933	-1.54074
169.9777	-0.71098	3.62639	-2.58263	0.70939	-1.54097
170.0115	-0.71102	3.62642	-2.58291	0.7094	-1.5412
170.045	-0.7111	3.62646	-2.58335	0.70946	-1.54131
170.0785	-0.71122	3.62651	-2.58405	0.70956	-1.54137
170.1112	-0.71134	3.62663	-2.58465	0.70962	-1.54147
170.145	-0.71146	3.62671	-2.58498	0.70969	-1.54166
170.178	-0.71156	3.62668	-2.58529	0.70984	-1.54193
170.2117	-0.71164	3.62662	-2.58557	0.71004	-1.54218
170.2443	-0.71172	3.62657	-2.58586	0.71032	-1.5424
170.2784	-0.7118	3.6266	-2.58618	0.7105	-1.54257
170.3109	-0.71188	3.62678	-2.58652	0.71059	-1.54277
170.3454	-0.71194	3.62697	-2.58742	0.71062	-1.54294
170.3779	-0.712	3.62705	-2.58869	0.71066	-1.54313

170.4118	-0.71206	3.62711	-2.58967	0.71075	-1.54335
170.4446	-0.71212	3.62724	-2.59007	0.71087	-1.54358
170.4779	-0.71218	3.62746	-2.59057	0.71104	-1.54374
170.5118	-0.71228	3.62852	-2.59151	0.7112	-1.54384
170.545	-0.71238	3.63028	-2.59289	0.71133	-1.54396
170.5784	-0.71246	3.63168	-2.5934	0.71138	-1.5441
170.6114	-0.71248	3.63273	-2.59377	0.71136	-1.5443
170.6446	-0.71248	3.63326	-2.59398	0.71137	-1.54453
170.6782	-0.7125	3.63349	-2.59419	0.71151	-1.54477
170.7117	-0.71274	3.63398	-2.59445	0.71163	-1.545
170.7452	-0.71326	3.63448	-2.59474	0.71176	-1.54523
170.7785	-0.71342	3.63492	-2.59504	0.71182	-1.54536
170.8116	-0.71344	3.63536	-2.59537	0.71172	-1.54548
170.8446	-0.71342	3.63564	-2.59568	0.71175	-1.54565
170.8785	-0.7133	3.63581	-2.59603	0.71187	-1.54584
170.9112	-0.71332	3.63591	-2.59678	0.712	-1.54609
170.9449	-0.7134	3.6359	-2.59774	0.71218	-1.54635
170.9786	-0.71352	3.63587	-2.59849	0.71225	-1.54659
171.0117	-0.7139	3.63596	-2.59896	0.71235	-1.54684
171.0453	-0.71402	3.6361	-2.59961	0.71245	-1.5471
171.0787	-0.71406	3.63622	-2.60051	0.71257	-1.54734
171.1116	-0.7141	3.6363	-2.60149	0.71282	-1.5475
171.1448	-0.71414	3.63612	-2.60197	0.71309	-1.54757
171.1781	-0.7142	3.63582	-2.60223	0.71316	-1.54764
171.2104	-0.71428	3.6356	-2.60245	0.7132	-1.54771
171.2443	-0.71434	3.63551	-2.6027	0.71323	-1.54778
171.2784	-0.71438	3.63552	-2.60303	0.7133	-1.54791
171.3121	-0.7144	3.6355	-2.60343	0.71338	-1.54804
171.3452	-0.71446	3.63545	-2.60381	0.71347	-1.54819
171.3784	-0.71454	3.63549	-2.6042	0.71351	-1.54838

171.4114	-0.7146	3.63556	-2.60454	0.71357	-1.54859
171.4445	-0.71462	3.63567	-2.60488	0.7137	-1.54884
171.4786	-0.71466	3.6358	-2.60519	0.71388	-1.5491
171.5108	-0.7147	3.63593	-2.6055	0.71404	-1.54929
171.5443	-0.71474	3.63633	-2.60586	0.71416	-1.54947
171.5787	-0.7148	3.63729	-2.6062	0.7142	-1.54957
171.6124	-0.71488	3.63843	-2.60652	0.71414	-1.54968
171.6453	-0.71494	3.63898	-2.60685	0.71415	-1.54997
171.6793	-0.71508	3.63949	-2.60718	0.71423	-1.55036
171.7112	-0.71528	3.64117	-2.60754	0.71438	-1.55062
171.7451	-0.71534	3.64361	-2.60792	0.7148	-1.5508
171.7782	-0.71542	3.64766	-2.60824	0.71523	-1.5508
171.8111	-0.7155	3.65034	-2.60853	0.71538	-1.55081
171.8454	-0.71556	3.65085	-2.60882	0.71547	-1.55092
171.8786	-0.71562	3.65138	-2.60912	0.71549	-1.55107
171.9113	-0.71584	3.65184	-2.60941	0.71547	-1.55126
171.9446	-0.71624	3.65199	-2.60966	0.71549	-1.55146
171.9786	-0.71666	3.65206	-2.60982	0.71556	-1.55157
172.0121	-0.71676	3.65202	-2.61004	0.71566	-1.55172
172.0459	-0.71682	3.65181	-2.61043	0.71581	-1.55186
172.0786	-0.71686	3.65178	-2.61178	0.71593	-1.552
172.1116	-0.71692	3.65181	-2.61387	0.71607	-1.55221
172.1451	-0.71698	3.65189	-2.61627	0.71623	-1.55263
172.1785	-0.71706	3.65196	-2.61719	0.71637	-1.55312
172.2108	-0.71712	3.65197	-2.61769	0.71641	-1.55344
172.2453	-0.71714	3.65197	-2.61804	0.7163	-1.55357
172.2796	-0.71718	3.65198	-2.61836	0.7162	-1.55337
172.3118	-0.71724	3.65205	-2.61864	0.71617	-1.55327
172.3444	-0.71732	3.65219	-2.61893	0.7163	-1.55333
172.3787	-0.71738	3.65264	-2.61925	0.71655	-1.55348

172.4112	-0.7174	3.65377	-2.6197	0.71696	-1.55368
172.4449	-0.71746	3.65443	-2.6201	0.71723	-1.55389
172.4783	-0.7175	3.6546	-2.62047	0.71739	-1.5541
172.5116	-0.7176	3.65473	-2.62085	0.7175	-1.55434
172.5453	-0.7178	3.65476	-2.6212	0.71759	-1.55448
172.5789	-0.71808	3.65485	-2.6216	0.7177	-1.55458
172.611	-0.71826	3.65506	-2.62218	0.71781	-1.55481
172.6447	-0.7183	3.65522	-2.62255	0.71794	-1.55595
172.6785	-0.71832	3.65564	-2.62285	0.71804	-1.55848
172.7109	-0.71826	3.65603	-2.62322	0.71817	-1.56035
172.745	-0.71824	3.65621	-2.62358	0.71828	-1.56065
172.7785	-0.7183	3.65639	-2.62395	0.71842	-1.56061
172.8112	-0.71838	3.65658	-2.62428	0.7185	-1.56044
172.8452	-0.7184	3.65677	-2.62449	0.71854	-1.56019
172.8776	-0.71846	3.65693	-2.62466	0.71854	-1.56006
172.911	-0.71852	3.65706	-2.62489	0.71862	-1.56008
172.9447	-0.7186	3.65699	-2.62524	0.71873	-1.56022
172.9774	-0.71868	3.65644	-2.62637	0.71887	-1.56054
173.011	-0.71876	3.65616	-2.62836	0.719	-1.56082
173.0444	-0.7188	3.65615	-2.63009	0.71913	-1.56108
173.0771	-0.71886	3.65629	-2.63056	0.71928	-1.56126
173.111	-0.71894	3.65699	-2.63087	0.71946	-1.56144
173.1445	-0.719	3.65727	-2.63107	0.71963	-1.56158
173.1783	-0.71906	3.65738	-2.63123	0.71974	-1.56162
173.2121	-0.71914	3.65747	-2.63139	0.71975	-1.56171
173.2446	-0.7192	3.65754	-2.6316	0.7197	-1.56192
173.2784	-0.71928	3.6576	-2.63187	0.71968	-1.56255
173.3114	-0.71936	3.65765	-2.6322	0.7197	-1.56309
173.3443	-0.71944	3.65784	-2.6325	0.71983	-1.56319
173.3784	-0.7195	3.658	-2.63272	0.71998	-1.56318

173.4117	-0.71954	3.65814	-2.6329	0.72013	-1.56322
173.4448	-0.71958	3.65829	-2.63305	0.72024	-1.5633
173.4779	-0.71964	3.65831	-2.63327	0.7203	-1.56338
173.5111	-0.71972	3.65838	-2.63355	0.72041	-1.56349
173.546	-0.71984	3.65857	-2.63381	0.72053	-1.56358
173.5782	-0.71996	3.65888	-2.63409	0.72067	-1.56371
173.6121	-0.72008	3.65936	-2.6344	0.72079	-1.56408
173.6452	-0.72022	3.66015	-2.6347	0.72089	-1.56428
173.6788	-0.72034	3.66063	-2.635	0.721-1.56443	
173.7118	-0.72044	3.66145	-2.63529	0.72112	-1.56454
173.7453	-0.72056	3.66209	-2.6356	0.72127	-1.56458
173.7786	-0.72064	3.66264	-2.63593	0.72137	-1.56466
173.8116	-0.7207	3.66302	-2.63684	0.72143	-1.56482
173.8452	-0.7207	3.66325	-2.63881	0.72155	-1.56503
173.8784	-0.72072	3.66336	-2.63998	0.72166	-1.56522
173.9114	-0.72076	3.66345	-2.64092	0.7218	-1.56536
173.9448	-0.72088	3.66356	-2.64129	0.72191	-1.56548
173.9782	-0.72122	3.66374	-2.64144	0.72201	-1.56562
174.0112	-0.72146	3.66395	-2.64158	0.72212	-1.56574
174.0447	-0.72154	3.6641	-2.64186	0.72216	-1.56586
174.0782	-0.72164	3.66423	-2.64302	0.72226	-1.56594
174.1118	-0.7217	3.66436	-2.64565	0.72236	-1.566
174.1445	-0.72174	3.66455	-2.64632	0.7224	-1.5661
174.1786	-0.7218	3.66485	-2.64662	0.72256	-1.56628
174.2118	-0.72184	3.66515	-2.64687	0.72272	-1.56651
174.2442	-0.72188	3.66537	-2.64717	0.72282	-1.56671
174.2778	-0.72194	3.66543	-2.64773	0.72297	-1.56682
174.312	-0.72202	3.66488	-2.64809	0.7231	-1.56685
174.3442	-0.7221	3.66431	-2.64827	0.72319	-1.56692
174.3778	-0.72222	3.66433	-2.64838	0.72346	-1.56707

174.4116	-0.72234	3.6645	-2.64854	0.72363	-1.56728
174.4442	-0.72242	3.6647	-2.64878	0.72374	-1.56764
174.4783	-0.72254	3.66506	-2.64902	0.72382	-1.56776
174.5112	-0.72264	3.66552	-2.6493	0.72377	-1.56785
174.545	-0.72272	3.66661	-2.64957	0.72367	-1.56793
174.5782	-0.72278	3.6682	-2.64981	0.72364	-1.56808
174.6117	-0.72286	3.66962	-2.65003	0.72357	-1.56829
174.6453	-0.7229	3.66997	-2.6502	0.72357	-1.56854
174.6787	-0.72298	3.67011	-2.65035	0.72369	-1.5687
174.7121	-0.72306	3.67017	-2.6506	0.72381	-1.56877
174.7452	-0.72316	3.67021	-2.65088	0.72388	-1.56882
174.7774	-0.72354	3.67032	-2.65135	0.72396	-1.56892
174.811	-0.7237	3.67048	-2.65165	0.72405	-1.56908
174.8452	-0.72378	3.67066	-2.65191	0.72412	-1.56918
174.8788	-0.72384	3.67088	-2.65217	0.72434	-1.56922
174.9123	-0.72388	3.67106	-2.65249	0.7245	-1.56924
174.9451	-0.72392	3.67119	-2.65344	0.7245	-1.56928
174.9779	-0.72396	3.67131	-2.65506	0.72454	-1.56945
175.0118	-0.724	3.67143	-2.65579	0.72454	-1.56965
175.0449	-0.72406	3.67157	-2.65617	0.72453	-1.56979
175.0778	-0.72414	3.67177	-2.6564	0.7247	-1.56985
175.1114	-0.72426	3.67195	-2.65661	0.72486	-1.56987
175.1444	-0.72436	3.67212	-2.65684	0.72498	-1.56987
175.1787	-0.72446	3.67233	-2.65712	0.72507	-1.57002
175.212	-0.72454	3.67259	-2.65749	0.7251	-1.57039
175.245	-0.72462	3.67278	-2.658	0.72513	-1.57141
175.2782	-0.72466	3.67285	-2.65831	0.72518	-1.57205
175.3116	-0.7247	3.67287	-2.65857	0.72523	-1.57225
175.3444	-0.72474	3.67291	-2.65882	0.72533	-1.57227
175.378	-0.7248	3.67299	-2.65912	0.72541	-1.57226

175.4106	-0.7249	3.67325	-2.65943	0.72559	-1.57228
175.4447	-0.72516	3.67355	-2.65971	0.72576	-1.57233
175.4788	-0.72542	3.67381	-2.65996	0.72581	-1.5724
175.5116	-0.72554	3.67435	-2.66021	0.72581	-1.57248
175.5449	-0.72562	3.67502	-2.66047	0.72586	-1.57251
175.577	-0.72566	3.67537	-2.66079	0.72595	-1.57263
175.6109	-0.72572	3.67558	-2.66109	0.72608	-1.57292
175.6441	-0.72578	3.67572	-2.66138	0.72621	-1.5732
175.6776	-0.72584	3.67591	-2.66174	0.72621	-1.57339
175.7118	-0.7259	3.67619	-2.66209	0.72608	-1.57357
175.7444	-0.72598	3.67657	-2.66242	0.72607	-1.57376
175.7777	-0.72624	3.67681	-2.66272	0.72614	-1.57397
175.8108	-0.72658	3.67695	-2.66299	0.72636	-1.57414
175.844	-0.72666	3.67712	-2.66325	0.72653	-1.57419
175.8783	-0.7267	3.67732	-2.66362	0.72658	-1.57418
175.9119	-0.72674	3.67753	-2.6647	0.72654	-1.57417
175.9449	-0.72682	3.67771	-2.66561	0.7265	-1.5742
175.9782	-0.72694	3.67784	-2.66615	0.72647	-1.57431
176.0114	-0.72706	3.67802	-2.66649	0.72651	-1.57442
176.0448	-0.72714	3.67831	-2.66685	0.72655	-1.57455
176.0772	-0.72722	3.6786	-2.66748	0.72655	-1.5747
176.112	-0.7273	3.67962	-2.66826	0.72655	-1.57481
176.1444	-0.72742	3.68032	-2.66864	0.72655	-1.57493
176.1785	-0.72752	3.68063	-2.66888	0.72648	-1.57502
176.2117	-0.7276	3.68083	-2.66911	0.72644	-1.57512
176.245	-0.72768	3.68096	-2.66938	0.7264	-1.57523
176.2776	-0.72772	3.68105	-2.66973	0.72625	-1.57533
176.3115	-0.72776	3.68125	-2.67009	0.72612	-1.57555
176.3453	-0.7278	3.68187	-2.67046	0.72589	-1.57591
176.3784	-0.72788	3.68341	-2.67075	0.72576	-1.57616

176.4117	-0.72818	3.68442	-2.67103	0.7257	-1.57637
176.4456	-0.7284	3.68487	-2.67133	0.72566	-1.57645
176.4777	-0.72852	3.68515	-2.67169	0.72554	-1.57637
176.5116	-0.72862	3.68555	-2.67211	0.72529	-1.57639
176.5448	-0.72872	3.68662	-2.67297	0.72499	-1.57647
176.578	-0.72882	3.68823	-2.67344	0.72442	-1.57659
176.6104	-0.72898	3.68943	-2.67378	0.72419	-1.57676
176.6437	-0.7291	3.68978	-2.6741	0.72384	-1.57692
176.6784	-0.72922	3.68994	-2.67441	0.72335	-1.57703
176.7118	-0.72934	3.69003	-2.67474	0.72306	-1.57711
176.7458	-0.72946	3.69012	-2.67509	0.72284	-1.57717
176.7775	-0.72958	3.69031	-2.67542	0.72267	-1.57721
176.811	-0.72974	3.69096	-2.67575	0.72254	-1.57731
176.8435	-0.72988	3.69193	-2.67616	0.72235	-1.57752
176.8772	-0.73	3.69232	-2.67687	0.72202	-1.57774
176.9106	-0.73012	3.69257	-2.67768	0.72092	-1.57794
176.9447	-0.73028	3.69275	-2.67886	0.72335	-1.57816
176.9778	-0.73042	3.69294	-2.68053	0.72306	-1.5783
177.0105	-0.73052	3.69306	-2.68129	0.72284	-1.57837
177.0438	-0.73064	3.69314	-2.68166	0.72267	-1.57831
177.0777	-0.73076	3.69319	-2.68203	0.72254	-1.57817
177.1092	-0.7309	3.69334	-2.68235	0.72235	-1.57821
177.1424	-0.73104	3.69359	-2.68268	0.72589	-1.57836
177.1752	-0.73116	3.69419	-2.68307	0.72576	-1.57859
177.2093	-0.73128	3.69528	-2.68346	0.7257	-1.57885
177.2416	-0.73142	3.69588	-2.68406	0.72566	-1.57901
177.2749	-0.73158	3.69615	-2.68472	0.72554	-1.57907
177.3085	-0.73172	3.69632	-2.68534	0.72529	-1.57904
177.342	-0.73184	3.69638	-2.68597	0.72499	-1.57901
177.3755	-0.73192	3.69643	-2.68654	0.72442	-1.57909

177.4089	-0.73202	3.69654	-2.68701	0.72405	-1.58004
177.4411	-0.73212	3.69667	-2.68743	0.72412	-1.58162
177.475	-0.73224	3.69684	-2.68777	0.72434	-1.5819
177.5089	-0.73238	3.69704	-2.68807	0.7245	-1.58196
177.5421	-0.7325	3.69731	-2.68847	0.71131	-1.58194
177.5756	-0.73262	3.69772	-2.68908	0.71243	-1.58194
177.6085	-0.7327	3.69799	-2.69015	0.71501	-1.58206
177.6424	-0.73282	3.69814	-2.69139	0.71892	-1.58221
177.6756	-0.73294	3.69817	-2.69192	0.72084	-1.58232
177.7098	-0.73308	3.69819	-2.69233	0.72225	-1.58244
177.7432	-0.7333	3.69829	-2.69273	0.72571	-1.58249
177.7762	-0.73364	3.6986	-2.69315	0.72916	-1.58247
177.8099	-0.73376	3.69948	-2.69356	0.73065	-1.58237
177.8432	-0.73384	3.70049	-2.69399	0.73005	-1.58207
177.8765	-0.73392	3.70079	-2.69433	0.72939	-1.58203
177.9102	-0.73398	3.70098	-2.6946	0.72896	-1.58215
177.9429	-0.7341	3.70103	-2.6949	0.72853	-1.5823
177.9764	-0.73424	3.70108	-2.69523	0.72836	-1.58247
178.01	-0.73438	3.7012	-2.69562	0.72853	-1.58267
178.0432	-0.7345	3.70139	-2.69609	0.72906	-1.58278
178.0771	-0.7346	3.70157	-2.69664	0.72966	-1.58289
178.1104	-0.7347	3.70175	-2.69737	0.73123	-1.58294
178.1431	-0.73478	3.702	-2.69821	0.73223	-1.58286
178.1758	-0.73484	3.7024	-2.69905	0.73351	-1.5828
178.2109	-0.73494	3.7031	-2.70044	0.73447	-1.58291
178.2444	-0.73516	3.70449	-2.7011	0.73501	-1.58311
178.2769	-0.73538	3.70506	-2.70154	0.7358	-1.58335
178.31	-0.7356	3.70528	-2.70192	0.73704	-1.58346
178.3439	-0.73576	3.70539	-2.70223	0.73827	-1.58355
178.3776	-0.73586	3.70547	-2.70257	0.7392	-1.58363

178.4117	-0.73596	3.70561	-2.70307	0.73971	-1.58366
178.4445	-0.736	3.70584	-2.70451	0.73985	-1.5837
178.4779	-0.73604	3.70662	-2.7071	0.73992	-1.58379
178.511	-0.73606	3.70719	-2.70875	0.74002	-1.58394
178.5448	-0.7361	3.70751	-2.70951	0.74004	-1.5841
178.5788	-0.73616	3.70798	-2.70991	0.74013	-1.58419
178.6119	-0.73624	3.70837	-2.7102	0.74096	-1.58416
178.6447	-0.73636	3.70856	-2.71046	0.74281	-1.58406
178.6788	-0.73646	3.70862	-2.71076	0.74394	-1.58405
178.711	-0.73656	3.70865	-2.71108	0.74452	-1.58412
178.7446	-0.73664	3.70872	-2.71143	0.74463	-1.58419
178.778	-0.73668	3.7089	-2.71233	0.74459	-1.58431
178.8109	-0.73676	3.70916	-2.71297	0.7446	-1.58443
178.8449	-0.73686	3.7094	-2.71339	0.74458	-1.58454
178.8775	-0.73702	3.70961	-2.71411	0.74457	-1.58469
178.9116	-0.7372	3.70984	-2.71457	0.74455	-1.58483
178.9448	-0.73734	3.71011	-2.71494	0.74464	-1.58494
178.9777	-0.73746	3.71041	-2.71532	0.74485	-1.58493
179.0122	-0.73756	3.71074	-2.71563	0.74521	-1.58484
179.0453	-0.73764	3.71139	-2.71589	0.74579	-1.58482
179.0786	-0.73772	3.71169	-2.71616	0.746-1.58486	
179.1117	-0.7378	3.71195	-2.71647	0.74612	-1.58499
179.1451	-0.73794	3.71219	-2.71685	0.74627	-1.58509
179.1782	-0.73808	3.71239	-2.71813	0.74638	-1.58514
179.2115	-0.73814	3.71259	-2.71937	0.74647	-1.58521
179.2453	-0.73816	3.71282	-2.72023	0.74651	-1.58528
179.2785	-0.73814	3.71303	-2.72142	0.74649	-1.58527
179.312	-0.73818	3.71323	-2.72243	0.74648	-1.58519
179.3448	-0.73828	3.71344	-2.72284	0.74646	-1.5851
179.3773	-0.73838	3.71371	-2.72318	0.7465	-1.58508

179.4114	-0.73844	3.71404	-2.72338	0.74655	-1.58515
179.446	-0.73854	3.71436	-2.72355	0.74677	-1.58521
179.4784	-0.73876	3.7146	-2.72377	0.74704	-1.58529
179.5116	-0.73938	3.71483	-2.72402	0.74714	-1.58539
179.5443	-0.73968	3.71507	-2.72436	0.74715	-1.58545
179.578	-0.73976	3.7153	-2.72503	0.74712	-1.5855
179.6118	-0.73982	3.71571	-2.7265	0.7471	-1.58551
179.645	-0.73986	3.71648	-2.72735	0.74713	-1.58552
179.6781	-0.73986	3.71712	-2.7277	0.74717	-1.58558
179.7121	-0.73986	3.71746	-2.72801	0.74718	-1.58573
179.7458	-0.7398	3.7178	-2.72831	0.74716	-1.58595
179.7783	-0.73978	3.71804	-2.72907	0.74717	-1.58614
179.812	-0.73982	3.71829	-2.73071	0.74724	-1.58627
179.8452	-0.73992	3.71859	-2.73156	0.74733	-1.58637
179.8784	-0.7403	3.71884	-2.73198	0.74744	-1.5864
179.9114	-0.74066	3.71912	-2.73234	0.74757	-1.58642
179.9454	-0.74076	3.71948	-2.73261	0.74767	-1.58648
179.9789	-0.7408	3.71978	-2.73289	0.7477	-1.58653
180.0119	-0.74082	3.72004	-2.7332	0.7477	-1.58662
180.0451	-0.74084	3.72024	-2.73351	0.74744	-1.58671
180.0784	-0.7409	3.72038	-2.73393	0.74742	-1.58676
180.1115	-0.74098	3.72053	-2.73466	0.7475	-1.58679
180.145	-0.74104	3.72073	-2.73564	0.74768	-1.58687
180.1791	-0.74112	3.72103	-2.73693	0.74789	-1.58699
180.2121	-0.7412	3.72134	-2.73768	0.74798	-1.58714
180.245	-0.74124	3.72169	-2.73804	0.74803	-1.58733
180.2782	-0.7413	3.72202	-2.73831	0.74806	-1.58752
180.311	-0.74136	3.72227	-2.73858	0.74796	-1.58767
180.3442	-0.74144	3.72242	-2.73891	0.74784	-1.5878
180.3786	-0.74154	3.72258	-2.73972	0.74783	-1.58792

180.4117	-0.74164	3.72271	-2.74111	0.74788	-1.58801
180.4454	-0.74172	3.72286	-2.74158	0.748	-1.58812
180.4783	-0.7418	3.7231	-2.74188	0.74821	-1.58818
180.5122	-0.74188	3.72339	-2.74209	0.74845	-1.58813
180.5445	-0.74196	3.72376	-2.7423	0.74896	-1.58796
180.5778	-0.74204	3.72452	-2.74253	0.74989	-1.58787
180.6109	-0.74208	3.72479	-2.74281	0.75036	-1.58792
180.6439	-0.7421	3.72506	-2.74338	0.75043	-1.58821
180.6778	-0.74212	3.72526	-2.74429	0.75042	-1.58877
180.7114	-0.74216	3.72543	-2.74483	0.7504	-1.58899
180.7439	-0.74222	3.72563	-2.74544	0.75041	-1.58914
180.7786	-0.74228	3.72598	-2.74587	0.75048	-1.58924
180.8116	-0.74232	3.72661	-2.74619	0.75058	-1.58928
180.8452	-0.74238	3.72702	-2.74653	0.75063	-1.58919
180.8782	-0.74242	3.72736	-2.74685	0.75067	-1.58906
180.9113	-0.74244	3.72767	-2.74716	0.75068	-1.58905
180.9452	-0.74246	3.72802	-2.74748	0.75067	-1.58909
180.9774	-0.74248	3.72902	-2.74807	0.75067	-1.58916
181.0111	-0.74252	3.73	-2.74868	0.7507	-1.58923
181.0447	-0.74254	3.73084	-2.74968	0.75073	-1.58932
181.0785	-0.74258	3.73146	-2.75088	0.75071	-1.58952
181.1122	-0.7426	3.73179	-2.75169	0.75067	-1.5897
181.1454	-0.74262	3.73212	-2.75262	0.75067	-1.5898
181.1786	-0.74264	3.73243	-2.75307	0.75069	-1.58986
181.2121	-0.7427	3.73274	-2.75338	0.75081	-1.58987
181.244	-0.74282	3.73305	-2.75363	0.75097	-1.58993
181.278	-0.74316	3.73339	-2.75388	0.75109	-1.59004
181.3108	-0.74346	3.73375	-2.75416	0.75115	-1.59011
181.3436	-0.74356	3.73412	-2.75445	0.75111	-1.59019
181.3772	-0.74362	3.73468	-2.75477	0.75103	-1.59028

181.4118	-0.74368	3.73552	-2.75507	0.75096	-1.5904
181.4441	-0.7437	3.73603	-2.75529	0.75089	-1.59051
181.4778	-0.74372	3.73641	-2.75557	0.7509	-1.59052
181.5112	-0.74372	3.73666	-2.75608	0.75089	-1.59043
181.5447	-0.74376	3.73683	-2.75694	0.75084	-1.59024
181.5781	-0.74382	3.737	-2.75765	0.75084	-1.59008
181.612	-0.74392	3.7373	-2.7581	0.75089	-1.5901
181.6454	-0.74396	3.73776	-2.75834	0.75096	-1.59018
181.6781	-0.74398	3.73813	-2.75851	0.75133	-1.59031
181.712	-0.74398	3.73838	-2.75867	0.75164	-1.59071
181.744	-0.744	3.73855	-2.75881	0.75175	-1.59112
181.7785	-0.74406	3.73868	-2.75899	0.7518	-1.59139
181.8115	-0.74424	3.73893	-2.7592	0.75178	-1.59146
181.8457	-0.74448	3.73932	-2.75941	0.75172	-1.59145
181.8788	-0.7446	3.74026	-2.75969	0.7517	-1.5914
181.9124	-0.7447	3.74073	-2.76	0.7517	-1.59131
181.9451	-0.74476	3.7409	-2.76075	0.7517	-1.59123
181.9782	-0.74478	3.74101	-2.76174	0.75166	-1.59123
182.0122	-0.74482	3.74113	-2.76242	0.75165	-1.59131
182.0454	-0.74484	3.74135	-2.76268	0.75163	-1.59144
182.0792	-0.74484	3.74163	-2.76297	0.75162	-1.59154
182.113	-0.74488	3.74191	-2.7633	0.75163	-1.59159
182.1464	-0.74494	3.74221	-2.76362	0.75159	-1.59163
182.1796	-0.74502	3.74251	-2.76396	0.75162	-1.59166
182.2125	-0.74506	3.7428	-2.76431	0.7517	-1.59166
182.2459	-0.74508	3.74302	-2.76465	0.75179	-1.59161
182.2782	-0.74512	3.74323	-2.76525	0.7519	-1.59157
182.3127	-0.74518	3.74358	-2.76573	0.75203	-1.59163
182.346	-0.74524	3.74483	-2.76597	0.75216	-1.59173
182.3793	-0.74528	3.7471	-2.76613	0.7523	-1.59178

182.4117	-0.7453	3.7496	-2.76627	0.75244	-1.59177
182.4455	-0.7453	3.7505	-2.76646	0.75248	-1.59175
182.4786	-0.74536	3.75073	-2.76725	0.75244	-1.59172
182.5131	-0.74544	3.75088	-2.76923	0.75239	-1.59173
182.546	-0.74554	3.751	-2.77001	0.75235	-1.5917
182.5793	-0.74562	3.75113	-2.77038	0.75243	-1.59169
182.6123	-0.74566	3.75134	-2.77067	0.75266	-1.59167
182.6454	-0.7457	3.75159	-2.77097	0.7528	-1.59166
182.6786	-0.74574	3.75186	-2.77132	0.75291	-1.59161
182.7123	-0.74576	3.75232	-2.77163	0.75303	-1.5916
182.7453	-0.7458	3.75314	-2.77195	0.75316	-1.59162
182.7781	-0.74584	3.75378	-2.77244	0.75332	-1.59166
182.812	-0.74586	3.75401	-2.77328	0.75348	-1.59168
182.845	-0.74592	3.75408	-2.77372	0.75357	-1.59161
182.8791	-0.74594	3.75414	-2.77403	0.7536	-1.59152
182.9122	-0.74596	3.75426	-2.77427	0.75363	-1.59148
182.9447	-0.746	3.75497	-2.77452	0.75367	-1.59163
182.9786	-0.74608	3.75734	-2.7748	0.7537	-1.59207
183.0119	-0.74612	3.75861	-2.77514	0.75368	-1.5923
183.0454	-0.74616	3.75887	-2.77576	0.7537	-1.59241
183.0783	-0.74618	3.75908	-2.77625	0.75371	-1.59243
183.1117	-0.74618	3.75934	-2.77666	0.75376	-1.59236
183.1452	-0.7462	3.75969	-2.77698	0.75384	-1.5924
183.1786	-0.74622	3.76028	-2.77722	0.75392	-1.59253
183.2119	-0.74626	3.76084	-2.77745	0.75398	-1.59288
183.2449	-0.74628	3.76123	-2.77771	0.75401	-1.59299
183.2785	-0.74632	3.76179	-2.77803	0.75392	-1.59296
183.3115	-0.74634	3.76228	-2.77833	0.75368	-1.59277
183.3452	-0.74636	3.76274	-2.77859	0.75358	-1.59261
183.3794	-0.7464	3.76307	-2.77881	0.75363	-1.59244

183.412	-0.74652	3.76332	-2.77905	0.75386	-1.5924
183.4449	-0.74676	3.76349	-2.77924	0.75448	-1.59239
183.4787	-0.74694	3.7636	-2.77939	0.75473	-1.59241
183.5116	-0.74698	3.76367	-2.77956	0.75479	-1.59252
183.5452	-0.74698	3.76373	-2.77984	0.75485	-1.59259
183.5781	-0.74698	3.76387	-2.78018	0.75491	-1.59266
183.6116	-0.747	3.76408	-2.7806	0.75489	-1.59271
183.6446	-0.74702	3.76435	-2.78091	0.75491	-1.59273
183.6782	-0.74706	3.76469	-2.7811	0.75493	-1.59274
183.7118	-0.74712	3.76501	-2.78129	0.75498	-1.59276
183.7451	-0.7472	3.76525	-2.78152	0.75507	-1.59278
183.7788	-0.74726	3.76547	-2.7817	0.75514	-1.59284
183.8119	-0.74728	3.76563	-2.7819	0.75526	-1.59292
183.8446	-0.74732	3.76582	-2.78212	0.75537	-1.59299
183.8775	-0.74738	3.76609	-2.78233	0.75547	-1.59305
183.9108	-0.74744	3.76643	-2.78257	0.75558	-1.59311
183.9446	-0.74754	3.76725	-2.78283	0.75567	-1.59318
183.9784	-0.7476	3.76757	-2.78313	0.7557	-1.59322
184.0119	-0.7476	3.76767	-2.78343	0.75569	-1.59324
184.0442	-0.74756	3.76753	-2.78369	0.75568	-1.59322
184.0778	-0.74756	3.7675	-2.78388	0.75561	-1.59319
184.1111	-0.74758	3.76775	-2.78403	0.75559	-1.5932
184.1449	-0.74764	3.76923	-2.78417	0.75564	-1.59322
184.1781	-0.74766	3.77296	-2.78433	0.75561	-1.59319
184.2115	-0.7477	3.77524	-2.78459	0.75562	-1.59318
184.2448	-0.74772	3.77637	-2.78486	0.75563	-1.59317
184.2781	-0.74776	3.77697	-2.7853	0.75565	-1.59316
184.3111	-0.7478	3.77776	-2.78564	0.75575	-1.5932
184.3448	-0.7478	3.77815	-2.78585	0.75589	-1.59326
184.3777	-0.74778	3.7785	-2.78605	0.75594	-1.5933

184.4112	-0.74784	3.77877	-2.78628	0.75595	-1.59341
184.4451	-0.7479	3.77899	-2.7865	0.75594	-1.59351
184.4784	-0.74796	3.77923	-2.78686	0.75591	-1.59352
184.5114	-0.74802	3.77944	-2.7874	0.75592	-1.59351
184.5451	-0.74808	3.77957	-2.78799	0.75595	-1.59345
184.5783	-0.74818	3.77968	-2.78861	0.75599	-1.59344
184.6117	-0.7483	3.77978	-2.78896	0.75602	-1.5935
184.6451	-0.74836	3.77987	-2.78922	0.7561	-1.59356
184.6784	-0.7484	3.78	-2.78948	0.75617	-1.59355
184.7115	-0.74842	3.78015	-2.78978	0.75618	-1.59355
184.7455	-0.74846	3.78028	-2.79009	0.75614	-1.5935
184.7793	-0.7485	3.78037	-2.79034	0.75606	-1.59343
184.8115	-0.74852	3.78043	-2.79052	0.75596	-1.59332
184.8446	-0.74856	3.78051	-2.79065	0.75597	-1.59322
184.878	-0.74856	3.78067	-2.79085	0.7561	-1.59324
184.9112	-0.74852	3.7809	-2.7914	0.75621	-1.59337
184.945	-0.74854	3.78113	-2.7922	0.75629	-1.5935
184.9784	-0.74856	3.78132	-2.79271	0.75629	-1.59361
185.0123	-0.7486	3.78142	-2.79301	0.75626	-1.5936
185.0457	-0.7487	3.78147	-2.79321	0.75631	-1.59358
185.0792	-0.74874	3.78153	-2.79341	0.75642	-1.59361
185.1118	-0.74876	3.78164	-2.79366	0.75657	-1.59363
185.1454	-0.7488	3.7818	-2.79393	0.75667	-1.59374
185.1776	-0.74884	3.78201	-2.79424	0.75666	-1.5938
185.2112	-0.74888	3.78334	-2.79453	0.75657	-1.59383
185.2444	-0.7489	3.78423	-2.7948	0.75647	-1.59387
185.2786	-0.74896	3.78453	-2.79509	0.75645	-1.59385
185.3122	-0.749	3.78499	-2.79548	0.75645	-1.59382
185.345	-0.74902	3.78533	-2.79579	0.75644	-1.59381
185.3788	-0.749	3.78572	-2.79609	0.75638	-1.59375

185.4121	-0.74884	3.78618	-2.79627	0.75619	-1.5937
185.4455	-0.74884	3.78638	-2.79637	0.75609	-1.59363
185.4788	-0.74892	3.78657	-2.79634	0.75604	-1.59358
185.5125	-0.74912	3.78679	-2.79634	0.7561	-1.59358
185.5456	-0.74926	3.78704	-2.79636	0.75625	-1.59354
185.5787	-0.7493	3.78772	-2.79647	0.75666	-1.59347
185.6121	-0.7493	3.78836	-2.79659	0.75679	-1.5935
185.6449	-0.7493	3.78891	-2.79693	0.75681	-1.59351
185.6785	-0.74934	3.78966	-2.79737	0.75678	-1.59358
185.7117	-0.74938	3.79026	-2.79832	0.75672	-1.59363
185.7457	-0.7494	3.79049	-2.79981	0.7567	-1.59357
185.7782	-0.7494	3.79069	-2.80074	0.75668	-1.59353
185.8112	-0.74944	3.79079	-2.80098	0.75664	-1.59354
185.845	-0.7495	3.79091	-2.80118	0.75667	-1.59354
185.8785	-0.74954	3.79114	-2.80134	0.75666	-1.59353
185.9118	-0.74952	3.79168	-2.80155	0.75666	-1.59347
185.9452	-0.74946	3.7933	-2.80174	0.75664	-1.59343
185.9788	-0.74944	3.79458	-2.80186	0.75658	-1.59341
186.0122	-0.74944	3.7948	-2.80199	0.75657	-1.59338
186.0449	-0.7495	3.79499	-2.80217	0.75659	-1.59332
186.0789	-0.74956	3.79507	-2.80244	0.75659	-1.5932
186.1117	-0.74956	3.79508	-2.80274	0.75655	-1.5931
186.1449	-0.7496	3.79519	-2.80288	0.75645	-1.59306
186.1781	-0.74966	3.79531	-2.80304	0.75635	-1.59302
186.2123	-0.7497	3.79538	-2.80327	0.75628	-1.59292
186.2452	-0.74974	3.79552	-2.80352	0.7563	-1.59284
186.2797	-0.74976	3.7957	-2.80375	0.75639	-1.59282
186.3125	-0.74978	3.79588	-2.80381	0.75665	-1.59286
186.3452	-0.7498	3.79605	-2.80376	0.75677	-1.59292
186.3785	-0.74984	3.7962	-2.80392	0.75686	-1.59298

186.4121	-0.74986	3.79631	-2.80417	0.75694	-1.59296
186.4452	-0.74986	3.7967	-2.8045	0.75698	-1.59289
186.4794	-0.74984	3.79812	-2.80478	0.75697	-1.5928
186.5126	-0.74982	3.8004	-2.80496	0.75703	-1.5927
186.5458	-0.74982	3.80318	-2.80512	0.75712	-1.59264
186.5785	-0.74986	3.80401	-2.80532	0.75723	-1.5927
186.6118	-0.74988	3.80427	-2.80551	0.75731	-1.59276
186.6454	-0.74988	3.80445	-2.80572	0.75726	-1.59274
186.6792	-0.74986	3.80458	-2.80594	0.75706	-1.5926
186.7127	-0.74988	3.80472	-2.80619	0.757	-1.59243
186.7455	-0.7499	3.80489	-2.80646	0.75703	-1.59236
186.7791	-0.74996	3.80517	-2.80674	0.75713	-1.59236
186.8112	-0.75004	3.80547	-2.80703	0.75727	-1.59242
186.8456	-0.75014	3.80562	-2.80732	0.75733	-1.59242
186.8778	-0.75024	3.80575	-2.80759	0.75738	-1.59236
186.9112	-0.75026	3.80592	-2.80783	0.75743	-1.59224
186.9451	-0.75024	3.80611	-2.80809	0.75745	-1.59211
186.979	-0.7502	3.80631	-2.80835	0.7574	-1.59203
187.0125	-0.75012	3.80647	-2.80866	0.75736	-1.59197
187.0462	-0.75006	3.80658	-2.80891	0.75736	-1.59192
187.079	-0.75002	3.80671	-2.80911	0.75745	-1.59188
187.1119	-0.75	3.80692	-2.80933	0.75759	-1.59183
187.1443	-0.75	3.80718	-2.8096	0.75789	-1.59183
187.178	-0.75028	3.80736	-2.80988	0.75806	-1.59187
187.2123	-0.7505	3.80752	-2.81015	0.75815	-1.59194
187.2447	-0.75058	3.80775	-2.81039	0.75819	-1.592
187.2787	-0.7506	3.80796	-2.81055	0.75818	-1.59207
187.3125	-0.75052	3.80815	-2.81076	0.75815	-1.59209
187.3458	-0.75038	3.80836	-2.81102	0.75821	-1.592
187.3787	-0.75026	3.80849	-2.81127	0.75835	-1.59189

187.4122	-0.7502	3.80861	-2.81161	0.7585	-1.59175
187.4464	-0.7502	3.80874	-2.81188	0.75865	-1.59167
187.4784	-0.75024	3.80888	-2.81201	0.75872	-1.59159
187.5124	-0.75036	3.809	-2.81219	0.75872	-1.5915
187.5456	-0.75046	3.80911	-2.81239	0.75874	-1.59146
187.5787	-0.75048	3.80929	-2.81261	0.75882	-1.59149
187.6123	-0.75052	3.8095	-2.81285	0.75893	-1.59157
187.6452	-0.75056	3.80976	-2.81306	0.75906	-1.59164
187.6792	-0.75058	3.81029	-2.81328	0.75915	-1.59162
187.7118	-0.75054	3.81066	-2.81395	0.75922	-1.5916
187.745	-0.75026	3.81099	-2.81506	0.75925	-1.59159
187.7783	-0.75002	3.8114	-2.81567	0.75932	-1.59157
187.8125	-0.75	3.81165	-2.81588	0.75939	-1.59151
187.8452	-0.75	3.81186	-2.81599	0.75945	-1.59147
187.8789	-0.75002	3.81206	-2.81613	0.75956	-1.59145
187.9119	-0.75004	3.81226	-2.81638	0.75965	-1.59151
187.946	-0.75006	3.81249	-2.81667	0.7597	-1.59155
187.9788	-0.75008	3.81275	-2.81693	0.75979	-1.59157
188.0124	-0.75012	3.81322	-2.81717	0.75987	-1.59153
188.0456	-0.75014	3.81417	-2.81735	0.75997	-1.59149
188.0784	-0.75014	3.81524	-2.81745	0.76009	-1.59148
188.1115	-0.75014	3.81563	-2.81762	0.76019	-1.59142
188.145	-0.75014	3.81589	-2.81785	0.76024	-1.59138
188.1793	-0.75012	3.81619	-2.81804	0.76029	-1.5914
188.2113	-0.75012	3.8165	-2.81821	0.76035	-1.59143
188.245	-0.7501	3.81677	-2.81833	0.7604	-1.5915
188.2784	-0.75008	3.81701	-2.81841	0.76048	-1.59151
188.3116	-0.75006	3.81726	-2.81863	0.76061	-1.5915
188.3451	-0.75006	3.81764	-2.82015	0.76073	-1.59148
188.3795	-0.75008	3.81847	-2.82337	0.76089	-1.59148

188.4121	-0.75012	3.81908	-2.82527	0.76101	-1.59152
188.4454	-0.75018	3.81943	-2.82587	0.7611	-1.59156
188.4791	-0.7502	3.81966	-2.826	0.76118	-1.59161
188.512	-0.75022	3.81989	-2.82604	0.7613	-1.59164
188.5453	-0.75022	3.82014	-2.82606	0.76148	-1.5915
188.5785	-0.7502	3.82041	-2.82612	0.7616	-1.59142
188.6118	-0.75022	3.82115	-2.82621	0.76161	-1.59139
188.6448	-0.75026	3.82184	-2.82637	0.76157	-1.59156
188.6794	-0.75024	3.82219	-2.82658	0.76154	-1.59216
188.7128	-0.75022	3.82253	-2.8268	0.76161	-1.59238
188.7459	-0.7502	3.82277	-2.82699	0.76173	-1.59232
188.7789	-0.7502	3.82291	-2.82713	0.76183	-1.59224
188.812	-0.75022	3.82299	-2.82736	0.76192	-1.59182
188.8456	-0.75024	3.82315	-2.82757	0.76205	-1.5916
188.8793	-0.75026	3.82337	-2.8278	0.76224	-1.59153
188.9119	-0.75026	3.82381	-2.82799	0.76243	-1.59148
188.9453	-0.75028	3.82448	-2.8281	0.76254	-1.59144
188.9774	-0.7503	3.82477	-2.82829	0.76251	-1.59143
189.0129	-0.75032	3.82501	-2.82851	0.76224	-1.59135
189.0459	-0.7503	3.8253	-2.82877	0.76219	-1.59124
189.0788	-0.7503	3.82556	-2.82928	0.76224	-1.59122
189.1122	-0.75032	3.82585	-2.8297	0.76235	-1.59122
189.1452	-0.75034	3.82618	-2.83003	0.7625	-1.59121
189.179	-0.75036	3.82649	-2.83024	0.76261	-1.59116
189.2122	-0.7504	3.82667	-2.83034	0.76265	-1.59106
189.2447	-0.75044	3.82684	-2.83041	0.76272	-1.59086
189.278	-0.75044	3.82707	-2.83044	0.76284	-1.5908
189.3113	-0.75042	3.82733	-2.83051	0.76295	-1.59073
189.3447	-0.75038	3.82762	-2.83065	0.76309	-1.59067
189.3784	-0.75034	3.82786	-2.83089	0.76322	-1.59066

189.4125	-0.75032	3.82803	-2.83155	0.76331	-1.59068
189.4456	-0.7503	3.82818	-2.83216	0.76335	-1.59067
189.4786	-0.7503	3.82837	-2.83246	0.76337	-1.59064
189.5124	-0.75032	3.82855	-2.8327	0.76336	-1.59054
189.5452	-0.75036	3.82877	-2.83297	0.76342	-1.59044
189.5782	-0.75042	3.82902	-2.83326	0.76353	-1.59032
189.6118	-0.75044	3.82924	-2.83349	0.76364	-1.59021
189.6452	-0.75046	3.82944	-2.83373	0.76378	-1.59015
189.6786	-0.75048	3.82972	-2.83404	0.76396	-1.59011
189.7122	-0.75048	3.8306	-2.83428	0.76416	-1.59016
189.7459	-0.75046	3.83214	-2.83455	0.76435	-1.59024
189.7783	-0.7504	3.8338	-2.83481	0.76442	-1.59027
189.8114	-0.75036	3.83433	-2.835	0.76437	-1.59028
189.846	-0.75032	3.83455	-2.83515	0.76439	-1.59023
189.8792	-0.75028	3.83472	-2.83532	0.76451	-1.59019
189.9117	-0.75024	3.83489	-2.83547	0.76464	-1.59022
189.946	-0.75022	3.83509	-2.8356	0.76477	-1.59023
189.9794	-0.7502	3.83526	-2.83579	0.76488	-1.5902
190.0125	-0.75024	3.83551	-2.83602	0.76501	-1.59016
190.0452	-0.75028	3.83587	-2.83624	0.76519	-1.59008
190.0792	-0.75034	3.83697	-2.83648	0.76532	-1.59008
190.1119	-0.75034	3.83918	-2.8367	0.76542	-1.59015
190.1457	-0.75032	3.84024	-2.83695	0.76555	-1.59018
190.1789	-0.75032	3.84054	-2.83747	0.76569	-1.59016
190.2125	-0.7503	3.84068	-2.83815	0.76586	-1.5901
190.2449	-0.75032	3.84077	-2.83882	0.76604	-1.58999
190.2783	-0.75034	3.84093	-2.83901	0.76612	-1.58987
190.3125	-0.75036	3.84124	-2.83905	0.76609	-1.58982
190.3458	-0.75036	3.842	-2.83903	0.76601	-1.58981
190.3791	-0.7504	3.8429	-2.83898	0.76597	-1.58985

190.4126	-0.75042	3.84339	-2.83904	0.76609	-1.58991
190.4448	-0.75042	3.84358	-2.83915	0.76625	-1.58995
190.4782	-0.7504	3.84368	-2.83942	0.76643	-1.58993
190.5118	-0.75038	3.84378	-2.8405	0.76652	-1.58993
190.5458	-0.75036	3.8439	-2.84139	0.76654	-1.58993
190.5789	-0.75038	3.84404	-2.84173	0.76651	-1.5899
190.6121	-0.75042	3.84419	-2.84188	0.76649	-1.58986
190.6452	-0.75044	3.84438	-2.84196	0.7665	-1.58986
190.6793	-0.75044	3.84472	-2.84211	0.76652	-1.58989
190.7125	-0.75042	3.84499	-2.84234	0.76659	-1.58995
190.7459	-0.7504	3.84522	-2.84259	0.76725	-1.59001
190.7778	-0.75038	3.84542	-2.84291	0.76788	-1.59002
190.8112	-0.75034	3.84564	-2.84316	0.76817	-1.59003
190.8456	-0.75024	3.84606	-2.84341	0.76827	-1.59
190.8787	-0.7501	3.84659	-2.84367	0.76826	-1.58999
190.9131	-0.74998	3.84709	-2.84392	0.76819	-1.58999
190.9463	-0.74992	3.84822	-2.84415	0.76822	-1.58989
190.9794	-0.7499	3.84886	-2.84448	0.76828	-1.58967
191.0126	-0.74988	3.8494	-2.84471	0.7683	-1.58961
191.0454	-0.74982	3.84972	-2.84492	0.7683	-1.58962
191.0784	-0.74978	3.85	-2.84517	0.76811	-1.58975
191.112	-0.74976	3.85025	-2.84538	0.76752	-1.58989
191.1459	-0.74976	3.8506	-2.84563	0.76729	-1.58995
191.1792	-0.74978	3.85089	-2.84592	0.76725	-1.58996
191.2122	-0.7498	3.85109	-2.84626	0.76729	-1.5899
191.246	-0.7498	3.85132	-2.84723	0.76736	-1.58946
191.2791	-0.7498	3.85157	-2.84784	0.76739	-1.58885
191.3126	-0.7498	3.85187	-2.84811	0.7674	-1.58871
191.3457	-0.74978	3.85278	-2.84835	0.76744	-1.58873
191.3787	-0.74974	3.85339	-2.8485	0.76753	-1.58887

191.4118	-0.74968	3.85362	-2.84857	0.76762	-1.58892
191.4451	-0.74964	3.85373	-2.84864	0.76766	-1.5889
191.4794	-0.74964	3.85381	-2.84869	0.76757	-1.58887
191.5125	-0.7496	3.85383	-2.84878	0.76749	-1.58887
191.5455	-0.74958	3.85394	-2.84895	0.76749	-1.58885
191.5791	-0.74938	3.85408	-2.84926	0.76755	-1.5887
191.6115	-0.74928	3.85419	-2.85016	0.76755	-1.58849
191.6454	-0.74926	3.85427	-2.85144	0.7675	-1.58833
191.6794	-0.74926	3.85436	-2.85223	0.76735	-1.58821
191.7125	-0.74922	3.8545	-2.85269	0.76719	-1.58819
191.7451	-0.7492	3.85472	-2.85294	0.76714	-1.58824
191.7787	-0.74916	3.85495	-2.8531	0.76711	-1.5883
191.8125	-0.74912	3.8552	-2.85325	0.76716	-1.58832
191.8459	-0.74908	3.85568	-2.8534	0.76763	-1.58817
191.8792	-0.749	3.85639	-2.85355	0.76798	-1.58748
191.9125	-0.74892	3.85735	-2.85373	0.76803	-1.58736
191.9456	-0.74886	3.85803	-2.85404	0.76797	-1.58732
191.9784	-0.74884	3.85841	-2.85429	0.76773	-1.58737
192.0111	-0.74886	3.85864	-2.85456	0.76758	-1.58743
192.0452	-0.74886	3.85887	-2.85509	0.76756	-1.58716
192.0787	-0.74884	3.85908	-2.85548	0.76755	-1.58629
192.1124	-0.7488	3.85929	-2.85577	0.76757	-1.58542
192.1461	-0.74876	3.8597	-2.85605	0.76761	-1.5847
192.1784	-0.7487	3.86061	-2.85627	0.76764	-1.58426
192.2127	-0.74866	3.86198	-2.85644	0.76769	-1.58417
192.2448	-0.74864	3.86475	-2.85661	0.7677	-1.58406
192.2785	-0.74858	3.86651	-2.85675	0.76773	-1.584
192.3115	-0.74854	3.867	-2.85697	0.76779	-1.58394
192.3451	-0.7485	3.86726	-2.85728	0.76777	-1.58384
192.3792	-0.74848	3.86743	-2.85757	0.76769	-1.58371

192.4132	-0.74842	3.86761	-2.85779	0.76762	-1.58366
192.4457	-0.74836	3.86816	-2.85796	0.76771	-1.58364
192.4785	-0.74816	3.86926	-2.85813	0.76814	-1.58367
192.5133	-0.748	3.86994	-2.8584	0.76855	-1.58383
192.5463	-0.74792	3.87038	-2.85871	0.76869	-1.58389
192.5795	-0.7479	3.87069	-2.85901	0.76867	-1.58394
192.6125	-0.74786	3.87096	-2.85929	0.76862	-1.58397
192.6444	-0.7478	3.87114	-2.85961	0.76862	-1.58397
192.6782	-0.74774	3.87129	-2.8603	0.76864	-1.58396
192.7116	-0.74768	3.87148	-2.86124	0.7687	-1.58386
192.7453	-0.7476	3.87168	-2.86171	0.76874	-1.58352
192.7791	-0.74756	3.87194	-2.86207	0.76874	-1.58339
192.8124	-0.74748	3.87223	-2.86237	0.7688	-1.58332
192.8456	-0.74738	3.87247	-2.86259	0.76884	-1.5835
192.8794	-0.74732	3.87285	-2.86282	0.76889	-1.58378
192.9123	-0.74728	3.87327	-2.86302	0.76896	-1.58377
192.9452	-0.74722	3.8736	-2.86326	0.76909	-1.58368
192.9786	-0.74718	3.87423	-2.86358	0.76921	-1.58358
193.012	-0.74708	3.87458	-2.86402	0.76928	-1.58356
193.0451	-0.74698	3.87486	-2.86489	0.76924	-1.58373
193.0783	-0.74692	3.87511	-2.86644	0.7692	-1.58393
193.1112	-0.74684	3.87529	-2.86873	0.76918	-1.58394
193.1449	-0.74678	3.87542	-2.87069	0.76928	-1.58384
193.1797	-0.74672	3.87562	-2.87143	0.76942	-1.58373
193.2121	-0.74662	3.87586	-2.87176	0.76953	-1.58363
193.2459	-0.74656	3.87614	-2.87207	0.76961	-1.58366
193.2785	-0.74654	3.8764	-2.87234	0.76967	-1.58374
193.3107	-0.74654	3.87666	-2.87264	0.76967	-1.58379
193.3444	-0.74654	3.87695	-2.87293	0.76973	-1.58371
193.3784	-0.74648	3.87775	-2.87314	0.76977	-1.58338

193.4114	-0.7464	3.87836	-2.87346	0.76976	-1.58299
193.4446	-0.7463	3.87865	-2.87399	0.7697	-1.5829
193.4789	-0.74616	3.87877	-2.87492	0.76907	-1.58301
193.5115	-0.74608	3.87882	-2.87648	0.76819	-1.58308
193.5463	-0.74598	3.87883	-2.8778	0.76809	-1.58302
193.5791	-0.74588	3.87899	-2.87916	0.76794	-1.58258
193.6114	-0.74582	3.87941	-2.88049	0.76787	-1.58168
193.6442	-0.74576	3.88037	-2.88217	0.76781	-1.5811
193.6777	-0.74568	3.88089	-2.88297	0.76742	-1.58104
193.7119	-0.7456	3.88118	-2.88349	0.76724	-1.58104
193.7448	-0.74552	3.88139	-2.88382	0.7672	-1.58106
193.7783	-0.74542	3.88151	-2.88415	0.76715	-1.58105
193.8114	-0.74532	3.88163	-2.88455	0.7672	-1.58102
193.8449	-0.74518	3.88176	-2.88535	0.76719	-1.58104
193.8784	-0.74492	3.88186	-2.88629	0.76715	-1.58107
193.912	-0.7447	3.8821	-2.88676	0.76708	-1.58115
193.9457	-0.74462	3.88282	-2.88775	0.76699	-1.58118
193.9786	-0.74454	3.88337	-2.88976	0.76694	-1.58106
194.0118	-0.74446	3.88371	-2.89368	0.76695	-1.58092
194.0453	-0.74438	3.88398	-2.89829	0.76711	-1.58079
194.0787	-0.7443	3.88427	-2.90025	0.76724	-1.58074
194.1119	-0.74422	3.88501	-2.90076	0.76726	-1.58084
194.145	-0.7442	3.88671	-2.90141	0.76721	-1.58086
194.1789	-0.74416	3.88728	-2.90214	0.76707	-1.58082
194.2122	-0.7441	3.88787	-2.90485	0.76693	-1.58075
194.2455	-0.74402	3.88825	-2.90854	0.76684	-1.58065
194.2791	-0.7439	3.88854	-2.90995	0.76683	-1.58056
194.3123	-0.7438	3.88884	-2.91148	0.76684	-1.5805
194.3448	-0.74372	3.88915	-2.91273	0.76687	-1.58041
194.3794	-0.7436	3.88951	-2.91332	0.76688	-1.58035

194.4121	-0.74342	3.89076	-2.91373	0.76693	-1.58032
194.446	-0.74318	3.8912	-2.91408	0.76695	-1.58035
194.4787	-0.74286	3.89149	-2.91443	0.76693	-1.58041
194.5122	-0.74272	3.89172	-2.91492	0.76692	-1.58044
194.5462	-0.74264	3.89196	-2.91551	0.7669	-1.58041
194.5794	-0.74256	3.89242	-2.9159	0.76689	-1.58024
194.6127	-0.74246	3.89339	-2.91627	0.7669	-1.58
194.6458	-0.74236	3.89393	-2.91674	0.76694	-1.57994
194.6791	-0.74226	3.8946	-2.91789	0.76701	-1.57992
194.7124	-0.74216	3.89486	-2.9192	0.76709	-1.57994
194.7455	-0.74208	3.89505	-2.92012	0.7672	-1.57999
194.7784	-0.74196	3.89533	-2.92117	0.76726	-1.57998
194.8126	-0.74182	3.89568	-2.92167	0.76735	-1.57994
194.8458	-0.74166	3.89672	-2.92204	0.7674	-1.57989
194.8788	-0.74132	3.898-2.9227	0.76736	-1.57982	
194.9125	-0.74106	3.89833	-2.92425	0.76732	-1.57982
194.9454	-0.74092	3.89856	-2.92638	0.76726	-1.57979
194.9794	-0.74076	3.89879	-2.92955	0.76722	-1.57969
195.013	-0.74058	3.89901	-2.93202	0.76722	-1.57953
195.0456	-0.74046	3.89933	-2.93321	0.76713	-1.57939
195.0785	-0.74036	3.89967	-2.9338	0.76687	-1.57928
195.1128	-0.74026	3.9005	-2.93422	0.76682	-1.57919
195.1445	-0.74016	3.90128	-2.93466	0.76683	-1.57906
195.1794	-0.74004	3.90168	-2.93553	0.7669	-1.57889
195.2127	-0.73994	3.90221	-2.93787	0.76698	-1.57875
195.2453	-0.73984	3.90275	-2.94239	0.76699	-1.57866
195.2788	-0.73976	3.9031	-2.94484	0.76692	-1.5786
195.3119	-0.73972	3.90345	-2.9455	0.76689	-1.57853
195.3449	-0.73964	3.90372	-2.94611	0.76688	-1.57847
195.3782	-0.7395	3.9039	-2.94649	0.76691	-1.57844

195.4115	-0.73926	3.90412	-2.94694	0.76696	-1.57838
195.4442	-0.7387	3.90434	-2.94813	0.76699	-1.57828
195.4774	-0.73802	3.90458	-2.94918	0.76697	-1.57816
195.511	-0.73746	3.90484	-2.94954	0.76693	-1.57804
195.545	-0.73724	3.90511	-2.95009	0.76692	-1.57799
195.5784	-0.7371	3.90529	-2.95058	0.76695	-1.57804
195.6116	-0.73698	3.90546	-2.95095	0.76702	-1.57802
195.6446	-0.73688	3.90563	-2.95134	0.76713	-1.57799
195.678	-0.7368	3.906	-2.95166	0.76725	-1.57794
195.7105	-0.73676	3.90764	-2.95186	0.76729	-1.57793
195.7432	-0.7367	3.90994	-2.95201	0.76721	-1.57803
195.7771	-0.73664	3.91106	-2.95223	0.76695	-1.57806
195.8111	-0.73654	3.91143	-2.95283	0.7663	-1.57796
195.8443	-0.73646	3.9117	-2.9541	0.76591	-1.57774
195.8786	-0.73638	3.91204	-2.95521	0.76588	-1.5776
195.9114	-0.7363	3.91251	-2.95588	0.76595	-1.57754
195.945	-0.73592	3.9132	-2.95682	0.76611	-1.57757
195.9777	-0.73528	3.91417	-2.95801	0.7665	-1.57761
196.0112	-0.73488	3.91466	-2.95902	0.76654	-1.57757
196.0439	-0.73472	3.91494	-2.95947	0.7665	-1.57747
196.0782	-0.73466	3.91519	-2.95974	0.76637	-1.57732
196.1111	-0.73462	3.91543	-2.95998	0.76602	-1.57721
196.1447	-0.73458	3.91569	-2.96031	0.76553	-1.57715
196.1778	-0.73448	3.916	-2.96071	0.76536	-1.57713
196.2114	-0.7344	3.91664	-2.96122	0.76528	-1.57713
196.245	-0.73422	3.91719	-2.96164	0.76534	-1.57707
196.2772	-0.73378	3.91813	-2.96197	0.76546	-1.57698
196.3104	-0.7336	3.91885	-2.96229	0.76549	-1.57692
196.3434	-0.73348	3.9192	-2.96262	0.76541	-1.57694
196.379	-0.7334	3.91948	-2.96293	0.76529	-1.57703

196.4121	-0.73336	3.91967	-2.96321	0.76523	-1.57708
196.4453	-0.73332	3.9198	-2.96346	0.76517	-1.57706

Figure S30

Control	SD	Untreated	SD	SA	SD	L-SA	SD	D-SA	SD
1	0.15553	4.8338	0.44621	1.0543	0.06476	0.75342	0.1061	1.239	0.05429

Figure S31

Control	SD	Untreated	SD	SA	SD	L-SA	SD	D-SA	SD
1	0.04396	0.88232	0.16798	1.30618	0.21792	1.50181	0.11735	1.2586	0.17143
